# Supplementary figures and images for: A novel eukaryotic RdRP-dependent small RNA pathway represses antiviral immunity by controlling an ERK pathway component in the black-legged tick (part 1 of 3)
Source: PLoS One. 2023 Mar 30;18(3):e0281195. doi: 10.1371/journal.pone.0281195 (PMC10062562; doi:10.1371/journal.pone.0281195)

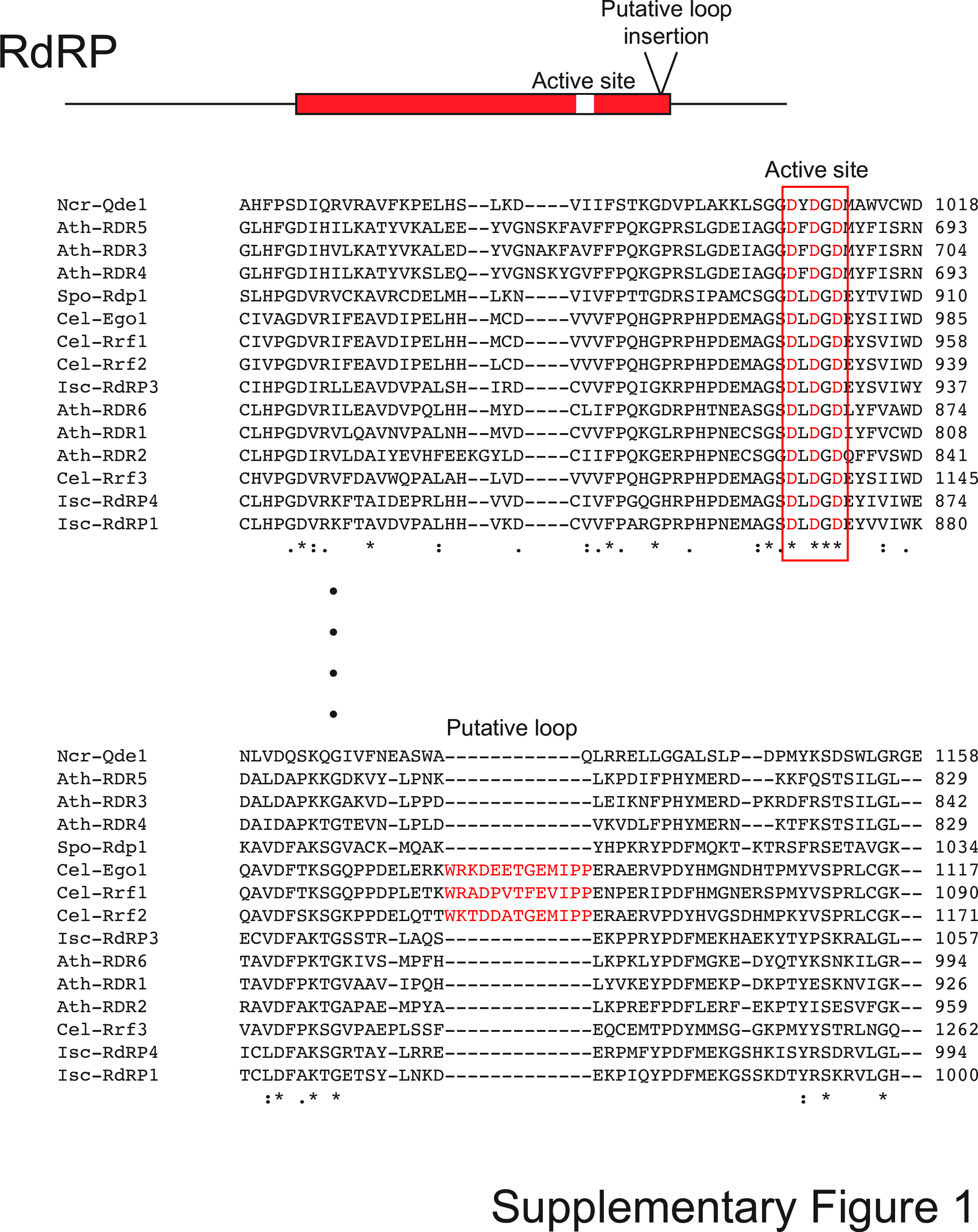

Supplement: S1 Fig — The sequences of RdRP homologs from Fission yeast (Spo), Neurospora (Ncr), Arabidopsis (Ath), worm (Cel) and tick (Isc) were aligned by MUSCLE <https://www.ebi.ac.uk/Tools/msa/muscle/> and the regions near the catalytically active site and the Rrf1-specific putative loop are shown. The accession numbers of the gene sequences can be found in S1 Table. (TIF) [file pone.0281195.s001.tif]

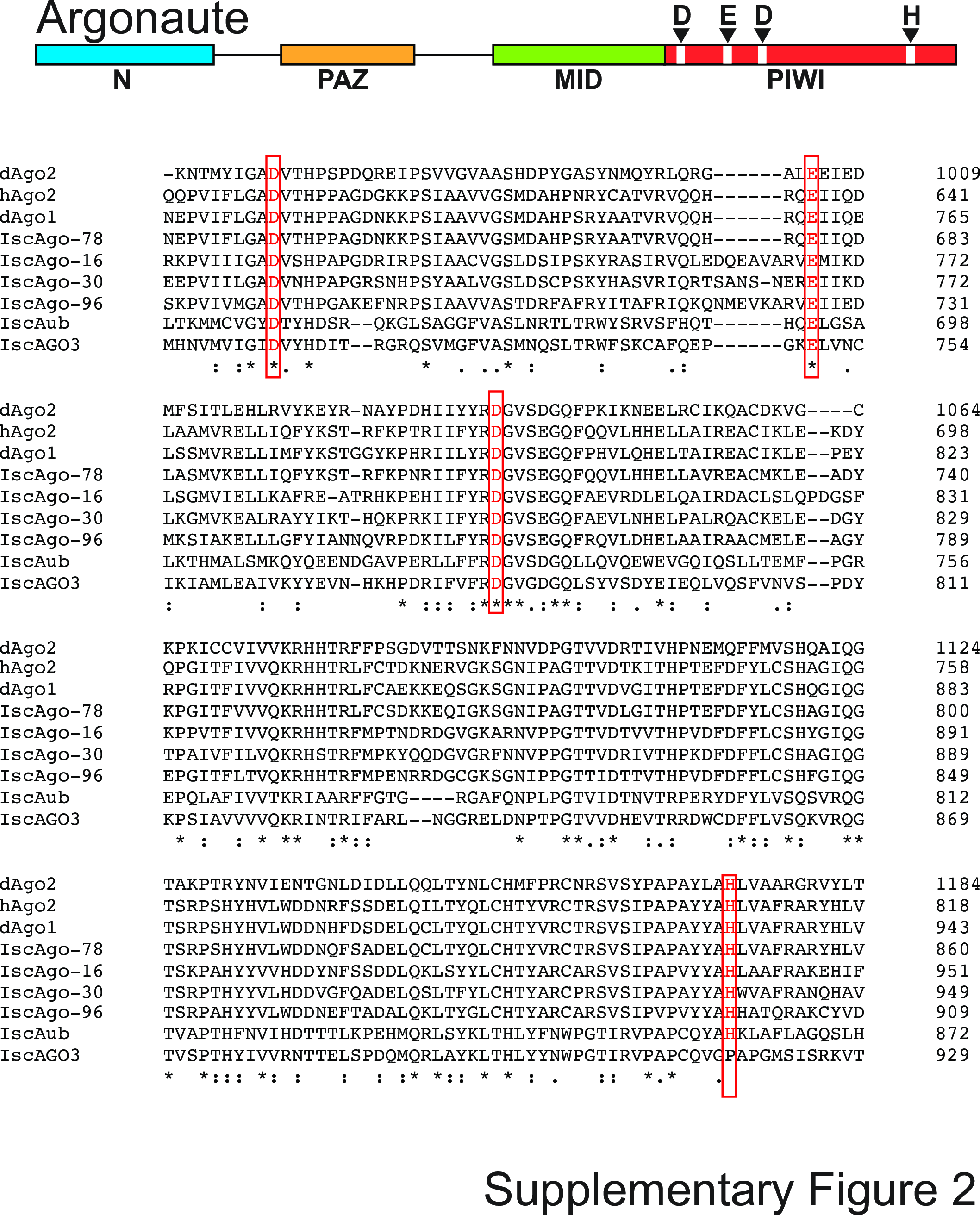

Supplement: S2 Fig — The sequences of AGO homologs from Drosophila (dAGOs), human (hAGOs) and tick (IscAGOs) were aligned by CLUSTAL Omega < https://www.ebi.ac.uk/Tools/msa/clustalo/> and the region containing the catalytic residues is shown. The conserved DEDH residues are highlighted in red. The accession numbers of the gene sequences can be found in S1 Table. (TIF) [file pone.0281195.s002.tif]

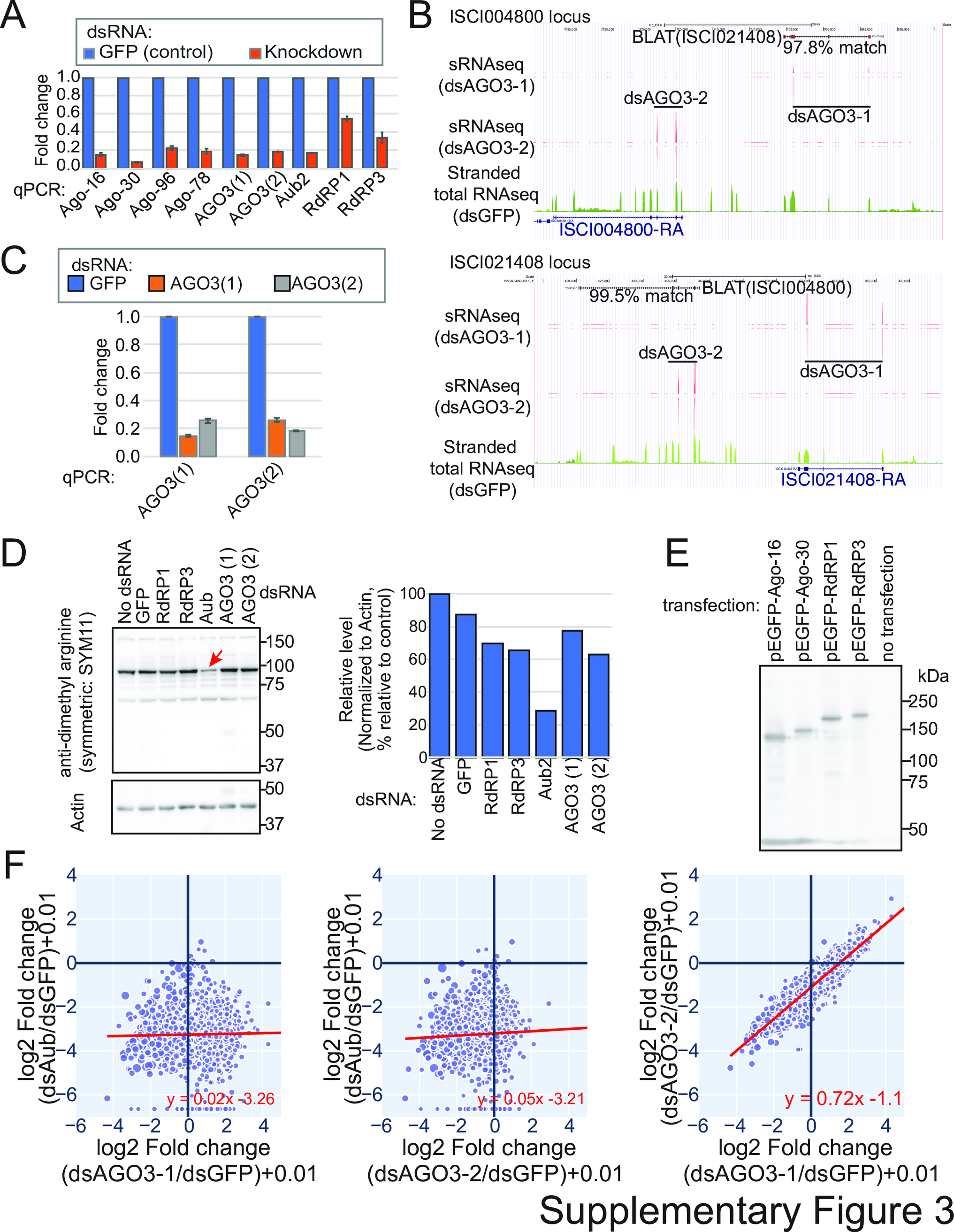

Supplement: S3 Fig — (A) Verification of gene knockdown. RNA samples isolated from ISE6 cells transfected with the indicated dsRNAs were used for qPCR verification. The qPCR primers were designed to quantify the expression of the cognate gene but avoided the region corresponding to the introduced dsRNA sequence. The values were normalized by the Actin expression and further normalized to the value in the dsGFP control sample. Four technical replicates were made and the averages and the standard deviations are shown. (B) ISCI021408 and ISCI004800 correspond to two fragments of the IscAGO3 gene. There are two loci in the ISE6 assembly that match with ISCI021408 and ISCI004800 genes at high homologies (>97.8%) and the gene structures were conserved in the two loci, suggesting that these two loci may represent a very recent gene duplication or a genome assembly artifact. (C) qPCR verification of AGO3 knockdown against ISCI021408 (dsAGO3-1) and ISCI004800 (dsAGO3-2). Note that transfection of either dsRNA construct resulted in strong reduction of both ISCI021408 and ISCI004800 qPCR amplicons, indicating the dsRNA constructs reduce both genes. Based on the observations in (B), we consider the two loci as a single gene, IscAGO3. (D) Western blot analysis for the PIWI proteins detected by anti-symmetric dimethyl arginine antibody, SYM11. ISE6 cells were treated with the indicated dsRNAs and total protein was extracted and separated on an SDS-PAGE gel. The proteins were detected by SYM11, which recognizes symmetric dimethyl arginine that is a characteristic of PIWI proteins and some other RNA binding proteins [32]. The quantified values of the band at ~100kDa were normalized to the value of the Actin band and shown in the bar chart next to the Western blotting panel. Note the strong reduction of the 100kDa band upon Aub KD, suggesting that this band corresponded to the Aub protein. (E) HEK293T cells were transfected with the indicated construct and total protein samples were subjected to Western [file pone.0281195.s003.tif]

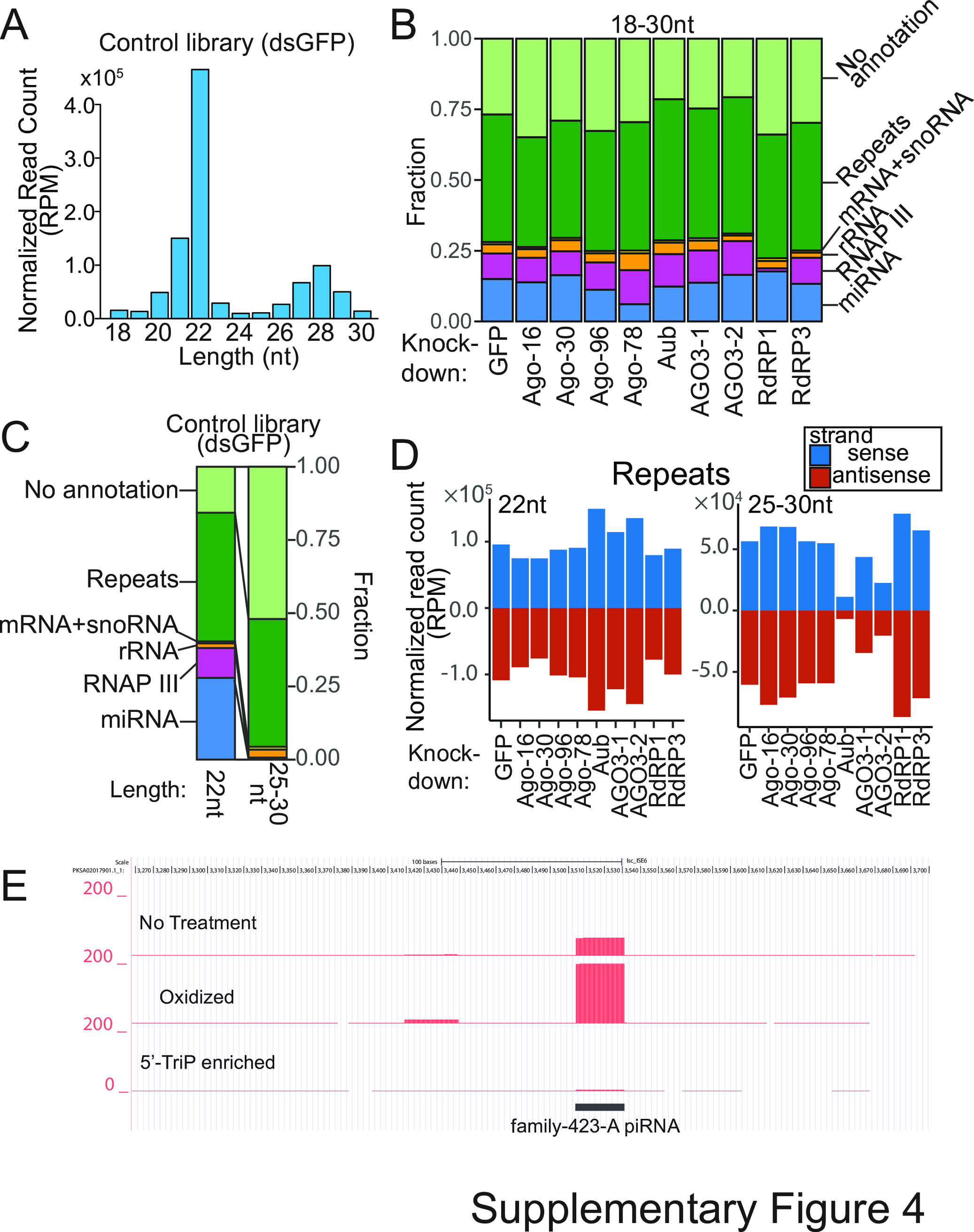

Supplement: S4 Fig — (A) Size distribution of sRNA reads. Genome-matching sRNA reads of each length in the control (dsGFP) library were counted and normalized by the number of reads of all lengths and expressed as RPM (Reads Per Million mapped reads). (B) Percent stacked-bar-chart showing the composition of sRNAs in the knockdown libraries. The fractions of sRNAs derived from miRNAs (blue), RNAP III transcribed genes (pink), rRNAs (orange), snoRNAs + mRNAs (yellow), repeats (green) and sRNAs that did not belong to any of the categories (light green) are shown. 18-30nt reads were used. Note that the same bars are shown in Fig 2A for dsGFP, dsAgo-78, dsRdRP1 and dsRdRP3. (C) Percent stacked-bar-chart showing the composition of sRNAs in the control (dsGFP) library. 22nt (left bar) and 25-30nt (right bar) reads were used. (D) Normalized read count (RPM) of sense and antisense (22nt reads, left; 25-30nt reads, right) that were derived from repeats in the indicated knockdown libraries are shown in blue and red, respectively. Negative values were given to antisense read counts. (E) UCSC Genome Browser screenshot of the TE family-423 region. A strong peak of a piRNA (family-423-A) was observed in the control library, which was prepared with the standard protocol. This peak was even higher in the library prepared after oxidization of the total RNA, supporting that this RNA species have a 2’-O-me group similar to piRNAs in other organisms. These species were depleted when 5’-mono-phosphorylated species were removed, suggesting that this RNA species had a 5’-mono-phosphate group. (TIF) [file pone.0281195.s004.tif]

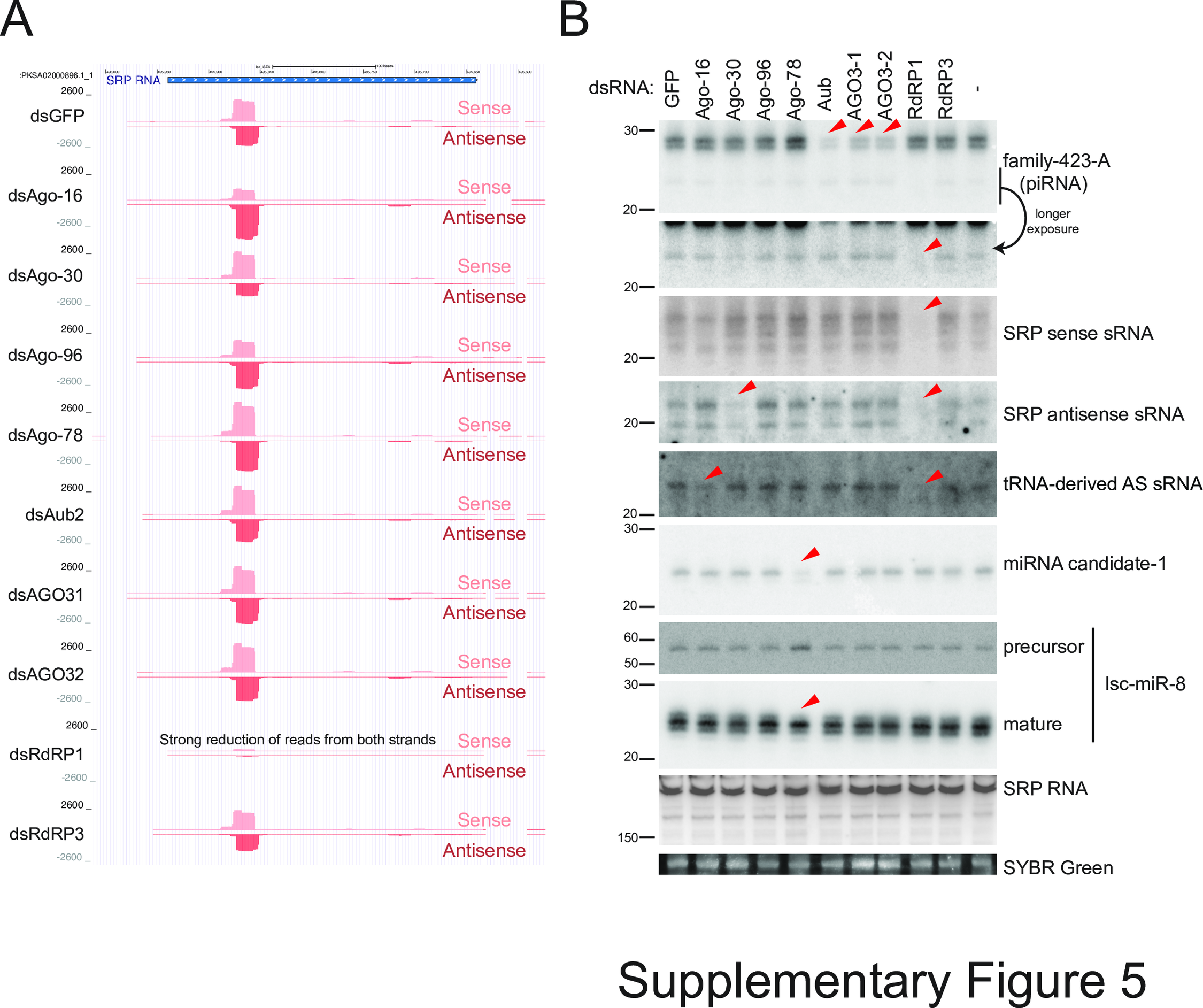

Supplement: S5 Fig — (A) UCSC screenshot at the SRP RNA locus shown as an example of a locus producing RdRP1-dependent sRNAs. The tracks show sRNA mapping densities in the indicated libraries from the sense (upward) and antisense (downward) strands of the locus with respect to the direction of the transcription. (B) Verification of sRNA library analysis by Northern blotting of representative sRNA species. 10ug total RNA from each of the indicated knockdown samples was loaded, and the membrane was incubated with the indicated probes. The probe sequences can be found in S5 Table. The >150nt (SRP RNA), ~50-60nt (pre-mir-8) and ~20-30nt (other panels) areas of the blots are cropped and shown. miRNA-candidate-1 was not in miRBase but identified by visual inspection of mapping data as well as RNA secondary structure prediction for its precursor structure. The full analysis for novel miRNA genes will be published elsewhere. The SYBR Green II staining result is shown as the loading control. Red arrowheads show the bands that showed a clear reduction in the knockdown lanes. (TIF) [file pone.0281195.s005.tif]

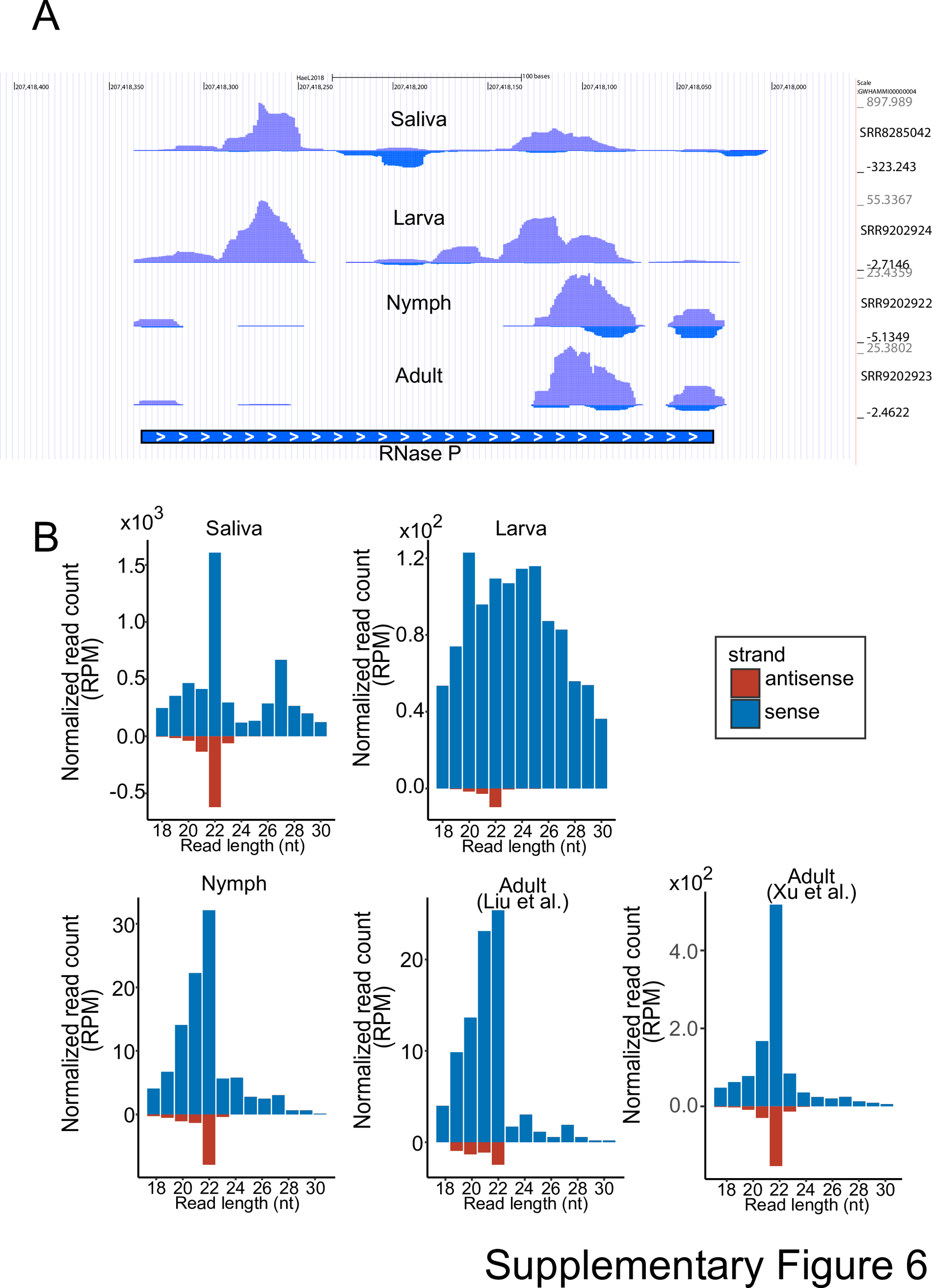

Supplement: S6 Fig — longicornis. (A) UCSC genome browser screenshot of the RNase P locus in the H. longicornis genome. sRNA mapping density is shown. The upper (purple) and lower (blue) signals represent sense and antisense reads. The sRNAseq data are from previous studies [43,44]. (B) Size distribution of sRNAs mapping to the representative RNAP III-dependent genes (RNase P, RNase MRP and SRP RNA) of H. longicornis. The upper left and lower right panels are reanalysis of data from [43] and [45], respectively. Other panels are using [44]. For both (A) and (B), the saliva data are also shown in Fig 2E and 2F. (TIF) [file pone.0281195.s006.tif]

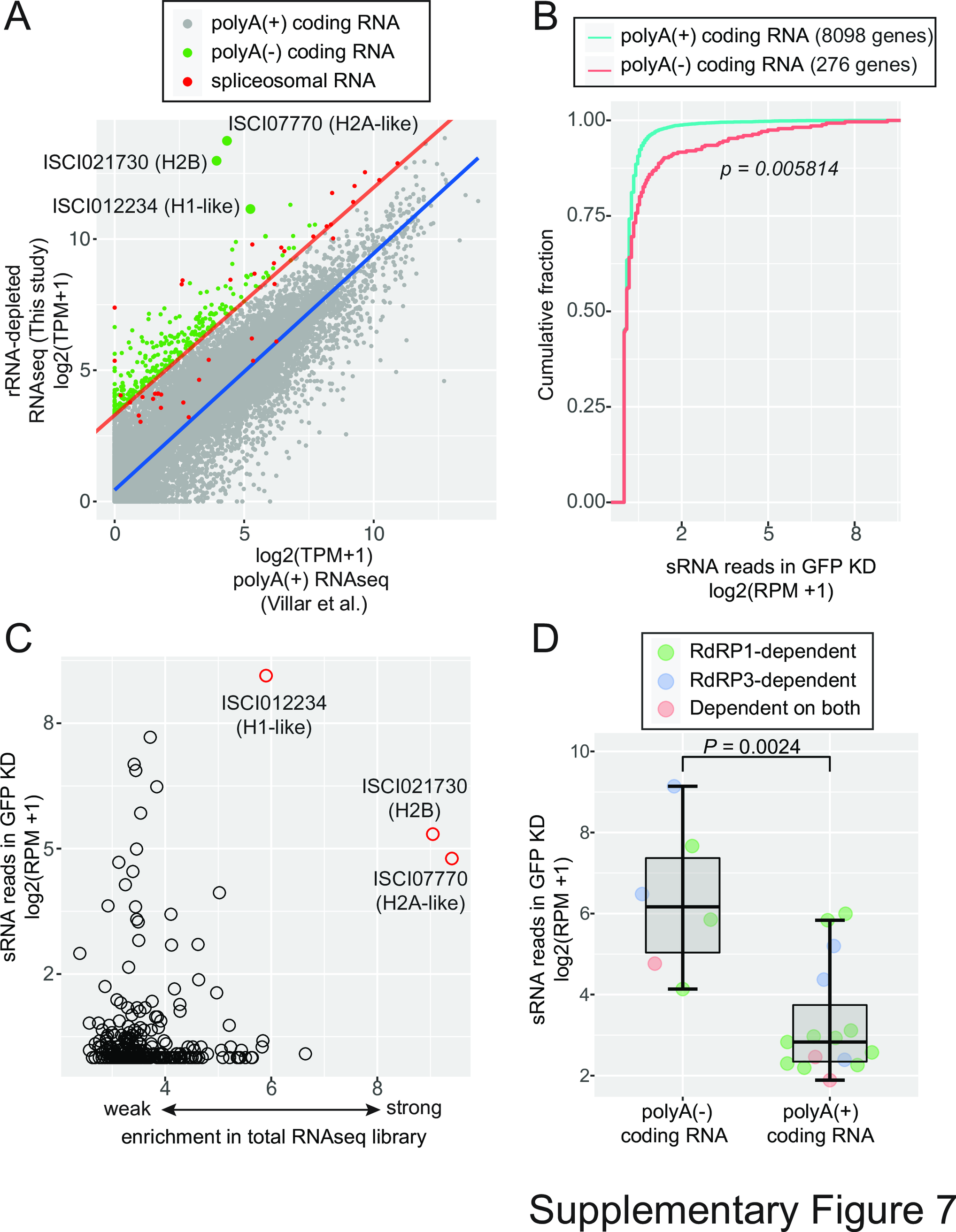

Supplement: S7 Fig — (A) Identification of mRNAs enriched in rRNA-depleted RNAseq libraries. Log2 of the average TPM values from ISE6 cells in total RNAseq libraries (this study; transfected with dsGFP) or polyA-enriched RNAseq libraries (control samples)[50]. To determine the cut off value, the linear regression line of spliceosomal RNA datapoints (red) was used. The green and grey dots represent mRNAs that were judged as polyA(-) and polyA(+) coding RNAs, respectively. Because of the enrichment cut off, polyA(-) coding RNAs had at least log2(TPM+1)> 3.288885. Therefore, only polyA(+) coding RNAs with log2(TPM+1)> 3.288885 were used for analysis in (B) to account for gene expression levels. The histone homologs found in the polyA(-) group are highlighted by larger dots and their gene IDs are shown. (B) The distributions of sRNA production values of polyA(-) (Pink line) and polyA(+) (Green line) coding RNAs are shown in a CDF plot. The RPM normalized sRNA counts for polyA(-) and polyA(+) coding RNAs were used. The X-axis shows log2 of the RPM value +1 in the sRNA libraries using ISE6 cells transfected with dsGFP. The p-value was calculated by the Wilcoxon test. (C) The sRNA production value (log2 of RPM value +1) of each polyA (-) coding gene was plotted against the enrichment factor in the total RNAseq library (log2(total RNAseq TPM +1)–log2(polyA RNAseq +1)). There are many genes showing strong enrichment in the total RNAseq library while not producing many sRNAs. The histone homologs found in the polyA(-) group are highlighted by red circles and their gene IDs are shown. (D) RdRP-dependent sRNA production from polyA(+) and (-) groups. The loci producing sRNAs that are RdRP1-dependent (green), RdRP3-dependent (blue) and dependent on both RdRPs (pink) are shown. The genes were grouped based on the analysis shown in Fig 3. Y-axis shows the sRNA level in the control dsGFP library (log2 of RPM+1). The p-value was calculated by the Wilcoxon test. (TIF) [file pone.0281195.s007.tif]

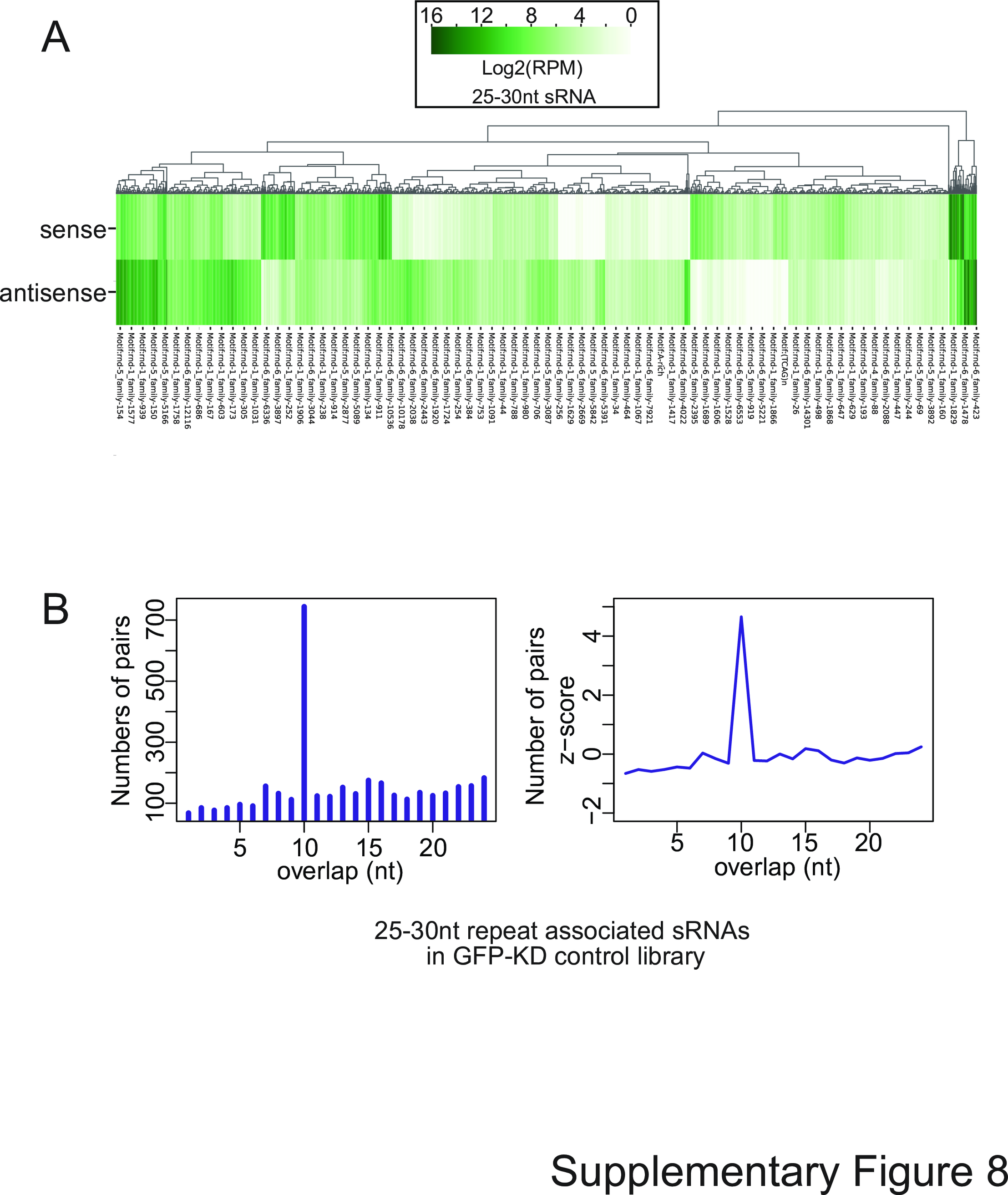

Supplement: S8 Fig — (A) Heat map of sense/antisense 25-30nt sRNA levels from individual repeat families in the control library. Repeats with at least 16 RPM 25-30nt reads were used. (B) The 5’-5’ overlap between sense and antisense repeat-derived 25-30nt sRNAs was analyzed and the numbers of read pairs with 1-25nt overlaps (left) and the significance of the overlap (Z-score: right) are shown. A significant signal was observed at 10nt (Z score> 2.58 corresponds to p<0.01). (TIF) [file pone.0281195.s008.tif]

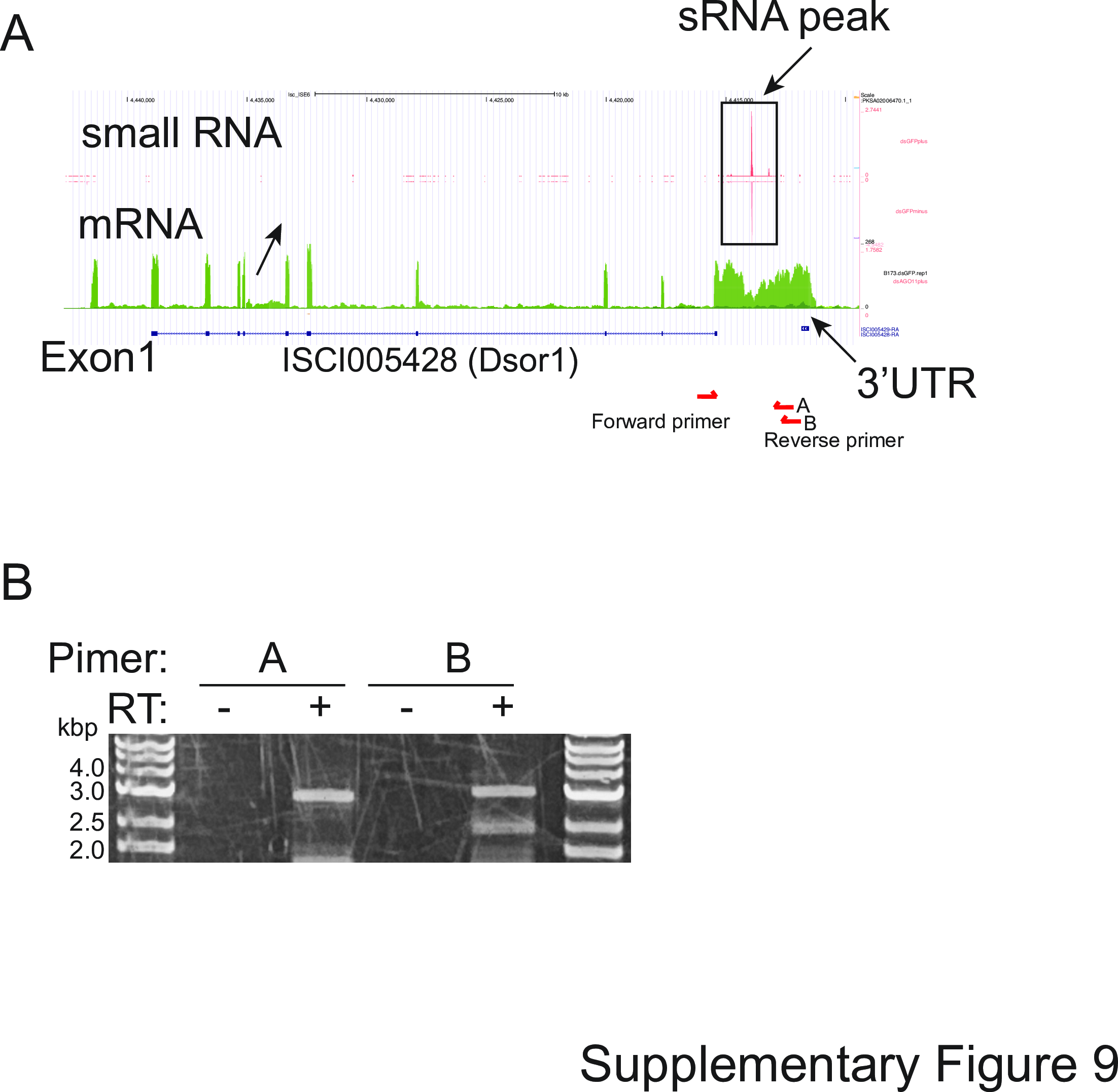

Supplement: S9 Fig — (A) UCSC genome browser screenshot of the Dsor1 (ISCI005428) locus in the I.scapularis genome. sRNAseq read density (upper) and stranded total RNAseq read density from the control libraries are shown. The positions of RT-PCR primers used in (B) are shown. The forward primer was designed against the 3’-end of the coding region, and the reverse primers were designed against the putative 3’UTR sequence. (B) RT-PCR results. Using the primers illustrated in (A), RT-PCR was performed with (+) or without (-) adding reverse transcriptase in the RT reaction. Both primer pairs amplified the expected fragments in a reverse-transcription dependent manner, confirming the extended sequence represents the 3’UTR of Dsor1. The band amplified with pair B was cloned and sequenced, and used for the luciferase assays shown in Fig 6D. (TIF) [file pone.0281195.s009.tif]

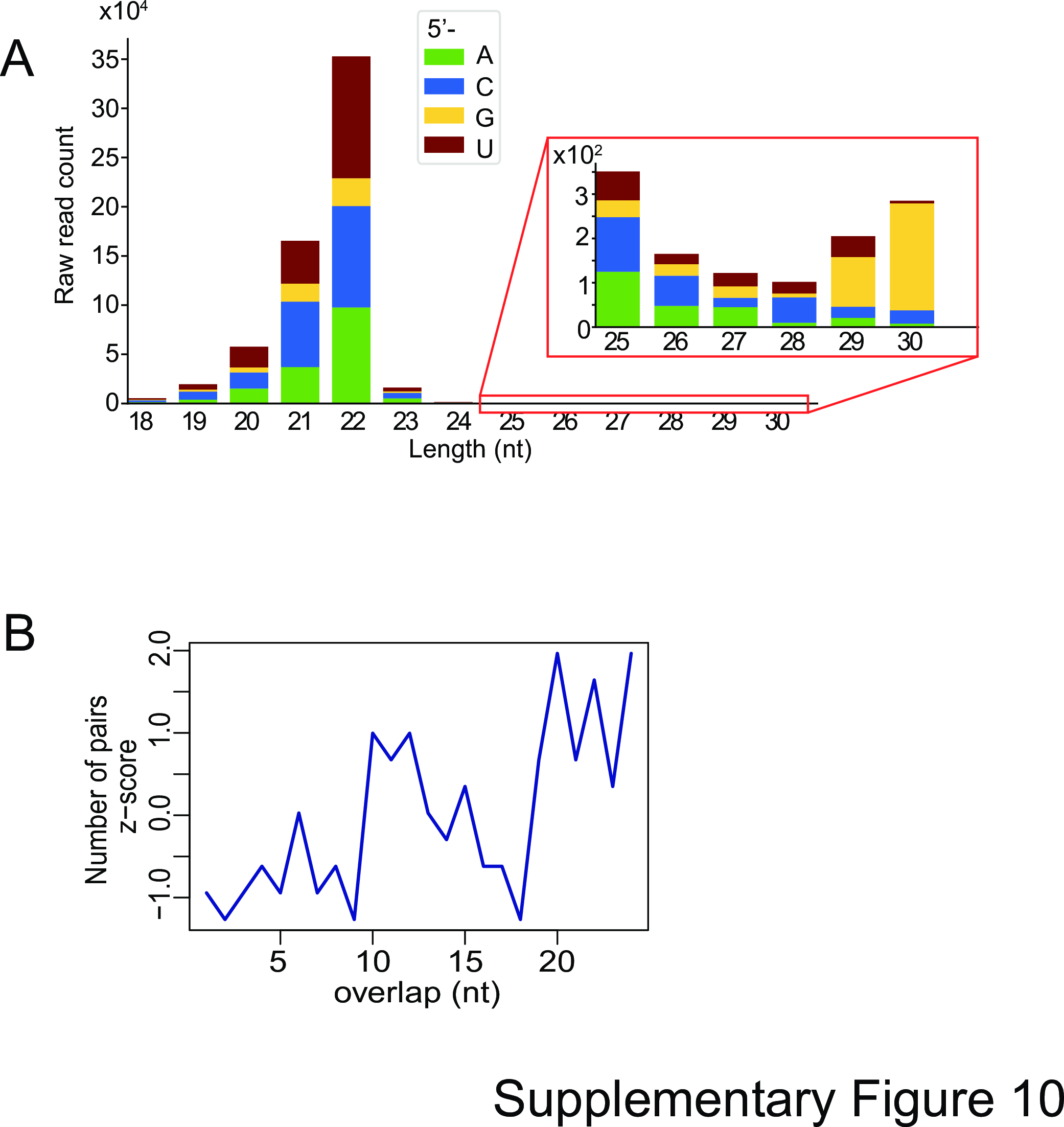

Supplement: S10 Fig — (A) Viral sequence contigs were assembled using ISE6 sRNA library data and reads were remapped to the contigs. Reads were grouped based on the 5’ nucleotides and the size distribution is shown. The X- and Y-axes show the size and the raw read count, respectively. The inset shows an enlarged view of the 25-30nt window with a smaller Y-scale. (B) The 5’-5’ overlaps between sense and antisense virus-derived 25-30nt sRNAs were analyzed and the number of read pairs with 1-25nt overlaps (left) and the significance of the overlap (Z-score: right) are shown. No significant signal was observed at 10nt, suggesting the lack of ping-pong amplification of viral sRNAs. (TIF) [file pone.0281195.s010.tif]

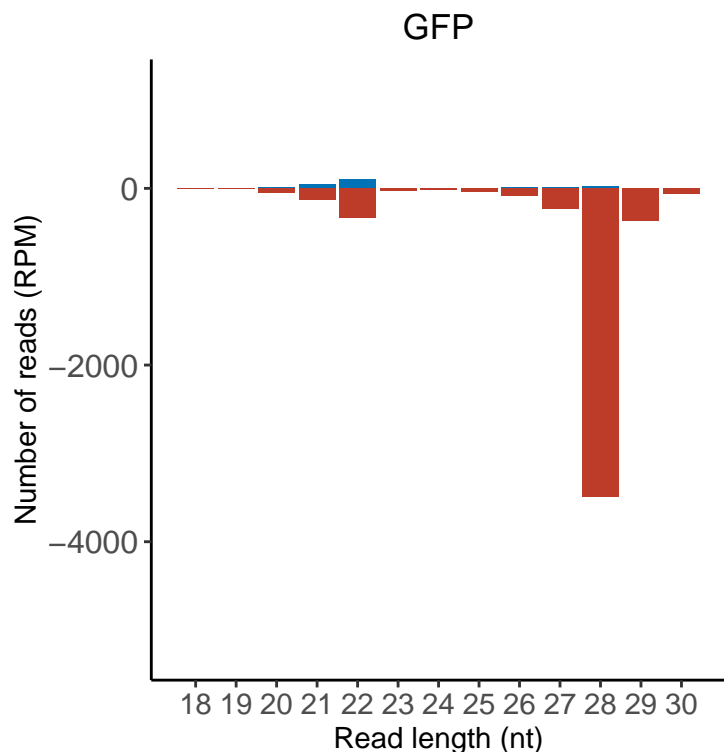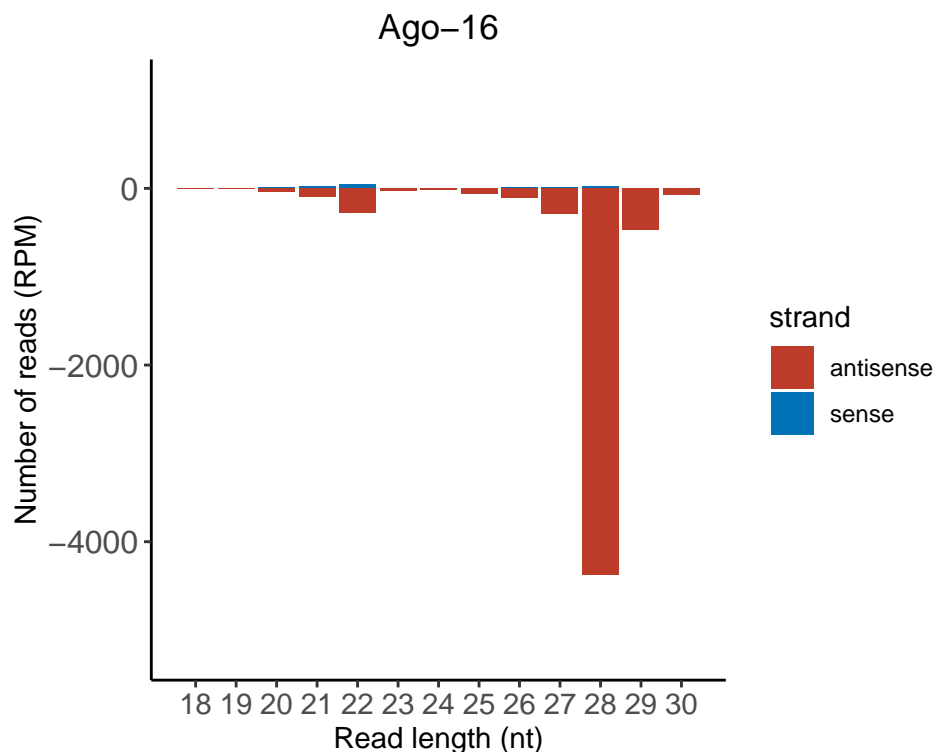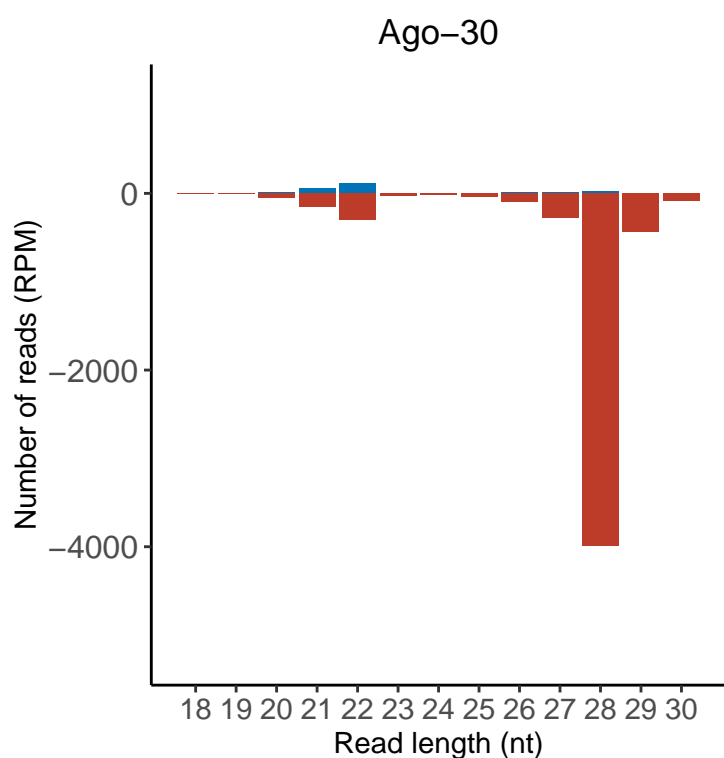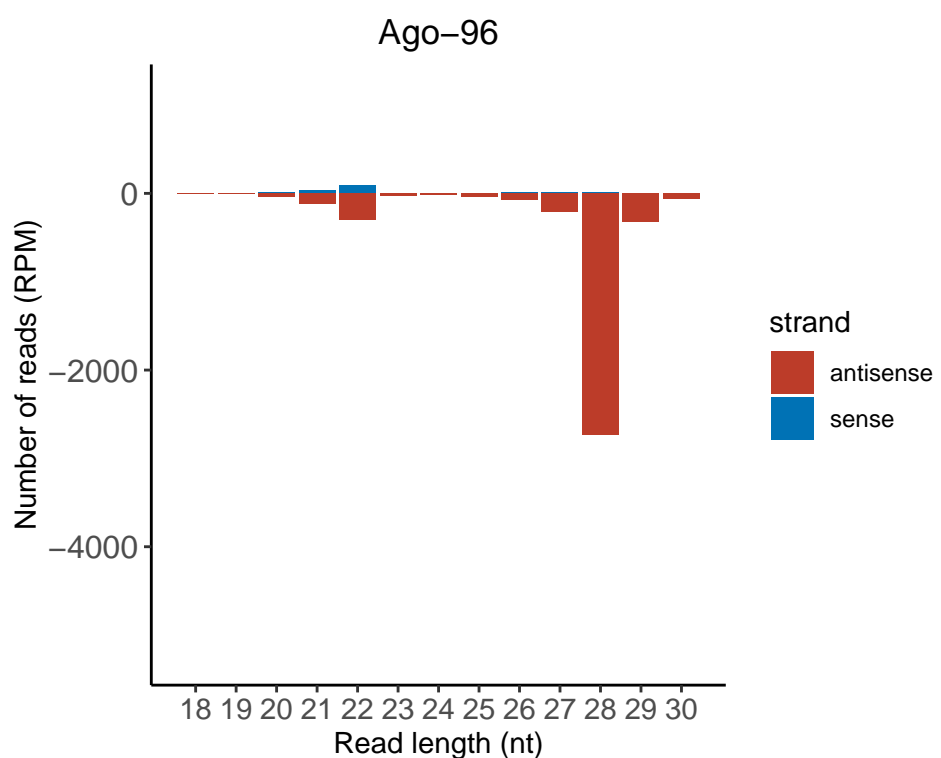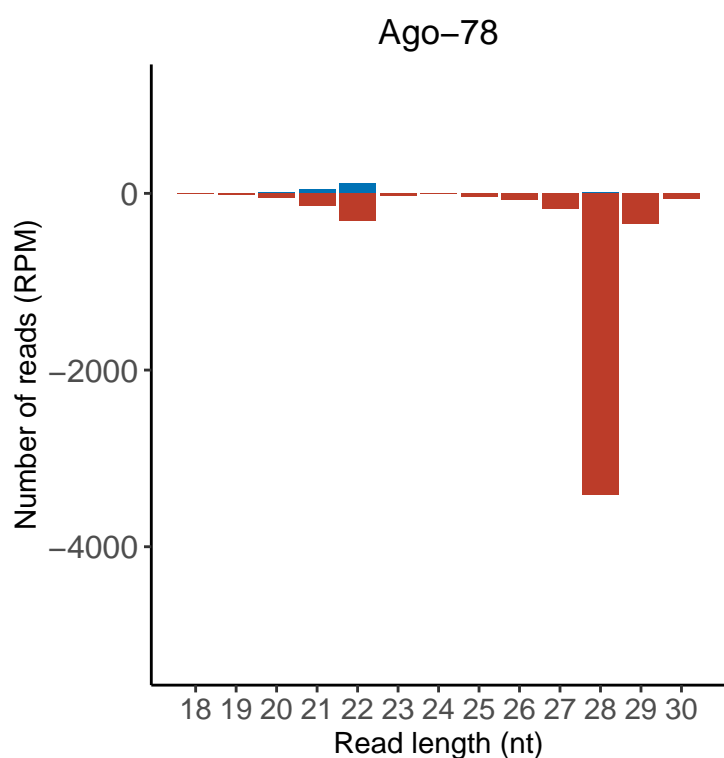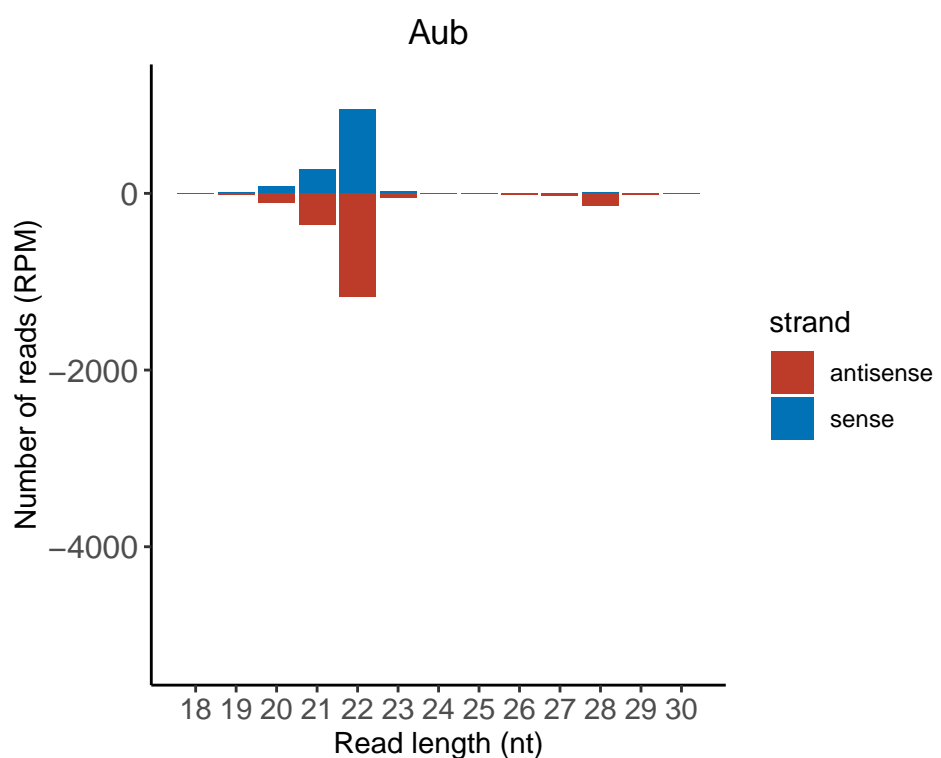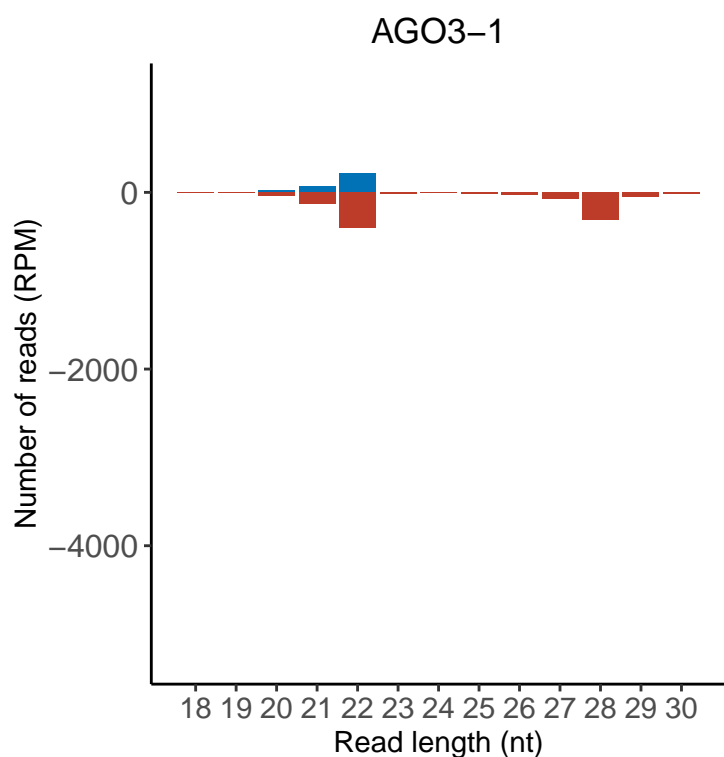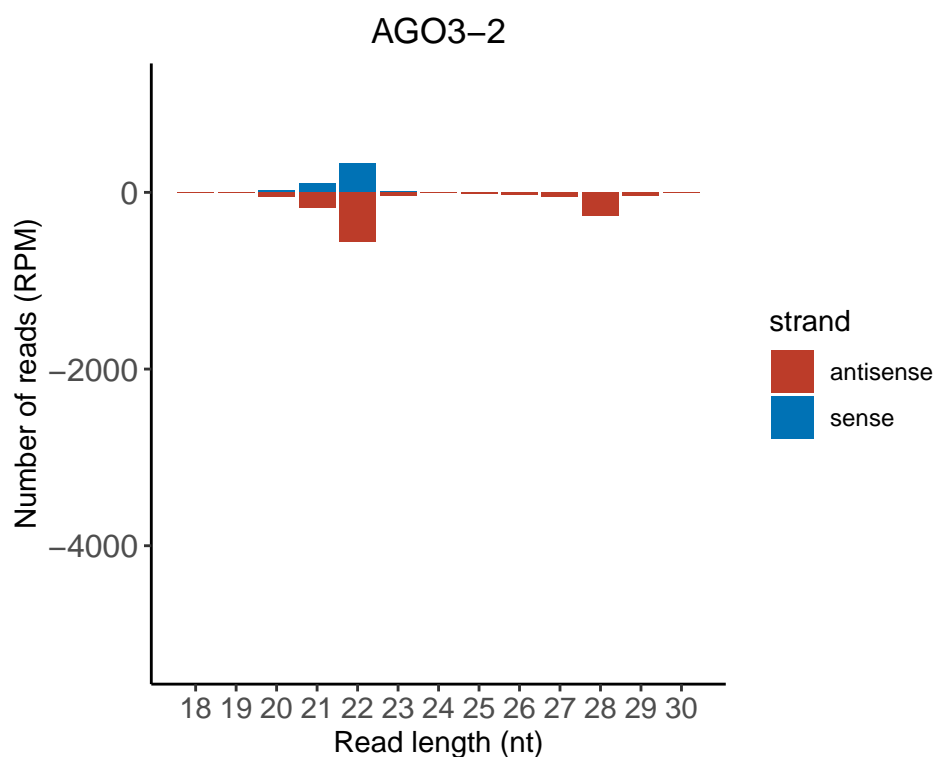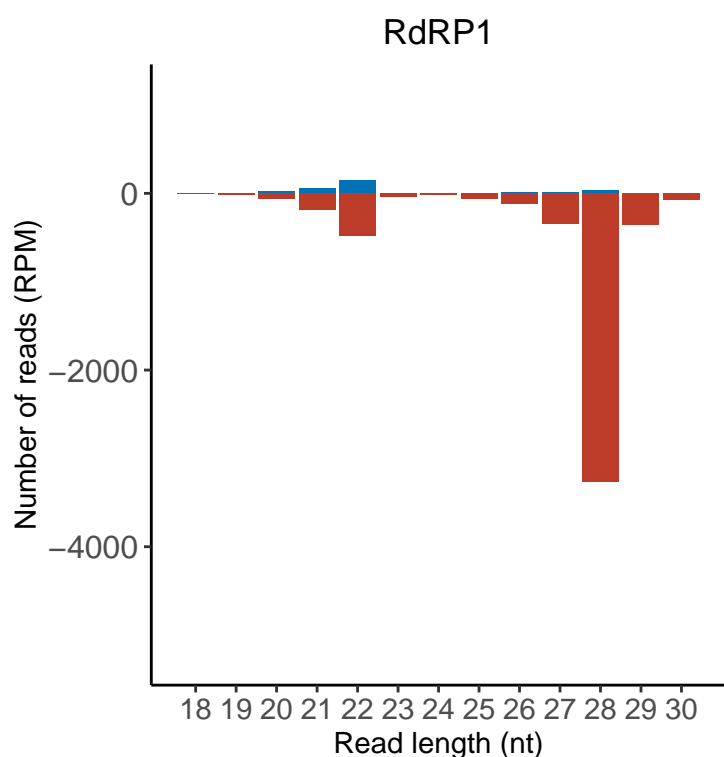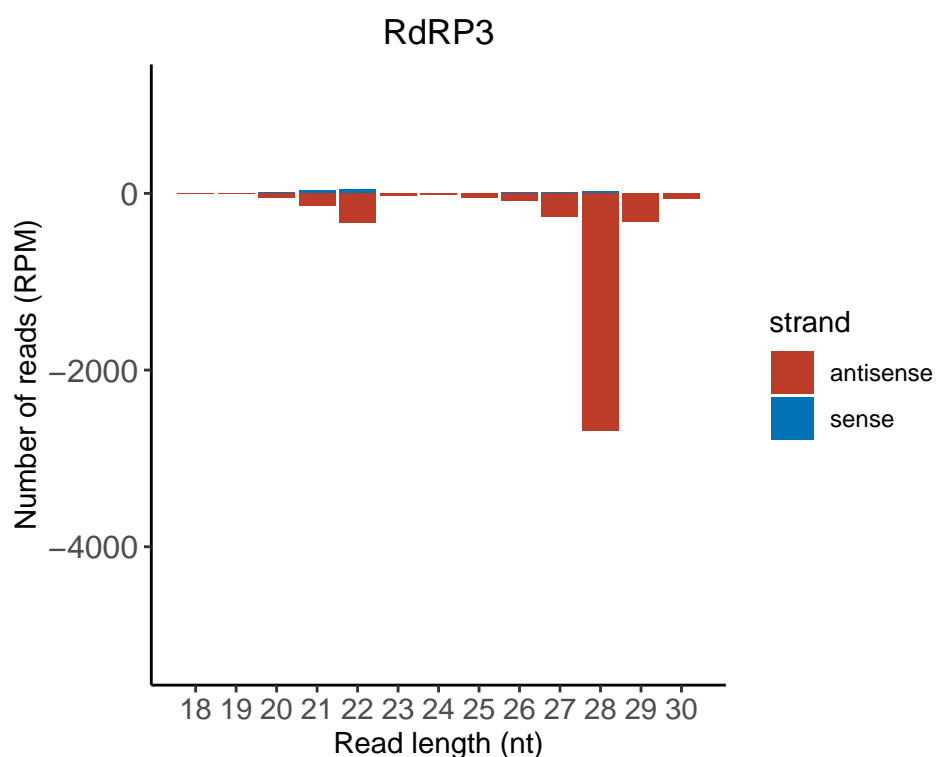

Supplement: S1 Data — On the Normalized and Relative levels pages, normalized read counts (RPM) and relative levels compared to the control (dsGFP) library were used. TEs with more than 50RPM and Coding Genes with more than 3.5RPM on average in KD libraries are included in this website. For viral sequences, reference sequences were generated by assembling sRNA sequences (See the Analysis of sRNAseq data section in Materials and methods.) and size distributions of sRNA reads mapped to each contig are shown. Reads were first grouped by their 5’ nucleotides, and reads of each length were counted. Full tables including TEs and Coding genes with fewer reads are in the “FullTable” folder. (ZIP) [file pone.0281195.s017.zip › SupplementaryData/Links/PDFs/Motif:rnd-5_family-5812TE_lengthdistribution.pdf]

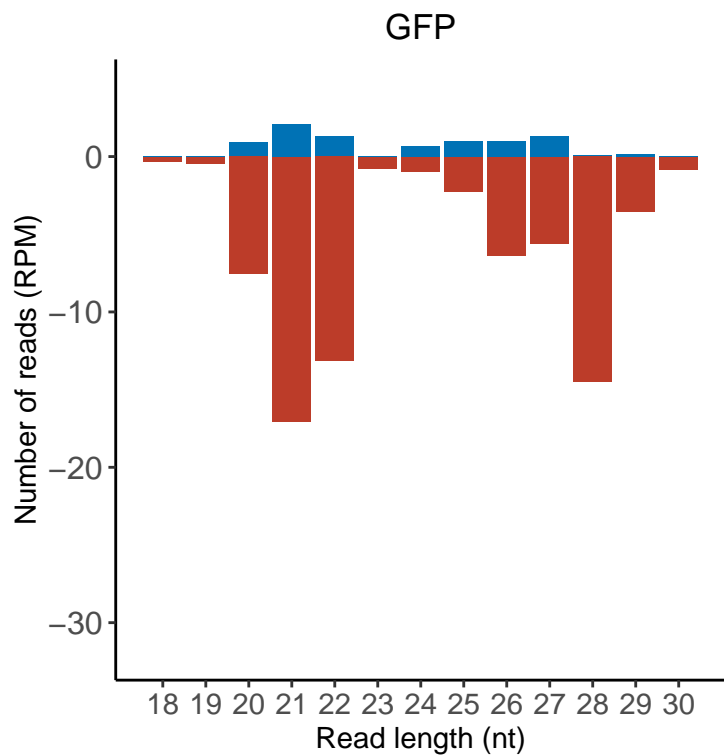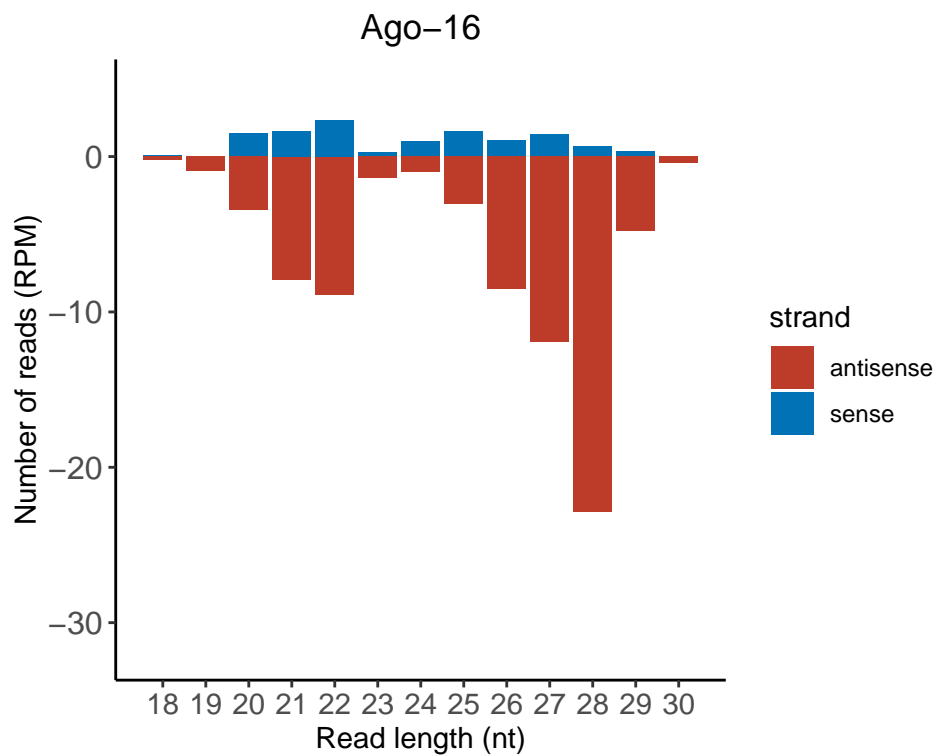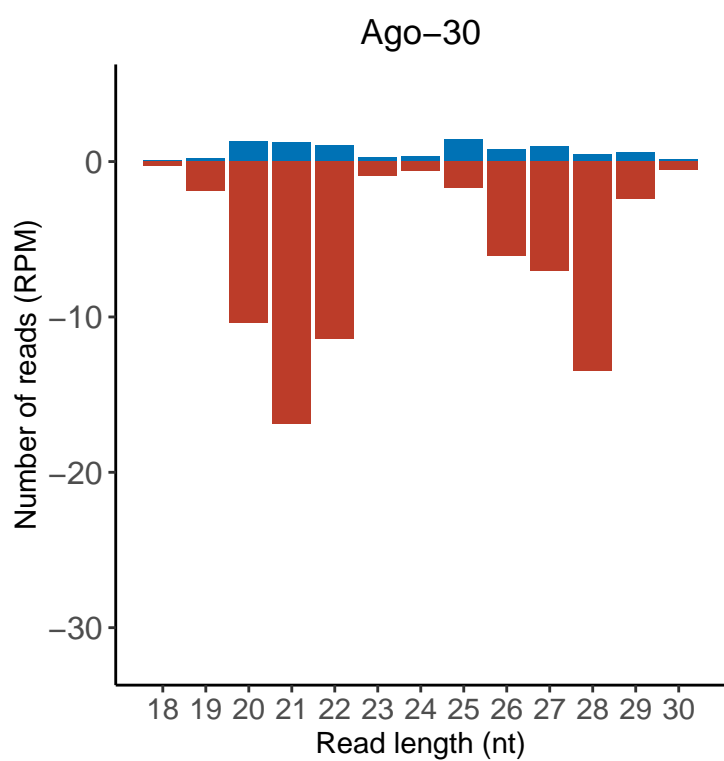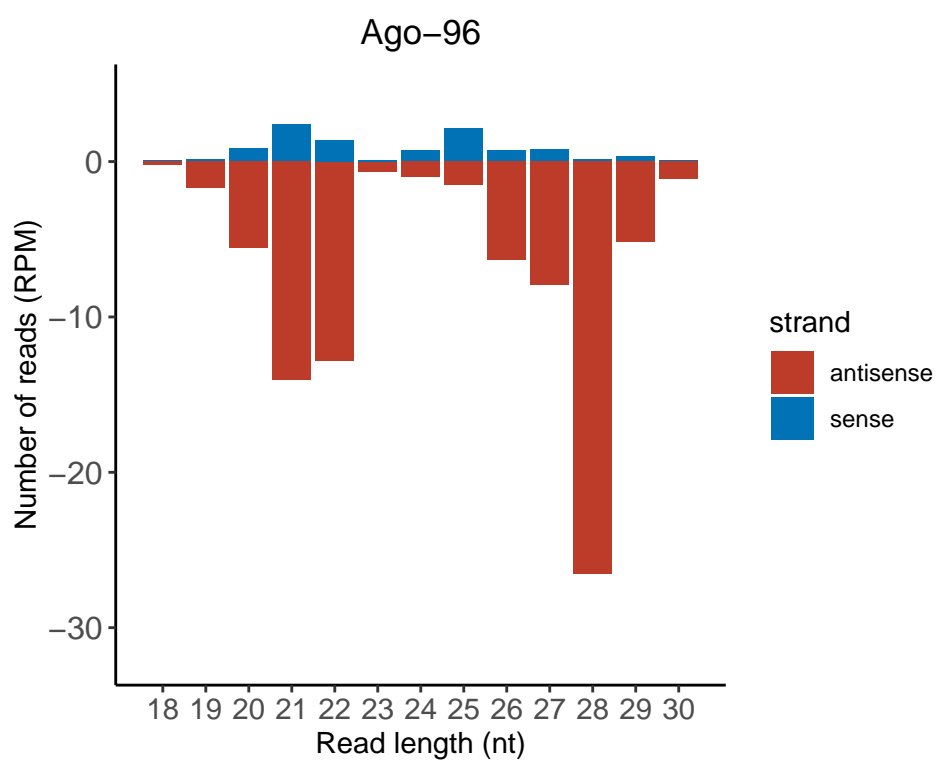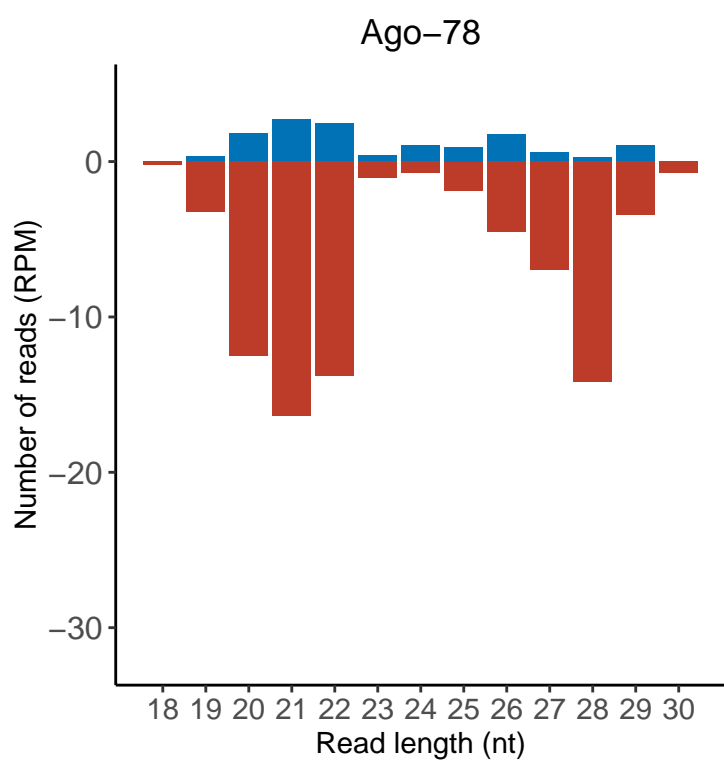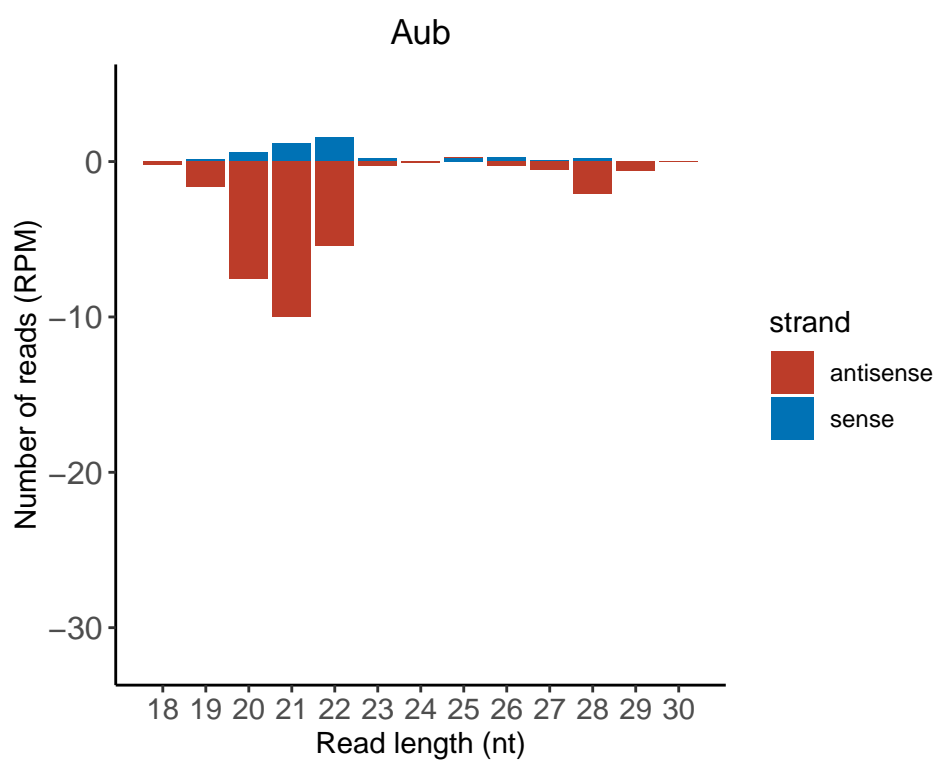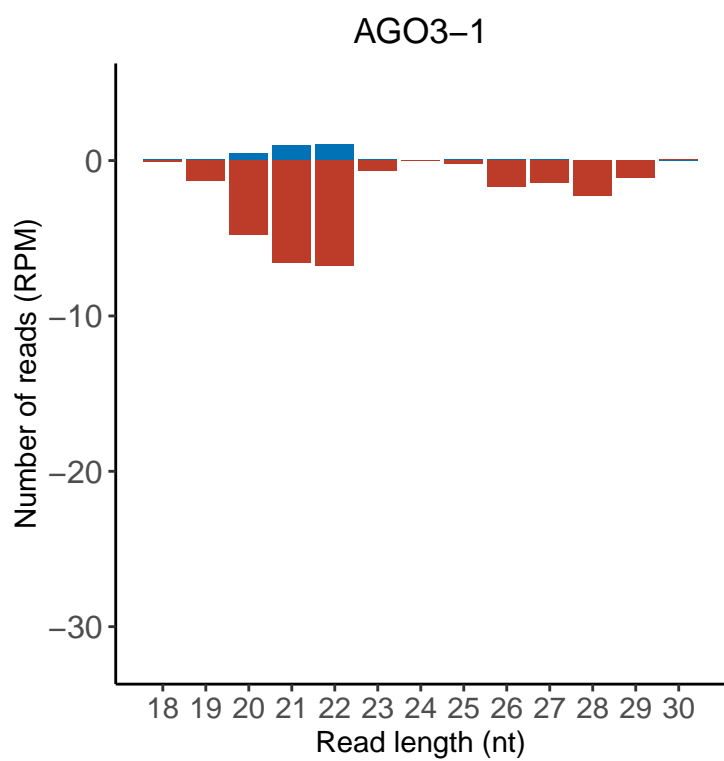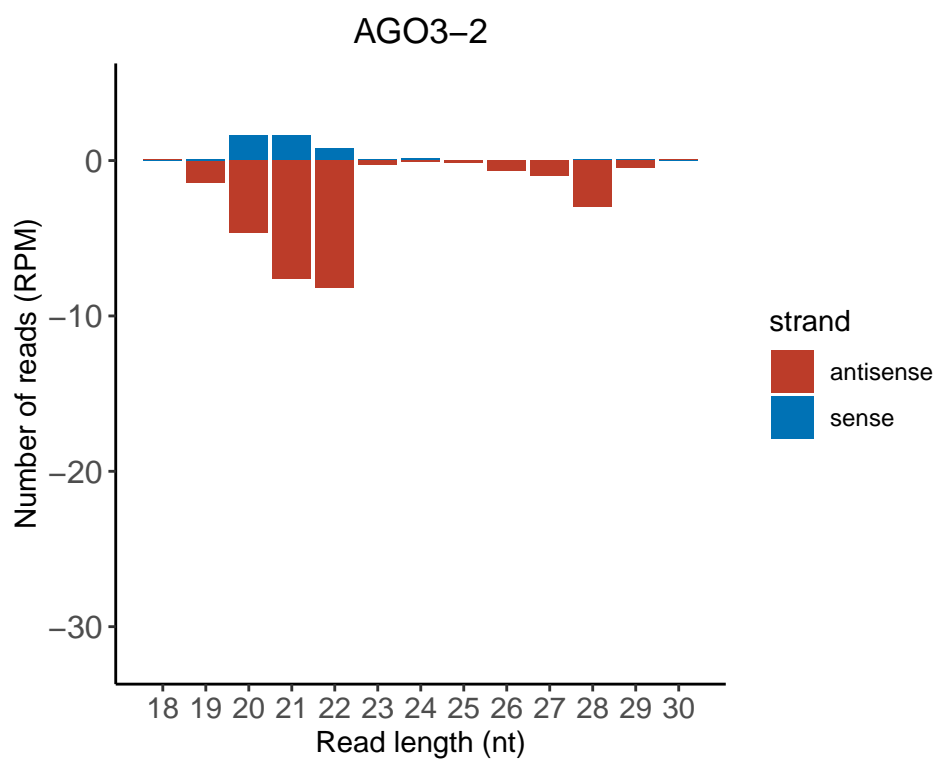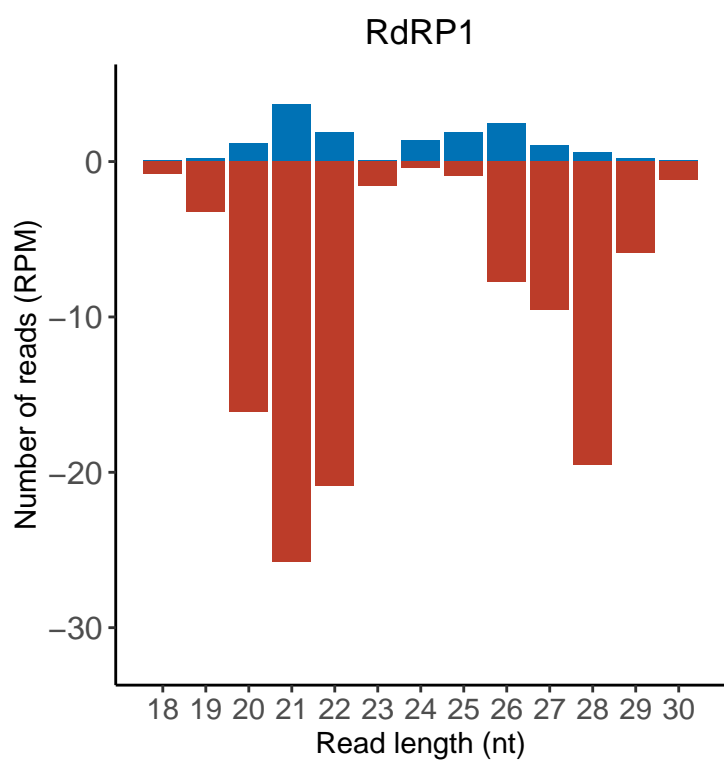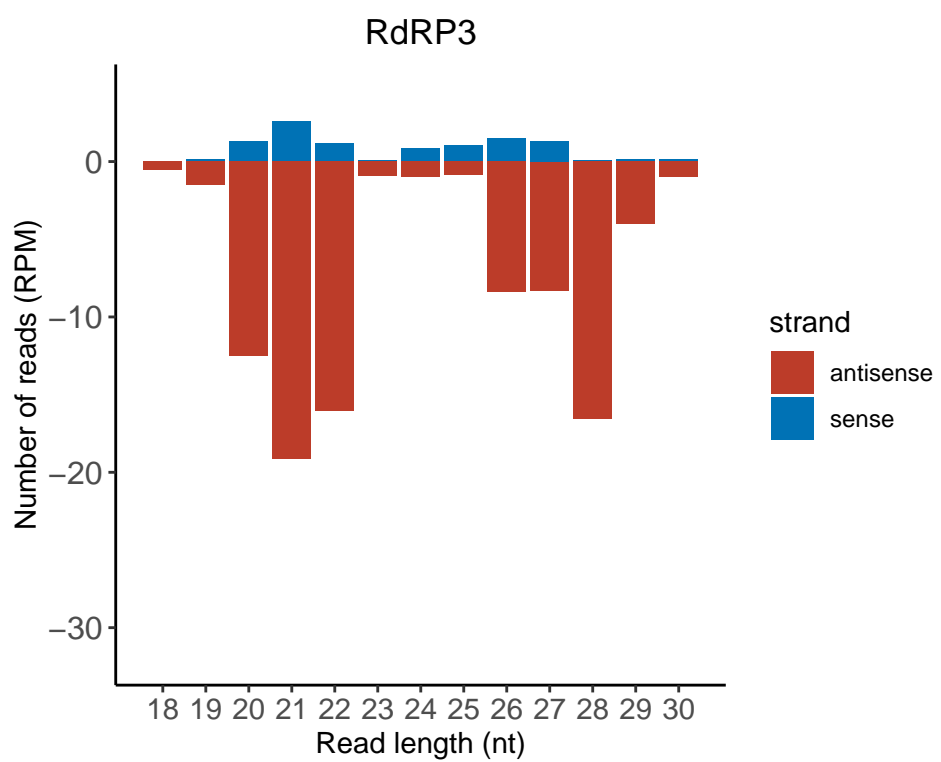

Supplement: S1 Data — On the Normalized and Relative levels pages, normalized read counts (RPM) and relative levels compared to the control (dsGFP) library were used. TEs with more than 50RPM and Coding Genes with more than 3.5RPM on average in KD libraries are included in this website. For viral sequences, reference sequences were generated by assembling sRNA sequences (See the Analysis of sRNAseq data section in Materials and methods.) and size distributions of sRNA reads mapped to each contig are shown. Reads were first grouped by their 5’ nucleotides, and reads of each length were counted. Full tables including TEs and Coding genes with fewer reads are in the “FullTable” folder. (ZIP) [file pone.0281195.s017.zip › SupplementaryData/Links/PDFs/Motif:rnd-6_family-2714TE_lengthdistribution.pdf]

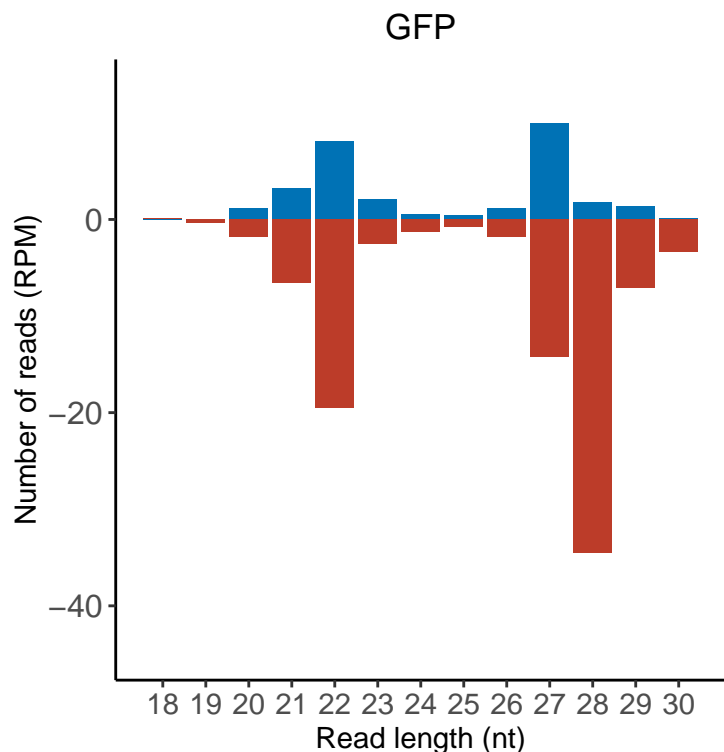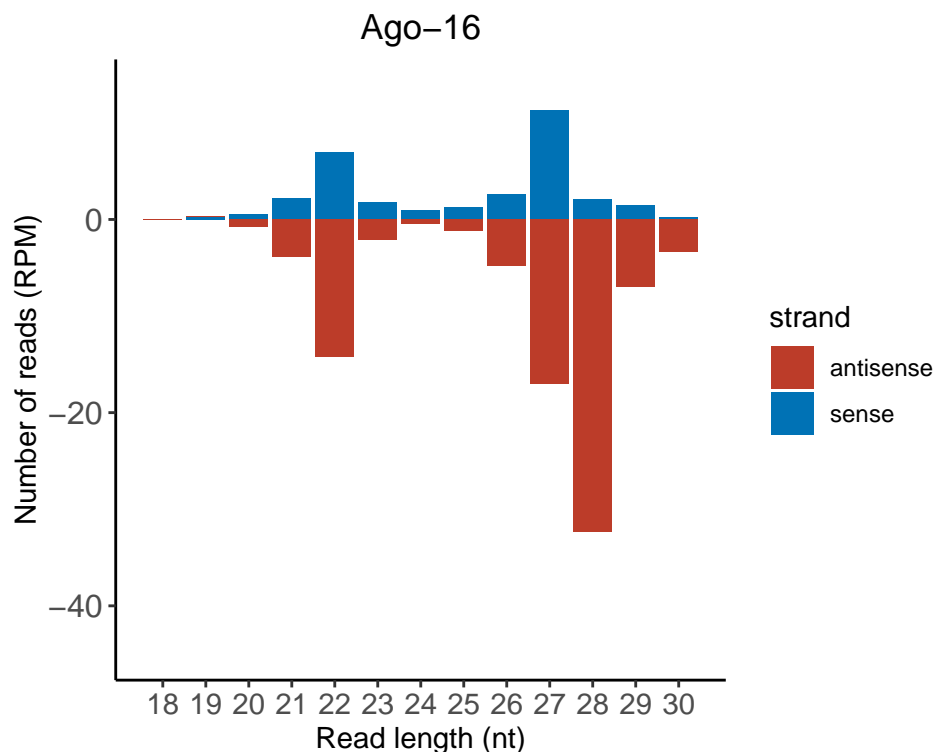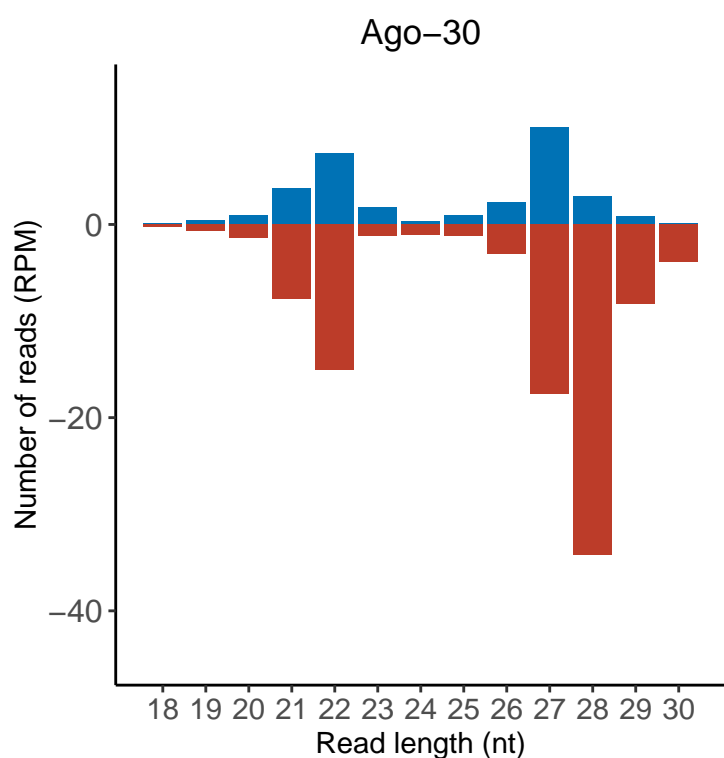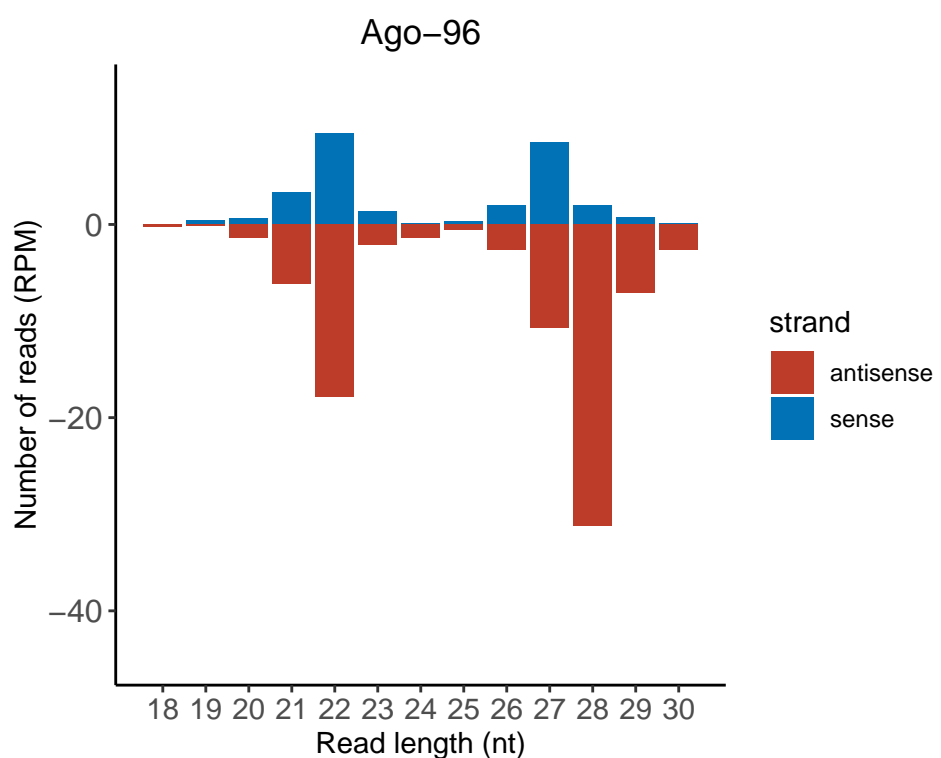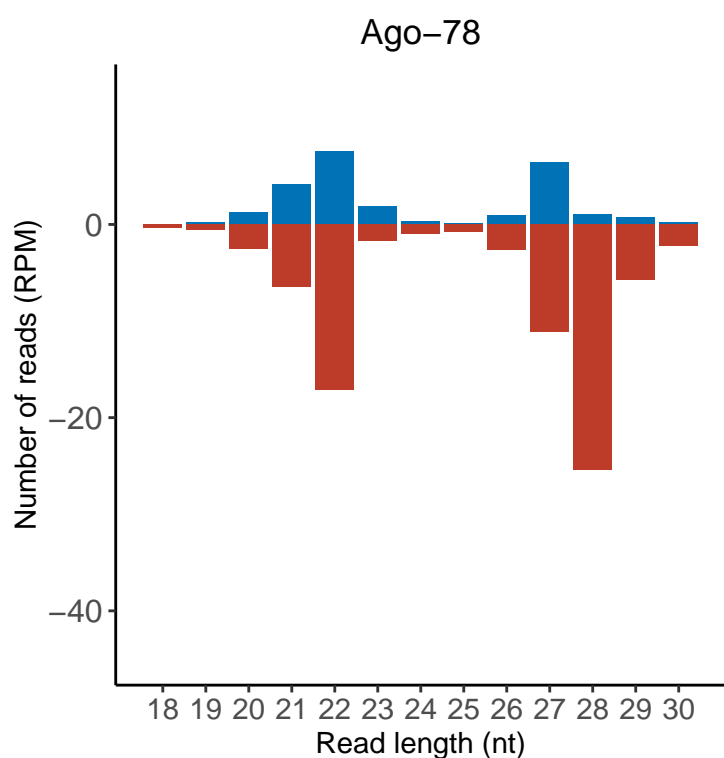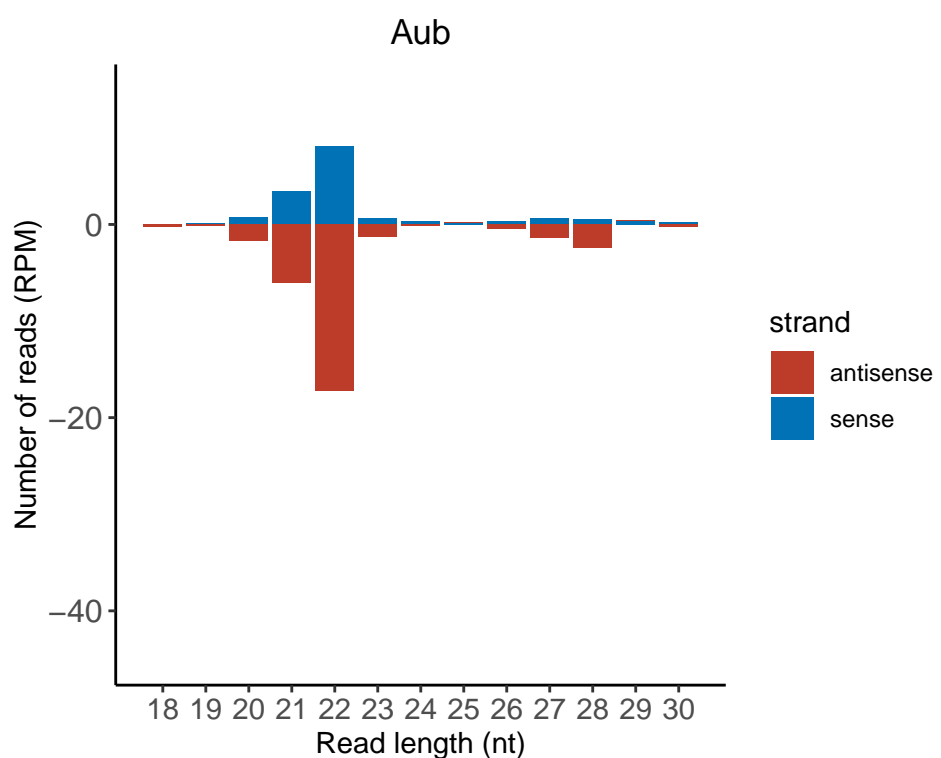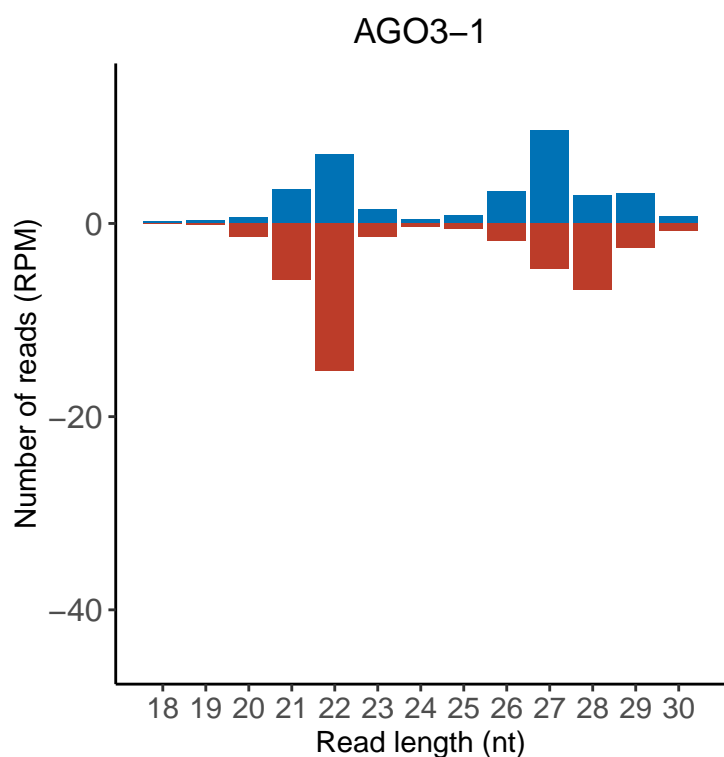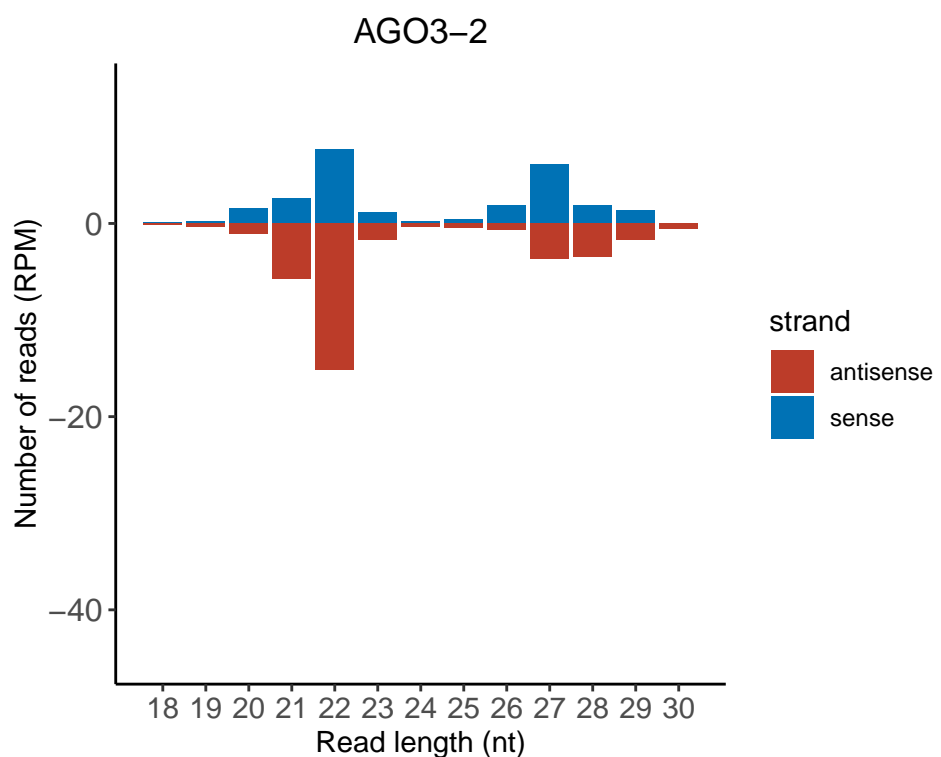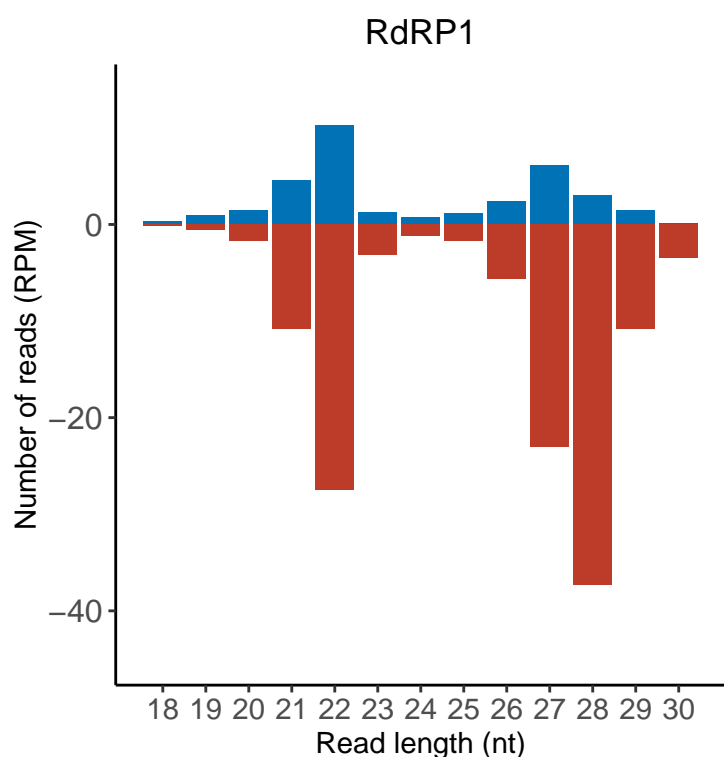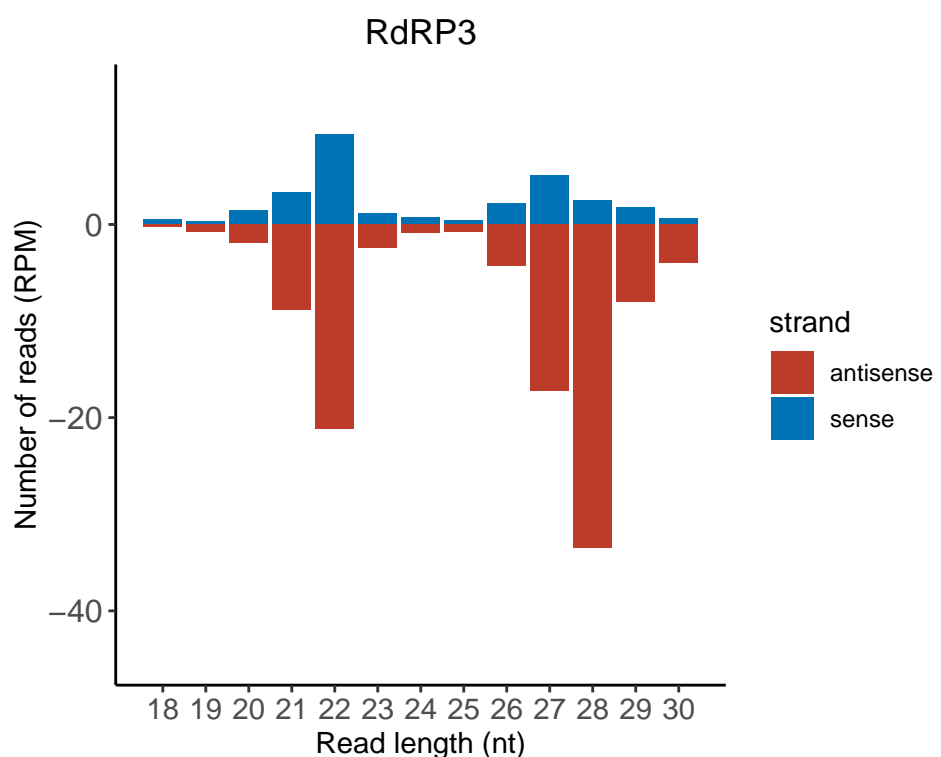

Supplement: S1 Data — On the Normalized and Relative levels pages, normalized read counts (RPM) and relative levels compared to the control (dsGFP) library were used. TEs with more than 50RPM and Coding Genes with more than 3.5RPM on average in KD libraries are included in this website. For viral sequences, reference sequences were generated by assembling sRNA sequences (See the Analysis of sRNAseq data section in Materials and methods.) and size distributions of sRNA reads mapped to each contig are shown. Reads were first grouped by their 5’ nucleotides, and reads of each length were counted. Full tables including TEs and Coding genes with fewer reads are in the “FullTable” folder. (ZIP) [file pone.0281195.s017.zip › SupplementaryData/Links/PDFs/Motif:rnd-1_family-603TE_lengthdistribution.pdf]

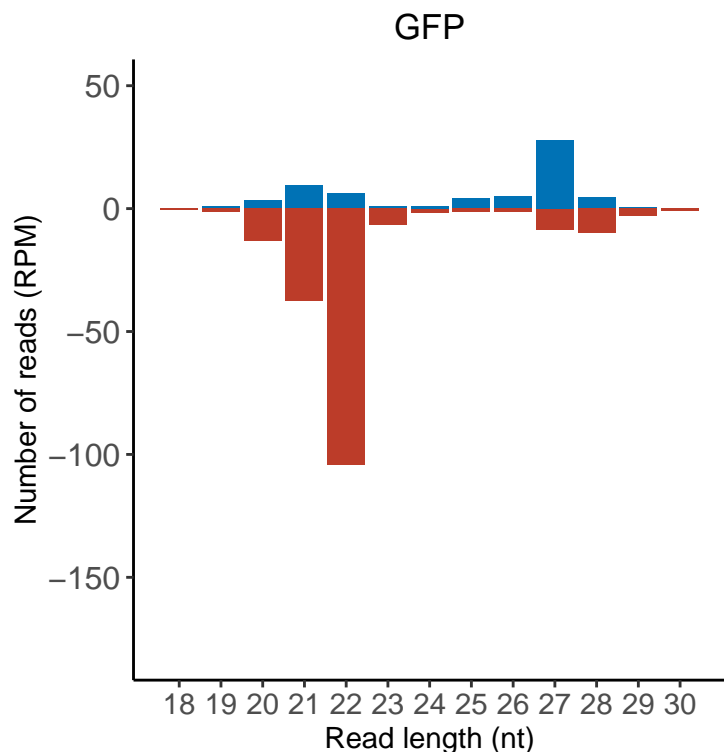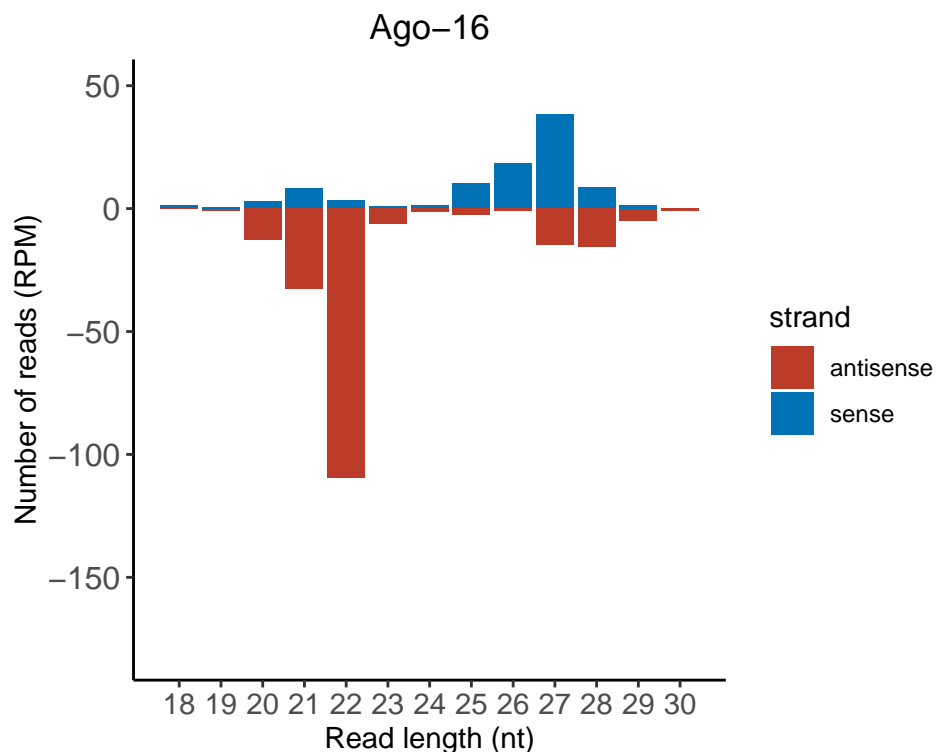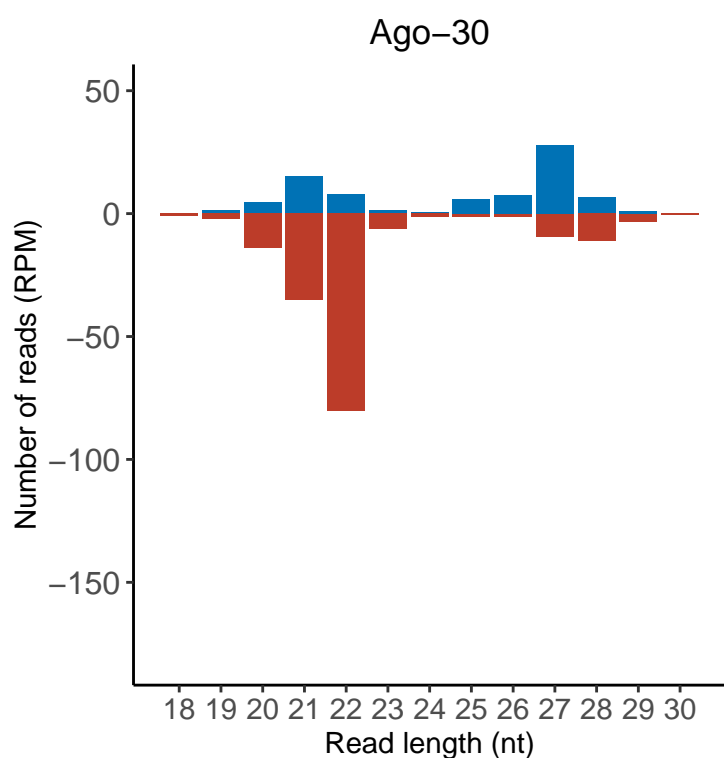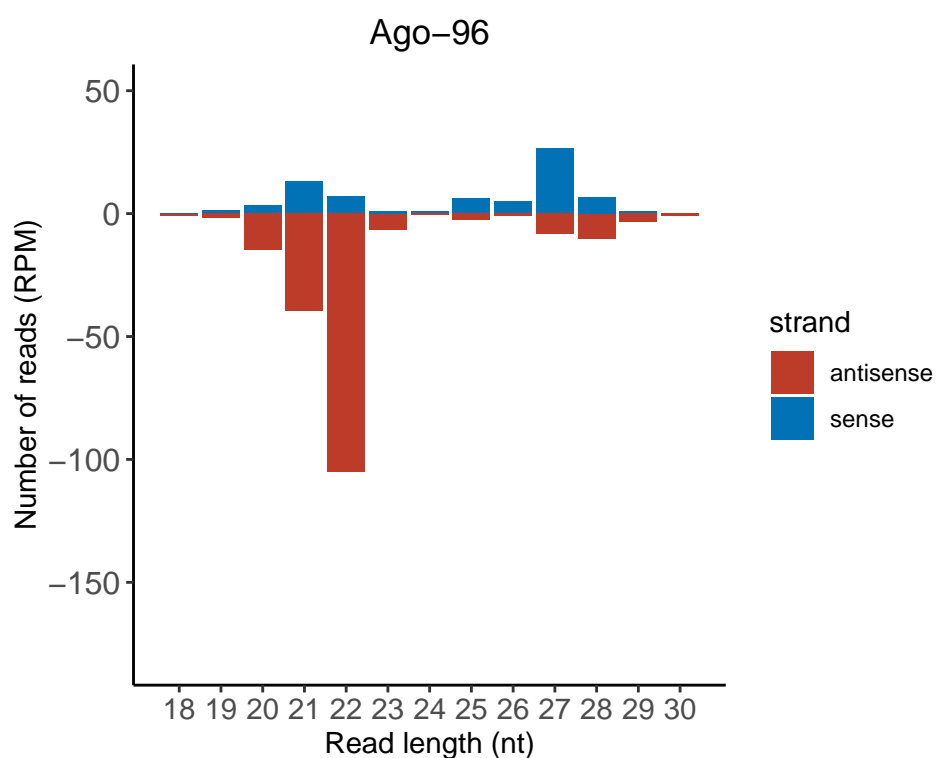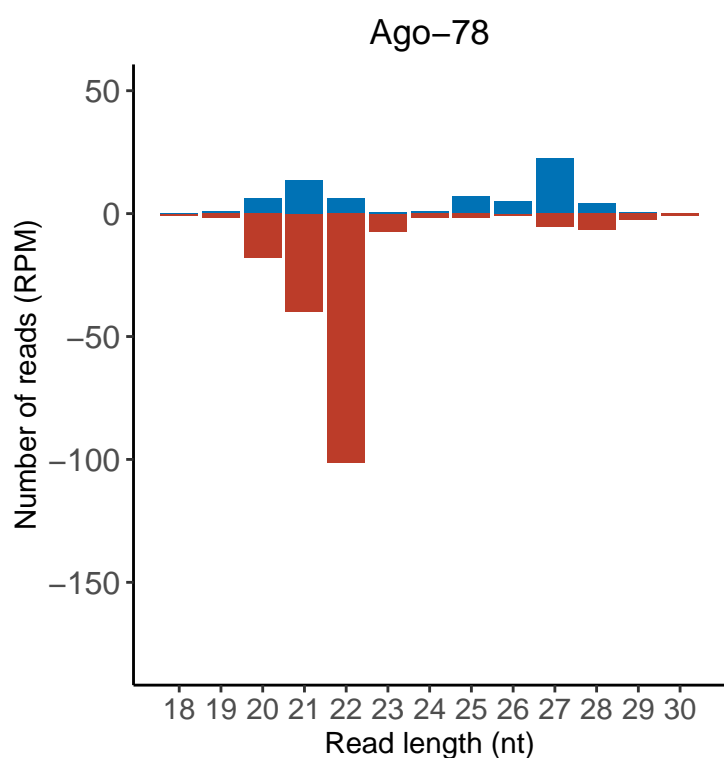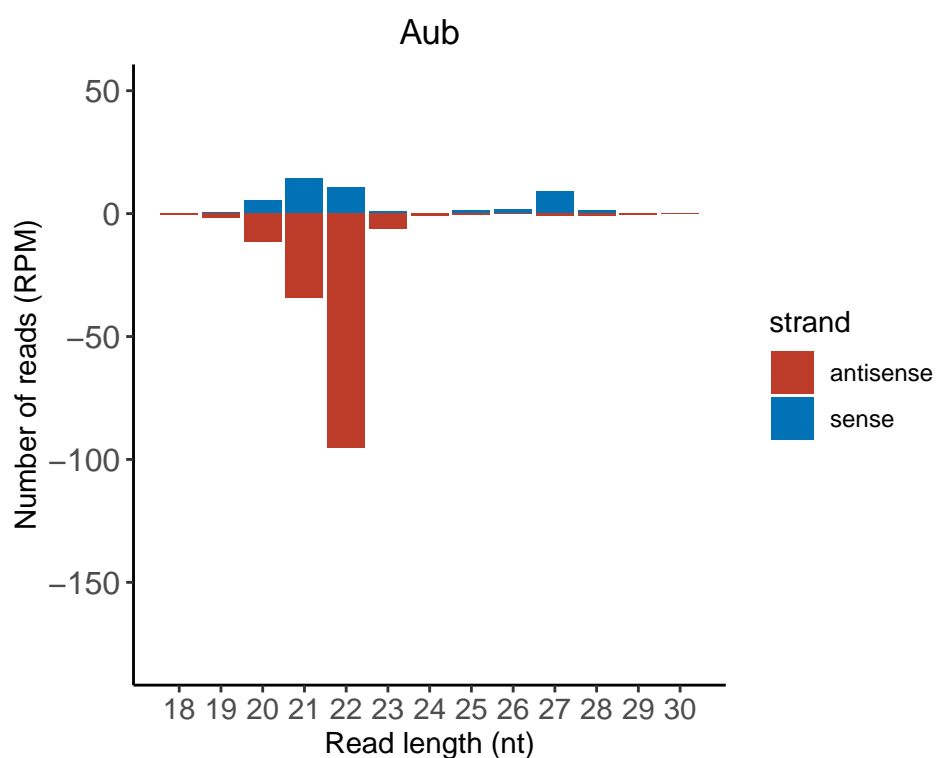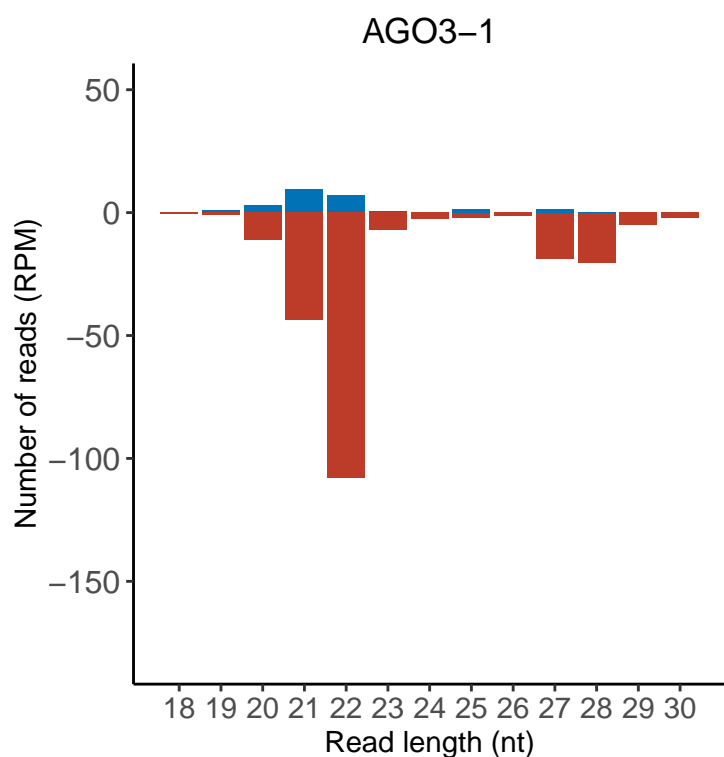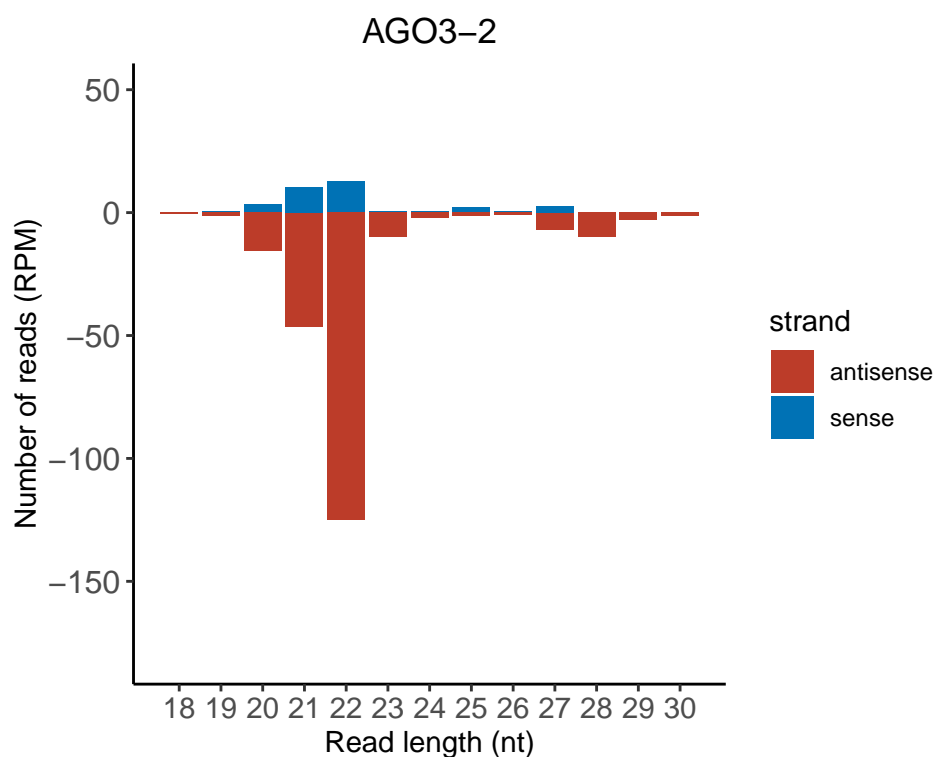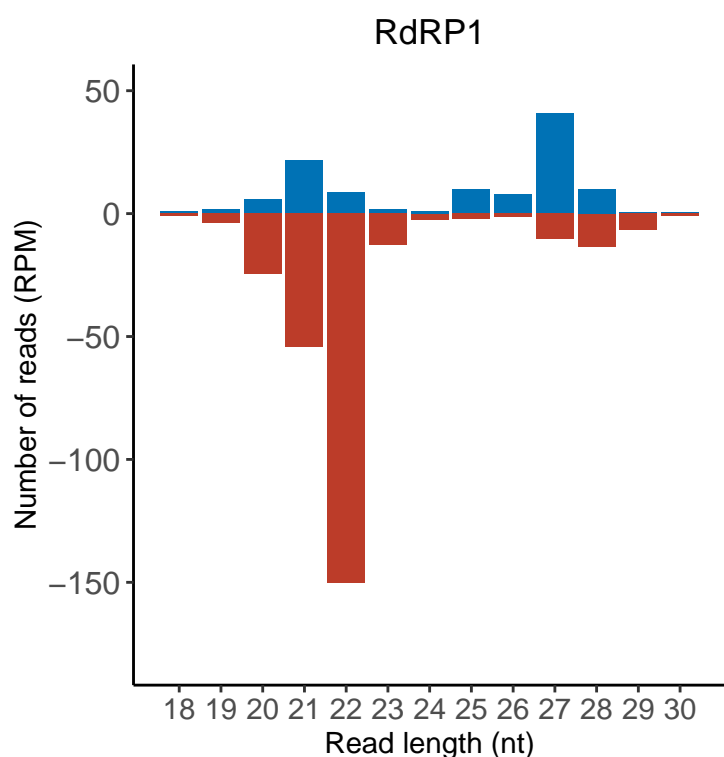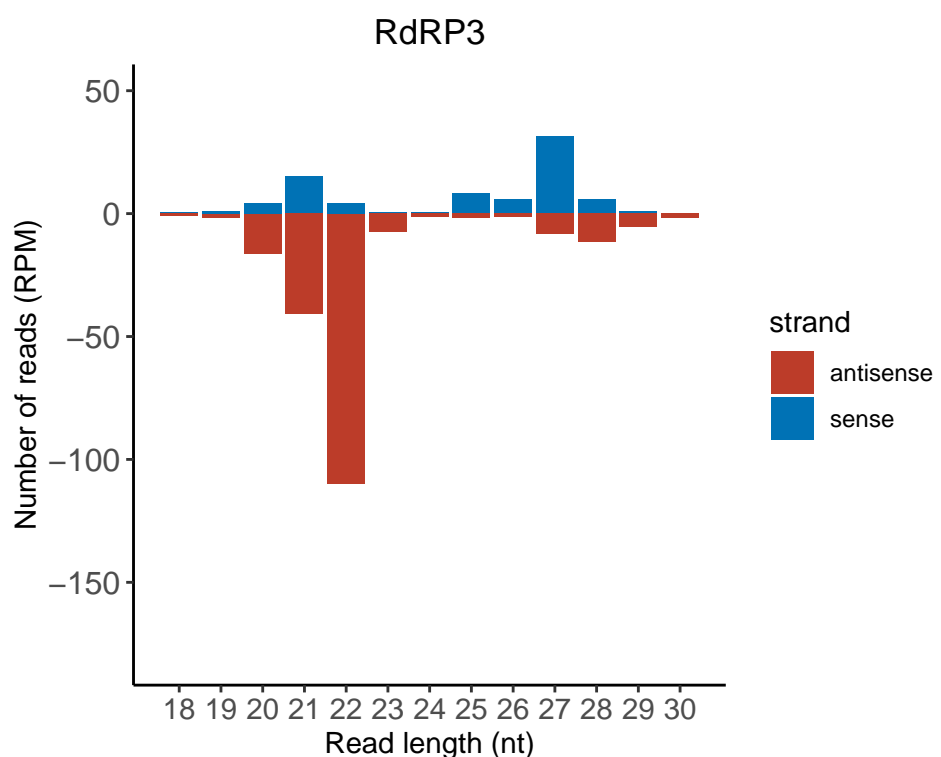

Supplement: S1 Data — On the Normalized and Relative levels pages, normalized read counts (RPM) and relative levels compared to the control (dsGFP) library were used. TEs with more than 50RPM and Coding Genes with more than 3.5RPM on average in KD libraries are included in this website. For viral sequences, reference sequences were generated by assembling sRNA sequences (See the Analysis of sRNAseq data section in Materials and methods.) and size distributions of sRNA reads mapped to each contig are shown. Reads were first grouped by their 5’ nucleotides, and reads of each length were counted. Full tables including TEs and Coding genes with fewer reads are in the “FullTable” folder. (ZIP) [file pone.0281195.s017.zip › SupplementaryData/Links/PDFs/Motif:rnd-6_family-12152TE_lengthdistribution.pdf]

GFP

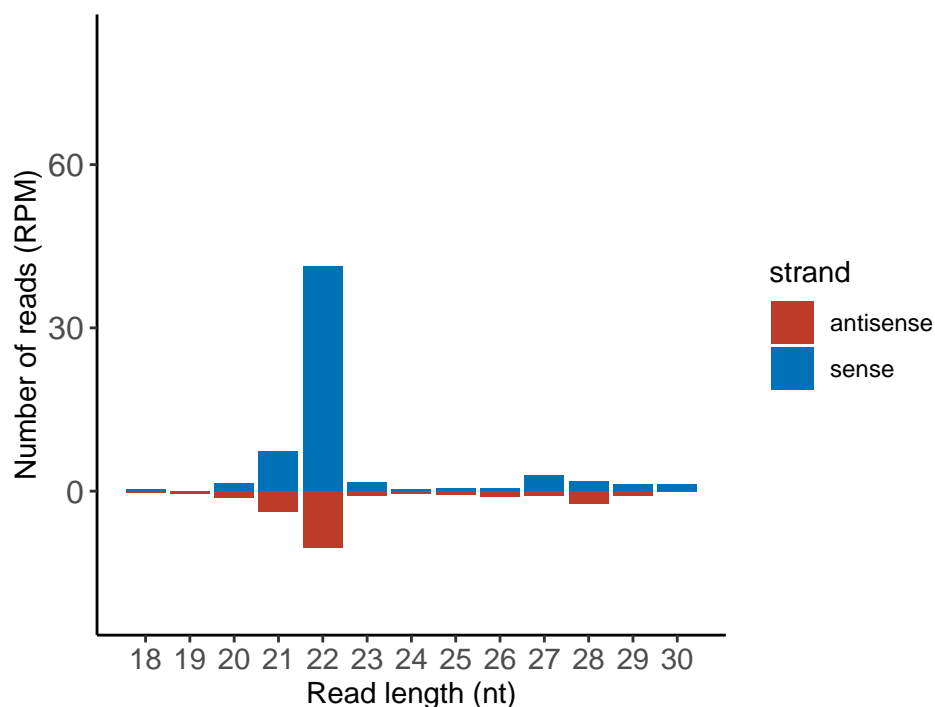

Ago-16

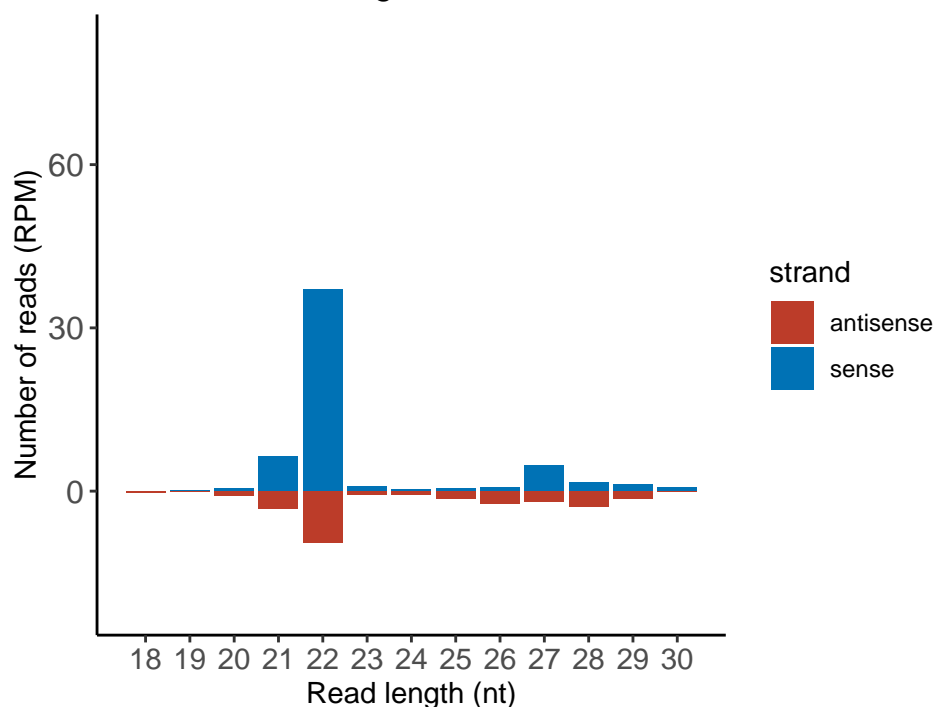

Ago-30

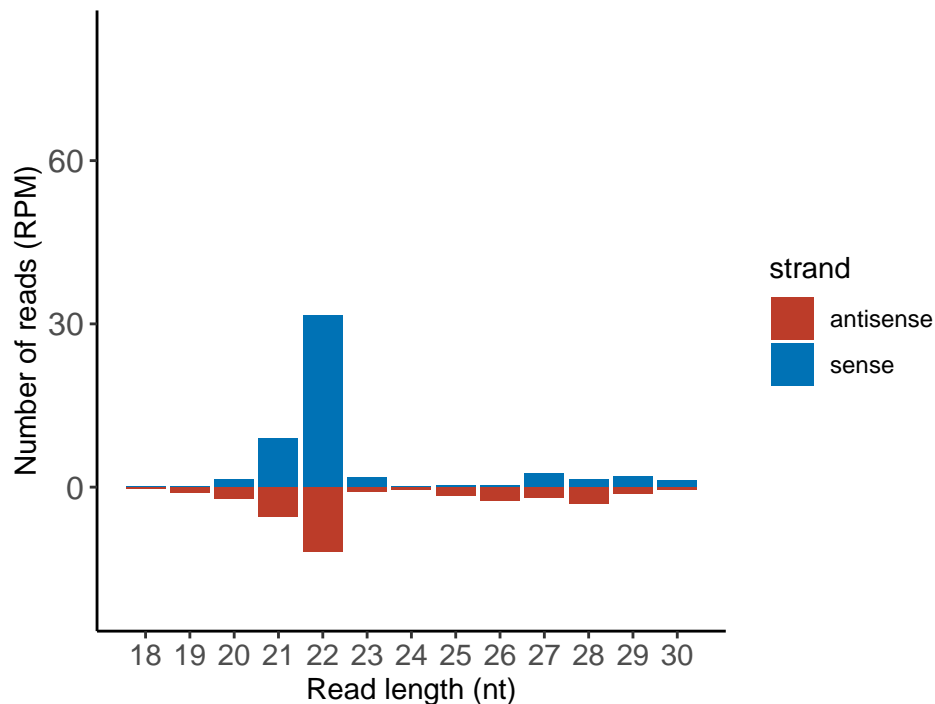

Ago-96

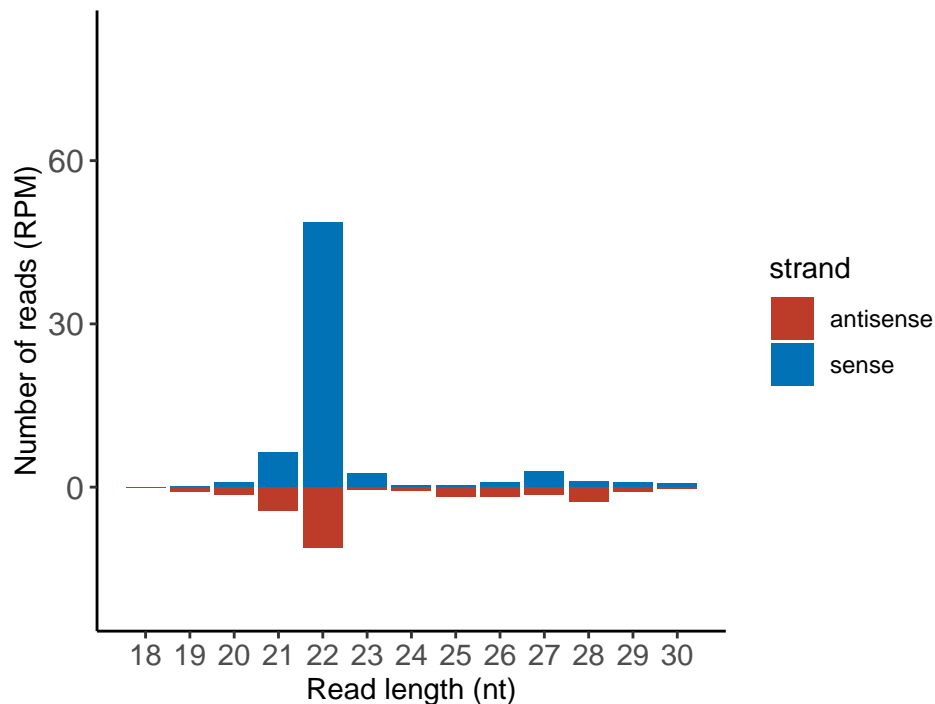

Ago-78

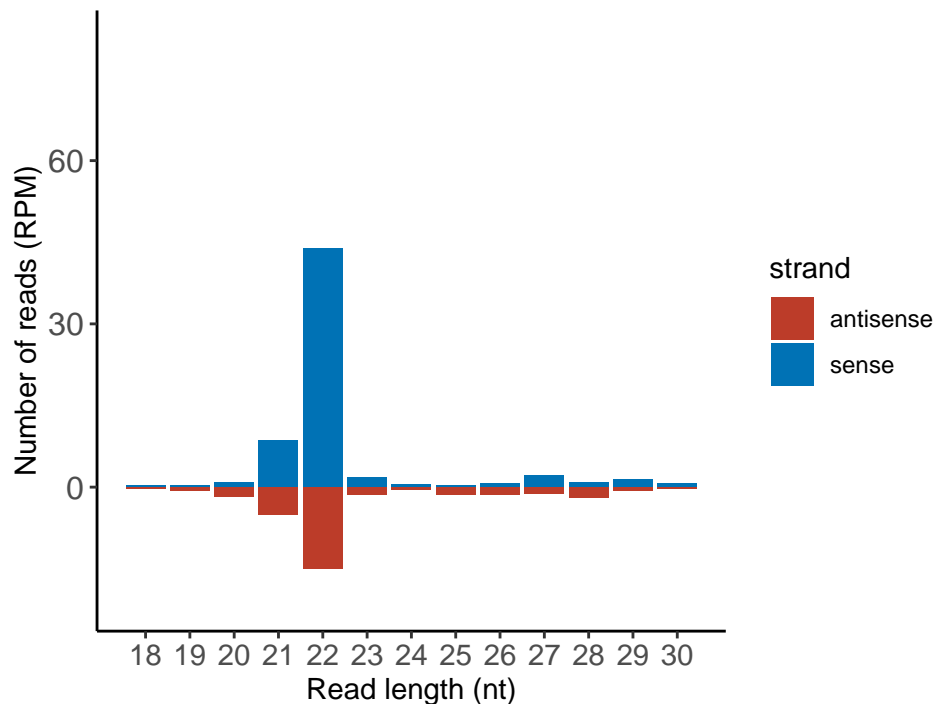

Aub

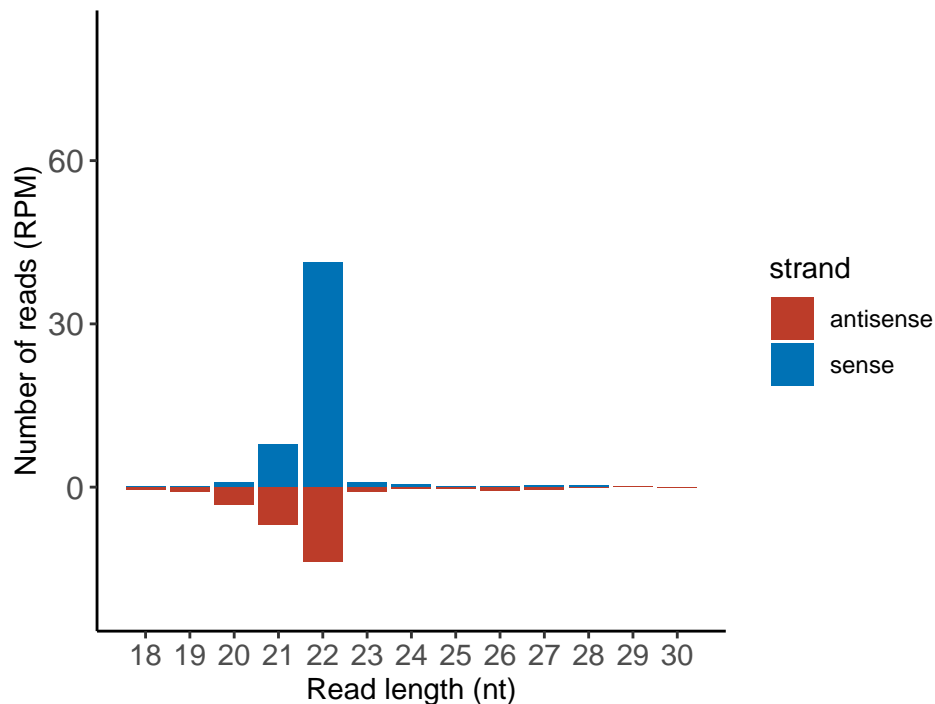

AGO3-1

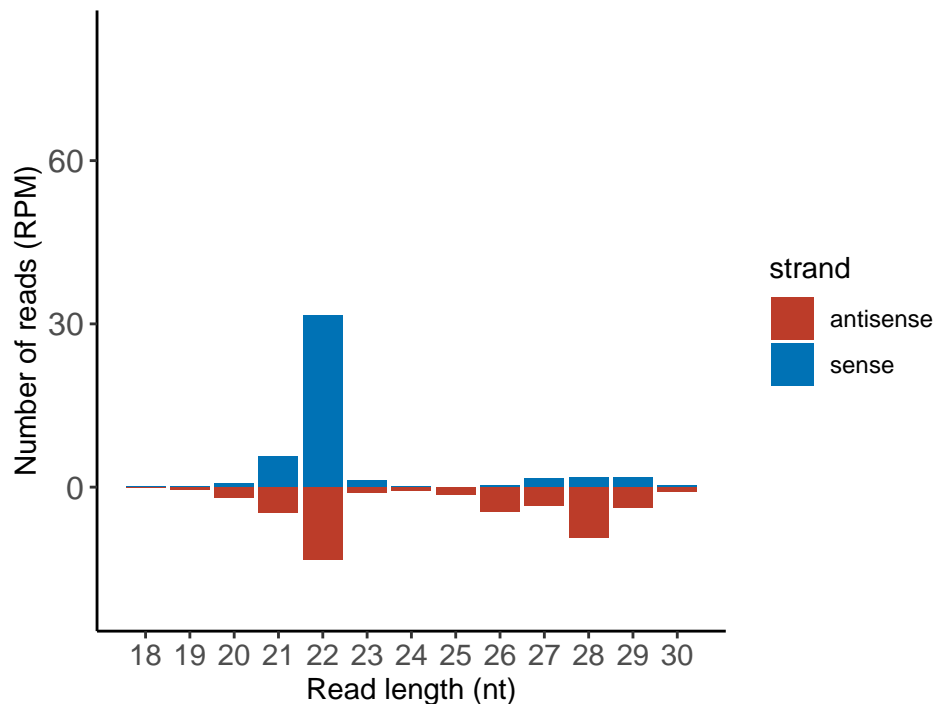

AGO3-2

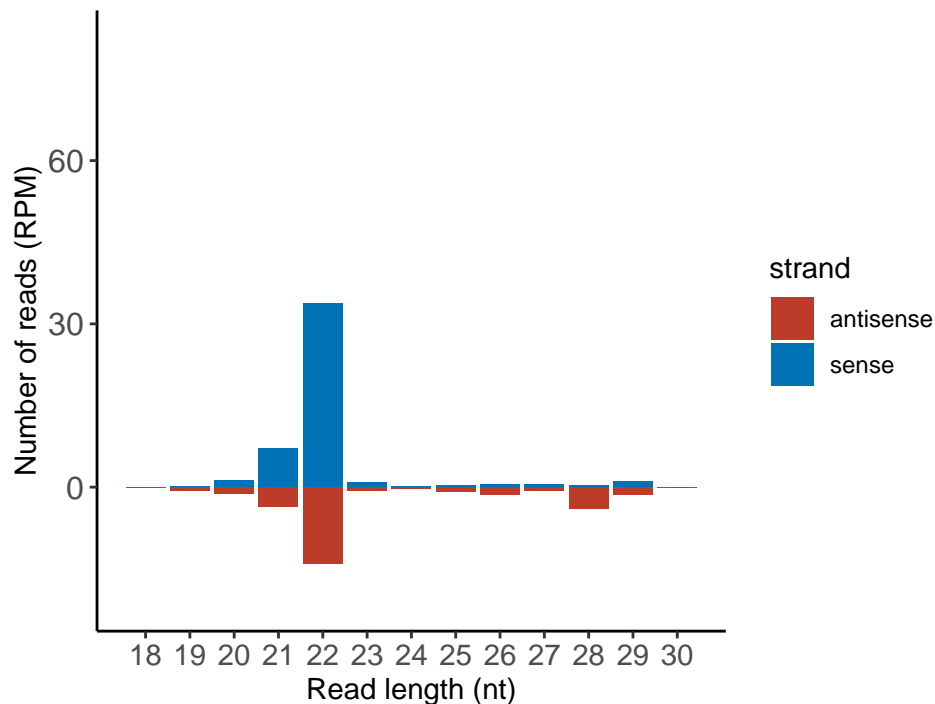

RdRP1

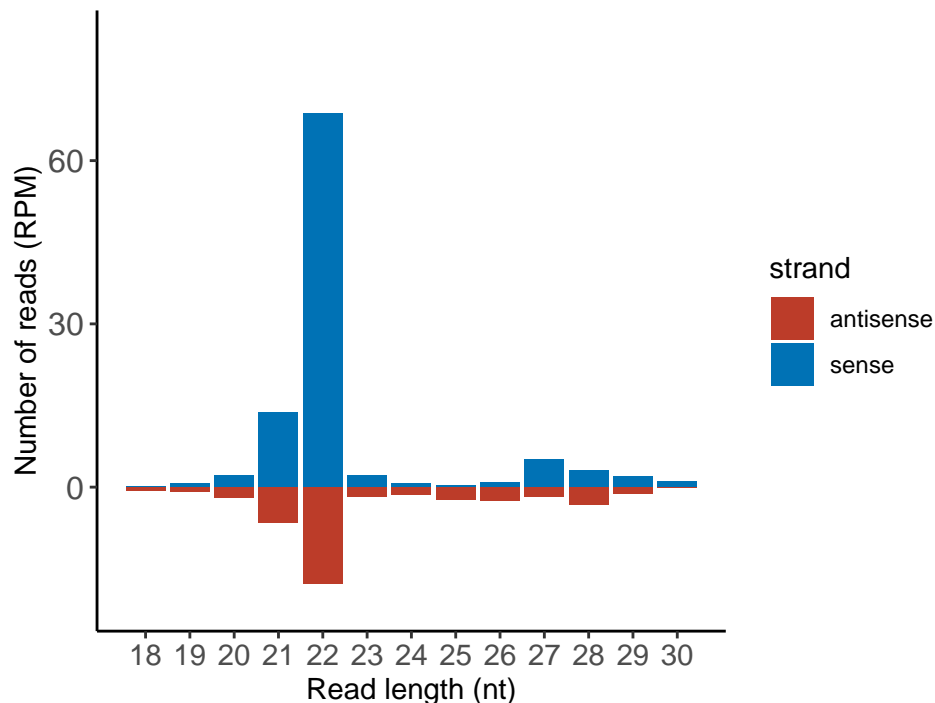

RdRP3

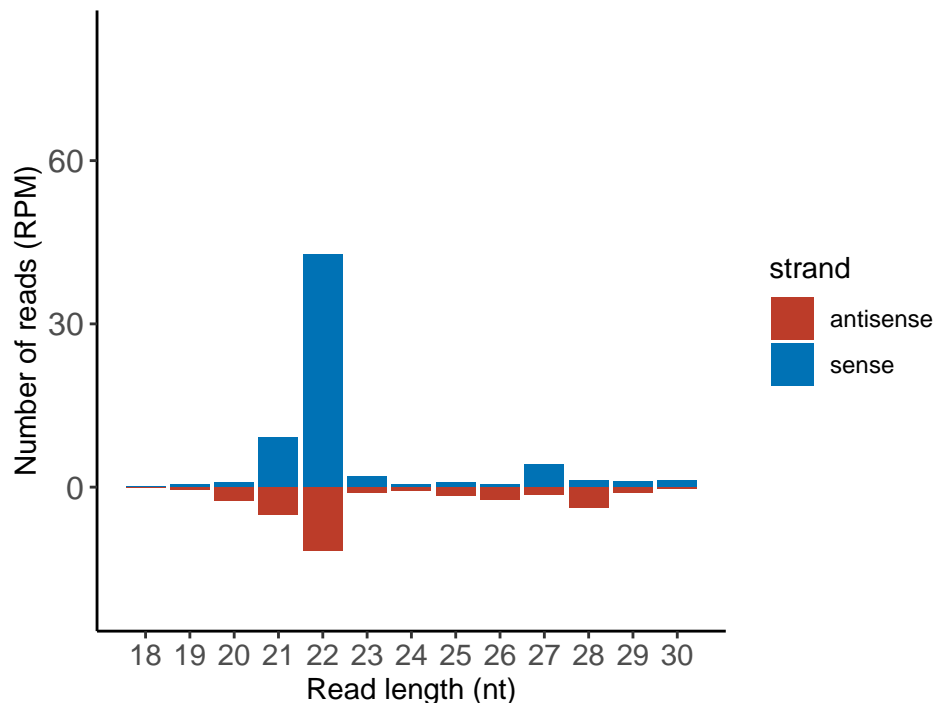

Supplement: S1 Data — On the Normalized and Relative levels pages, normalized read counts (RPM) and relative levels compared to the control (dsGFP) library were used. TEs with more than 50RPM and Coding Genes with more than 3.5RPM on average in KD libraries are included in this website. For viral sequences, reference sequences were generated by assembling sRNA sequences (See the Analysis of sRNAseq data section in Materials and methods.) and size distributions of sRNA reads mapped to each contig are shown. Reads were first grouped by their 5’ nucleotides, and reads of each length were counted. Full tables including TEs and Coding genes with fewer reads are in the “FullTable” folder. (ZIP) [file pone.0281195.s017.zip › SupplementaryData/Links/PDFs/Motif:rnd-5_family-4863TE_lengthdistribution.pdf]

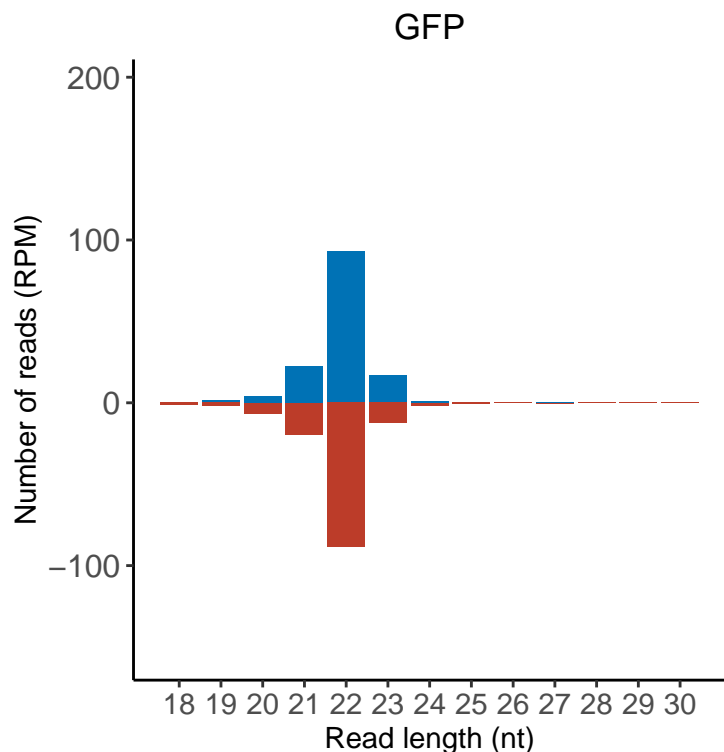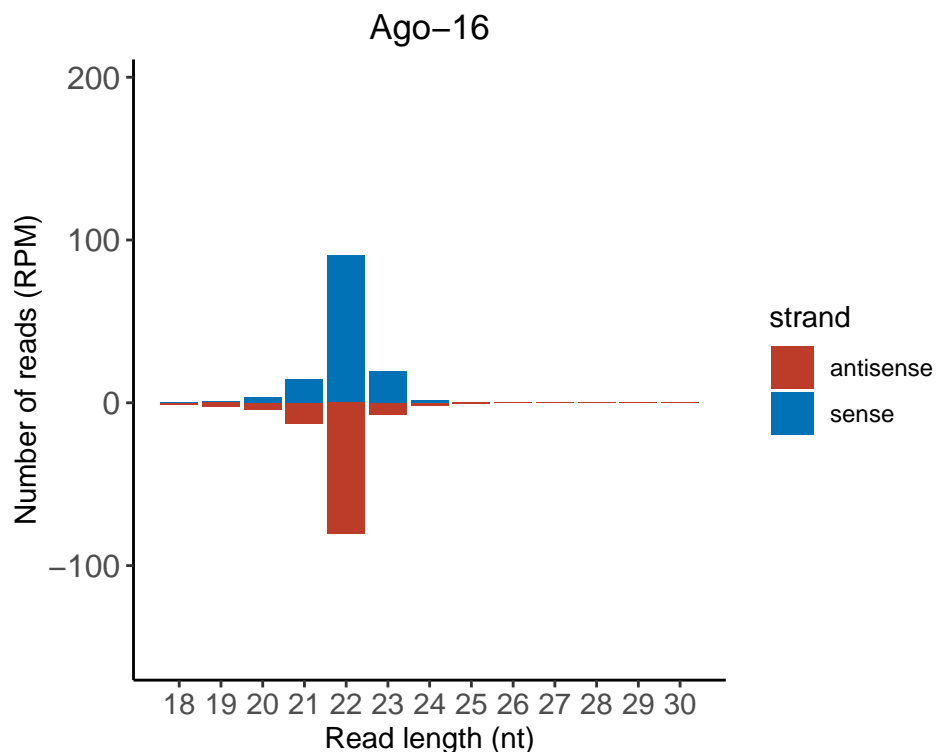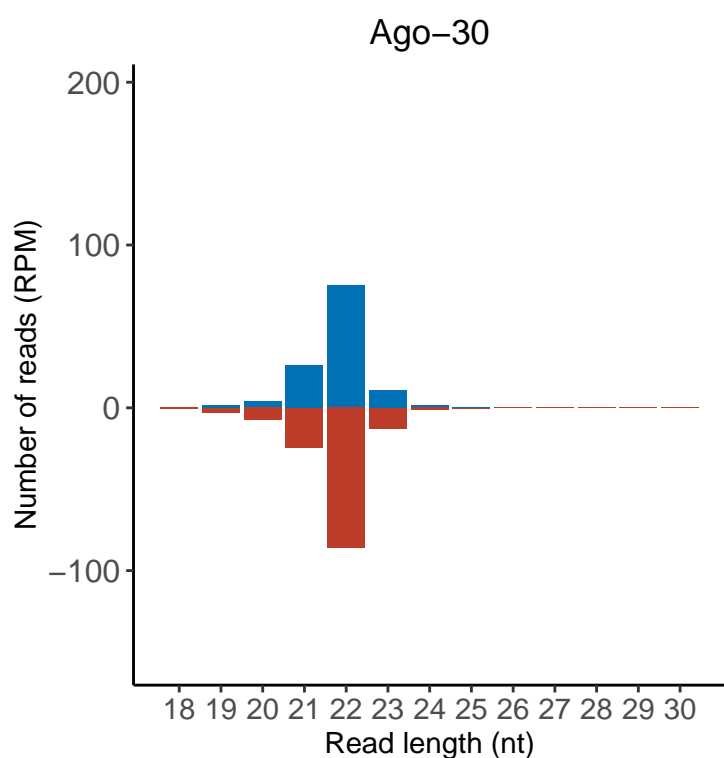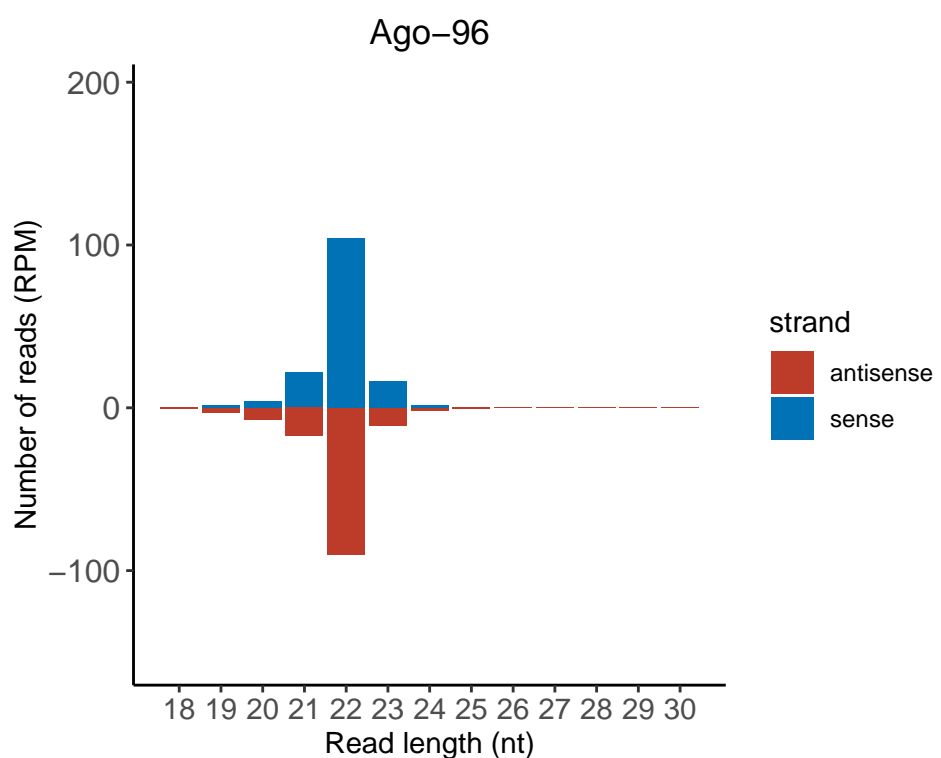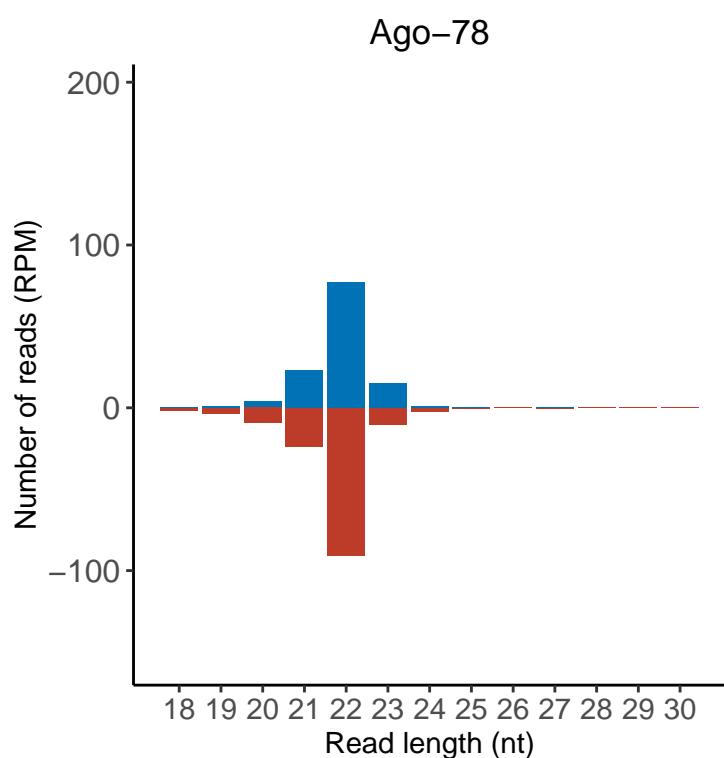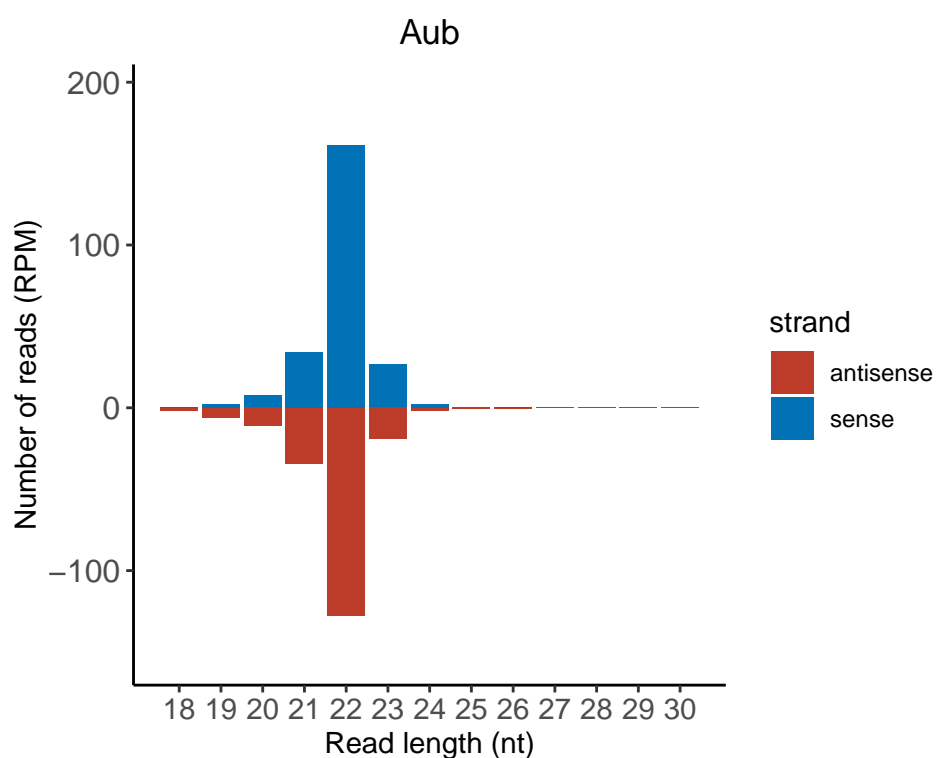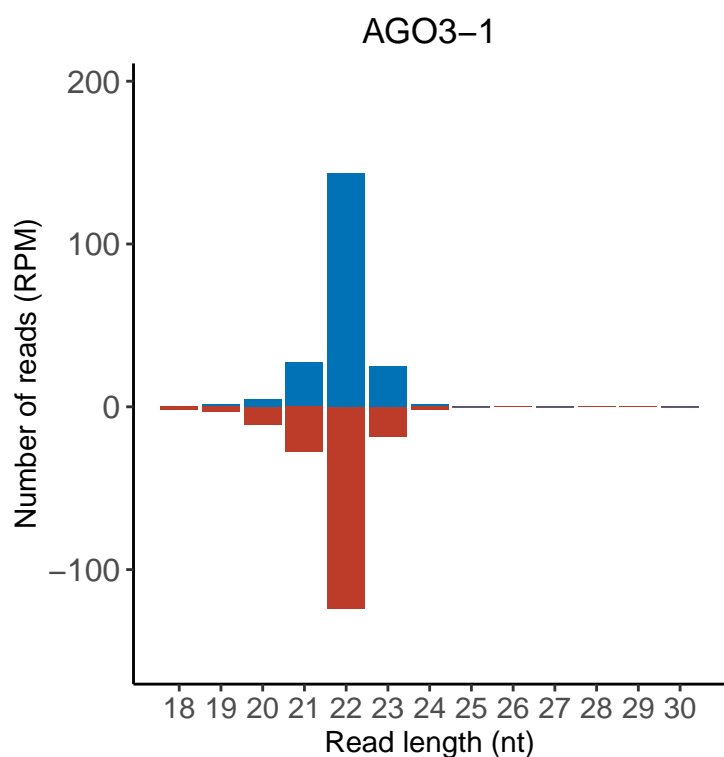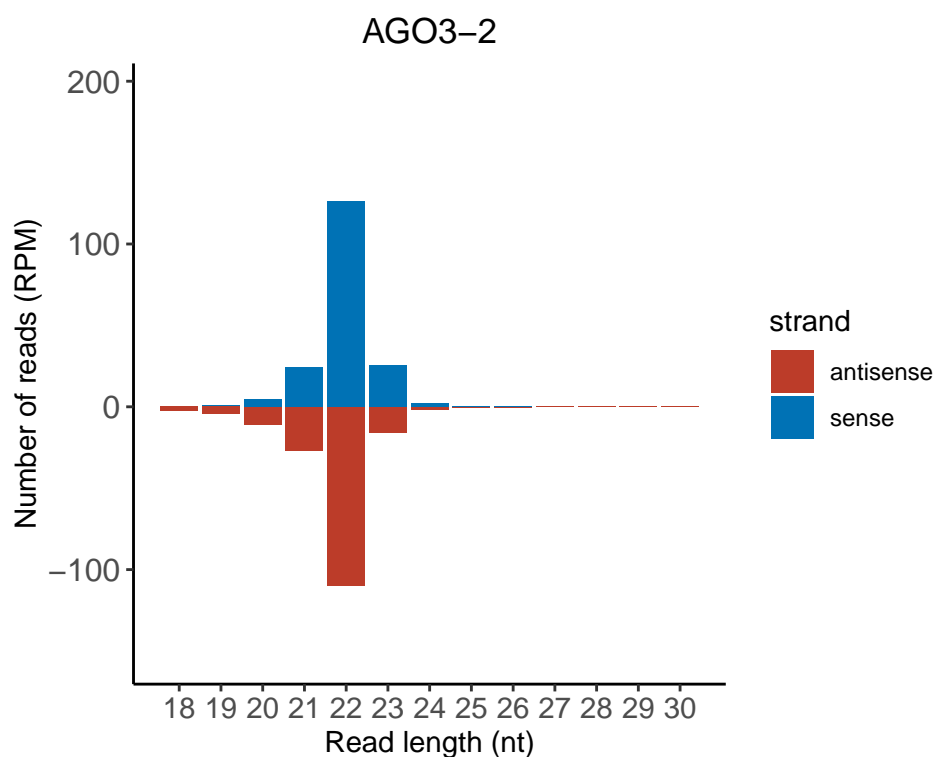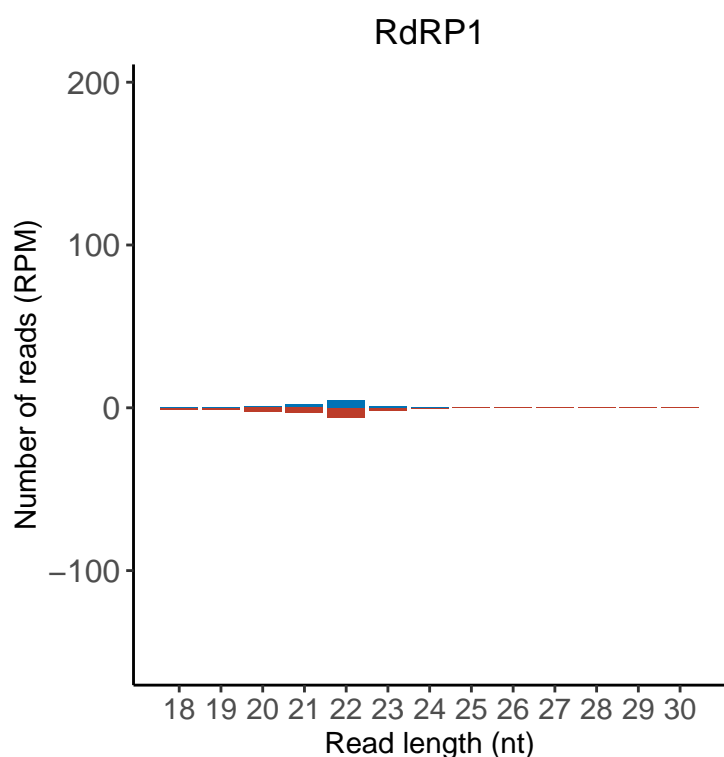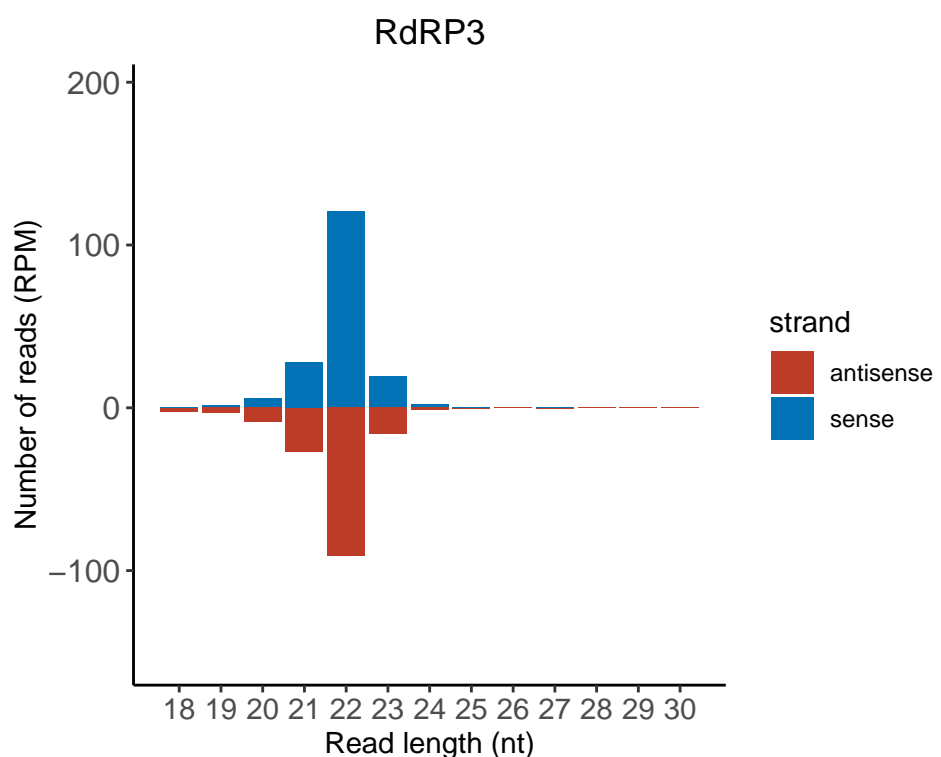

Supplement: S1 Data — On the Normalized and Relative levels pages, normalized read counts (RPM) and relative levels compared to the control (dsGFP) library were used. TEs with more than 50RPM and Coding Genes with more than 3.5RPM on average in KD libraries are included in this website. For viral sequences, reference sequences were generated by assembling sRNA sequences (See the Analysis of sRNAseq data section in Materials and methods.) and size distributions of sRNA reads mapped to each contig are shown. Reads were first grouped by their 5’ nucleotides, and reads of each length were counted. Full tables including TEs and Coding genes with fewer reads are in the “FullTable” folder. (ZIP) [file pone.0281195.s017.zip › SupplementaryData/Links/PDFs/Motif:rnd-5_family-1090TE_lengthdistribution.pdf]

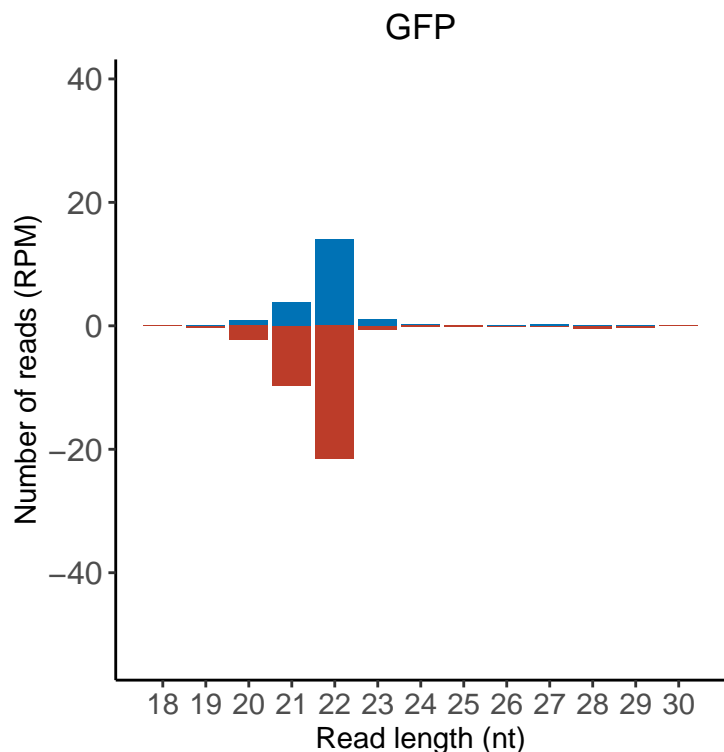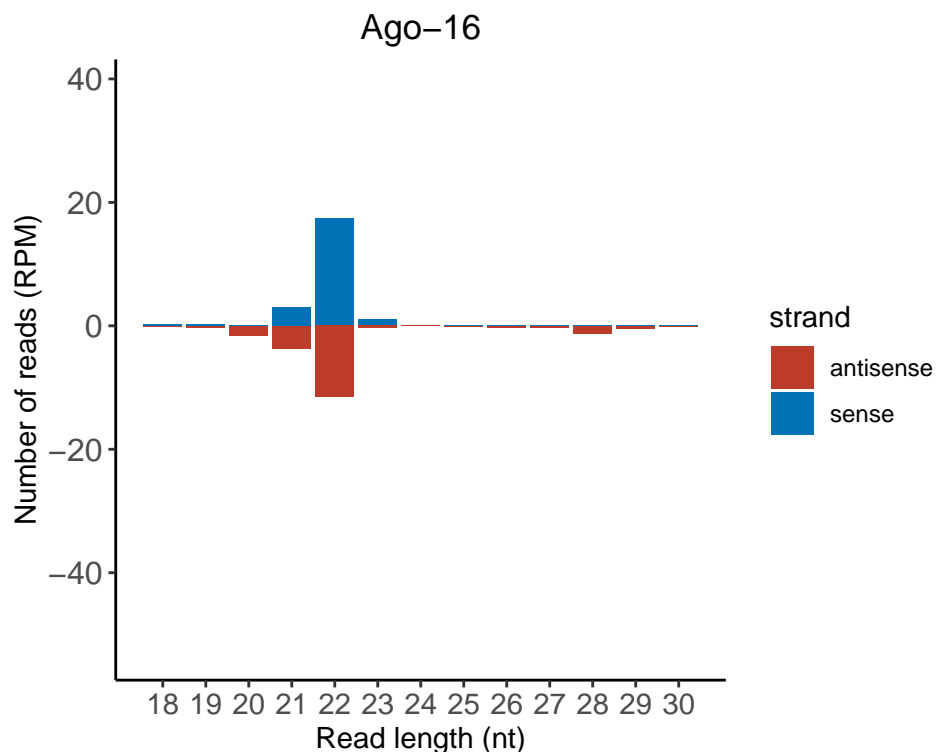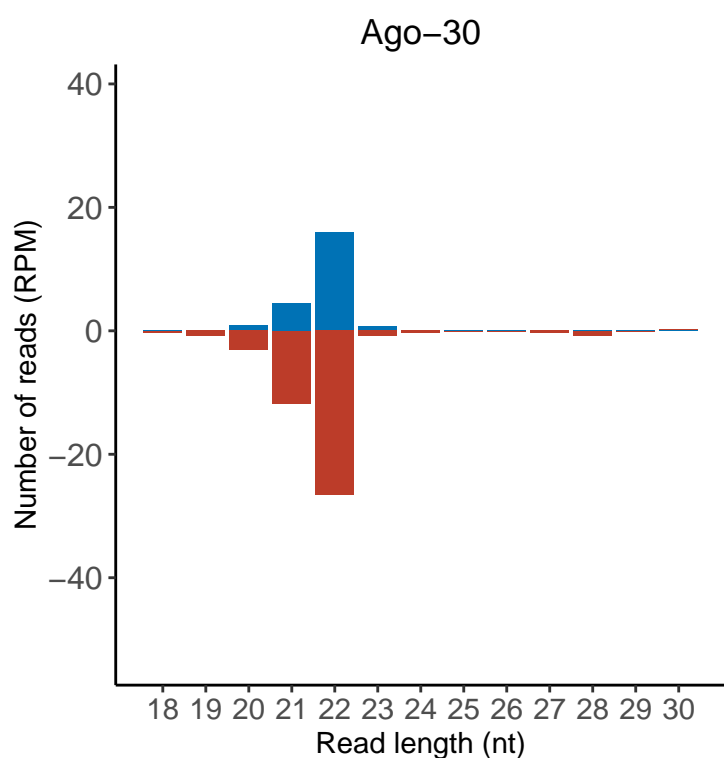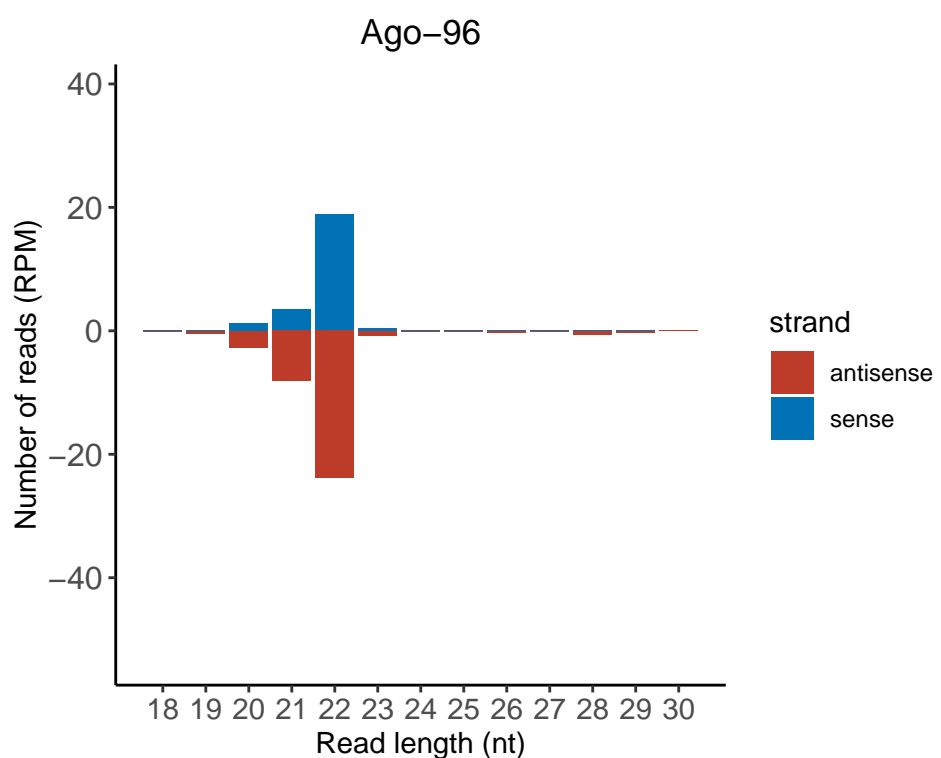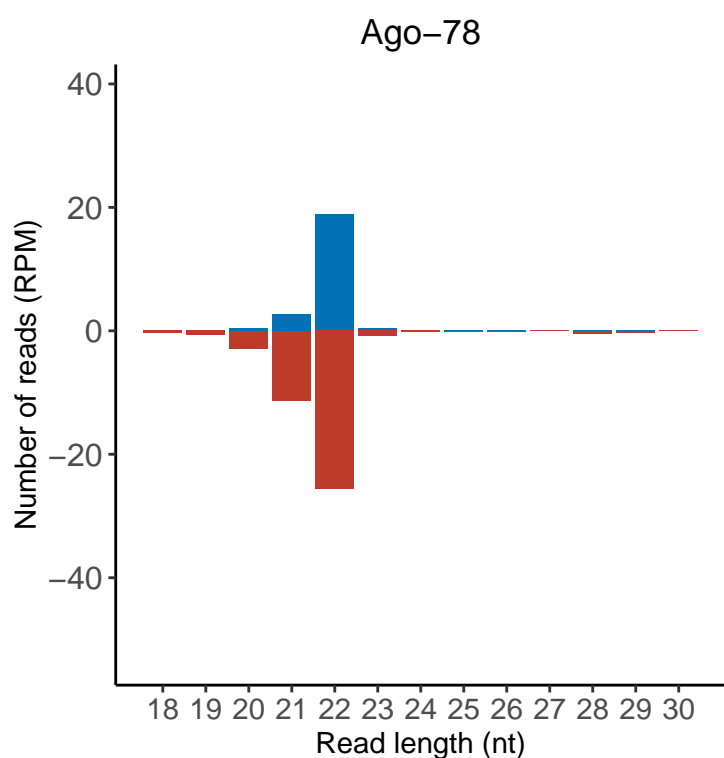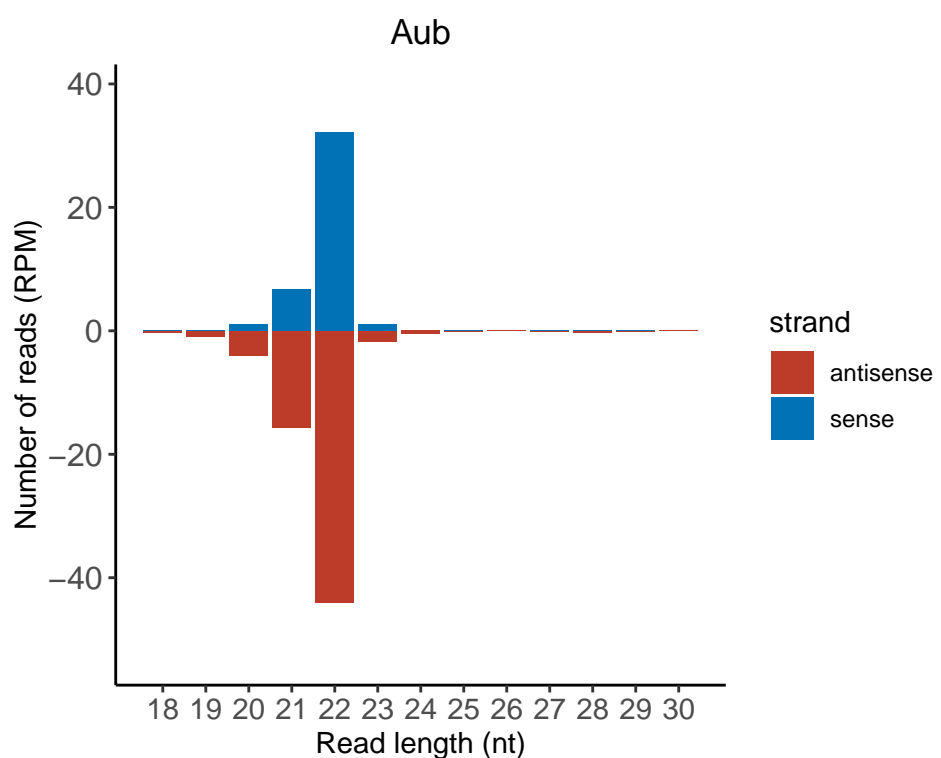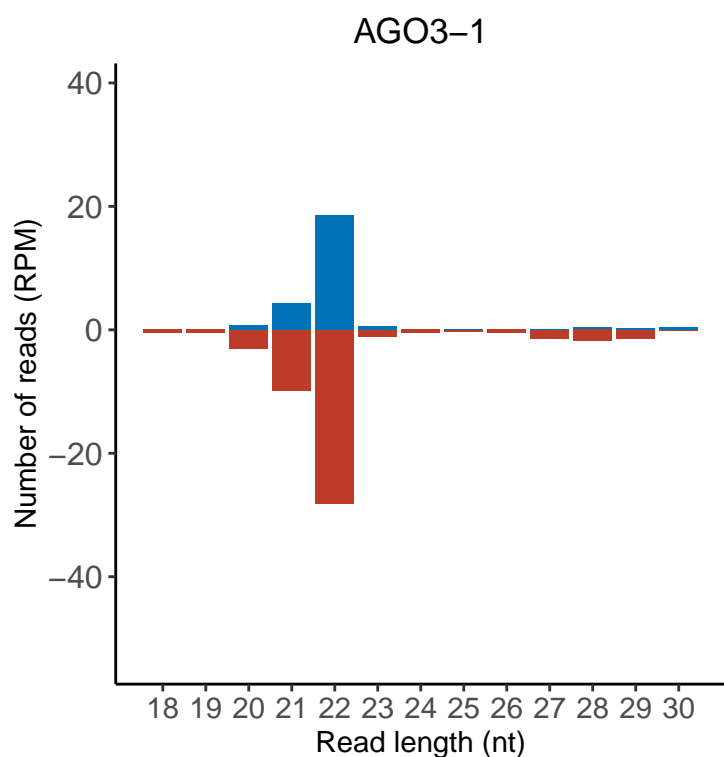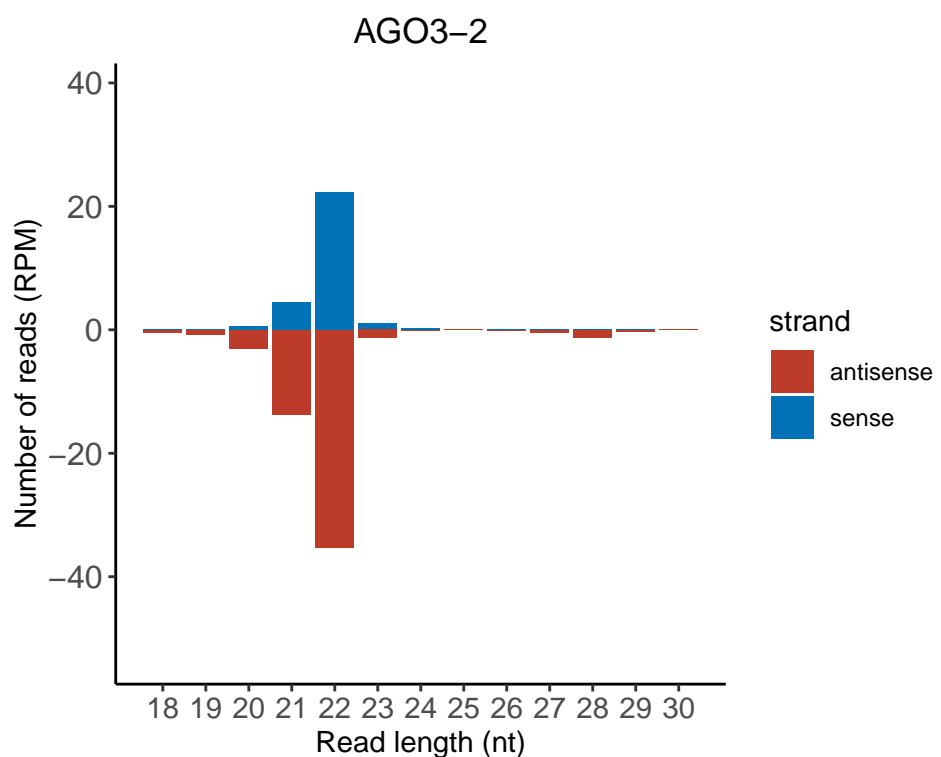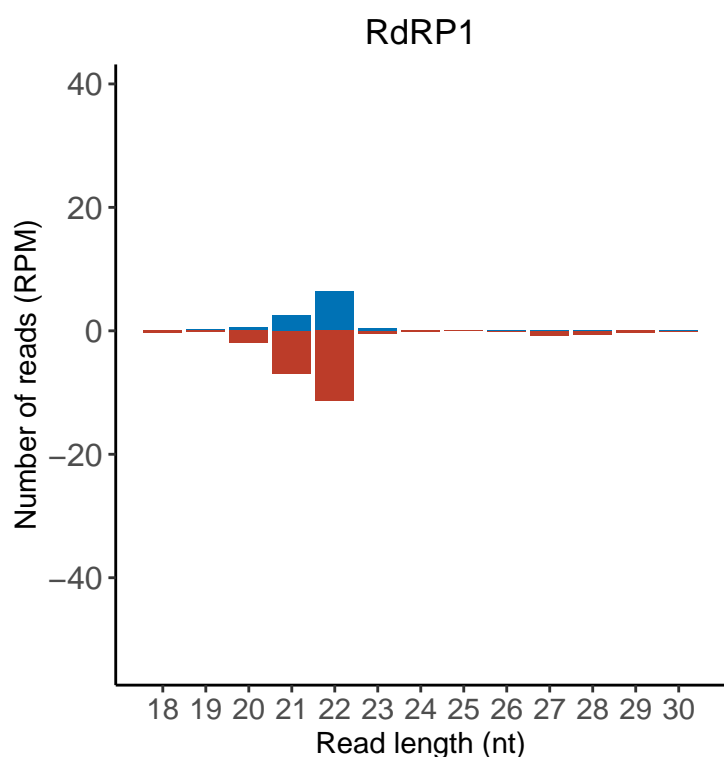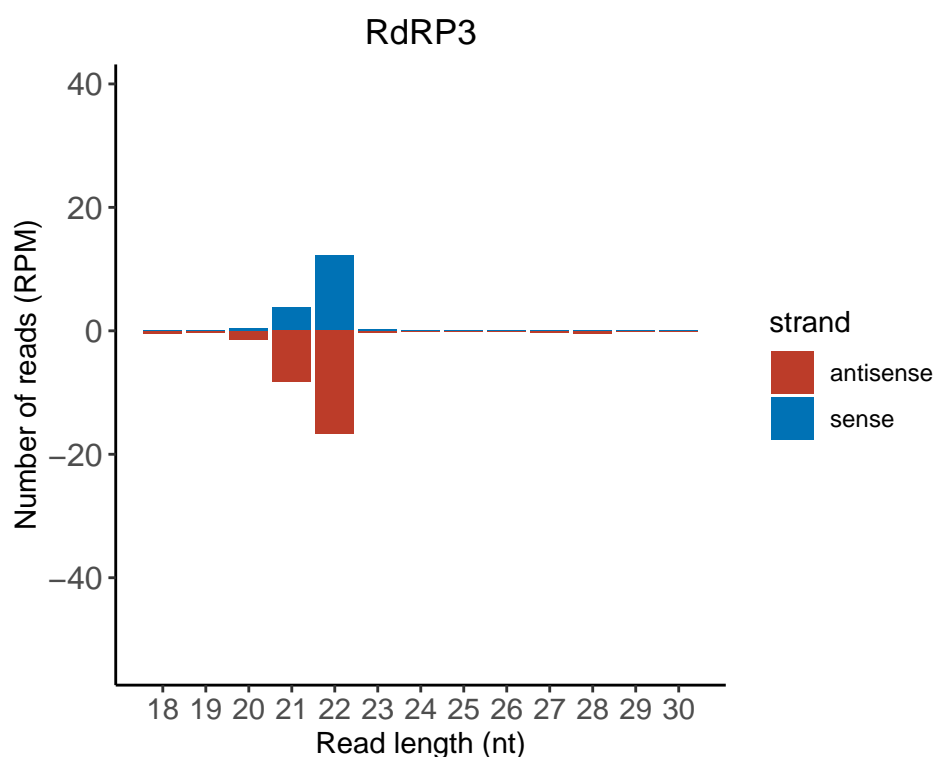

Supplement: S1 Data — On the Normalized and Relative levels pages, normalized read counts (RPM) and relative levels compared to the control (dsGFP) library were used. TEs with more than 50RPM and Coding Genes with more than 3.5RPM on average in KD libraries are included in this website. For viral sequences, reference sequences were generated by assembling sRNA sequences (See the Analysis of sRNAseq data section in Materials and methods.) and size distributions of sRNA reads mapped to each contig are shown. Reads were first grouped by their 5’ nucleotides, and reads of each length were counted. Full tables including TEs and Coding genes with fewer reads are in the “FullTable” folder. (ZIP) [file pone.0281195.s017.zip › SupplementaryData/Links/PDFs/Motif:rnd-6_family-906TE_lengthdistribution.pdf]

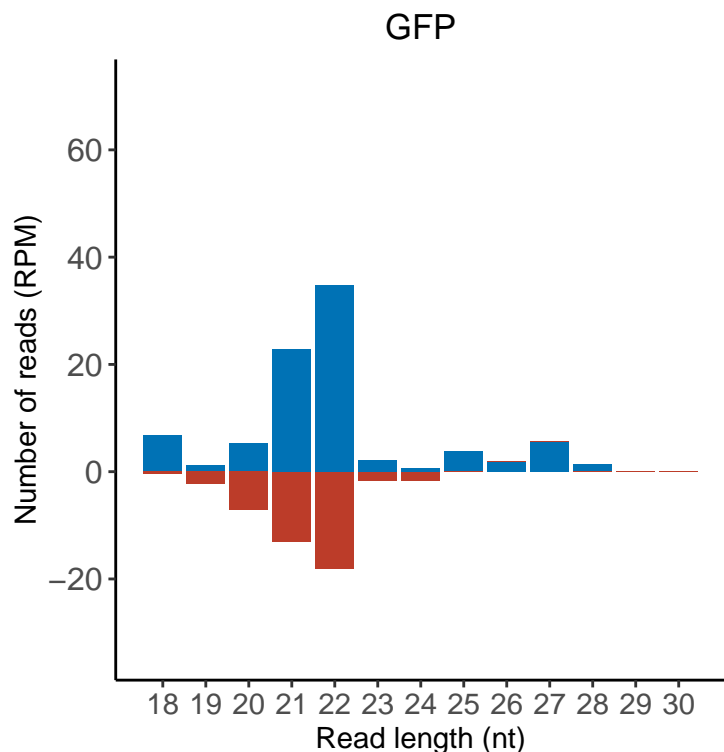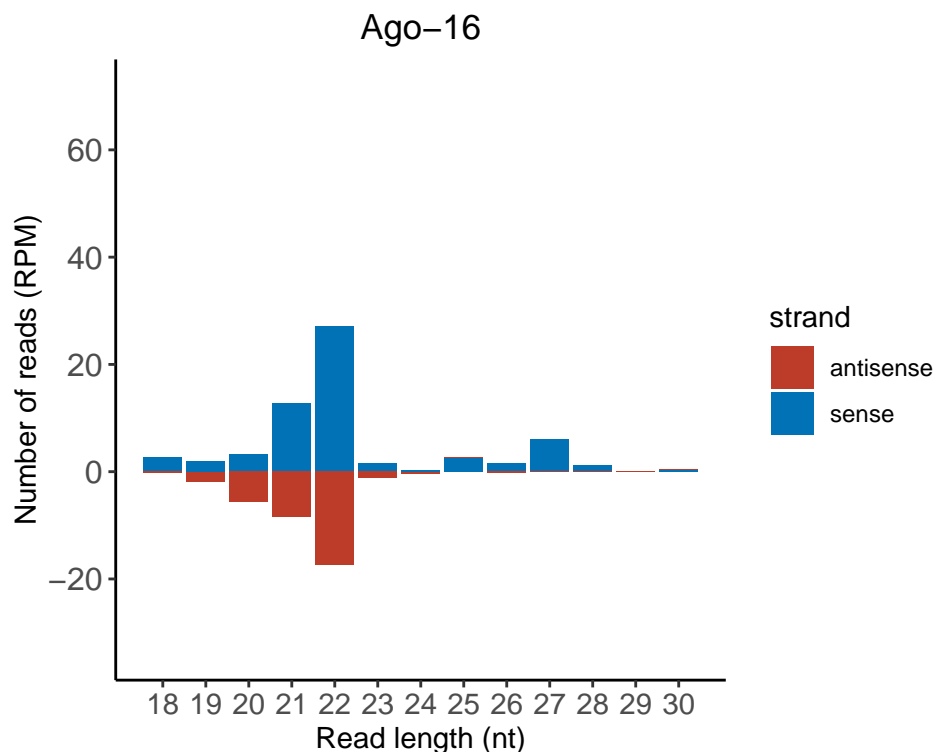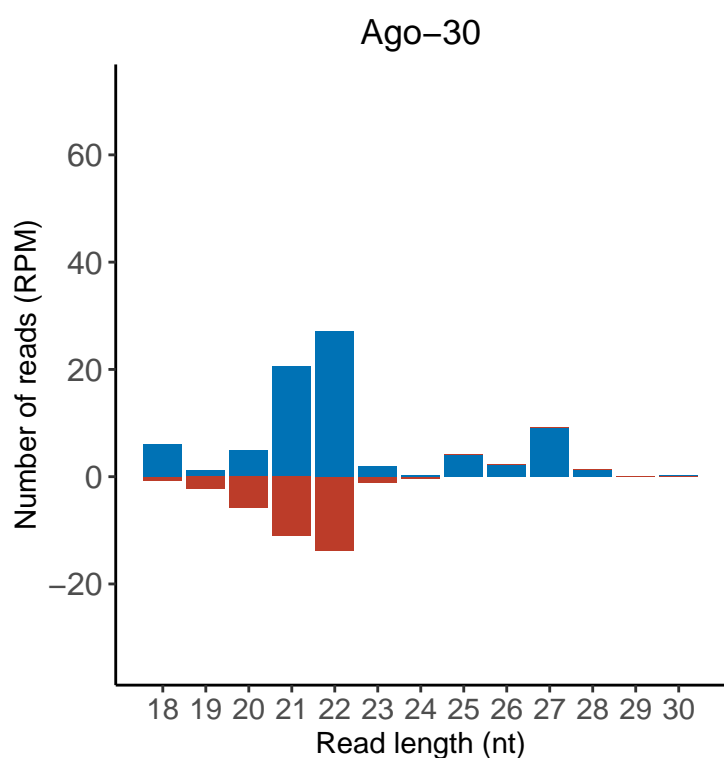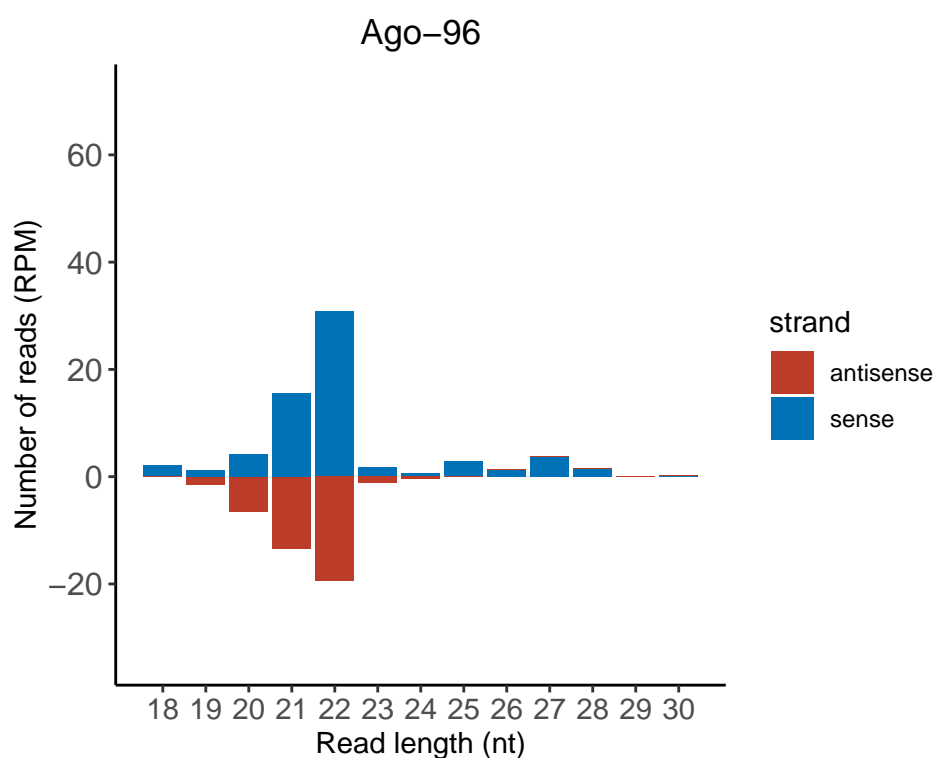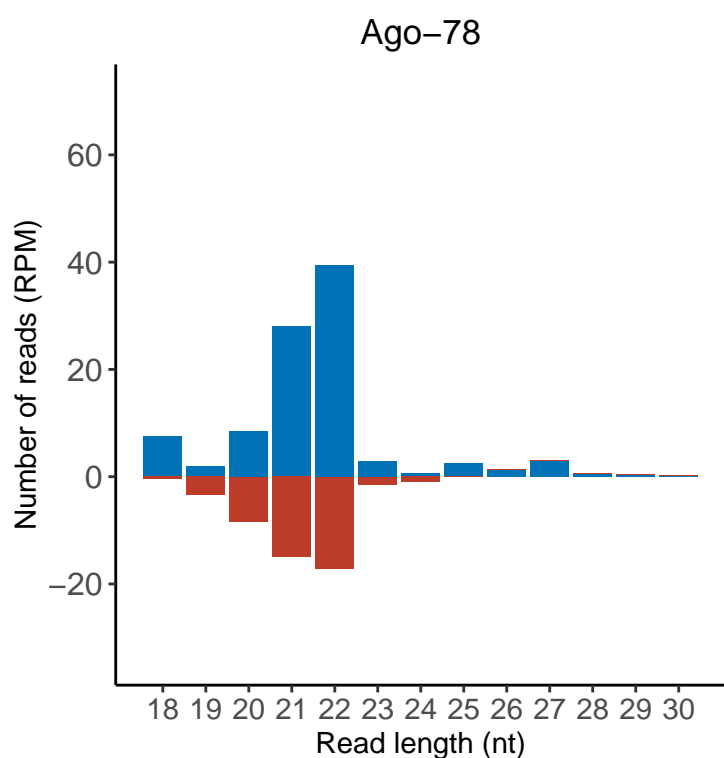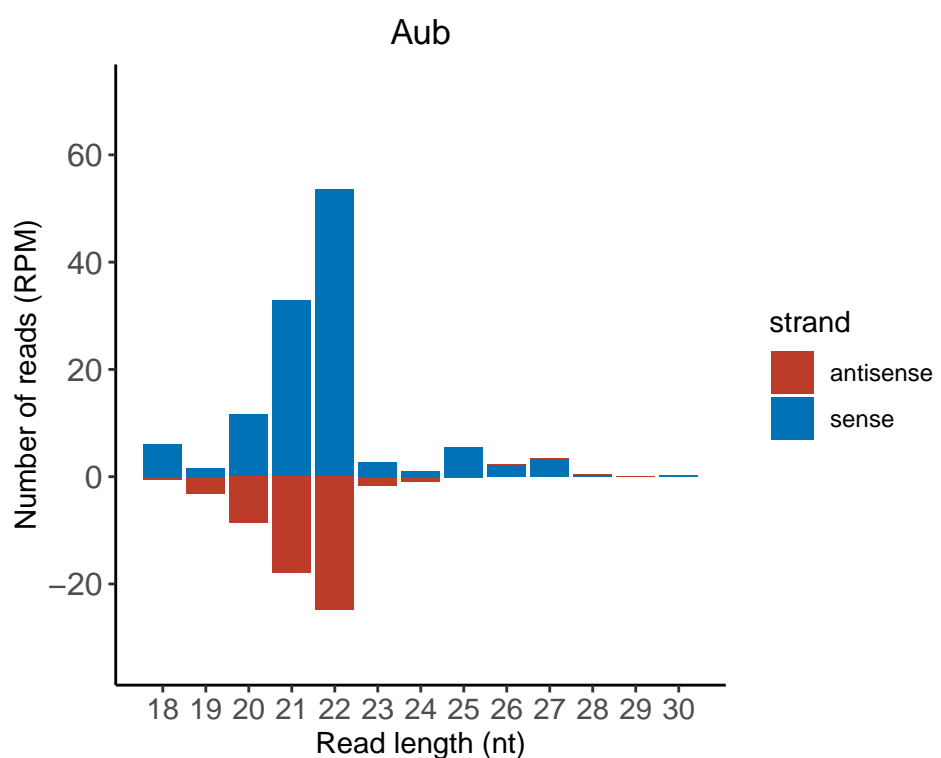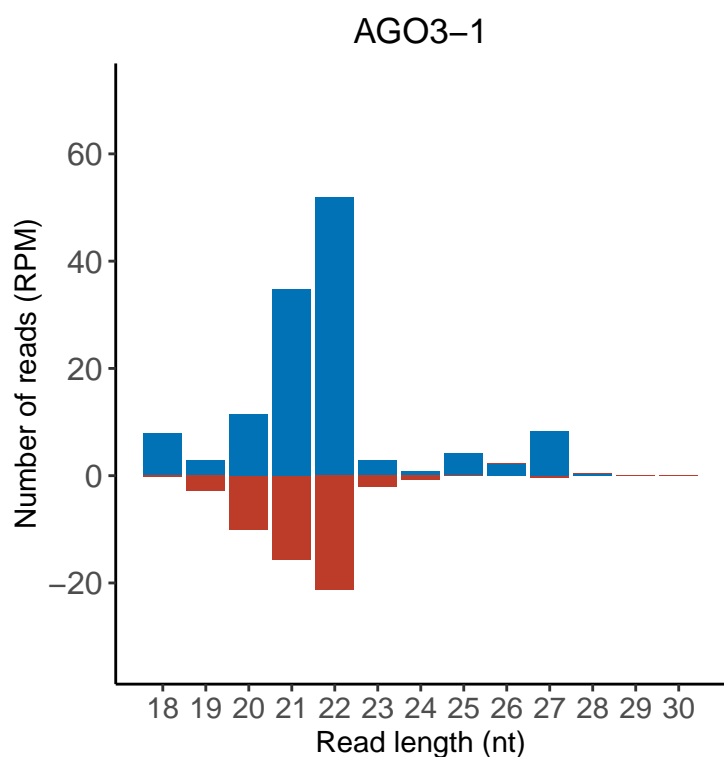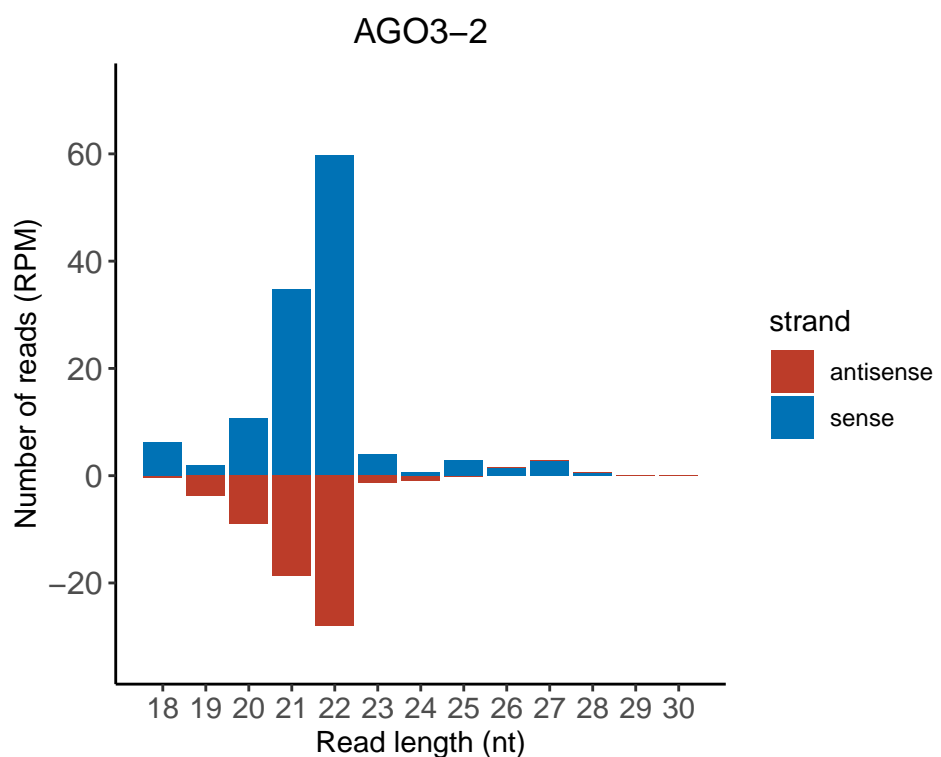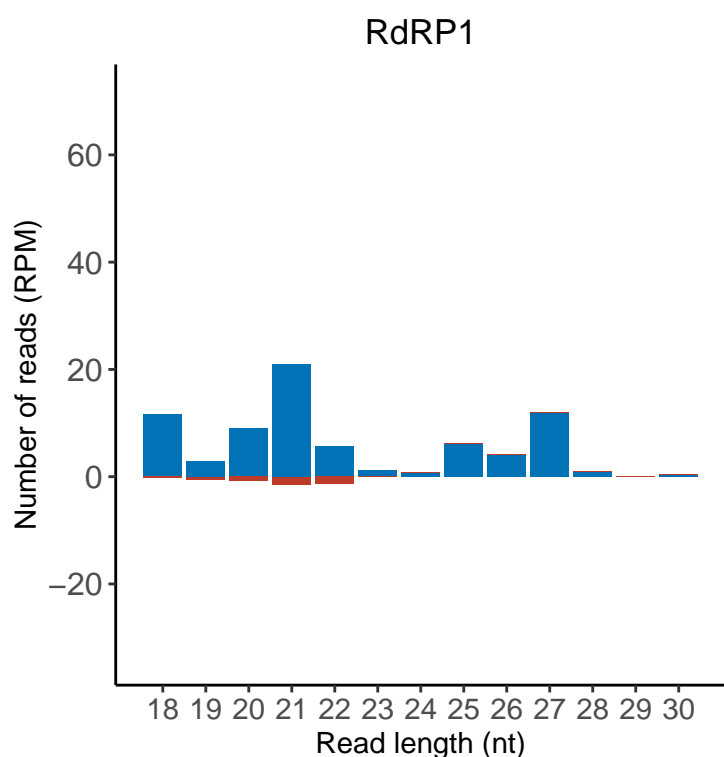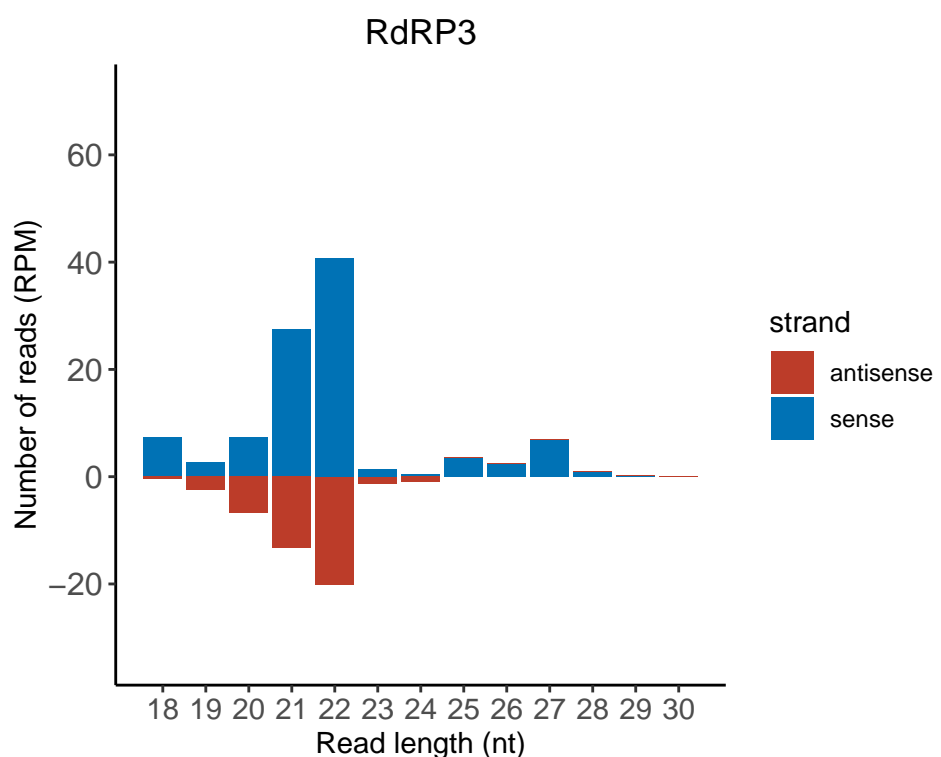

Supplement: S1 Data — On the Normalized and Relative levels pages, normalized read counts (RPM) and relative levels compared to the control (dsGFP) library were used. TEs with more than 50RPM and Coding Genes with more than 3.5RPM on average in KD libraries are included in this website. For viral sequences, reference sequences were generated by assembling sRNA sequences (See the Analysis of sRNAseq data section in Materials and methods.) and size distributions of sRNA reads mapped to each contig are shown. Reads were first grouped by their 5’ nucleotides, and reads of each length were counted. Full tables including TEs and Coding genes with fewer reads are in the “FullTable” folder. (ZIP) [file pone.0281195.s017.zip › SupplementaryData/Links/PDFs/Motif:rnd-5_family-1764TE_lengthdistribution.pdf]

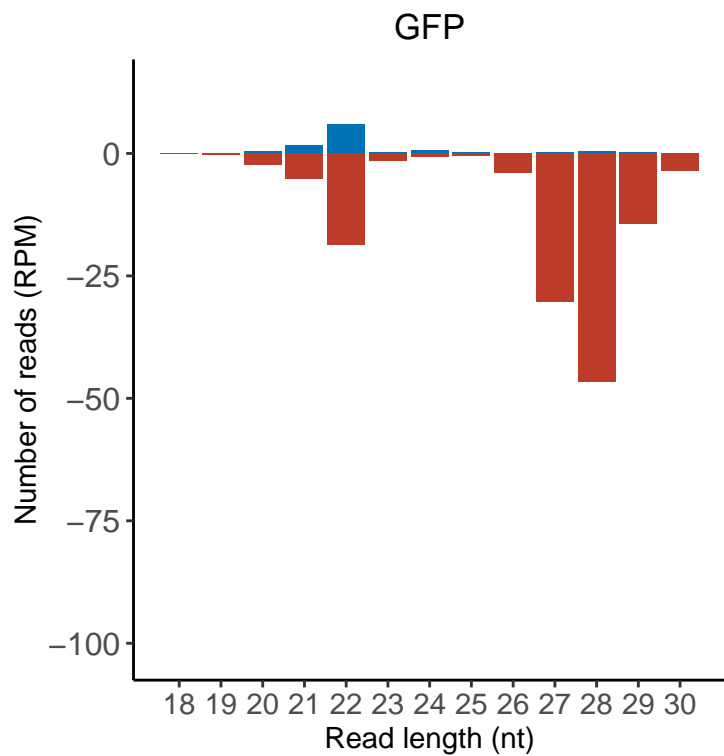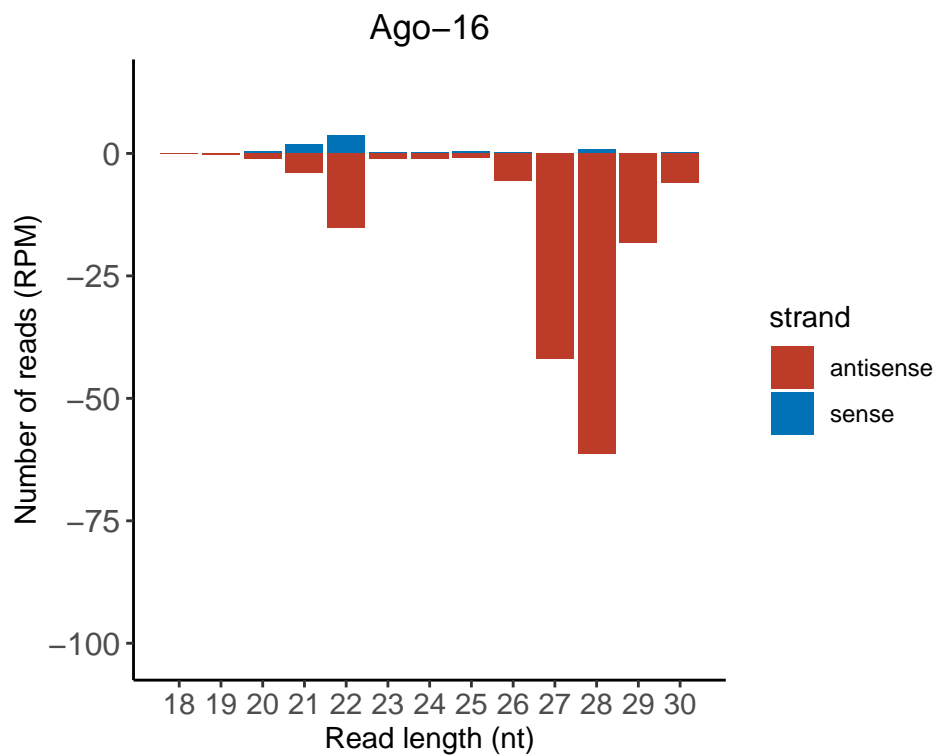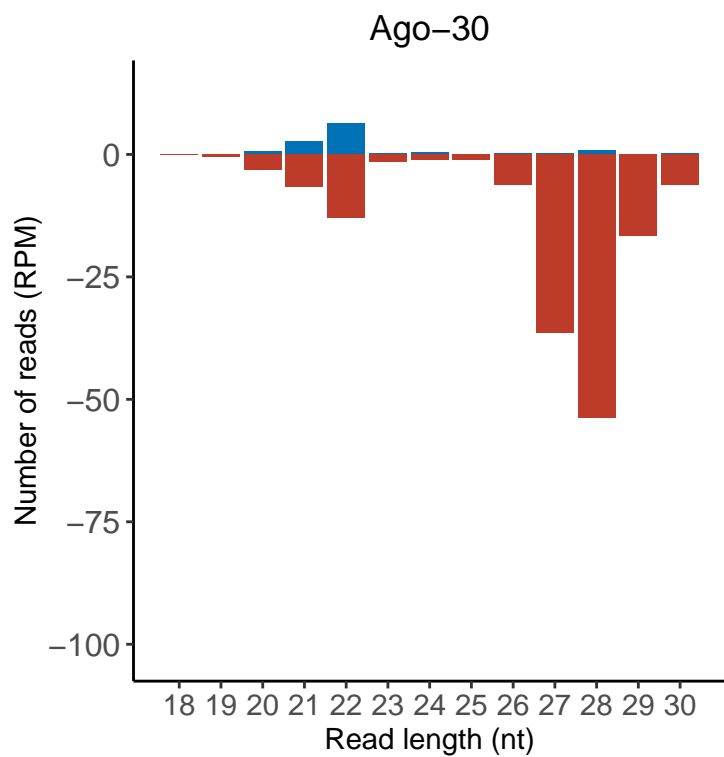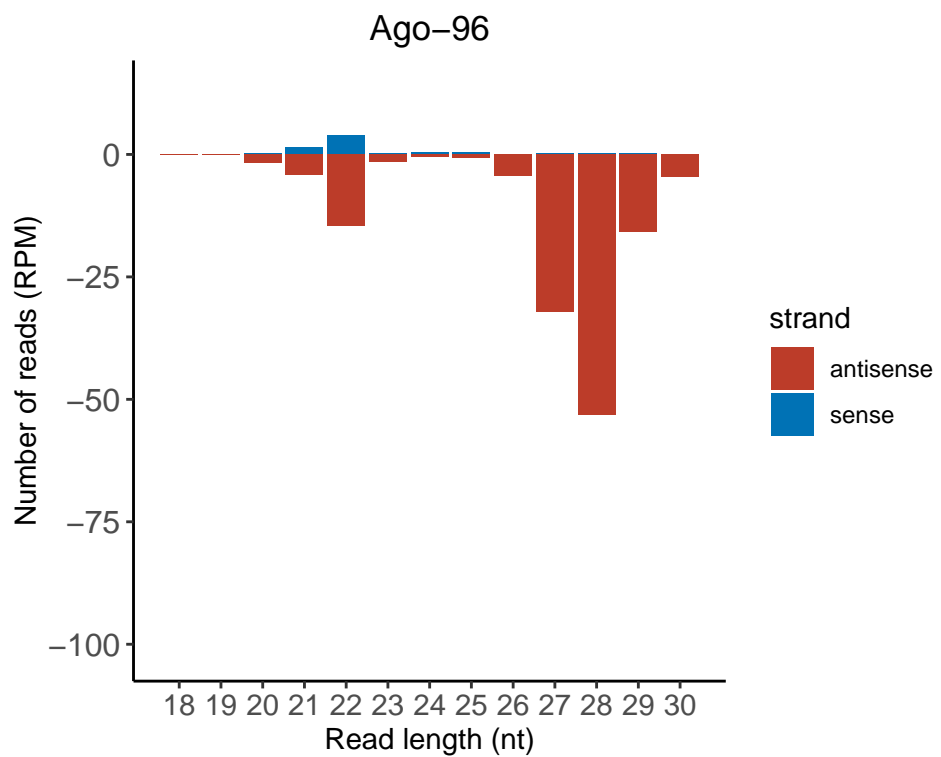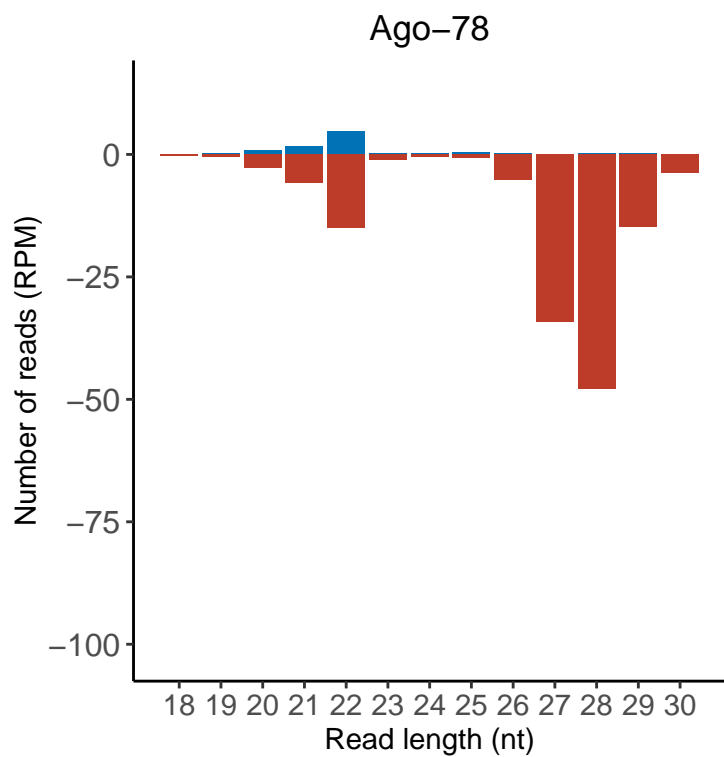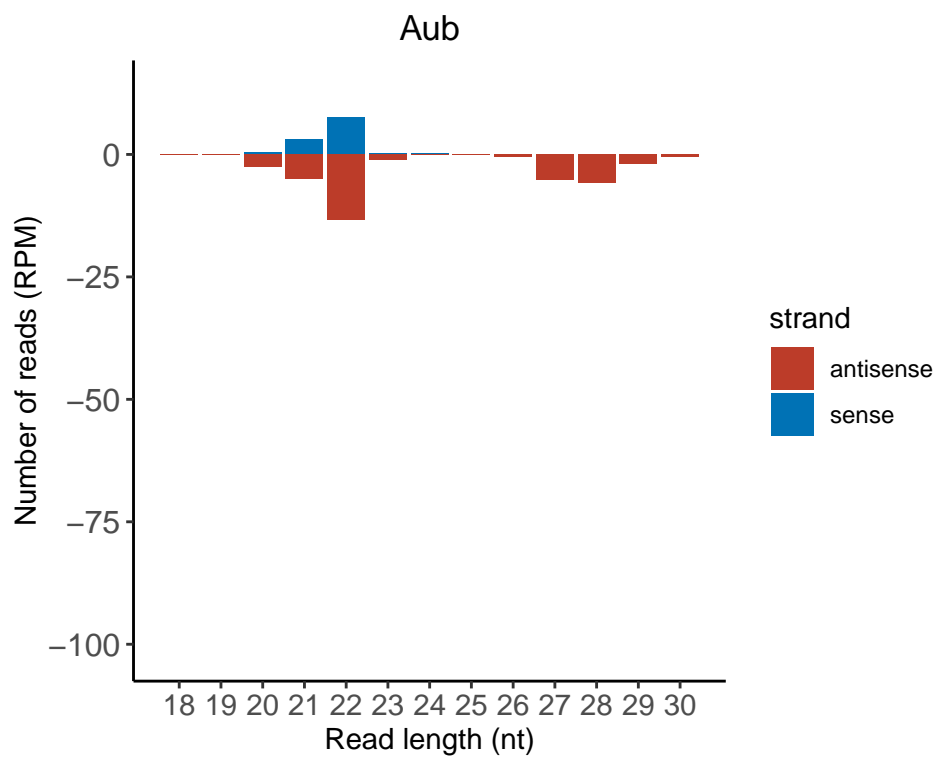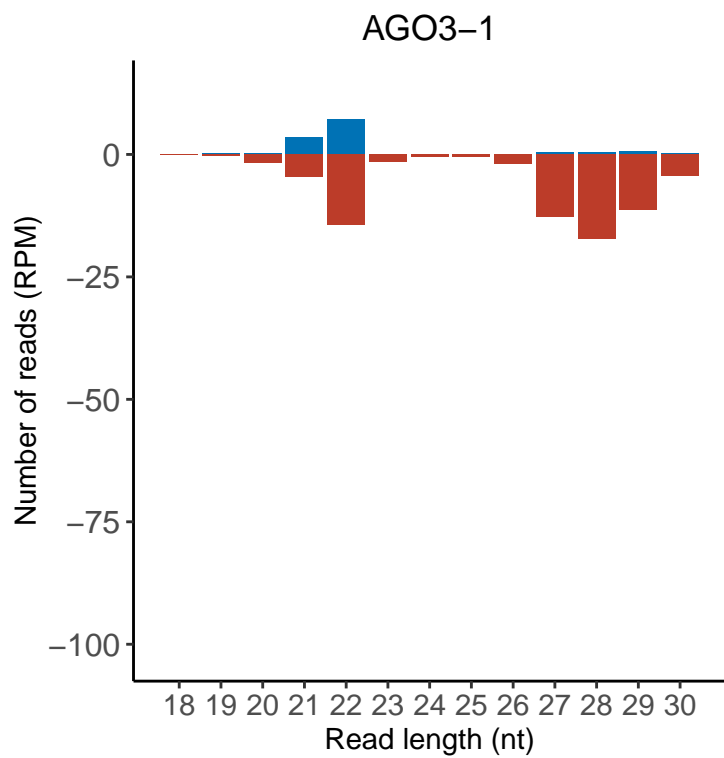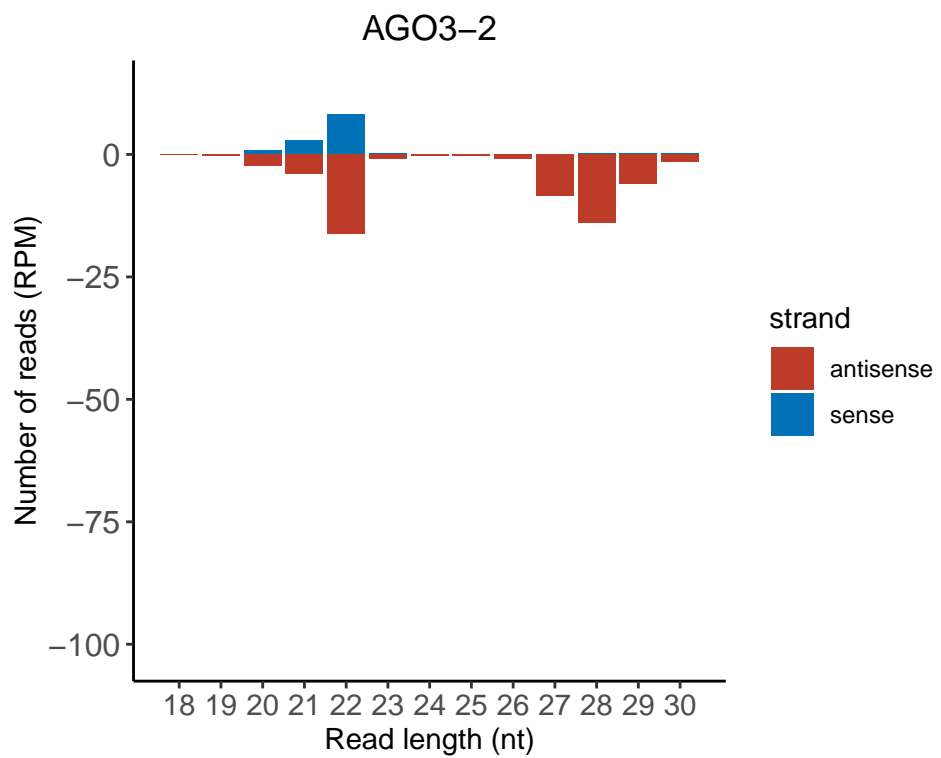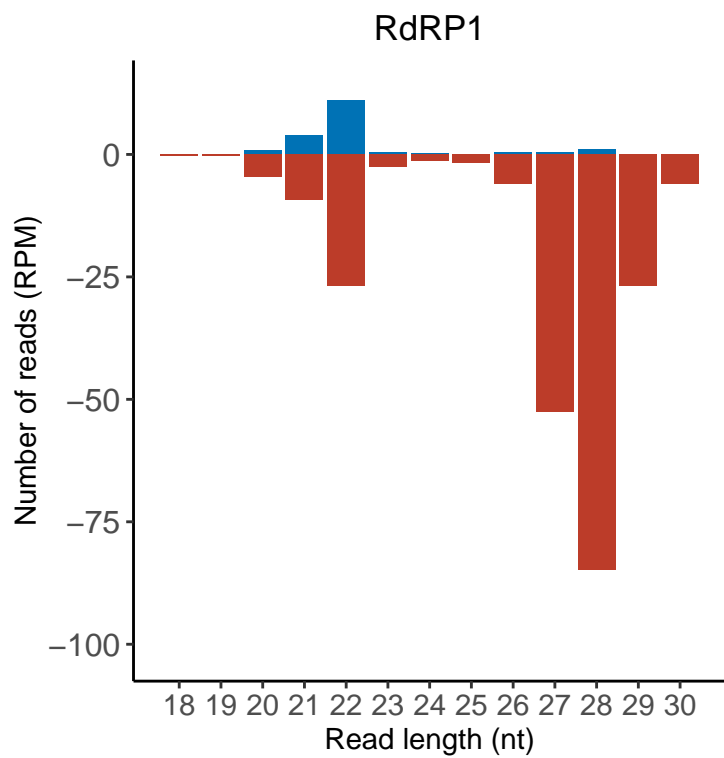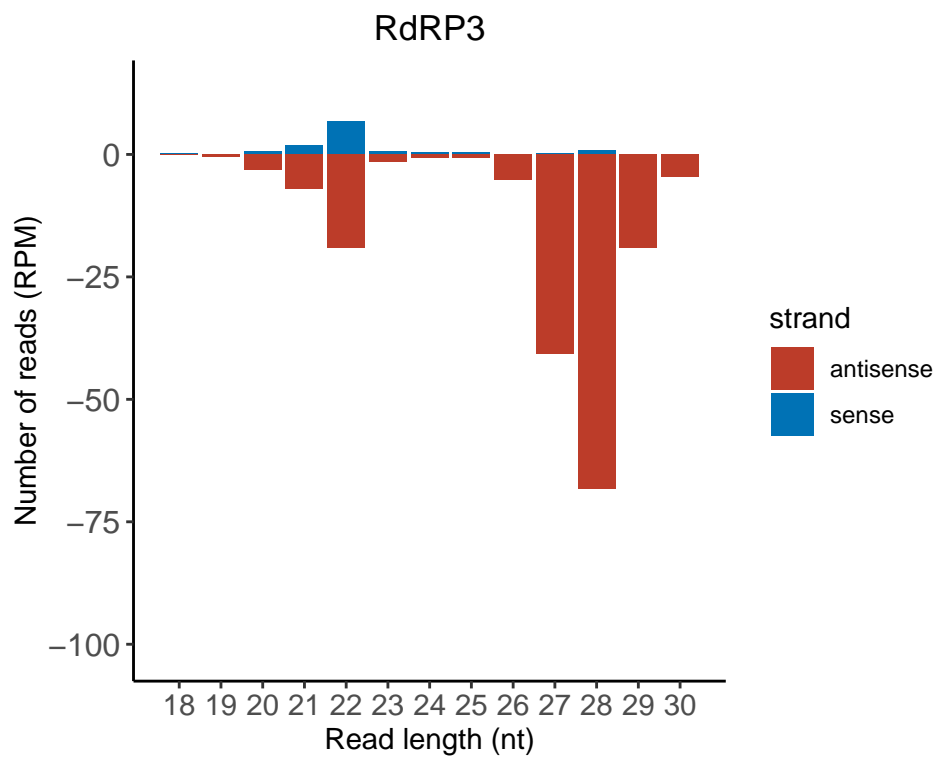

Supplement: S1 Data — On the Normalized and Relative levels pages, normalized read counts (RPM) and relative levels compared to the control (dsGFP) library were used. TEs with more than 50RPM and Coding Genes with more than 3.5RPM on average in KD libraries are included in this website. For viral sequences, reference sequences were generated by assembling sRNA sequences (See the Analysis of sRNAseq data section in Materials and methods.) and size distributions of sRNA reads mapped to each contig are shown. Reads were first grouped by their 5’ nucleotides, and reads of each length were counted. Full tables including TEs and Coding genes with fewer reads are in the “FullTable” folder. (ZIP) [file pone.0281195.s017.zip › SupplementaryData/Links/PDFs/Motif:rnd-1_family-173TE_lengthdistribution.pdf]

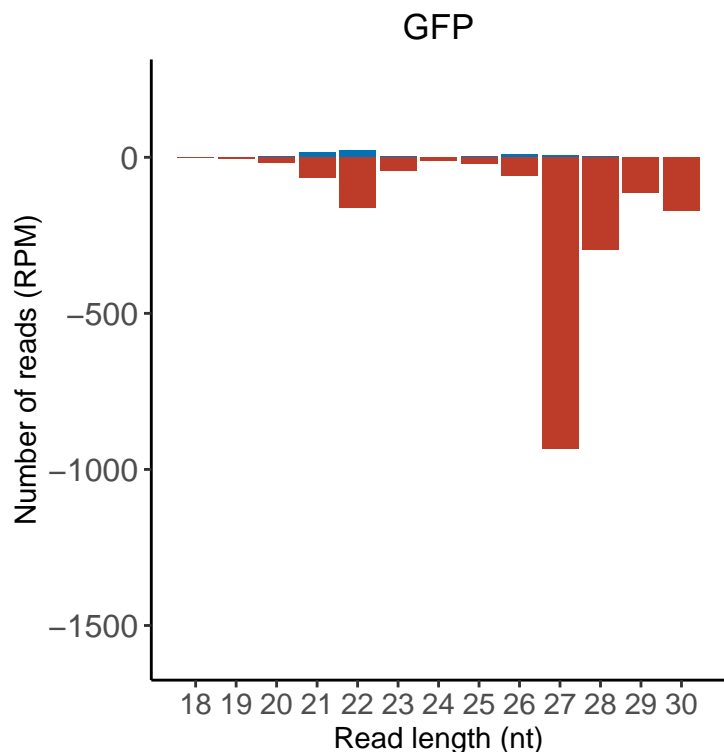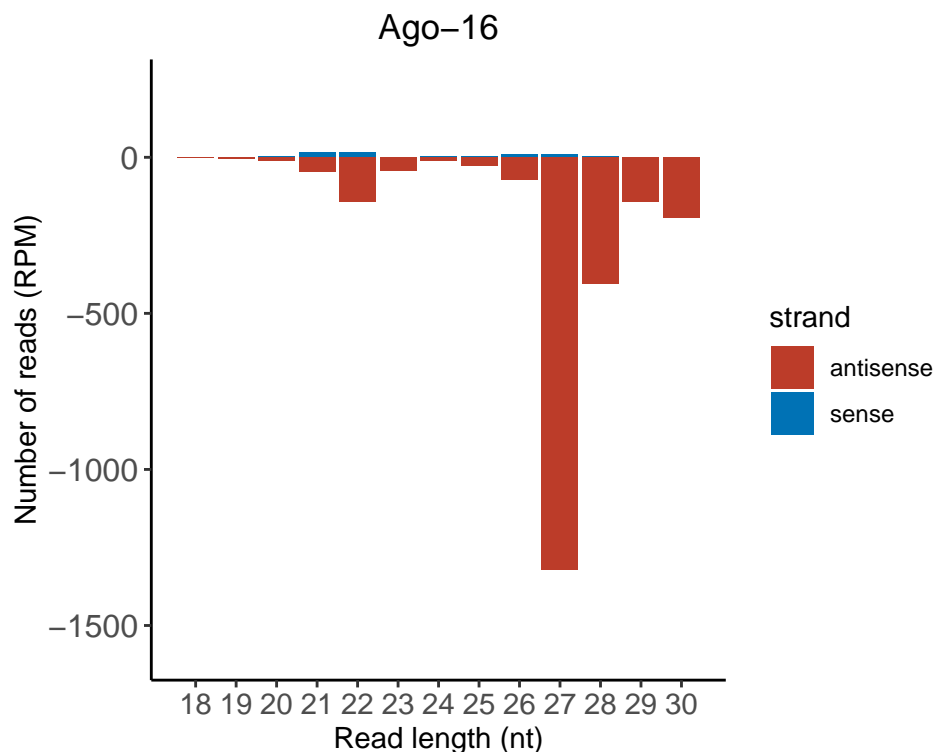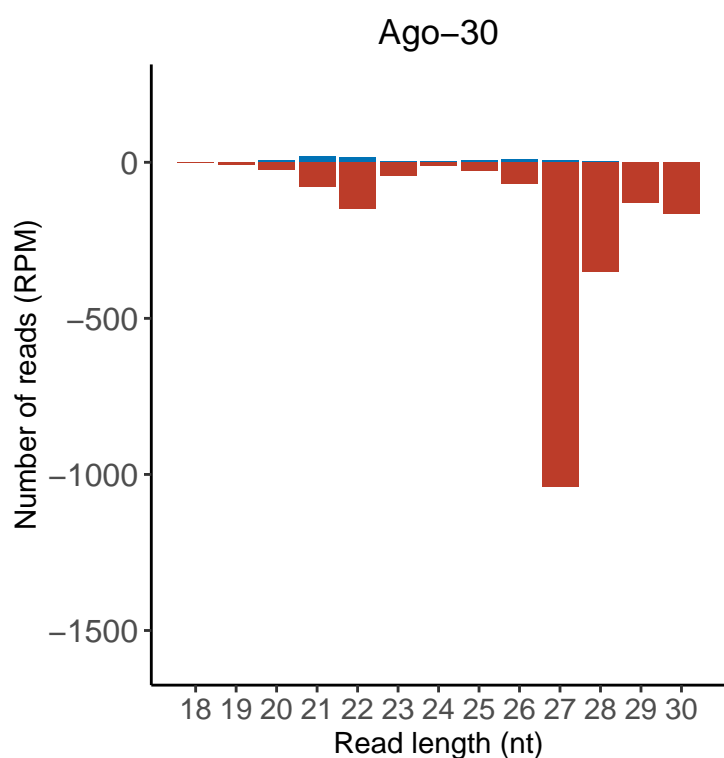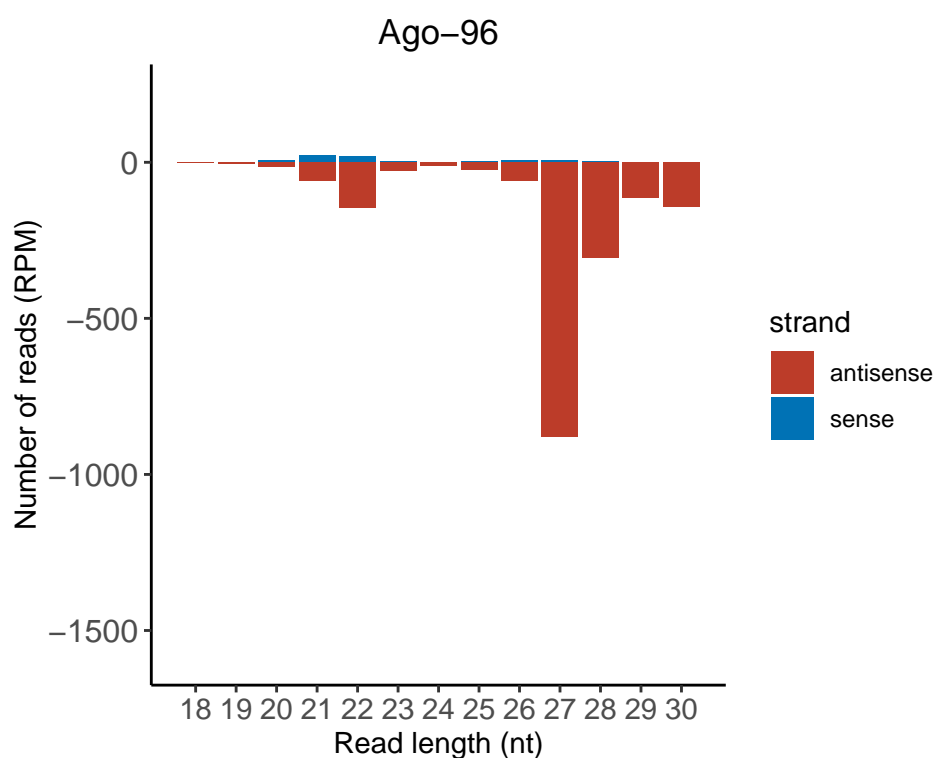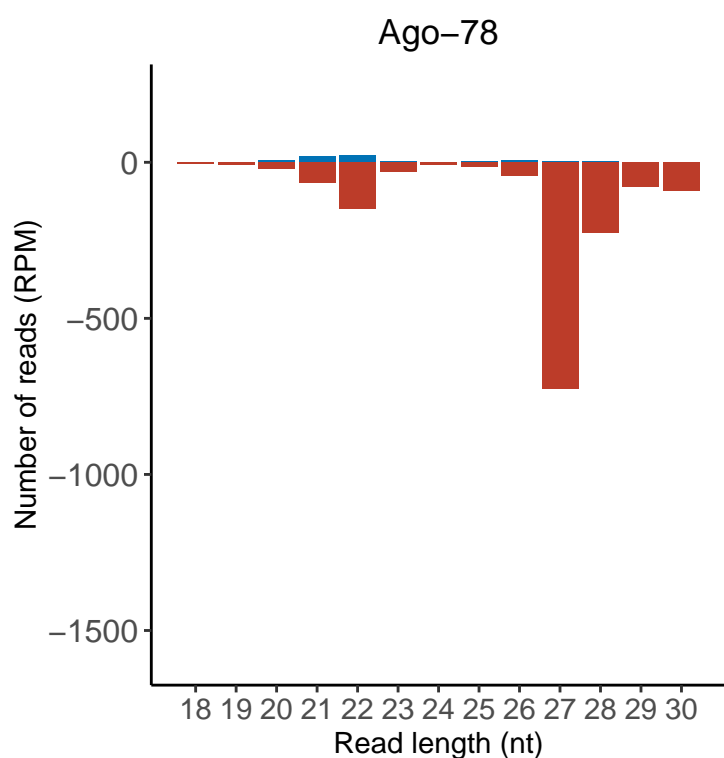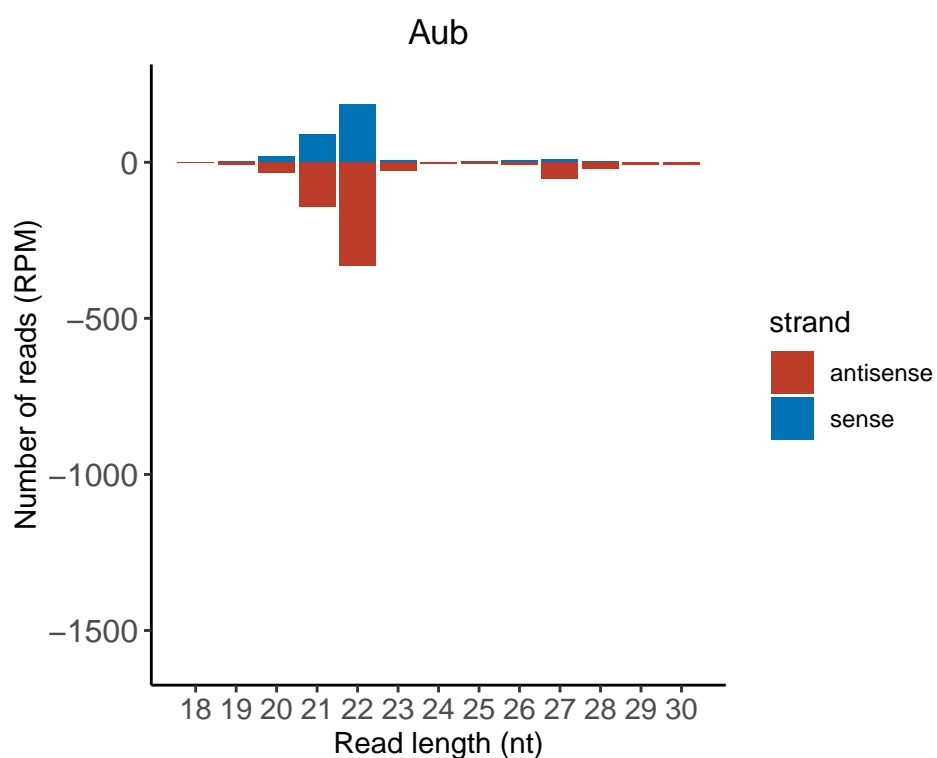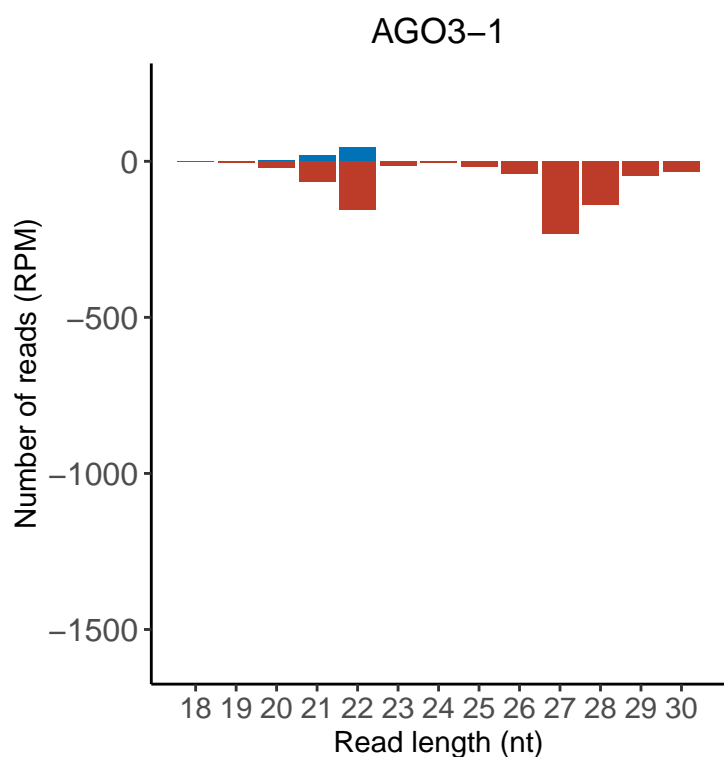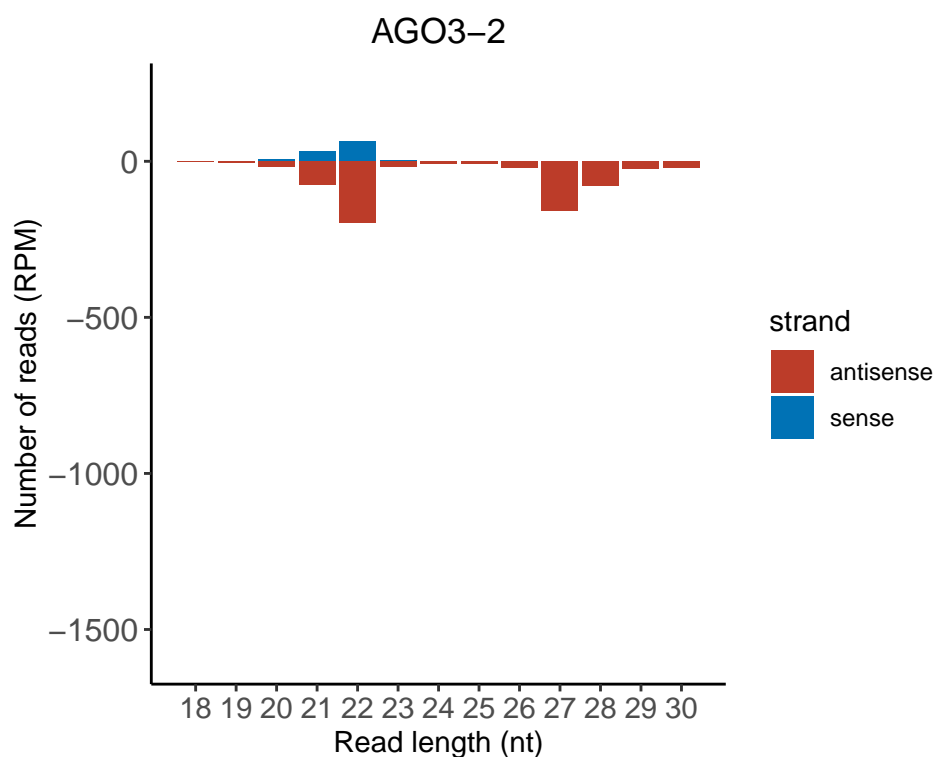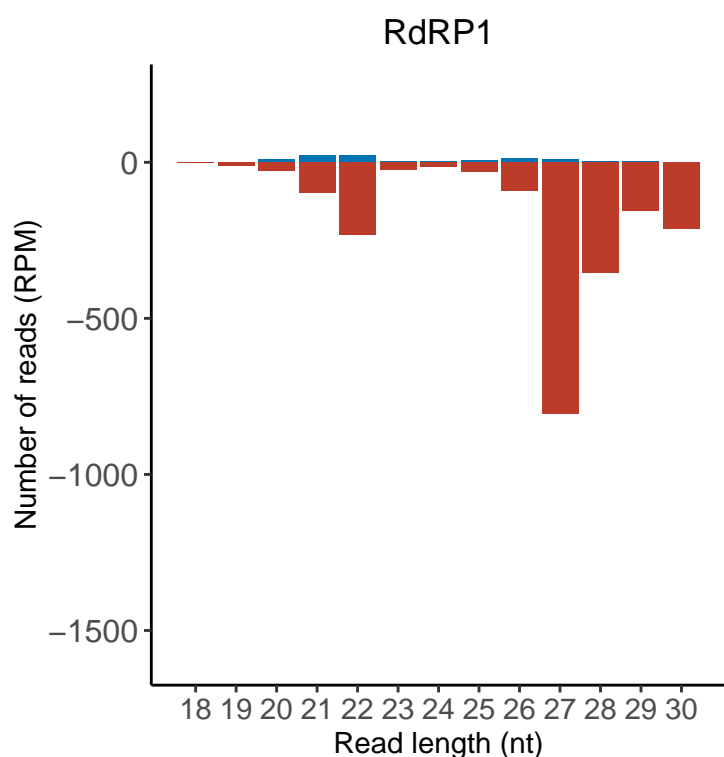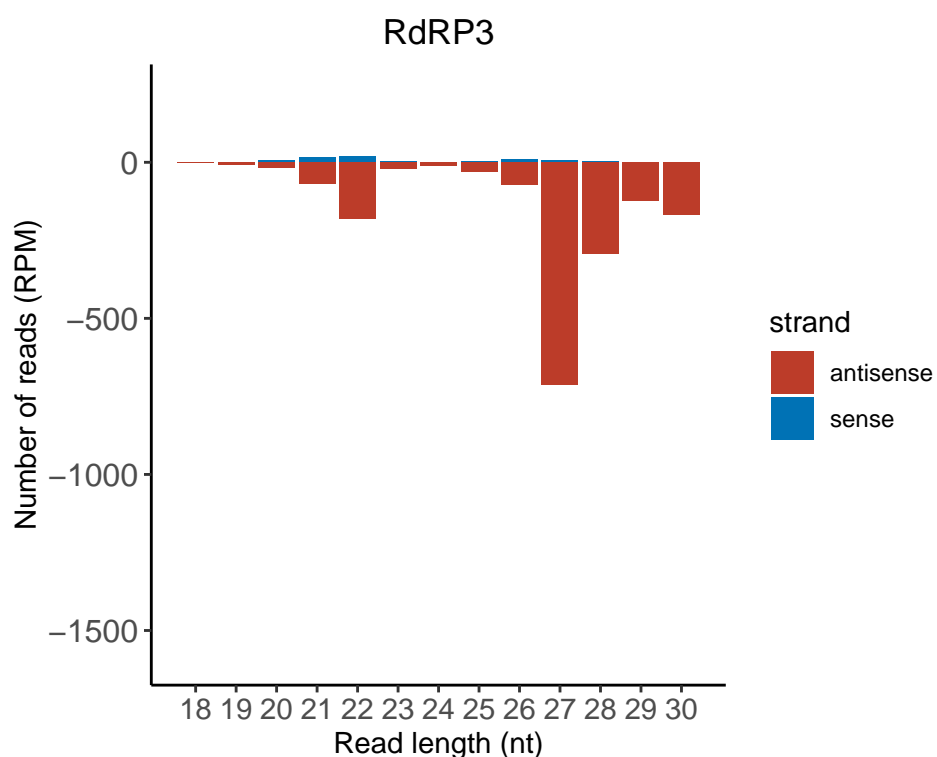

Supplement: S1 Data — On the Normalized and Relative levels pages, normalized read counts (RPM) and relative levels compared to the control (dsGFP) library were used. TEs with more than 50RPM and Coding Genes with more than 3.5RPM on average in KD libraries are included in this website. For viral sequences, reference sequences were generated by assembling sRNA sequences (See the Analysis of sRNAseq data section in Materials and methods.) and size distributions of sRNA reads mapped to each contig are shown. Reads were first grouped by their 5’ nucleotides, and reads of each length were counted. Full tables including TEs and Coding genes with fewer reads are in the “FullTable” folder. (ZIP) [file pone.0281195.s017.zip › SupplementaryData/Links/PDFs/Motif:rnd-6_family-4430TE_lengthdistribution.pdf]

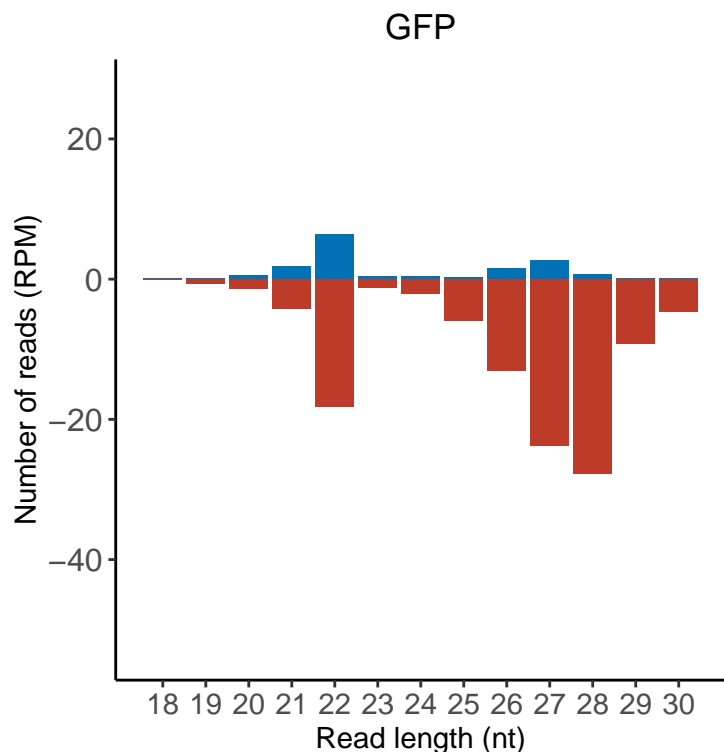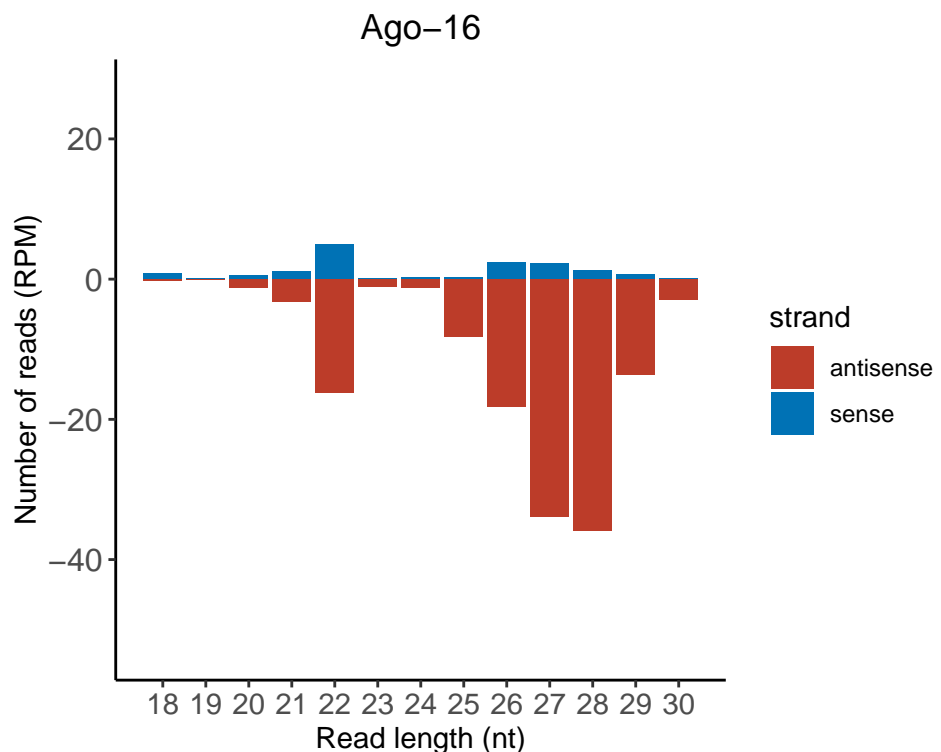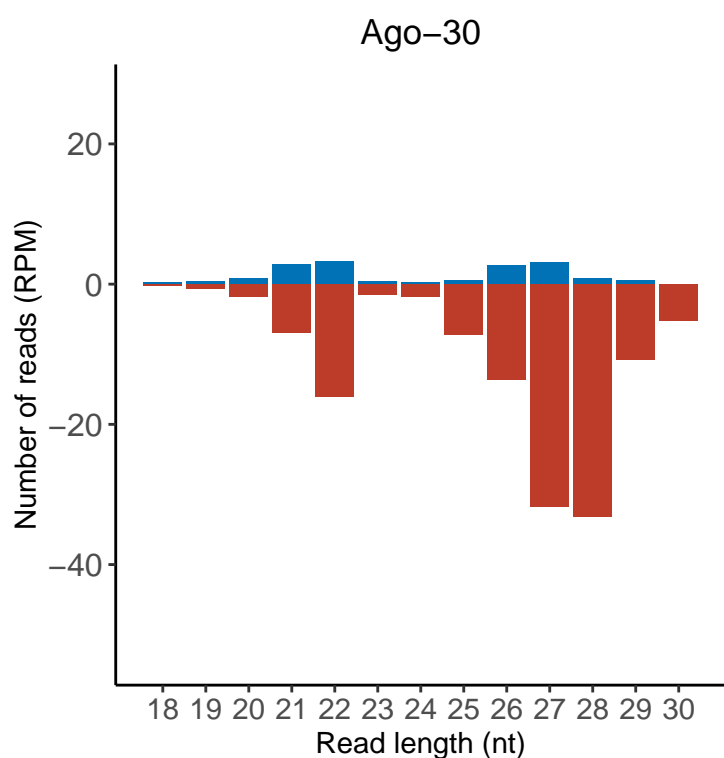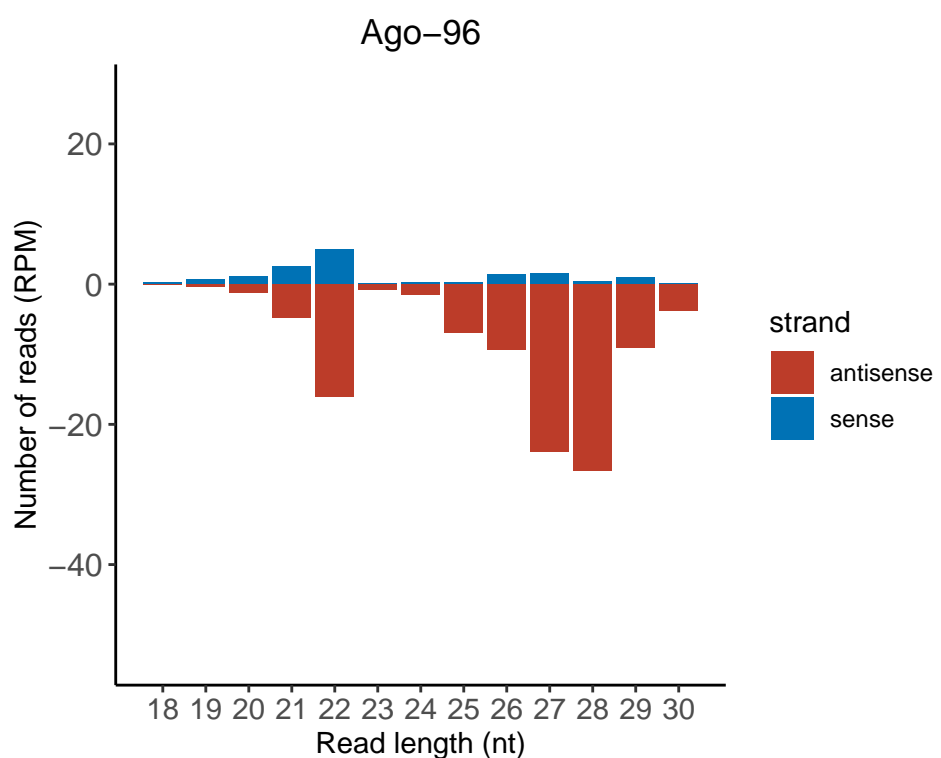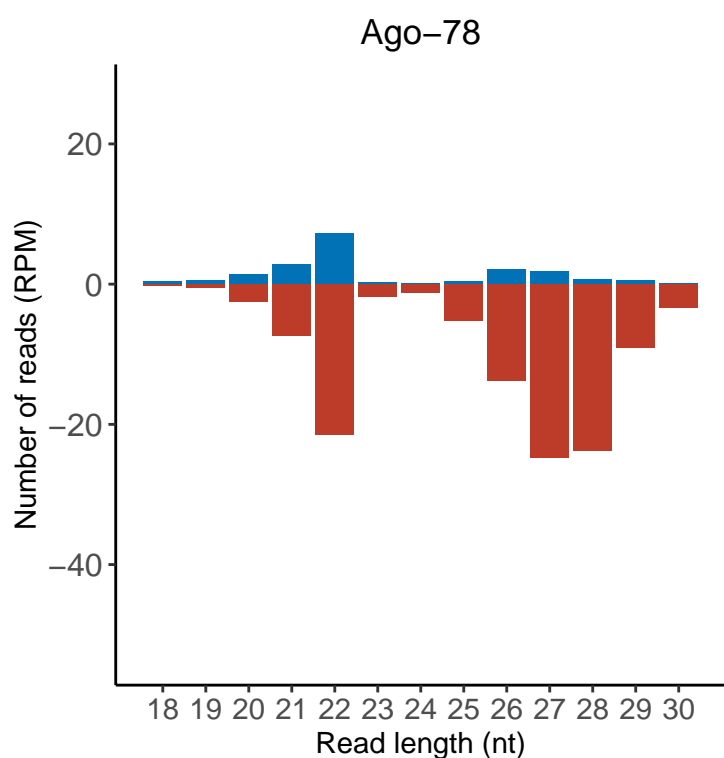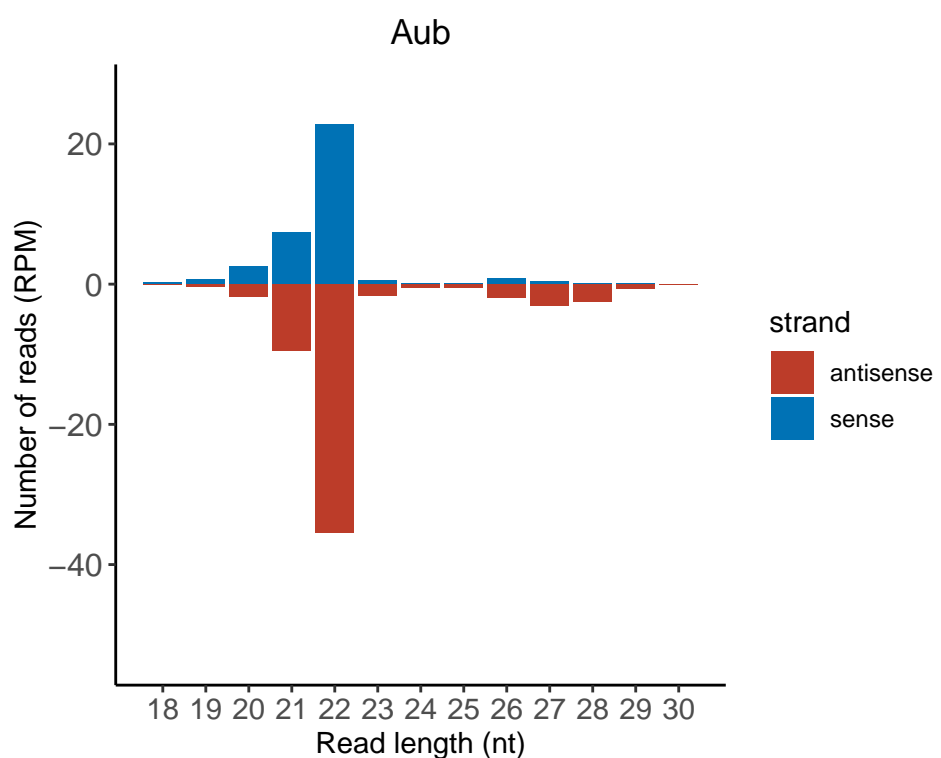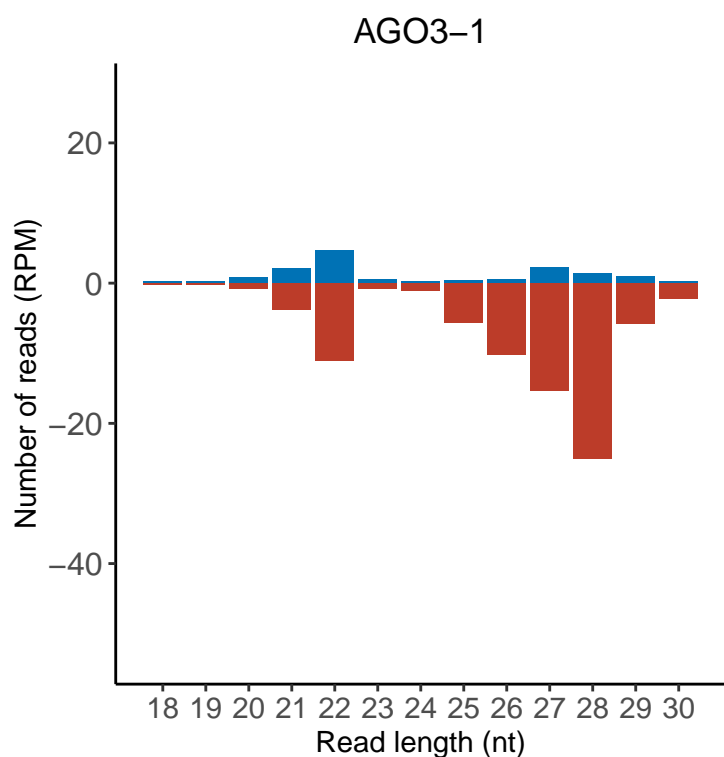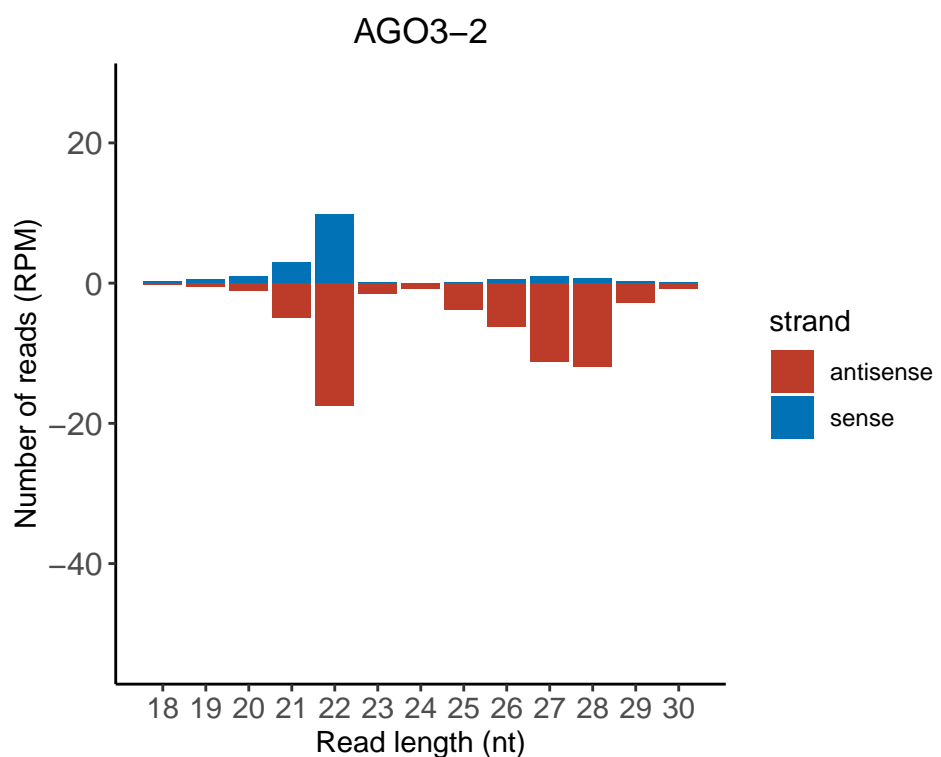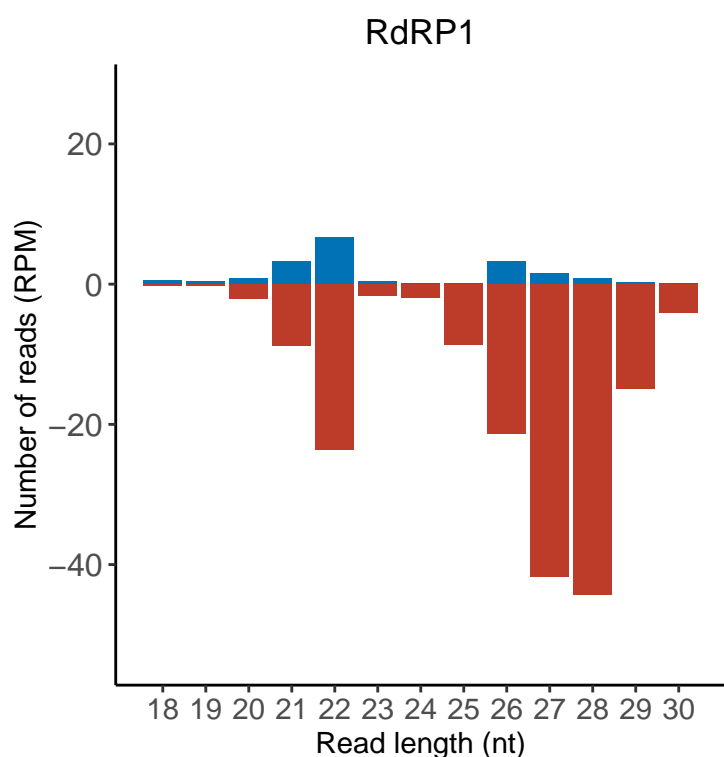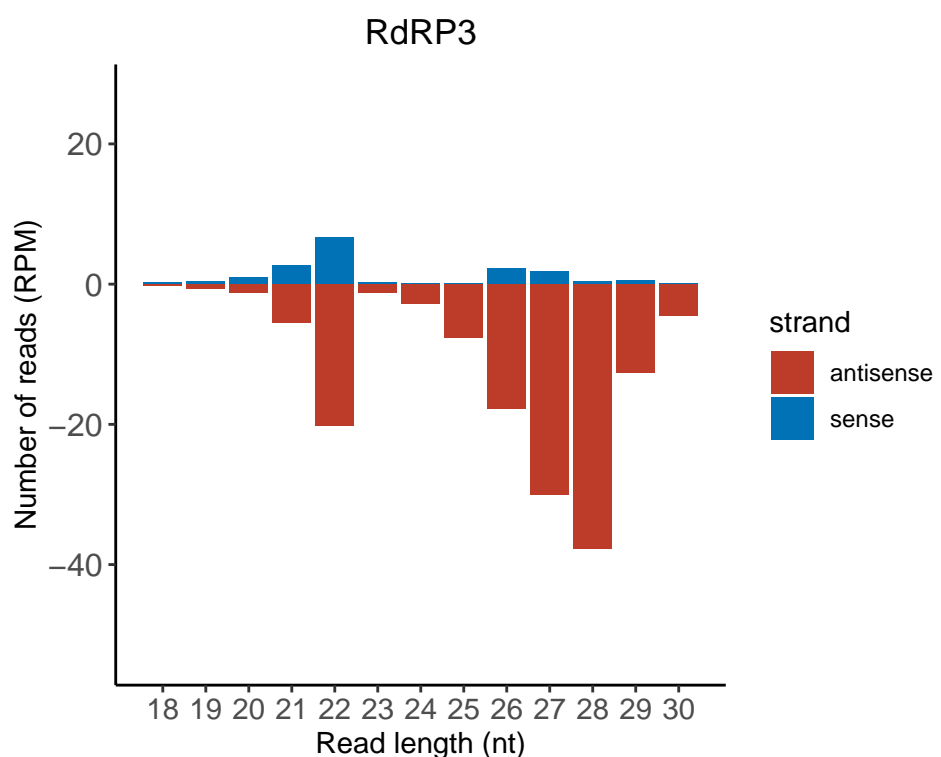

Supplement: S1 Data — On the Normalized and Relative levels pages, normalized read counts (RPM) and relative levels compared to the control (dsGFP) library were used. TEs with more than 50RPM and Coding Genes with more than 3.5RPM on average in KD libraries are included in this website. For viral sequences, reference sequences were generated by assembling sRNA sequences (See the Analysis of sRNAseq data section in Materials and methods.) and size distributions of sRNA reads mapped to each contig are shown. Reads were first grouped by their 5’ nucleotides, and reads of each length were counted. Full tables including TEs and Coding genes with fewer reads are in the “FullTable” folder. (ZIP) [file pone.0281195.s017.zip › SupplementaryData/Links/PDFs/Motif:rnd-6_family-7992TE_lengthdistribution.pdf]

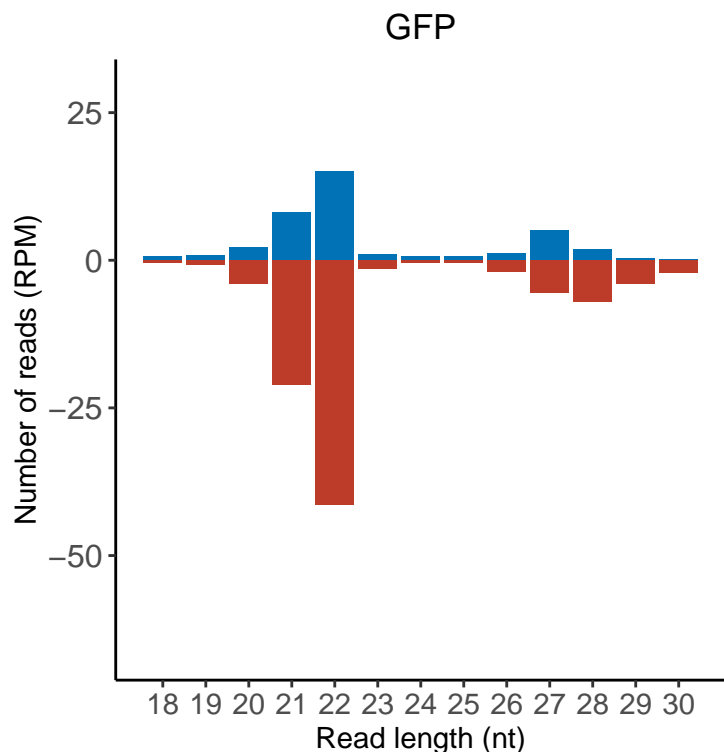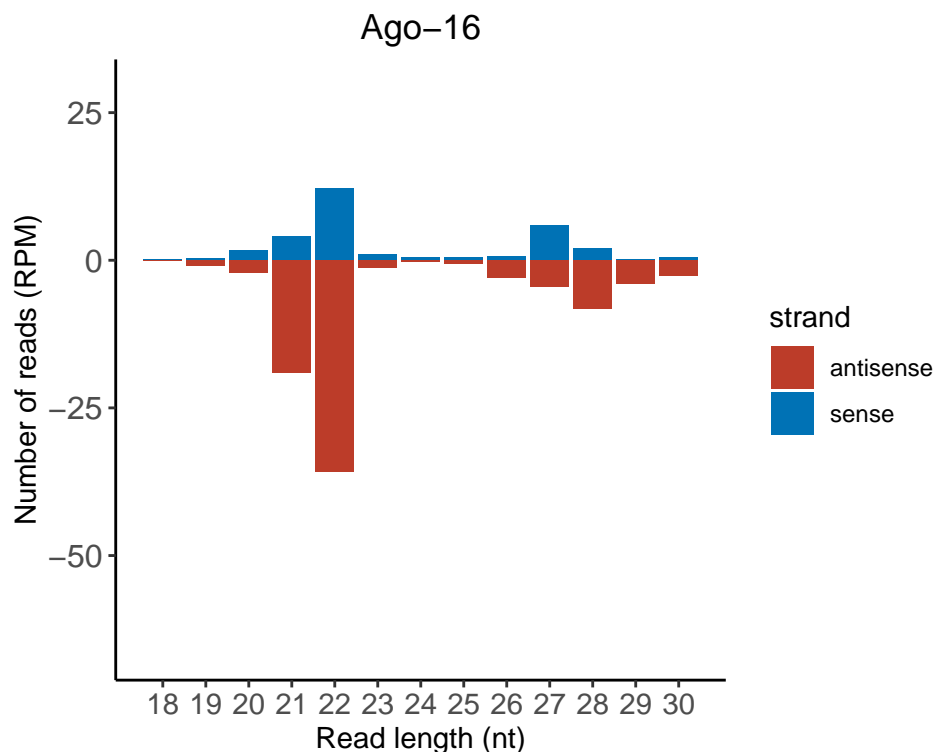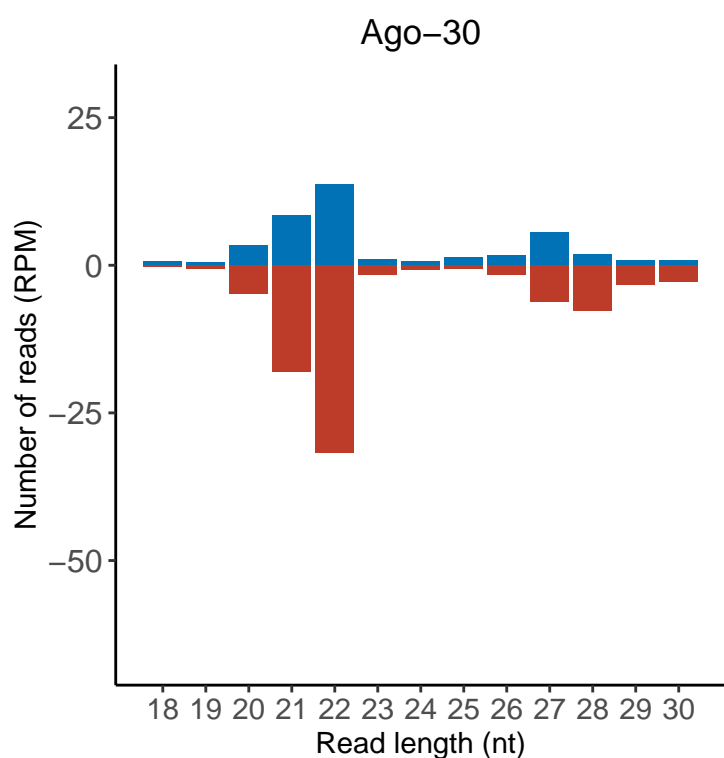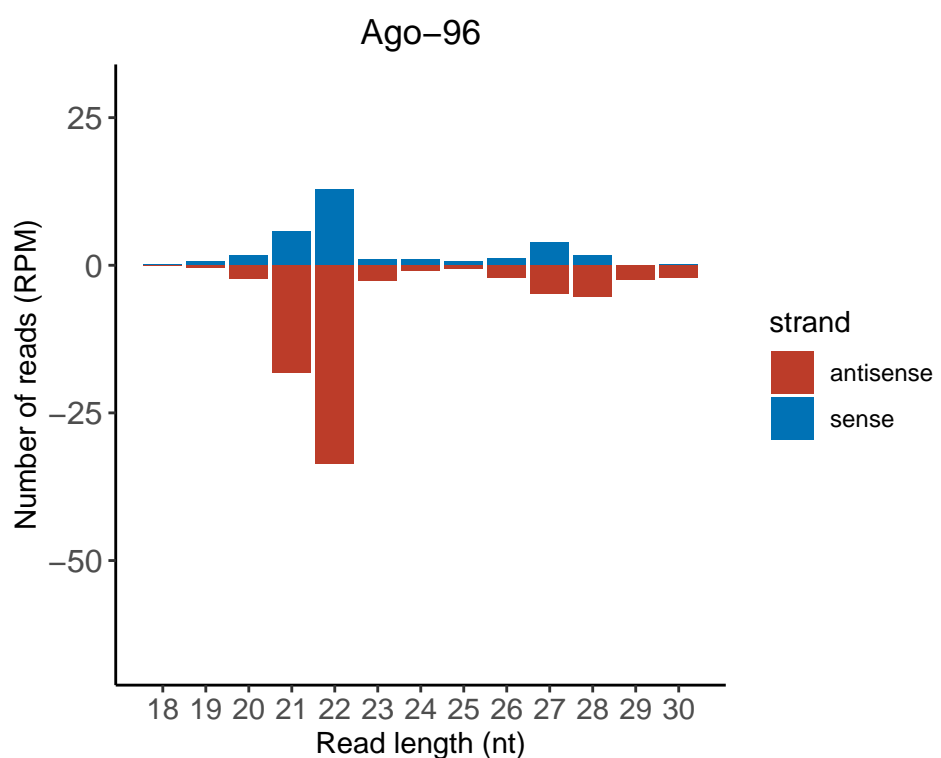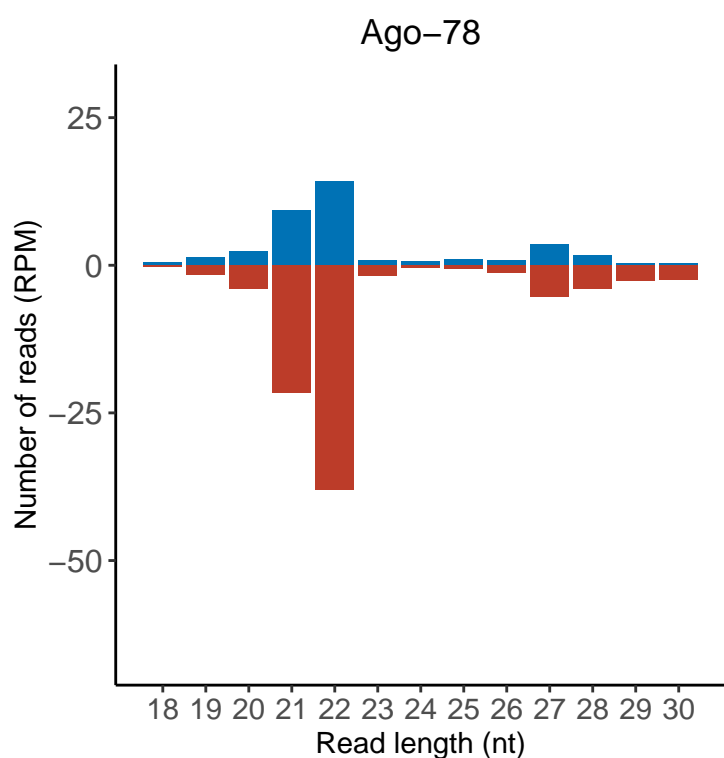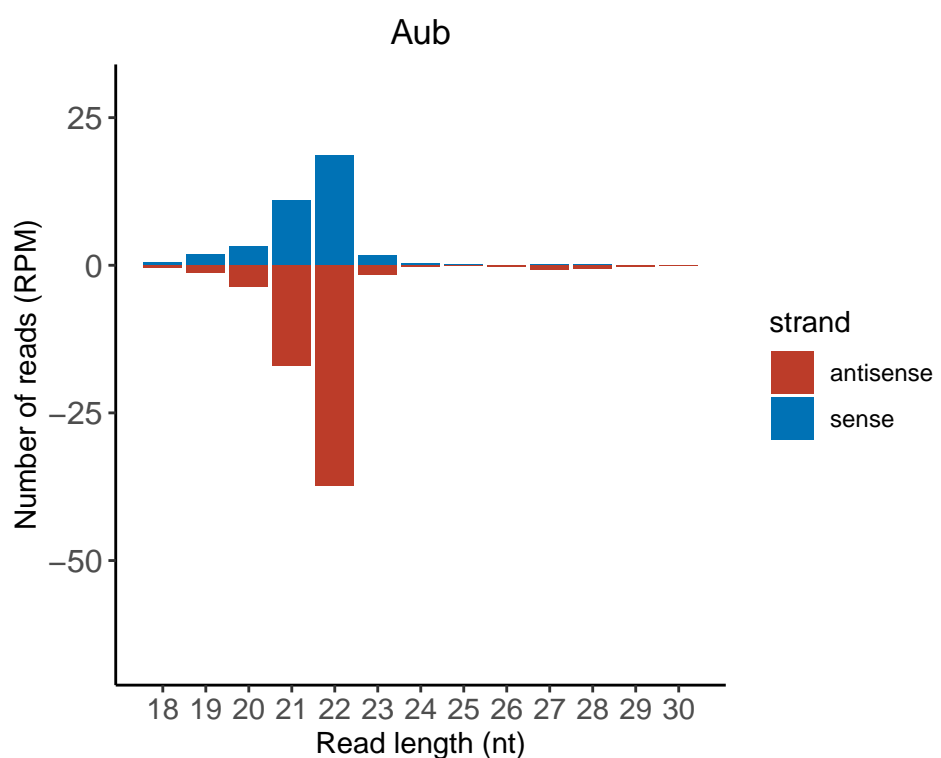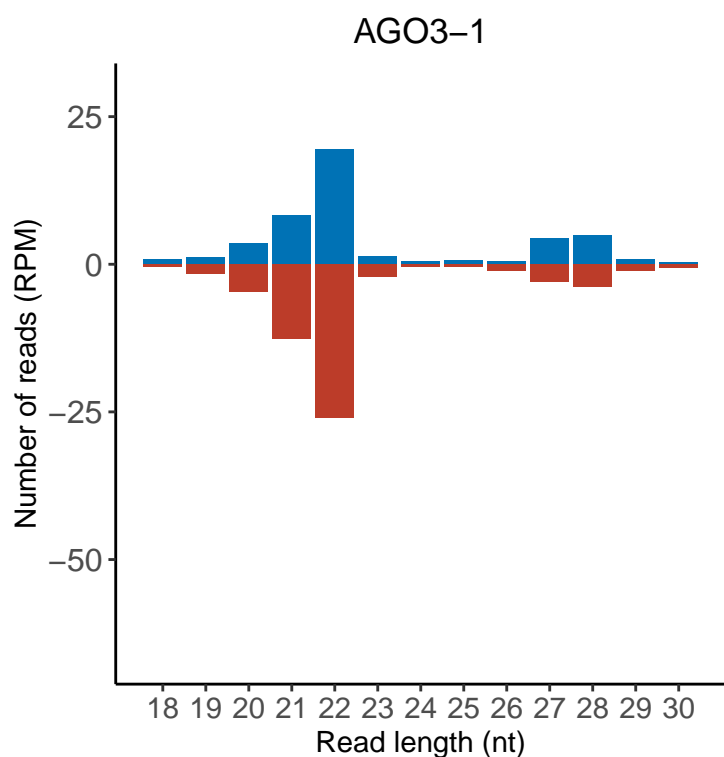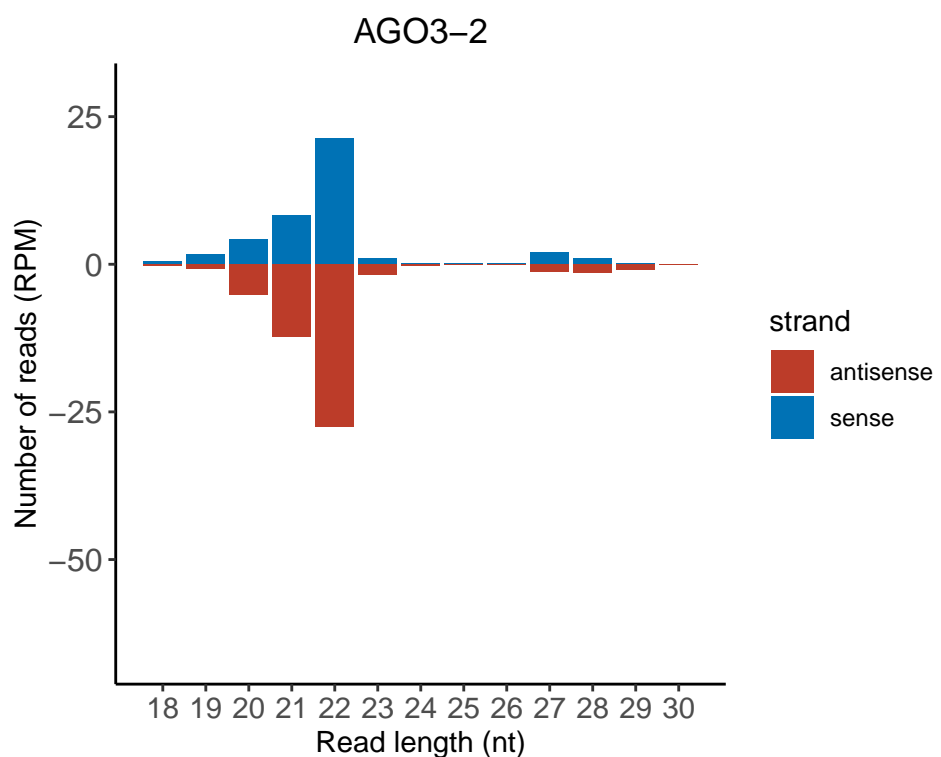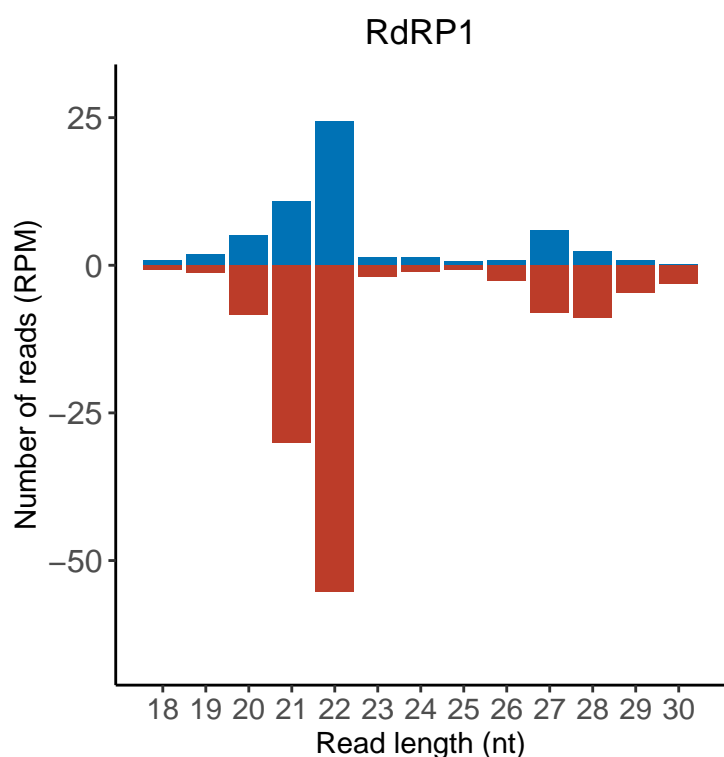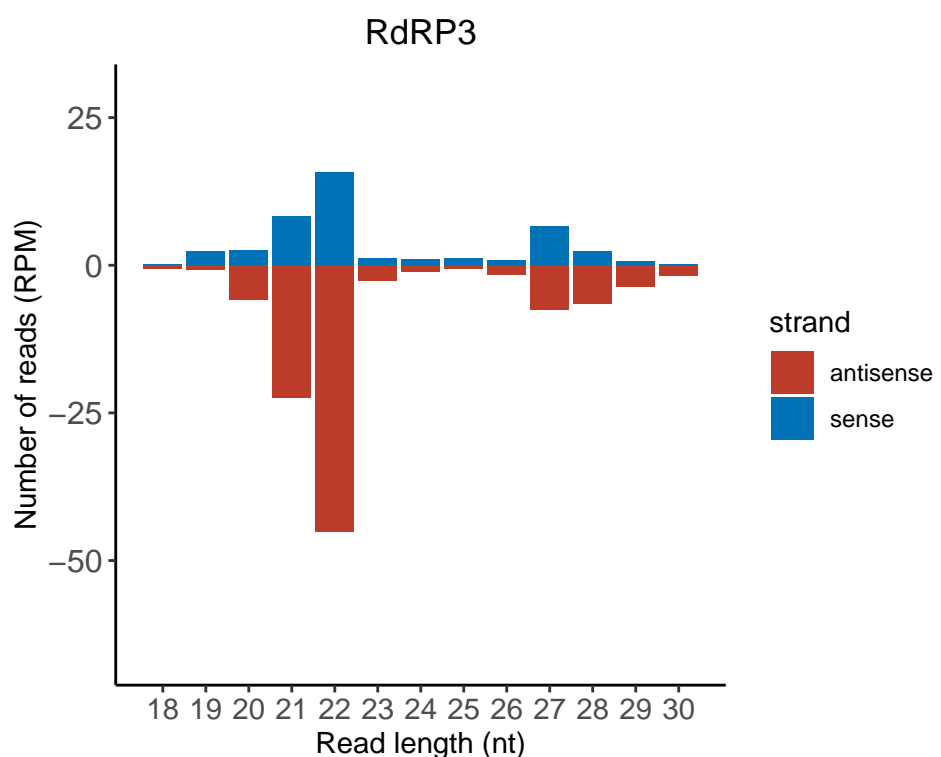

Supplement: S1 Data — On the Normalized and Relative levels pages, normalized read counts (RPM) and relative levels compared to the control (dsGFP) library were used. TEs with more than 50RPM and Coding Genes with more than 3.5RPM on average in KD libraries are included in this website. For viral sequences, reference sequences were generated by assembling sRNA sequences (See the Analysis of sRNAseq data section in Materials and methods.) and size distributions of sRNA reads mapped to each contig are shown. Reads were first grouped by their 5’ nucleotides, and reads of each length were counted. Full tables including TEs and Coding genes with fewer reads are in the “FullTable” folder. (ZIP) [file pone.0281195.s017.zip › SupplementaryData/Links/PDFs/Motif:rnd-1_family-731TE_lengthdistribution.pdf]

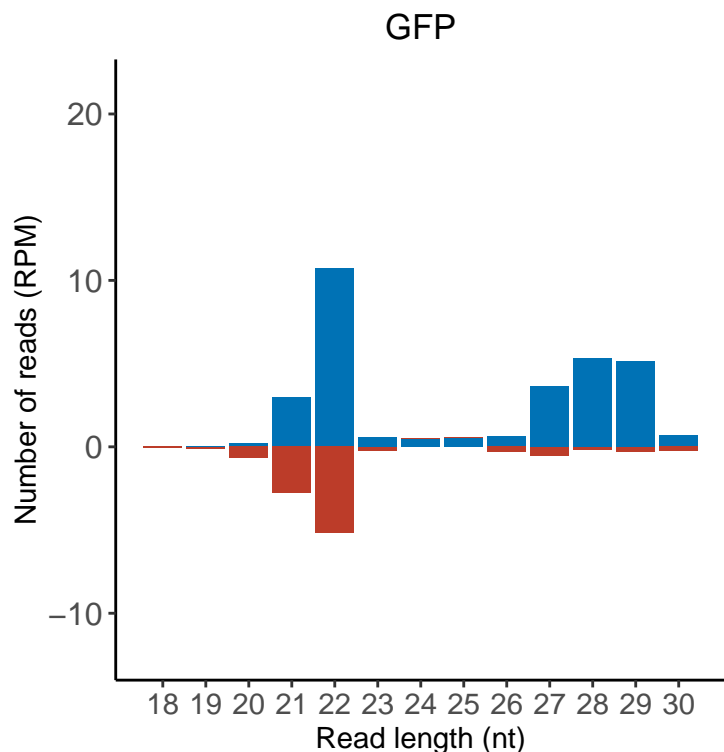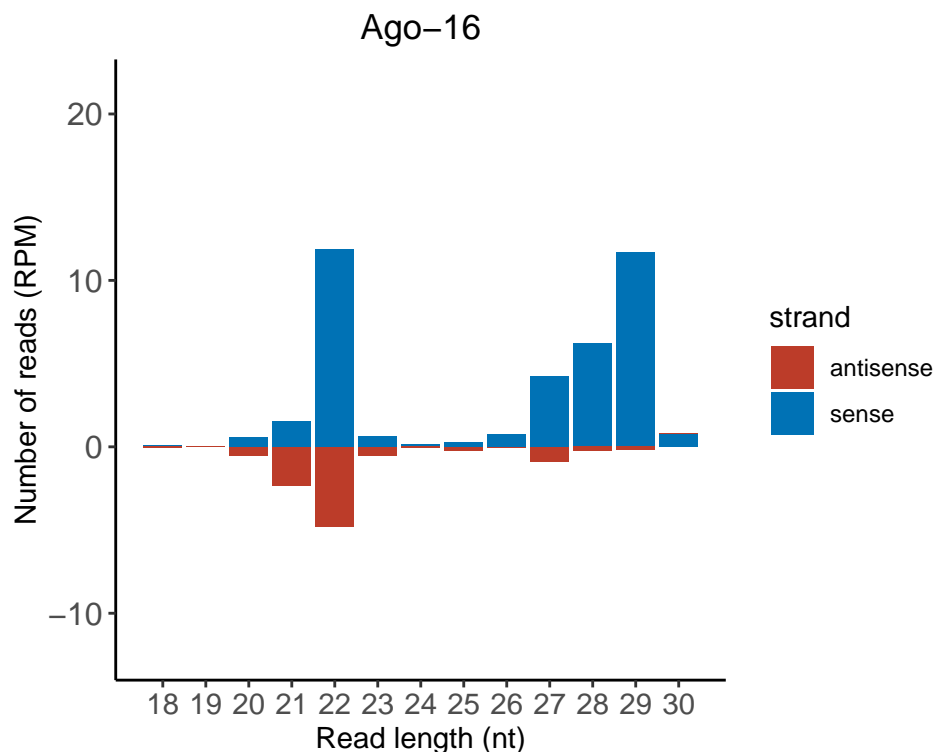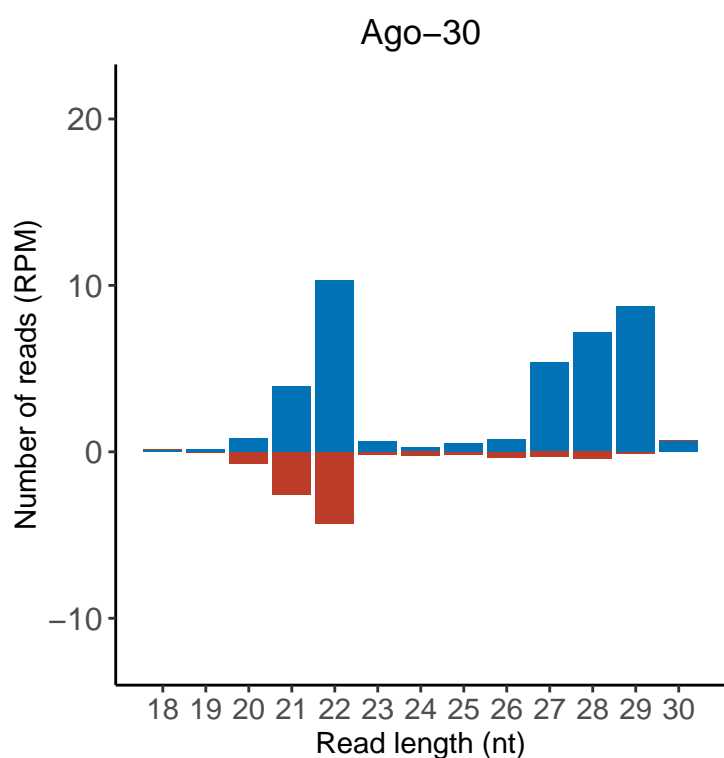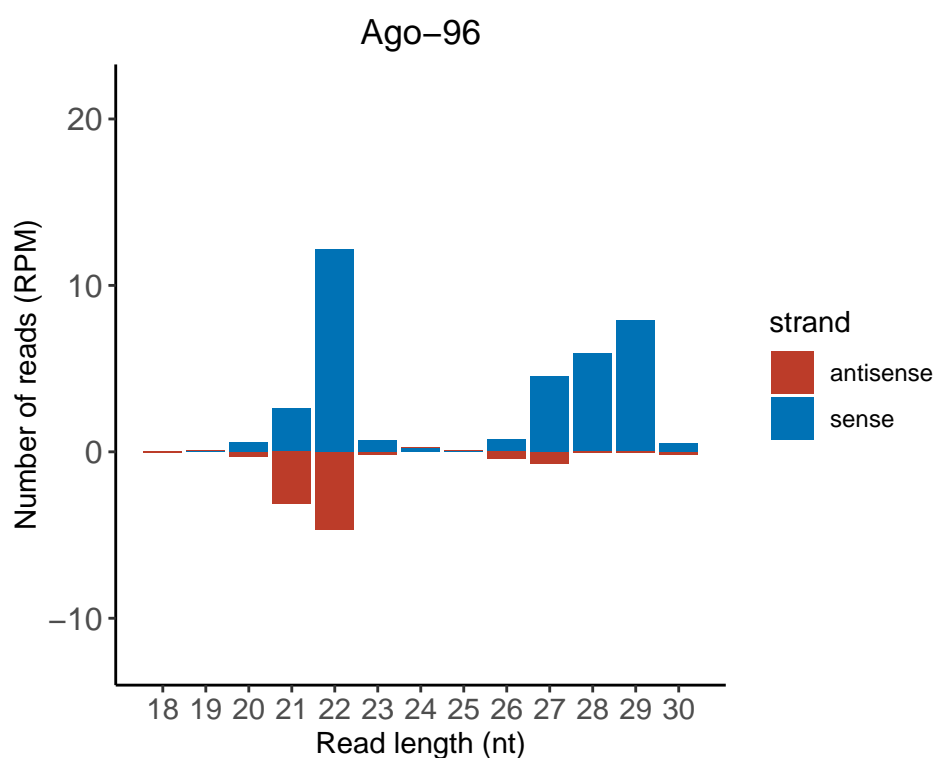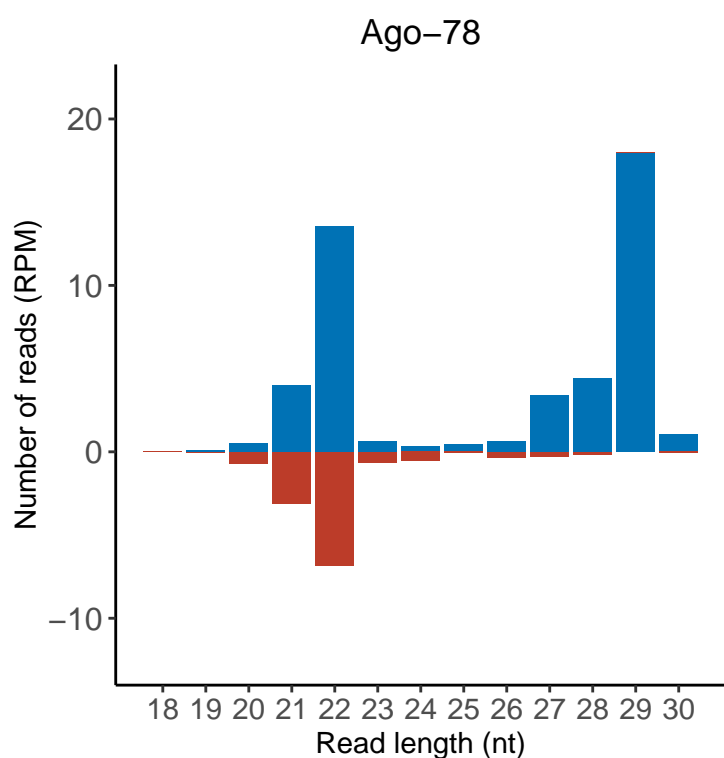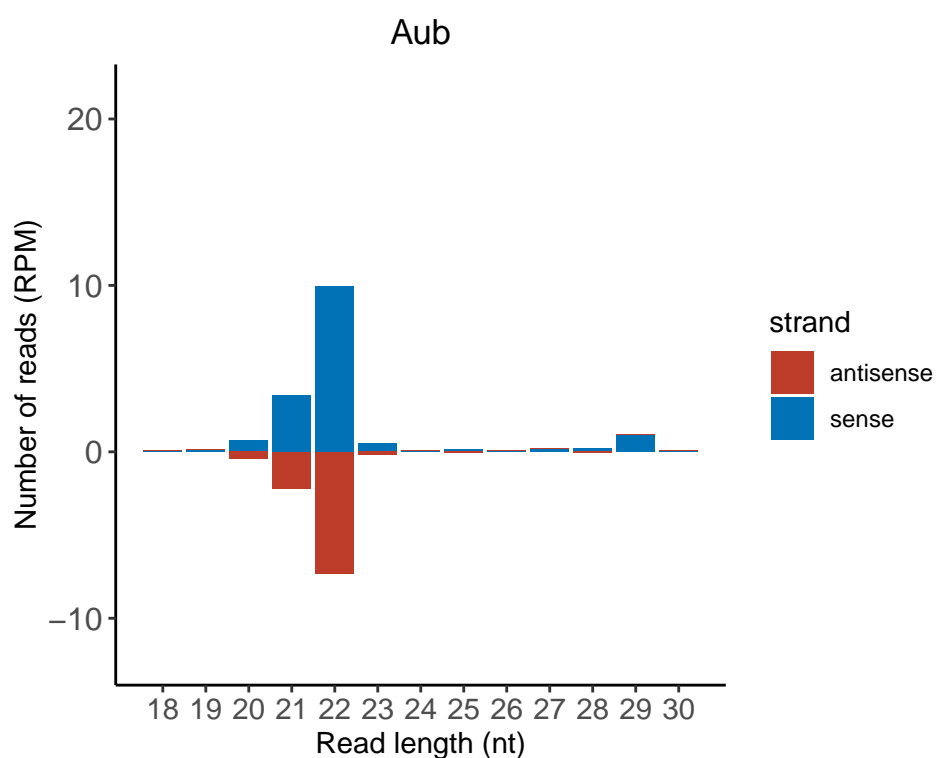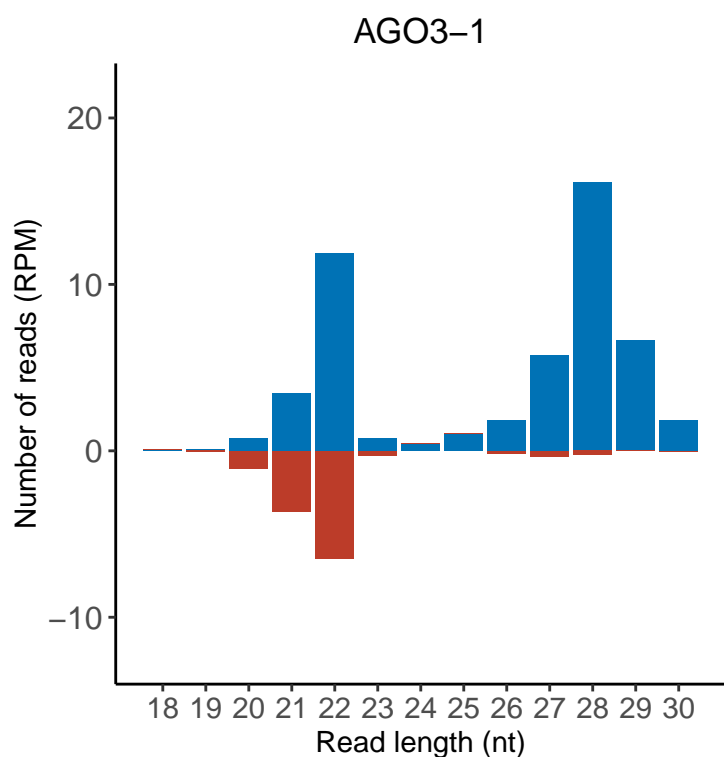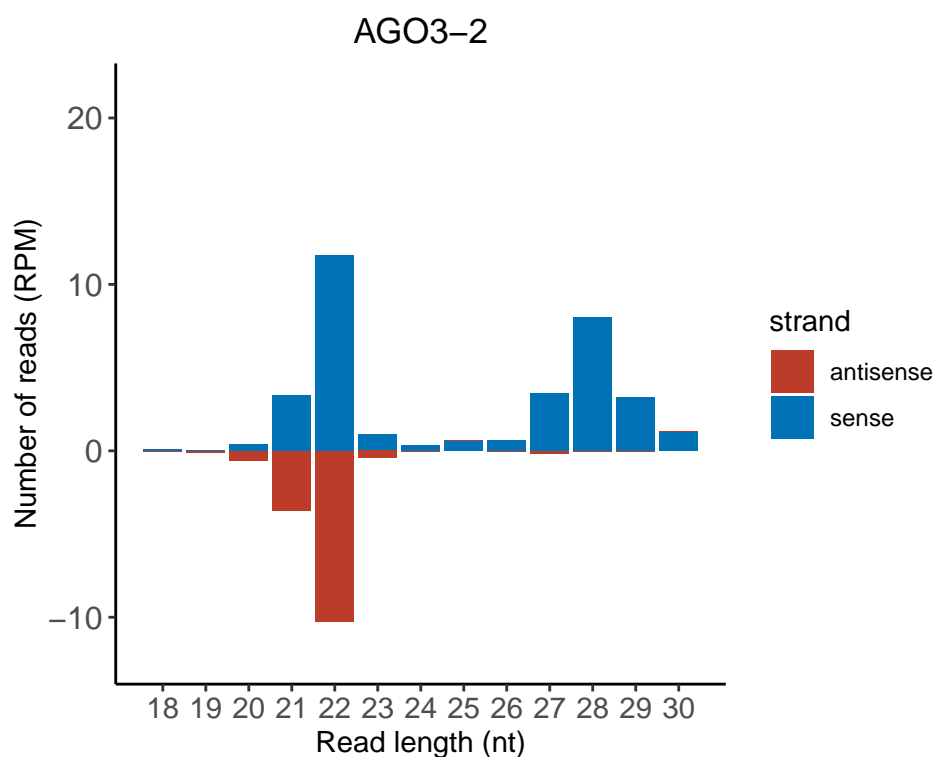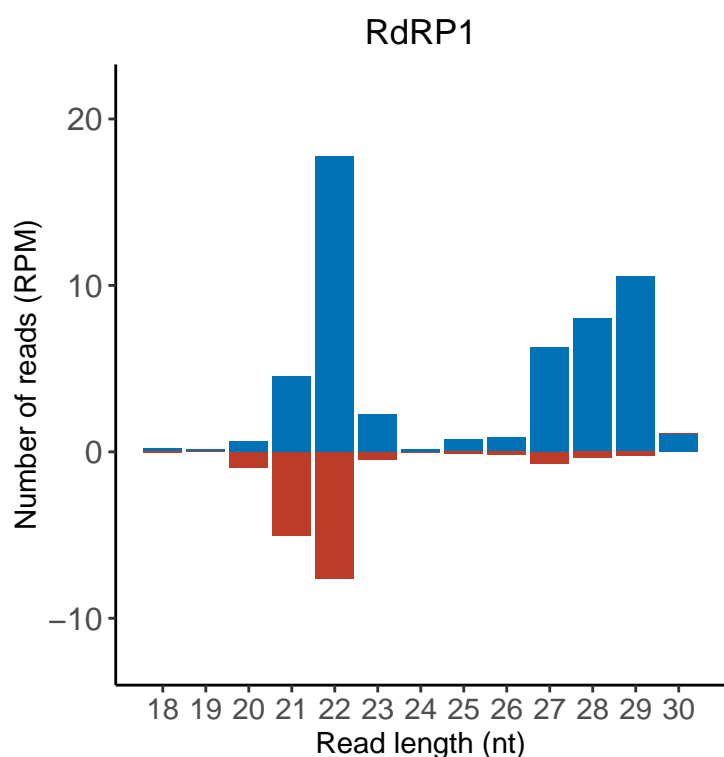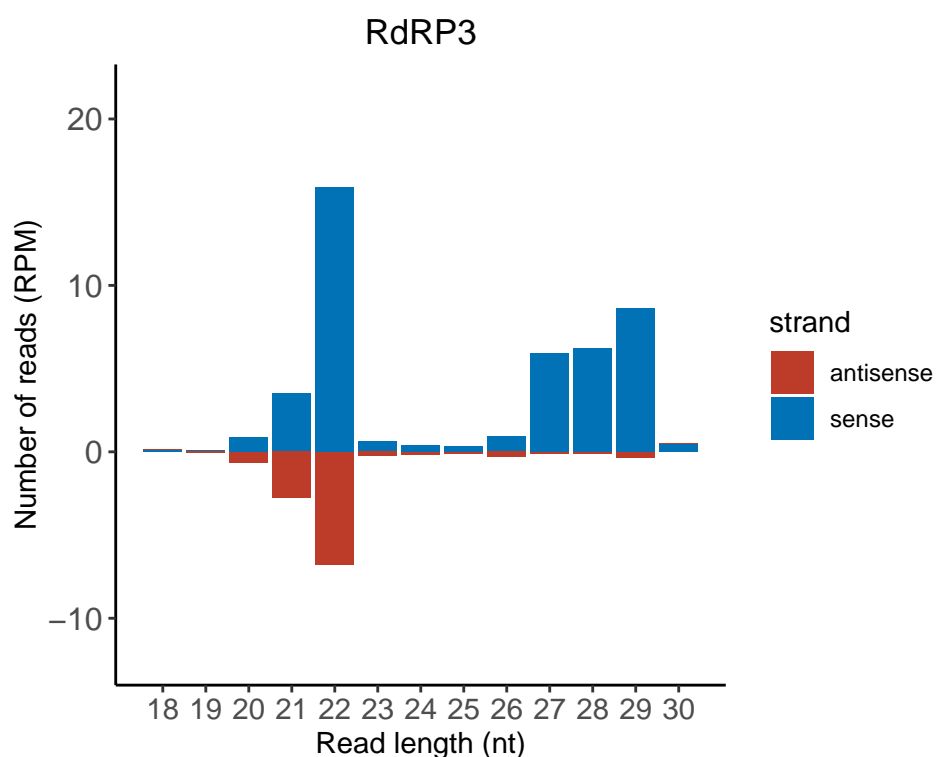

Supplement: S1 Data — On the Normalized and Relative levels pages, normalized read counts (RPM) and relative levels compared to the control (dsGFP) library were used. TEs with more than 50RPM and Coding Genes with more than 3.5RPM on average in KD libraries are included in this website. For viral sequences, reference sequences were generated by assembling sRNA sequences (See the Analysis of sRNAseq data section in Materials and methods.) and size distributions of sRNA reads mapped to each contig are shown. Reads were first grouped by their 5’ nucleotides, and reads of each length were counted. Full tables including TEs and Coding genes with fewer reads are in the “FullTable” folder. (ZIP) [file pone.0281195.s017.zip › SupplementaryData/Links/PDFs/Motif:rnd-1_family-809TE_lengthdistribution.pdf]

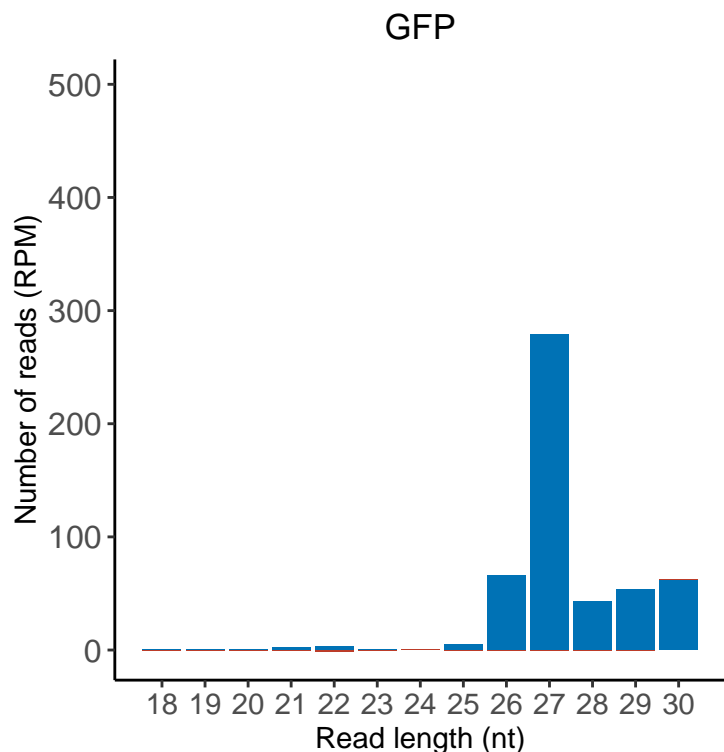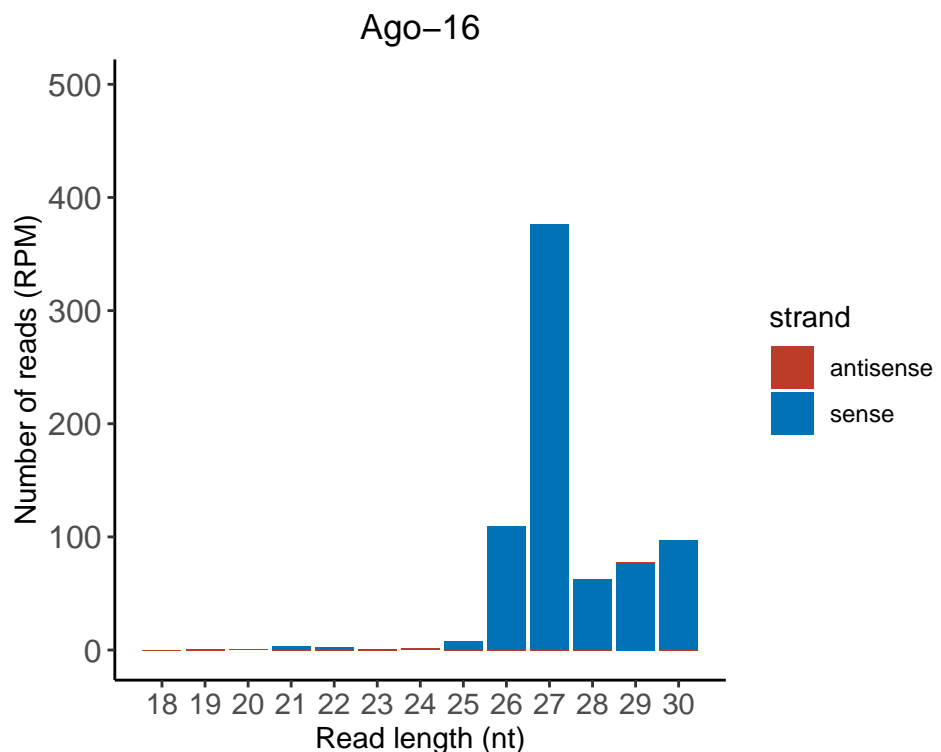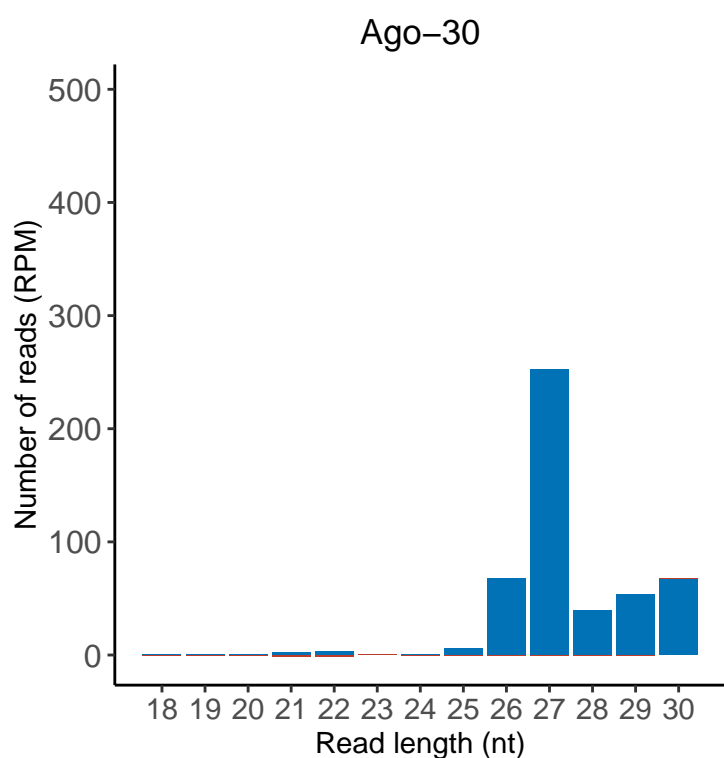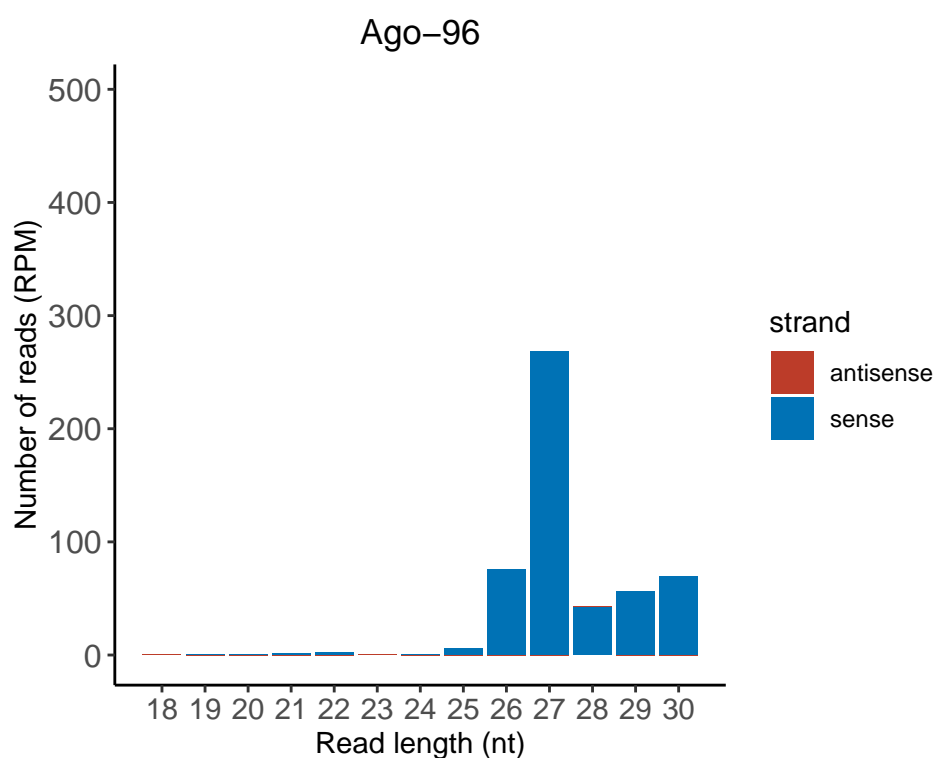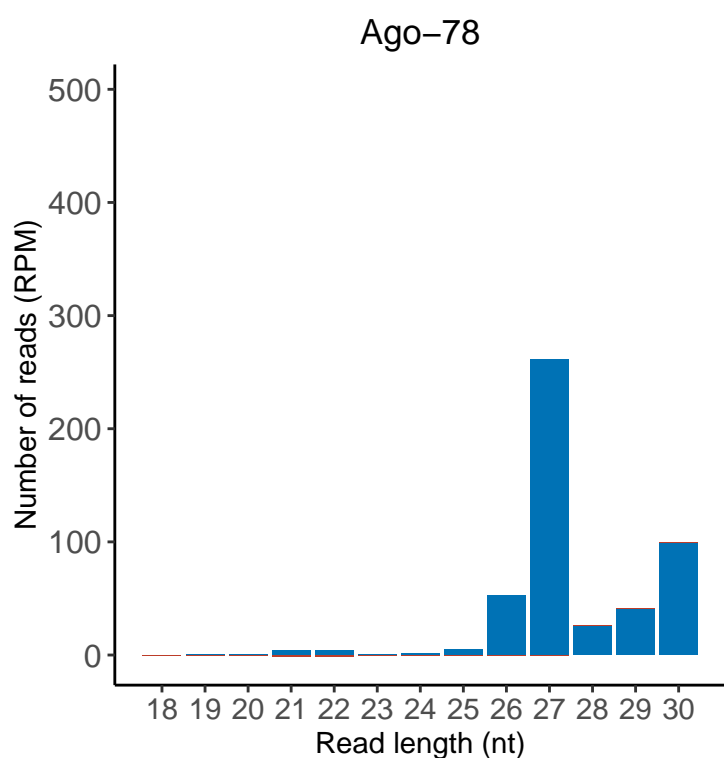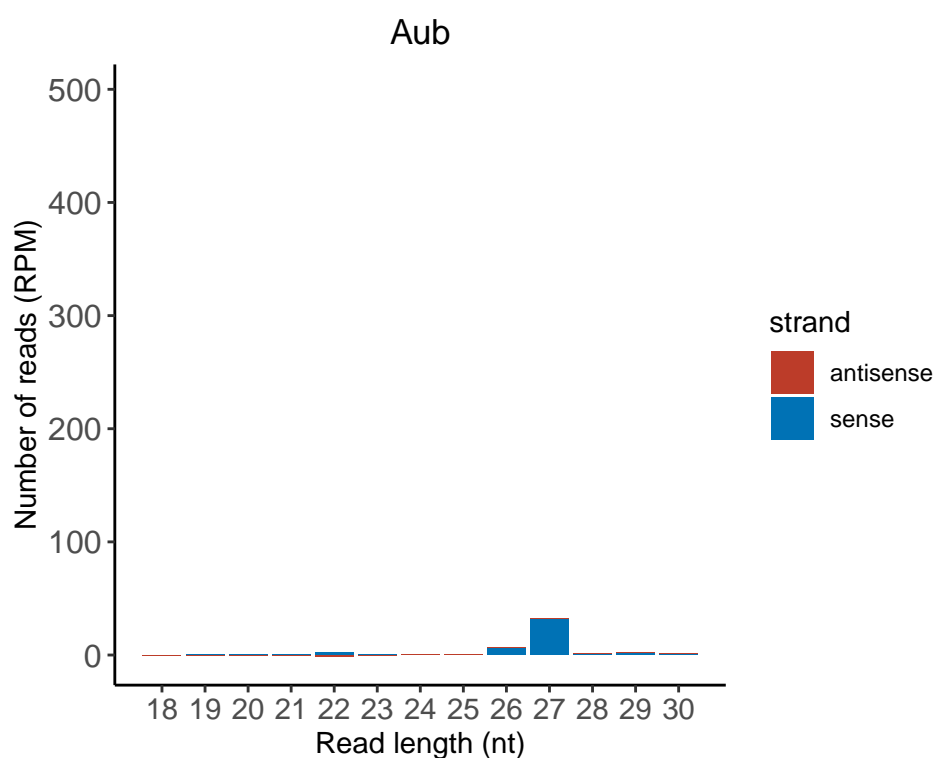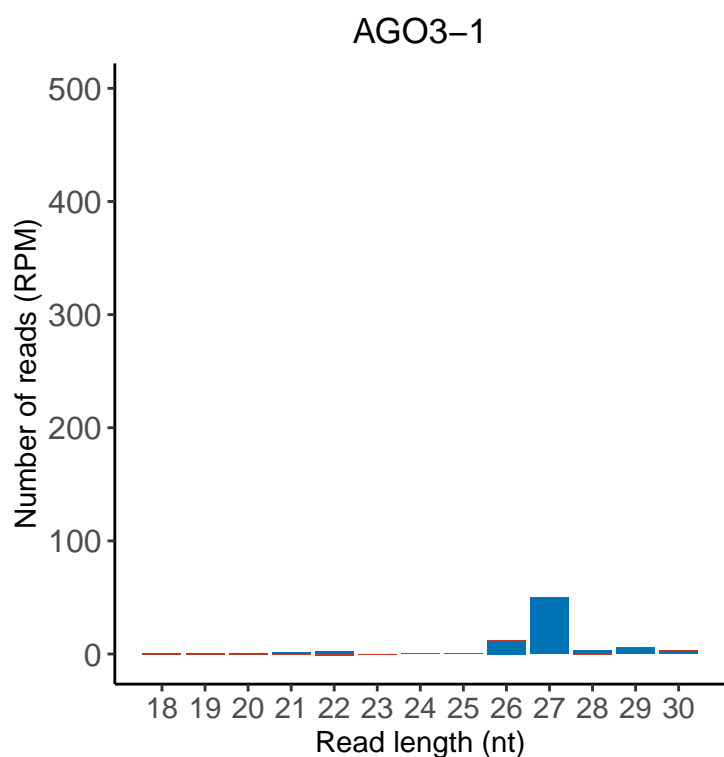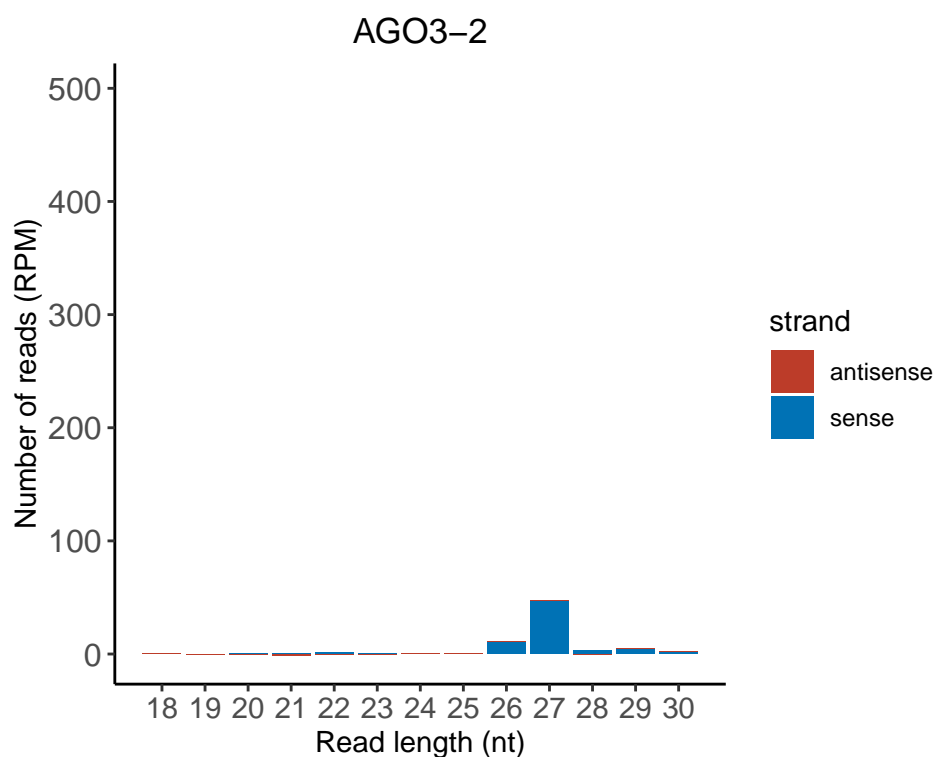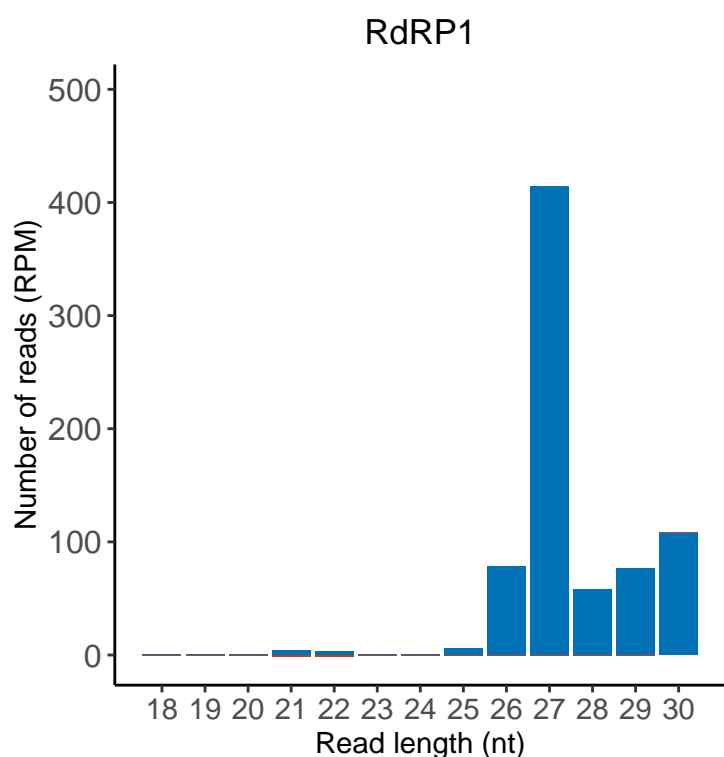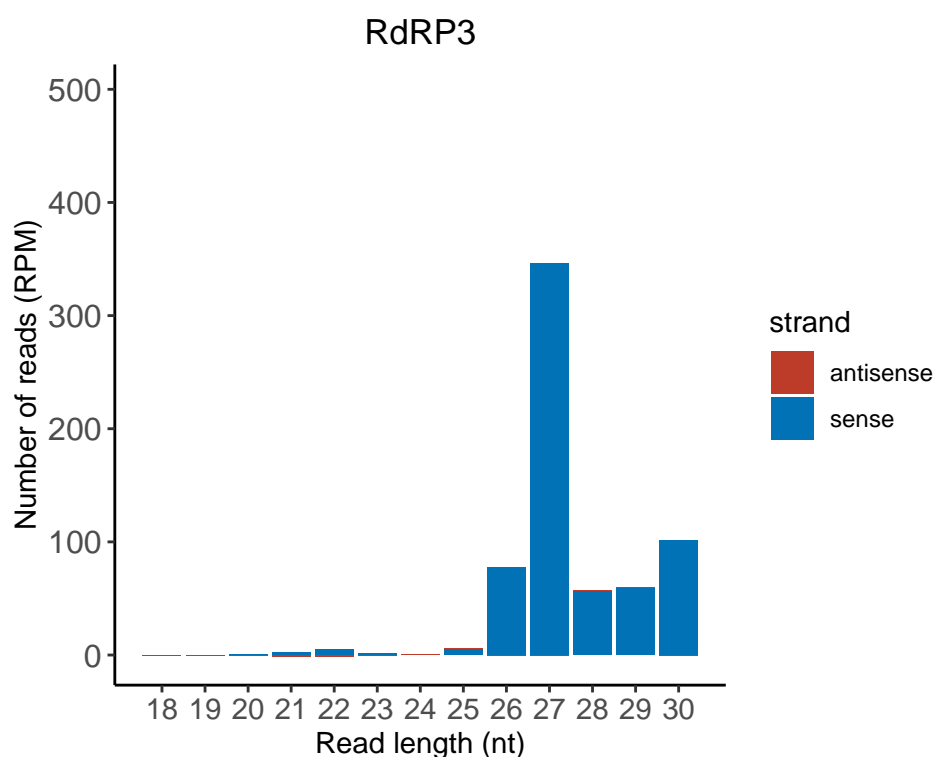

Supplement: S1 Data — On the Normalized and Relative levels pages, normalized read counts (RPM) and relative levels compared to the control (dsGFP) library were used. TEs with more than 50RPM and Coding Genes with more than 3.5RPM on average in KD libraries are included in this website. For viral sequences, reference sequences were generated by assembling sRNA sequences (See the Analysis of sRNAseq data section in Materials and methods.) and size distributions of sRNA reads mapped to each contig are shown. Reads were first grouped by their 5’ nucleotides, and reads of each length were counted. Full tables including TEs and Coding genes with fewer reads are in the “FullTable” folder. (ZIP) [file pone.0281195.s017.zip › SupplementaryData/Links/PDFs/Motif:rnd-1_family-1070TE_lengthdistribution.pdf]

GFP

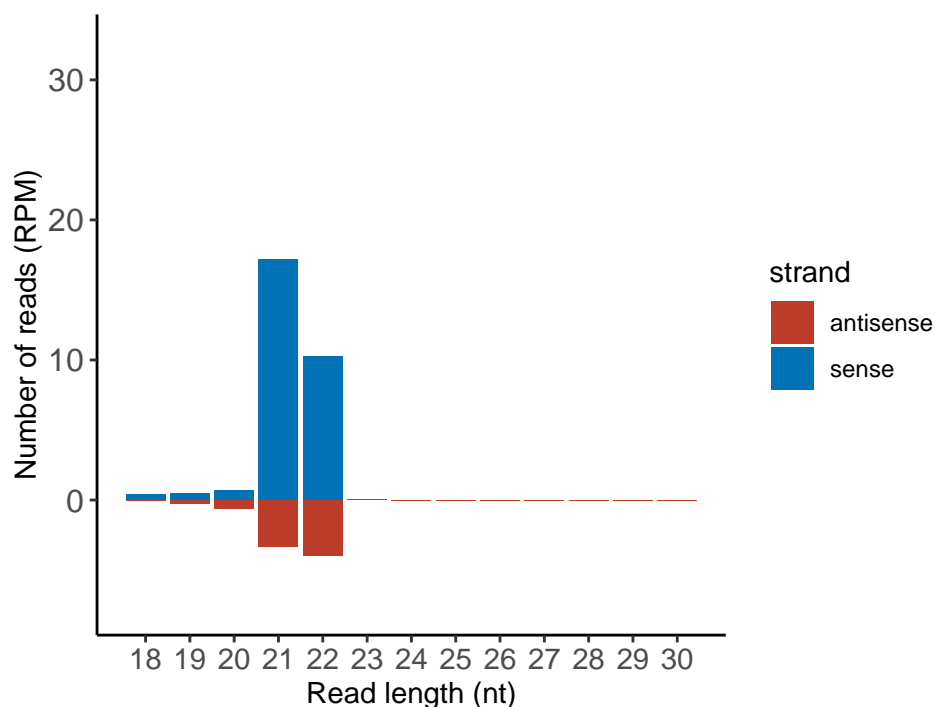

Ago-16

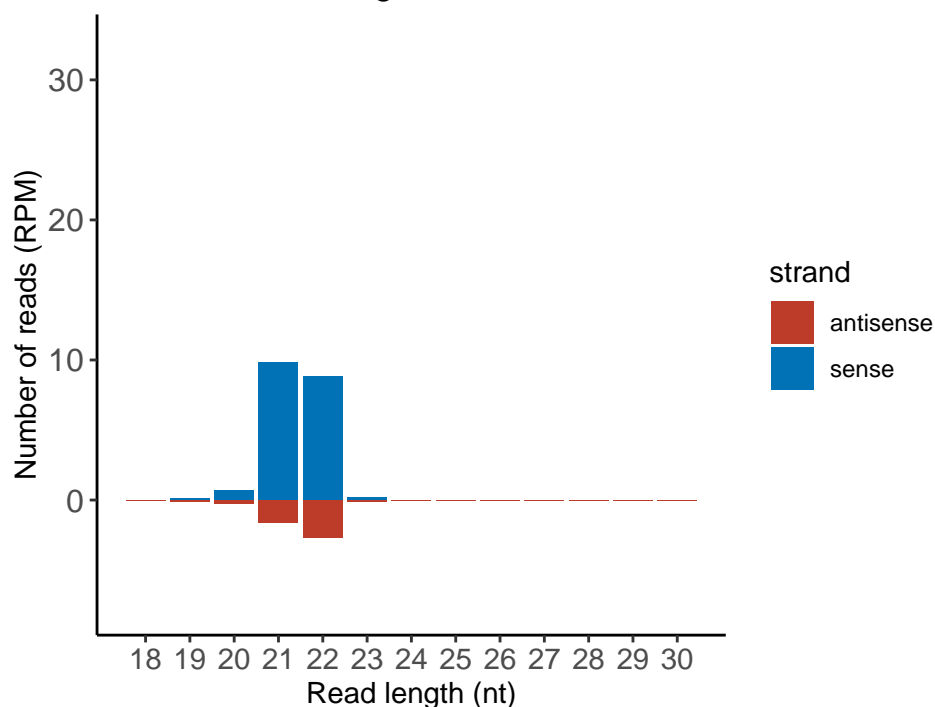

Ago-30

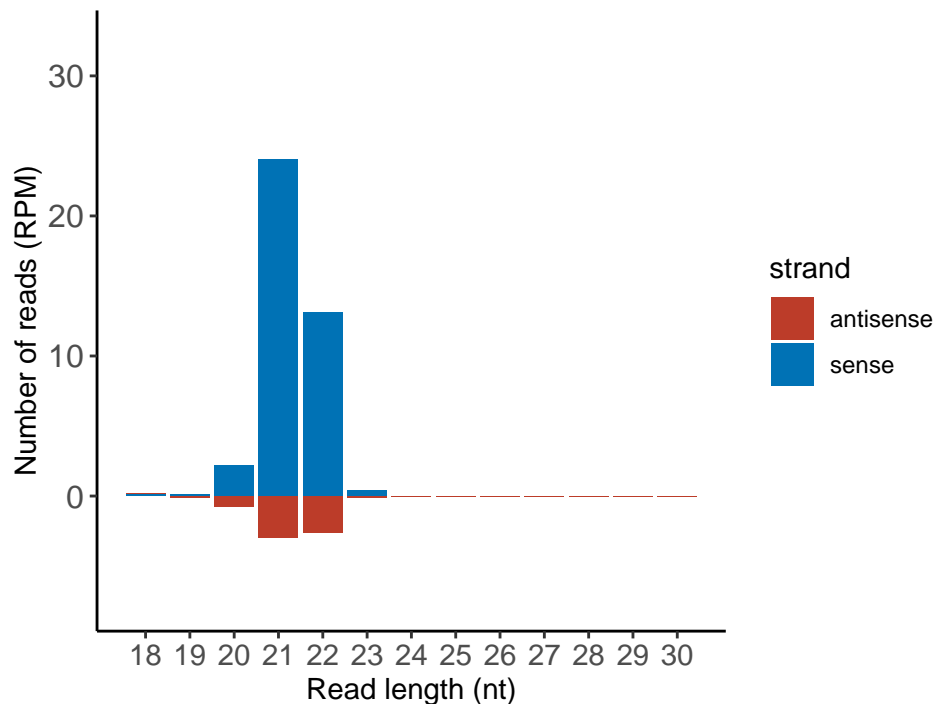

Ago-96

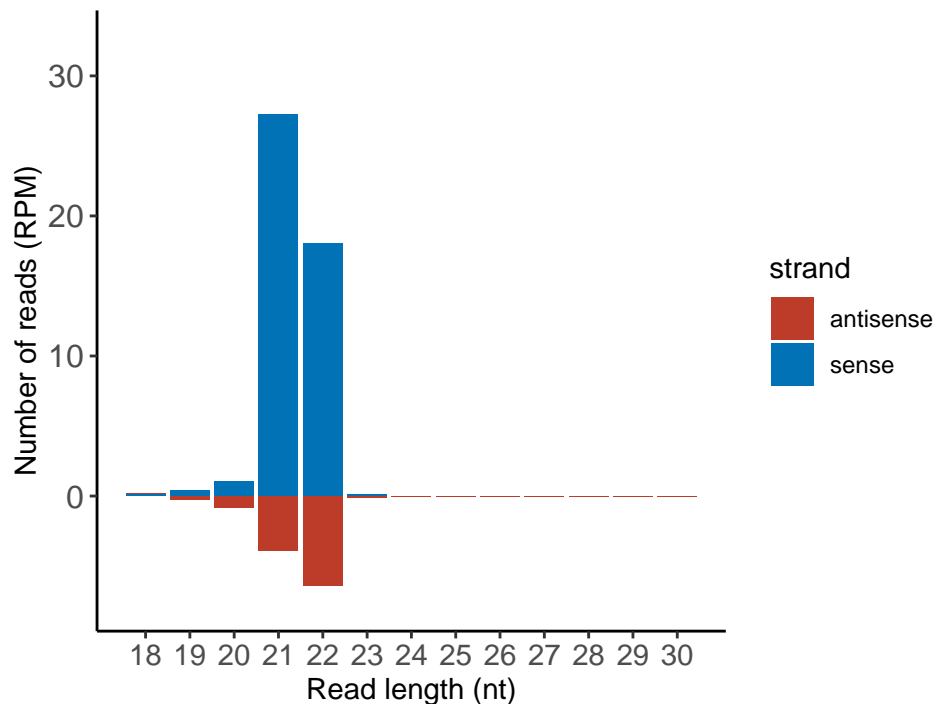

Ago-78

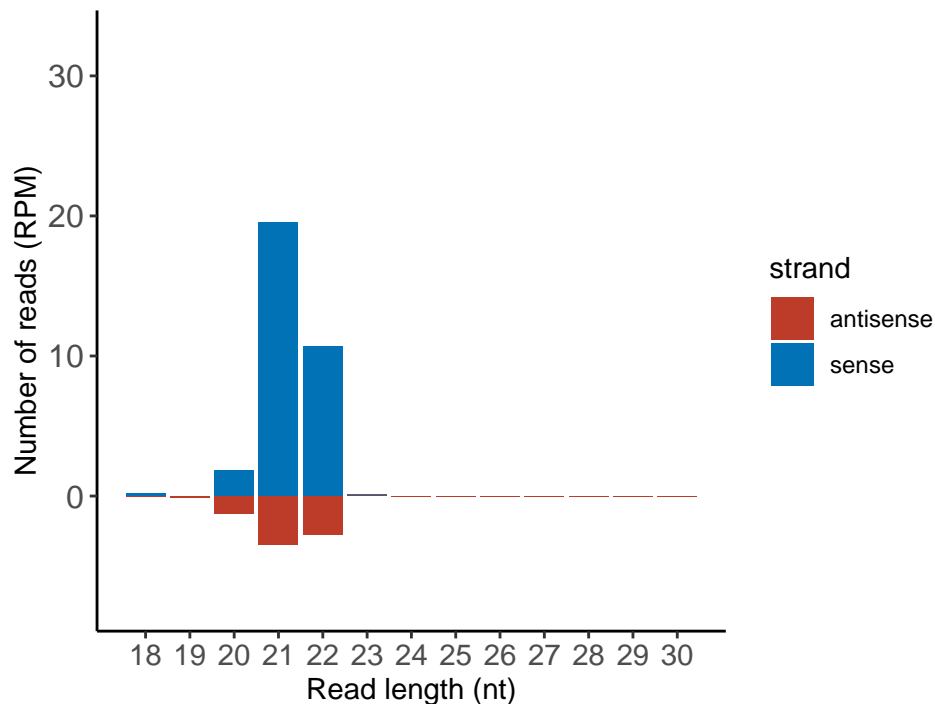

Aub

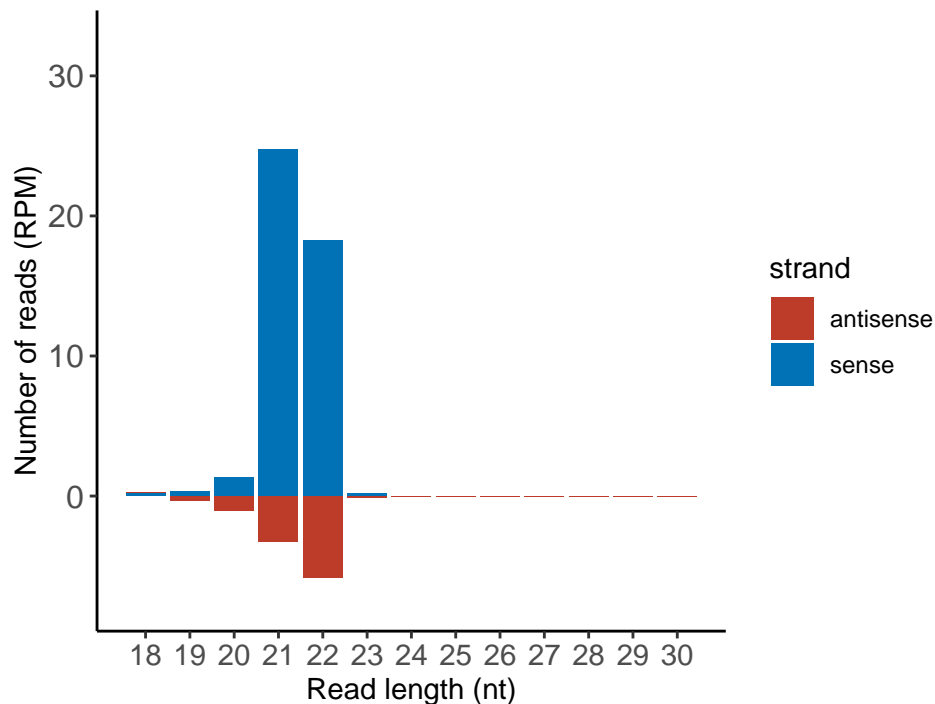

AGO3-1

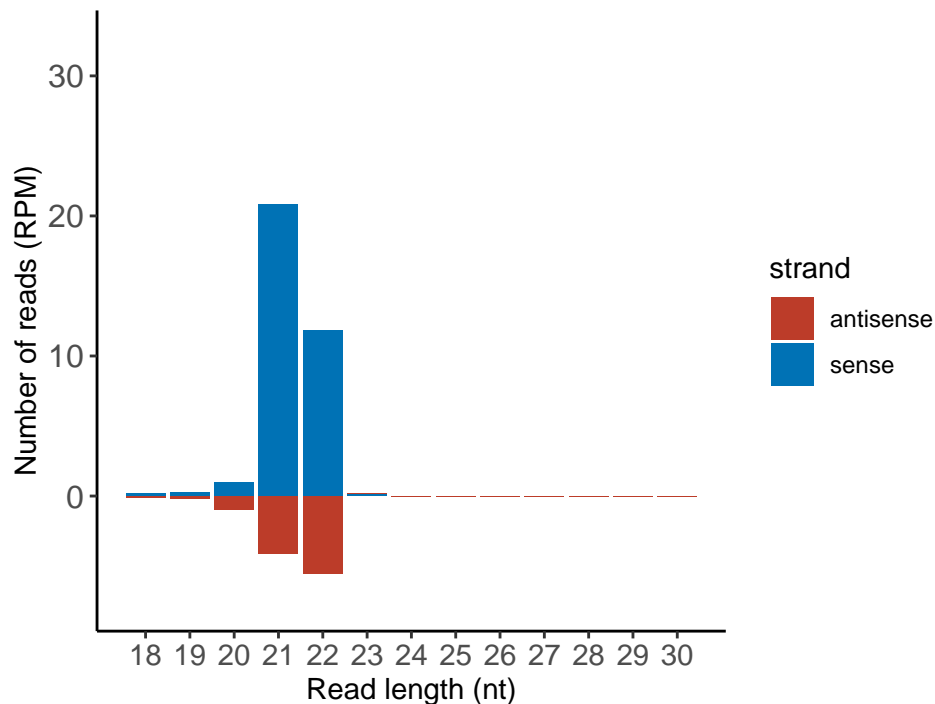

AGO3-2

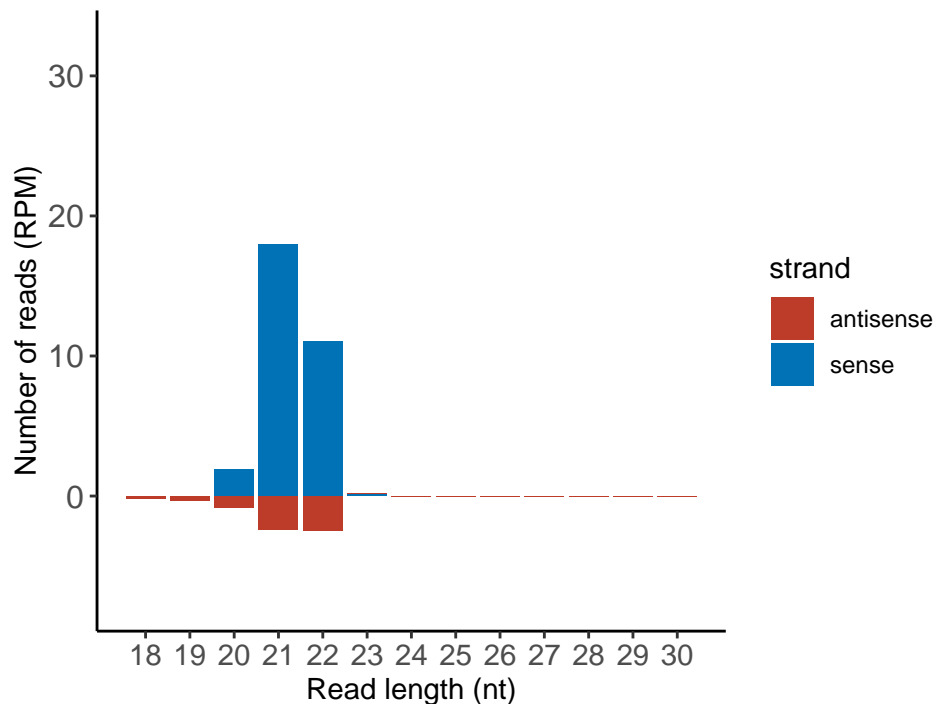

RdRP1

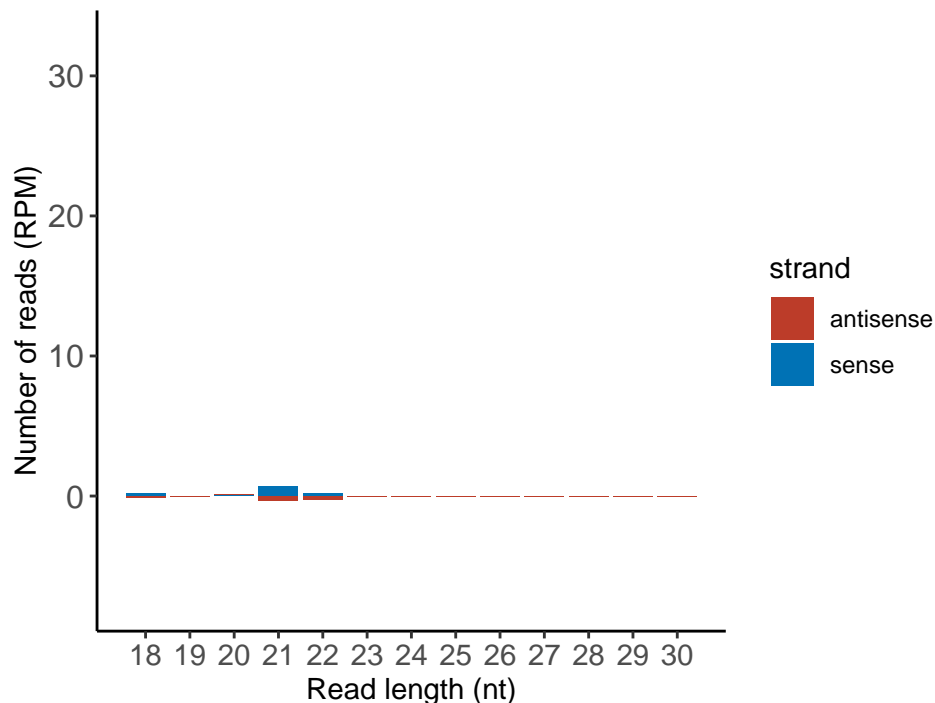

RdRP3

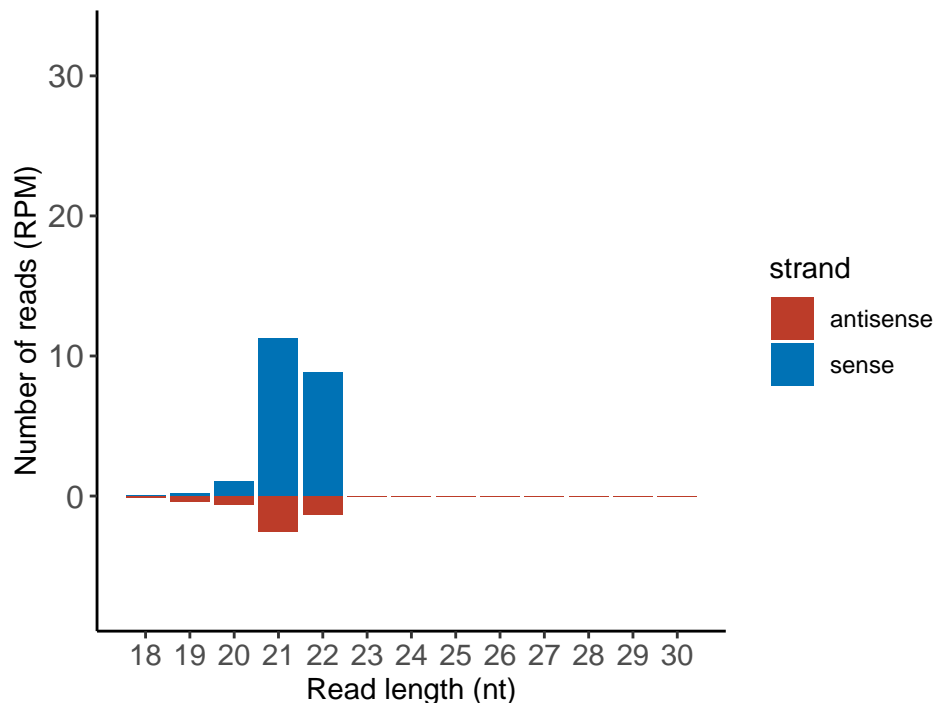

Supplement: S1 Data — On the Normalized and Relative levels pages, normalized read counts (RPM) and relative levels compared to the control (dsGFP) library were used. TEs with more than 50RPM and Coding Genes with more than 3.5RPM on average in KD libraries are included in this website. For viral sequences, reference sequences were generated by assembling sRNA sequences (See the Analysis of sRNAseq data section in Materials and methods.) and size distributions of sRNA reads mapped to each contig are shown. Reads were first grouped by their 5’ nucleotides, and reads of each length were counted. Full tables including TEs and Coding genes with fewer reads are in the “FullTable” folder. (ZIP) [file pone.0281195.s017.zip › SupplementaryData/Links/PDFs/ISCI007332.RACDS_lengthdistribution.pdf]

GFP

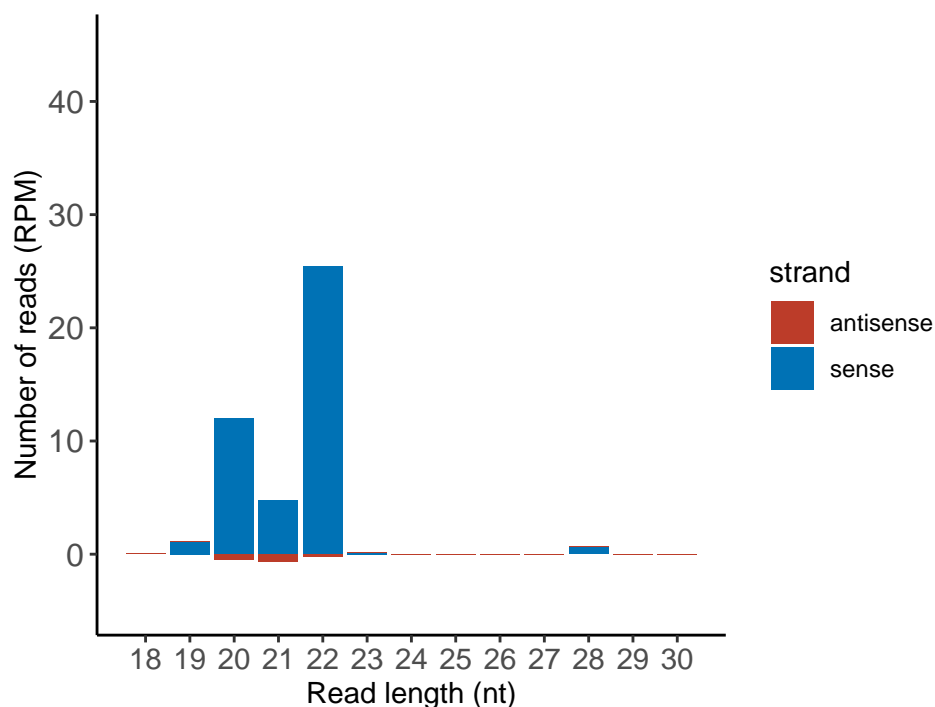

Ago-16

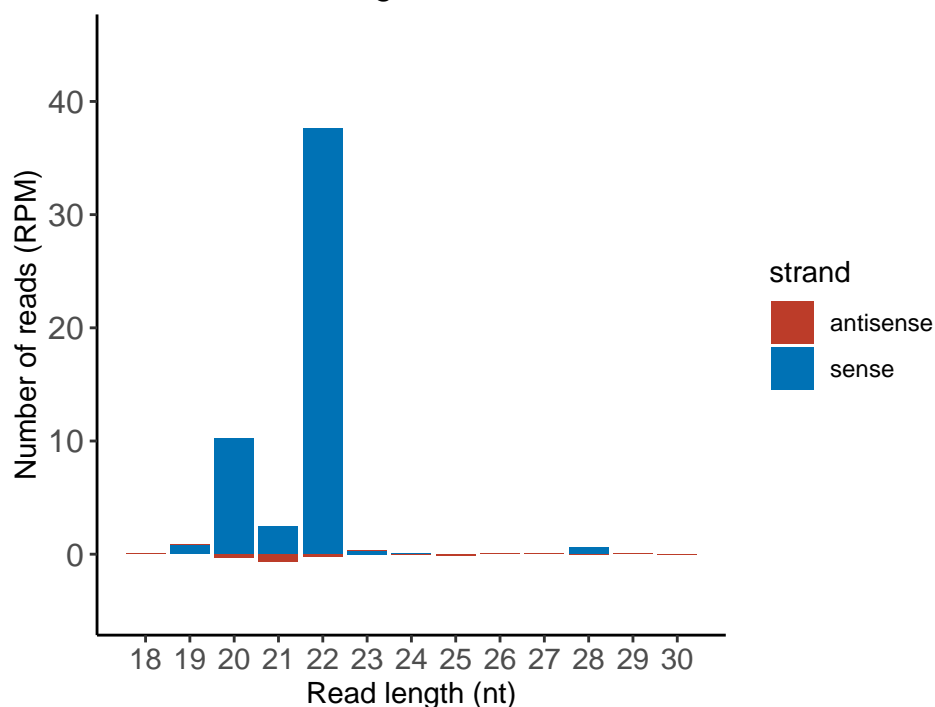

Ago-30

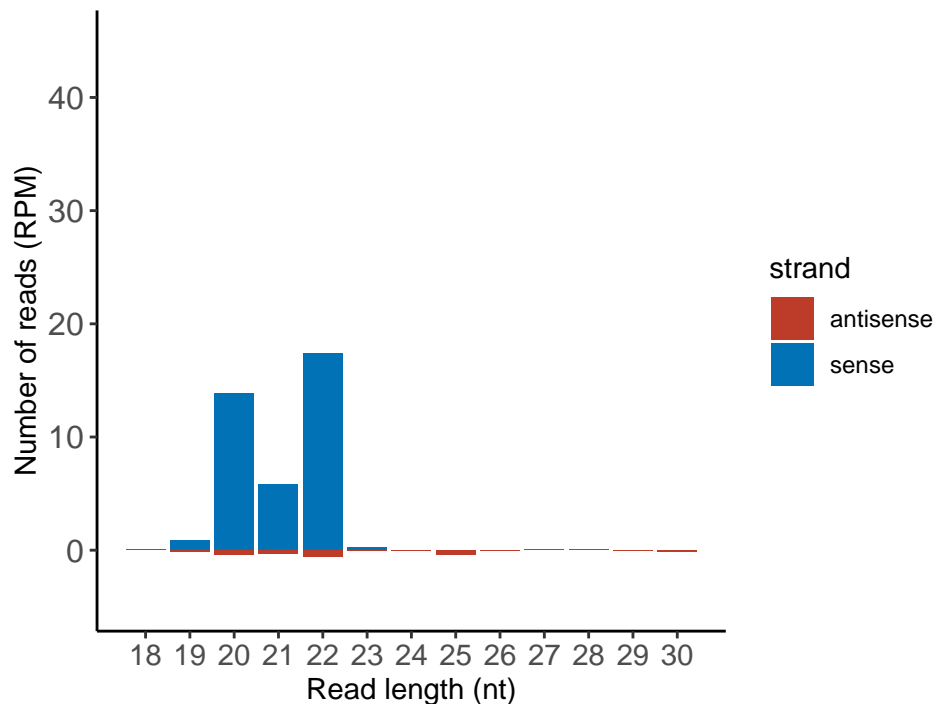

Ago-96

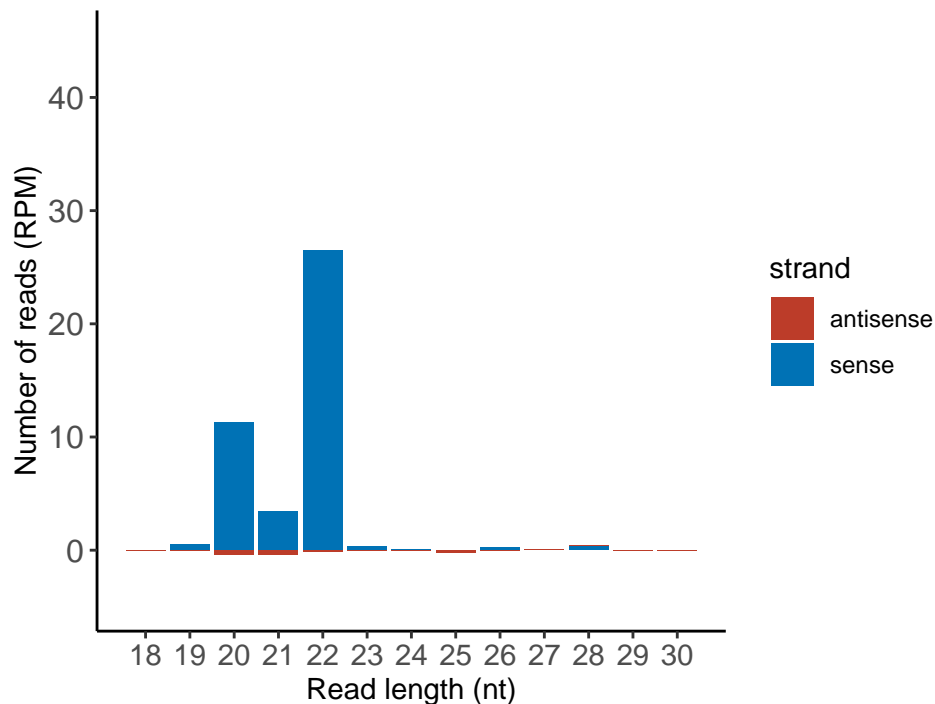

Ago-78

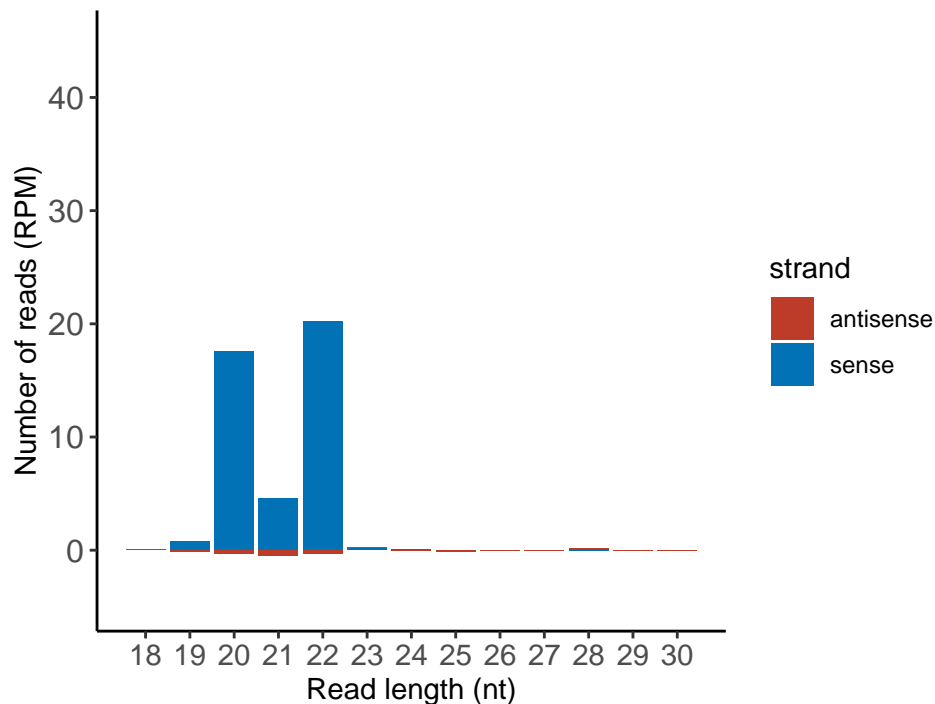

Aub

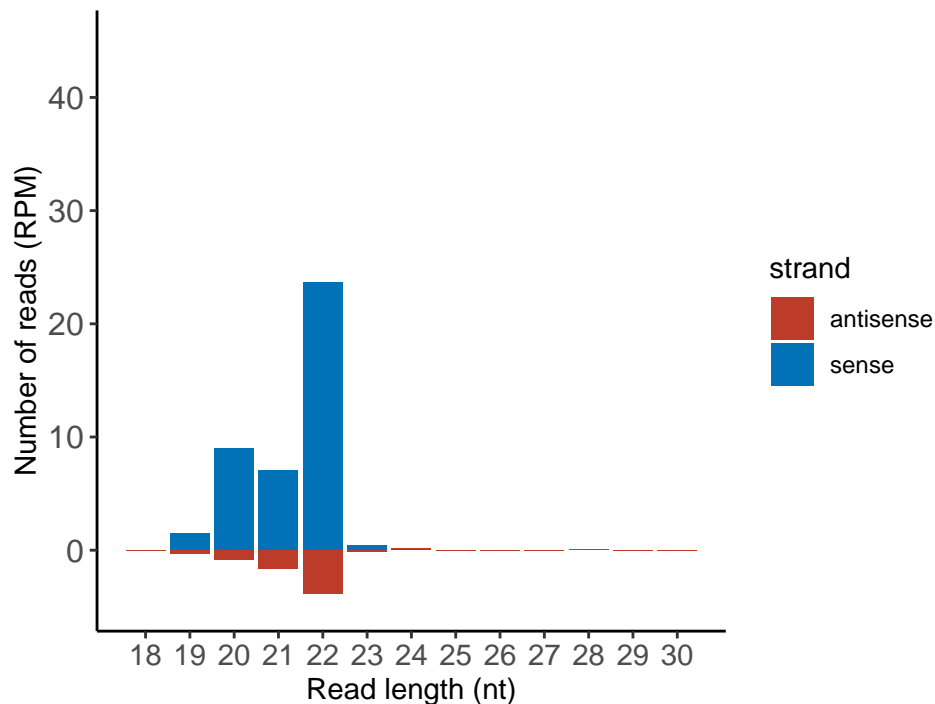

AGO3-1

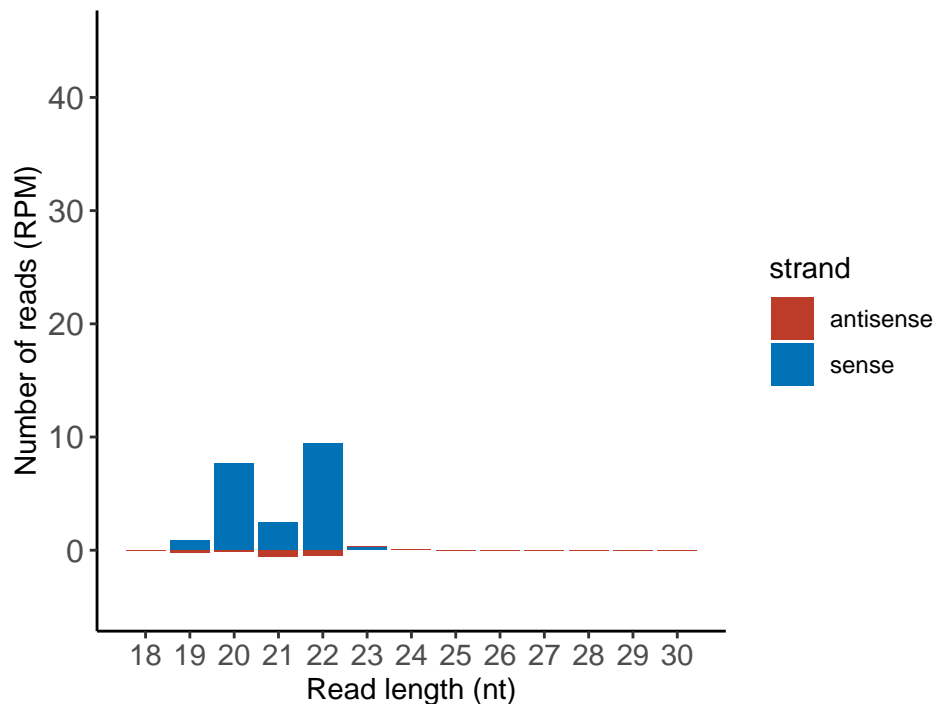

AGO3-2

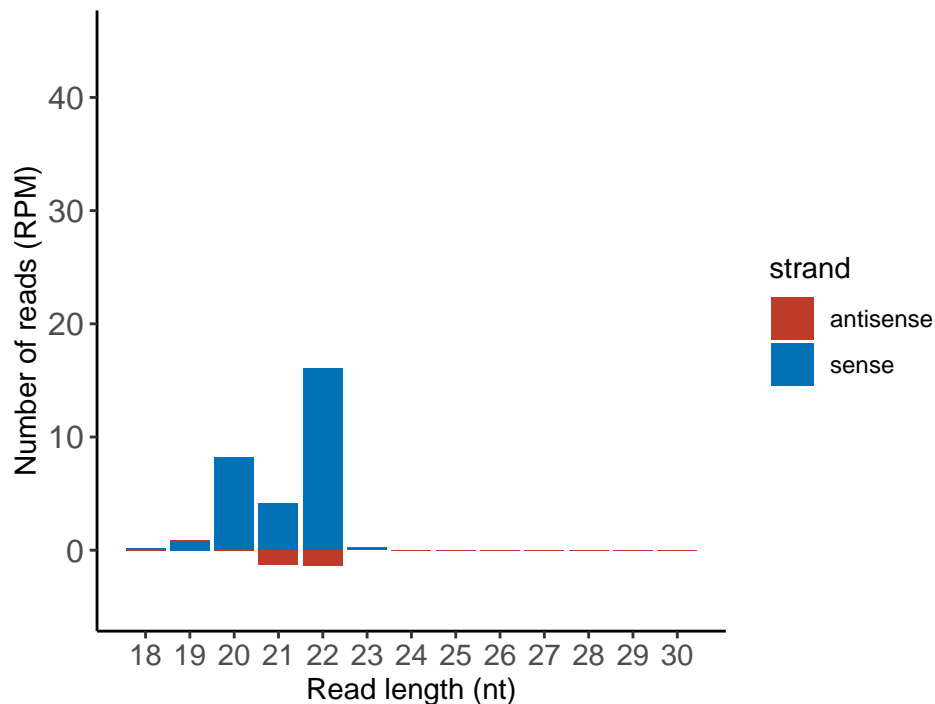

RdRP1

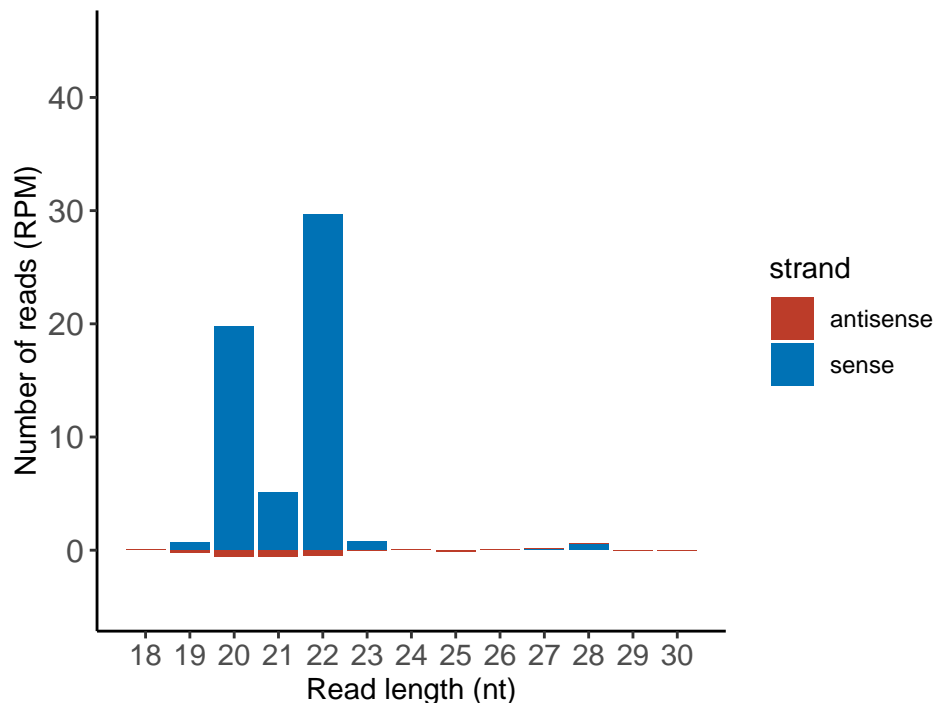

RdRP3

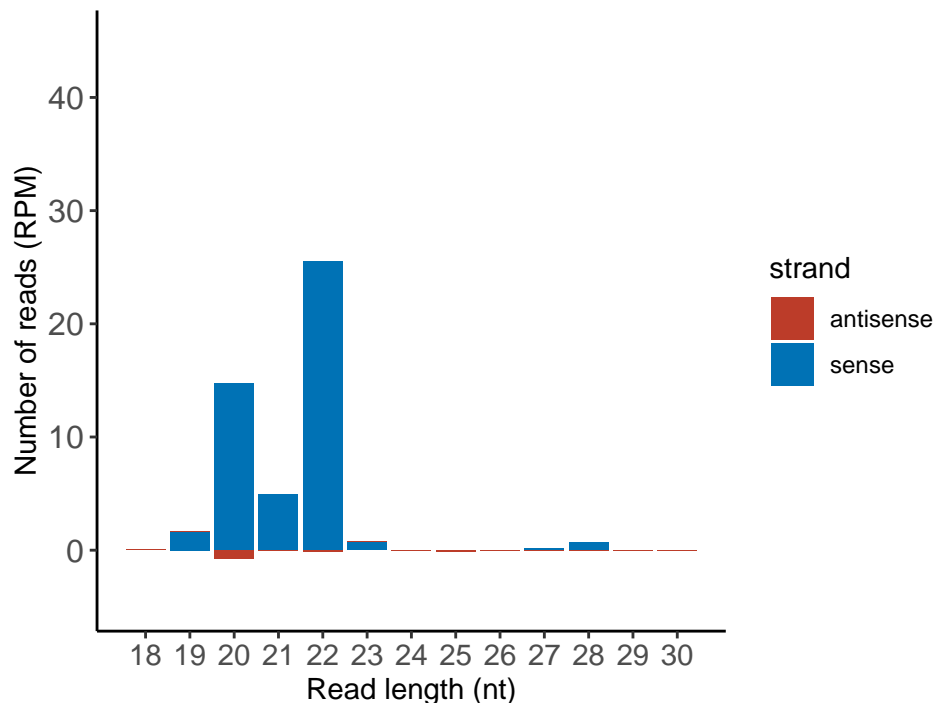

Supplement: S1 Data — On the Normalized and Relative levels pages, normalized read counts (RPM) and relative levels compared to the control (dsGFP) library were used. TEs with more than 50RPM and Coding Genes with more than 3.5RPM on average in KD libraries are included in this website. For viral sequences, reference sequences were generated by assembling sRNA sequences (See the Analysis of sRNAseq data section in Materials and methods.) and size distributions of sRNA reads mapped to each contig are shown. Reads were first grouped by their 5’ nucleotides, and reads of each length were counted. Full tables including TEs and Coding genes with fewer reads are in the “FullTable” folder. (ZIP) [file pone.0281195.s017.zip › SupplementaryData/Links/PDFs/ISCI018019.RACDS_lengthdistribution.pdf]

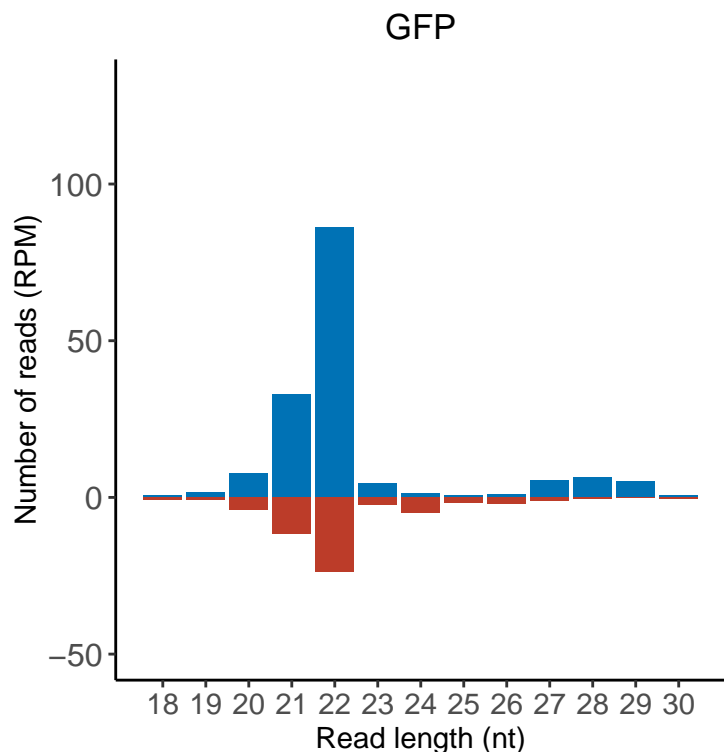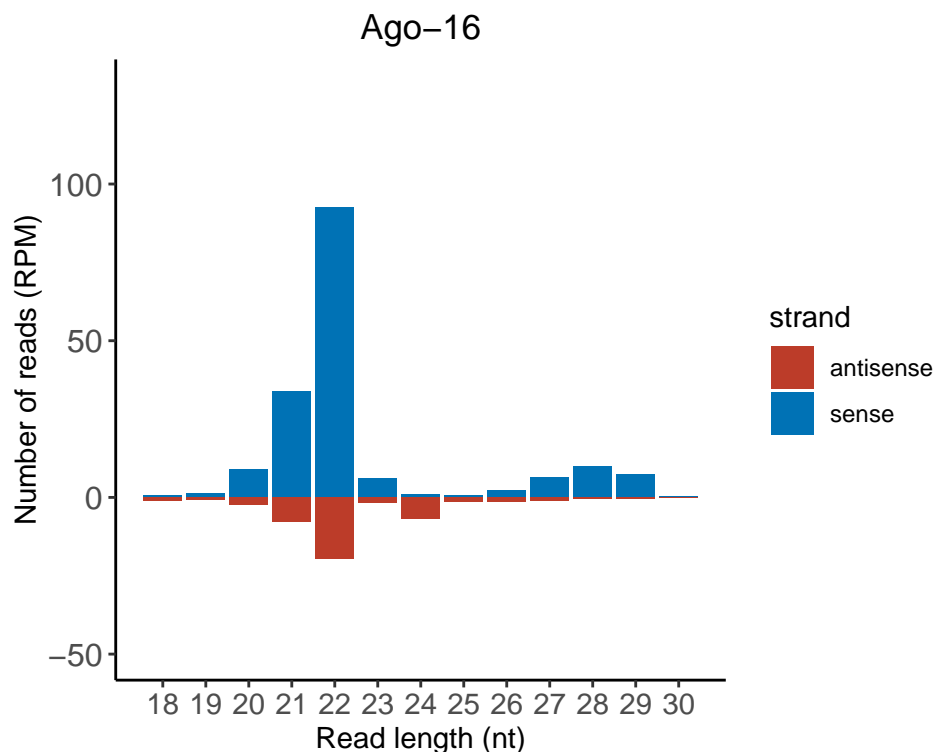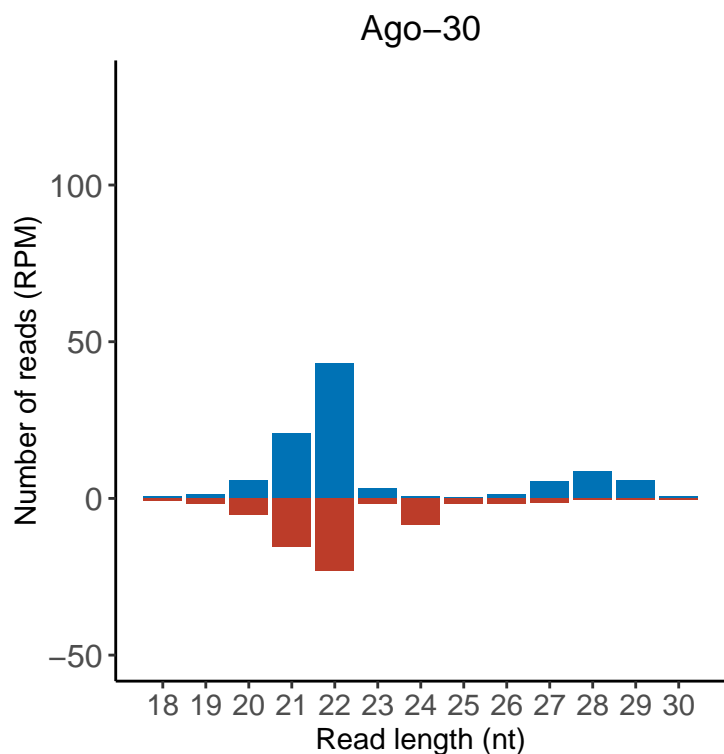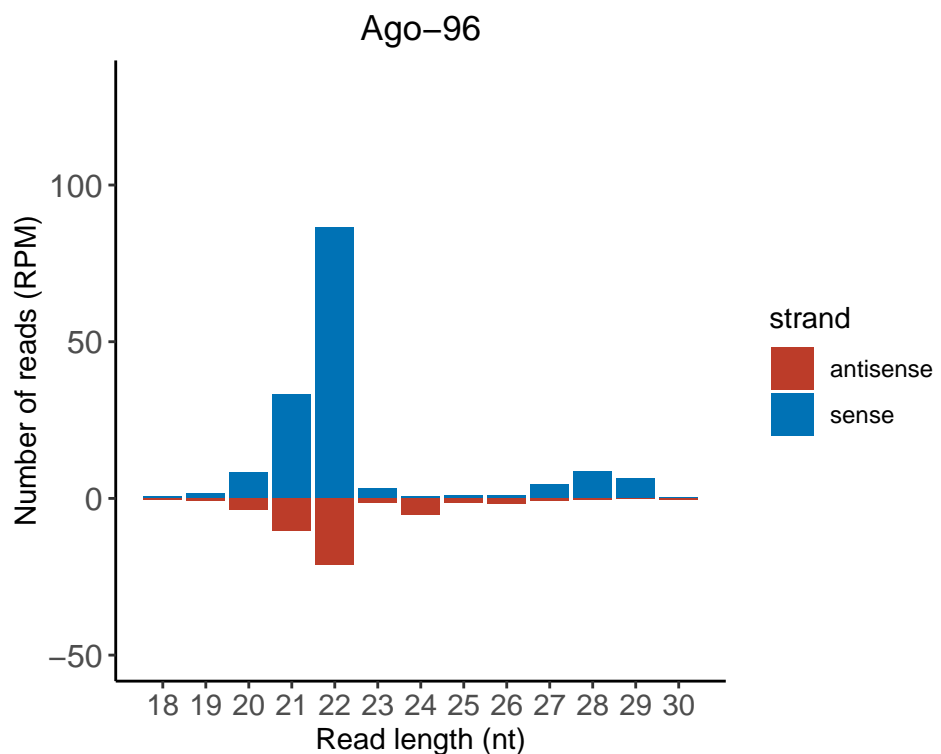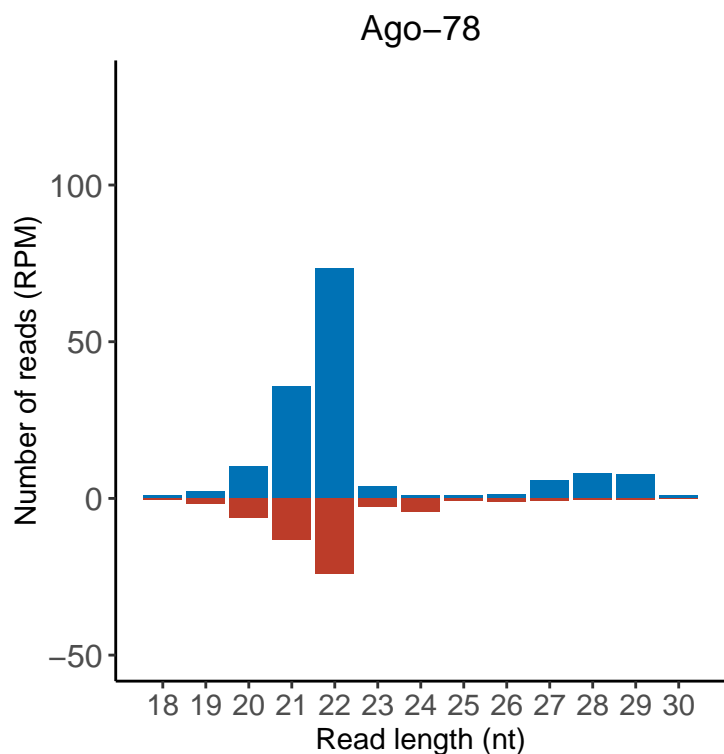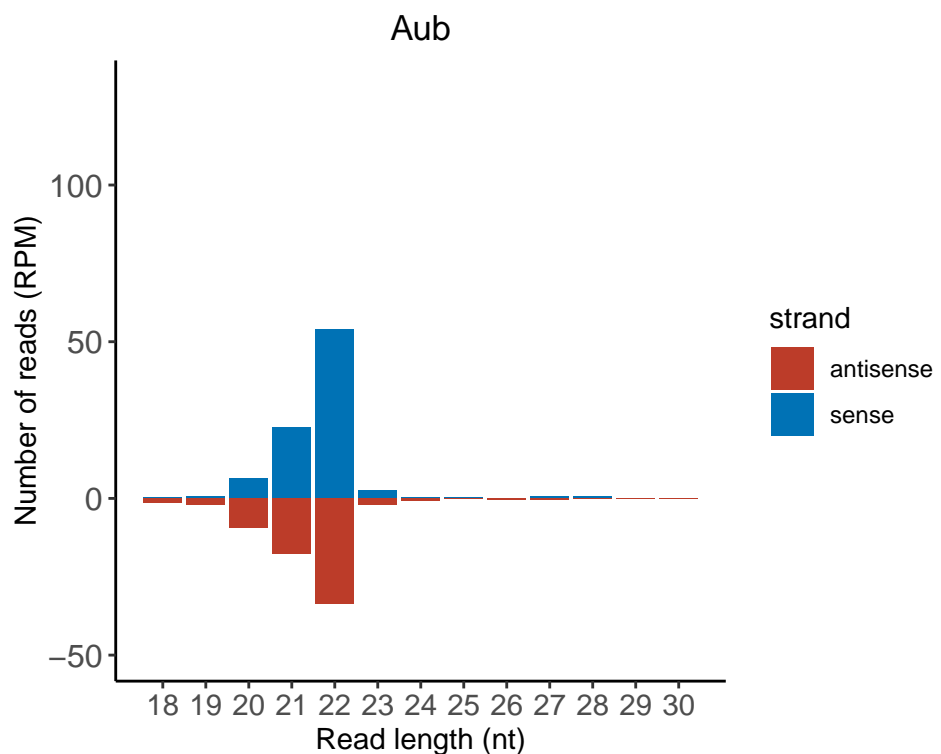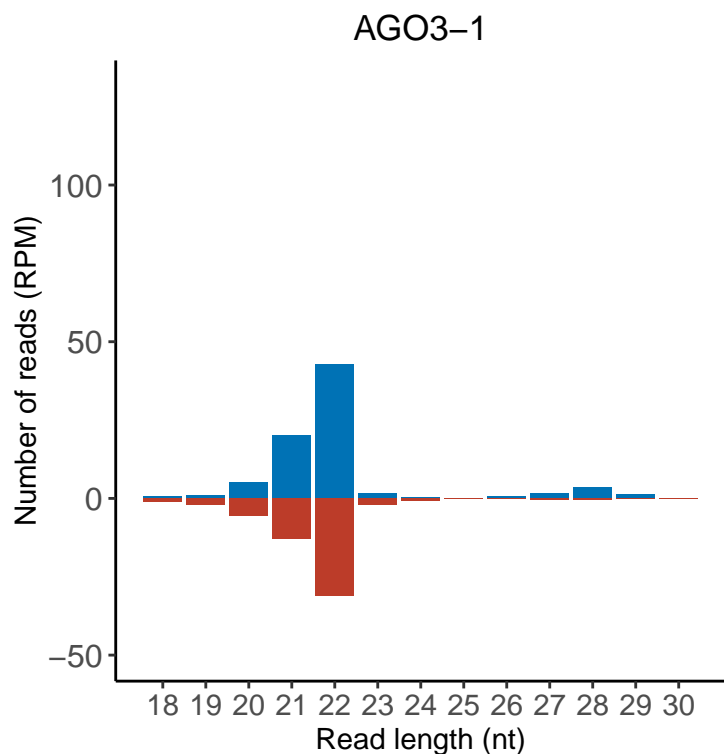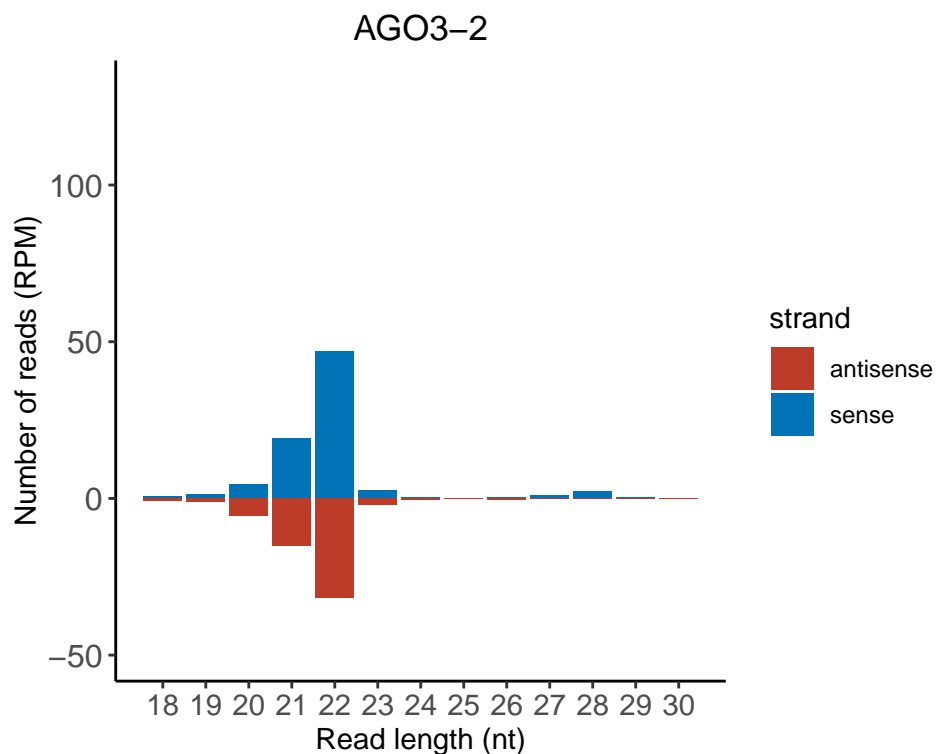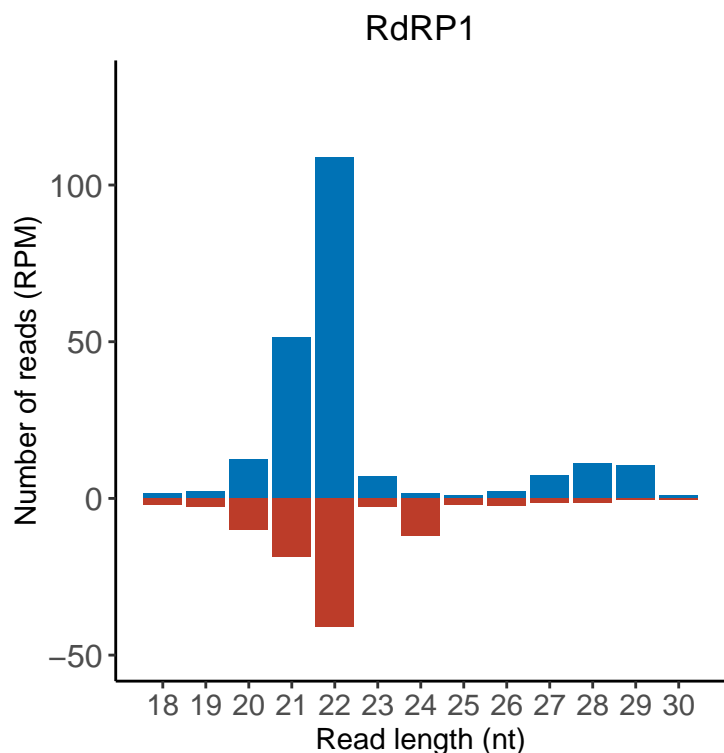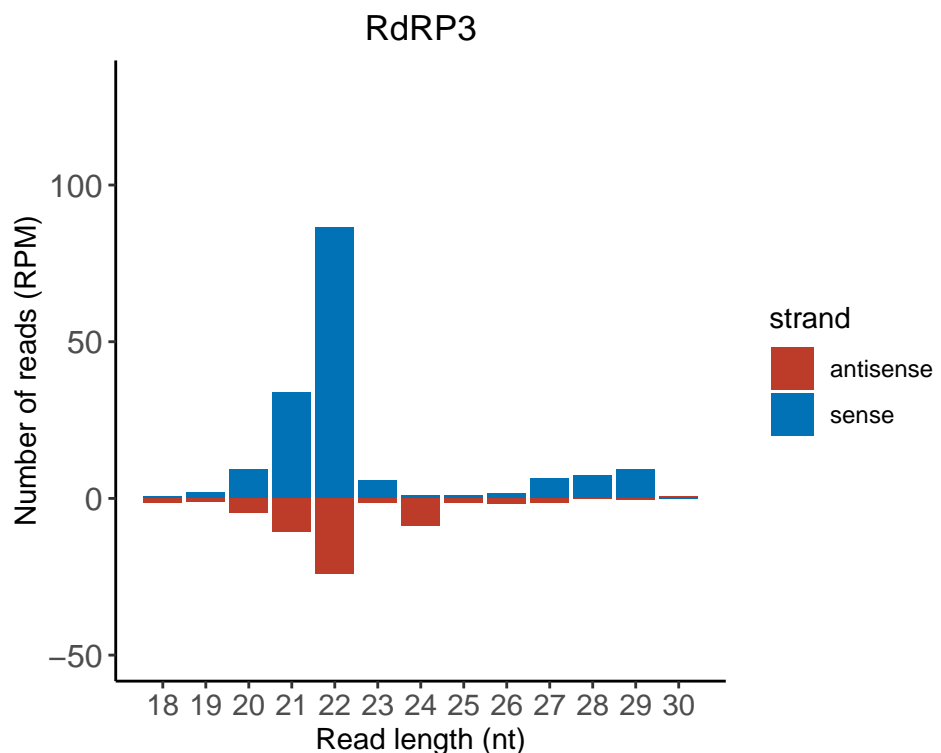

Supplement: S1 Data — On the Normalized and Relative levels pages, normalized read counts (RPM) and relative levels compared to the control (dsGFP) library were used. TEs with more than 50RPM and Coding Genes with more than 3.5RPM on average in KD libraries are included in this website. For viral sequences, reference sequences were generated by assembling sRNA sequences (See the Analysis of sRNAseq data section in Materials and methods.) and size distributions of sRNA reads mapped to each contig are shown. Reads were first grouped by their 5’ nucleotides, and reads of each length were counted. Full tables including TEs and Coding genes with fewer reads are in the “FullTable” folder. (ZIP) [file pone.0281195.s017.zip › SupplementaryData/Links/PDFs/Motif:rnd-1_family-955TE_lengthdistribution.pdf]

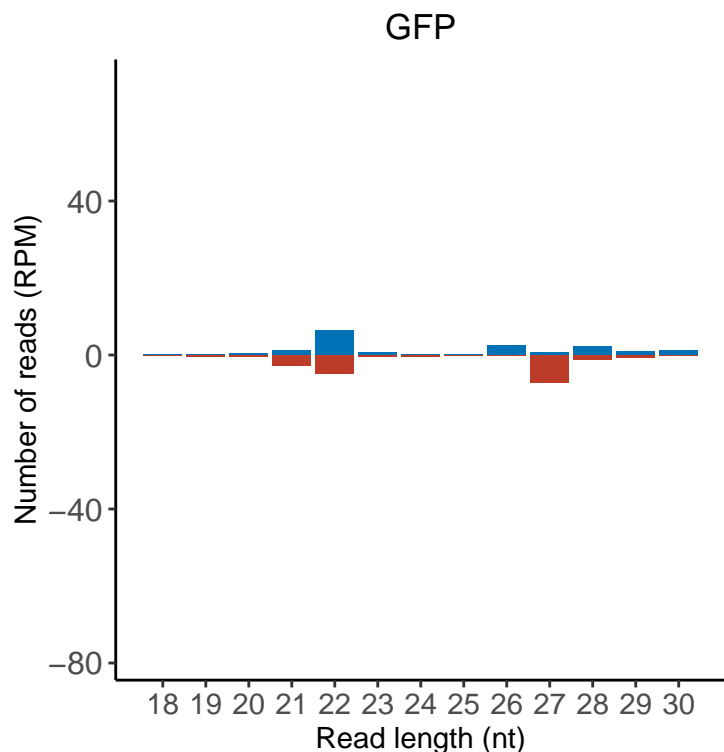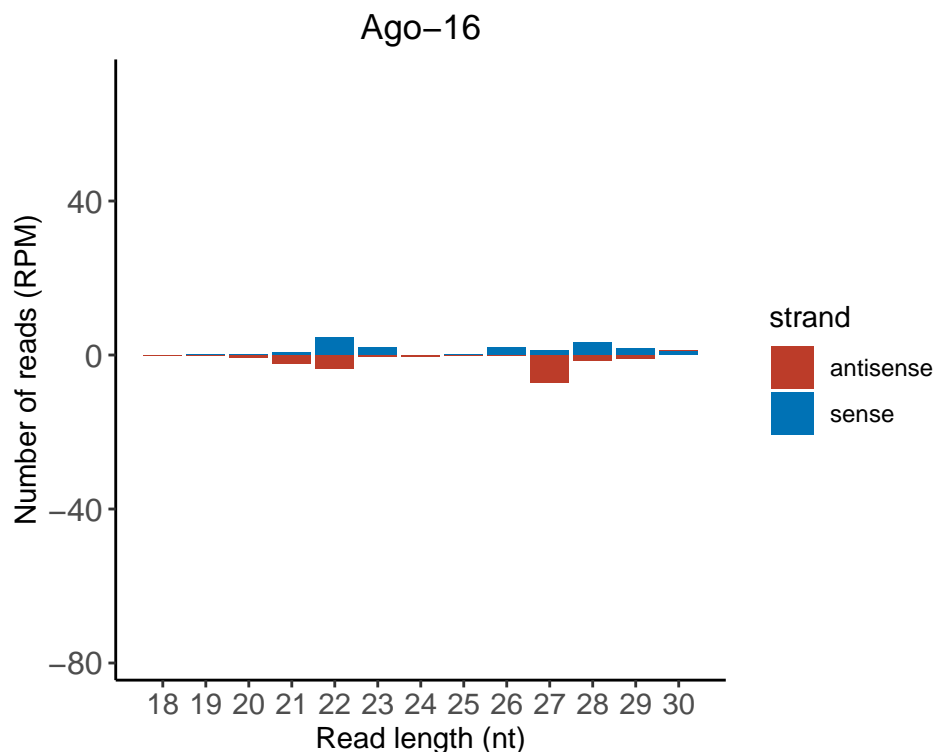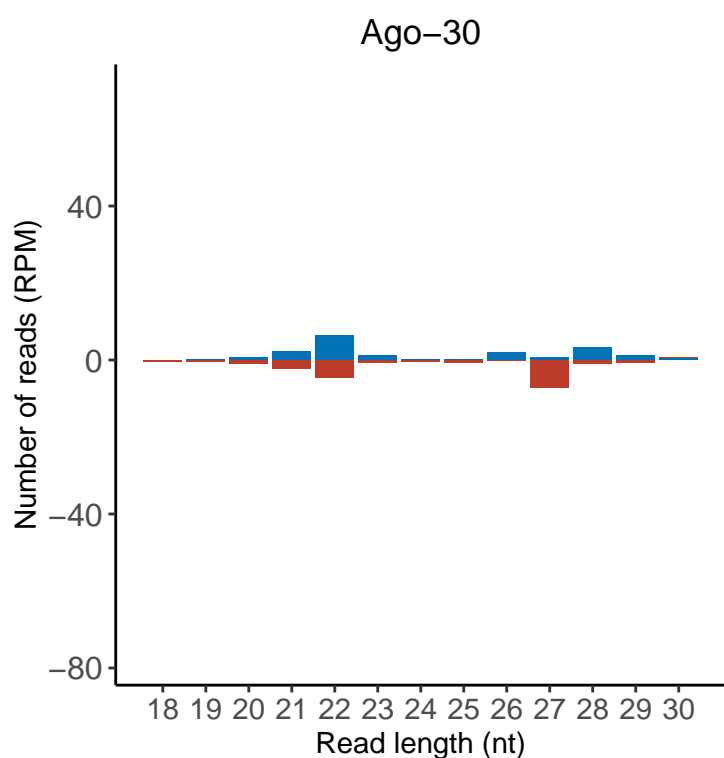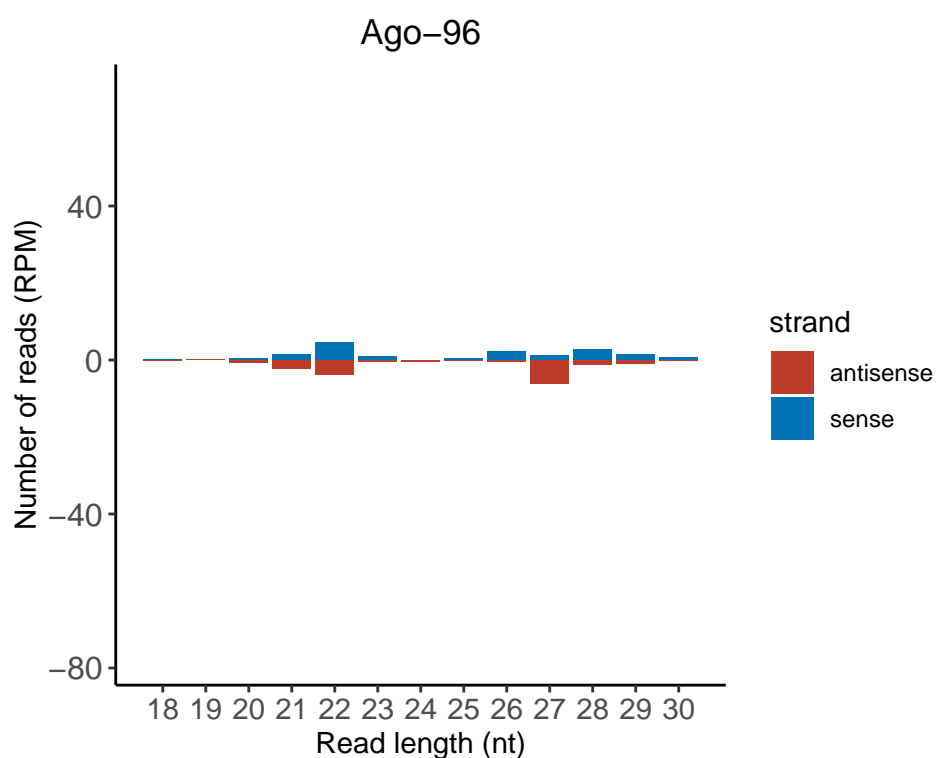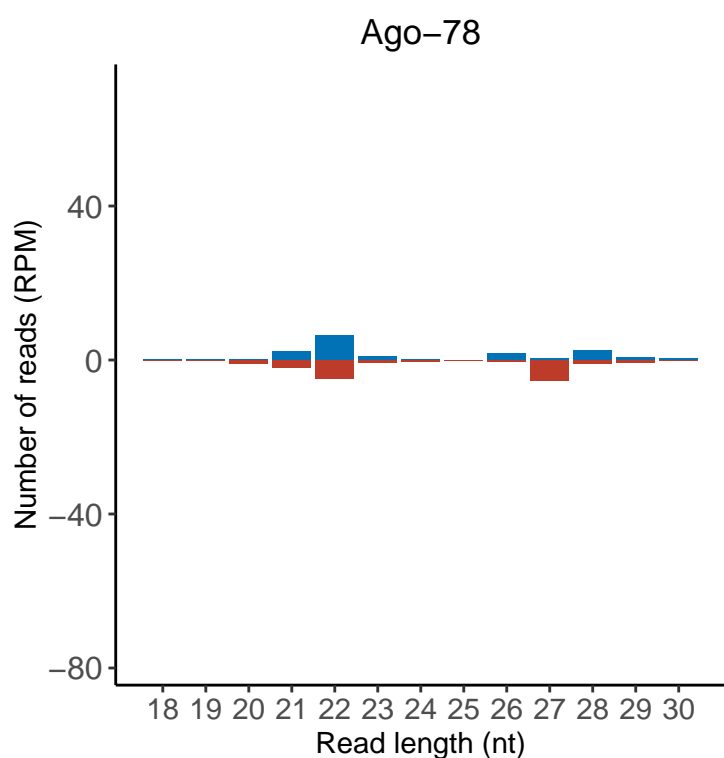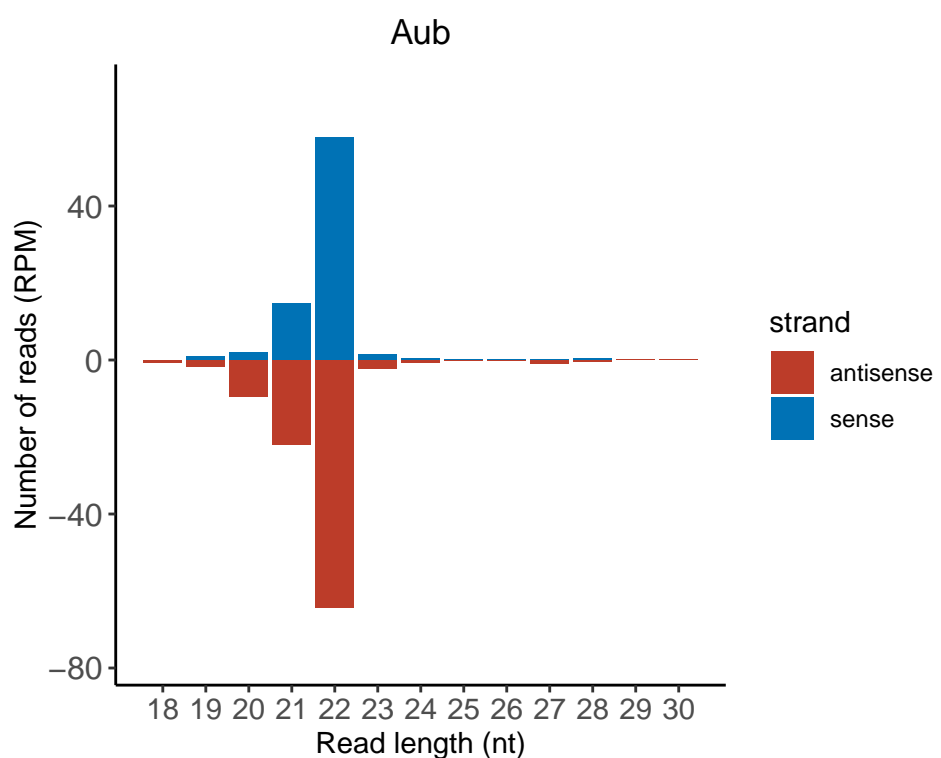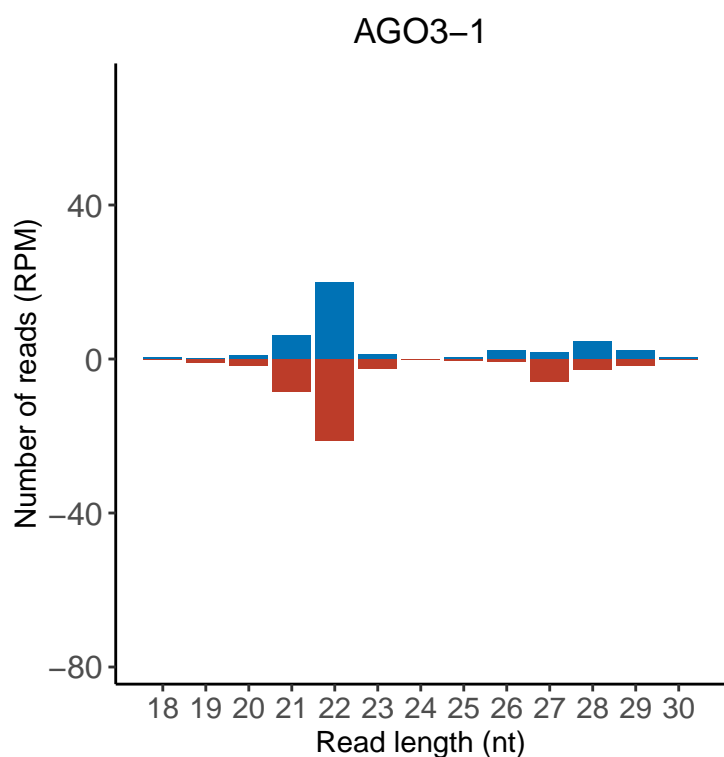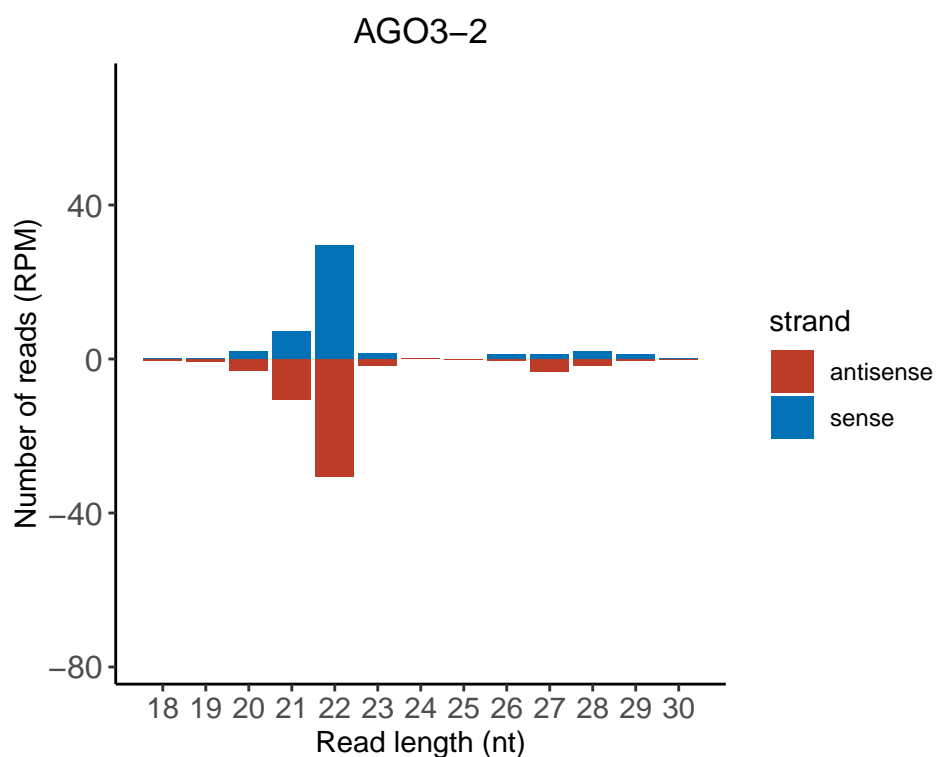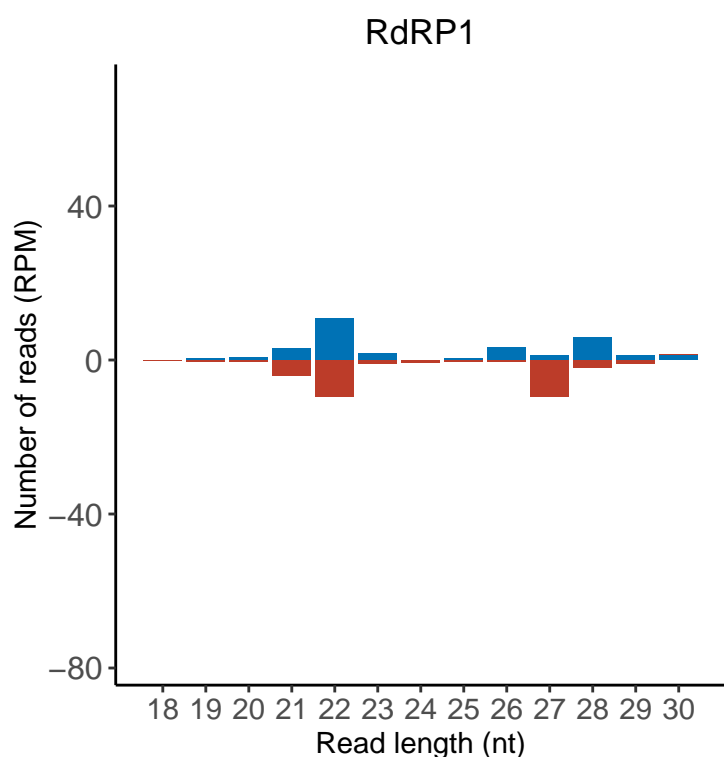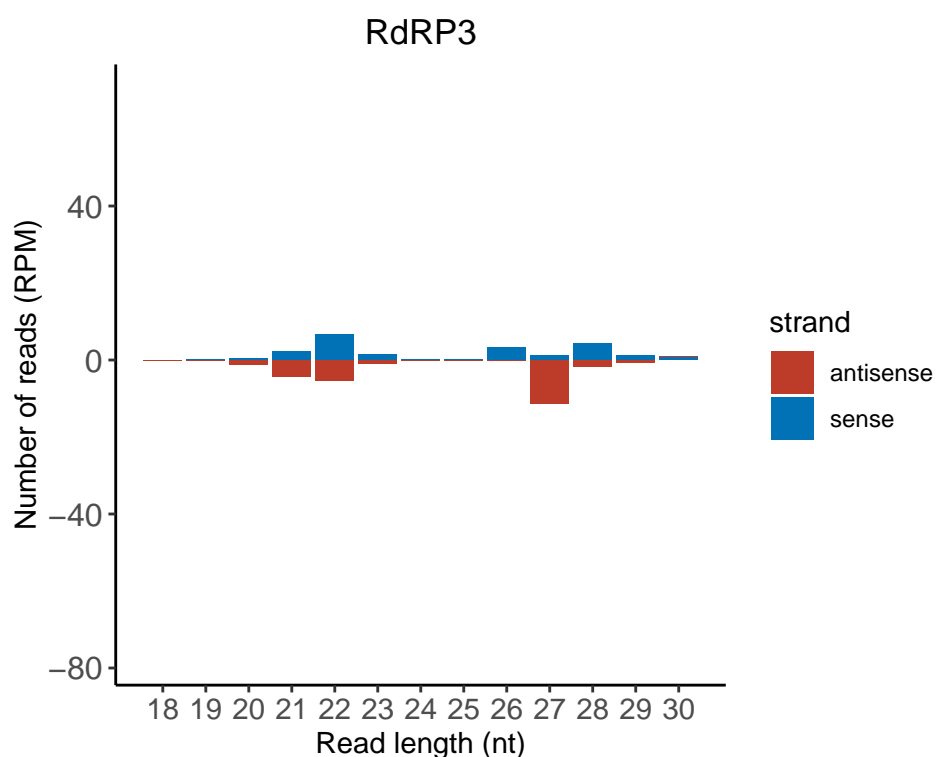

Supplement: S1 Data — On the Normalized and Relative levels pages, normalized read counts (RPM) and relative levels compared to the control (dsGFP) library were used. TEs with more than 50RPM and Coding Genes with more than 3.5RPM on average in KD libraries are included in this website. For viral sequences, reference sequences were generated by assembling sRNA sequences (See the Analysis of sRNAseq data section in Materials and methods.) and size distributions of sRNA reads mapped to each contig are shown. Reads were first grouped by their 5’ nucleotides, and reads of each length were counted. Full tables including TEs and Coding genes with fewer reads are in the “FullTable” folder. (ZIP) [file pone.0281195.s017.zip › SupplementaryData/Links/PDFs/Motif:rnd-6_family-7183TE_lengthdistribution.pdf]

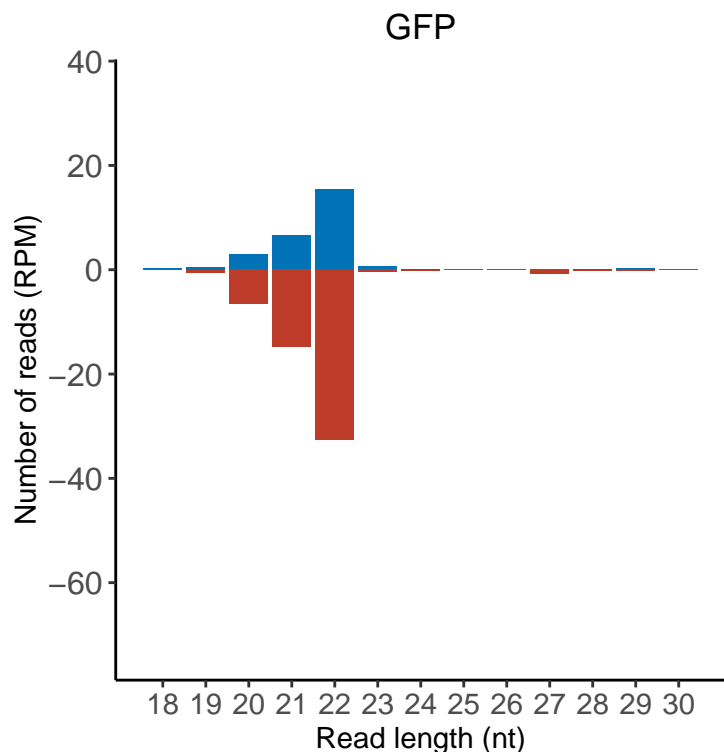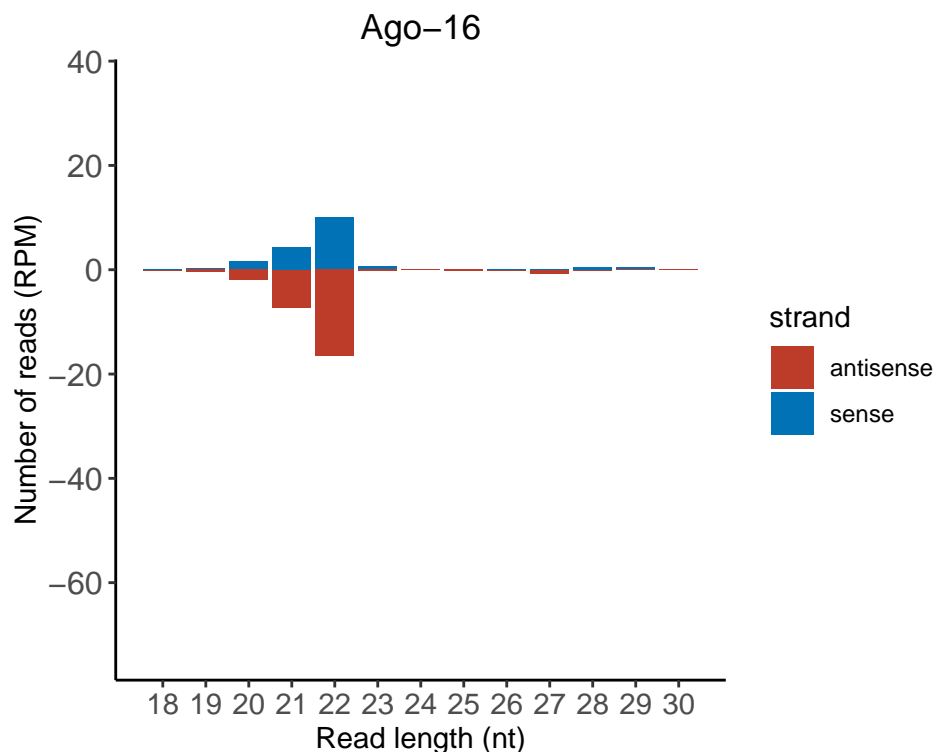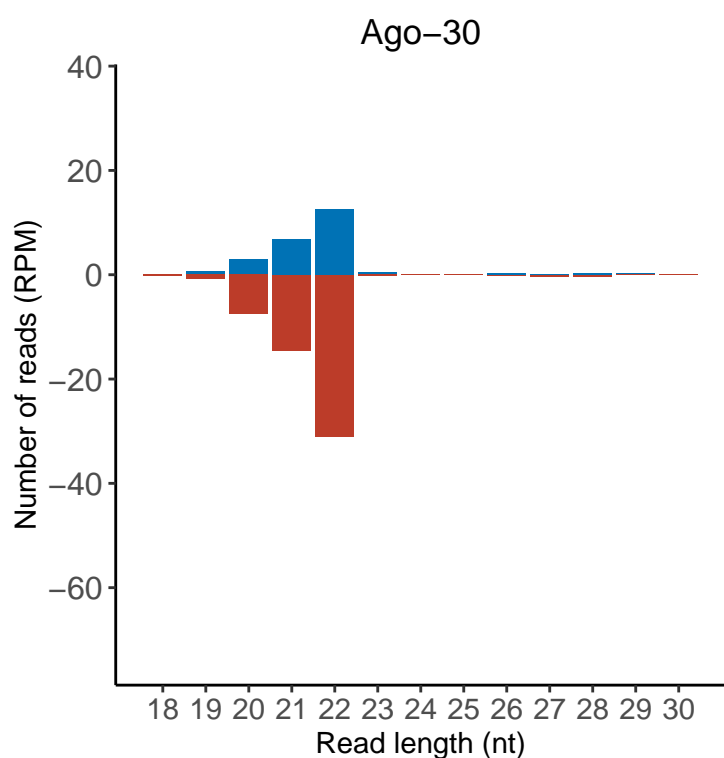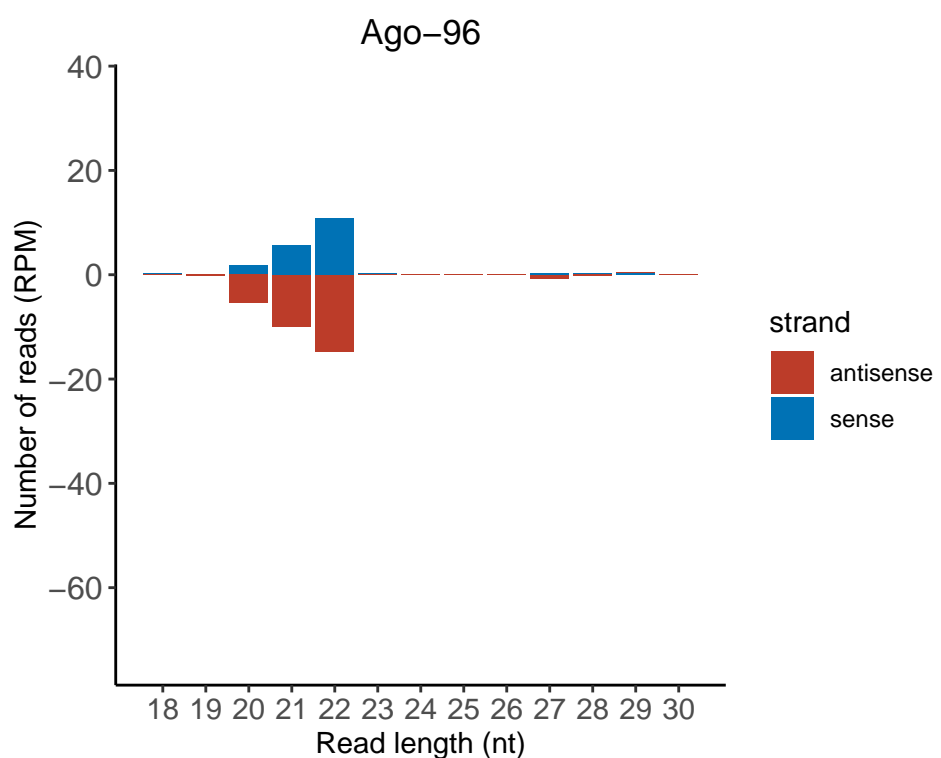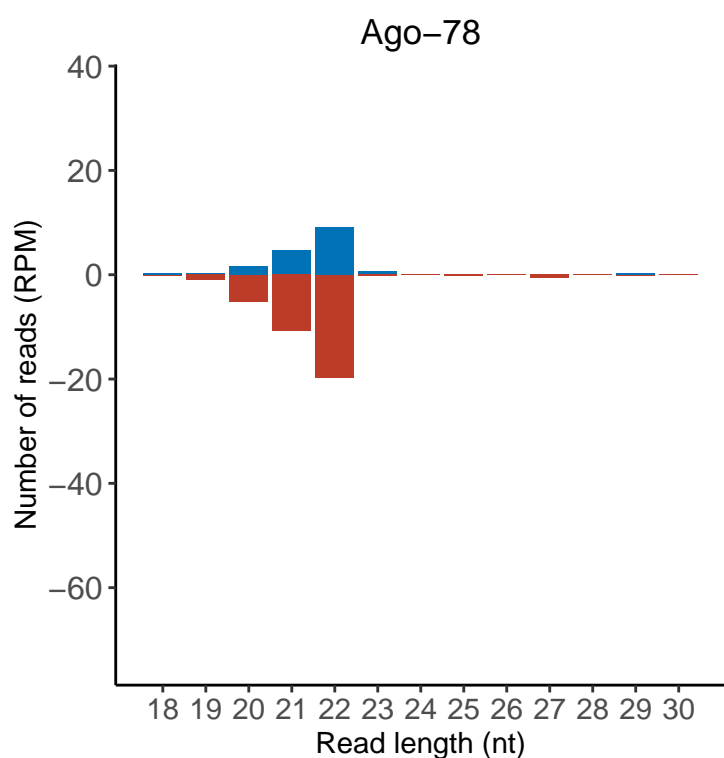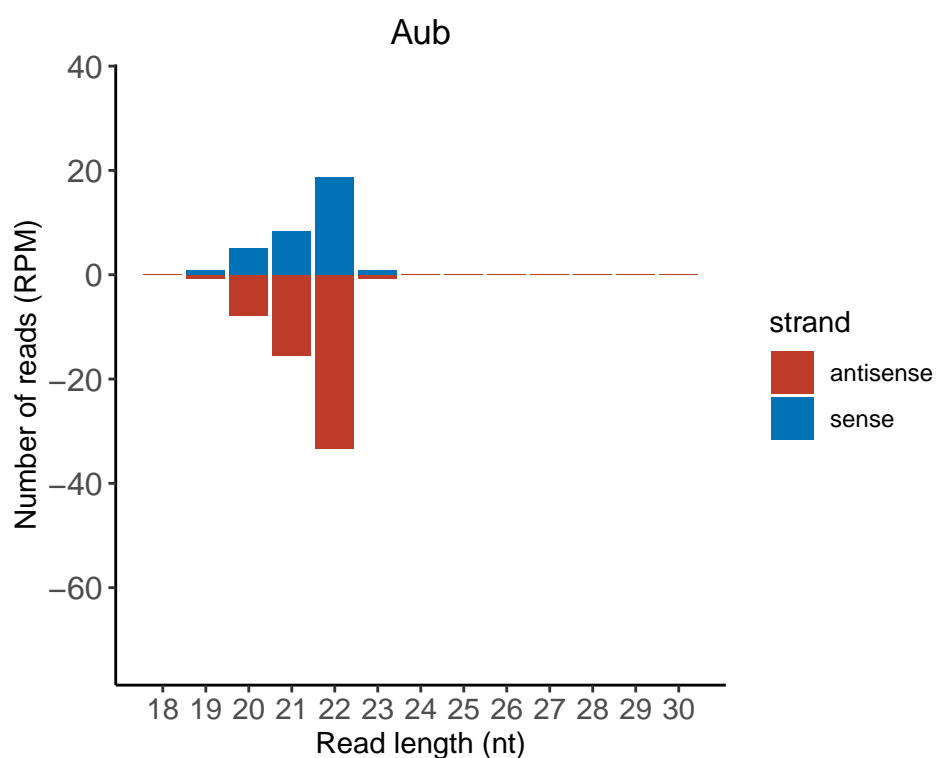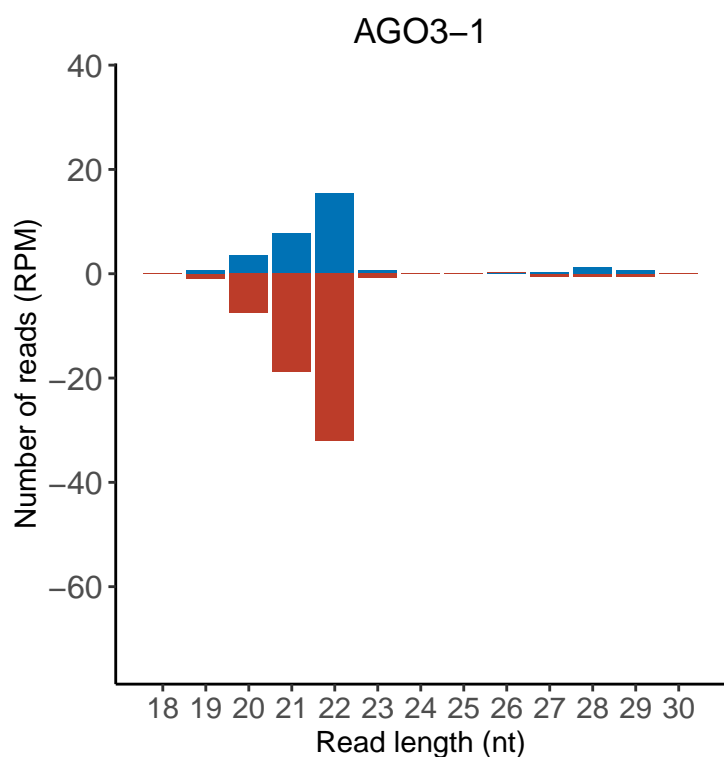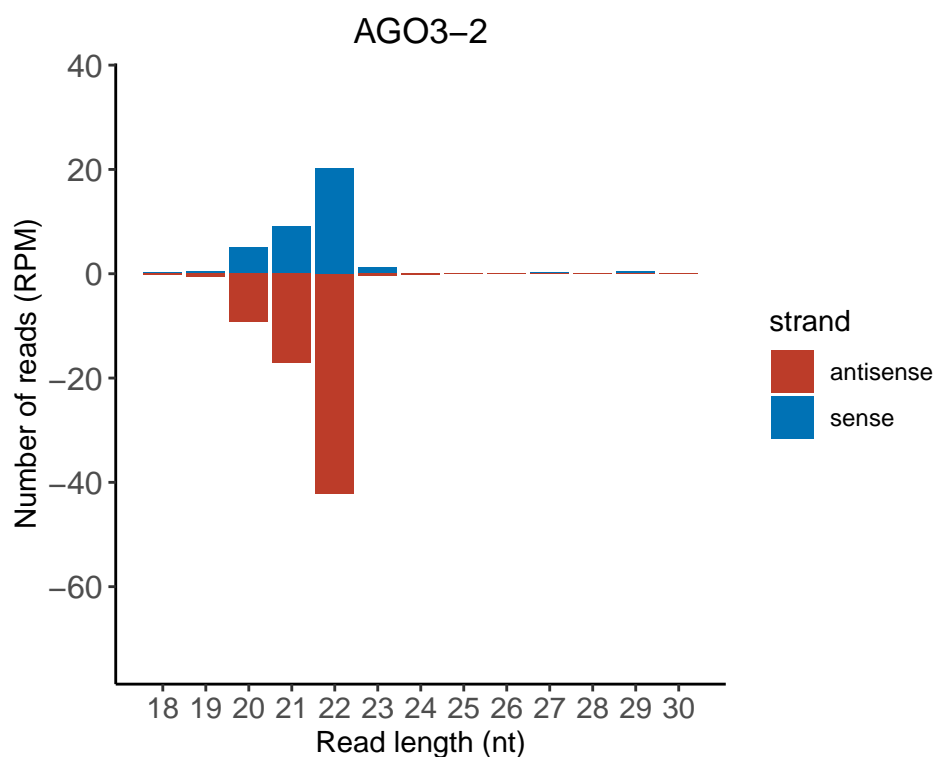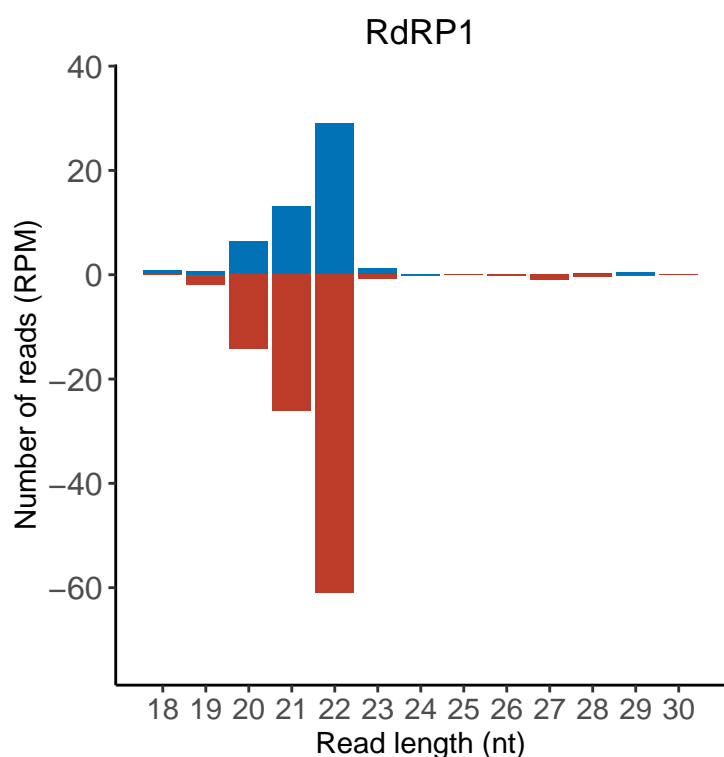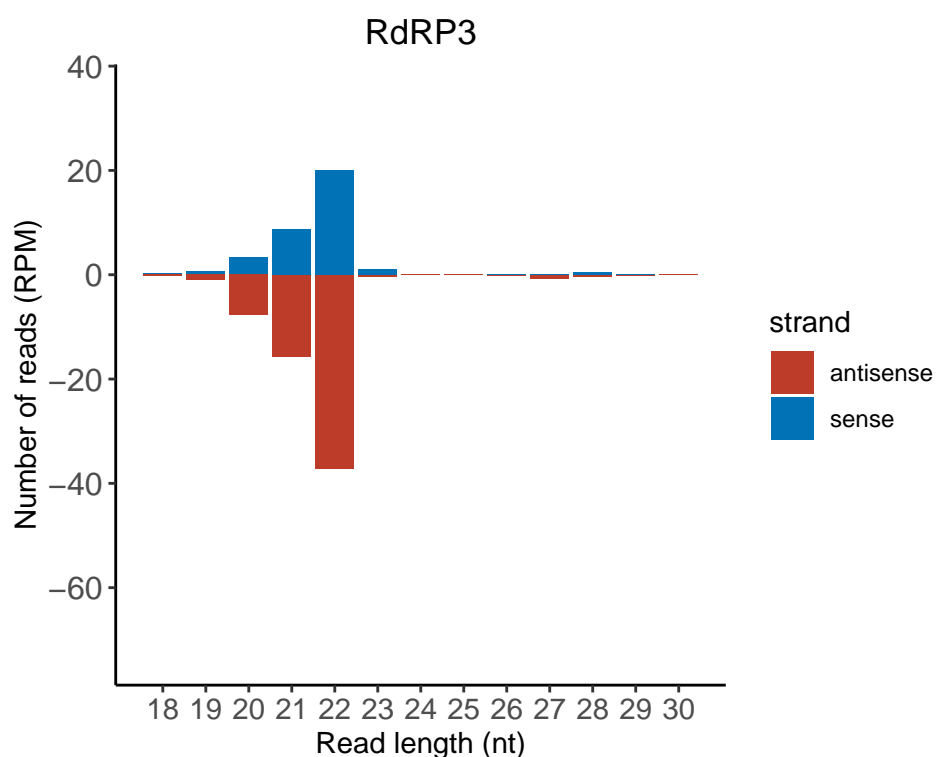

Supplement: S1 Data — On the Normalized and Relative levels pages, normalized read counts (RPM) and relative levels compared to the control (dsGFP) library were used. TEs with more than 50RPM and Coding Genes with more than 3.5RPM on average in KD libraries are included in this website. For viral sequences, reference sequences were generated by assembling sRNA sequences (See the Analysis of sRNAseq data section in Materials and methods.) and size distributions of sRNA reads mapped to each contig are shown. Reads were first grouped by their 5’ nucleotides, and reads of each length were counted. Full tables including TEs and Coding genes with fewer reads are in the “FullTable” folder. (ZIP) [file pone.0281195.s017.zip › SupplementaryData/Links/PDFs/Motif:rnd-1_family-393TE_lengthdistribution.pdf]

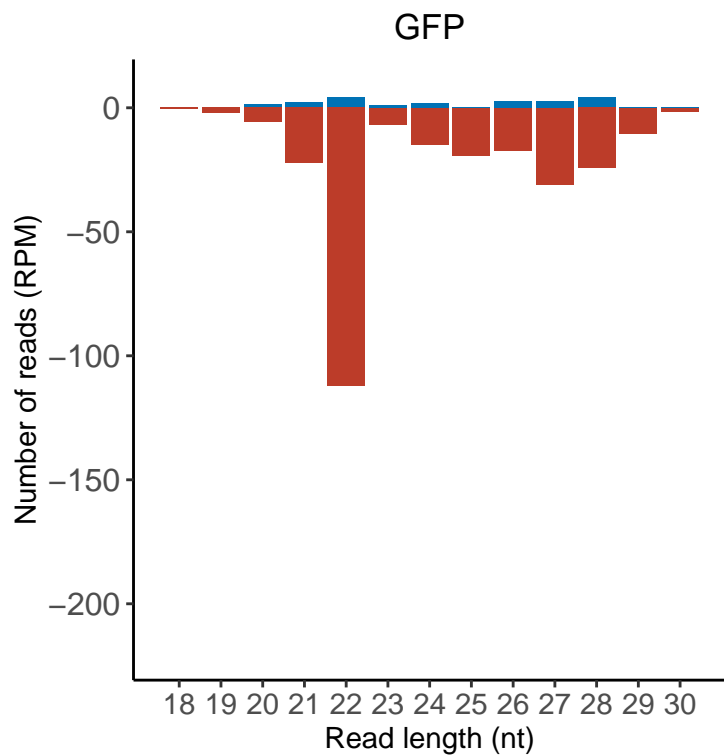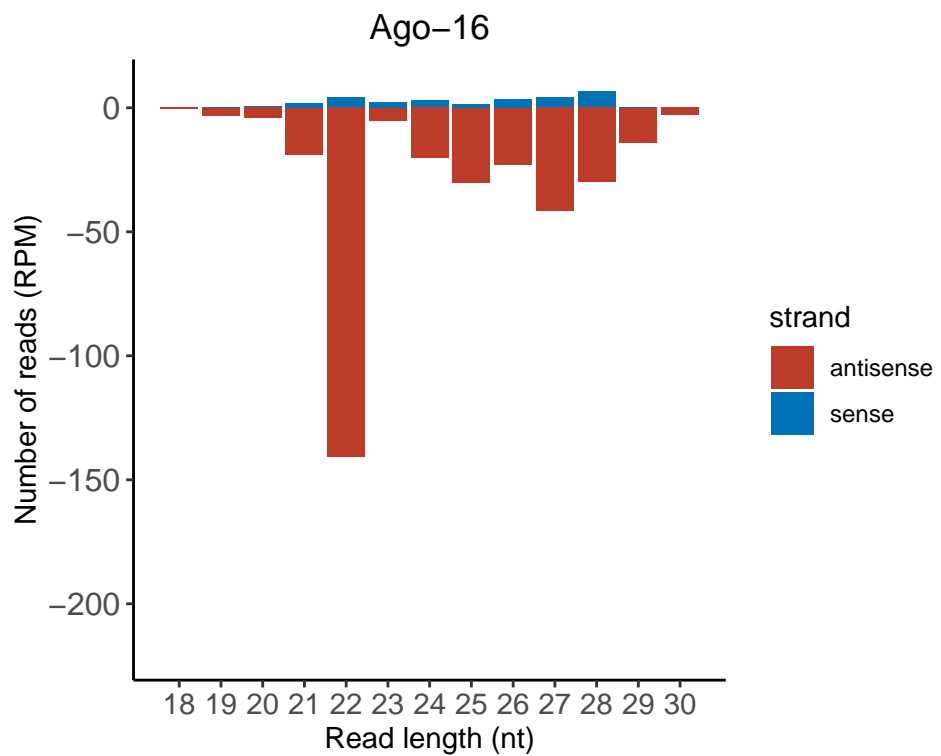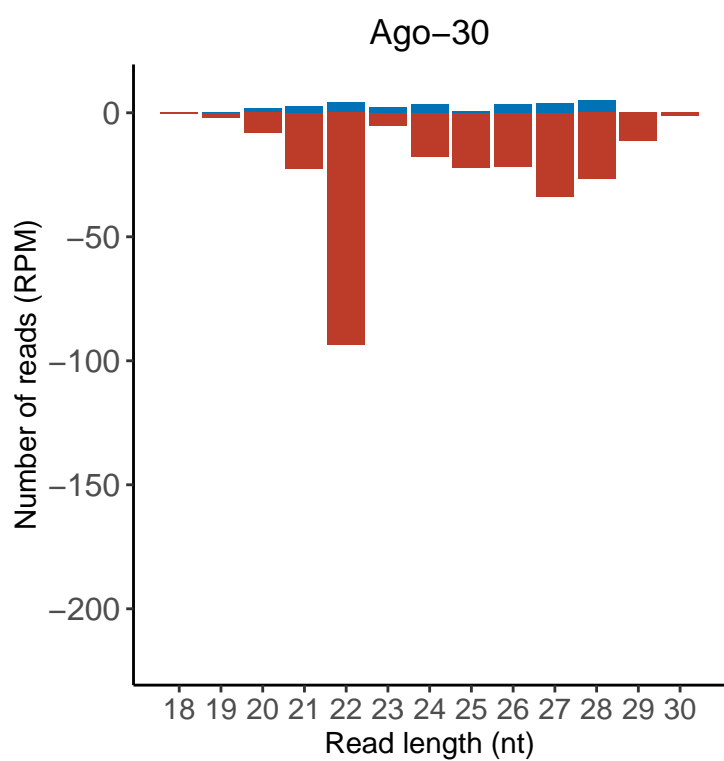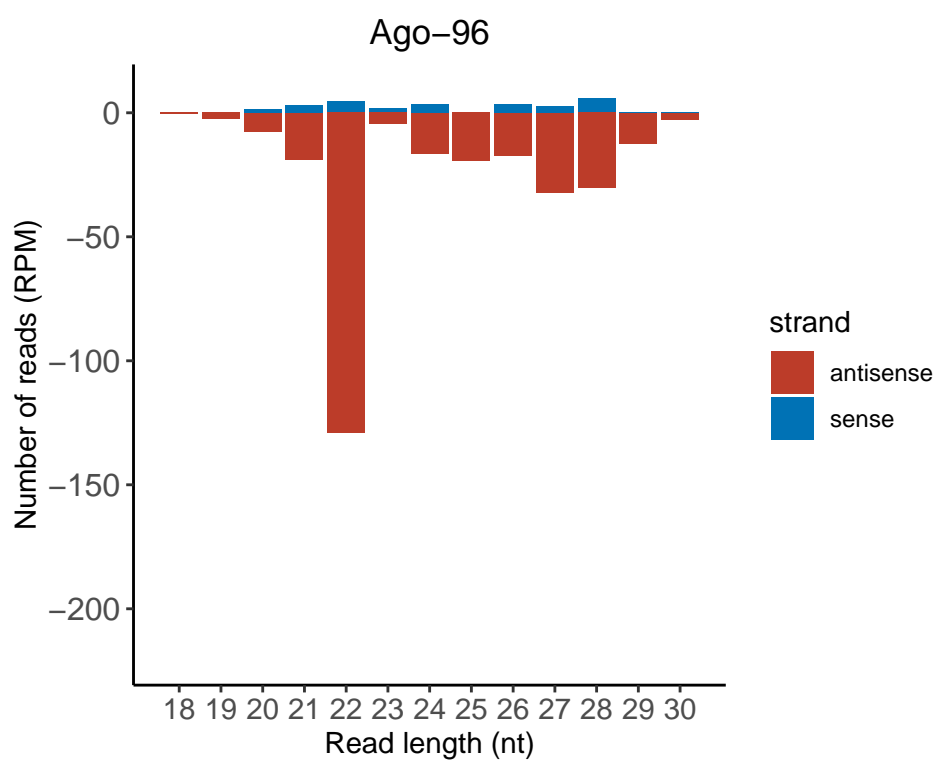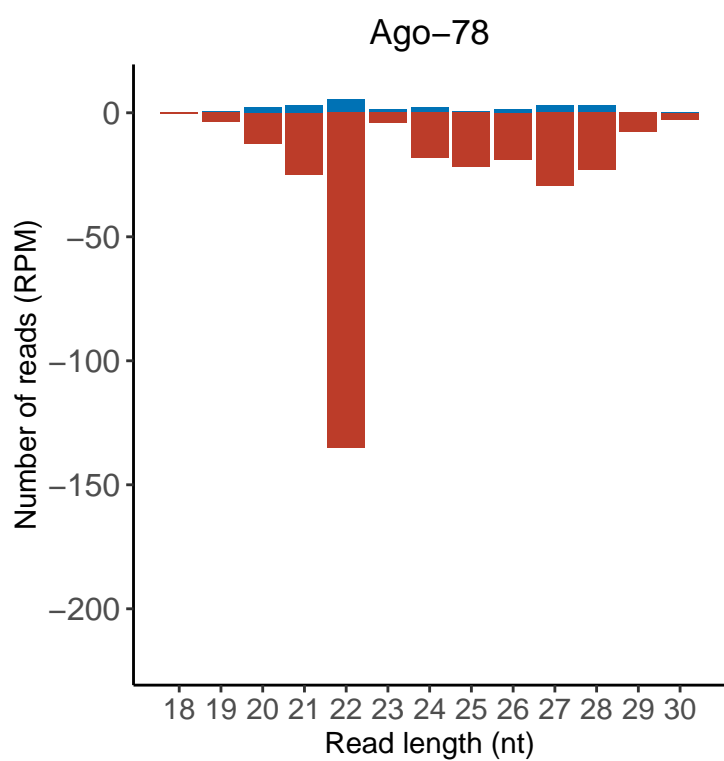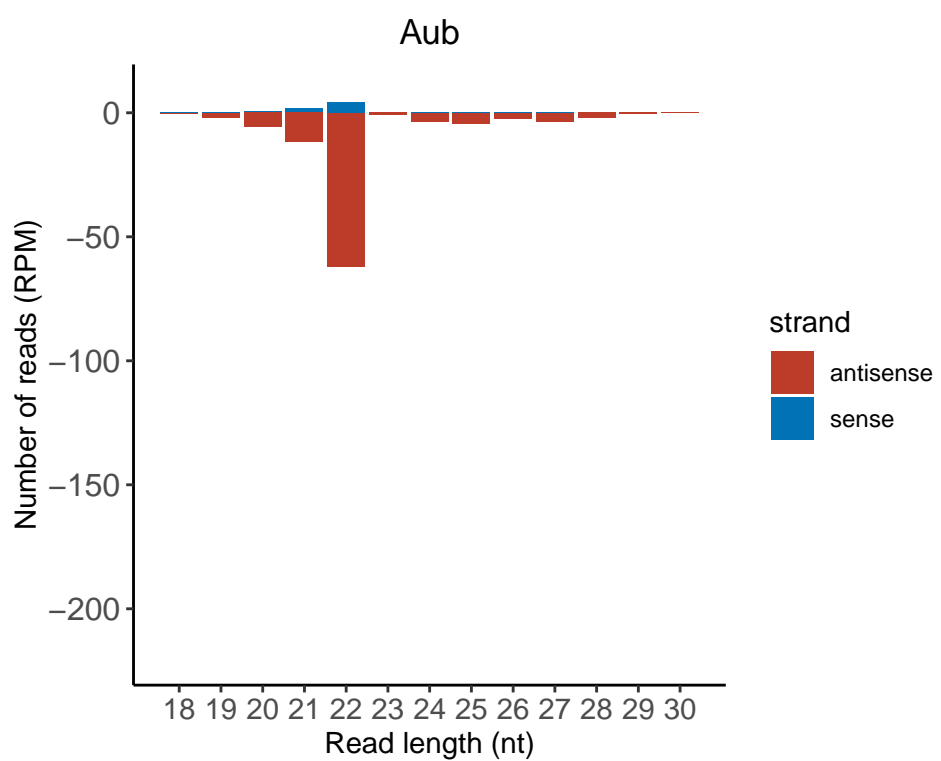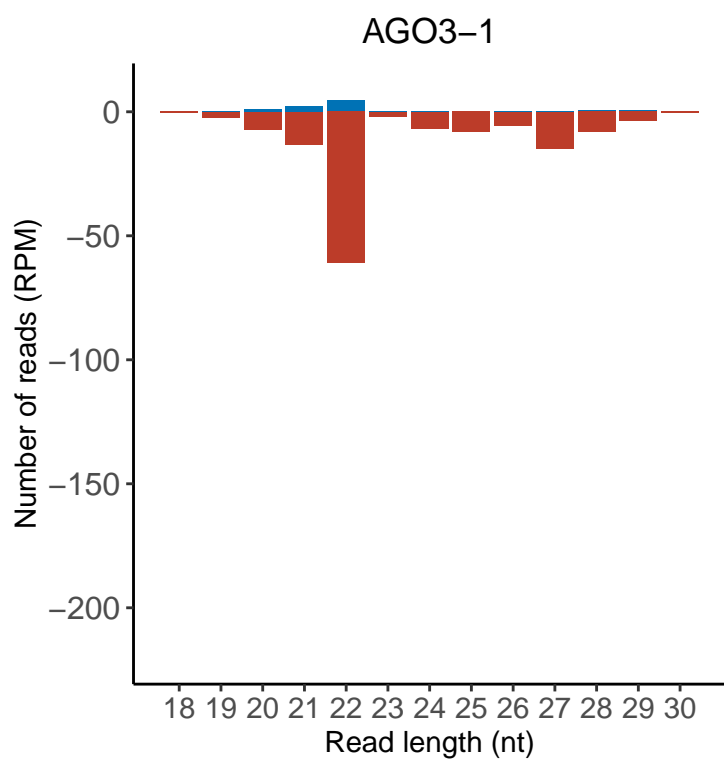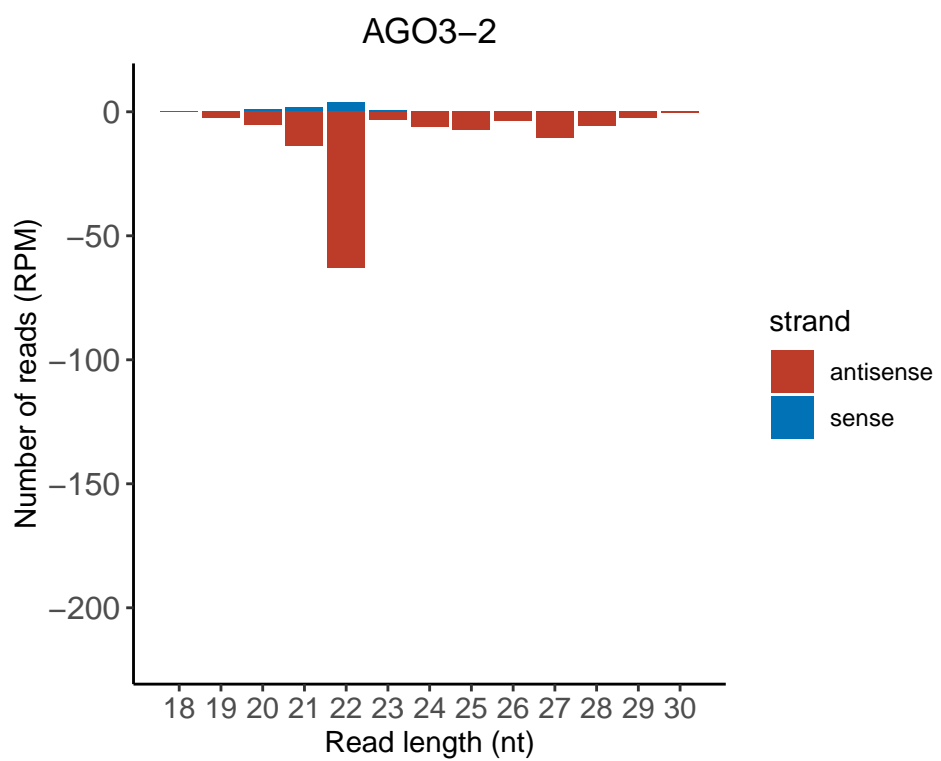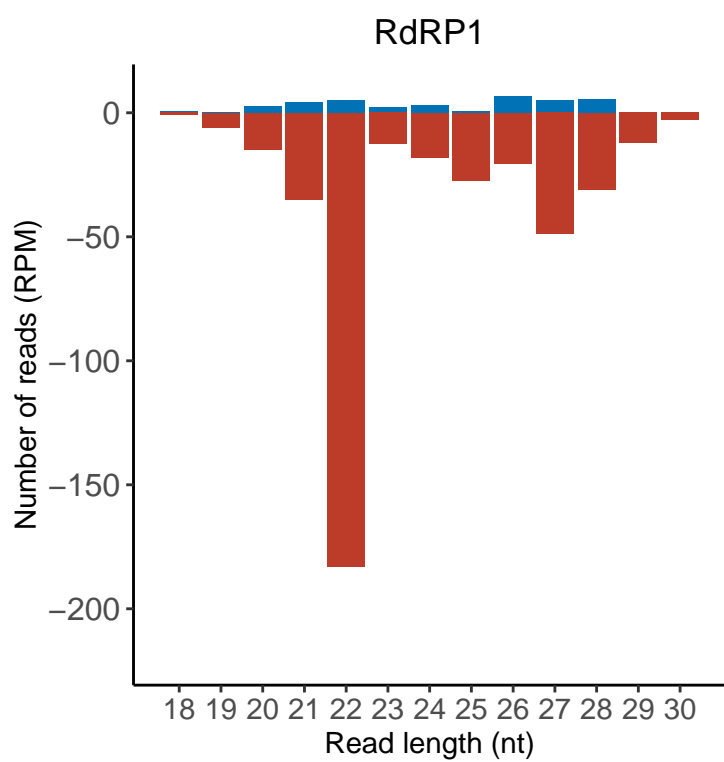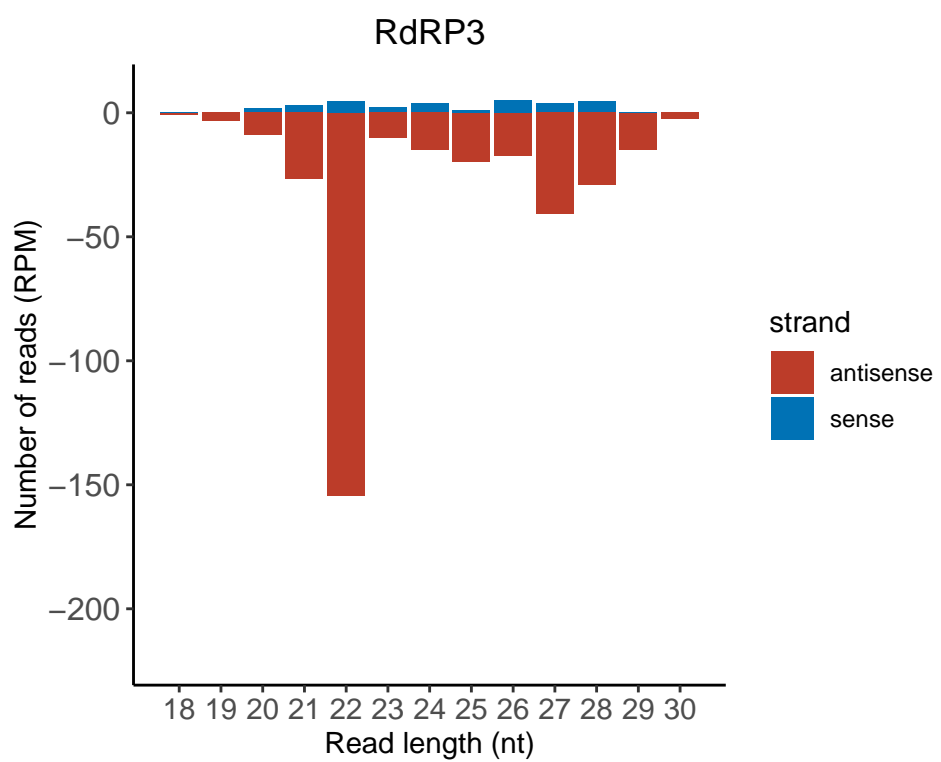

Supplement: S1 Data — On the Normalized and Relative levels pages, normalized read counts (RPM) and relative levels compared to the control (dsGFP) library were used. TEs with more than 50RPM and Coding Genes with more than 3.5RPM on average in KD libraries are included in this website. For viral sequences, reference sequences were generated by assembling sRNA sequences (See the Analysis of sRNAseq data section in Materials and methods.) and size distributions of sRNA reads mapped to each contig are shown. Reads were first grouped by their 5’ nucleotides, and reads of each length were counted. Full tables including TEs and Coding genes with fewer reads are in the “FullTable” folder. (ZIP) [file pone.0281195.s017.zip › SupplementaryData/Links/PDFs/Motif:rnd-5_family-990TE_lengthdistribution.pdf]

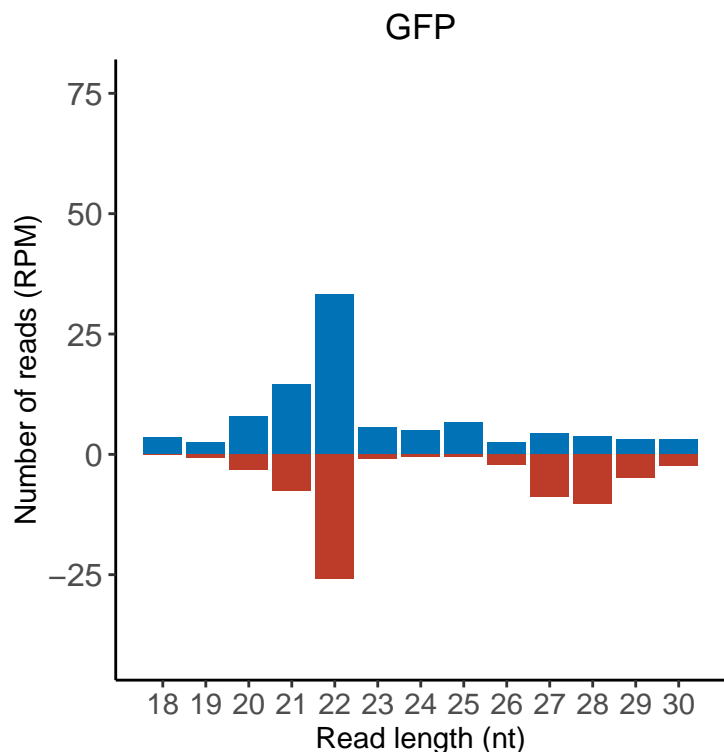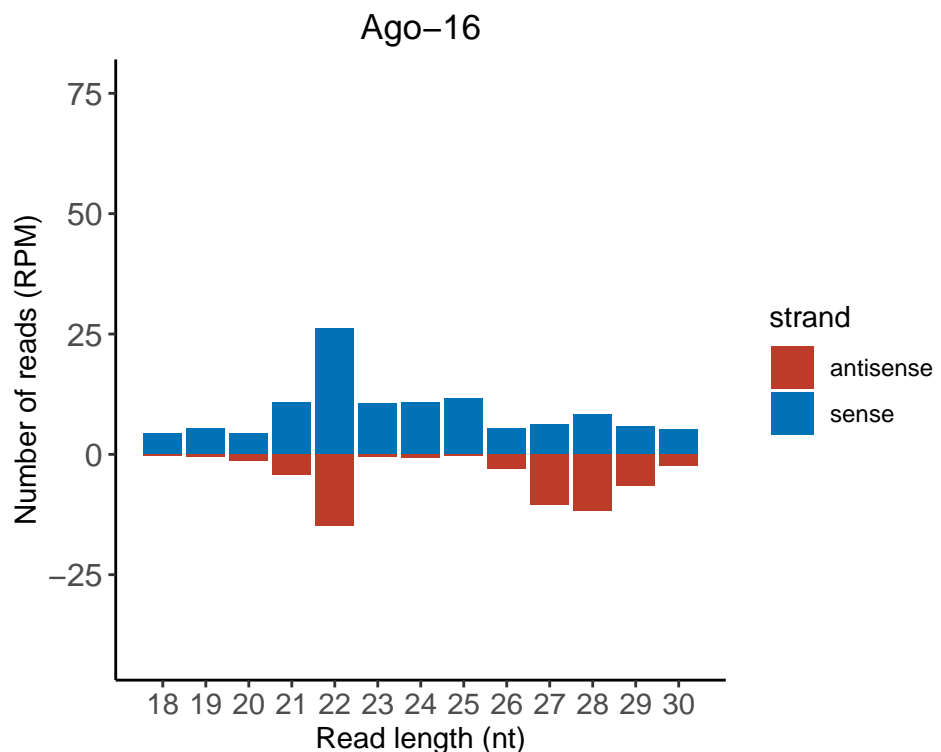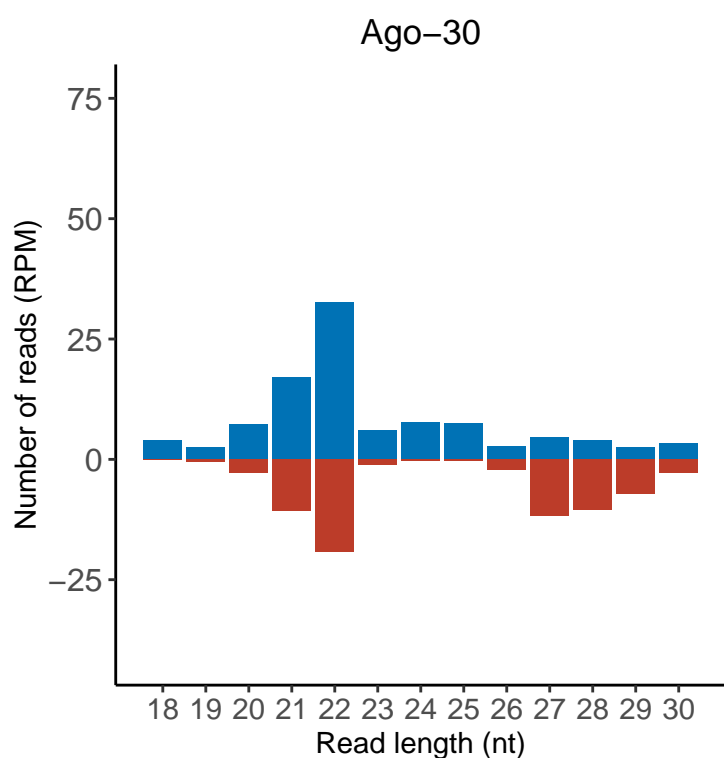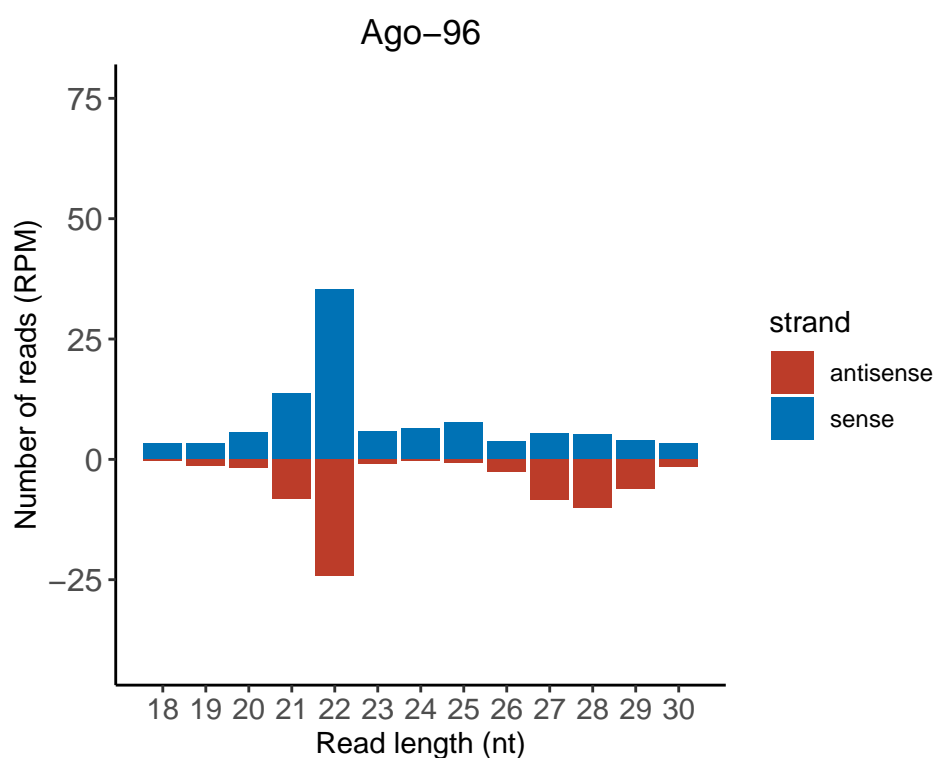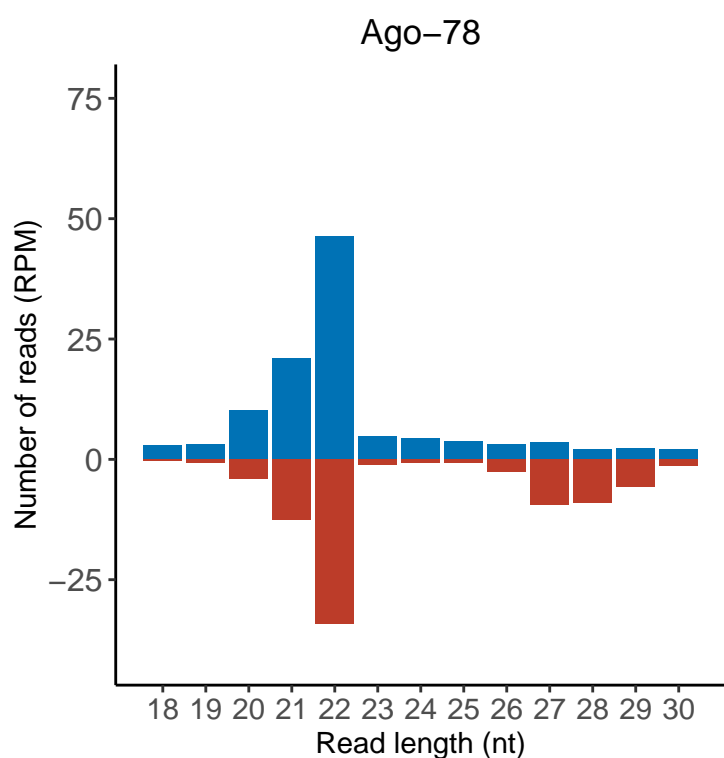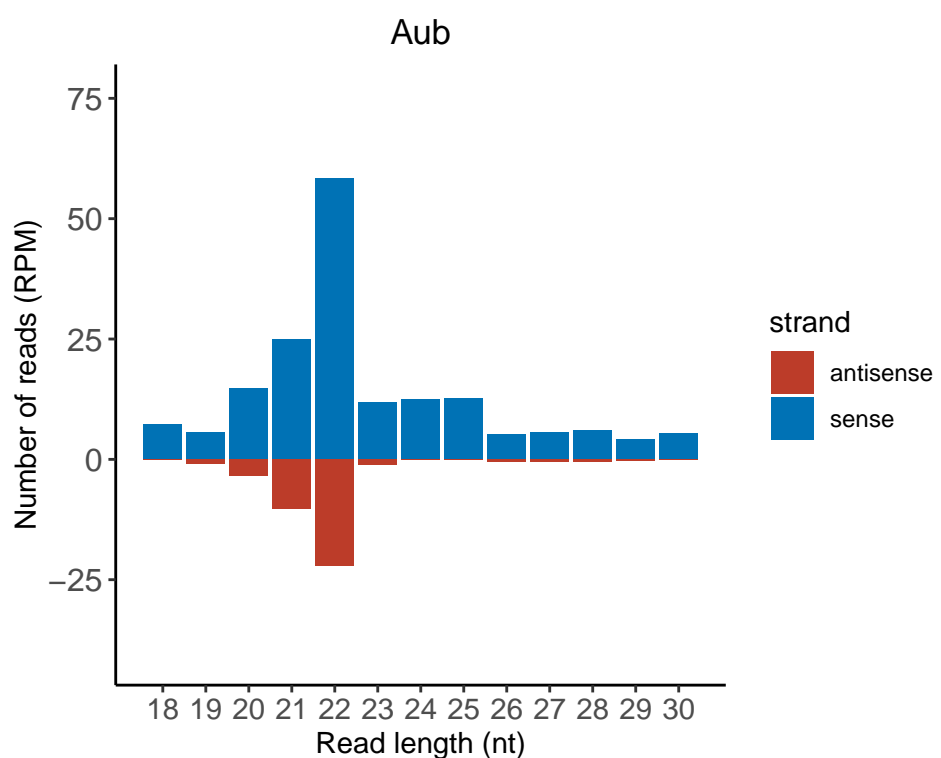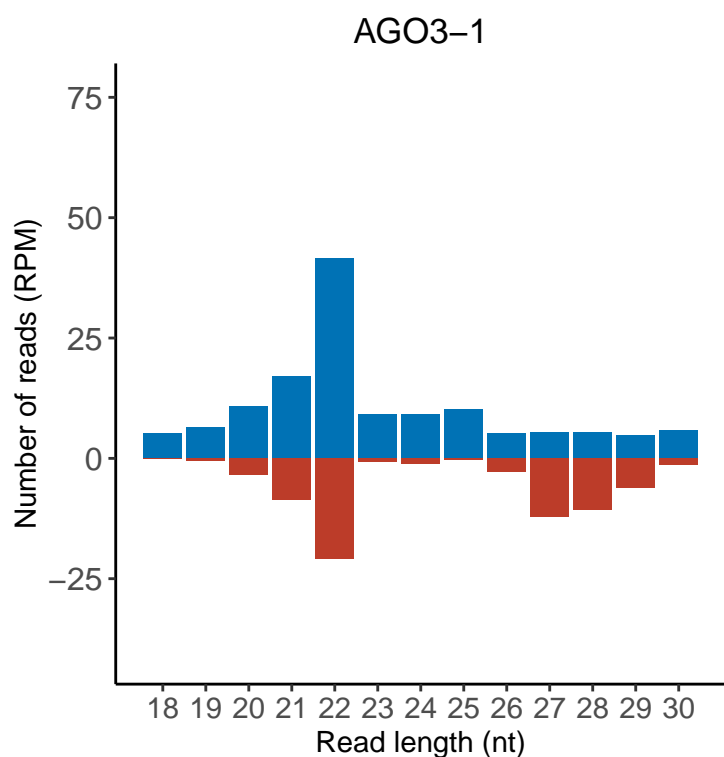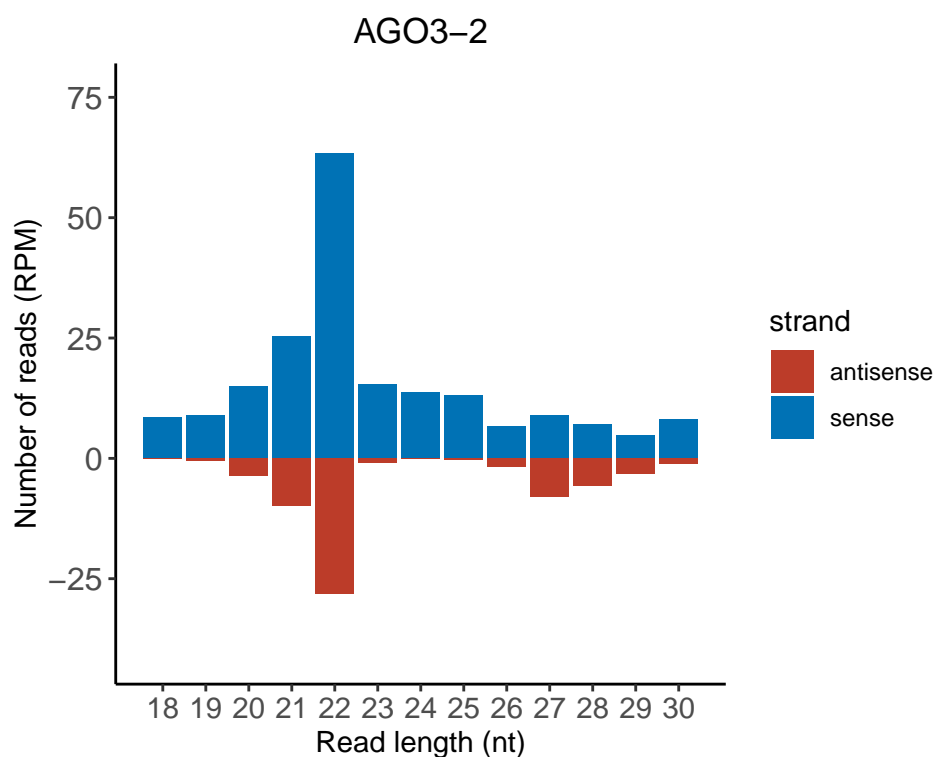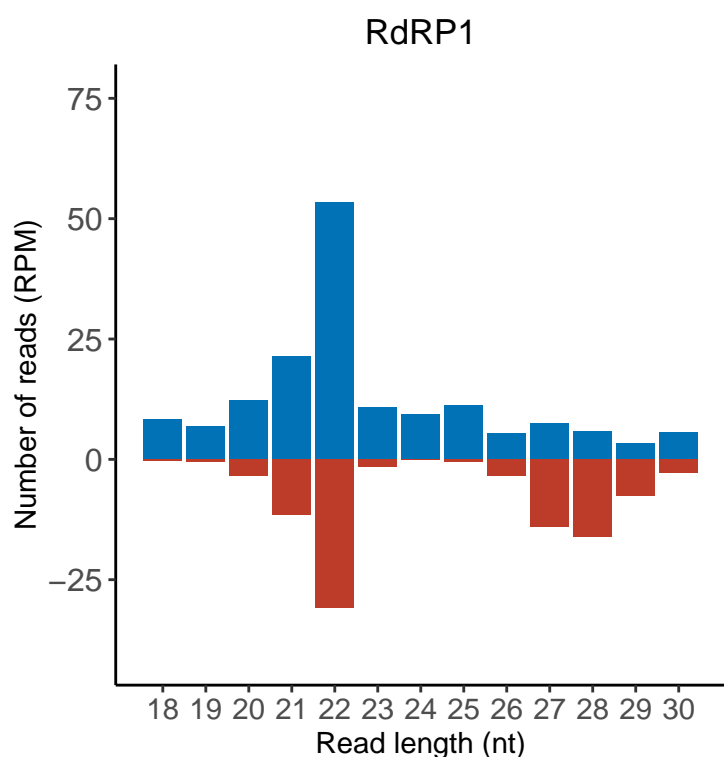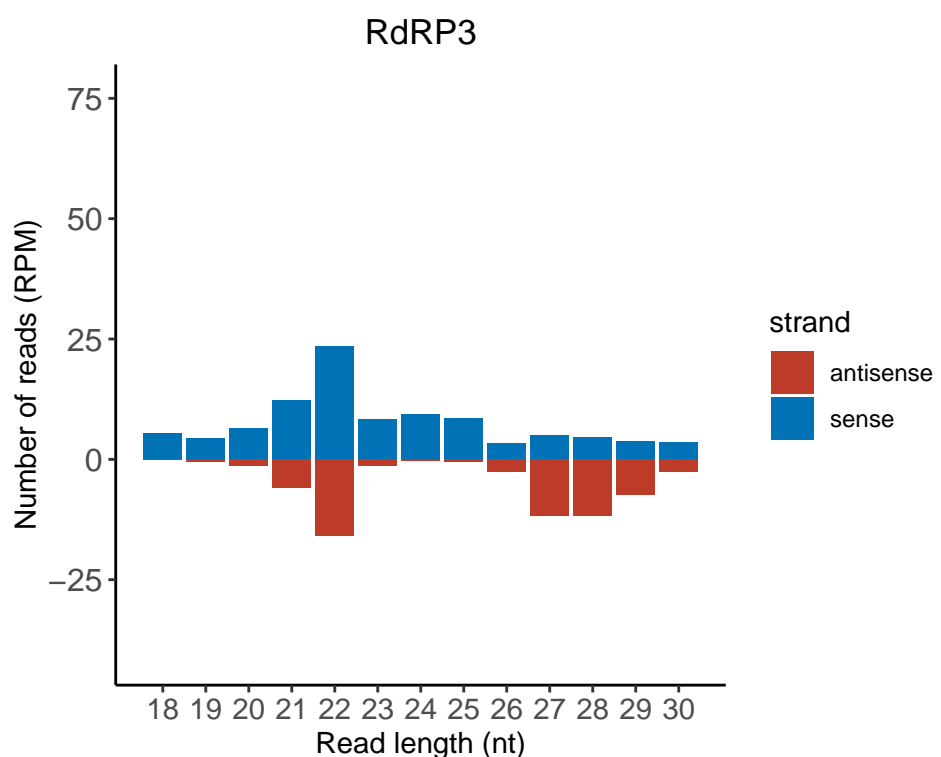

Supplement: S1 Data — On the Normalized and Relative levels pages, normalized read counts (RPM) and relative levels compared to the control (dsGFP) library were used. TEs with more than 50RPM and Coding Genes with more than 3.5RPM on average in KD libraries are included in this website. For viral sequences, reference sequences were generated by assembling sRNA sequences (See the Analysis of sRNAseq data section in Materials and methods.) and size distributions of sRNA reads mapped to each contig are shown. Reads were first grouped by their 5’ nucleotides, and reads of each length were counted. Full tables including TEs and Coding genes with fewer reads are in the “FullTable” folder. (ZIP) [file pone.0281195.s017.zip › SupplementaryData/Links/PDFs/Motif:rnd-1_family-1557TE_lengthdistribution.pdf]

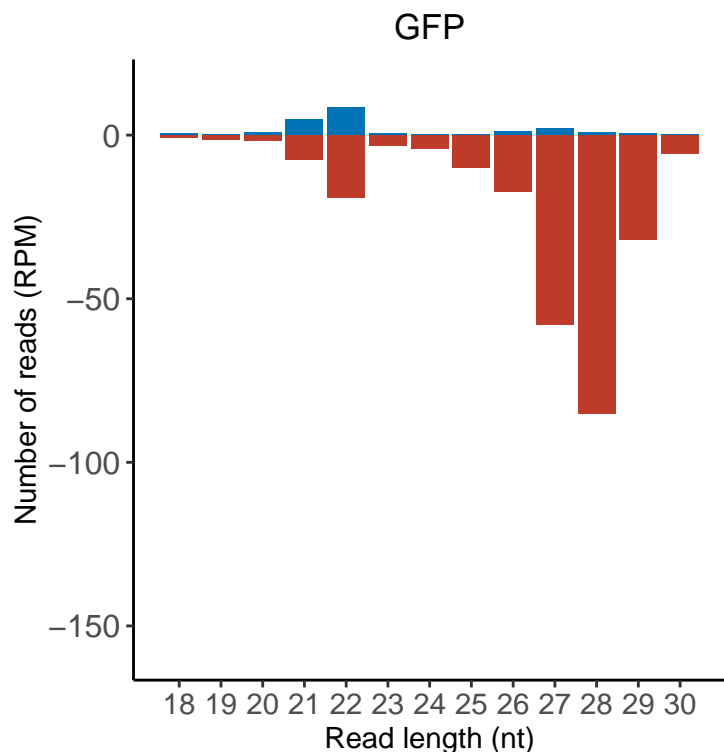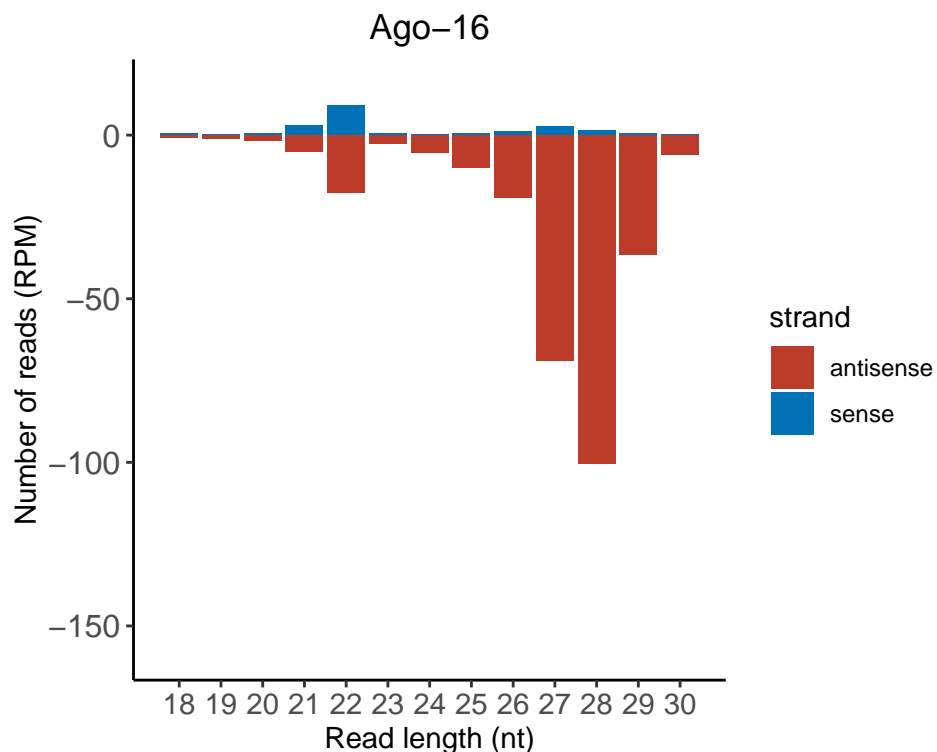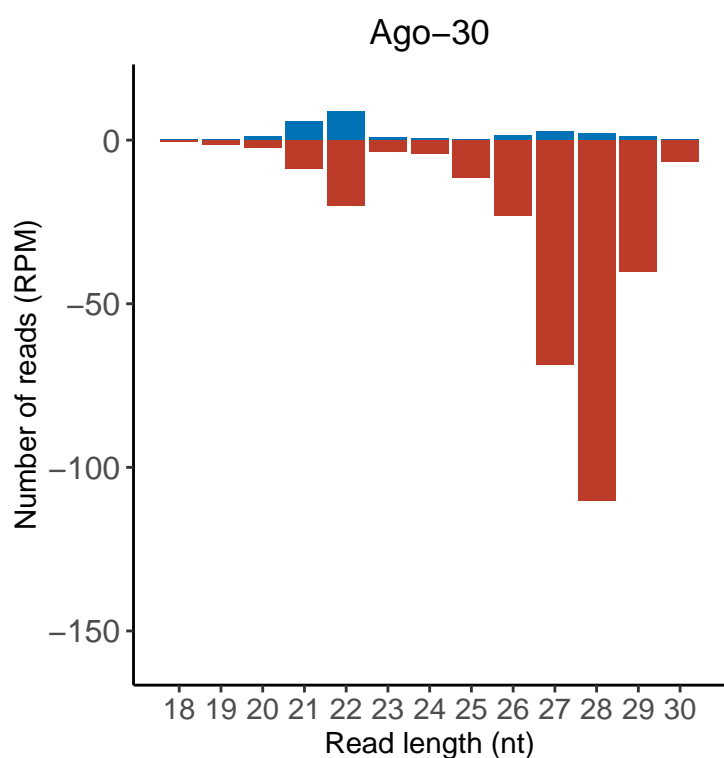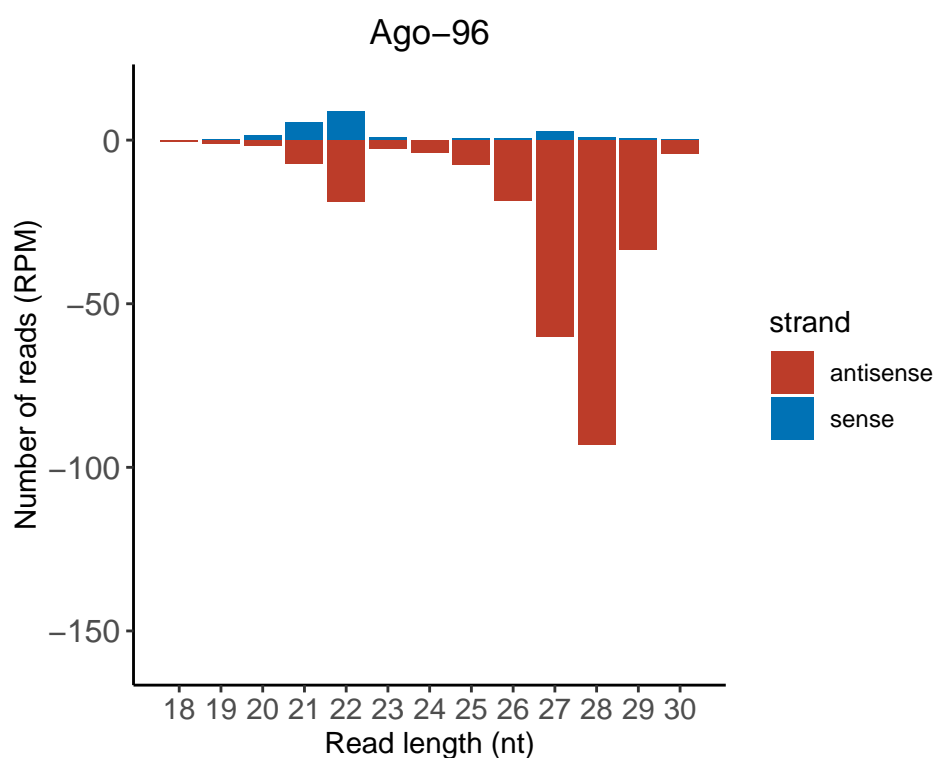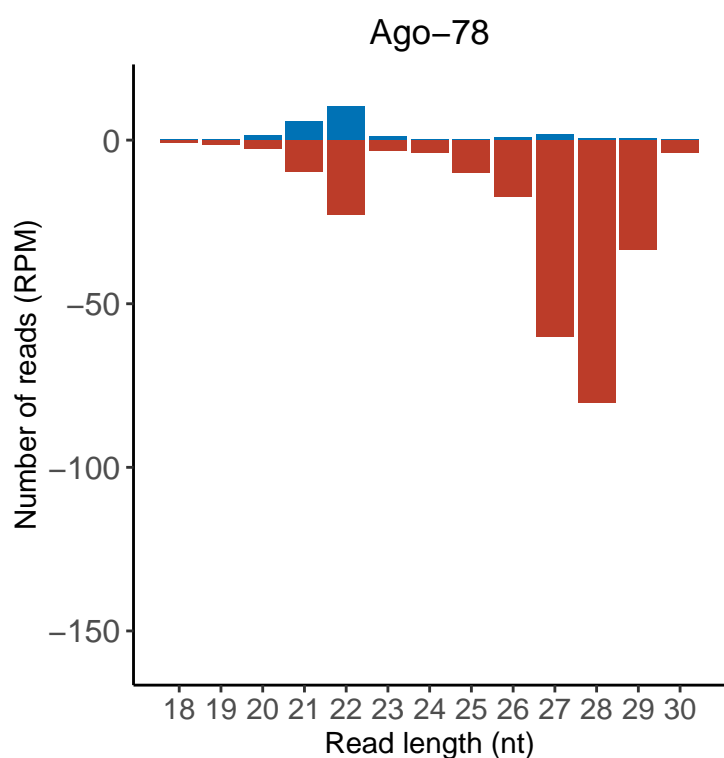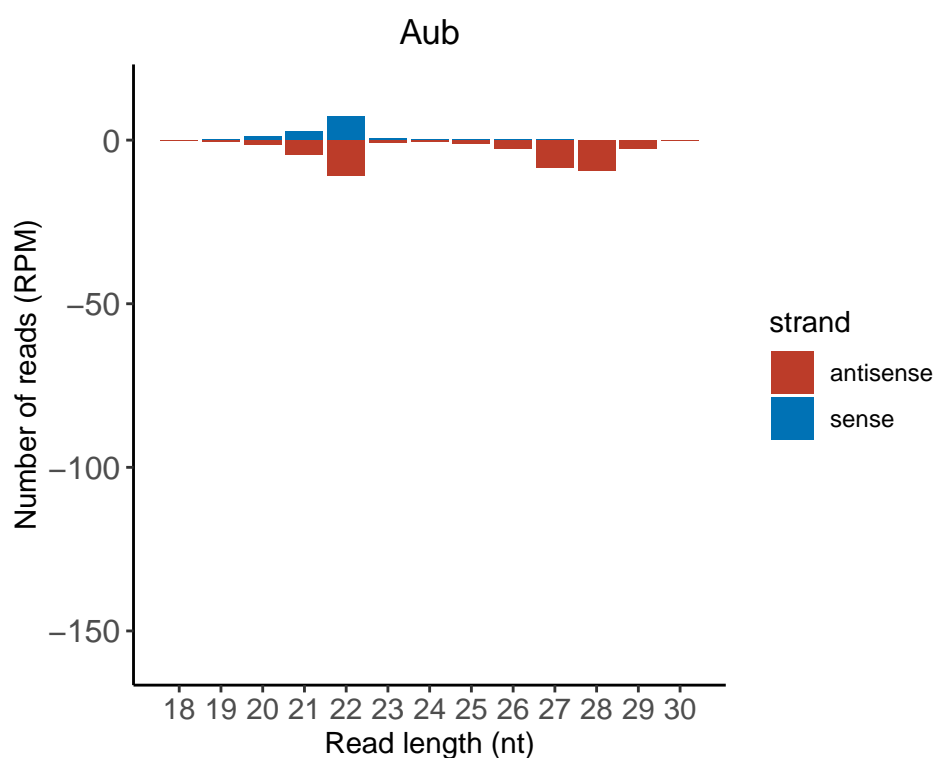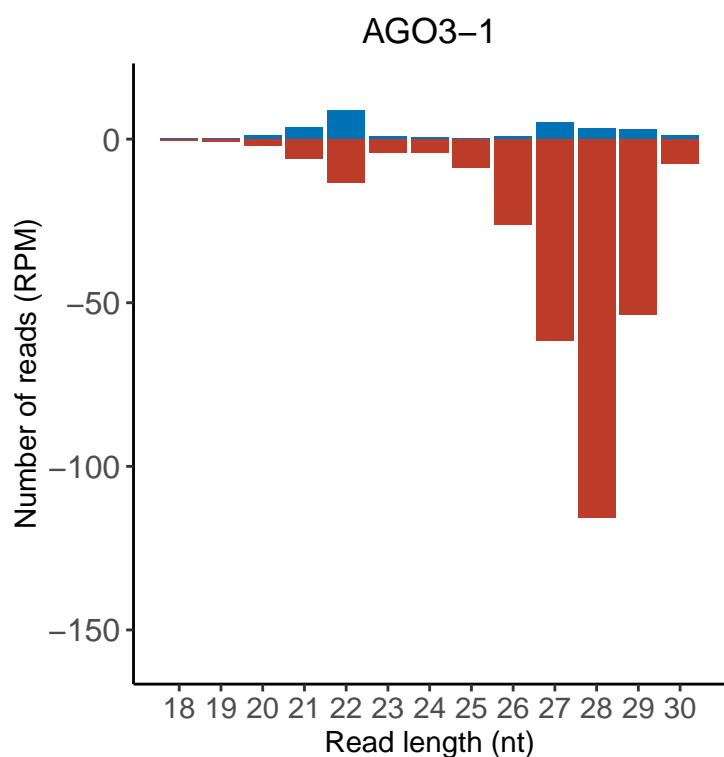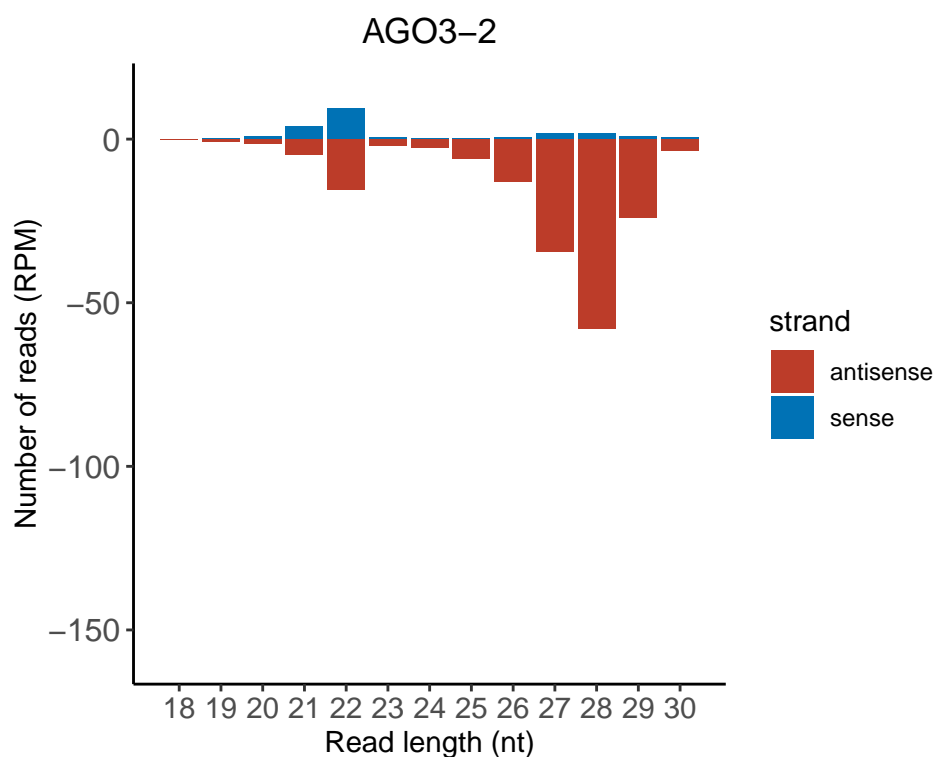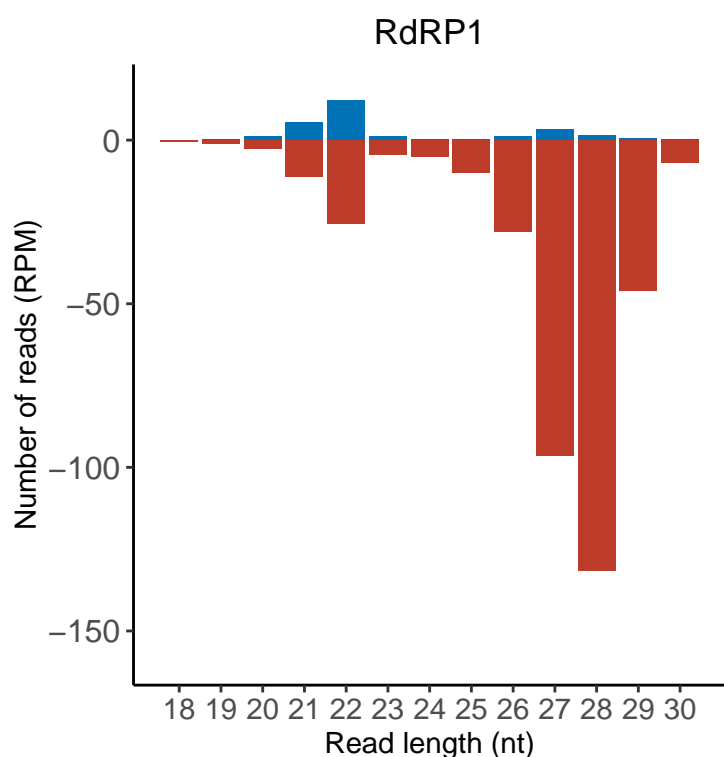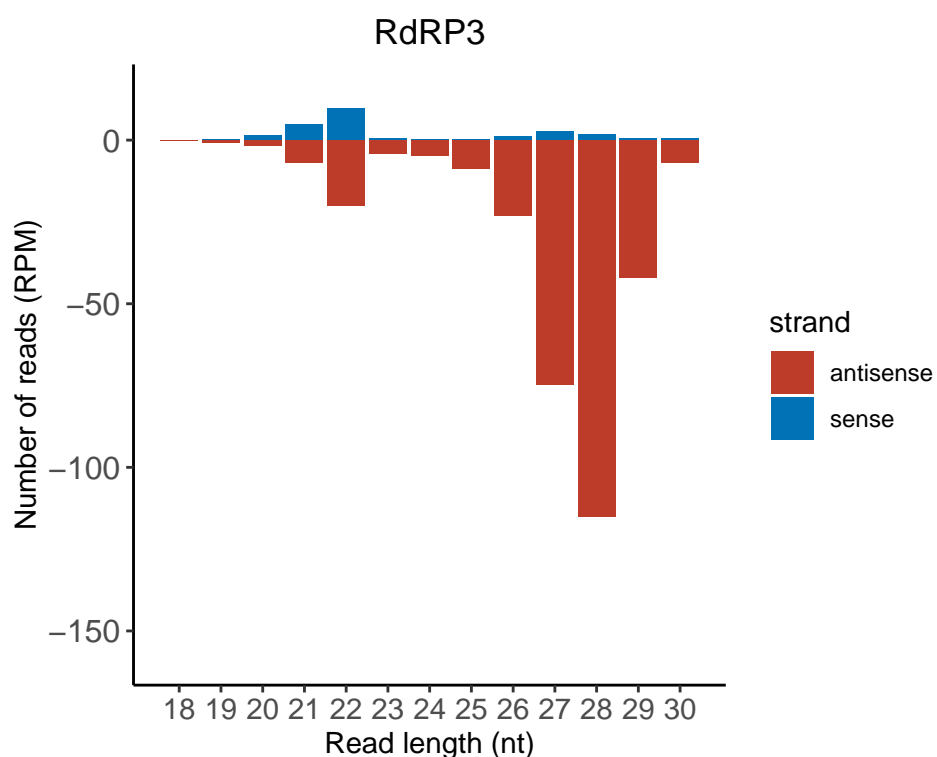

Supplement: S1 Data — On the Normalized and Relative levels pages, normalized read counts (RPM) and relative levels compared to the control (dsGFP) library were used. TEs with more than 50RPM and Coding Genes with more than 3.5RPM on average in KD libraries are included in this website. For viral sequences, reference sequences were generated by assembling sRNA sequences (See the Analysis of sRNAseq data section in Materials and methods.) and size distributions of sRNA reads mapped to each contig are shown. Reads were first grouped by their 5’ nucleotides, and reads of each length were counted. Full tables including TEs and Coding genes with fewer reads are in the “FullTable” folder. (ZIP) [file pone.0281195.s017.zip › SupplementaryData/Links/PDFs/Motif:rnd-1_family-669TE_lengthdistribution.pdf]

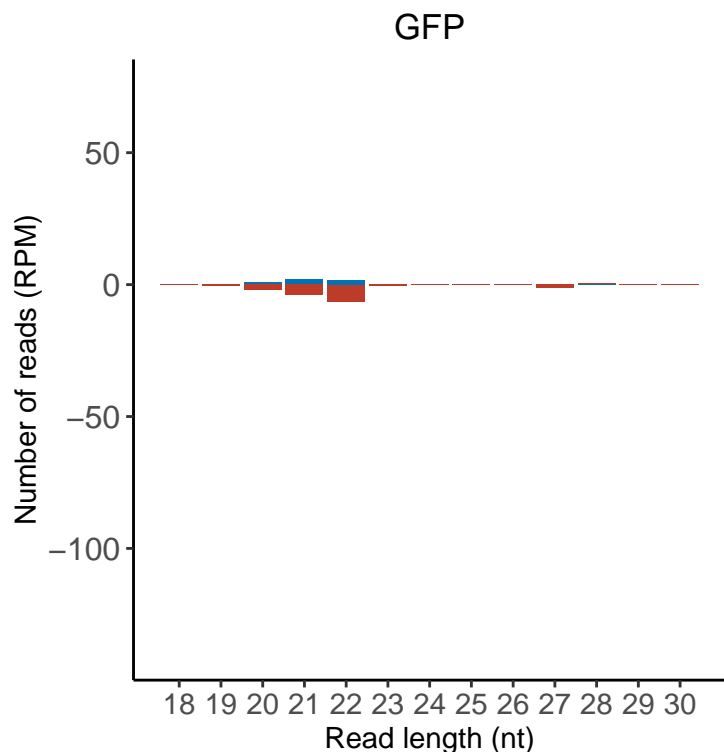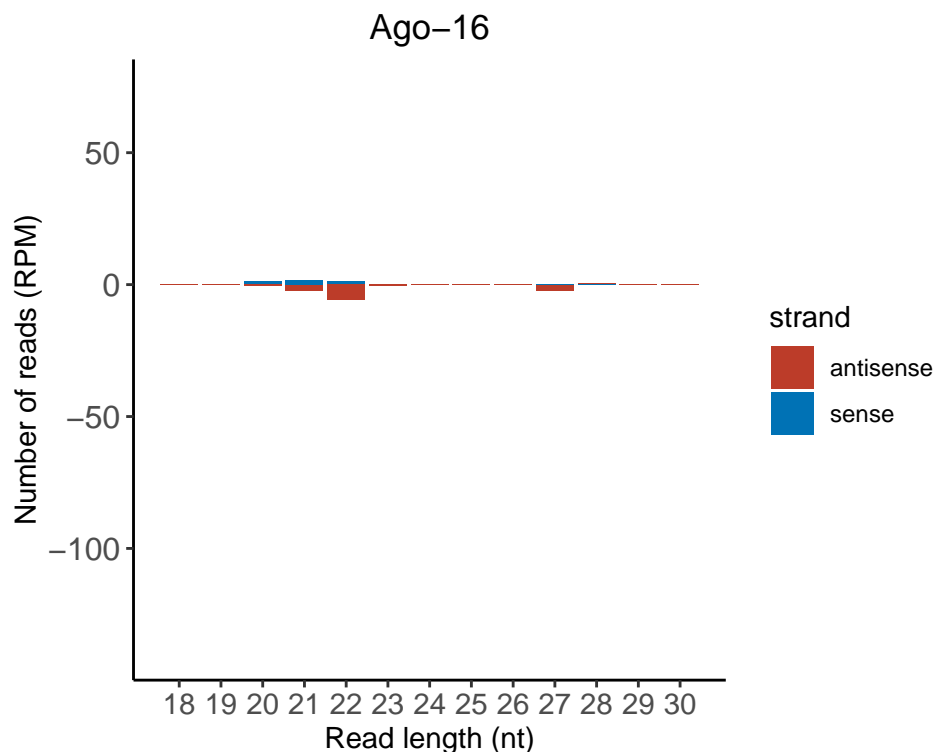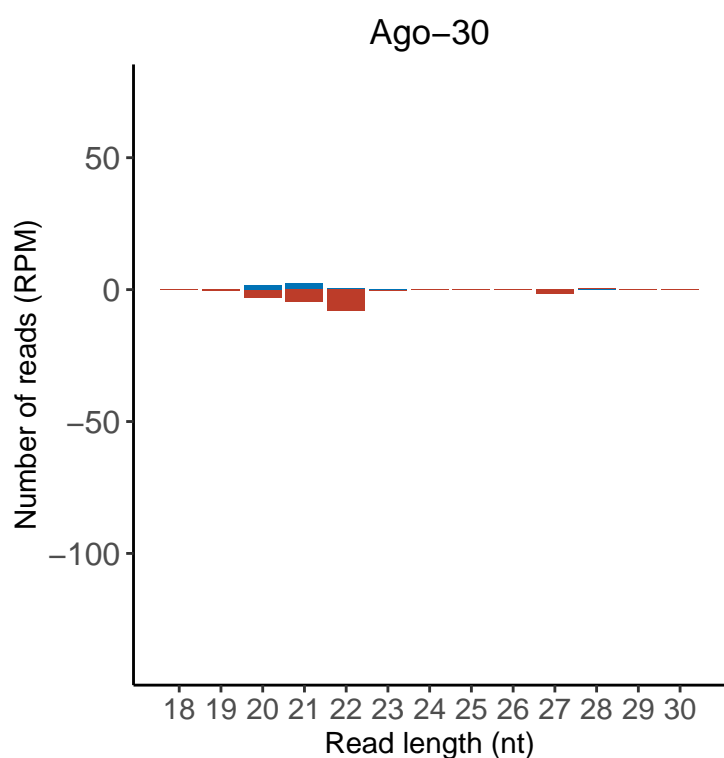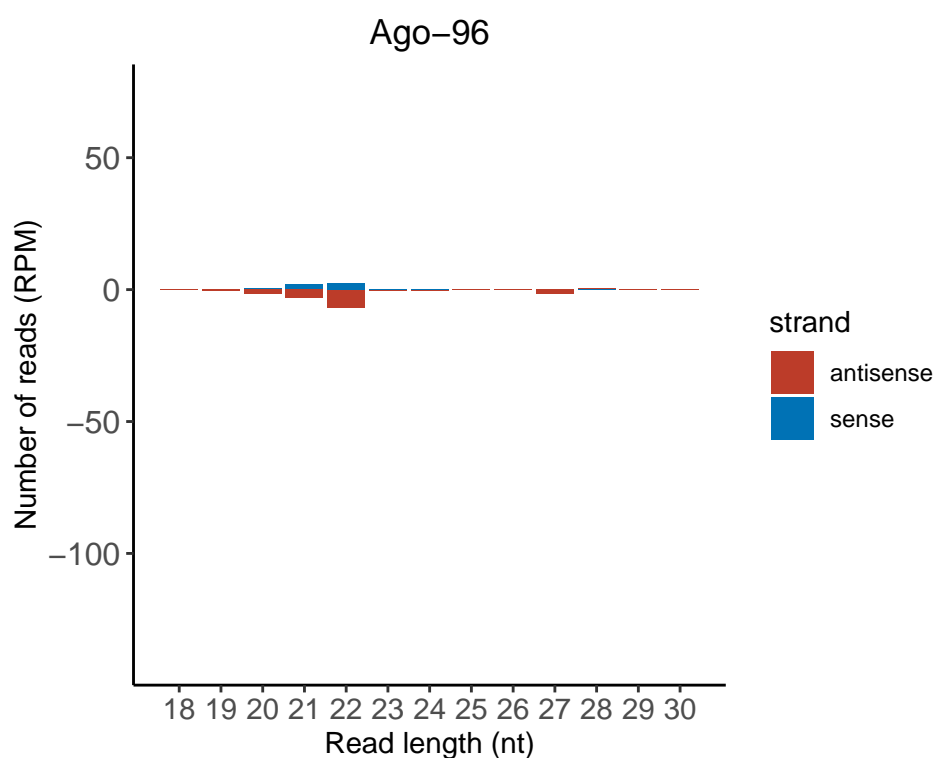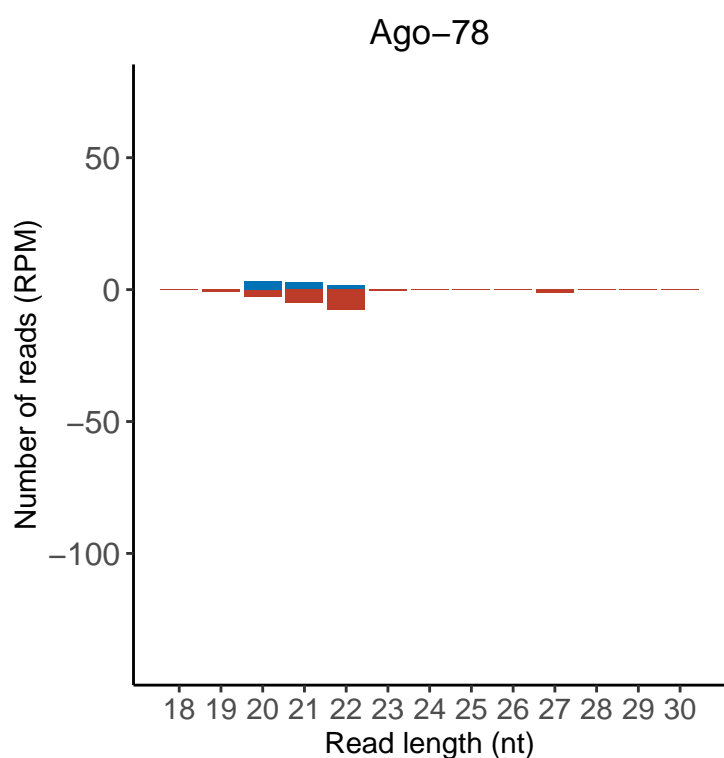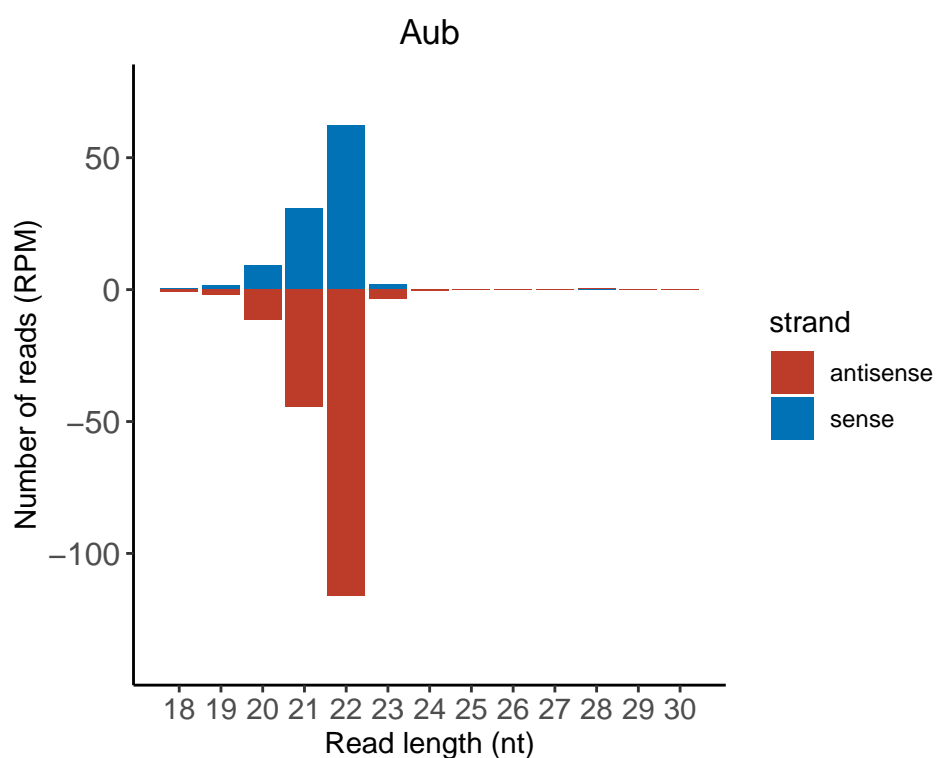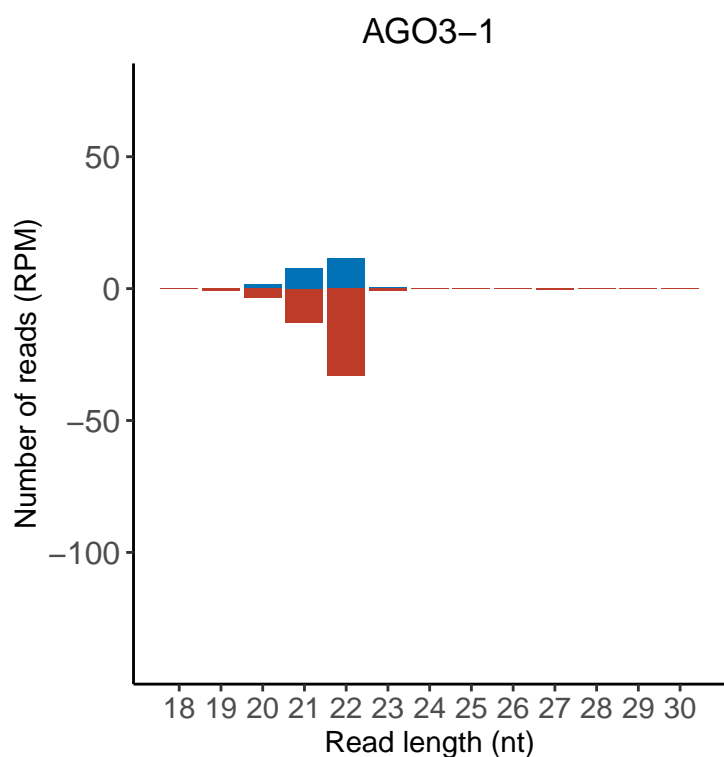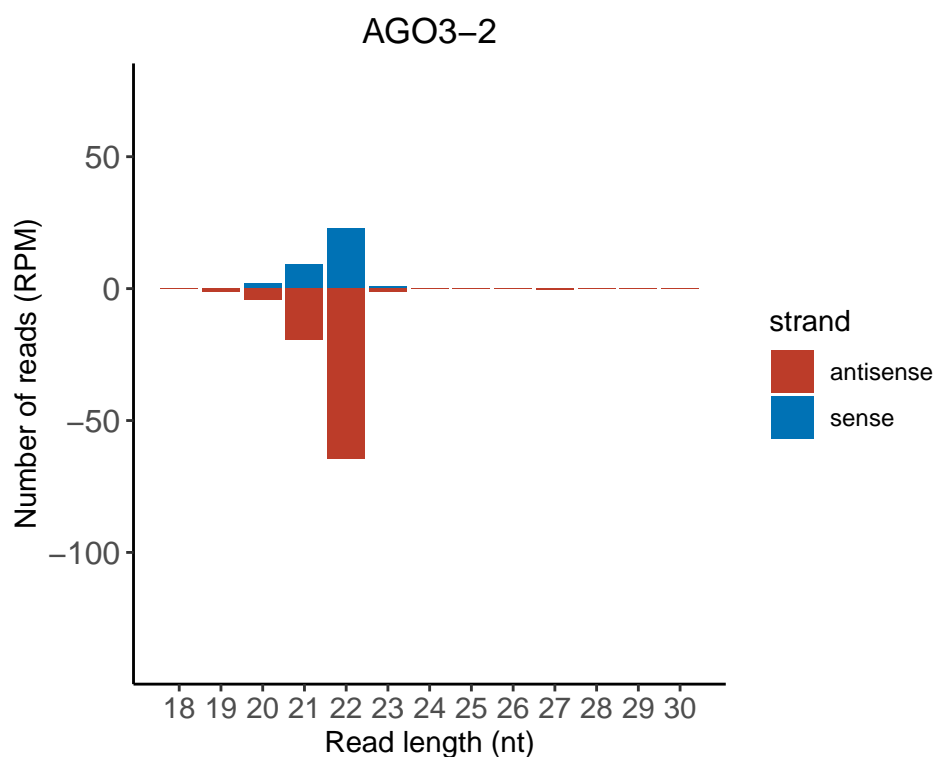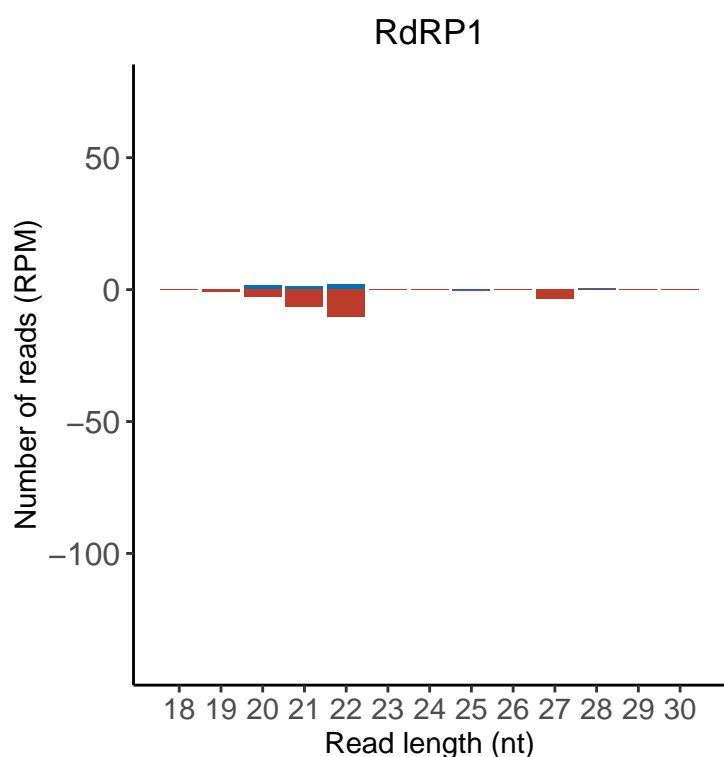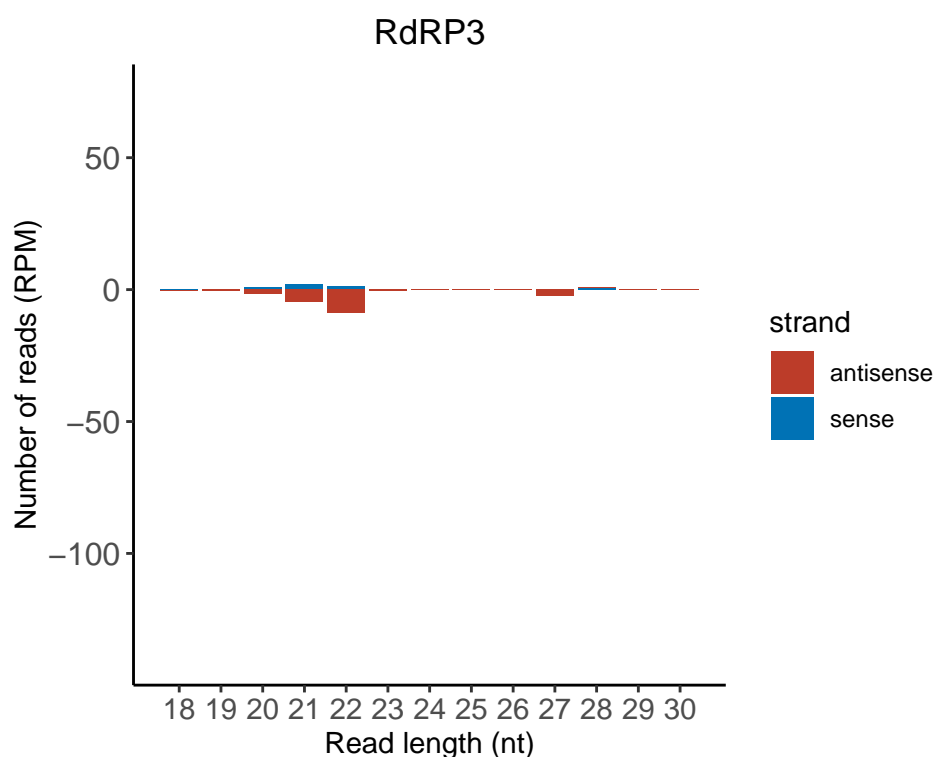

Supplement: S1 Data — On the Normalized and Relative levels pages, normalized read counts (RPM) and relative levels compared to the control (dsGFP) library were used. TEs with more than 50RPM and Coding Genes with more than 3.5RPM on average in KD libraries are included in this website. For viral sequences, reference sequences were generated by assembling sRNA sequences (See the Analysis of sRNAseq data section in Materials and methods.) and size distributions of sRNA reads mapped to each contig are shown. Reads were first grouped by their 5’ nucleotides, and reads of each length were counted. Full tables including TEs and Coding genes with fewer reads are in the “FullTable” folder. (ZIP) [file pone.0281195.s017.zip › SupplementaryData/Links/PDFs/ISCI003367.RACDS_lengthdistribution.pdf]

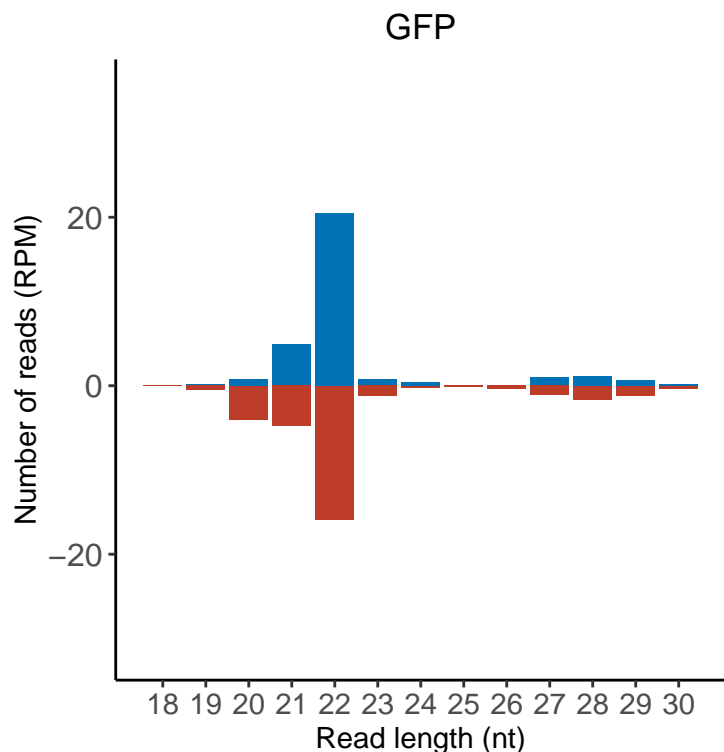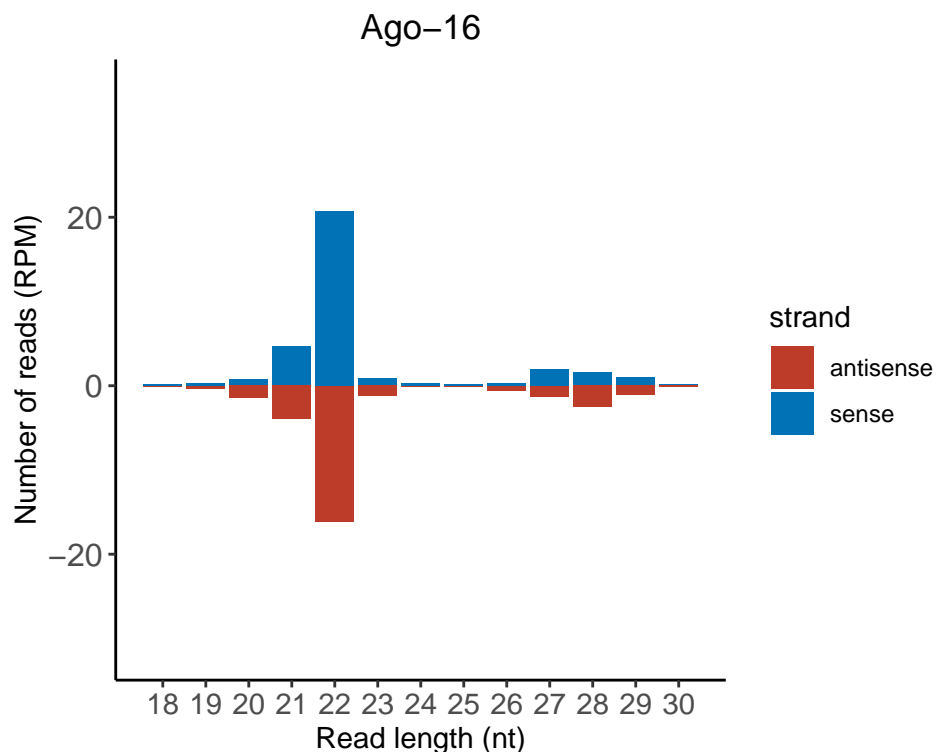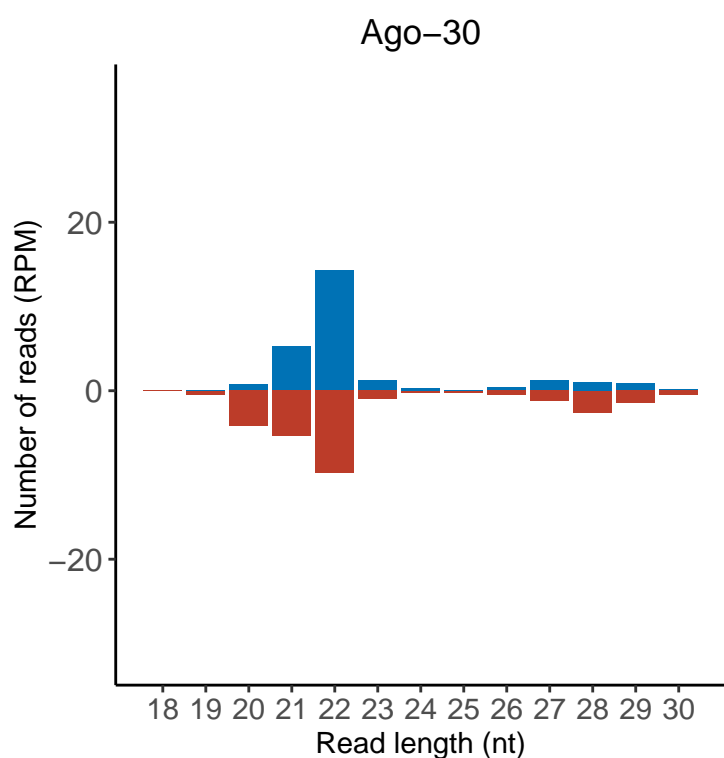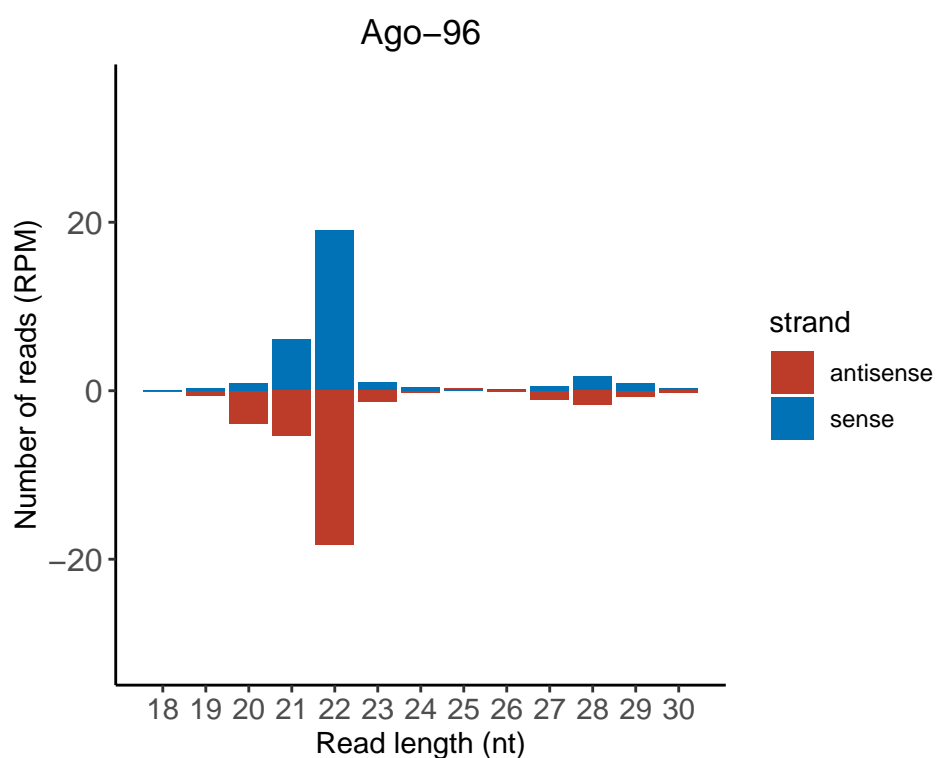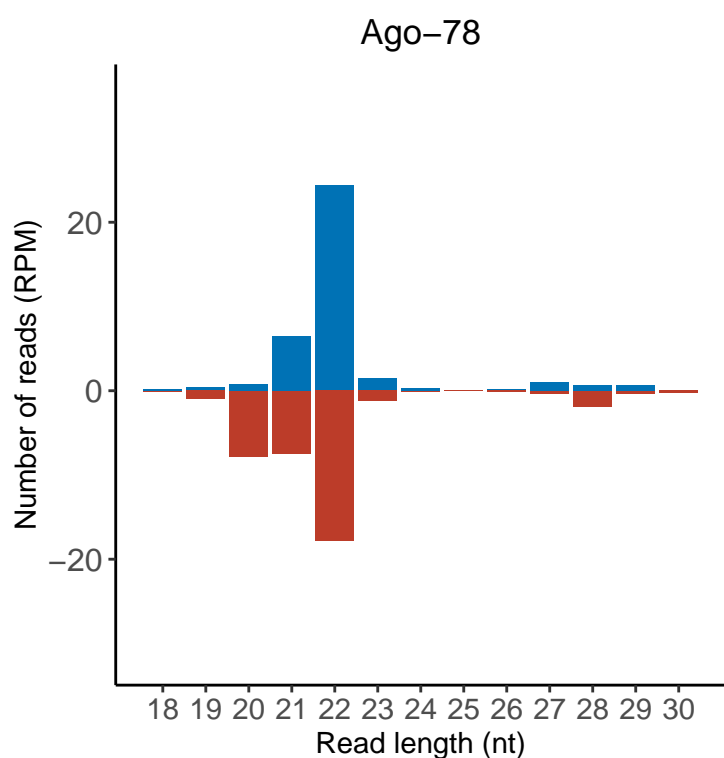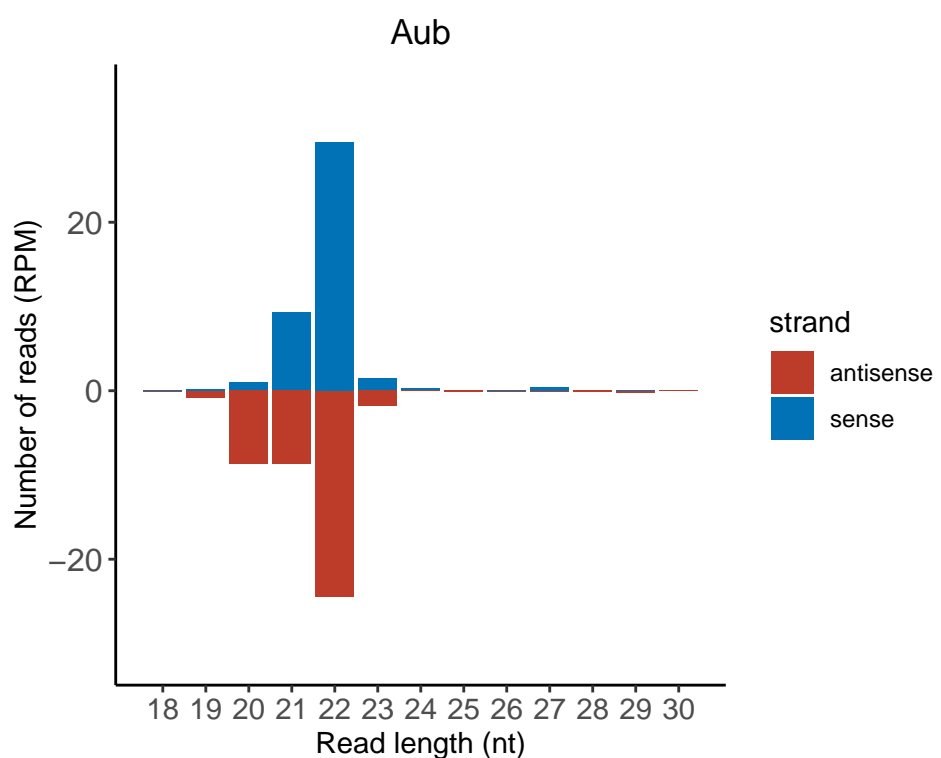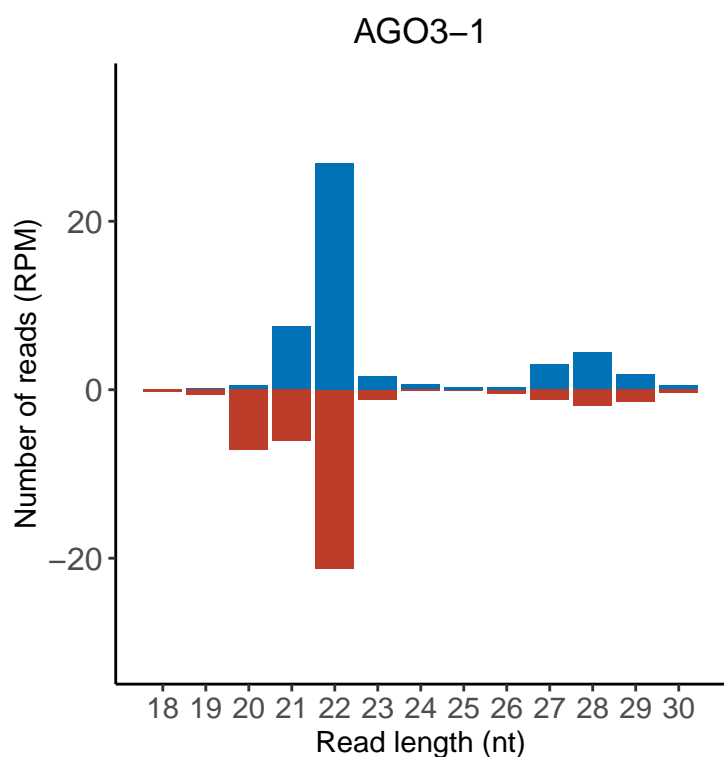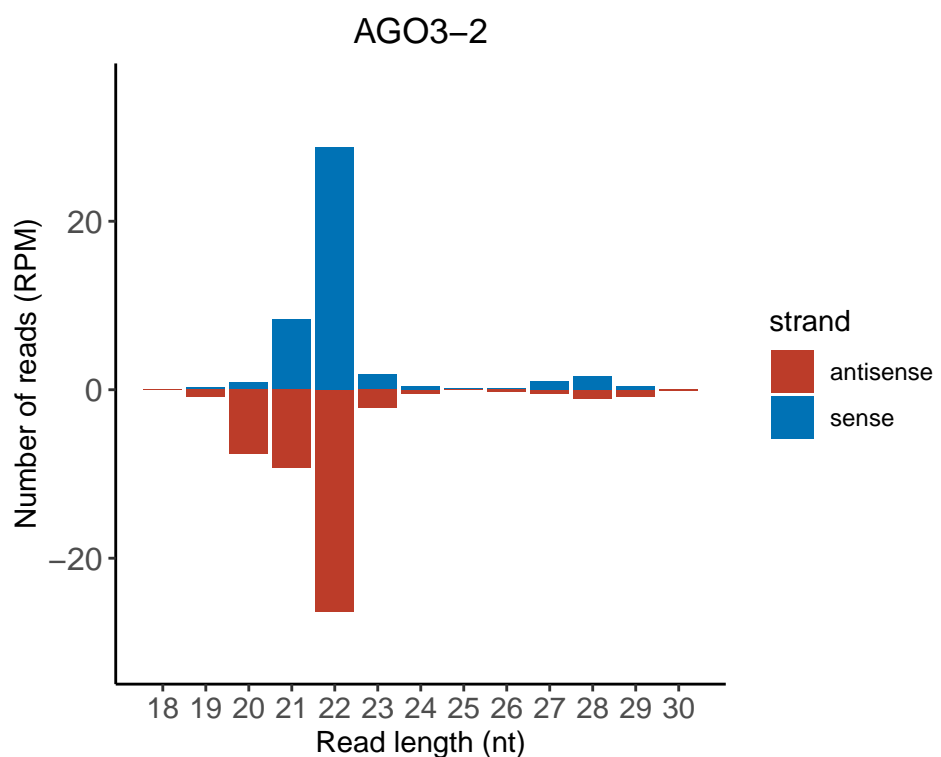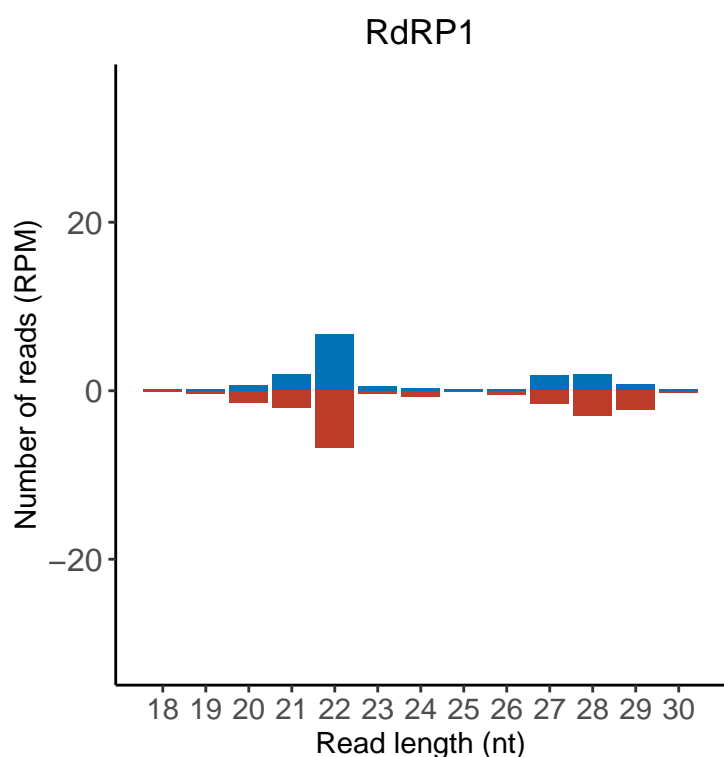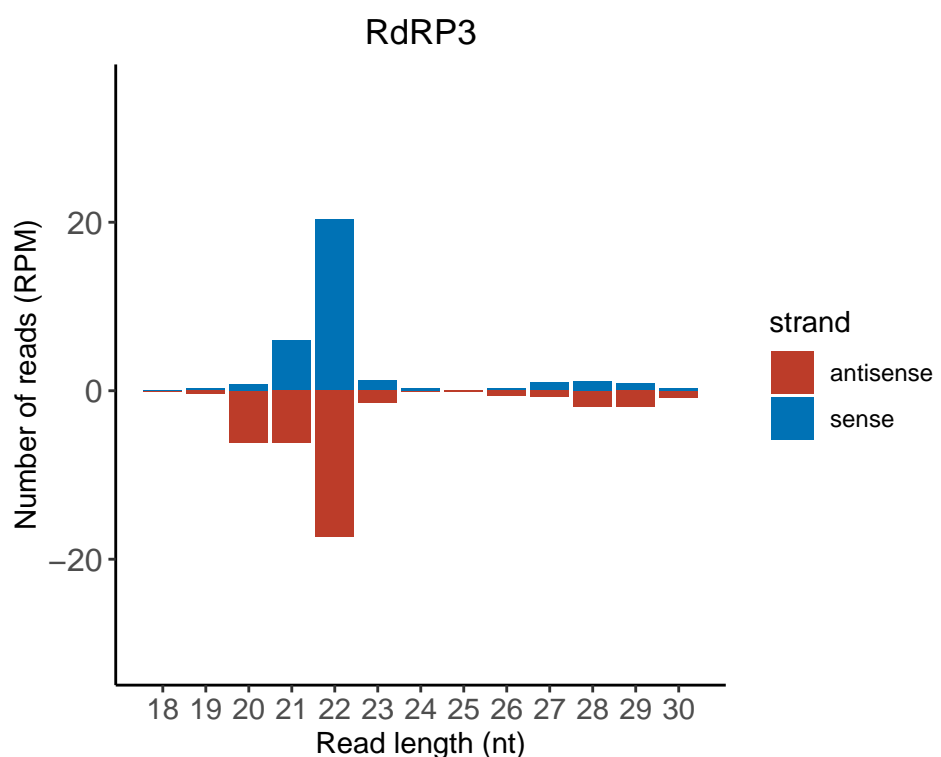

Supplement: S1 Data — On the Normalized and Relative levels pages, normalized read counts (RPM) and relative levels compared to the control (dsGFP) library were used. TEs with more than 50RPM and Coding Genes with more than 3.5RPM on average in KD libraries are included in this website. For viral sequences, reference sequences were generated by assembling sRNA sequences (See the Analysis of sRNAseq data section in Materials and methods.) and size distributions of sRNA reads mapped to each contig are shown. Reads were first grouped by their 5’ nucleotides, and reads of each length were counted. Full tables including TEs and Coding genes with fewer reads are in the “FullTable” folder. (ZIP) [file pone.0281195.s017.zip › SupplementaryData/Links/PDFs/Motif:rnd-1_family-607TE_lengthdistribution.pdf]

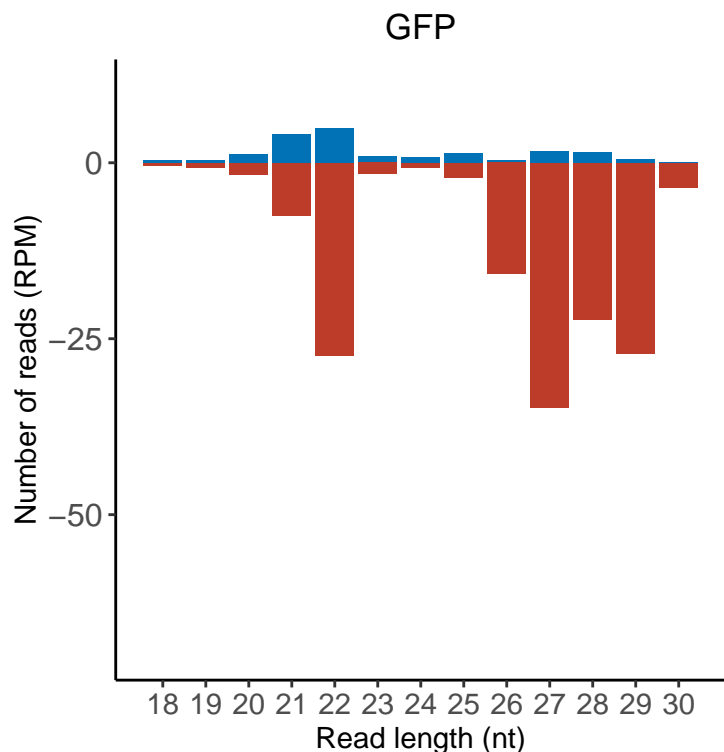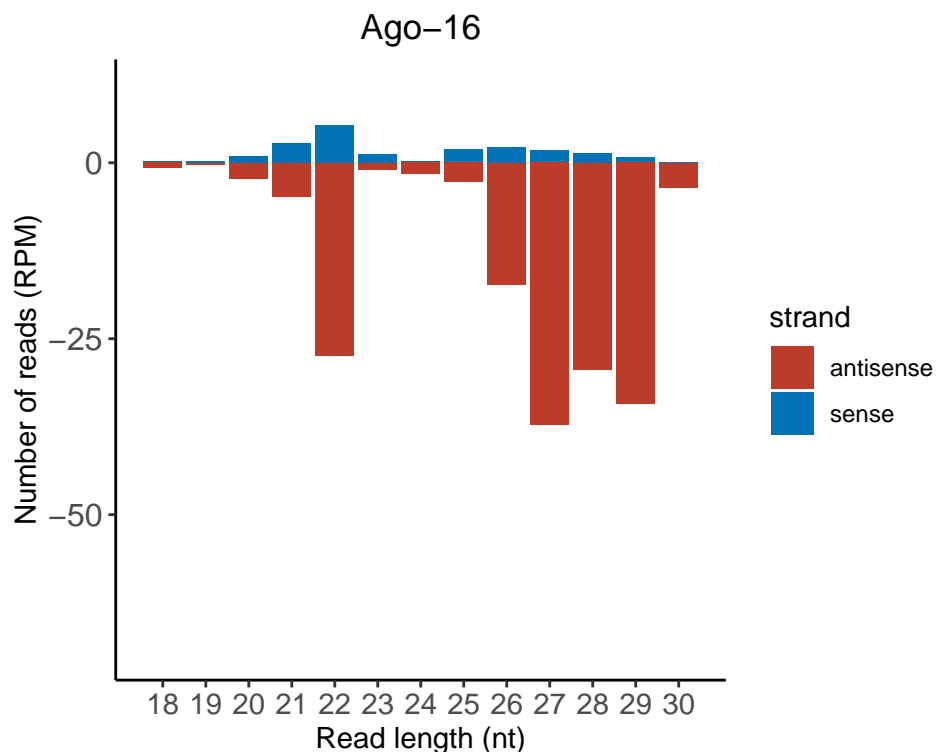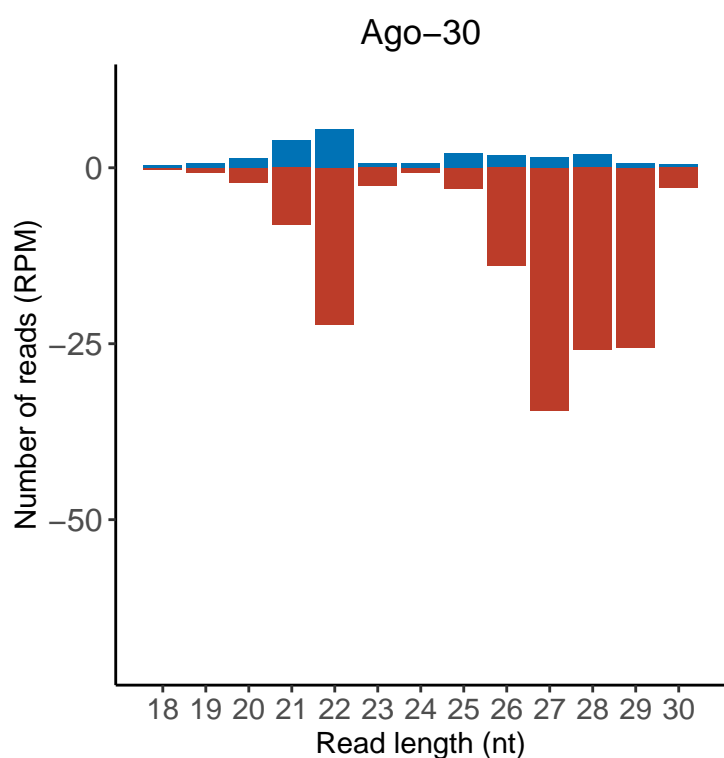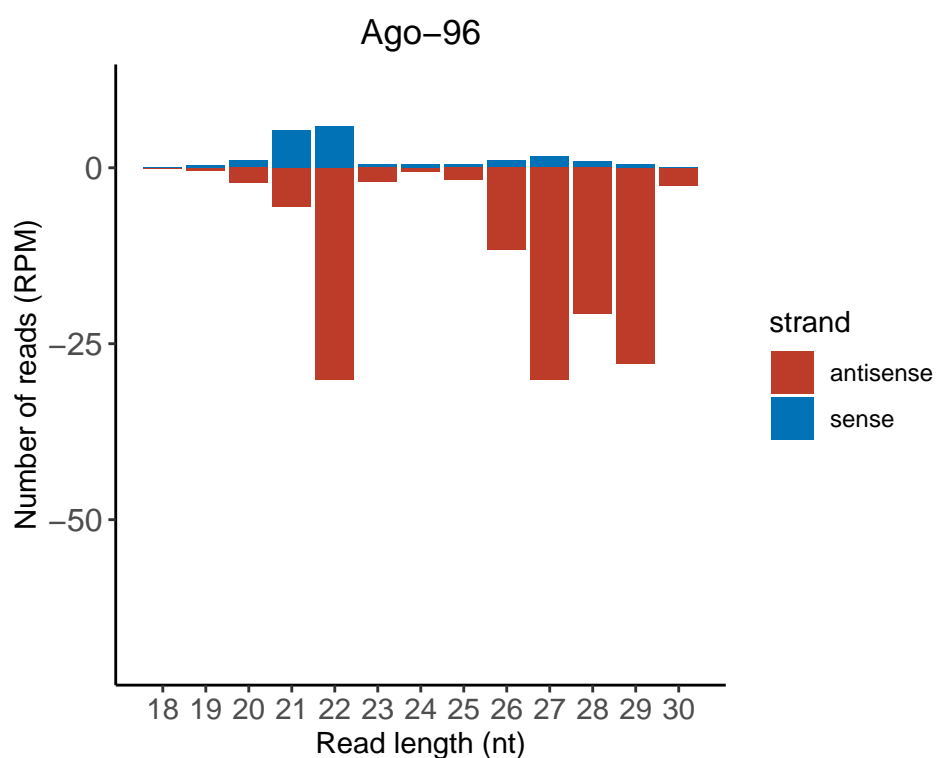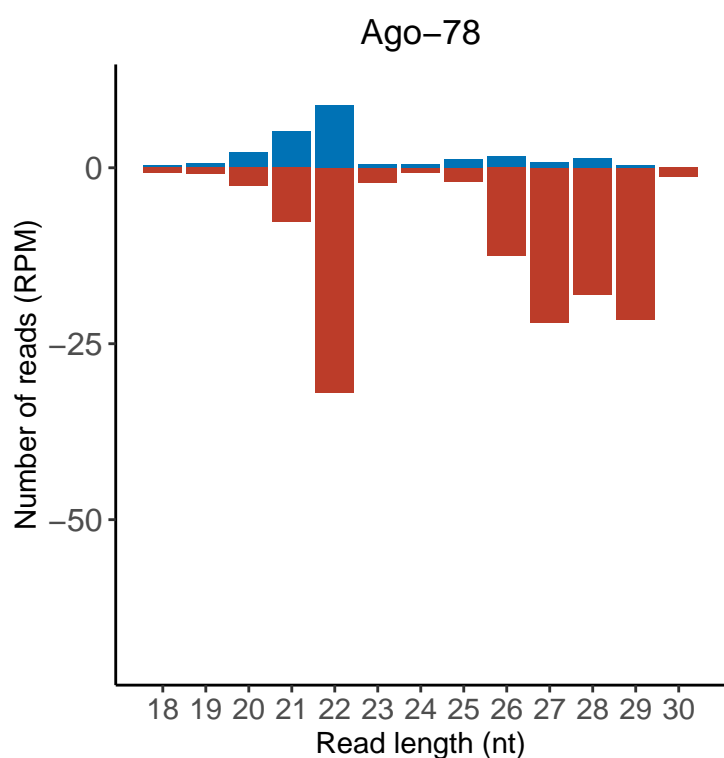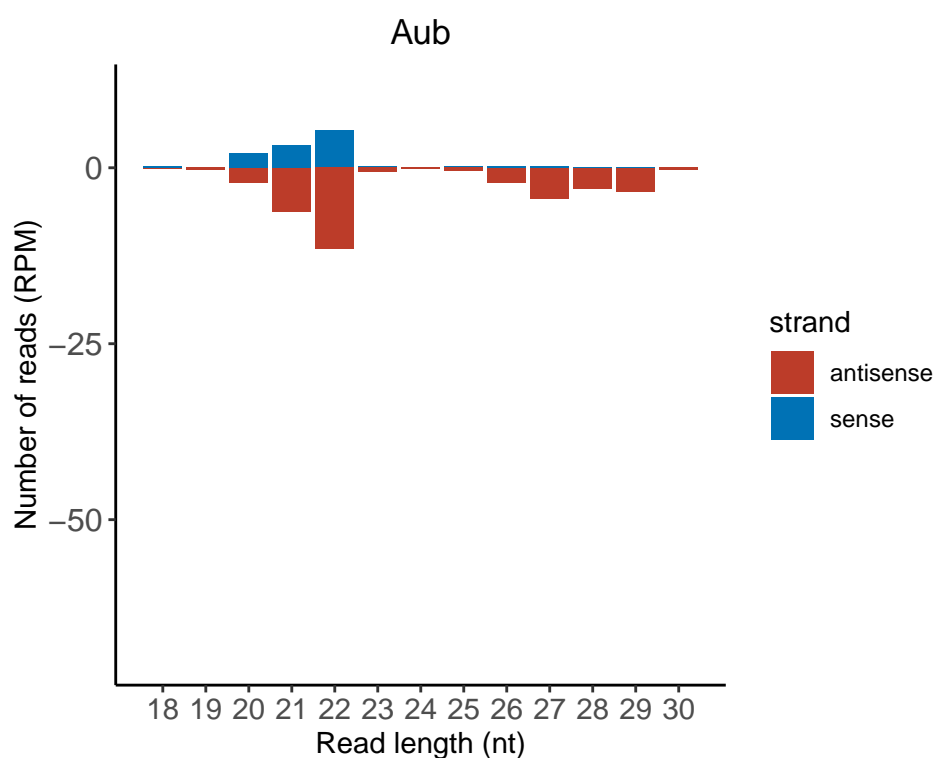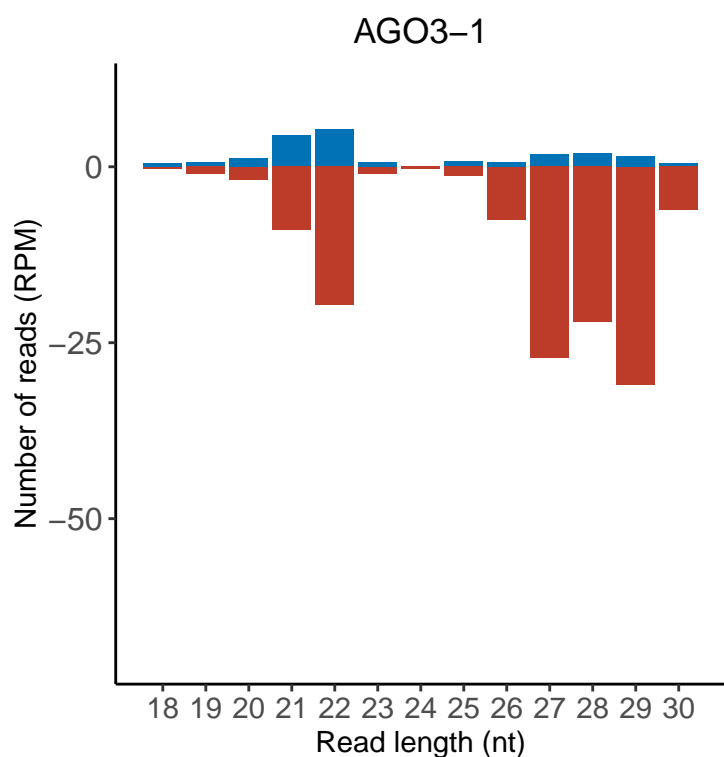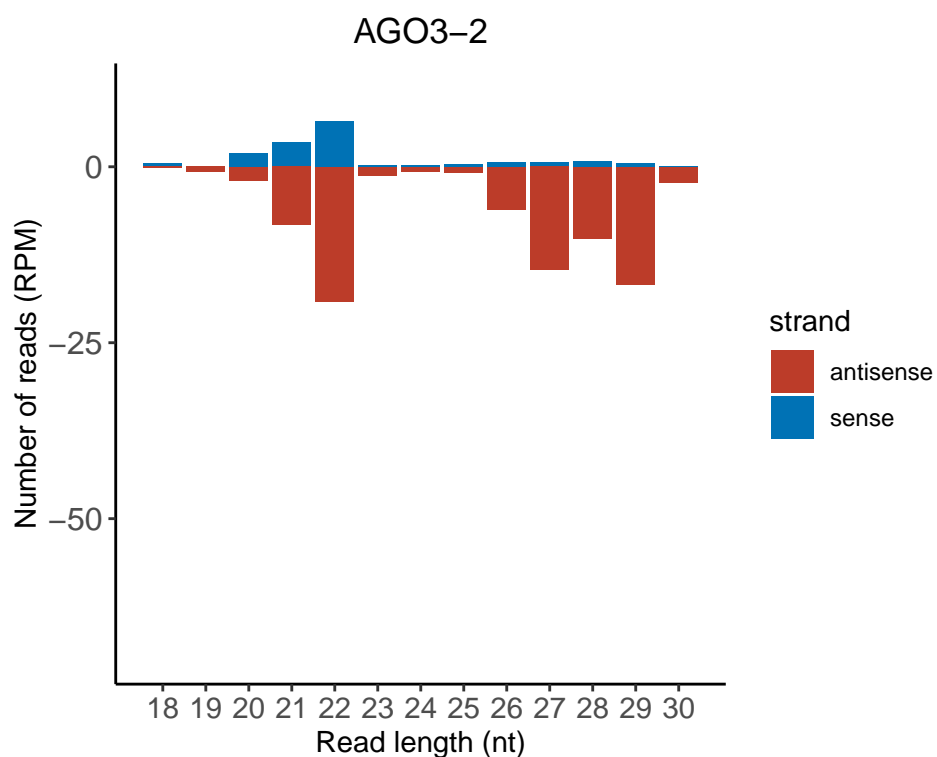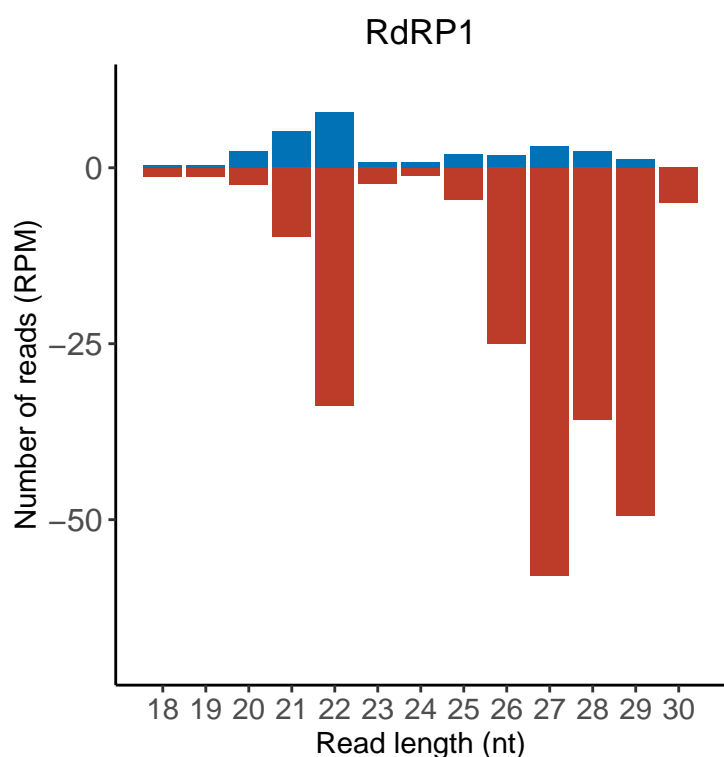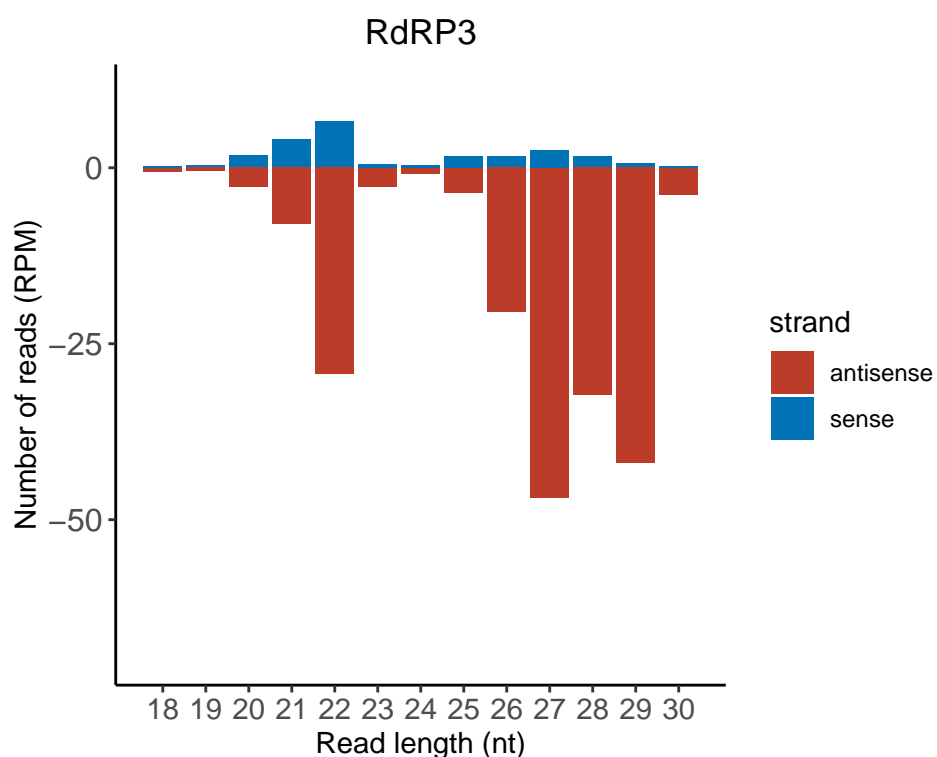

Supplement: S1 Data — On the Normalized and Relative levels pages, normalized read counts (RPM) and relative levels compared to the control (dsGFP) library were used. TEs with more than 50RPM and Coding Genes with more than 3.5RPM on average in KD libraries are included in this website. For viral sequences, reference sequences were generated by assembling sRNA sequences (See the Analysis of sRNAseq data section in Materials and methods.) and size distributions of sRNA reads mapped to each contig are shown. Reads were first grouped by their 5’ nucleotides, and reads of each length were counted. Full tables including TEs and Coding genes with fewer reads are in the “FullTable” folder. (ZIP) [file pone.0281195.s017.zip › SupplementaryData/Links/PDFs/Motif:rnd-5_family-3705TE_lengthdistribution.pdf]

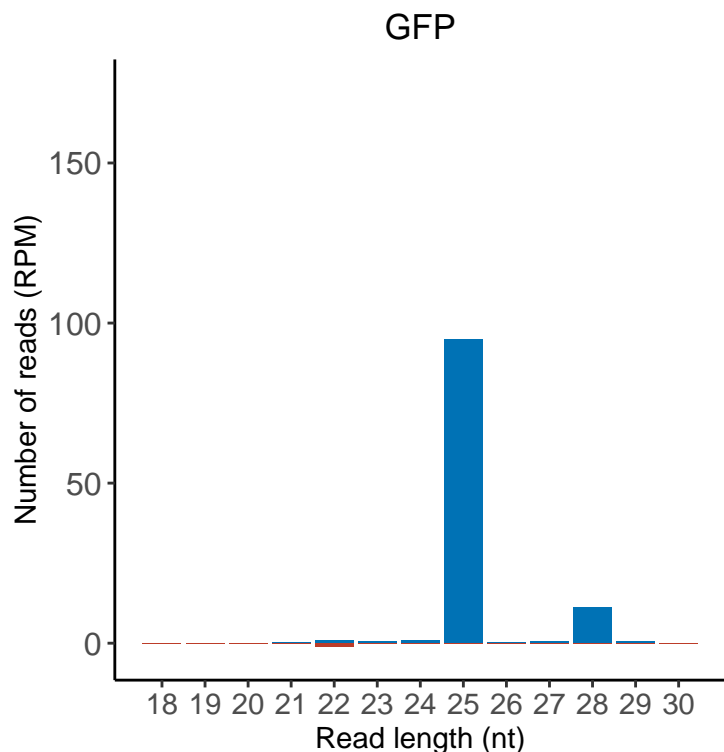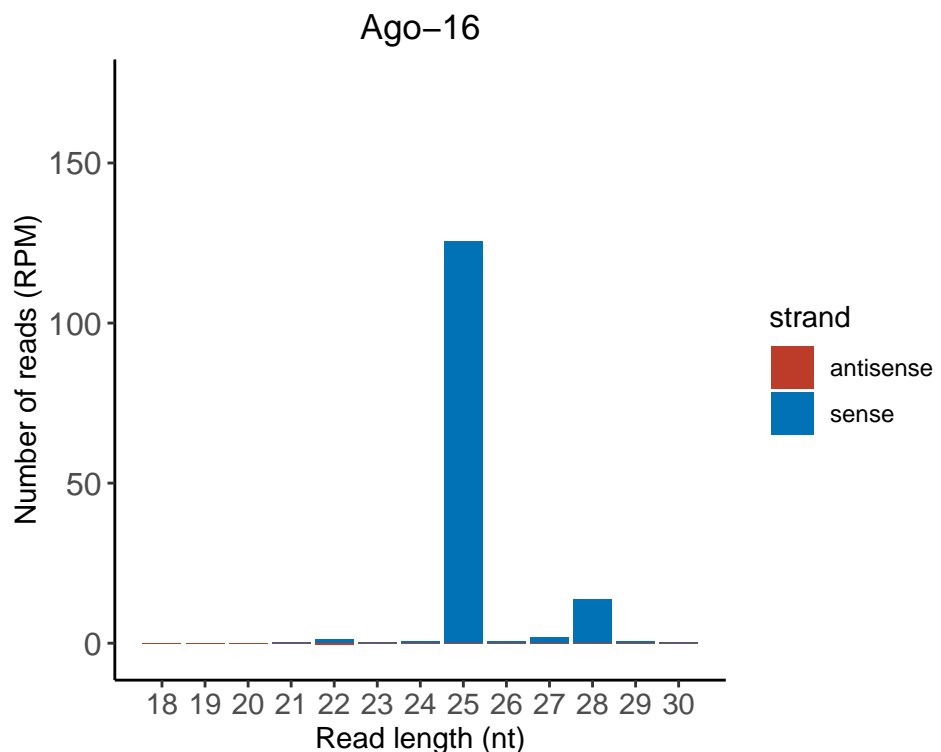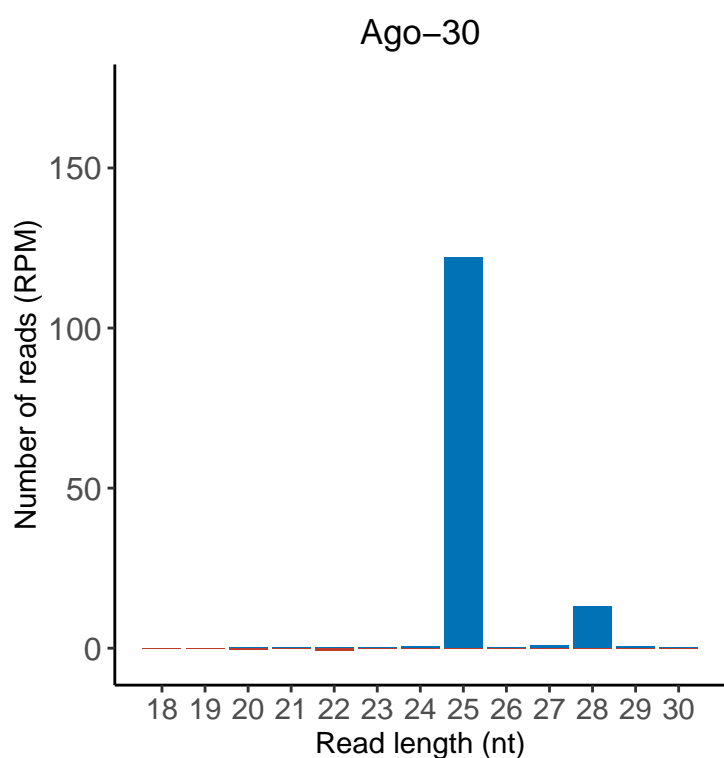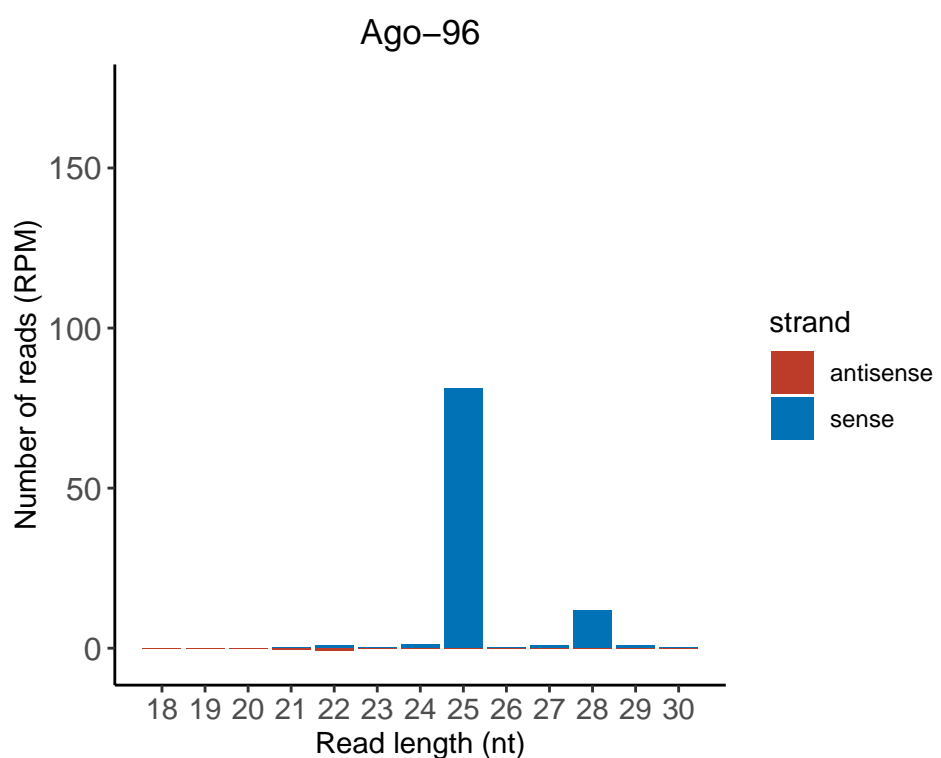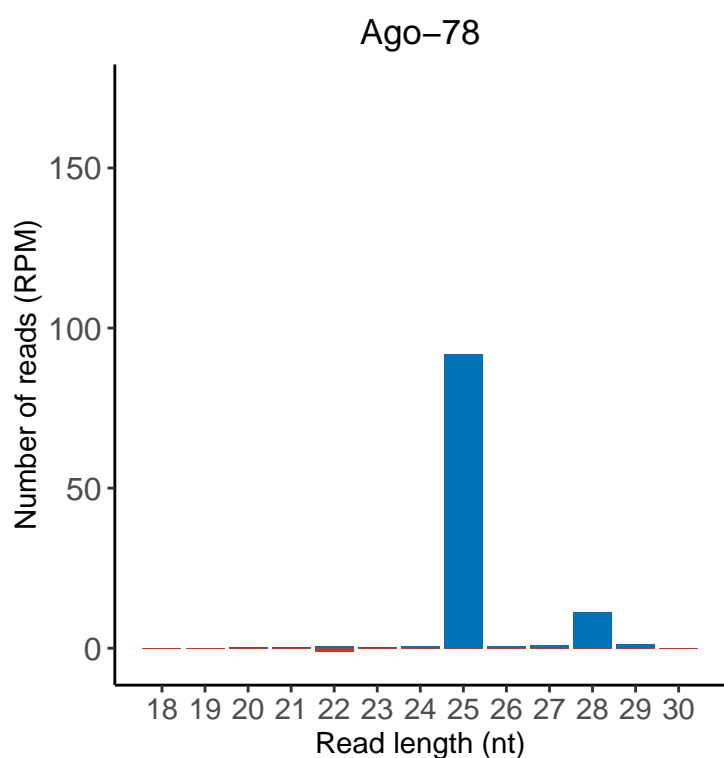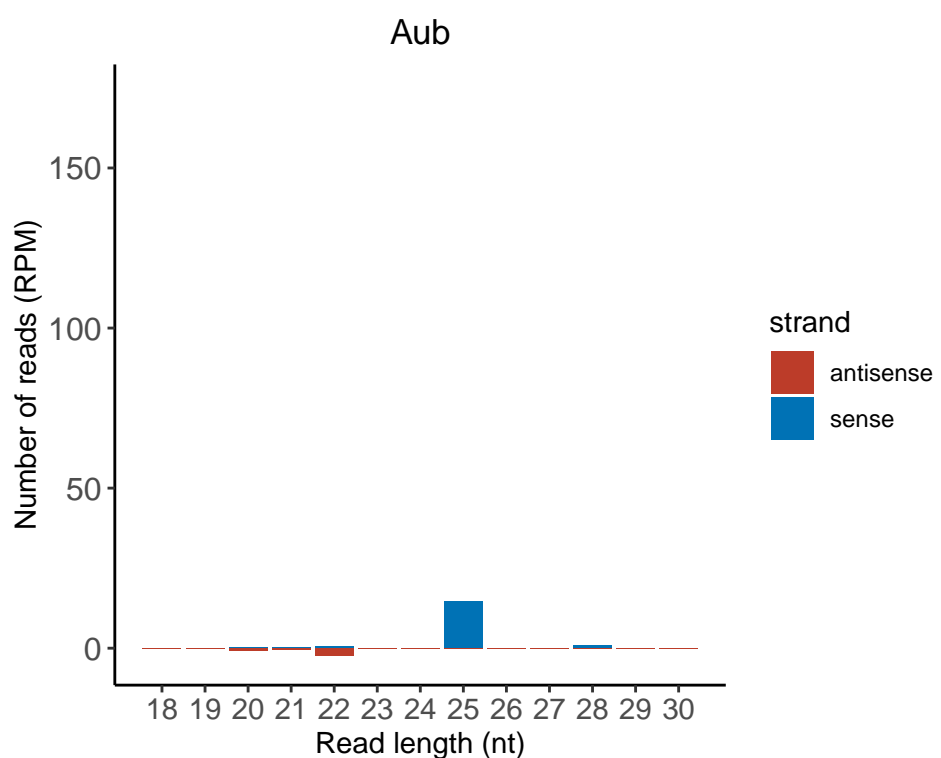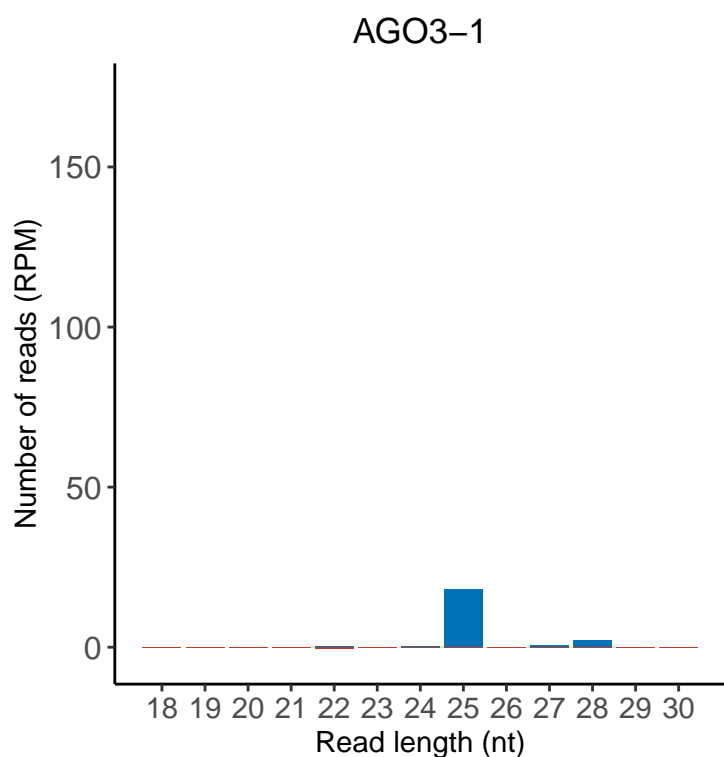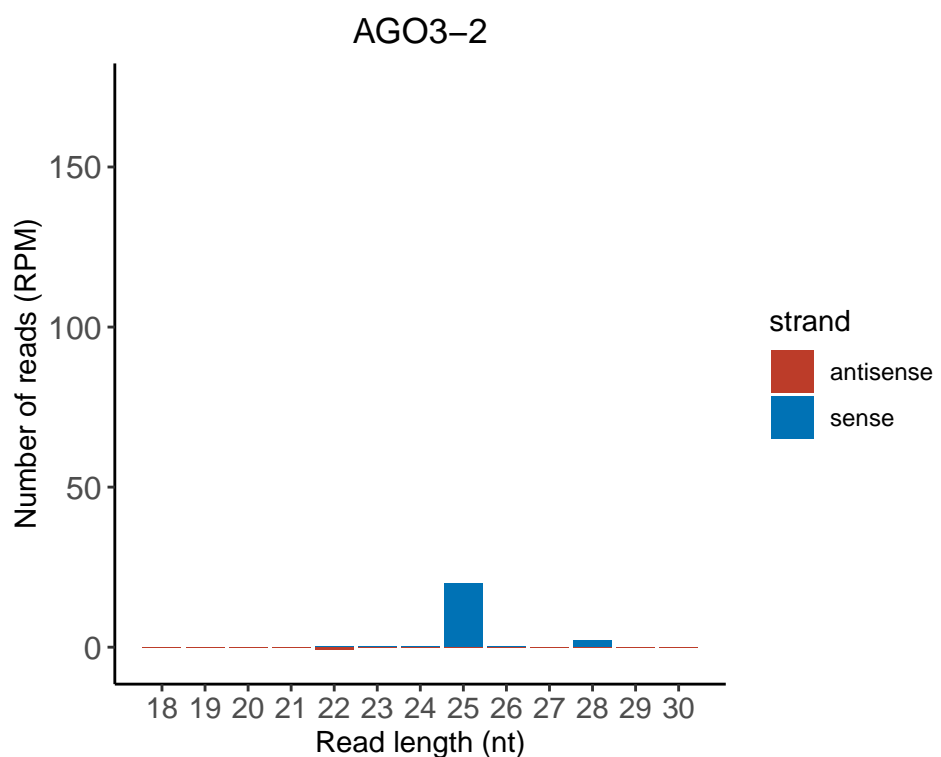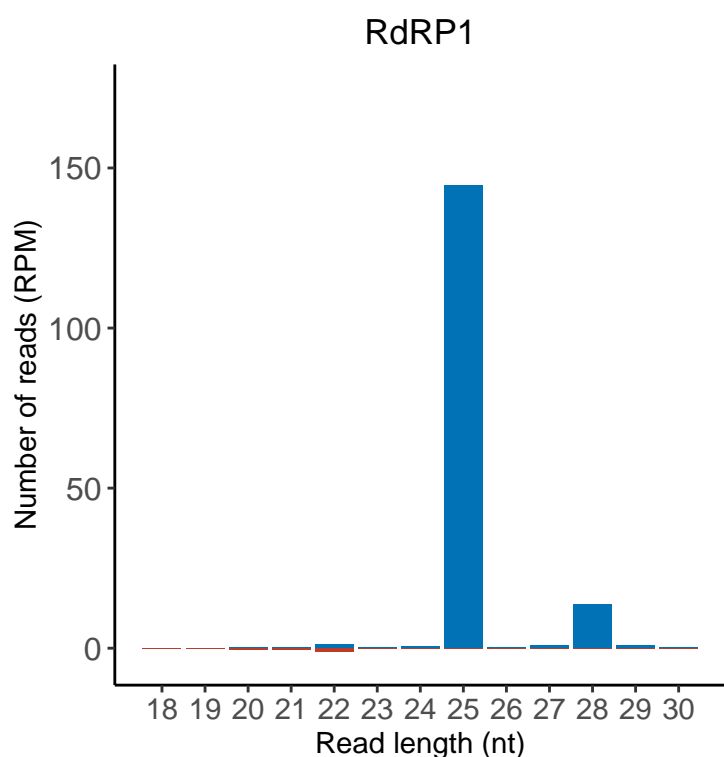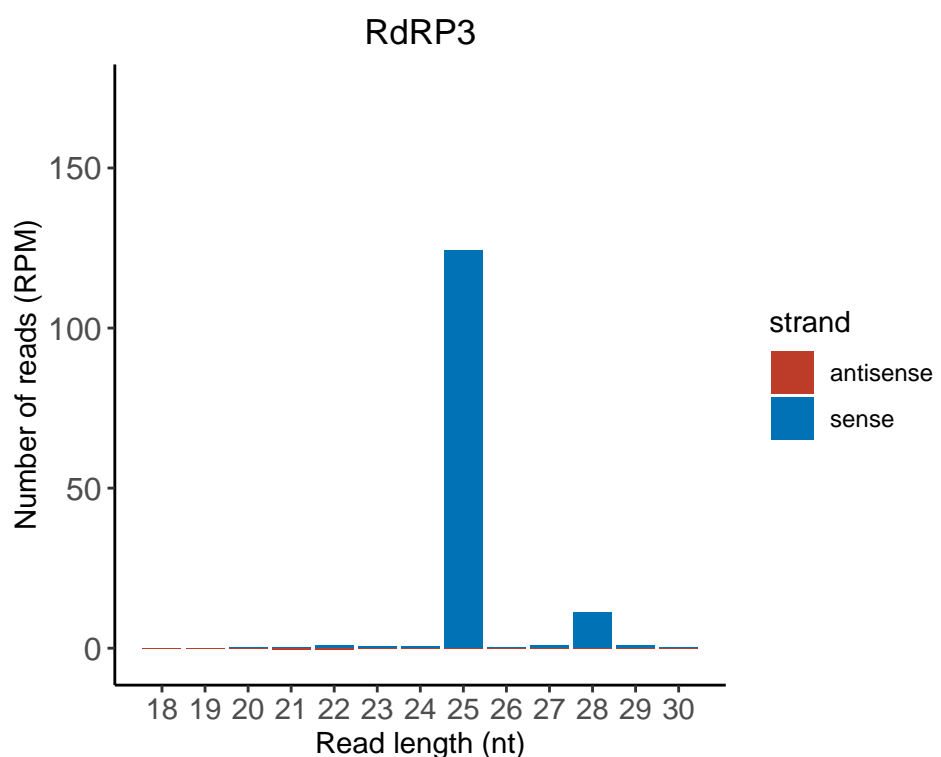

Supplement: S1 Data — On the Normalized and Relative levels pages, normalized read counts (RPM) and relative levels compared to the control (dsGFP) library were used. TEs with more than 50RPM and Coding Genes with more than 3.5RPM on average in KD libraries are included in this website. For viral sequences, reference sequences were generated by assembling sRNA sequences (See the Analysis of sRNAseq data section in Materials and methods.) and size distributions of sRNA reads mapped to each contig are shown. Reads were first grouped by their 5’ nucleotides, and reads of each length were counted. Full tables including TEs and Coding genes with fewer reads are in the “FullTable” folder. (ZIP) [file pone.0281195.s017.zip › SupplementaryData/Links/PDFs/Motif:rnd-1_family-1391TE_lengthdistribution.pdf]

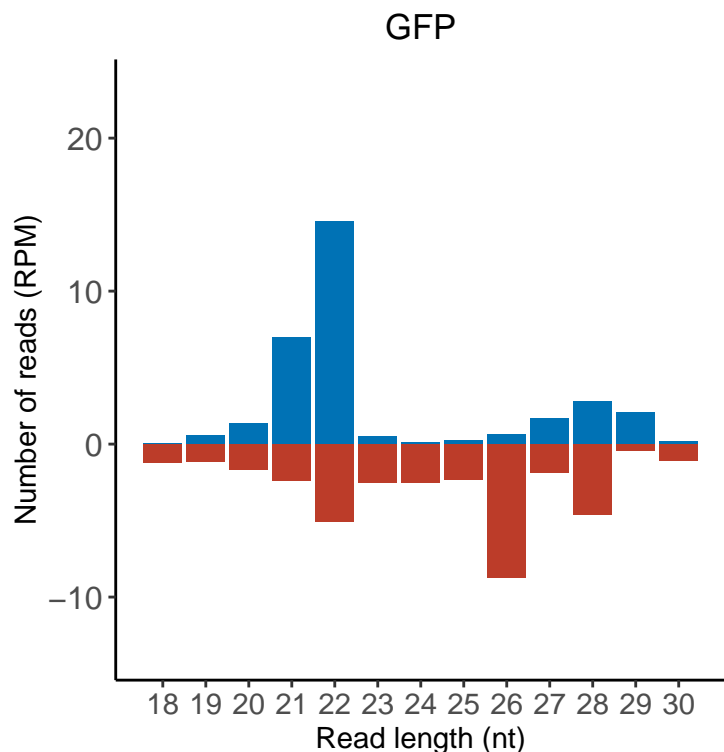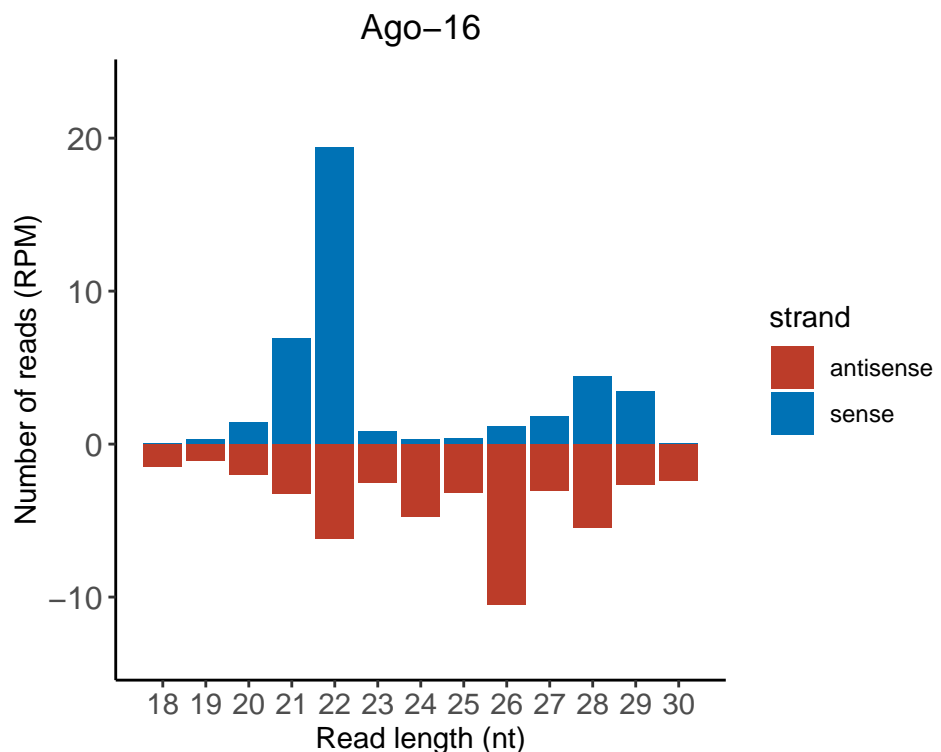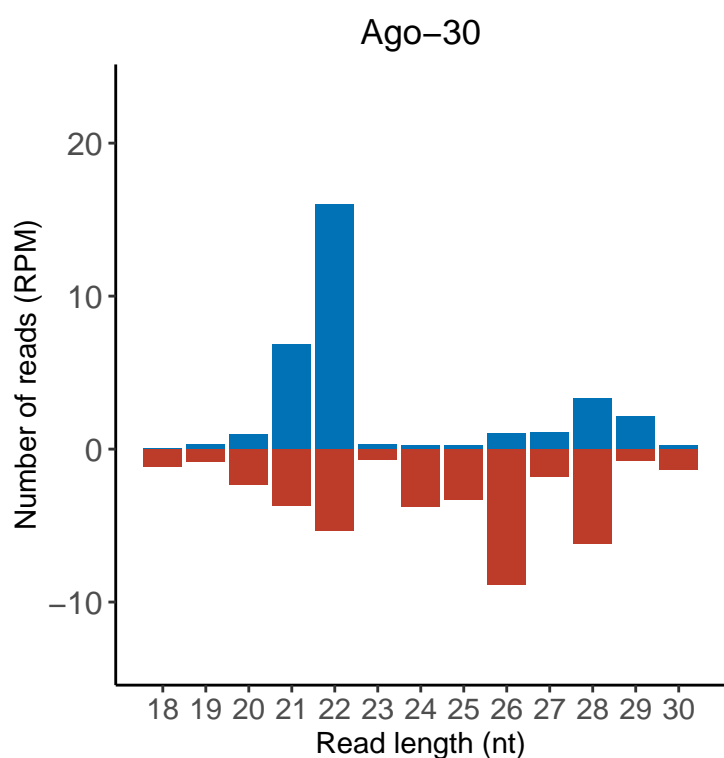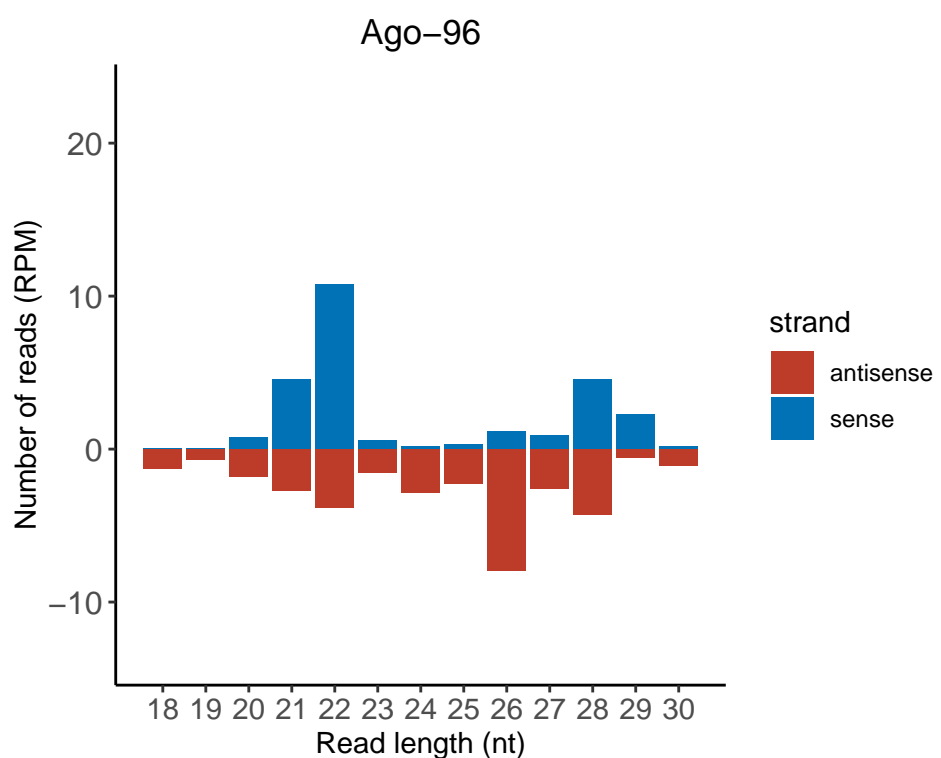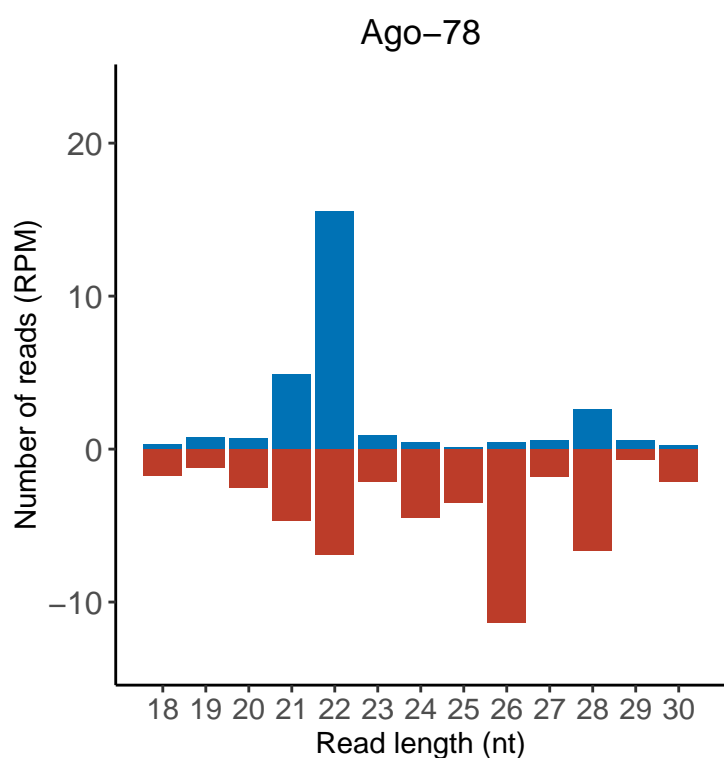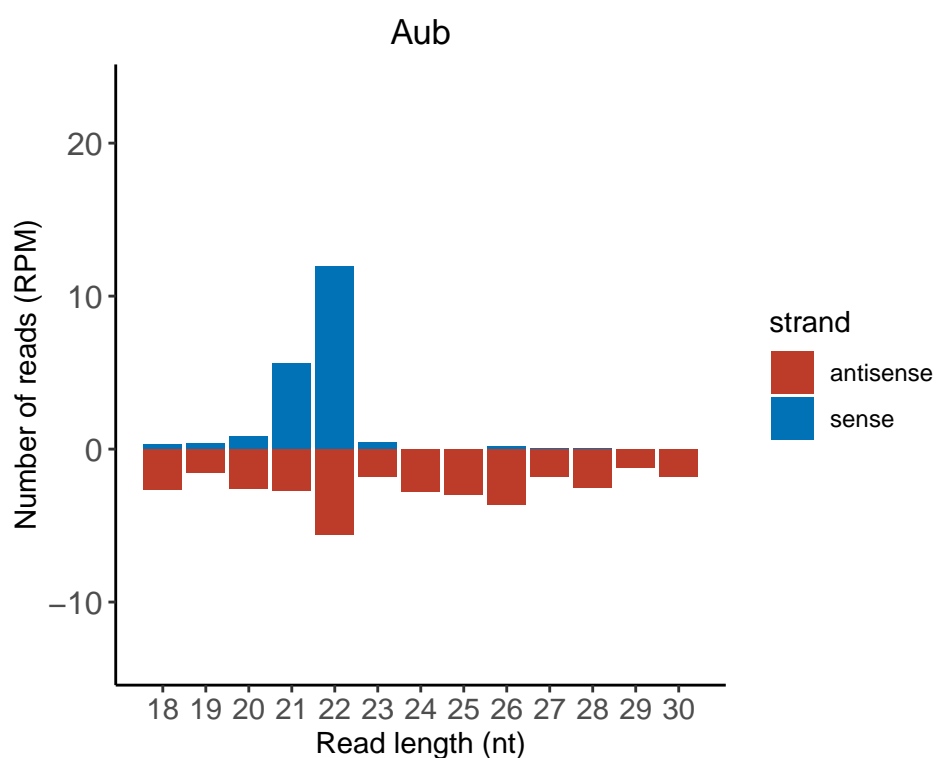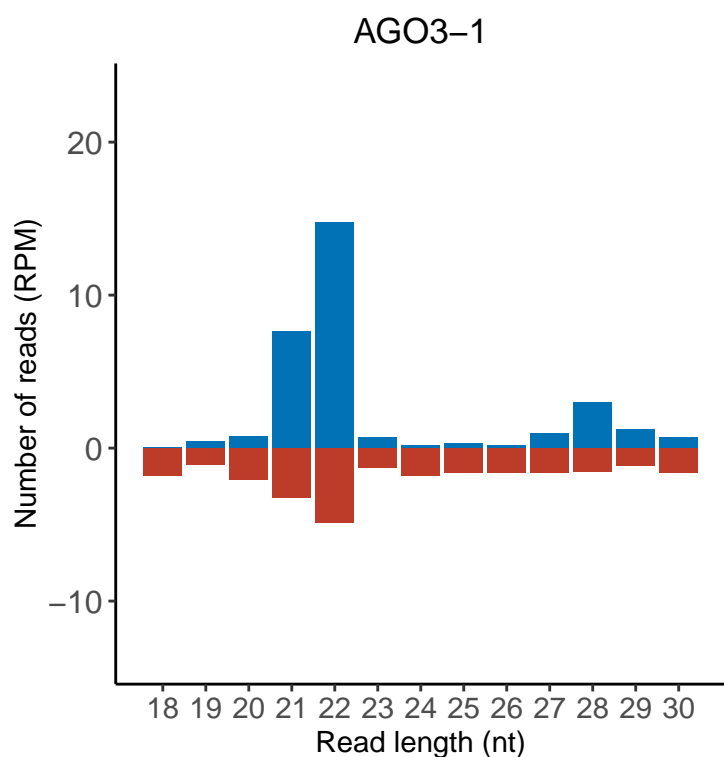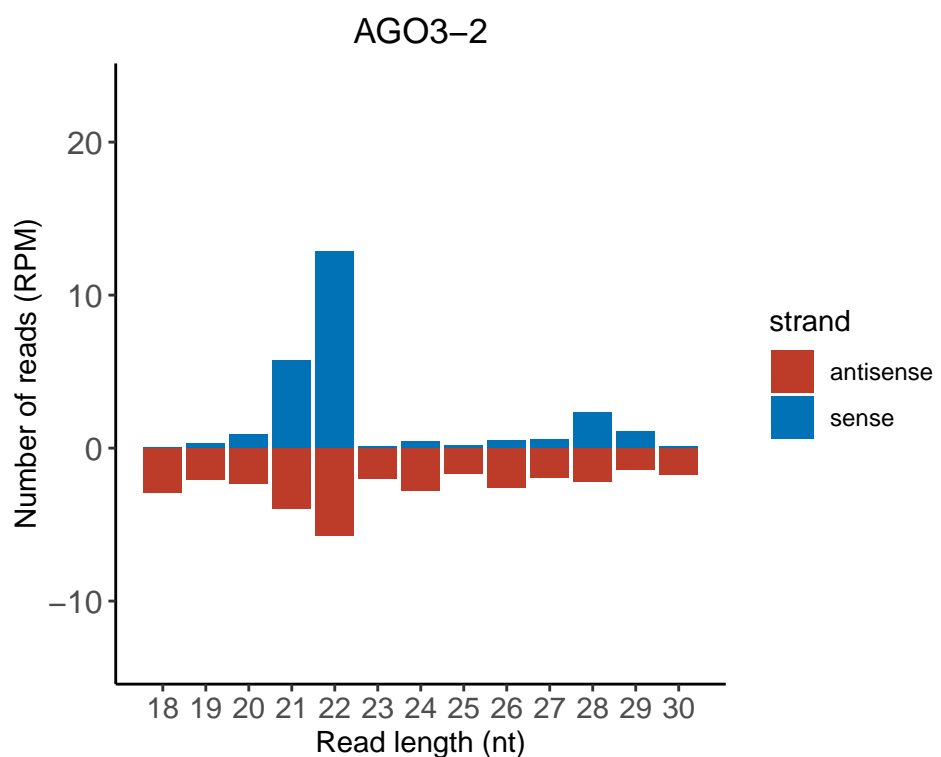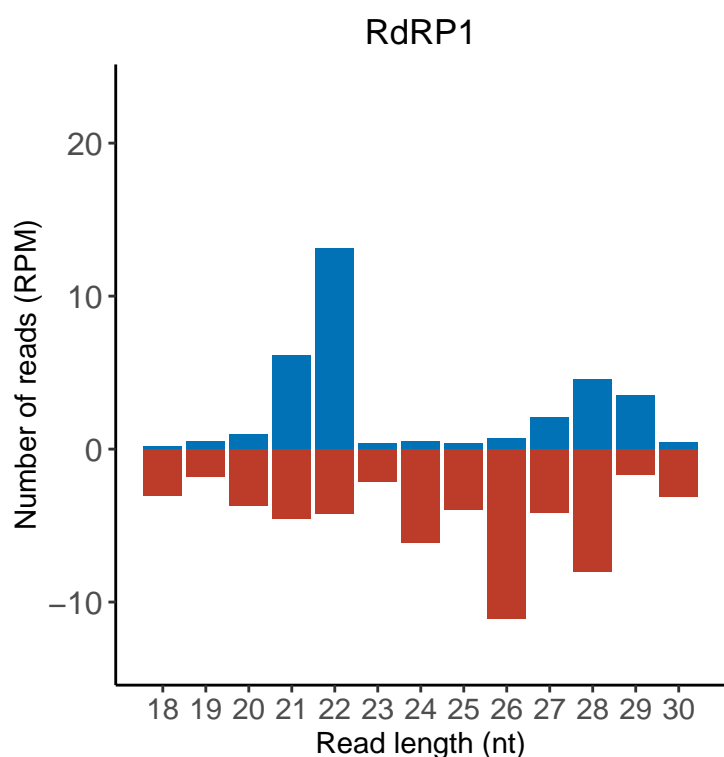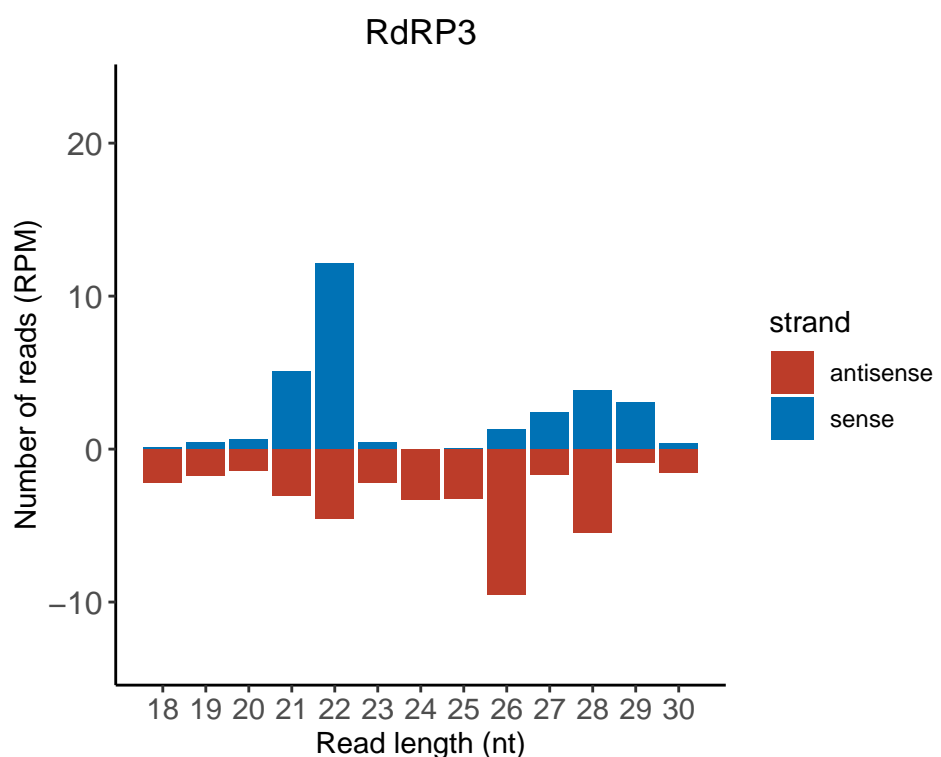

Supplement: S1 Data — On the Normalized and Relative levels pages, normalized read counts (RPM) and relative levels compared to the control (dsGFP) library were used. TEs with more than 50RPM and Coding Genes with more than 3.5RPM on average in KD libraries are included in this website. For viral sequences, reference sequences were generated by assembling sRNA sequences (See the Analysis of sRNAseq data section in Materials and methods.) and size distributions of sRNA reads mapped to each contig are shown. Reads were first grouped by their 5’ nucleotides, and reads of each length were counted. Full tables including TEs and Coding genes with fewer reads are in the “FullTable” folder. (ZIP) [file pone.0281195.s017.zip › SupplementaryData/Links/PDFs/Motif:rnd-6_family-902TE_lengthdistribution.pdf]

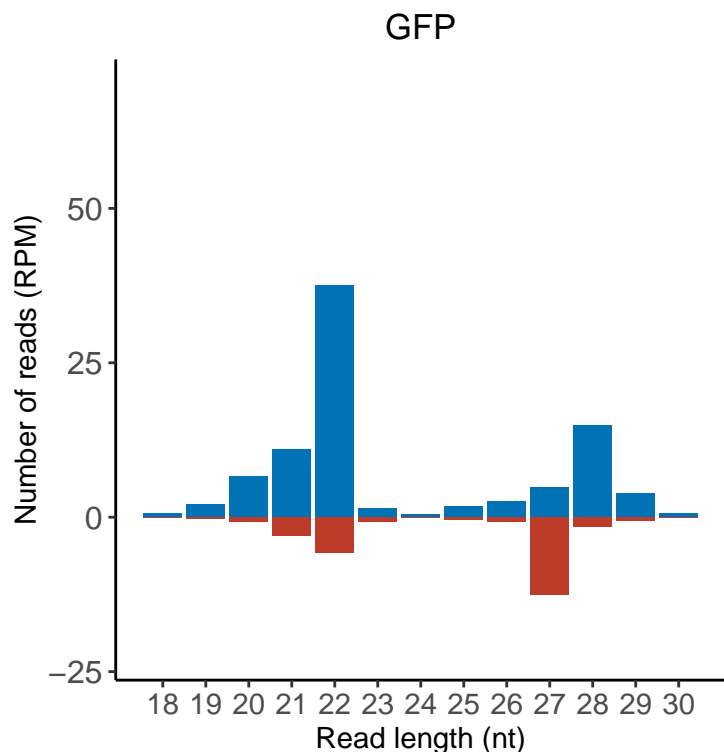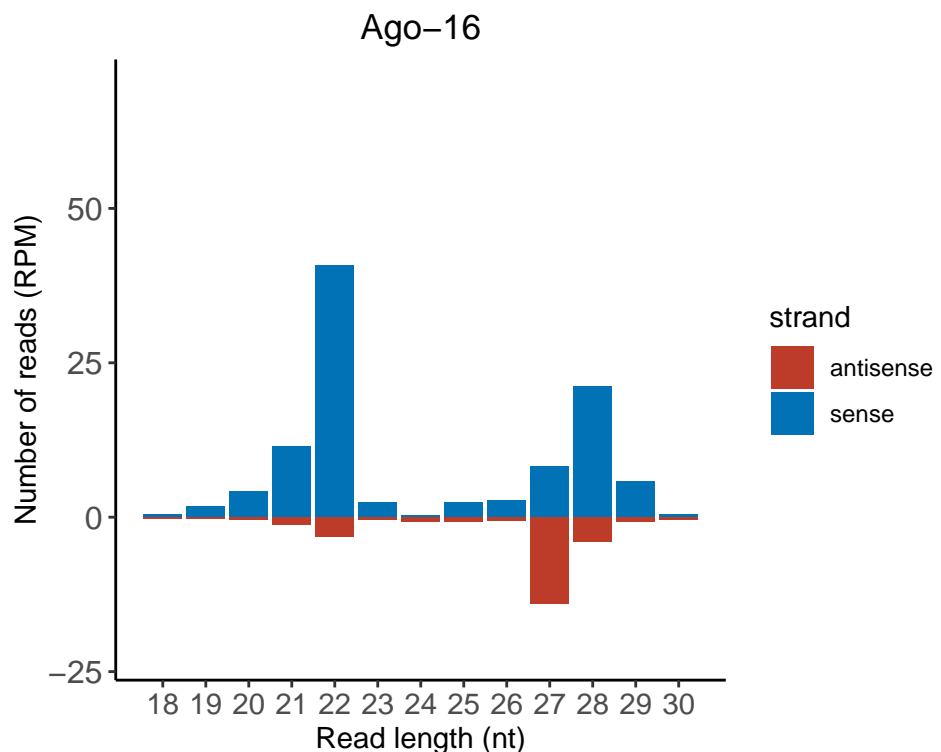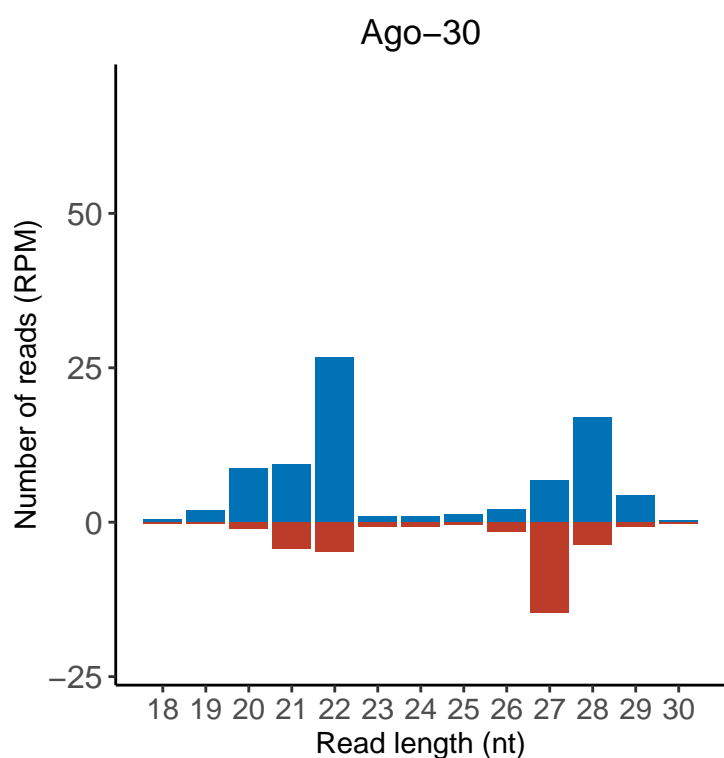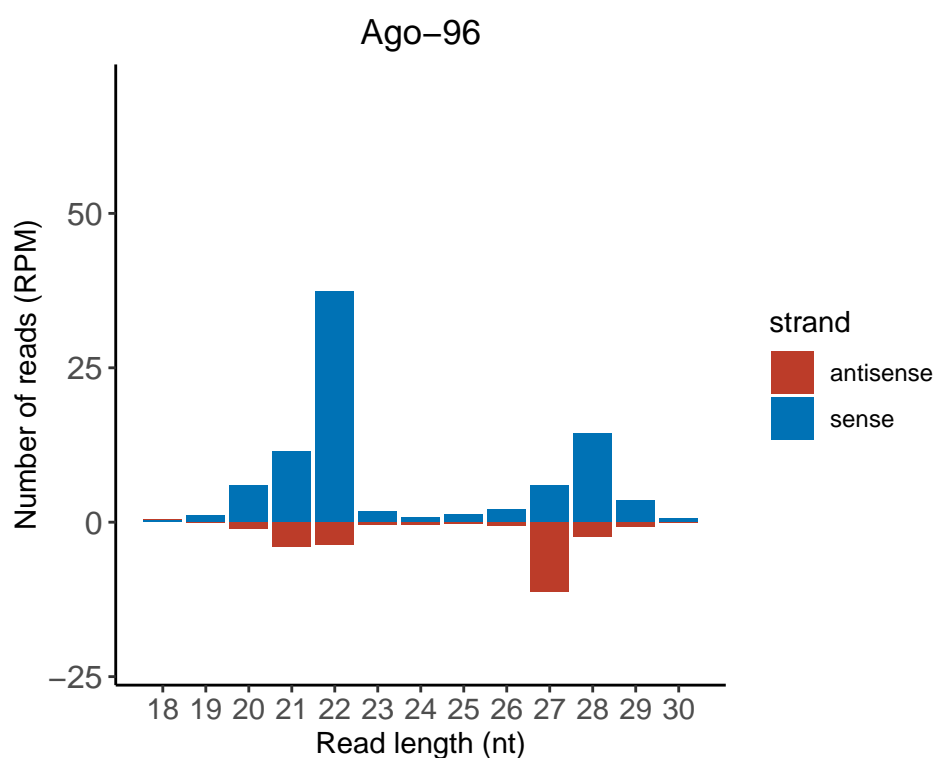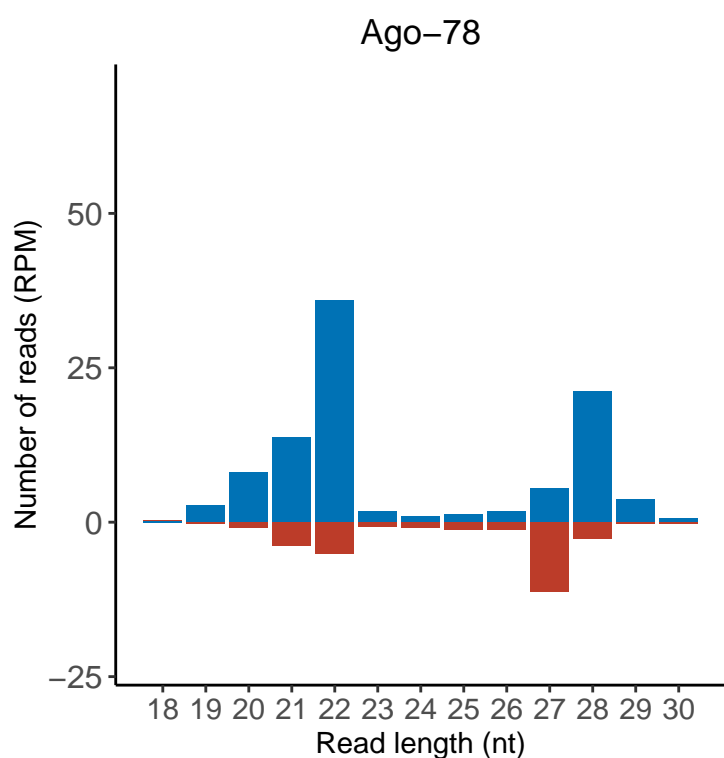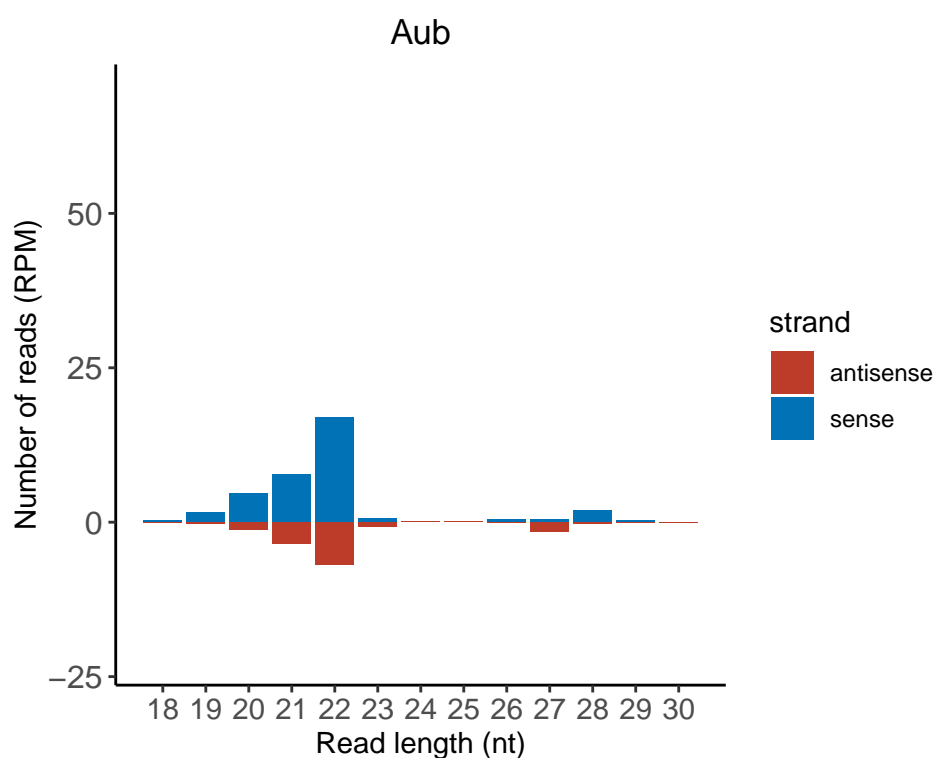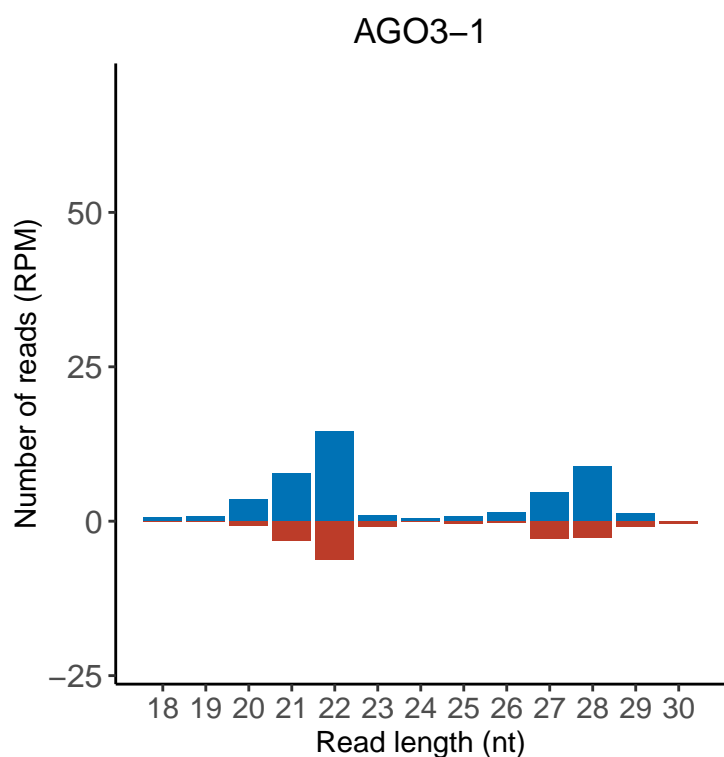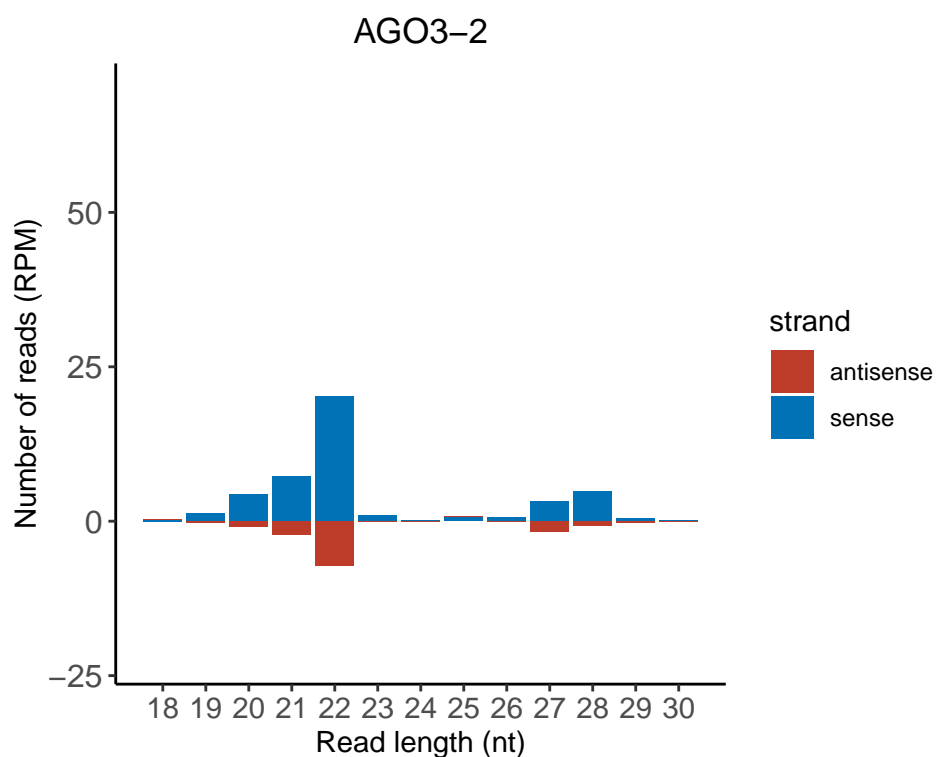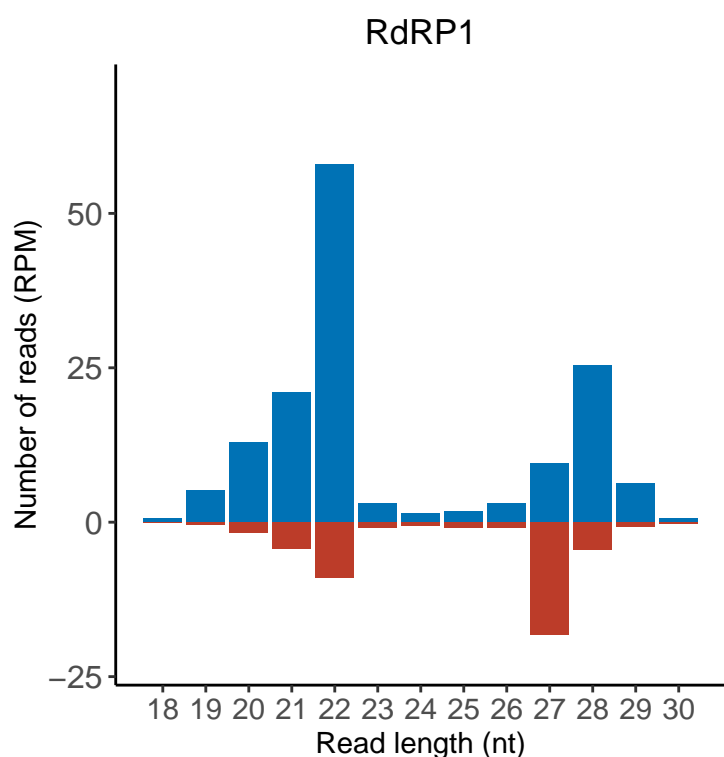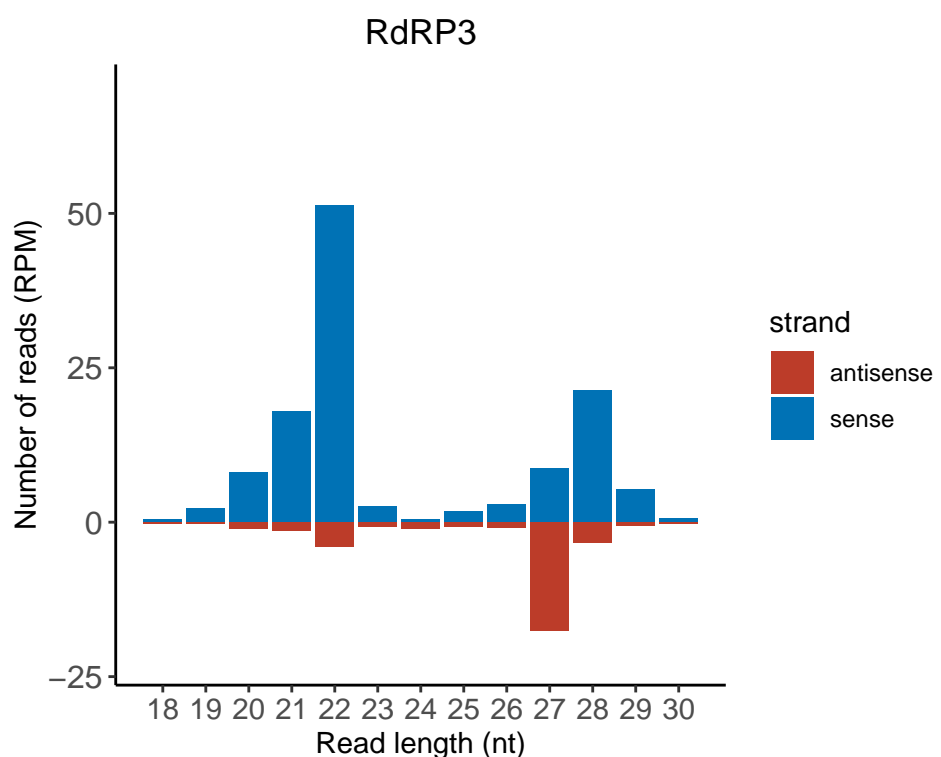

Supplement: S1 Data — On the Normalized and Relative levels pages, normalized read counts (RPM) and relative levels compared to the control (dsGFP) library were used. TEs with more than 50RPM and Coding Genes with more than 3.5RPM on average in KD libraries are included in this website. For viral sequences, reference sequences were generated by assembling sRNA sequences (See the Analysis of sRNAseq data section in Materials and methods.) and size distributions of sRNA reads mapped to each contig are shown. Reads were first grouped by their 5’ nucleotides, and reads of each length were counted. Full tables including TEs and Coding genes with fewer reads are in the “FullTable” folder. (ZIP) [file pone.0281195.s017.zip › SupplementaryData/Links/PDFs/Motif:rnd-5_family-2004TE_lengthdistribution.pdf]

GFP

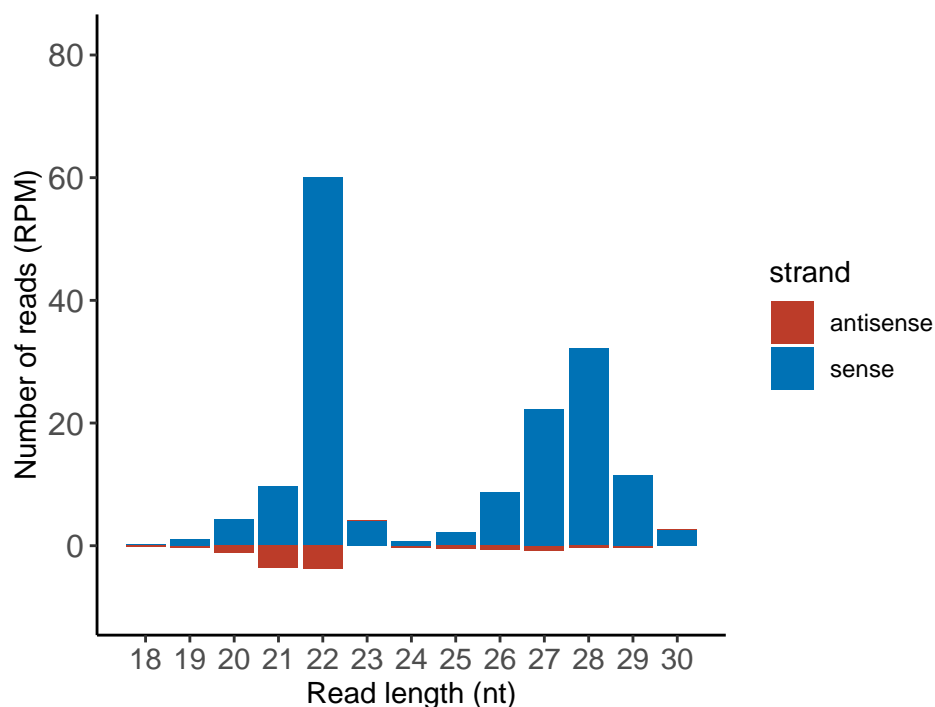

Ago-16

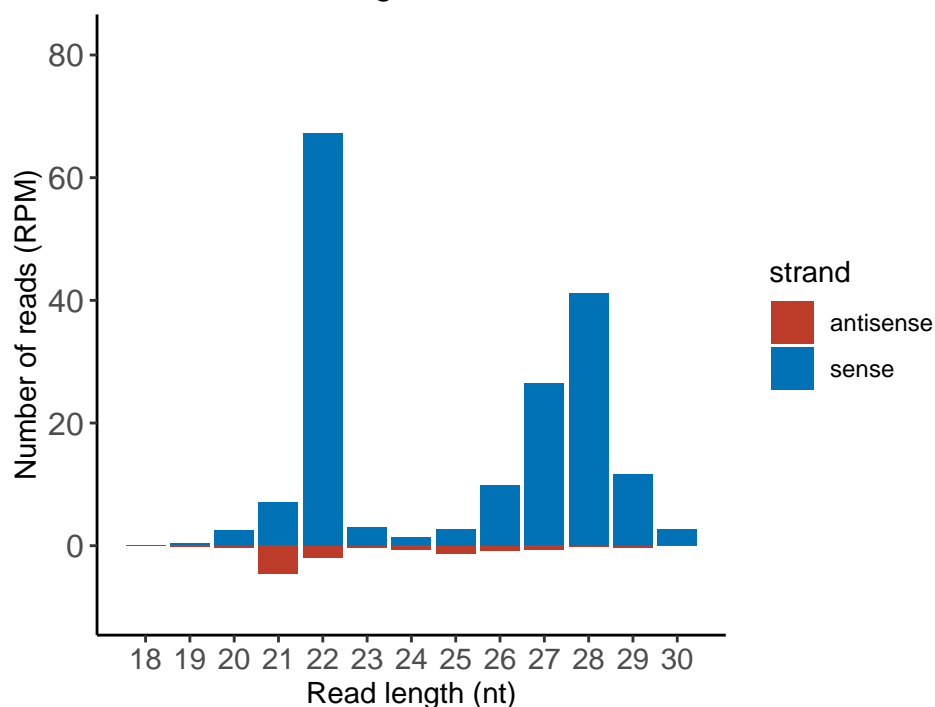

Ago-30

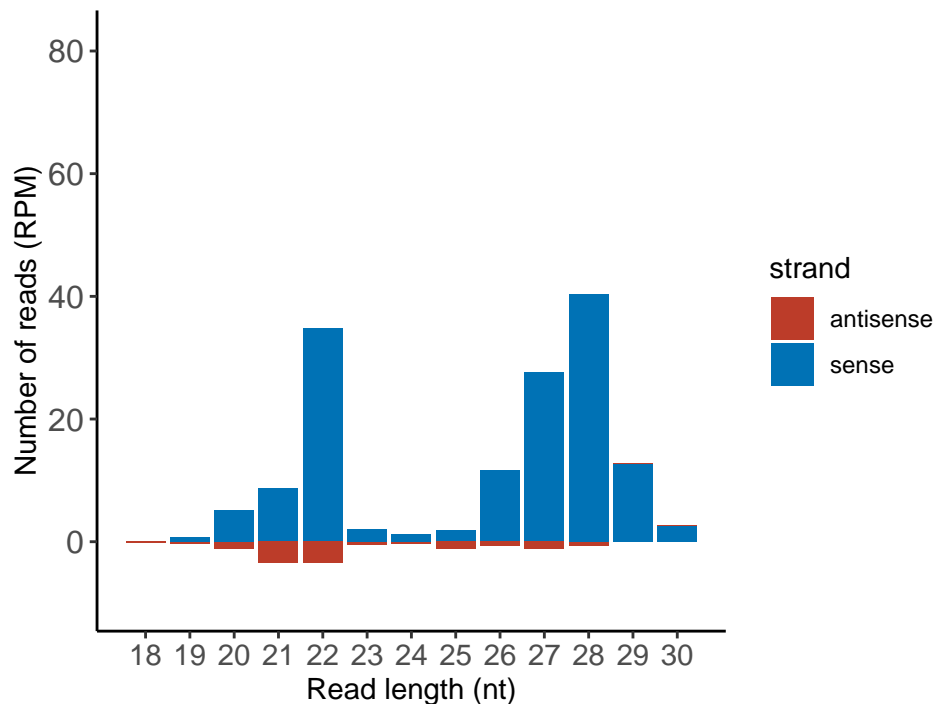

Ago-96

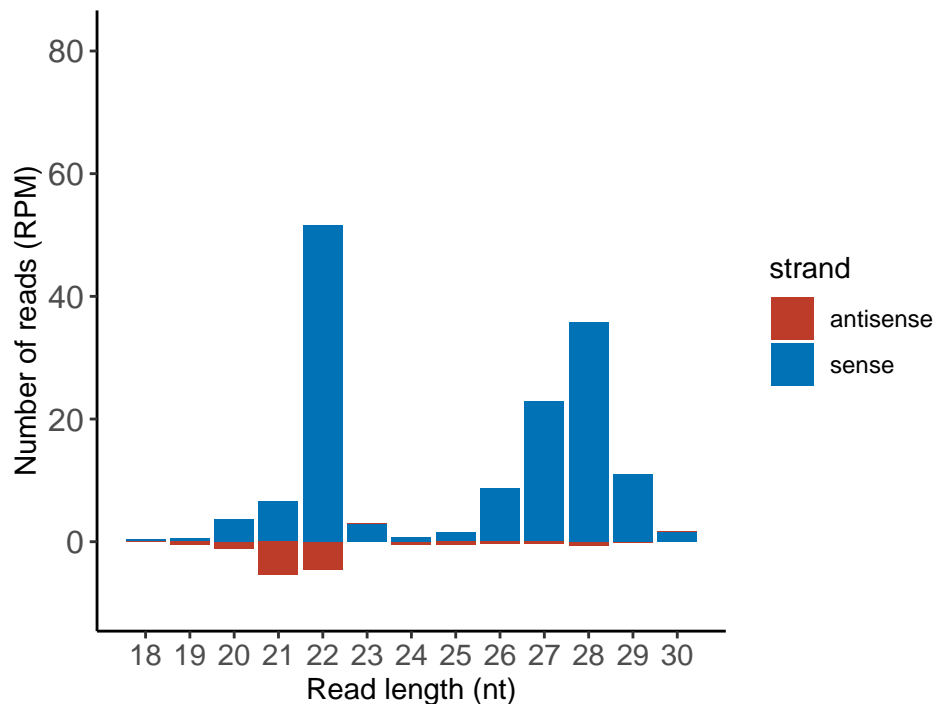

Ago-78

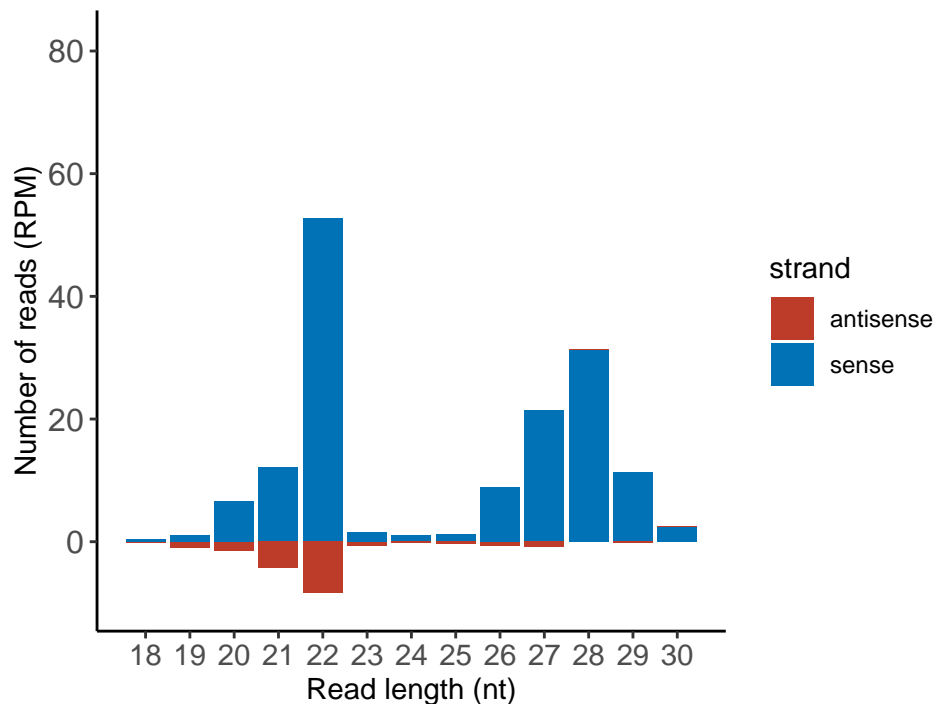

Aub

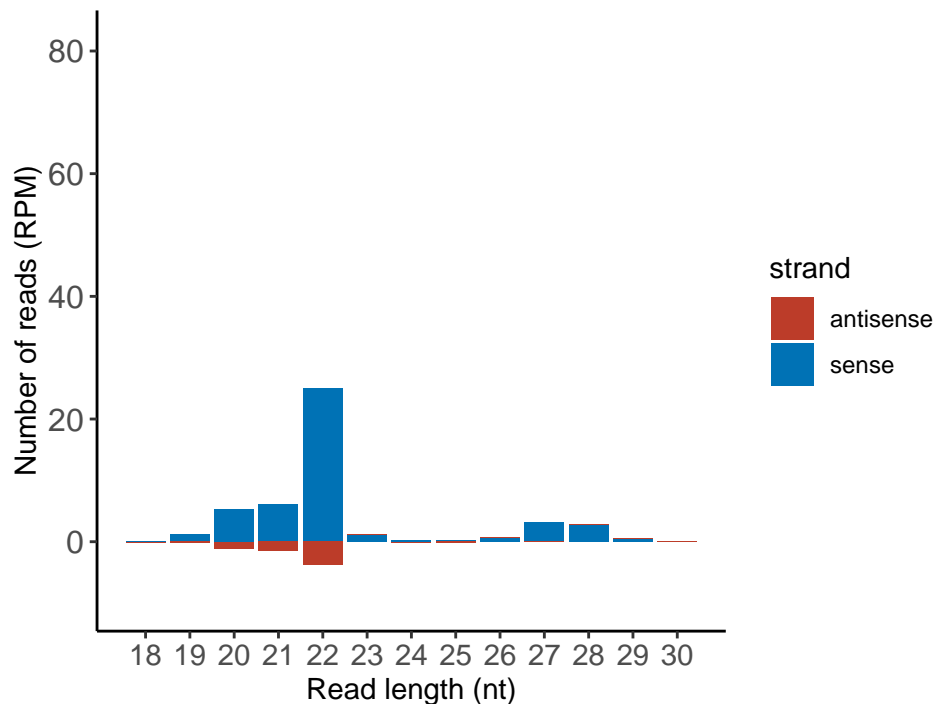

AGO3-1

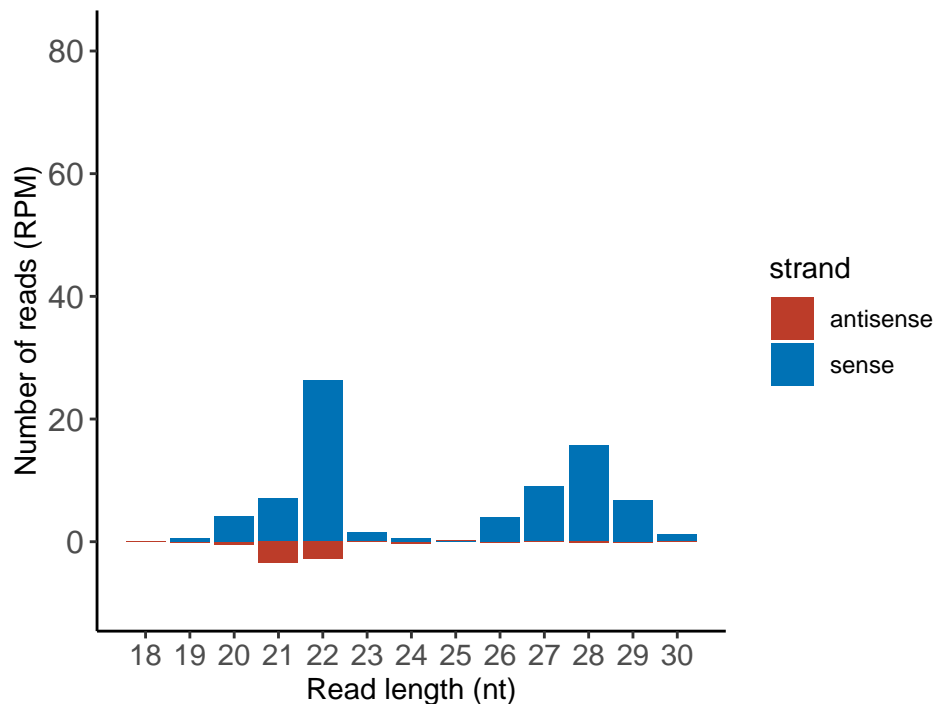

AGO3-2

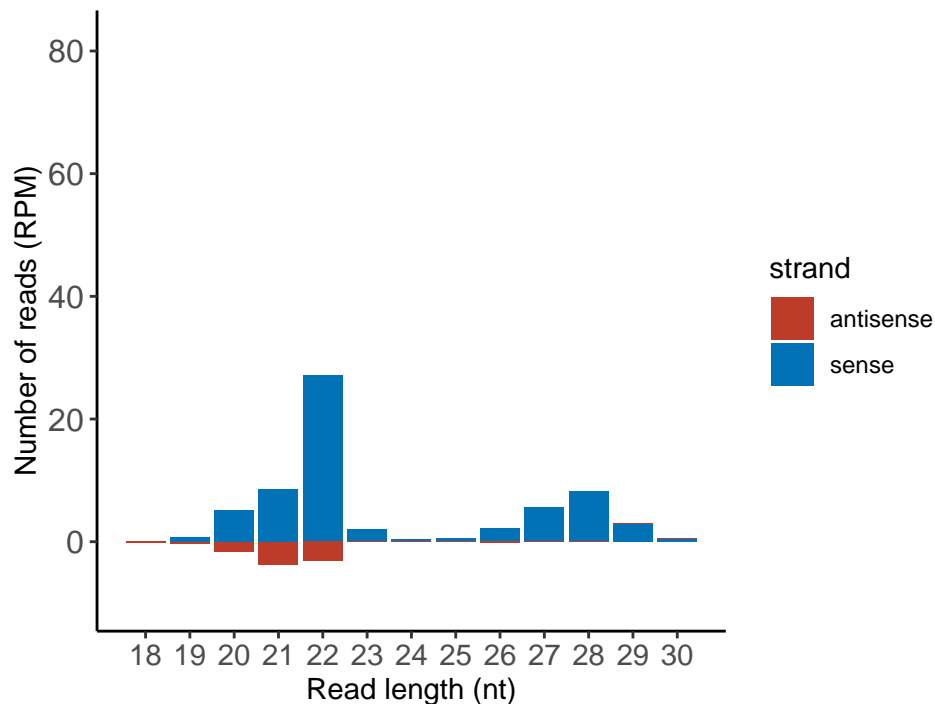

RdRP1

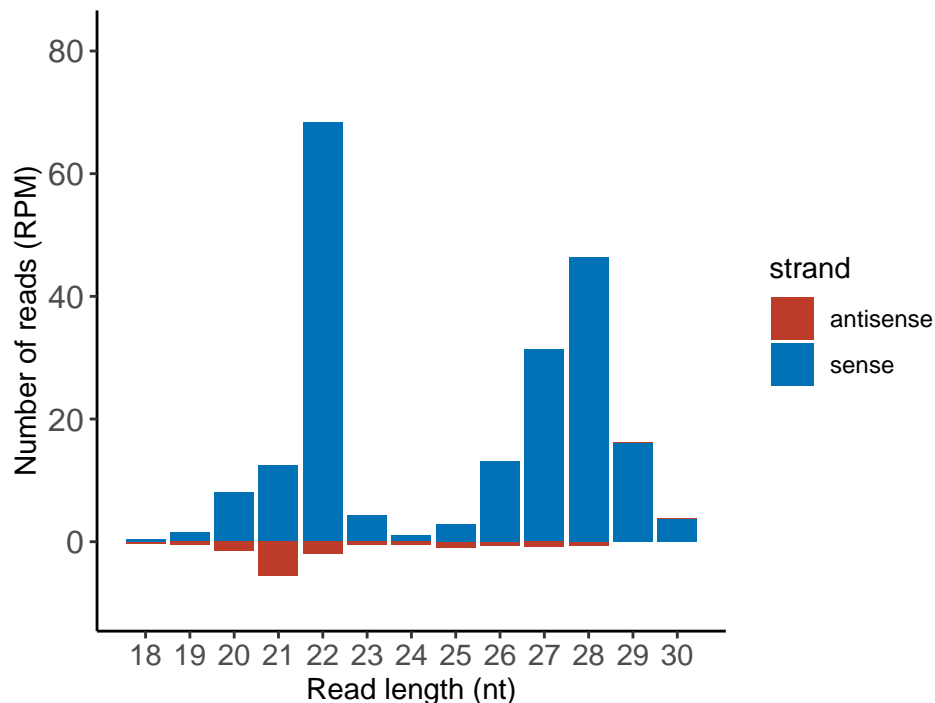

RdRP3

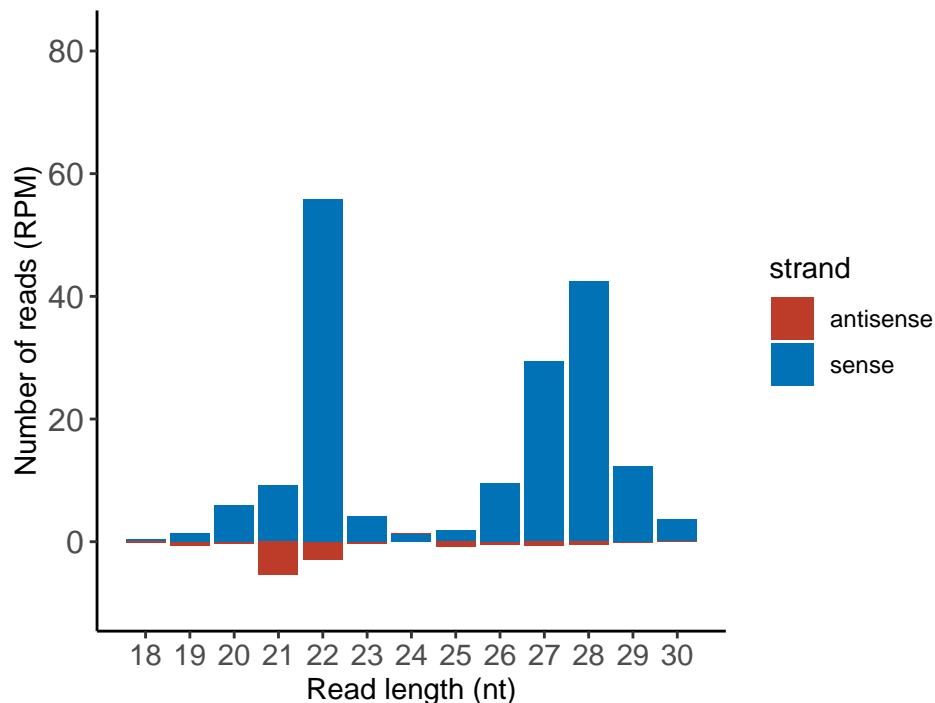

Supplement: S1 Data — On the Normalized and Relative levels pages, normalized read counts (RPM) and relative levels compared to the control (dsGFP) library were used. TEs with more than 50RPM and Coding Genes with more than 3.5RPM on average in KD libraries are included in this website. For viral sequences, reference sequences were generated by assembling sRNA sequences (See the Analysis of sRNAseq data section in Materials and methods.) and size distributions of sRNA reads mapped to each contig are shown. Reads were first grouped by their 5’ nucleotides, and reads of each length were counted. Full tables including TEs and Coding genes with fewer reads are in the “FullTable” folder. (ZIP) [file pone.0281195.s017.zip › SupplementaryData/Links/PDFs/Motif:rnd-6_family-4218TE_lengthdistribution.pdf]

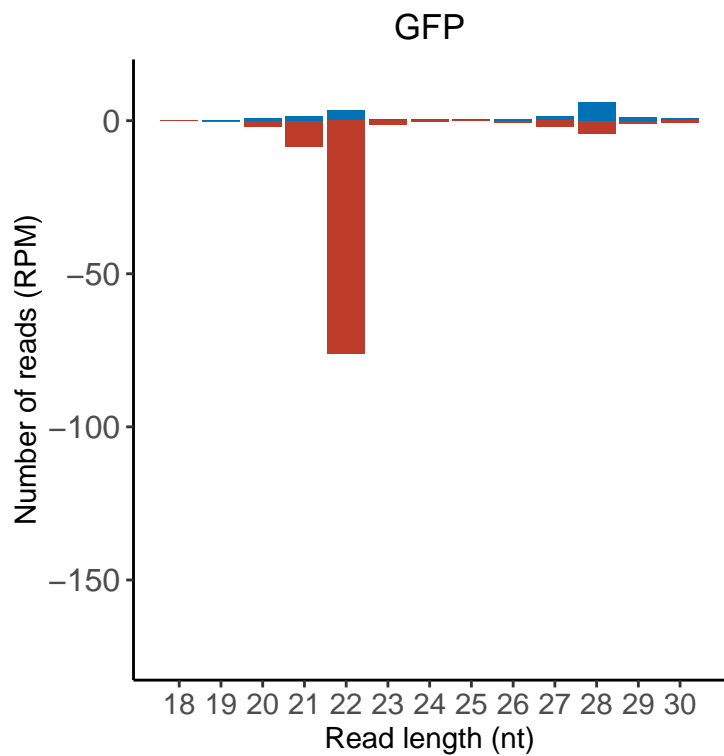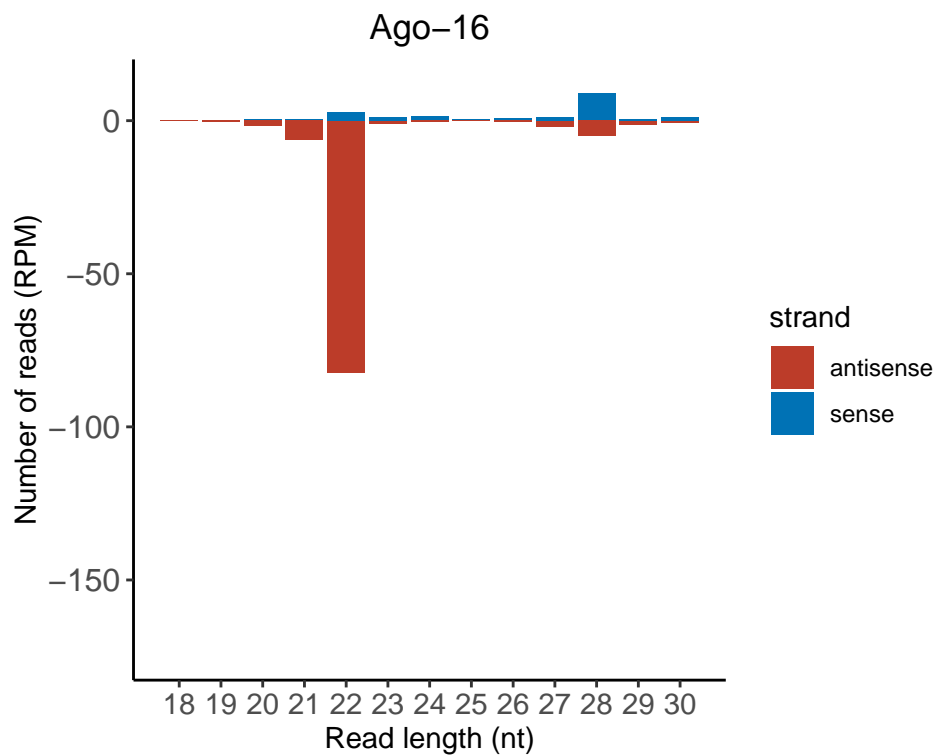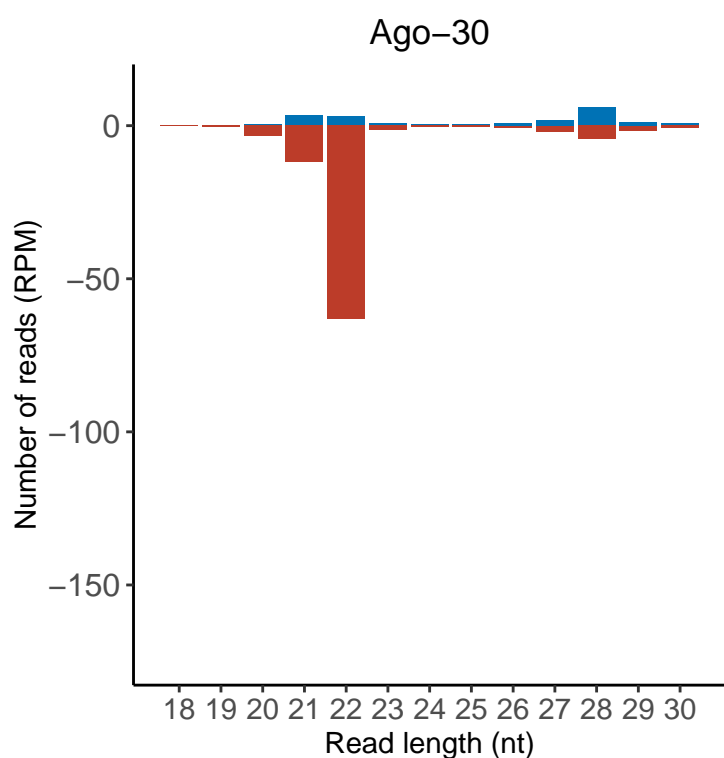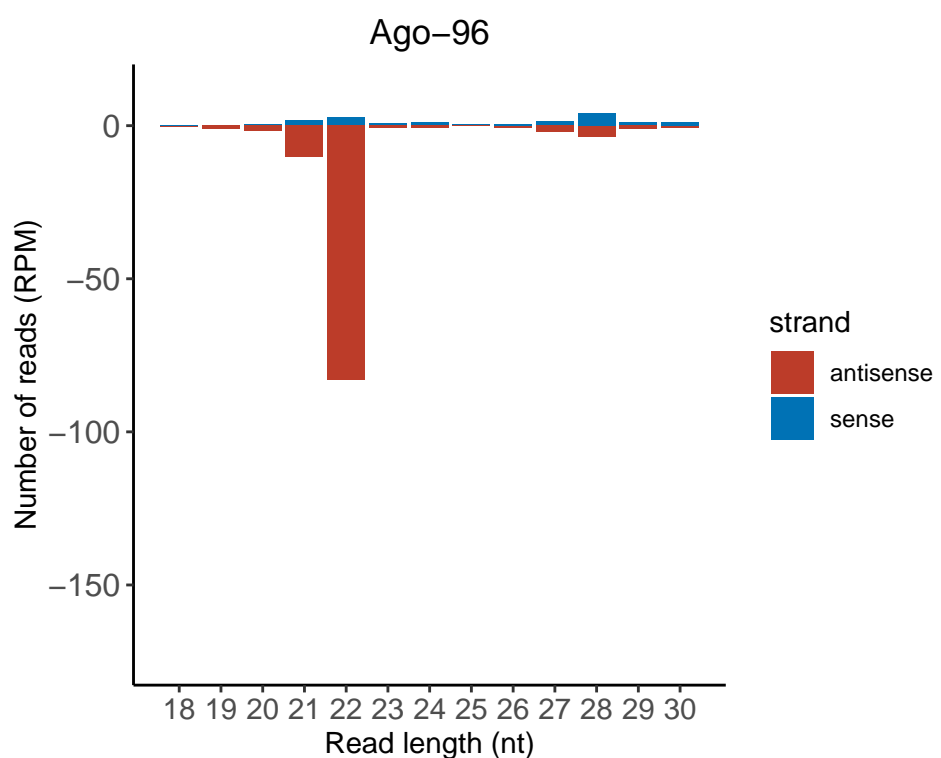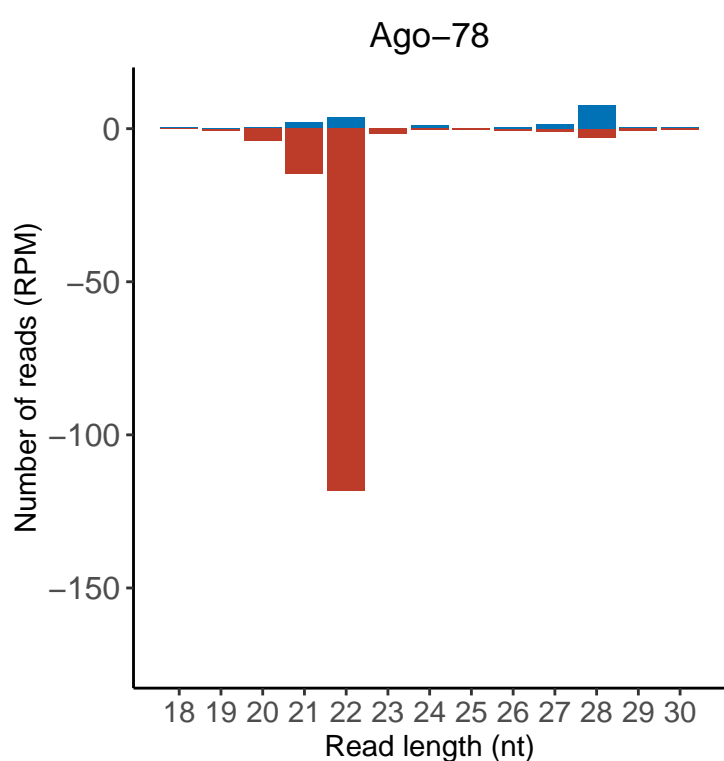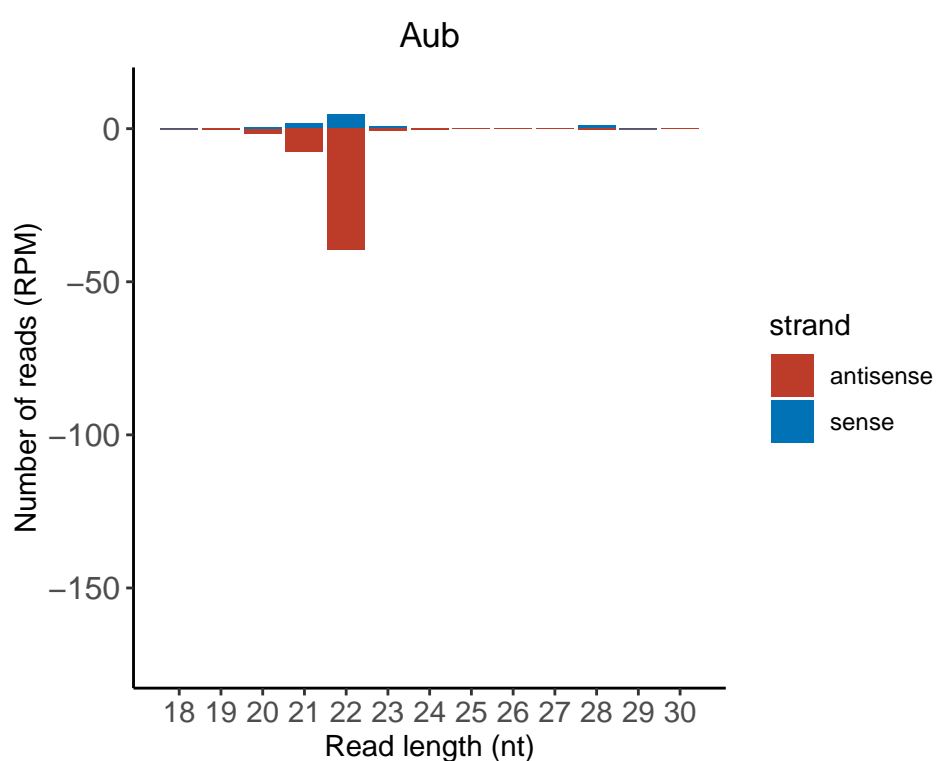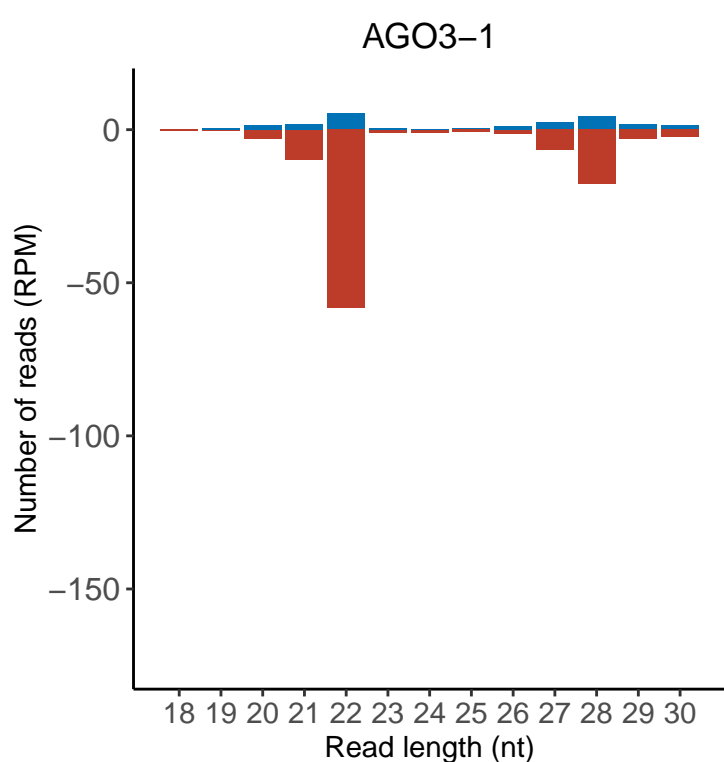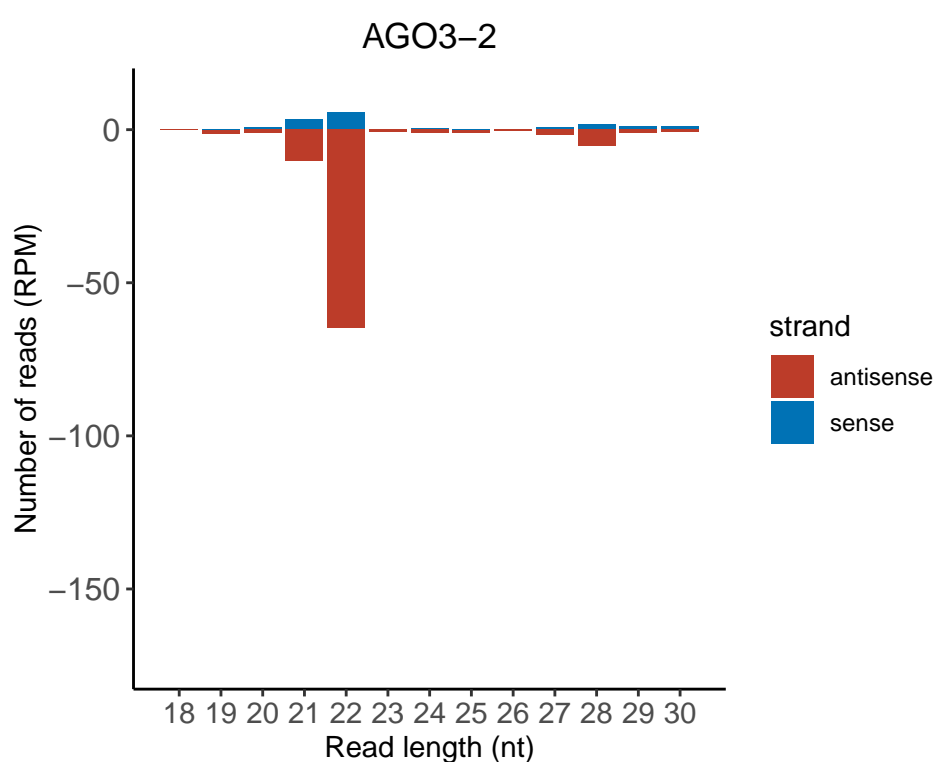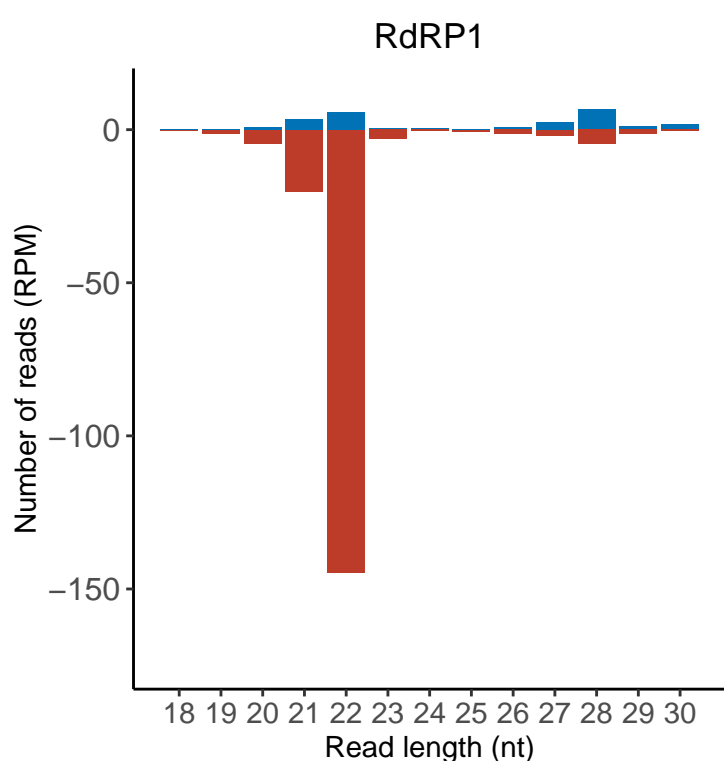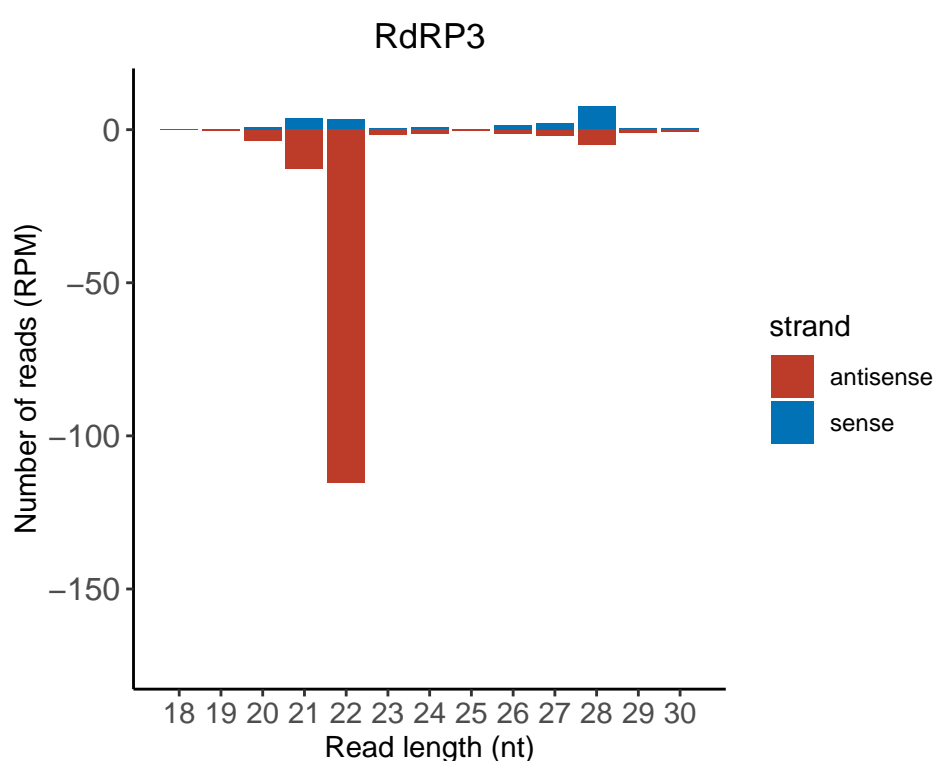

Supplement: S1 Data — On the Normalized and Relative levels pages, normalized read counts (RPM) and relative levels compared to the control (dsGFP) library were used. TEs with more than 50RPM and Coding Genes with more than 3.5RPM on average in KD libraries are included in this website. For viral sequences, reference sequences were generated by assembling sRNA sequences (See the Analysis of sRNAseq data section in Materials and methods.) and size distributions of sRNA reads mapped to each contig are shown. Reads were first grouped by their 5’ nucleotides, and reads of each length were counted. Full tables including TEs and Coding genes with fewer reads are in the “FullTable” folder. (ZIP) [file pone.0281195.s017.zip › SupplementaryData/Links/PDFs/Motif:rnd-6_family-2943TE_lengthdistribution.pdf]

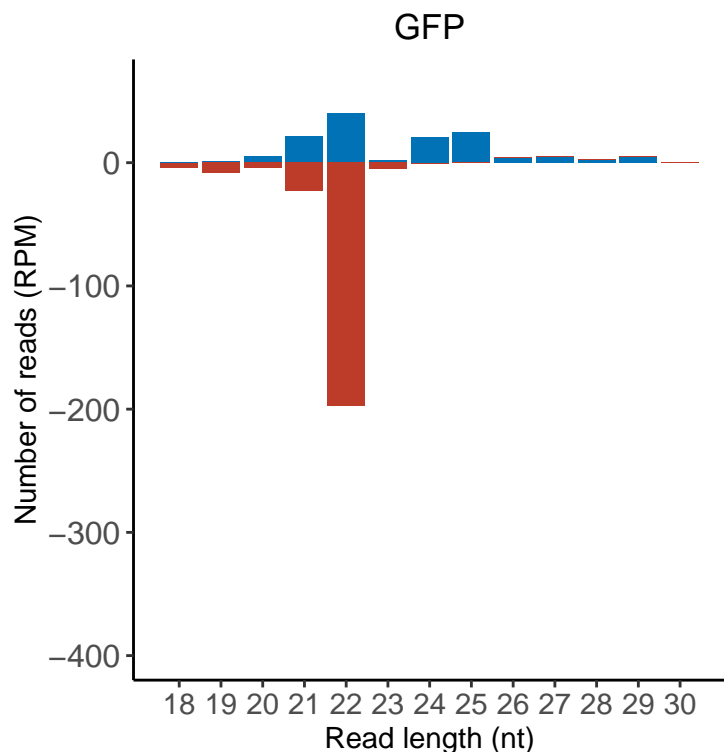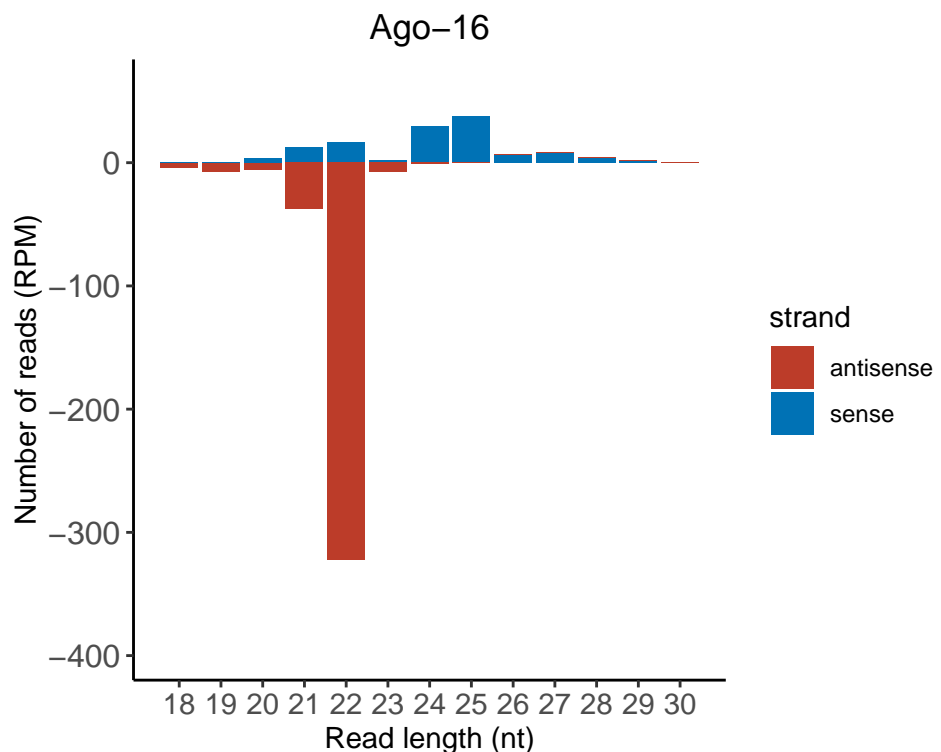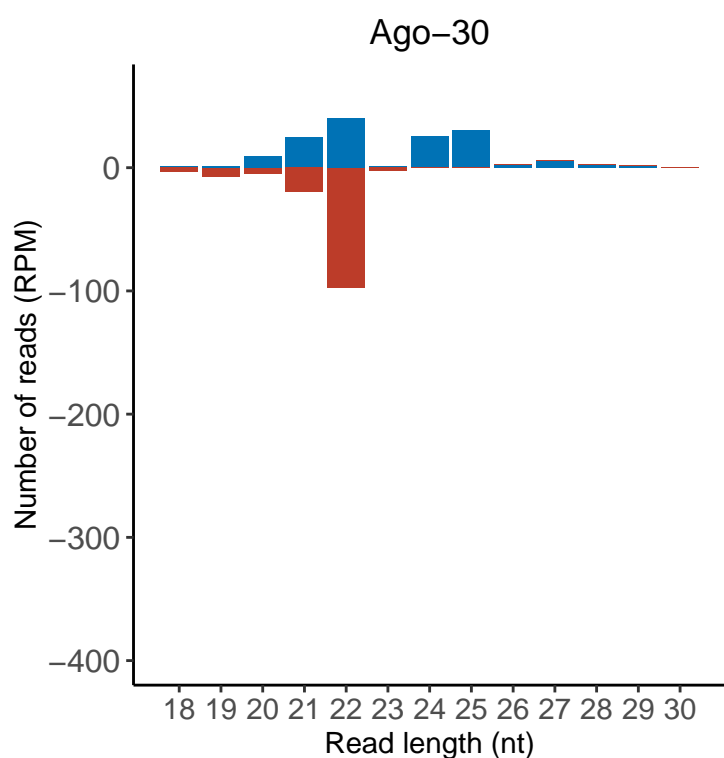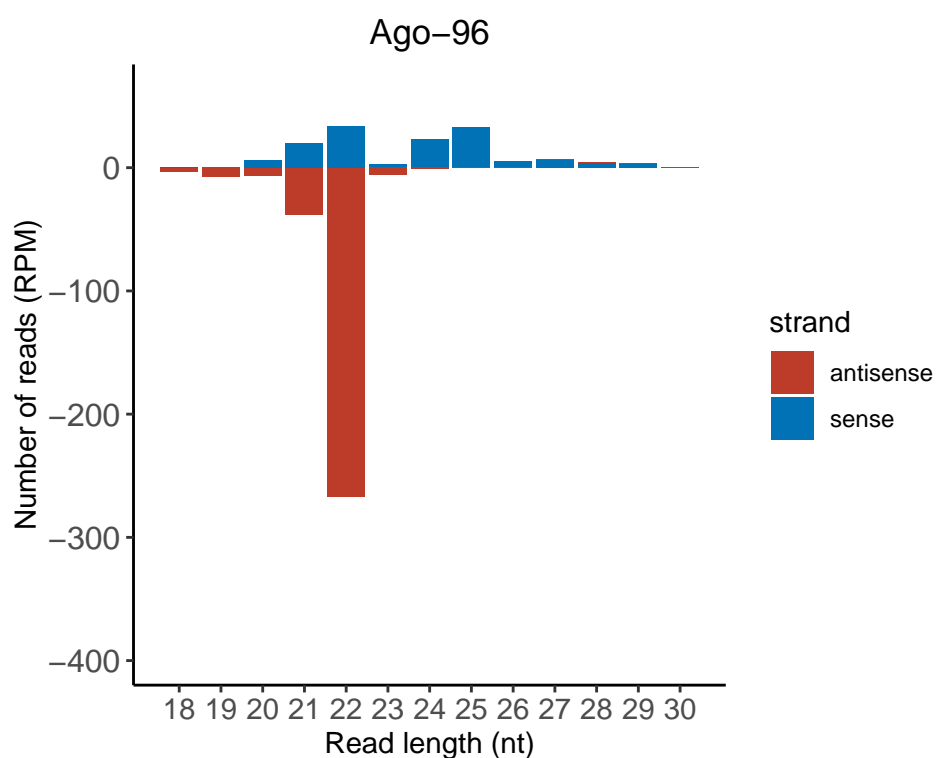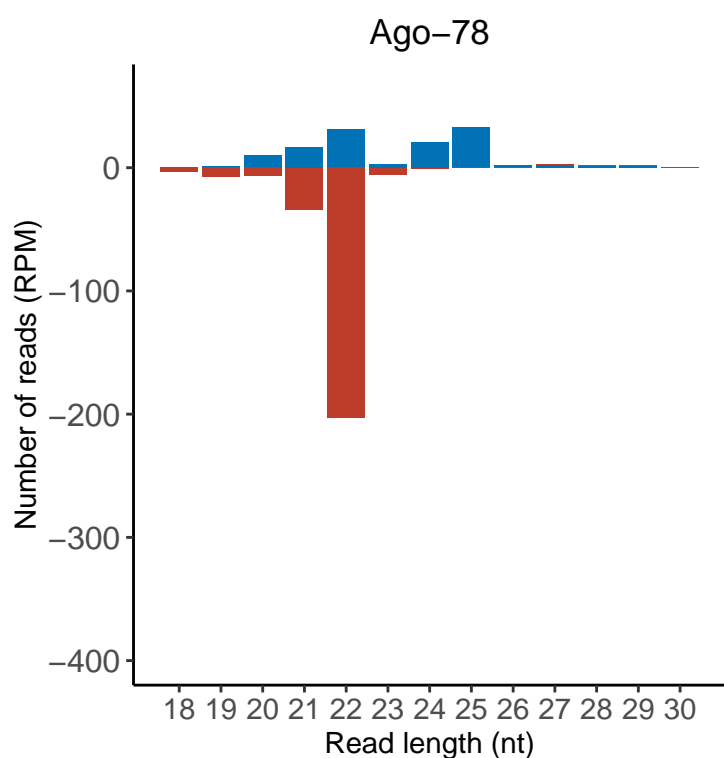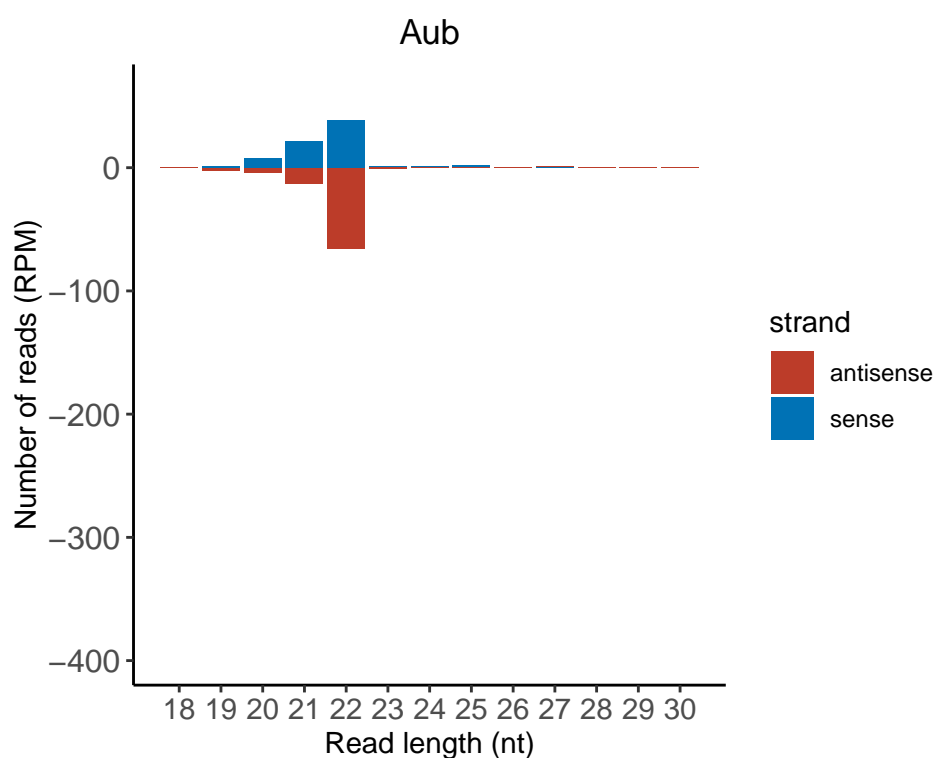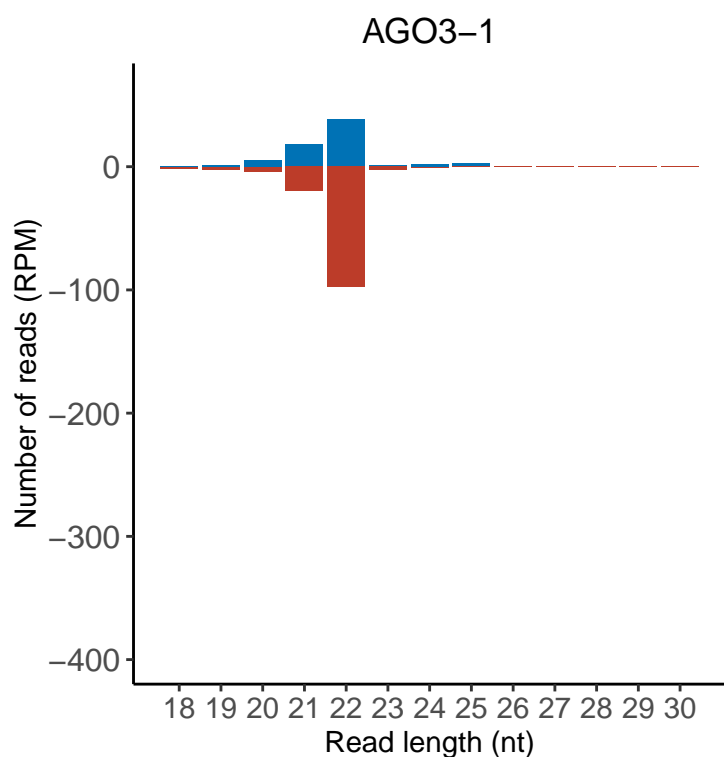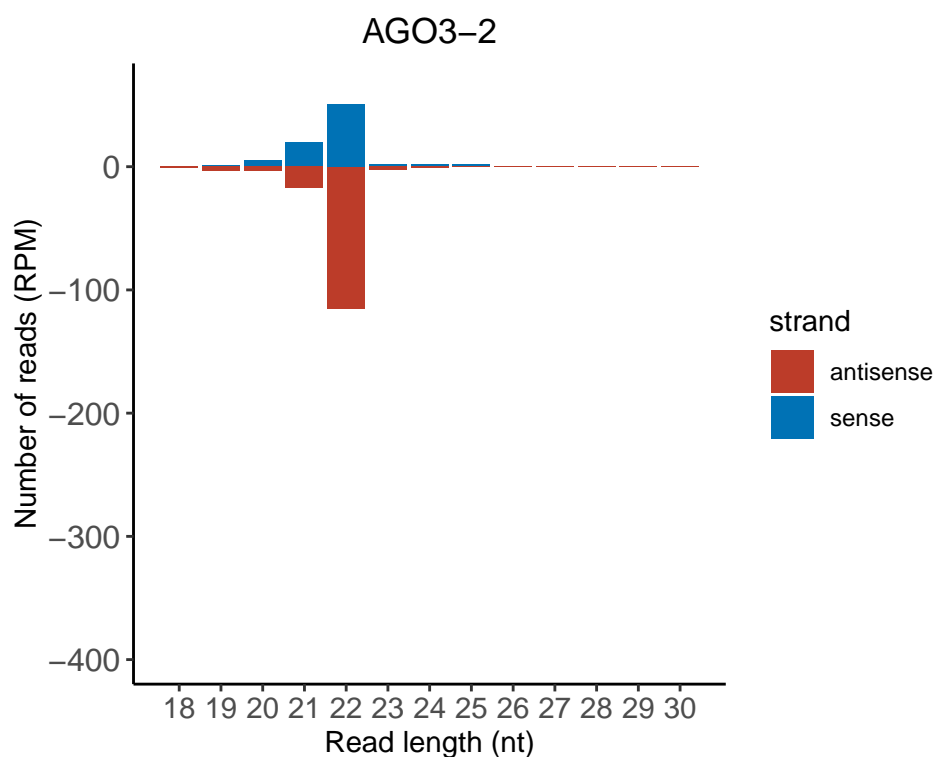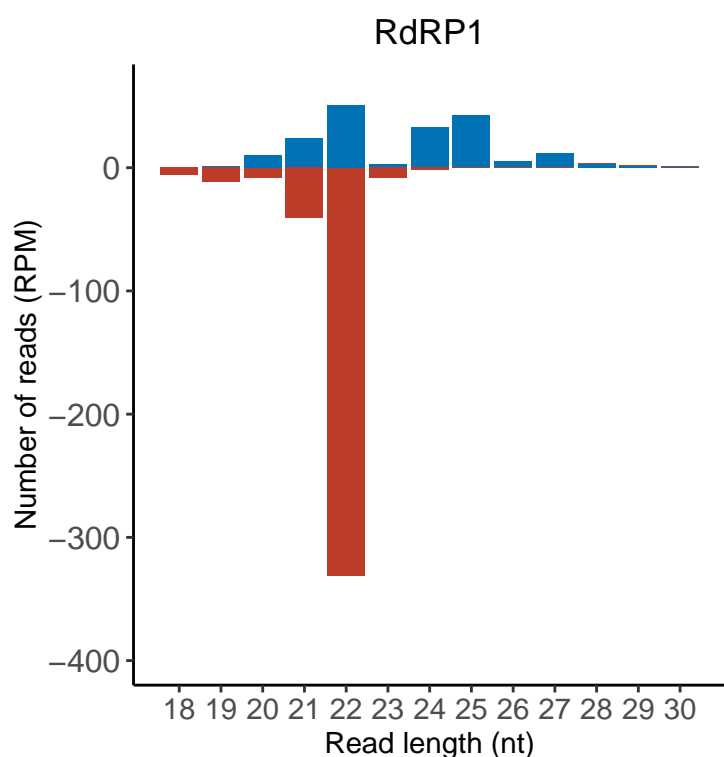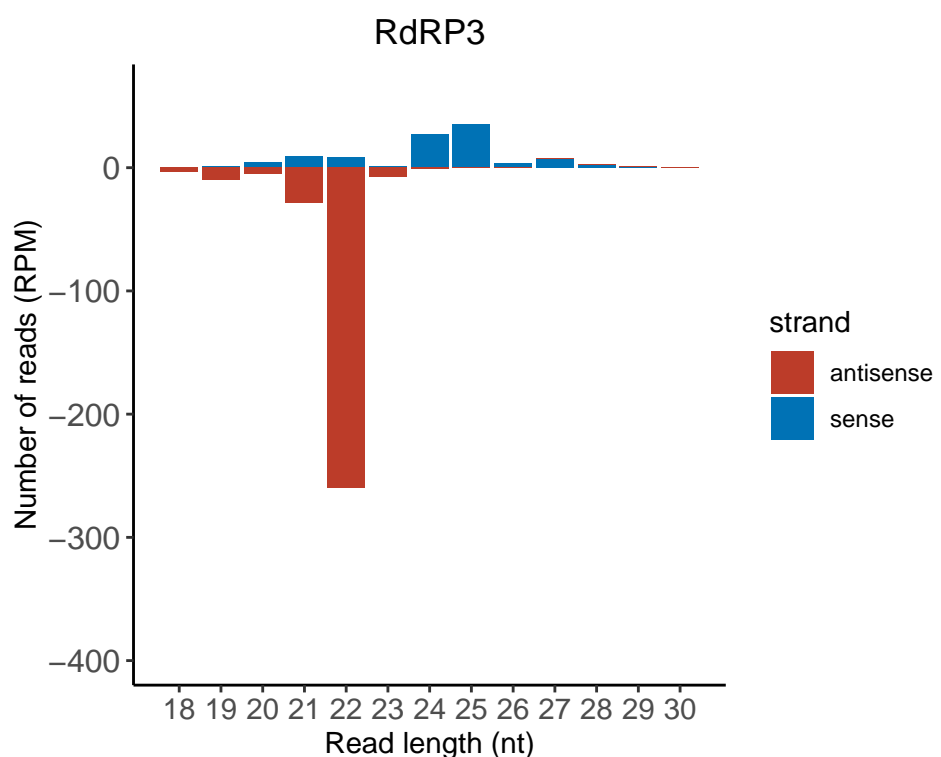

Supplement: S1 Data — On the Normalized and Relative levels pages, normalized read counts (RPM) and relative levels compared to the control (dsGFP) library were used. TEs with more than 50RPM and Coding Genes with more than 3.5RPM on average in KD libraries are included in this website. For viral sequences, reference sequences were generated by assembling sRNA sequences (See the Analysis of sRNAseq data section in Materials and methods.) and size distributions of sRNA reads mapped to each contig are shown. Reads were first grouped by their 5’ nucleotides, and reads of each length were counted. Full tables including TEs and Coding genes with fewer reads are in the “FullTable” folder. (ZIP) [file pone.0281195.s017.zip › SupplementaryData/Links/PDFs/ISCI005507.RACDS_lengthdistribution.pdf]

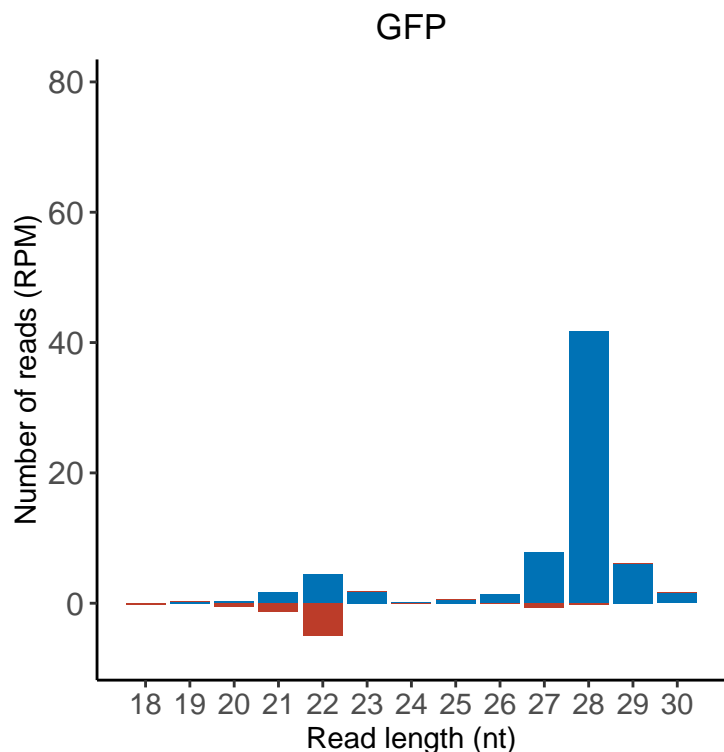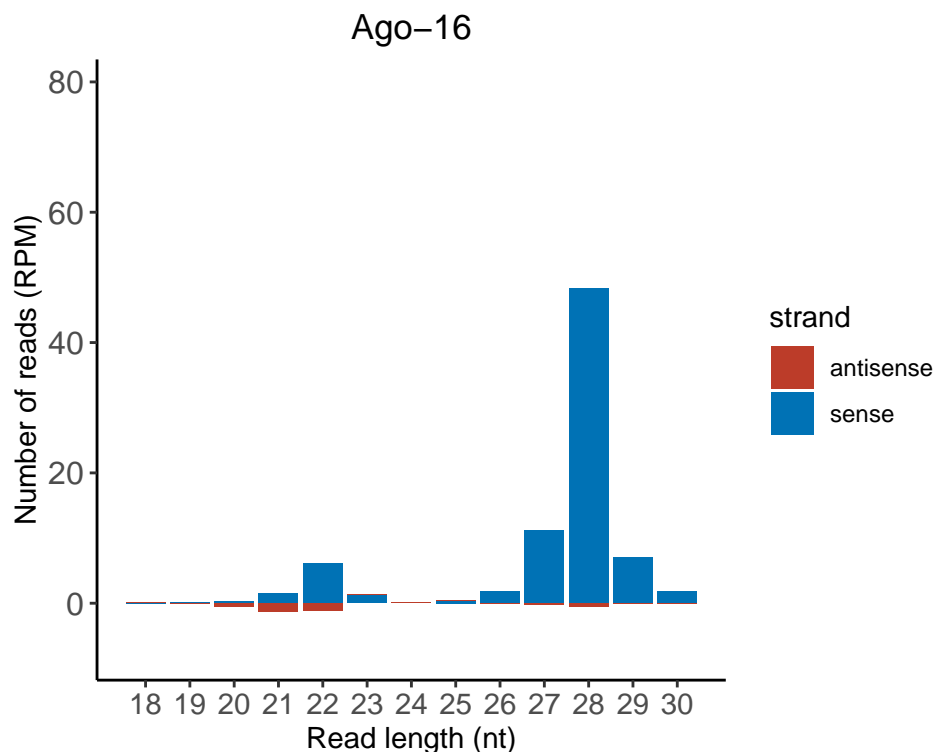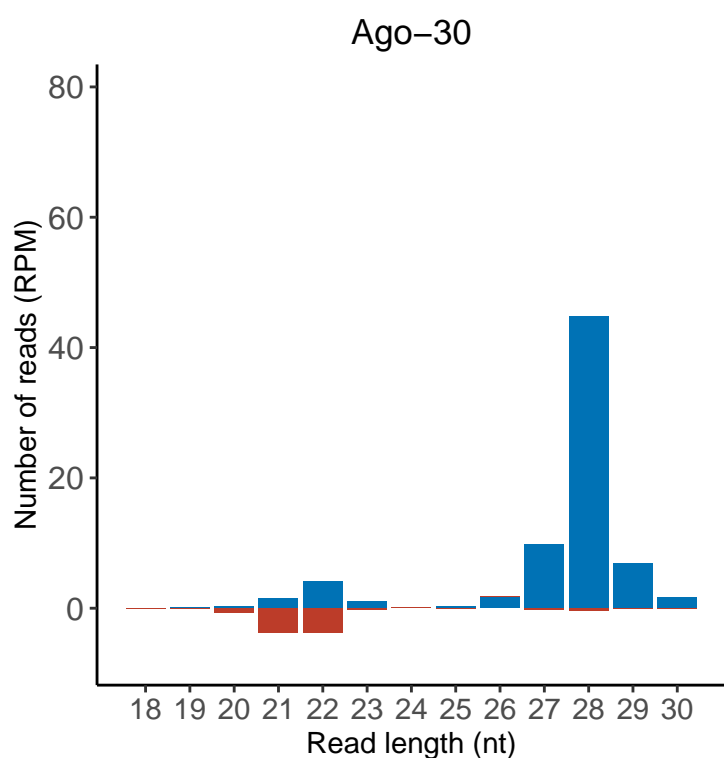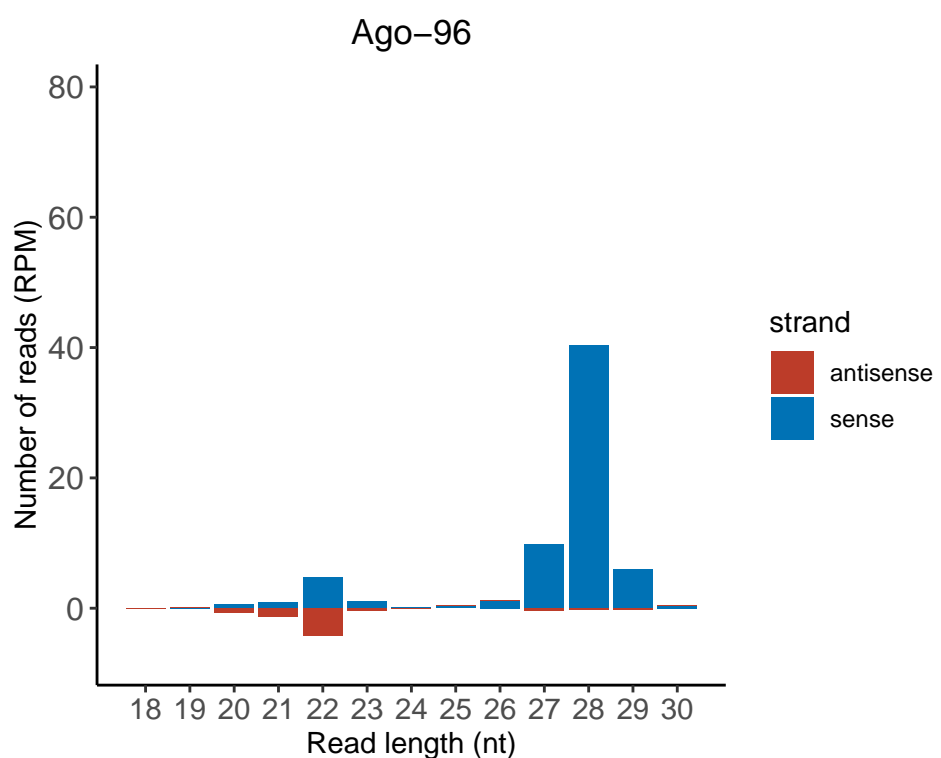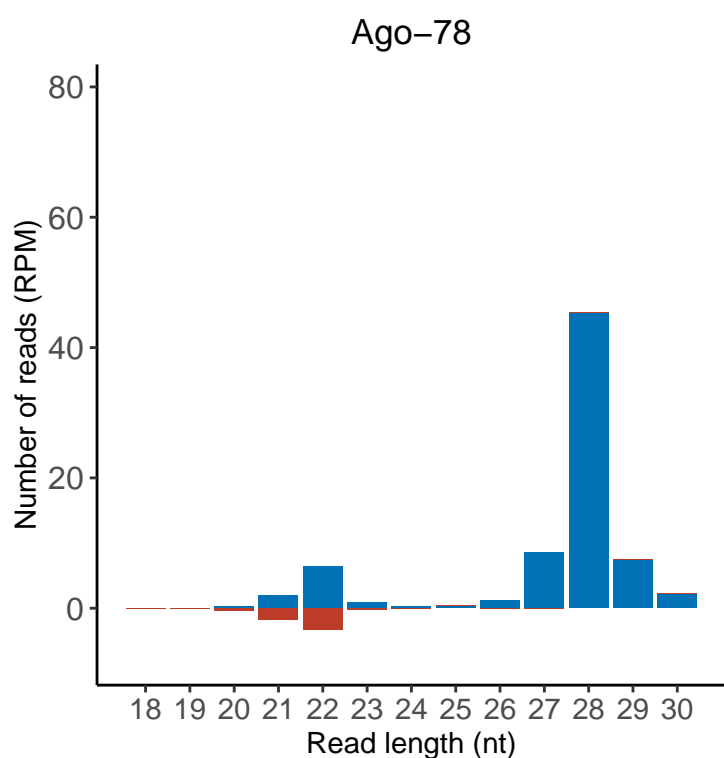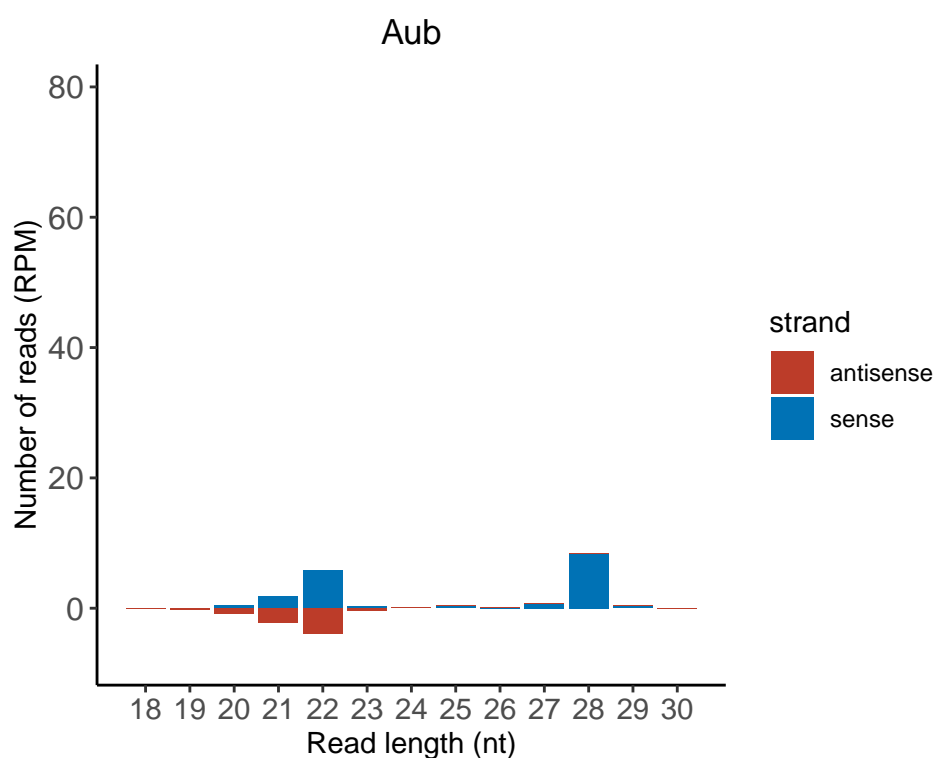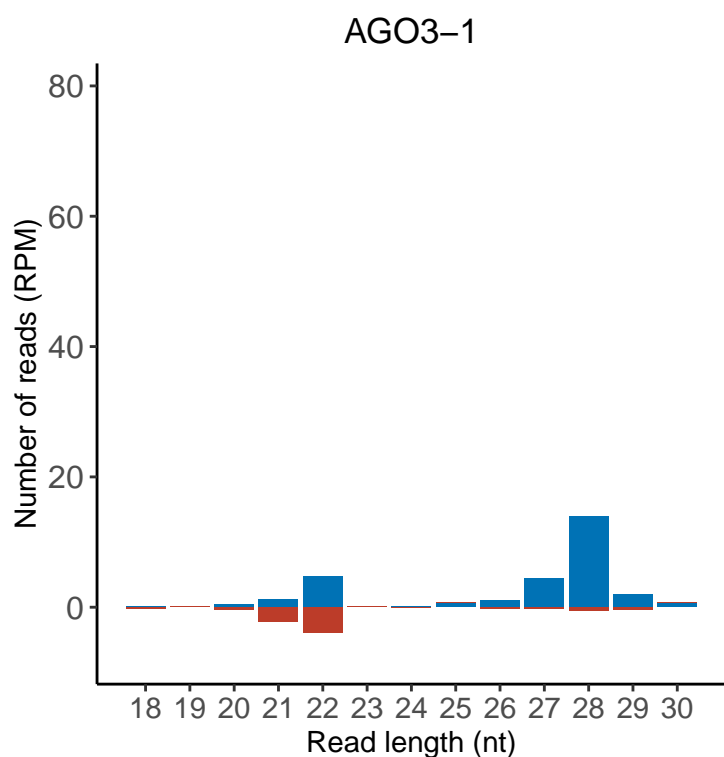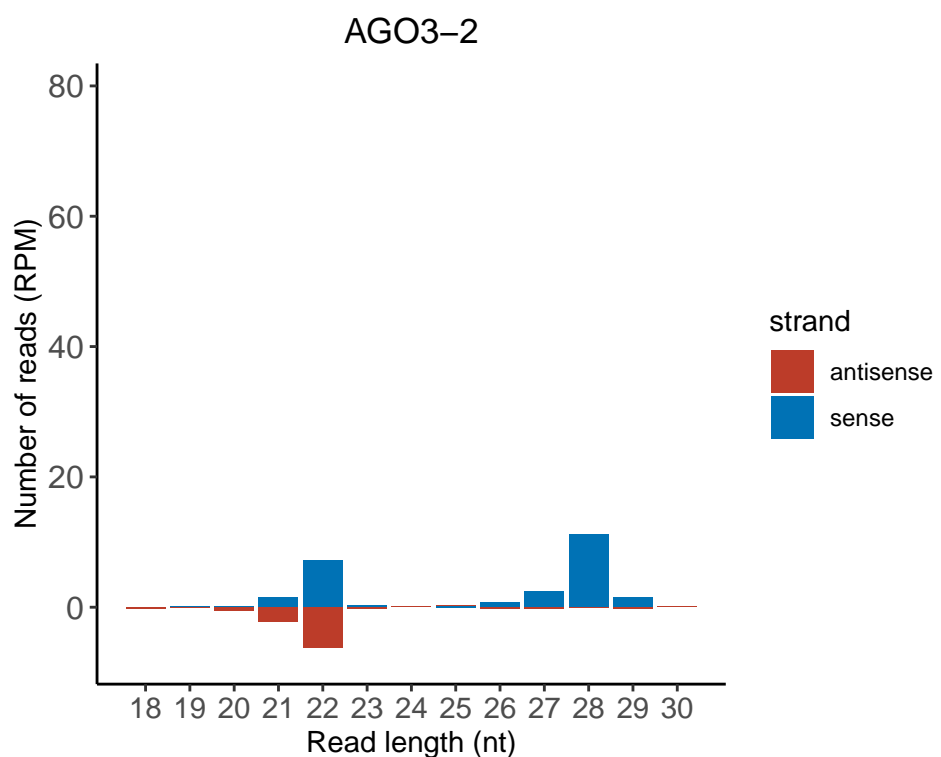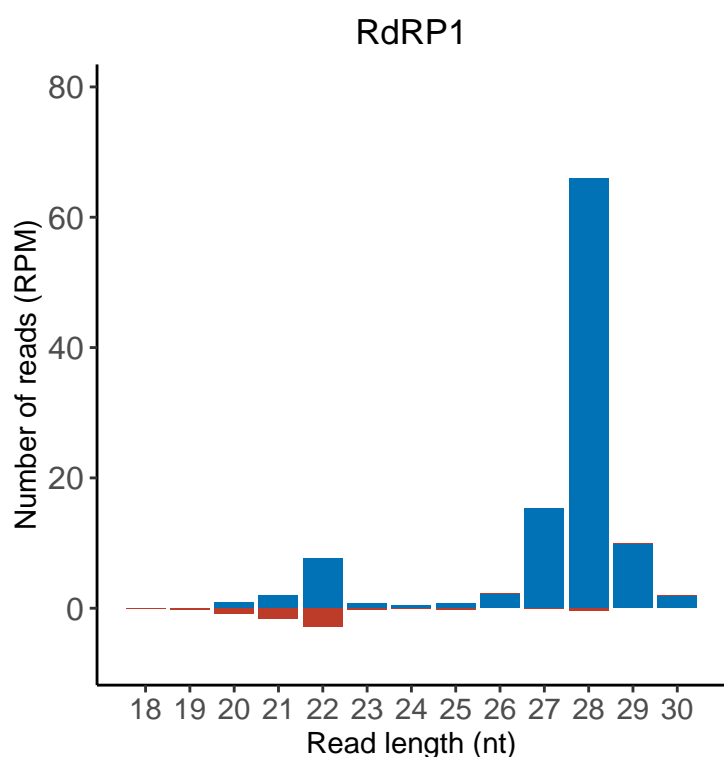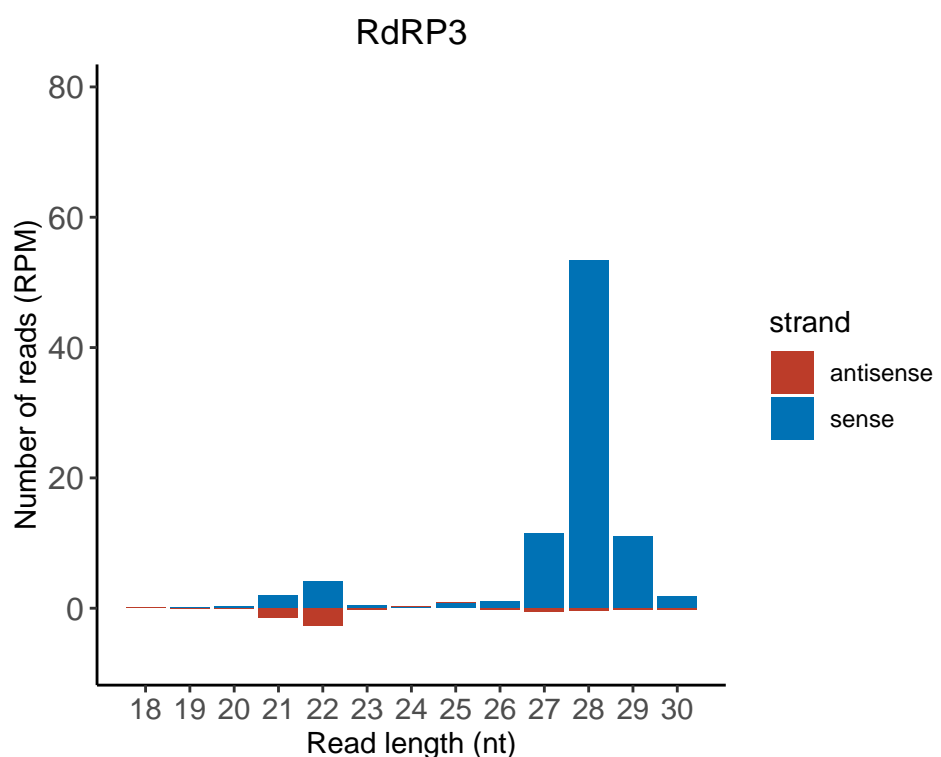

Supplement: S1 Data — On the Normalized and Relative levels pages, normalized read counts (RPM) and relative levels compared to the control (dsGFP) library were used. TEs with more than 50RPM and Coding Genes with more than 3.5RPM on average in KD libraries are included in this website. For viral sequences, reference sequences were generated by assembling sRNA sequences (See the Analysis of sRNAseq data section in Materials and methods.) and size distributions of sRNA reads mapped to each contig are shown. Reads were first grouped by their 5’ nucleotides, and reads of each length were counted. Full tables including TEs and Coding genes with fewer reads are in the “FullTable” folder. (ZIP) [file pone.0281195.s017.zip › SupplementaryData/Links/PDFs/Motif:rnd-5_family-2257TE_lengthdistribution.pdf]

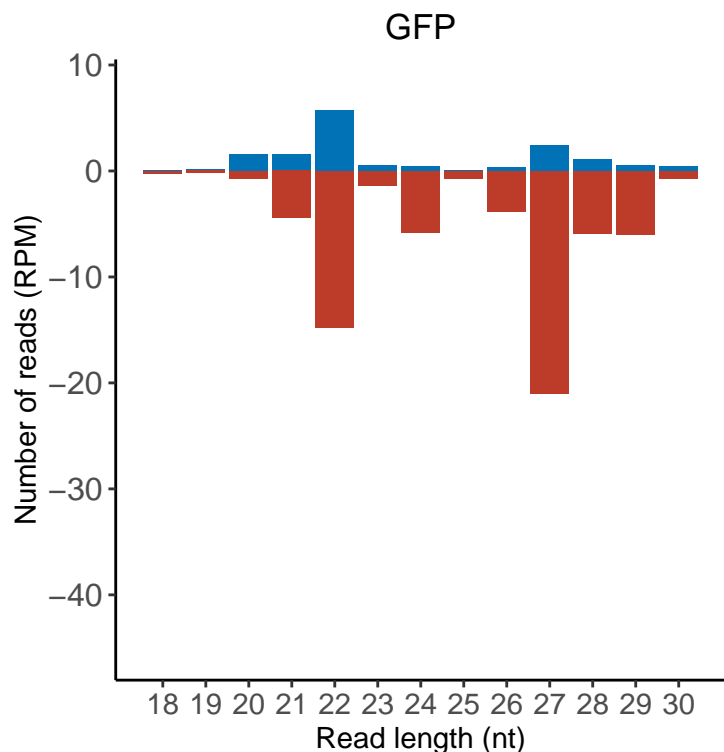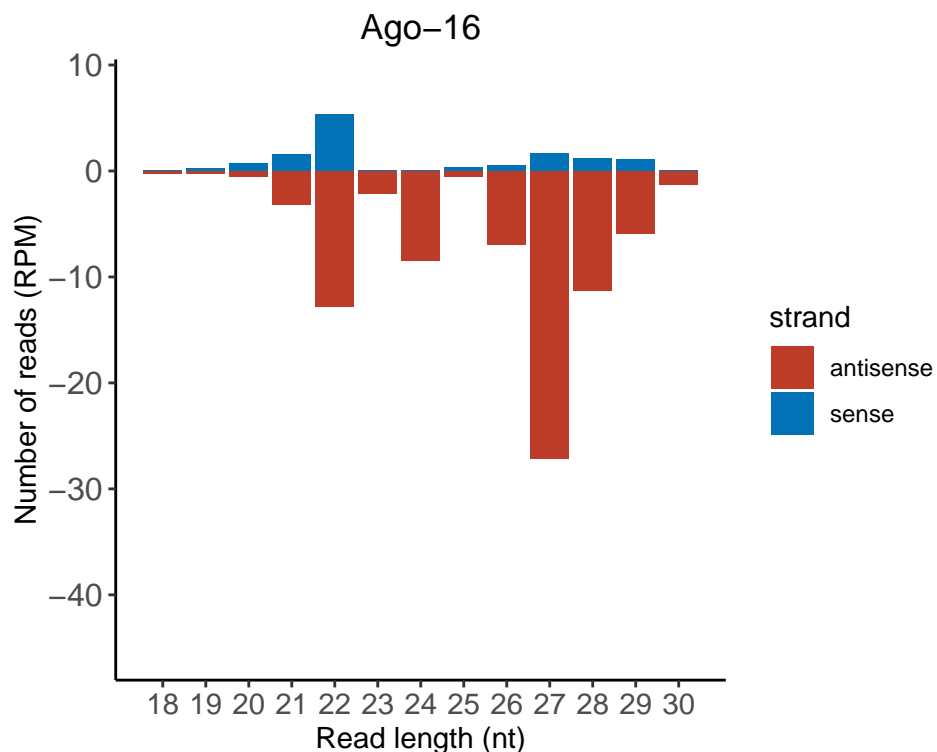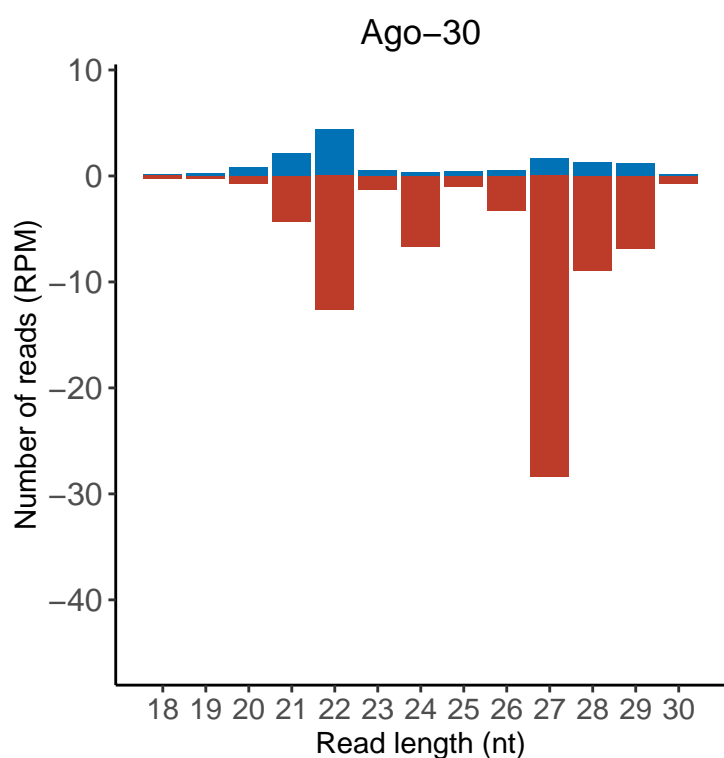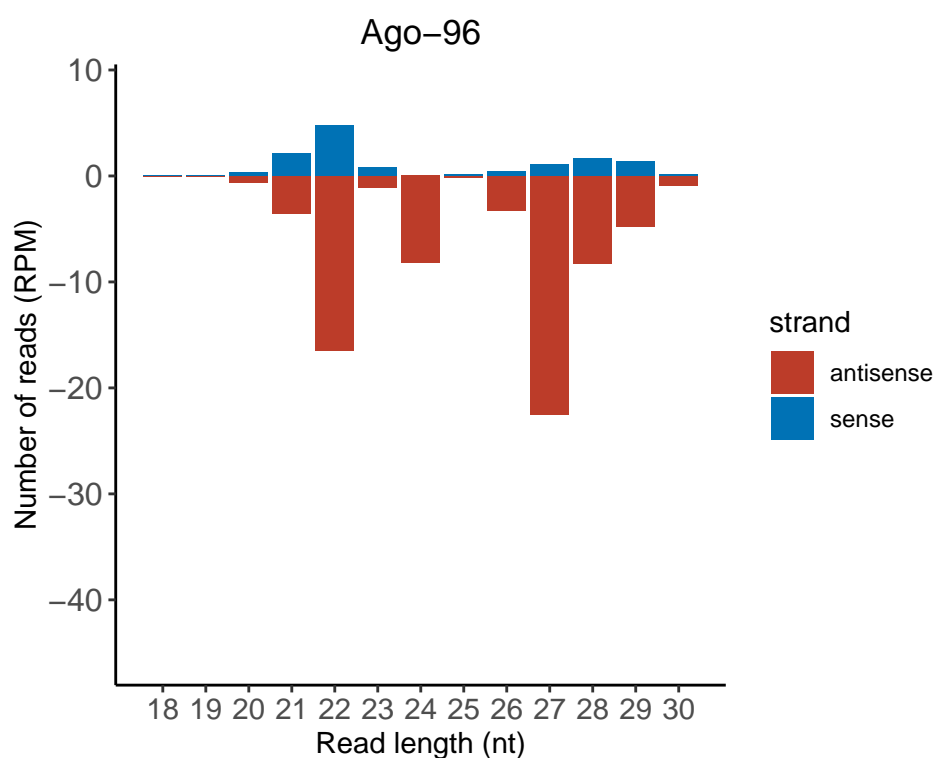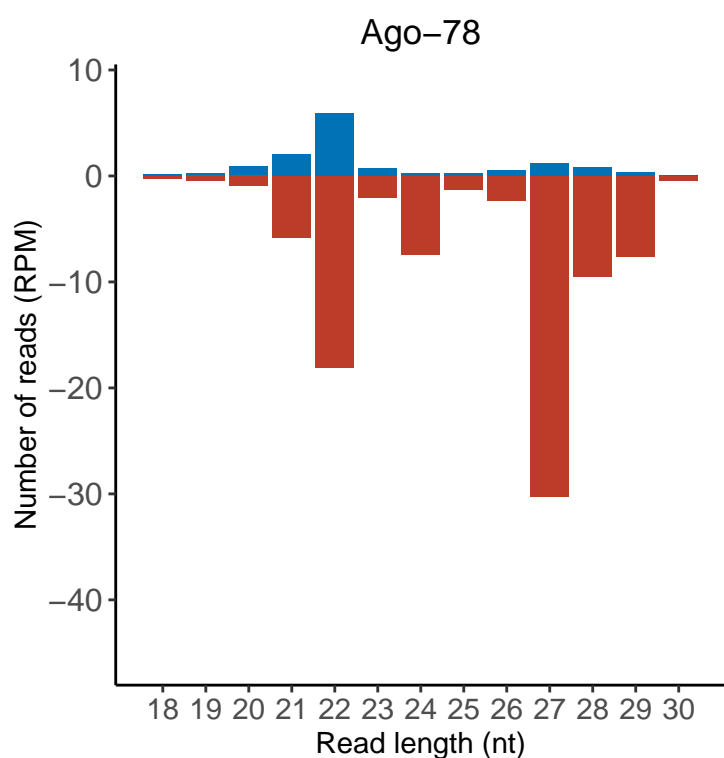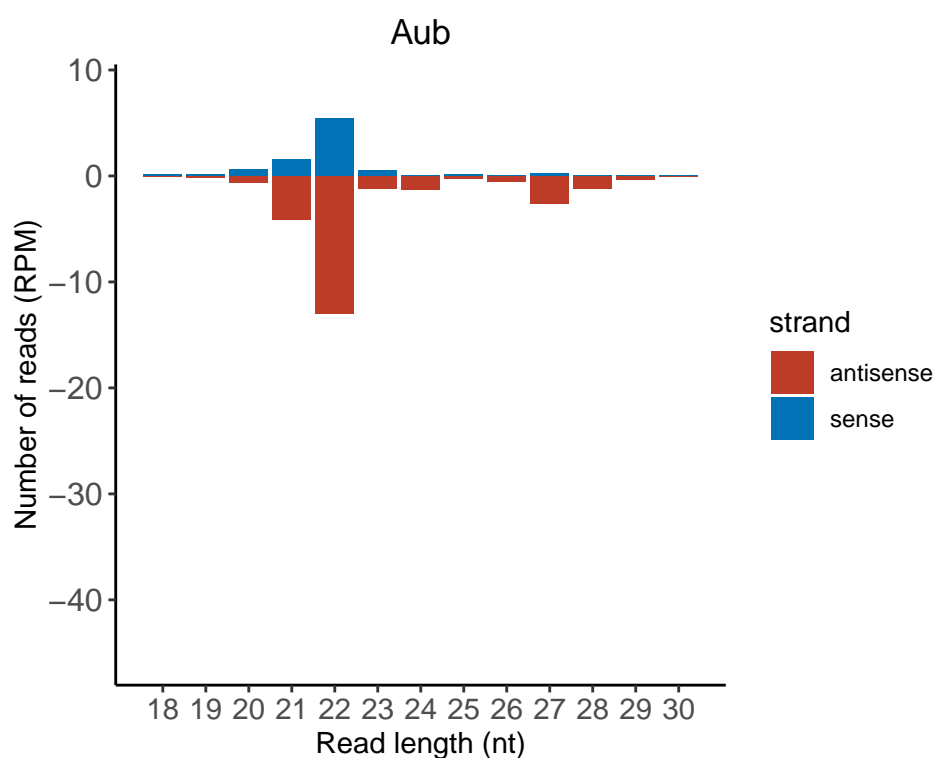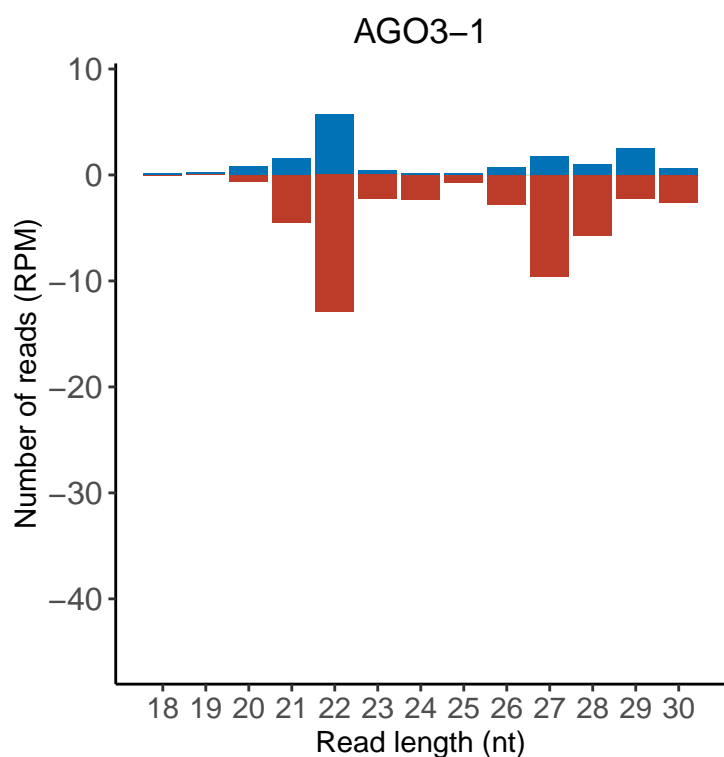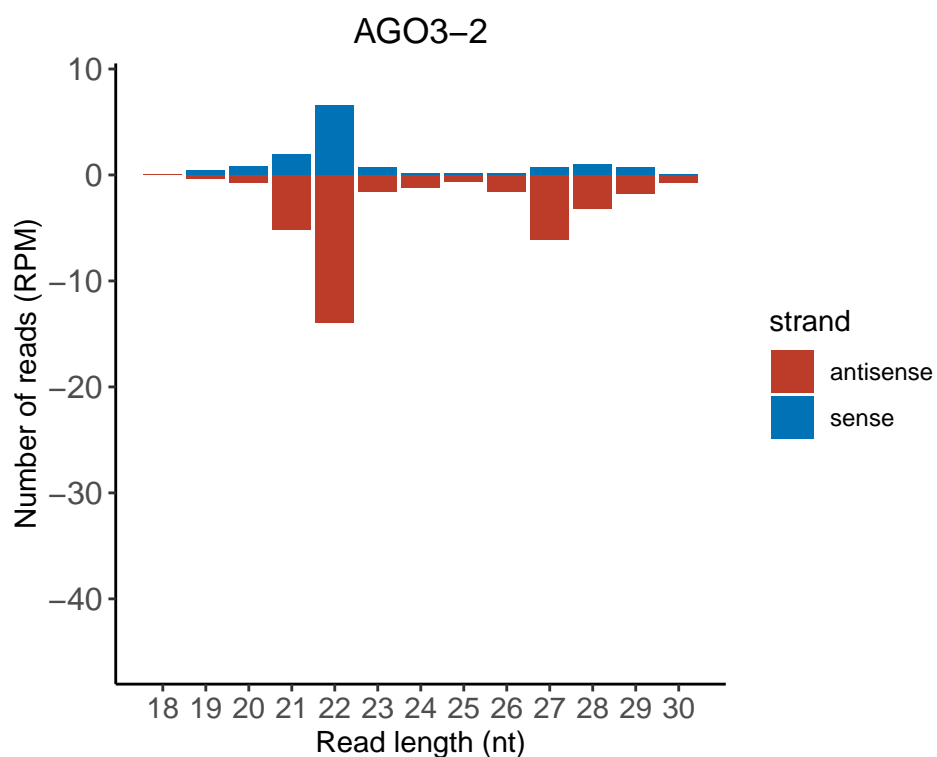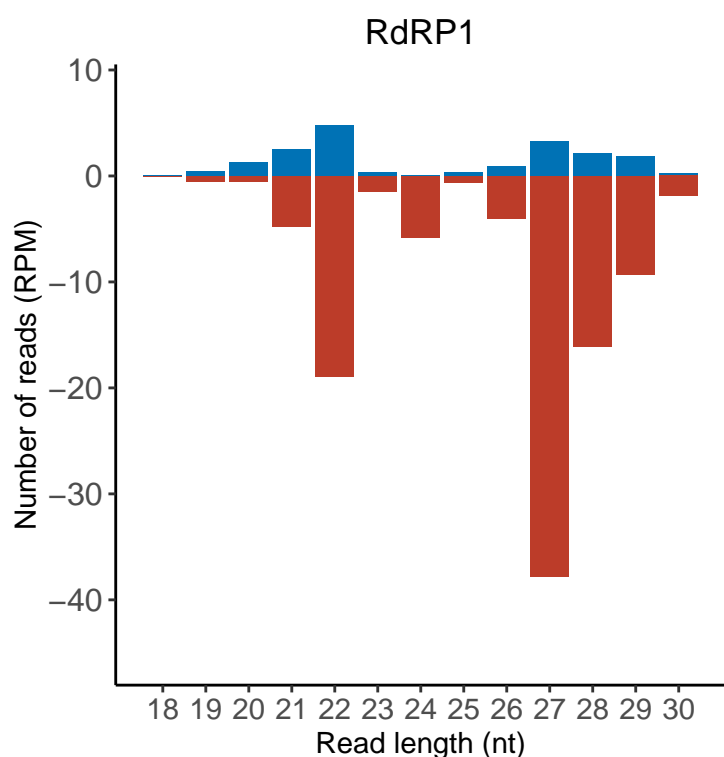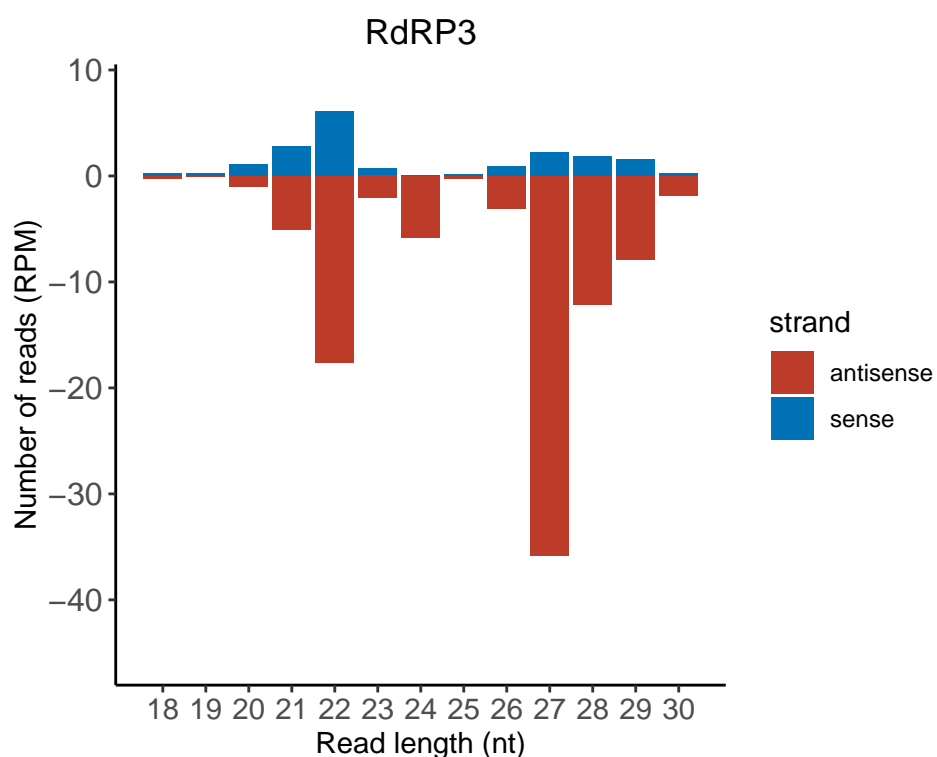

Supplement: S1 Data — On the Normalized and Relative levels pages, normalized read counts (RPM) and relative levels compared to the control (dsGFP) library were used. TEs with more than 50RPM and Coding Genes with more than 3.5RPM on average in KD libraries are included in this website. For viral sequences, reference sequences were generated by assembling sRNA sequences (See the Analysis of sRNAseq data section in Materials and methods.) and size distributions of sRNA reads mapped to each contig are shown. Reads were first grouped by their 5’ nucleotides, and reads of each length were counted. Full tables including TEs and Coding genes with fewer reads are in the “FullTable” folder. (ZIP) [file pone.0281195.s017.zip › SupplementaryData/Links/PDFs/Motif:rnd-1_family-1128TE_lengthdistribution.pdf]

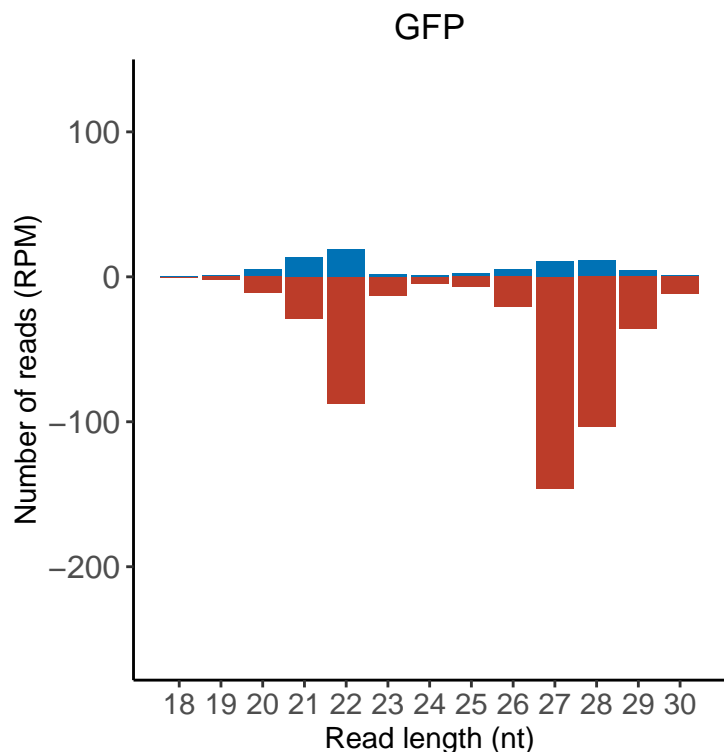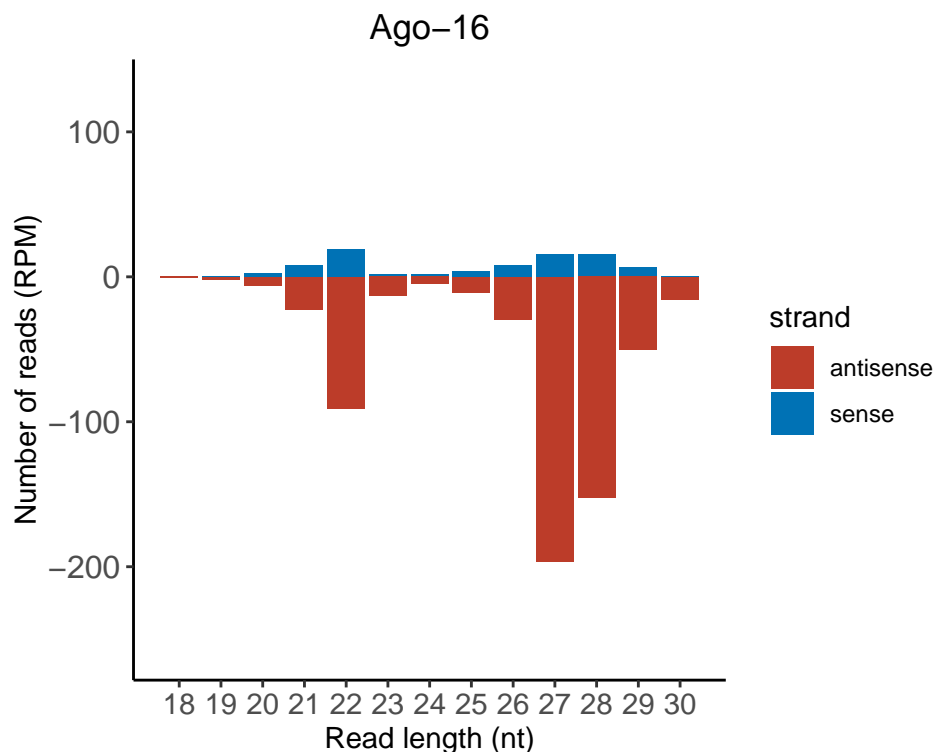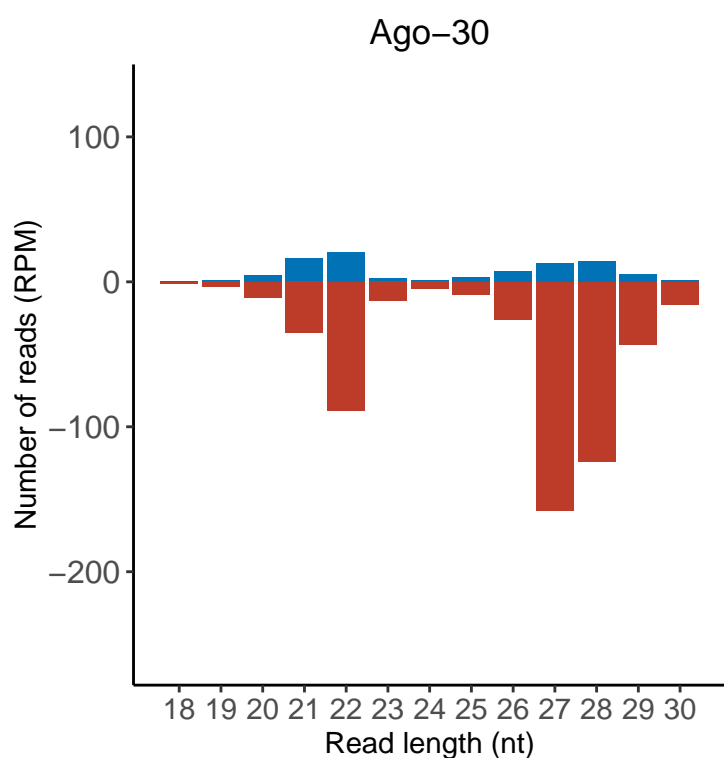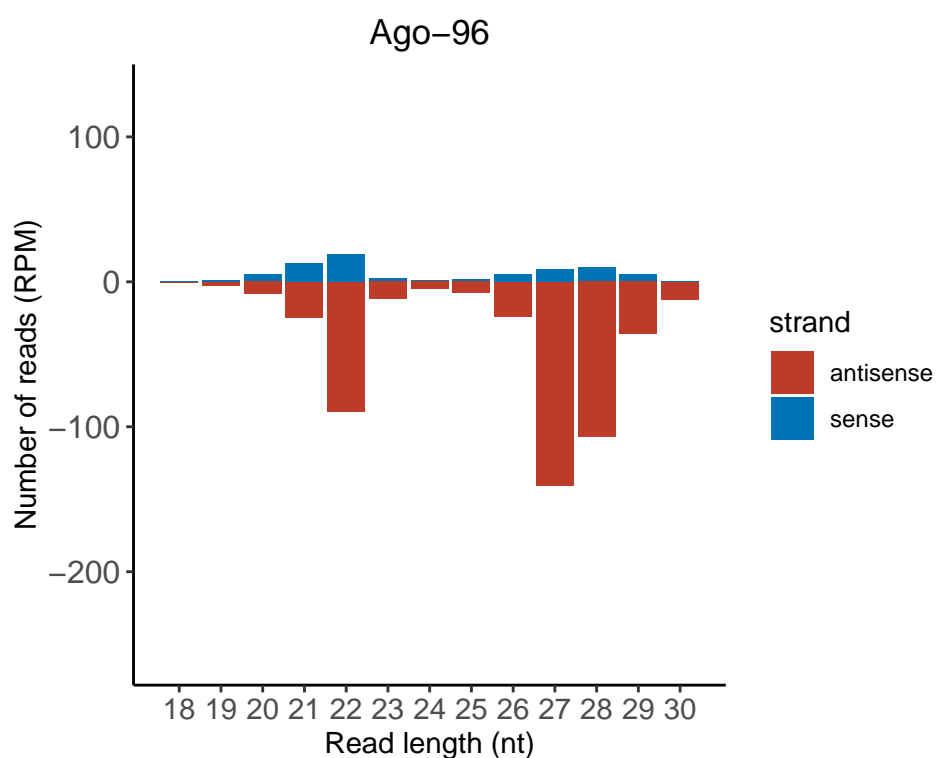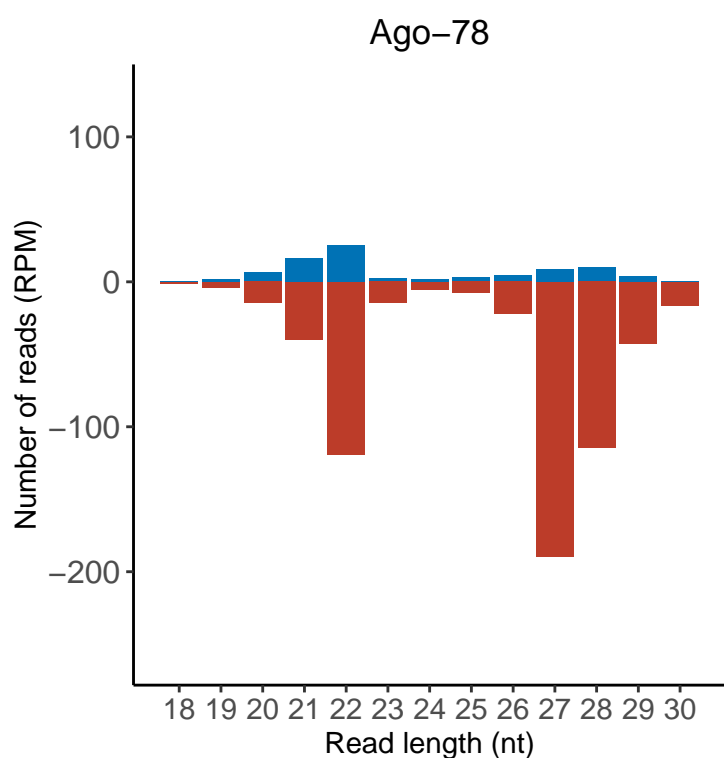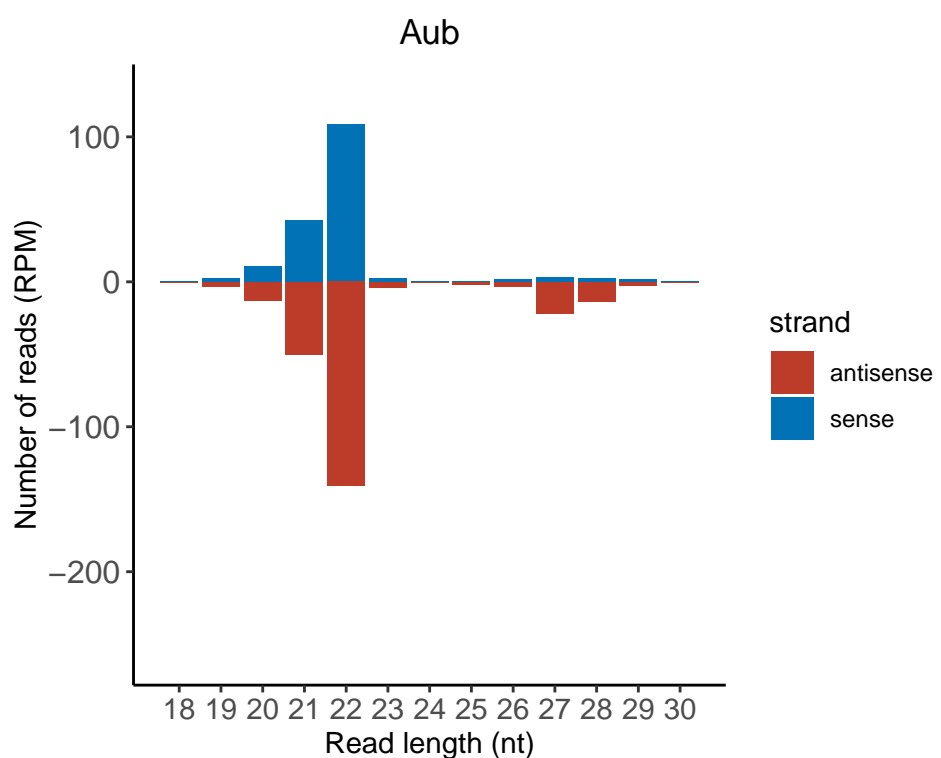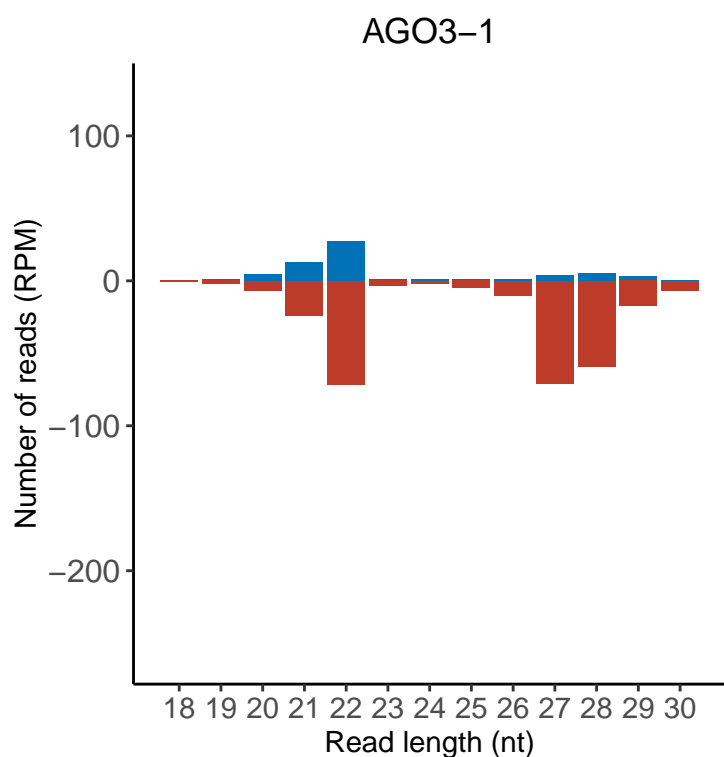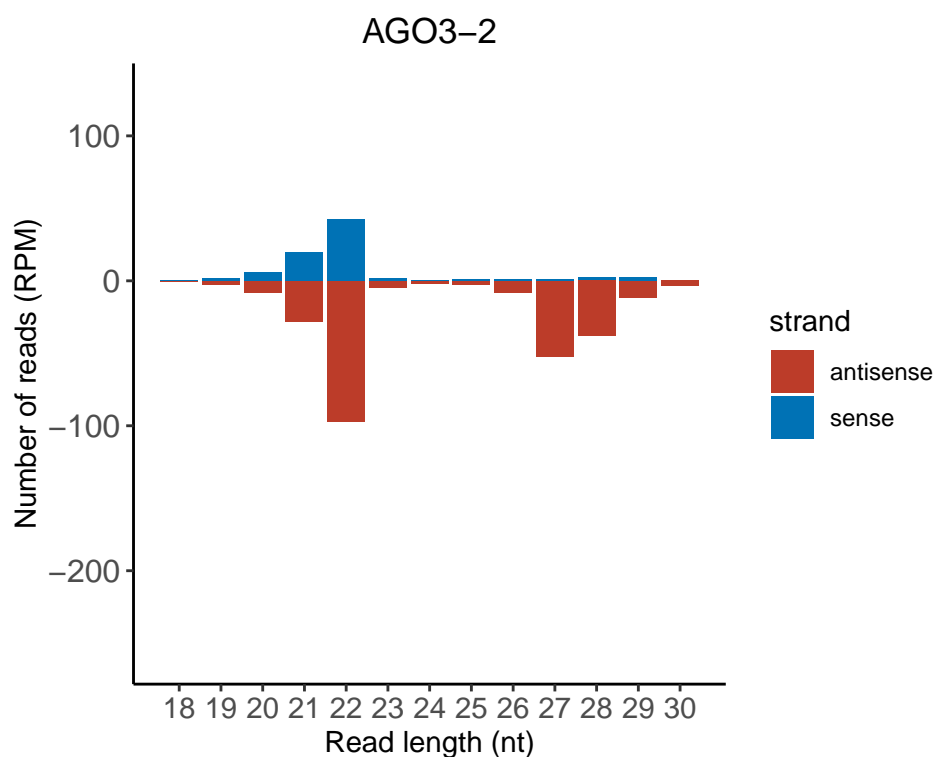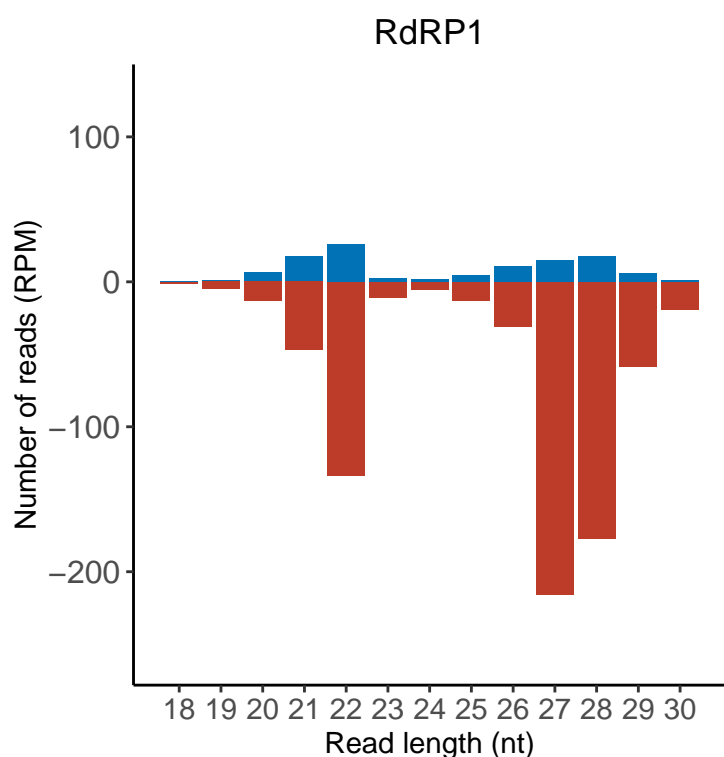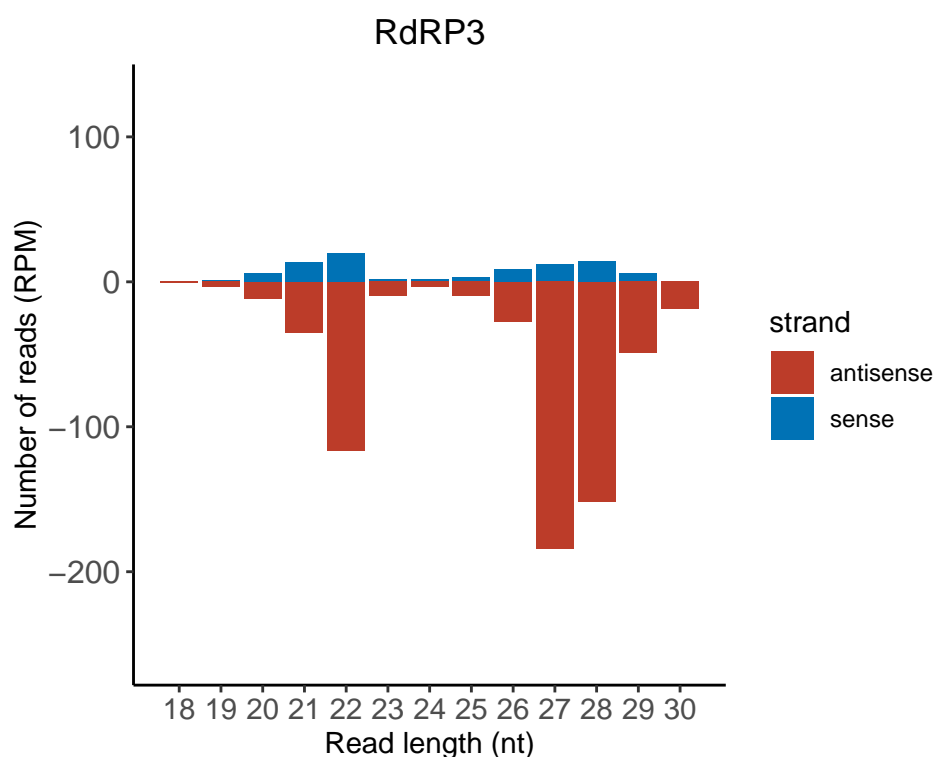

Supplement: S1 Data — On the Normalized and Relative levels pages, normalized read counts (RPM) and relative levels compared to the control (dsGFP) library were used. TEs with more than 50RPM and Coding Genes with more than 3.5RPM on average in KD libraries are included in this website. For viral sequences, reference sequences were generated by assembling sRNA sequences (See the Analysis of sRNAseq data section in Materials and methods.) and size distributions of sRNA reads mapped to each contig are shown. Reads were first grouped by their 5’ nucleotides, and reads of each length were counted. Full tables including TEs and Coding genes with fewer reads are in the “FullTable” folder. (ZIP) [file pone.0281195.s017.zip › SupplementaryData/Links/PDFs/Motif:rnd-1_family-463TE_lengthdistribution.pdf]

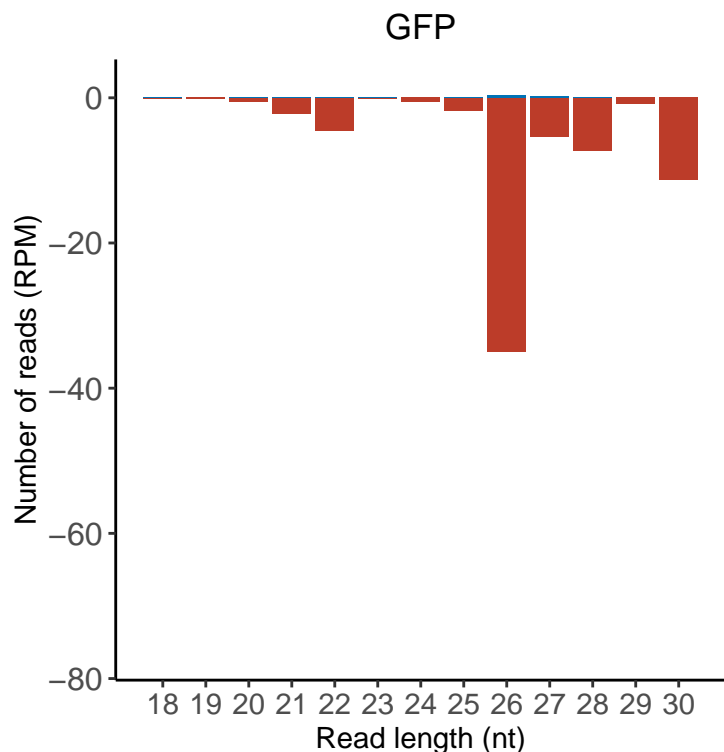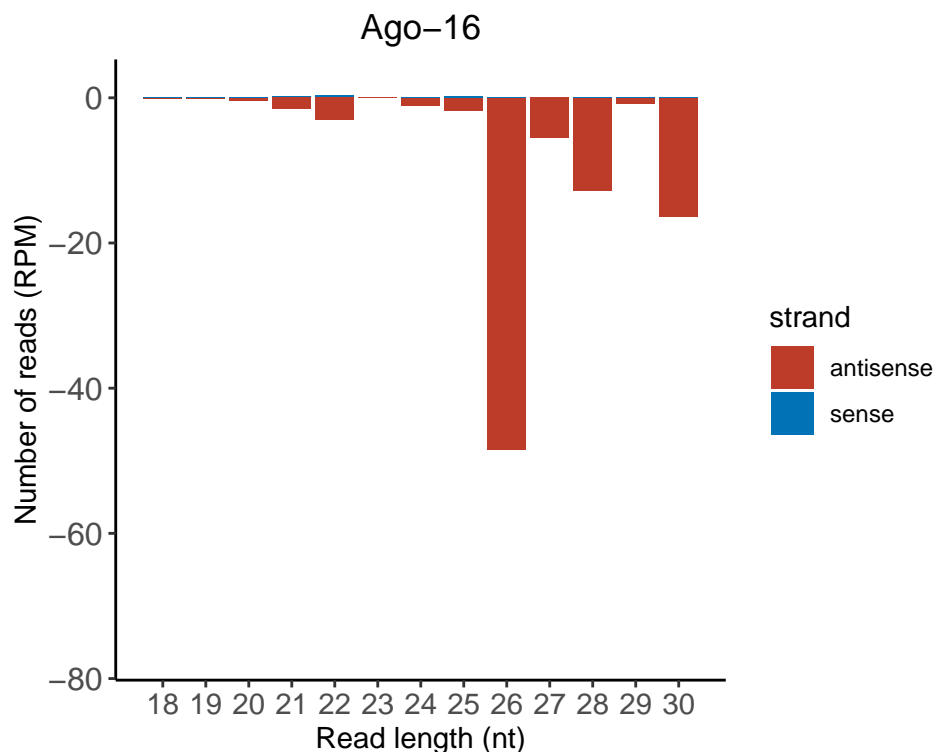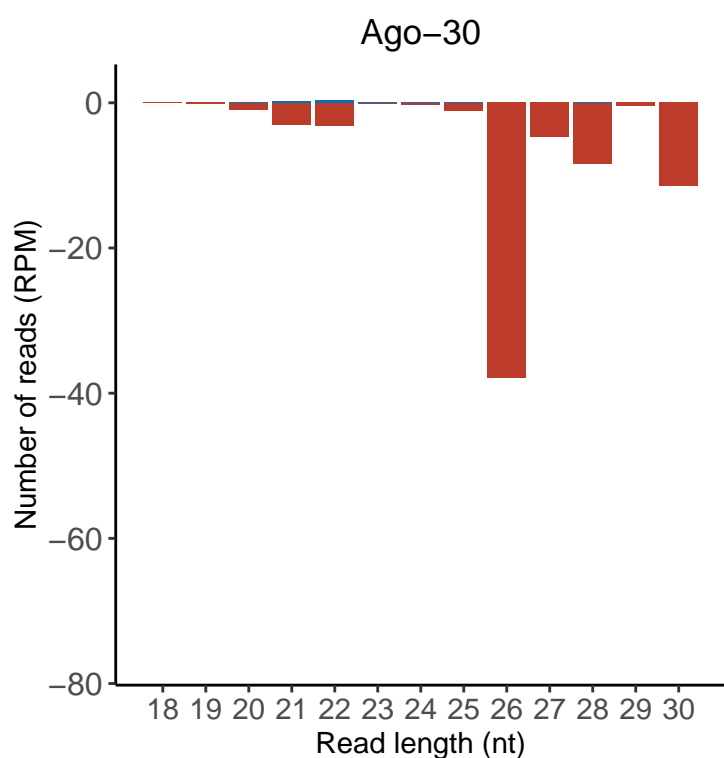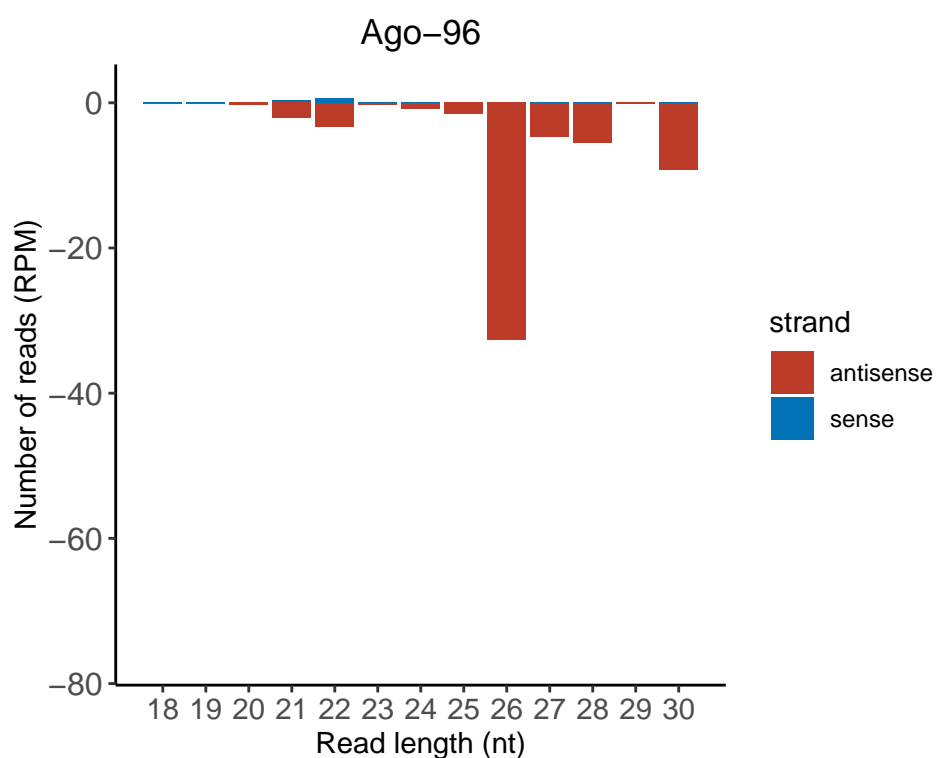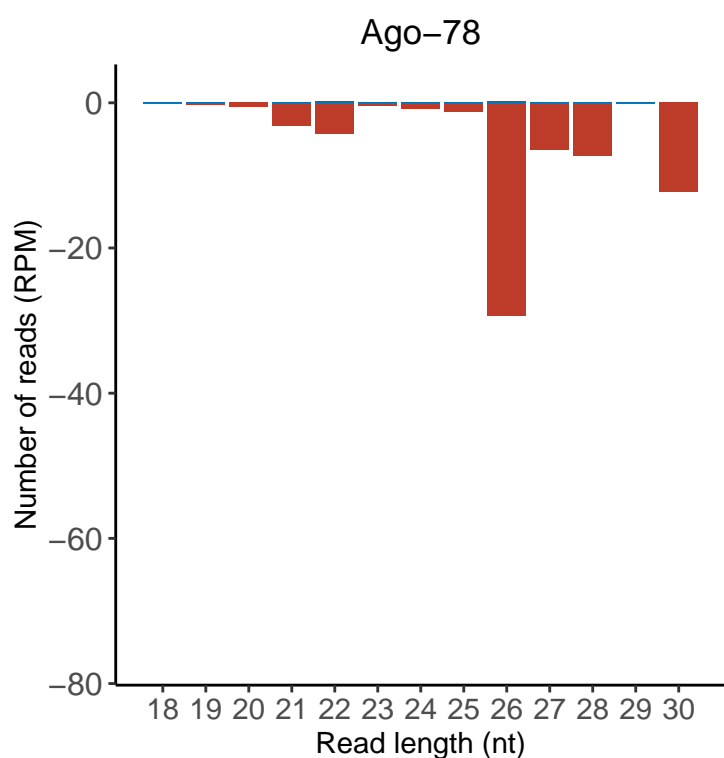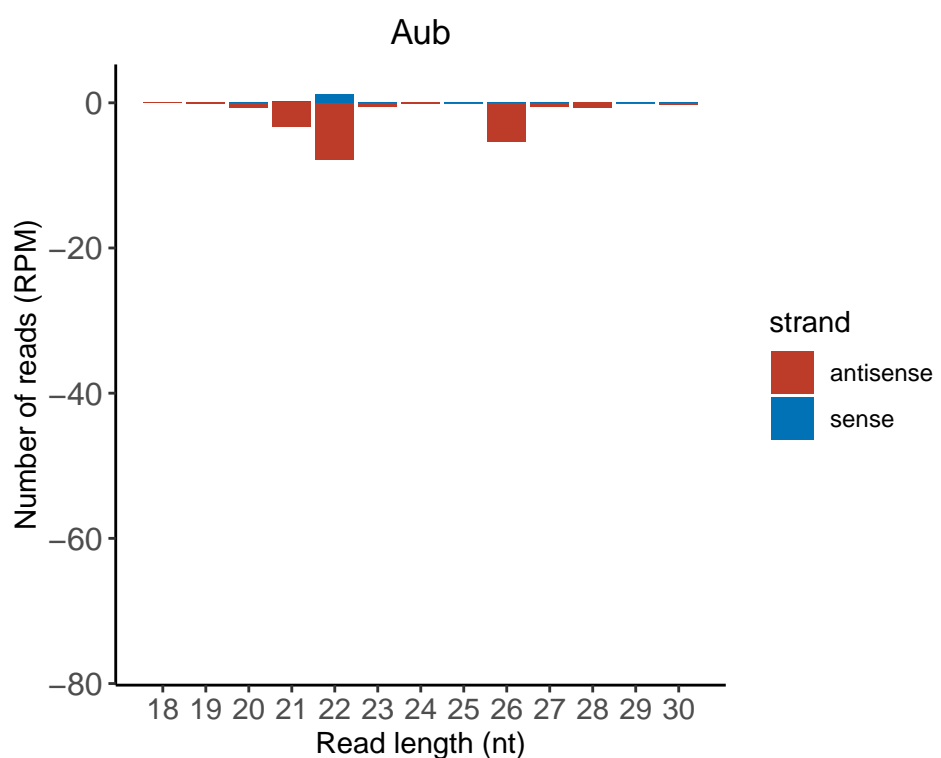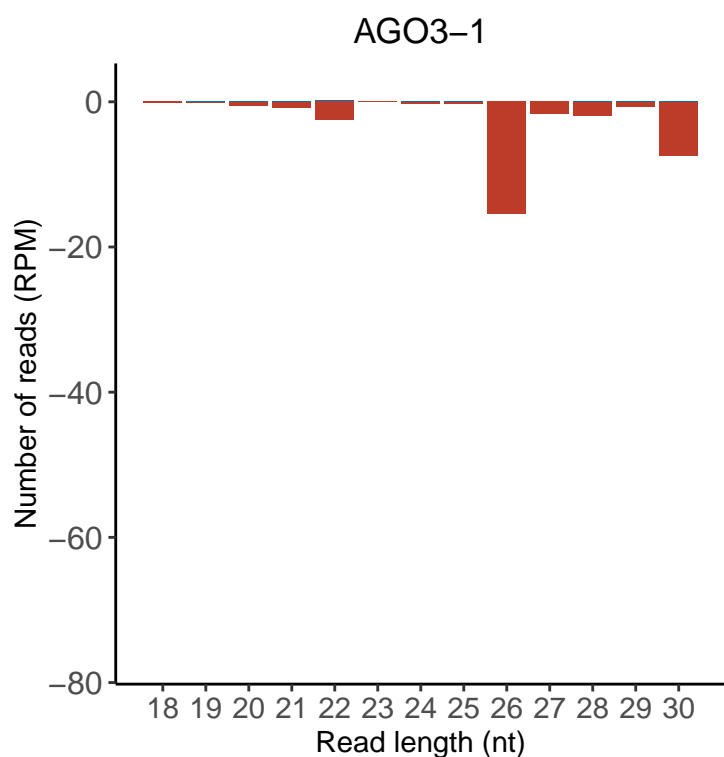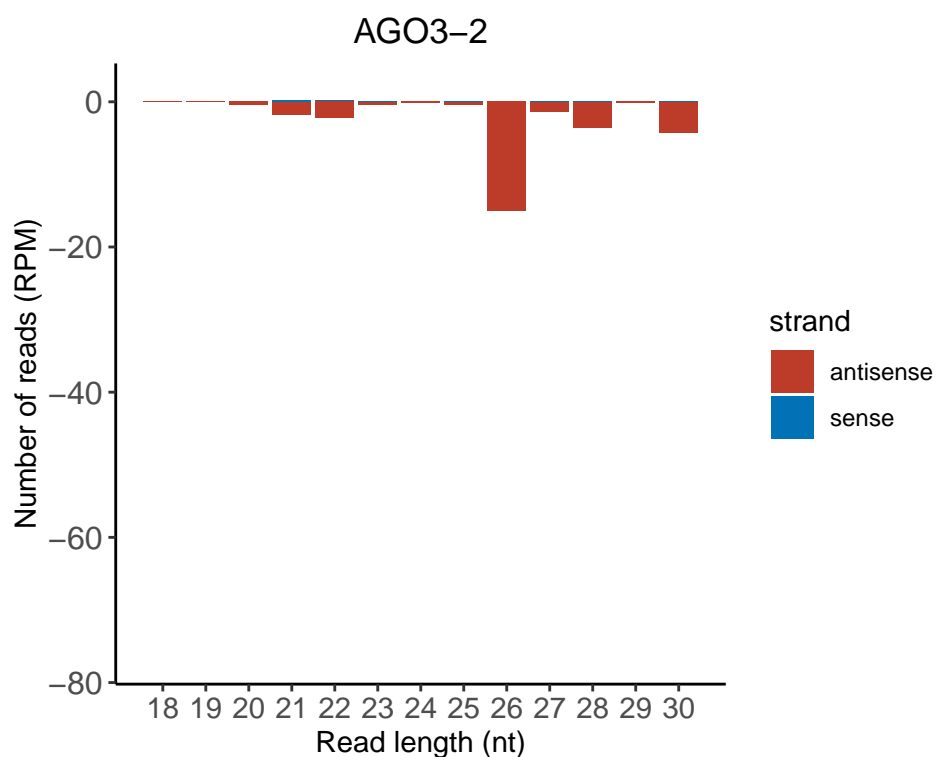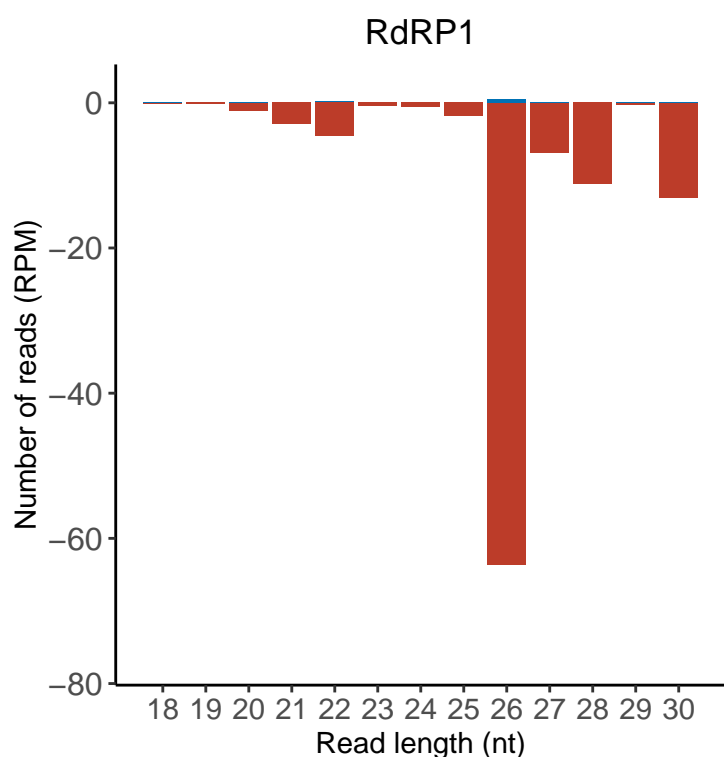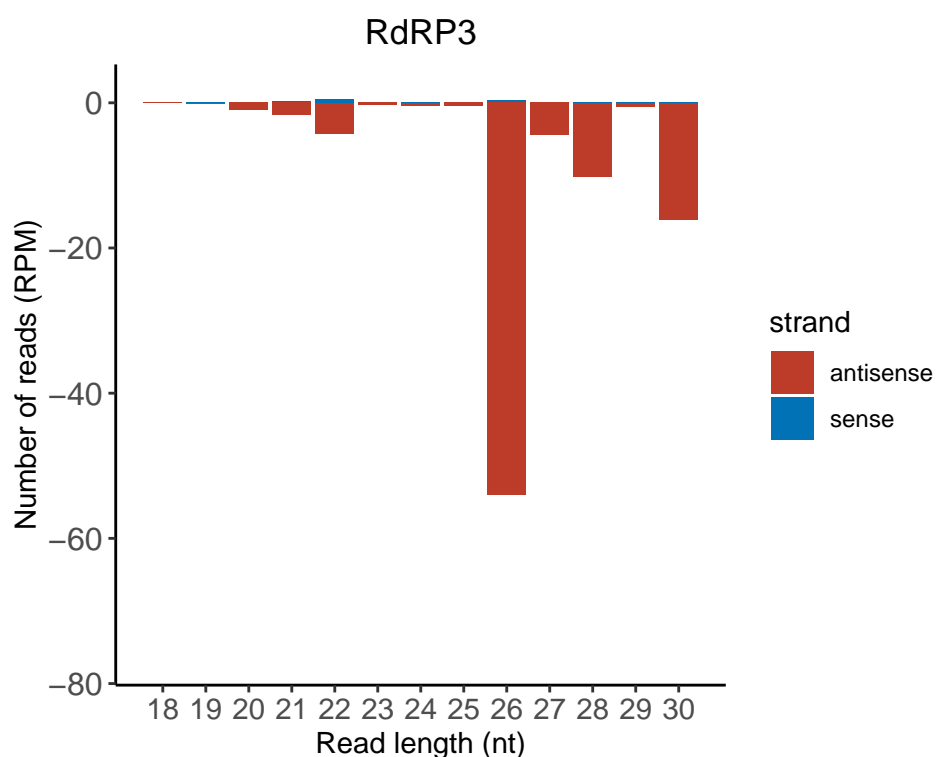

Supplement: S1 Data — On the Normalized and Relative levels pages, normalized read counts (RPM) and relative levels compared to the control (dsGFP) library were used. TEs with more than 50RPM and Coding Genes with more than 3.5RPM on average in KD libraries are included in this website. For viral sequences, reference sequences were generated by assembling sRNA sequences (See the Analysis of sRNAseq data section in Materials and methods.) and size distributions of sRNA reads mapped to each contig are shown. Reads were first grouped by their 5’ nucleotides, and reads of each length were counted. Full tables including TEs and Coding genes with fewer reads are in the “FullTable” folder. (ZIP) [file pone.0281195.s017.zip › SupplementaryData/Links/PDFs/ISCI004588.RACDS_lengthdistribution.pdf]

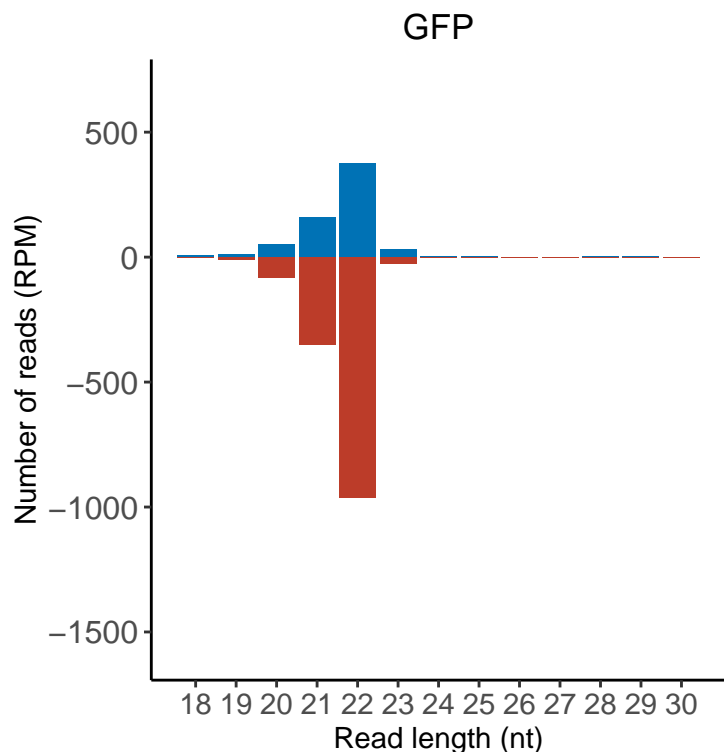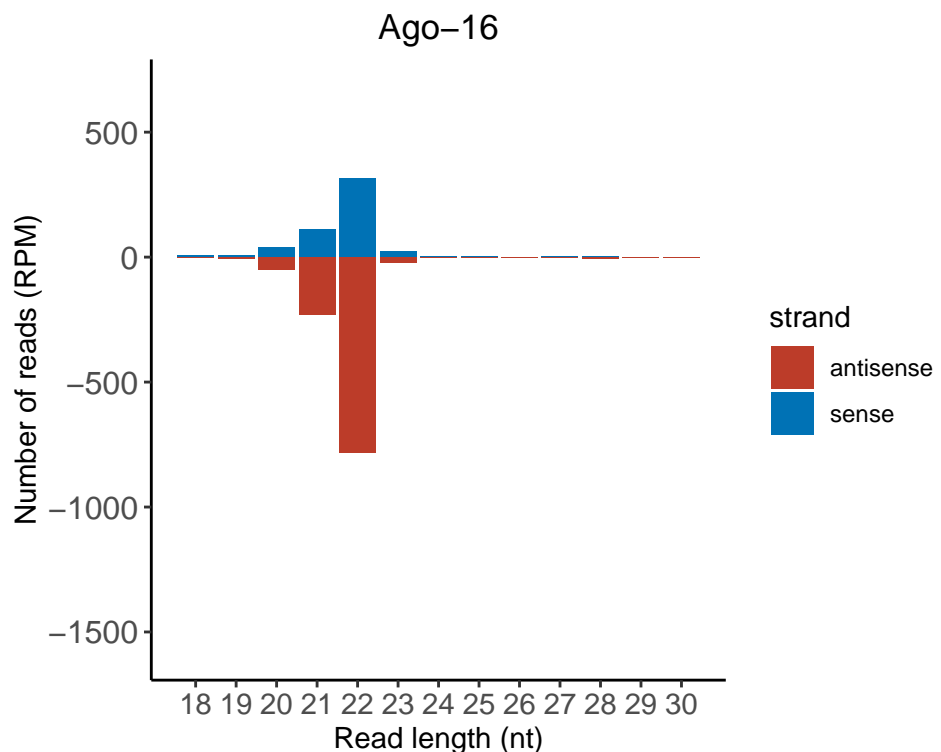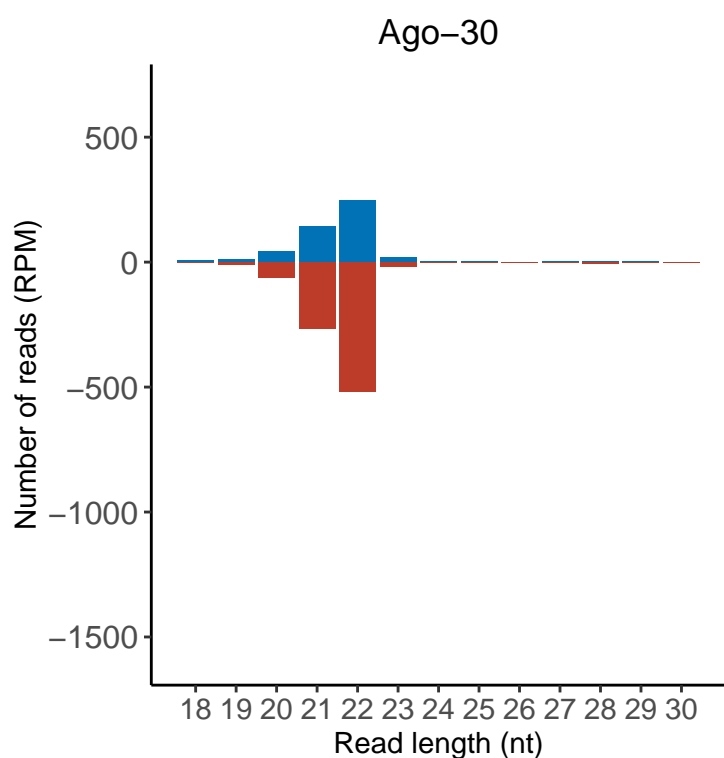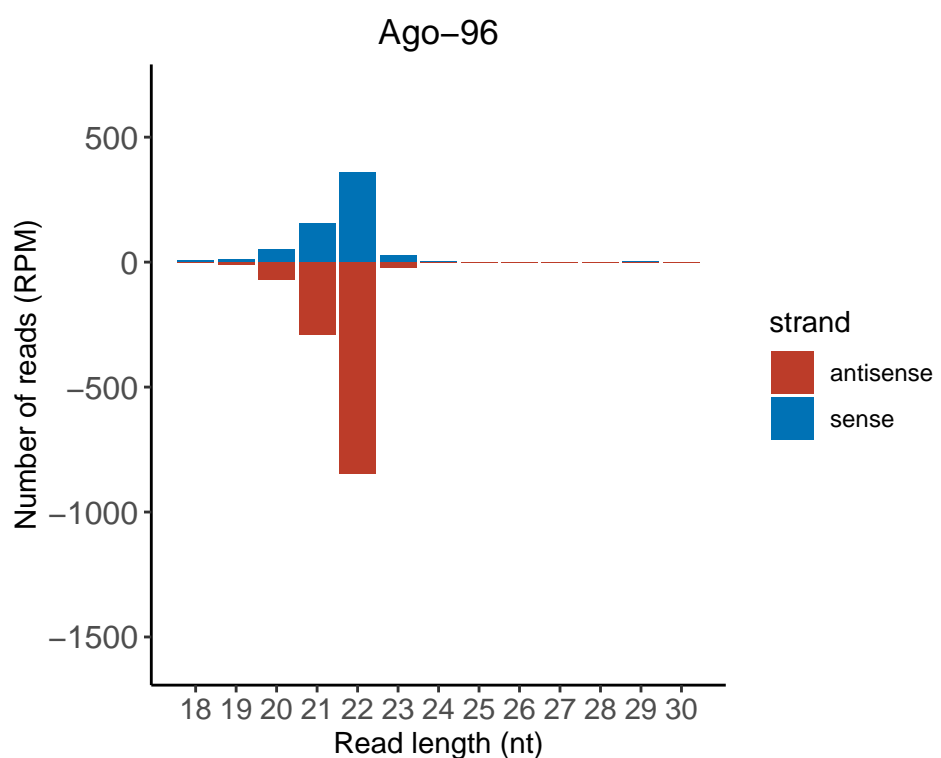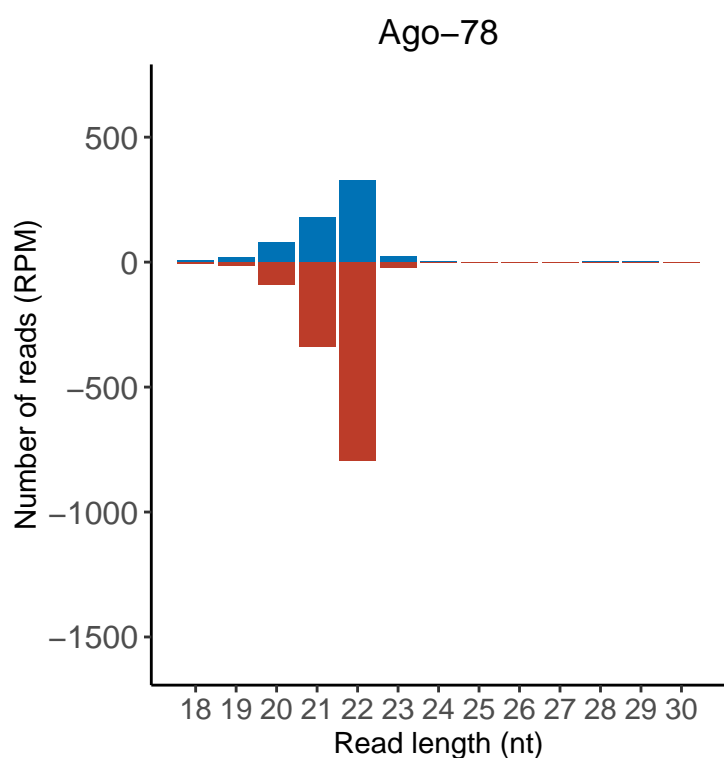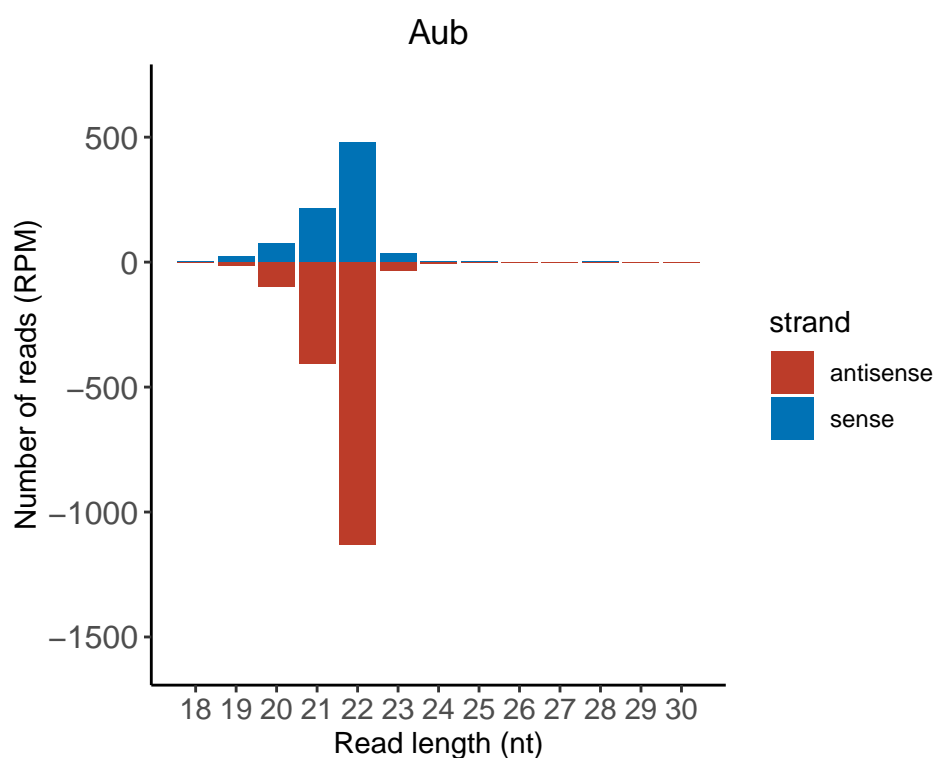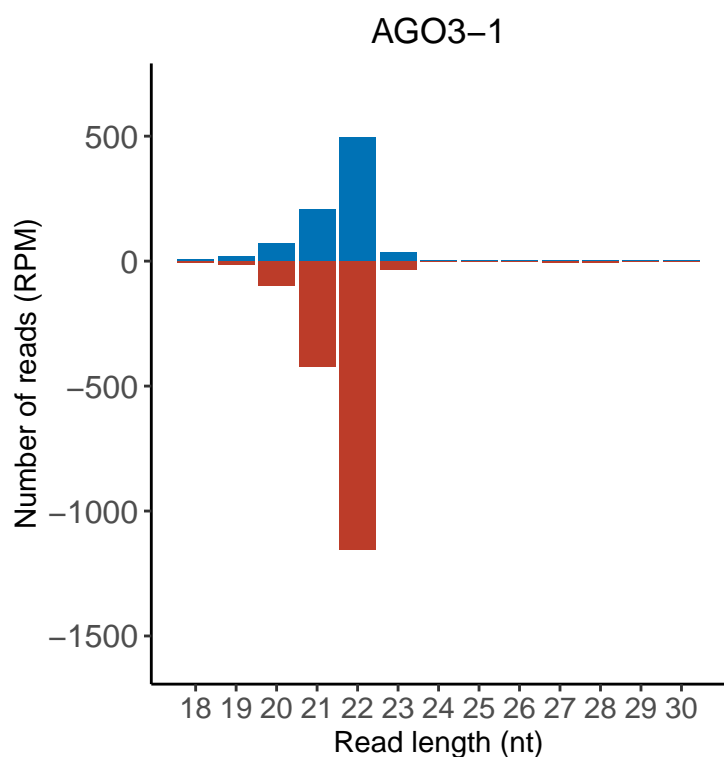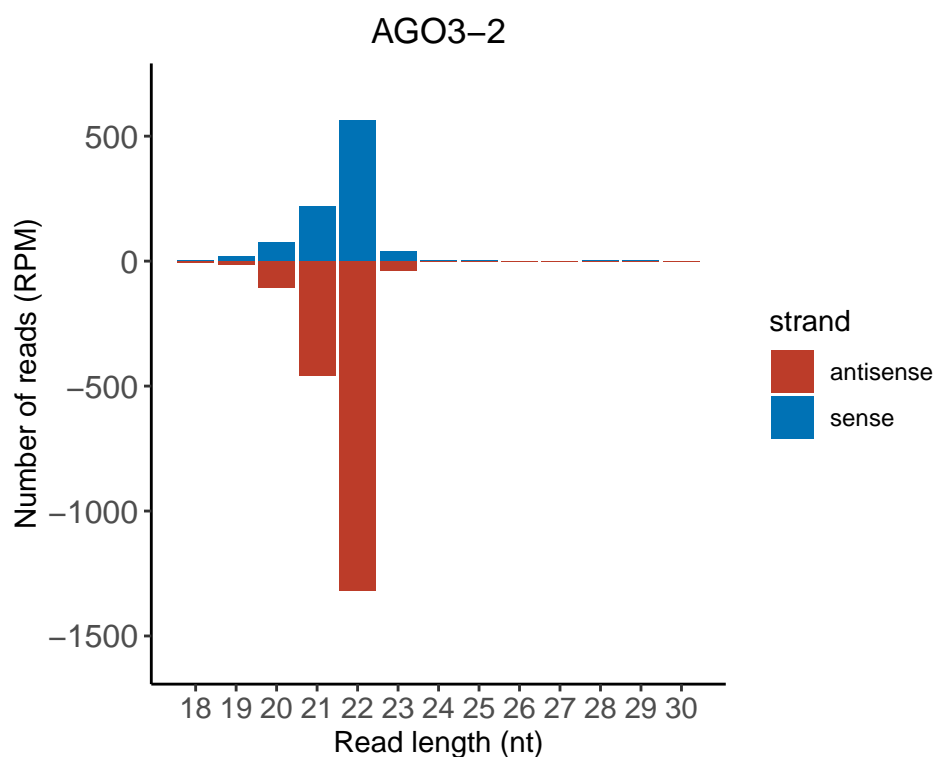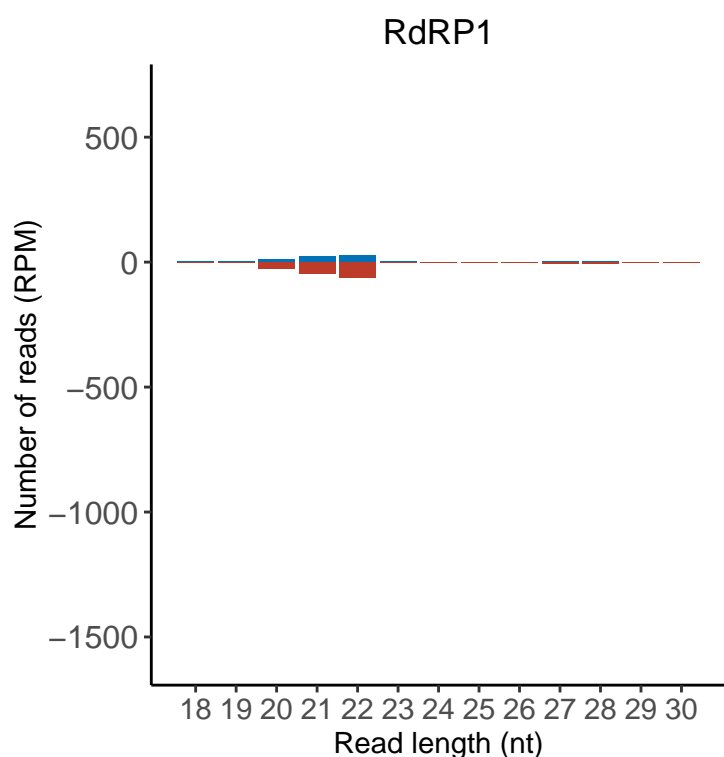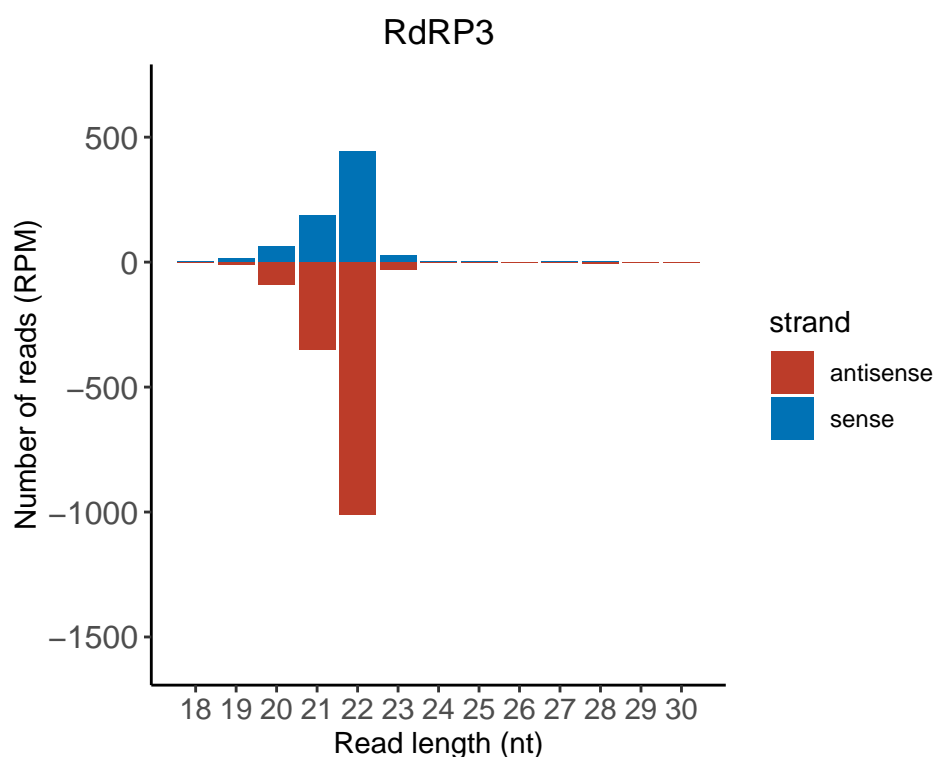

Supplement: S1 Data — On the Normalized and Relative levels pages, normalized read counts (RPM) and relative levels compared to the control (dsGFP) library were used. TEs with more than 50RPM and Coding Genes with more than 3.5RPM on average in KD libraries are included in this website. For viral sequences, reference sequences were generated by assembling sRNA sequences (See the Analysis of sRNAseq data section in Materials and methods.) and size distributions of sRNA reads mapped to each contig are shown. Reads were first grouped by their 5’ nucleotides, and reads of each length were counted. Full tables including TEs and Coding genes with fewer reads are in the “FullTable” folder. (ZIP) [file pone.0281195.s017.zip › SupplementaryData/Links/PDFs/Motif:rnd-4_family-408TE_lengthdistribution.pdf]

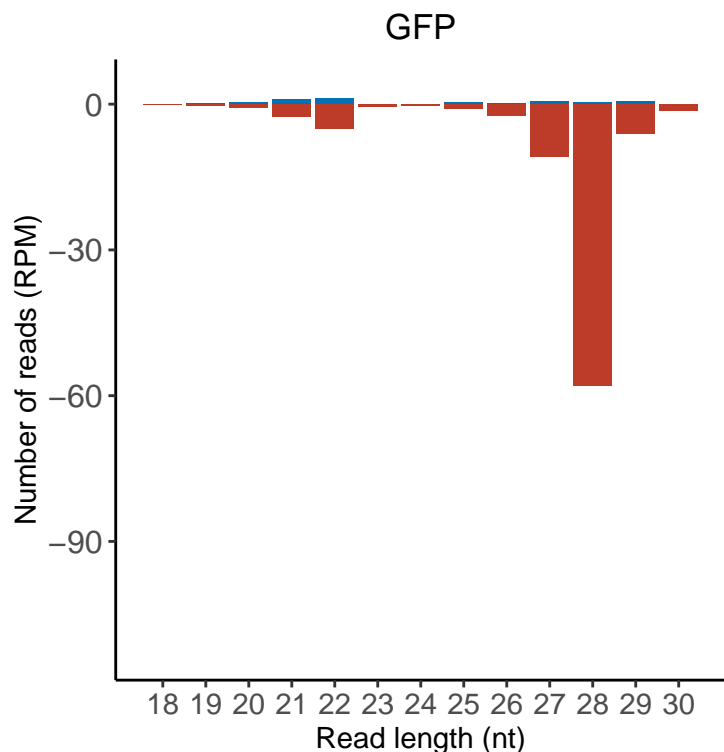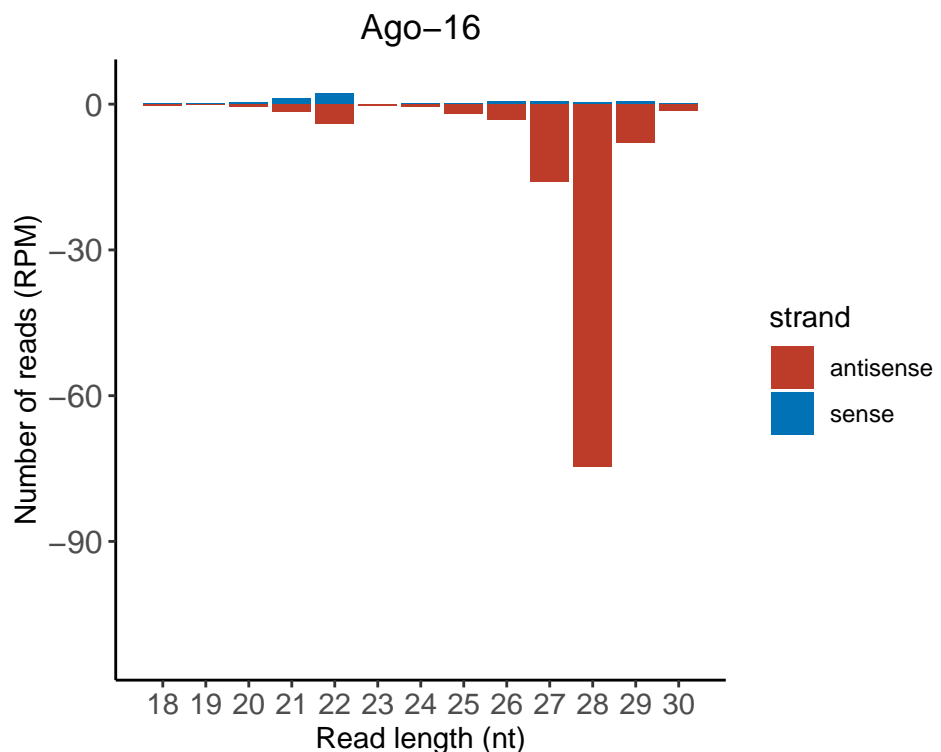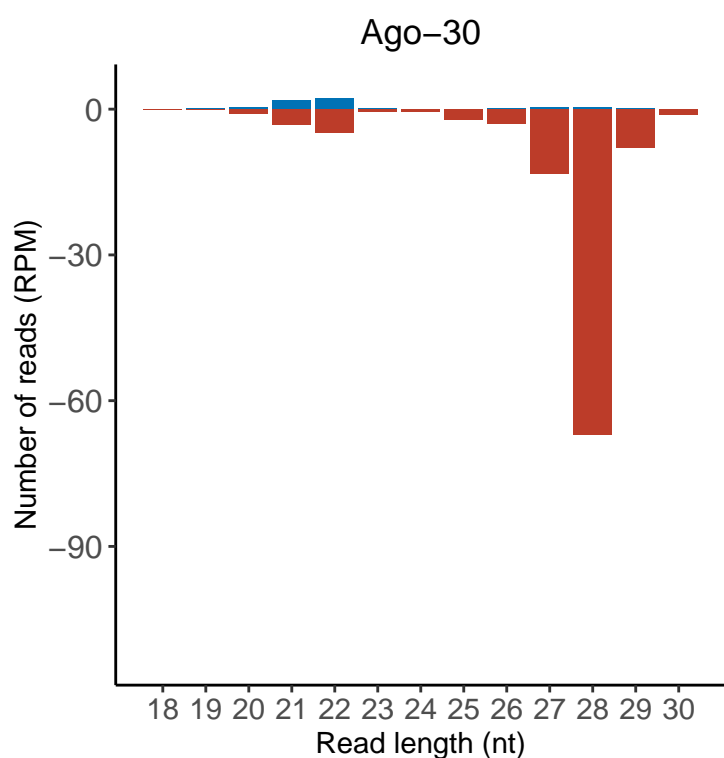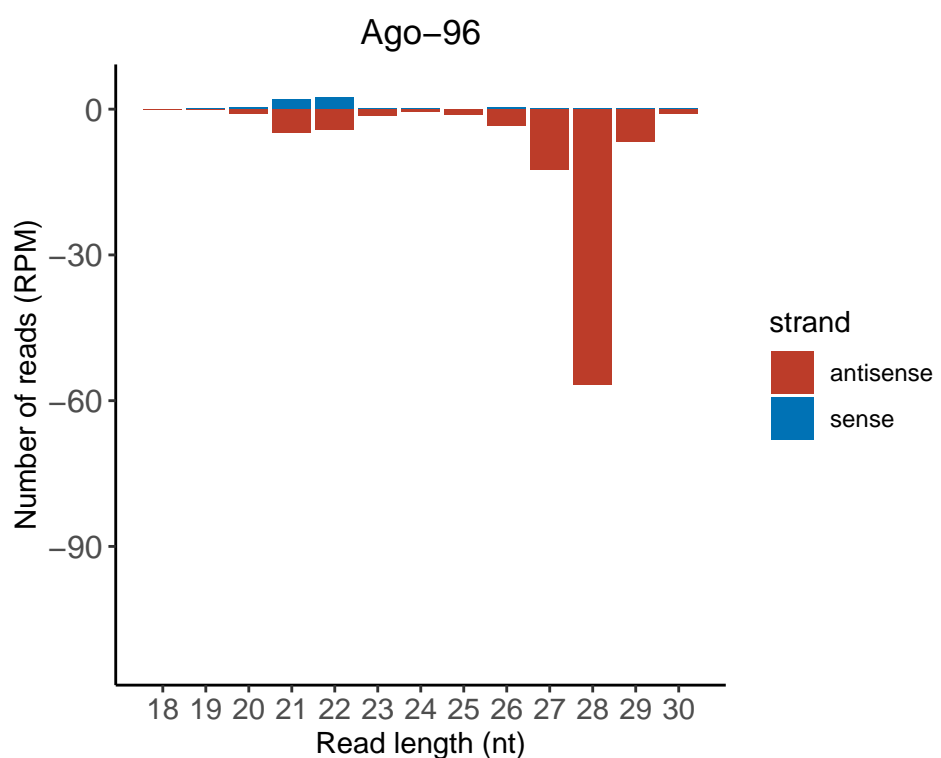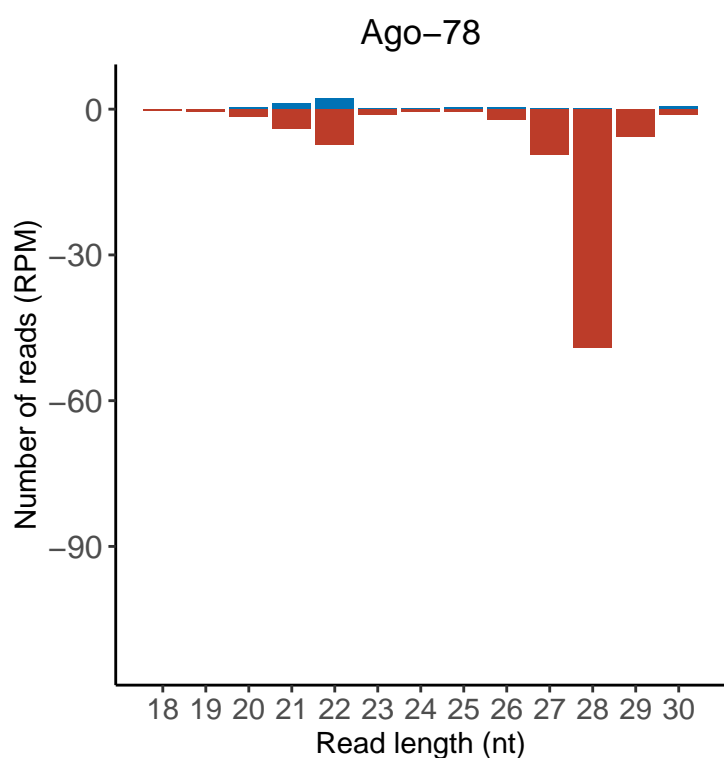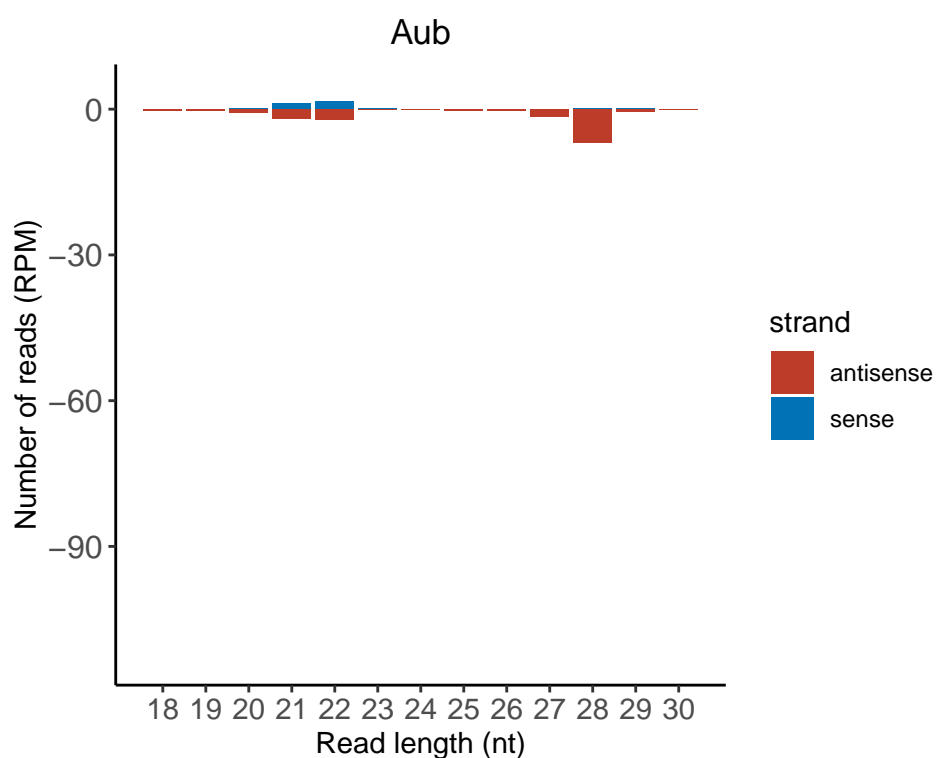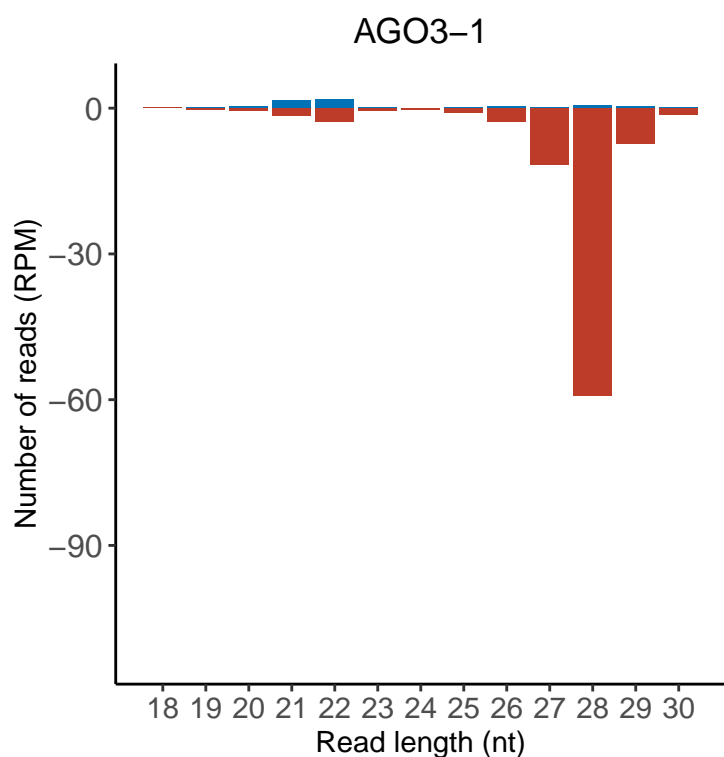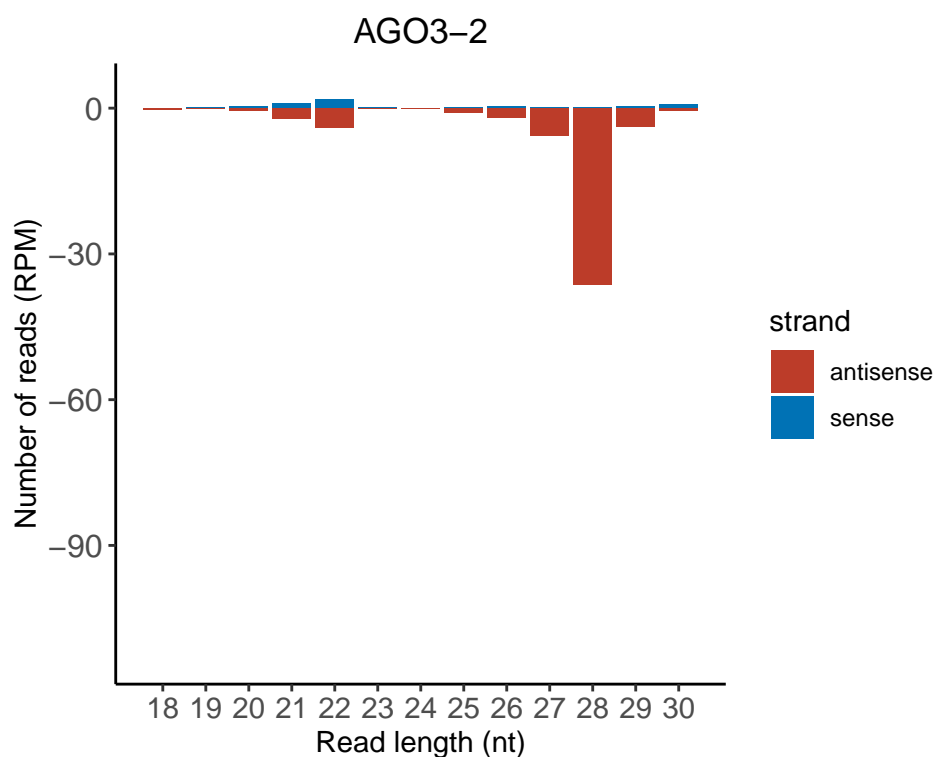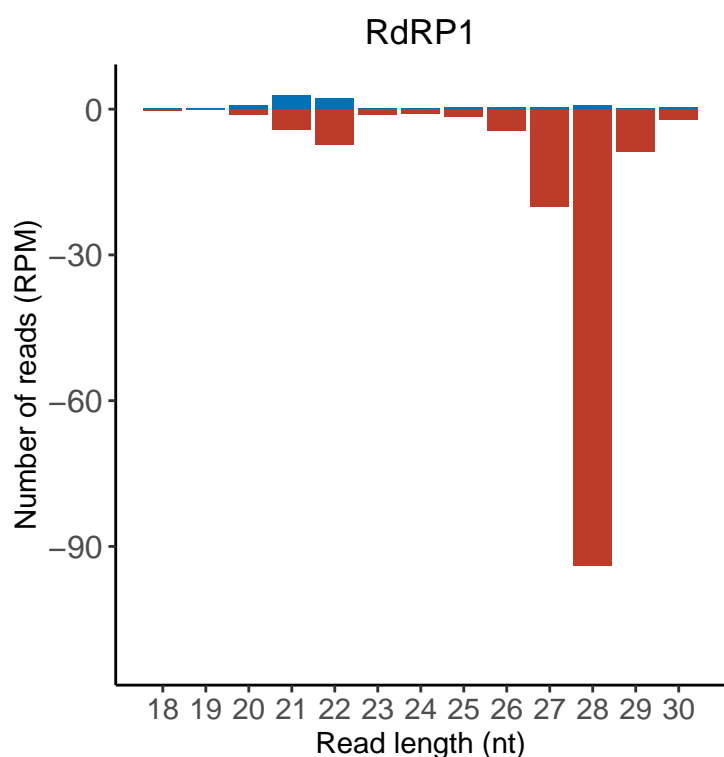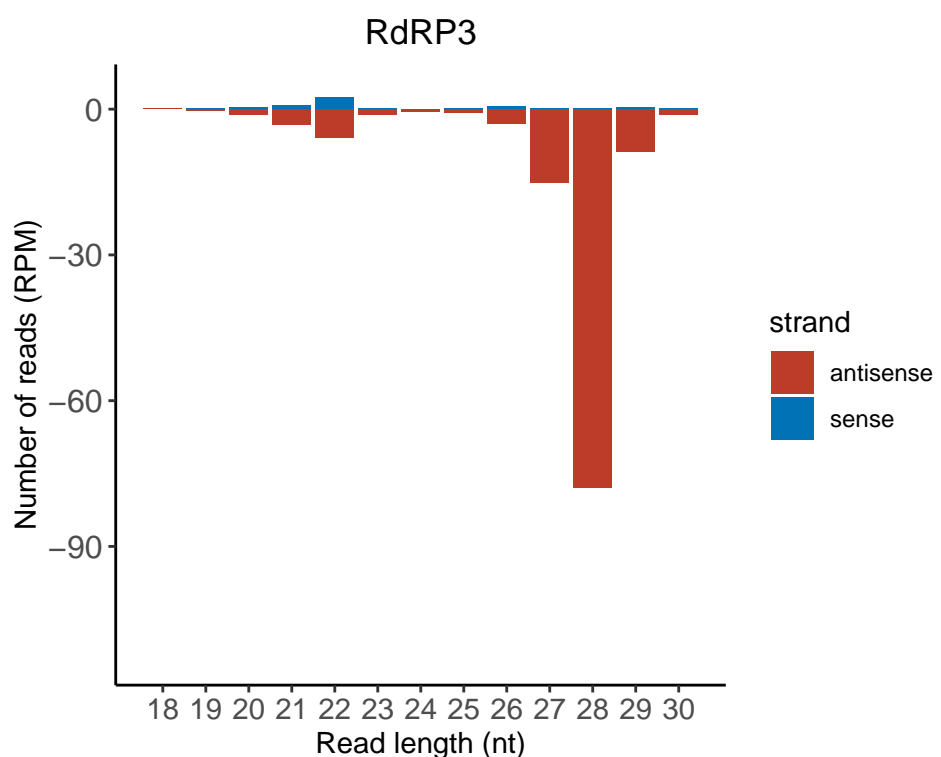

Supplement: S1 Data — On the Normalized and Relative levels pages, normalized read counts (RPM) and relative levels compared to the control (dsGFP) library were used. TEs with more than 50RPM and Coding Genes with more than 3.5RPM on average in KD libraries are included in this website. For viral sequences, reference sequences were generated by assembling sRNA sequences (See the Analysis of sRNAseq data section in Materials and methods.) and size distributions of sRNA reads mapped to each contig are shown. Reads were first grouped by their 5’ nucleotides, and reads of each length were counted. Full tables including TEs and Coding genes with fewer reads are in the “FullTable” folder. (ZIP) [file pone.0281195.s017.zip › SupplementaryData/Links/PDFs/Motif:rnd-6_family-2543TE_lengthdistribution.pdf]

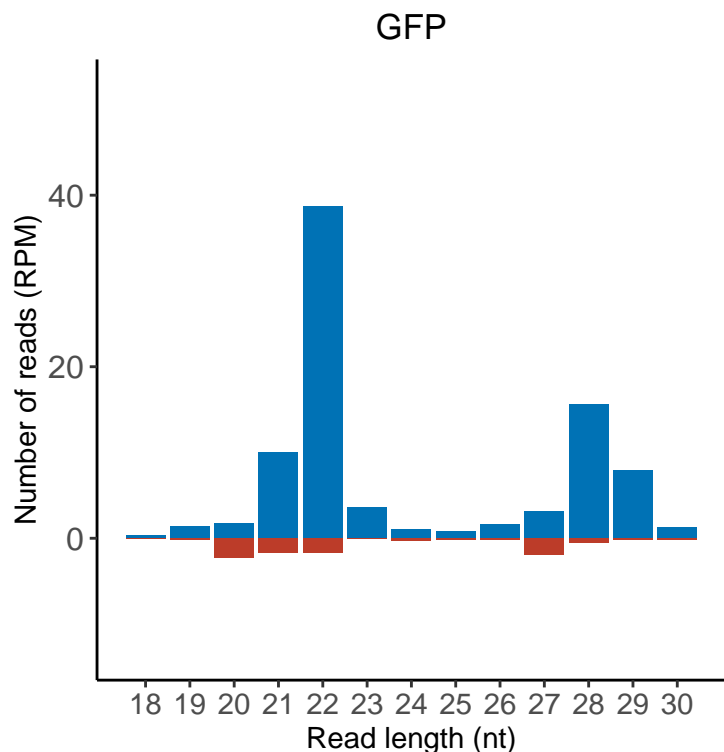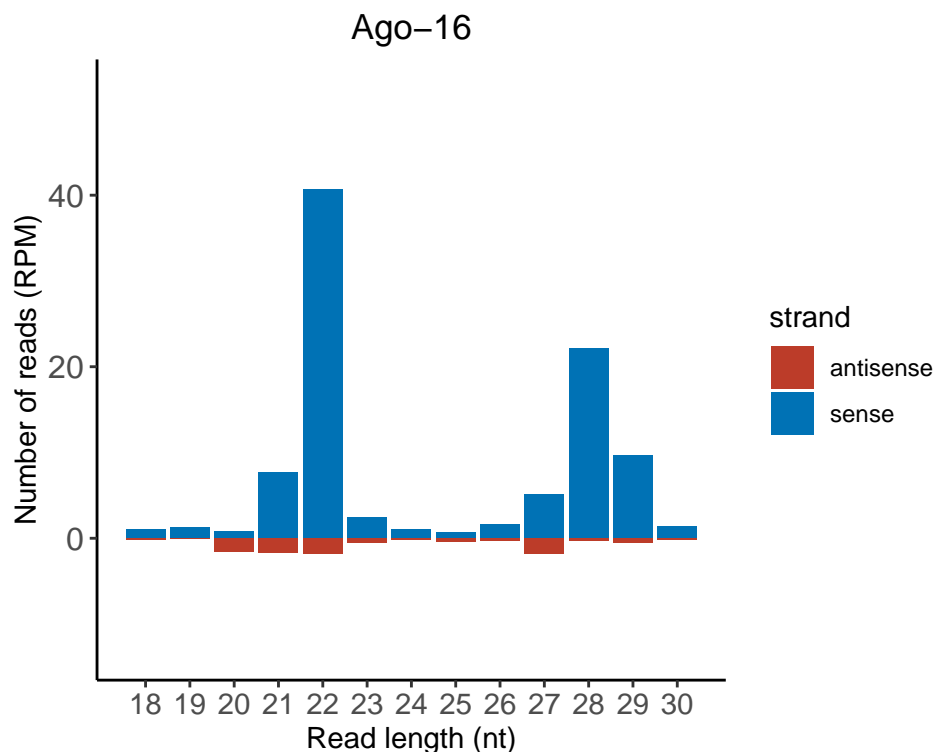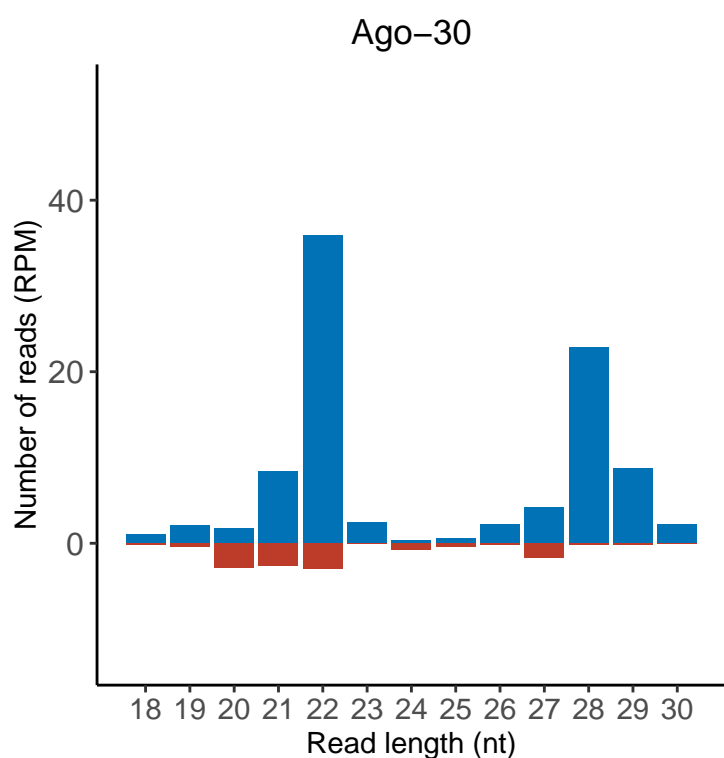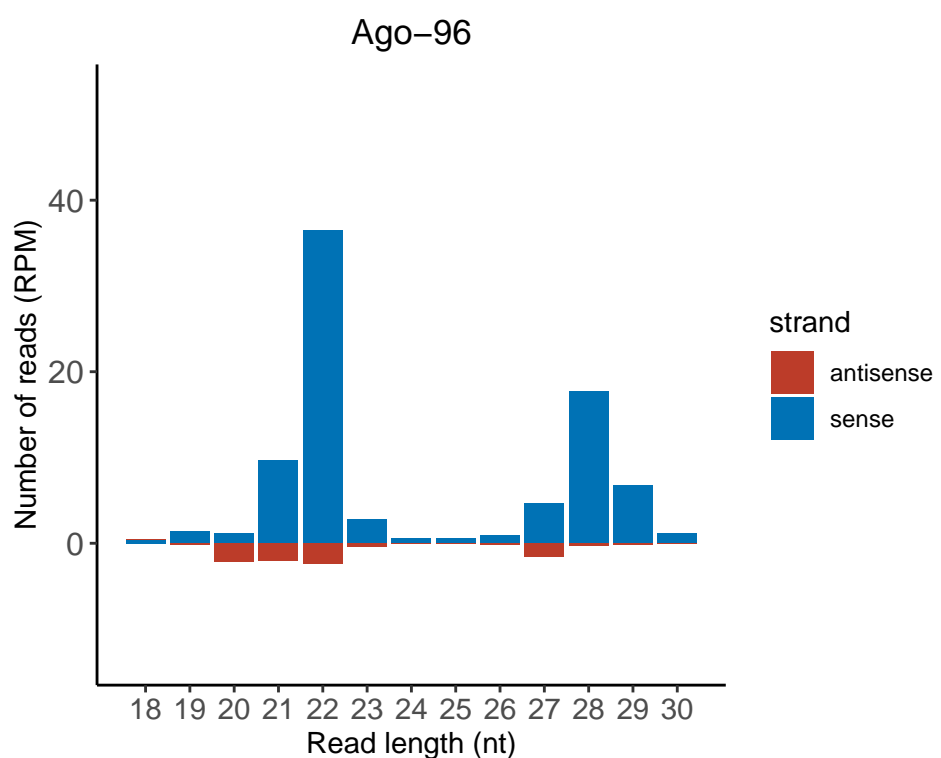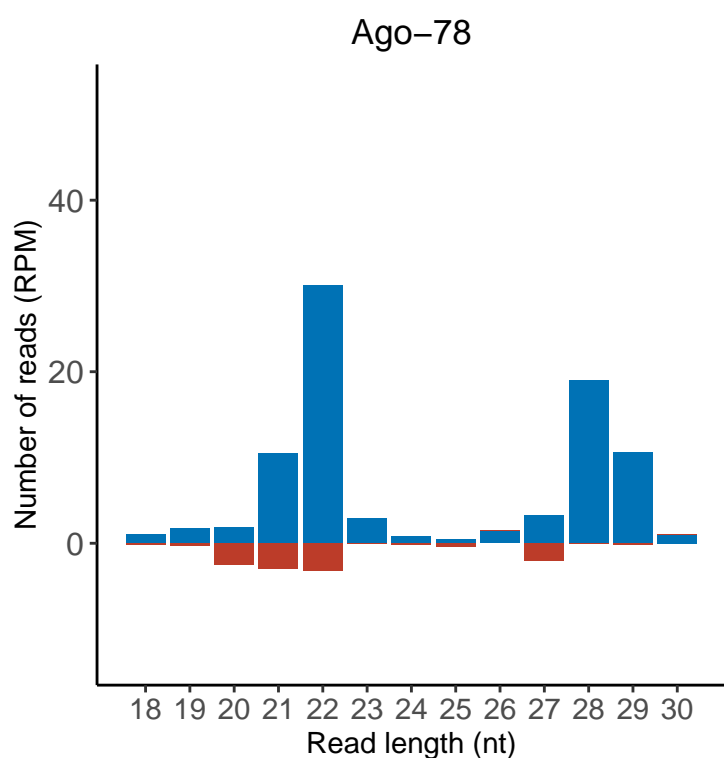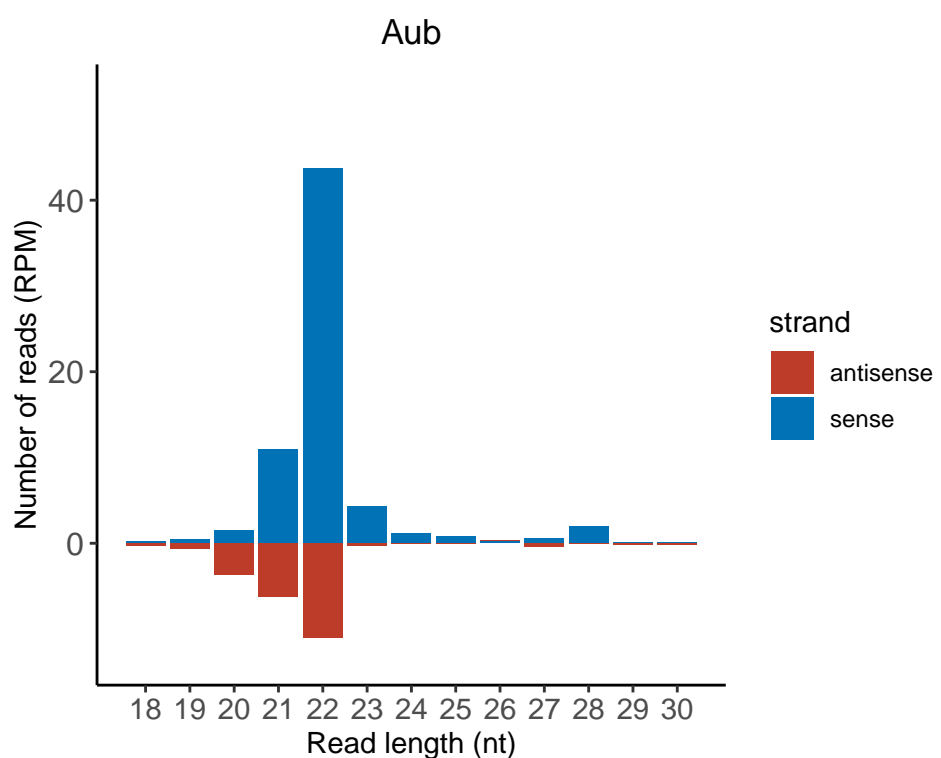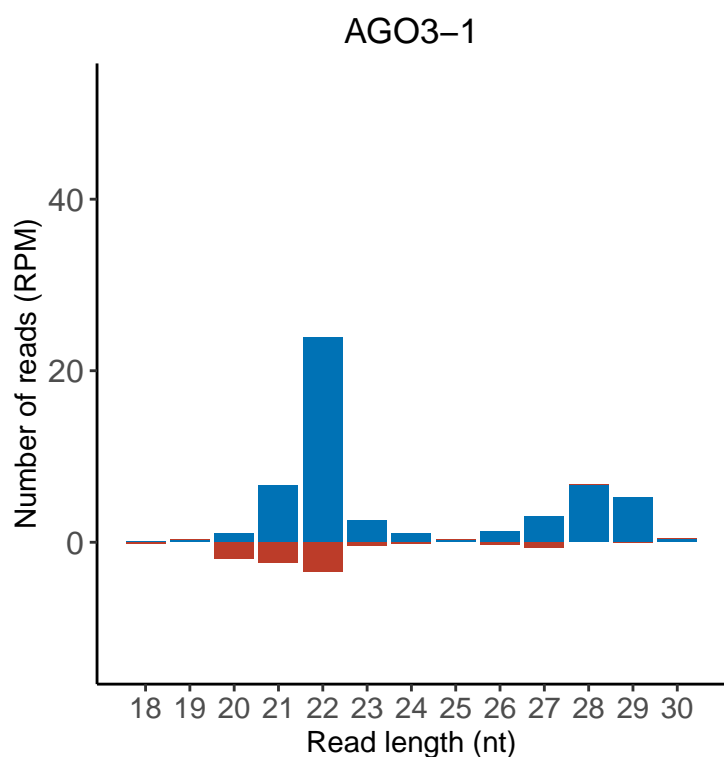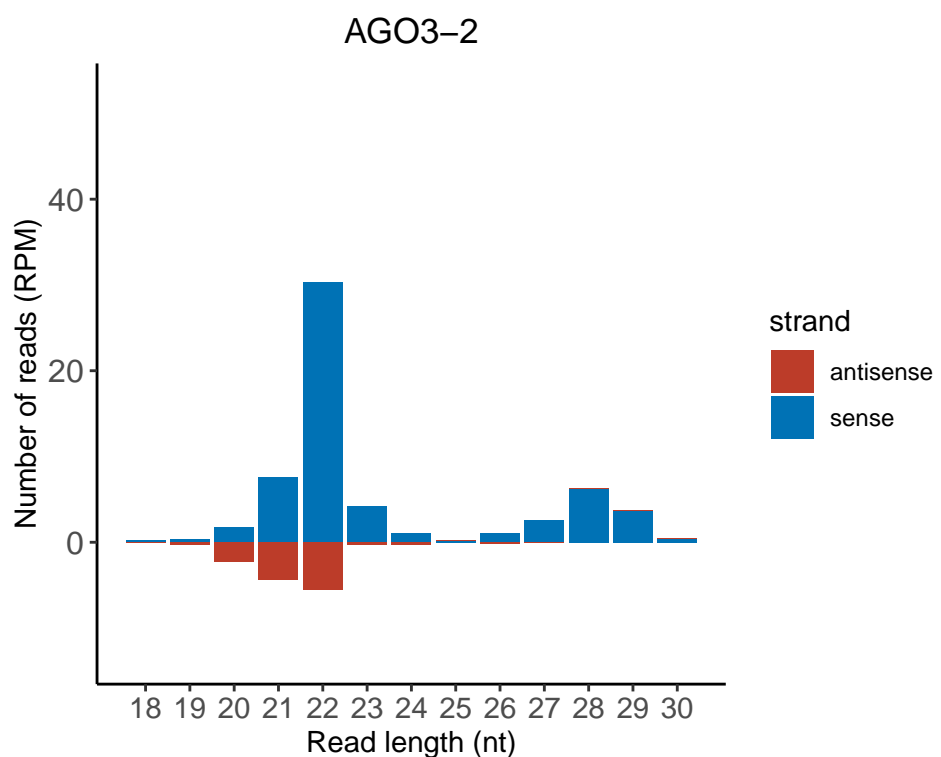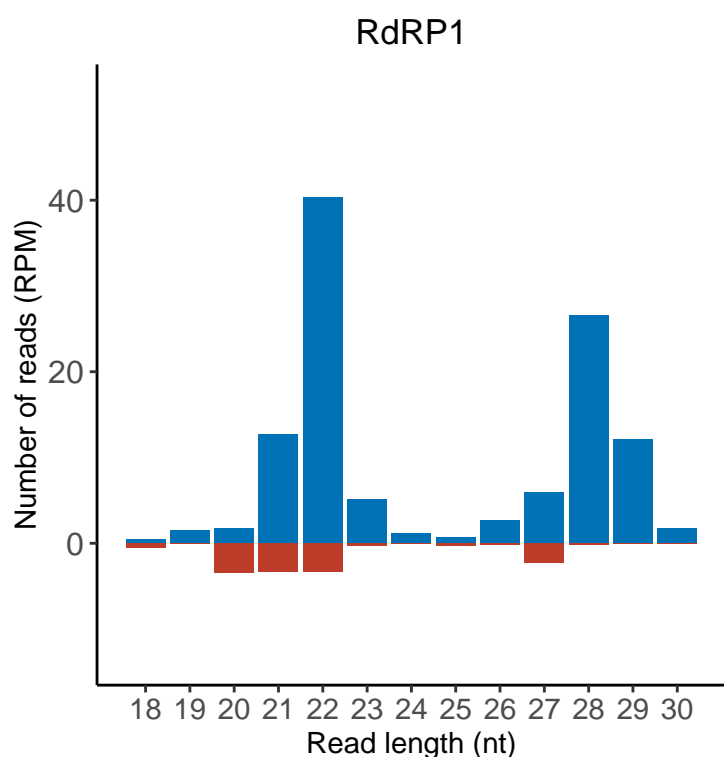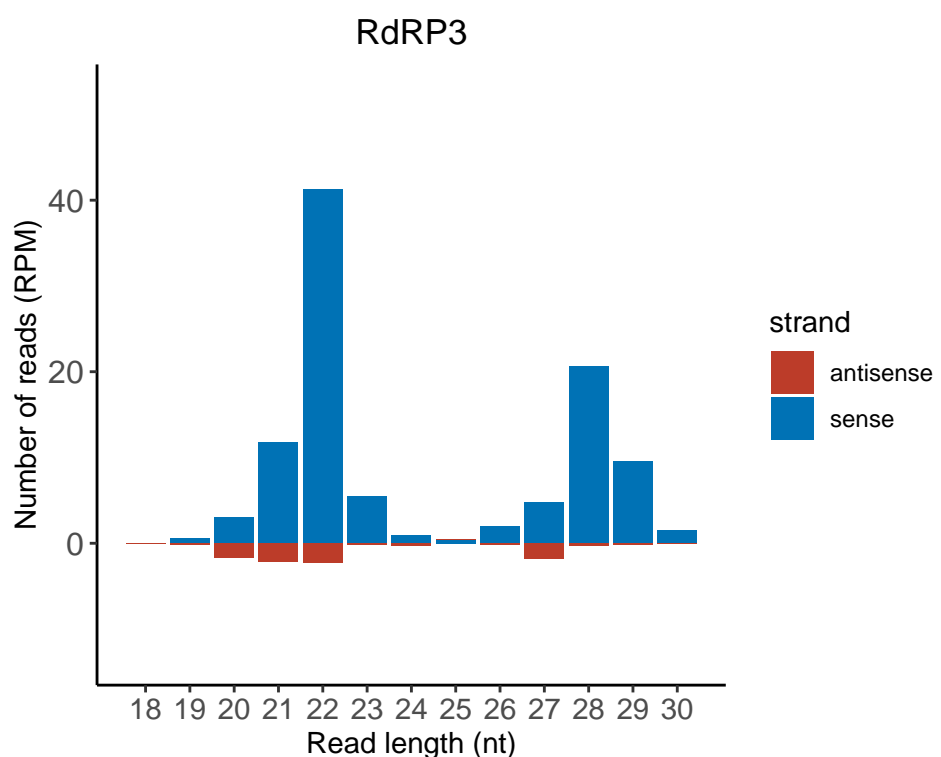

Supplement: S1 Data — On the Normalized and Relative levels pages, normalized read counts (RPM) and relative levels compared to the control (dsGFP) library were used. TEs with more than 50RPM and Coding Genes with more than 3.5RPM on average in KD libraries are included in this website. For viral sequences, reference sequences were generated by assembling sRNA sequences (See the Analysis of sRNAseq data section in Materials and methods.) and size distributions of sRNA reads mapped to each contig are shown. Reads were first grouped by their 5’ nucleotides, and reads of each length were counted. Full tables including TEs and Coding genes with fewer reads are in the “FullTable” folder. (ZIP) [file pone.0281195.s017.zip › SupplementaryData/Links/PDFs/Motif:rnd-6_family-5252TE_lengthdistribution.pdf]

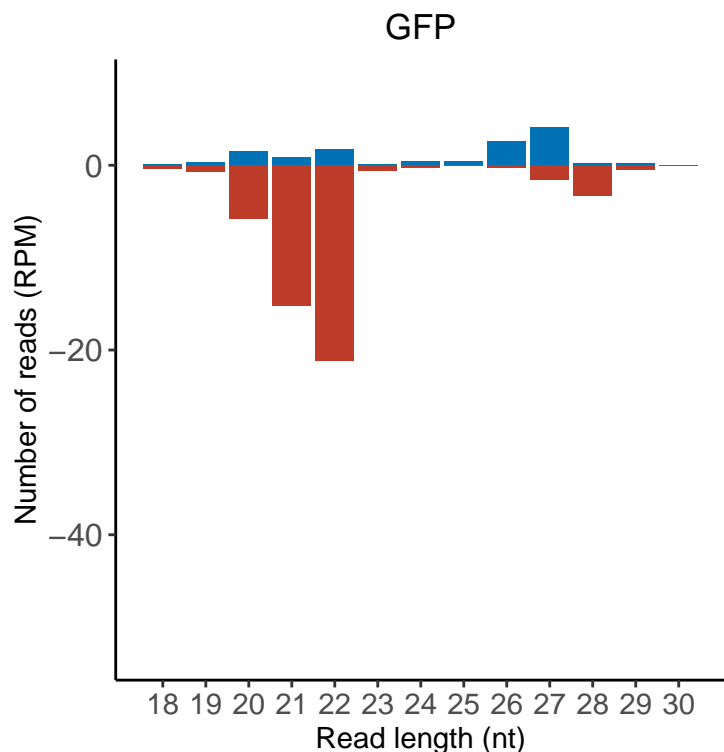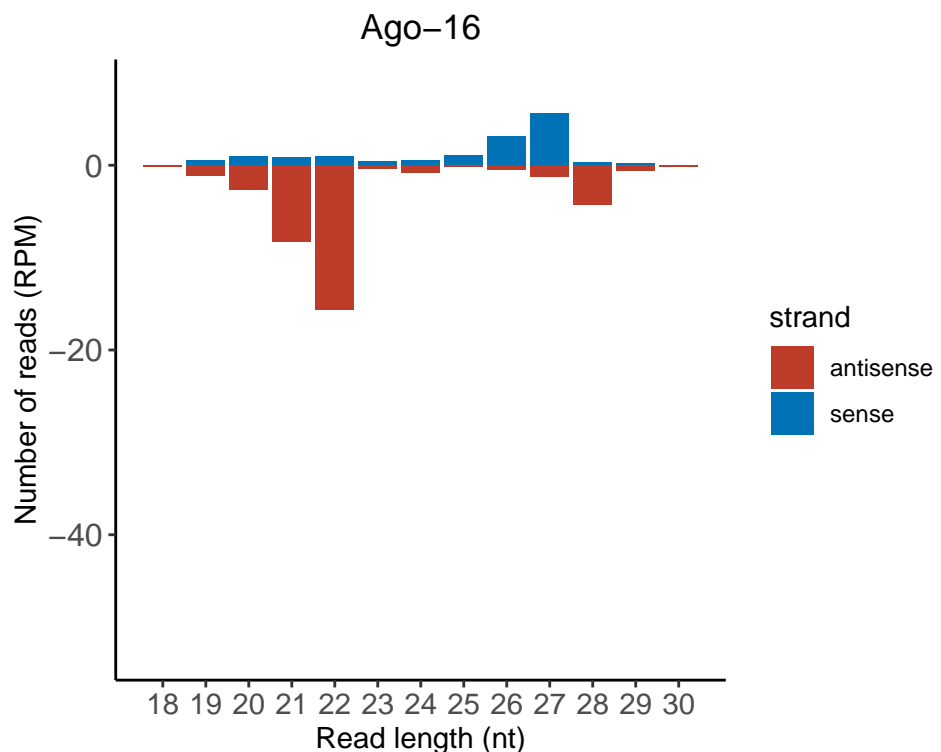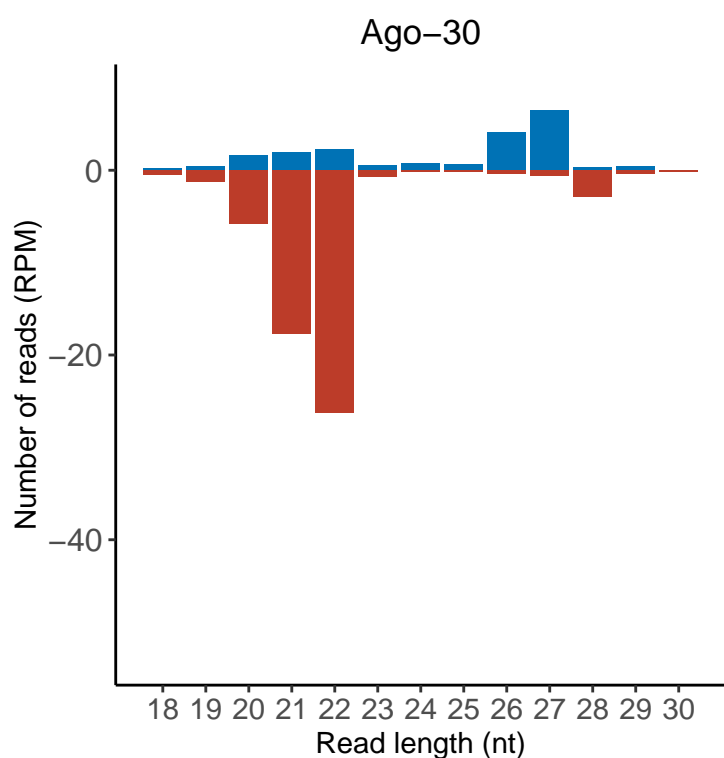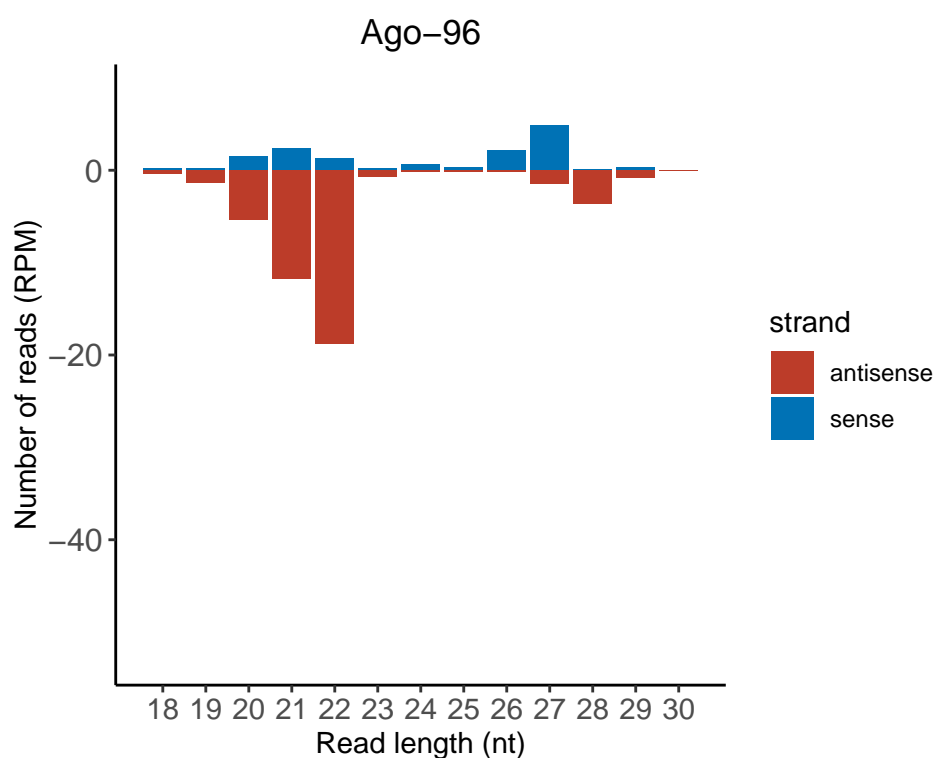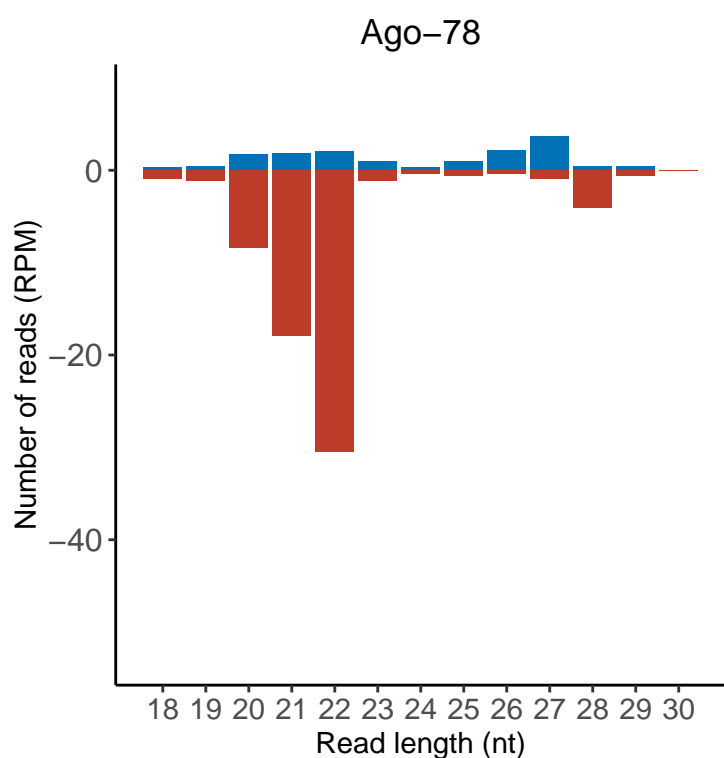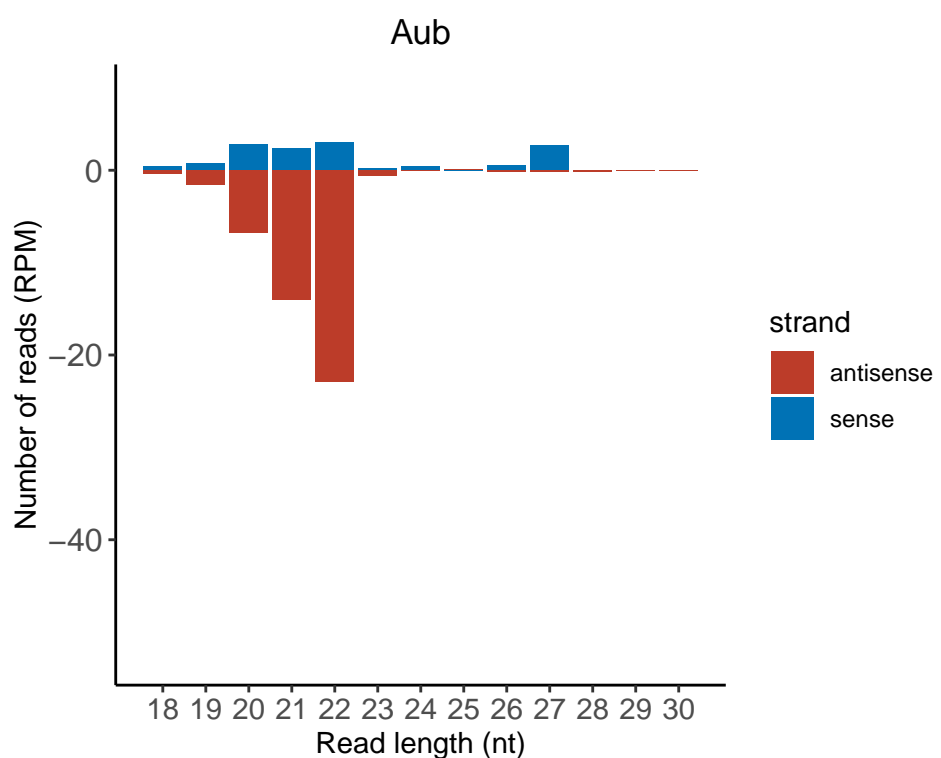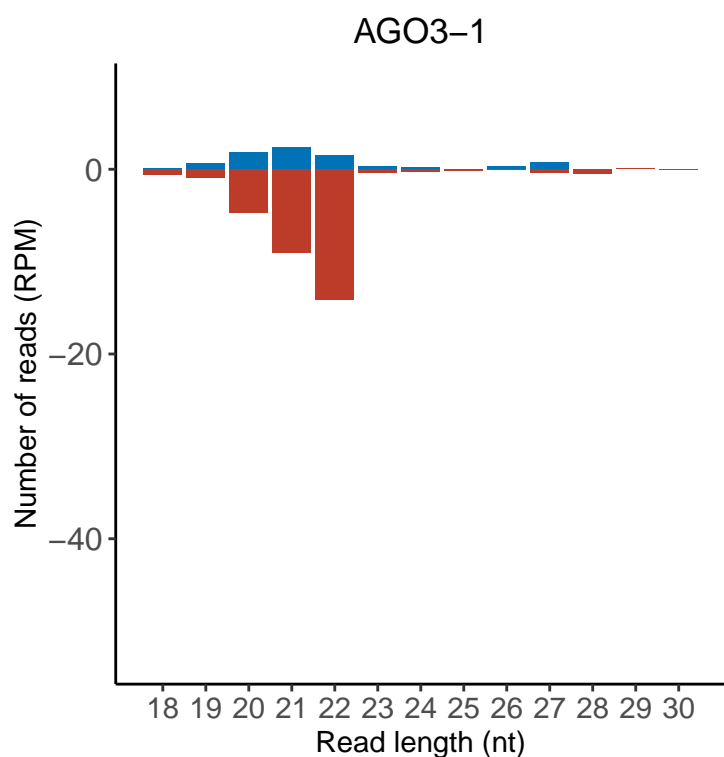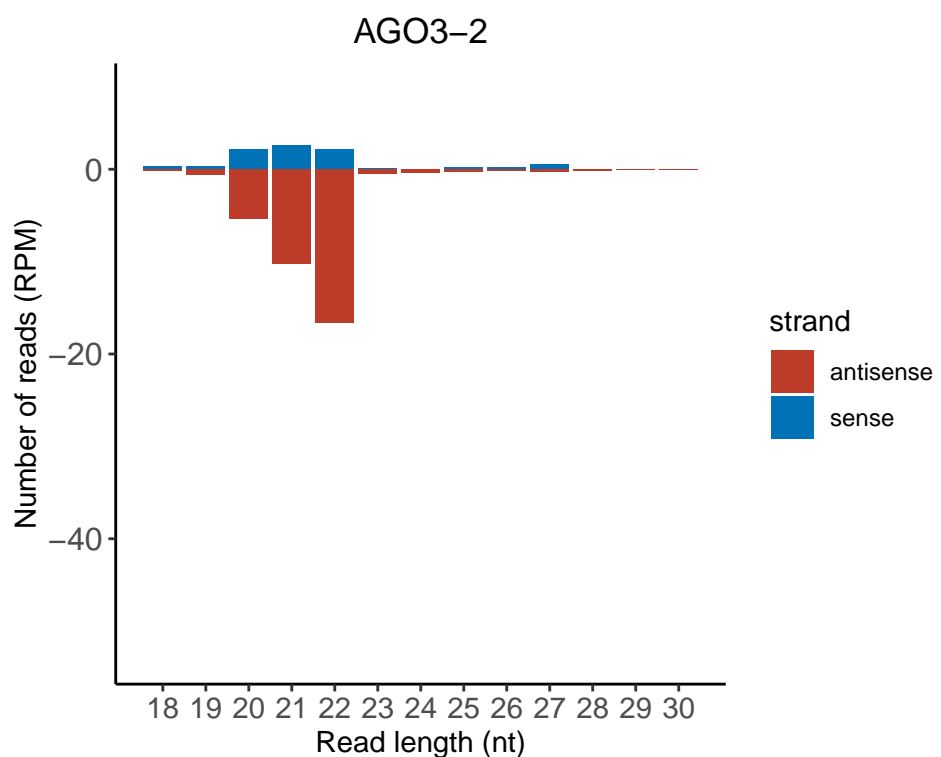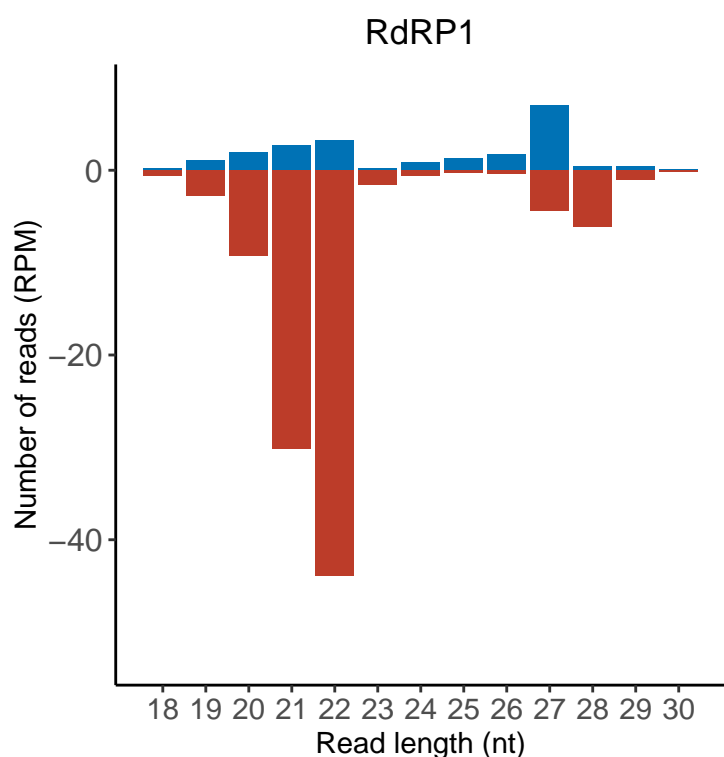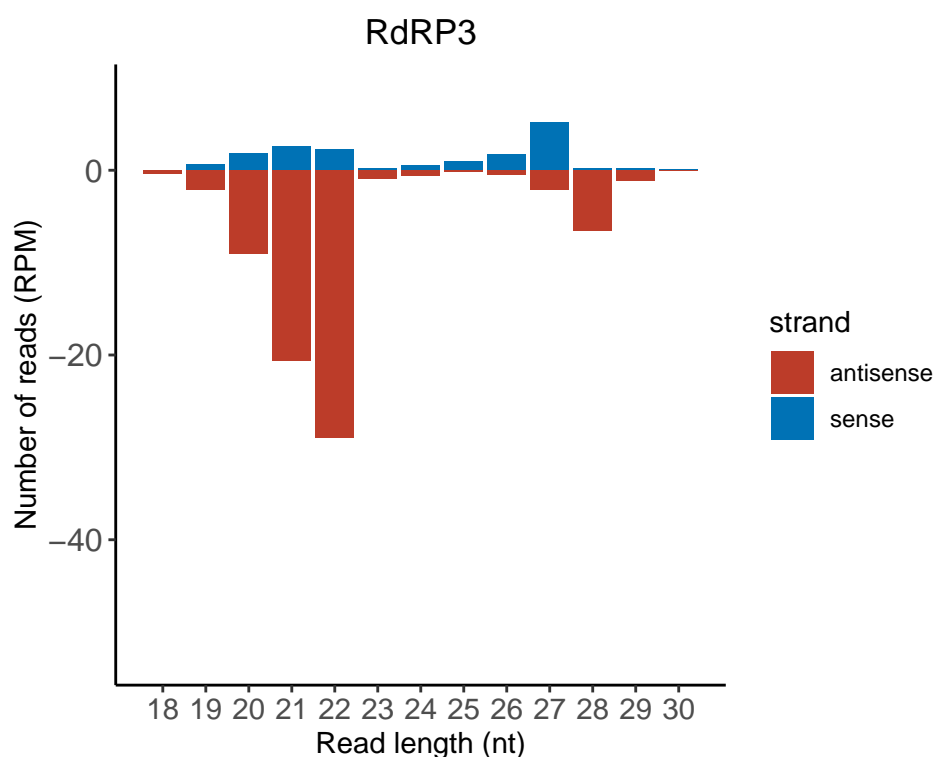

Supplement: S1 Data — On the Normalized and Relative levels pages, normalized read counts (RPM) and relative levels compared to the control (dsGFP) library were used. TEs with more than 50RPM and Coding Genes with more than 3.5RPM on average in KD libraries are included in this website. For viral sequences, reference sequences were generated by assembling sRNA sequences (See the Analysis of sRNAseq data section in Materials and methods.) and size distributions of sRNA reads mapped to each contig are shown. Reads were first grouped by their 5’ nucleotides, and reads of each length were counted. Full tables including TEs and Coding genes with fewer reads are in the “FullTable” folder. (ZIP) [file pone.0281195.s017.zip › SupplementaryData/Links/PDFs/Motif:rnd-6_family-3044TE_lengthdistribution.pdf]

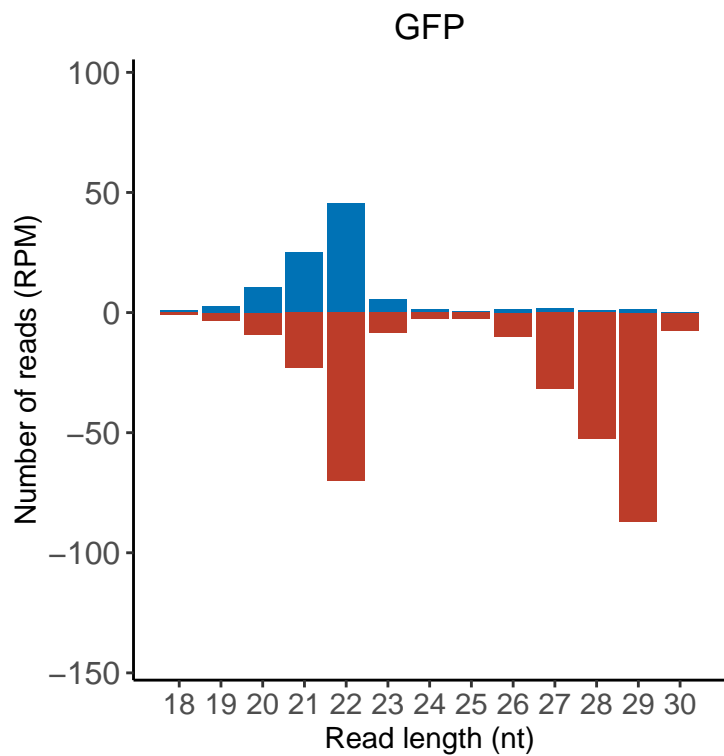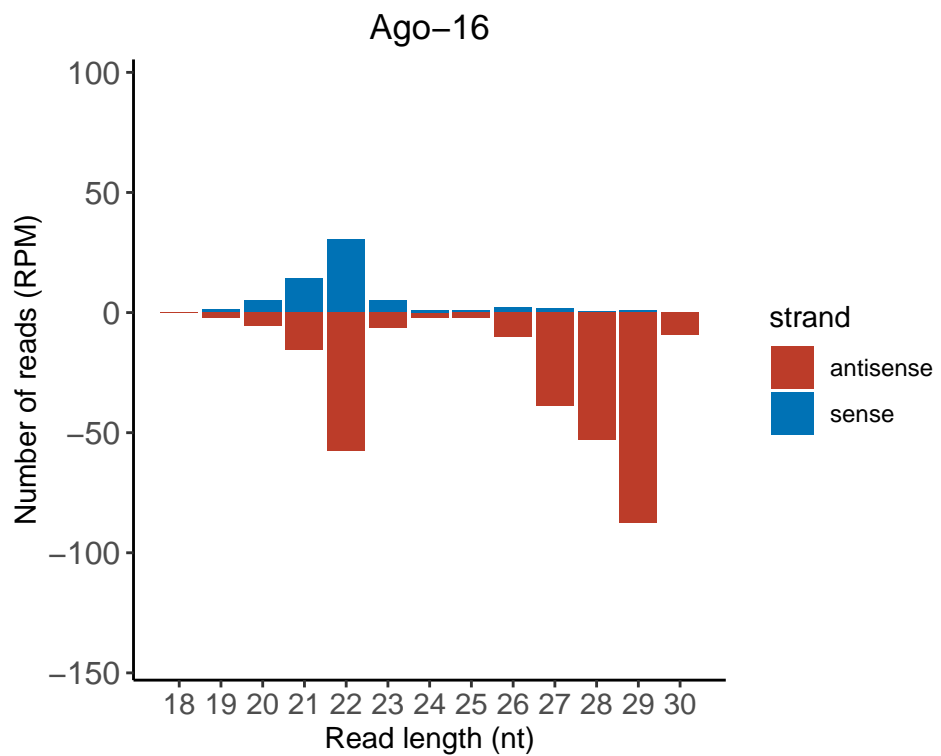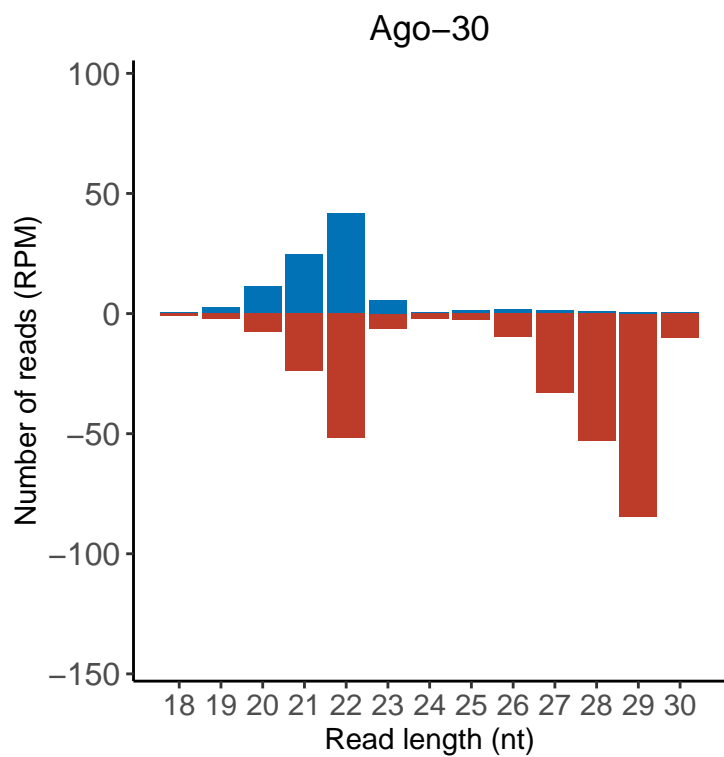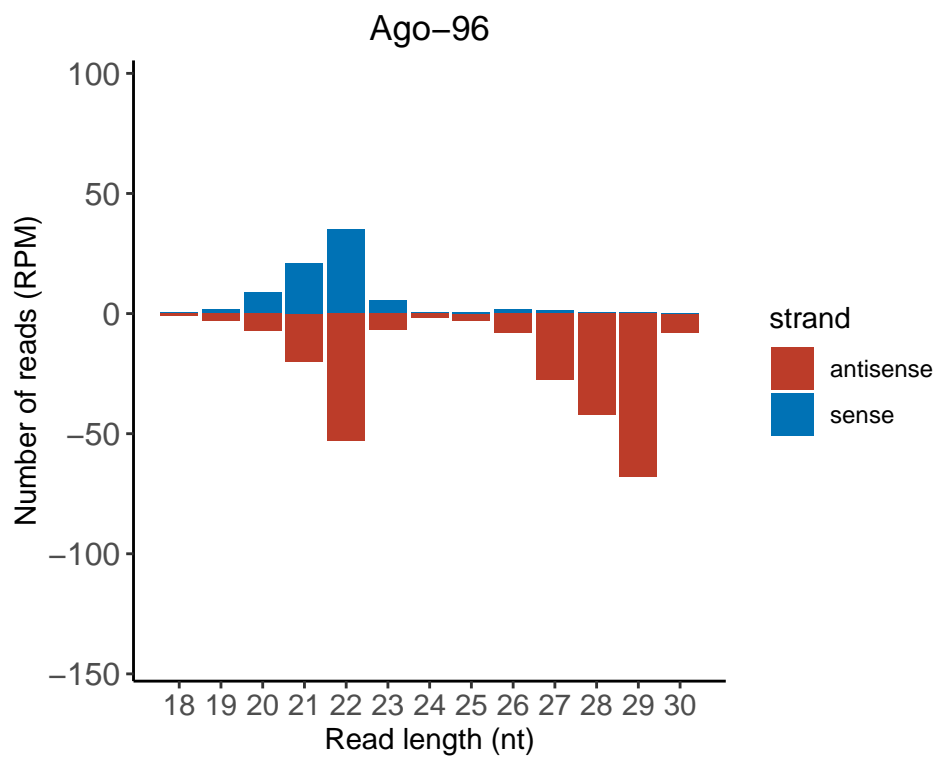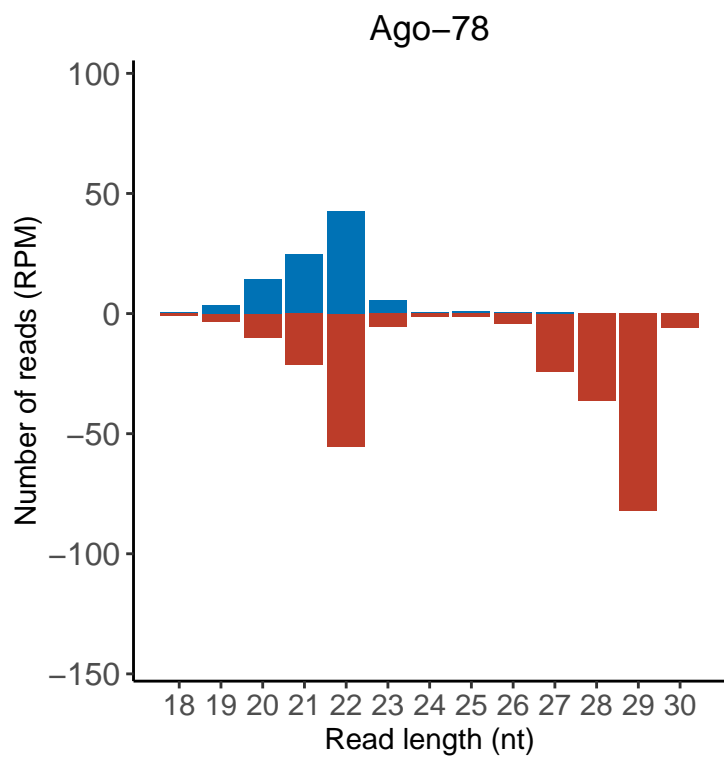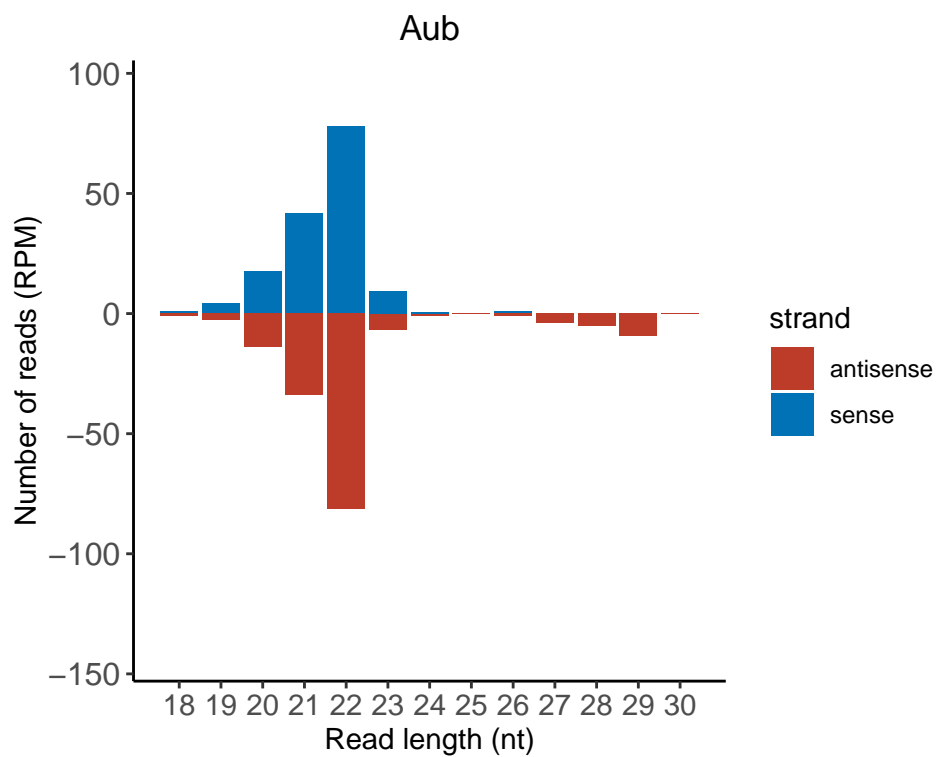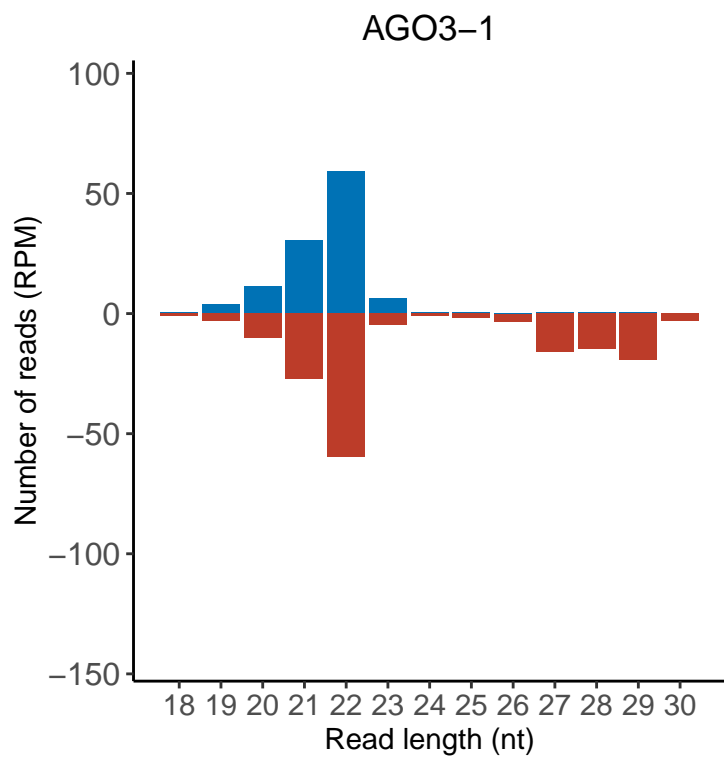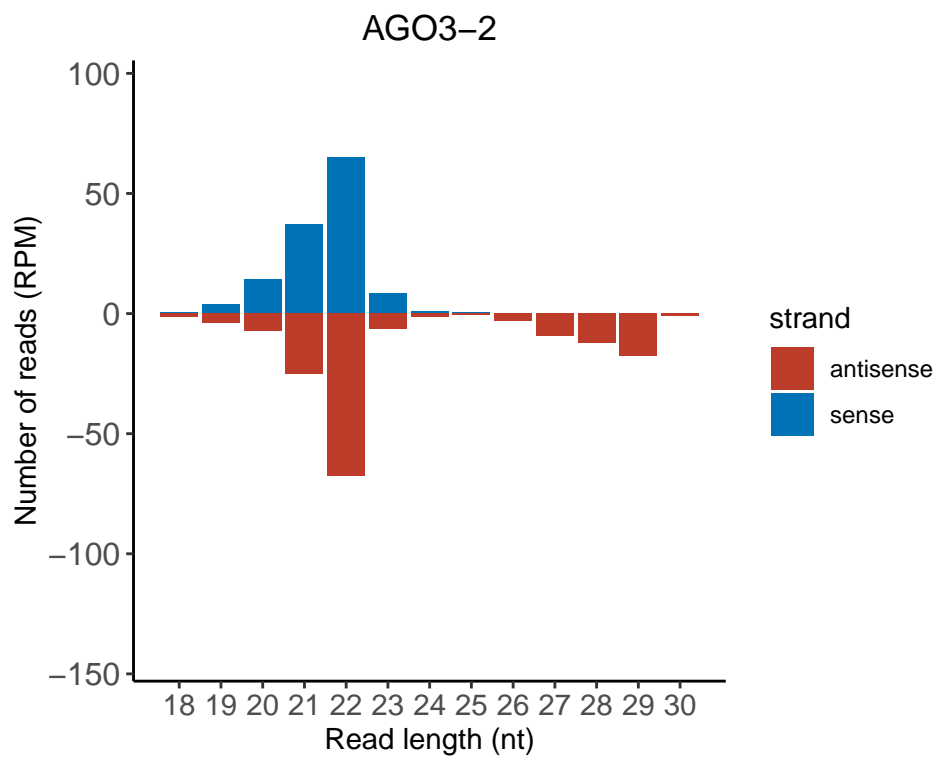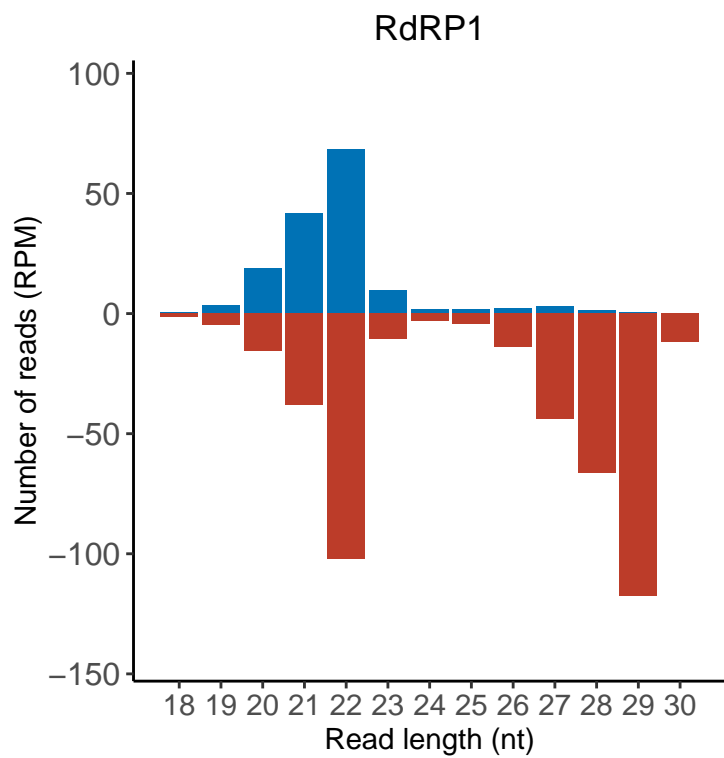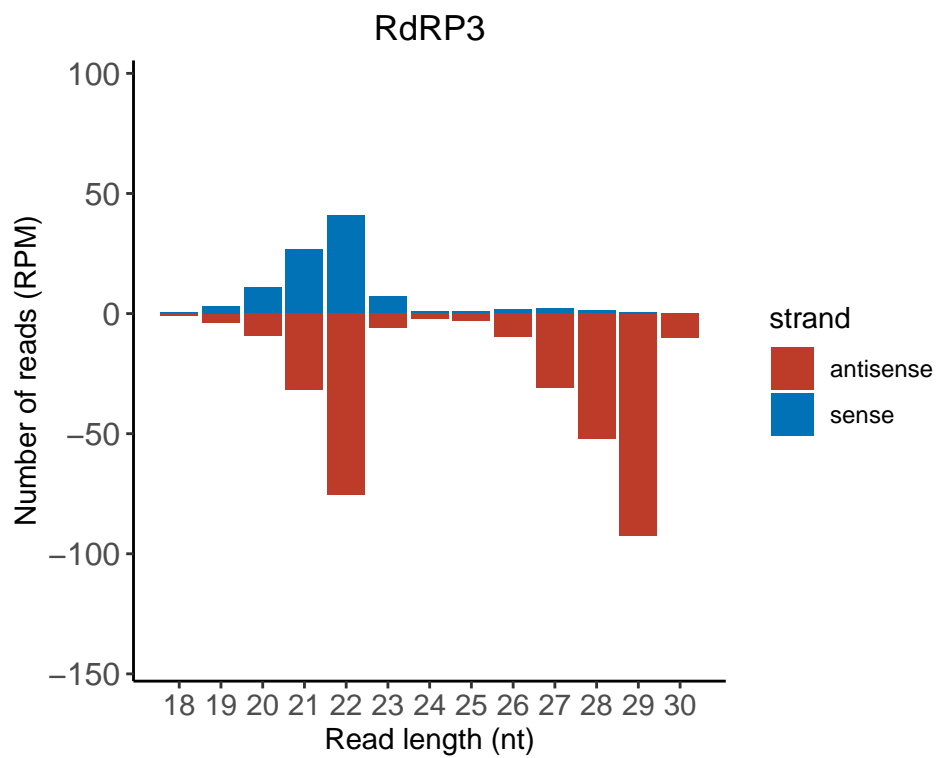

Supplement: S1 Data — On the Normalized and Relative levels pages, normalized read counts (RPM) and relative levels compared to the control (dsGFP) library were used. TEs with more than 50RPM and Coding Genes with more than 3.5RPM on average in KD libraries are included in this website. For viral sequences, reference sequences were generated by assembling sRNA sequences (See the Analysis of sRNAseq data section in Materials and methods.) and size distributions of sRNA reads mapped to each contig are shown. Reads were first grouped by their 5’ nucleotides, and reads of each length were counted. Full tables including TEs and Coding genes with fewer reads are in the “FullTable” folder. (ZIP) [file pone.0281195.s017.zip › SupplementaryData/Links/PDFs/Motif:rnd-6_family-75TE_lengthdistribution.pdf]

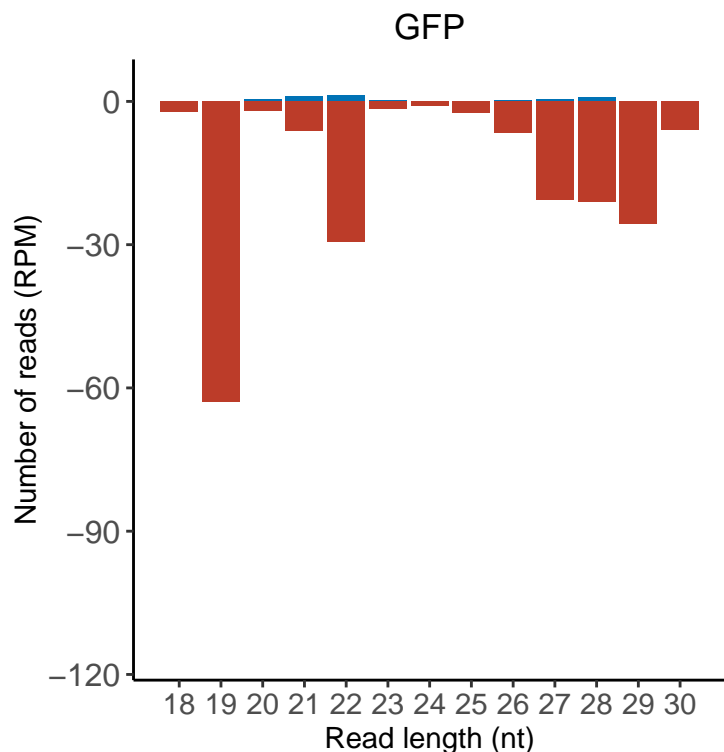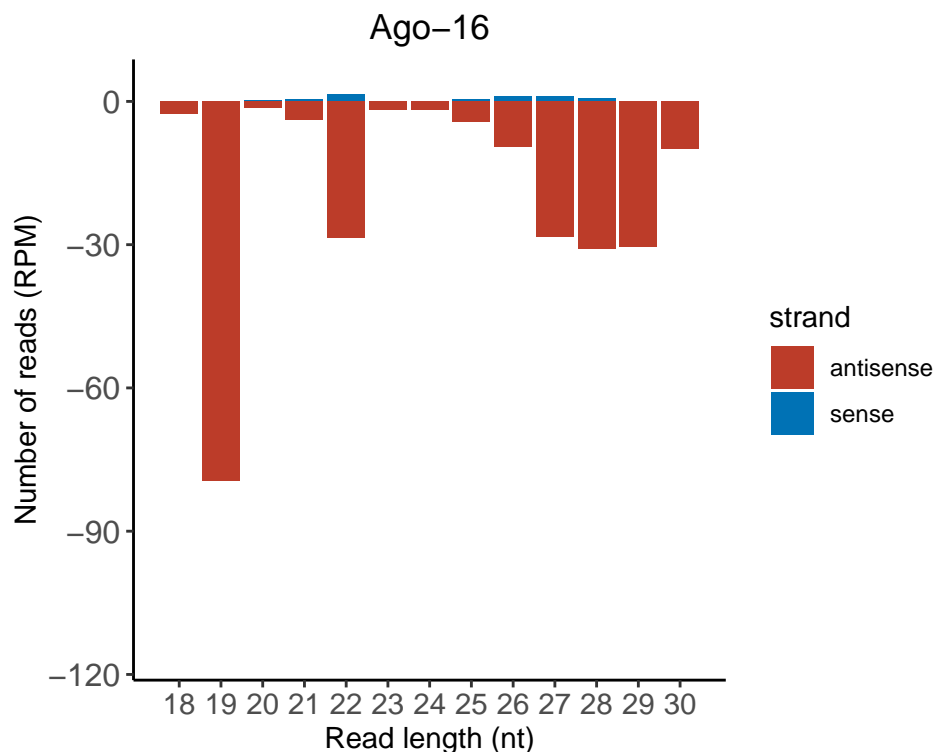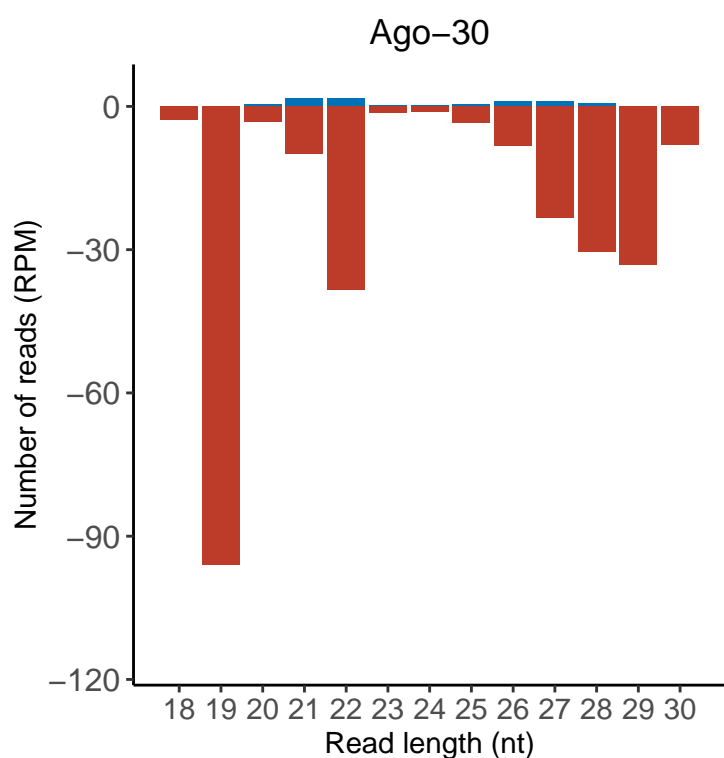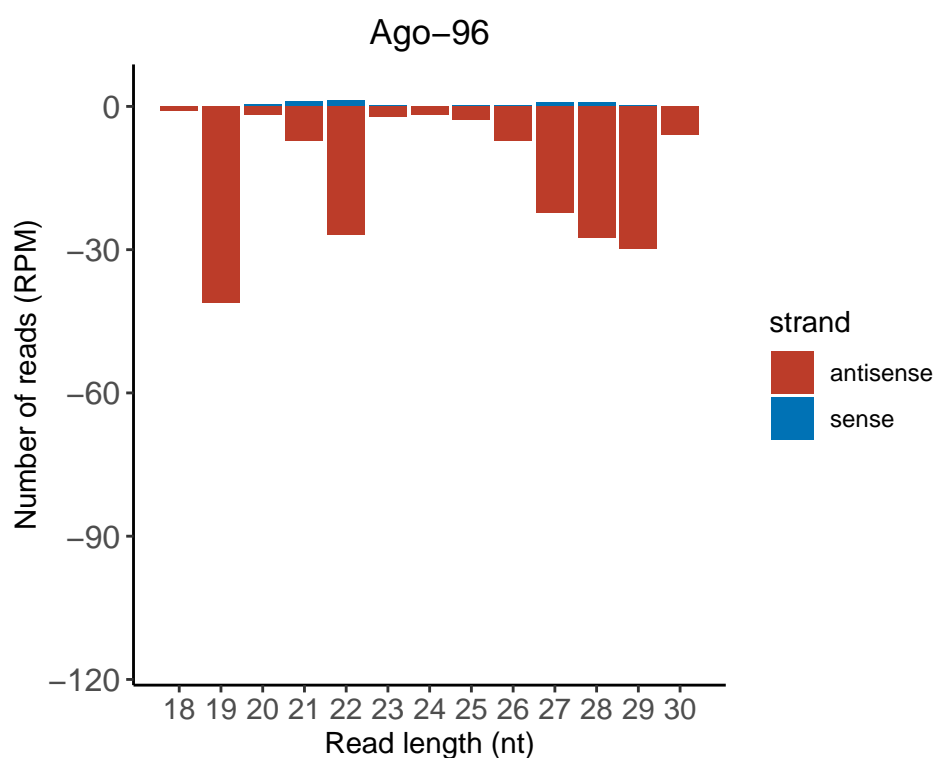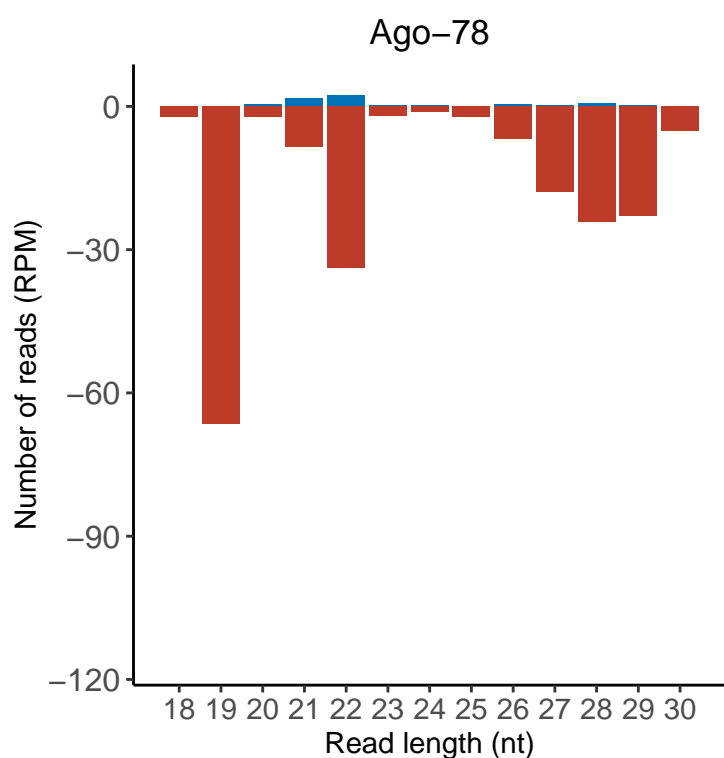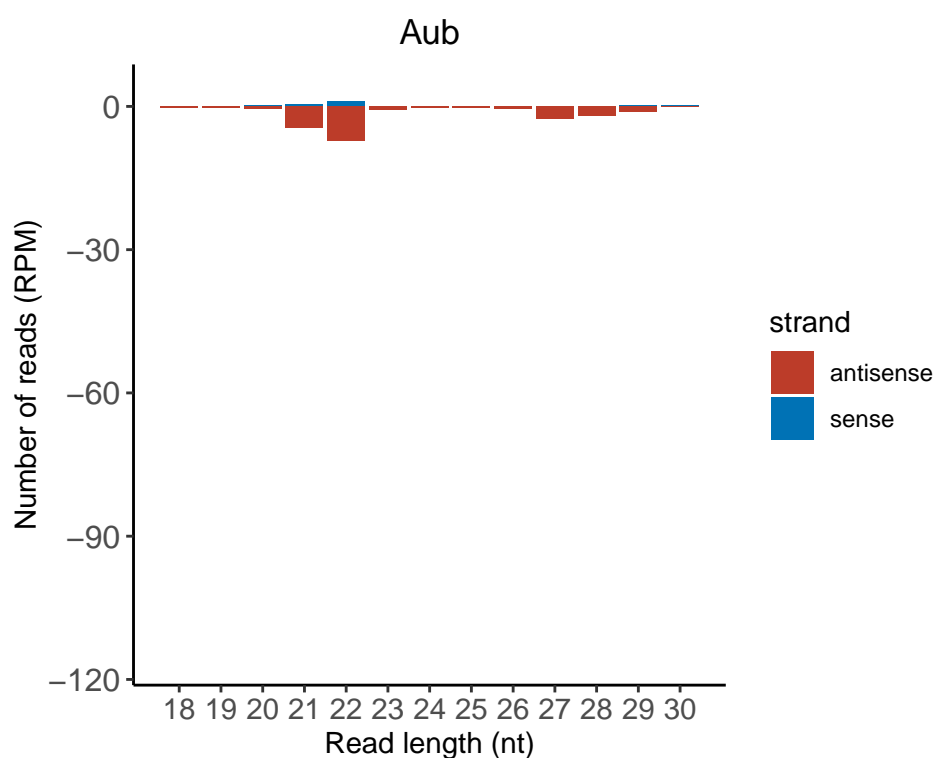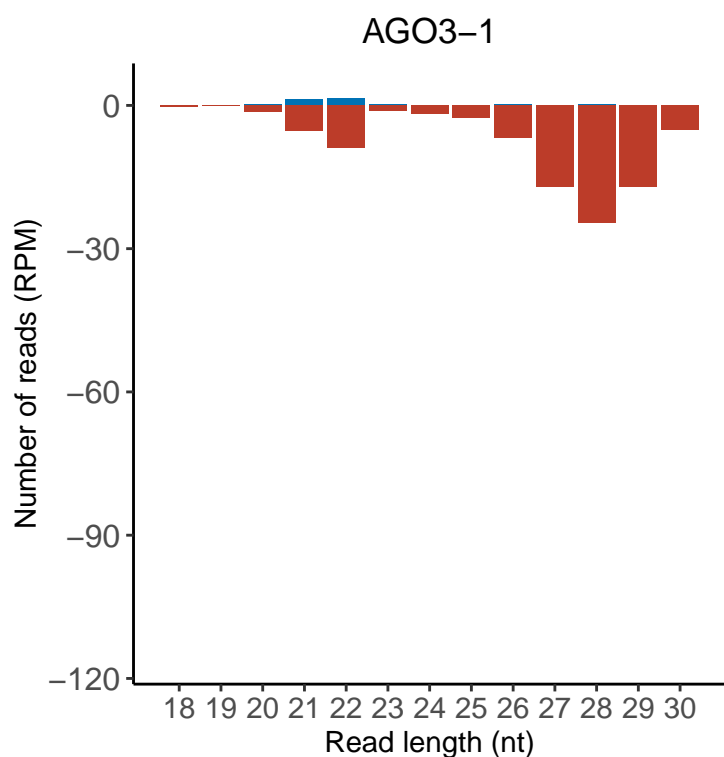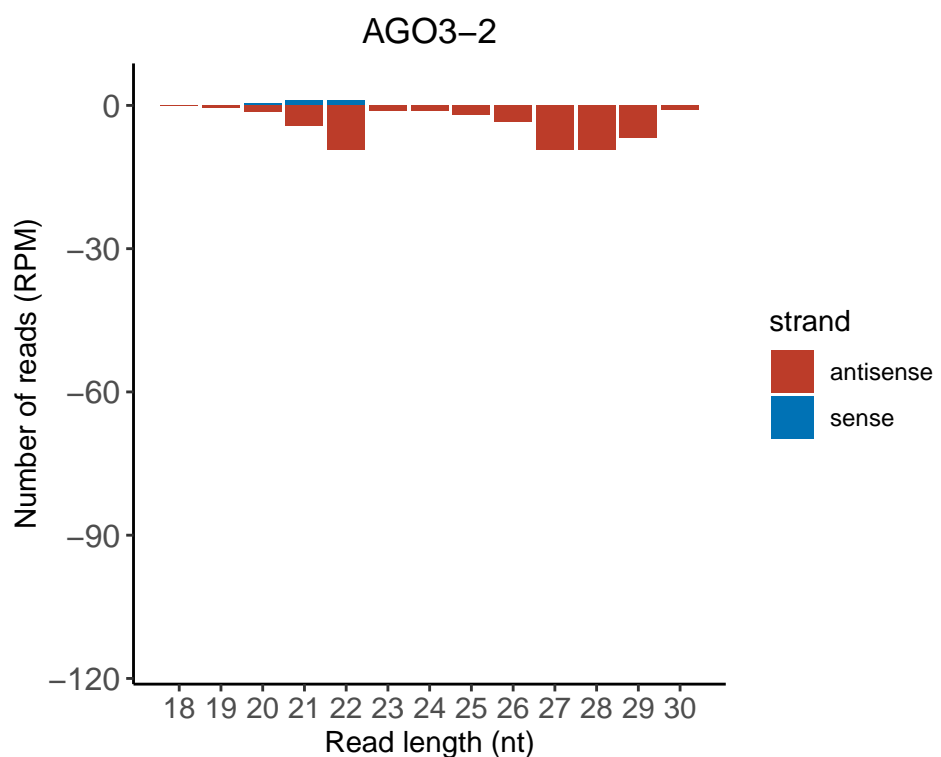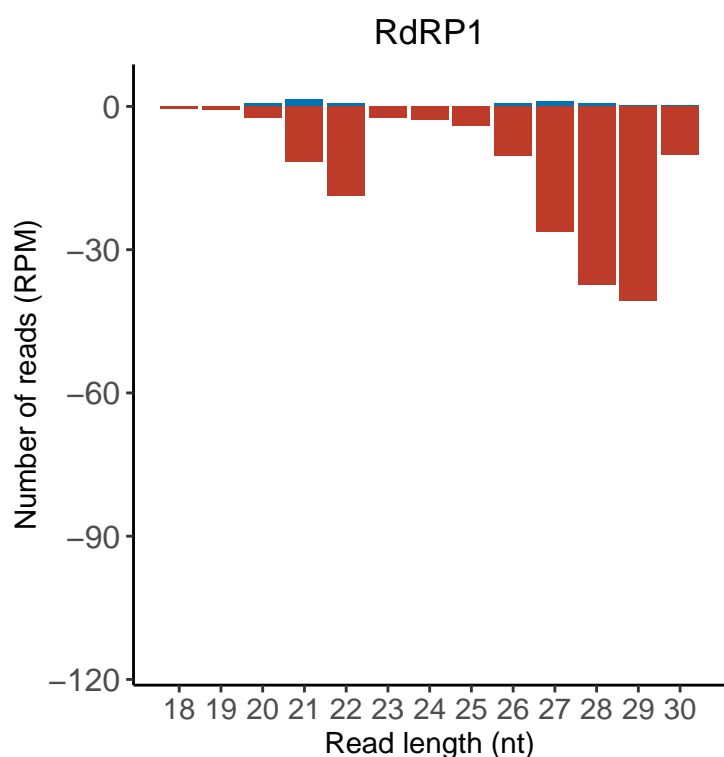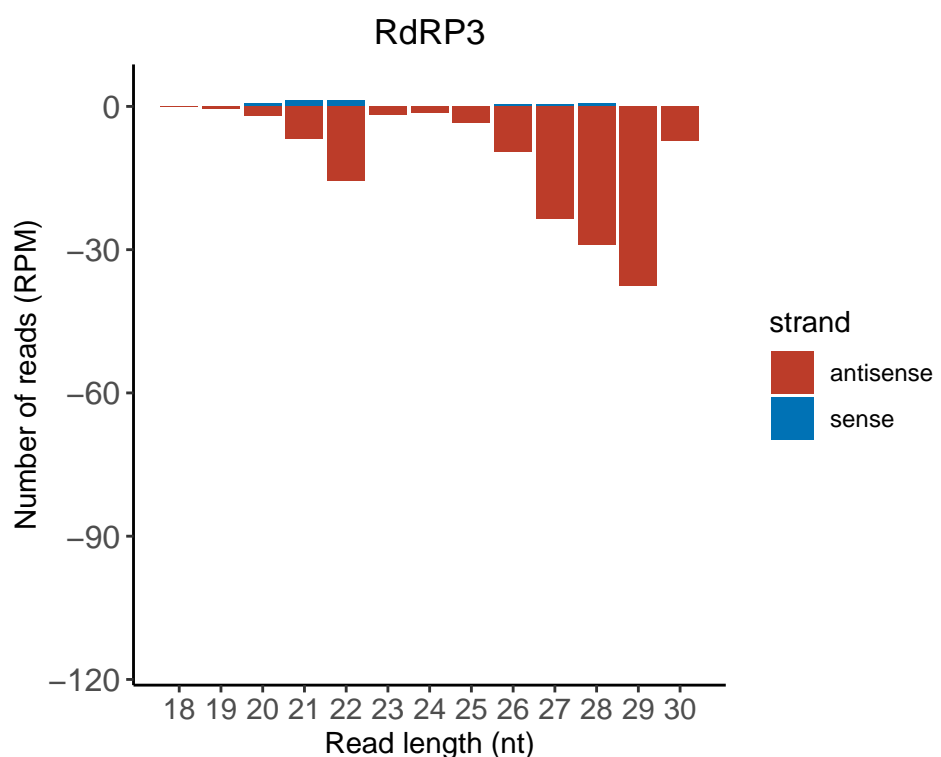

Supplement: S1 Data — On the Normalized and Relative levels pages, normalized read counts (RPM) and relative levels compared to the control (dsGFP) library were used. TEs with more than 50RPM and Coding Genes with more than 3.5RPM on average in KD libraries are included in this website. For viral sequences, reference sequences were generated by assembling sRNA sequences (See the Analysis of sRNAseq data section in Materials and methods.) and size distributions of sRNA reads mapped to each contig are shown. Reads were first grouped by their 5’ nucleotides, and reads of each length were counted. Full tables including TEs and Coding genes with fewer reads are in the “FullTable” folder. (ZIP) [file pone.0281195.s017.zip › SupplementaryData/Links/PDFs/Motif:rnd-6_family-5915TE_lengthdistribution.pdf]

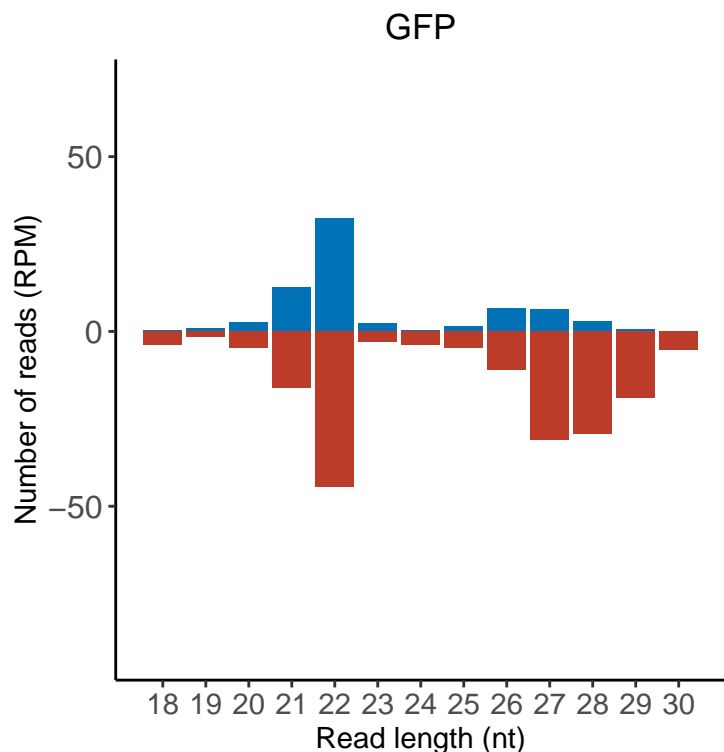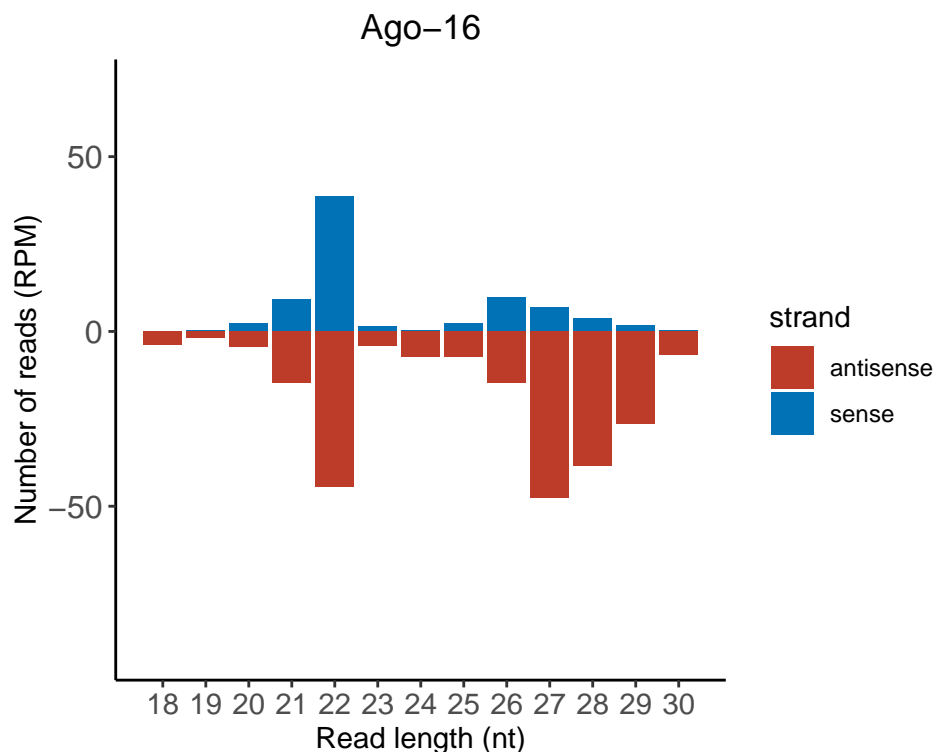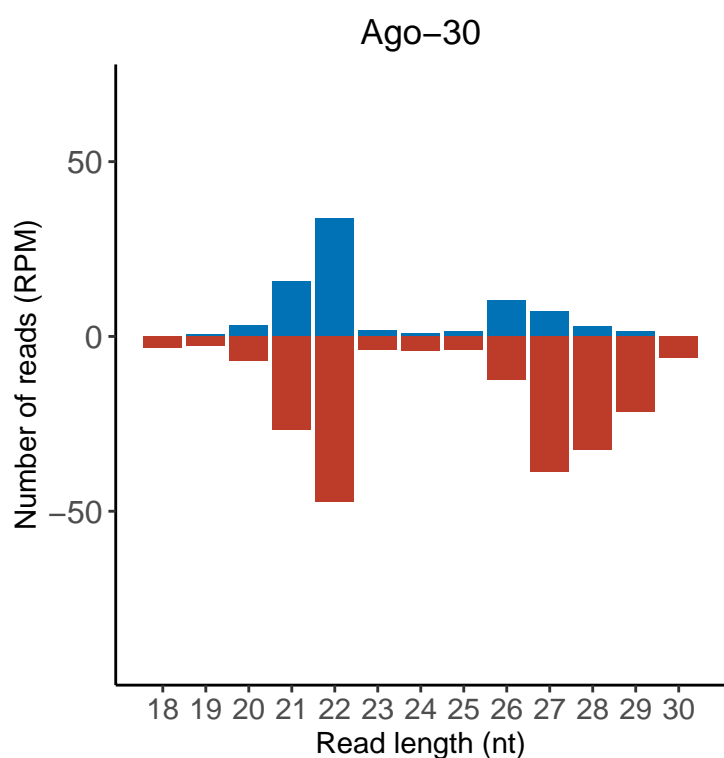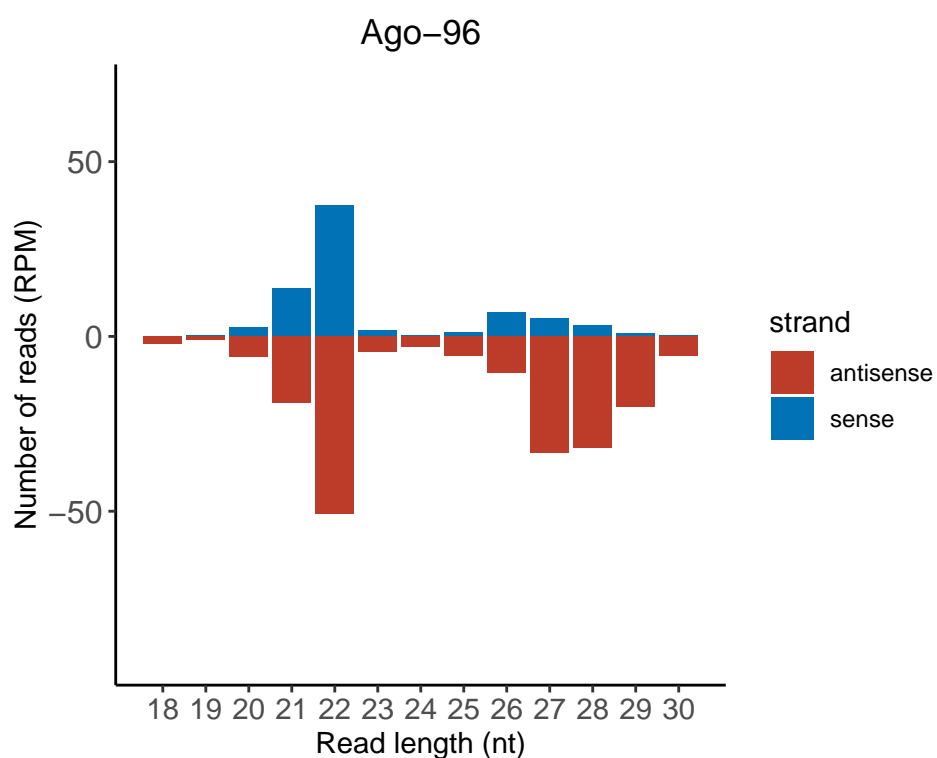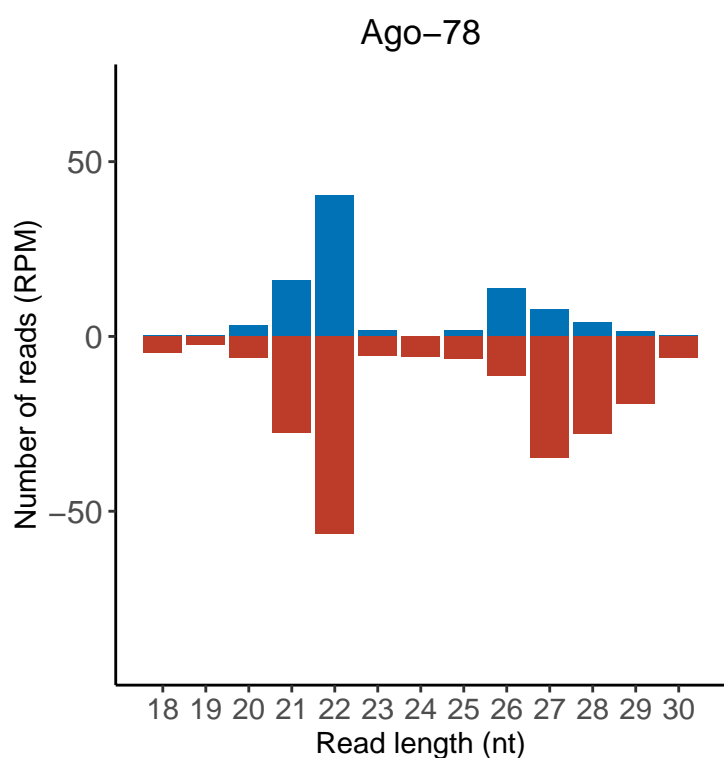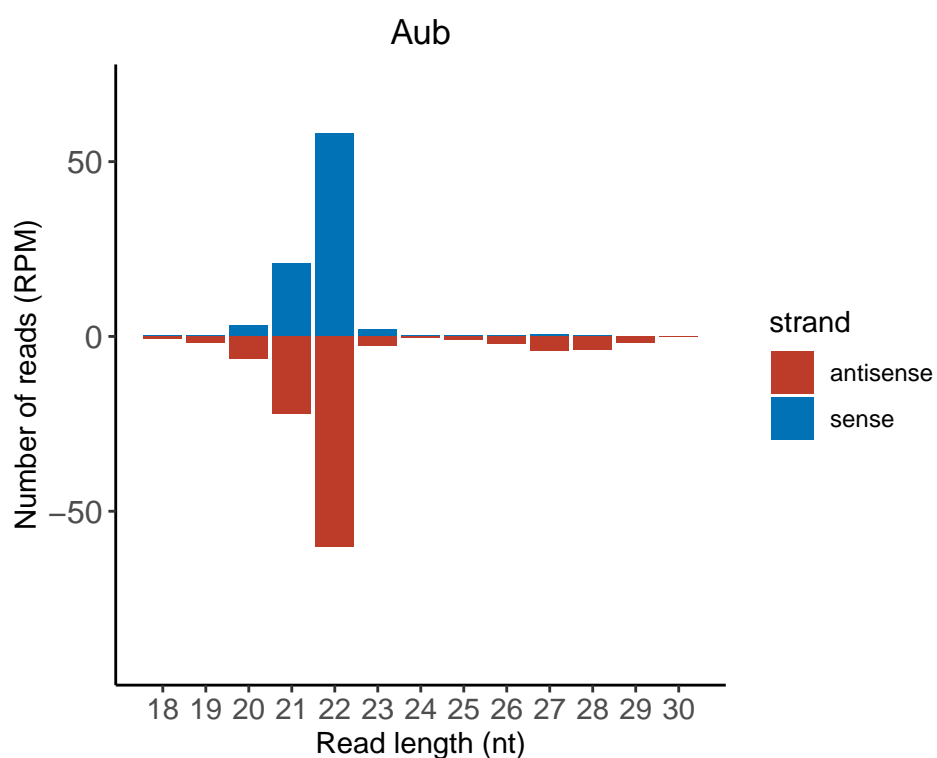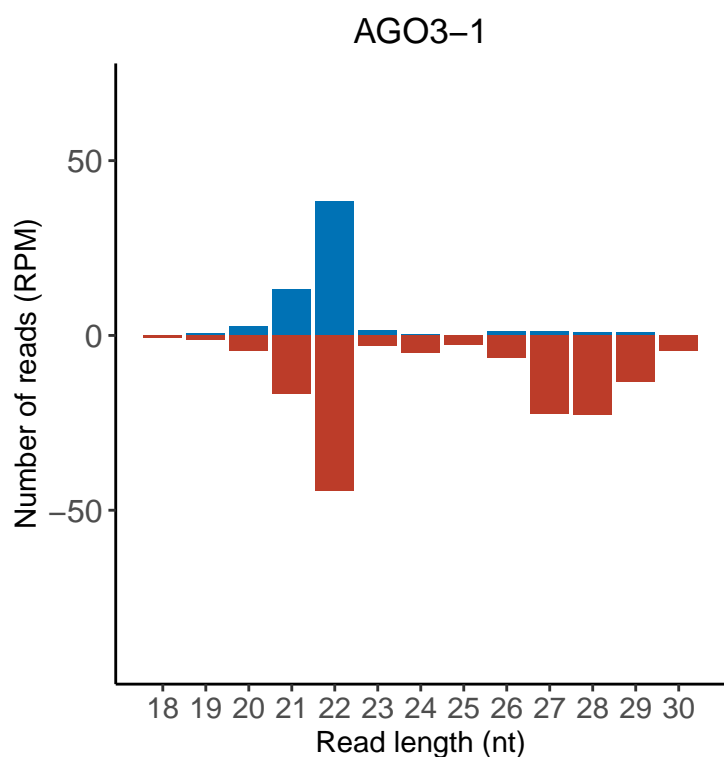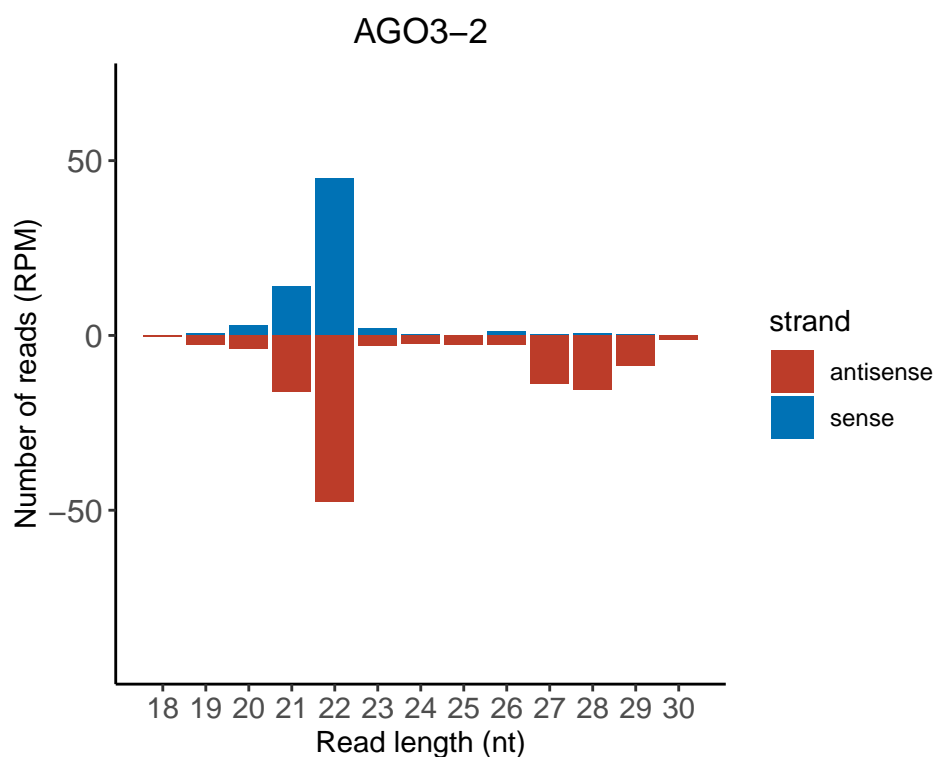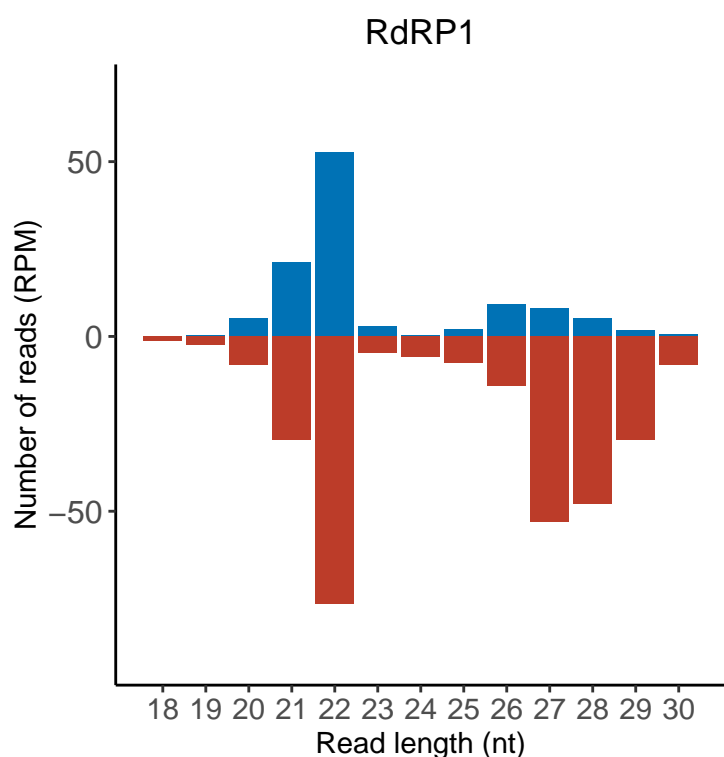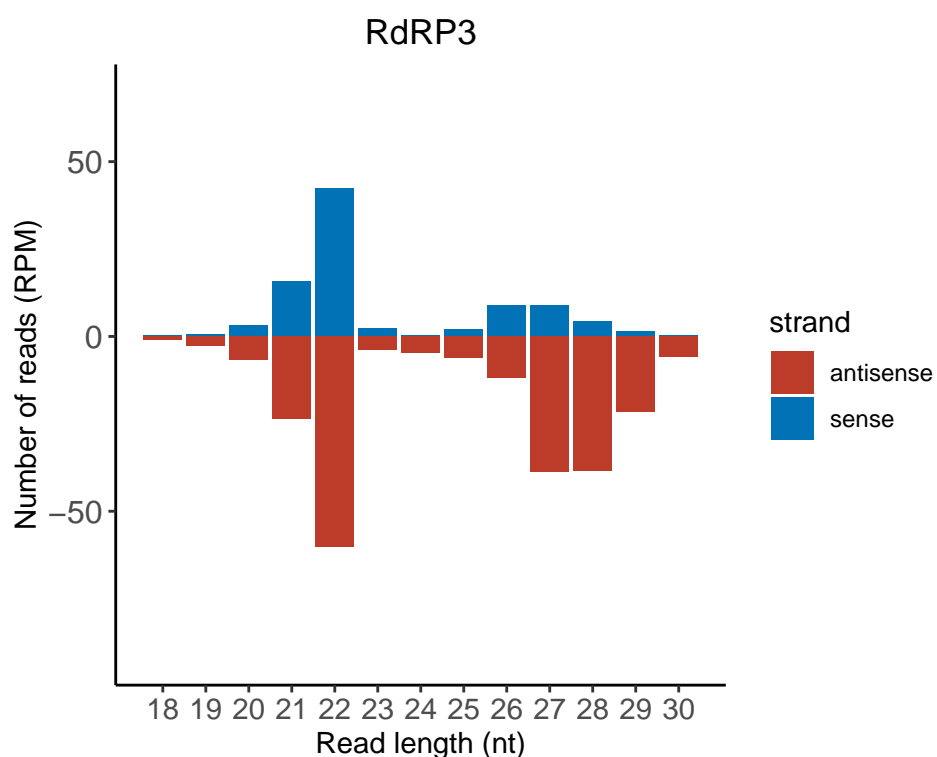

Supplement: S1 Data — On the Normalized and Relative levels pages, normalized read counts (RPM) and relative levels compared to the control (dsGFP) library were used. TEs with more than 50RPM and Coding Genes with more than 3.5RPM on average in KD libraries are included in this website. For viral sequences, reference sequences were generated by assembling sRNA sequences (See the Analysis of sRNAseq data section in Materials and methods.) and size distributions of sRNA reads mapped to each contig are shown. Reads were first grouped by their 5’ nucleotides, and reads of each length were counted. Full tables including TEs and Coding genes with fewer reads are in the “FullTable” folder. (ZIP) [file pone.0281195.s017.zip › SupplementaryData/Links/PDFs/Motif:rnd-6_family-9065TE_lengthdistribution.pdf]

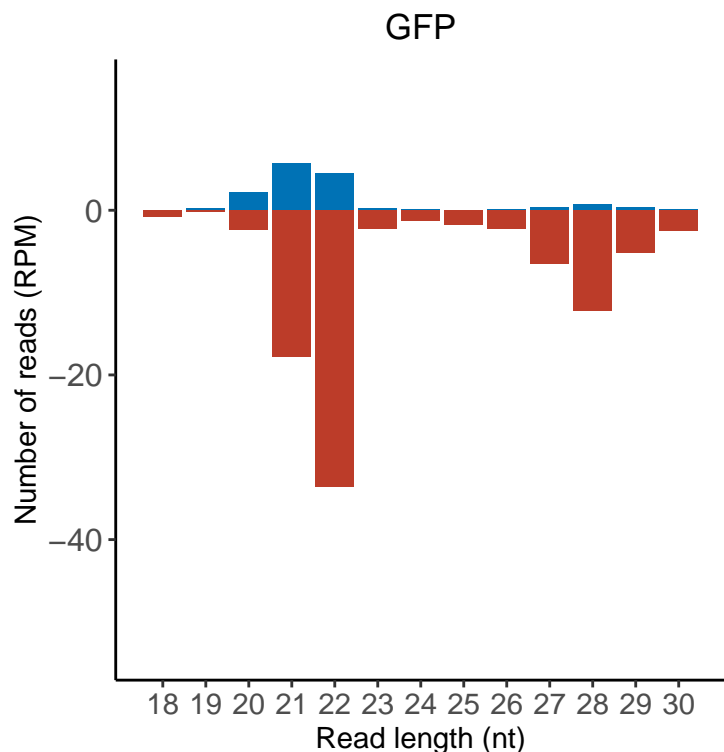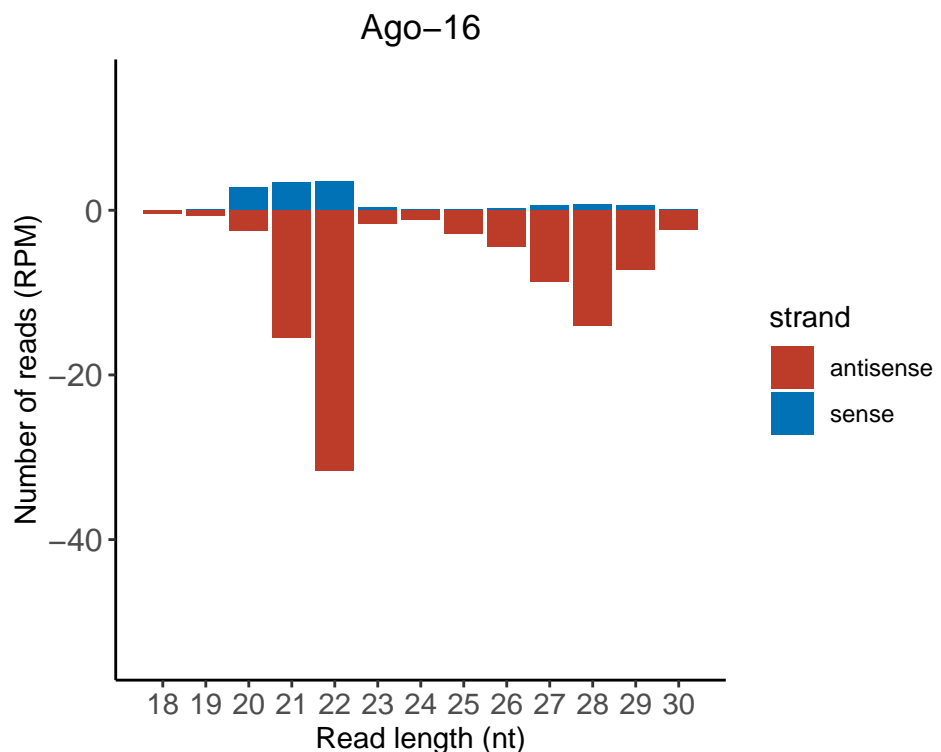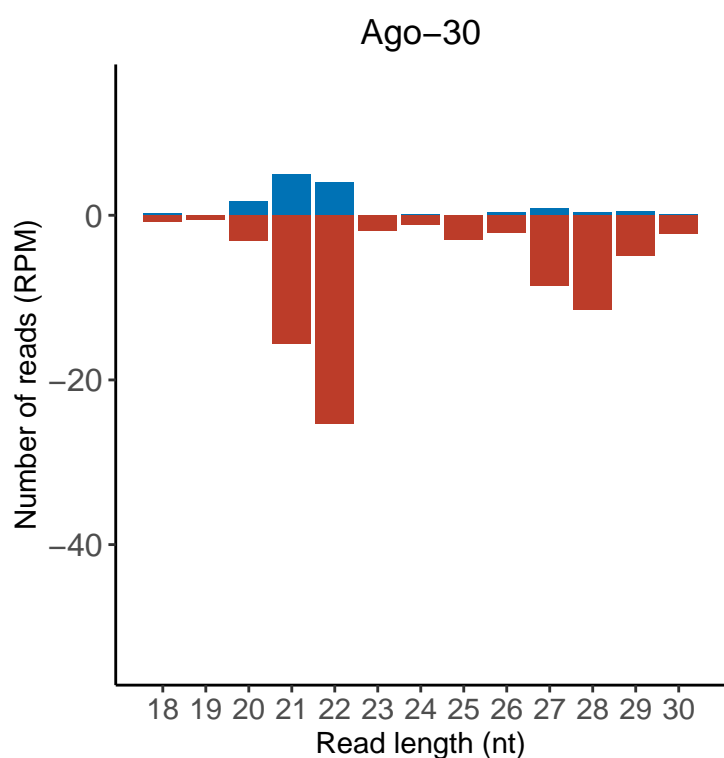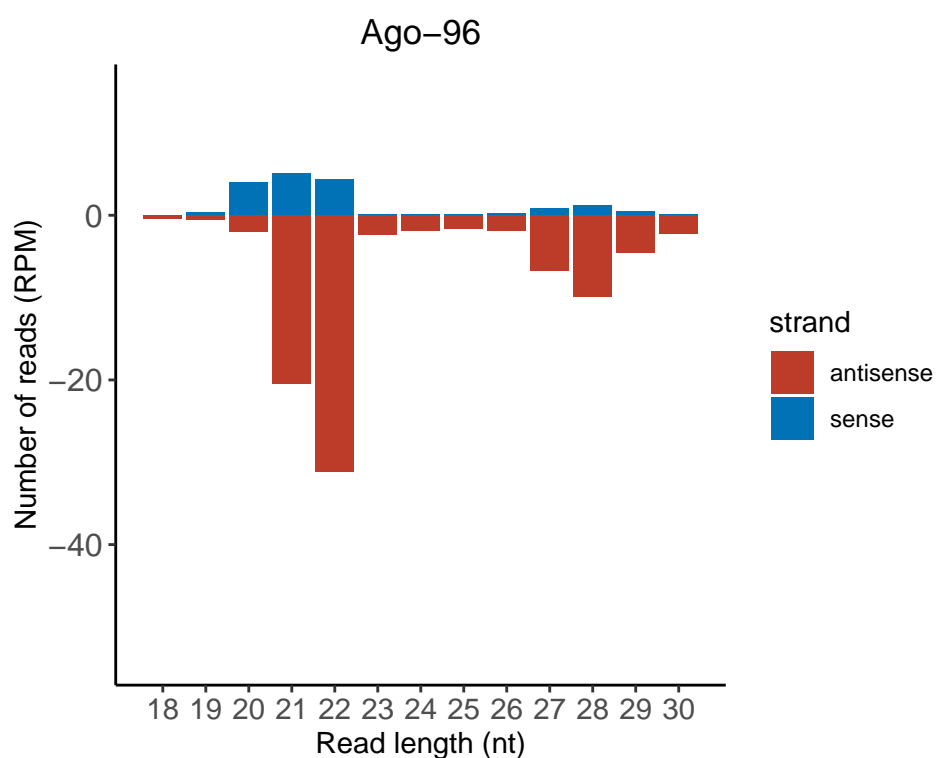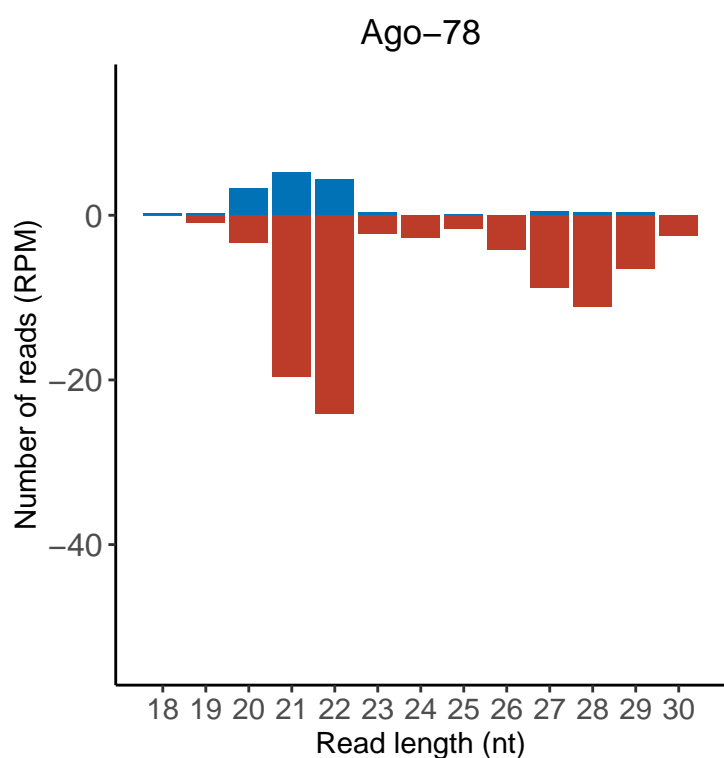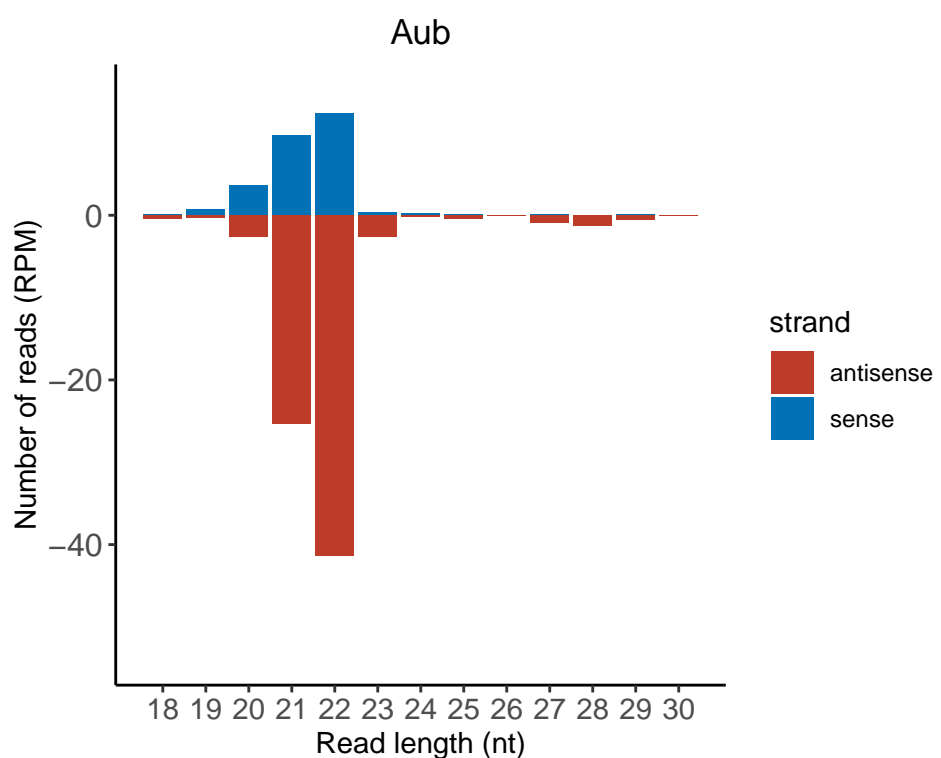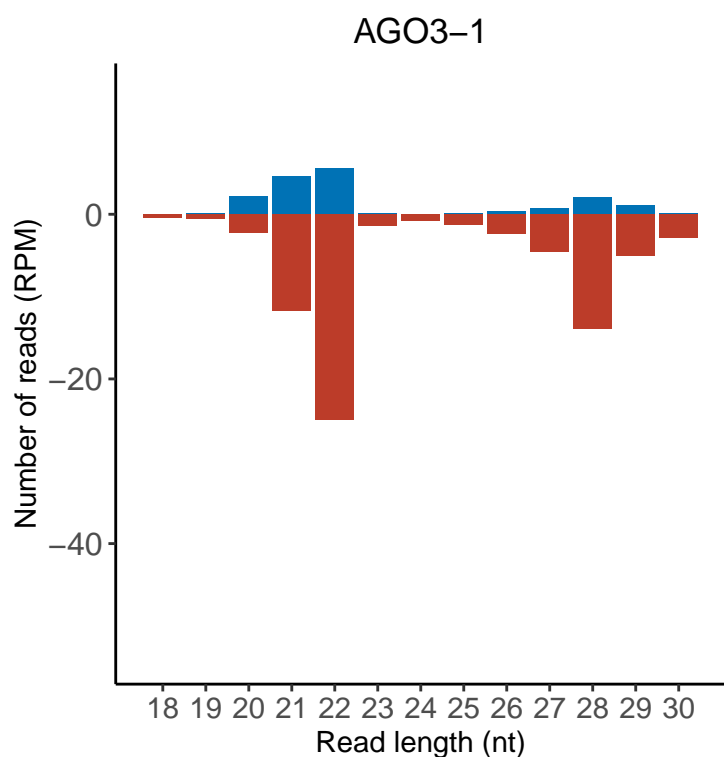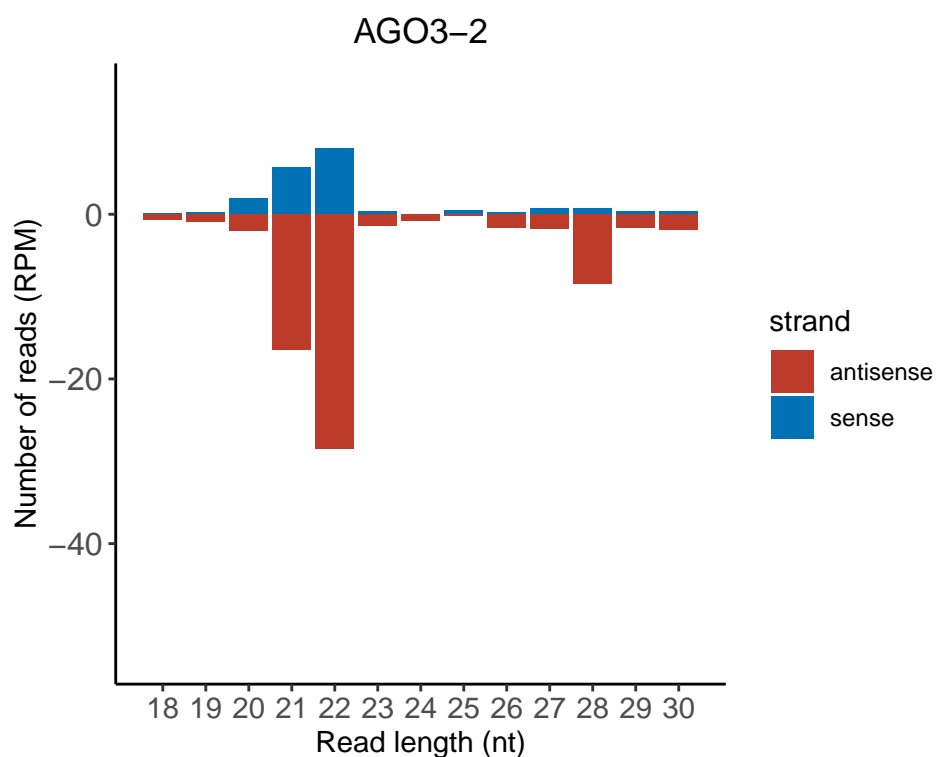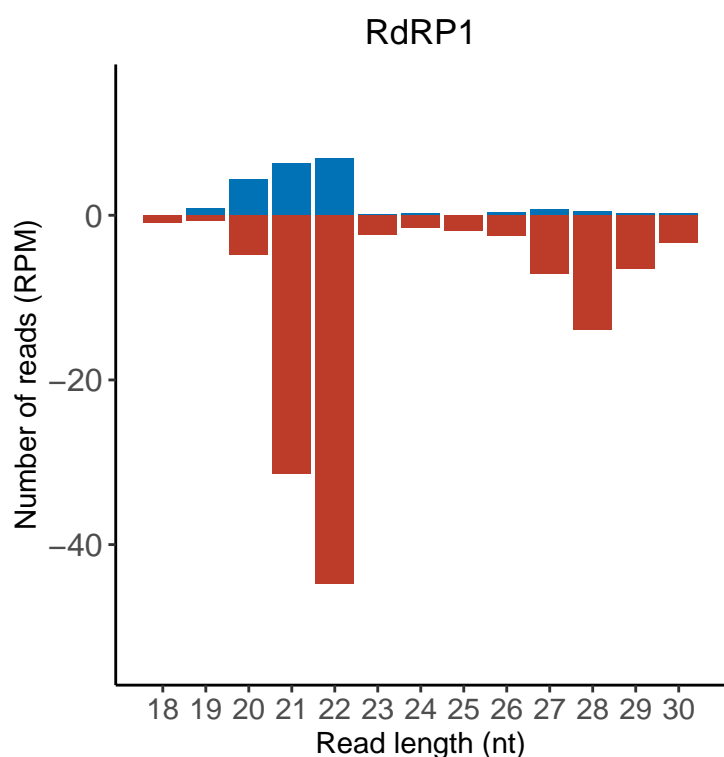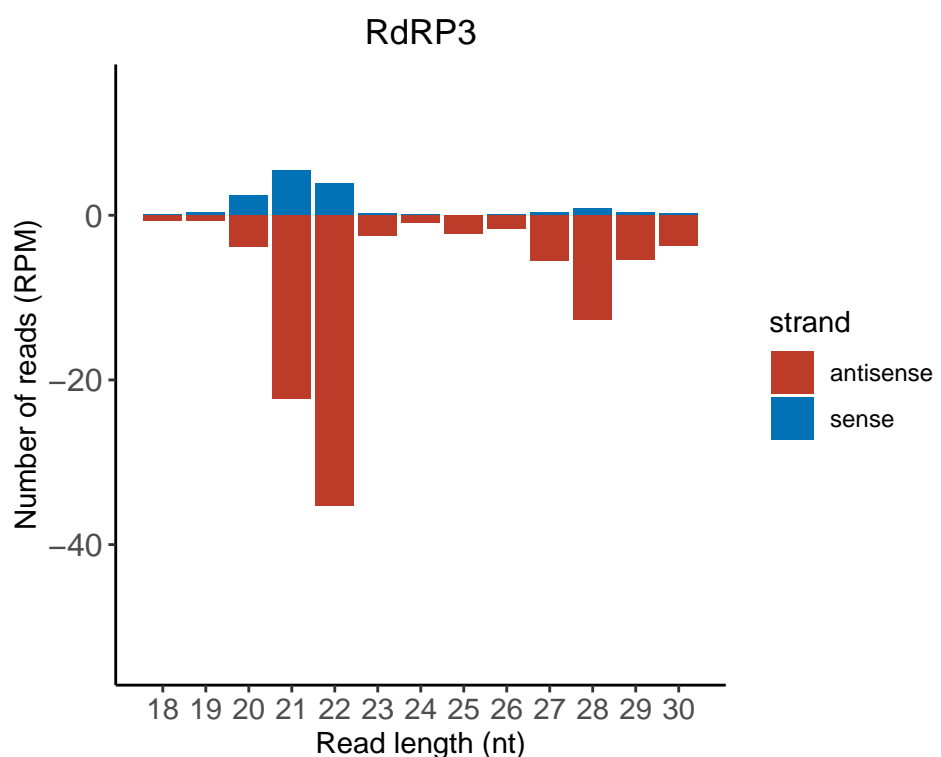

Supplement: S1 Data — On the Normalized and Relative levels pages, normalized read counts (RPM) and relative levels compared to the control (dsGFP) library were used. TEs with more than 50RPM and Coding Genes with more than 3.5RPM on average in KD libraries are included in this website. For viral sequences, reference sequences were generated by assembling sRNA sequences (See the Analysis of sRNAseq data section in Materials and methods.) and size distributions of sRNA reads mapped to each contig are shown. Reads were first grouped by their 5’ nucleotides, and reads of each length were counted. Full tables including TEs and Coding genes with fewer reads are in the “FullTable” folder. (ZIP) [file pone.0281195.s017.zip › SupplementaryData/Links/PDFs/Motif:rnd-1_family-122TE_lengthdistribution.pdf]

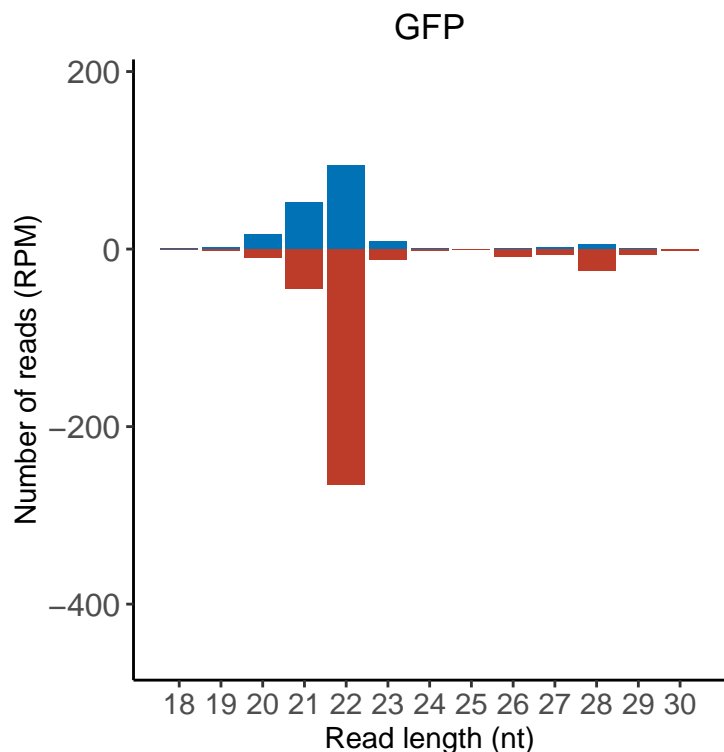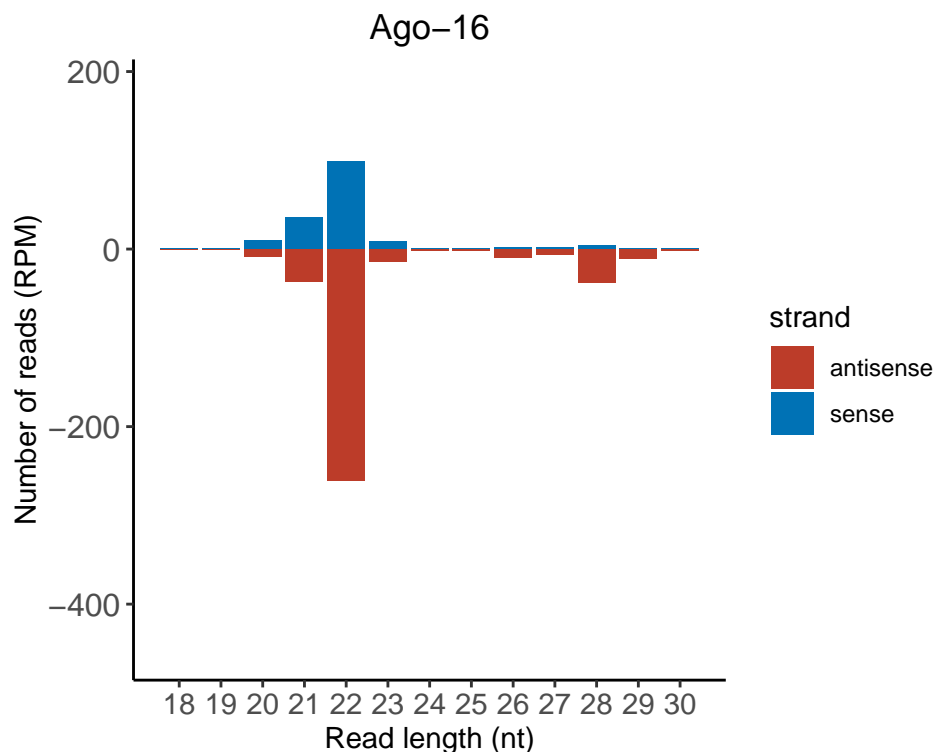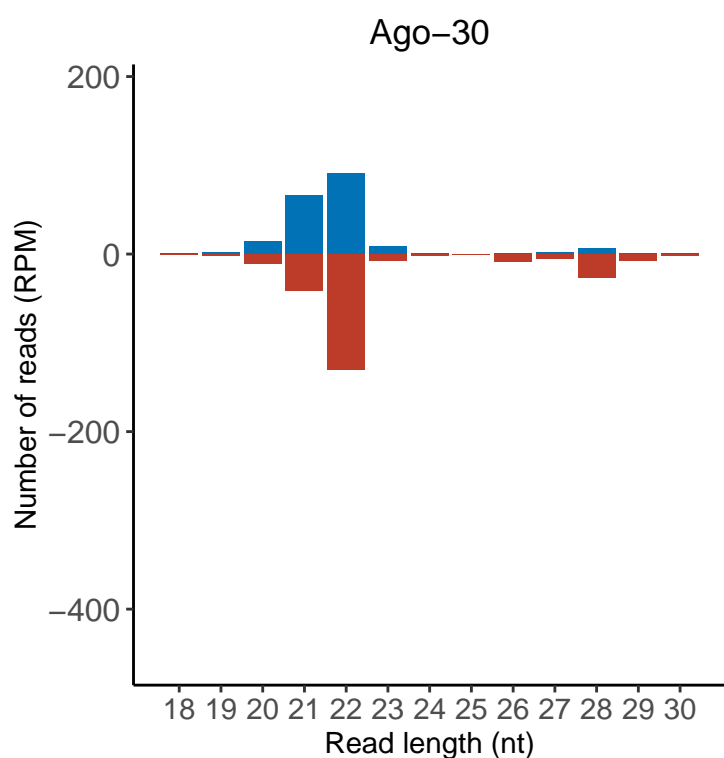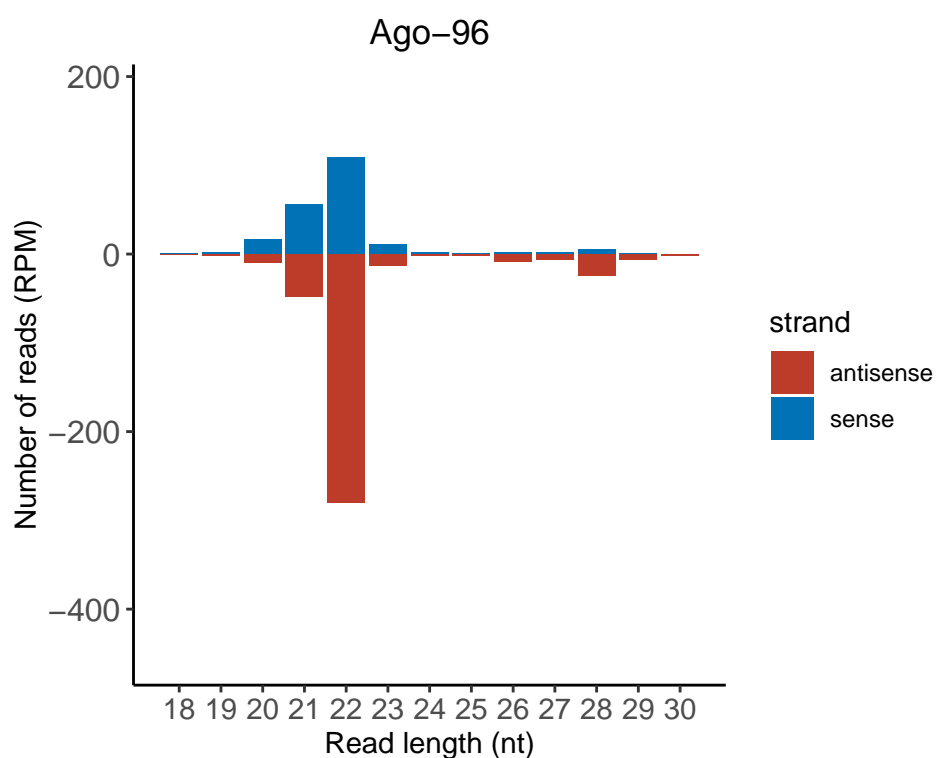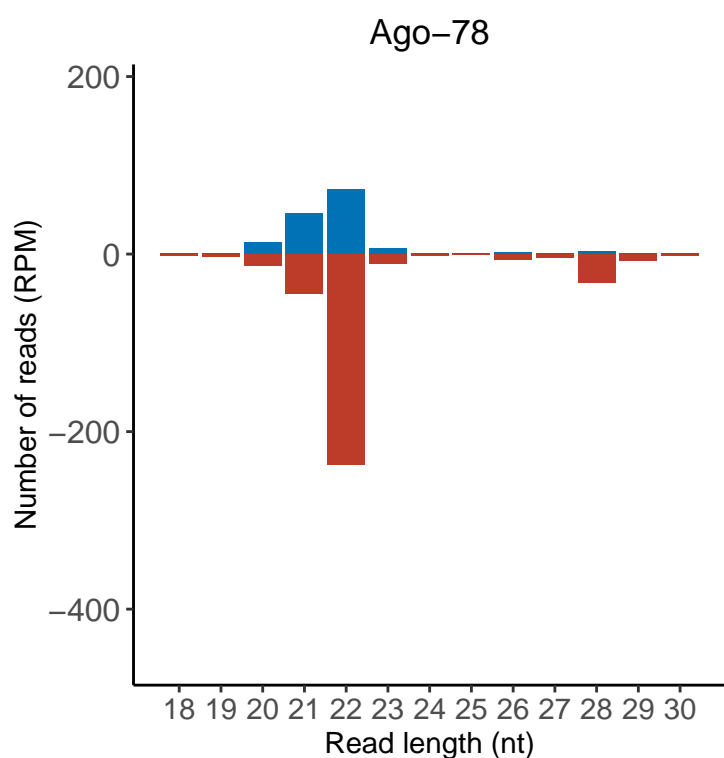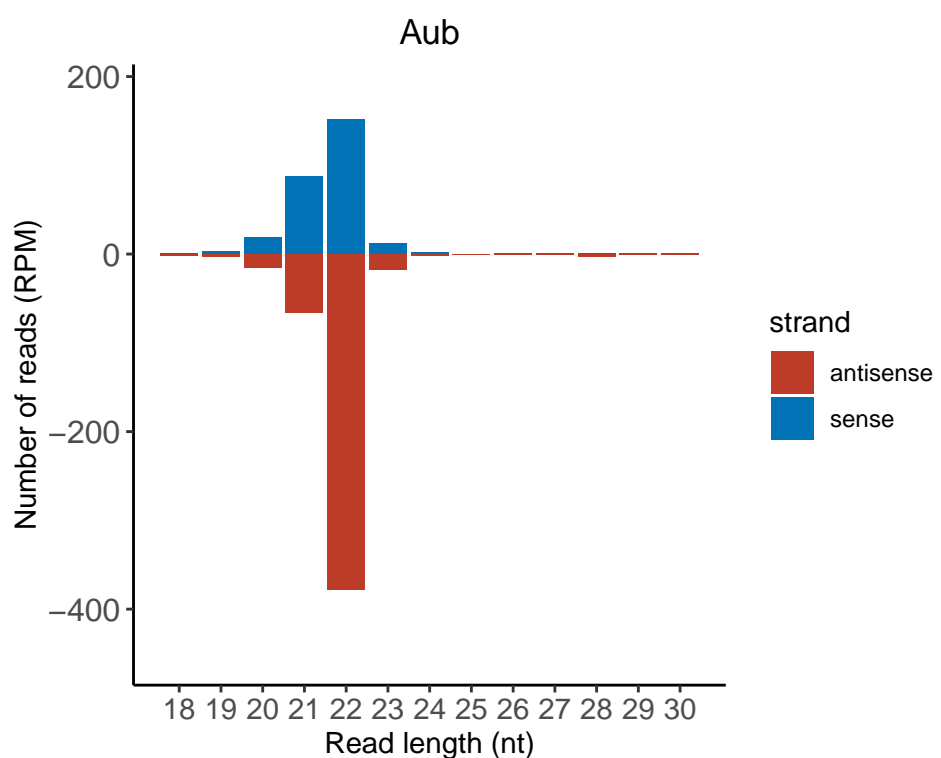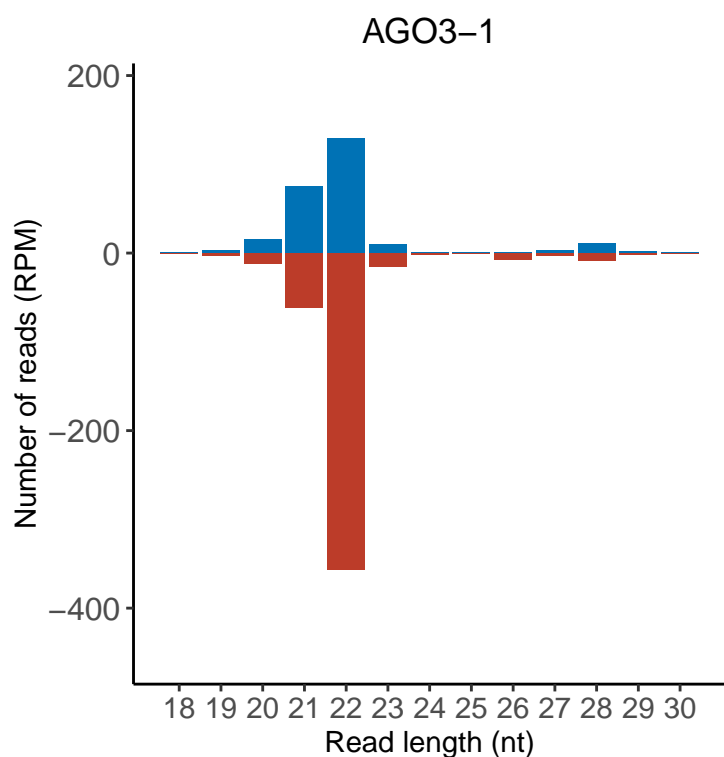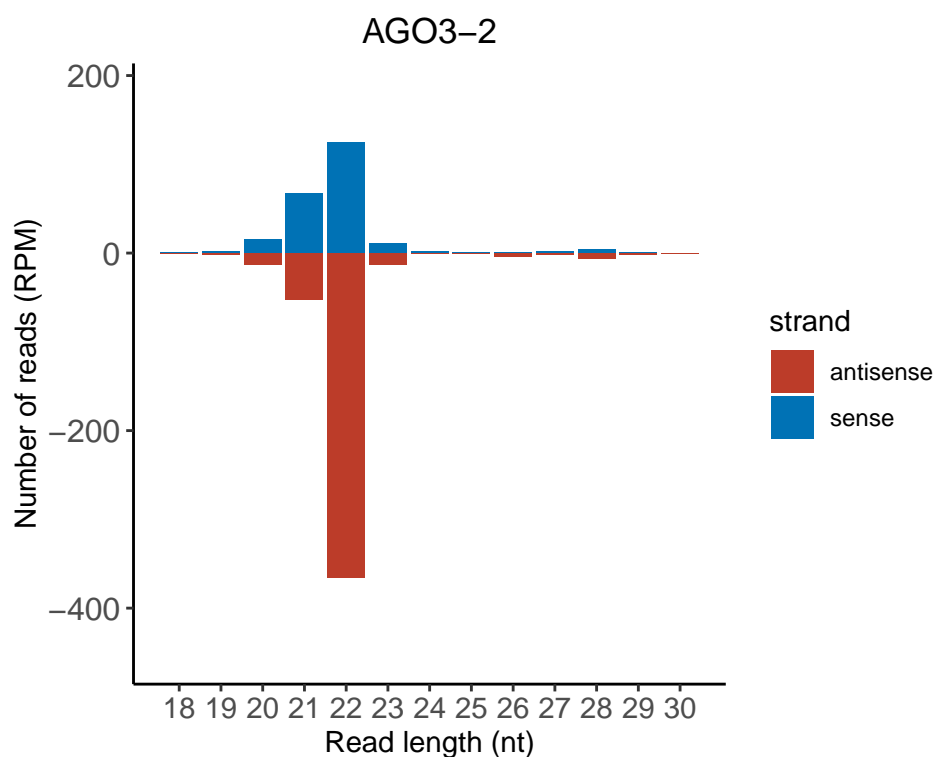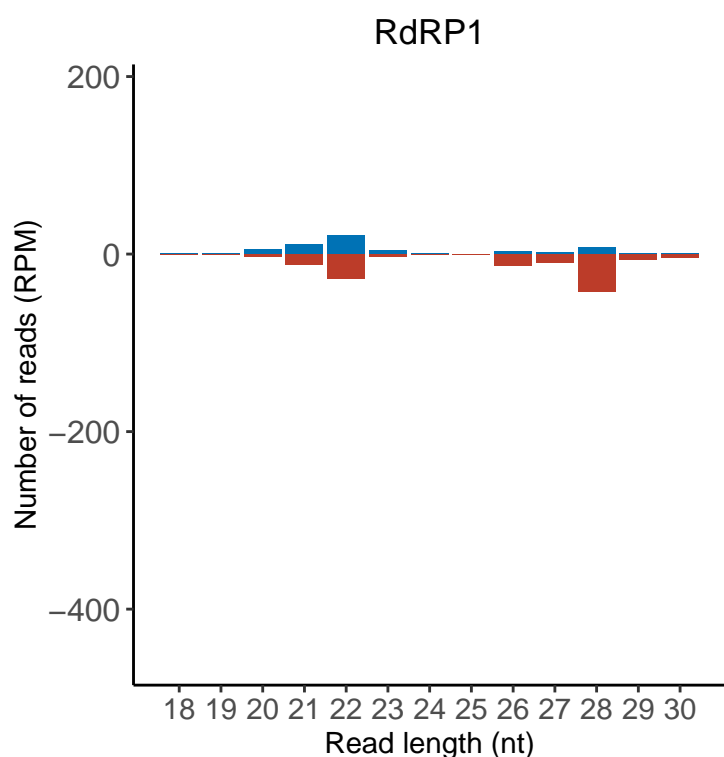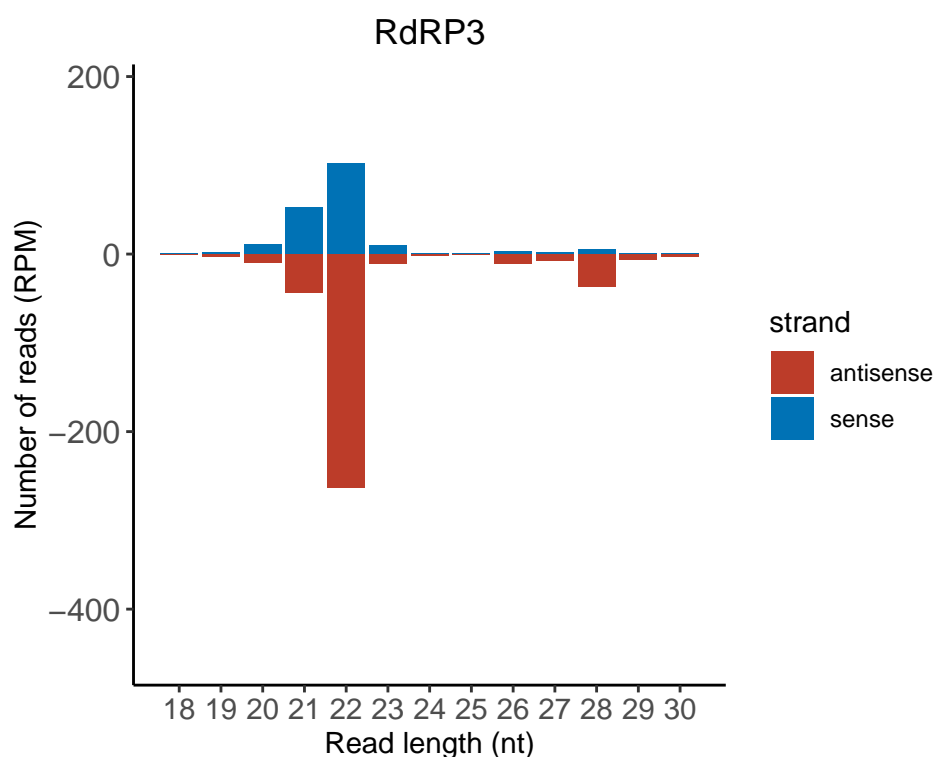

Supplement: S1 Data — On the Normalized and Relative levels pages, normalized read counts (RPM) and relative levels compared to the control (dsGFP) library were used. TEs with more than 50RPM and Coding Genes with more than 3.5RPM on average in KD libraries are included in this website. For viral sequences, reference sequences were generated by assembling sRNA sequences (See the Analysis of sRNAseq data section in Materials and methods.) and size distributions of sRNA reads mapped to each contig are shown. Reads were first grouped by their 5’ nucleotides, and reads of each length were counted. Full tables including TEs and Coding genes with fewer reads are in the “FullTable” folder. (ZIP) [file pone.0281195.s017.zip › SupplementaryData/Links/PDFs/Motif:rnd-1_family-96TE_lengthdistribution.pdf]

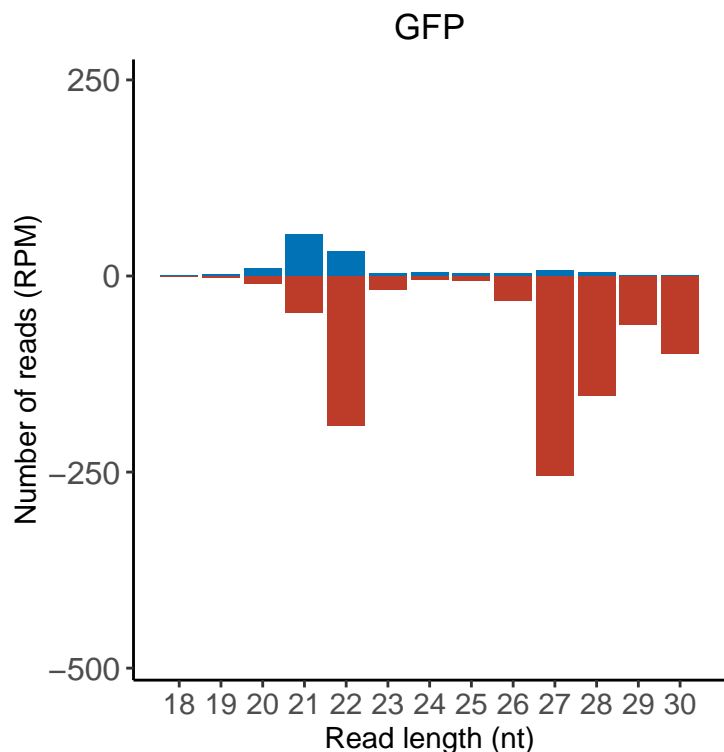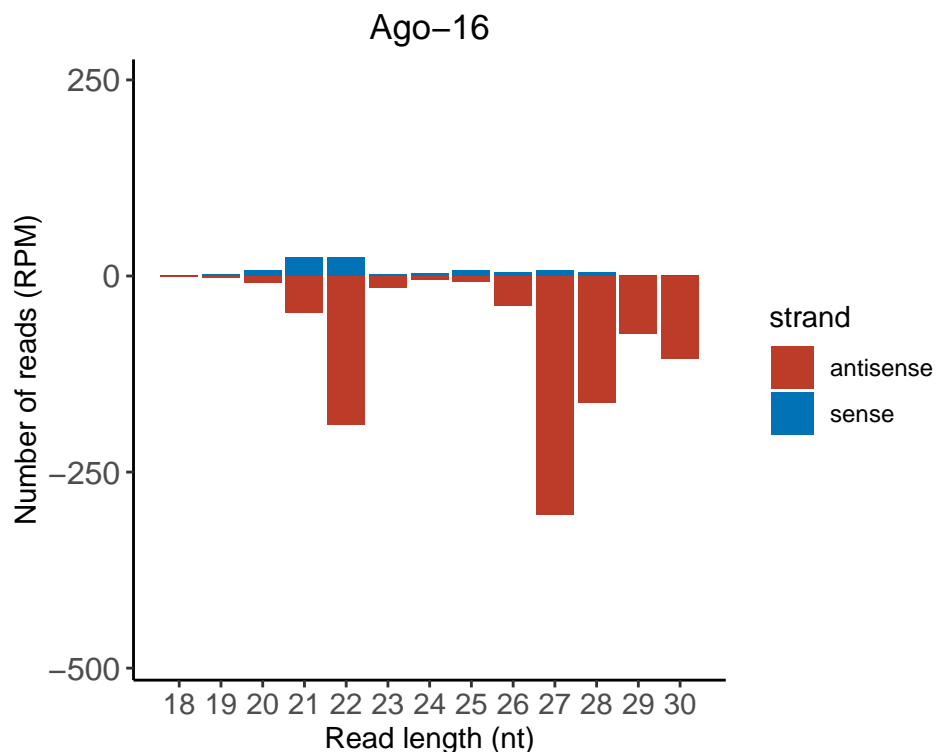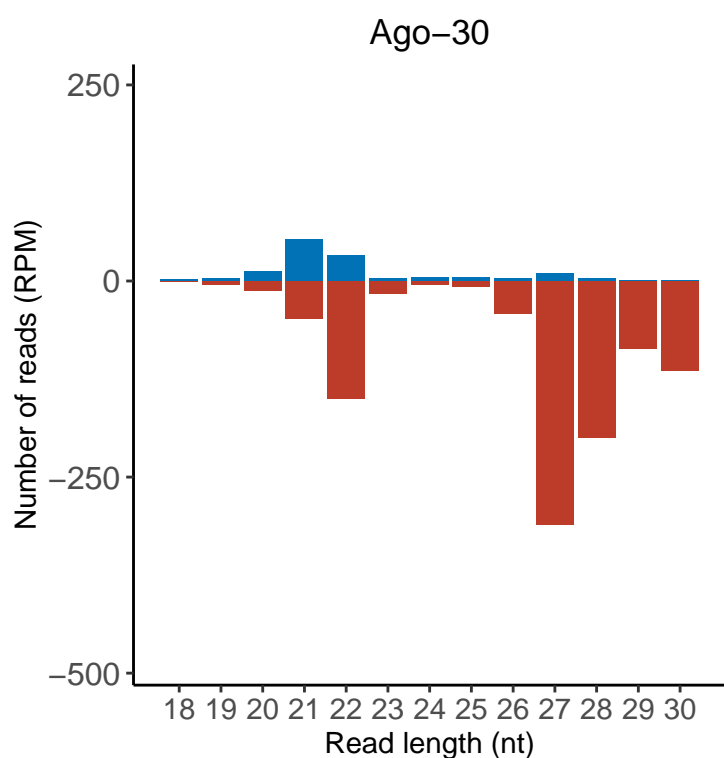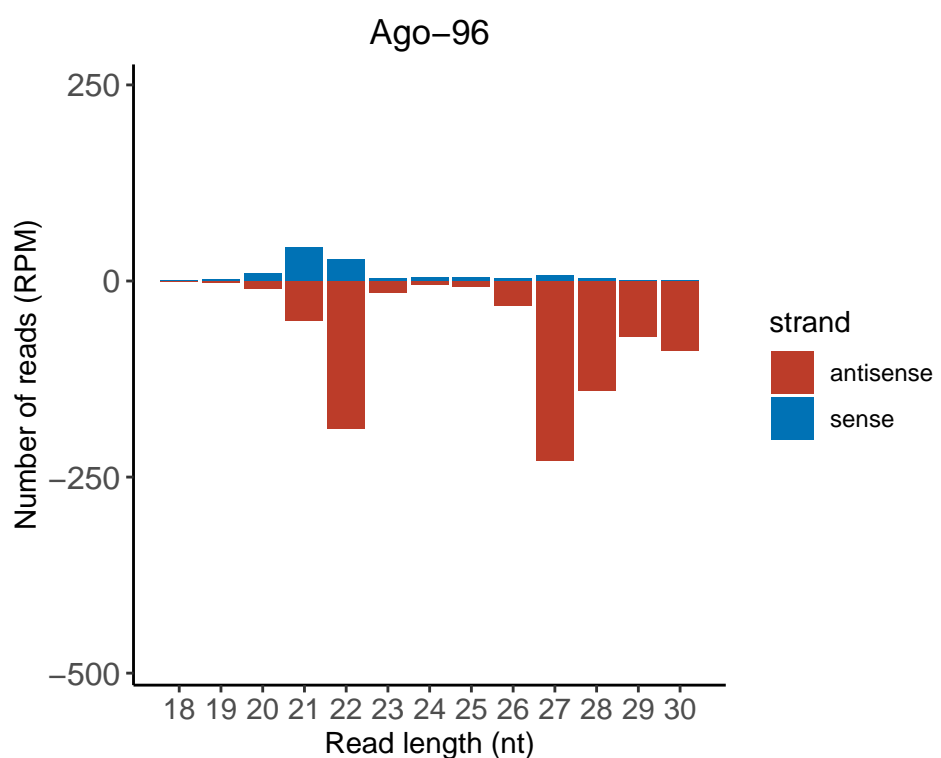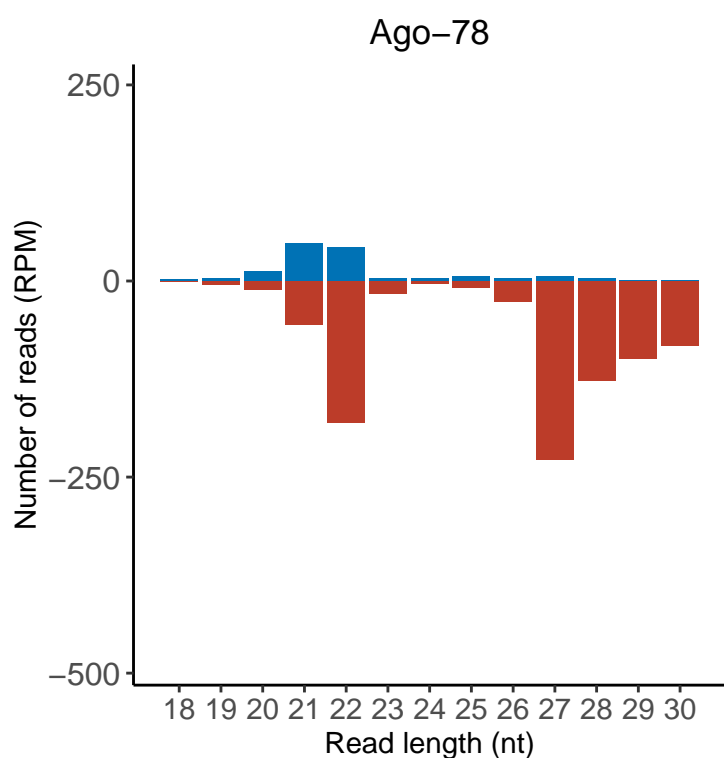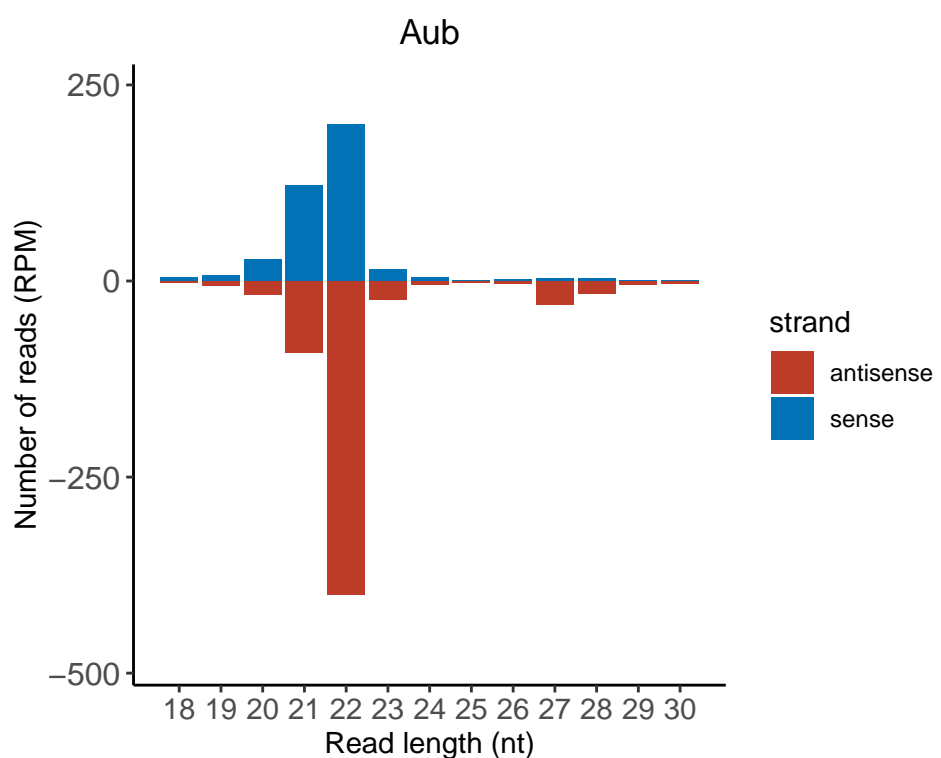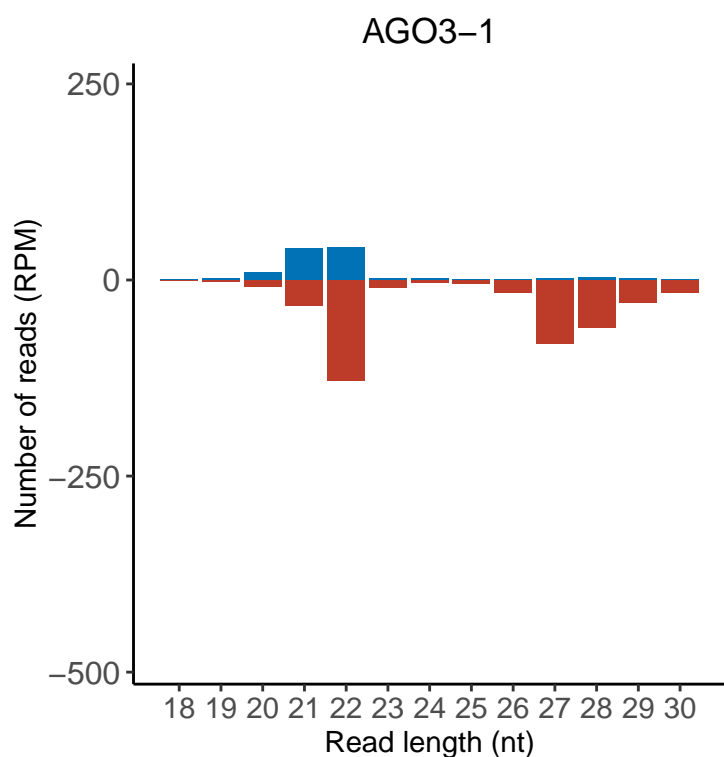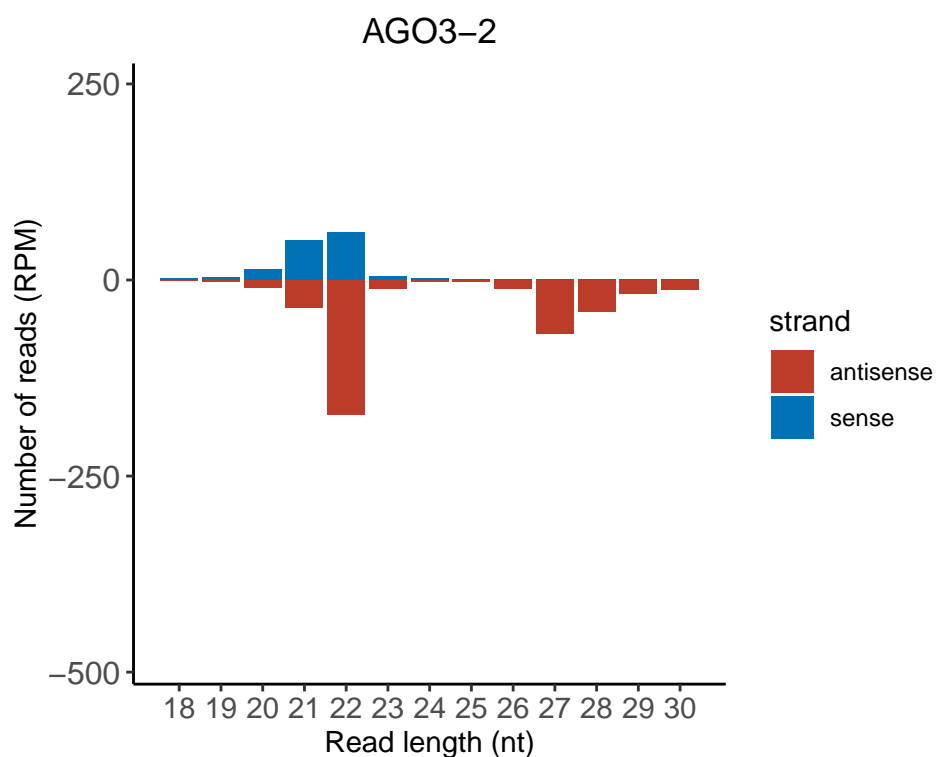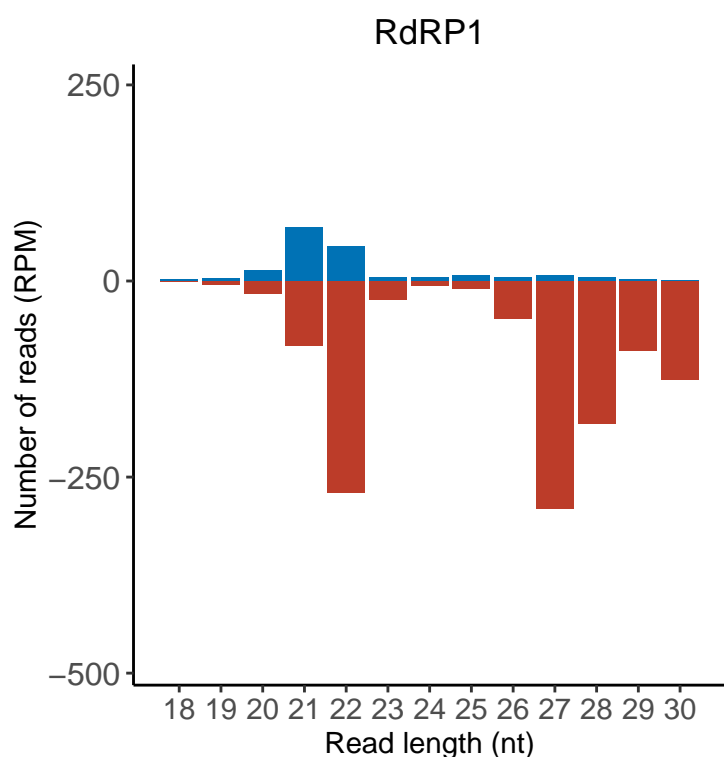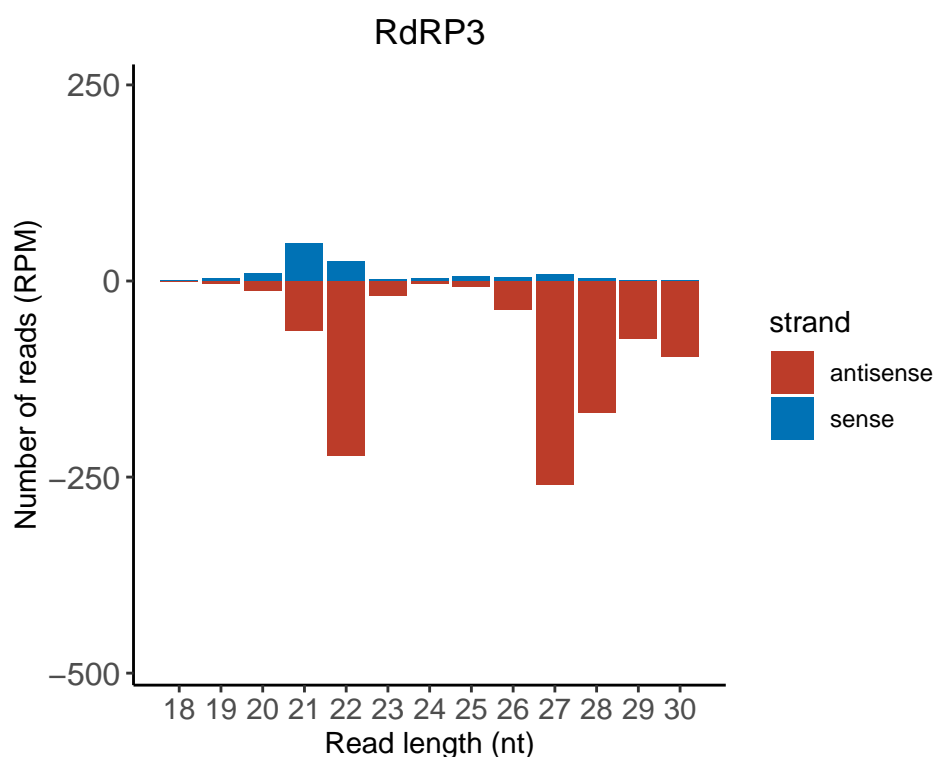

Supplement: S1 Data — On the Normalized and Relative levels pages, normalized read counts (RPM) and relative levels compared to the control (dsGFP) library were used. TEs with more than 50RPM and Coding Genes with more than 3.5RPM on average in KD libraries are included in this website. For viral sequences, reference sequences were generated by assembling sRNA sequences (See the Analysis of sRNAseq data section in Materials and methods.) and size distributions of sRNA reads mapped to each contig are shown. Reads were first grouped by their 5’ nucleotides, and reads of each length were counted. Full tables including TEs and Coding genes with fewer reads are in the “FullTable” folder. (ZIP) [file pone.0281195.s017.zip › SupplementaryData/Links/PDFs/Motif:rnd-5_family-154TE_lengthdistribution.pdf]

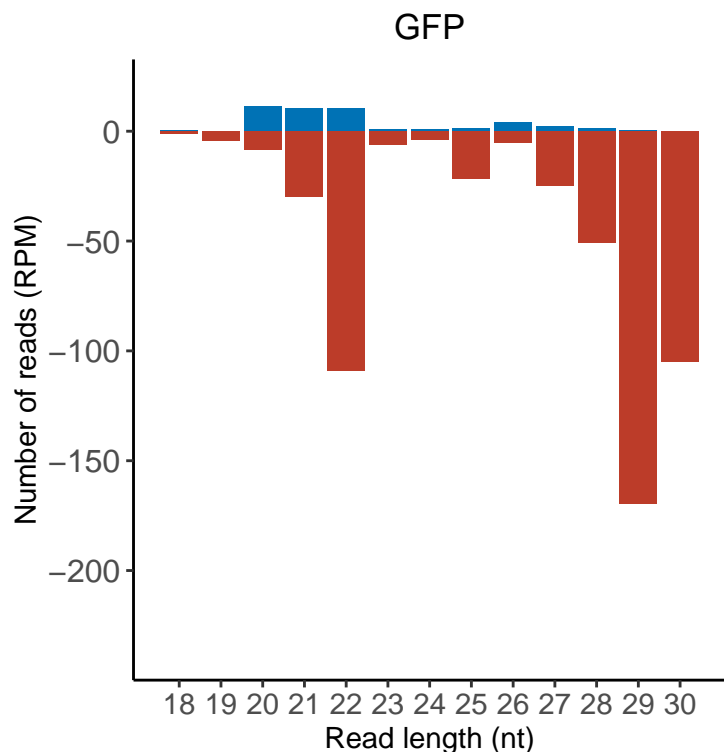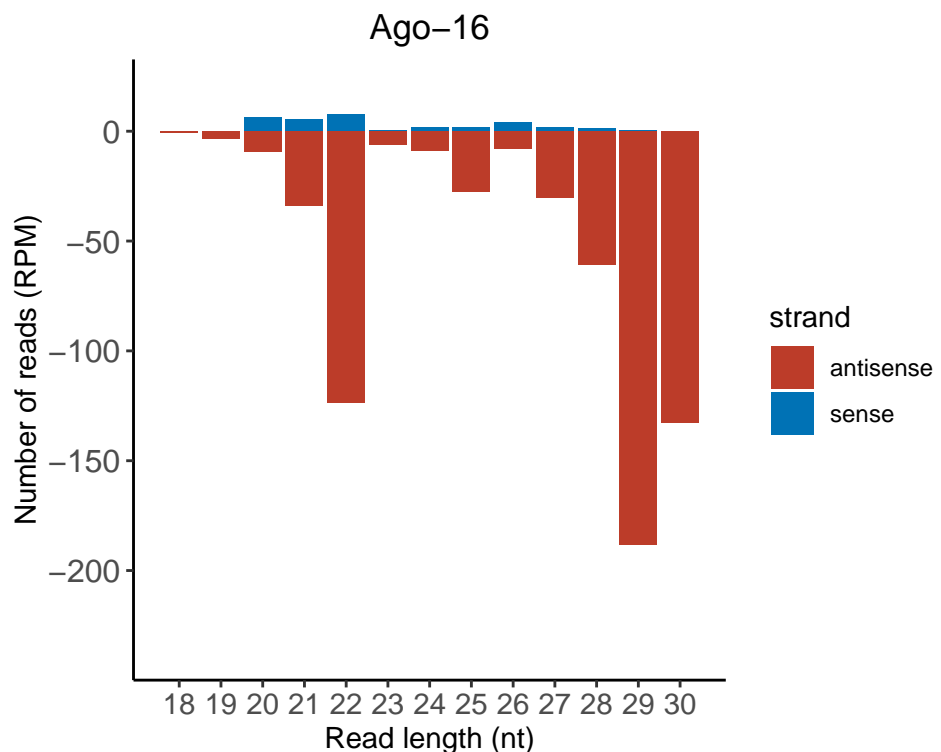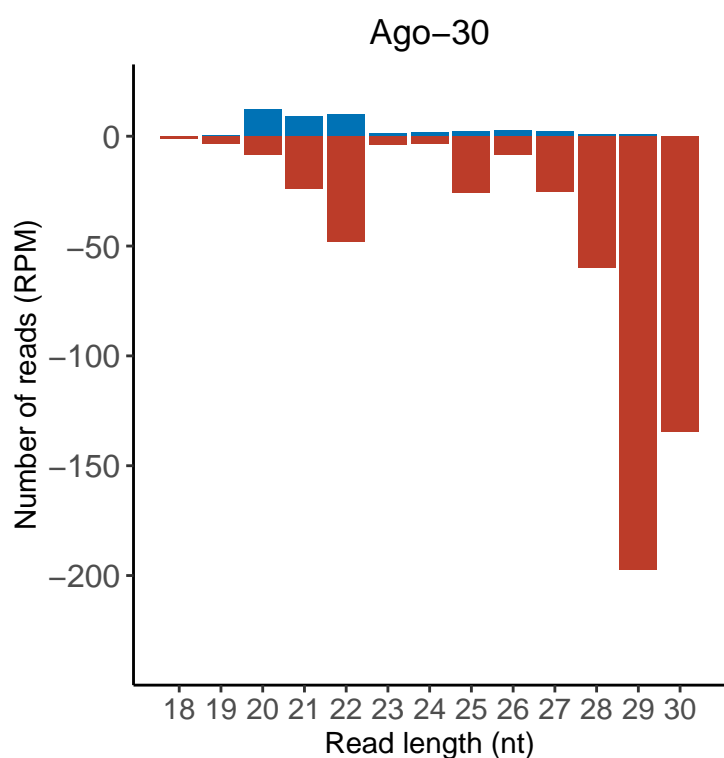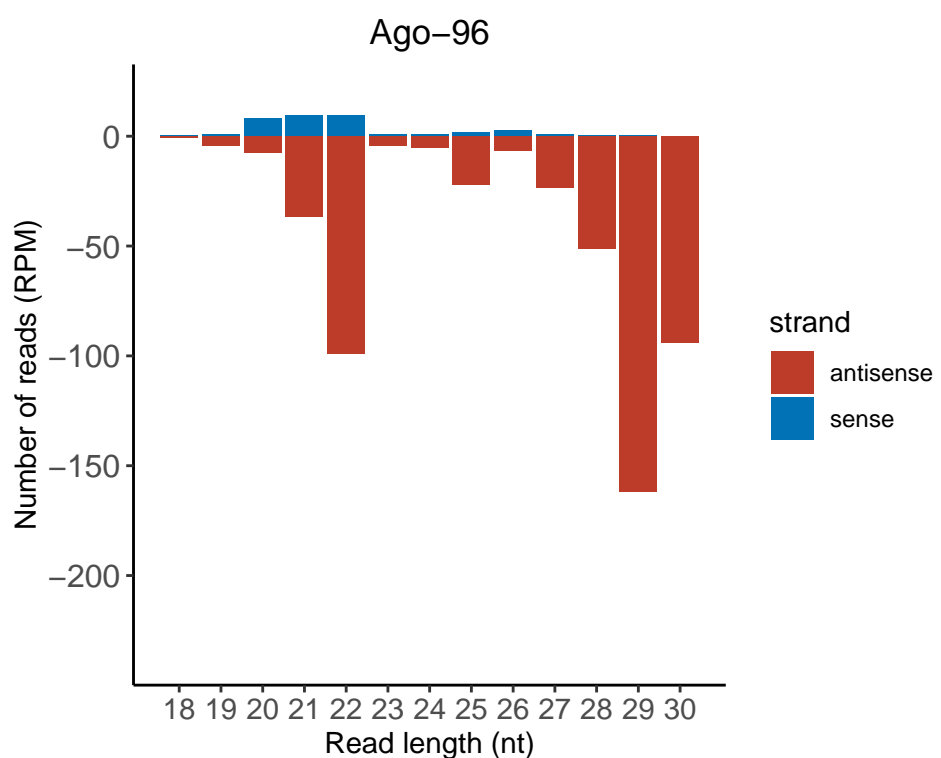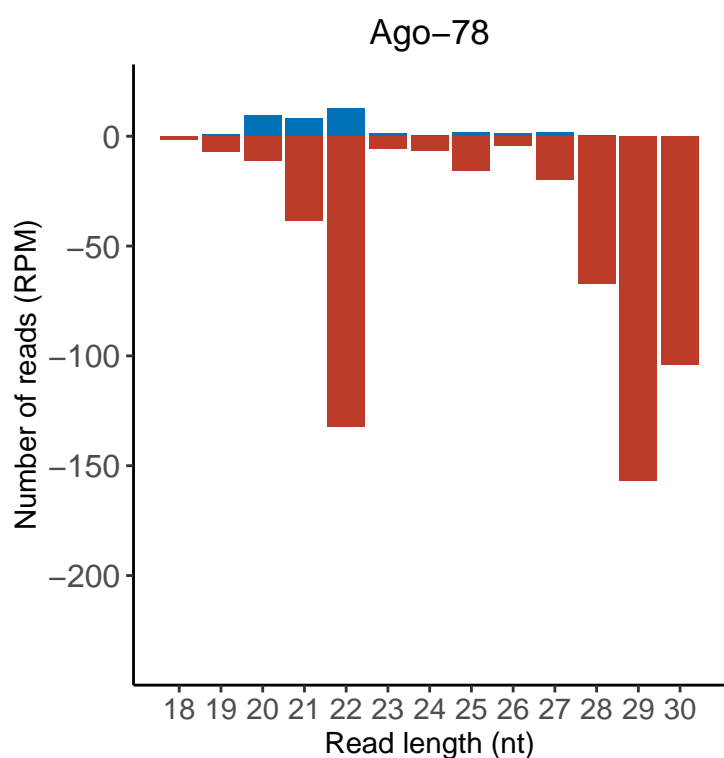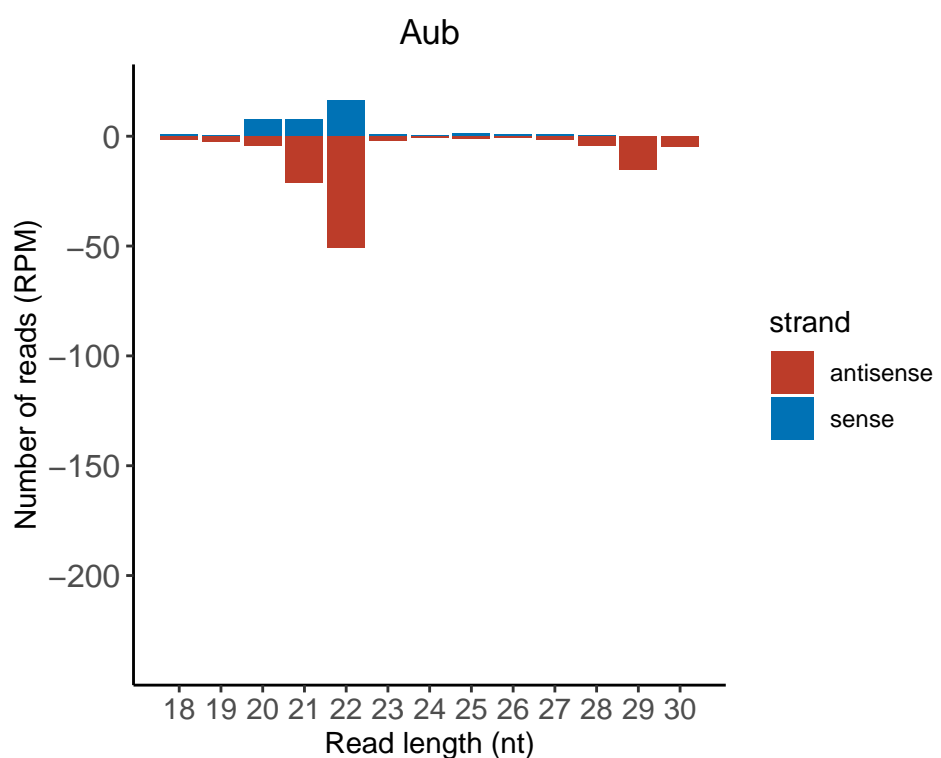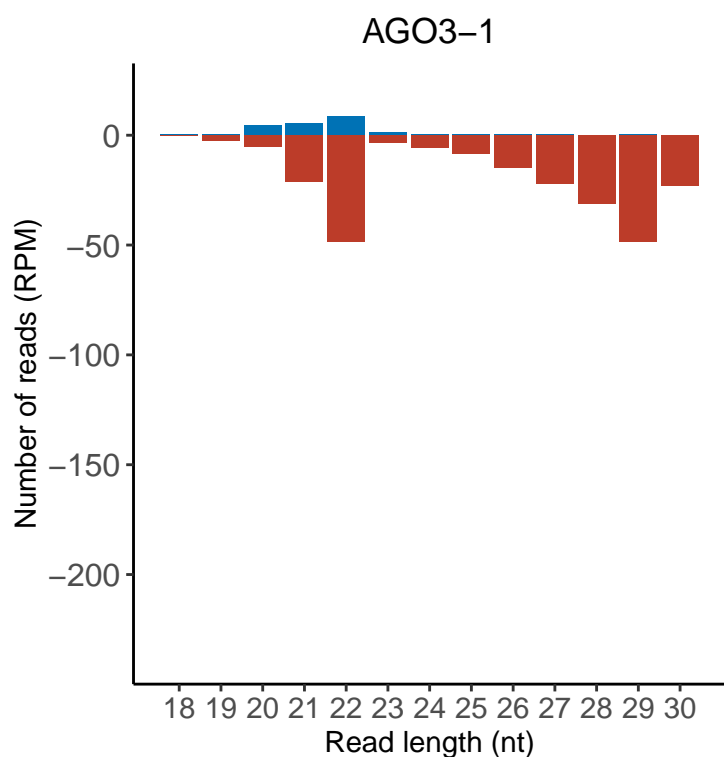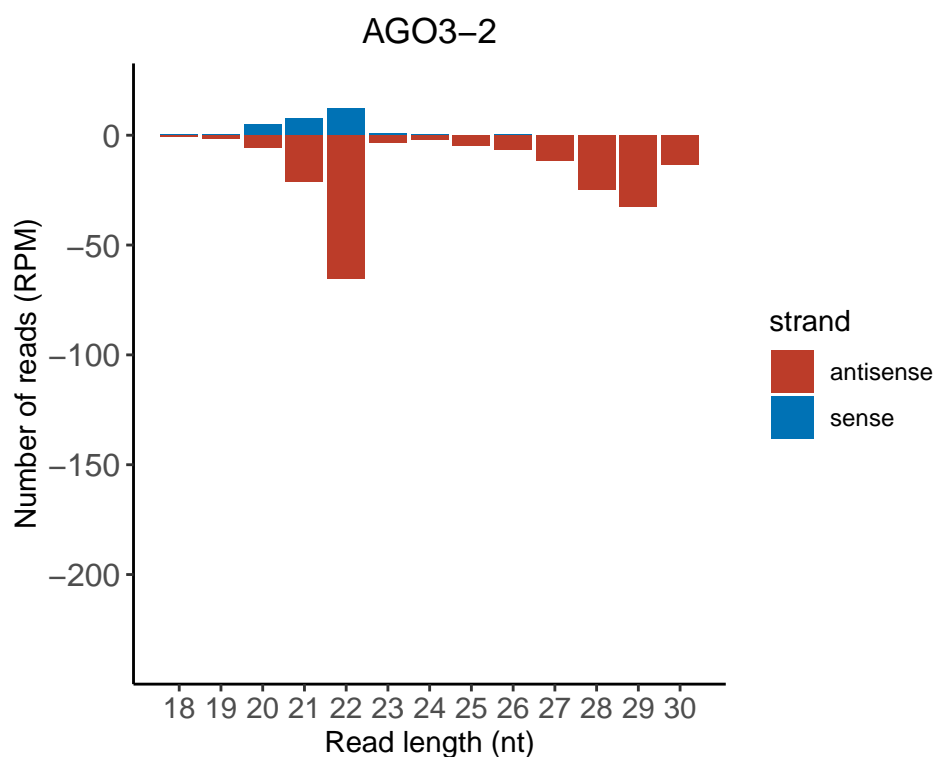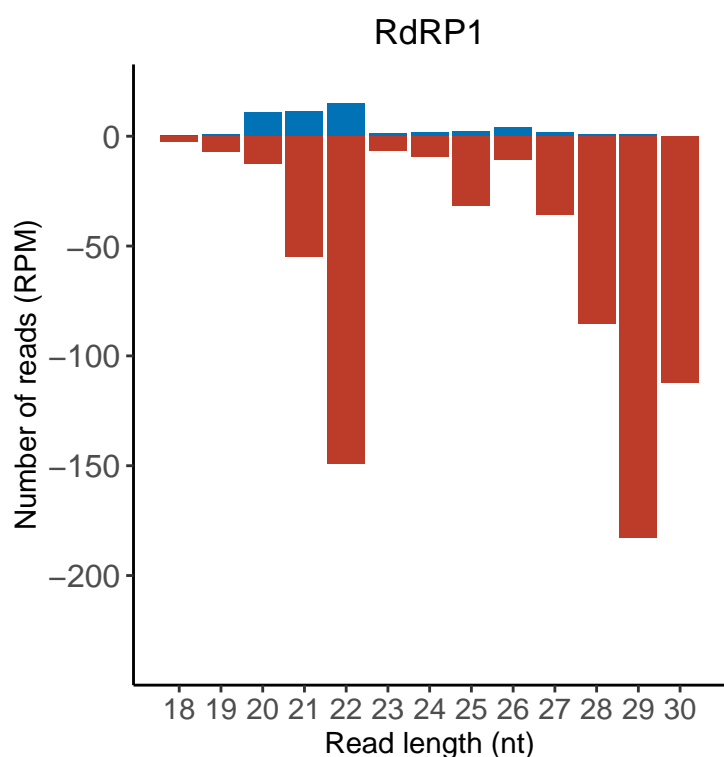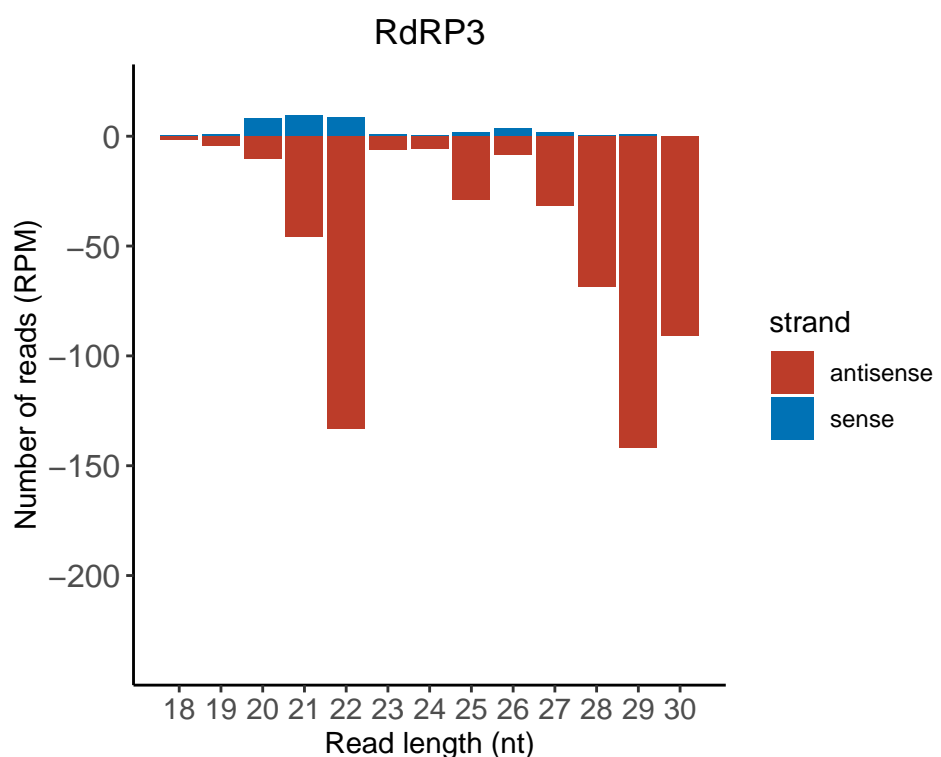

Supplement: S1 Data — On the Normalized and Relative levels pages, normalized read counts (RPM) and relative levels compared to the control (dsGFP) library were used. TEs with more than 50RPM and Coding Genes with more than 3.5RPM on average in KD libraries are included in this website. For viral sequences, reference sequences were generated by assembling sRNA sequences (See the Analysis of sRNAseq data section in Materials and methods.) and size distributions of sRNA reads mapped to each contig are shown. Reads were first grouped by their 5’ nucleotides, and reads of each length were counted. Full tables including TEs and Coding genes with fewer reads are in the “FullTable” folder. (ZIP) [file pone.0281195.s017.zip › SupplementaryData/Links/PDFs/Motif:rnd-5_family-1630TE_lengthdistribution.pdf]

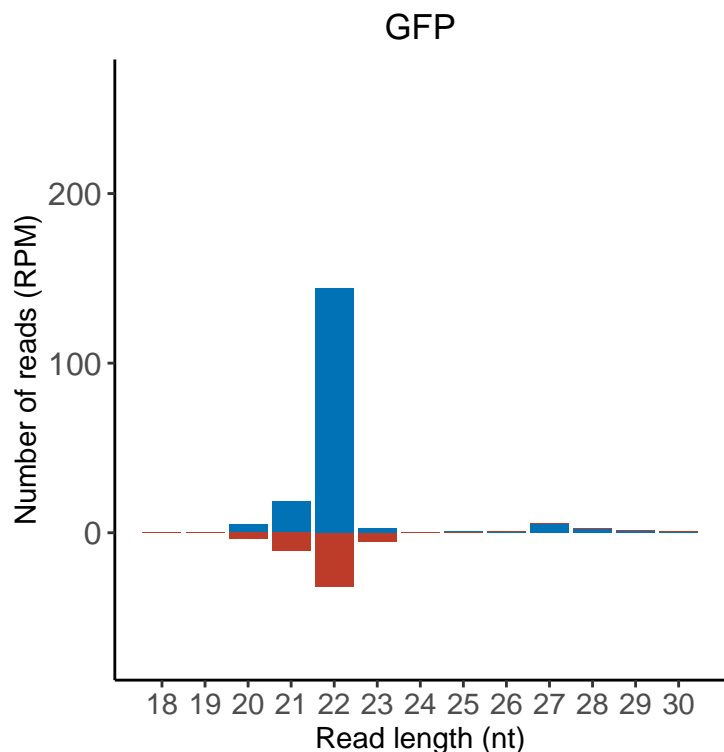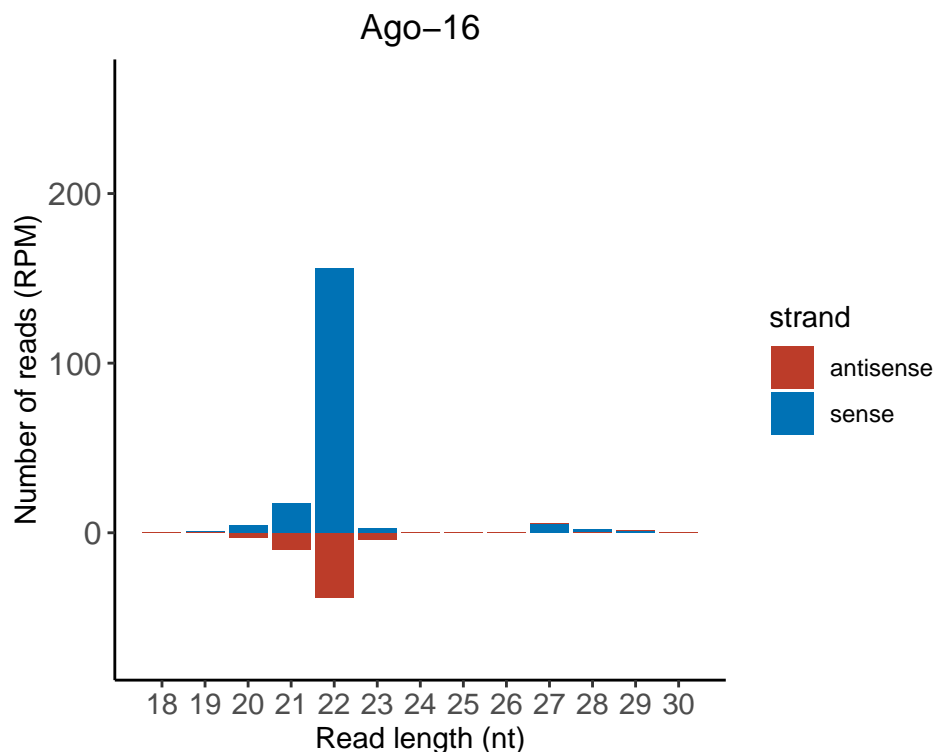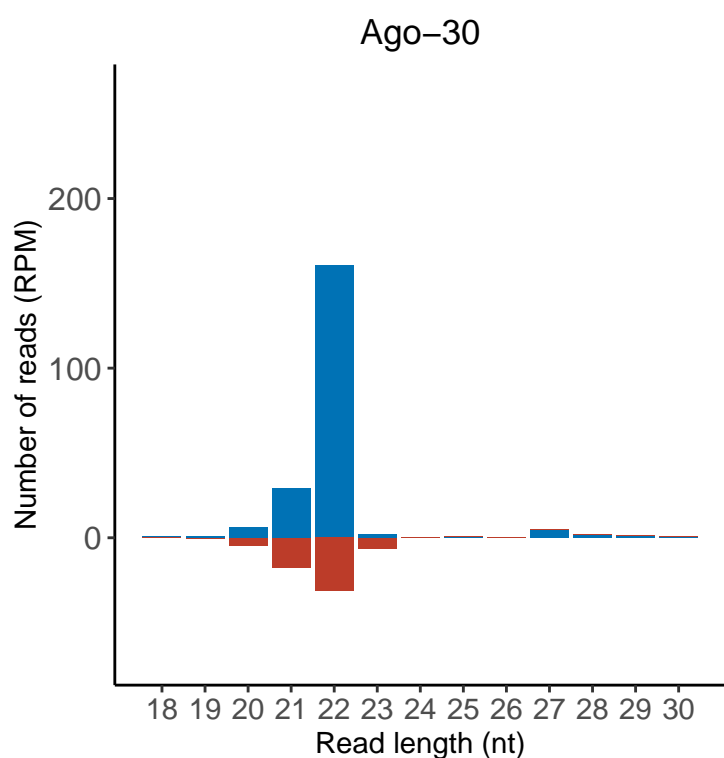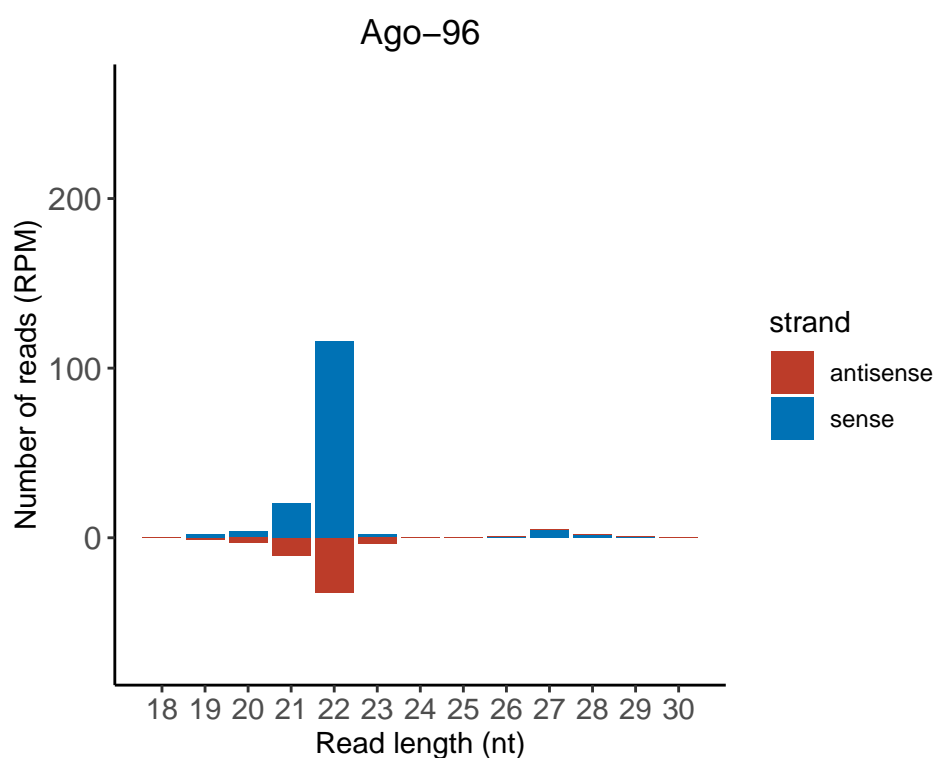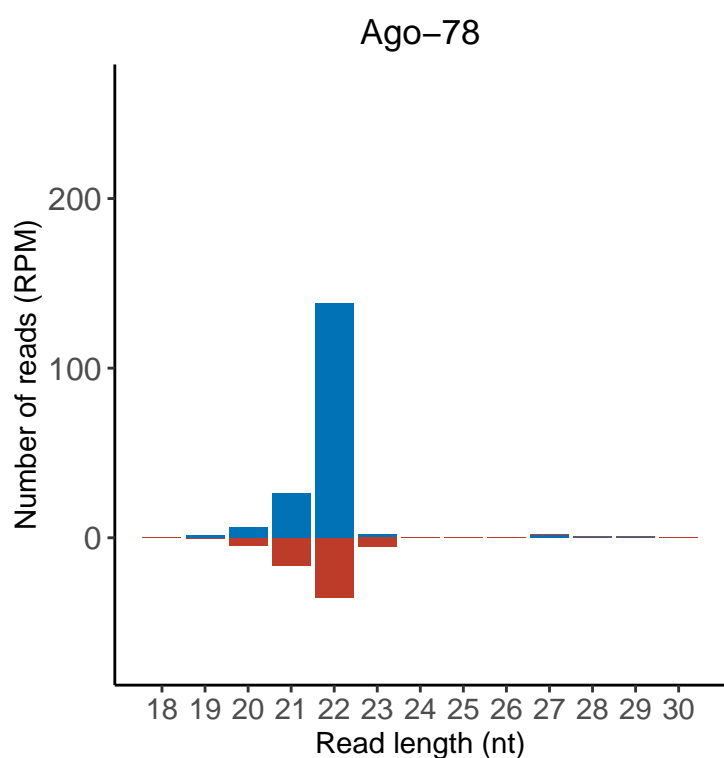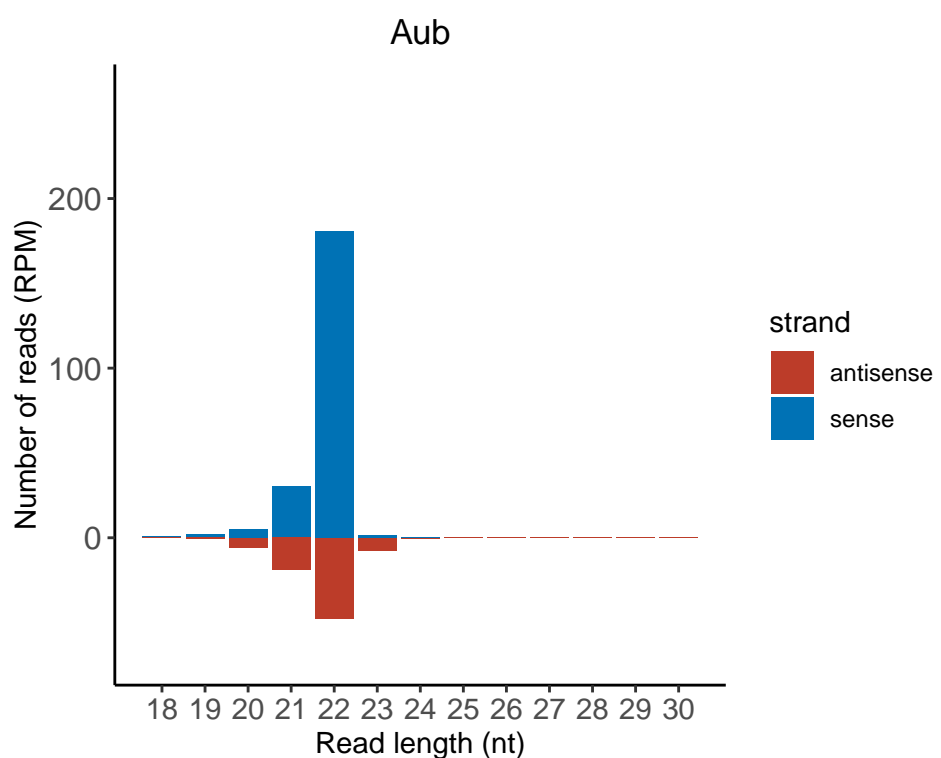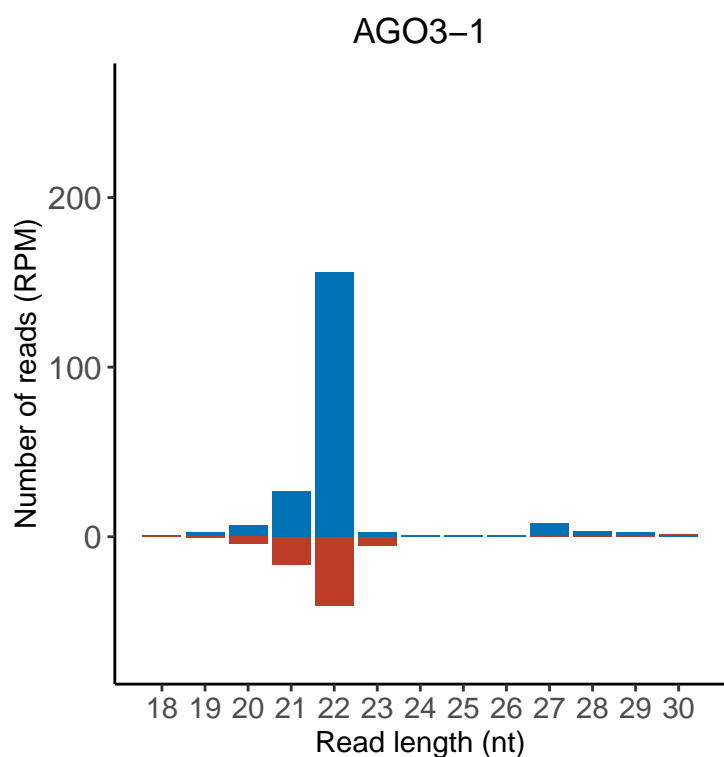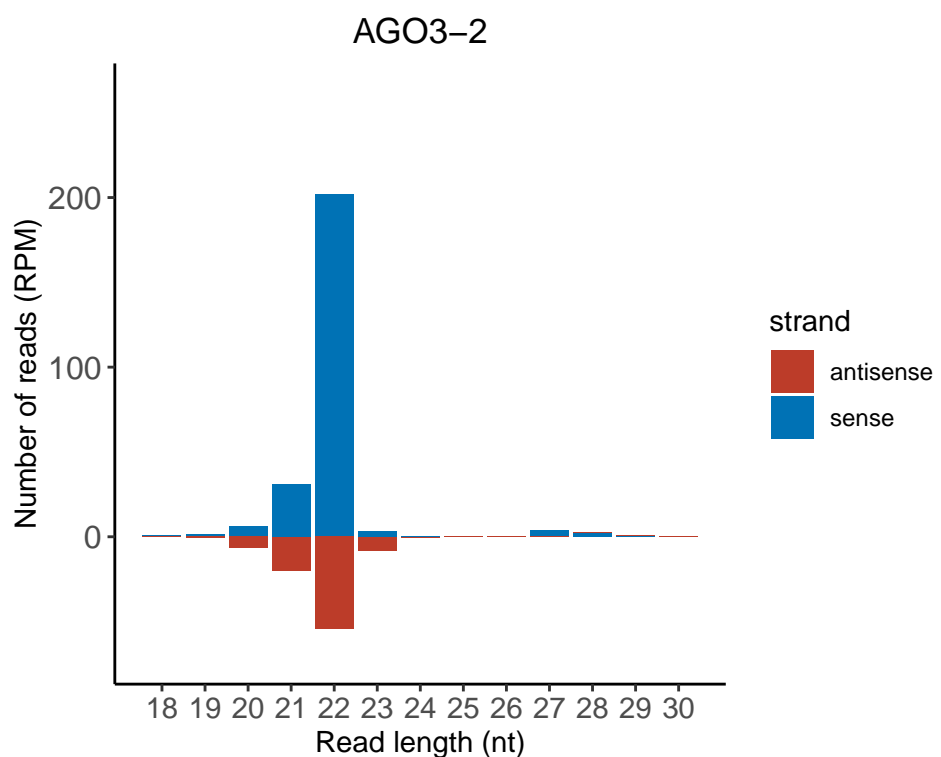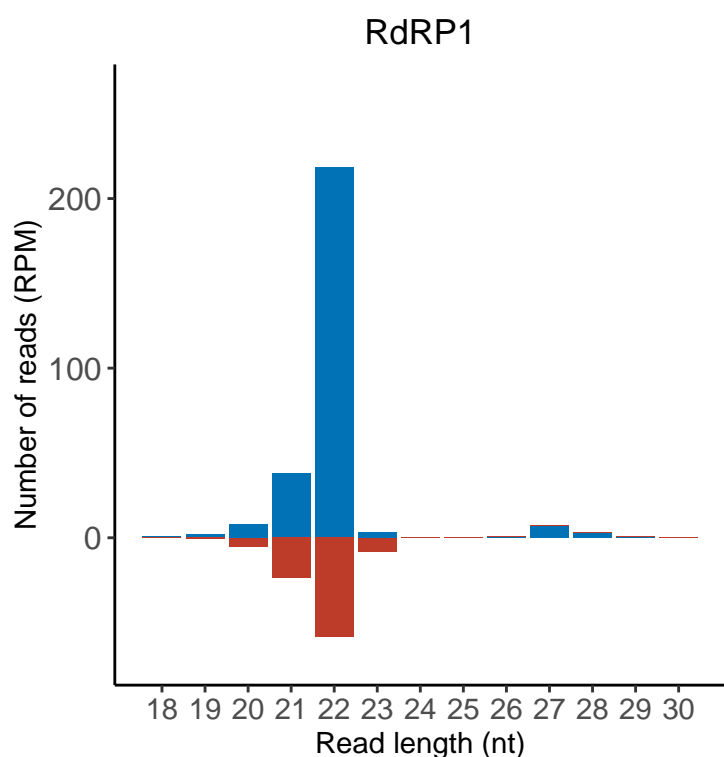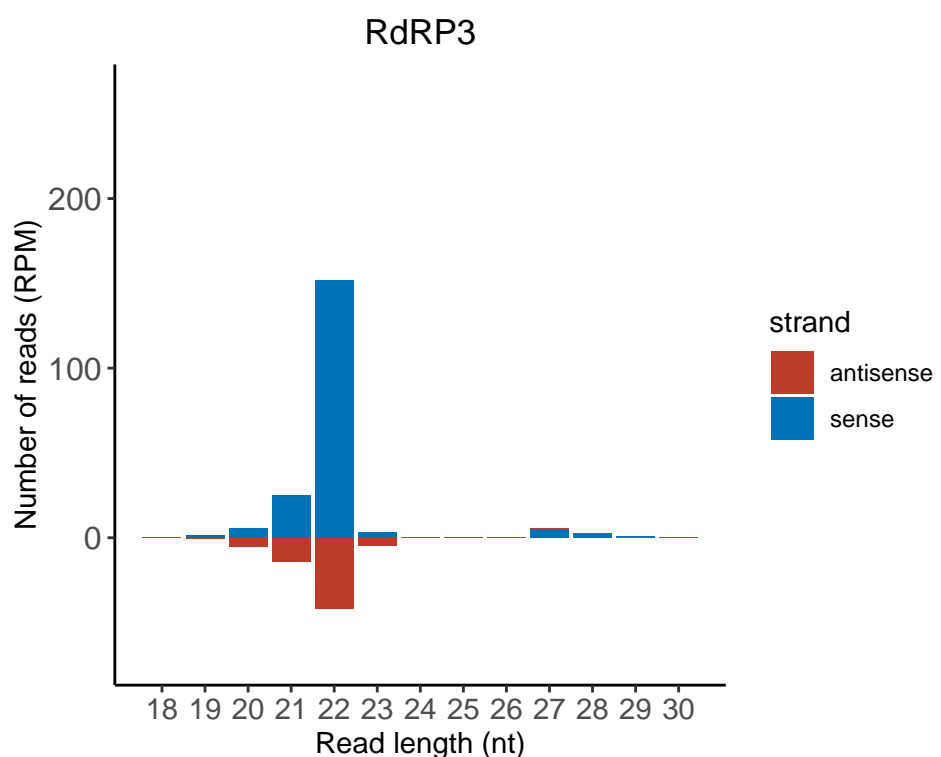

Supplement: S1 Data — On the Normalized and Relative levels pages, normalized read counts (RPM) and relative levels compared to the control (dsGFP) library were used. TEs with more than 50RPM and Coding Genes with more than 3.5RPM on average in KD libraries are included in this website. For viral sequences, reference sequences were generated by assembling sRNA sequences (See the Analysis of sRNAseq data section in Materials and methods.) and size distributions of sRNA reads mapped to each contig are shown. Reads were first grouped by their 5’ nucleotides, and reads of each length were counted. Full tables including TEs and Coding genes with fewer reads are in the “FullTable” folder. (ZIP) [file pone.0281195.s017.zip › SupplementaryData/Links/PDFs/Motif:rnd-1_family-1869TE_lengthdistribution.pdf]

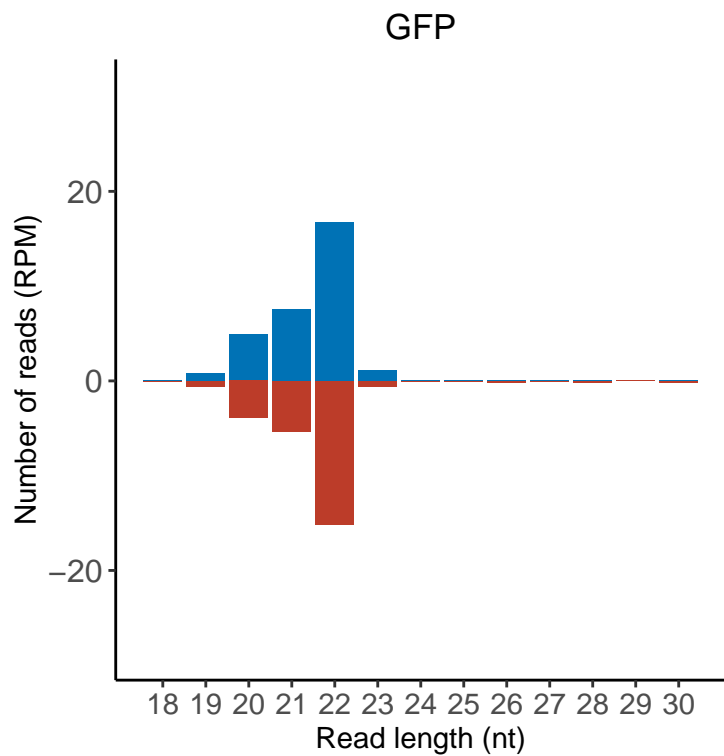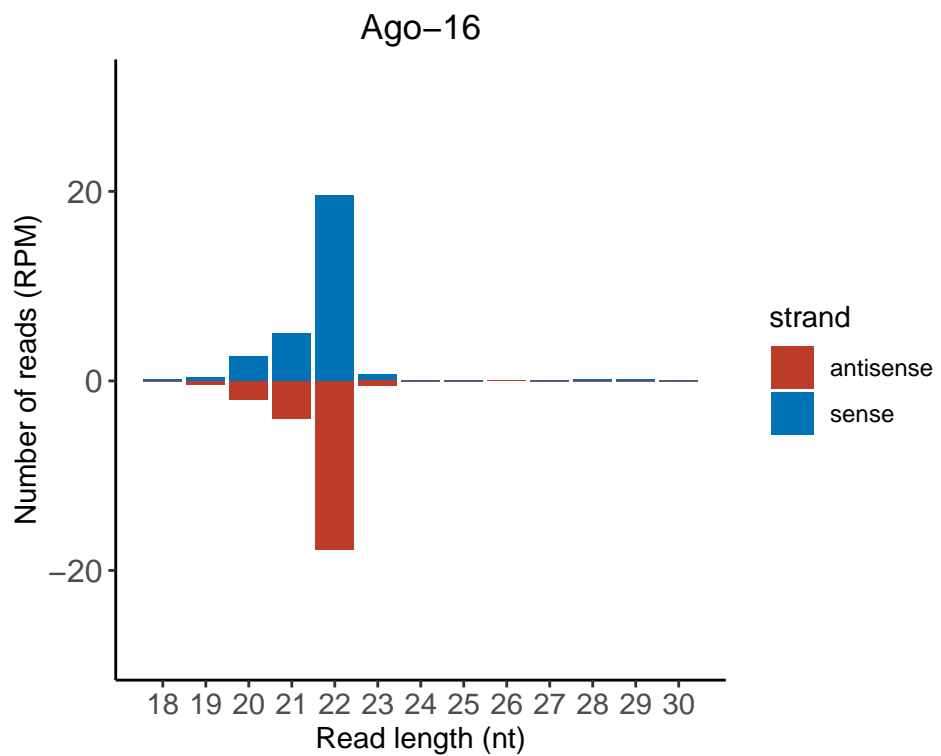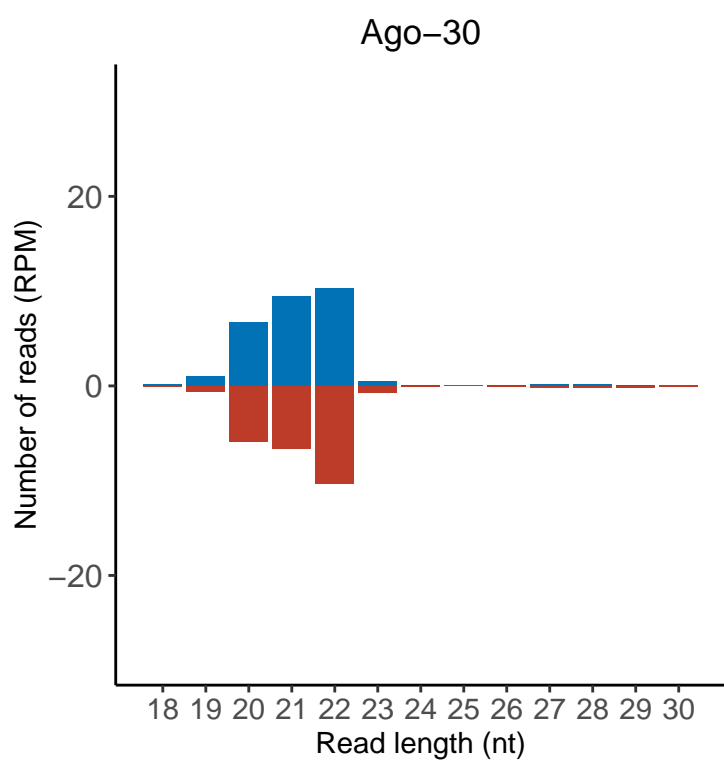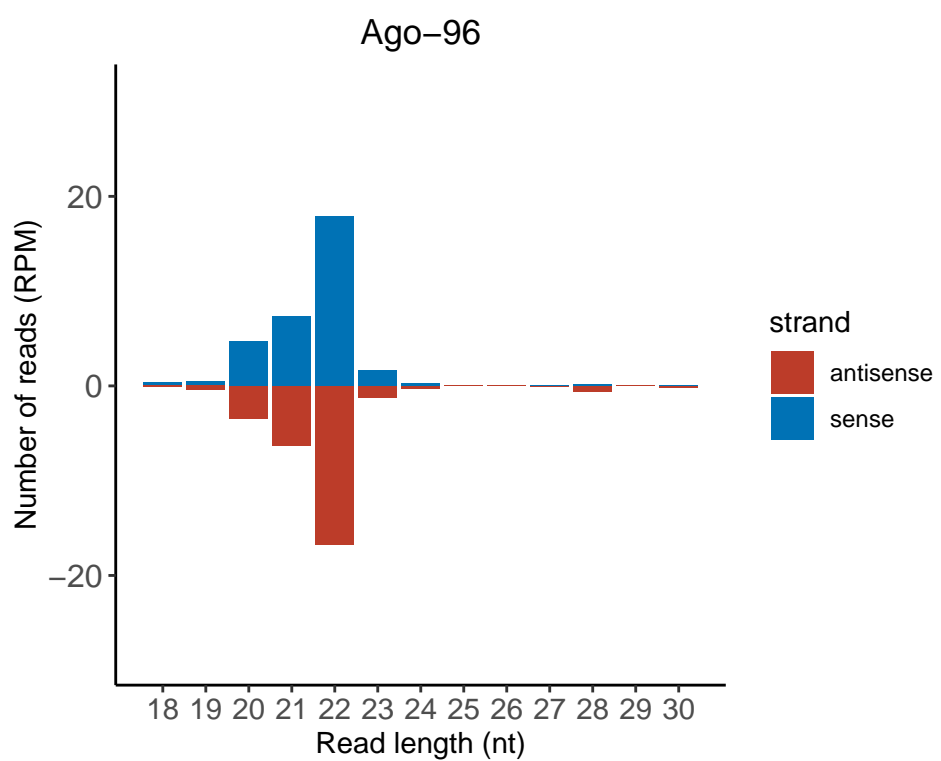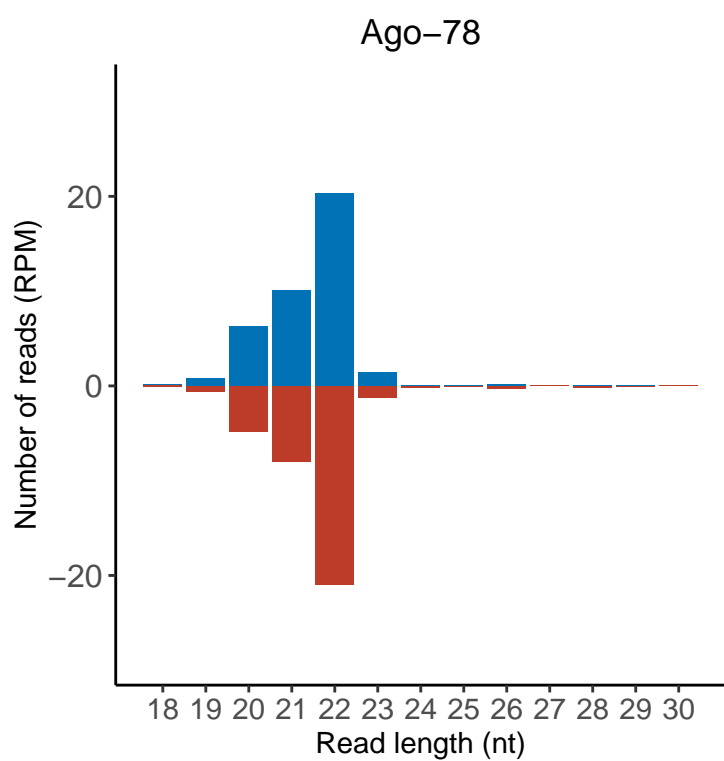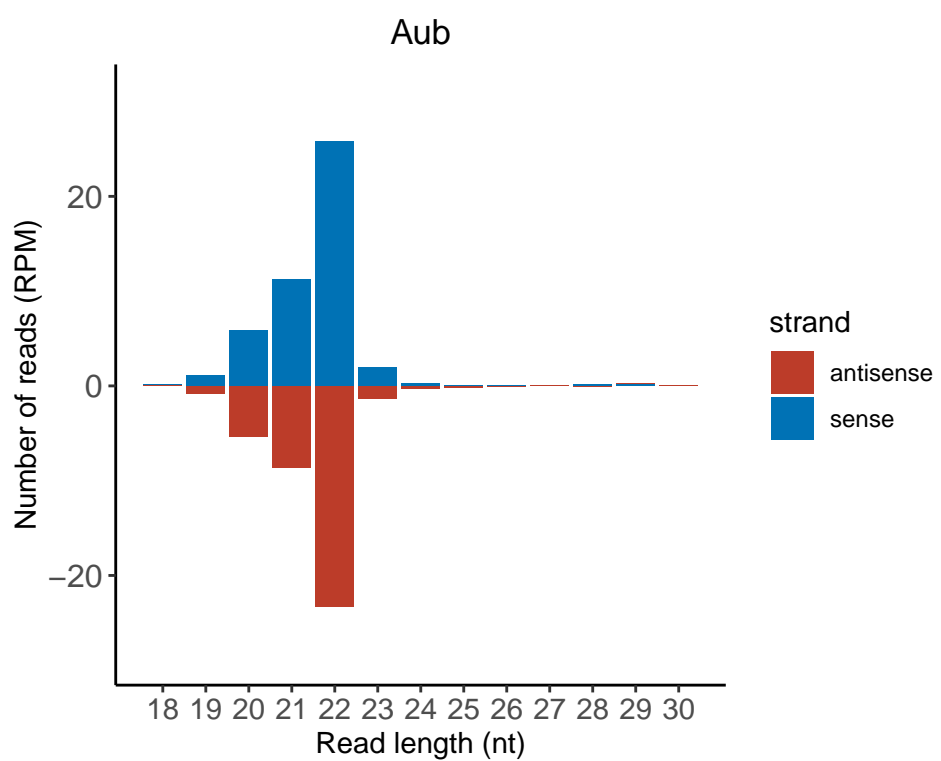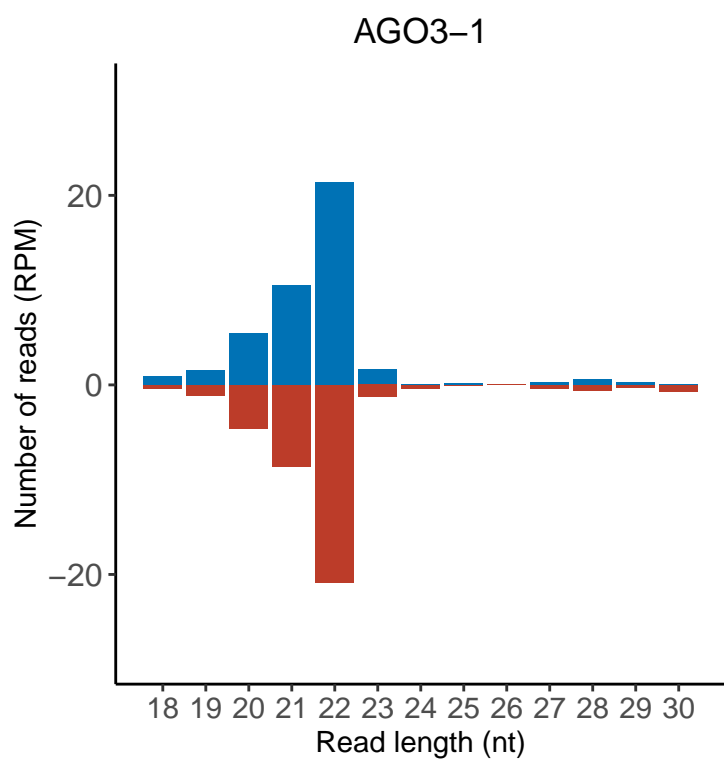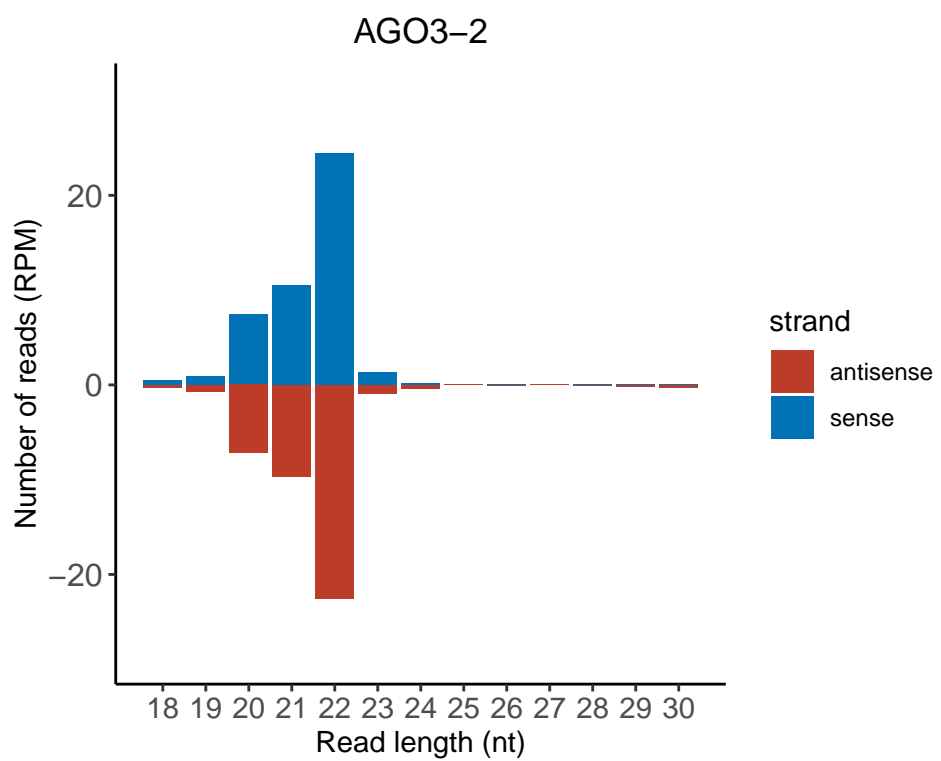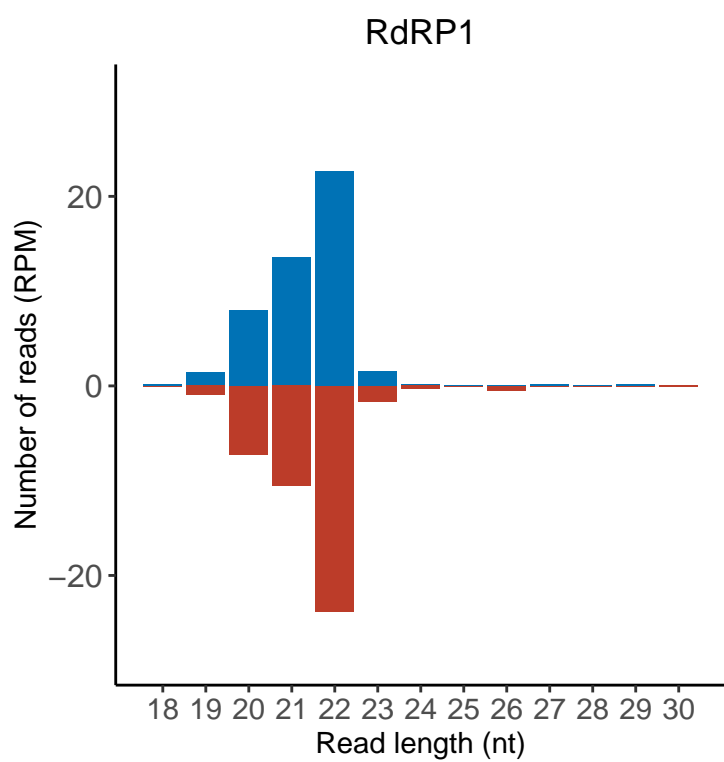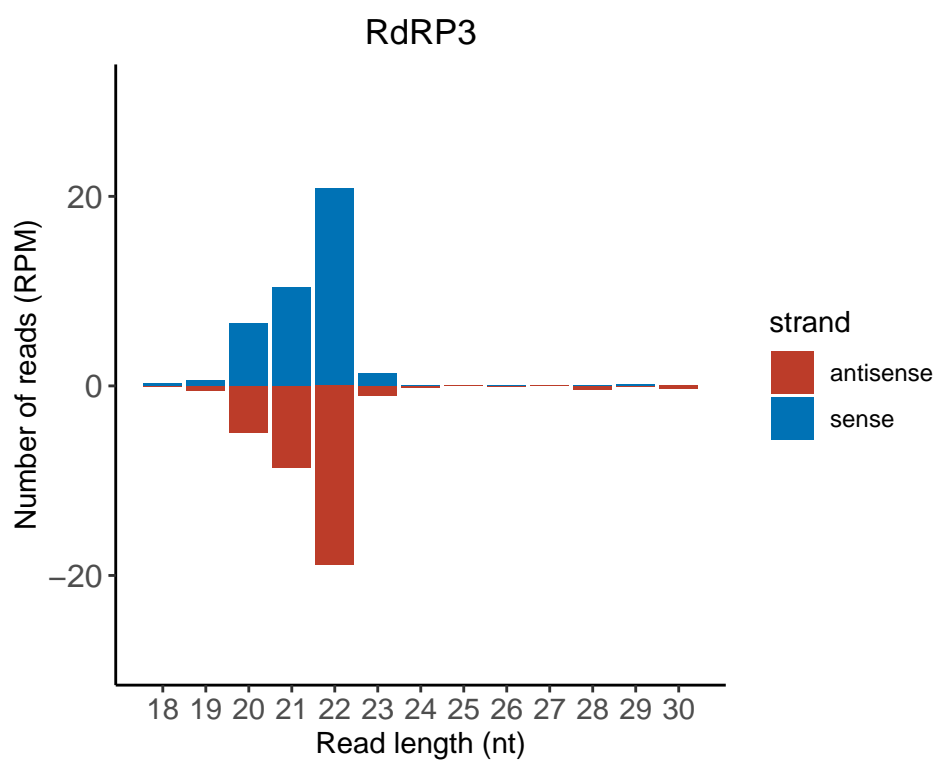

Supplement: S1 Data — On the Normalized and Relative levels pages, normalized read counts (RPM) and relative levels compared to the control (dsGFP) library were used. TEs with more than 50RPM and Coding Genes with more than 3.5RPM on average in KD libraries are included in this website. For viral sequences, reference sequences were generated by assembling sRNA sequences (See the Analysis of sRNAseq data section in Materials and methods.) and size distributions of sRNA reads mapped to each contig are shown. Reads were first grouped by their 5’ nucleotides, and reads of each length were counted. Full tables including TEs and Coding genes with fewer reads are in the “FullTable” folder. (ZIP) [file pone.0281195.s017.zip › SupplementaryData/Links/PDFs/Motif:rnd-3_family-857TE_lengthdistribution.pdf]

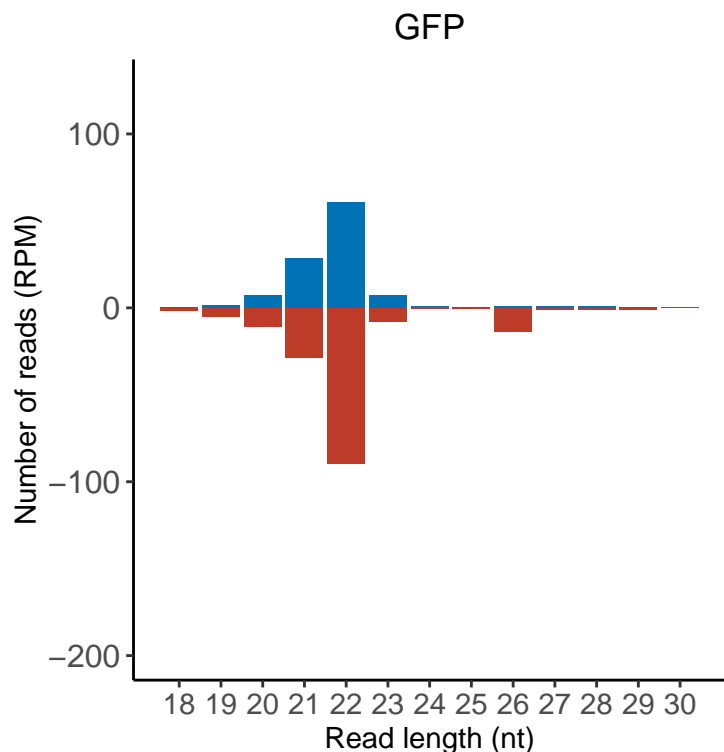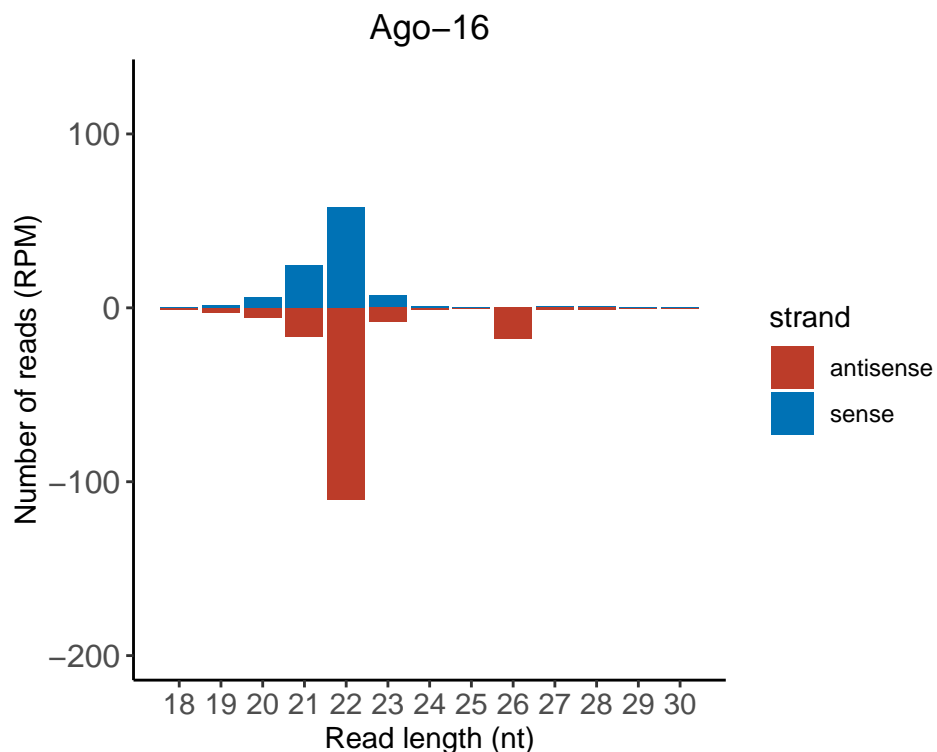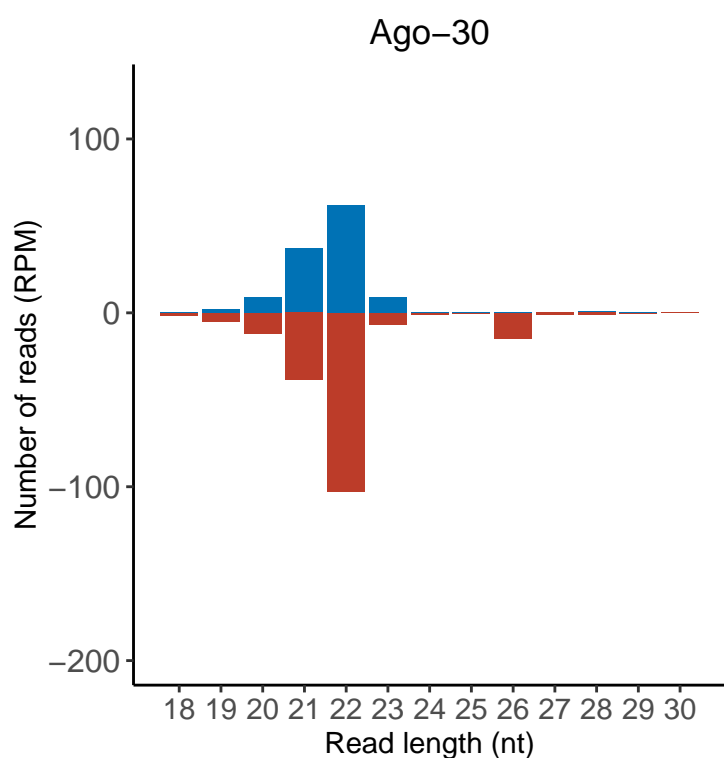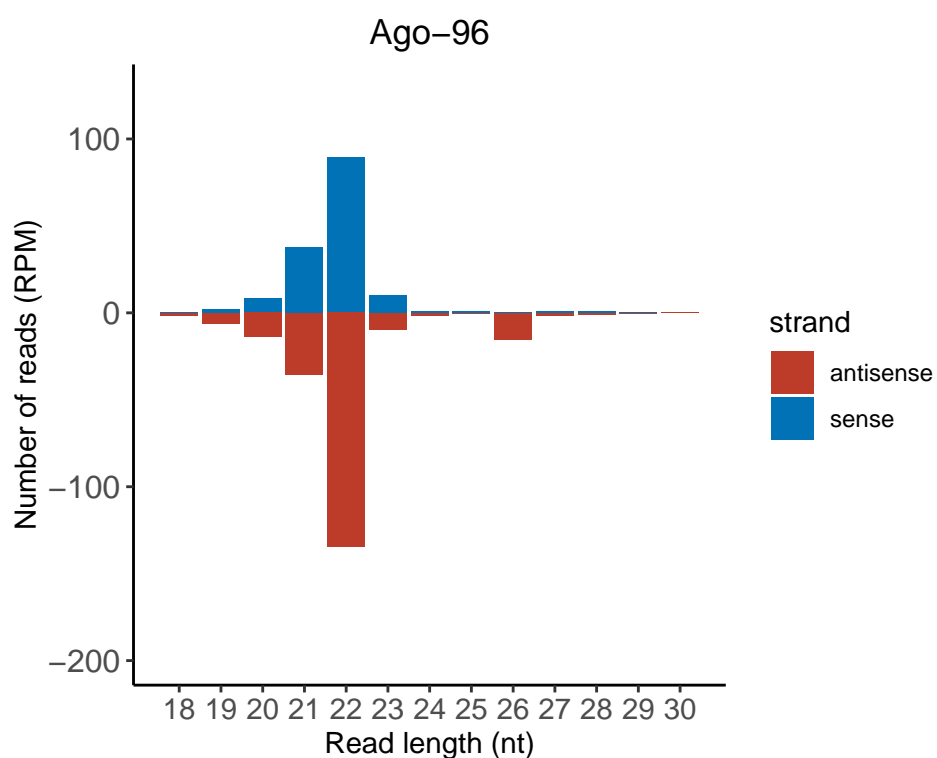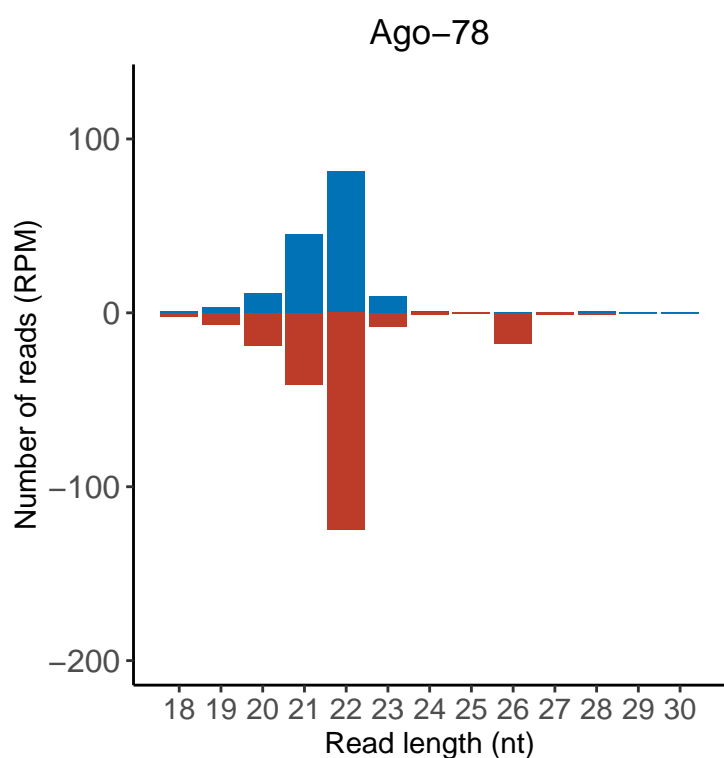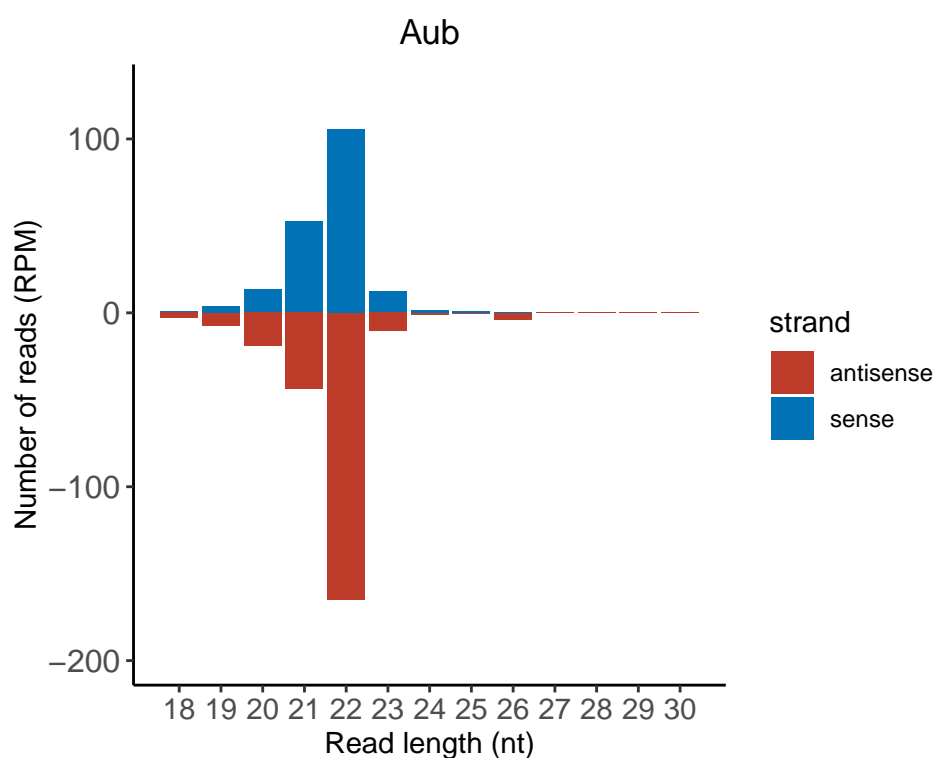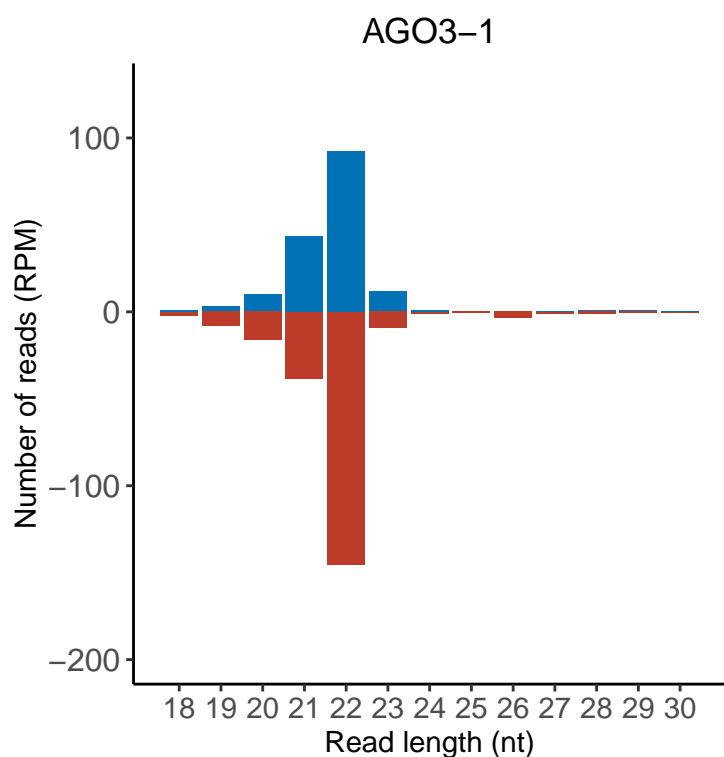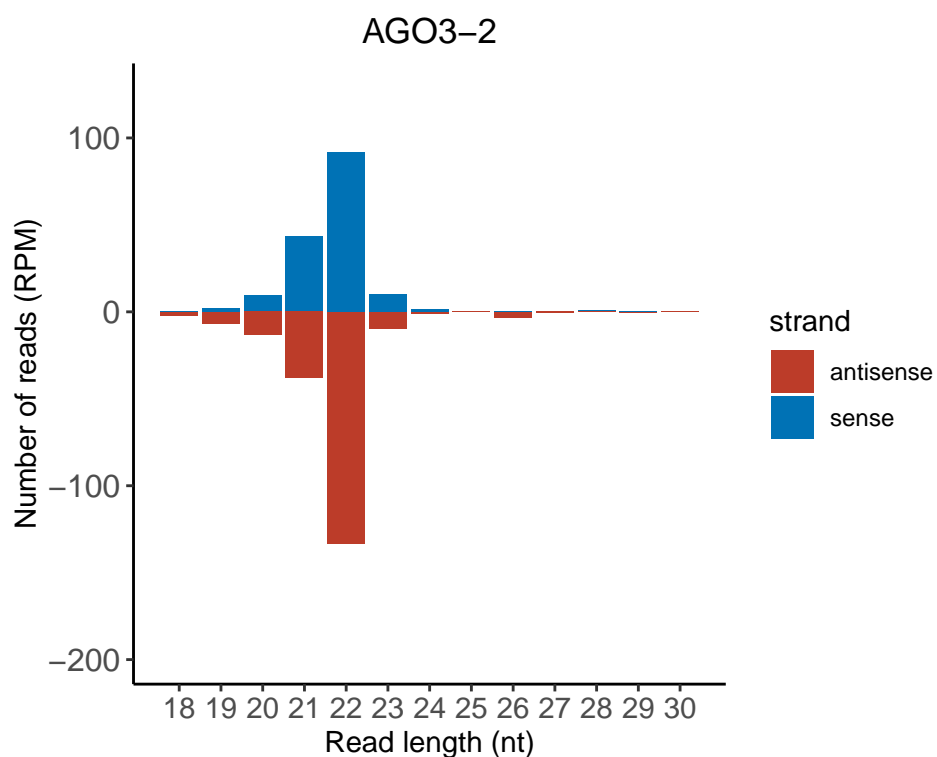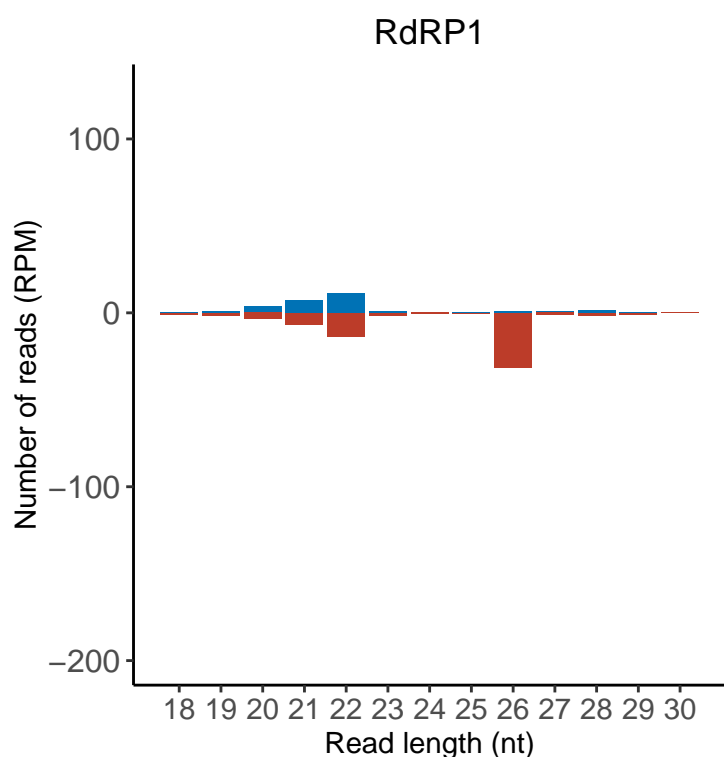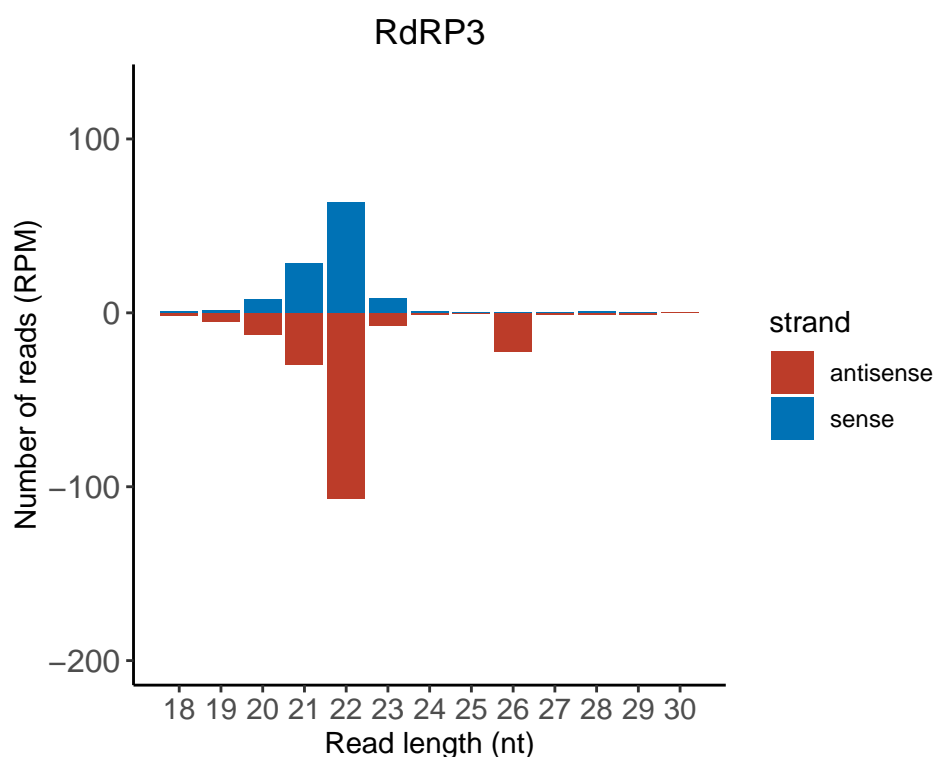

Supplement: S1 Data — On the Normalized and Relative levels pages, normalized read counts (RPM) and relative levels compared to the control (dsGFP) library were used. TEs with more than 50RPM and Coding Genes with more than 3.5RPM on average in KD libraries are included in this website. For viral sequences, reference sequences were generated by assembling sRNA sequences (See the Analysis of sRNAseq data section in Materials and methods.) and size distributions of sRNA reads mapped to each contig are shown. Reads were first grouped by their 5’ nucleotides, and reads of each length were counted. Full tables including TEs and Coding genes with fewer reads are in the “FullTable” folder. (ZIP) [file pone.0281195.s017.zip › SupplementaryData/Links/PDFs/Motif:rnd-1_family-12TE_lengthdistribution.pdf]

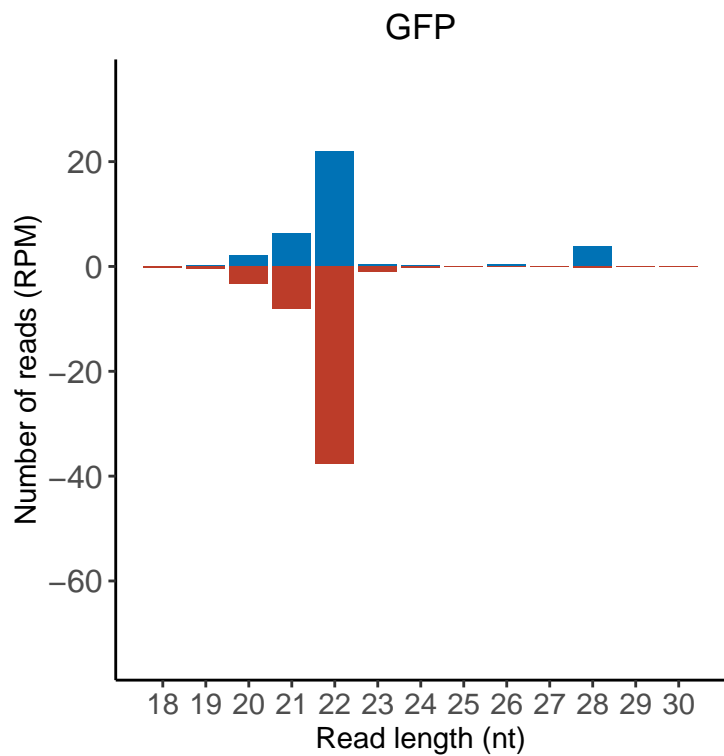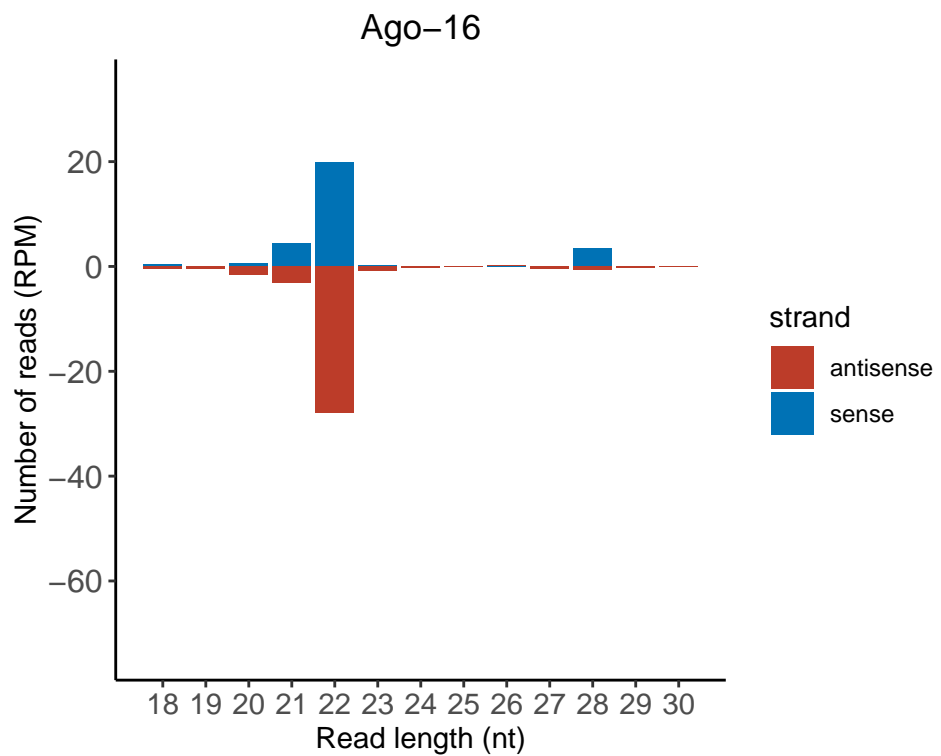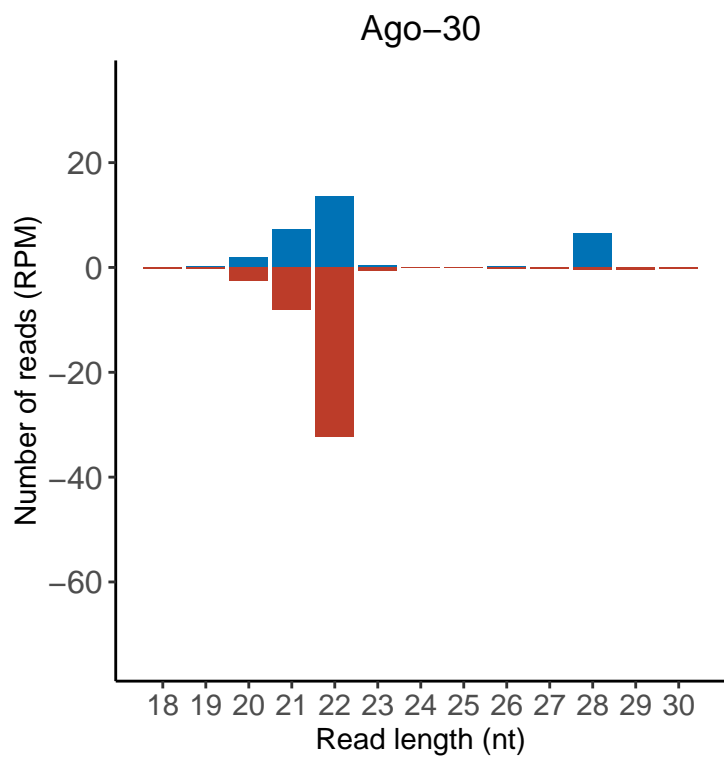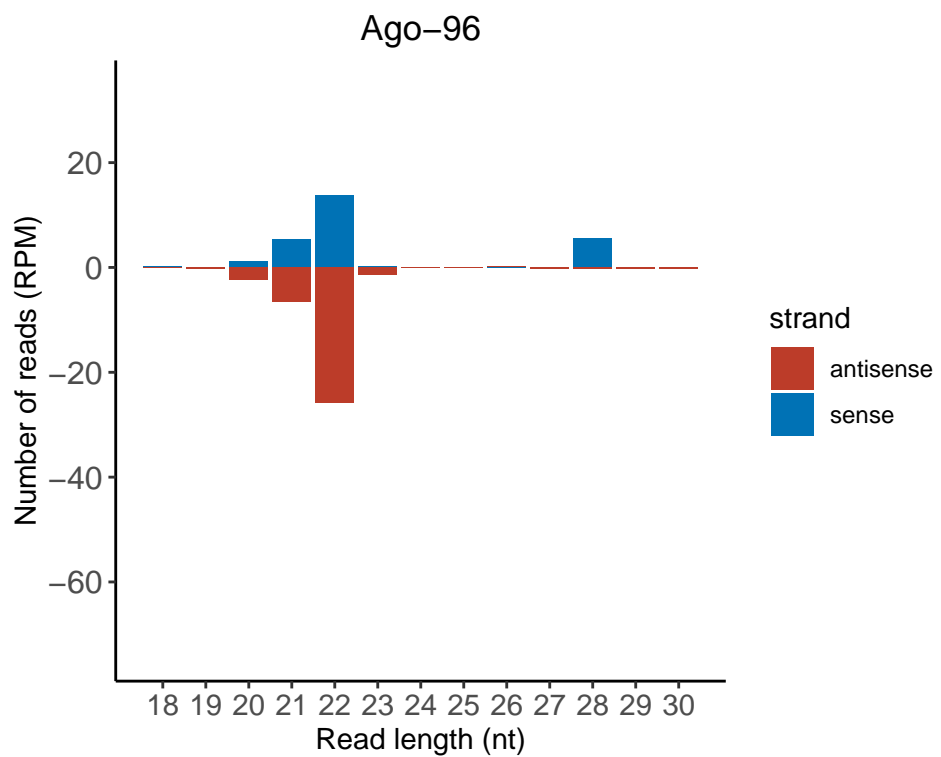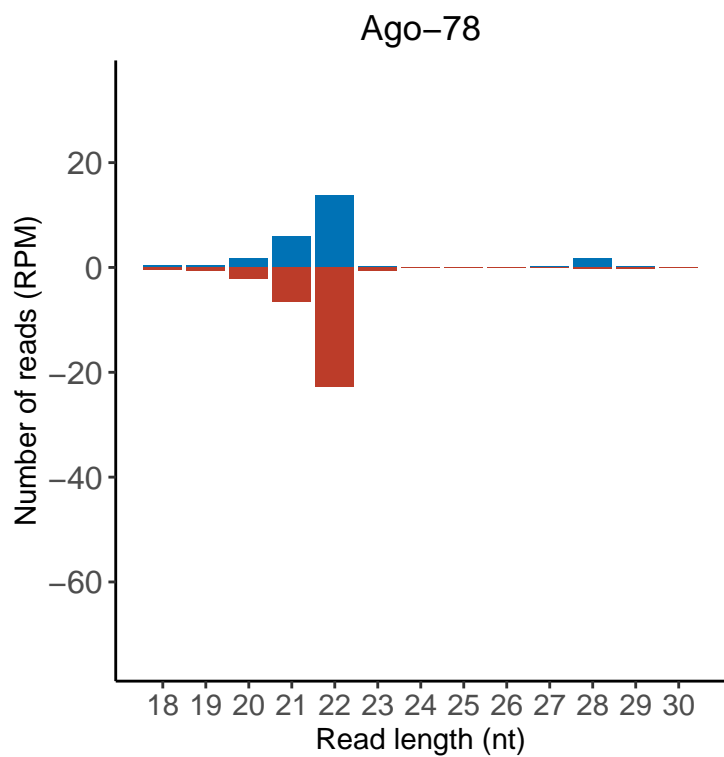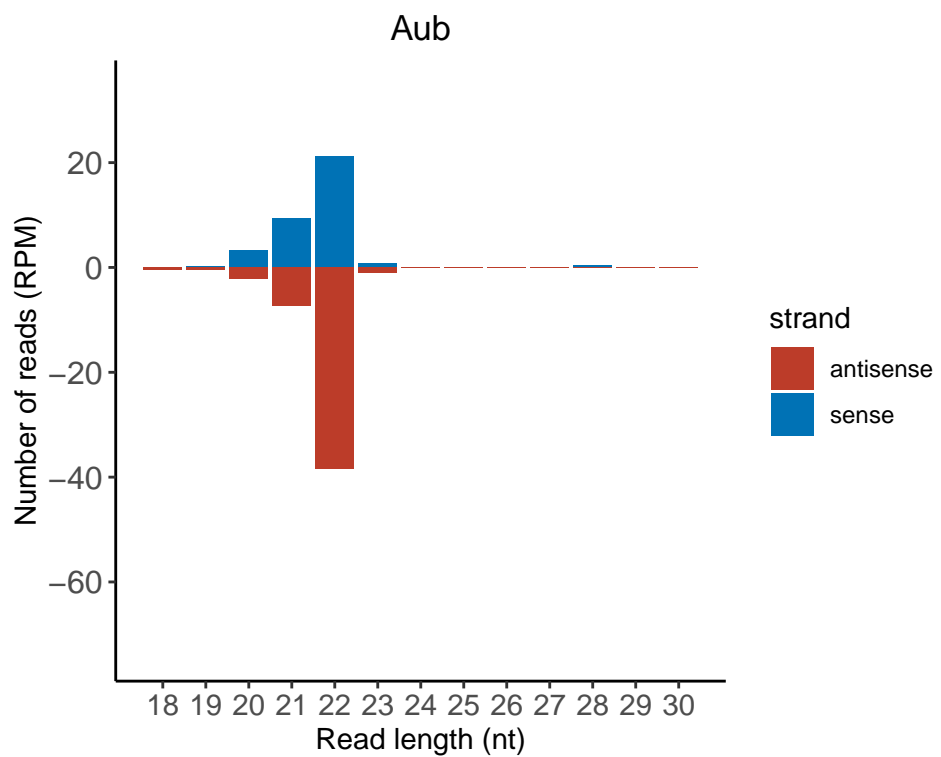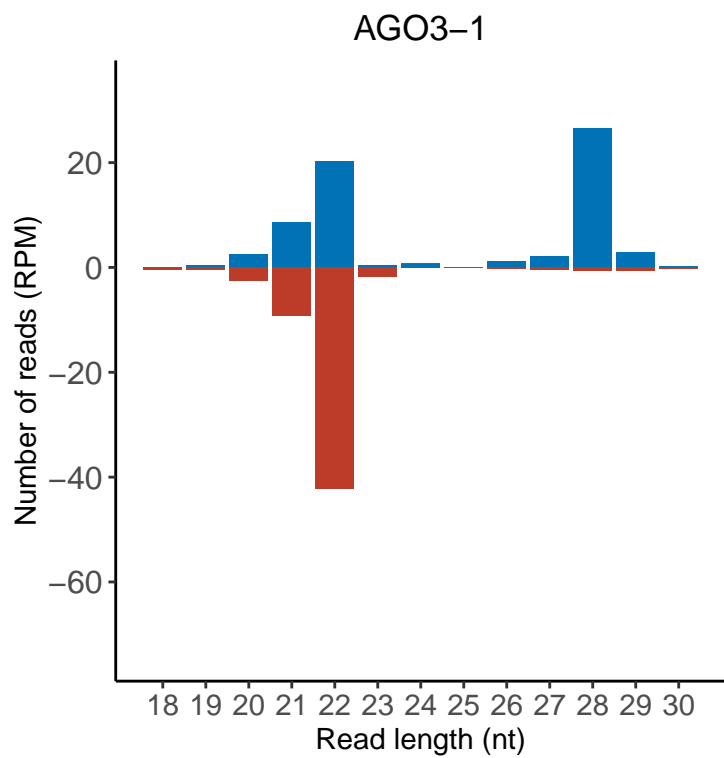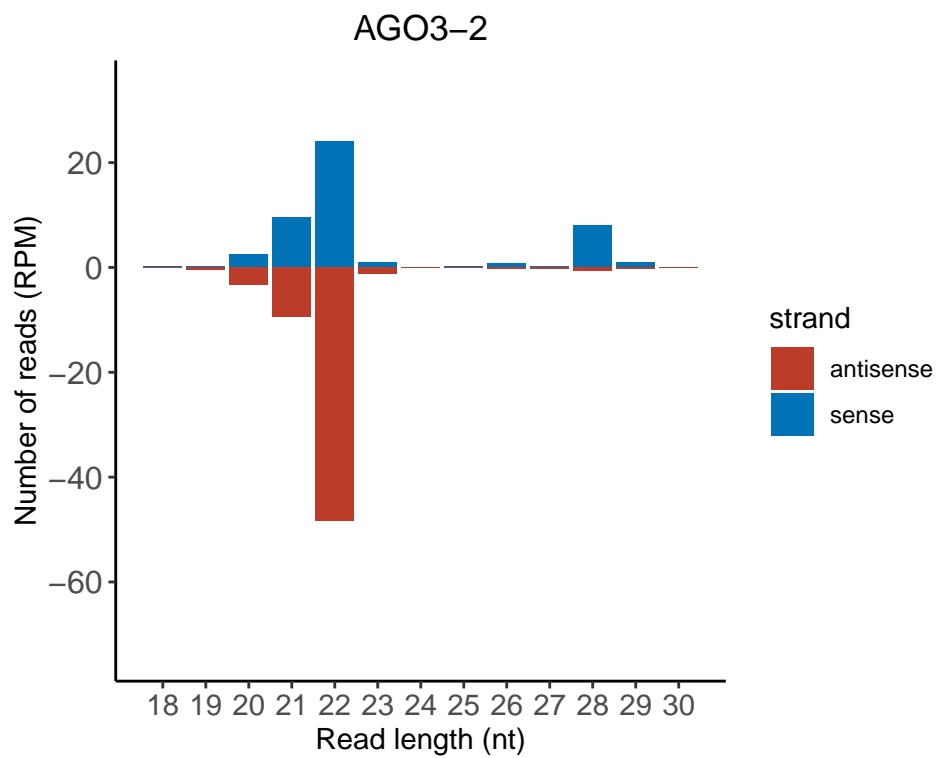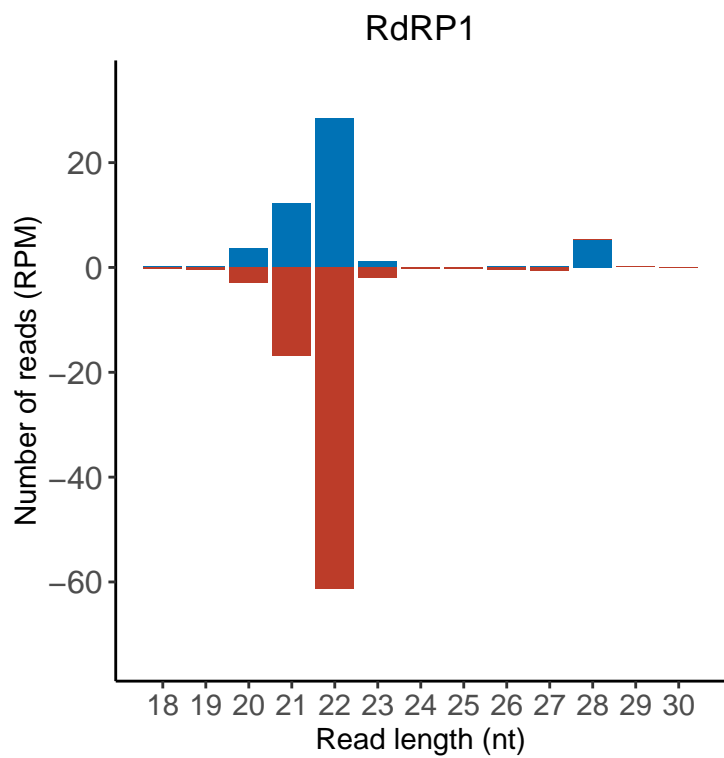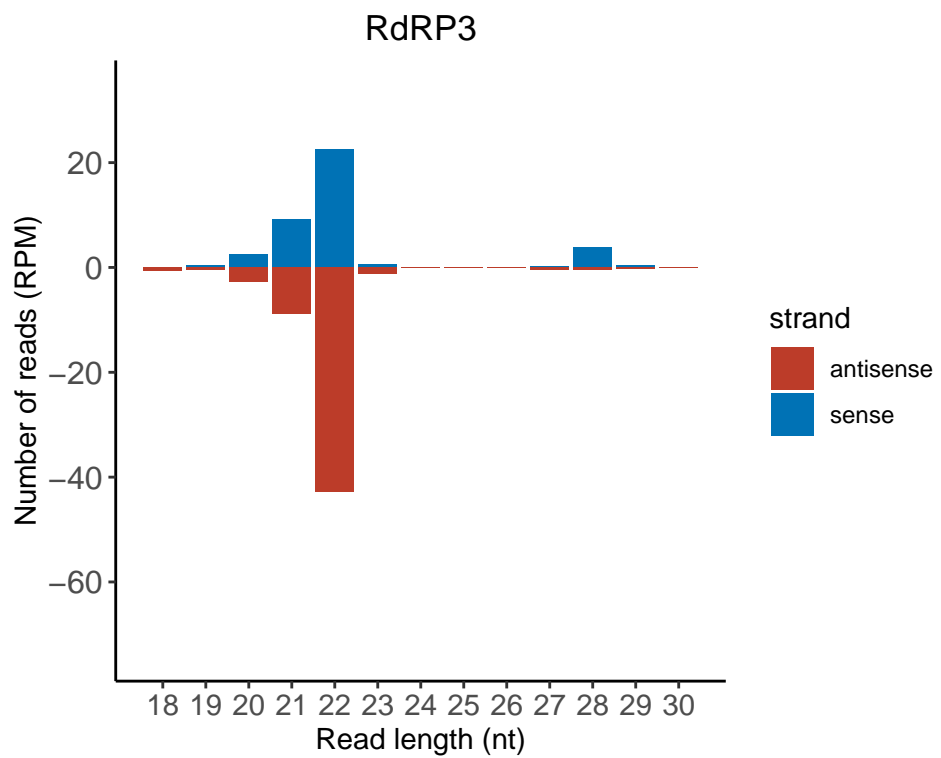

Supplement: S1 Data — On the Normalized and Relative levels pages, normalized read counts (RPM) and relative levels compared to the control (dsGFP) library were used. TEs with more than 50RPM and Coding Genes with more than 3.5RPM on average in KD libraries are included in this website. For viral sequences, reference sequences were generated by assembling sRNA sequences (See the Analysis of sRNAseq data section in Materials and methods.) and size distributions of sRNA reads mapped to each contig are shown. Reads were first grouped by their 5’ nucleotides, and reads of each length were counted. Full tables including TEs and Coding genes with fewer reads are in the “FullTable” folder. (ZIP) [file pone.0281195.s017.zip › SupplementaryData/Links/PDFs/Motif:rnd-6_family-1016TE_lengthdistribution.pdf]

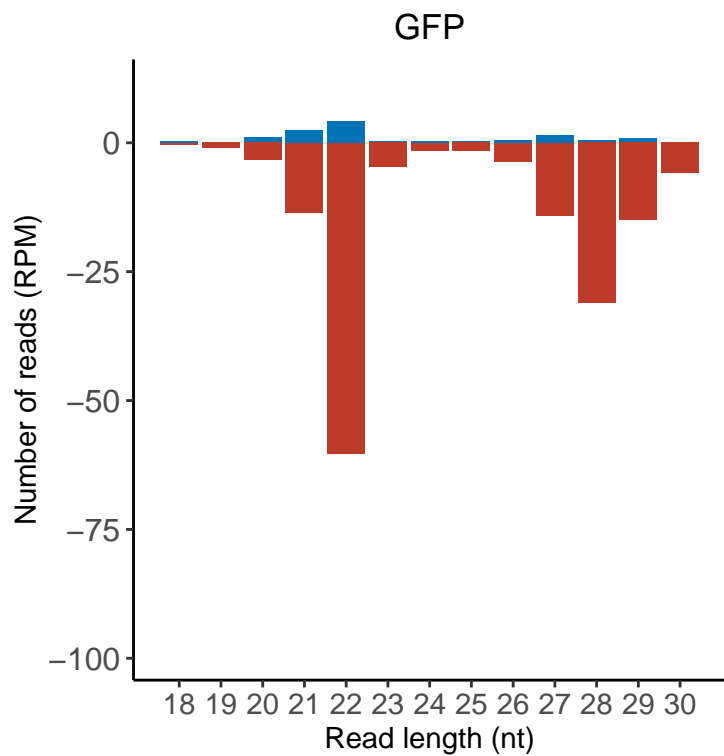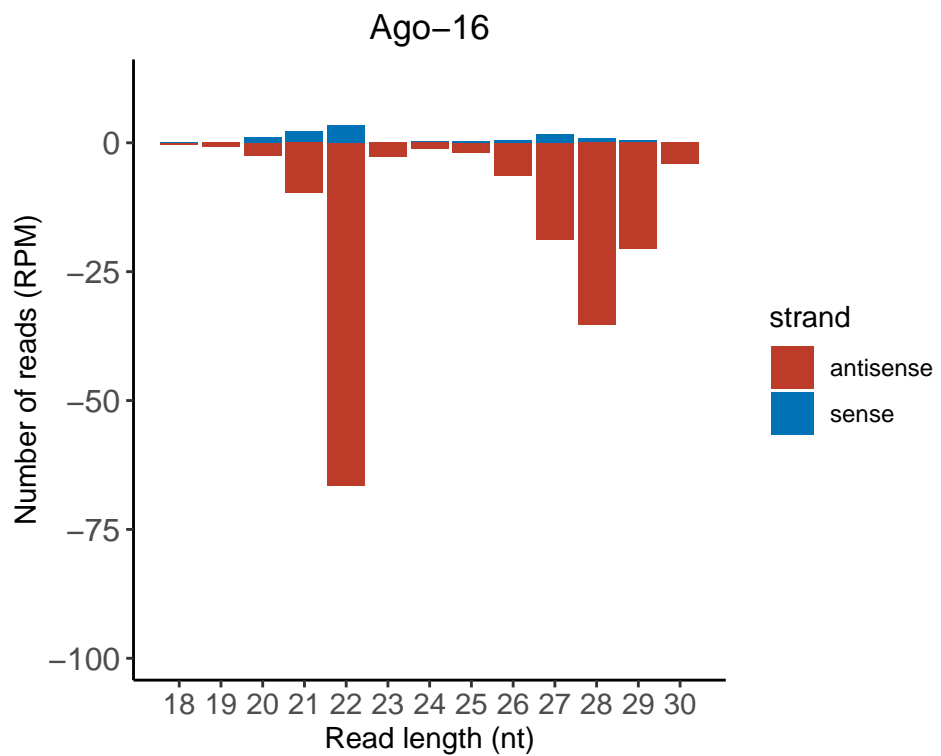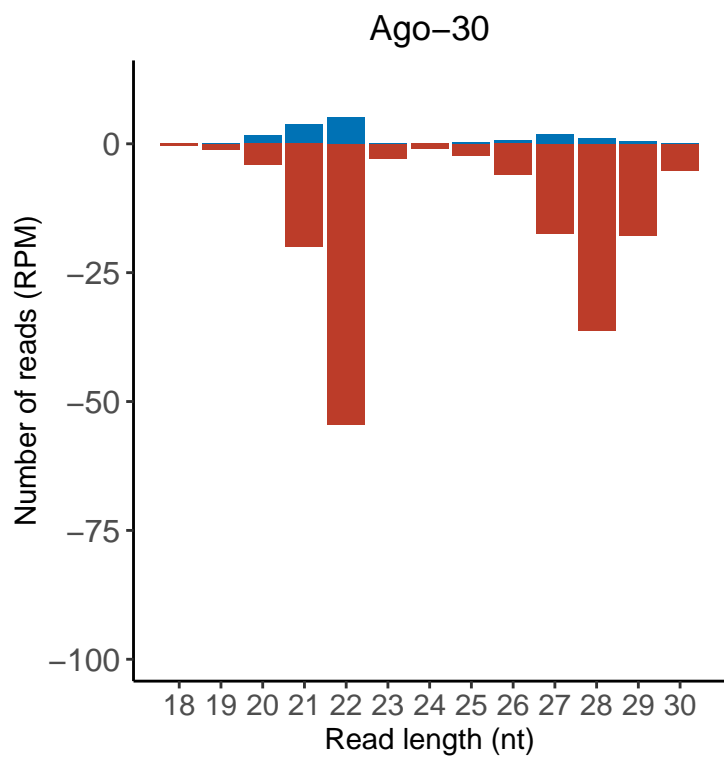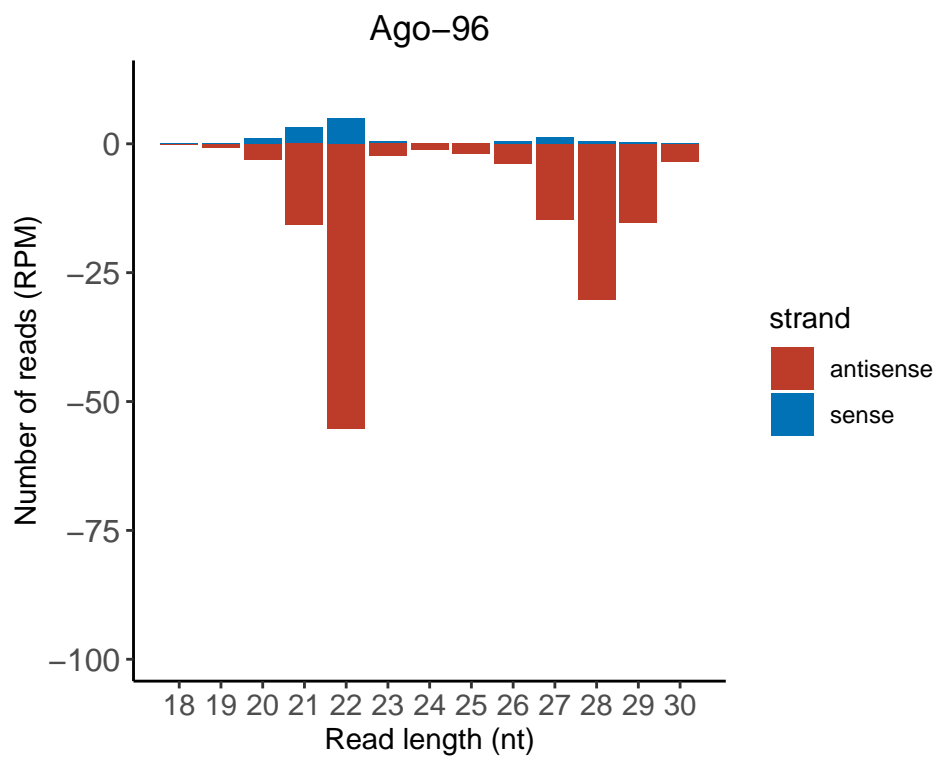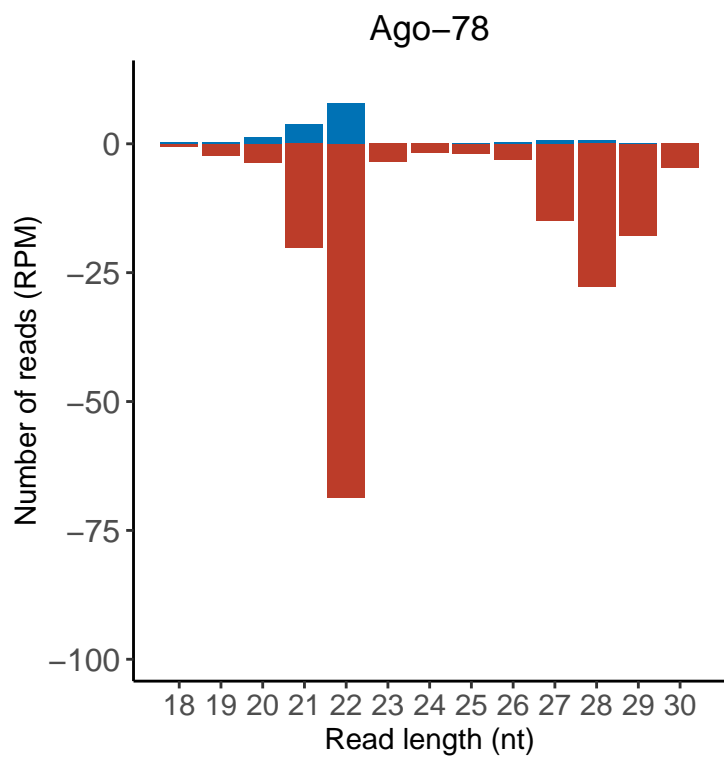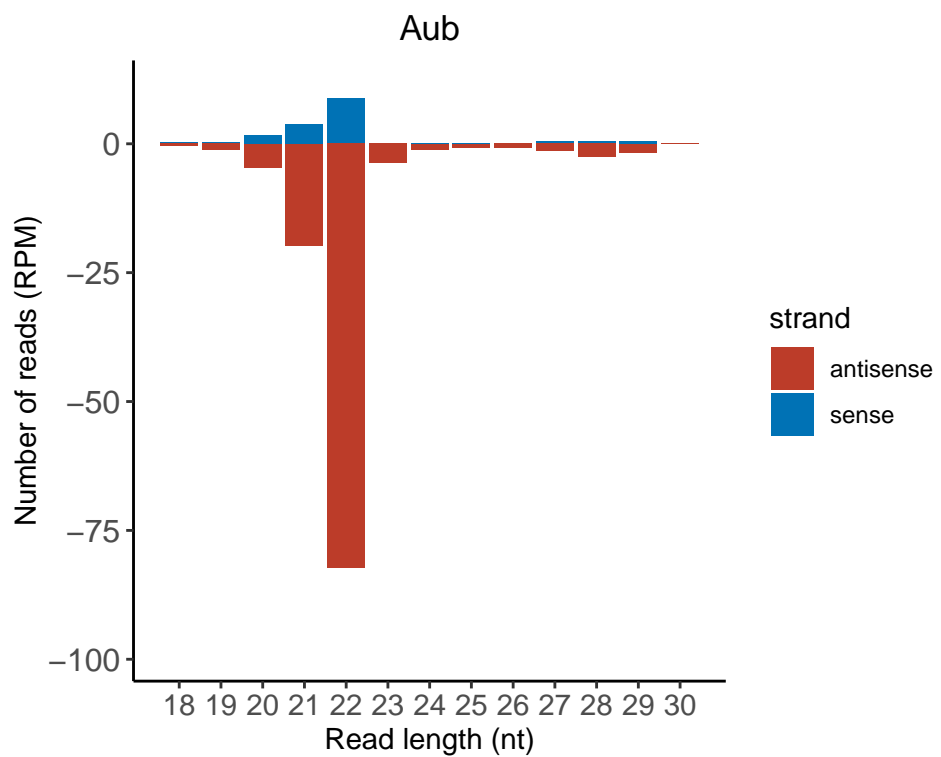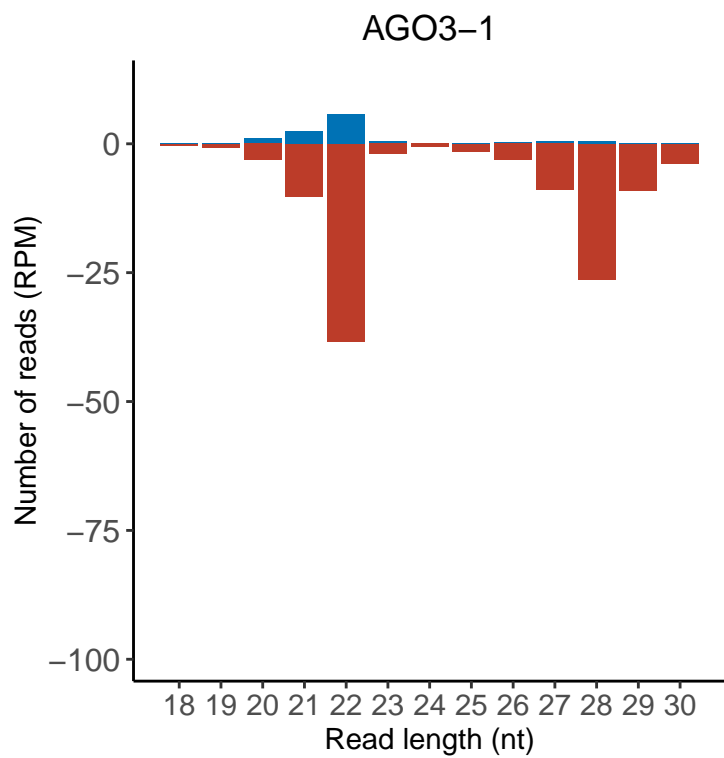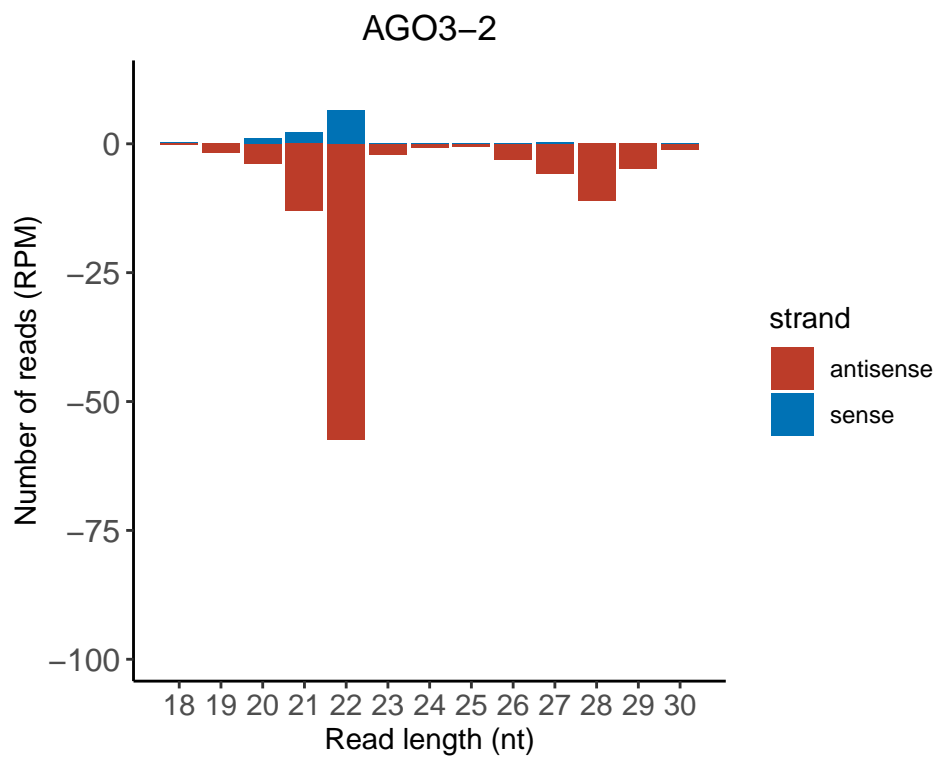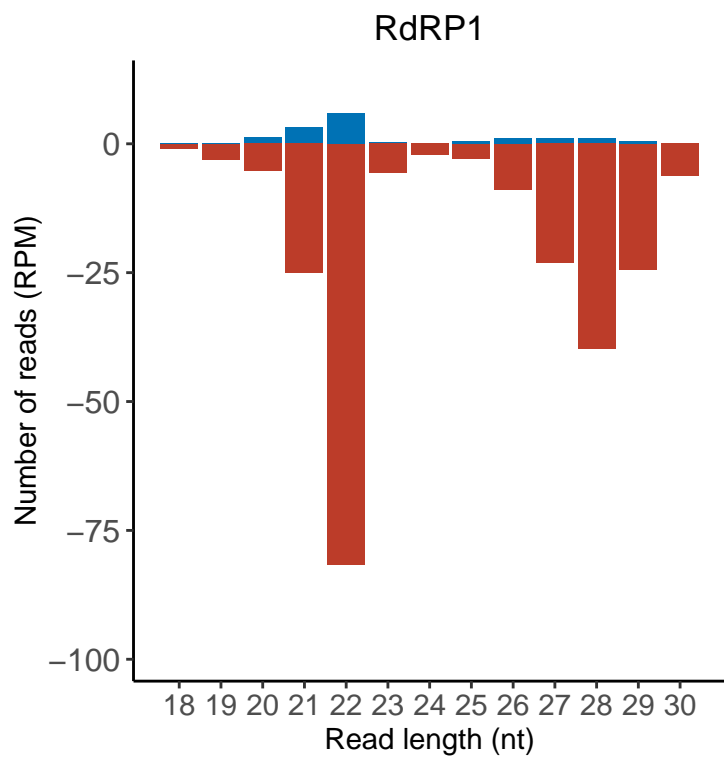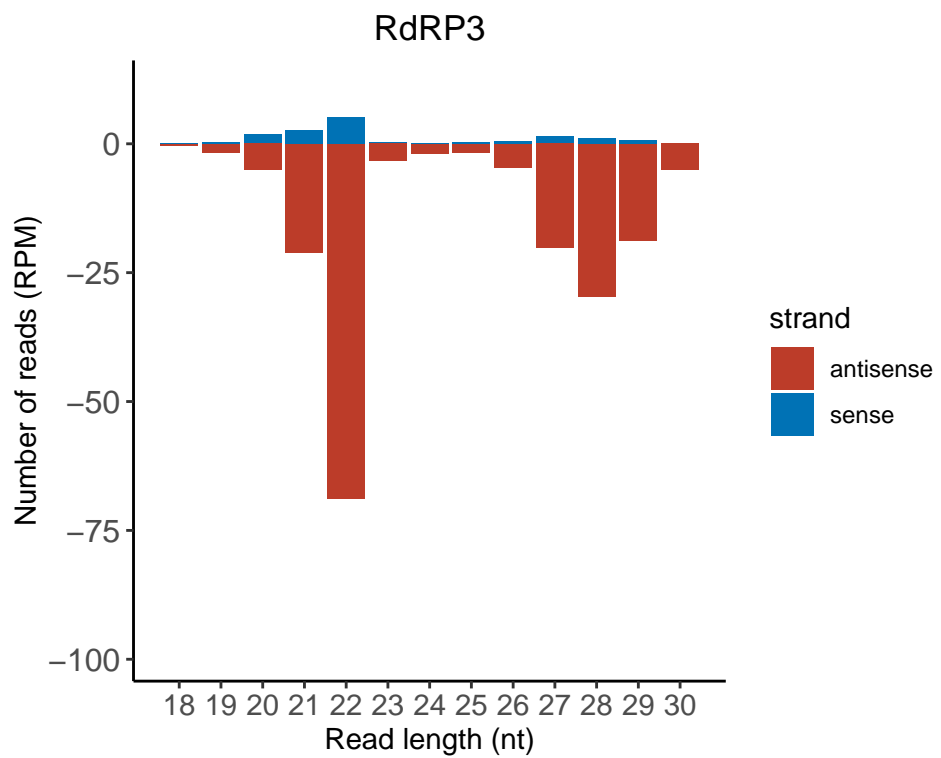

Supplement: S1 Data — On the Normalized and Relative levels pages, normalized read counts (RPM) and relative levels compared to the control (dsGFP) library were used. TEs with more than 50RPM and Coding Genes with more than 3.5RPM on average in KD libraries are included in this website. For viral sequences, reference sequences were generated by assembling sRNA sequences (See the Analysis of sRNAseq data section in Materials and methods.) and size distributions of sRNA reads mapped to each contig are shown. Reads were first grouped by their 5’ nucleotides, and reads of each length were counted. Full tables including TEs and Coding genes with fewer reads are in the “FullTable” folder. (ZIP) [file pone.0281195.s017.zip › SupplementaryData/Links/PDFs/Motif:rnd-1_family-274TE_lengthdistribution.pdf]

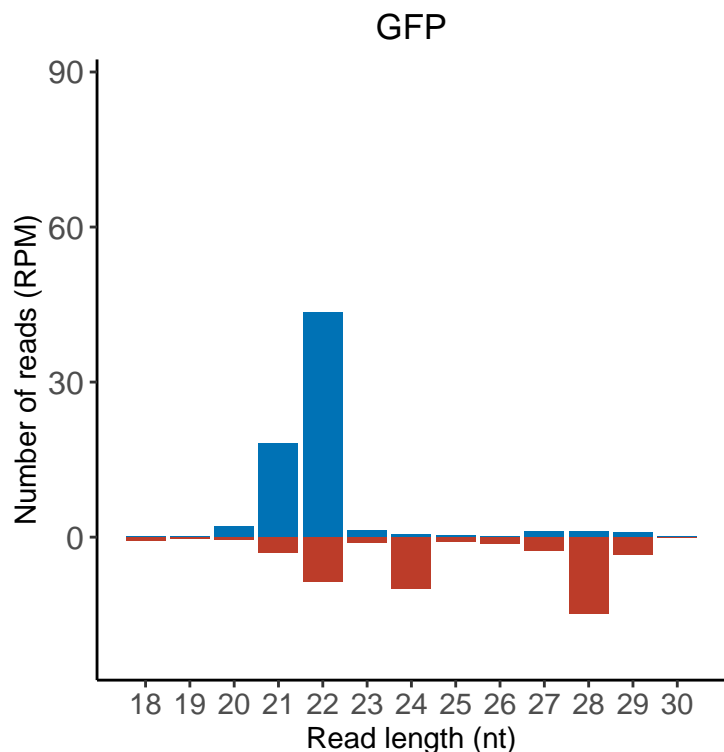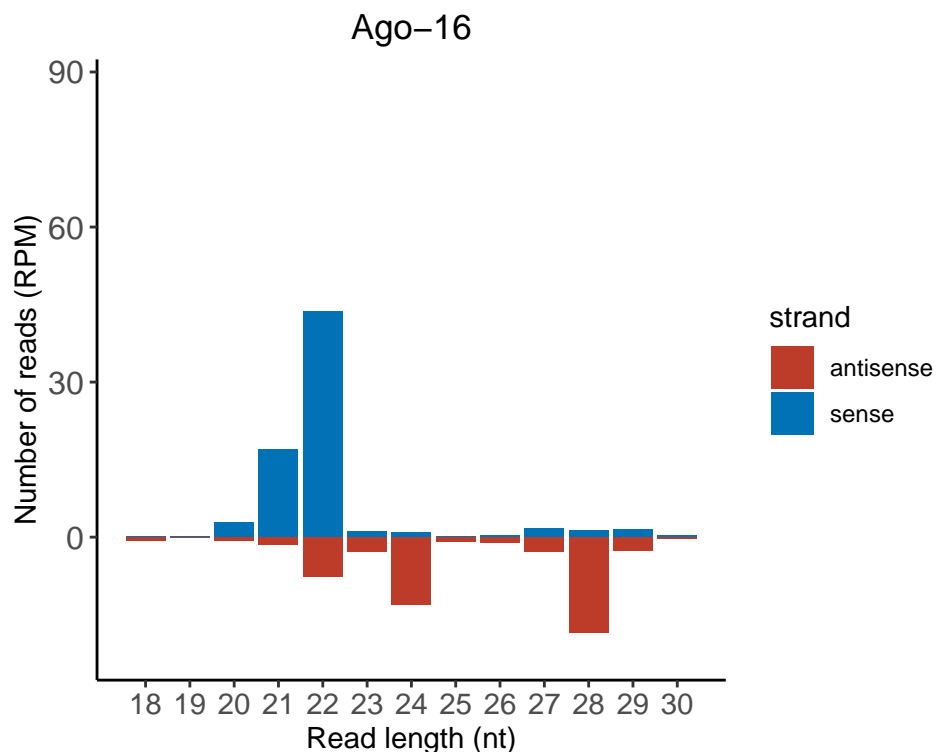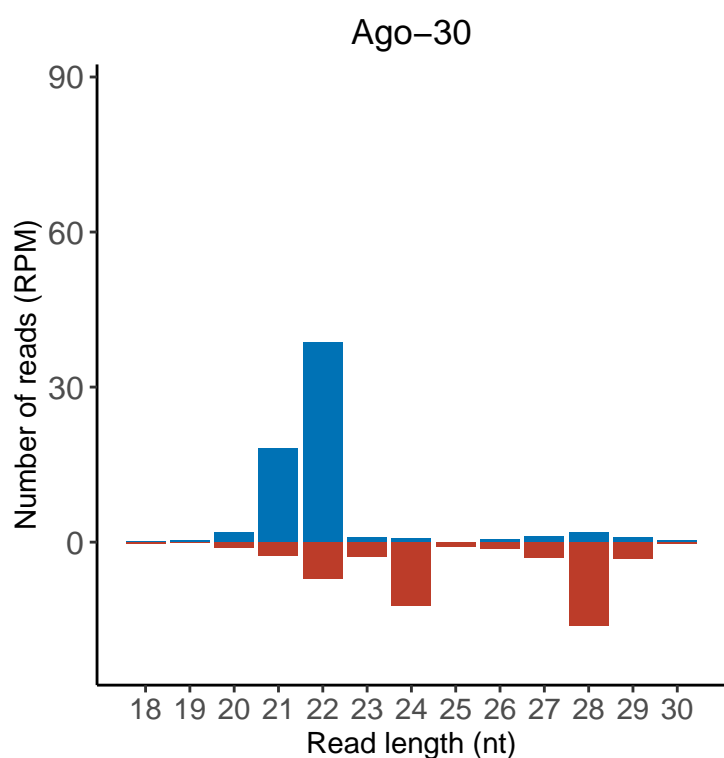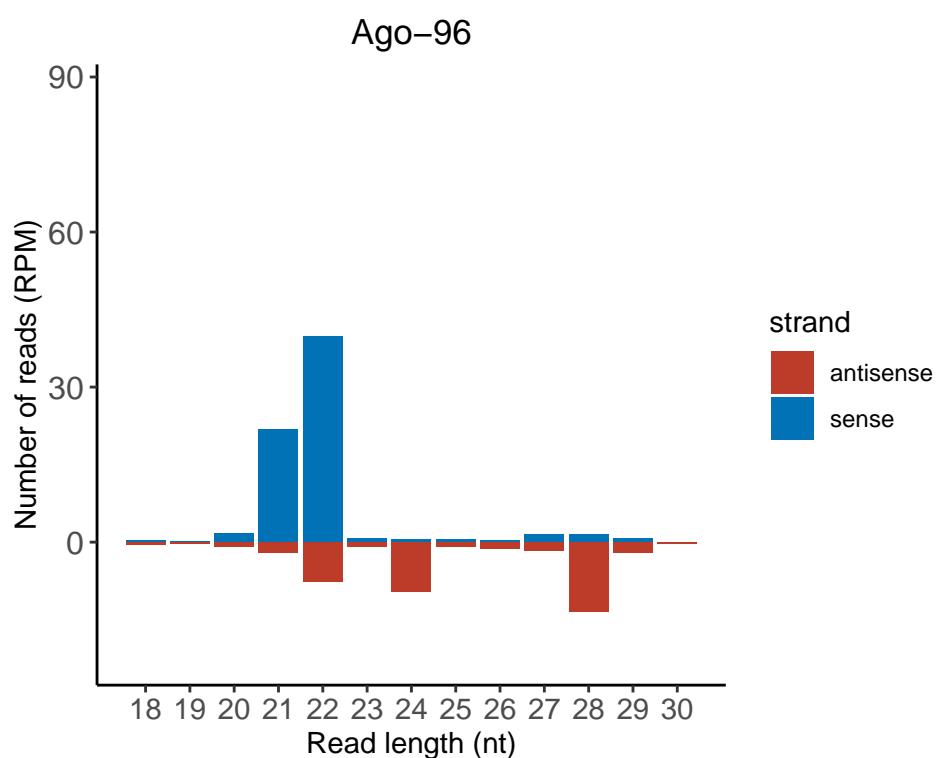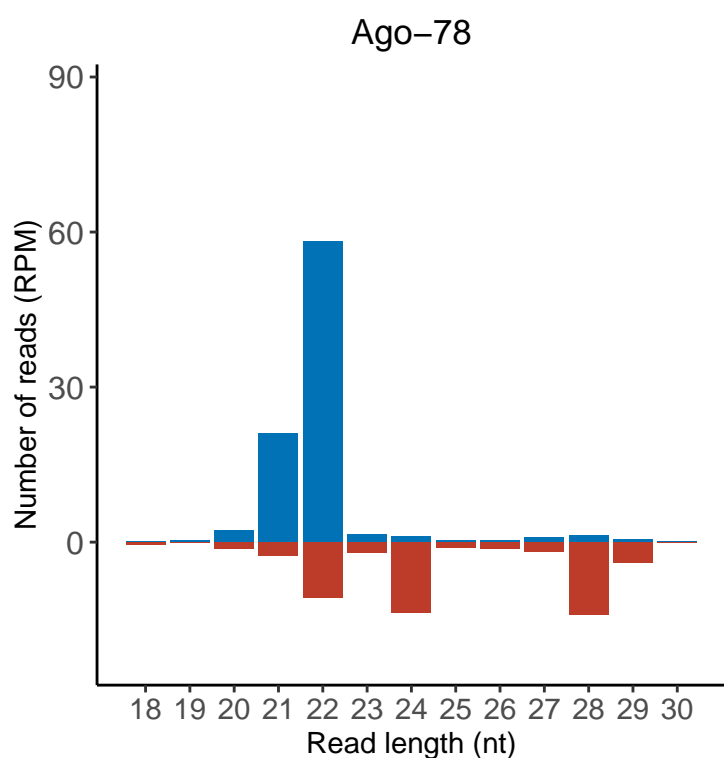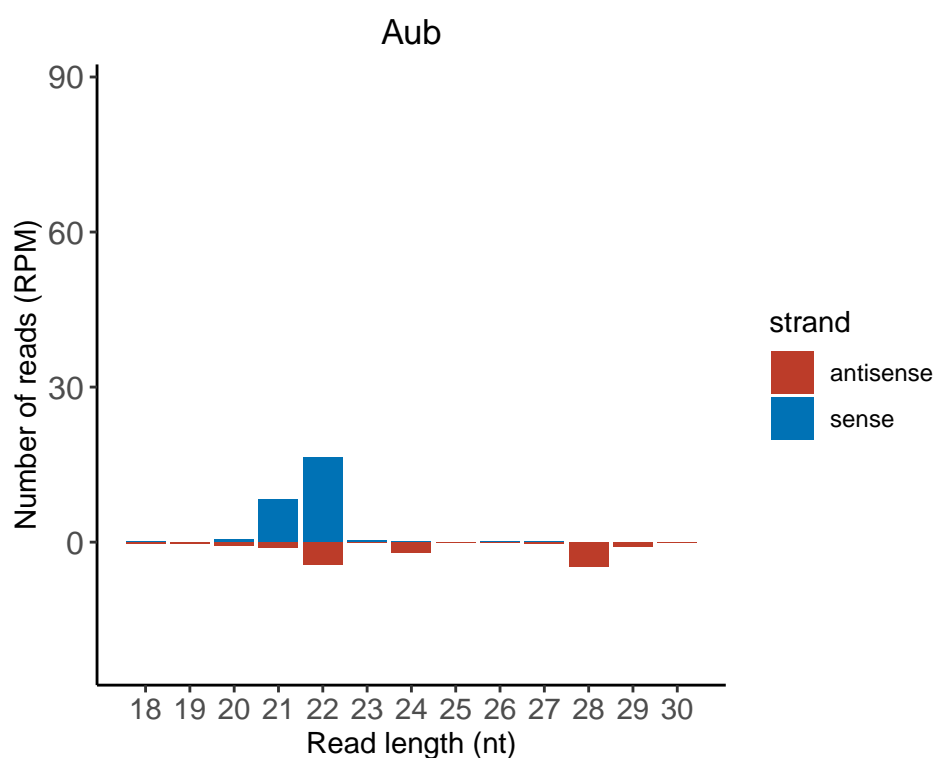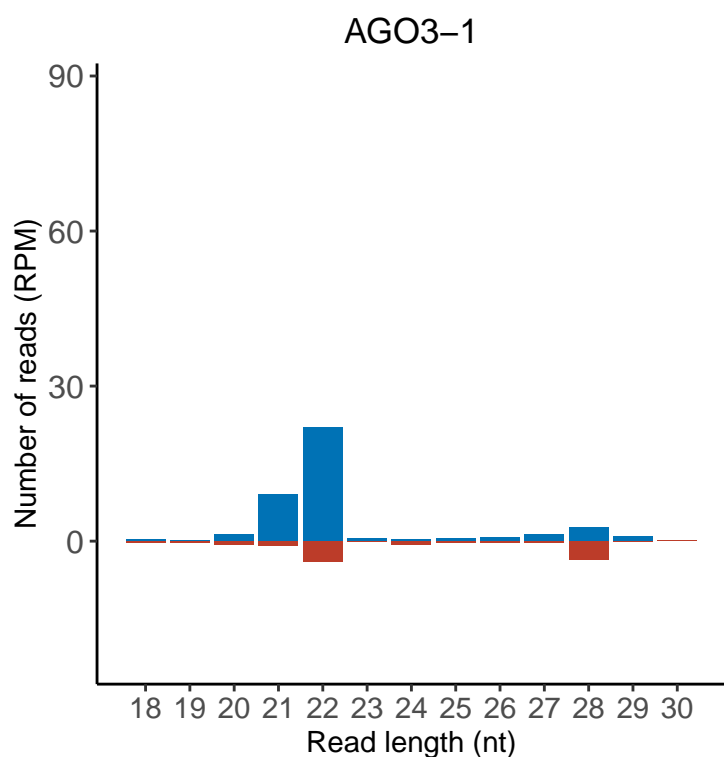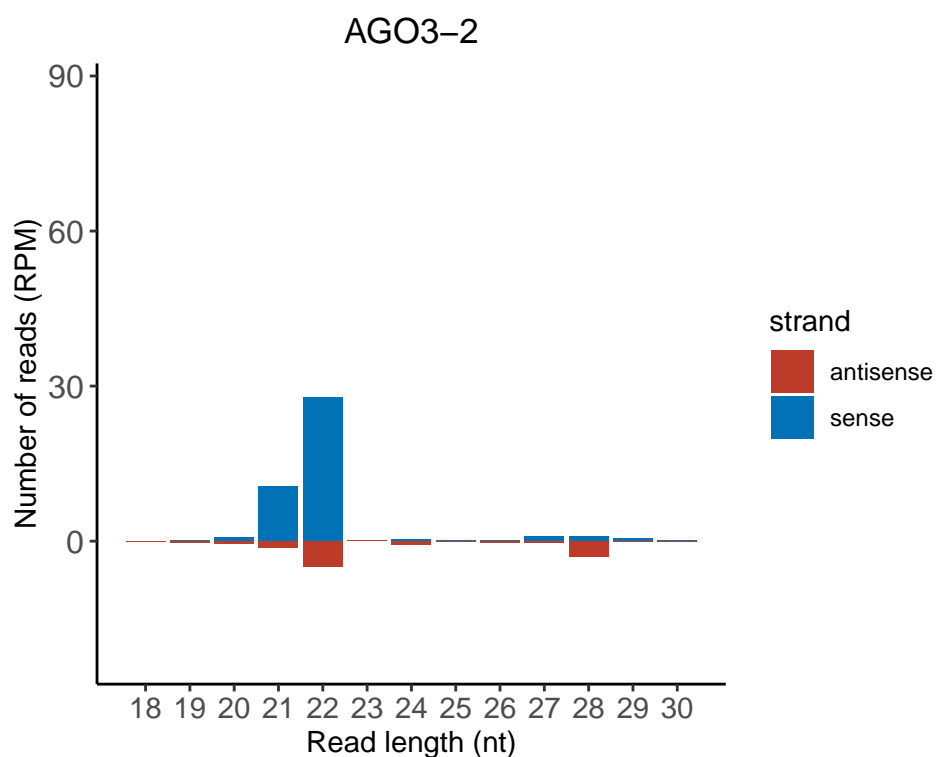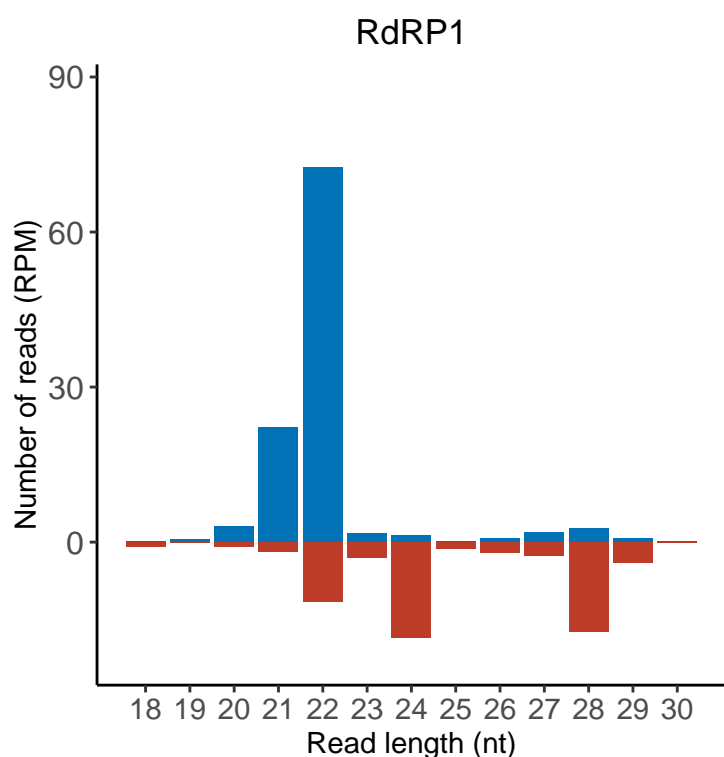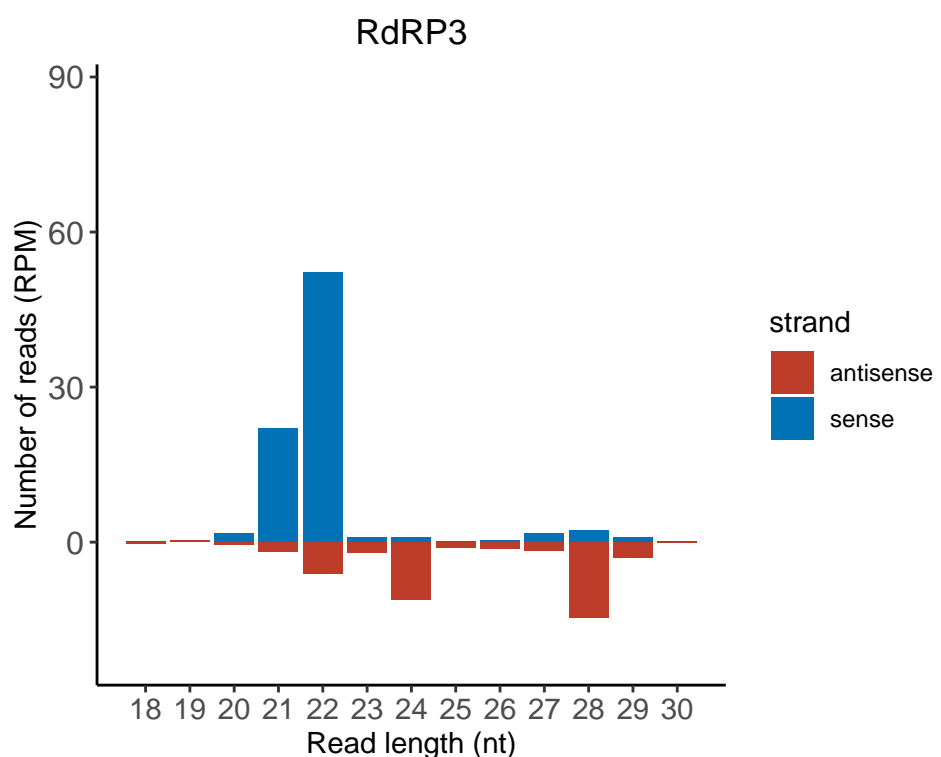

Supplement: S1 Data — On the Normalized and Relative levels pages, normalized read counts (RPM) and relative levels compared to the control (dsGFP) library were used. TEs with more than 50RPM and Coding Genes with more than 3.5RPM on average in KD libraries are included in this website. For viral sequences, reference sequences were generated by assembling sRNA sequences (See the Analysis of sRNAseq data section in Materials and methods.) and size distributions of sRNA reads mapped to each contig are shown. Reads were first grouped by their 5’ nucleotides, and reads of each length were counted. Full tables including TEs and Coding genes with fewer reads are in the “FullTable” folder. (ZIP) [file pone.0281195.s017.zip › SupplementaryData/Links/PDFs/Motif:rnd-1_family-401TE_lengthdistribution.pdf]

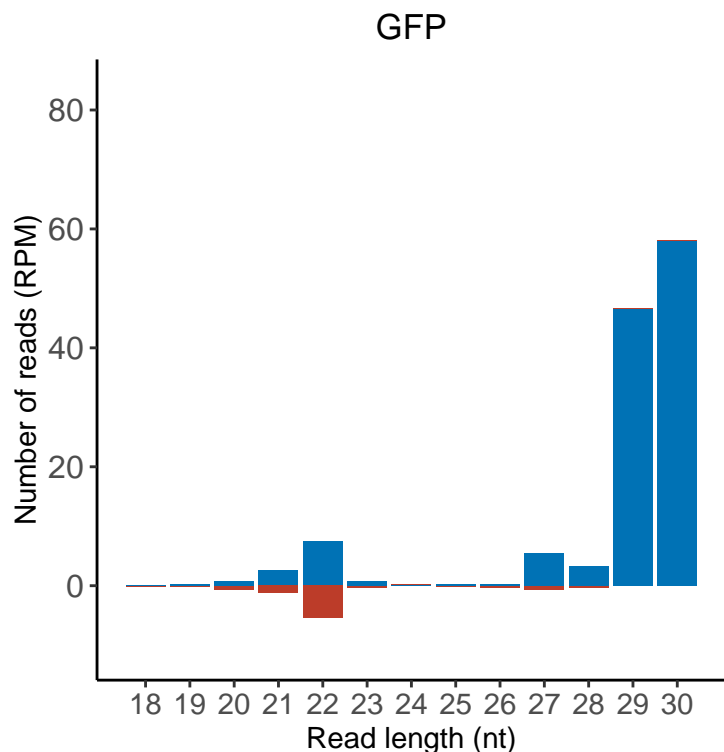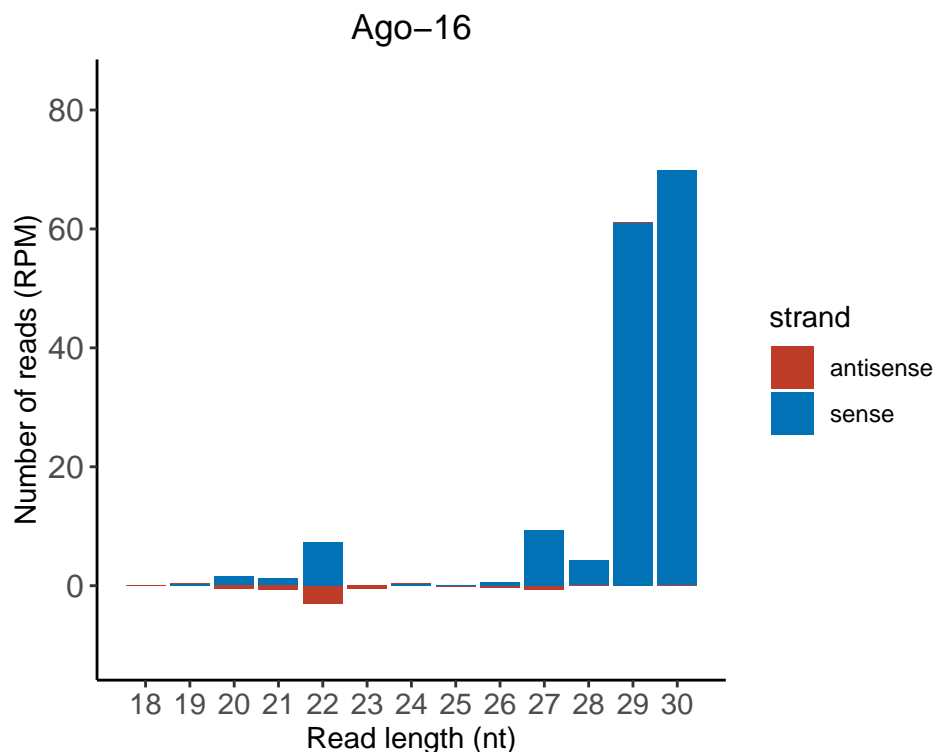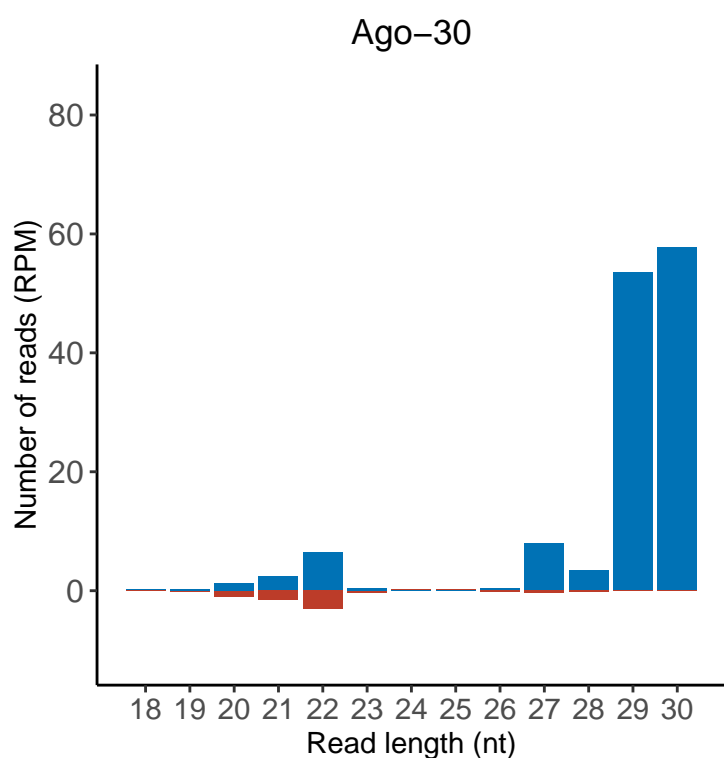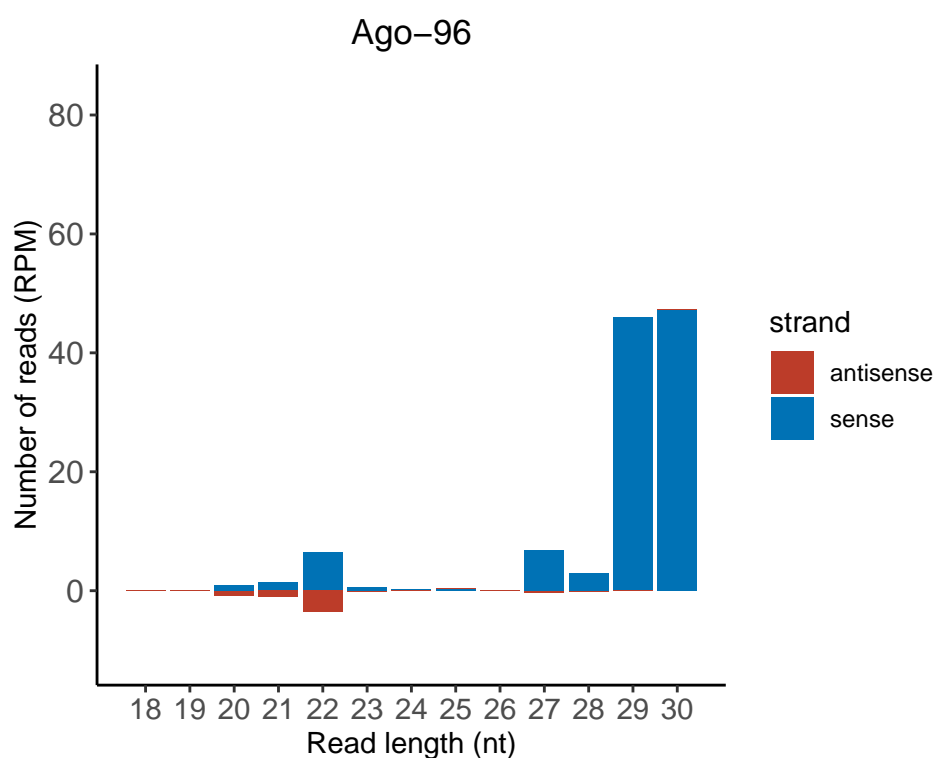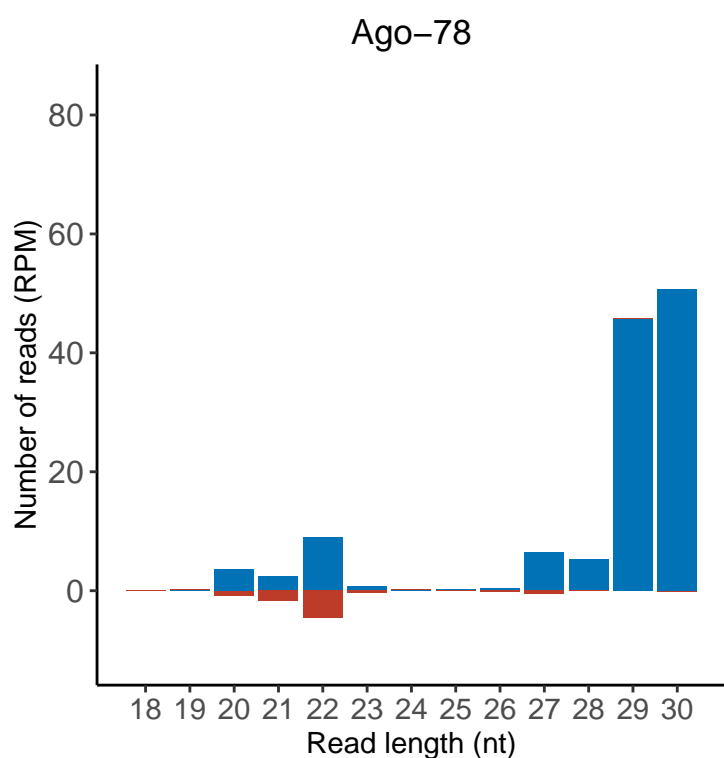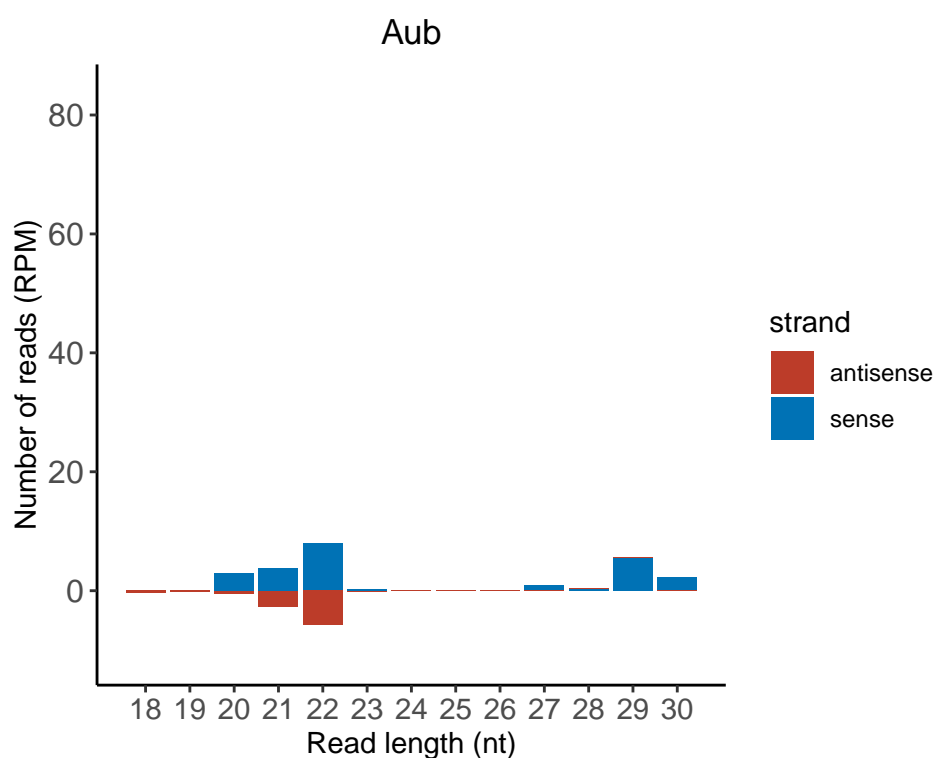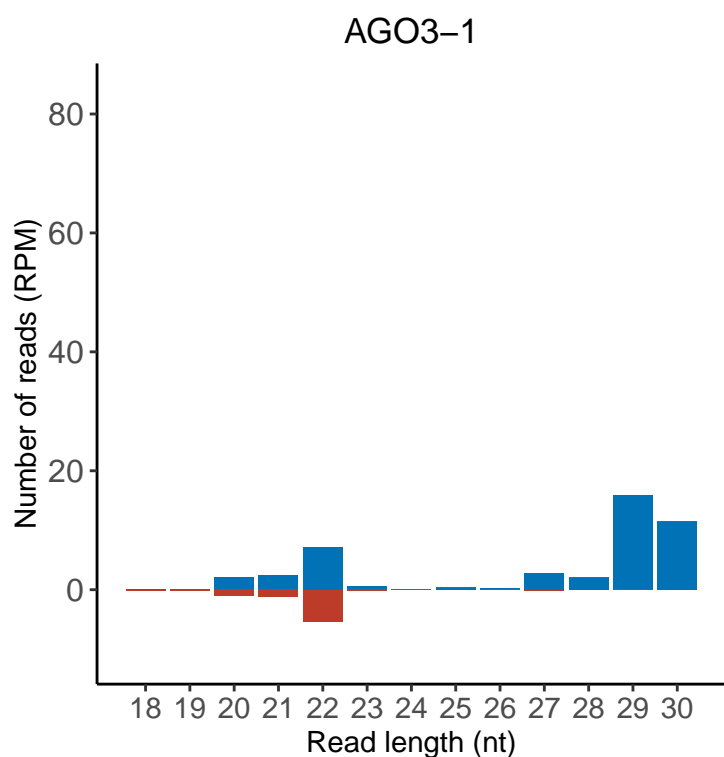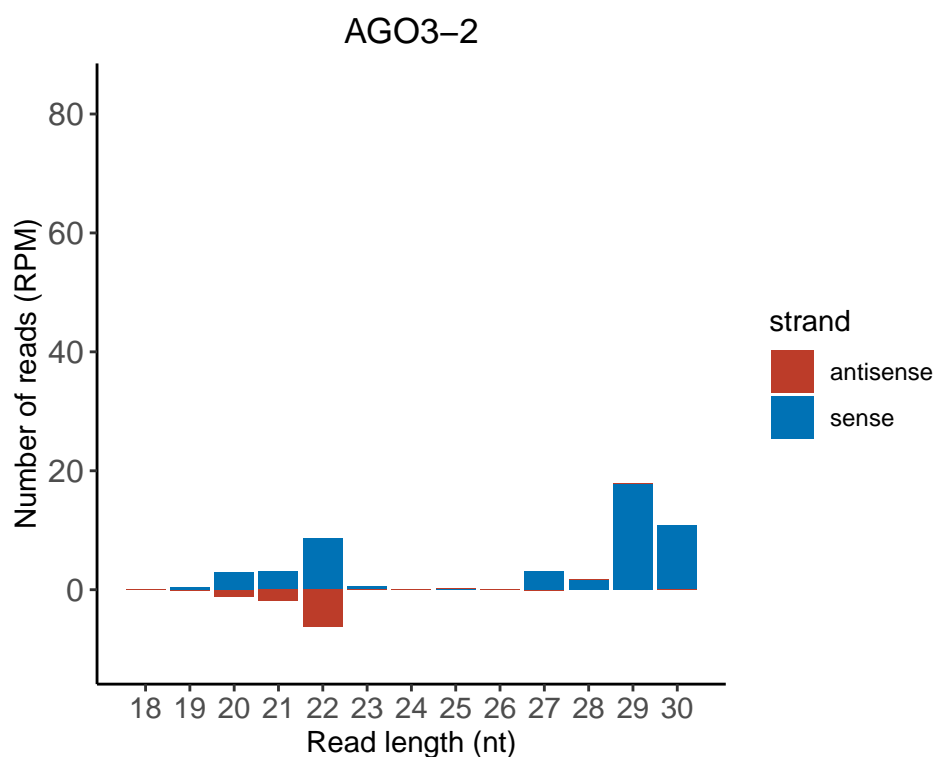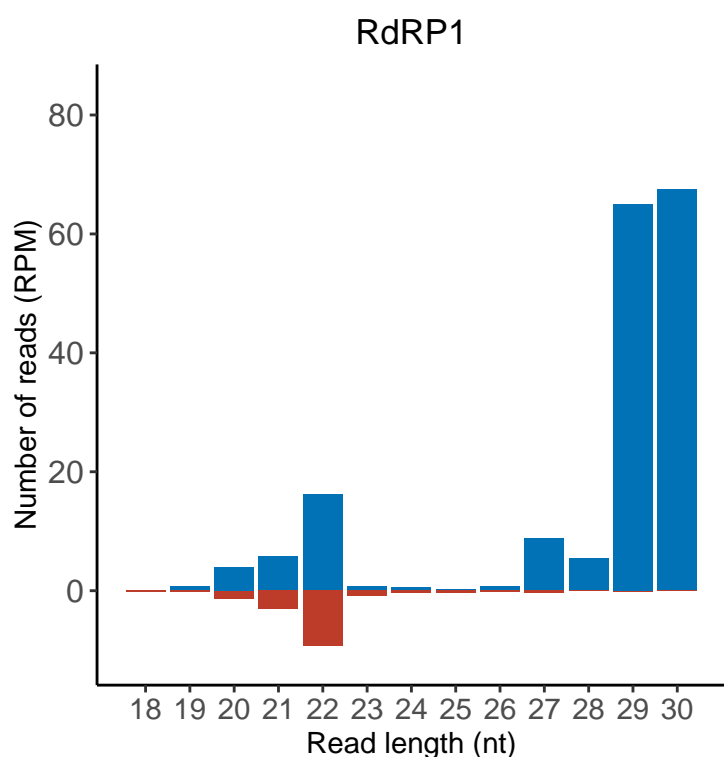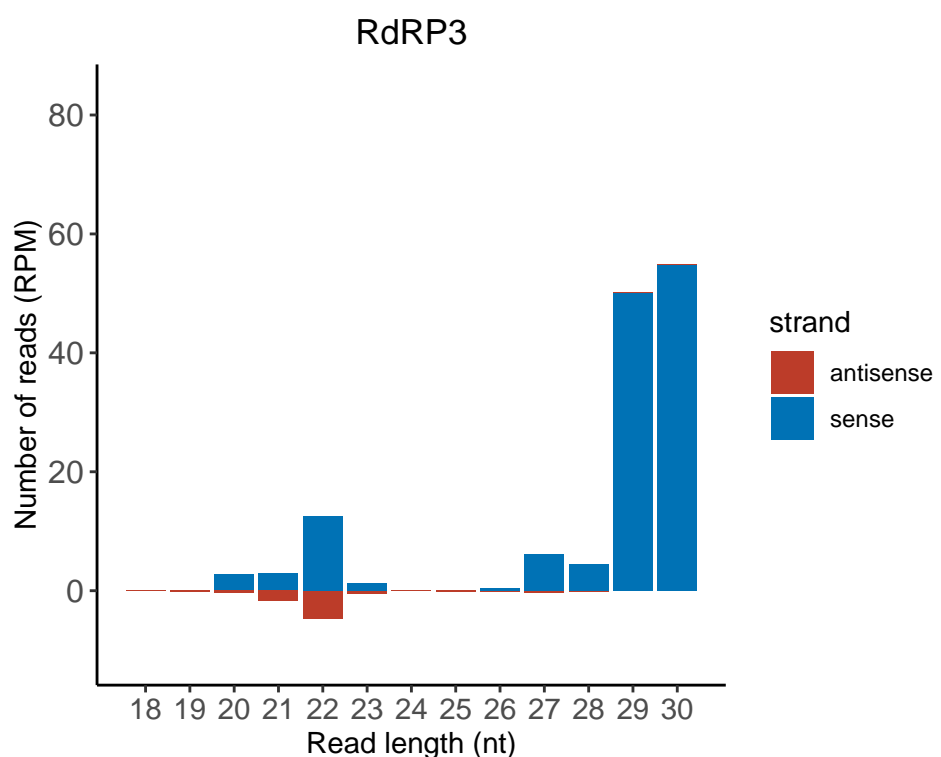

Supplement: S1 Data — On the Normalized and Relative levels pages, normalized read counts (RPM) and relative levels compared to the control (dsGFP) library were used. TEs with more than 50RPM and Coding Genes with more than 3.5RPM on average in KD libraries are included in this website. For viral sequences, reference sequences were generated by assembling sRNA sequences (See the Analysis of sRNAseq data section in Materials and methods.) and size distributions of sRNA reads mapped to each contig are shown. Reads were first grouped by their 5’ nucleotides, and reads of each length were counted. Full tables including TEs and Coding genes with fewer reads are in the “FullTable” folder. (ZIP) [file pone.0281195.s017.zip › SupplementaryData/Links/PDFs/Motif:rnd-6_family-12996TE_lengthdistribution.pdf]

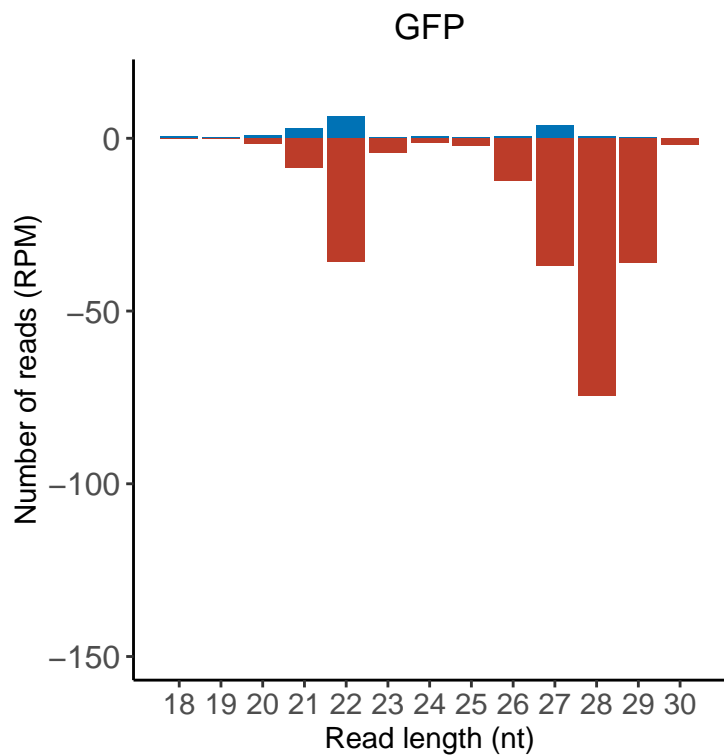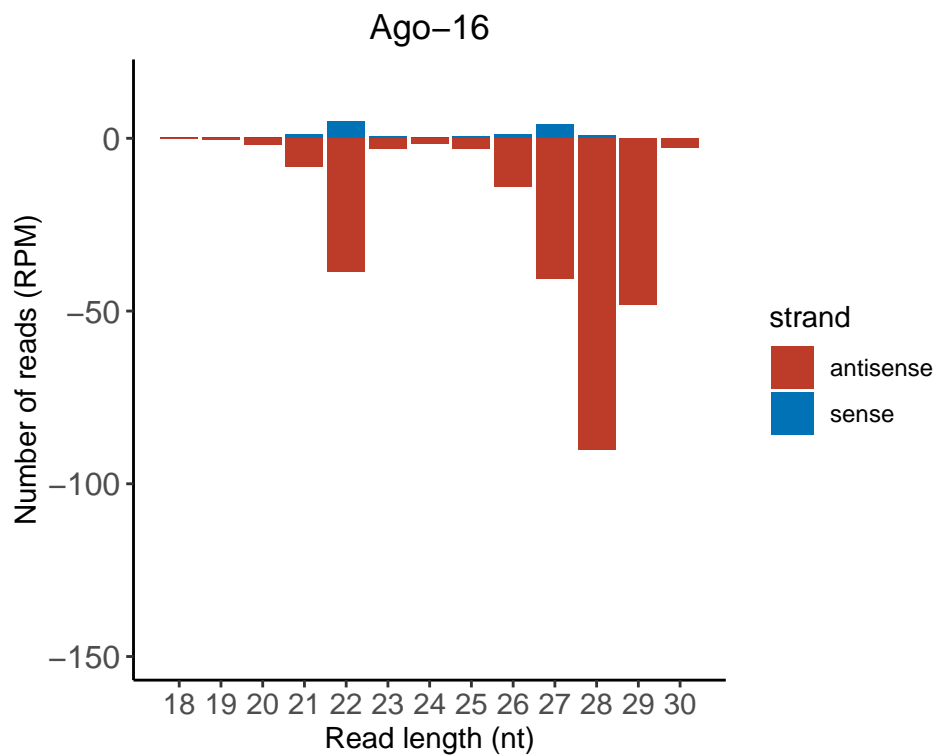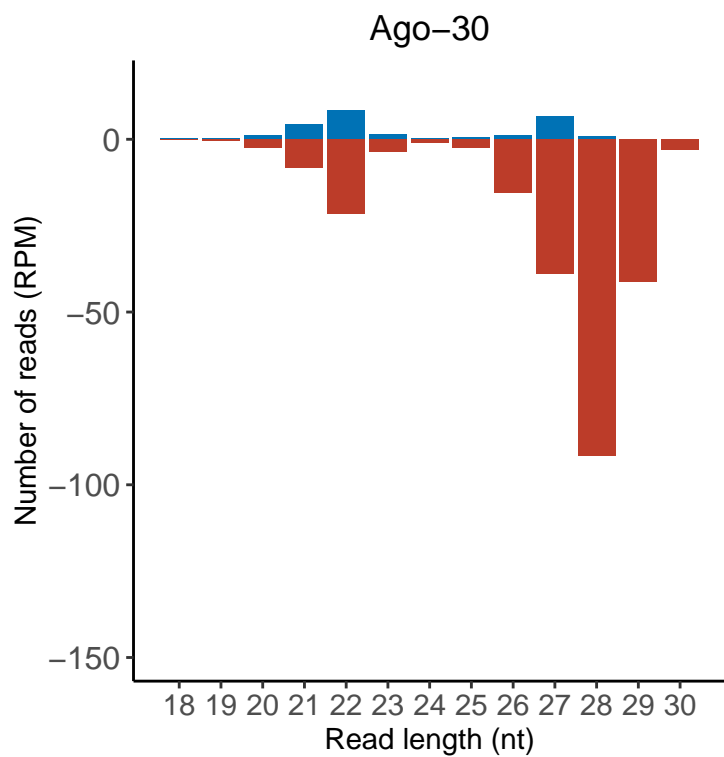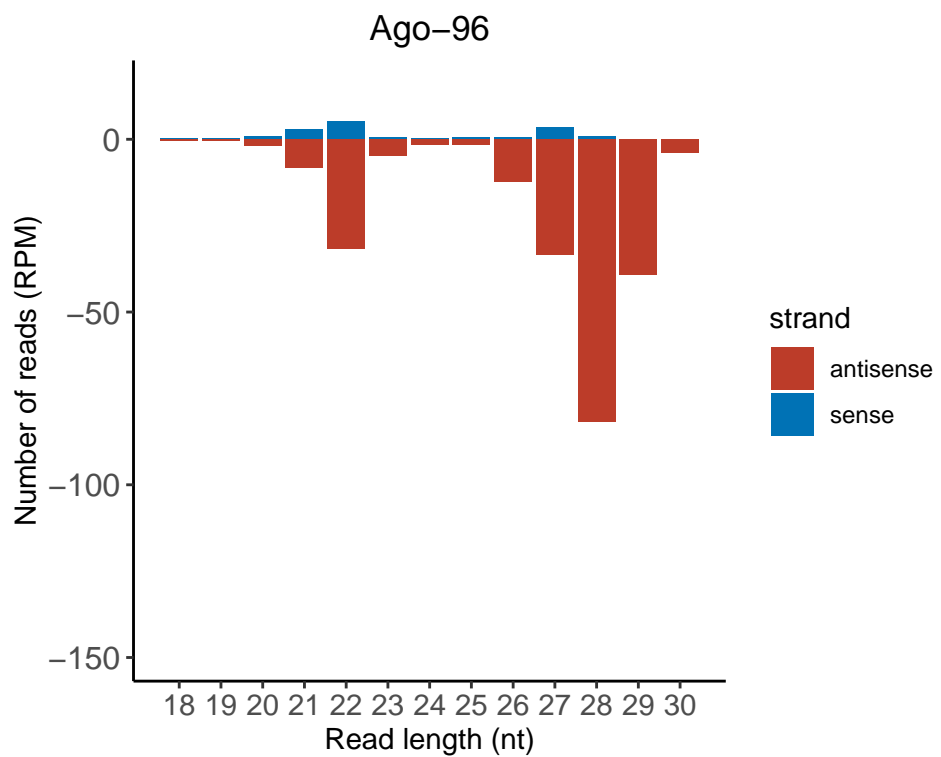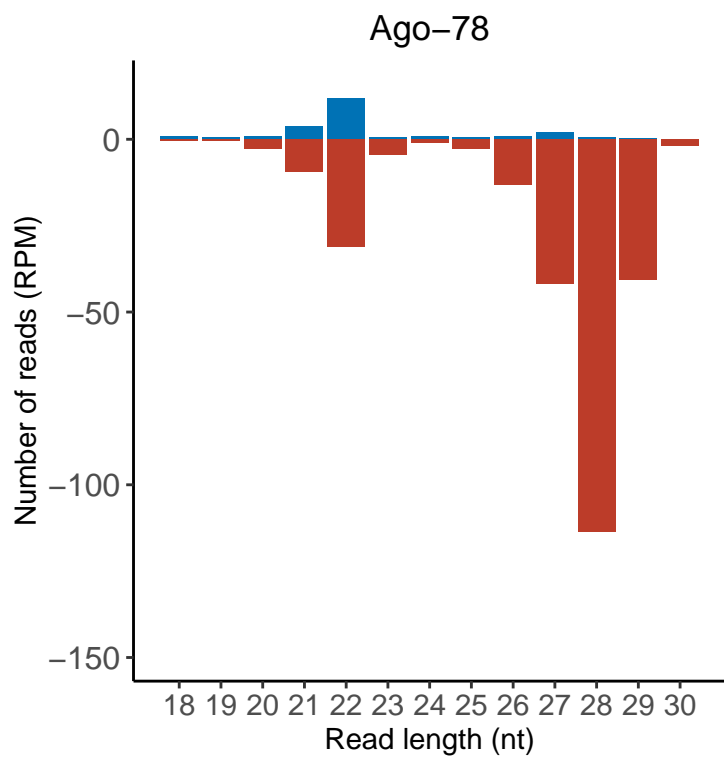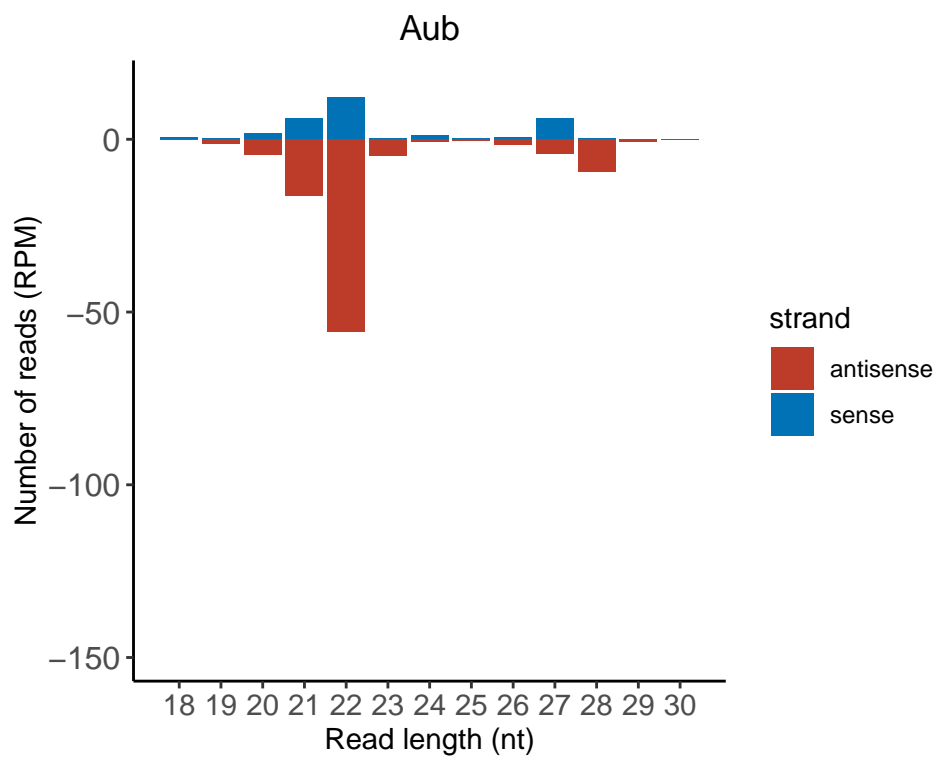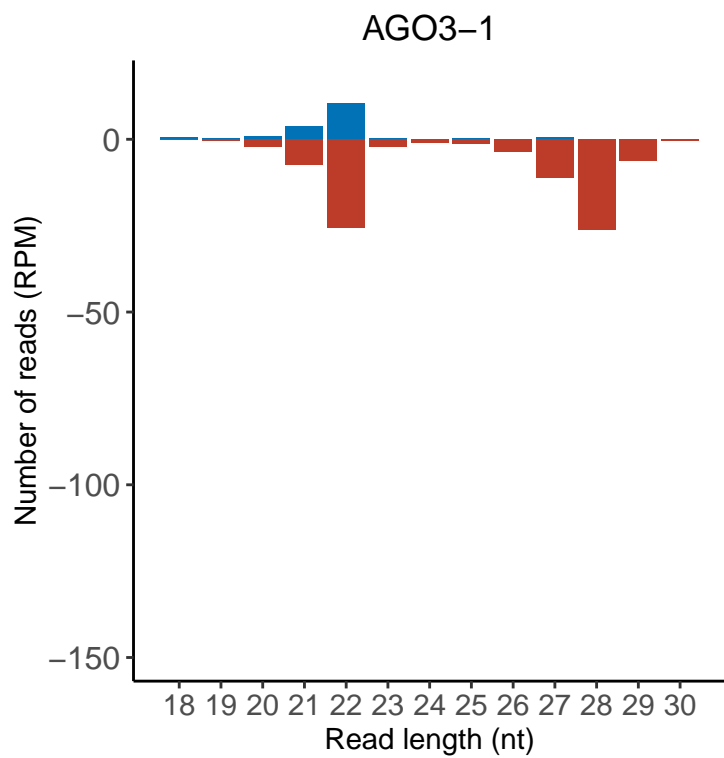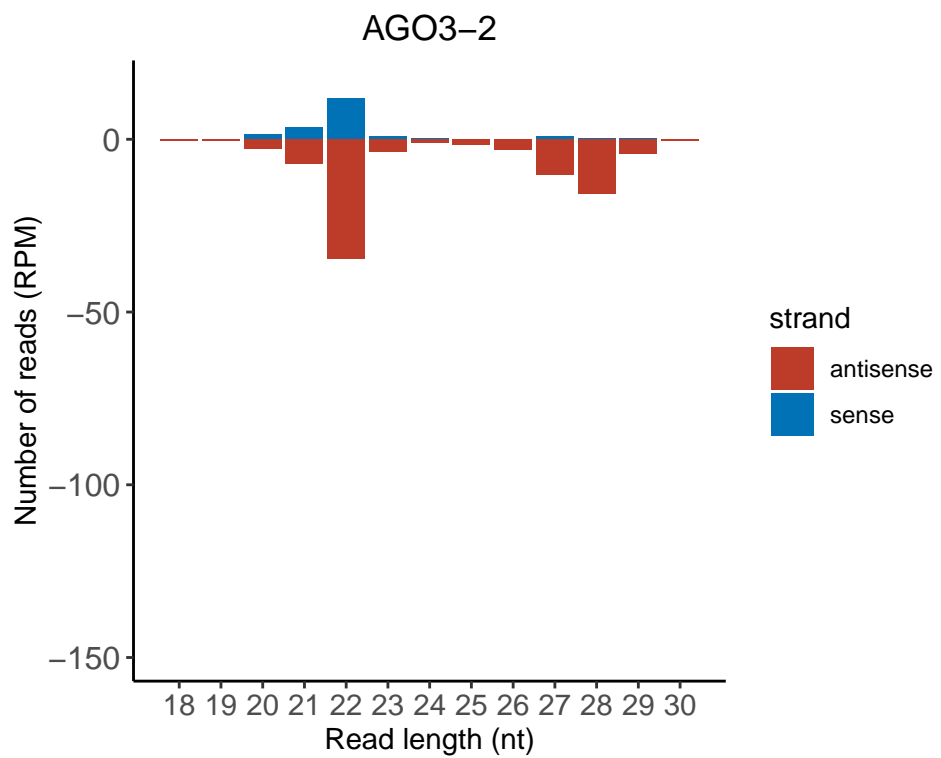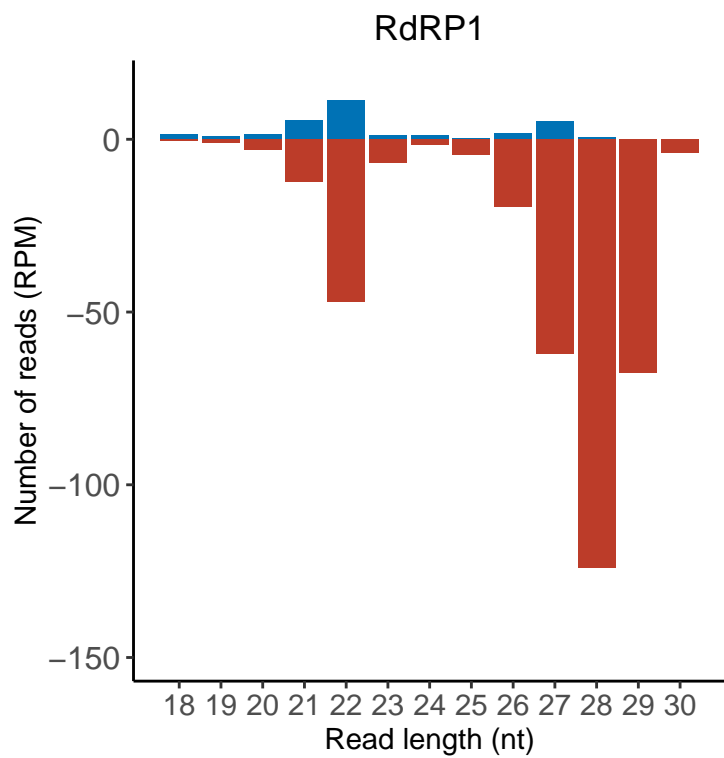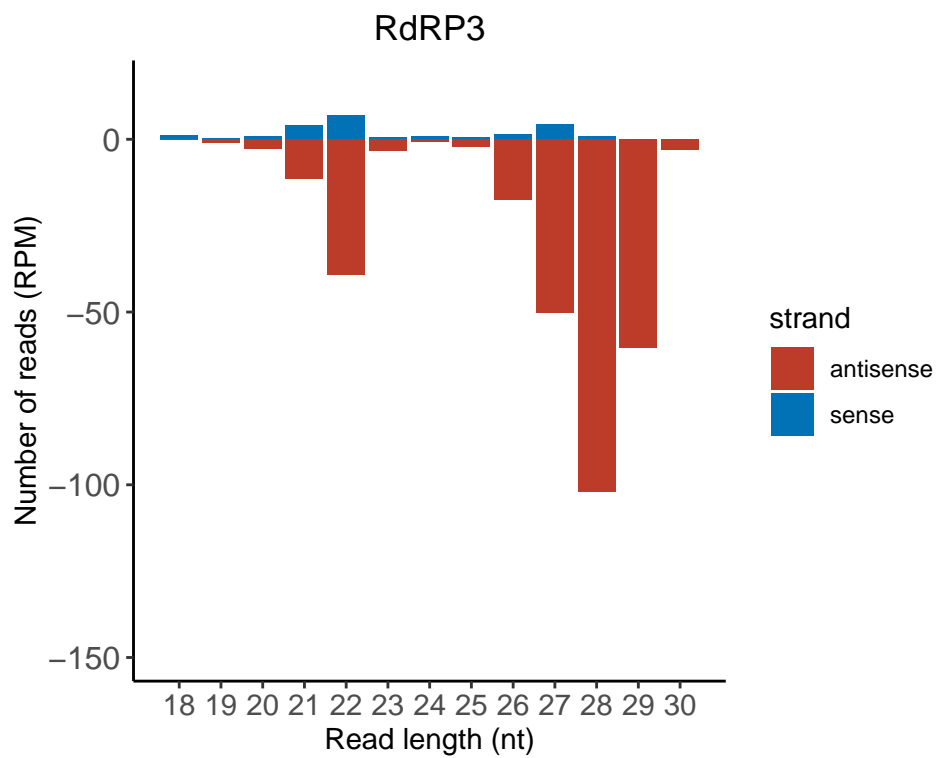

Supplement: S1 Data — On the Normalized and Relative levels pages, normalized read counts (RPM) and relative levels compared to the control (dsGFP) library were used. TEs with more than 50RPM and Coding Genes with more than 3.5RPM on average in KD libraries are included in this website. For viral sequences, reference sequences were generated by assembling sRNA sequences (See the Analysis of sRNAseq data section in Materials and methods.) and size distributions of sRNA reads mapped to each contig are shown. Reads were first grouped by their 5’ nucleotides, and reads of each length were counted. Full tables including TEs and Coding genes with fewer reads are in the “FullTable” folder. (ZIP) [file pone.0281195.s017.zip › SupplementaryData/Links/PDFs/Motif:rnd-1_family-243TE_lengthdistribution.pdf]

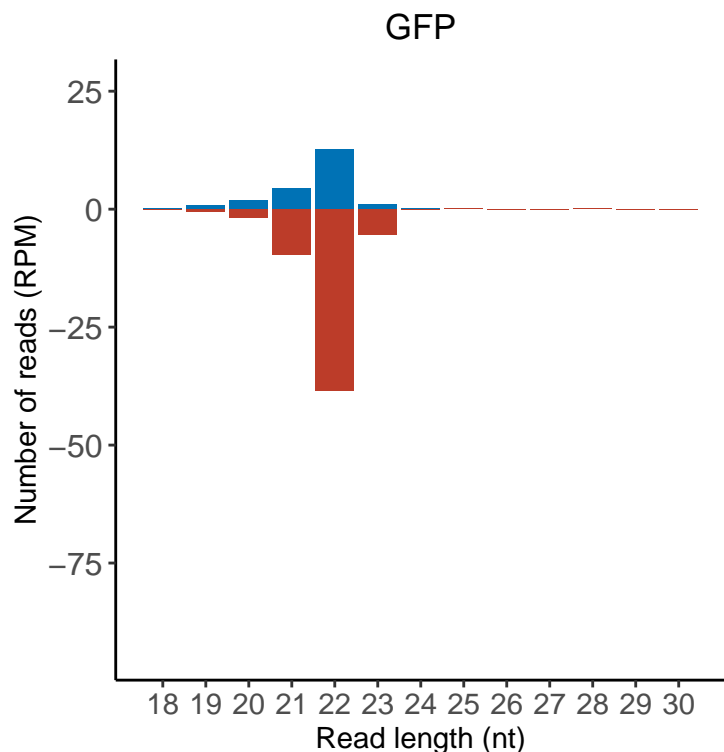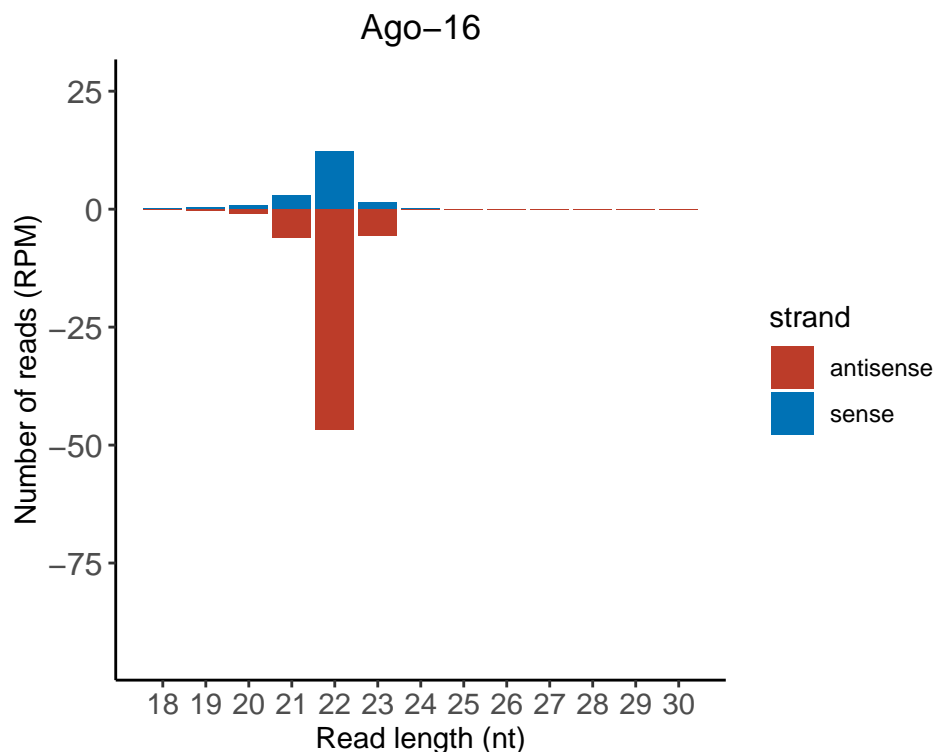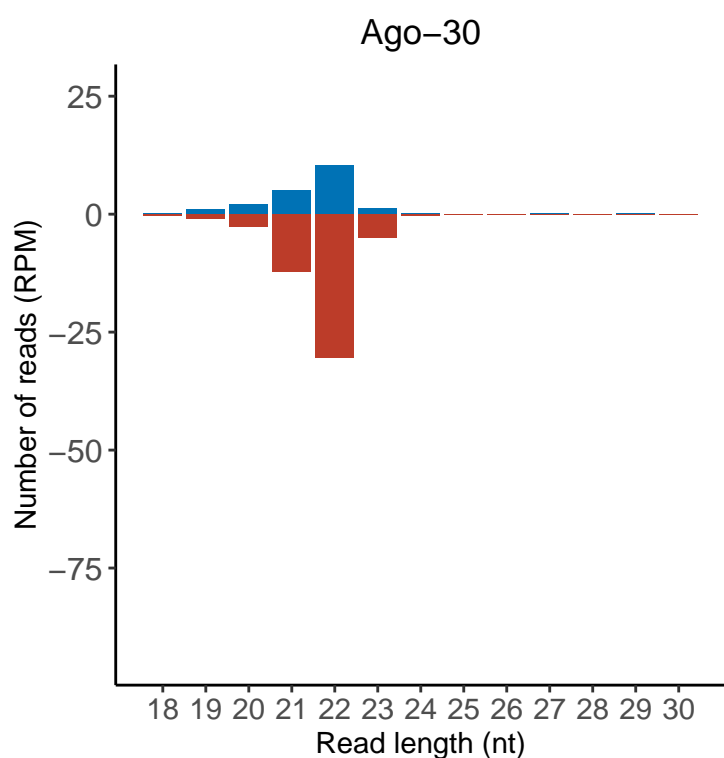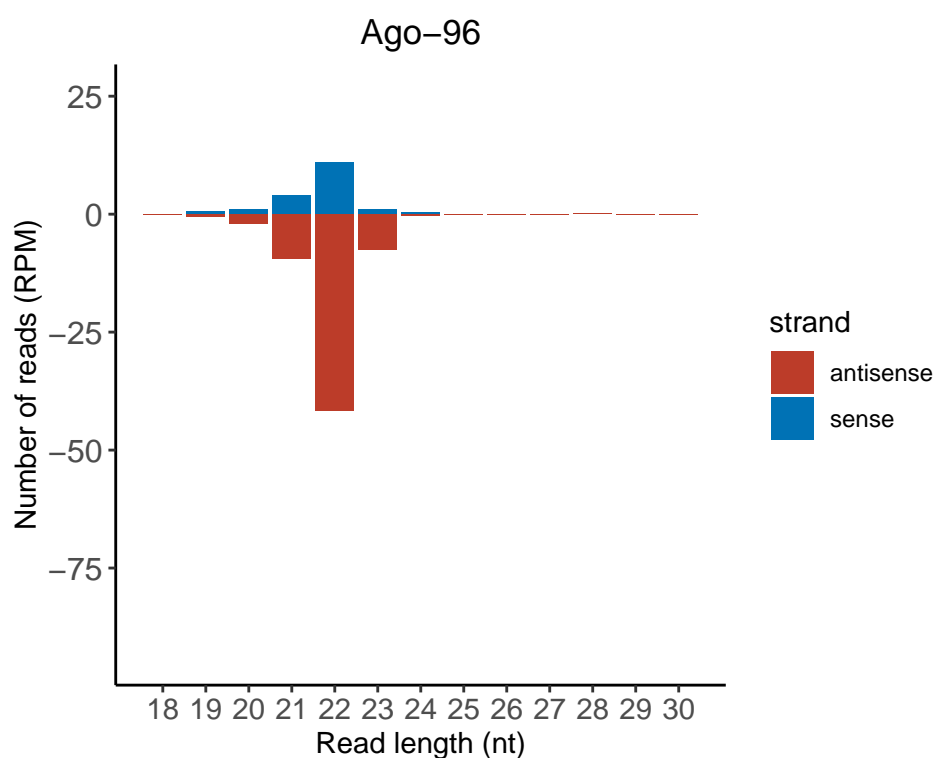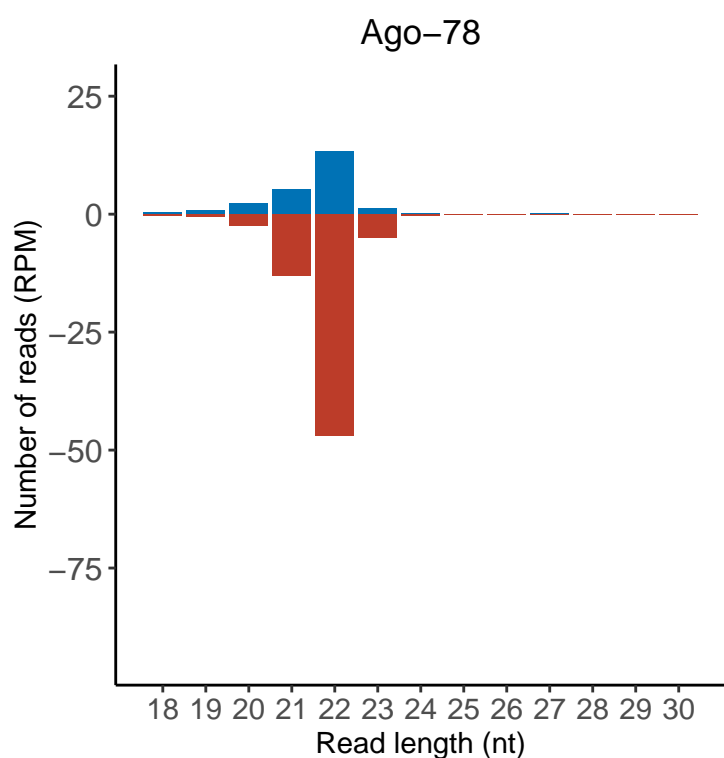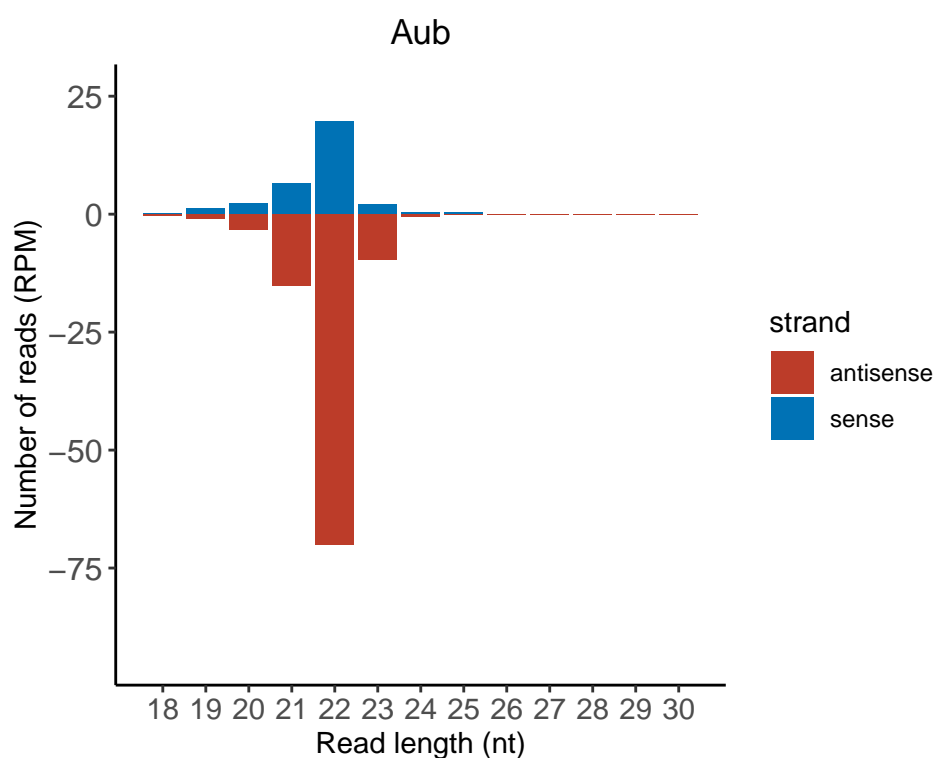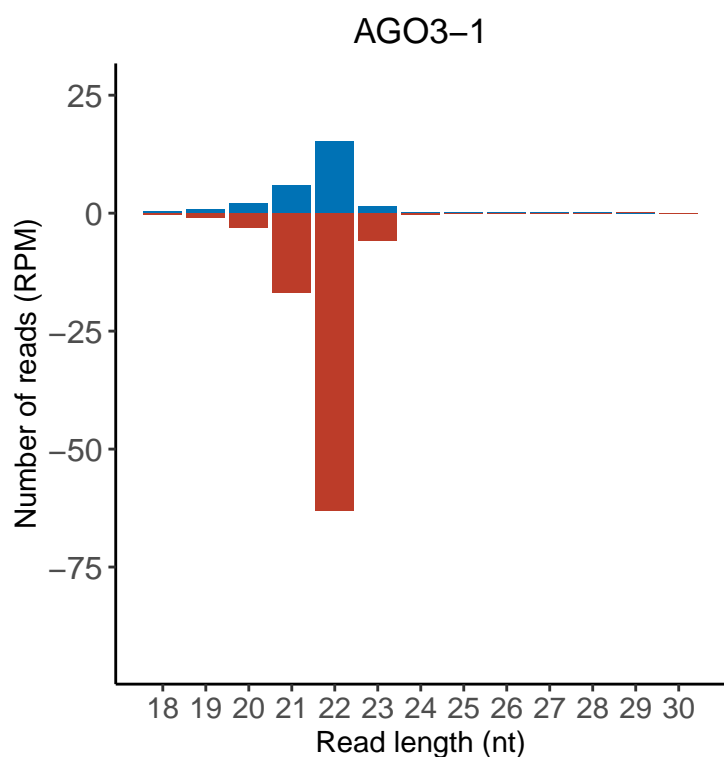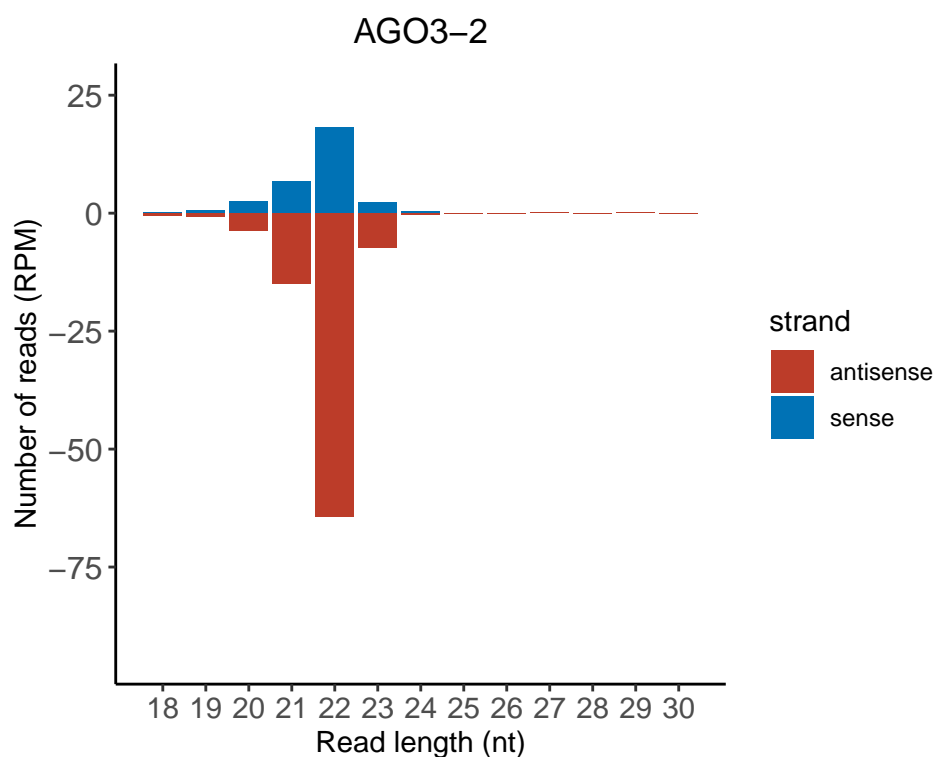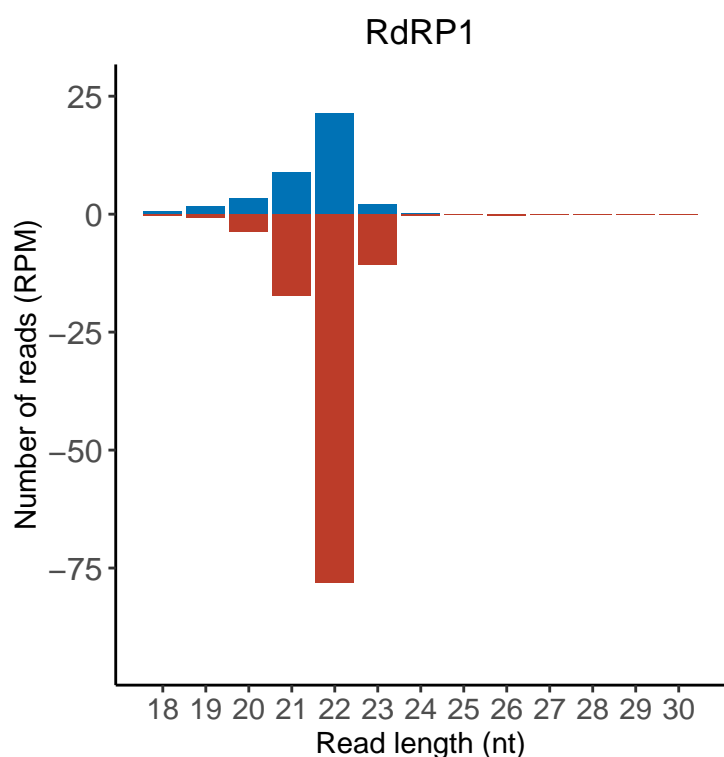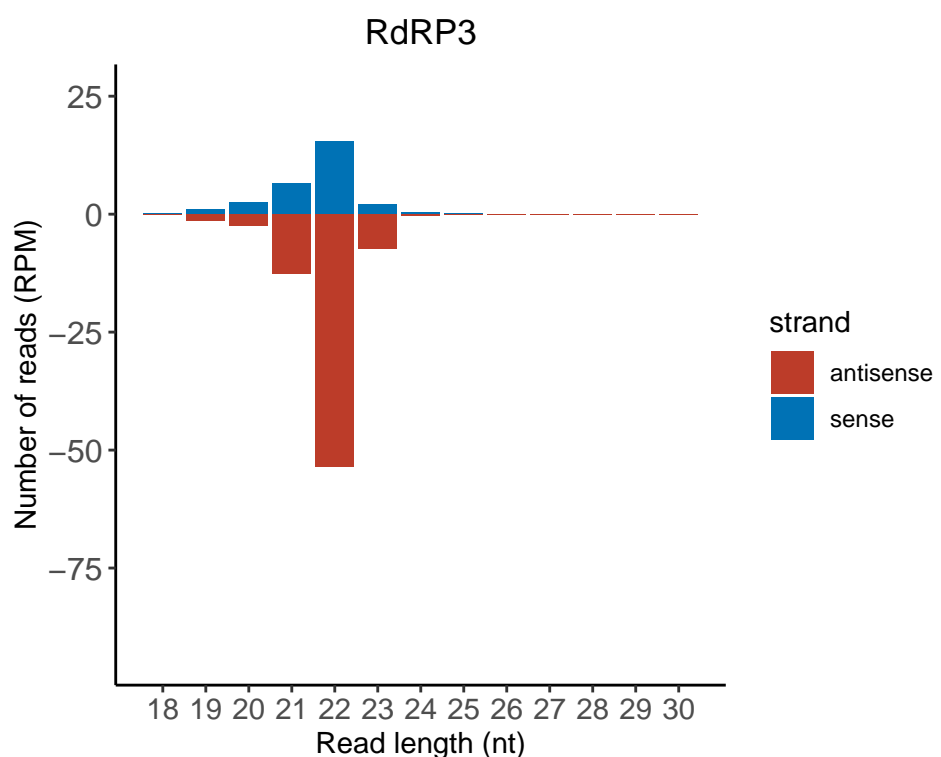

Supplement: S1 Data — On the Normalized and Relative levels pages, normalized read counts (RPM) and relative levels compared to the control (dsGFP) library were used. TEs with more than 50RPM and Coding Genes with more than 3.5RPM on average in KD libraries are included in this website. For viral sequences, reference sequences were generated by assembling sRNA sequences (See the Analysis of sRNAseq data section in Materials and methods.) and size distributions of sRNA reads mapped to each contig are shown. Reads were first grouped by their 5’ nucleotides, and reads of each length were counted. Full tables including TEs and Coding genes with fewer reads are in the “FullTable” folder. (ZIP) [file pone.0281195.s017.zip › SupplementaryData/Links/PDFs/Motif:rnd-6_family-2824TE_lengthdistribution.pdf]

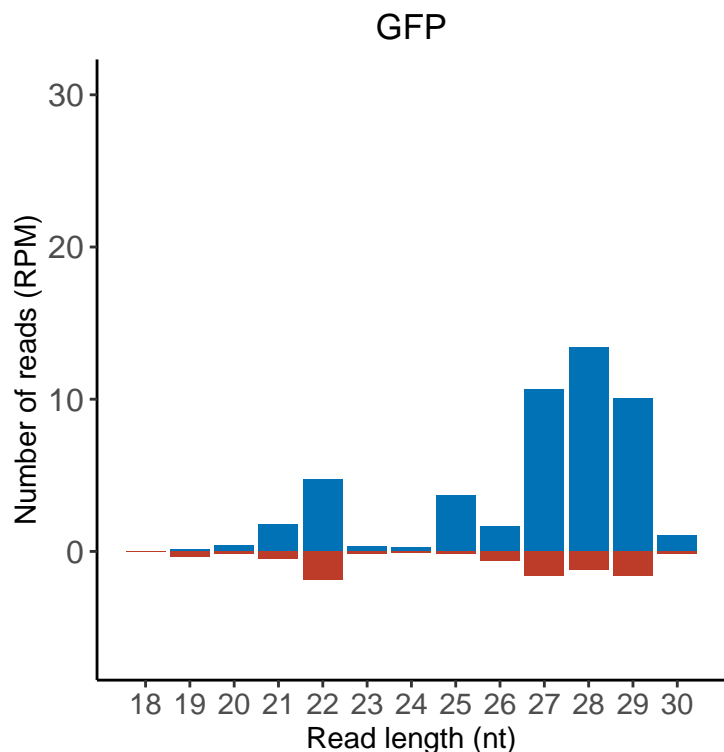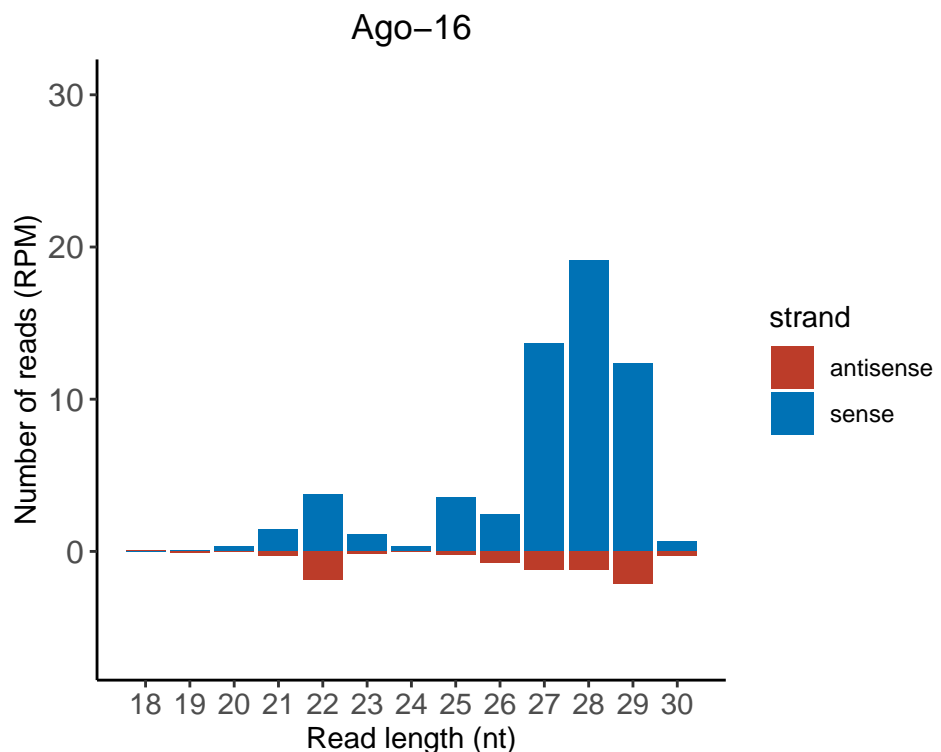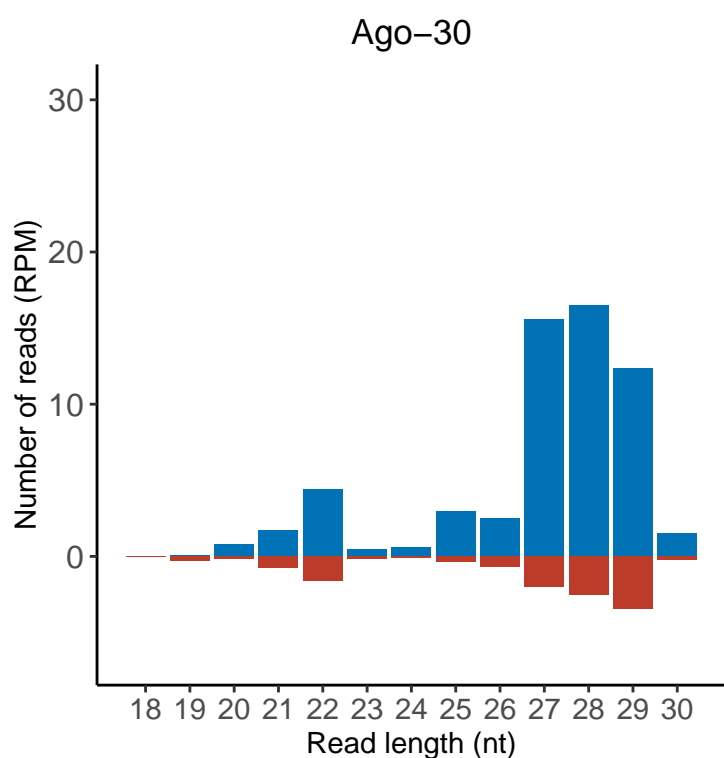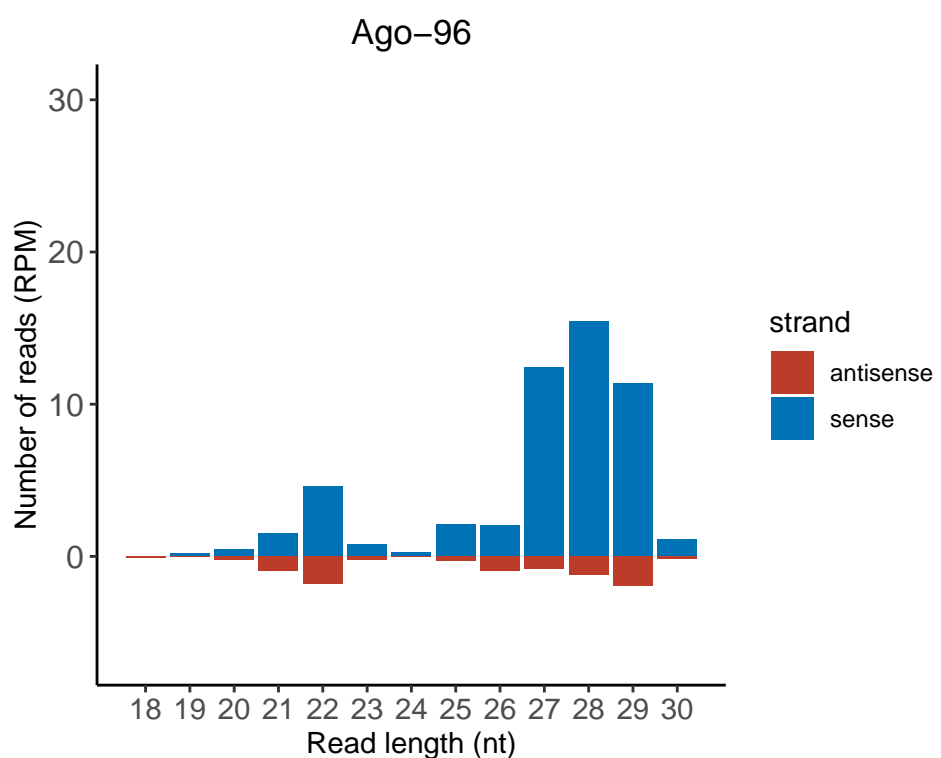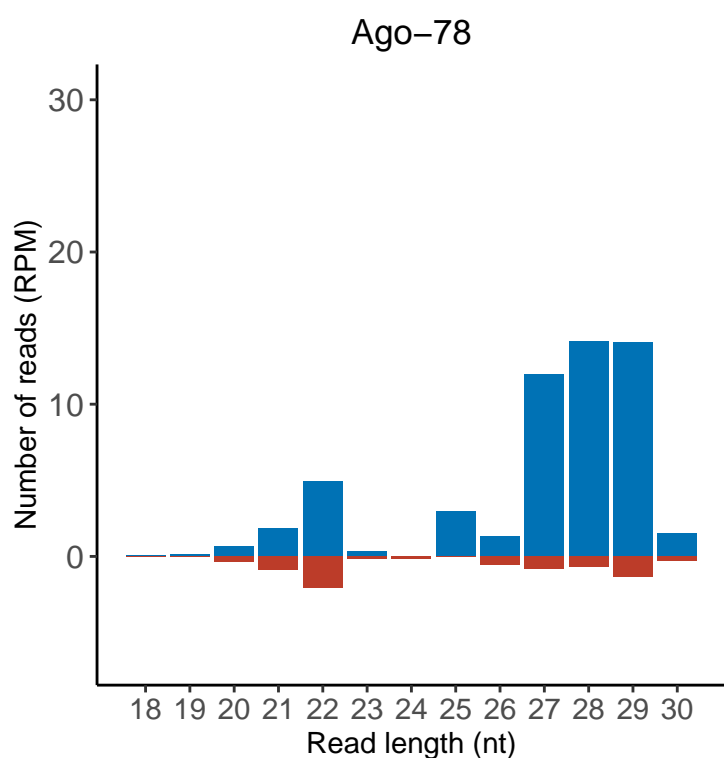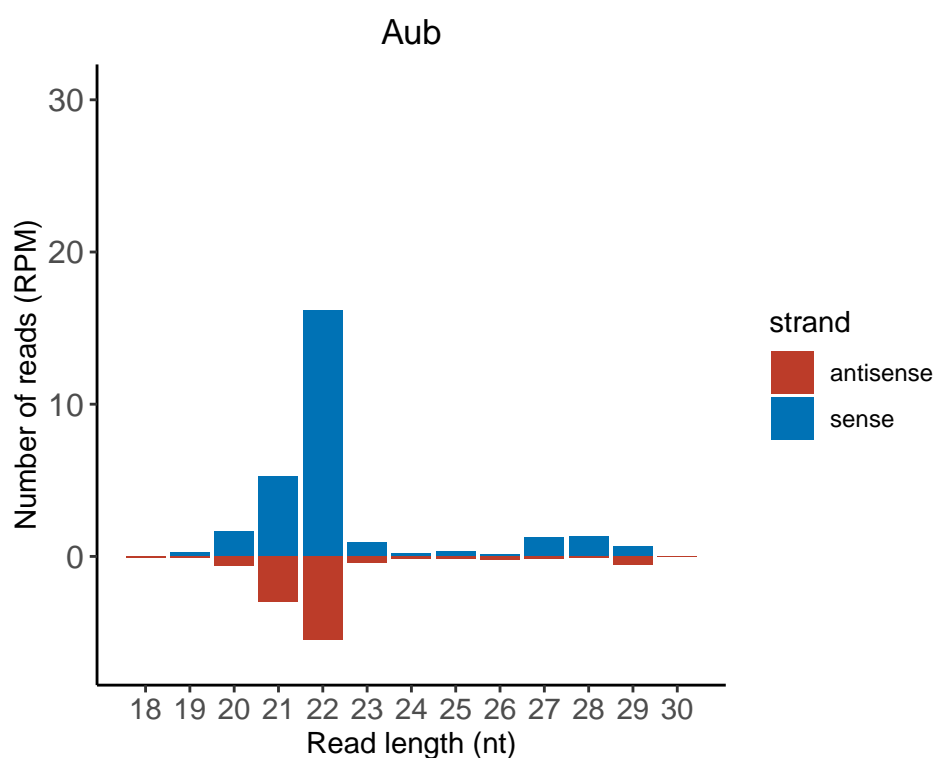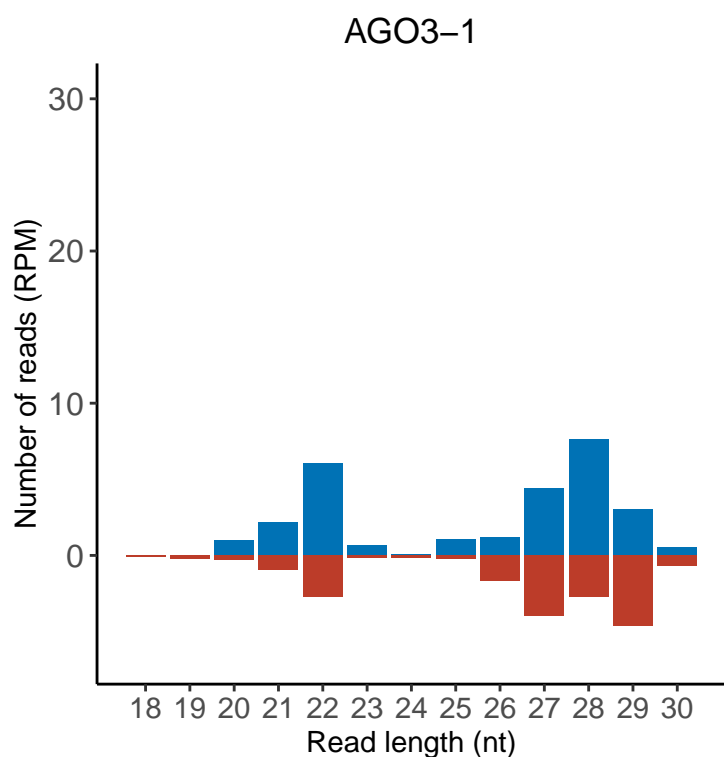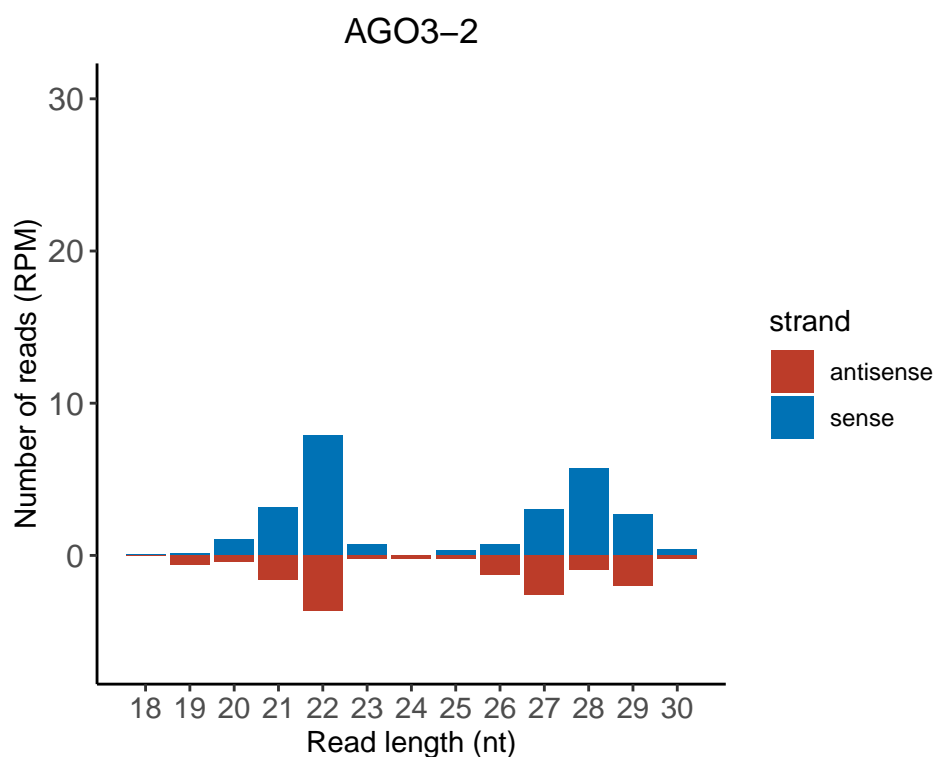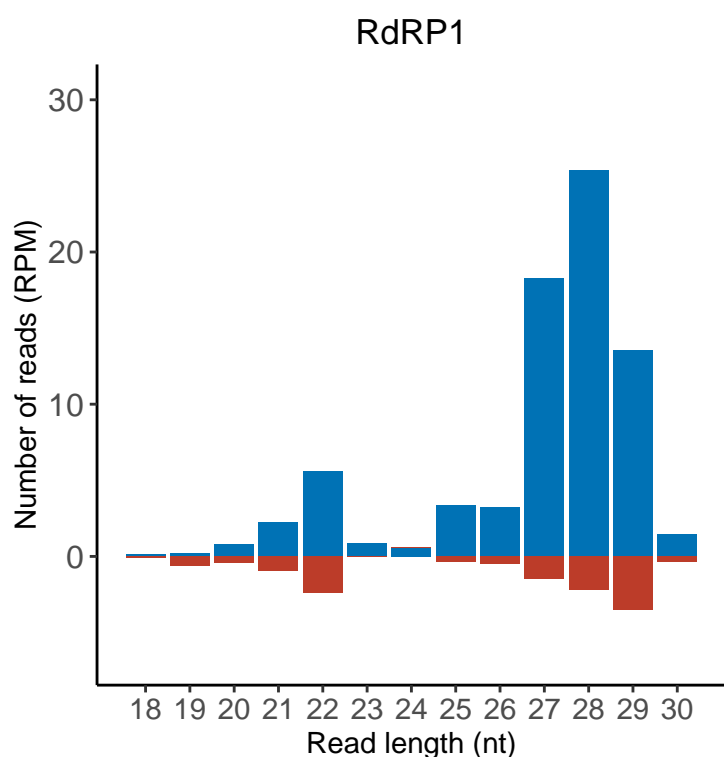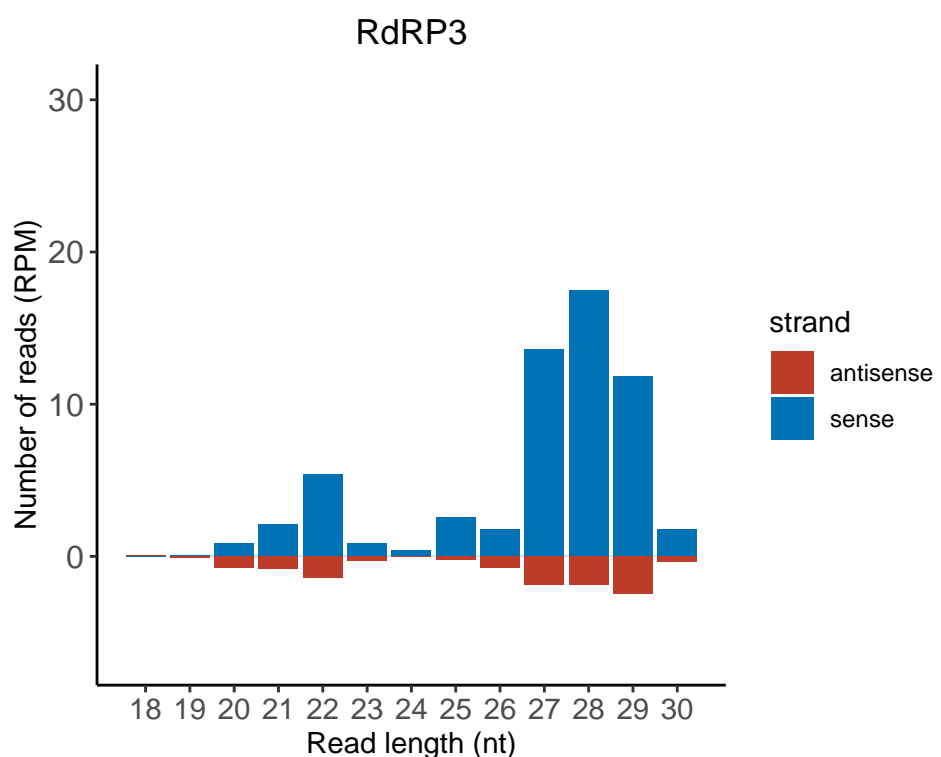

Supplement: S1 Data — On the Normalized and Relative levels pages, normalized read counts (RPM) and relative levels compared to the control (dsGFP) library were used. TEs with more than 50RPM and Coding Genes with more than 3.5RPM on average in KD libraries are included in this website. For viral sequences, reference sequences were generated by assembling sRNA sequences (See the Analysis of sRNAseq data section in Materials and methods.) and size distributions of sRNA reads mapped to each contig are shown. Reads were first grouped by their 5’ nucleotides, and reads of each length were counted. Full tables including TEs and Coding genes with fewer reads are in the “FullTable” folder. (ZIP) [file pone.0281195.s017.zip › SupplementaryData/Links/PDFs/Motif:rnd-1_family-1751TE_lengthdistribution.pdf]

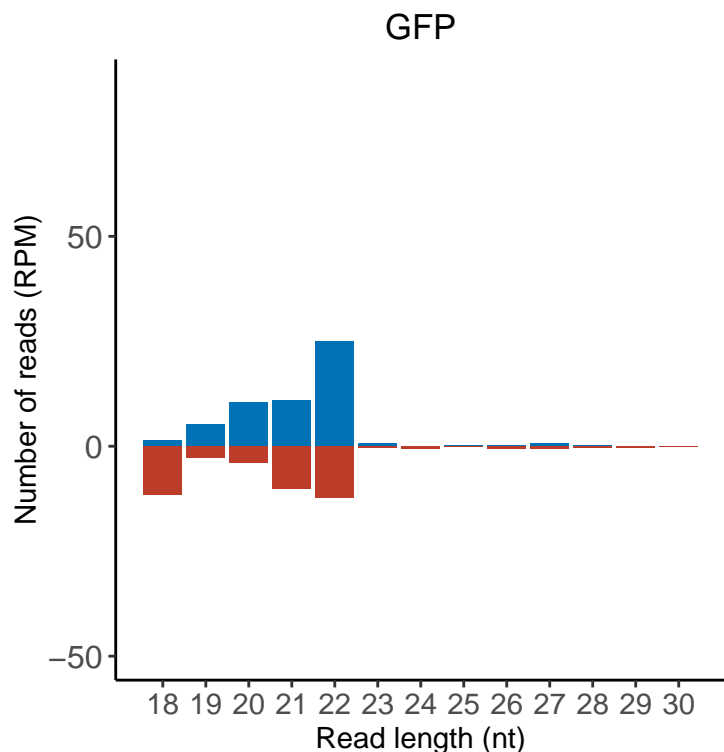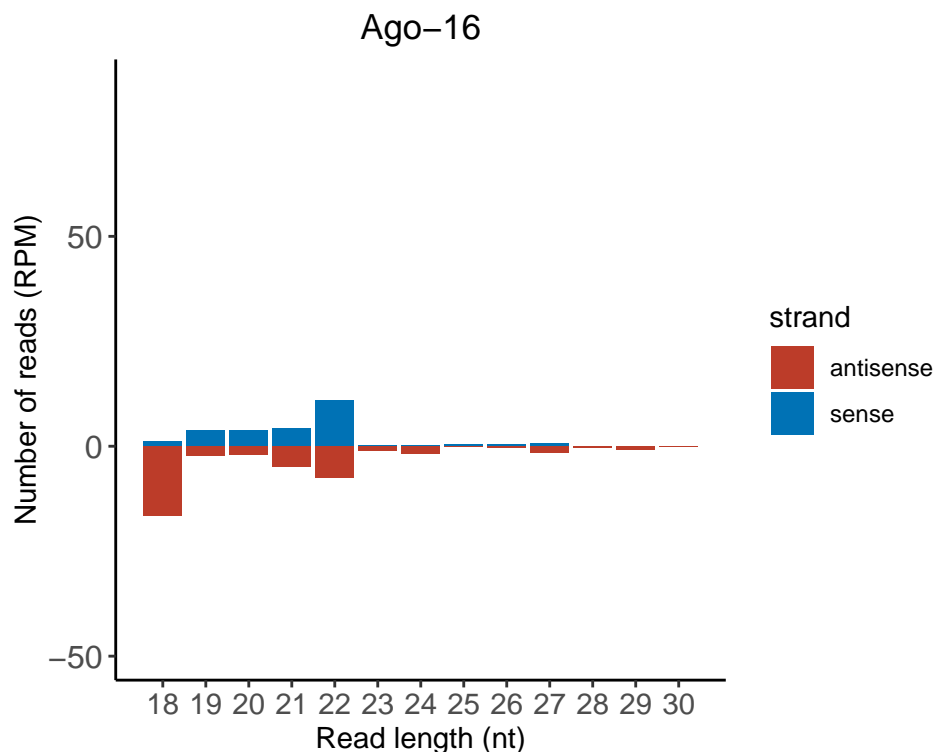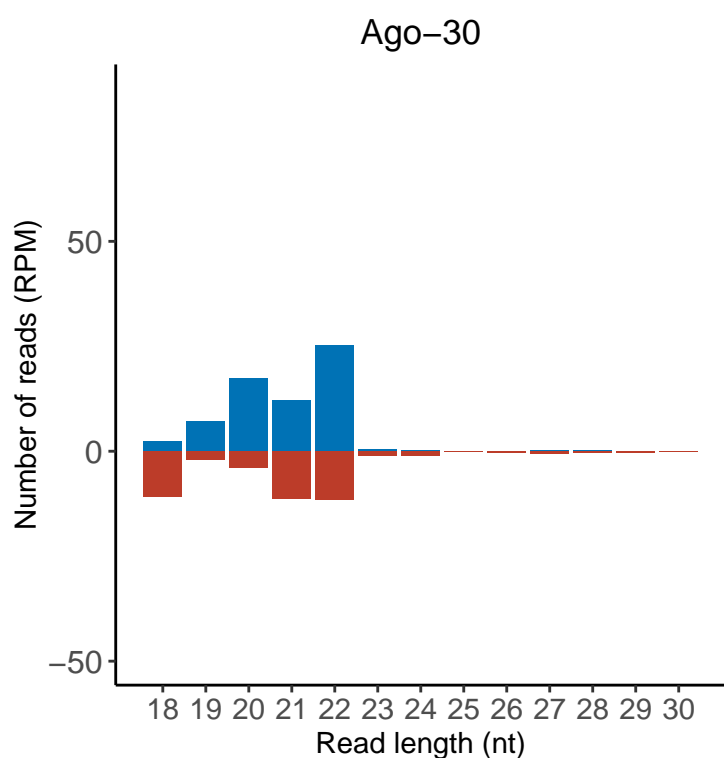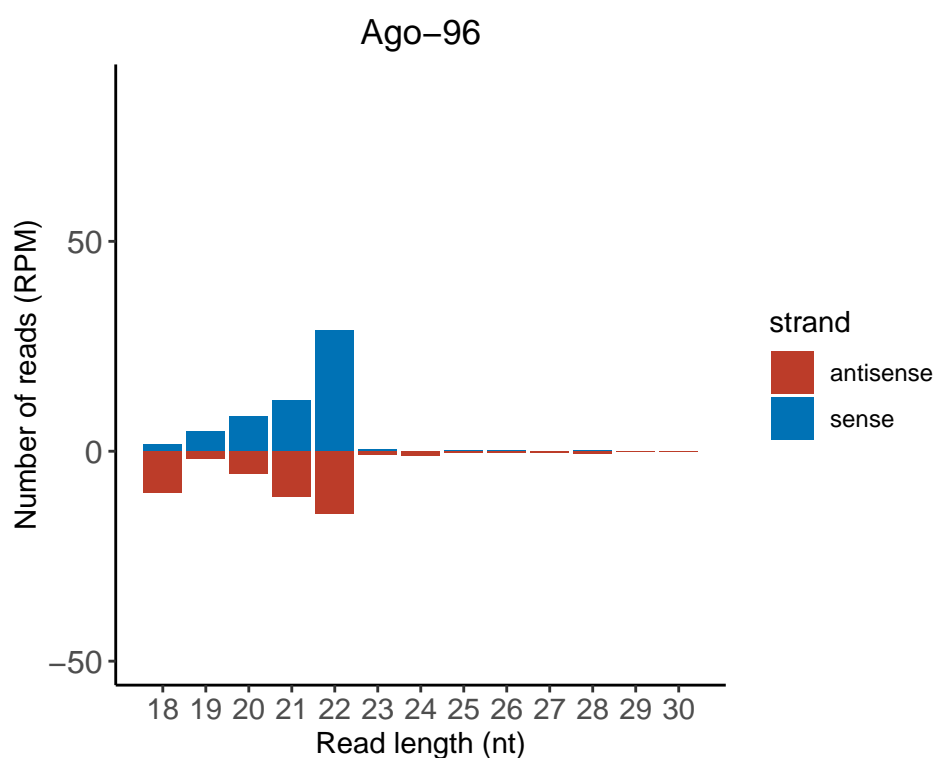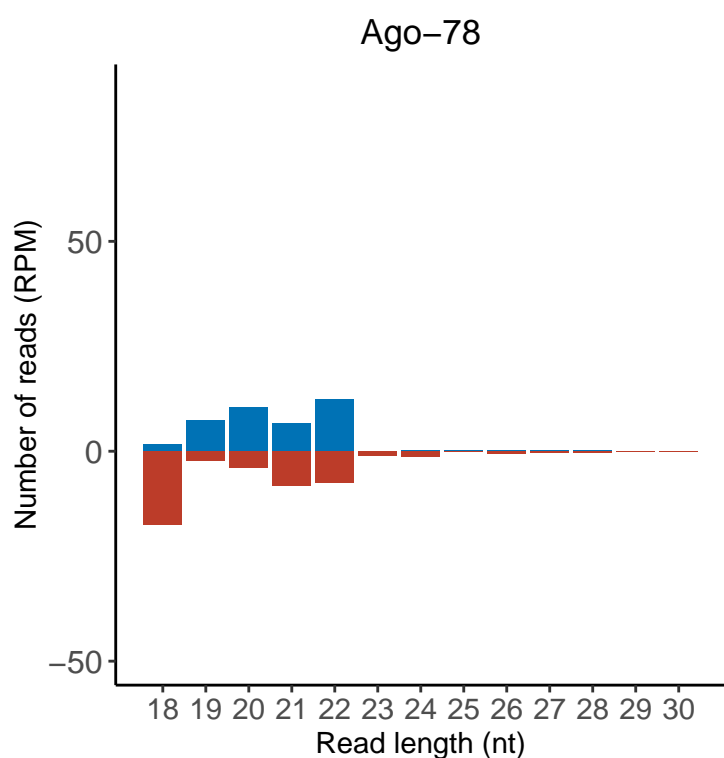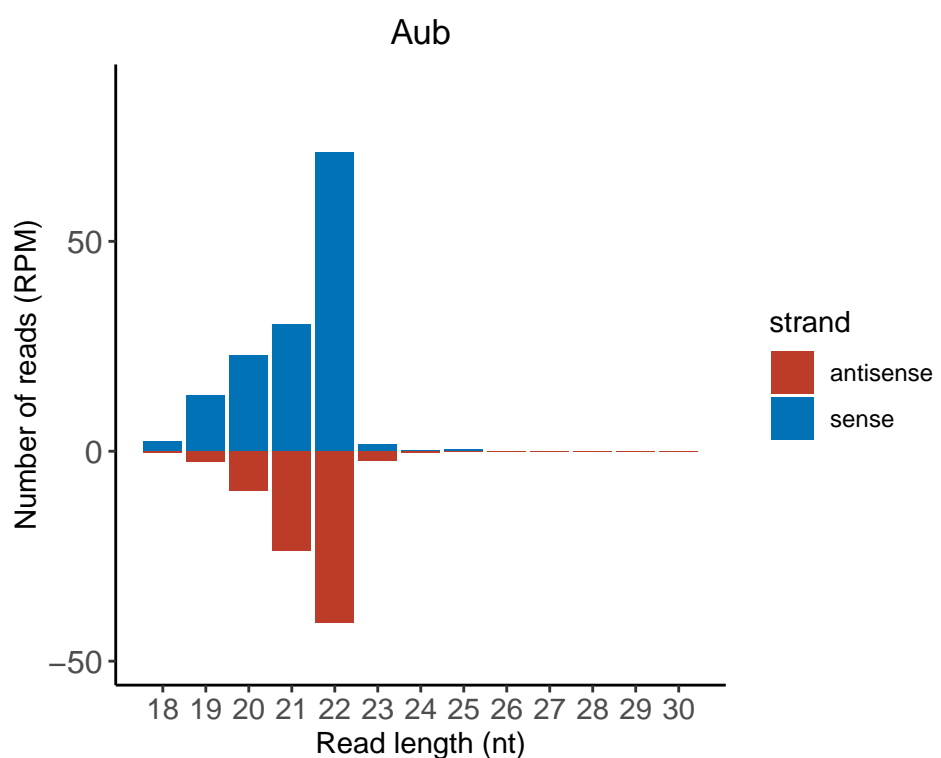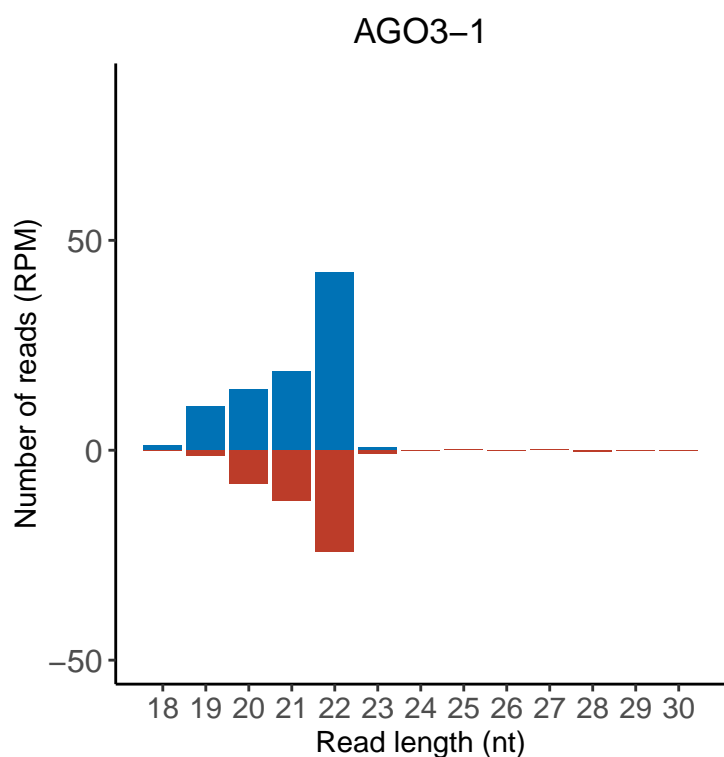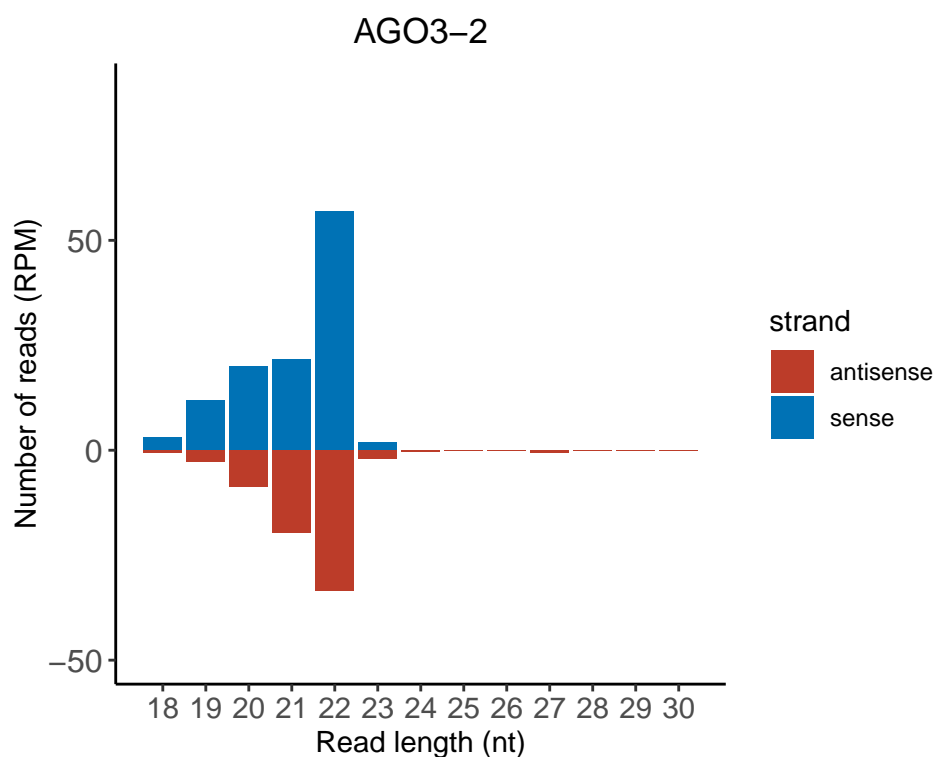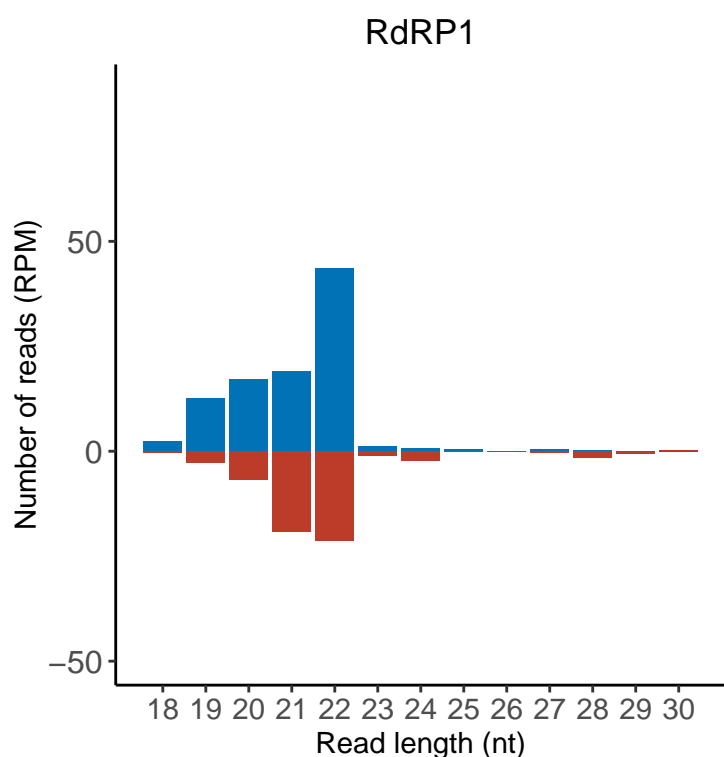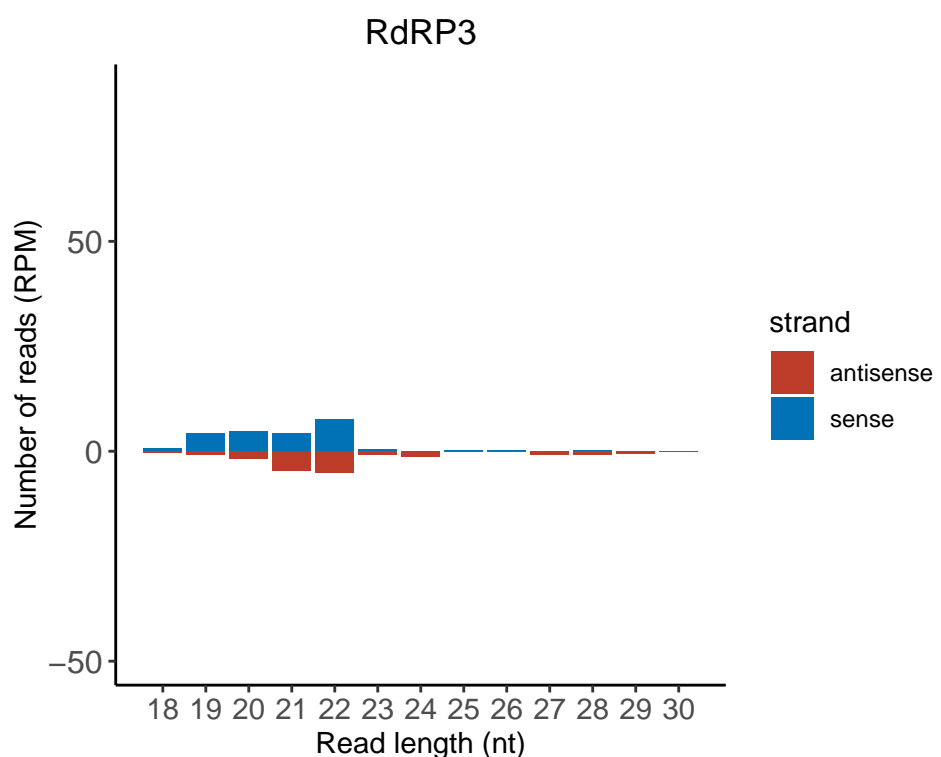

Supplement: S1 Data — On the Normalized and Relative levels pages, normalized read counts (RPM) and relative levels compared to the control (dsGFP) library were used. TEs with more than 50RPM and Coding Genes with more than 3.5RPM on average in KD libraries are included in this website. For viral sequences, reference sequences were generated by assembling sRNA sequences (See the Analysis of sRNAseq data section in Materials and methods.) and size distributions of sRNA reads mapped to each contig are shown. Reads were first grouped by their 5’ nucleotides, and reads of each length were counted. Full tables including TEs and Coding genes with fewer reads are in the “FullTable” folder. (ZIP) [file pone.0281195.s017.zip › SupplementaryData/Links/PDFs/Motif:rnd-6_family-10230TE_lengthdistribution.pdf]

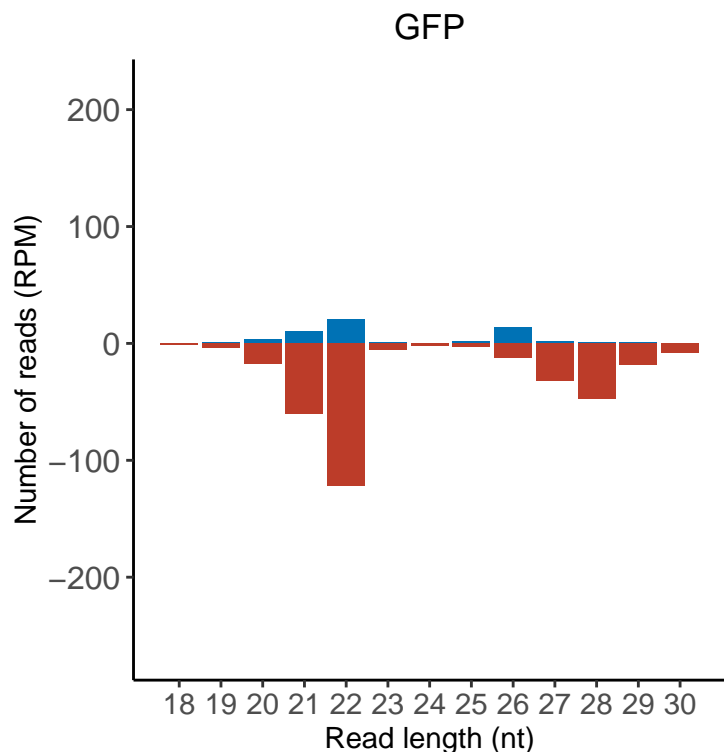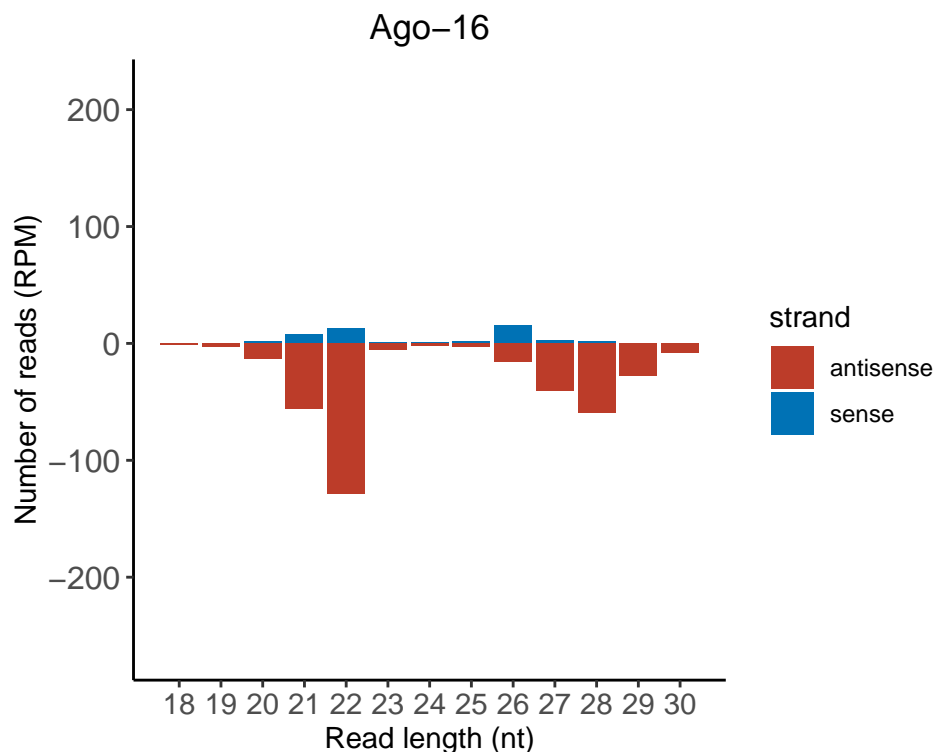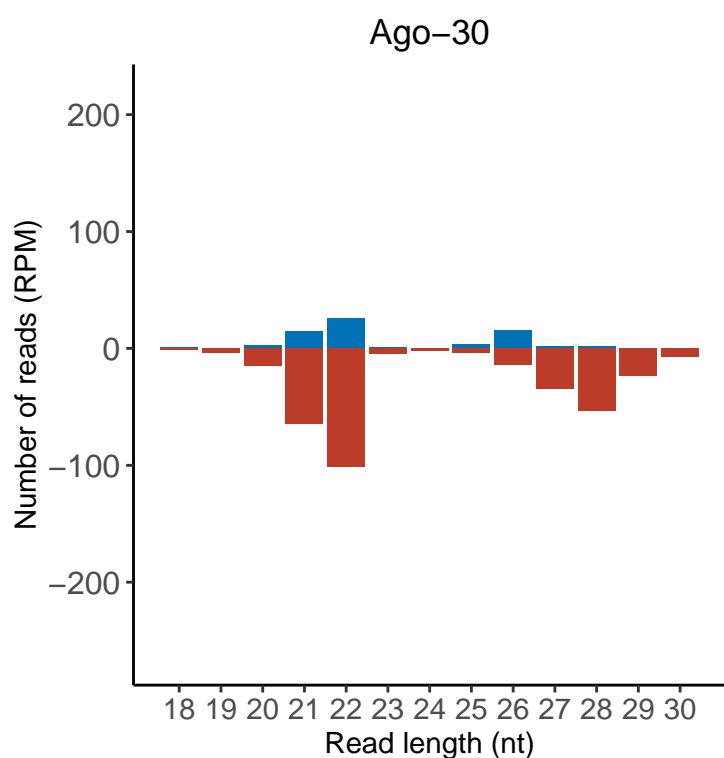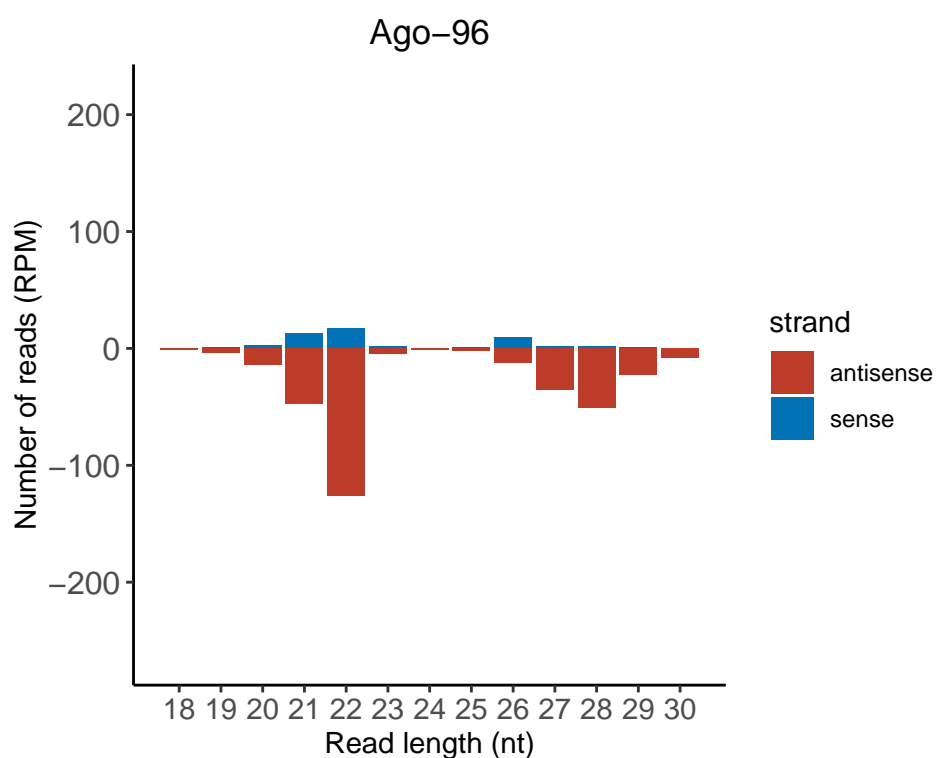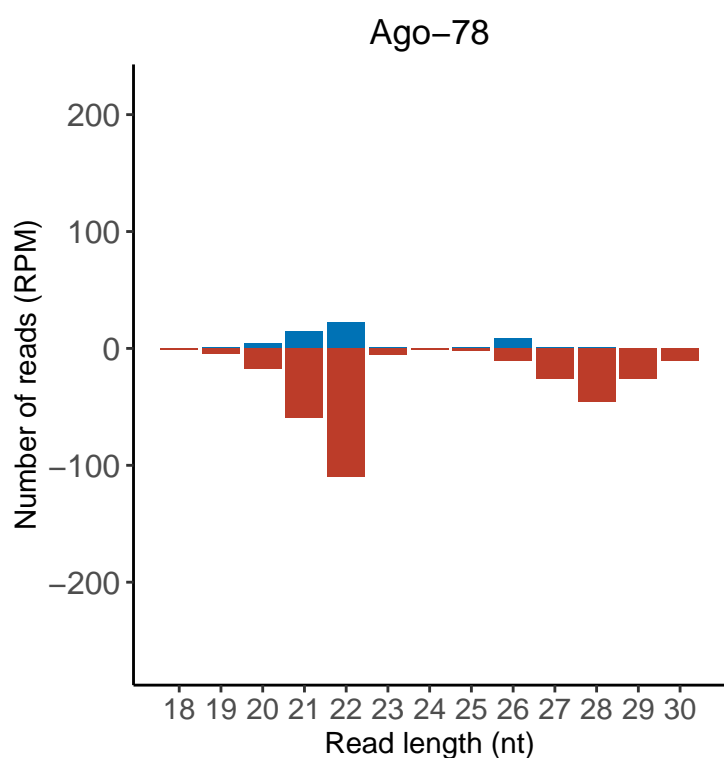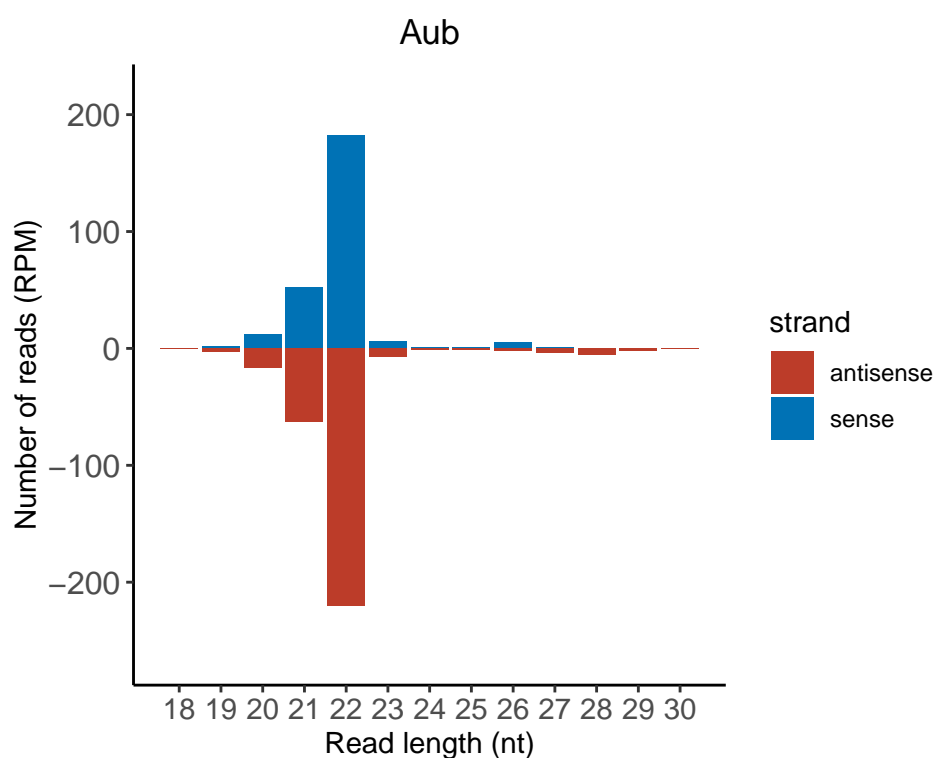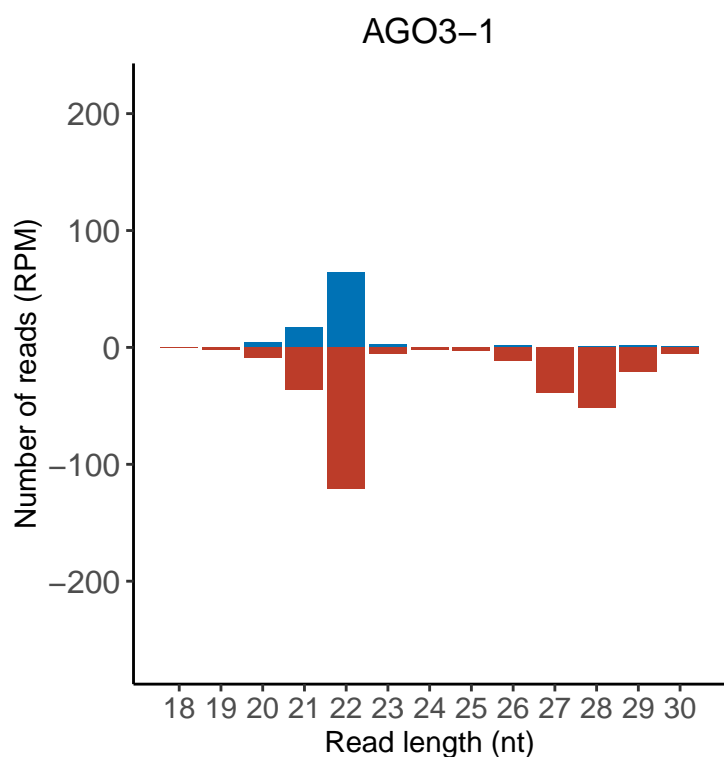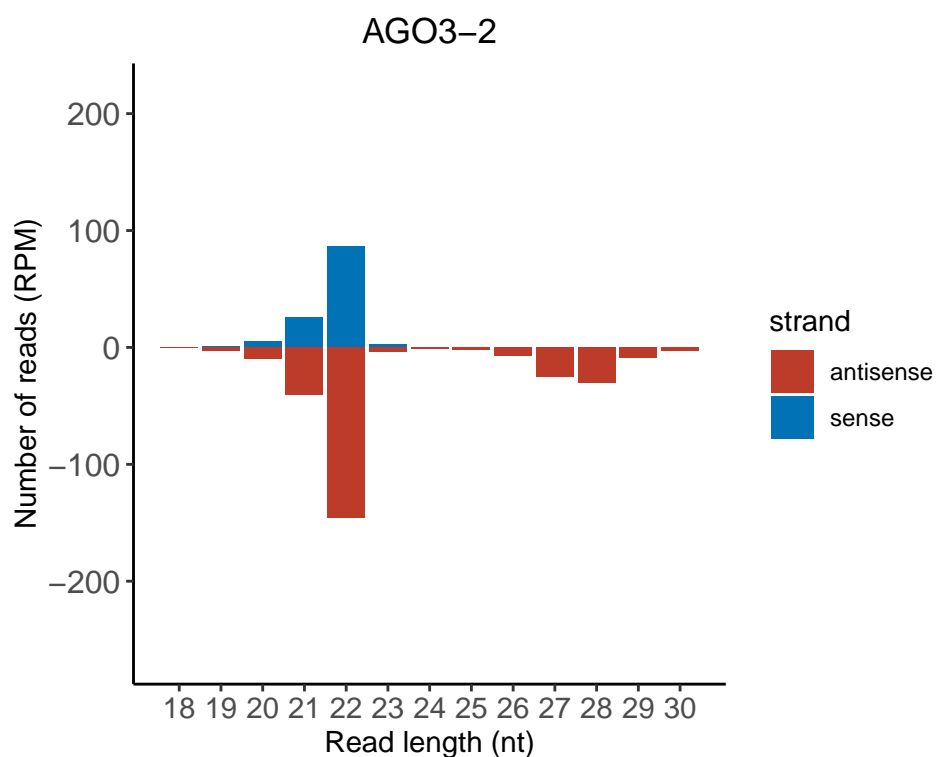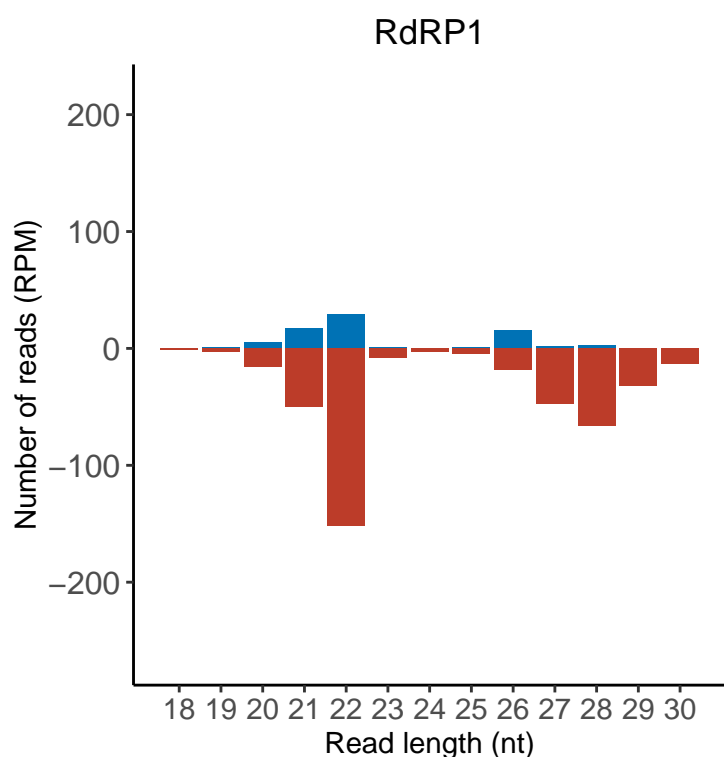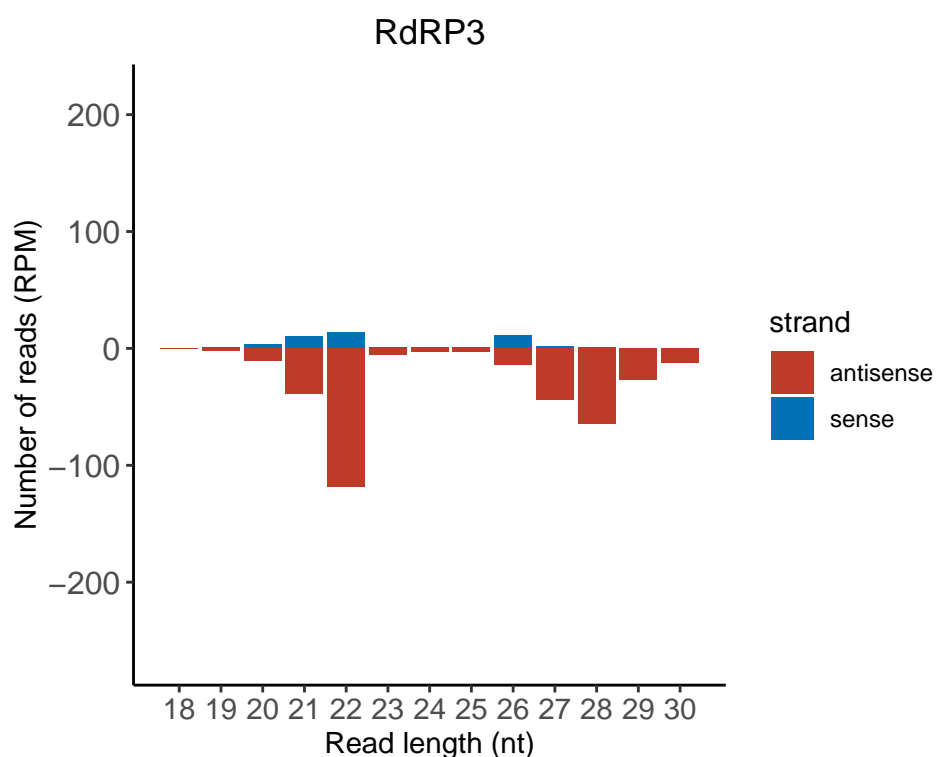

Supplement: S1 Data — On the Normalized and Relative levels pages, normalized read counts (RPM) and relative levels compared to the control (dsGFP) library were used. TEs with more than 50RPM and Coding Genes with more than 3.5RPM on average in KD libraries are included in this website. For viral sequences, reference sequences were generated by assembling sRNA sequences (See the Analysis of sRNAseq data section in Materials and methods.) and size distributions of sRNA reads mapped to each contig are shown. Reads were first grouped by their 5’ nucleotides, and reads of each length were counted. Full tables including TEs and Coding genes with fewer reads are in the “FullTable” folder. (ZIP) [file pone.0281195.s017.zip › SupplementaryData/Links/PDFs/Motif:rnd-1_family-1113TE_lengthdistribution.pdf]

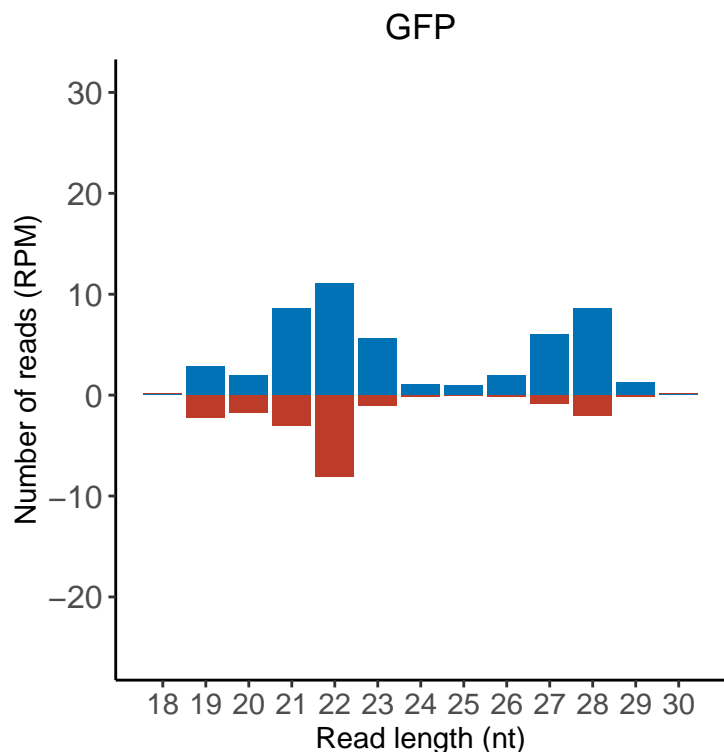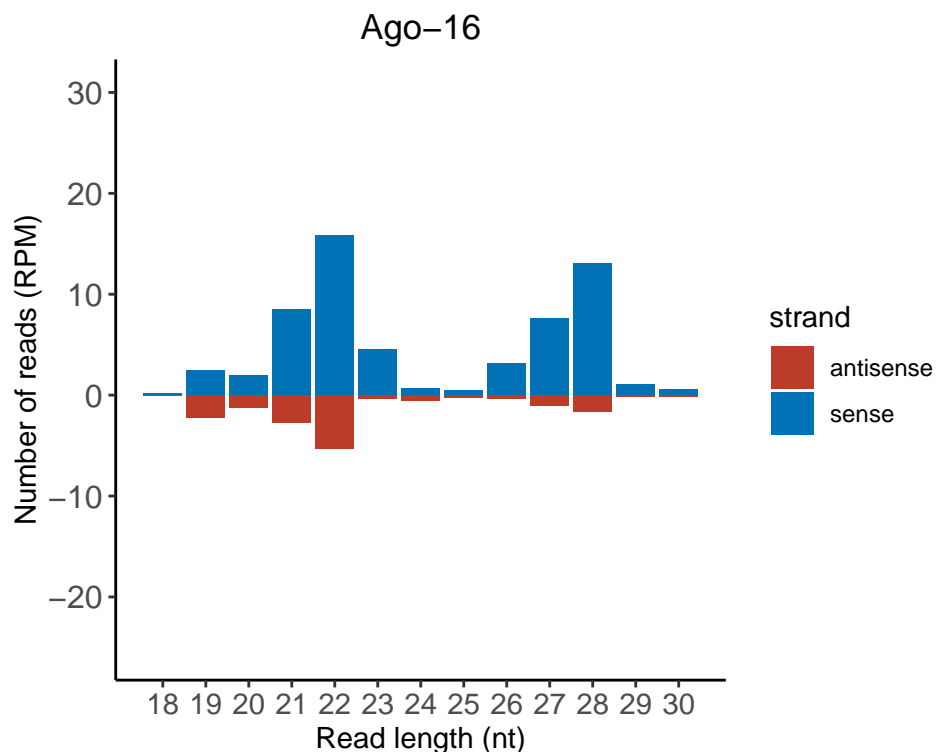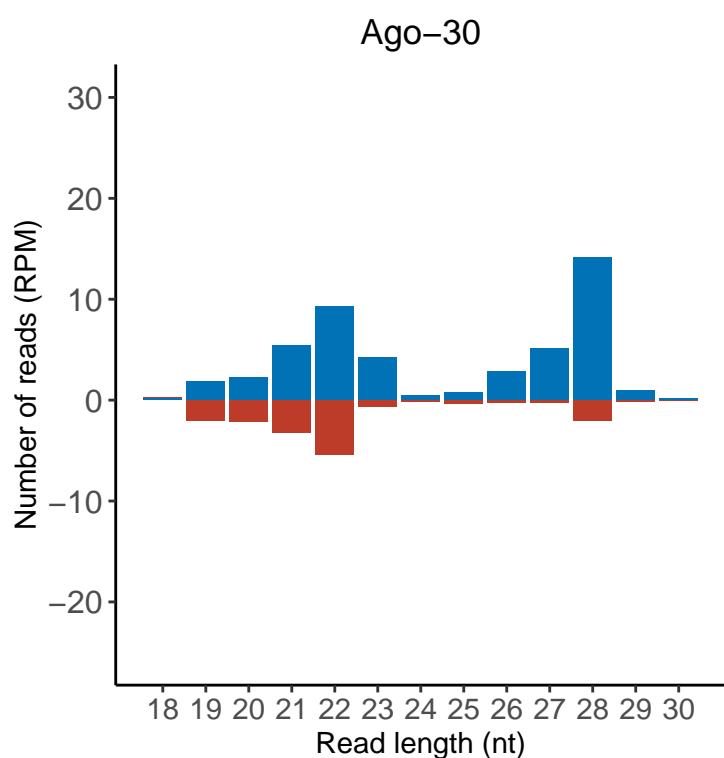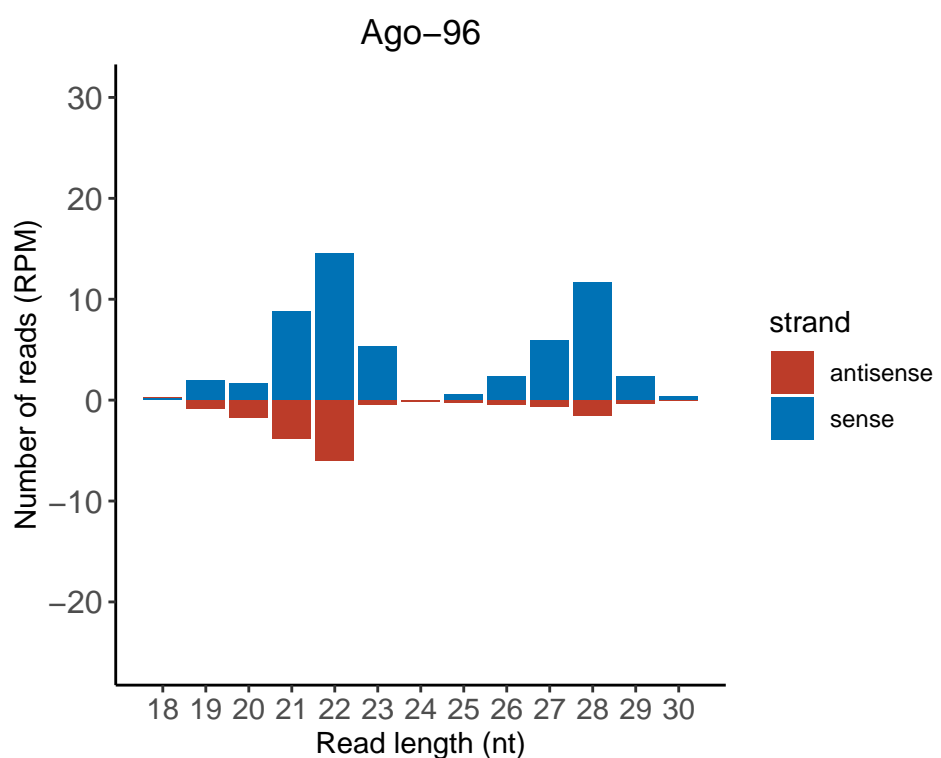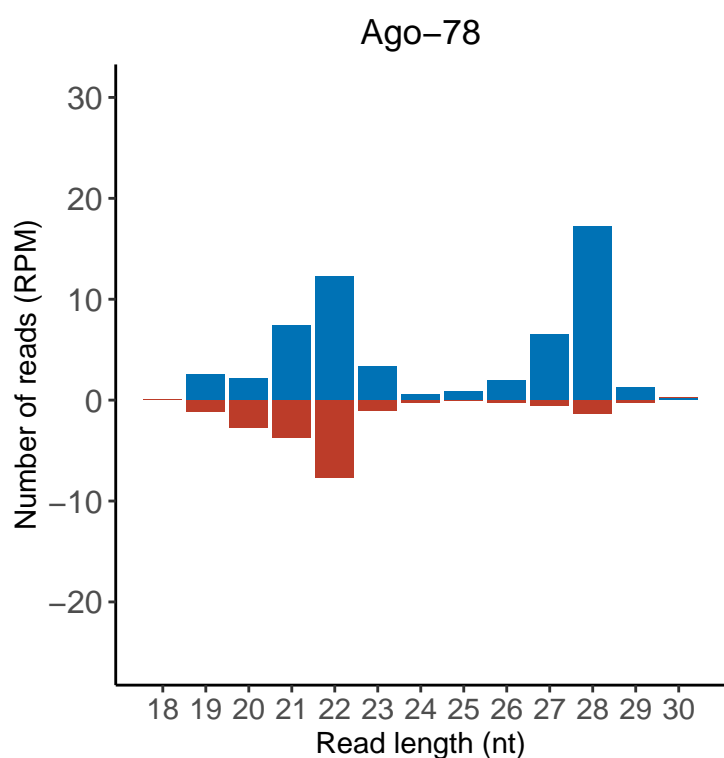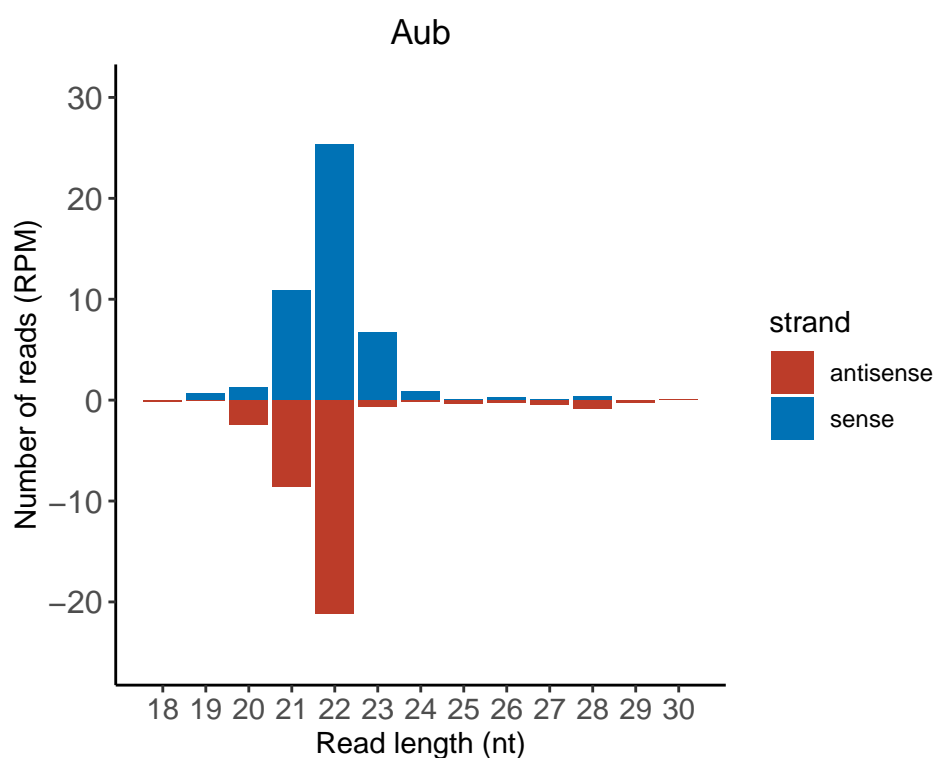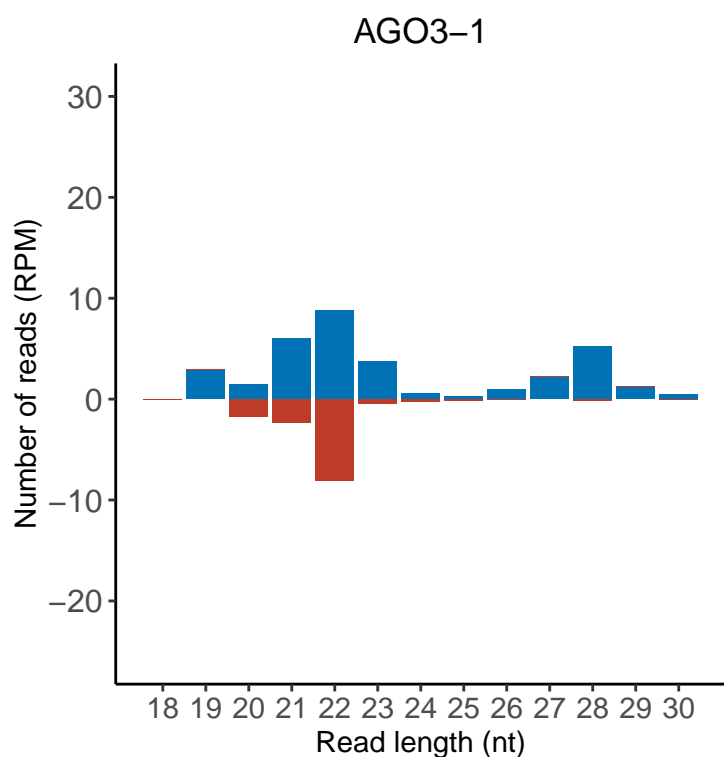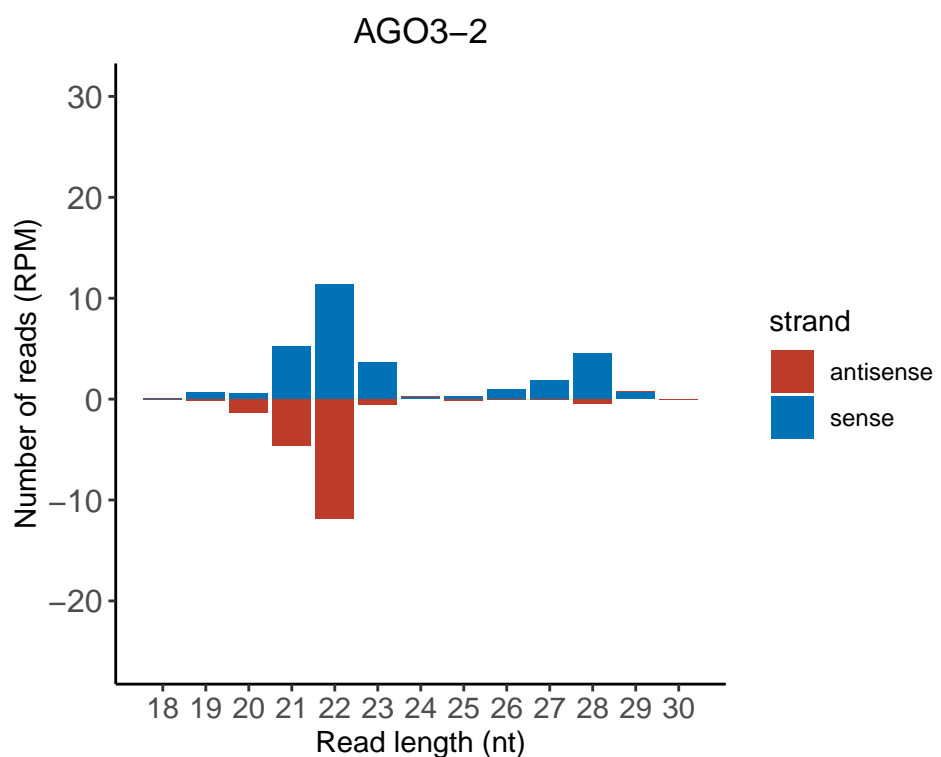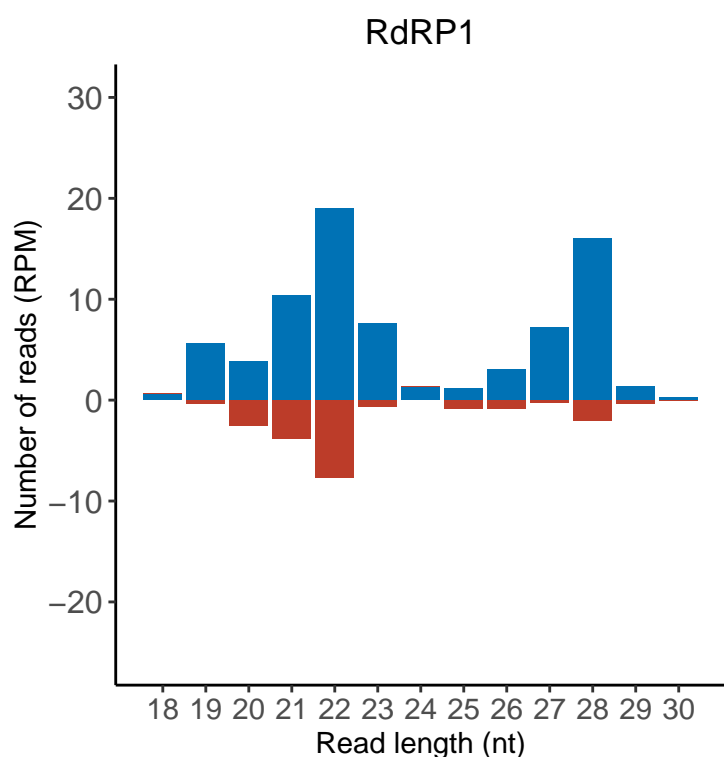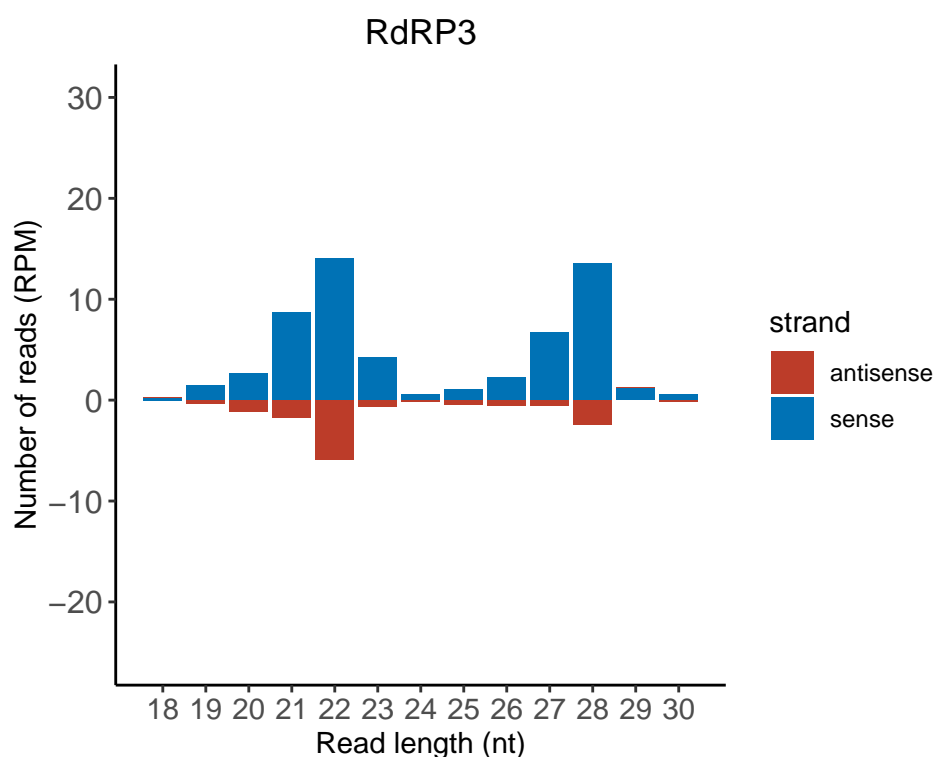

Supplement: S1 Data — On the Normalized and Relative levels pages, normalized read counts (RPM) and relative levels compared to the control (dsGFP) library were used. TEs with more than 50RPM and Coding Genes with more than 3.5RPM on average in KD libraries are included in this website. For viral sequences, reference sequences were generated by assembling sRNA sequences (See the Analysis of sRNAseq data section in Materials and methods.) and size distributions of sRNA reads mapped to each contig are shown. Reads were first grouped by their 5’ nucleotides, and reads of each length were counted. Full tables including TEs and Coding genes with fewer reads are in the “FullTable” folder. (ZIP) [file pone.0281195.s017.zip › SupplementaryData/Links/PDFs/Motif:rnd-5_family-2877TE_lengthdistribution.pdf]

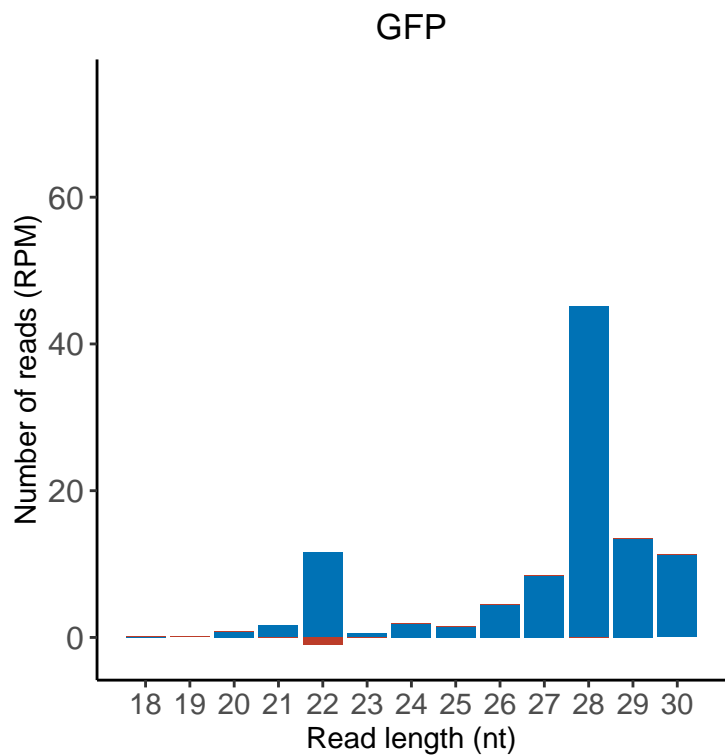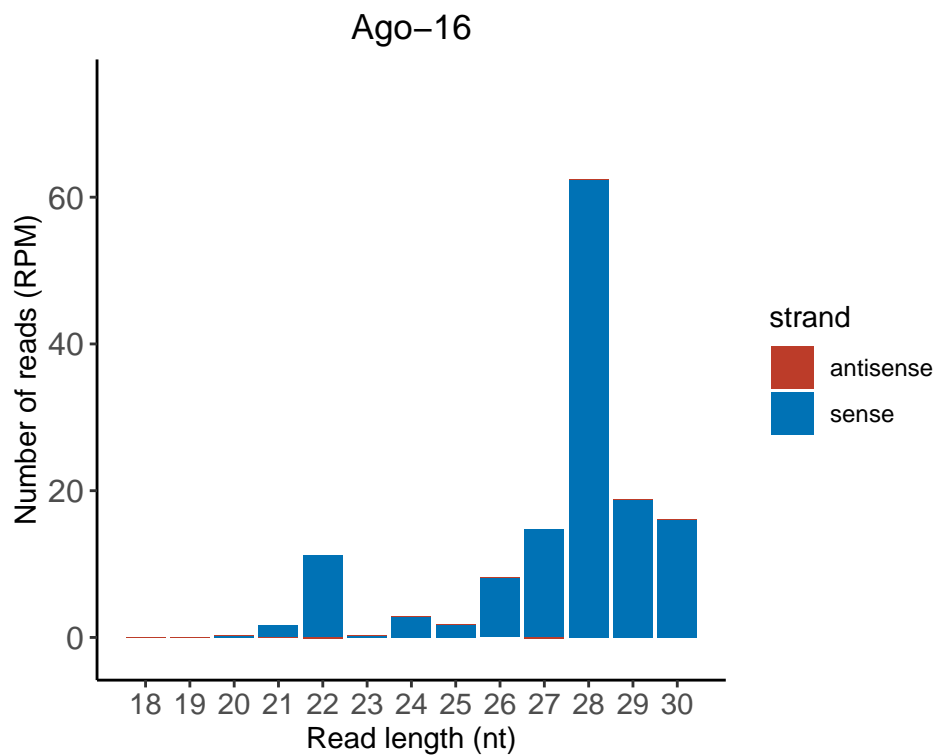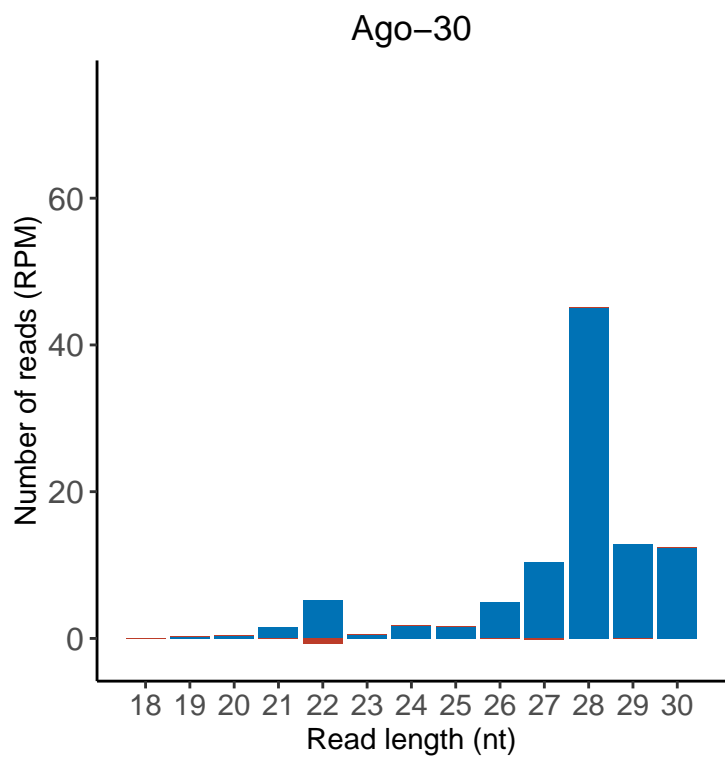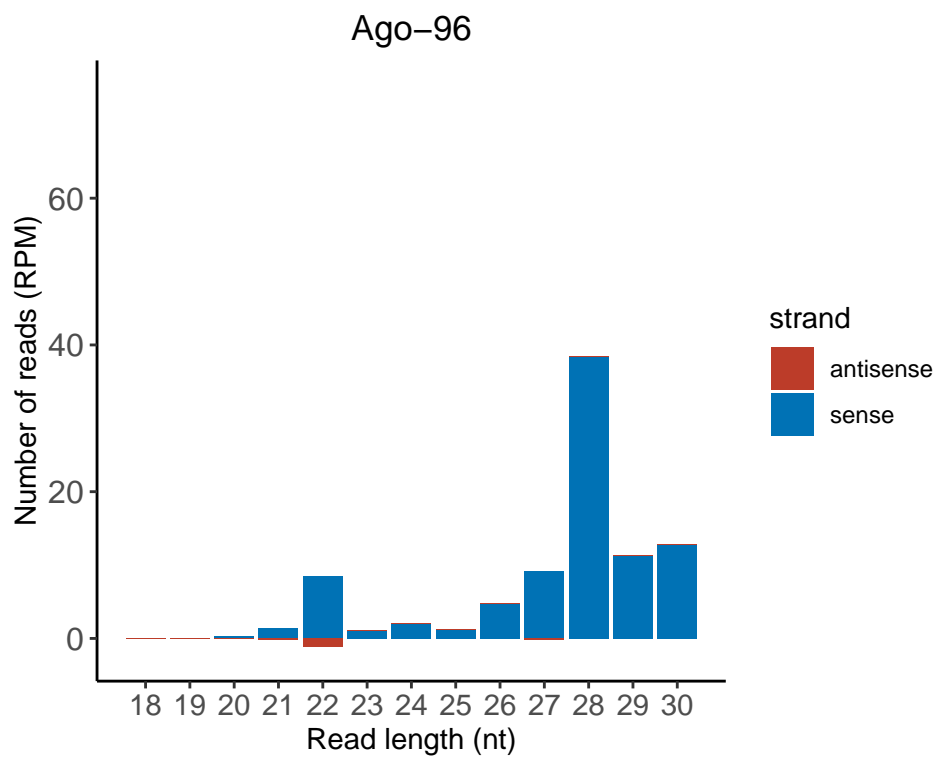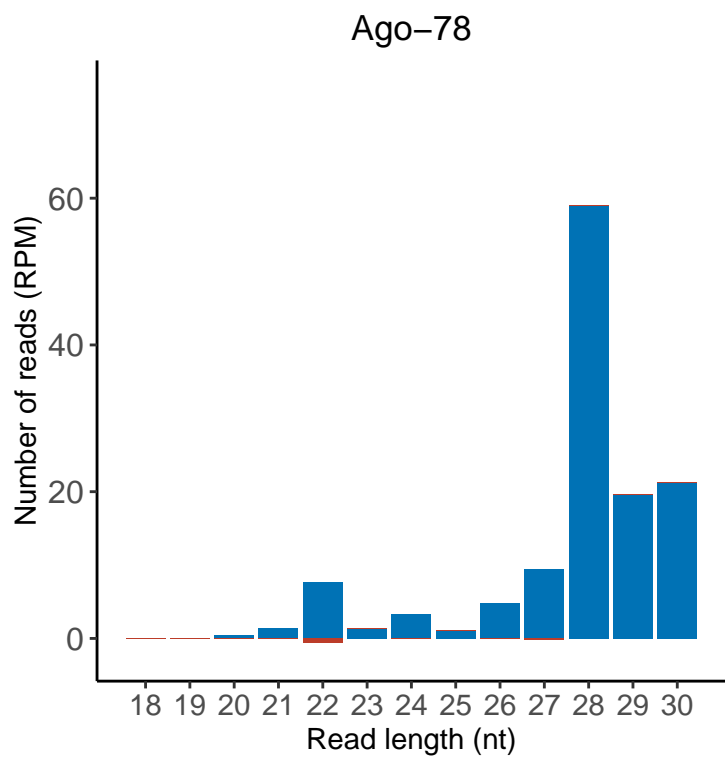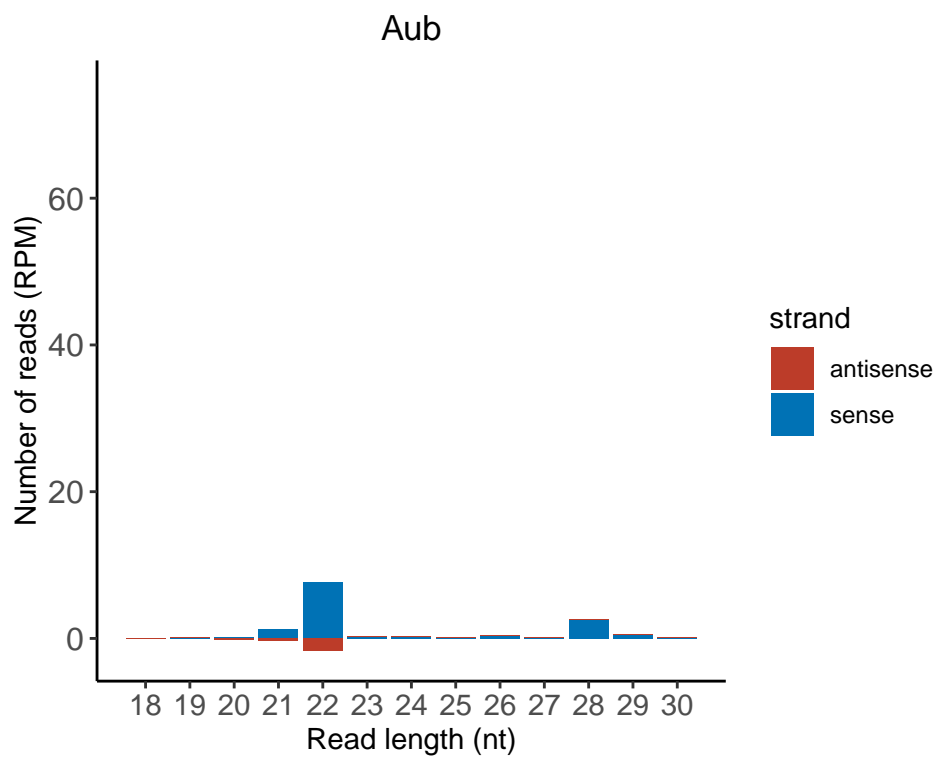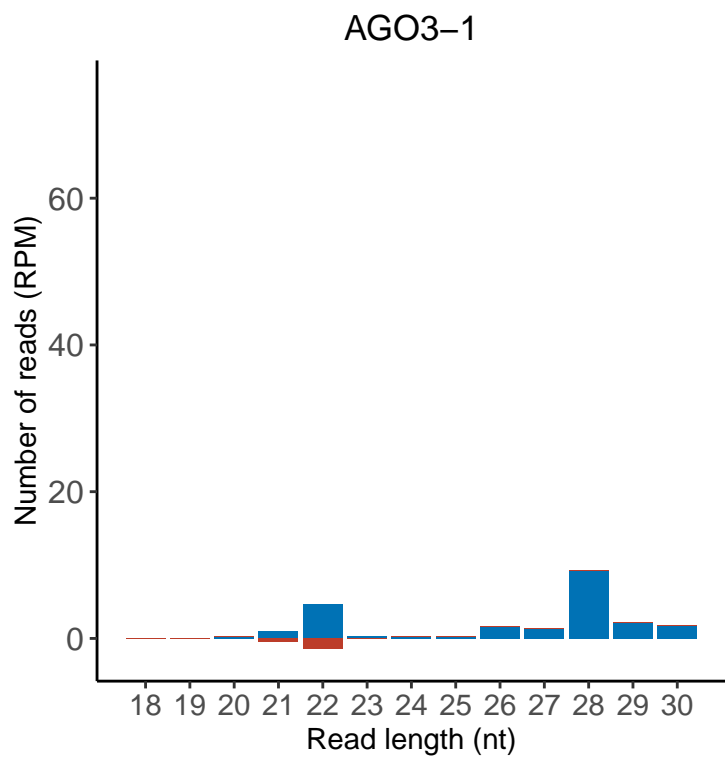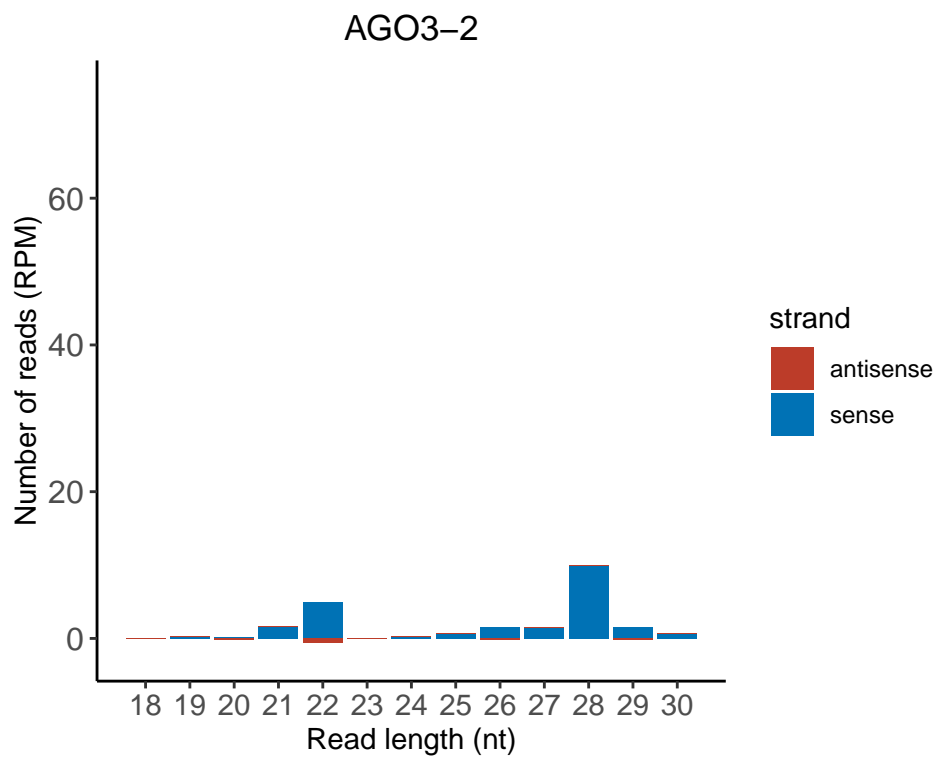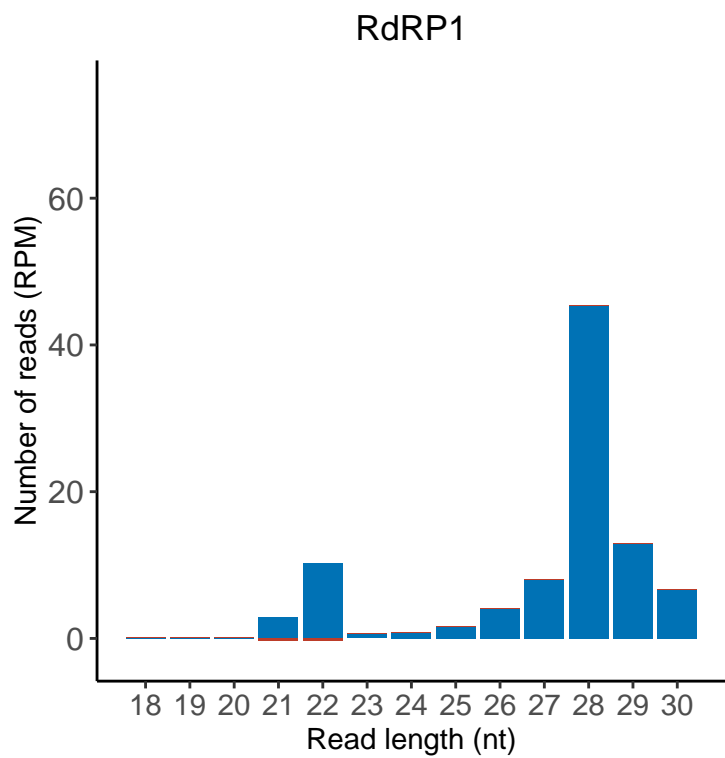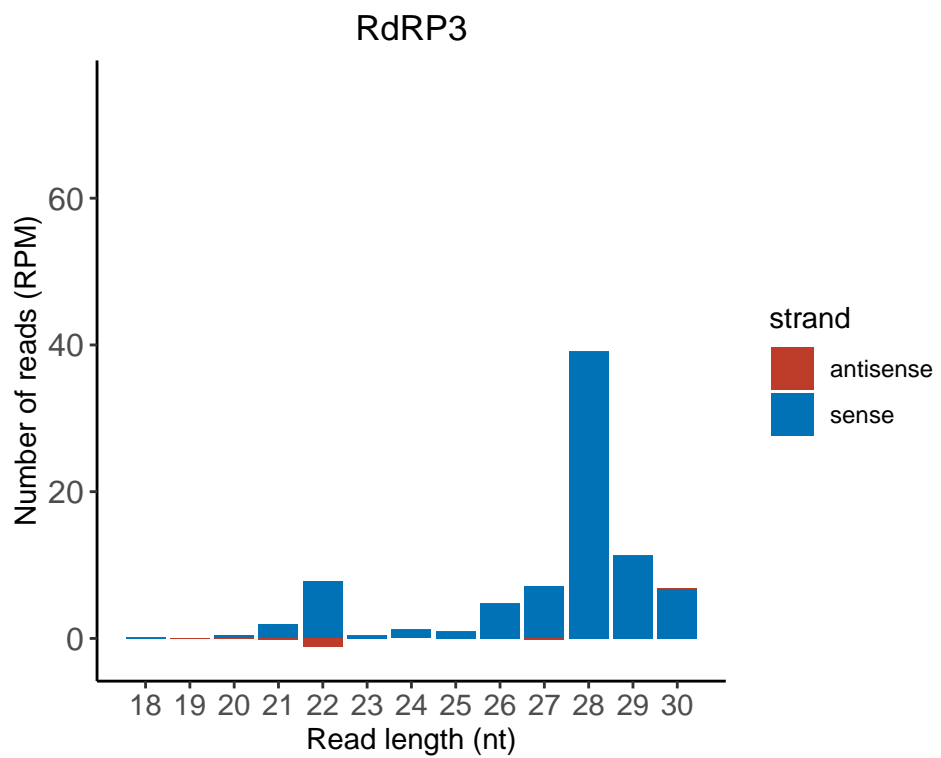

Supplement: S1 Data — On the Normalized and Relative levels pages, normalized read counts (RPM) and relative levels compared to the control (dsGFP) library were used. TEs with more than 50RPM and Coding Genes with more than 3.5RPM on average in KD libraries are included in this website. For viral sequences, reference sequences were generated by assembling sRNA sequences (See the Analysis of sRNAseq data section in Materials and methods.) and size distributions of sRNA reads mapped to each contig are shown. Reads were first grouped by their 5’ nucleotides, and reads of each length were counted. Full tables including TEs and Coding genes with fewer reads are in the “FullTable” folder. (ZIP) [file pone.0281195.s017.zip › SupplementaryData/Links/PDFs/Motif:rnd-6_family-10845TE_lengthdistribution.pdf]

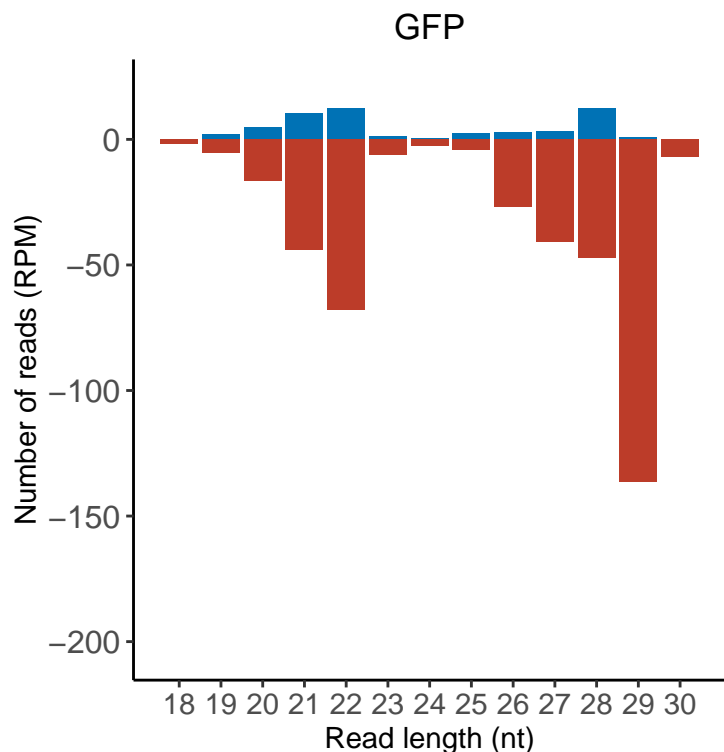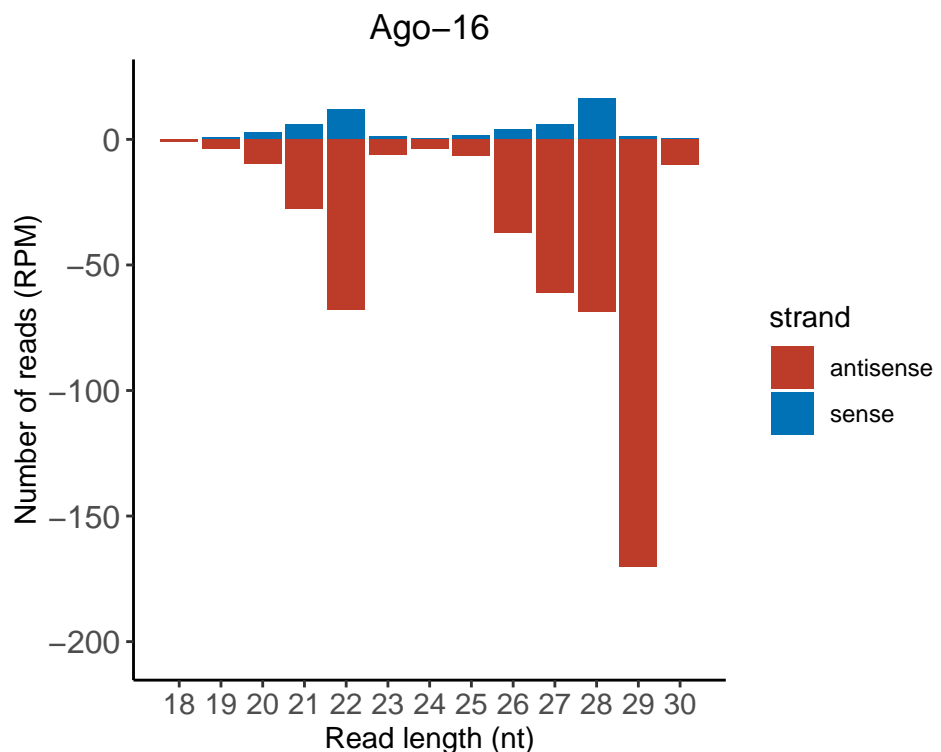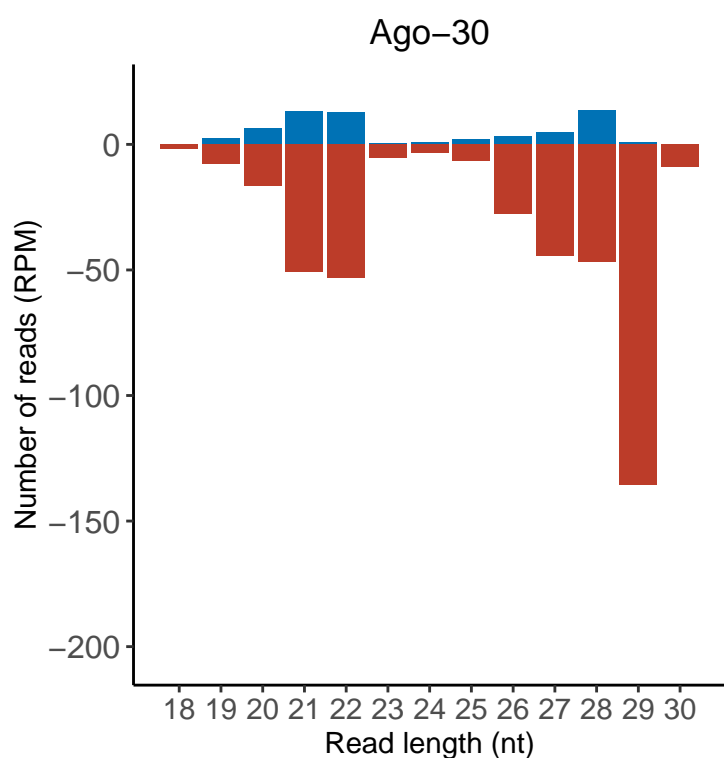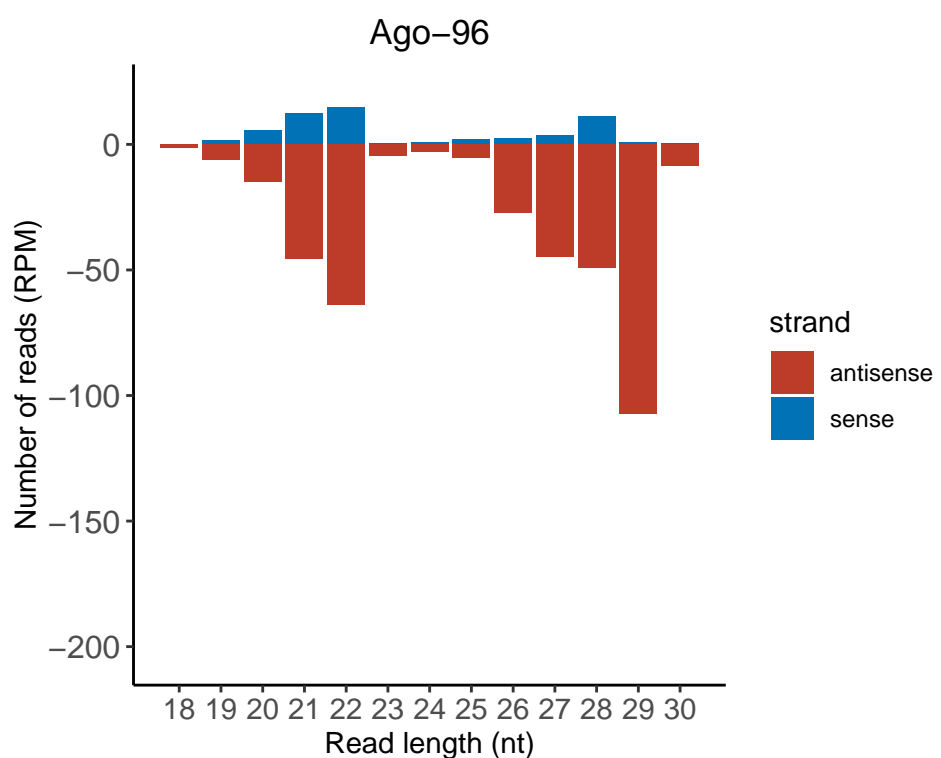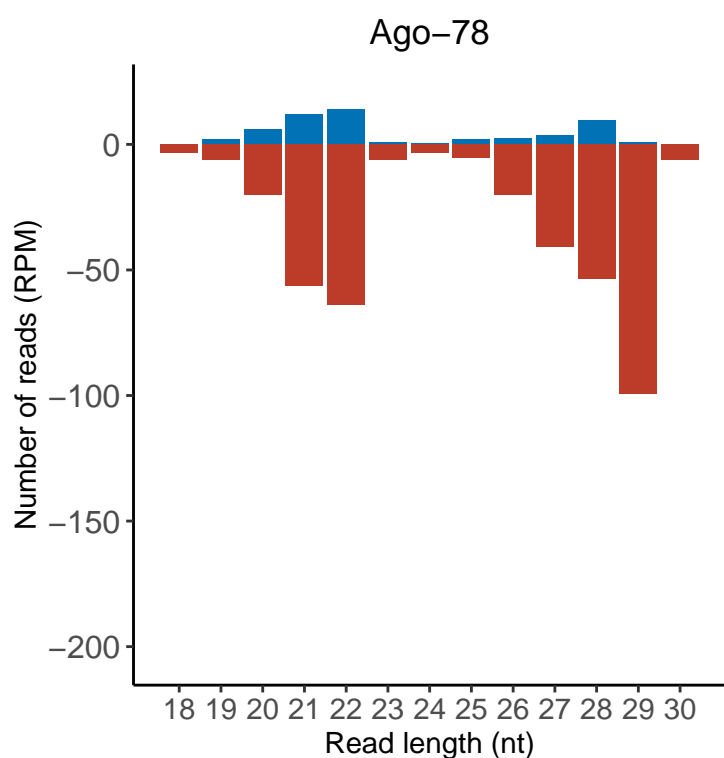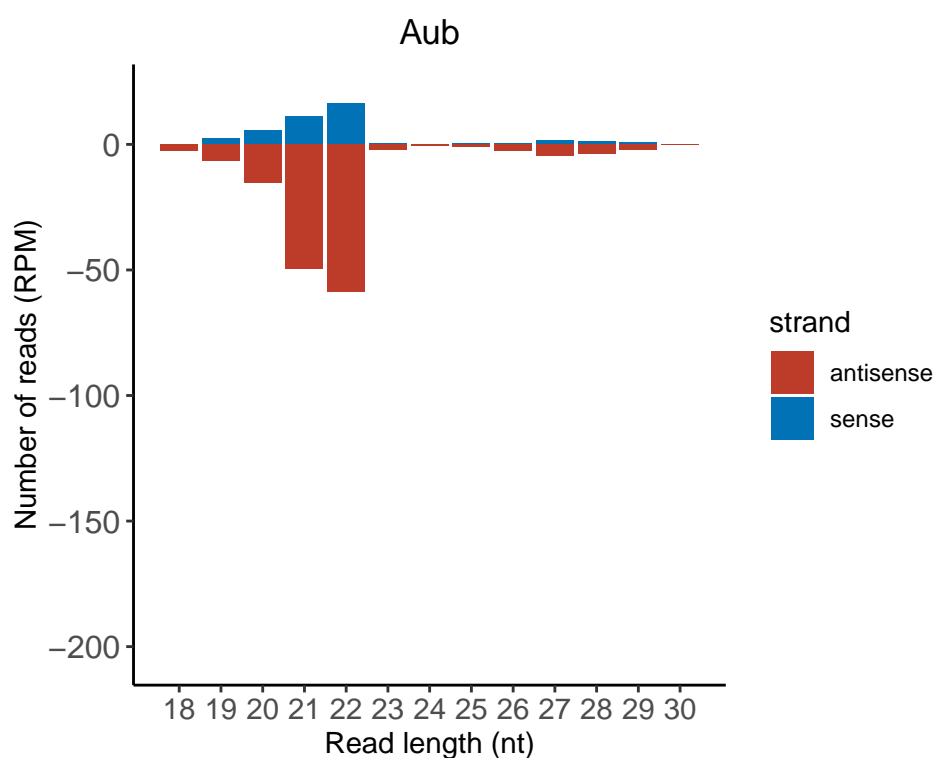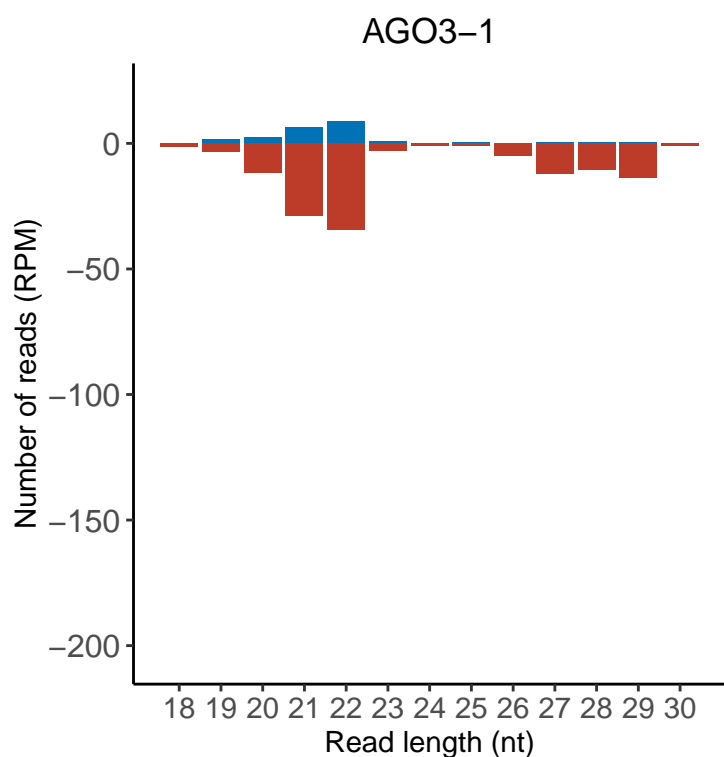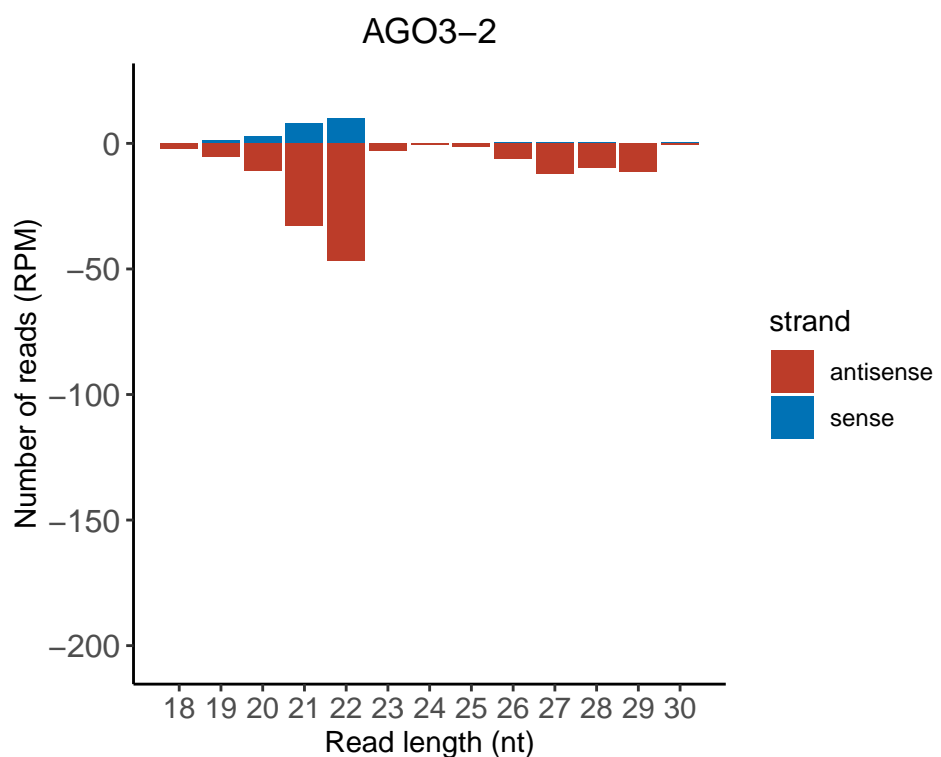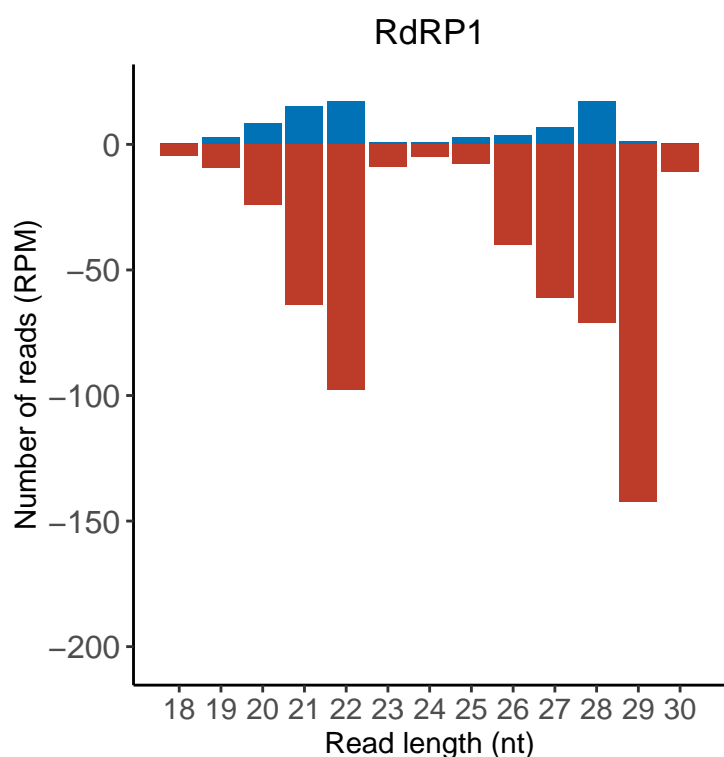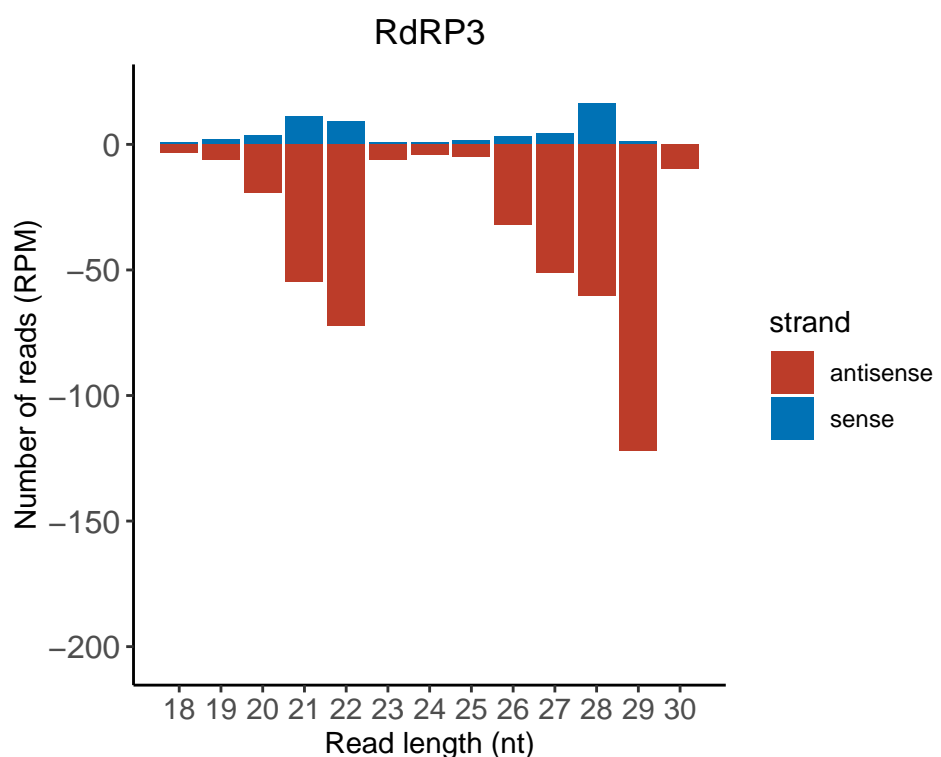

Supplement: S1 Data — On the Normalized and Relative levels pages, normalized read counts (RPM) and relative levels compared to the control (dsGFP) library were used. TEs with more than 50RPM and Coding Genes with more than 3.5RPM on average in KD libraries are included in this website. For viral sequences, reference sequences were generated by assembling sRNA sequences (See the Analysis of sRNAseq data section in Materials and methods.) and size distributions of sRNA reads mapped to each contig are shown. Reads were first grouped by their 5’ nucleotides, and reads of each length were counted. Full tables including TEs and Coding genes with fewer reads are in the “FullTable” folder. (ZIP) [file pone.0281195.s017.zip › SupplementaryData/Links/PDFs/Motif:rnd-6_family-3909TE_lengthdistribution.pdf]

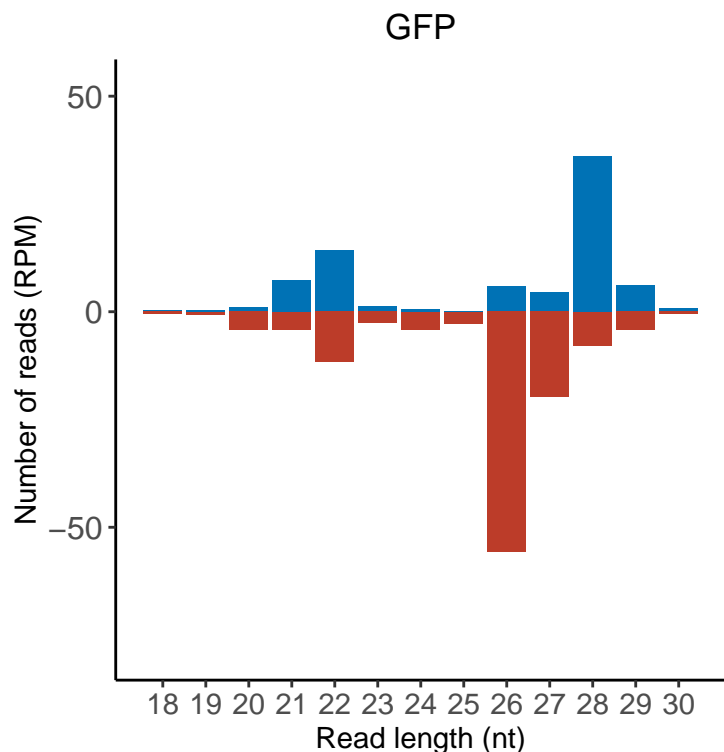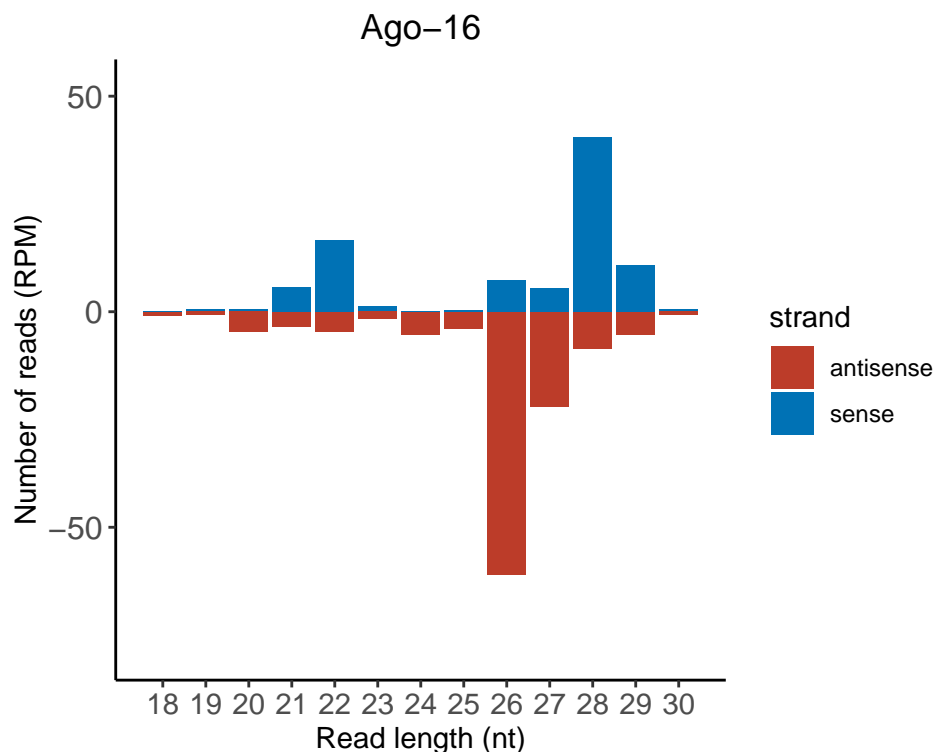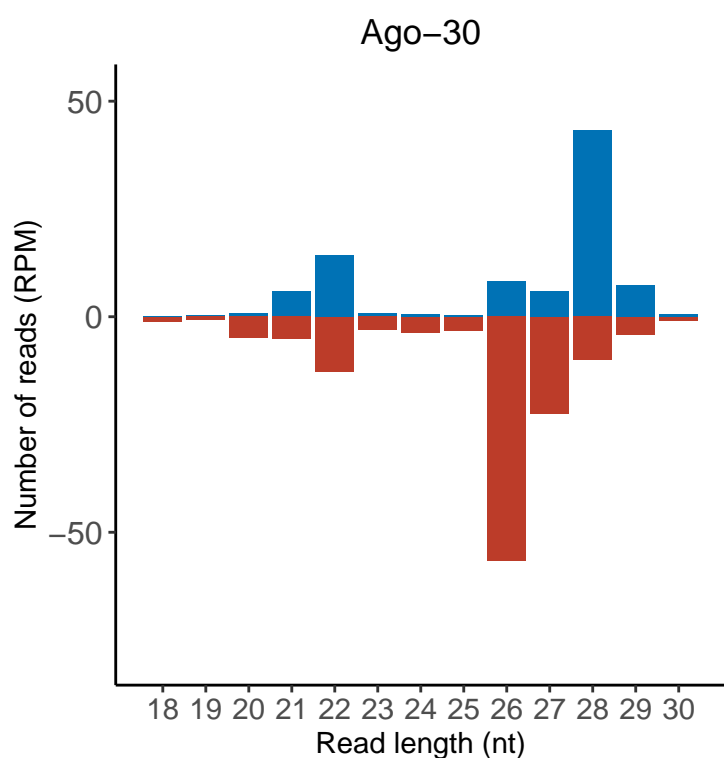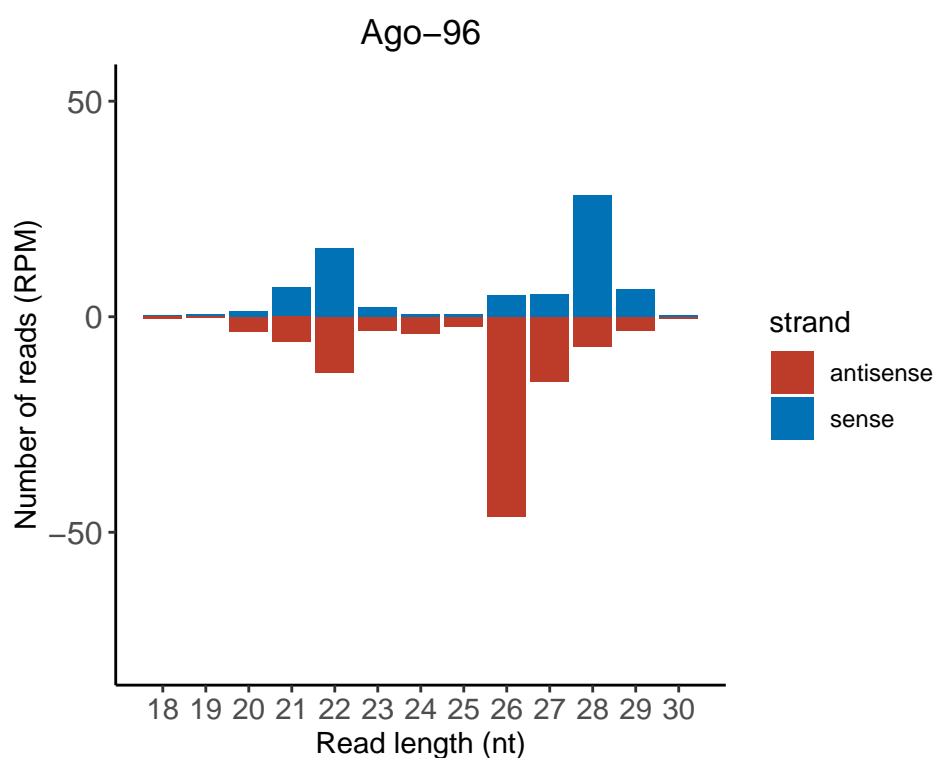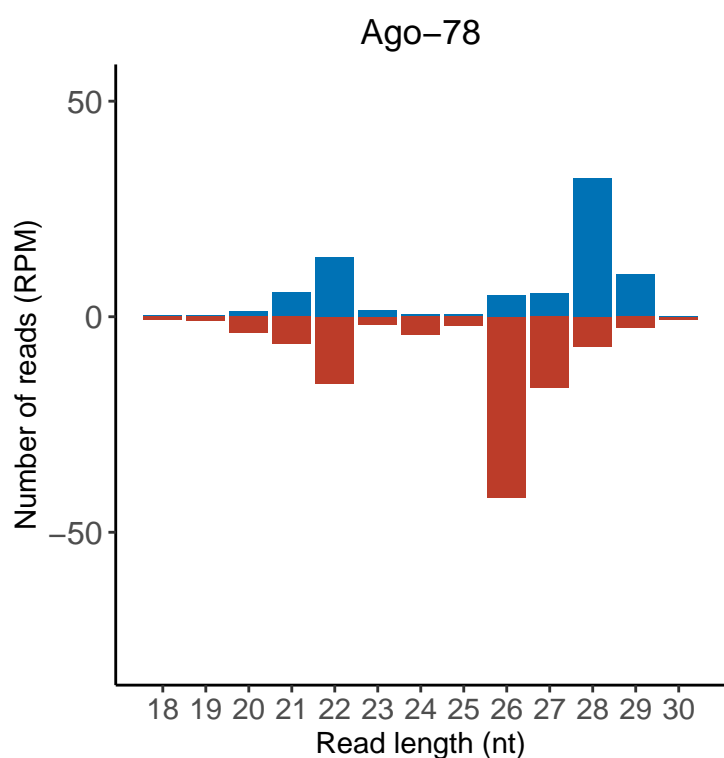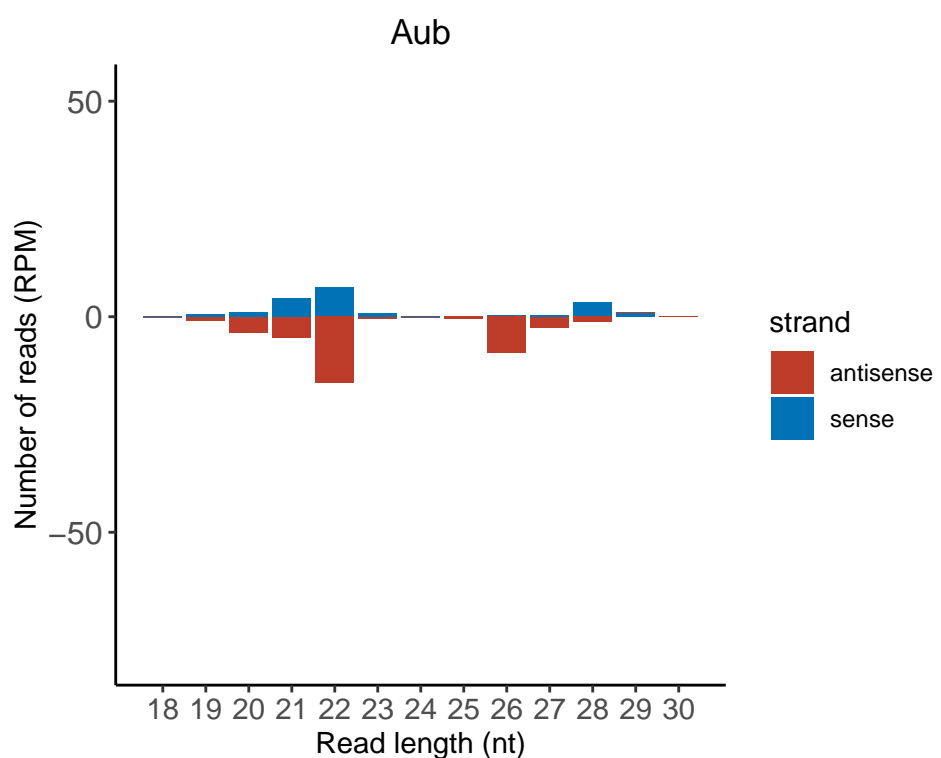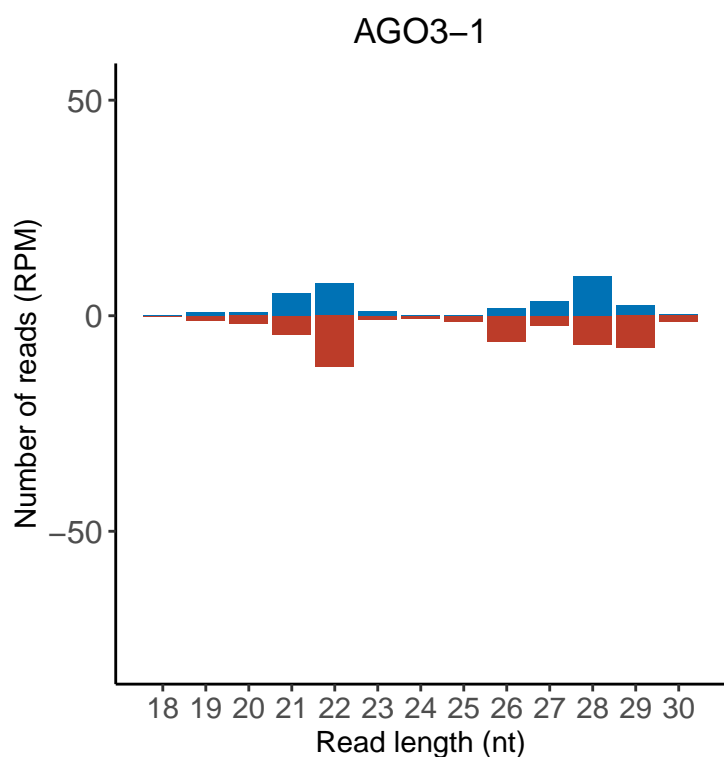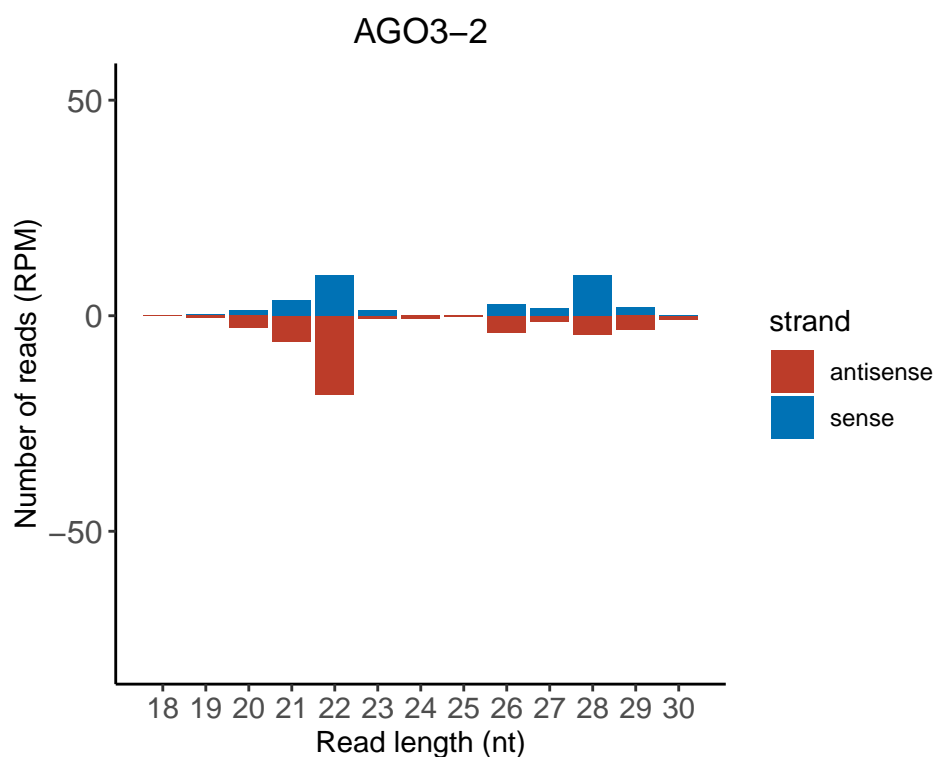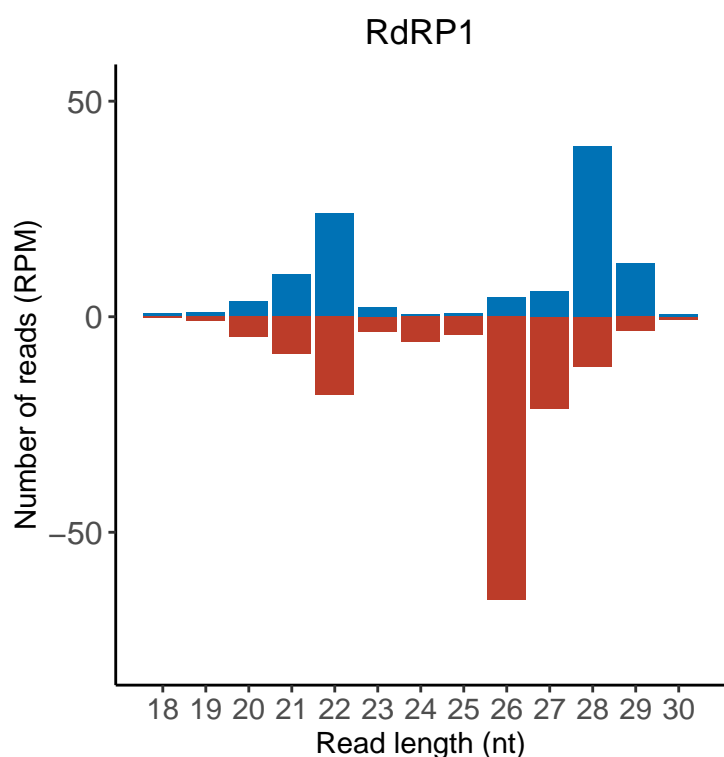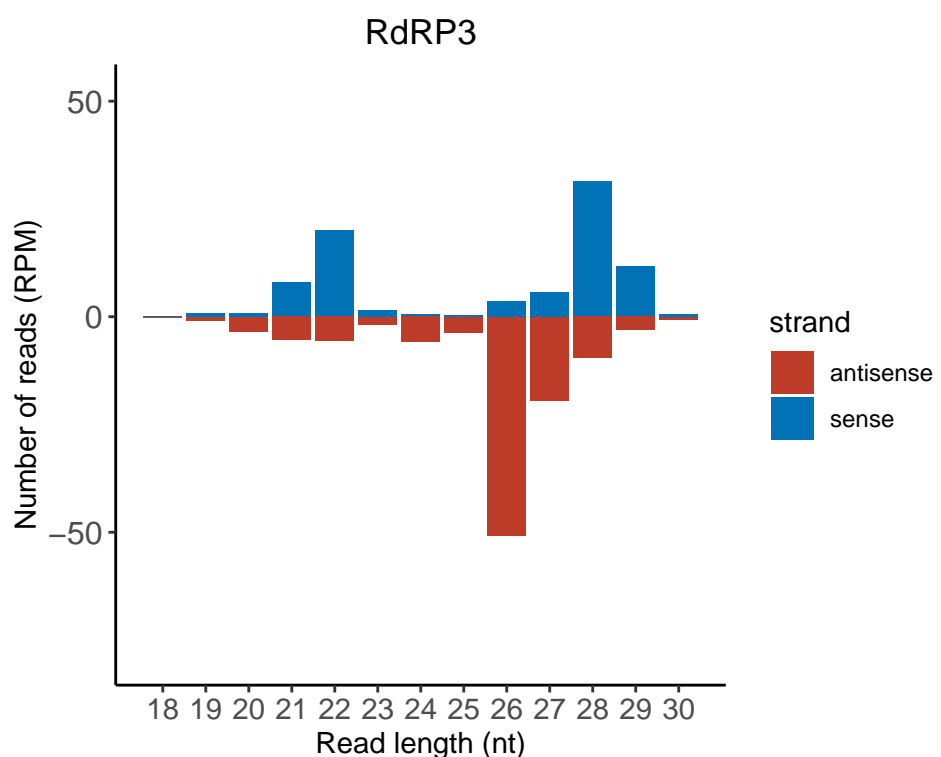

Supplement: S1 Data — On the Normalized and Relative levels pages, normalized read counts (RPM) and relative levels compared to the control (dsGFP) library were used. TEs with more than 50RPM and Coding Genes with more than 3.5RPM on average in KD libraries are included in this website. For viral sequences, reference sequences were generated by assembling sRNA sequences (See the Analysis of sRNAseq data section in Materials and methods.) and size distributions of sRNA reads mapped to each contig are shown. Reads were first grouped by their 5’ nucleotides, and reads of each length were counted. Full tables including TEs and Coding genes with fewer reads are in the “FullTable” folder. (ZIP) [file pone.0281195.s017.zip › SupplementaryData/Links/PDFs/Motif:rnd-6_family-12619TE_lengthdistribution.pdf]

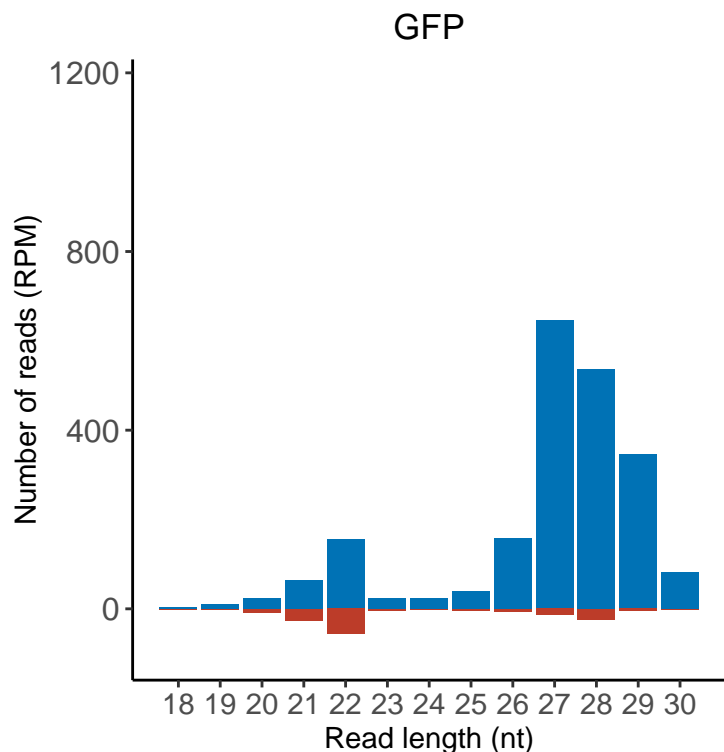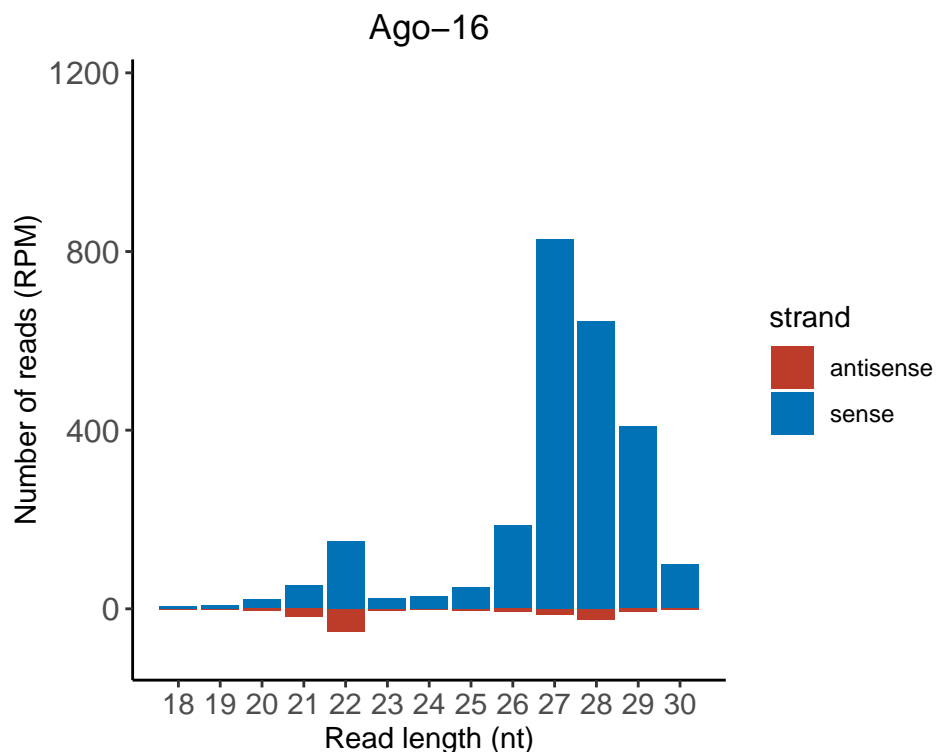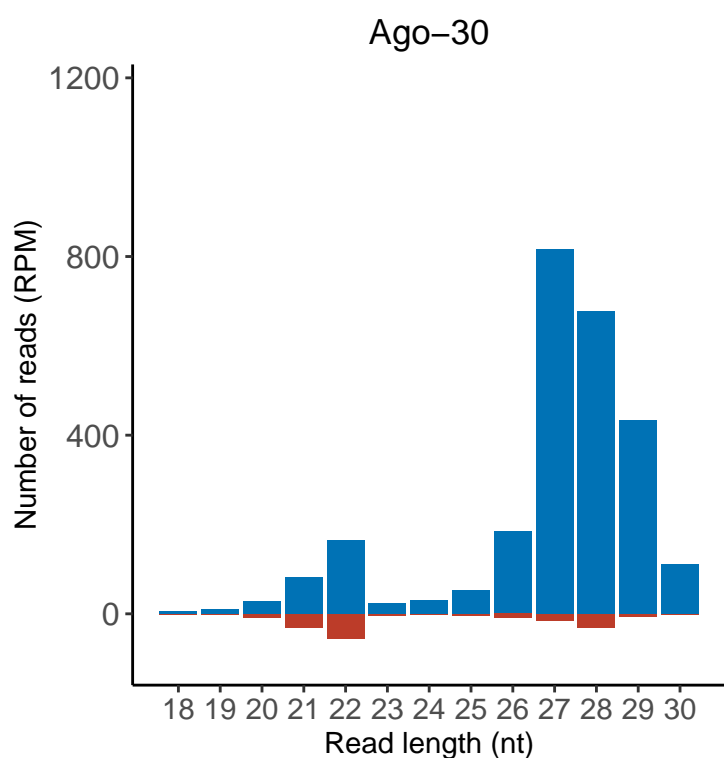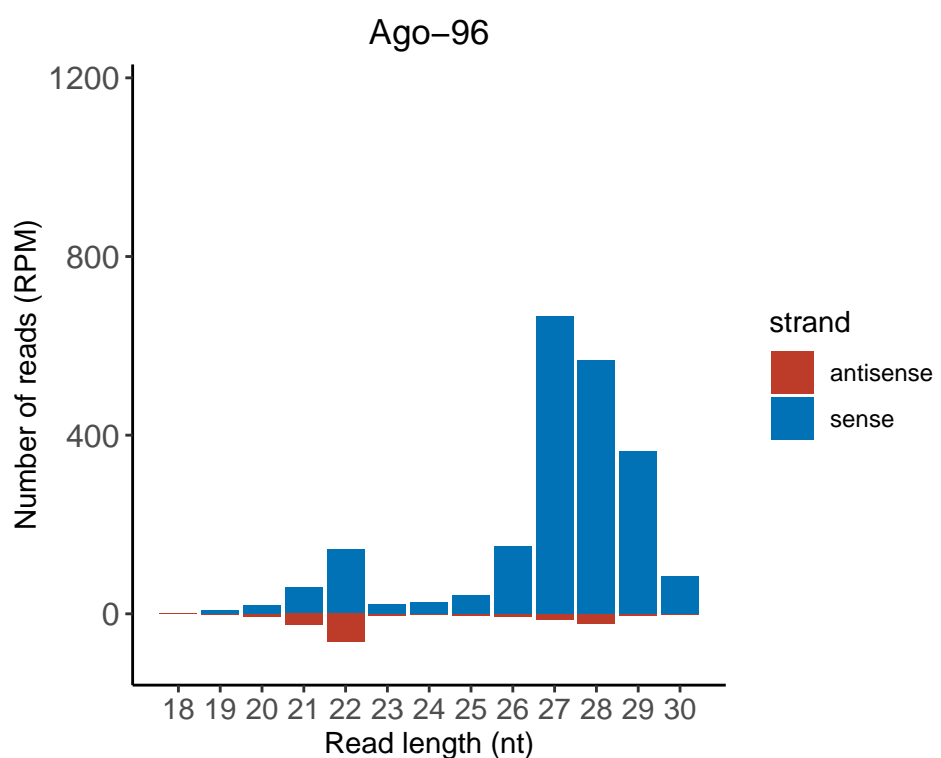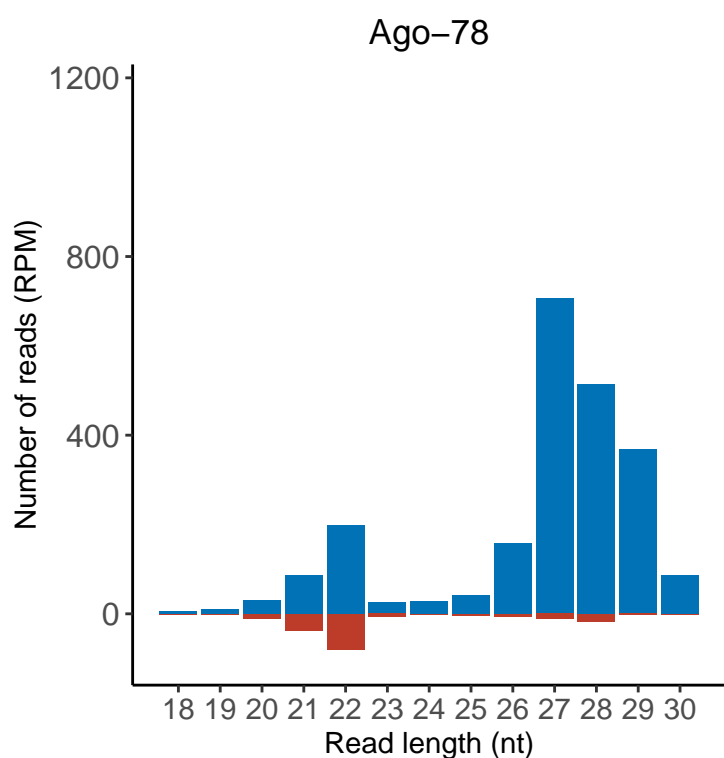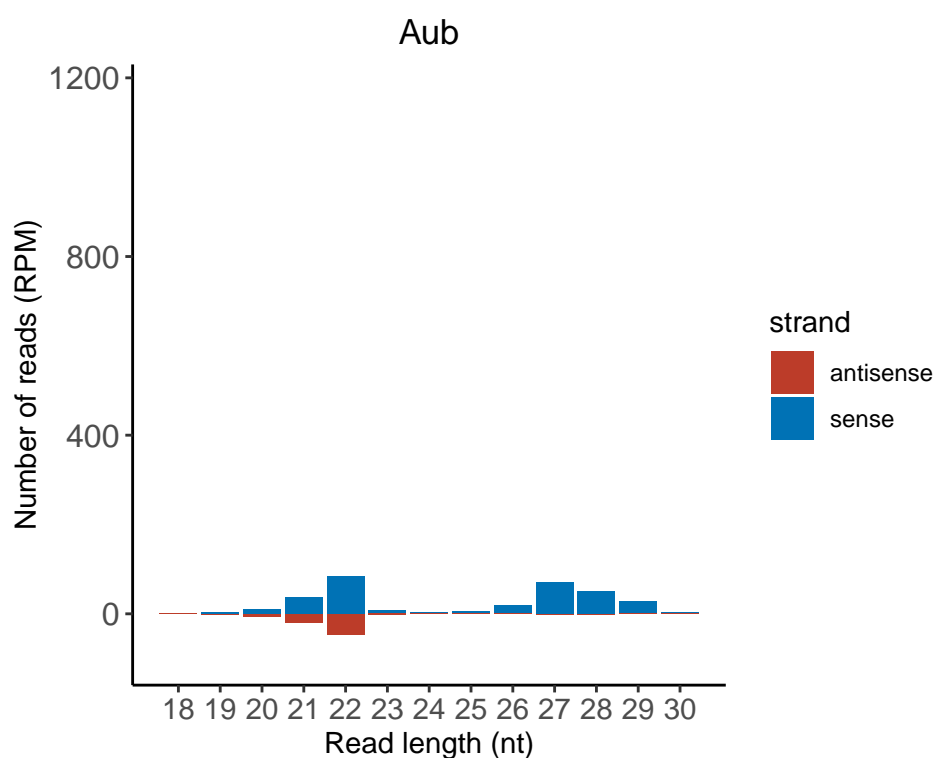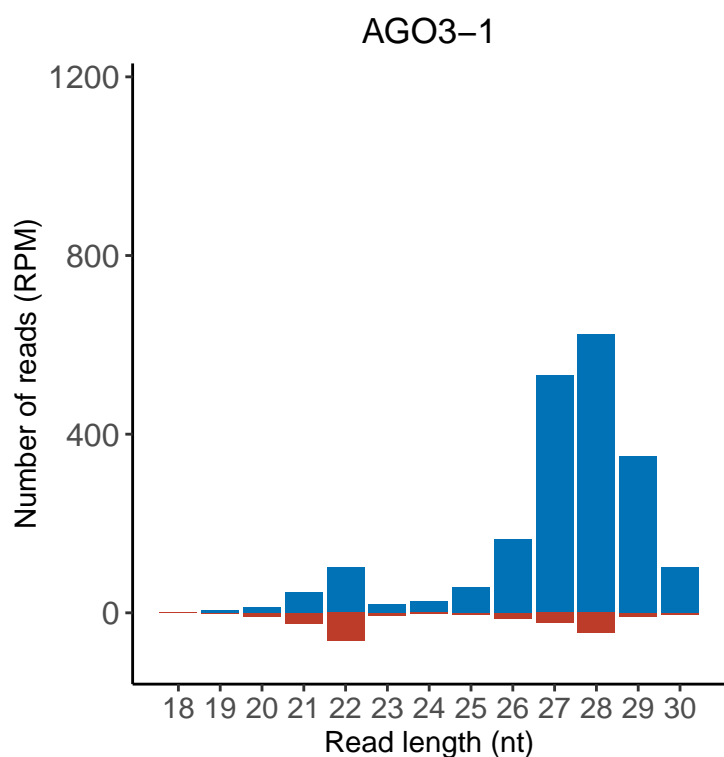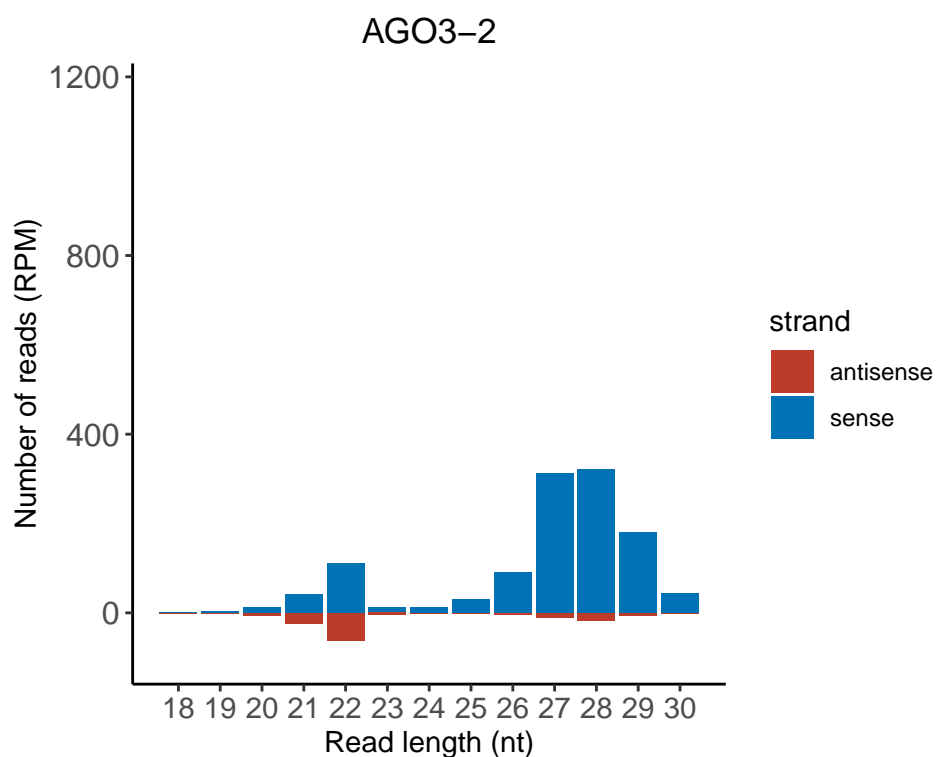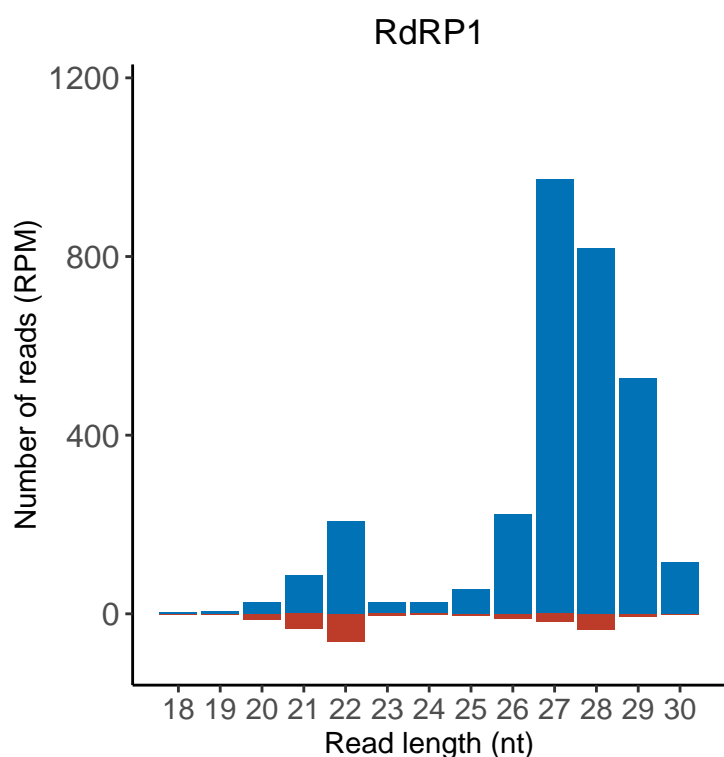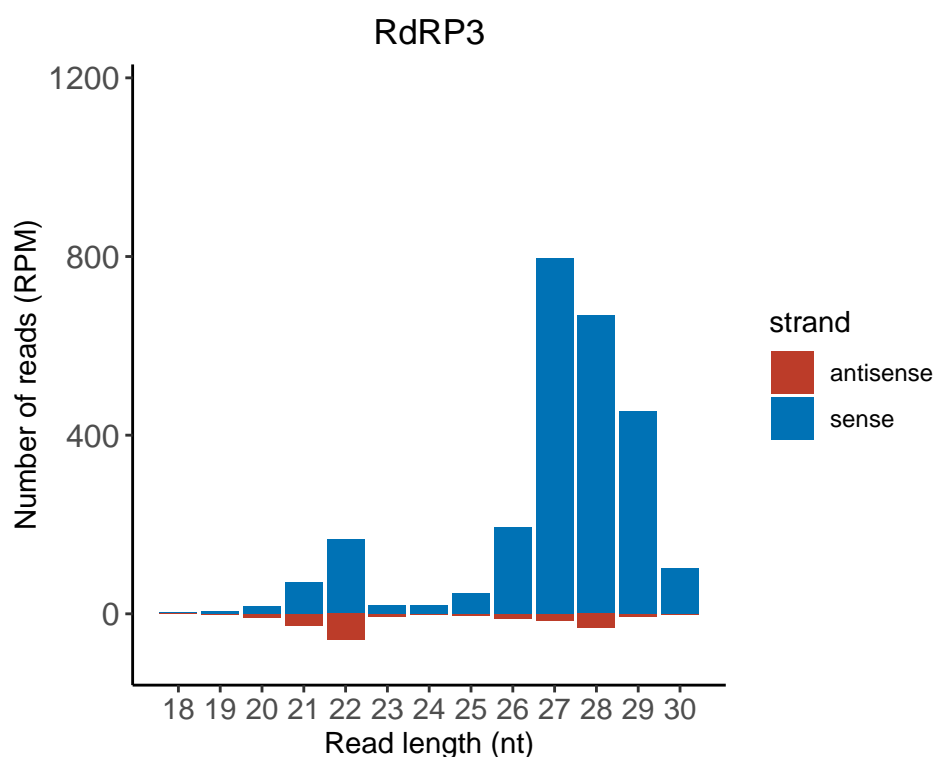

Supplement: S1 Data — On the Normalized and Relative levels pages, normalized read counts (RPM) and relative levels compared to the control (dsGFP) library were used. TEs with more than 50RPM and Coding Genes with more than 3.5RPM on average in KD libraries are included in this website. For viral sequences, reference sequences were generated by assembling sRNA sequences (See the Analysis of sRNAseq data section in Materials and methods.) and size distributions of sRNA reads mapped to each contig are shown. Reads were first grouped by their 5’ nucleotides, and reads of each length were counted. Full tables including TEs and Coding genes with fewer reads are in the “FullTable” folder. (ZIP) [file pone.0281195.s017.zip › SupplementaryData/Links/PDFs/Motif:rnd-6_family-46TE_lengthdistribution.pdf]

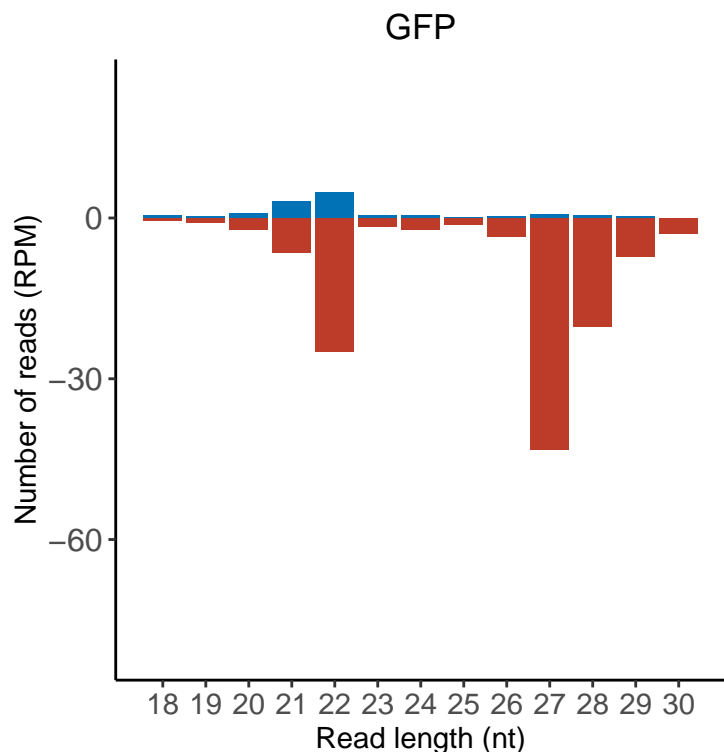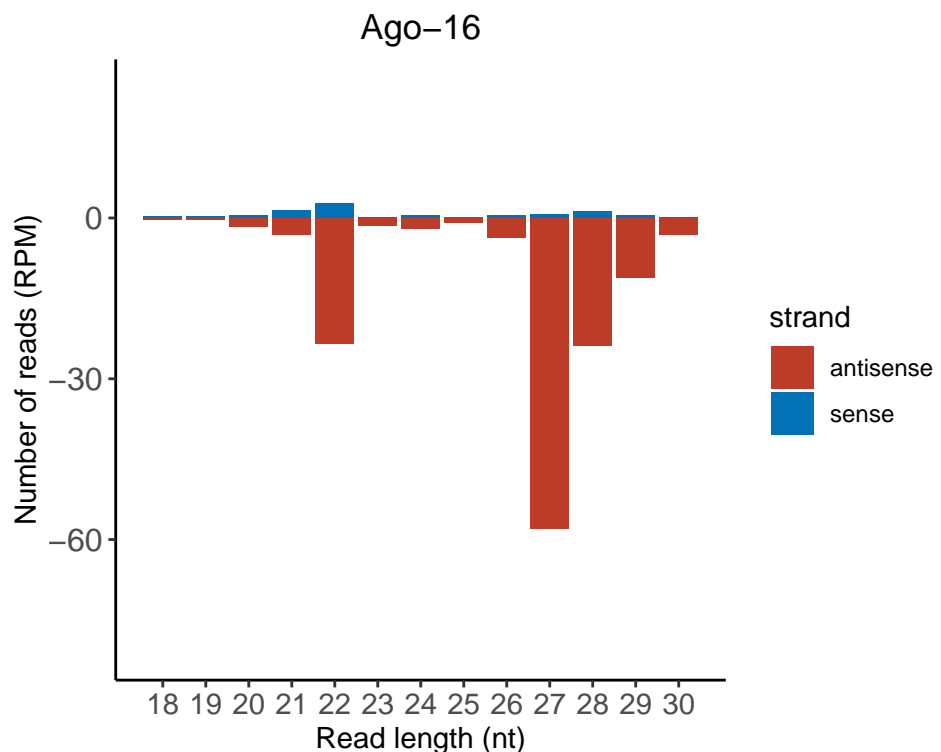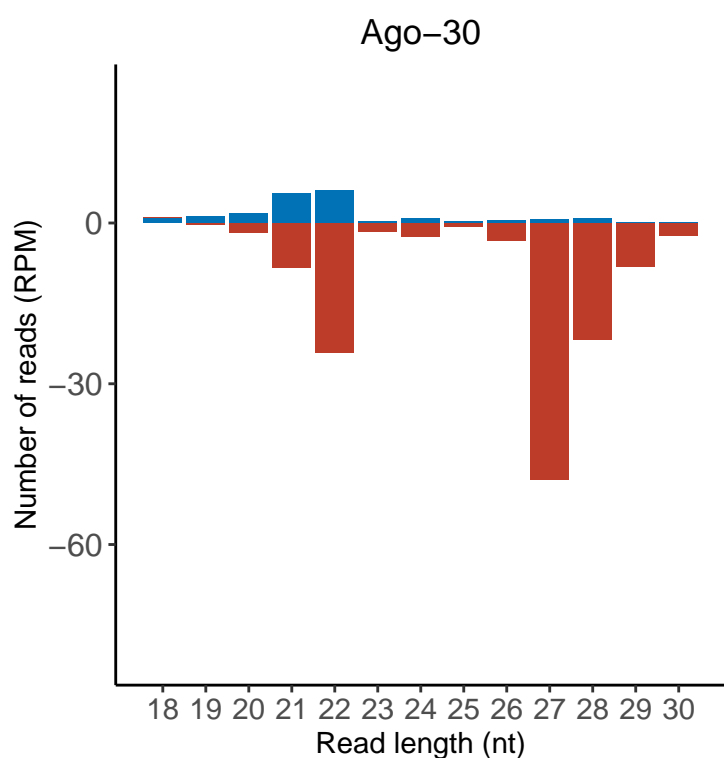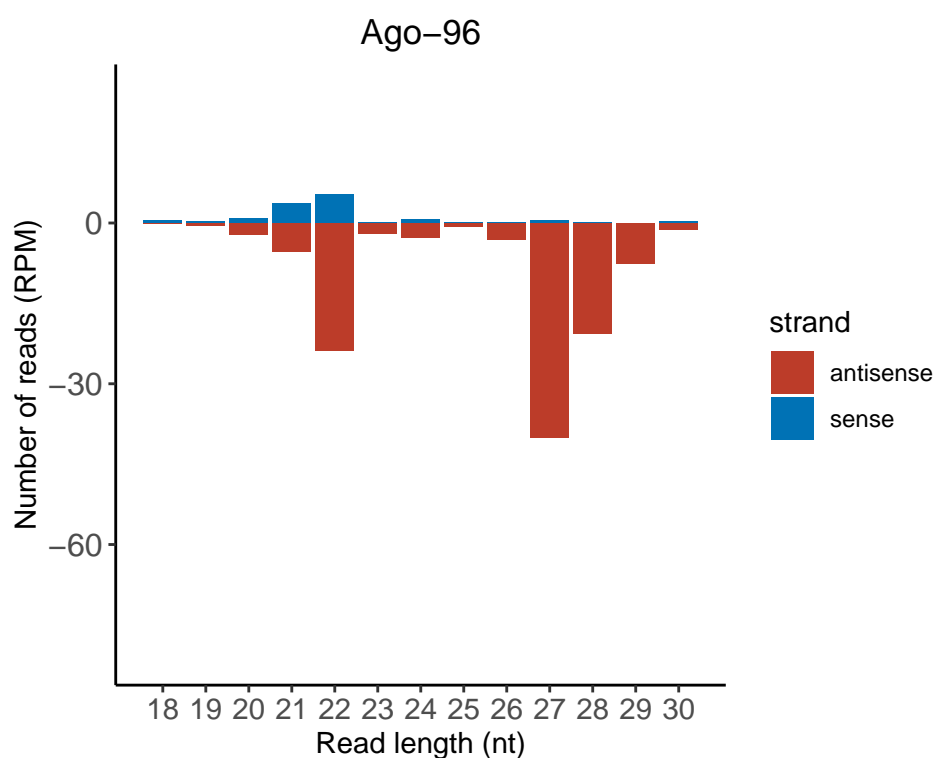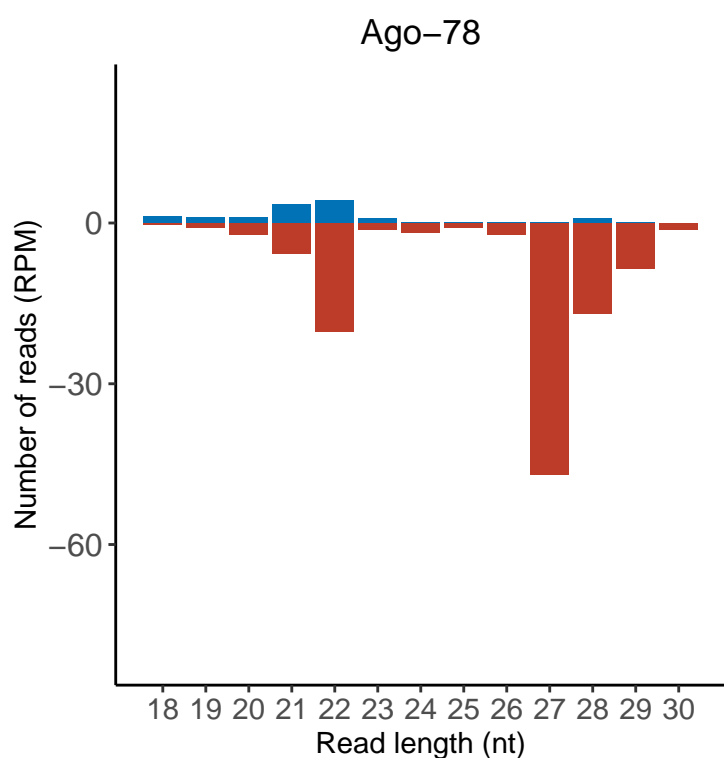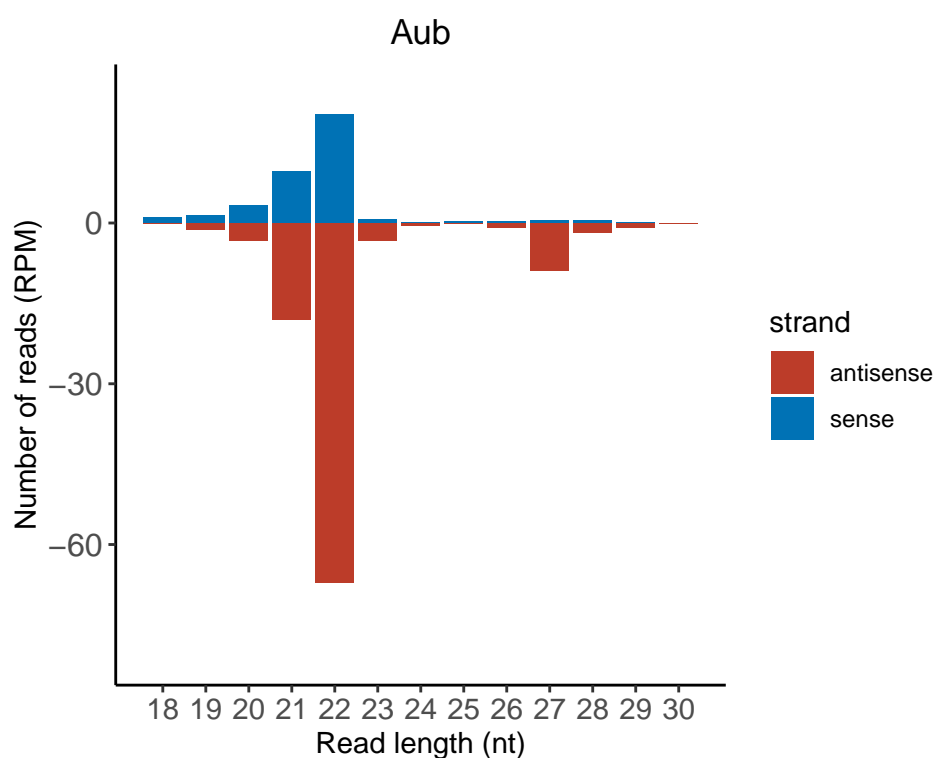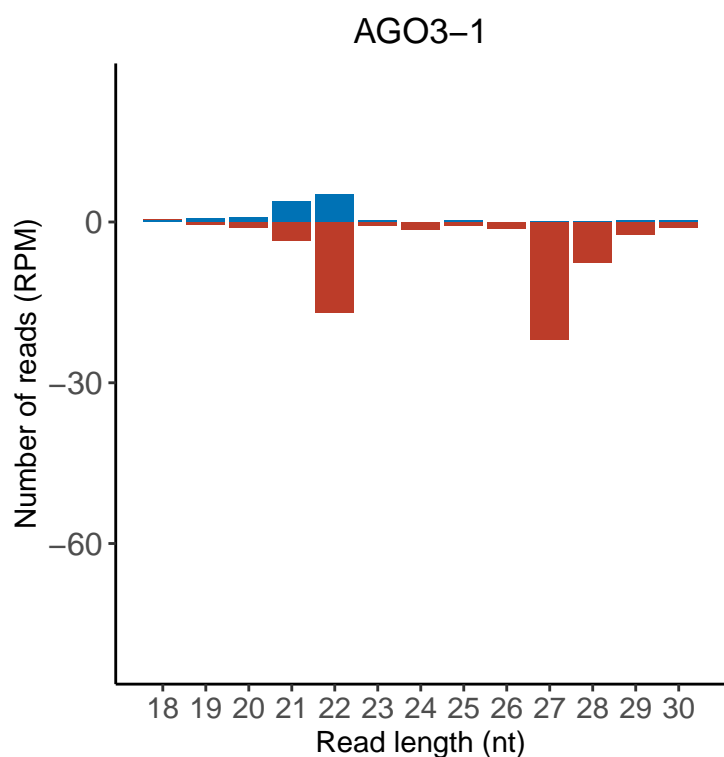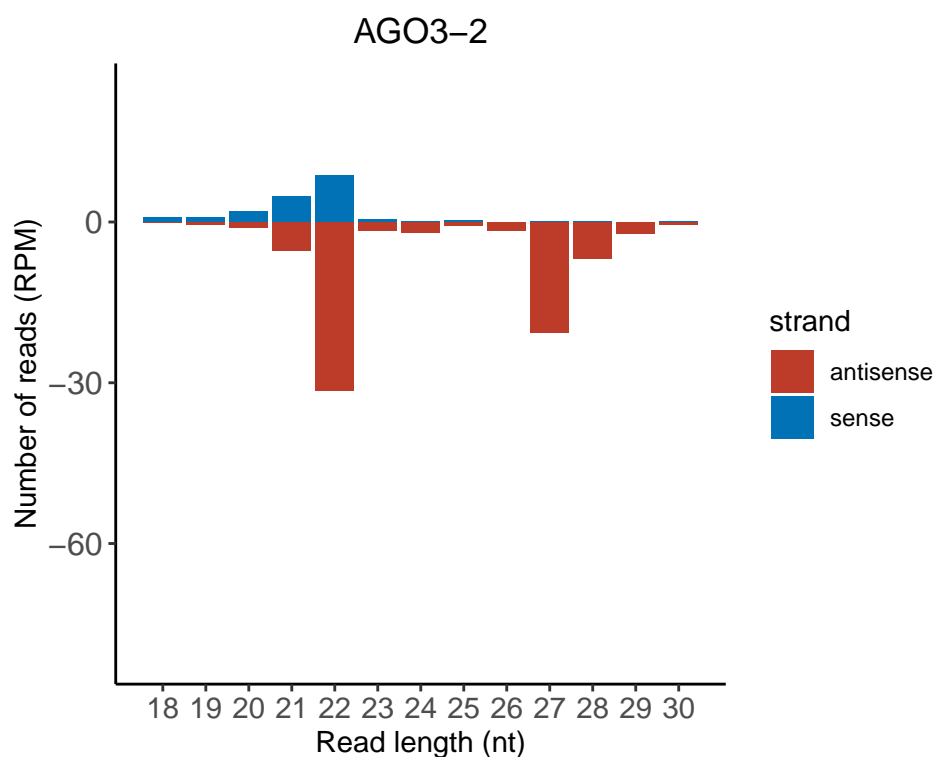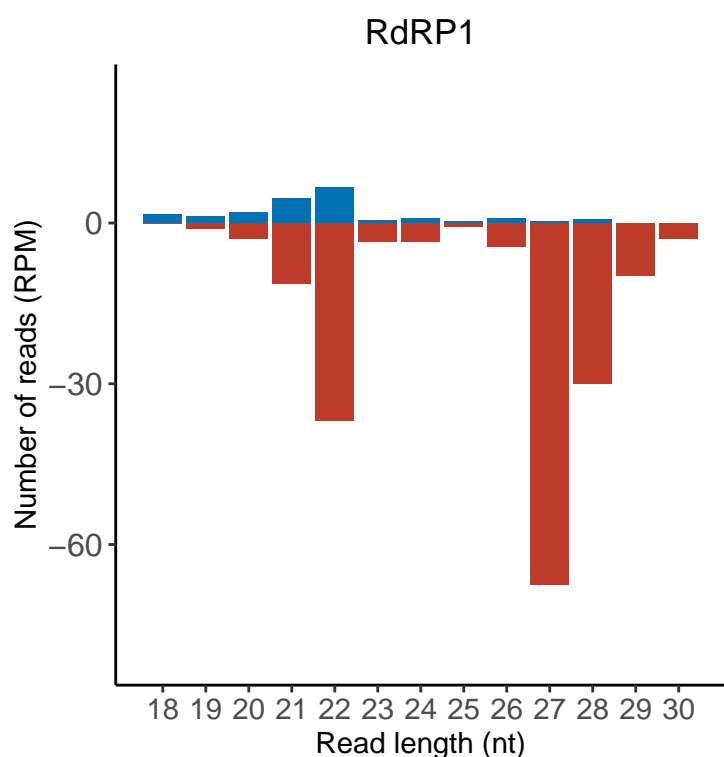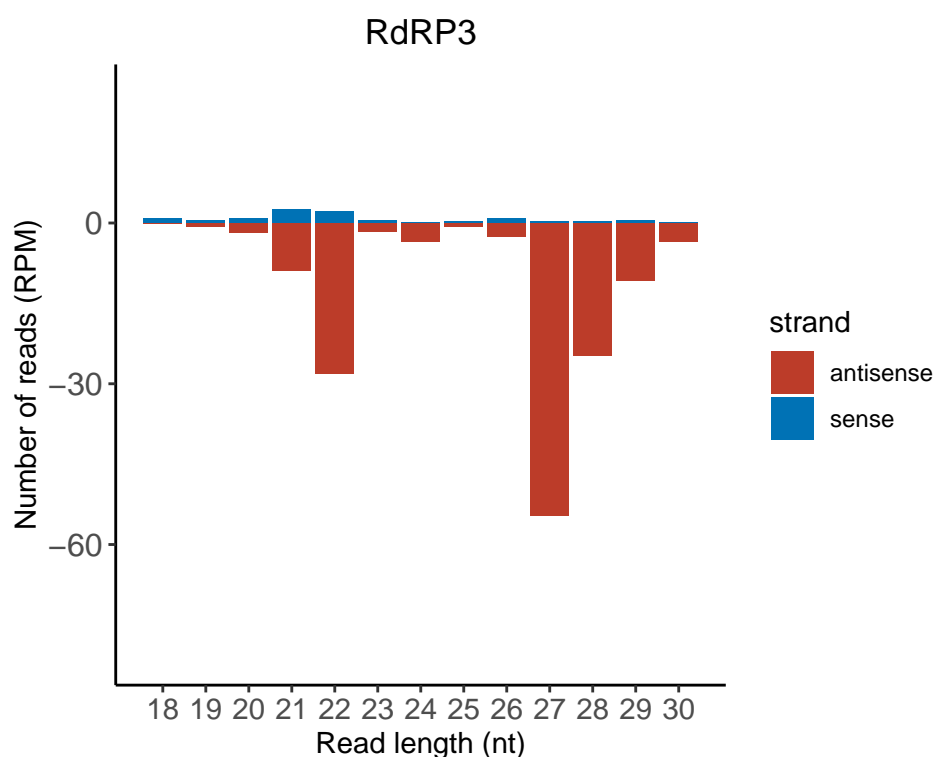

Supplement: S1 Data — On the Normalized and Relative levels pages, normalized read counts (RPM) and relative levels compared to the control (dsGFP) library were used. TEs with more than 50RPM and Coding Genes with more than 3.5RPM on average in KD libraries are included in this website. For viral sequences, reference sequences were generated by assembling sRNA sequences (See the Analysis of sRNAseq data section in Materials and methods.) and size distributions of sRNA reads mapped to each contig are shown. Reads were first grouped by their 5’ nucleotides, and reads of each length were counted. Full tables including TEs and Coding genes with fewer reads are in the “FullTable” folder. (ZIP) [file pone.0281195.s017.zip › SupplementaryData/Links/PDFs/ISCI024649.RACDS_lengthdistribution.pdf]

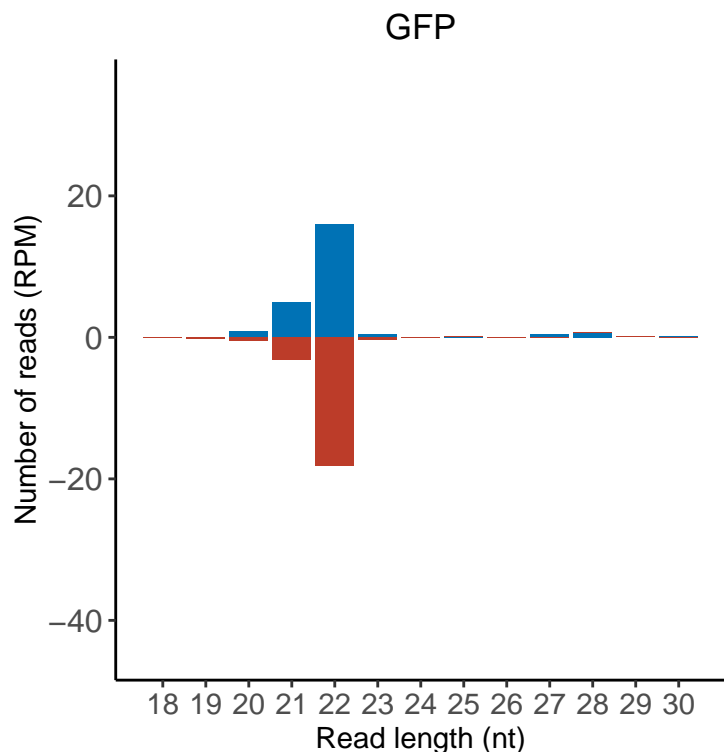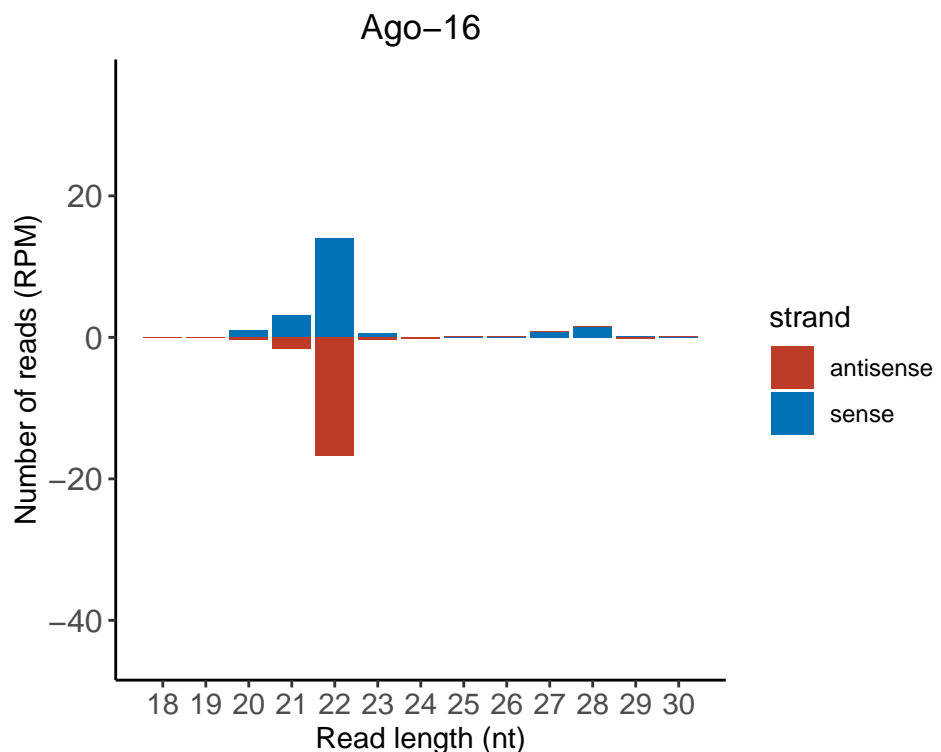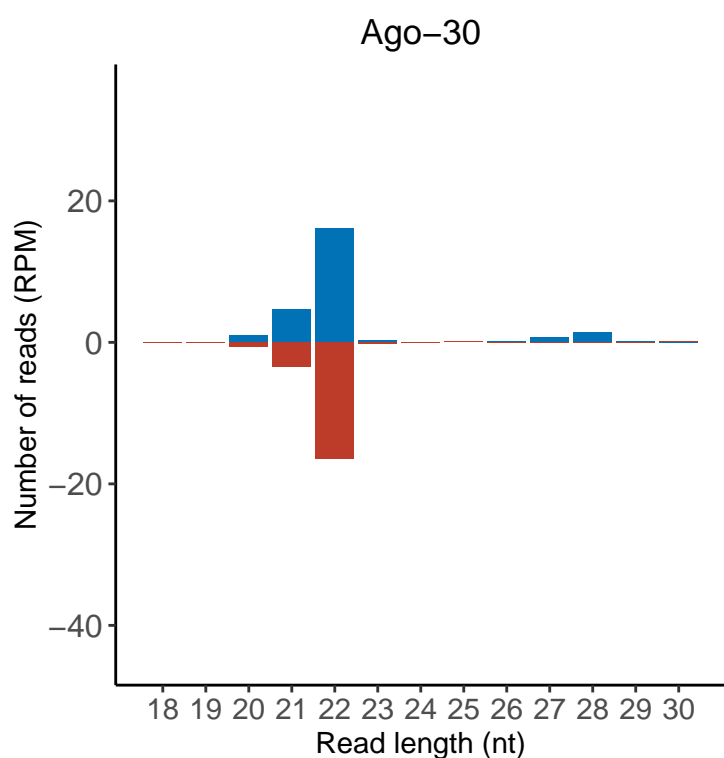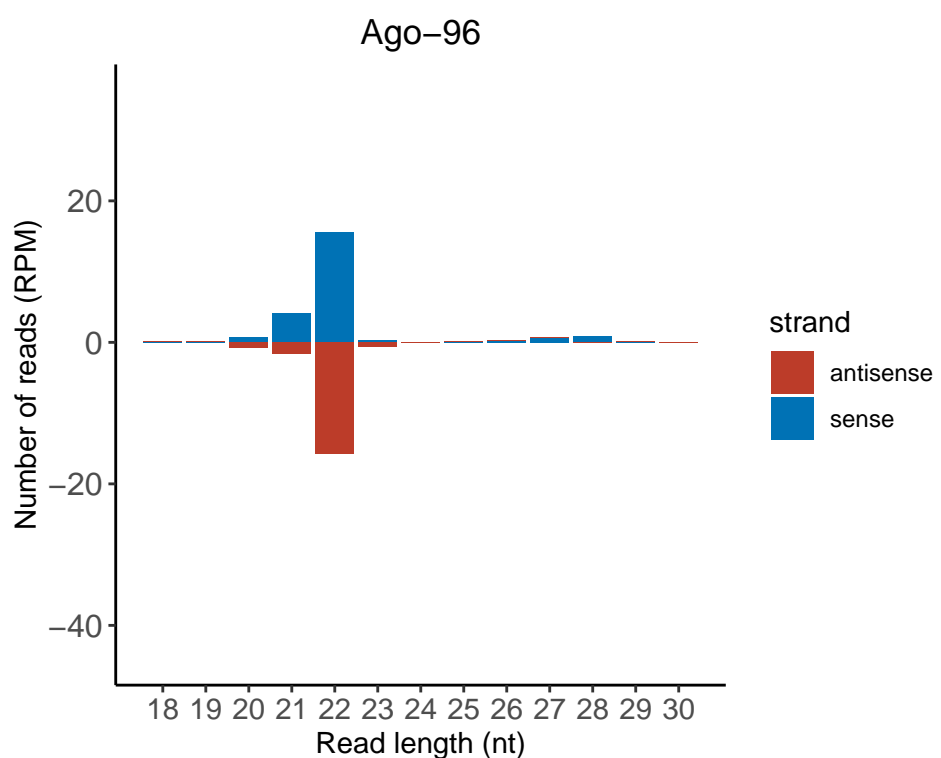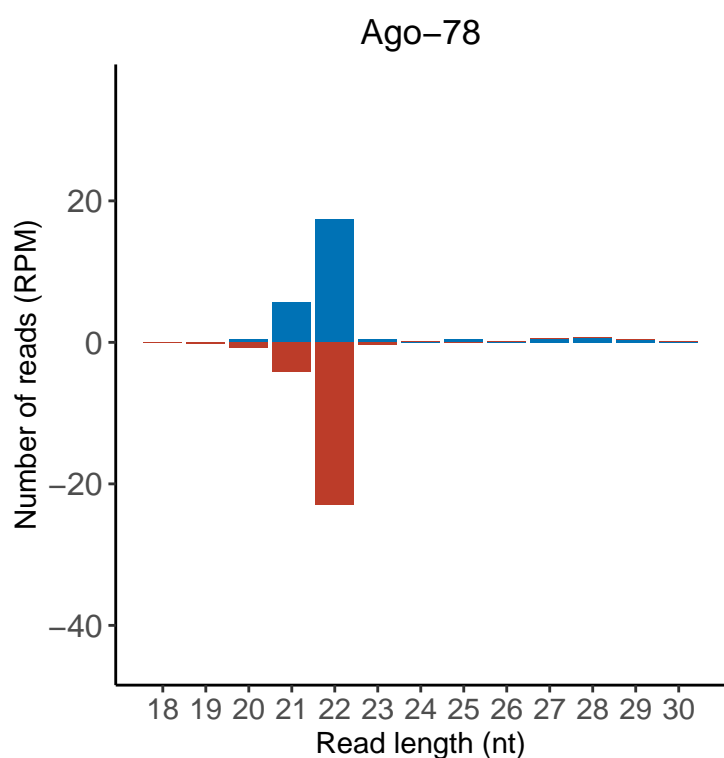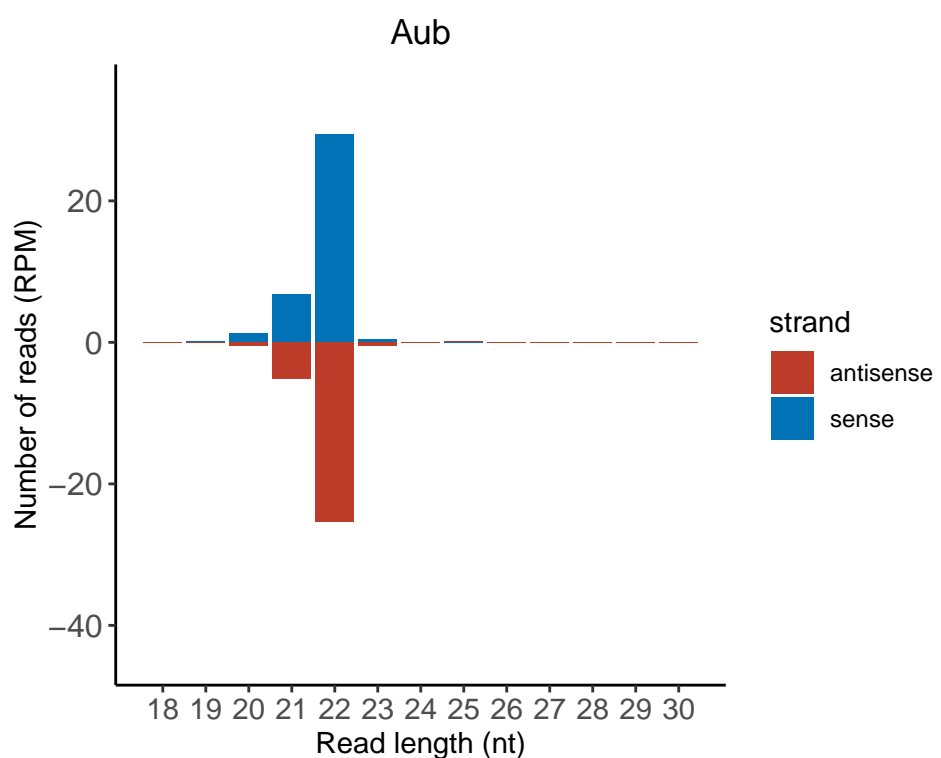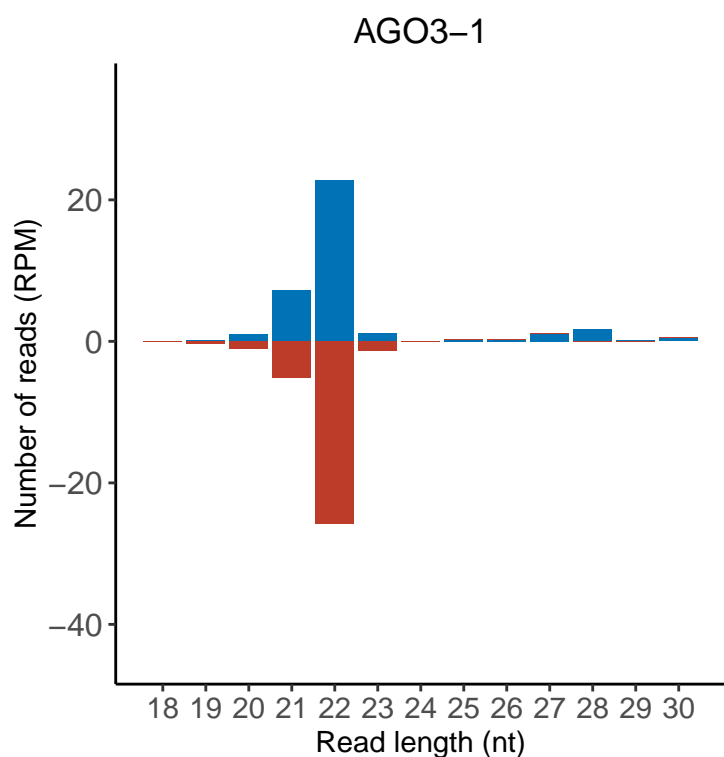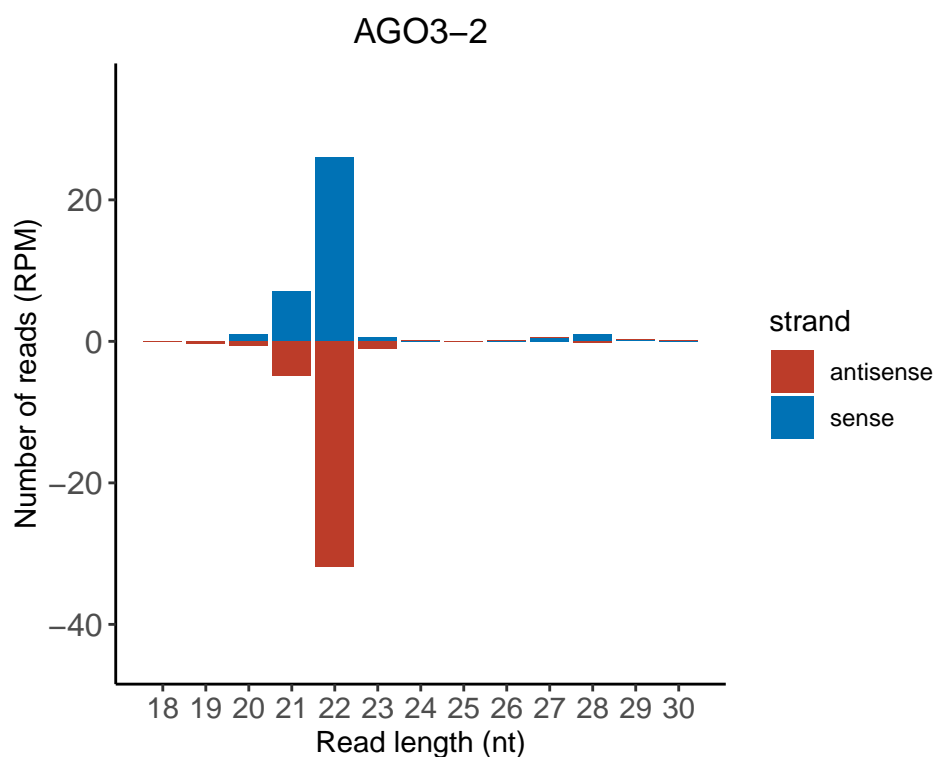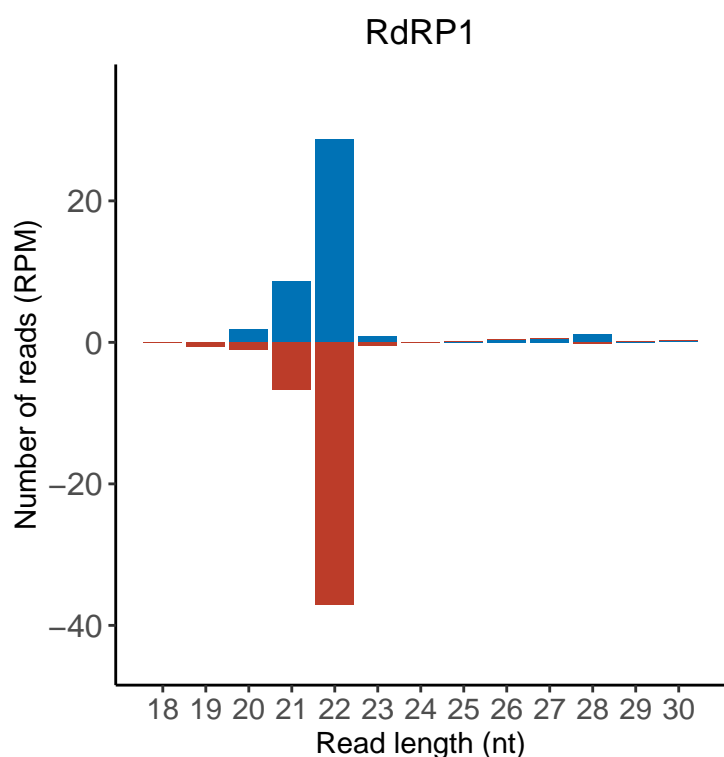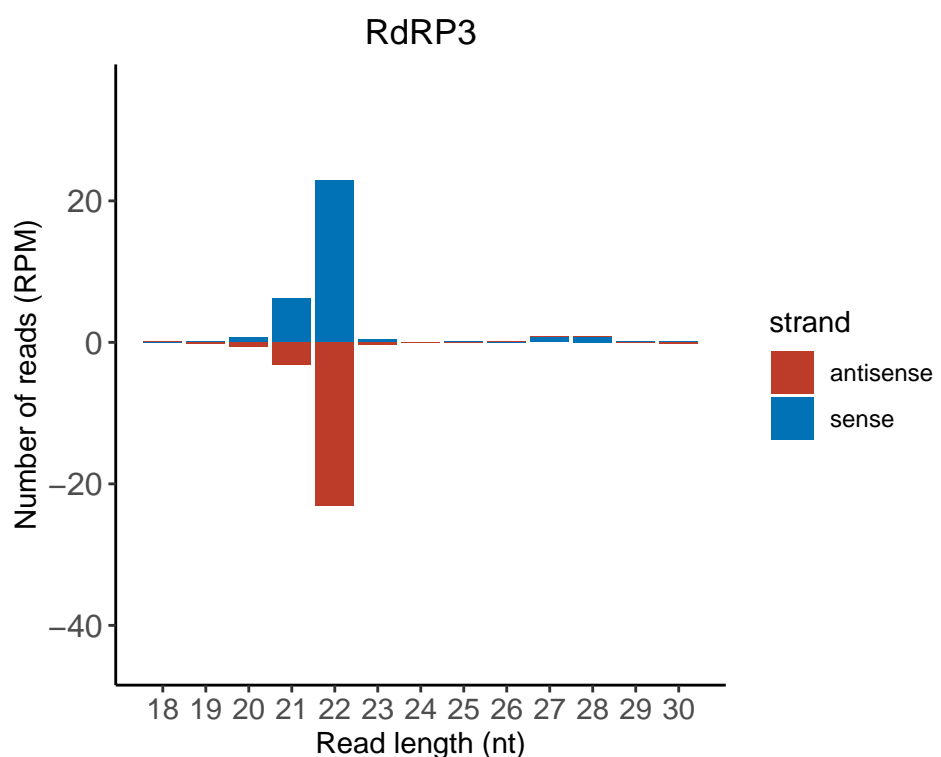

Supplement: S1 Data — On the Normalized and Relative levels pages, normalized read counts (RPM) and relative levels compared to the control (dsGFP) library were used. TEs with more than 50RPM and Coding Genes with more than 3.5RPM on average in KD libraries are included in this website. For viral sequences, reference sequences were generated by assembling sRNA sequences (See the Analysis of sRNAseq data section in Materials and methods.) and size distributions of sRNA reads mapped to each contig are shown. Reads were first grouped by their 5’ nucleotides, and reads of each length were counted. Full tables including TEs and Coding genes with fewer reads are in the “FullTable” folder. (ZIP) [file pone.0281195.s017.zip › SupplementaryData/Links/PDFs/Motif:rnd-6_family-71TE_lengthdistribution.pdf]

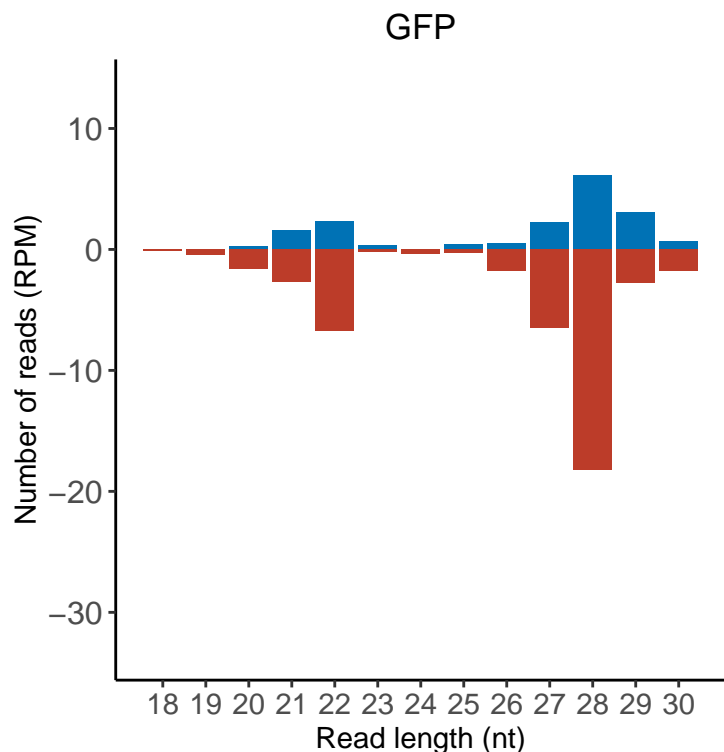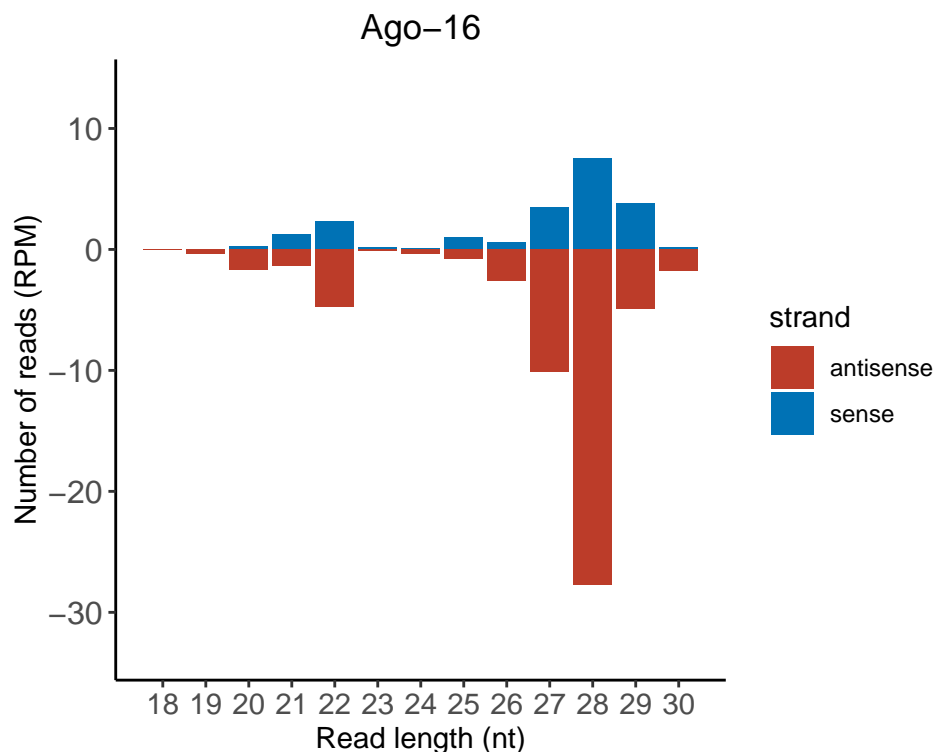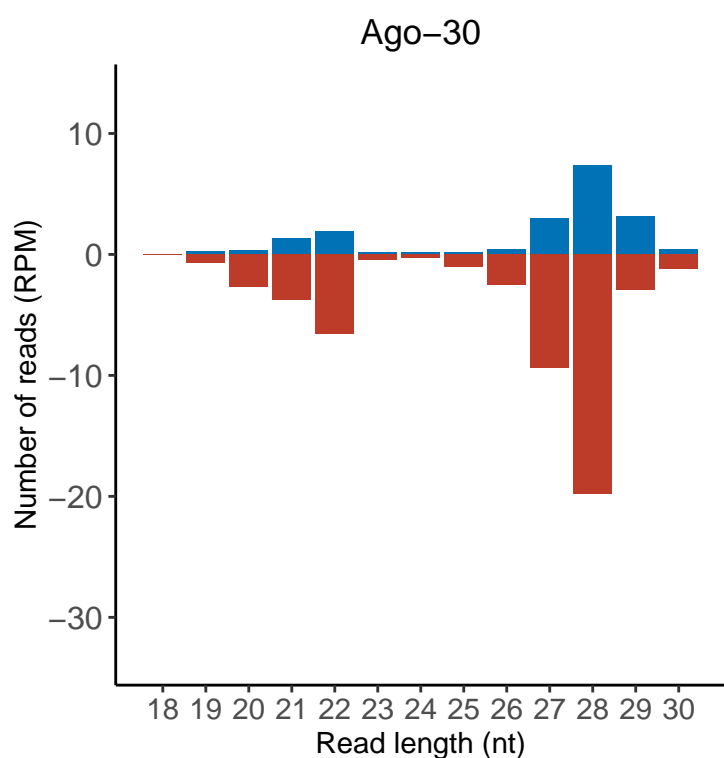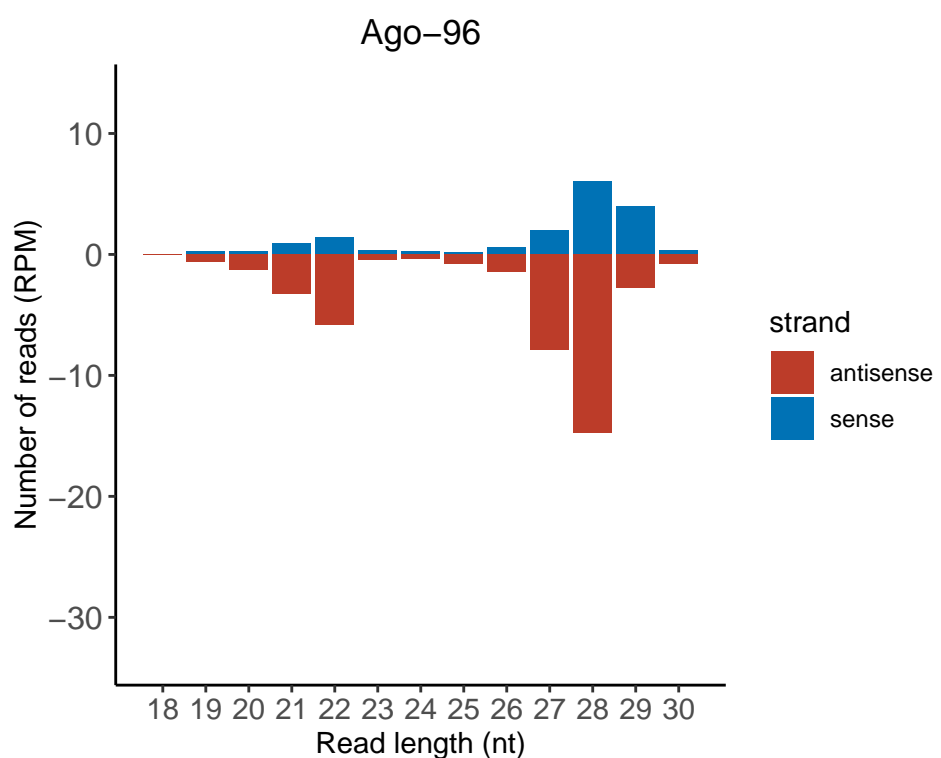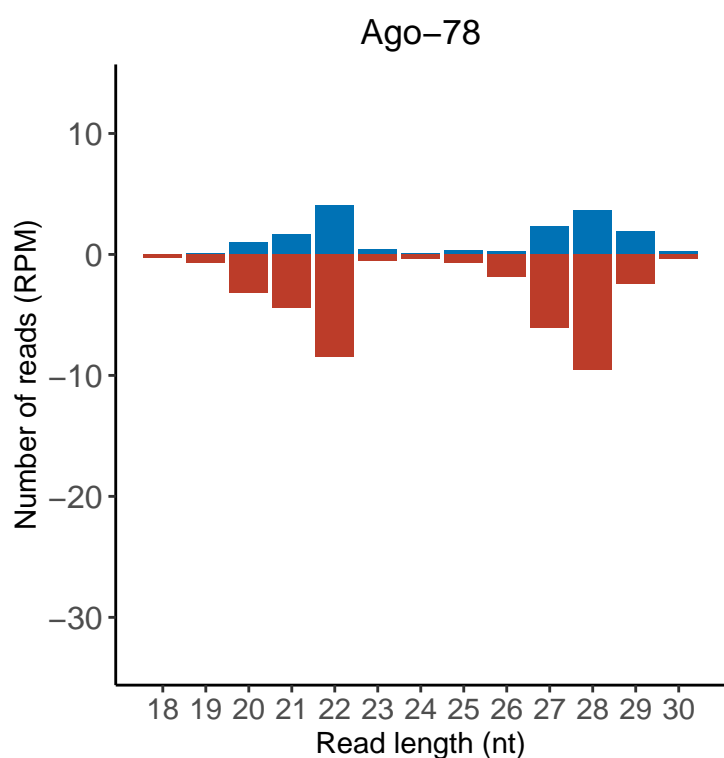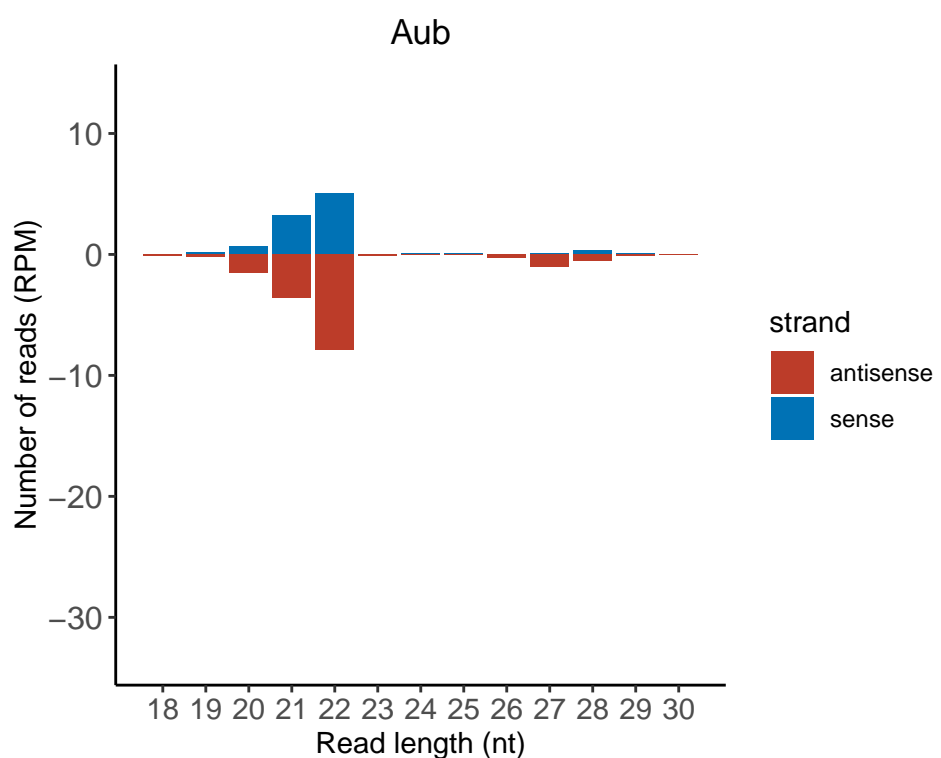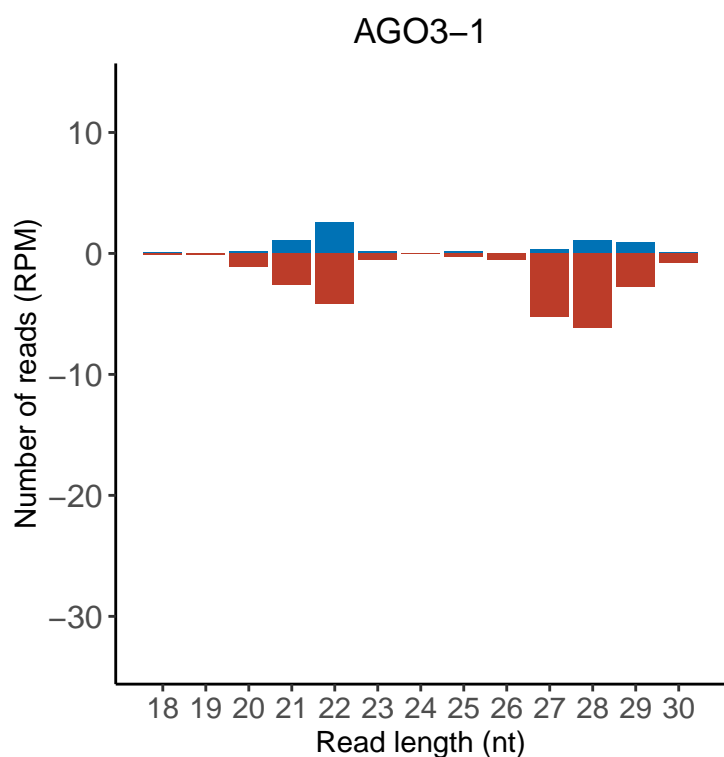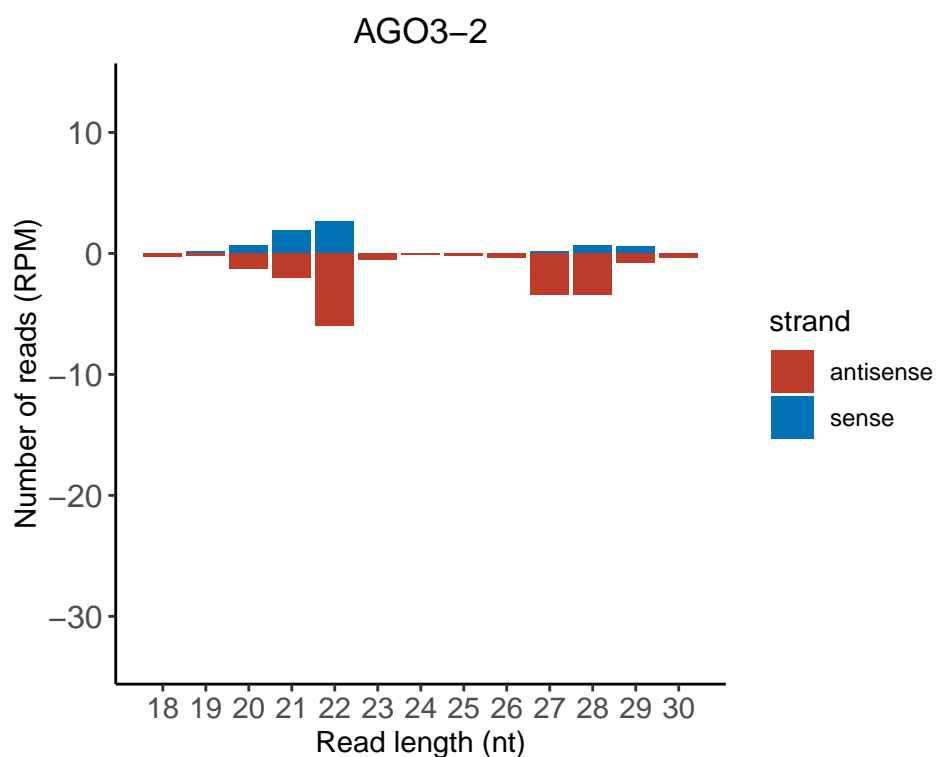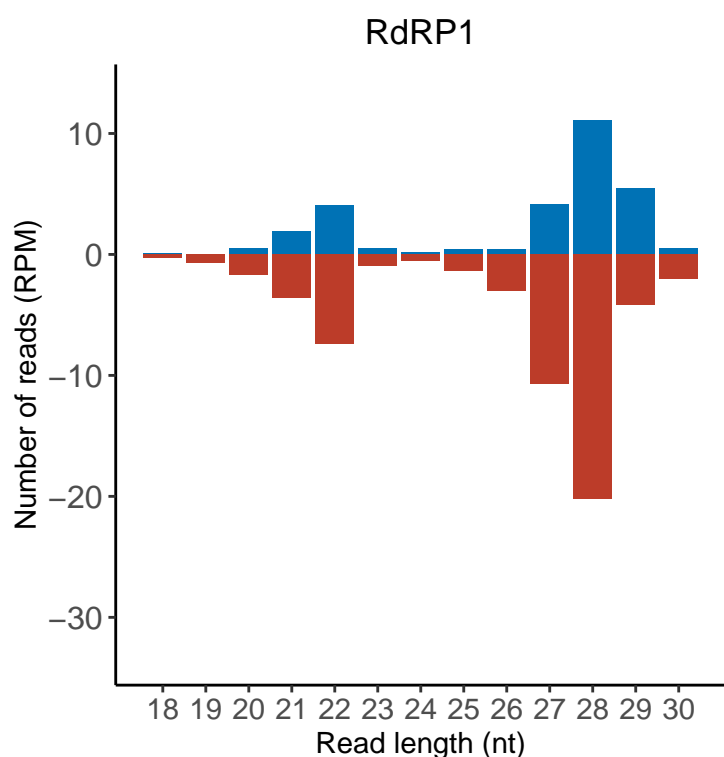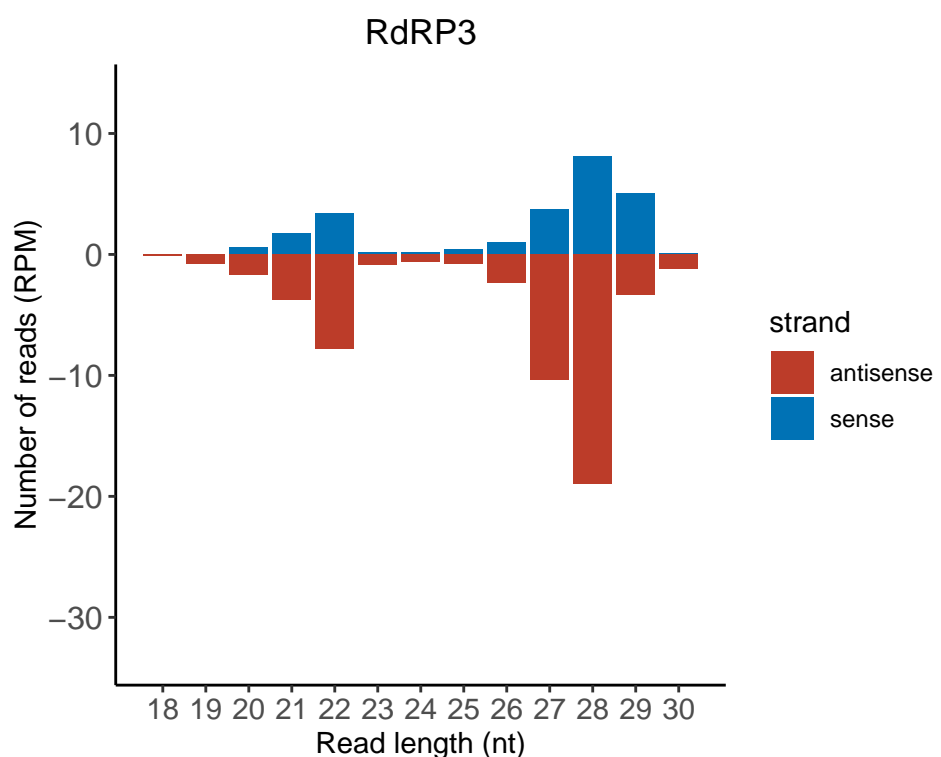

Supplement: S1 Data — On the Normalized and Relative levels pages, normalized read counts (RPM) and relative levels compared to the control (dsGFP) library were used. TEs with more than 50RPM and Coding Genes with more than 3.5RPM on average in KD libraries are included in this website. For viral sequences, reference sequences were generated by assembling sRNA sequences (See the Analysis of sRNAseq data section in Materials and methods.) and size distributions of sRNA reads mapped to each contig are shown. Reads were first grouped by their 5’ nucleotides, and reads of each length were counted. Full tables including TEs and Coding genes with fewer reads are in the “FullTable” folder. (ZIP) [file pone.0281195.s017.zip › SupplementaryData/Links/PDFs/Motif:rnd-1_family-1373TE_lengthdistribution.pdf]

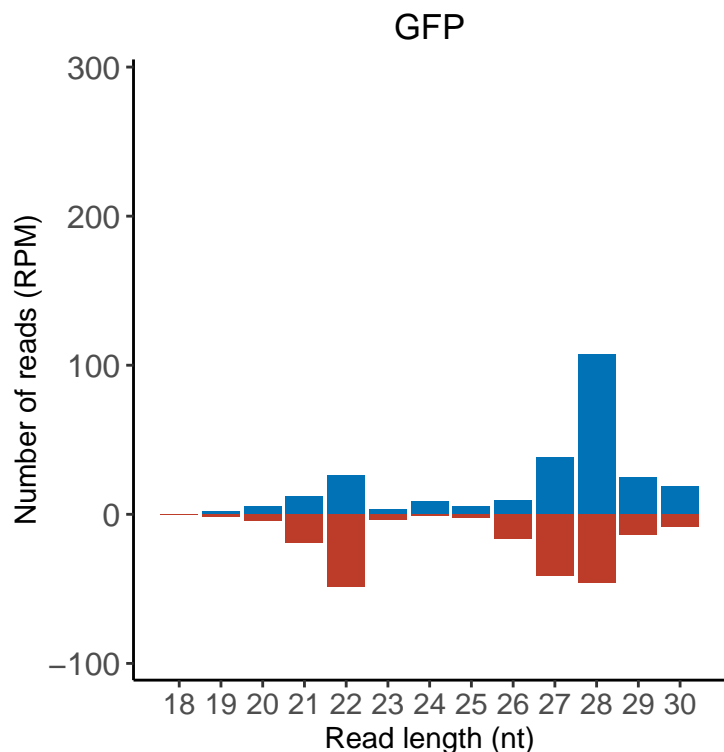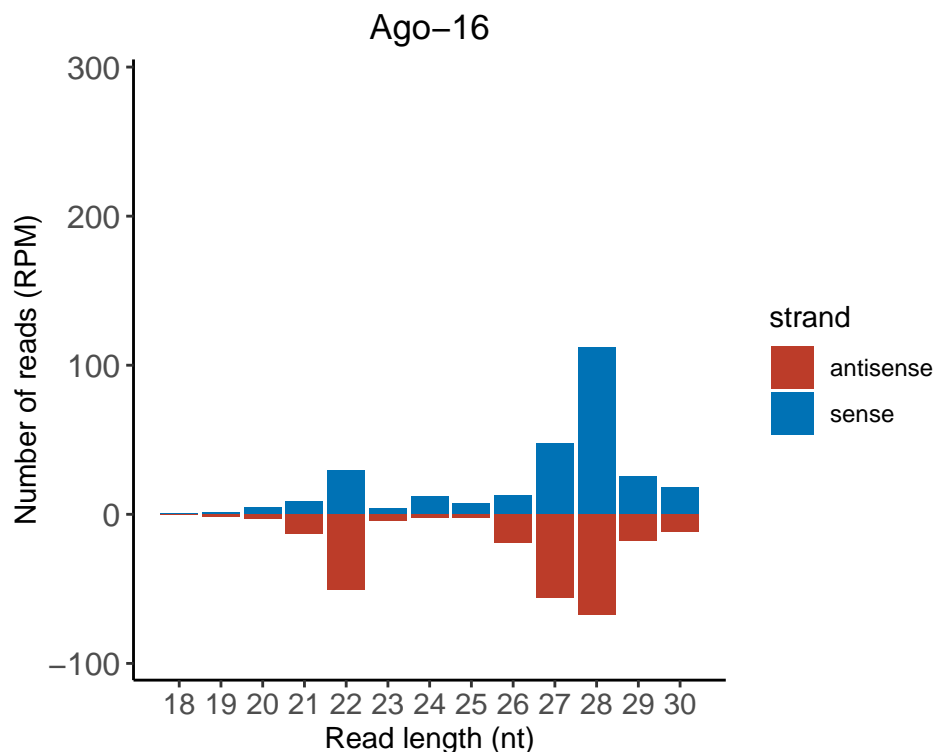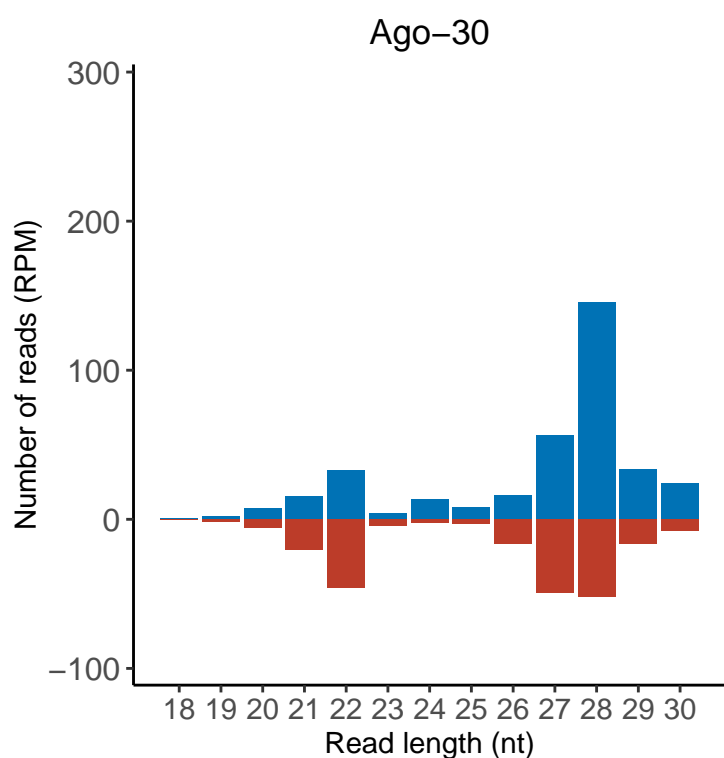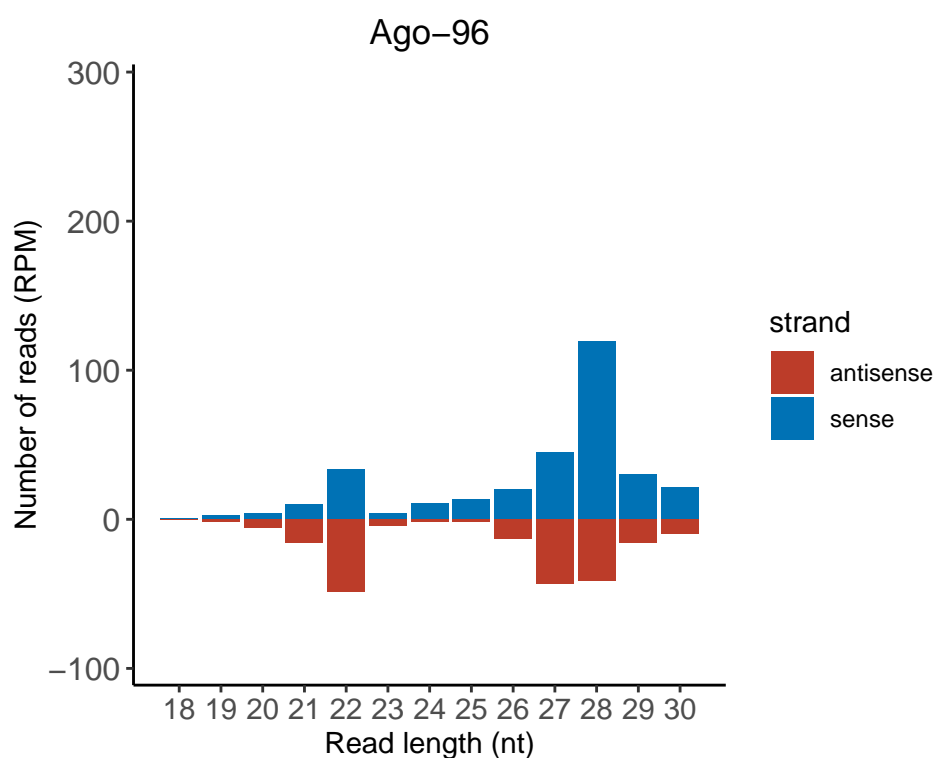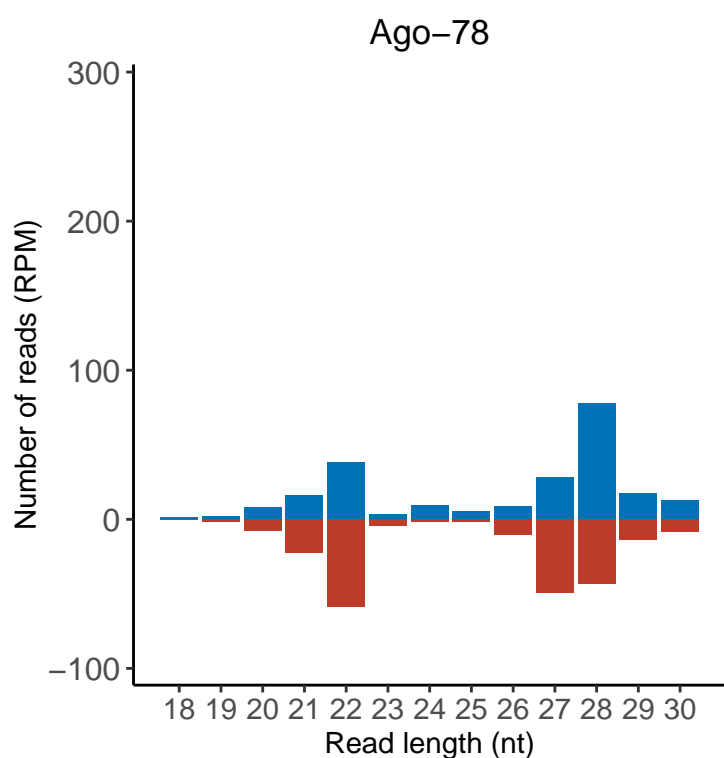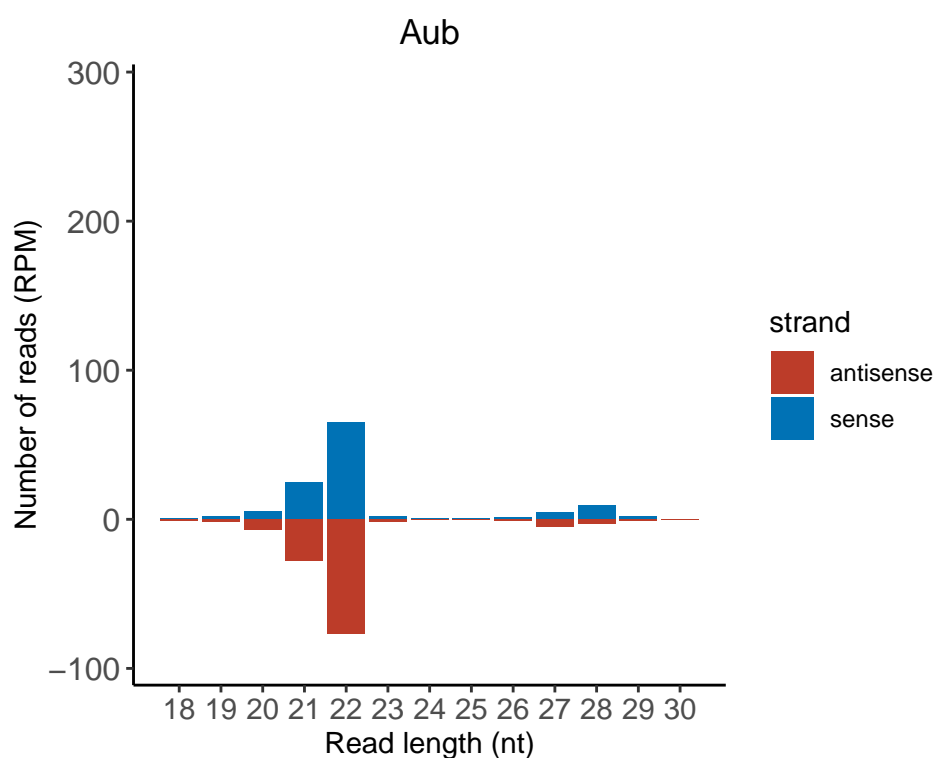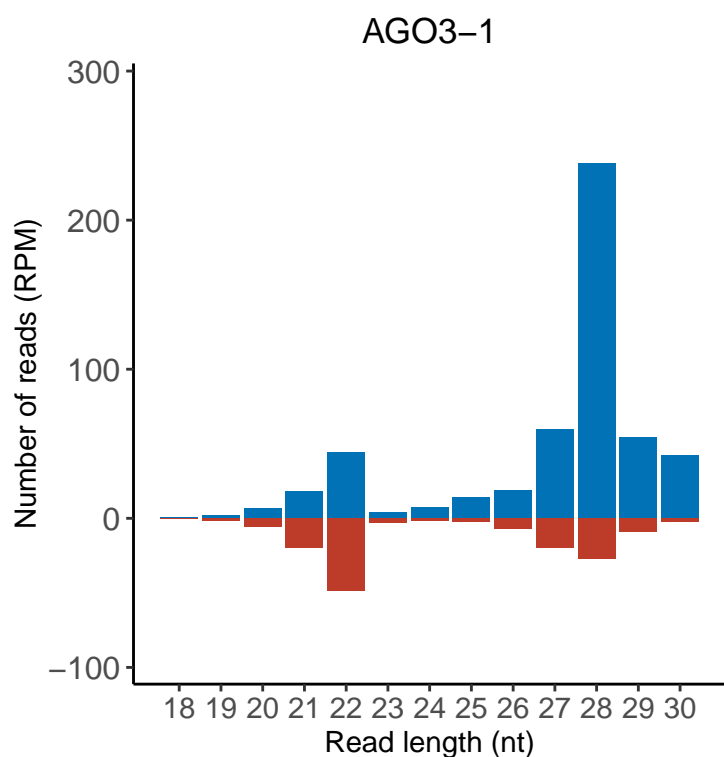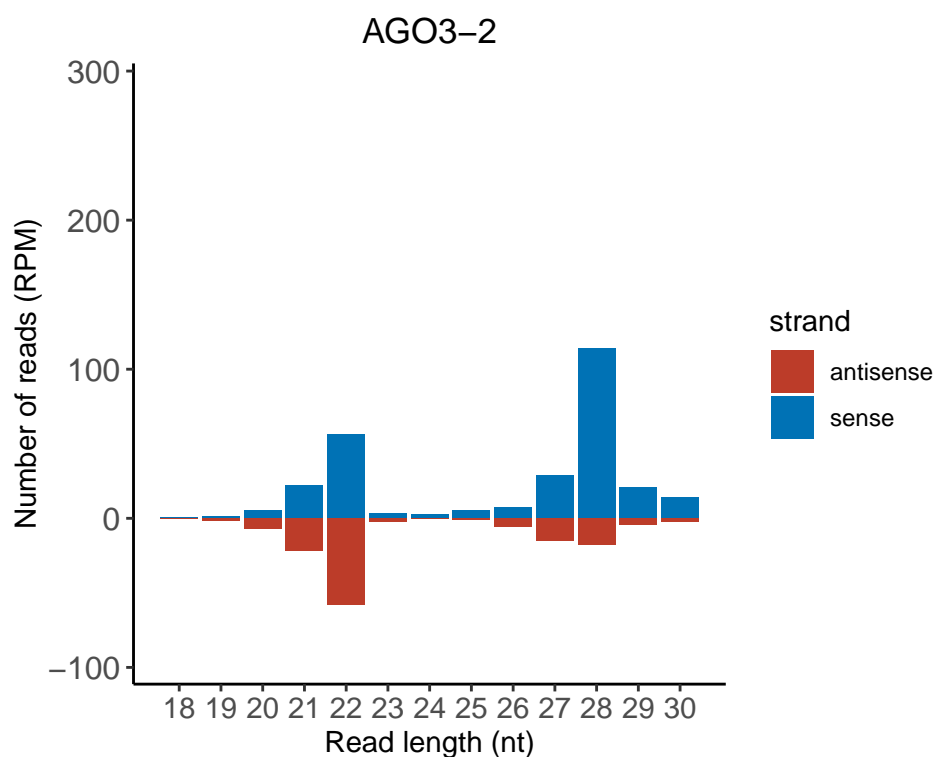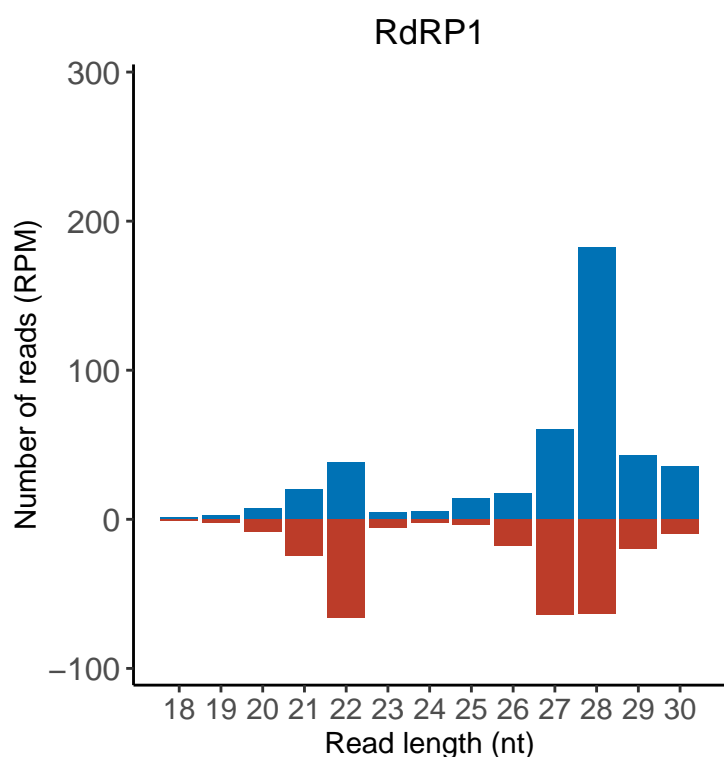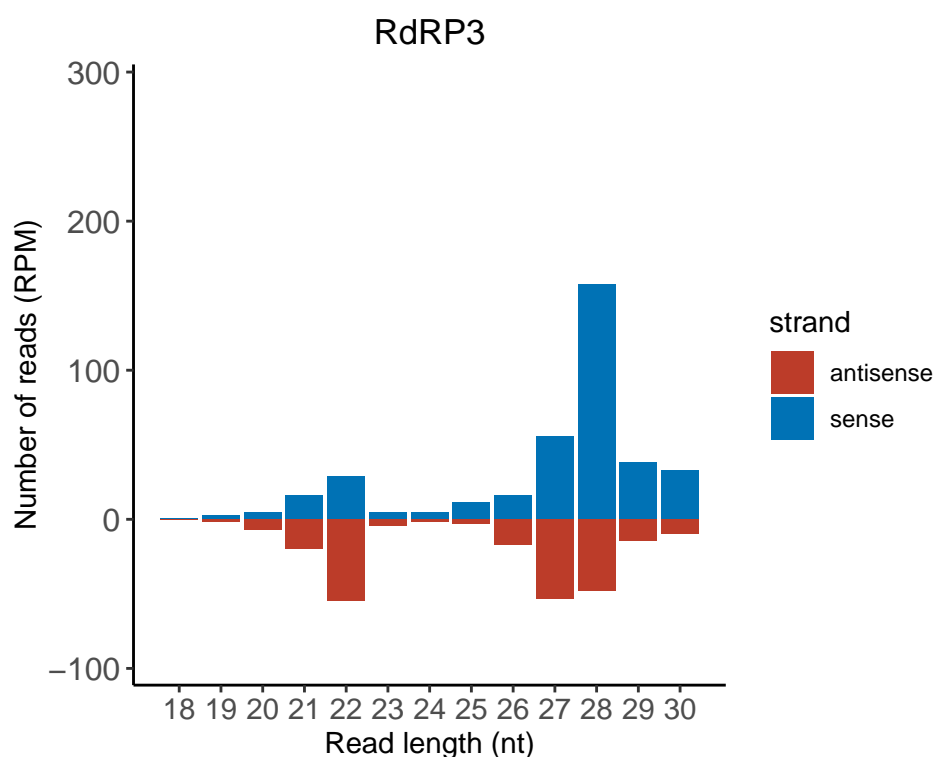

Supplement: S1 Data — On the Normalized and Relative levels pages, normalized read counts (RPM) and relative levels compared to the control (dsGFP) library were used. TEs with more than 50RPM and Coding Genes with more than 3.5RPM on average in KD libraries are included in this website. For viral sequences, reference sequences were generated by assembling sRNA sequences (See the Analysis of sRNAseq data section in Materials and methods.) and size distributions of sRNA reads mapped to each contig are shown. Reads were first grouped by their 5’ nucleotides, and reads of each length were counted. Full tables including TEs and Coding genes with fewer reads are in the “FullTable” folder. (ZIP) [file pone.0281195.s017.zip › SupplementaryData/Links/PDFs/Motif:rnd-1_family-1531TE_lengthdistribution.pdf]

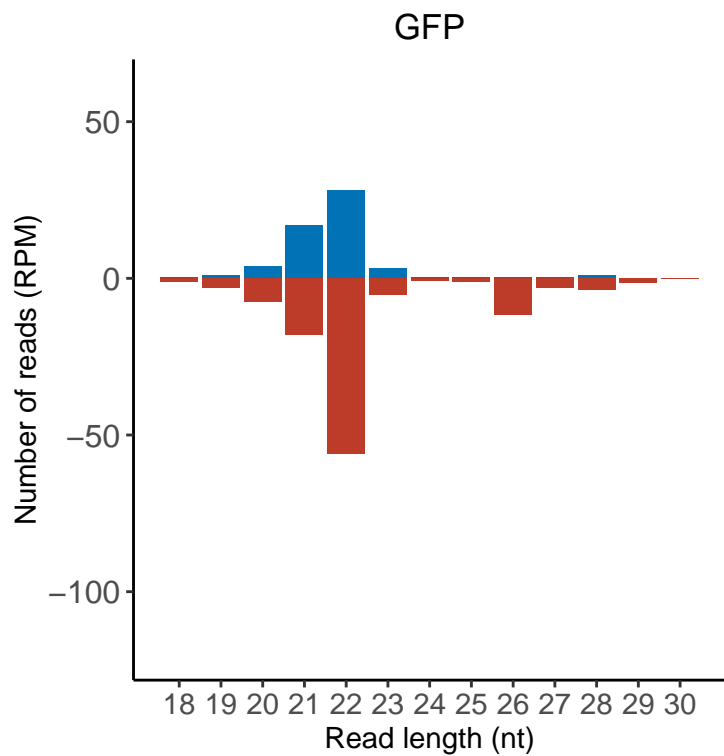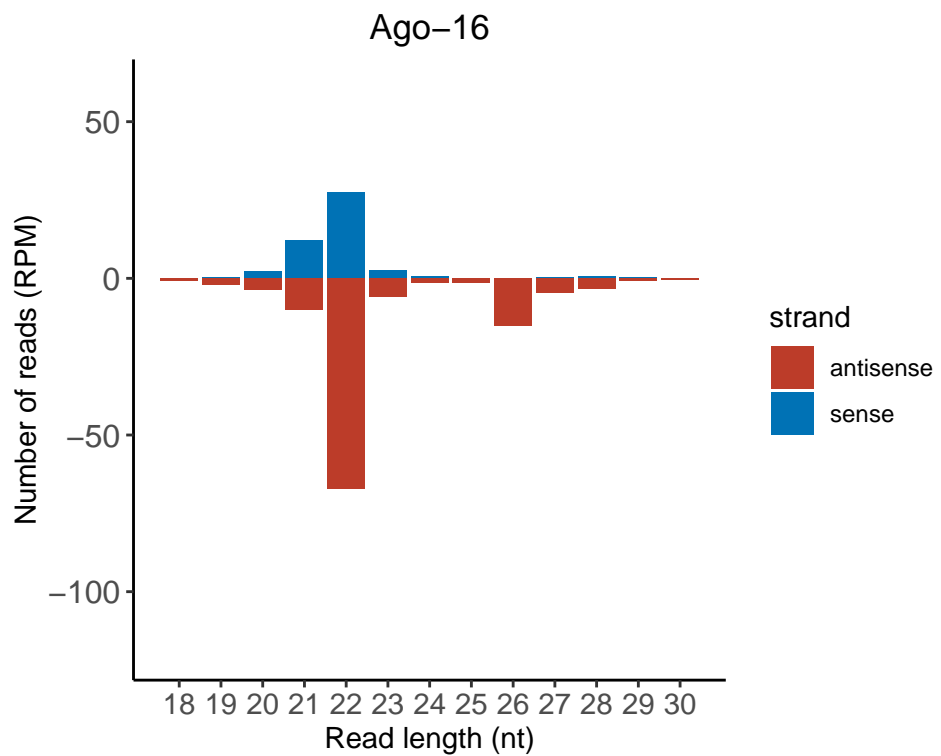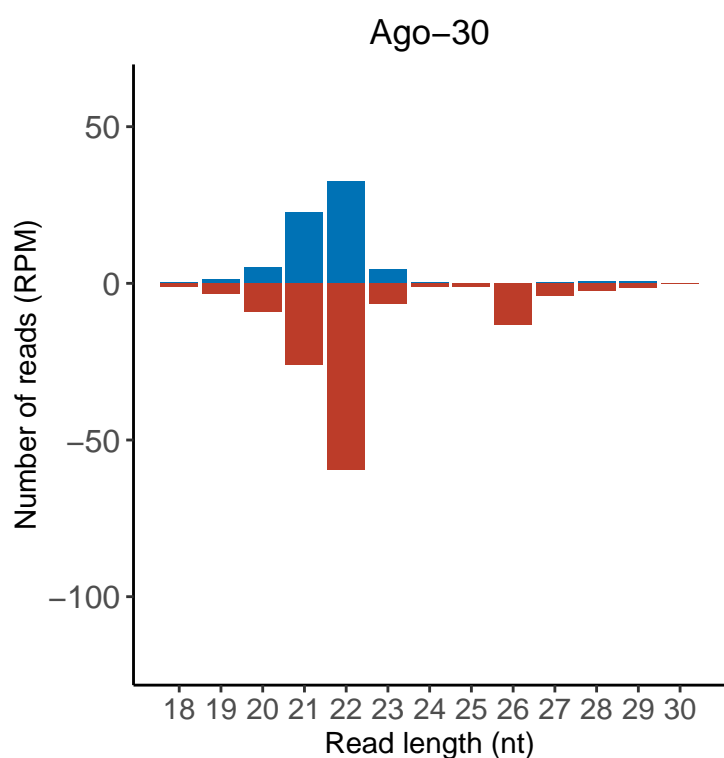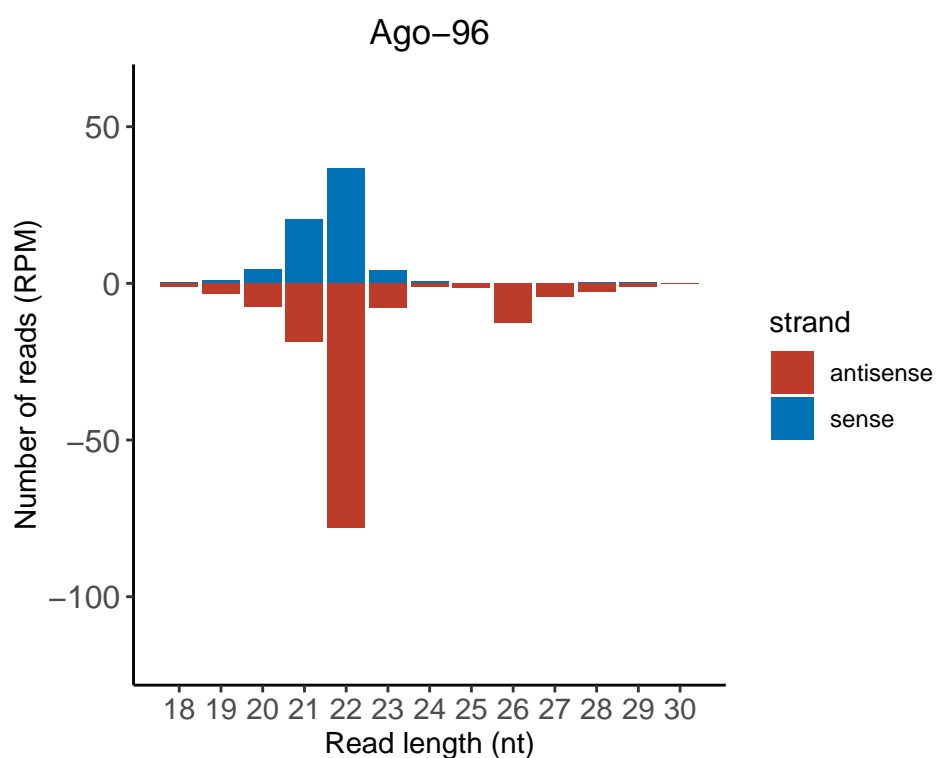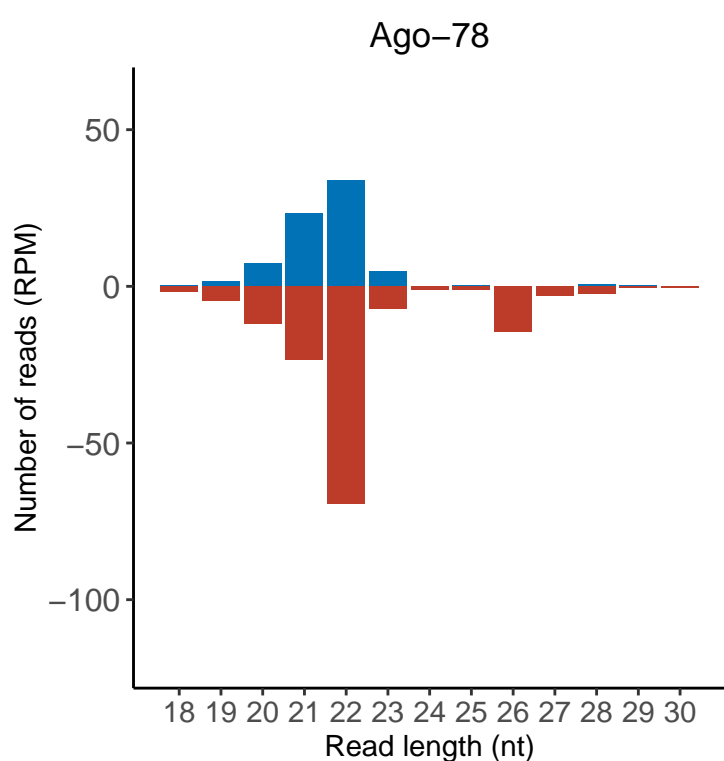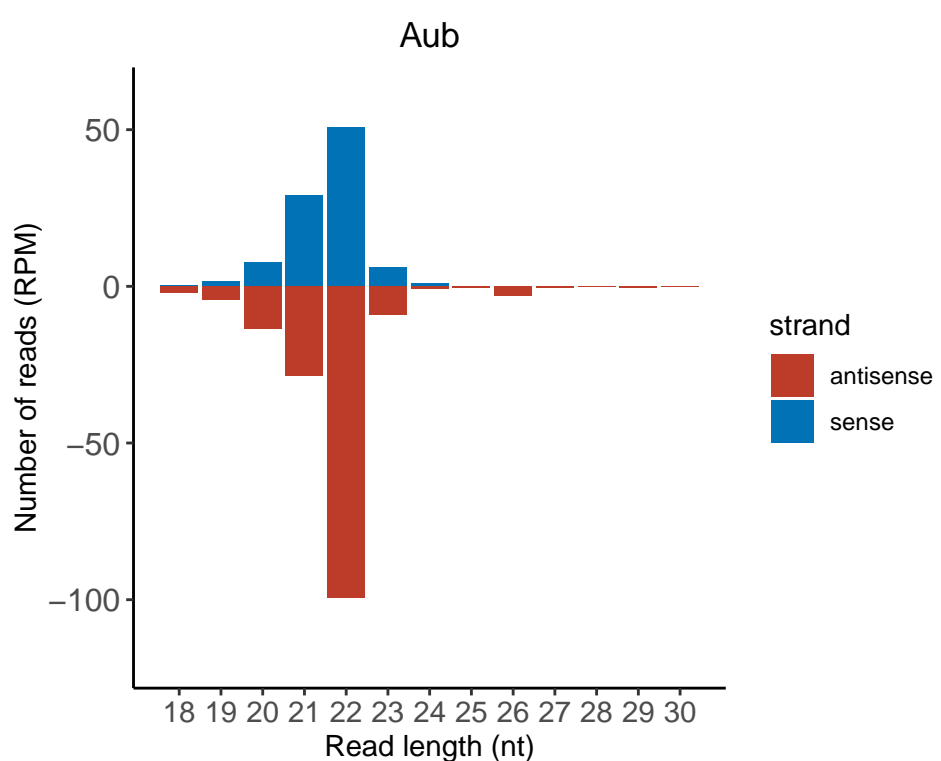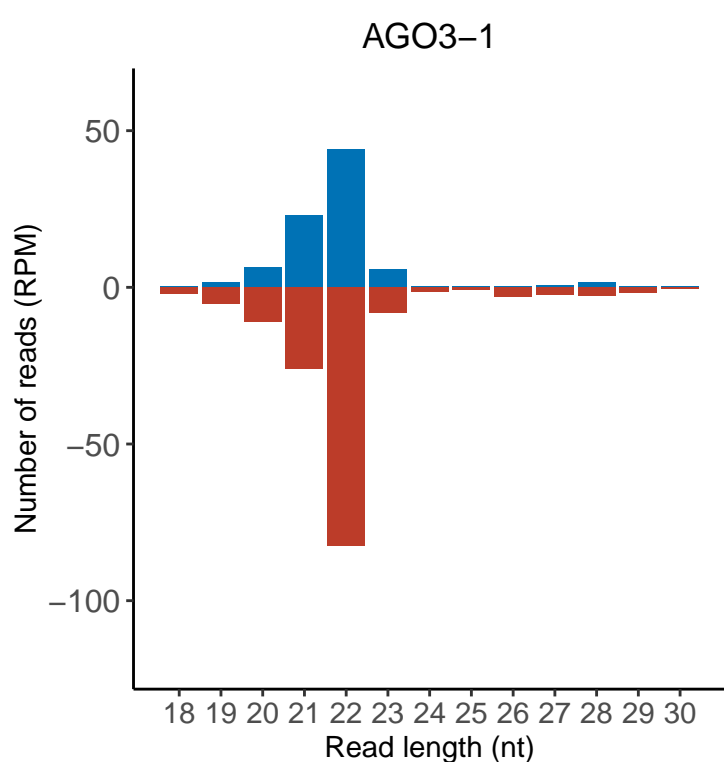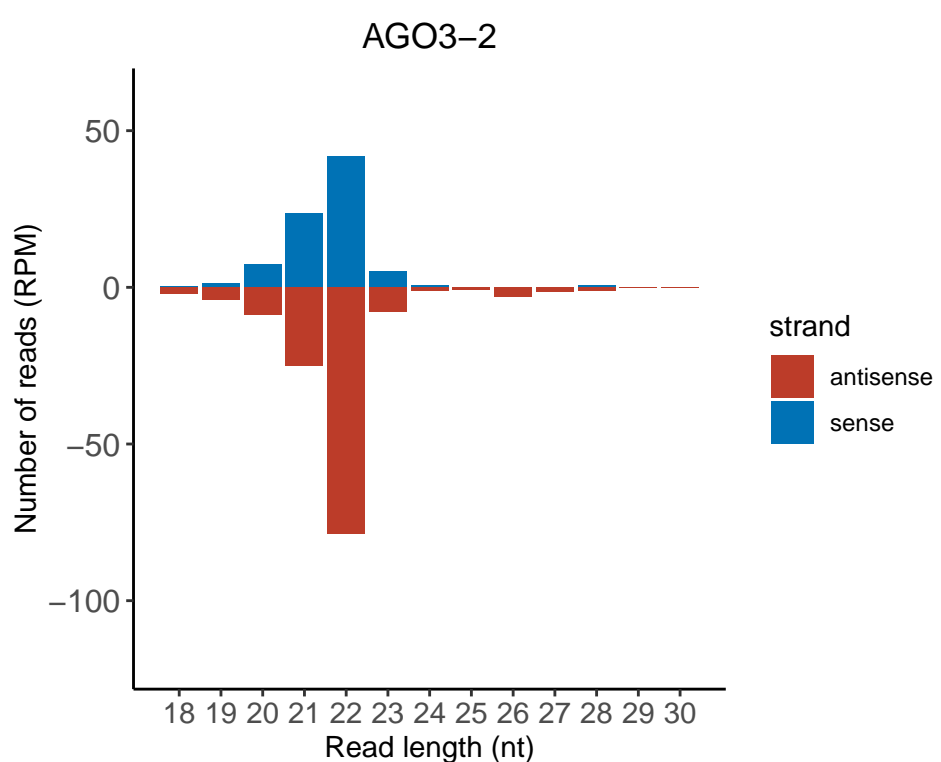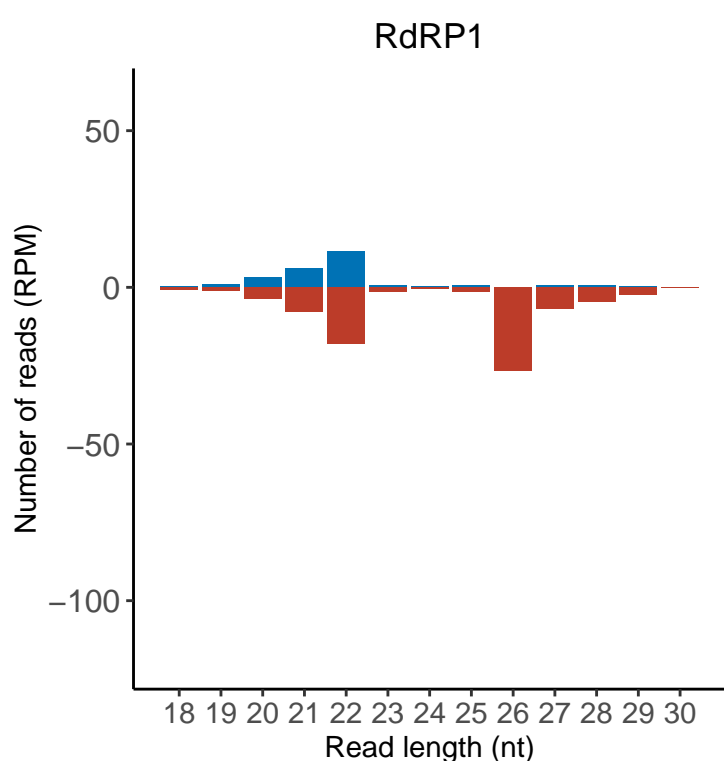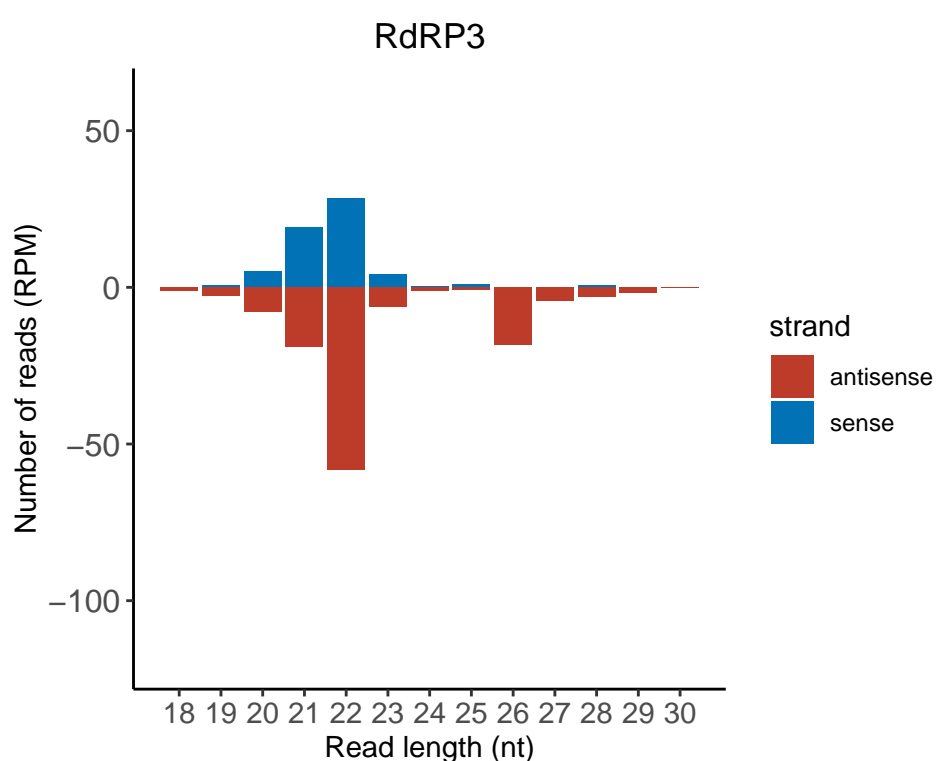

Supplement: S1 Data — On the Normalized and Relative levels pages, normalized read counts (RPM) and relative levels compared to the control (dsGFP) library were used. TEs with more than 50RPM and Coding Genes with more than 3.5RPM on average in KD libraries are included in this website. For viral sequences, reference sequences were generated by assembling sRNA sequences (See the Analysis of sRNAseq data section in Materials and methods.) and size distributions of sRNA reads mapped to each contig are shown. Reads were first grouped by their 5’ nucleotides, and reads of each length were counted. Full tables including TEs and Coding genes with fewer reads are in the “FullTable” folder. (ZIP) [file pone.0281195.s017.zip › SupplementaryData/Links/PDFs/Motif:rnd-1_family-16TE_lengthdistribution.pdf]

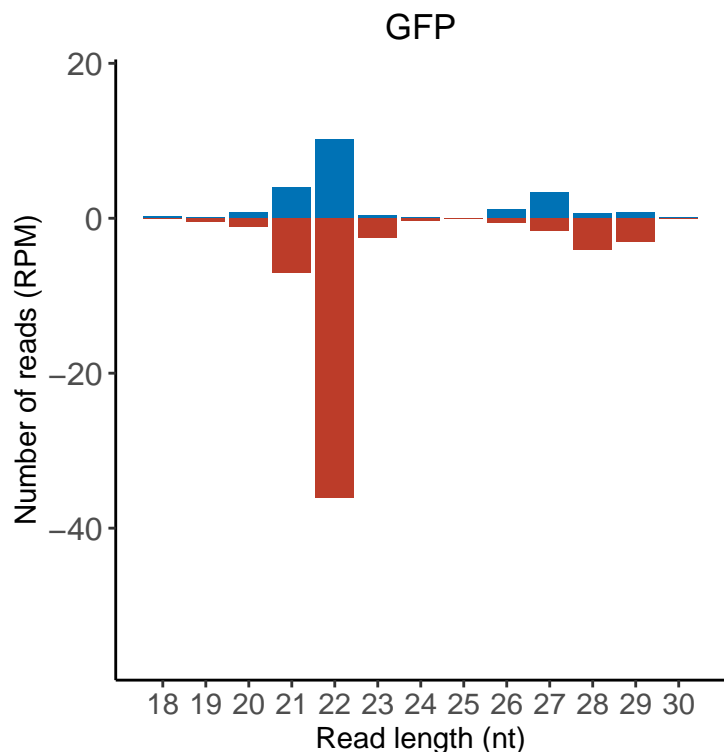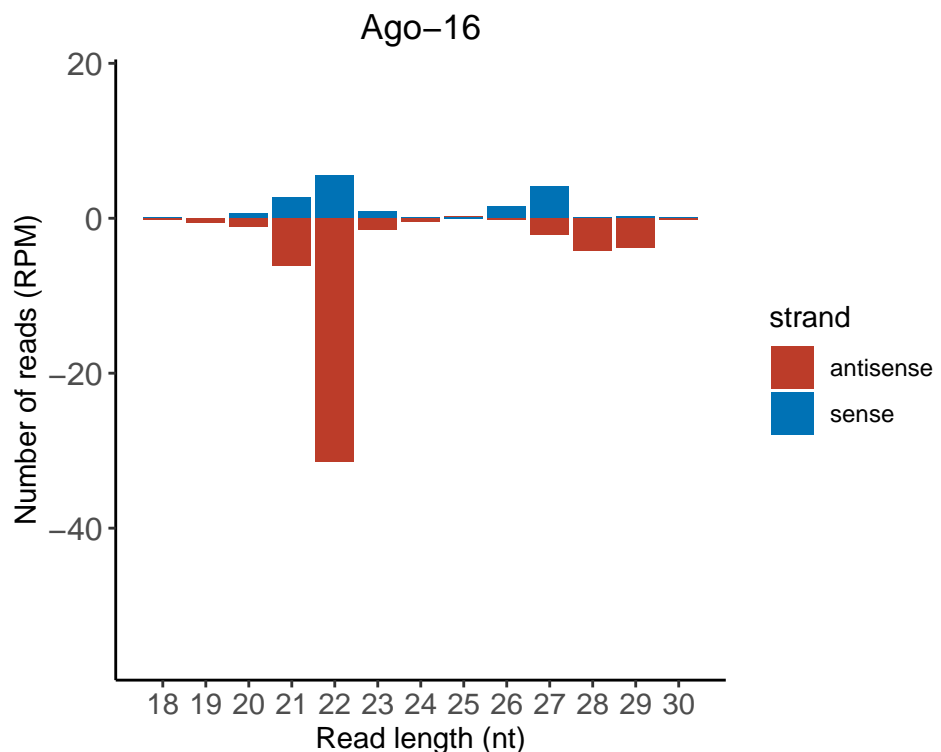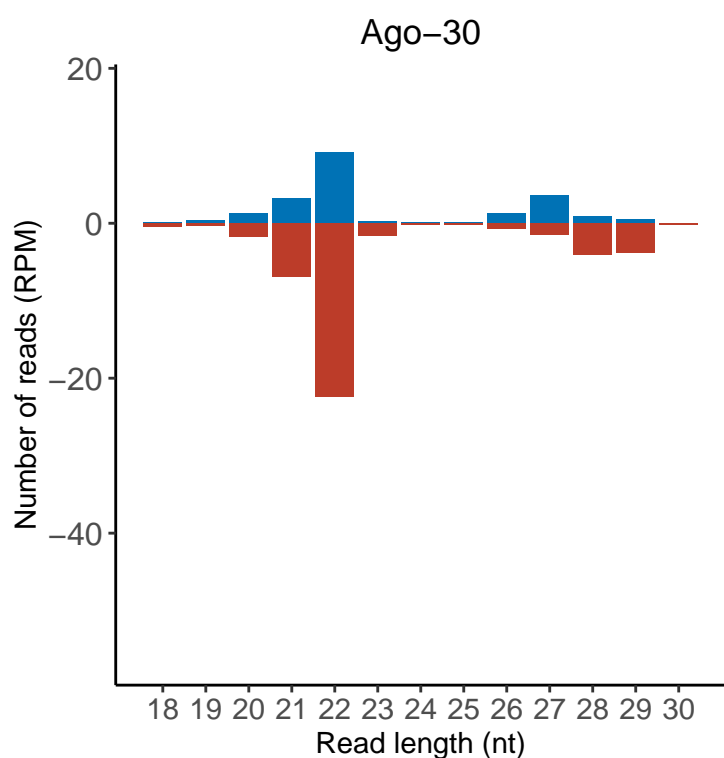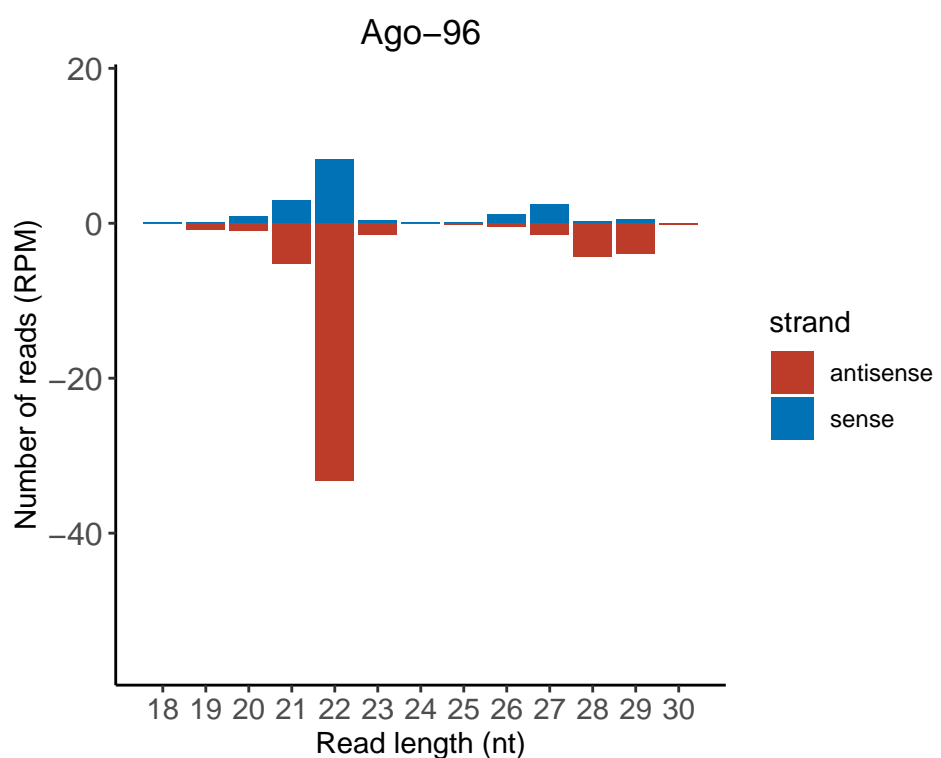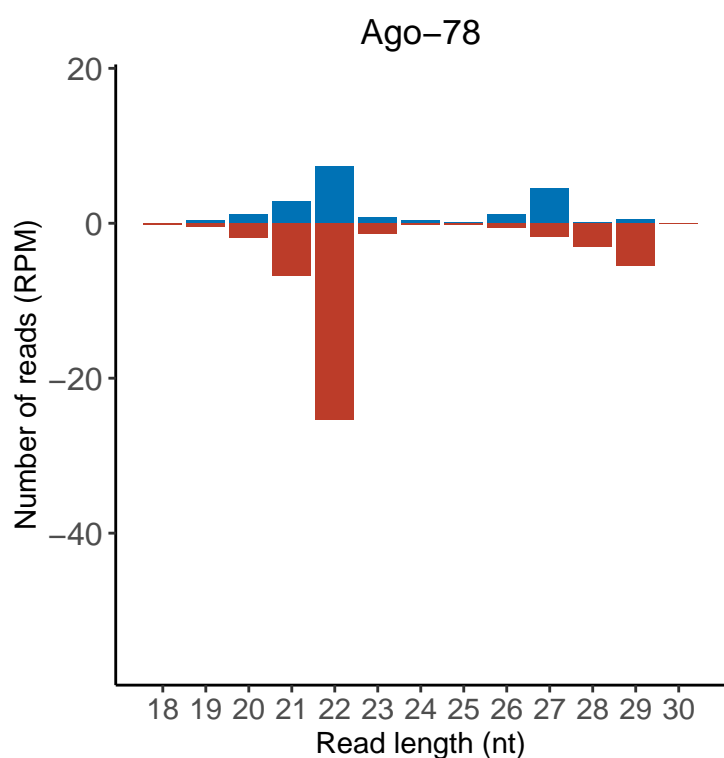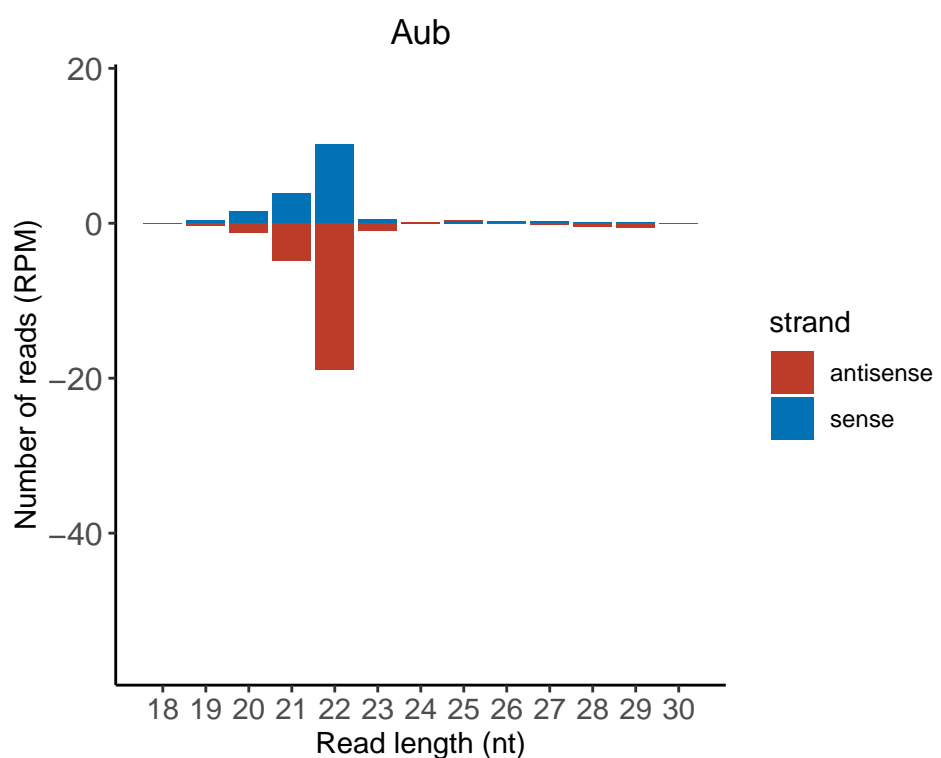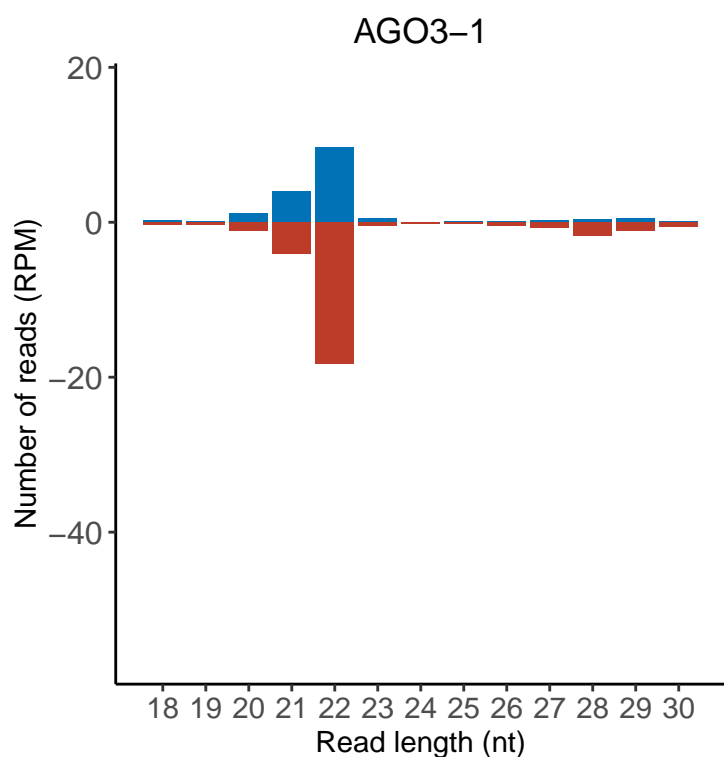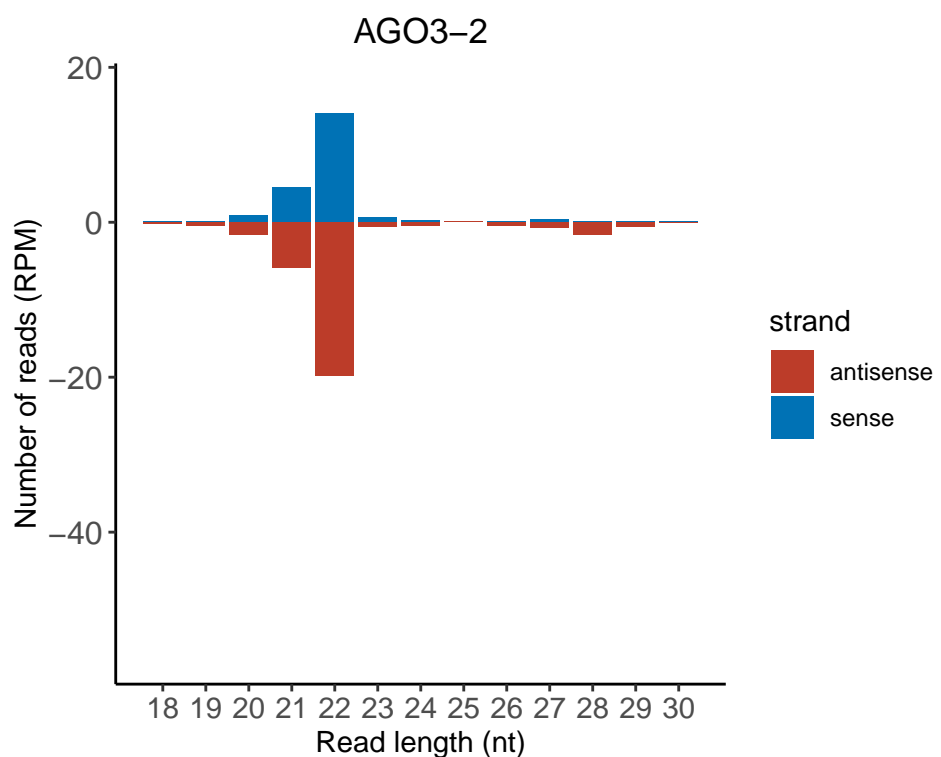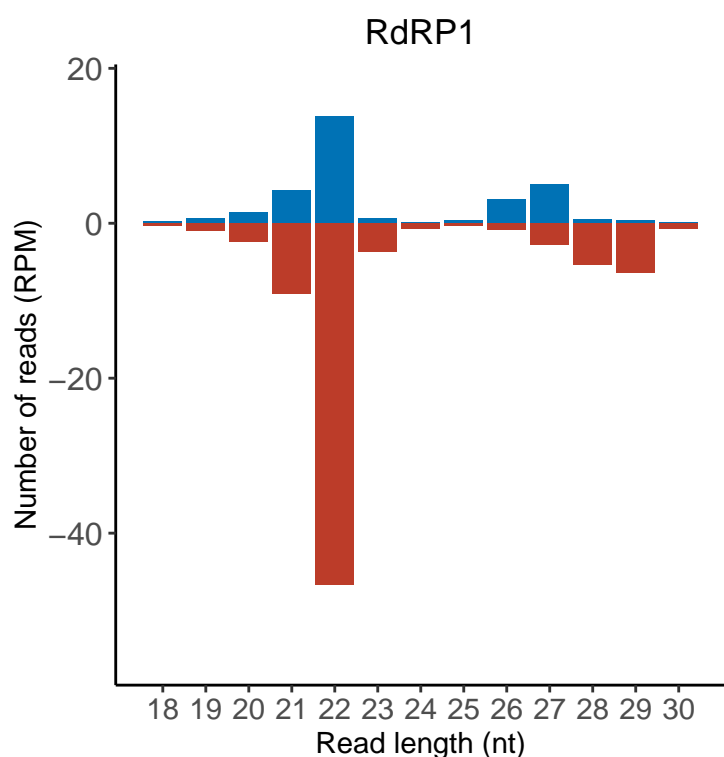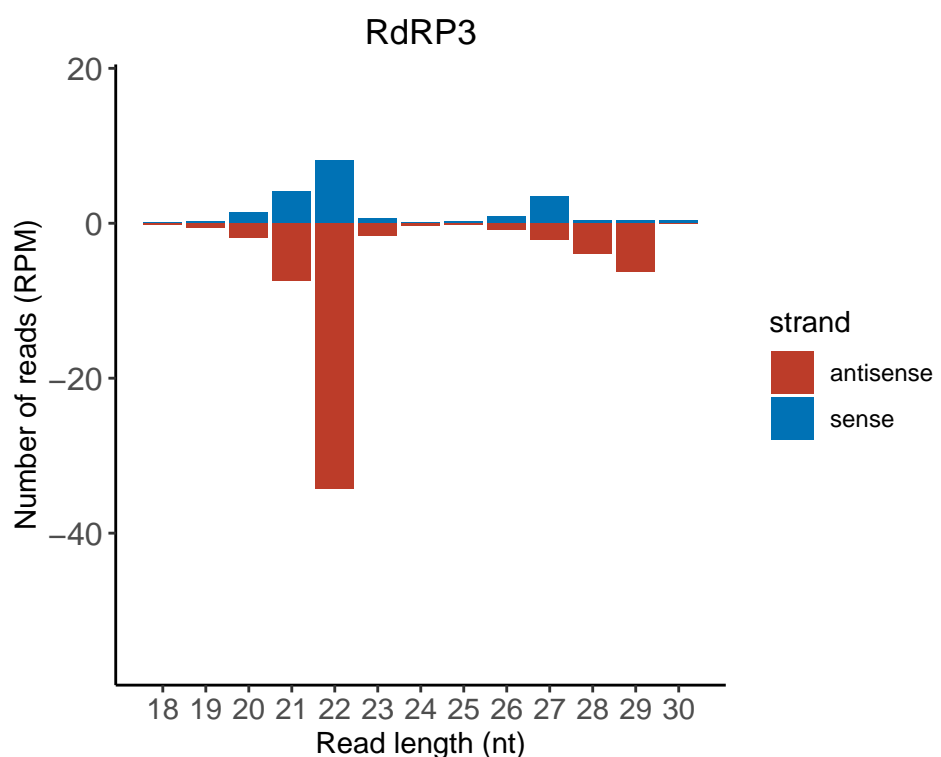

Supplement: S1 Data — On the Normalized and Relative levels pages, normalized read counts (RPM) and relative levels compared to the control (dsGFP) library were used. TEs with more than 50RPM and Coding Genes with more than 3.5RPM on average in KD libraries are included in this website. For viral sequences, reference sequences were generated by assembling sRNA sequences (See the Analysis of sRNAseq data section in Materials and methods.) and size distributions of sRNA reads mapped to each contig are shown. Reads were first grouped by their 5’ nucleotides, and reads of each length were counted. Full tables including TEs and Coding genes with fewer reads are in the “FullTable” folder. (ZIP) [file pone.0281195.s017.zip › SupplementaryData/Links/PDFs/Motif:rnd-6_family-3172TE_lengthdistribution.pdf]

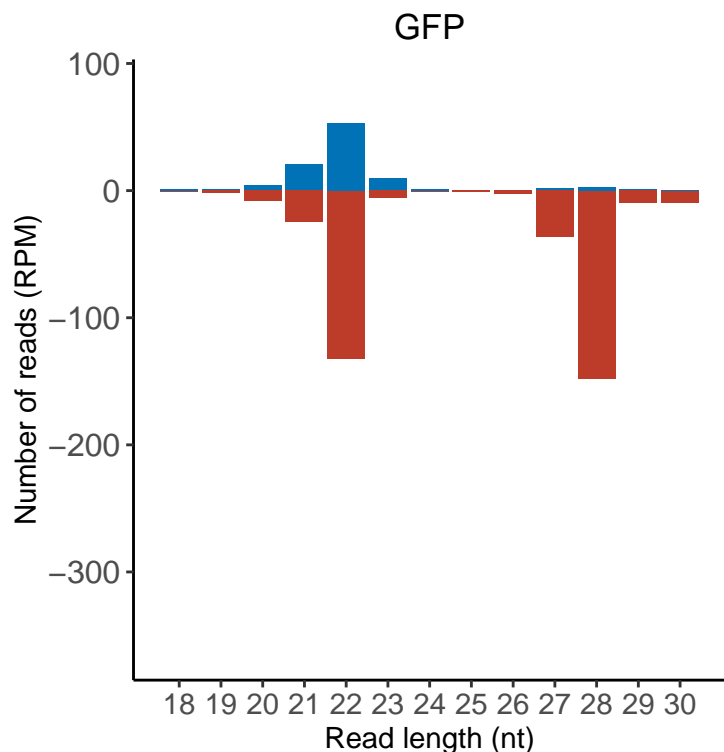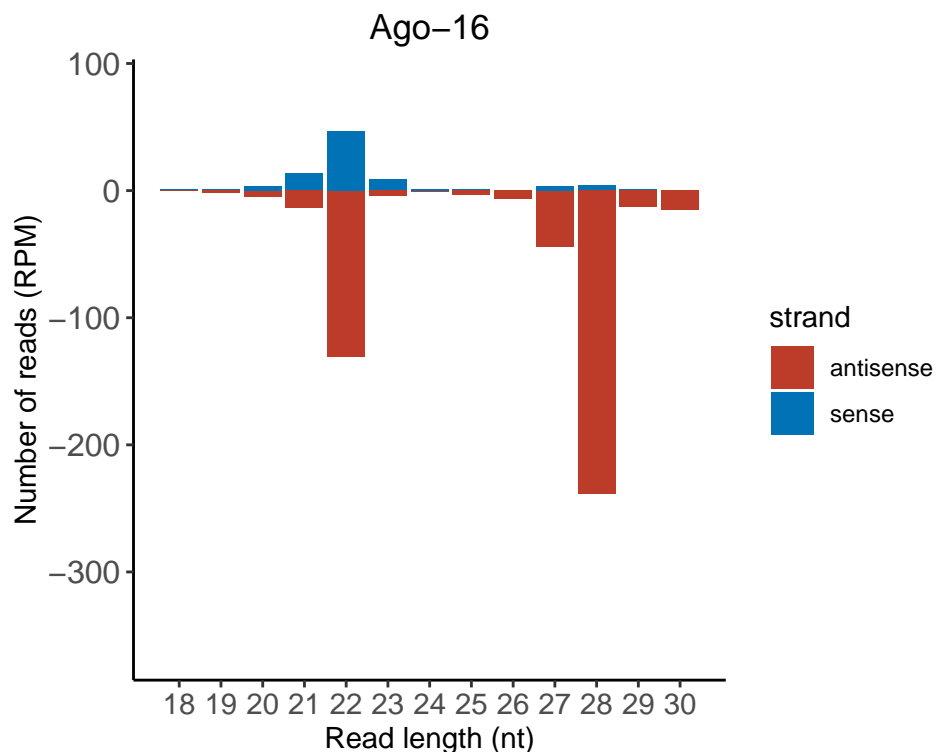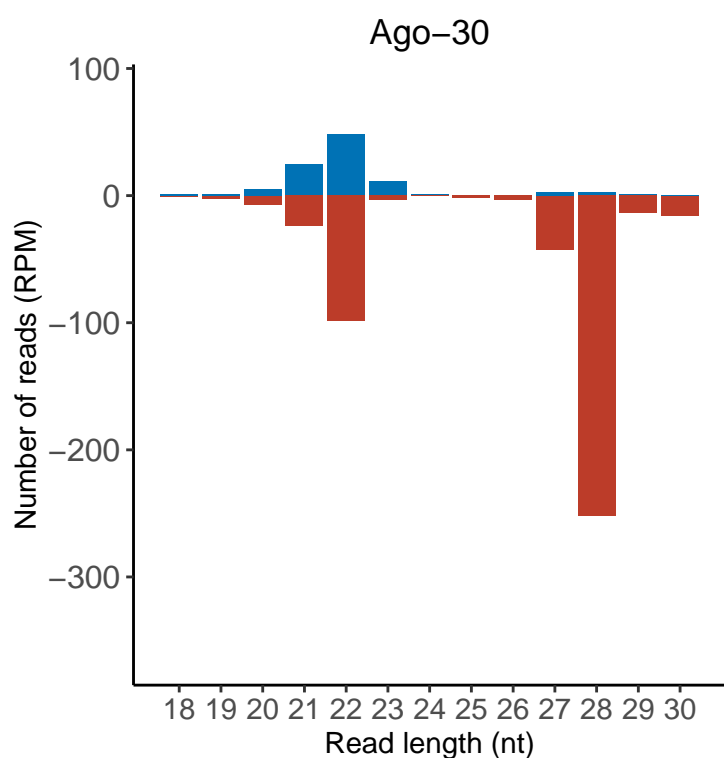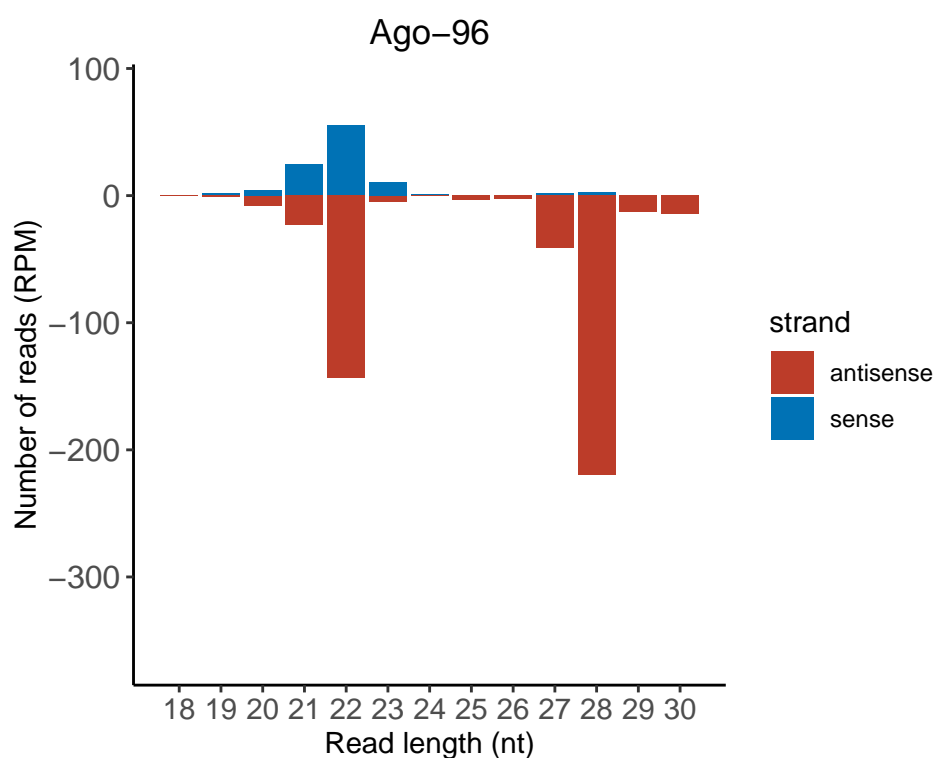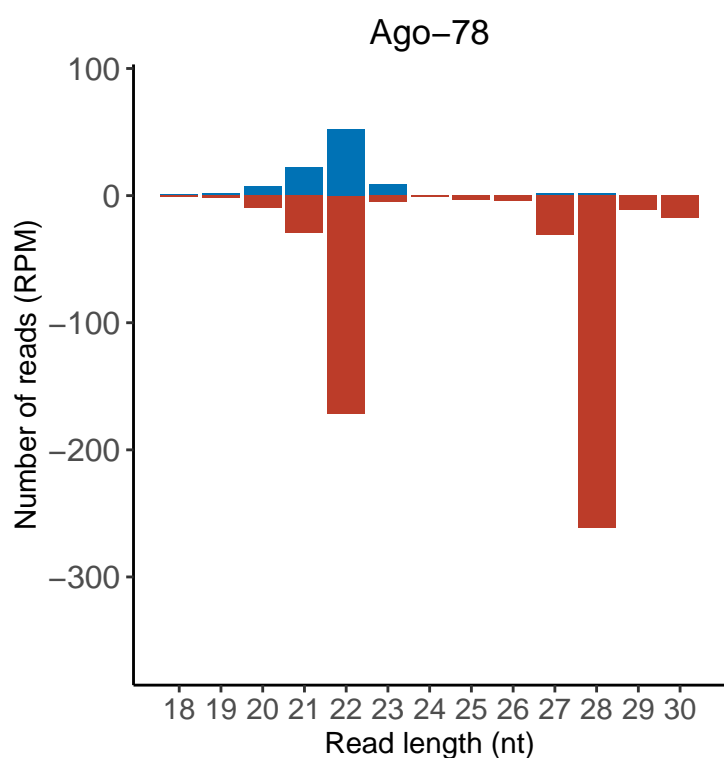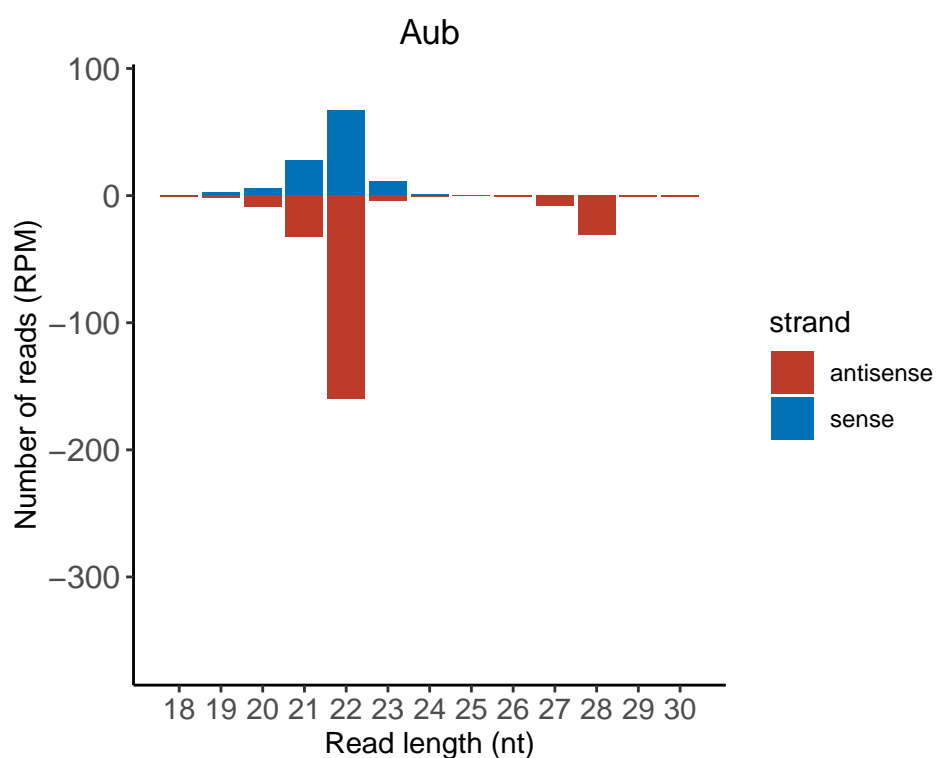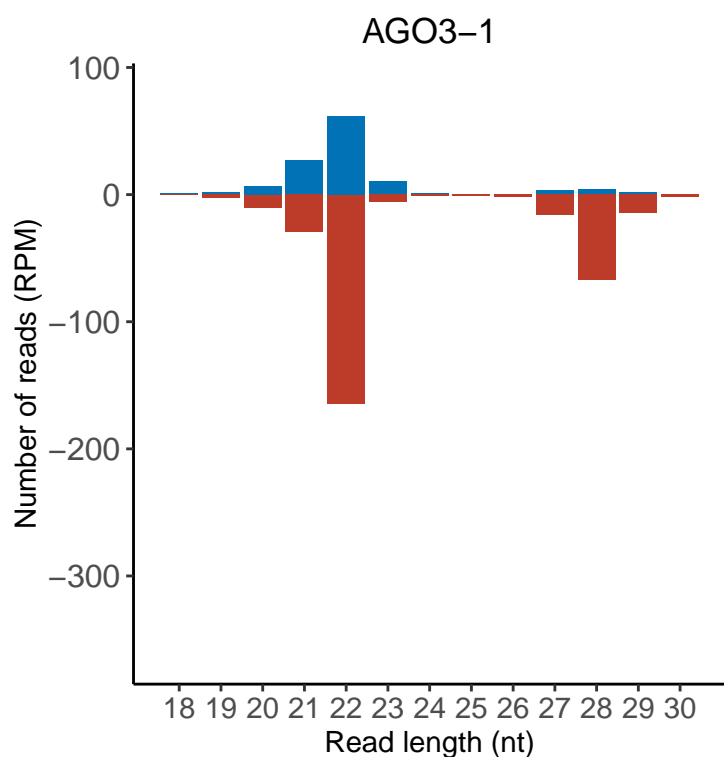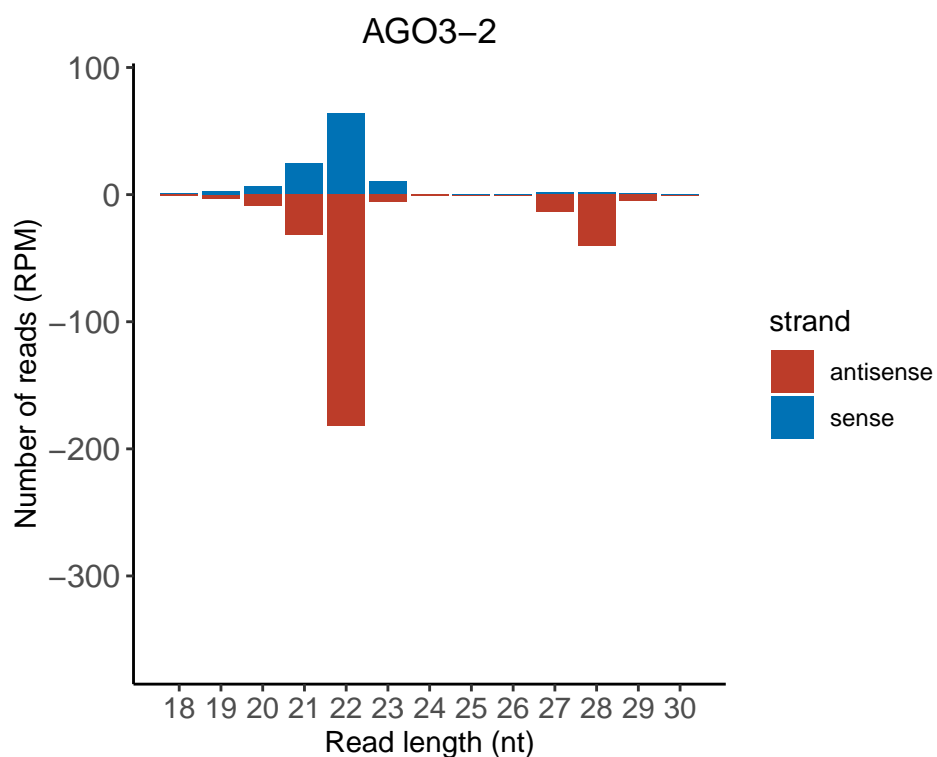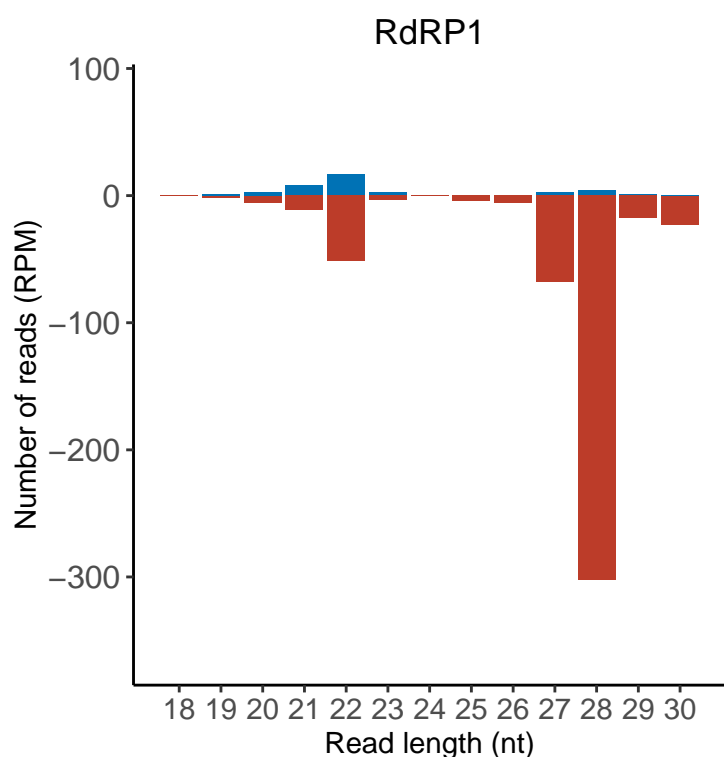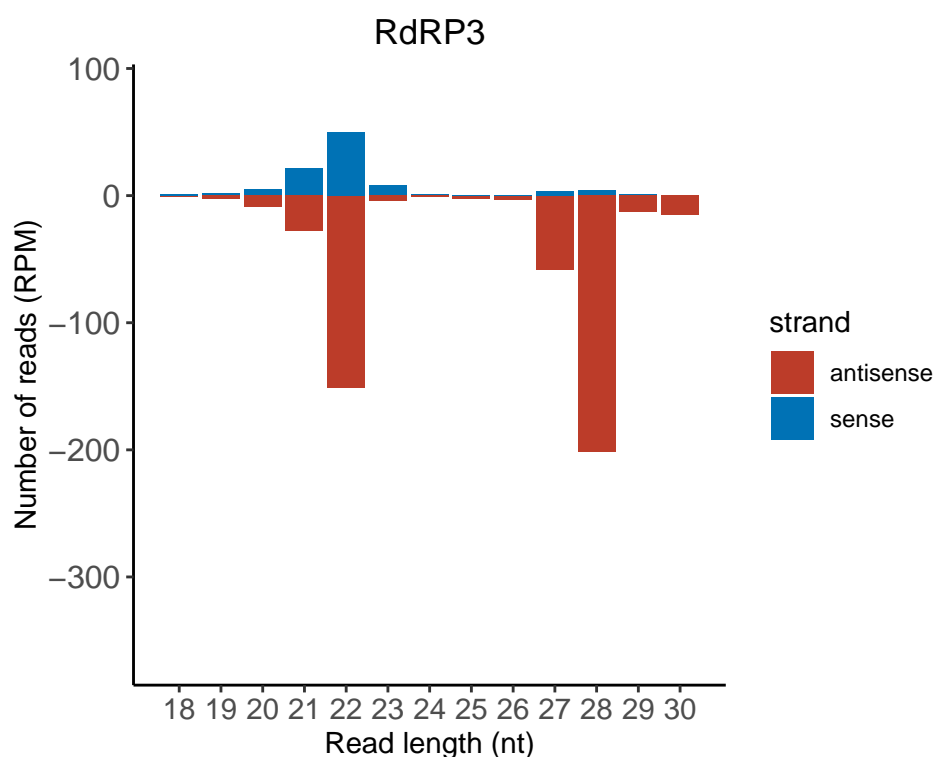

Supplement: S1 Data — On the Normalized and Relative levels pages, normalized read counts (RPM) and relative levels compared to the control (dsGFP) library were used. TEs with more than 50RPM and Coding Genes with more than 3.5RPM on average in KD libraries are included in this website. For viral sequences, reference sequences were generated by assembling sRNA sequences (See the Analysis of sRNAseq data section in Materials and methods.) and size distributions of sRNA reads mapped to each contig are shown. Reads were first grouped by their 5’ nucleotides, and reads of each length were counted. Full tables including TEs and Coding genes with fewer reads are in the “FullTable” folder. (ZIP) [file pone.0281195.s017.zip › SupplementaryData/Links/PDFs/Motif:rnd-5_family-150TE_lengthdistribution.pdf]

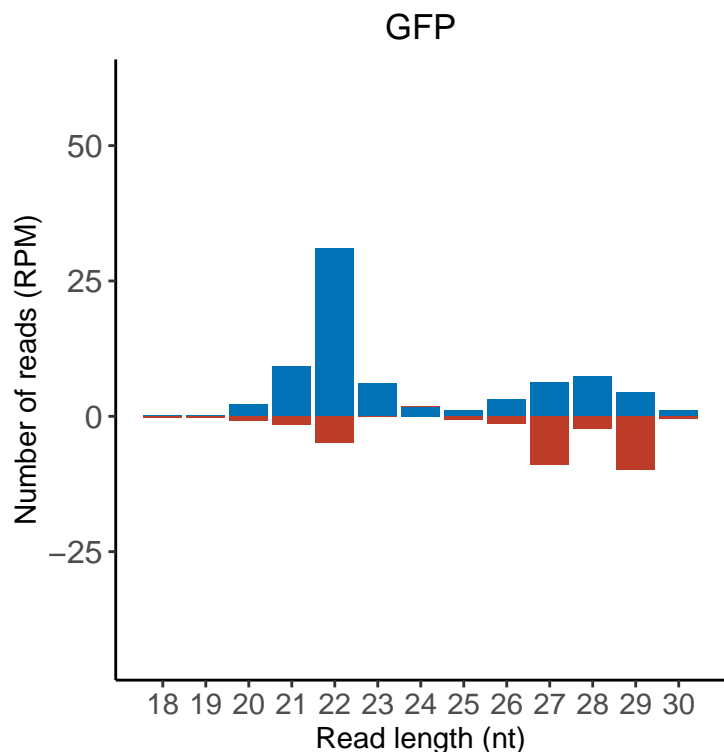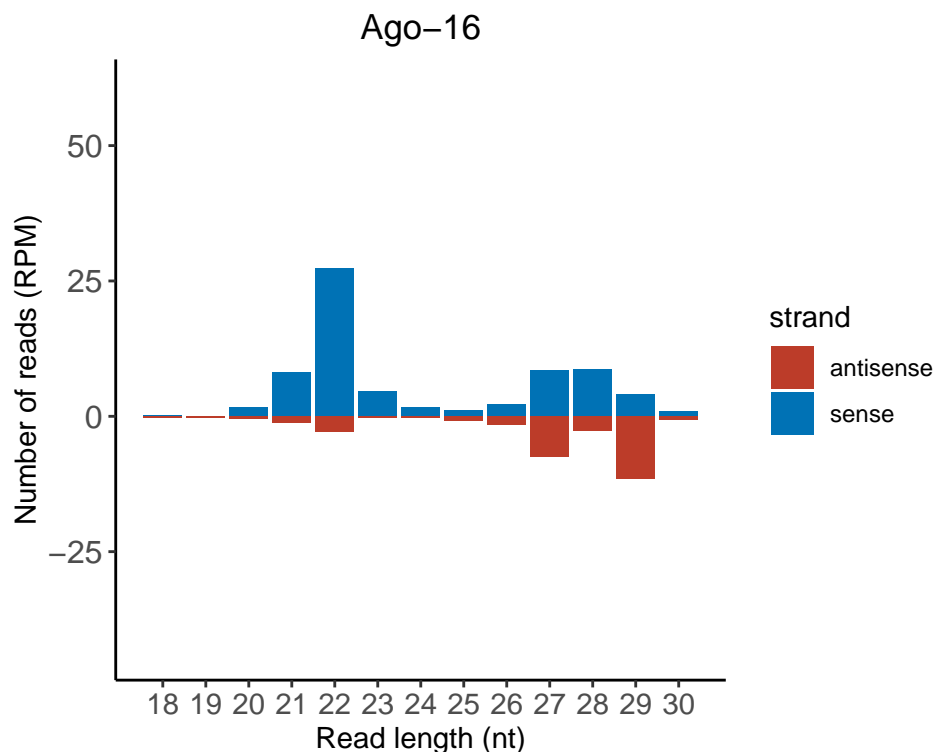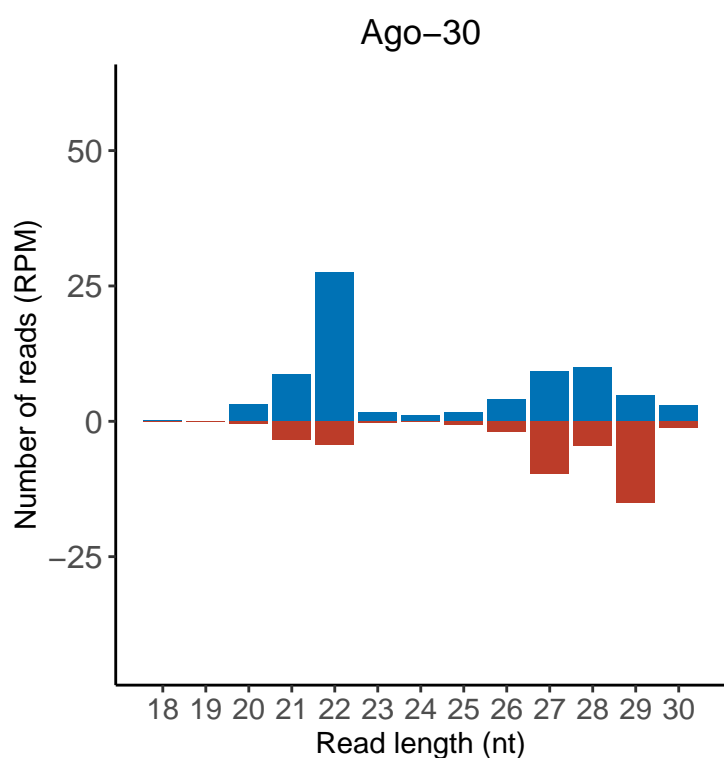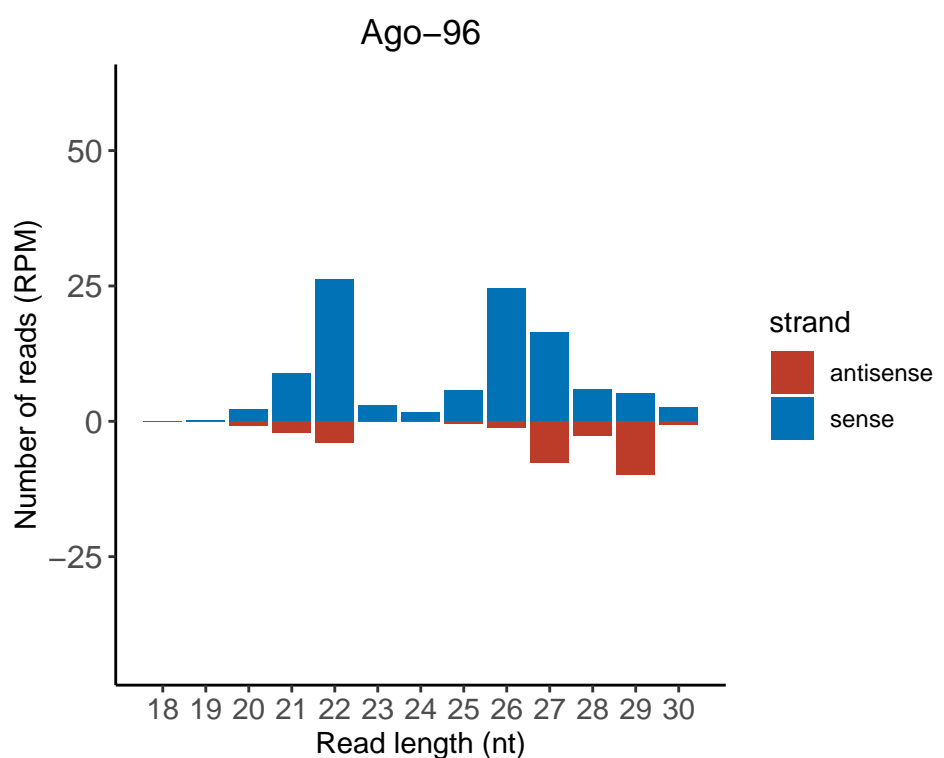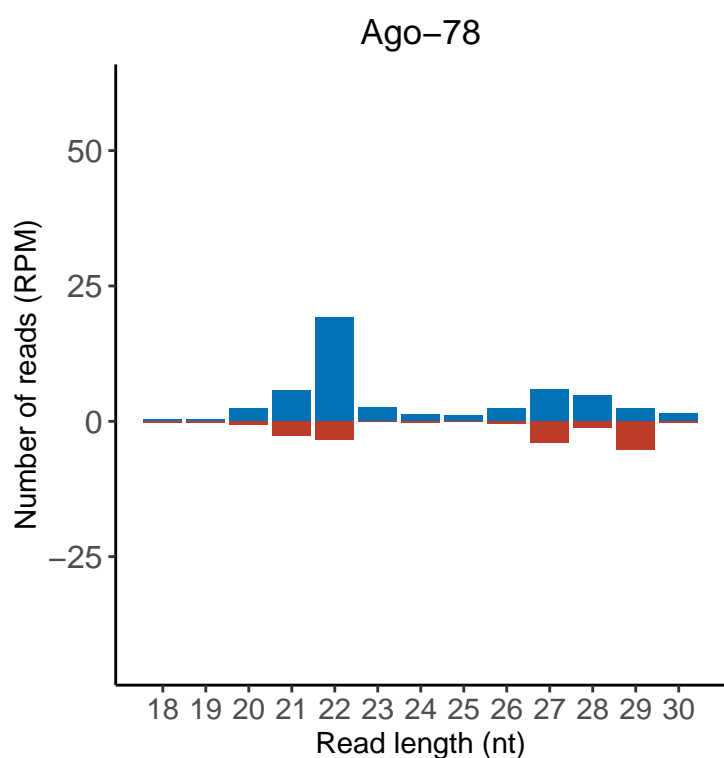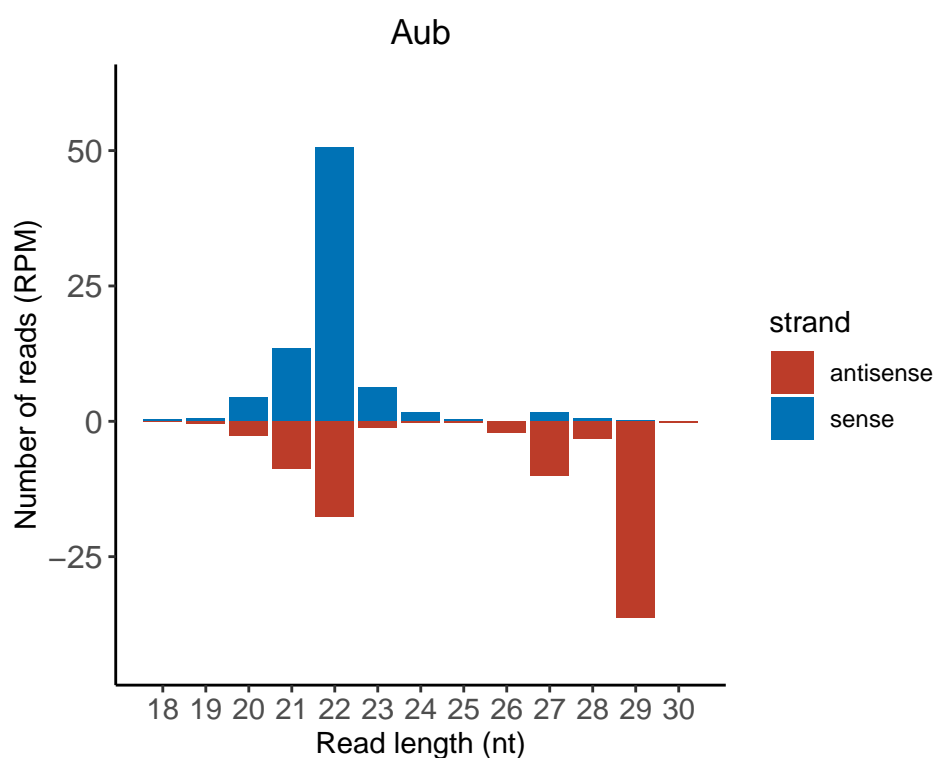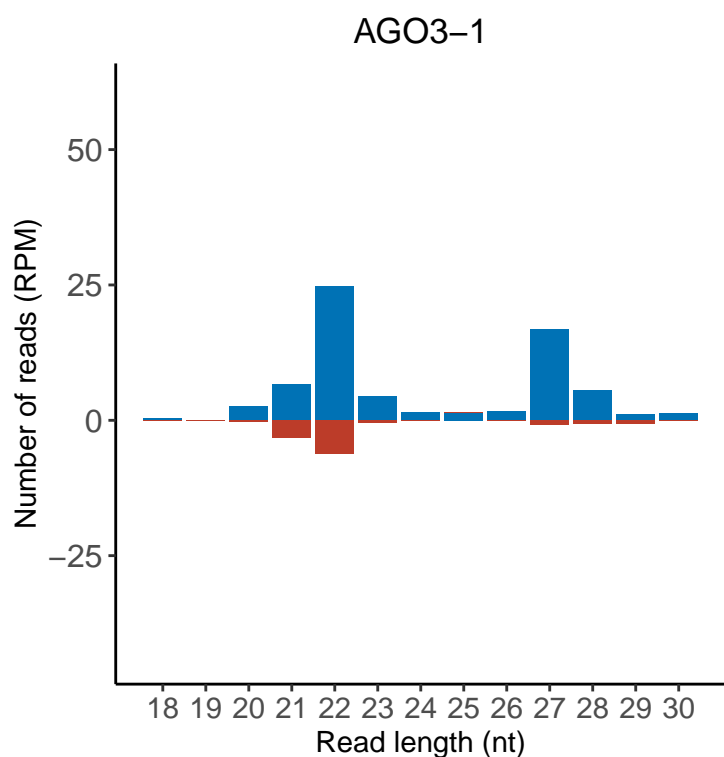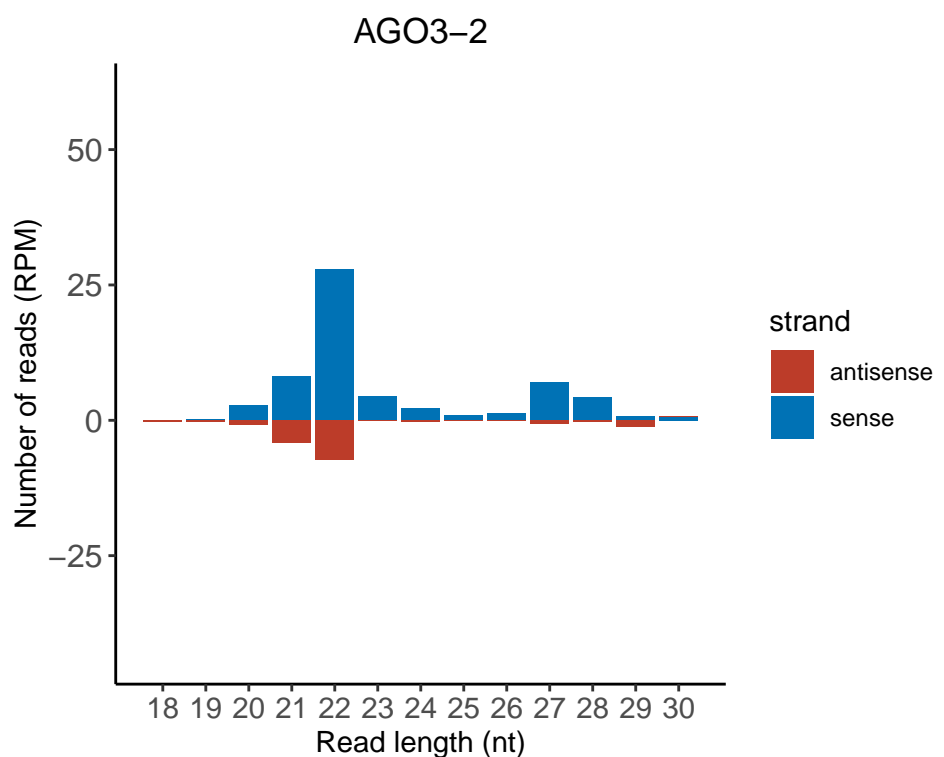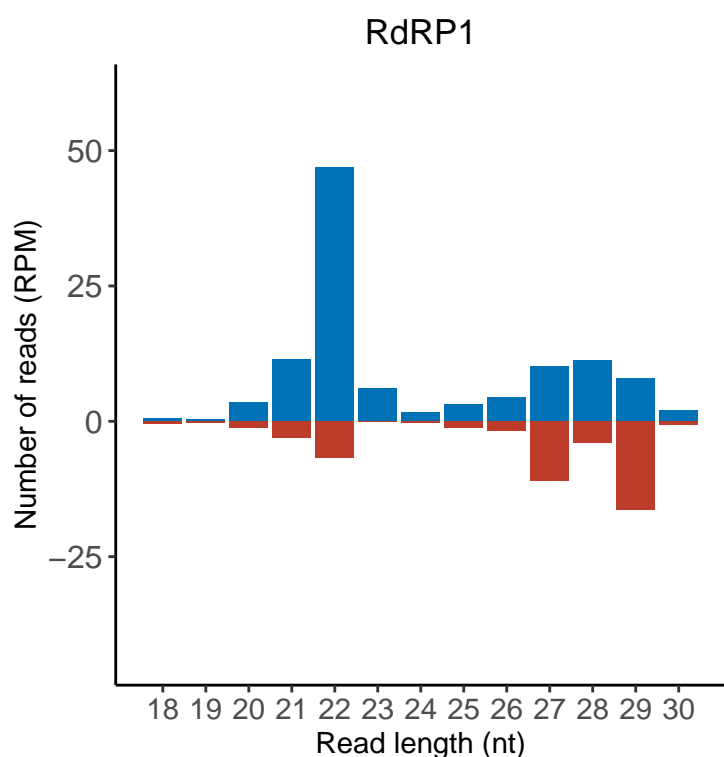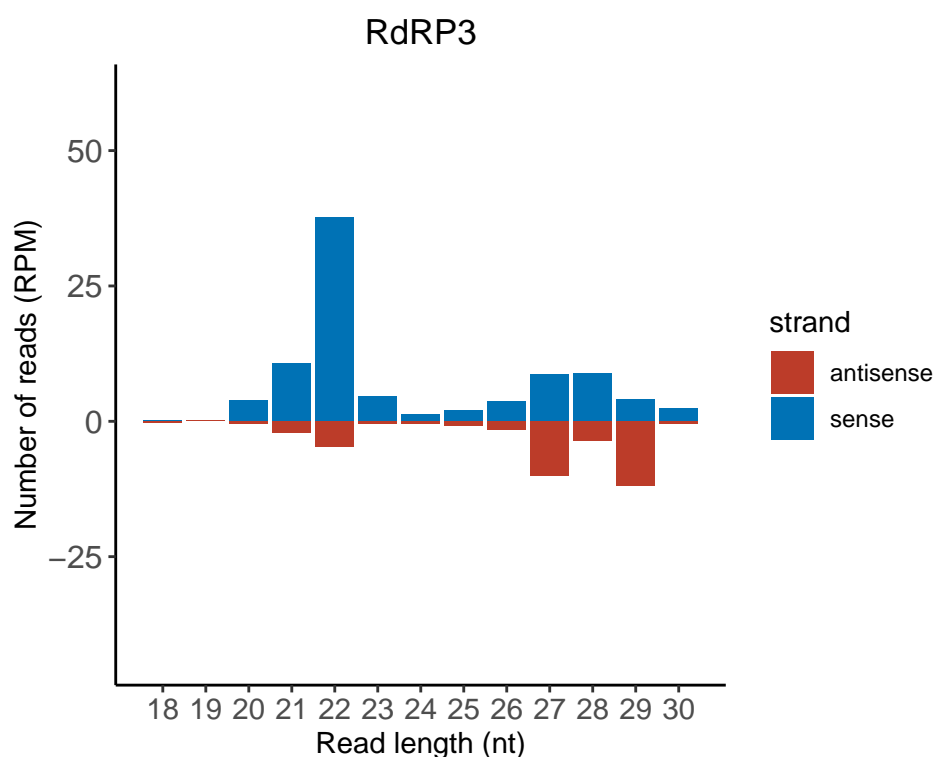

Supplement: S1 Data — On the Normalized and Relative levels pages, normalized read counts (RPM) and relative levels compared to the control (dsGFP) library were used. TEs with more than 50RPM and Coding Genes with more than 3.5RPM on average in KD libraries are included in this website. For viral sequences, reference sequences were generated by assembling sRNA sequences (See the Analysis of sRNAseq data section in Materials and methods.) and size distributions of sRNA reads mapped to each contig are shown. Reads were first grouped by their 5’ nucleotides, and reads of each length were counted. Full tables including TEs and Coding genes with fewer reads are in the “FullTable” folder. (ZIP) [file pone.0281195.s017.zip › SupplementaryData/Links/PDFs/Motif:rnd-6_family-1241TE_lengthdistribution.pdf]

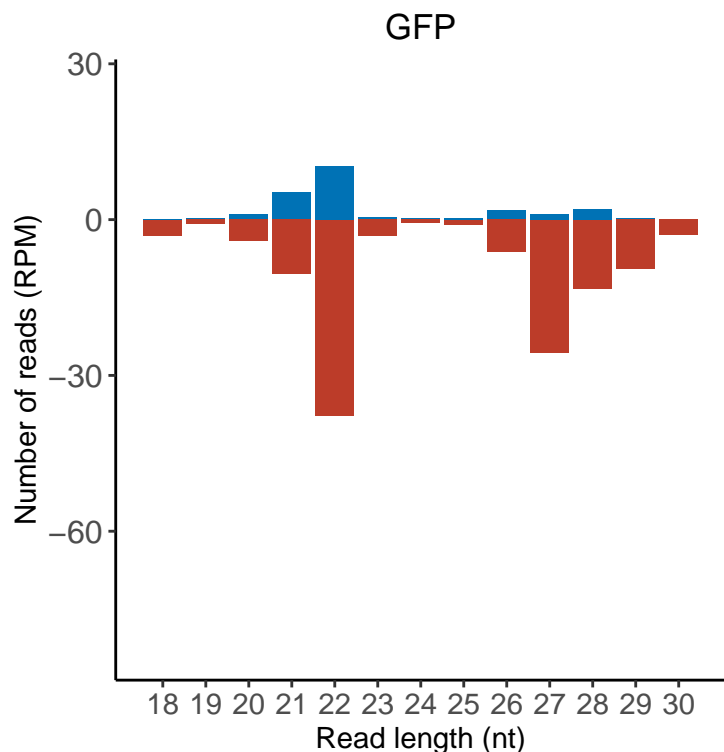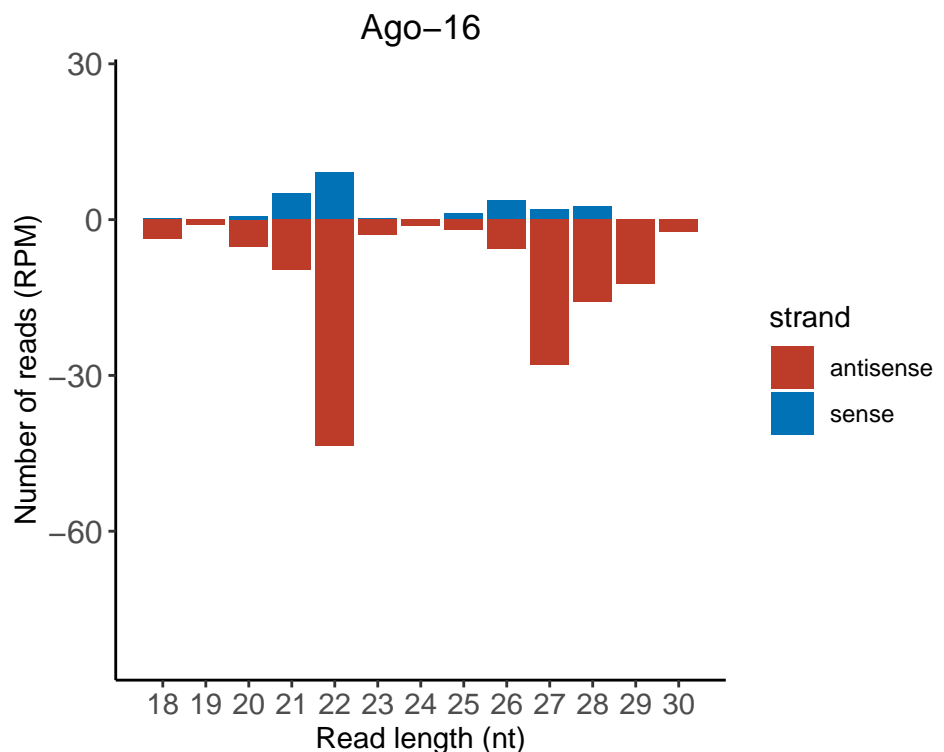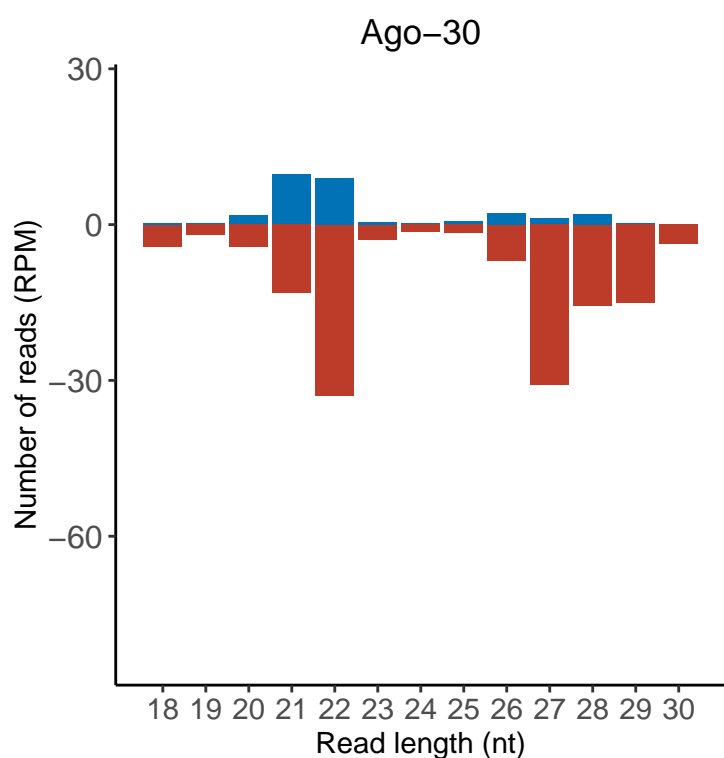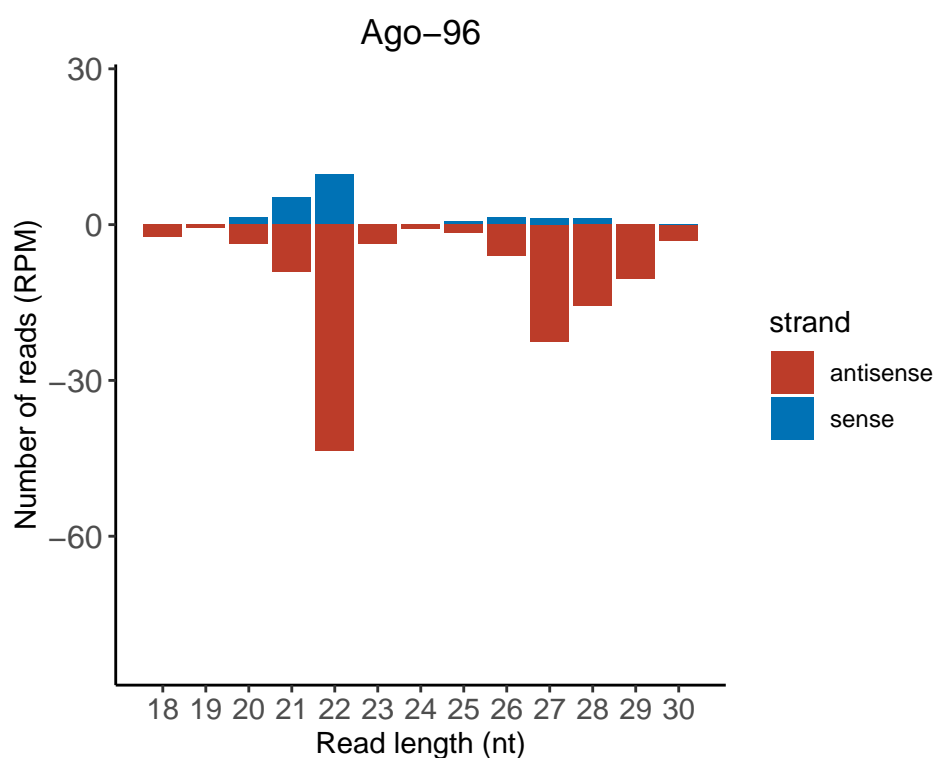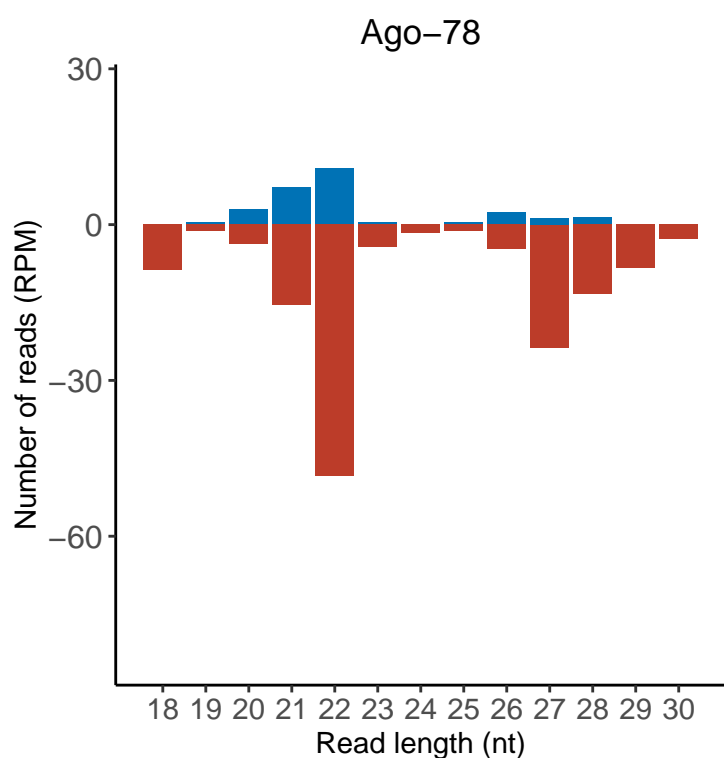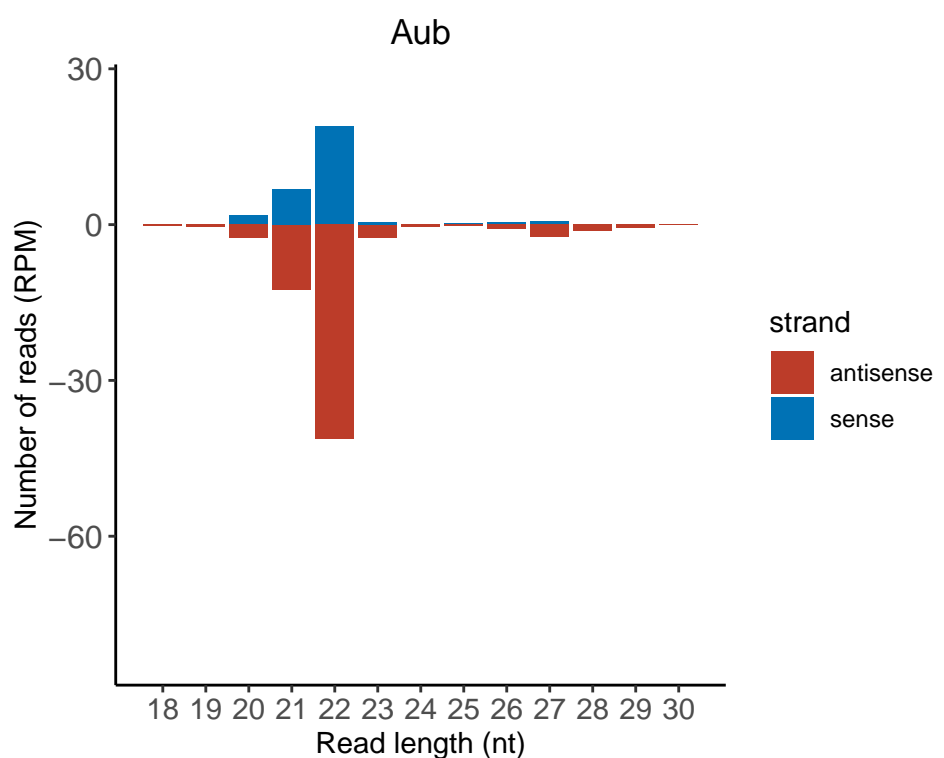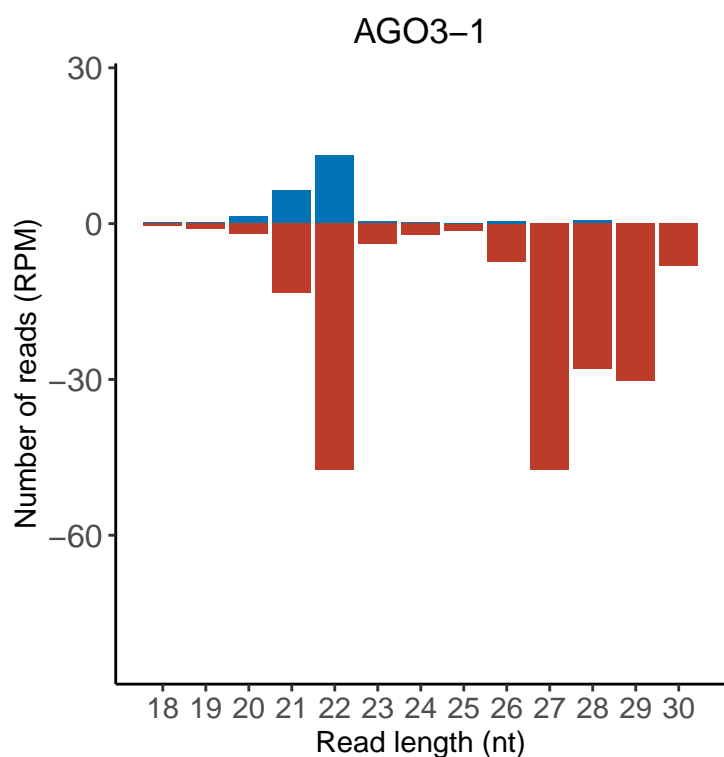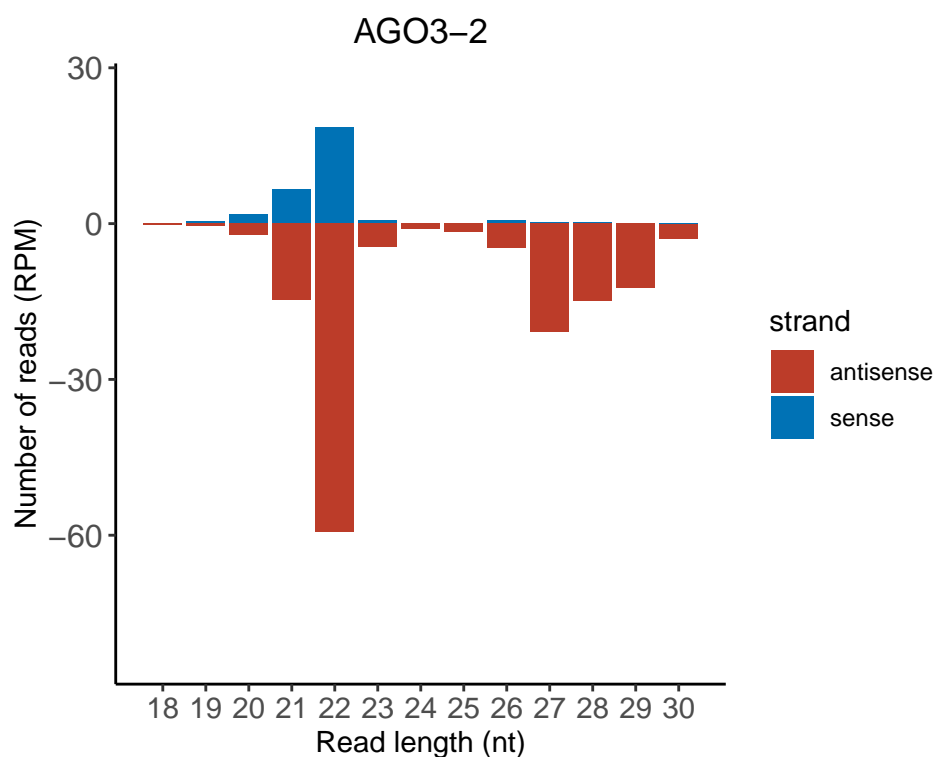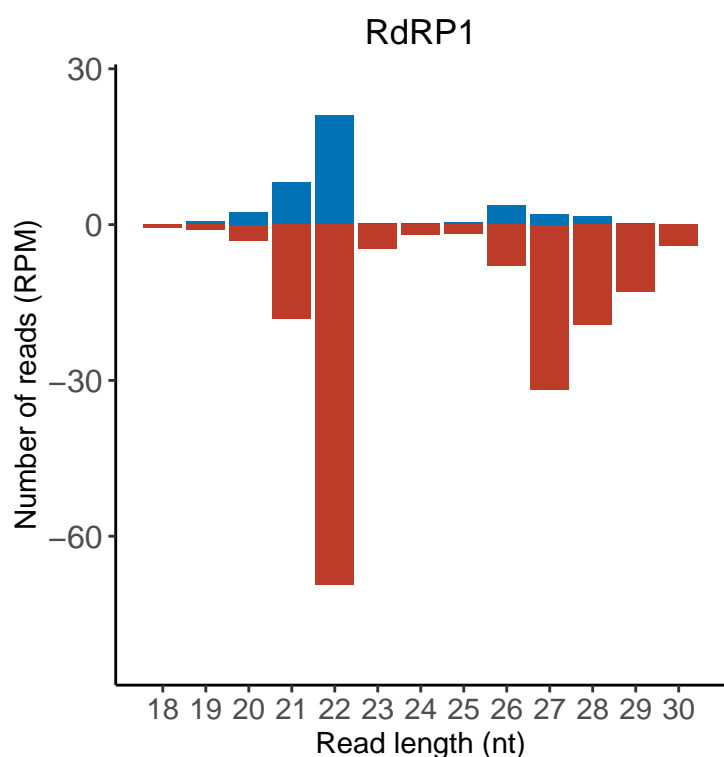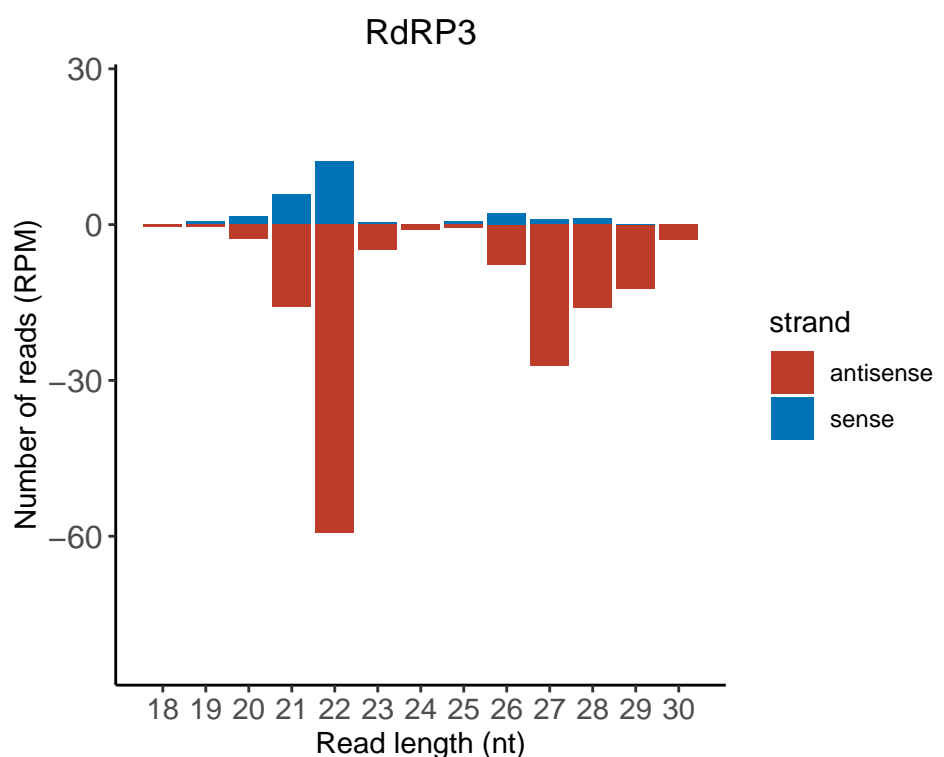

Supplement: S1 Data — On the Normalized and Relative levels pages, normalized read counts (RPM) and relative levels compared to the control (dsGFP) library were used. TEs with more than 50RPM and Coding Genes with more than 3.5RPM on average in KD libraries are included in this website. For viral sequences, reference sequences were generated by assembling sRNA sequences (See the Analysis of sRNAseq data section in Materials and methods.) and size distributions of sRNA reads mapped to each contig are shown. Reads were first grouped by their 5’ nucleotides, and reads of each length were counted. Full tables including TEs and Coding genes with fewer reads are in the “FullTable” folder. (ZIP) [file pone.0281195.s017.zip › SupplementaryData/Links/PDFs/Motif:rnd-6_family-1887TE_lengthdistribution.pdf]

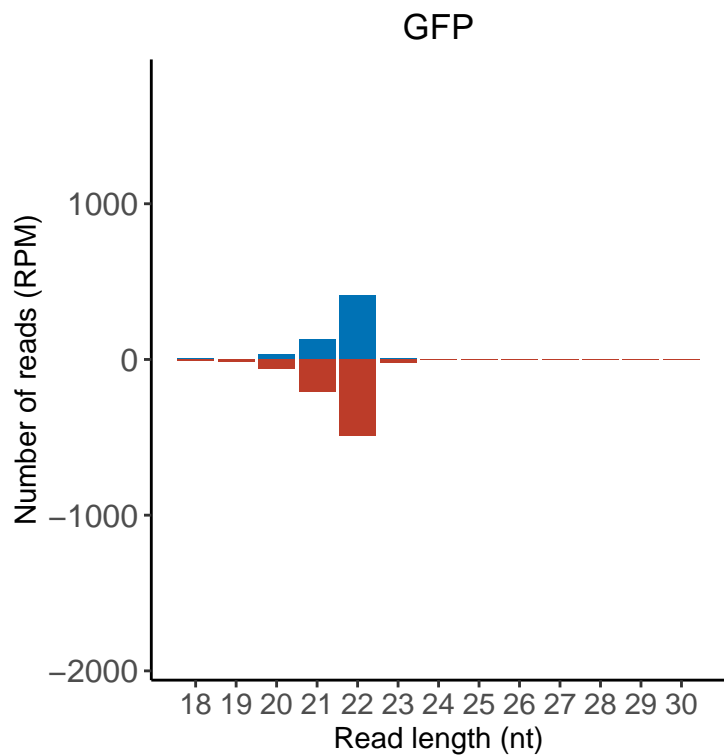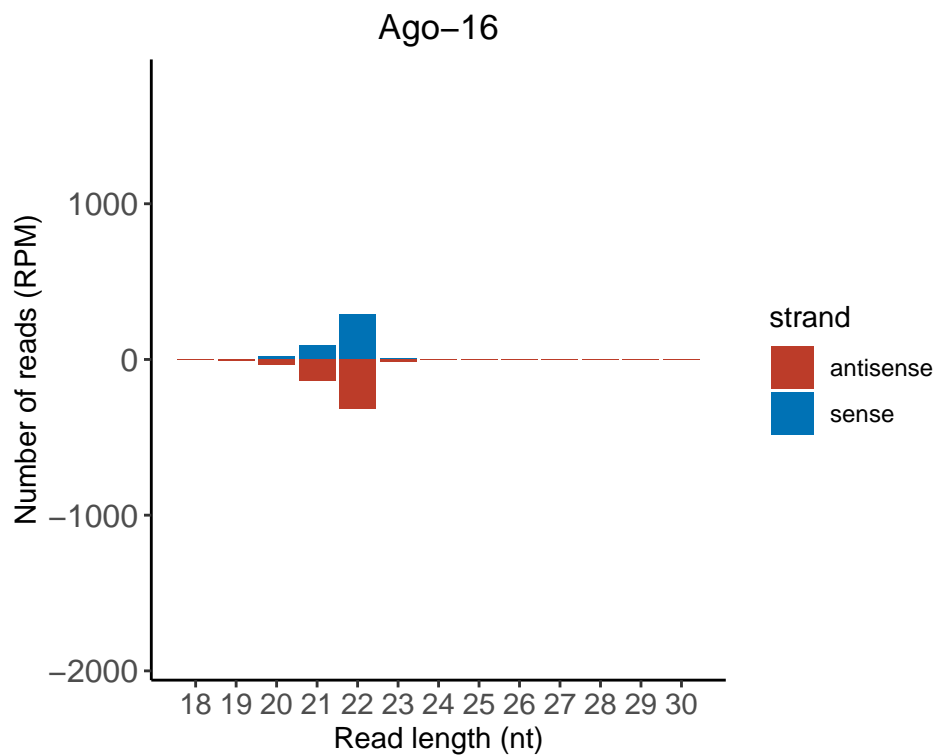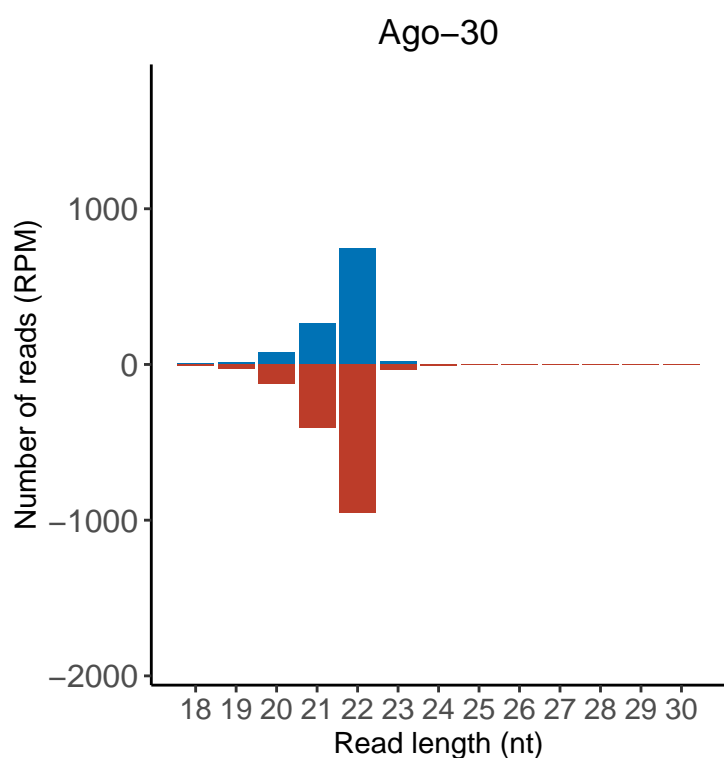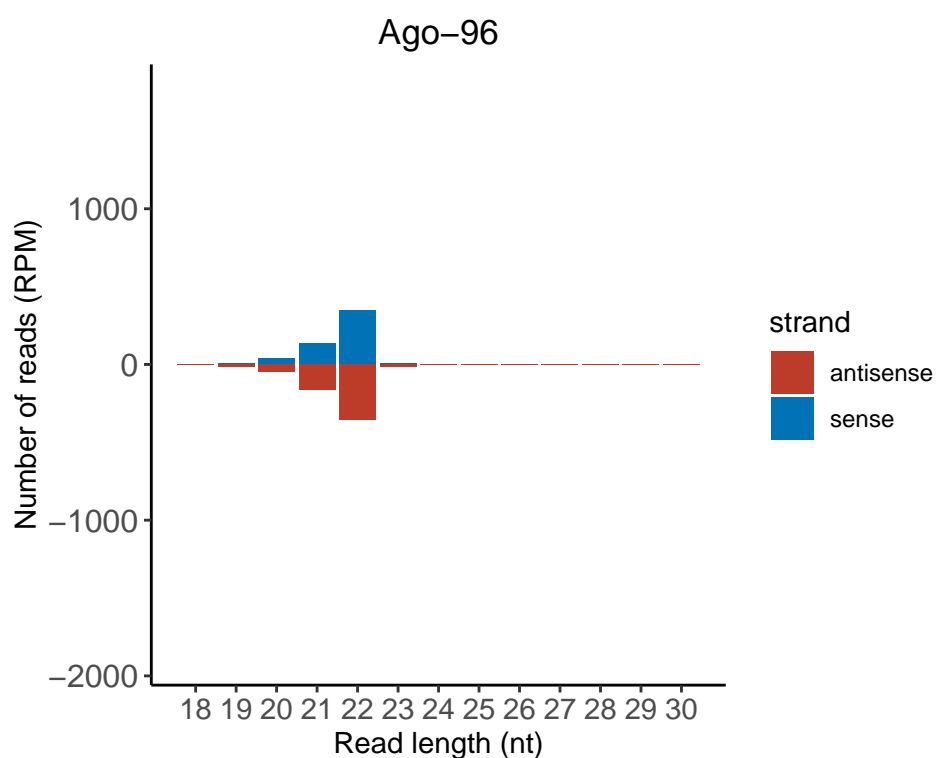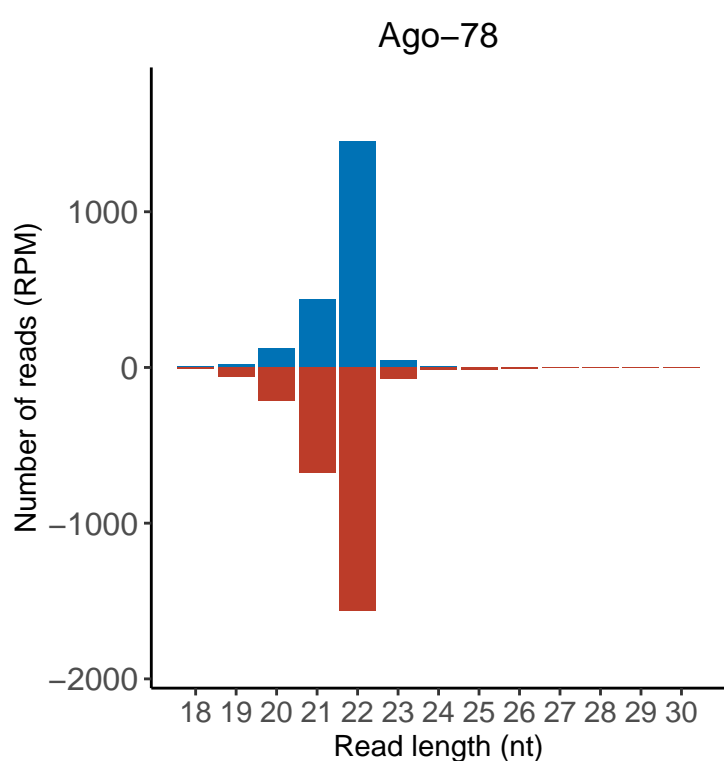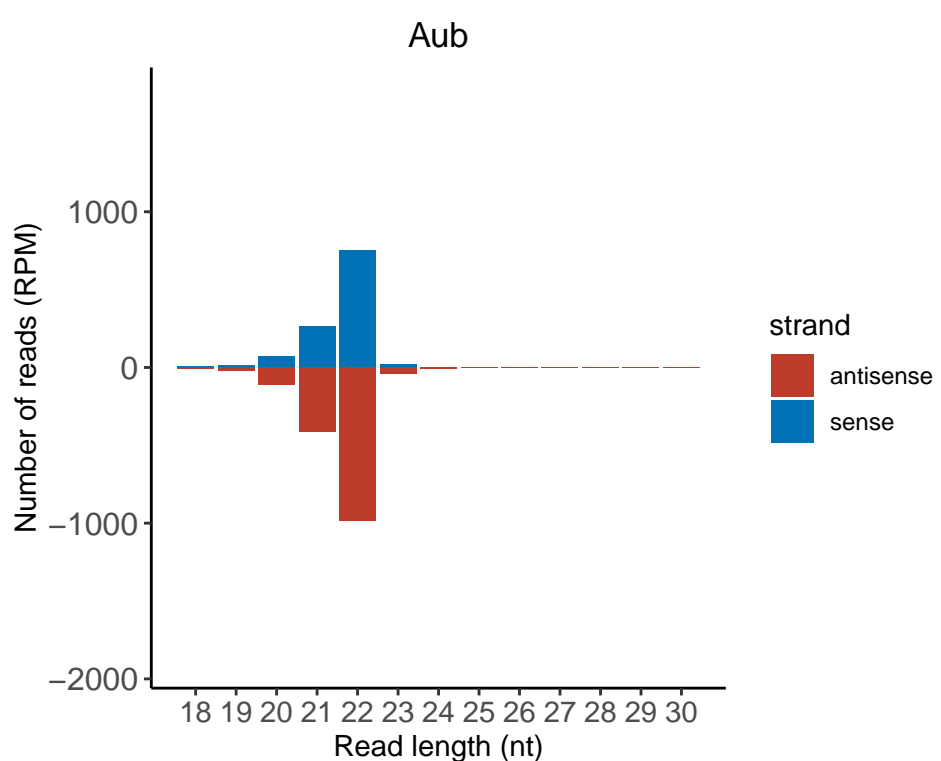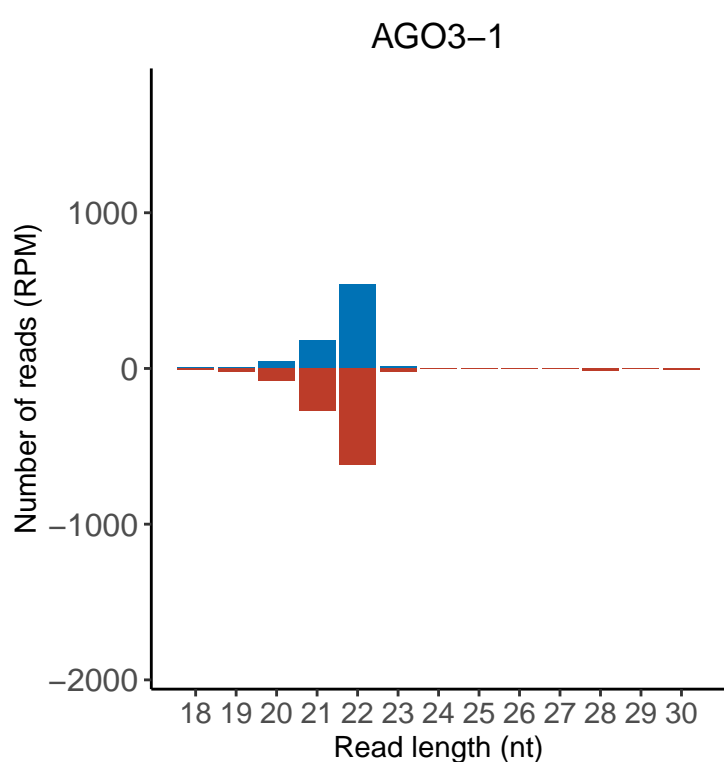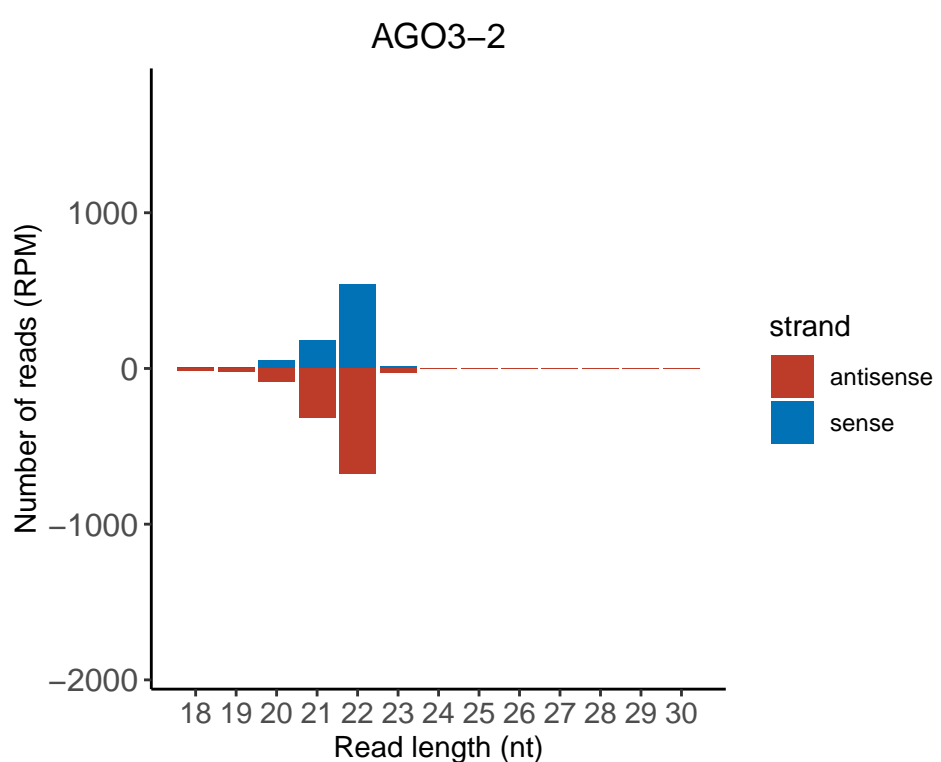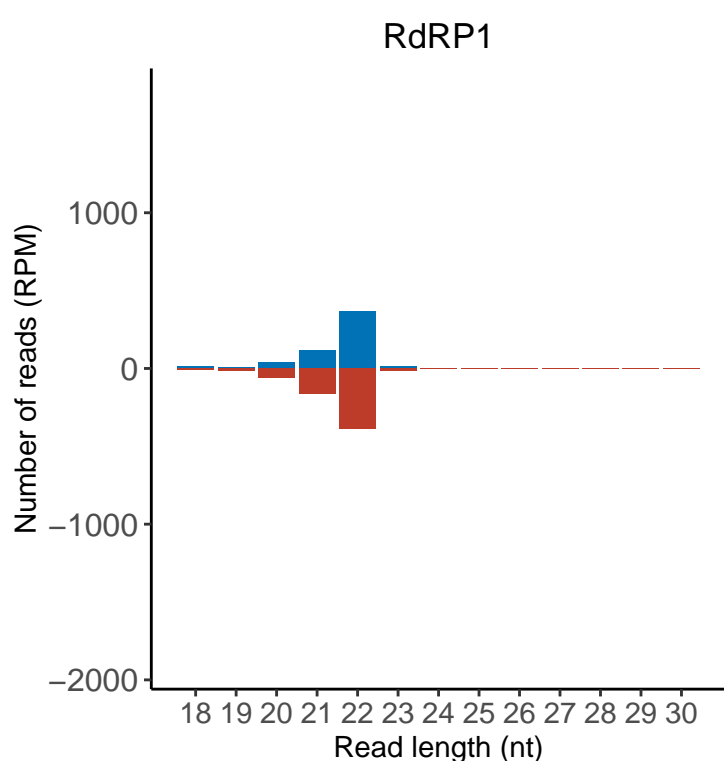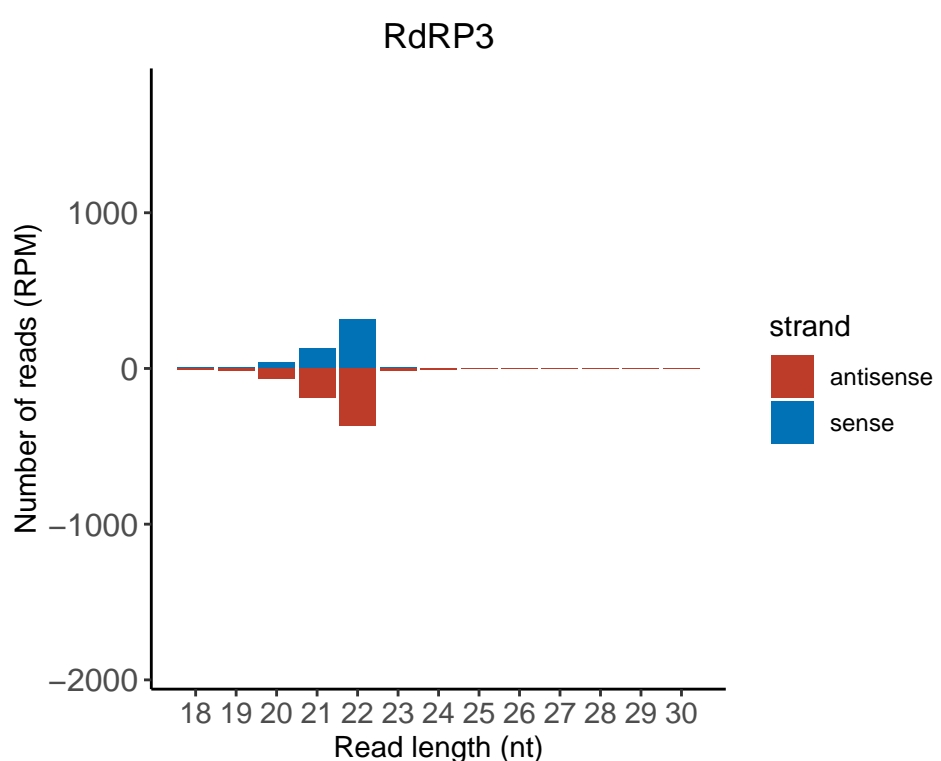

Supplement: S1 Data — On the Normalized and Relative levels pages, normalized read counts (RPM) and relative levels compared to the control (dsGFP) library were used. TEs with more than 50RPM and Coding Genes with more than 3.5RPM on average in KD libraries are included in this website. For viral sequences, reference sequences were generated by assembling sRNA sequences (See the Analysis of sRNAseq data section in Materials and methods.) and size distributions of sRNA reads mapped to each contig are shown. Reads were first grouped by their 5’ nucleotides, and reads of each length were counted. Full tables including TEs and Coding genes with fewer reads are in the “FullTable” folder. (ZIP) [file pone.0281195.s017.zip › SupplementaryData/Links/PDFs/Motif:rnd-6_family-4733TE_lengthdistribution.pdf]

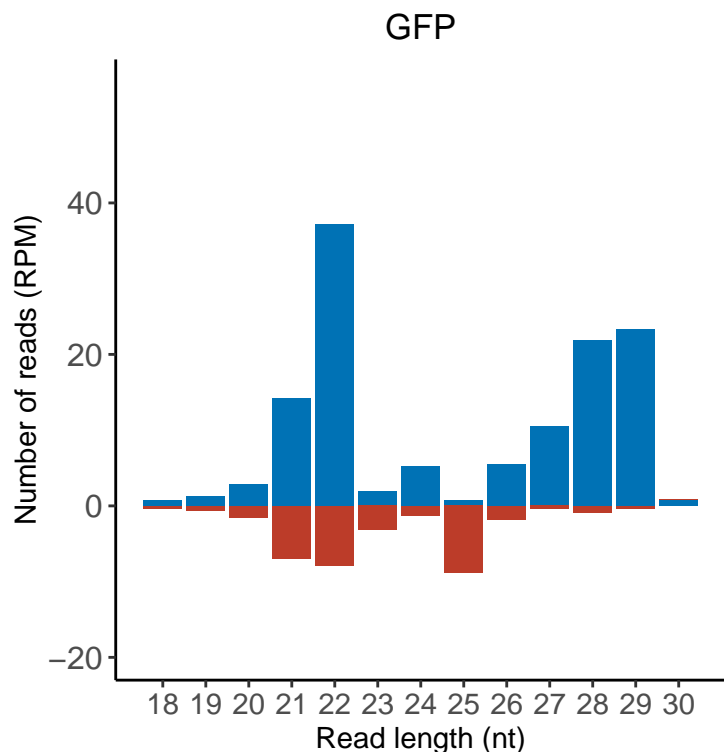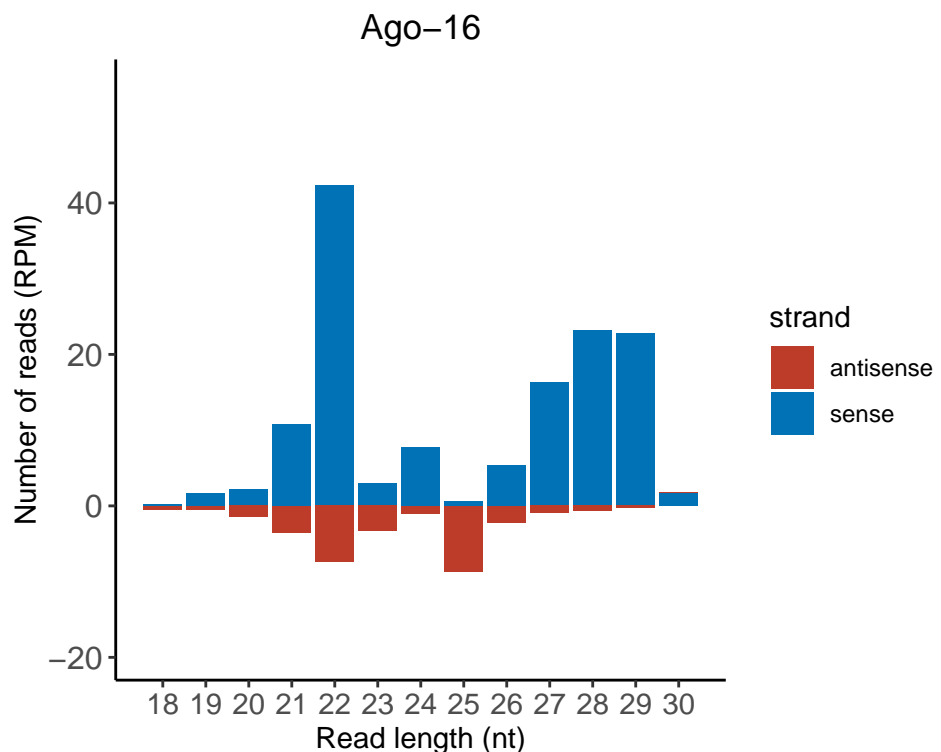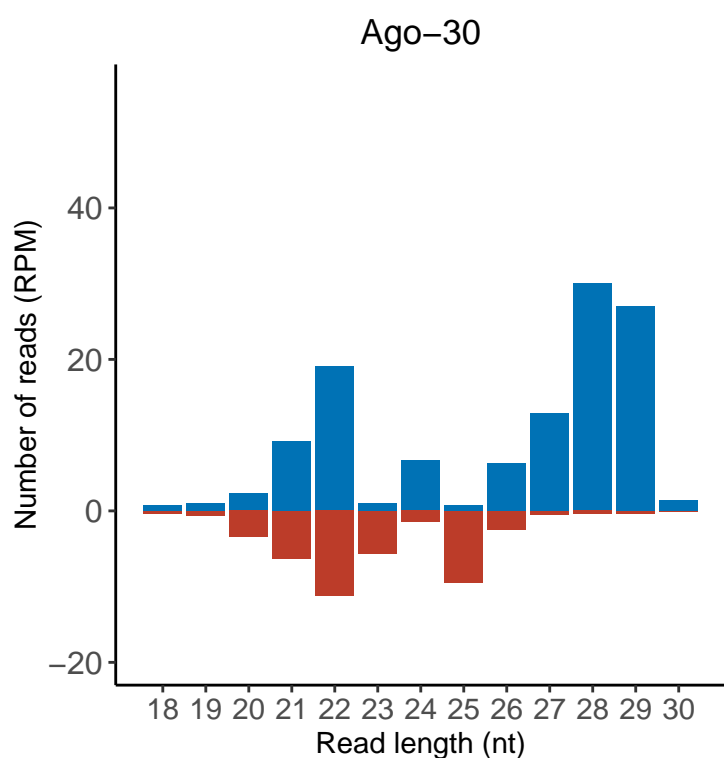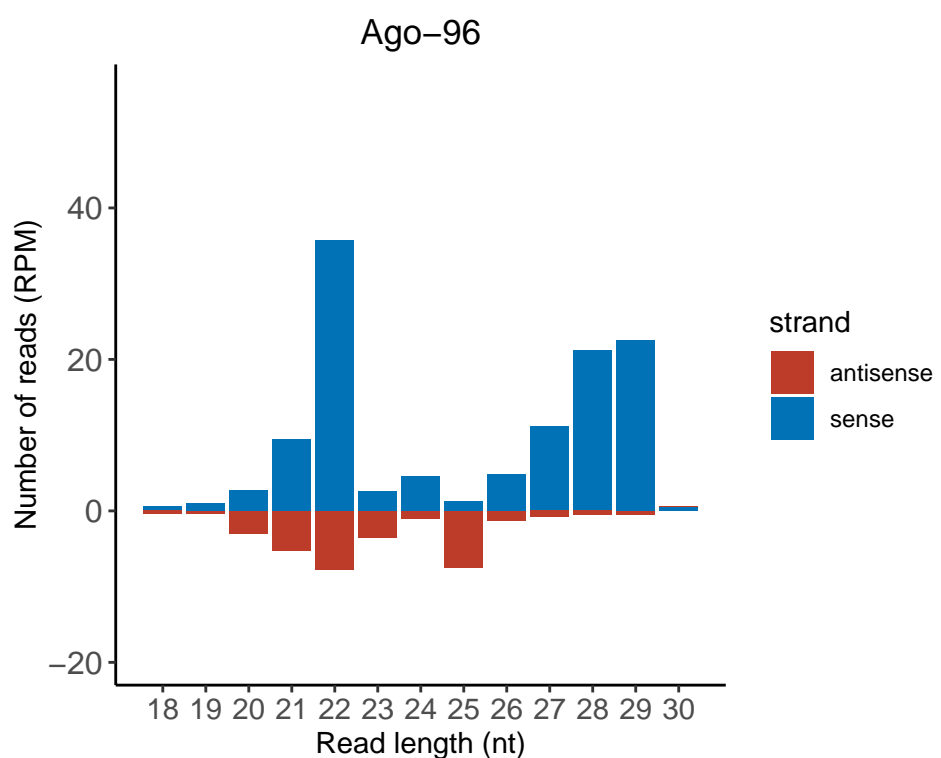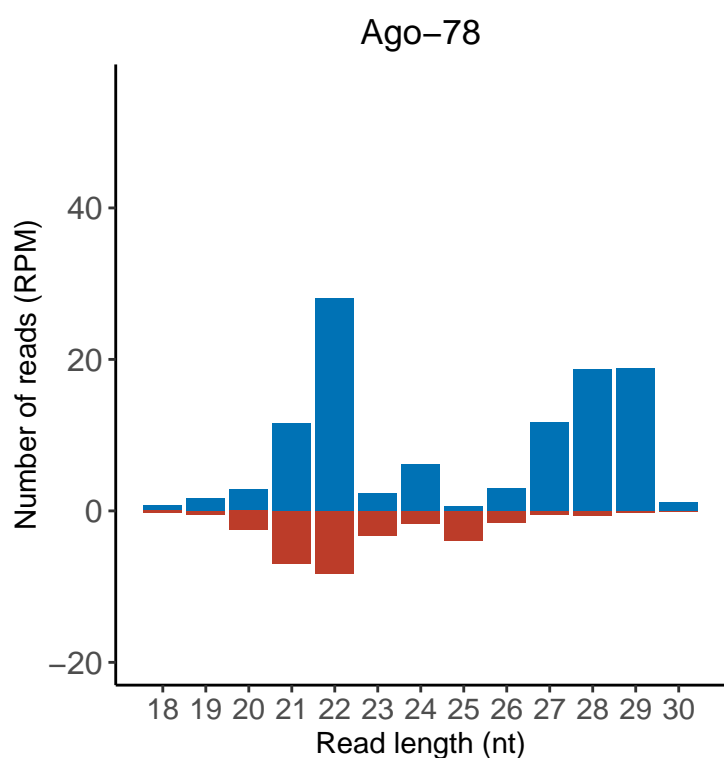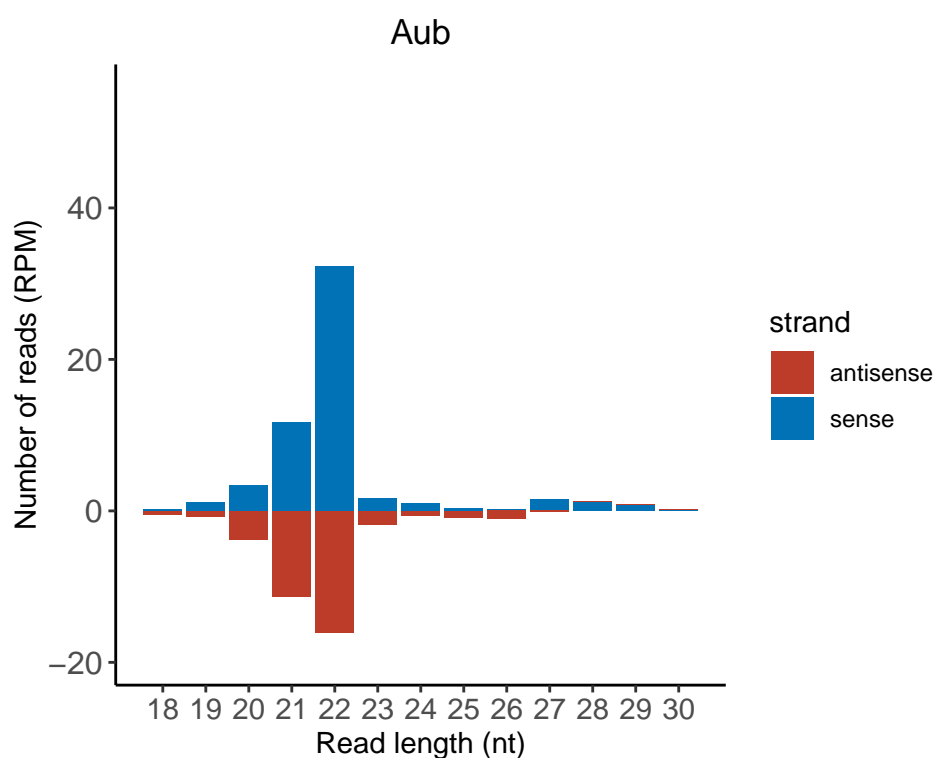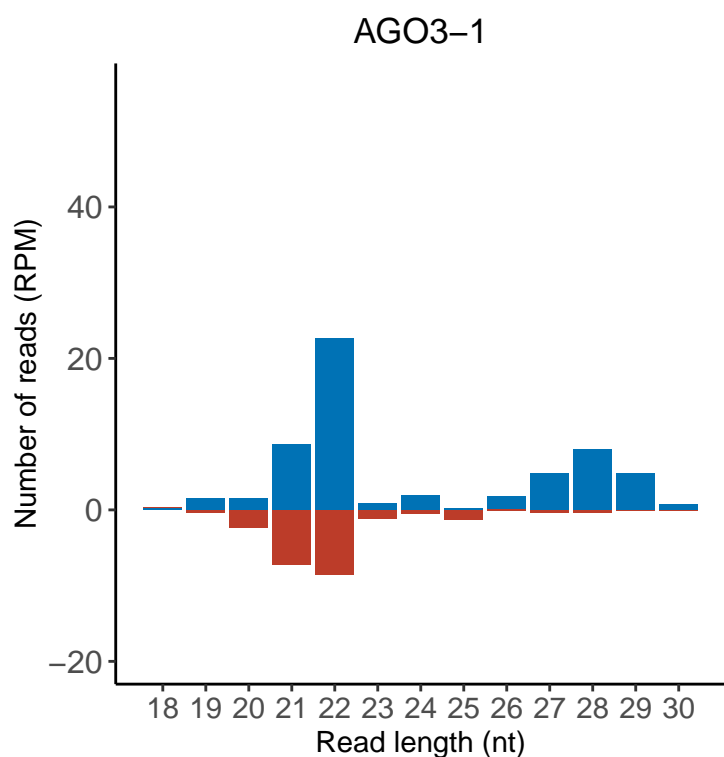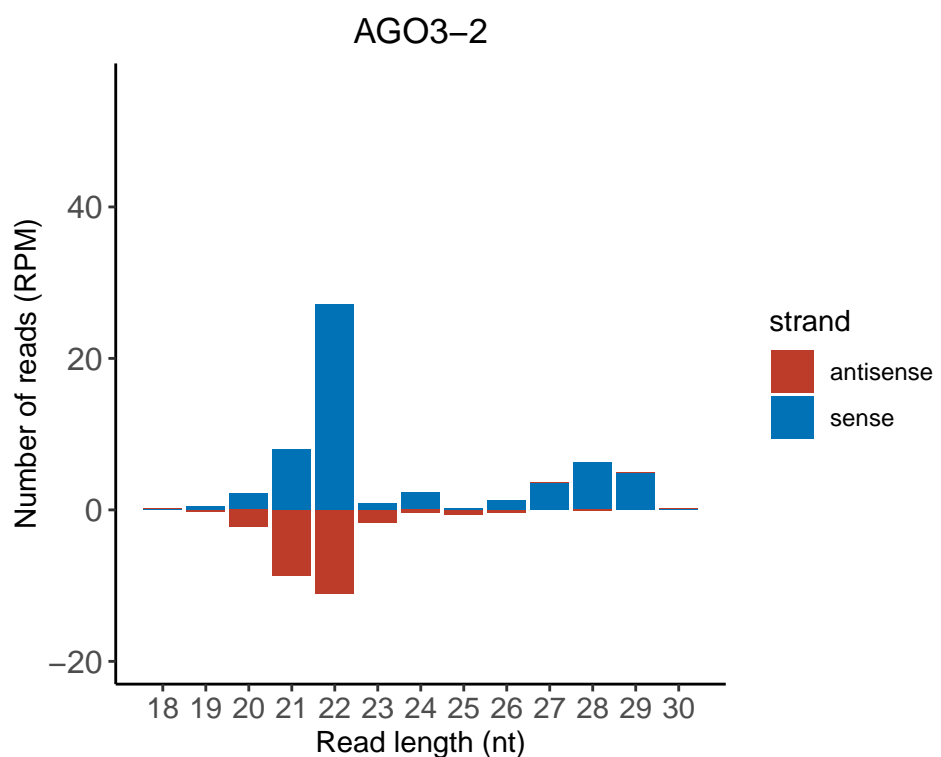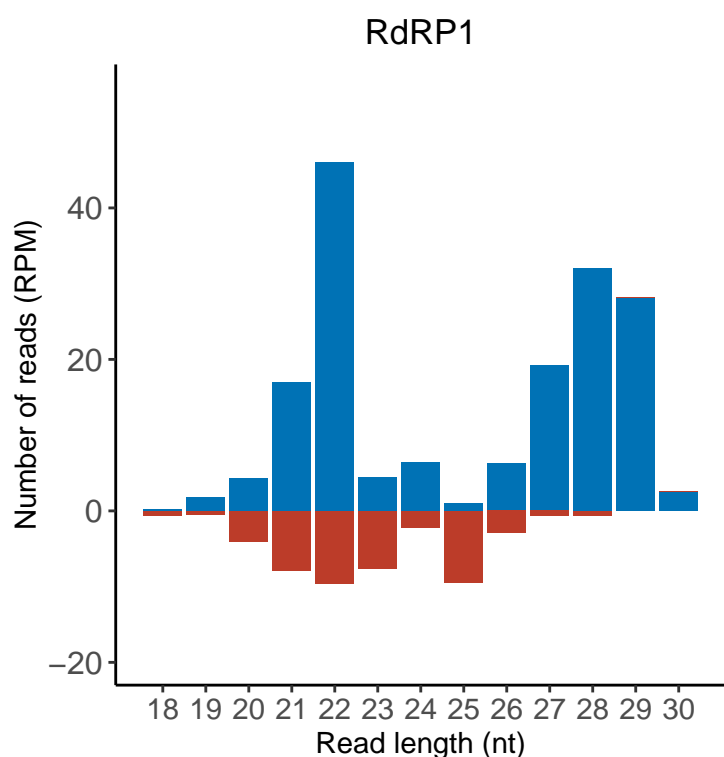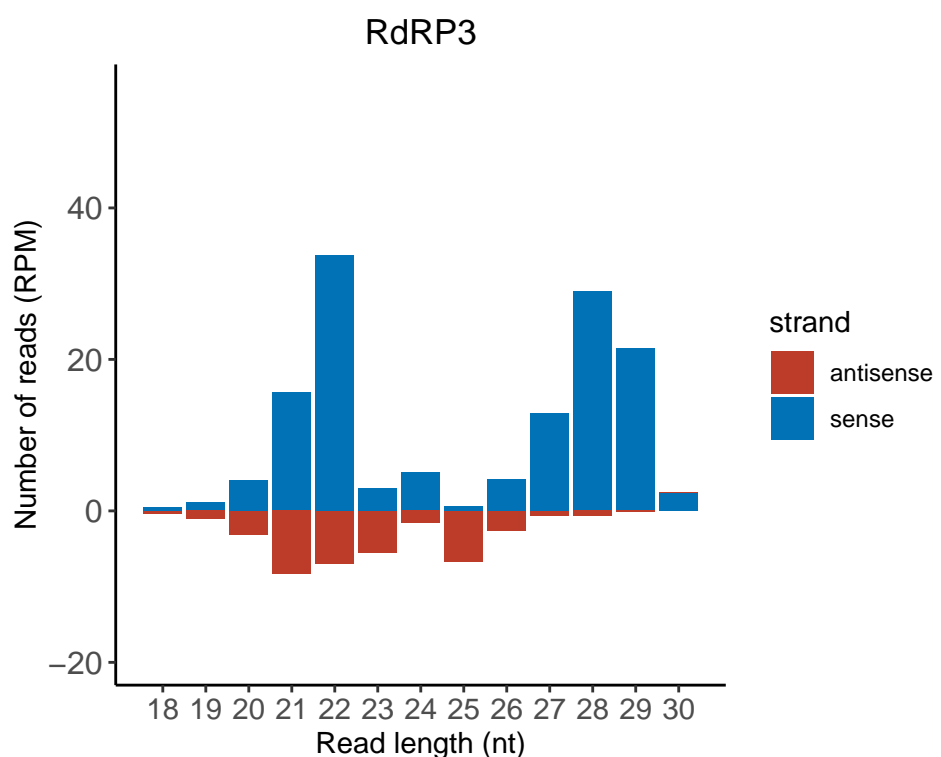

Supplement: S1 Data — On the Normalized and Relative levels pages, normalized read counts (RPM) and relative levels compared to the control (dsGFP) library were used. TEs with more than 50RPM and Coding Genes with more than 3.5RPM on average in KD libraries are included in this website. For viral sequences, reference sequences were generated by assembling sRNA sequences (See the Analysis of sRNAseq data section in Materials and methods.) and size distributions of sRNA reads mapped to each contig are shown. Reads were first grouped by their 5’ nucleotides, and reads of each length were counted. Full tables including TEs and Coding genes with fewer reads are in the “FullTable” folder. (ZIP) [file pone.0281195.s017.zip › SupplementaryData/Links/PDFs/Motif:rnd-6_family-5159TE_lengthdistribution.pdf]

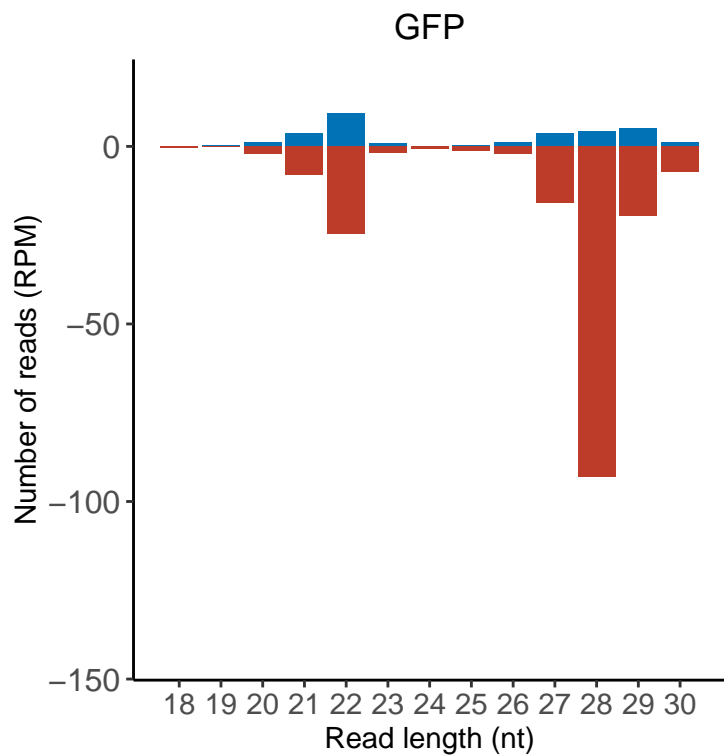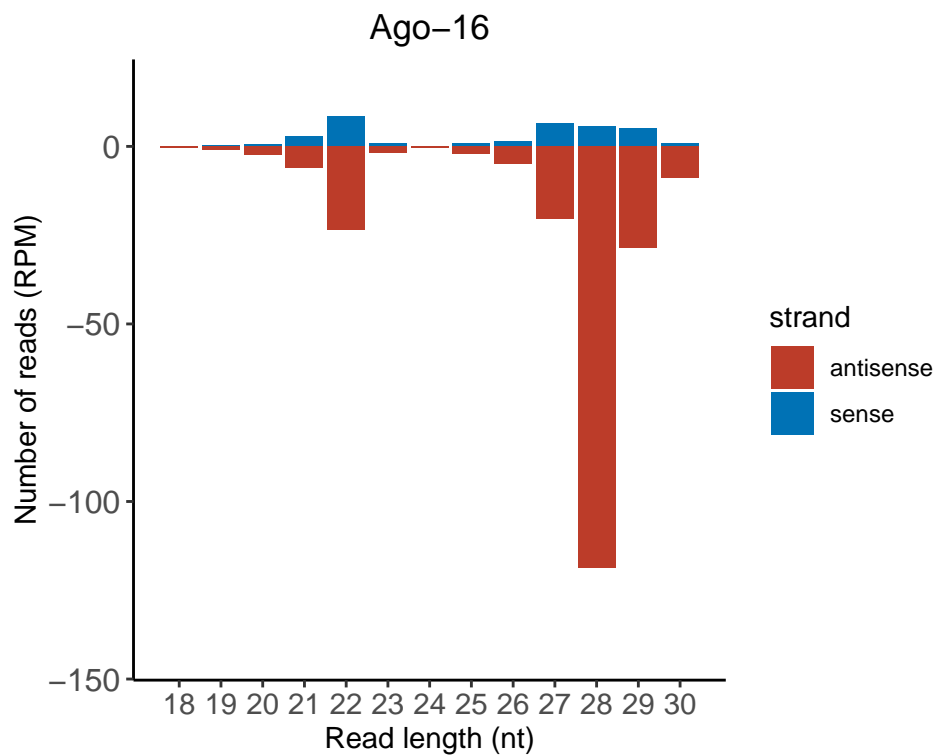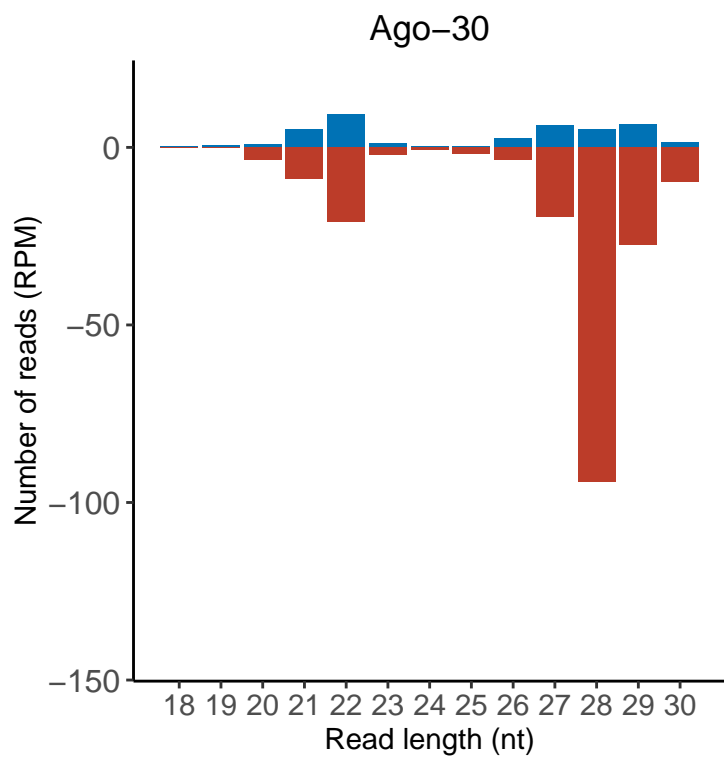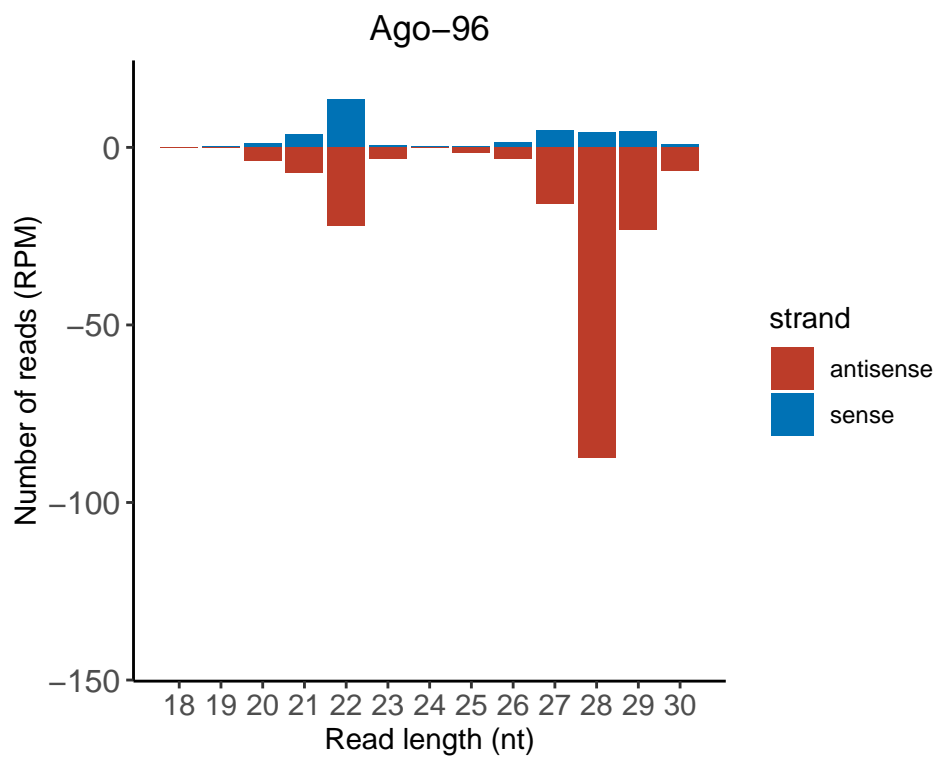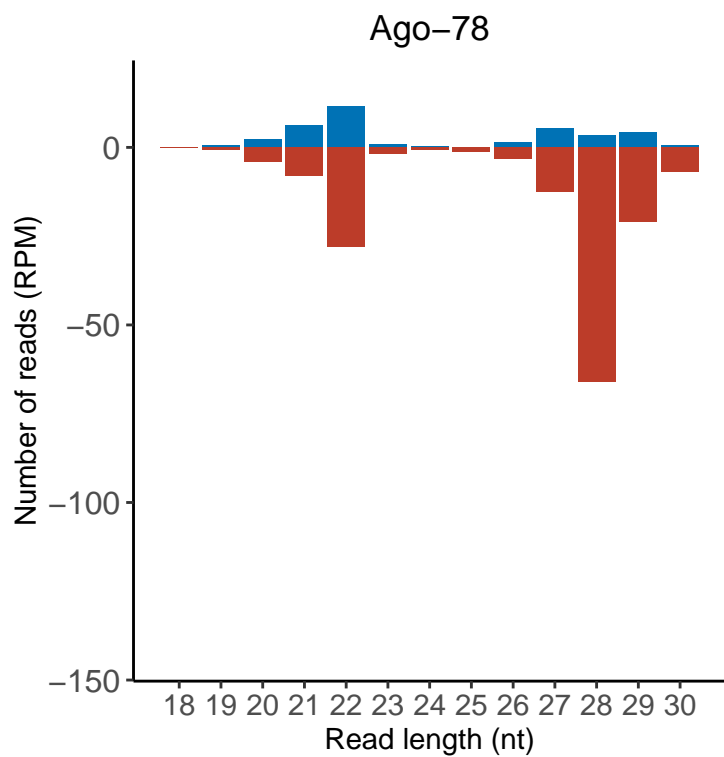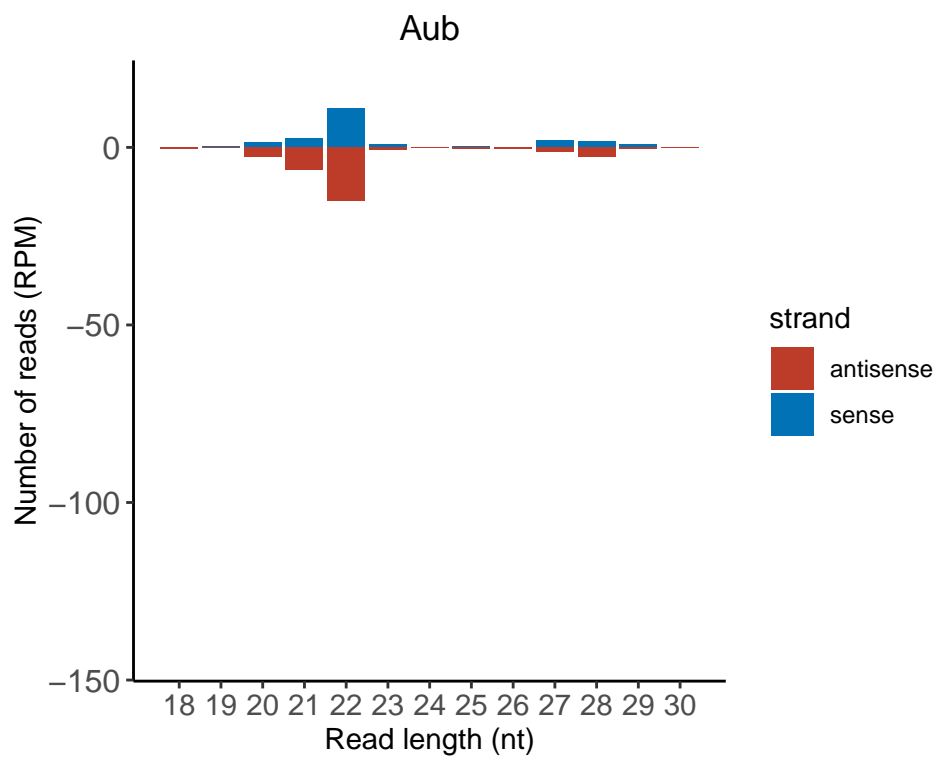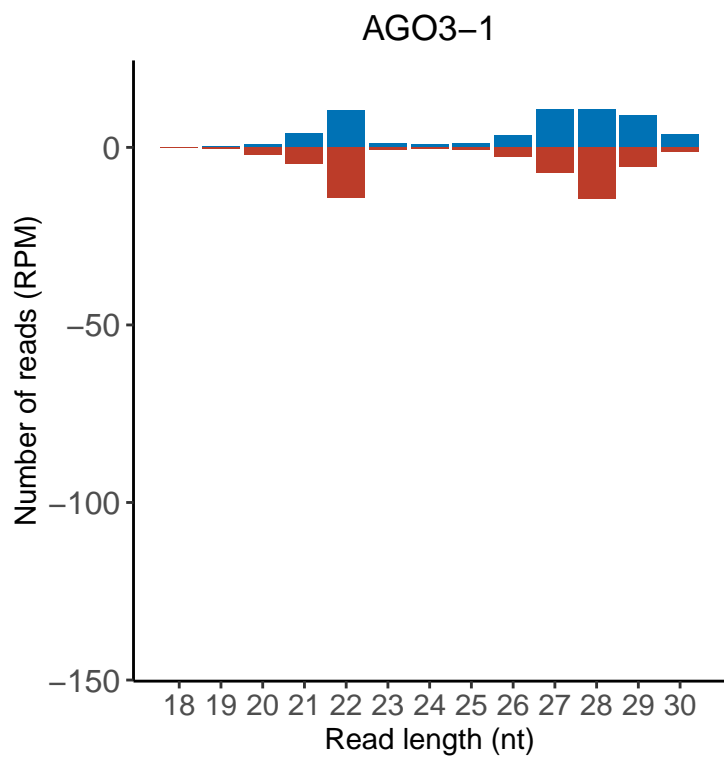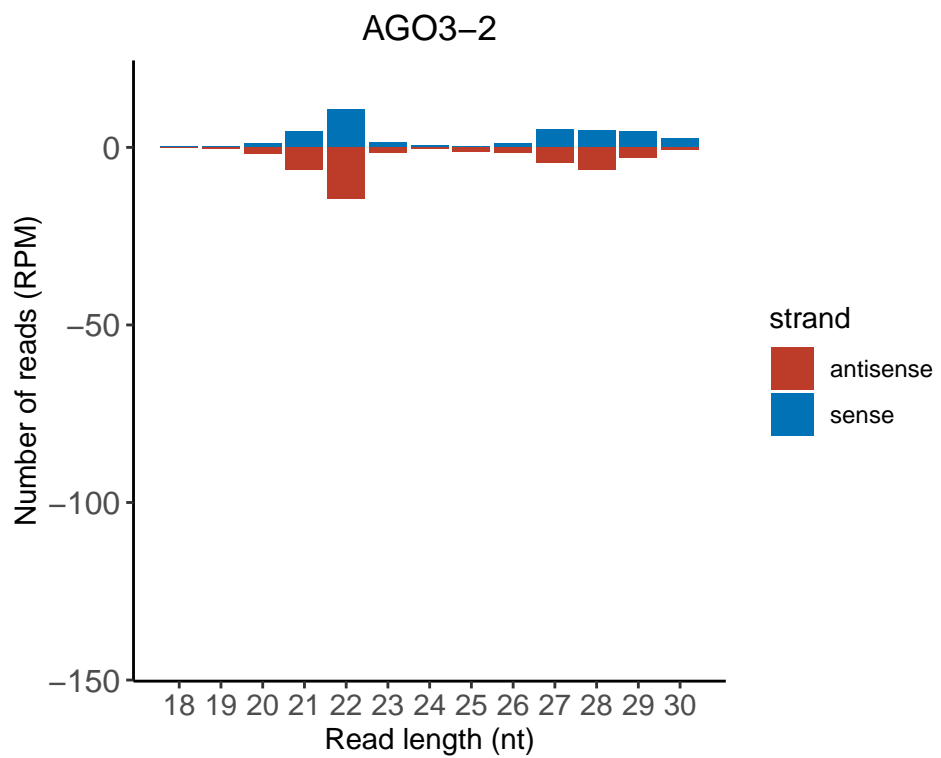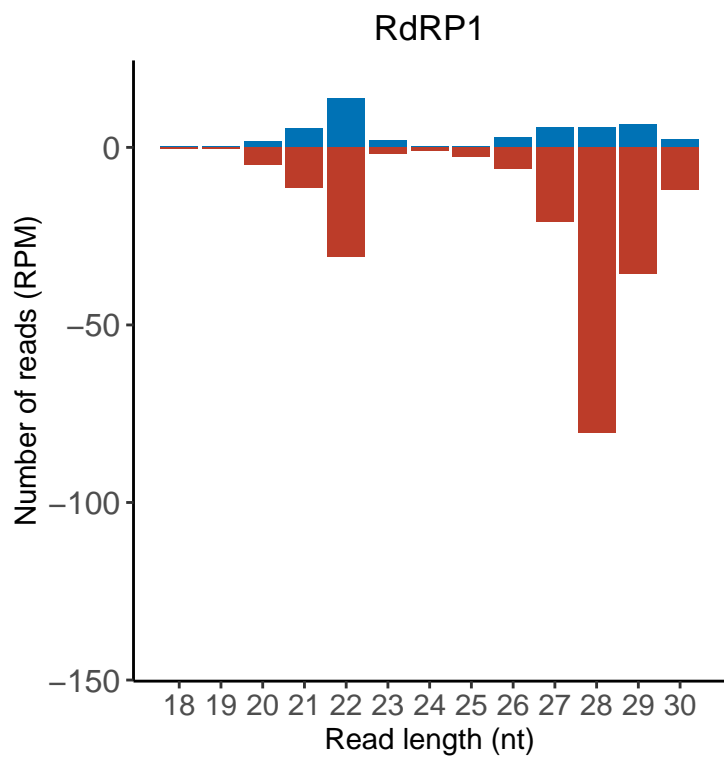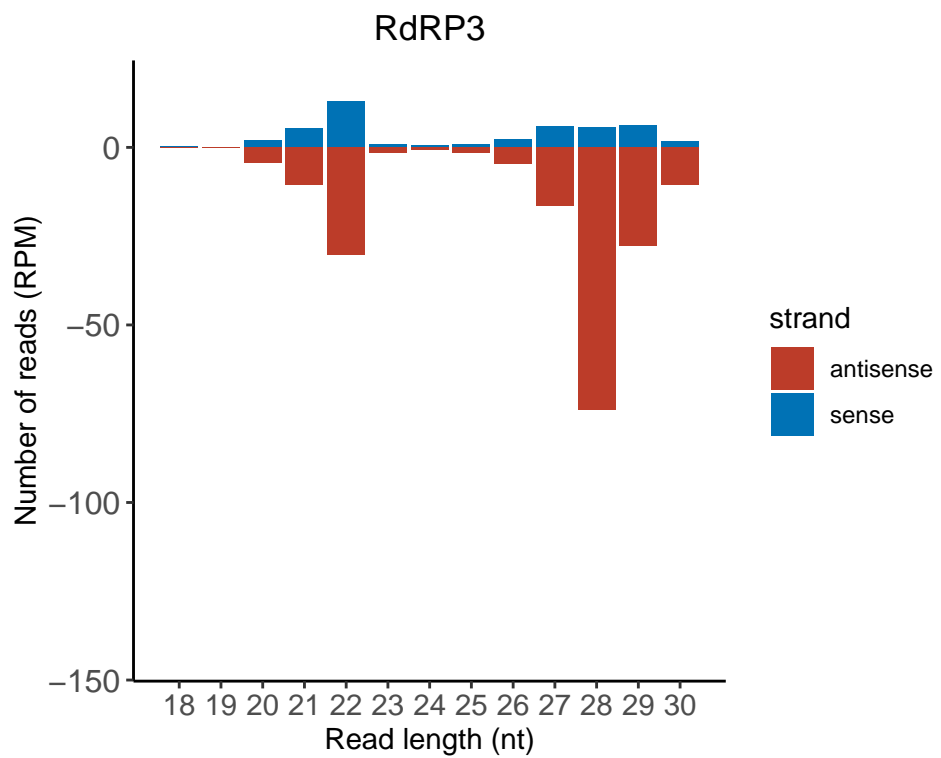

Supplement: S1 Data — On the Normalized and Relative levels pages, normalized read counts (RPM) and relative levels compared to the control (dsGFP) library were used. TEs with more than 50RPM and Coding Genes with more than 3.5RPM on average in KD libraries are included in this website. For viral sequences, reference sequences were generated by assembling sRNA sequences (See the Analysis of sRNAseq data section in Materials and methods.) and size distributions of sRNA reads mapped to each contig are shown. Reads were first grouped by their 5’ nucleotides, and reads of each length were counted. Full tables including TEs and Coding genes with fewer reads are in the “FullTable” folder. (ZIP) [file pone.0281195.s017.zip › SupplementaryData/Links/PDFs/Motif:rnd-1_family-270TE_lengthdistribution.pdf]

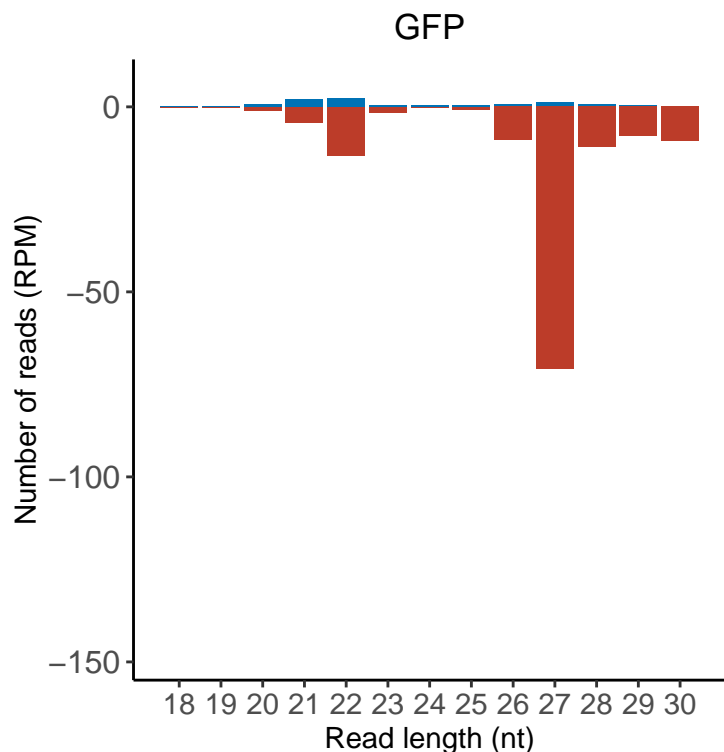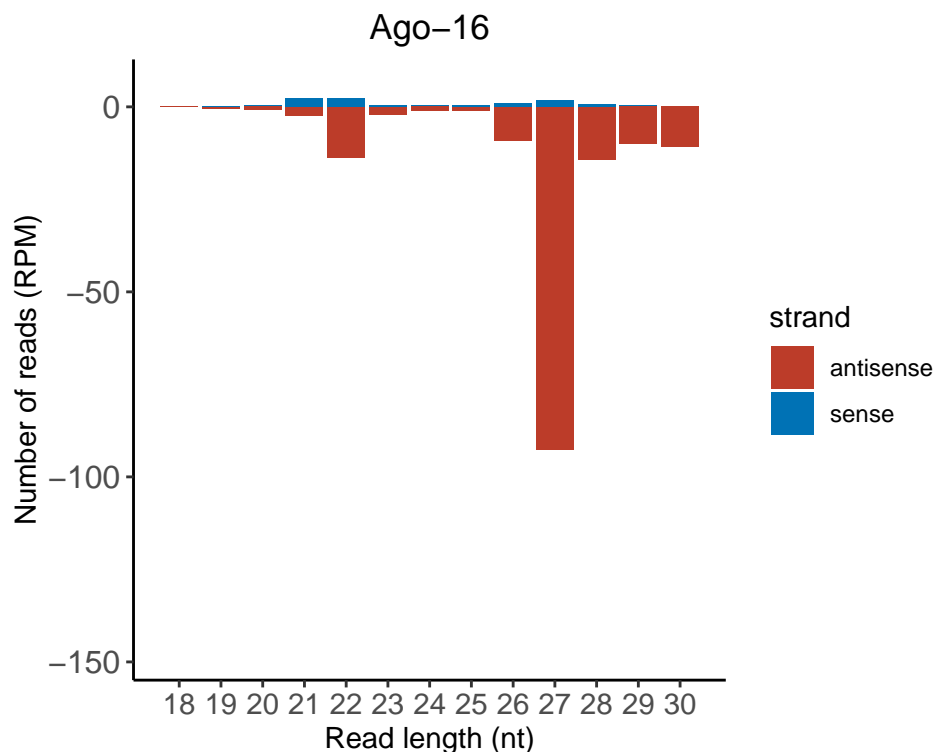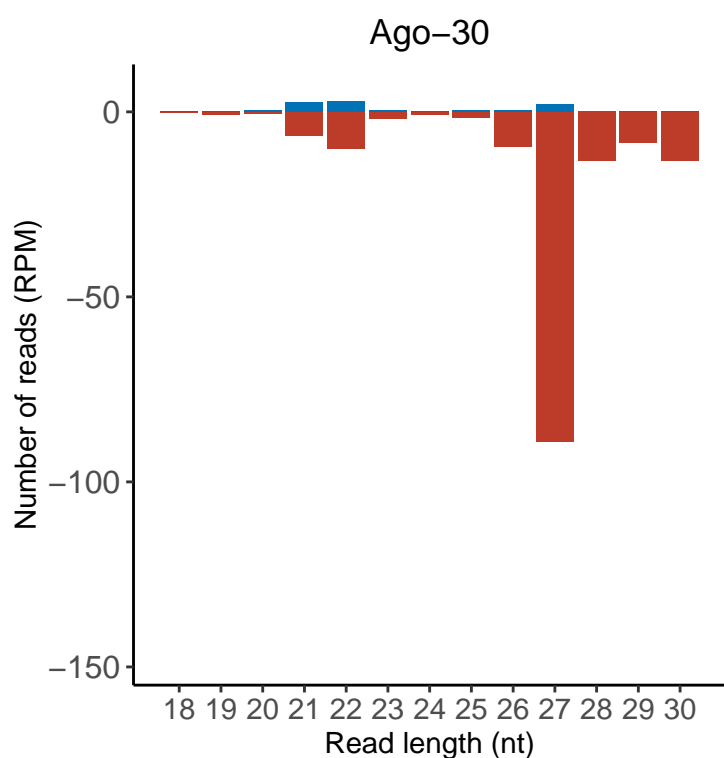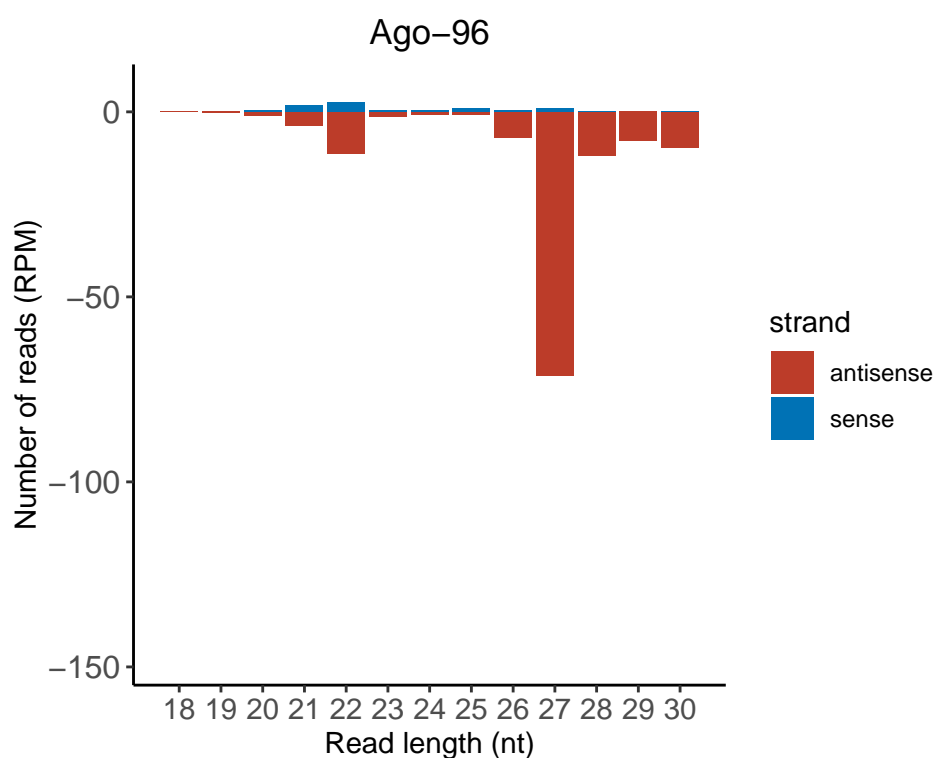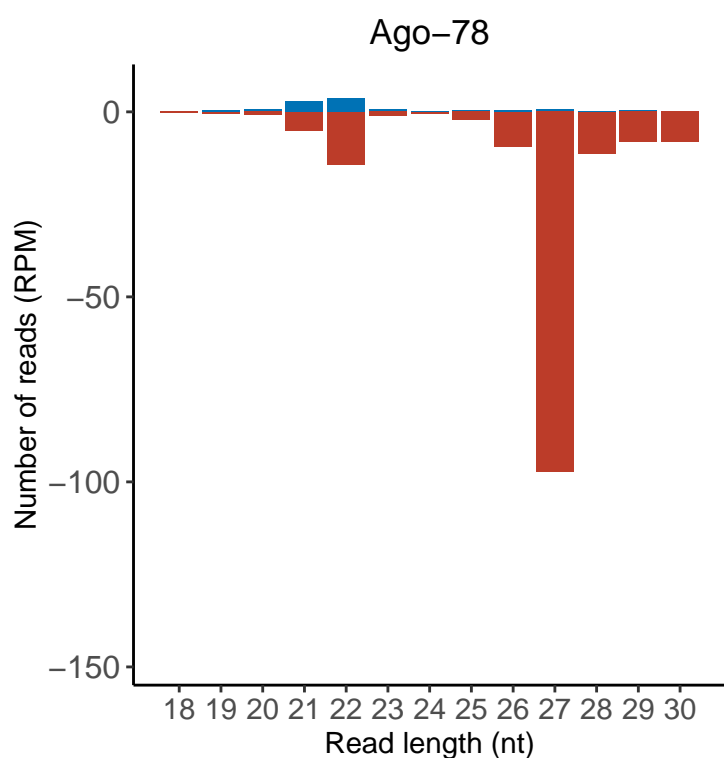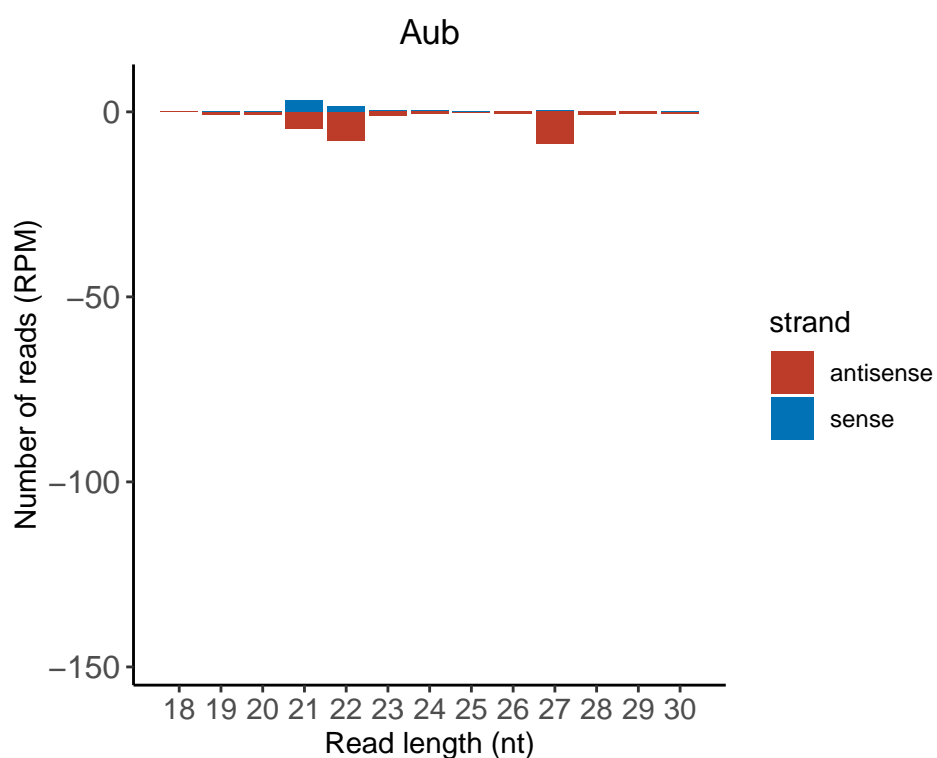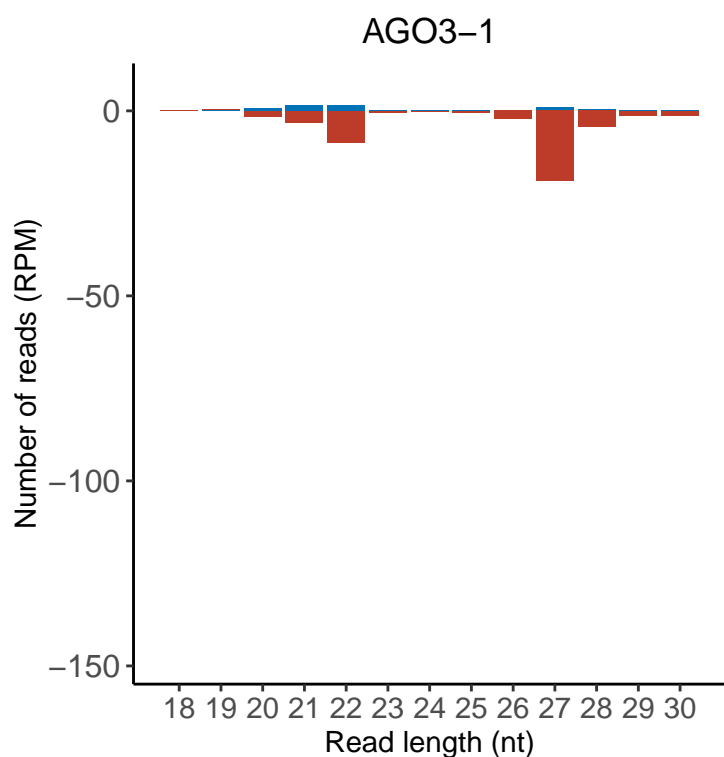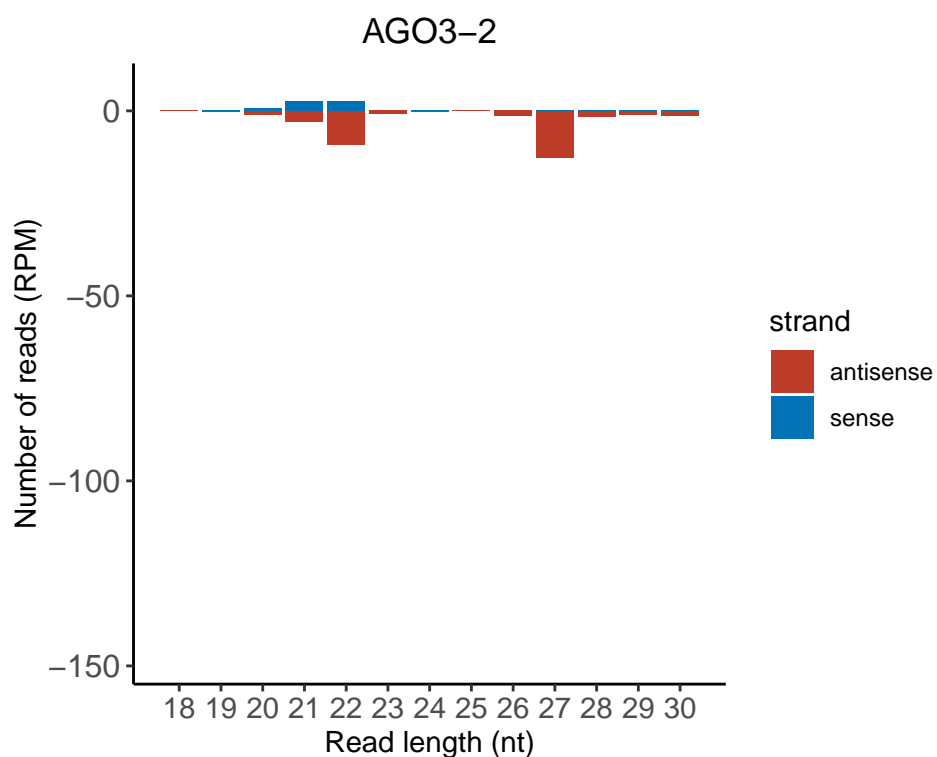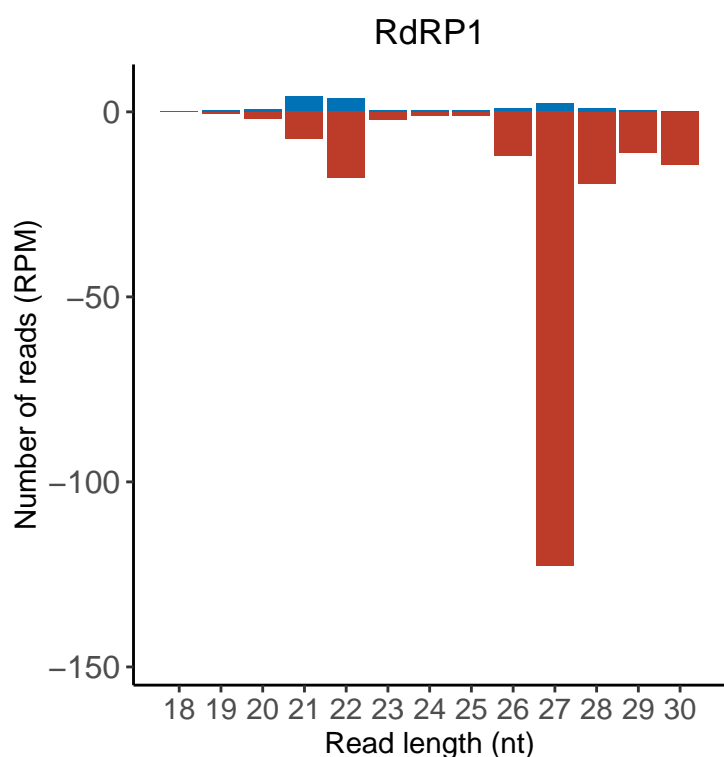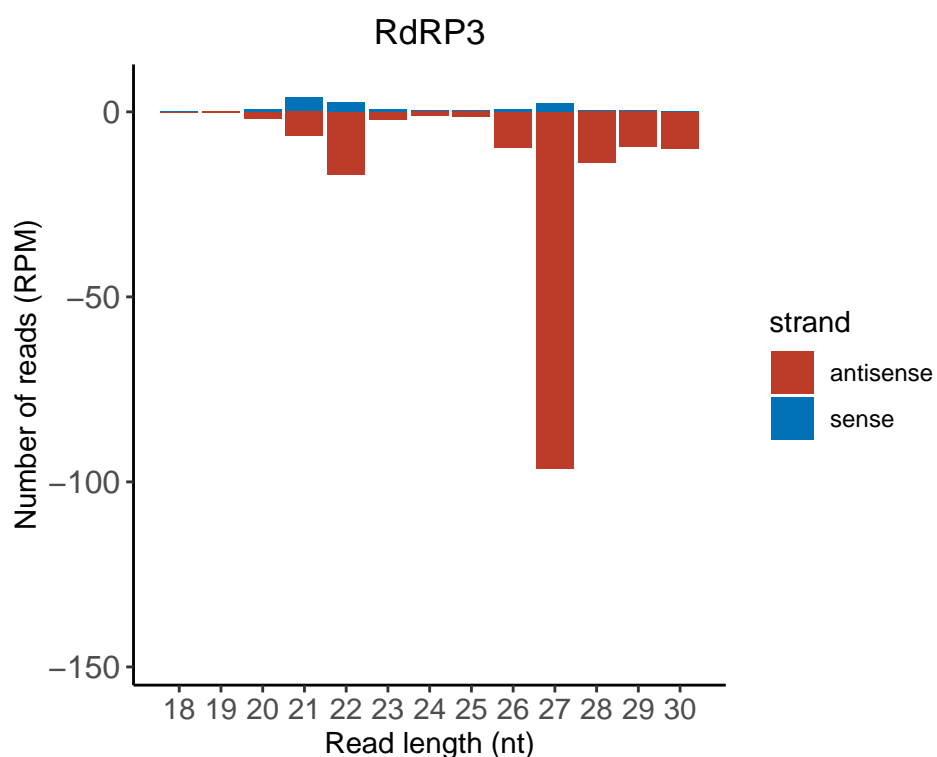

Supplement: S1 Data — On the Normalized and Relative levels pages, normalized read counts (RPM) and relative levels compared to the control (dsGFP) library were used. TEs with more than 50RPM and Coding Genes with more than 3.5RPM on average in KD libraries are included in this website. For viral sequences, reference sequences were generated by assembling sRNA sequences (See the Analysis of sRNAseq data section in Materials and methods.) and size distributions of sRNA reads mapped to each contig are shown. Reads were first grouped by their 5’ nucleotides, and reads of each length were counted. Full tables including TEs and Coding genes with fewer reads are in the “FullTable” folder. (ZIP) [file pone.0281195.s017.zip › SupplementaryData/Links/PDFs/Motif:rnd-1_family-832TE_lengthdistribution.pdf]

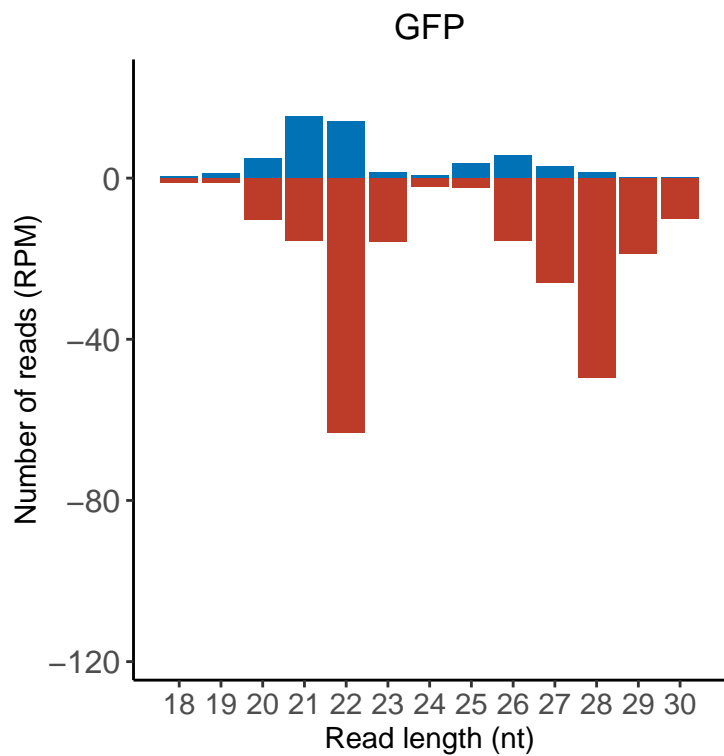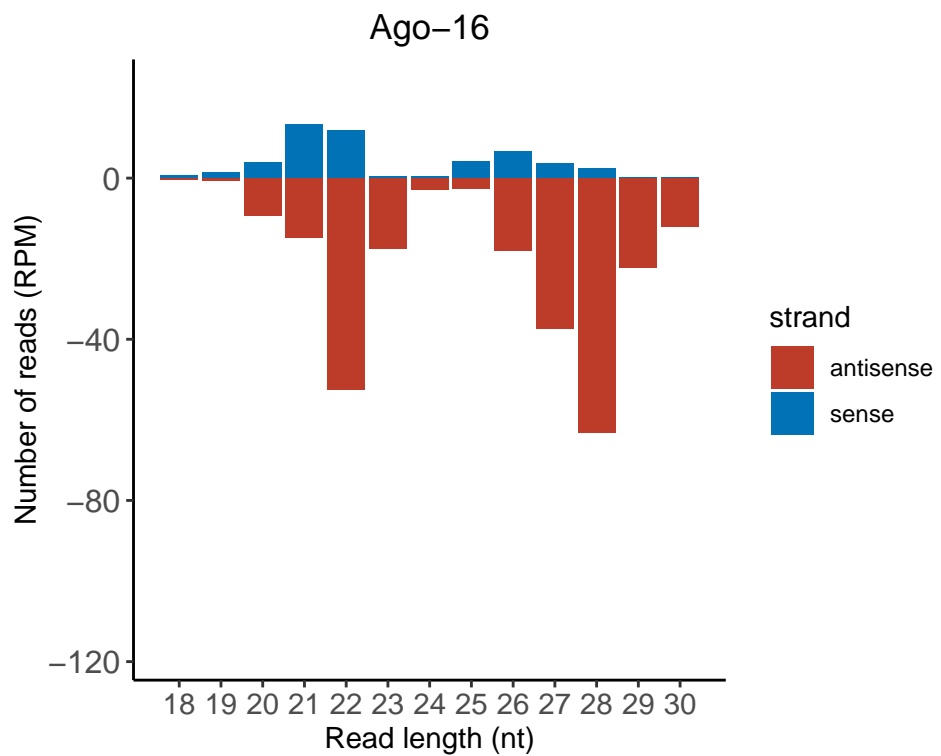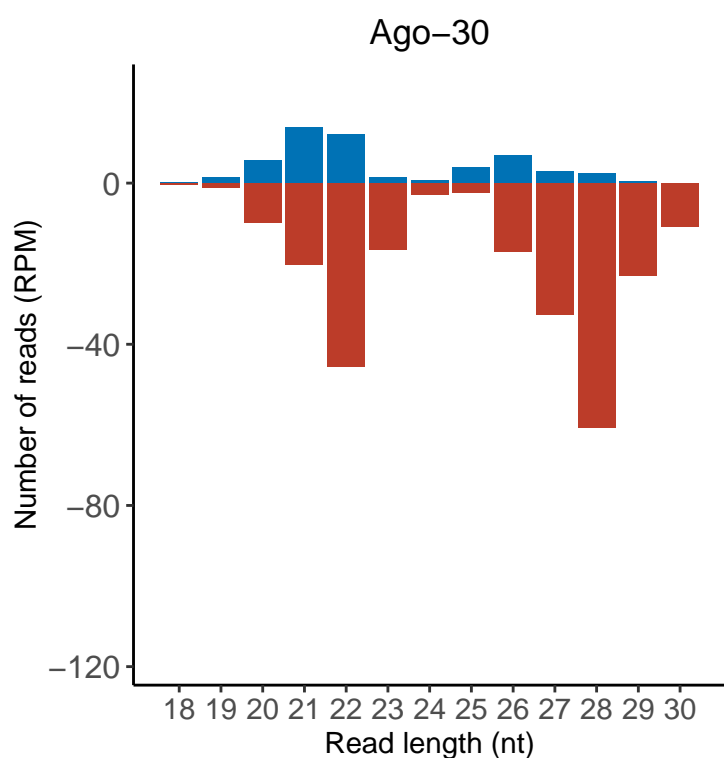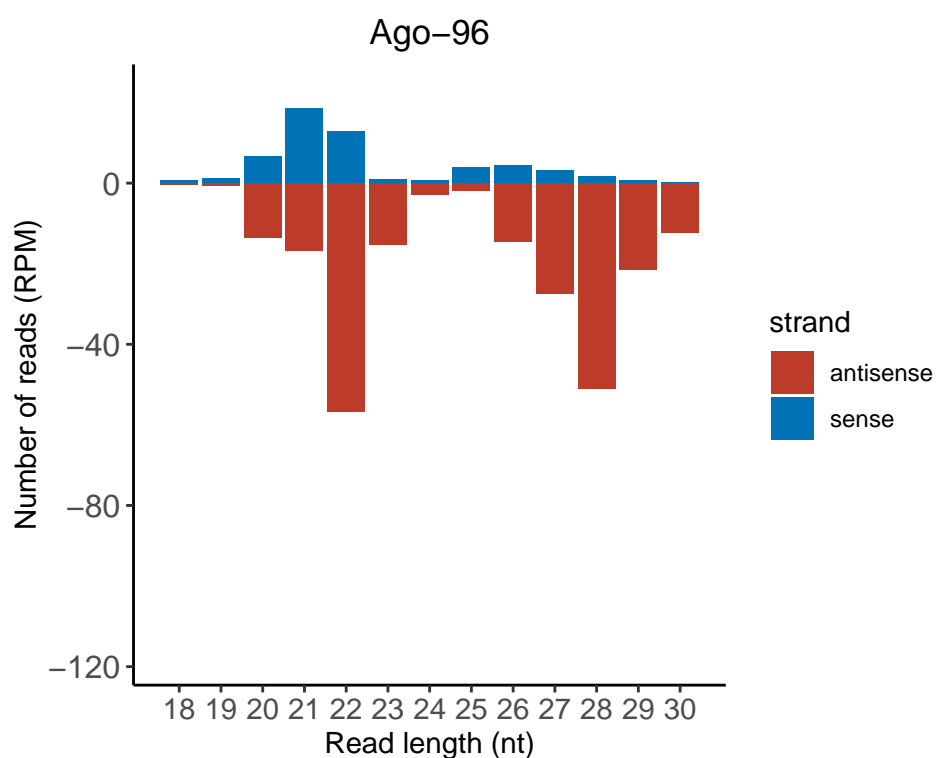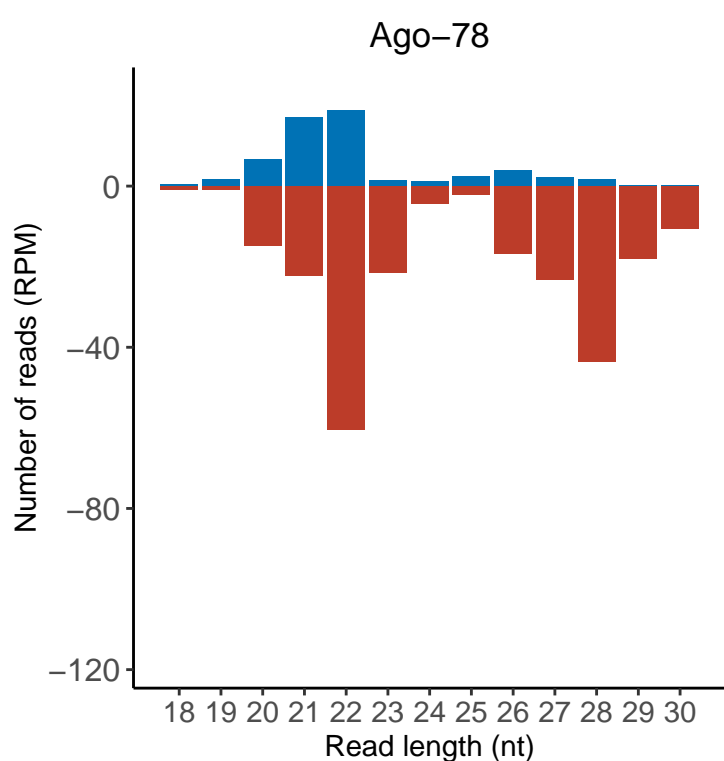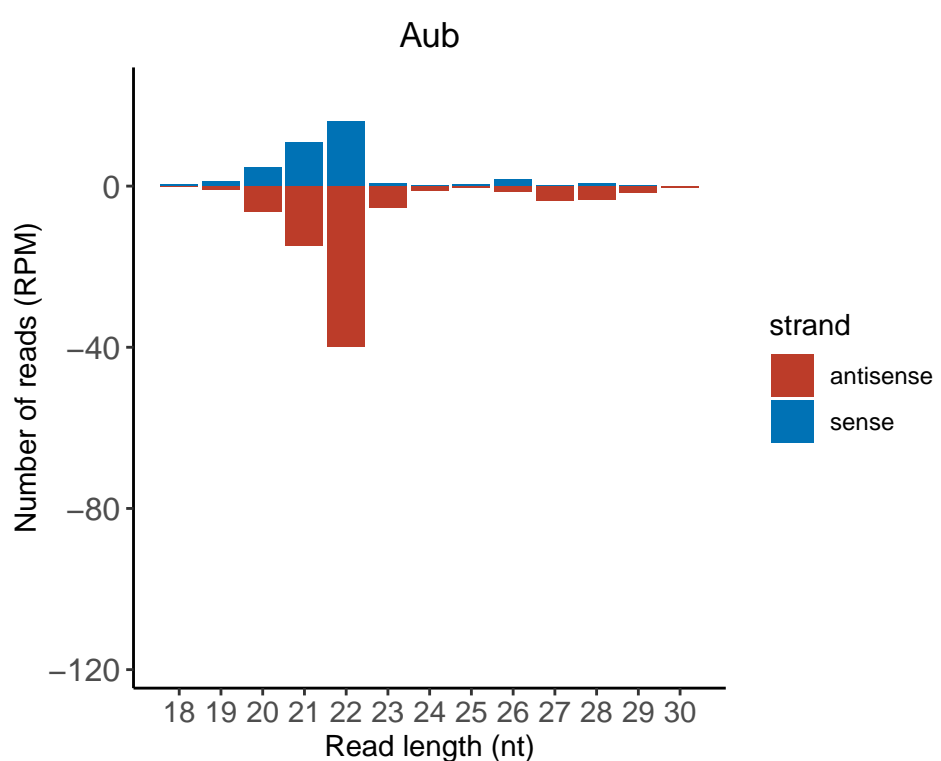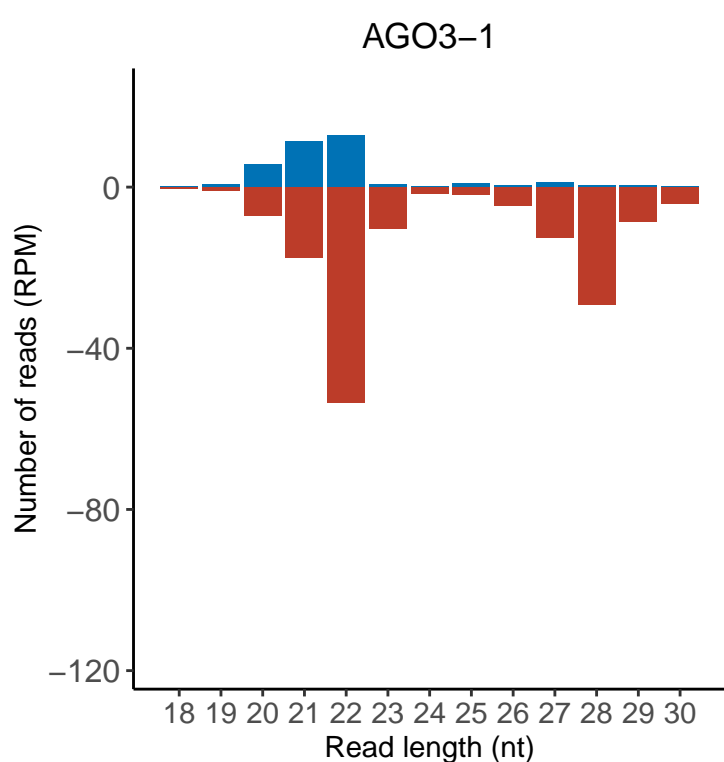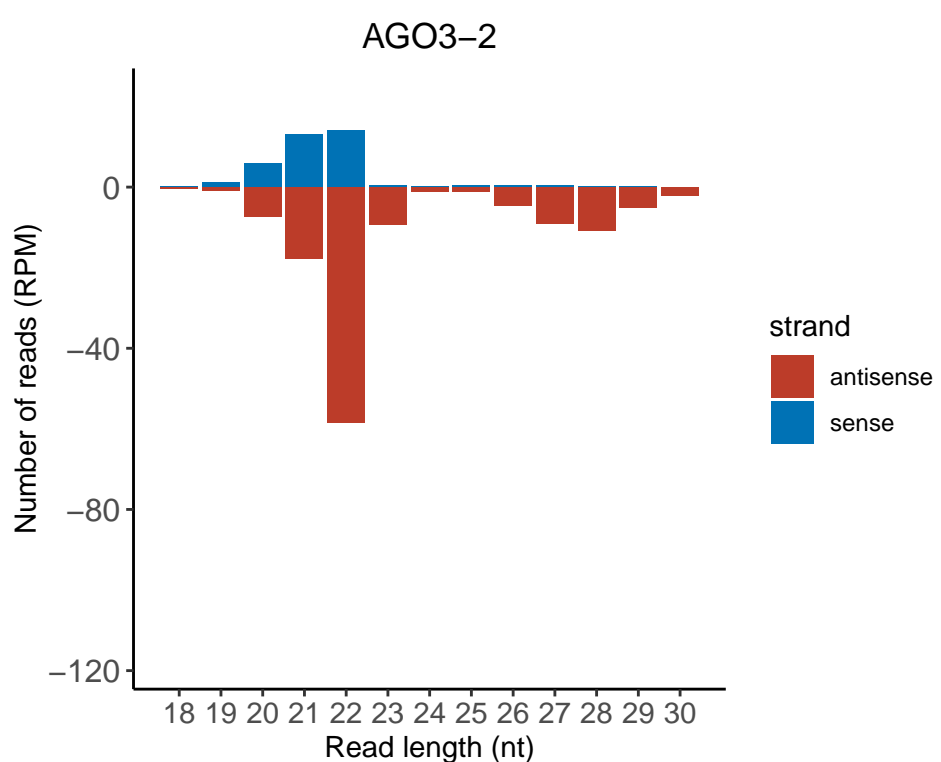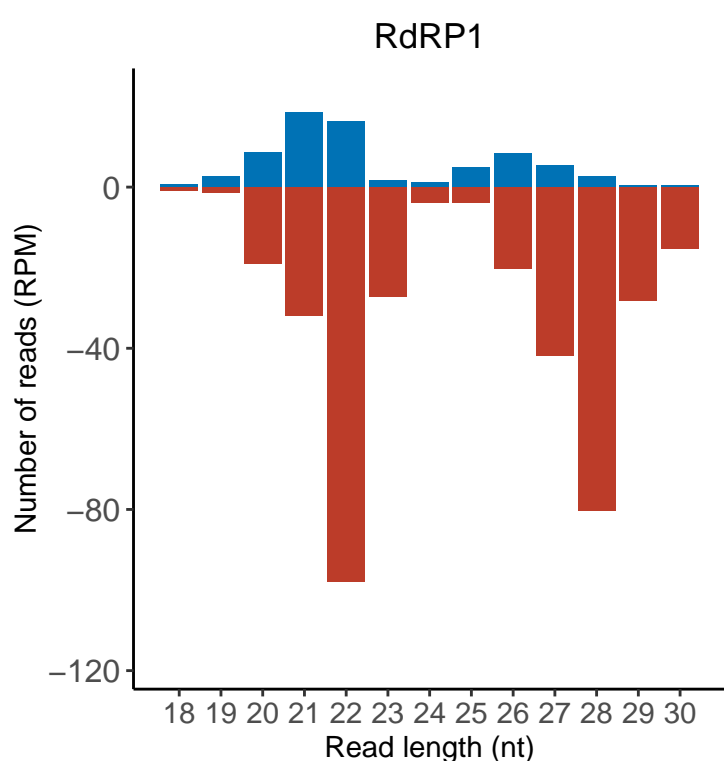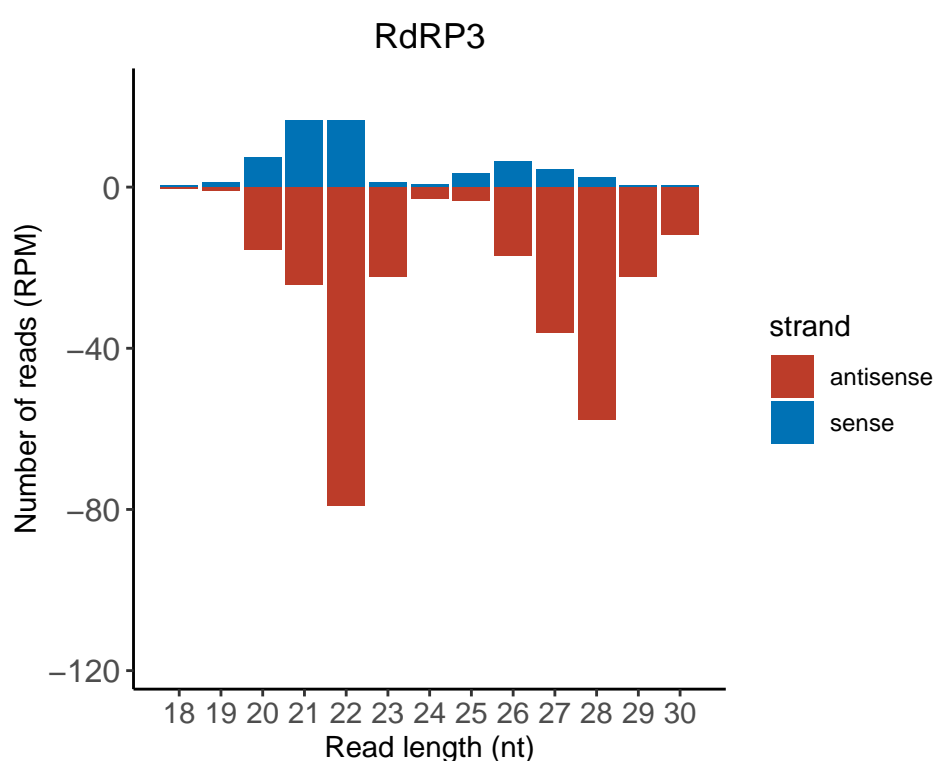

Supplement: S1 Data — On the Normalized and Relative levels pages, normalized read counts (RPM) and relative levels compared to the control (dsGFP) library were used. TEs with more than 50RPM and Coding Genes with more than 3.5RPM on average in KD libraries are included in this website. For viral sequences, reference sequences were generated by assembling sRNA sequences (See the Analysis of sRNAseq data section in Materials and methods.) and size distributions of sRNA reads mapped to each contig are shown. Reads were first grouped by their 5’ nucleotides, and reads of each length were counted. Full tables including TEs and Coding genes with fewer reads are in the “FullTable” folder. (ZIP) [file pone.0281195.s017.zip › SupplementaryData/Links/PDFs/Motif:rnd-6_family-4780TE_lengthdistribution.pdf]

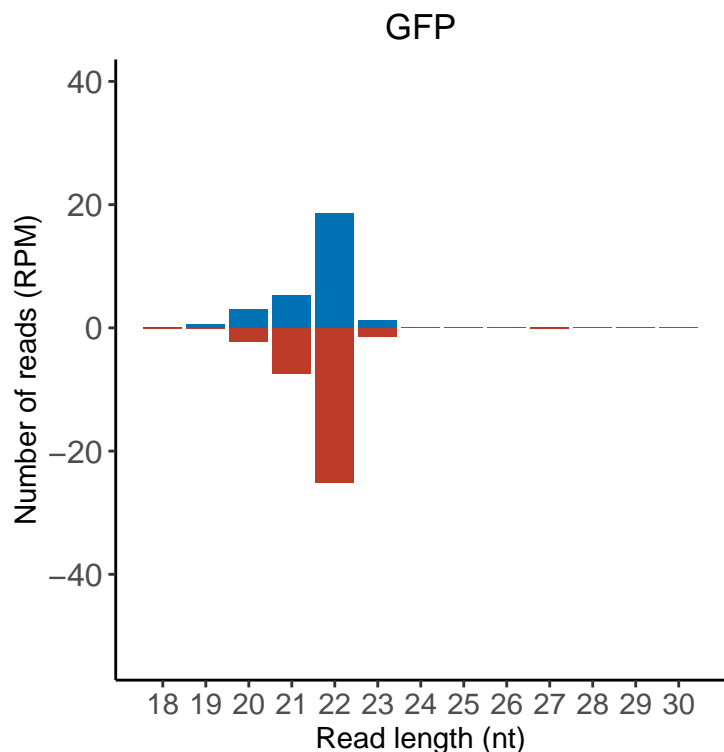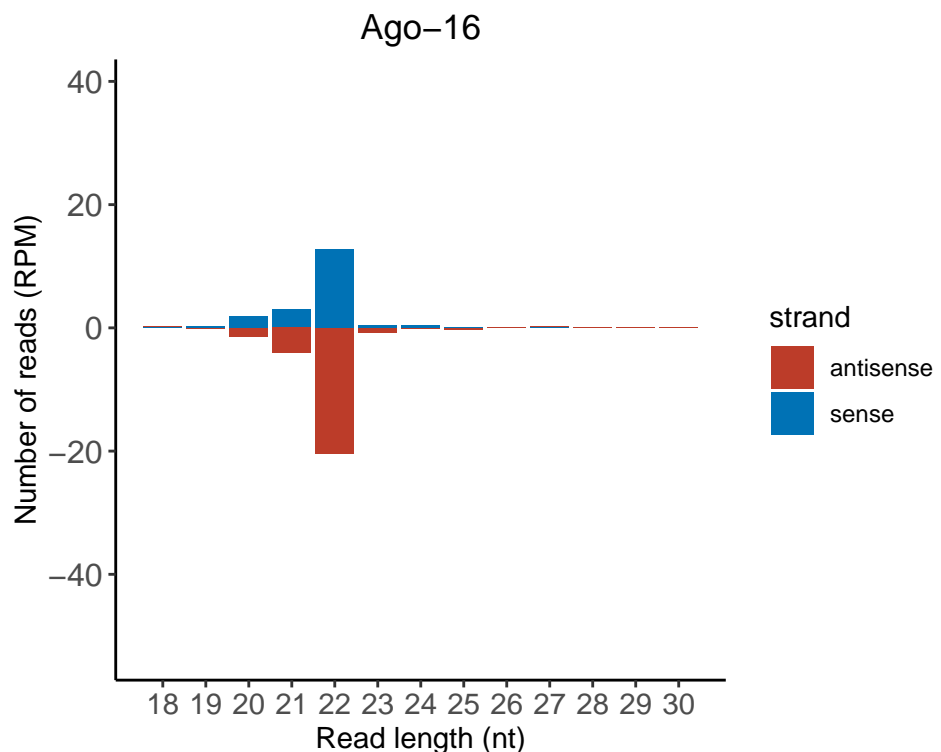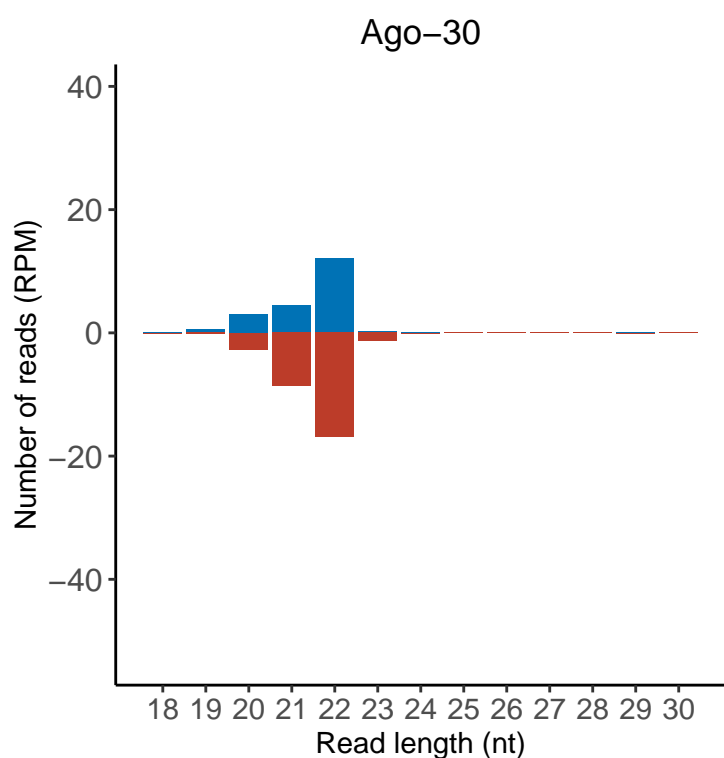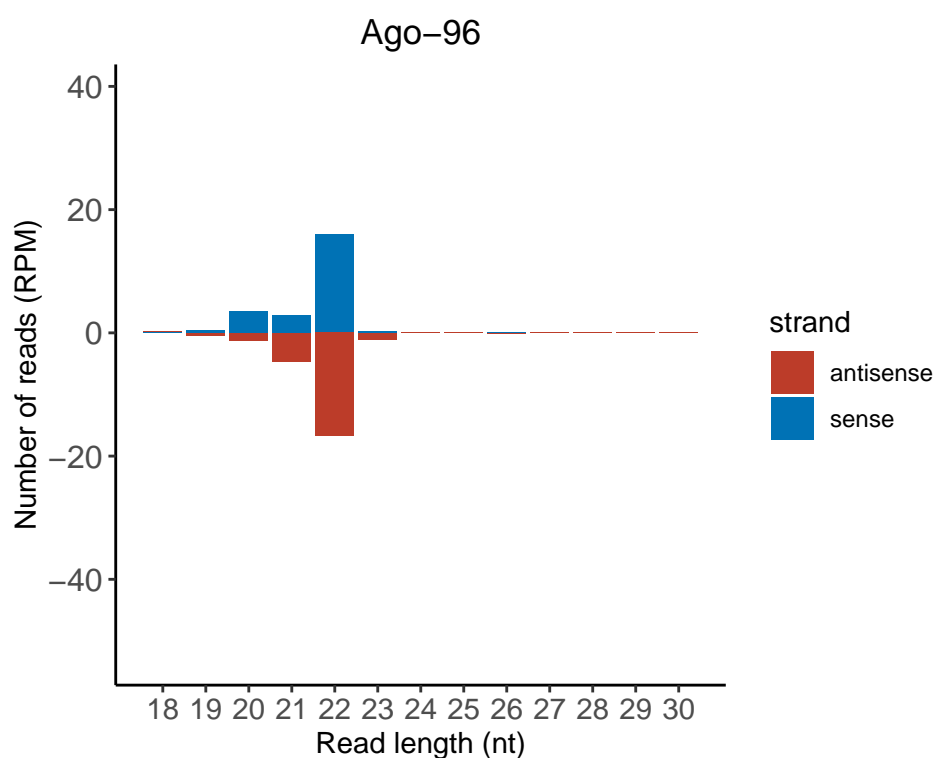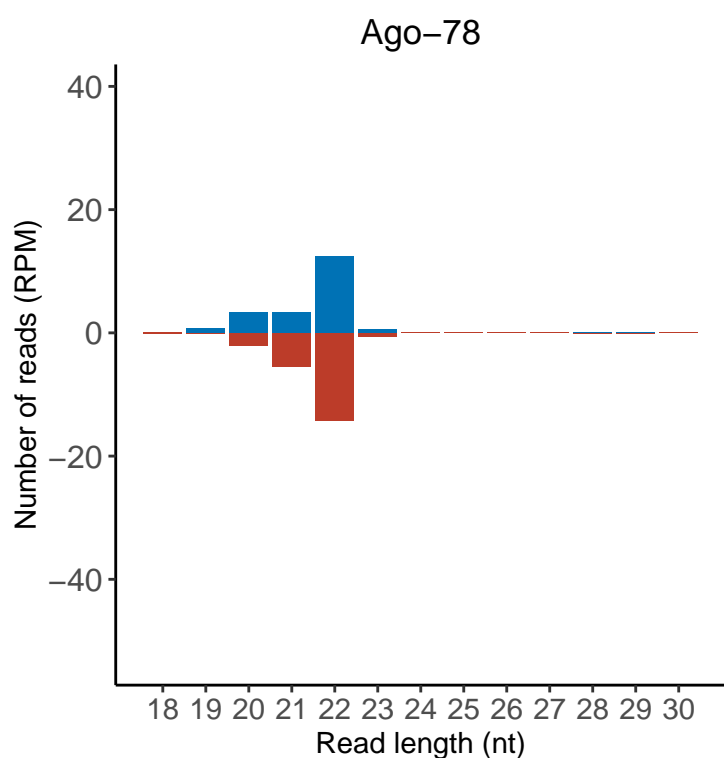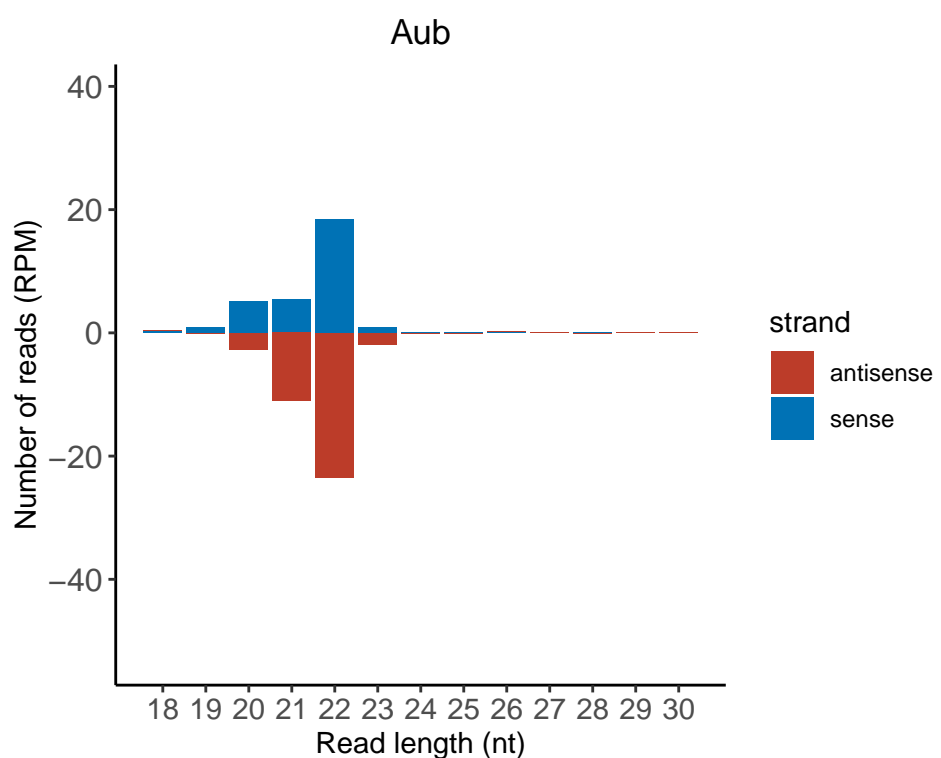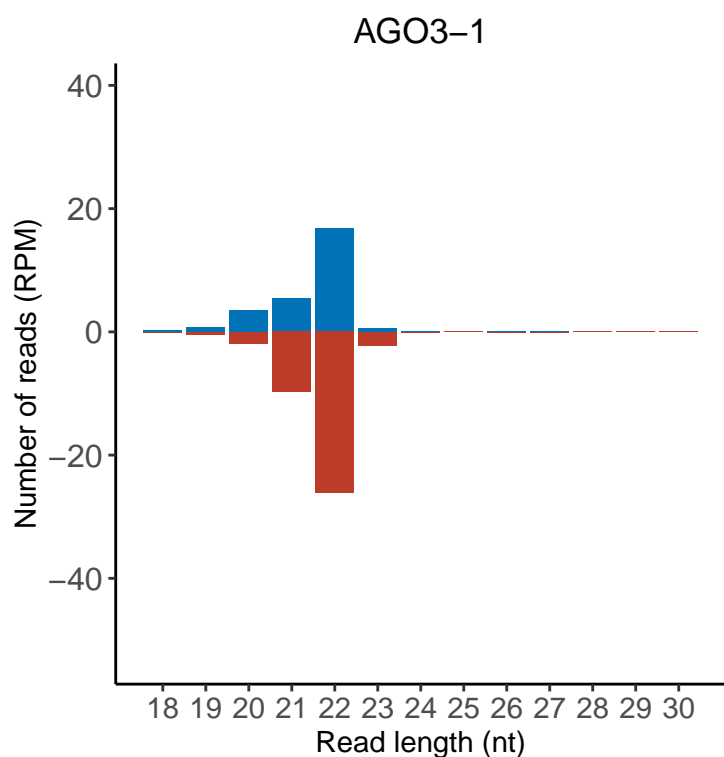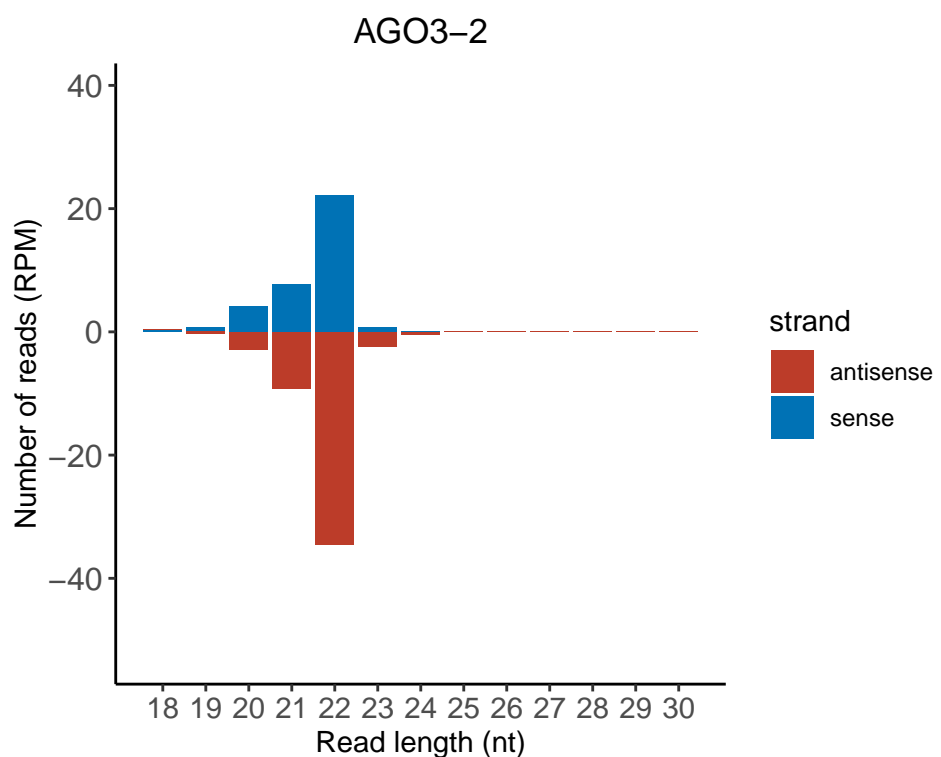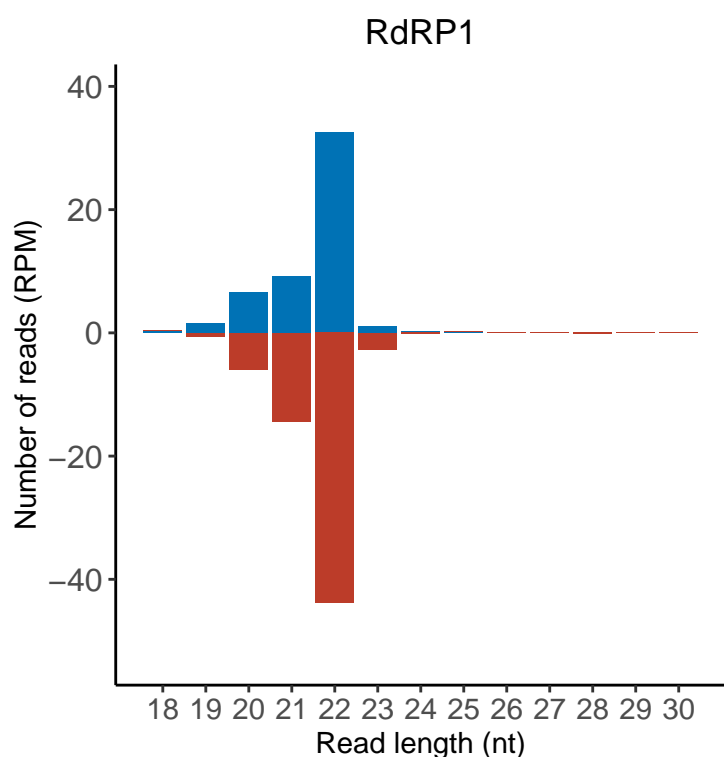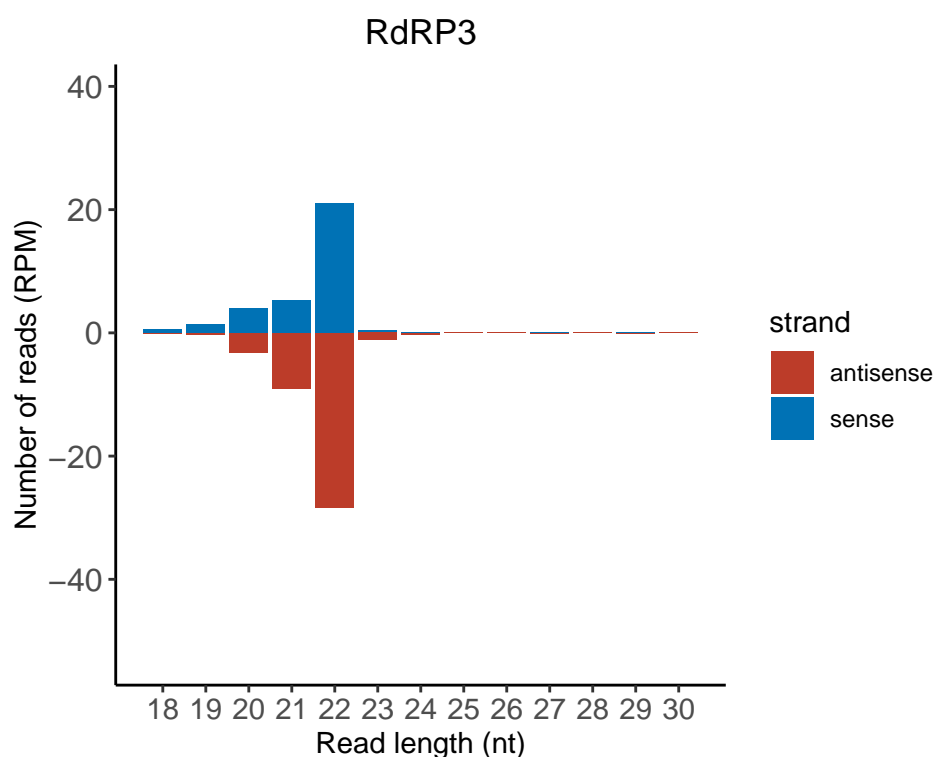

Supplement: S1 Data — On the Normalized and Relative levels pages, normalized read counts (RPM) and relative levels compared to the control (dsGFP) library were used. TEs with more than 50RPM and Coding Genes with more than 3.5RPM on average in KD libraries are included in this website. For viral sequences, reference sequences were generated by assembling sRNA sequences (See the Analysis of sRNAseq data section in Materials and methods.) and size distributions of sRNA reads mapped to each contig are shown. Reads were first grouped by their 5’ nucleotides, and reads of each length were counted. Full tables including TEs and Coding genes with fewer reads are in the “FullTable” folder. (ZIP) [file pone.0281195.s017.zip › SupplementaryData/Links/PDFs/Motif:rnd-6_family-2596TE_lengthdistribution.pdf]

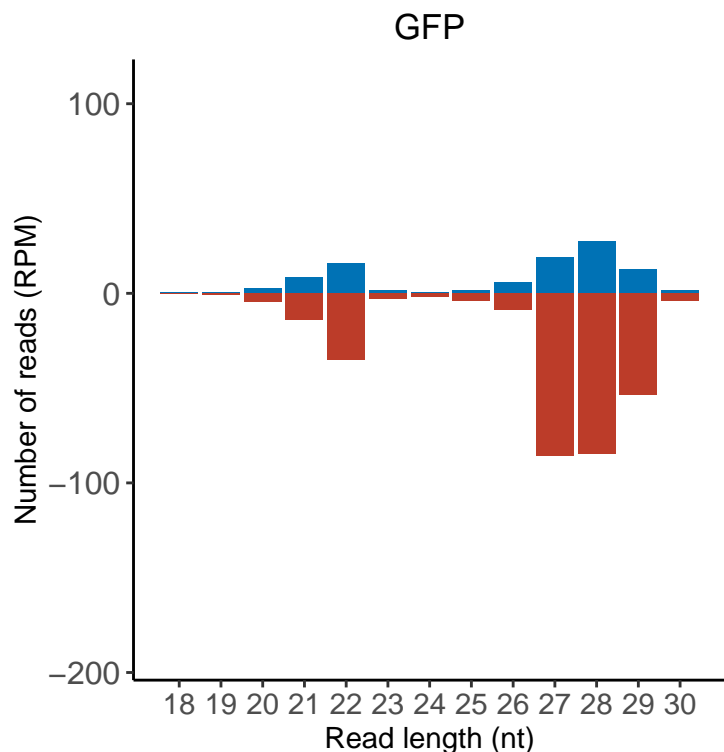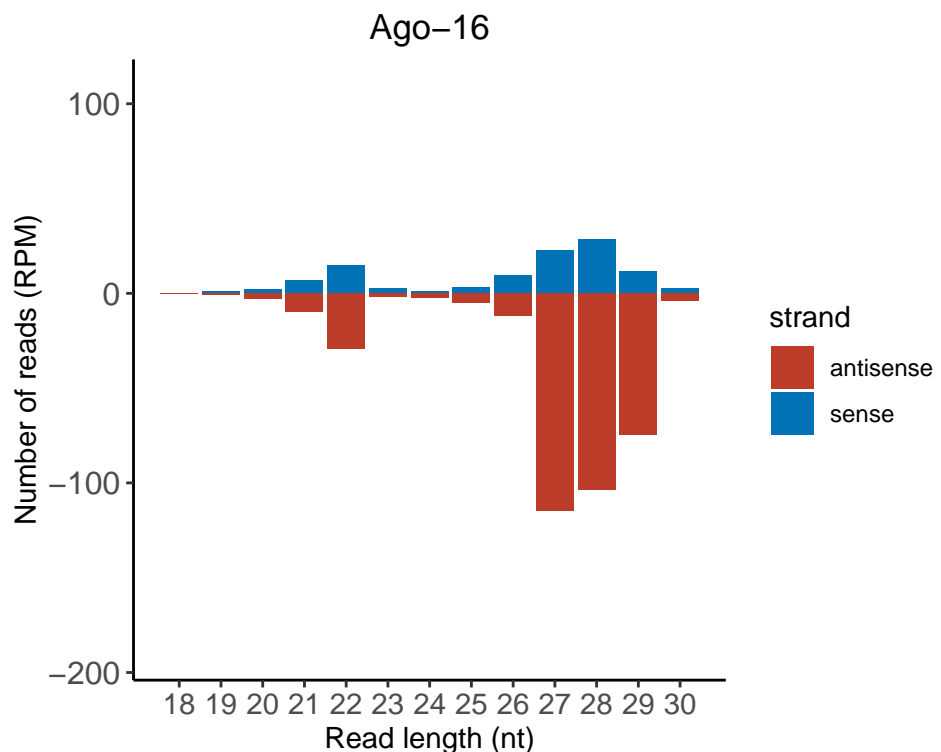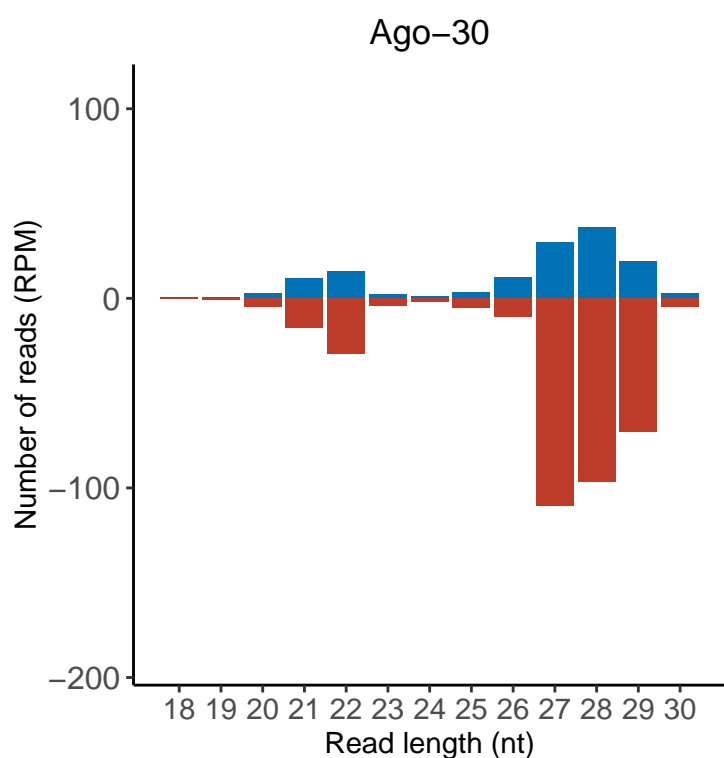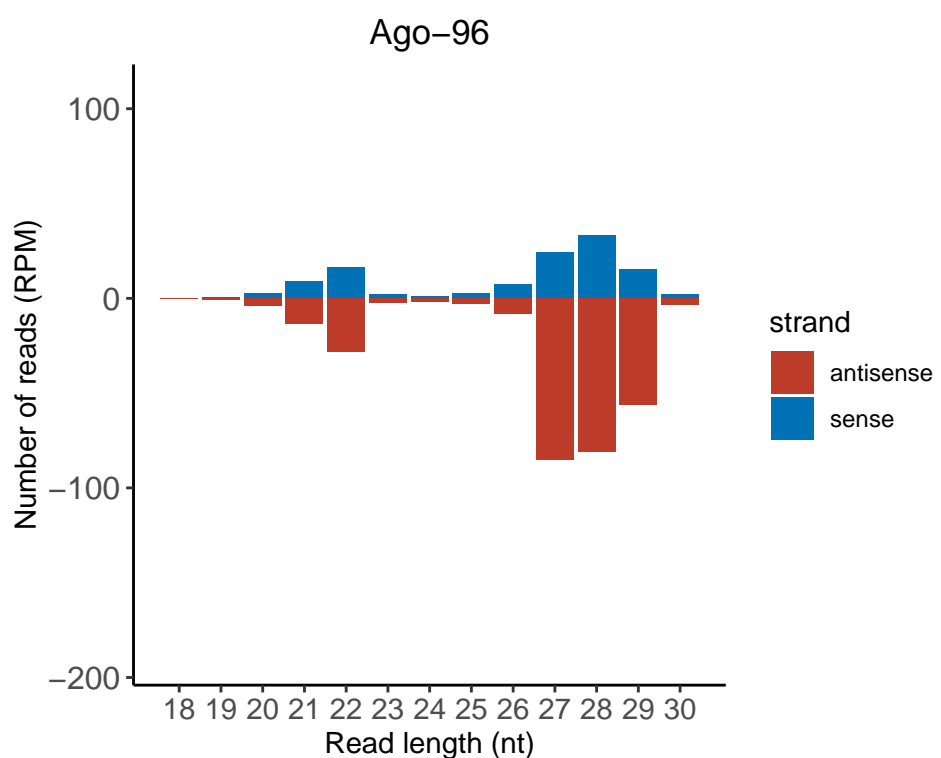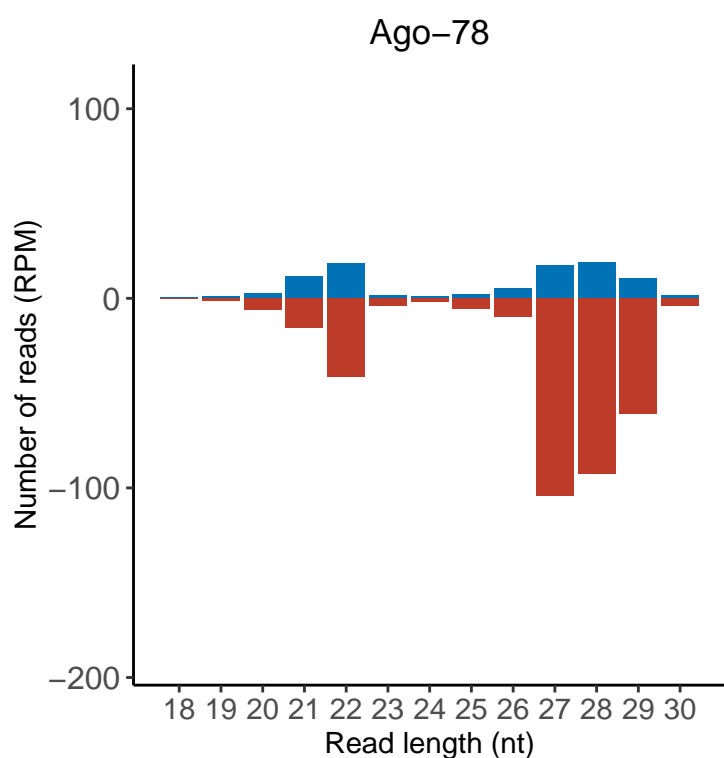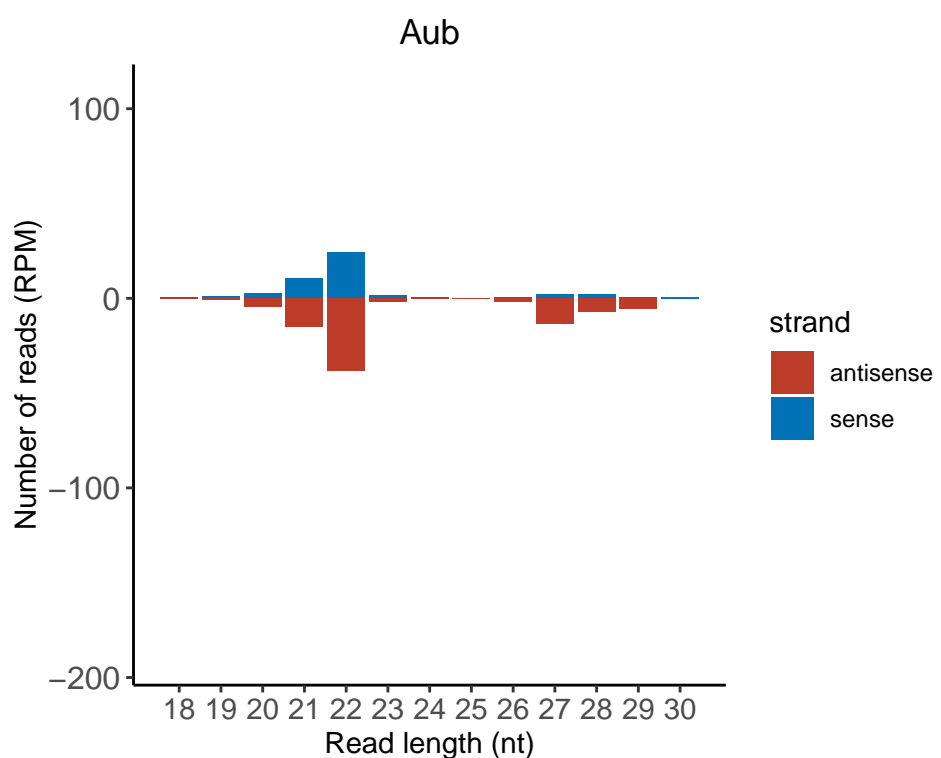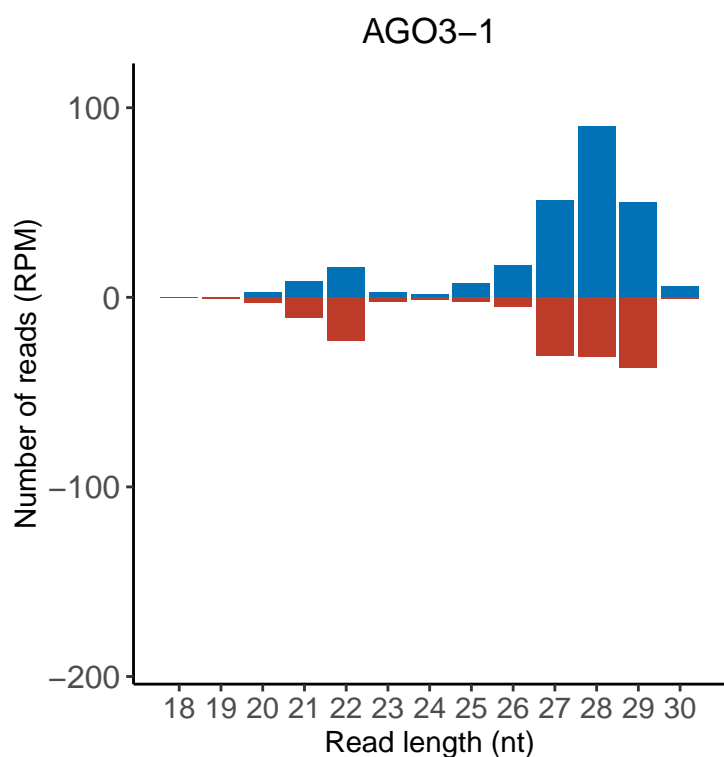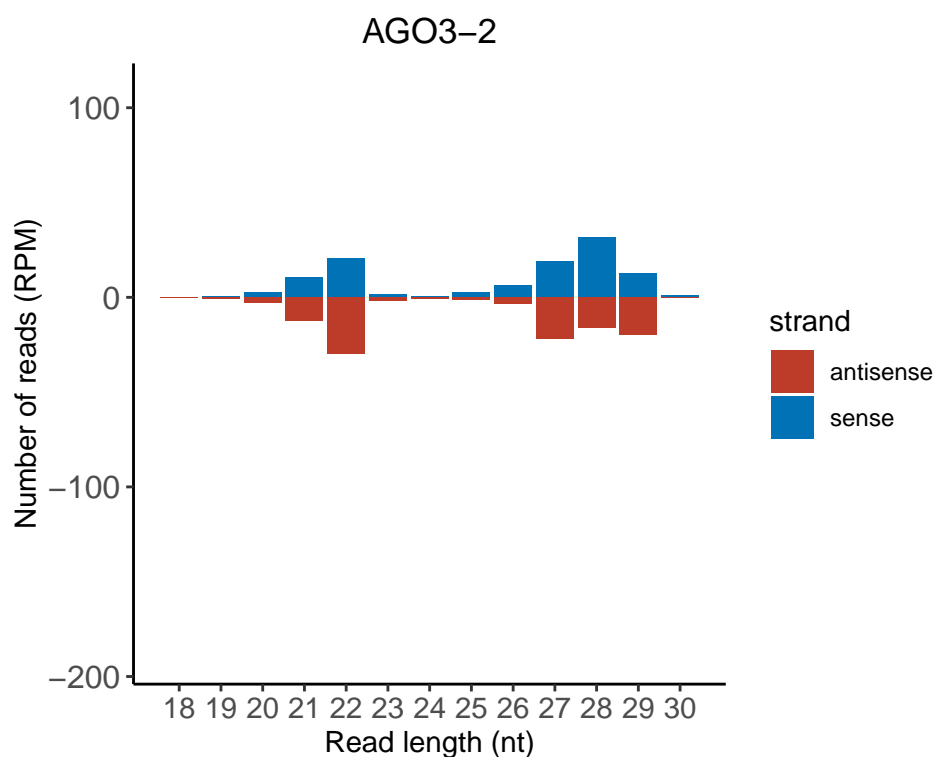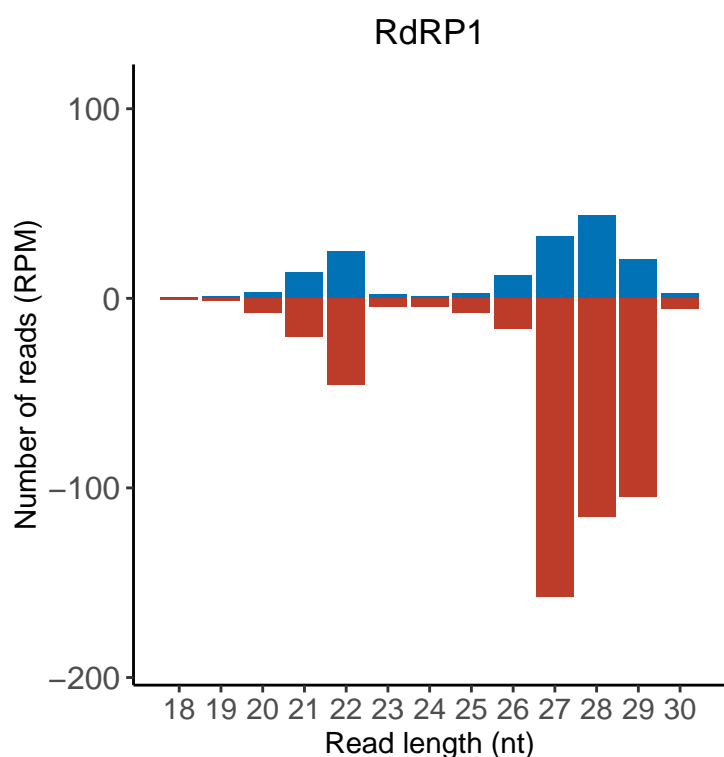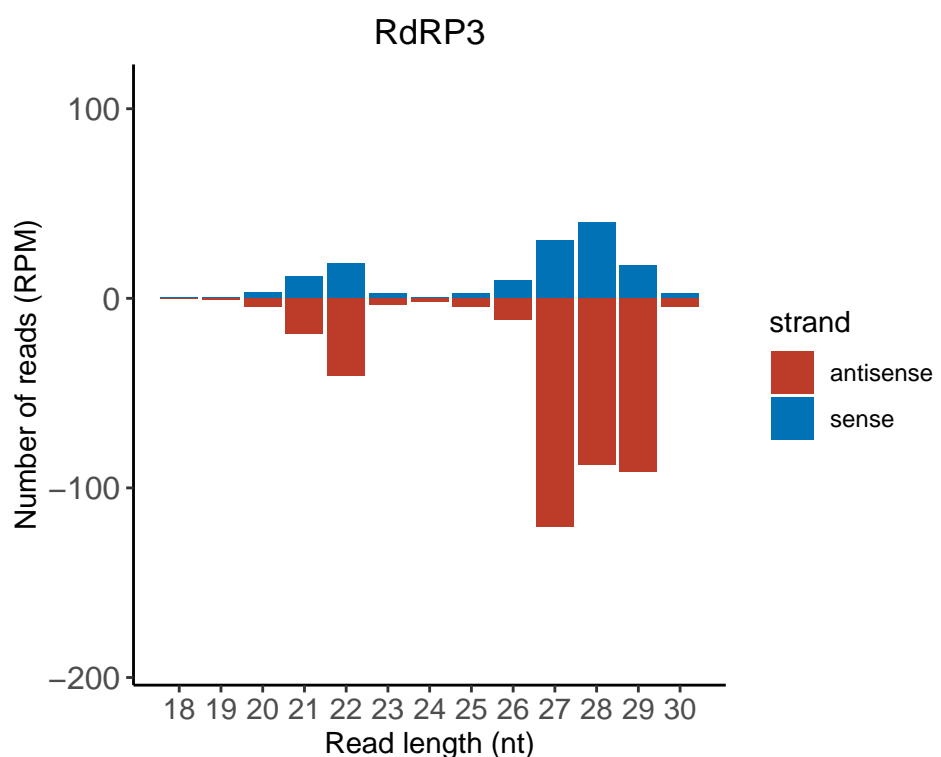

Supplement: S1 Data — On the Normalized and Relative levels pages, normalized read counts (RPM) and relative levels compared to the control (dsGFP) library were used. TEs with more than 50RPM and Coding Genes with more than 3.5RPM on average in KD libraries are included in this website. For viral sequences, reference sequences were generated by assembling sRNA sequences (See the Analysis of sRNAseq data section in Materials and methods.) and size distributions of sRNA reads mapped to each contig are shown. Reads were first grouped by their 5’ nucleotides, and reads of each length were counted. Full tables including TEs and Coding genes with fewer reads are in the “FullTable” folder. (ZIP) [file pone.0281195.s017.zip › SupplementaryData/Links/PDFs/Motif:rnd-1_family-1650TE_lengthdistribution.pdf]

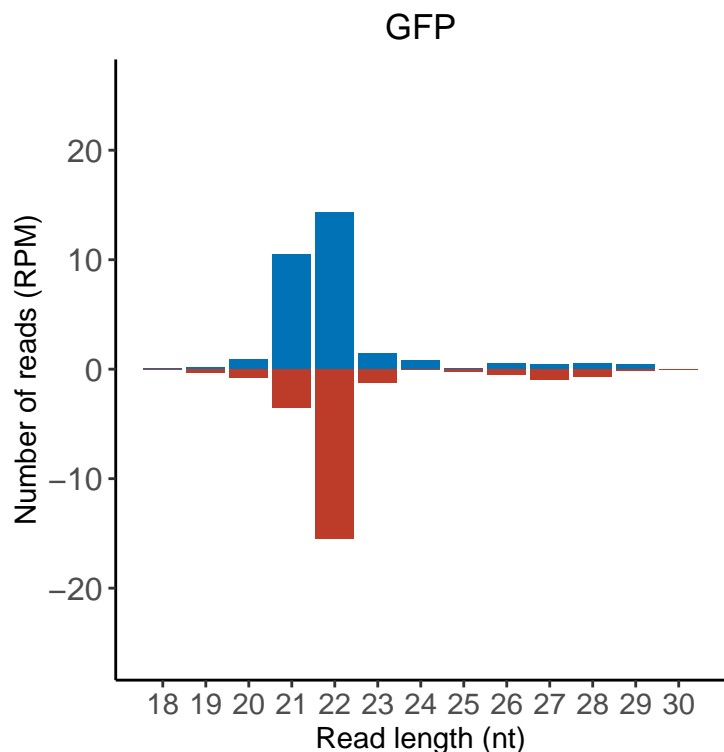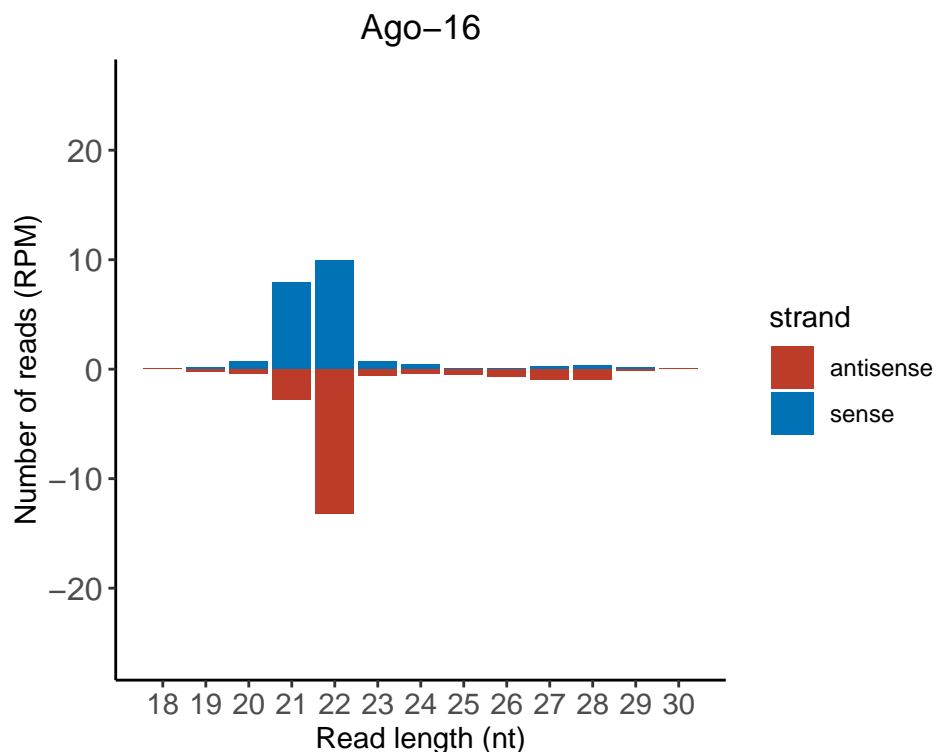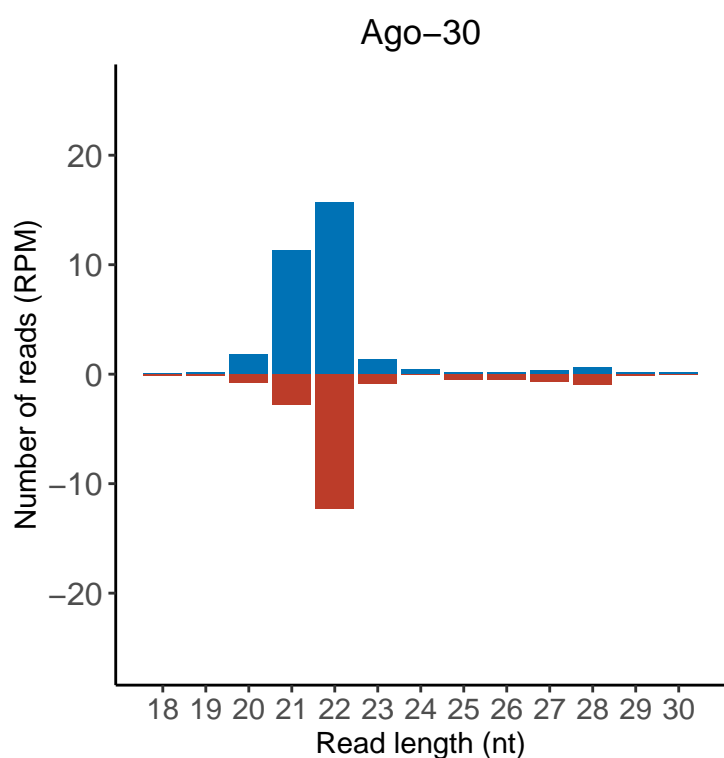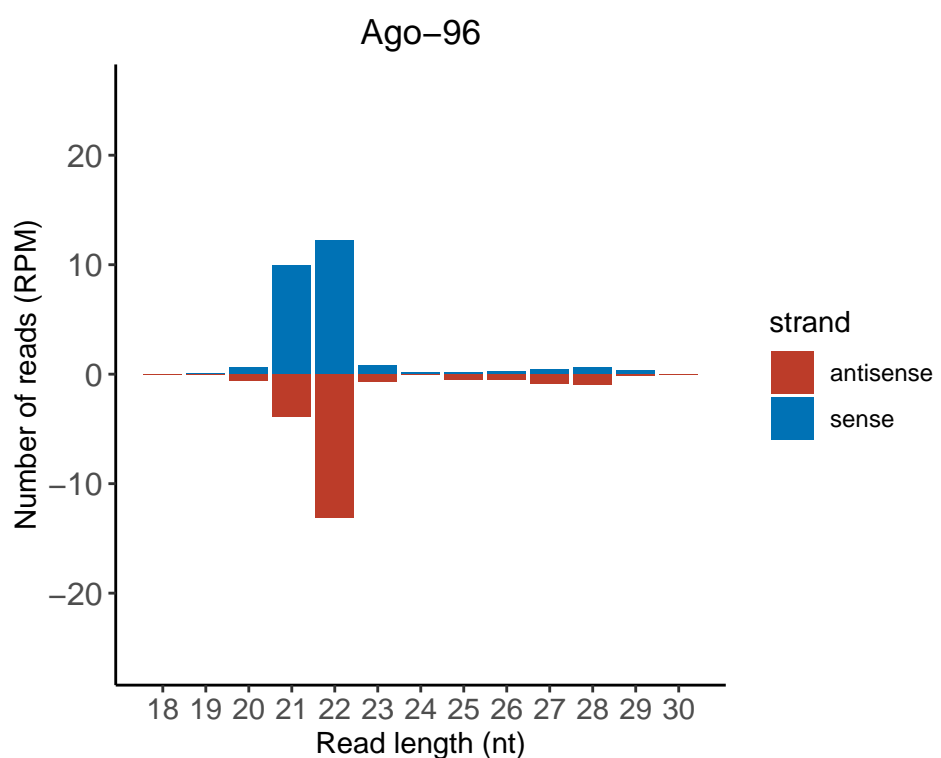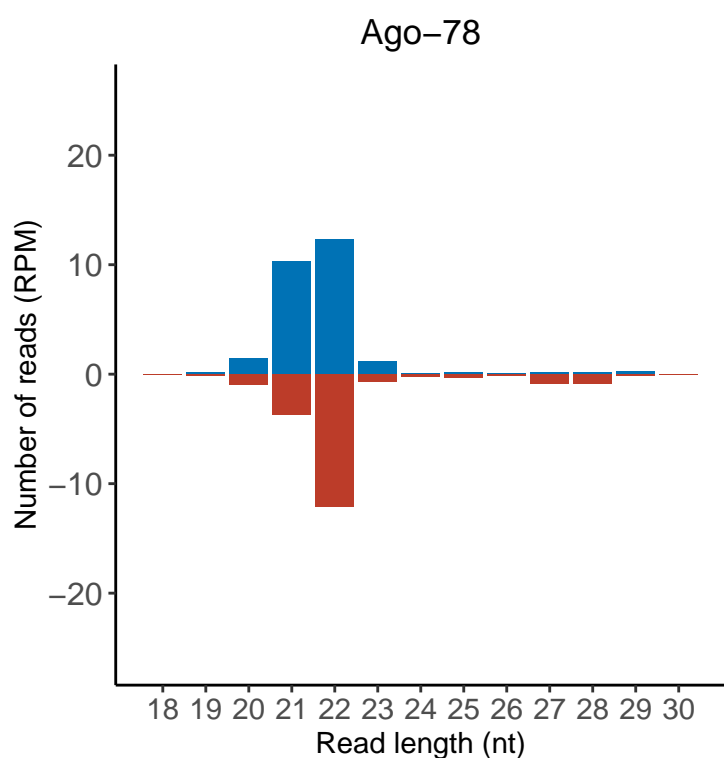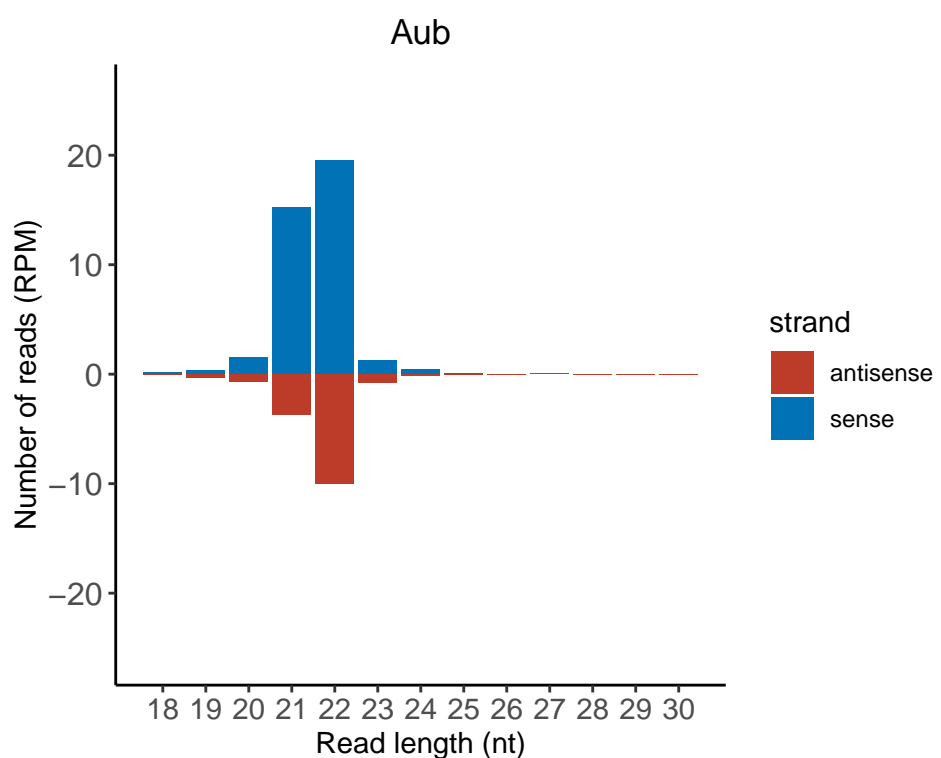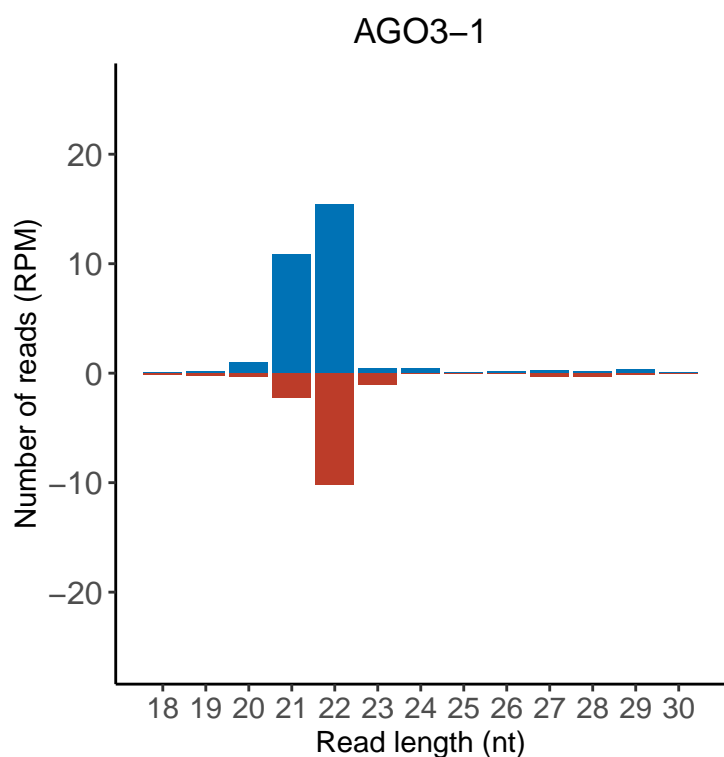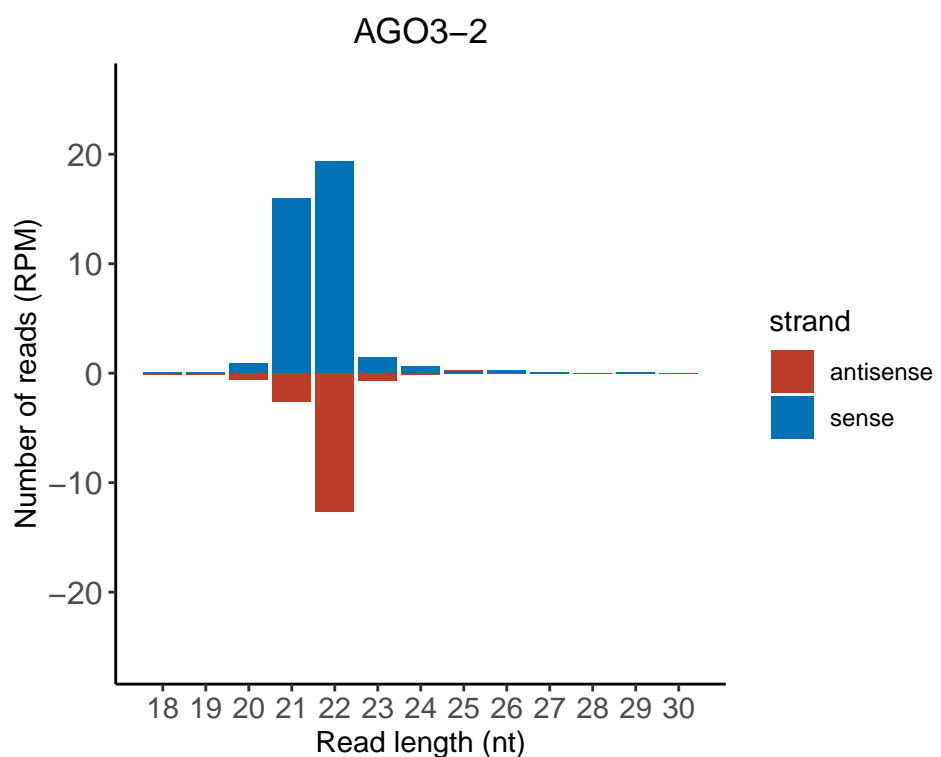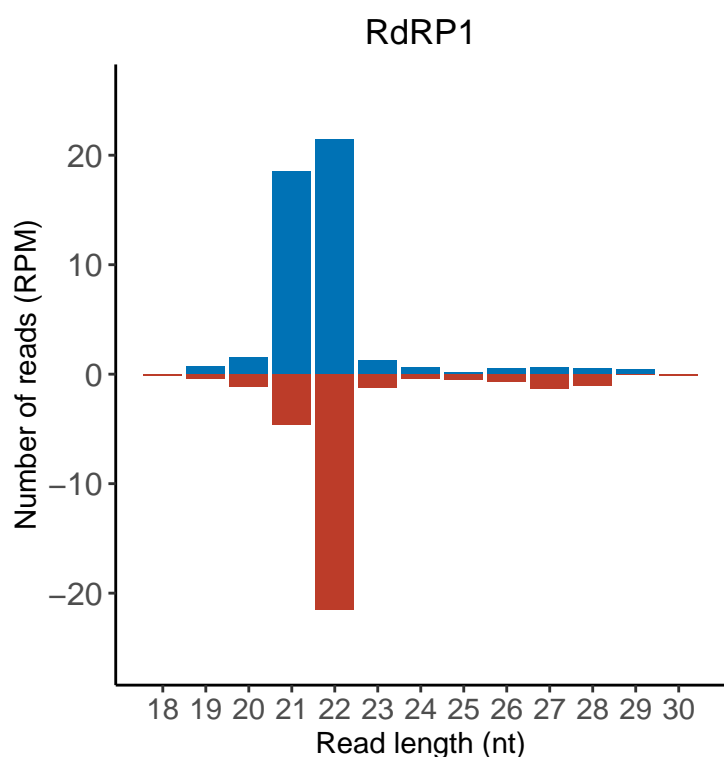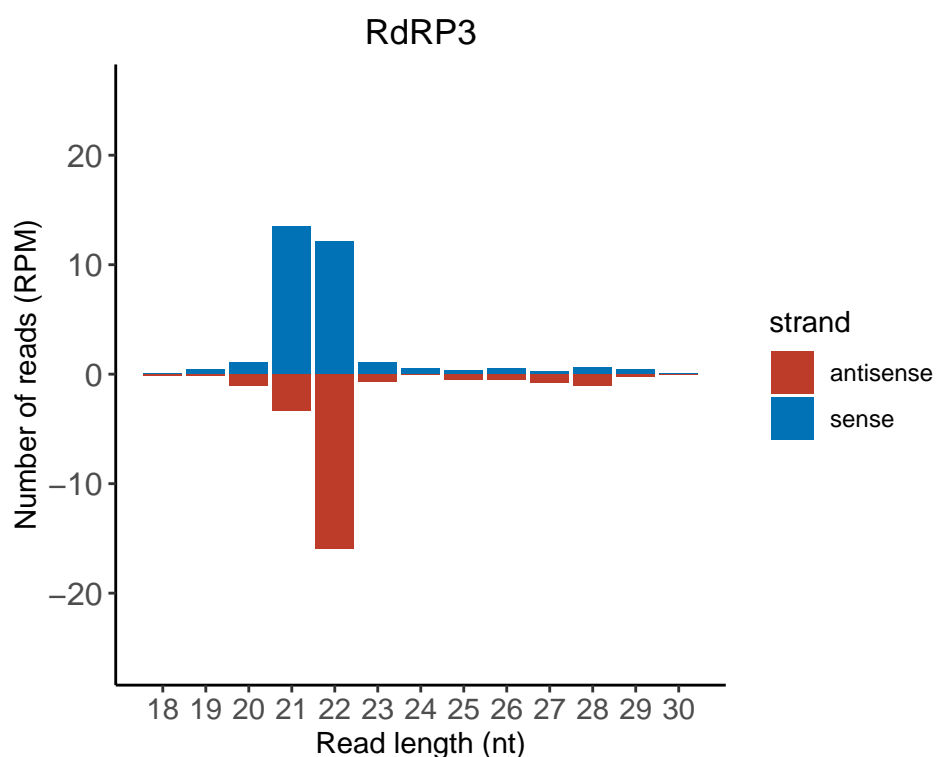

Supplement: S1 Data — On the Normalized and Relative levels pages, normalized read counts (RPM) and relative levels compared to the control (dsGFP) library were used. TEs with more than 50RPM and Coding Genes with more than 3.5RPM on average in KD libraries are included in this website. For viral sequences, reference sequences were generated by assembling sRNA sequences (See the Analysis of sRNAseq data section in Materials and methods.) and size distributions of sRNA reads mapped to each contig are shown. Reads were first grouped by their 5’ nucleotides, and reads of each length were counted. Full tables including TEs and Coding genes with fewer reads are in the “FullTable” folder. (ZIP) [file pone.0281195.s017.zip › SupplementaryData/Links/PDFs/Motif:rnd-4_family-281TE_lengthdistribution.pdf]

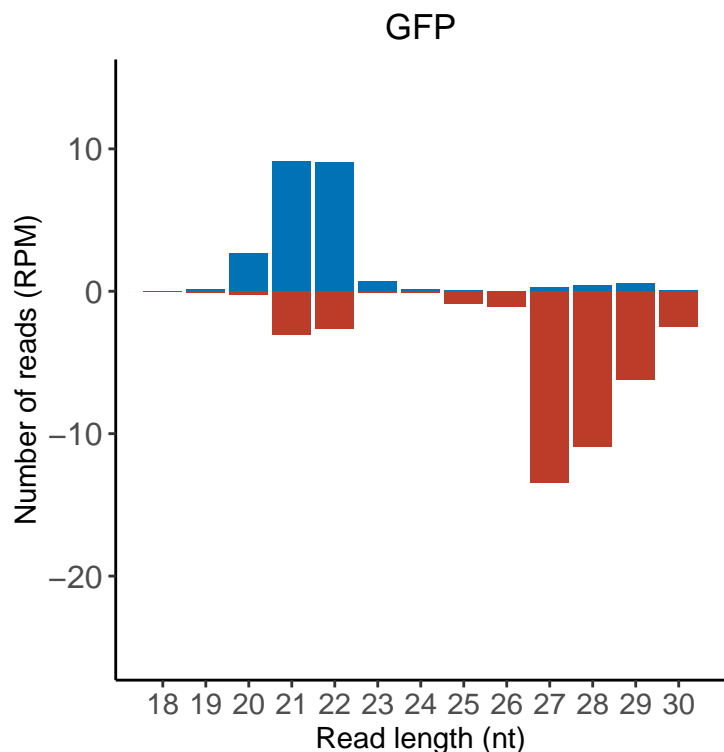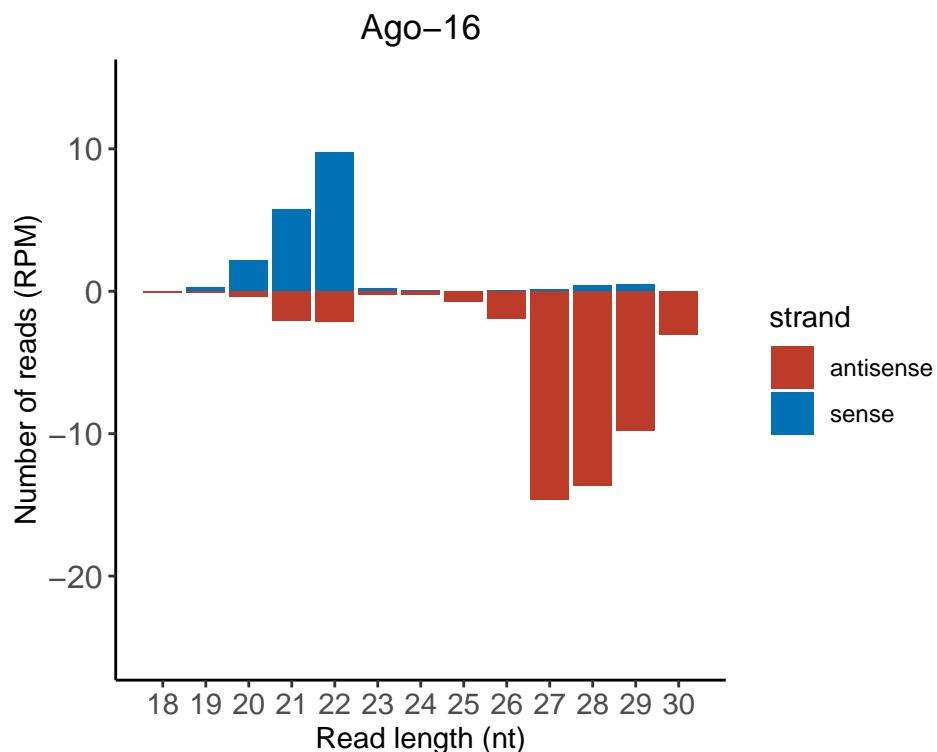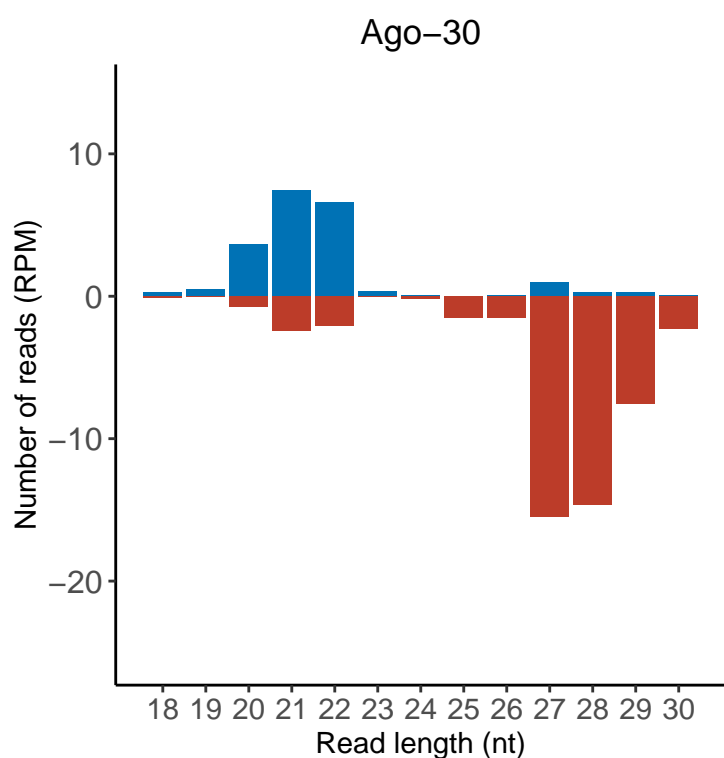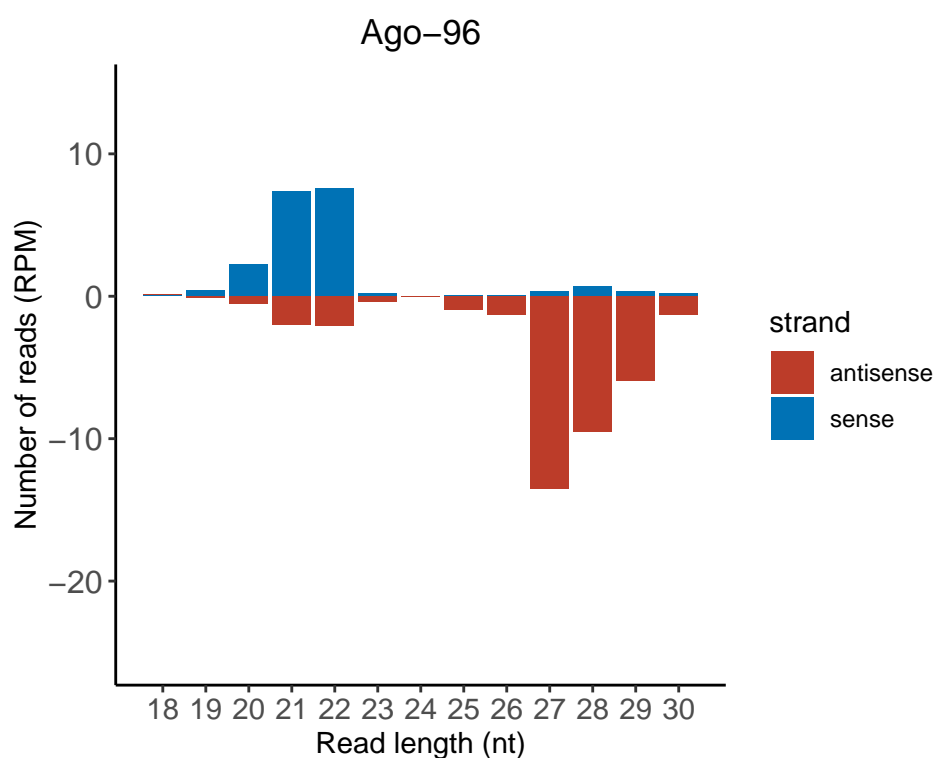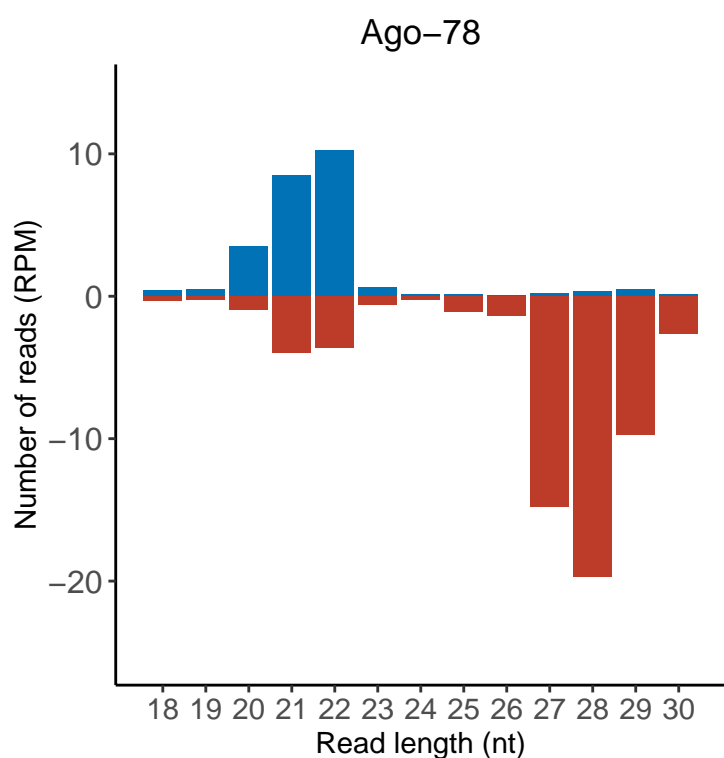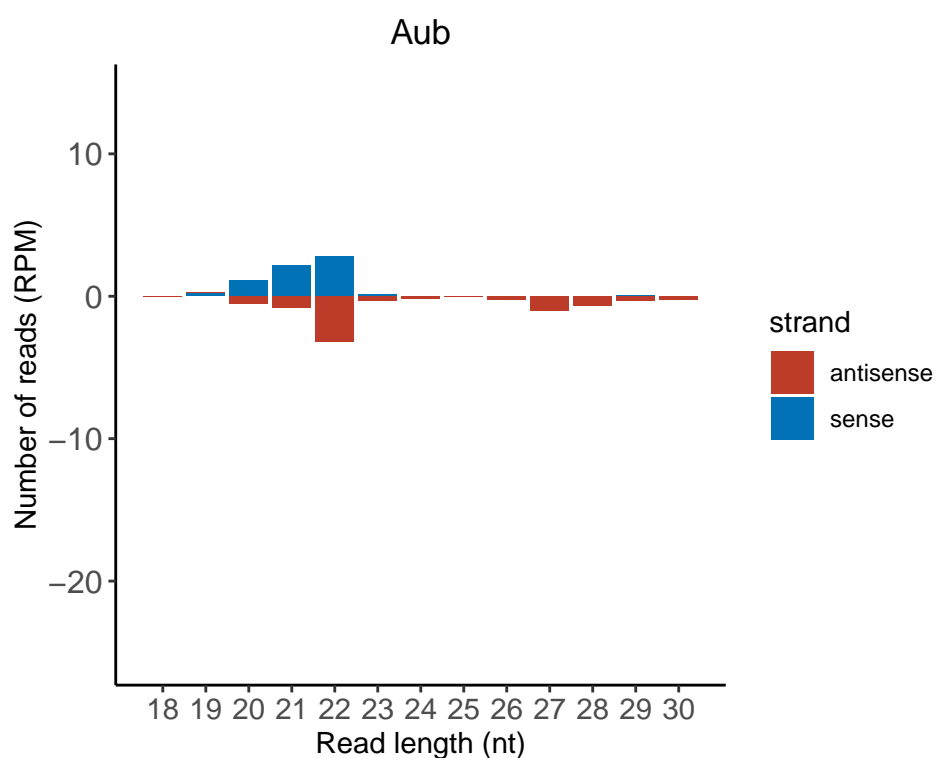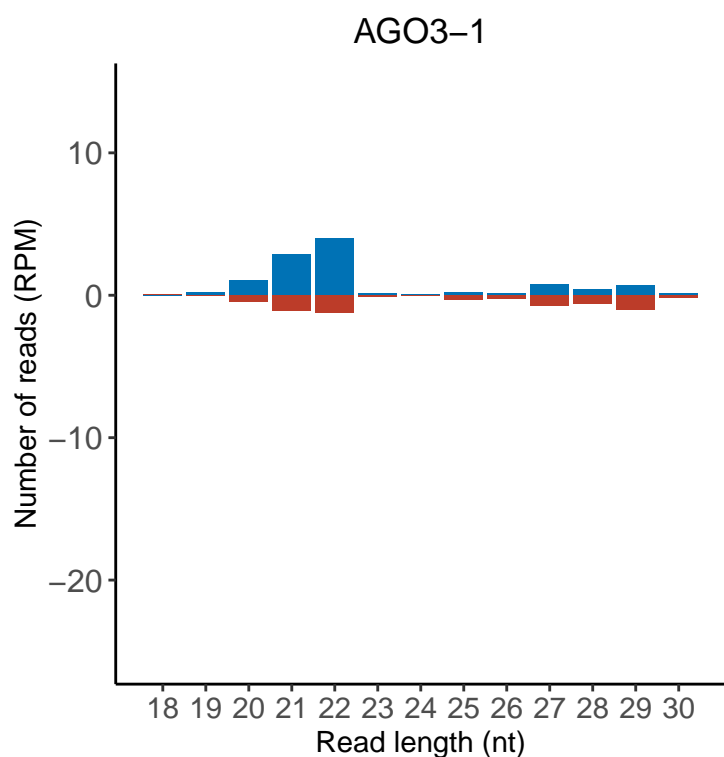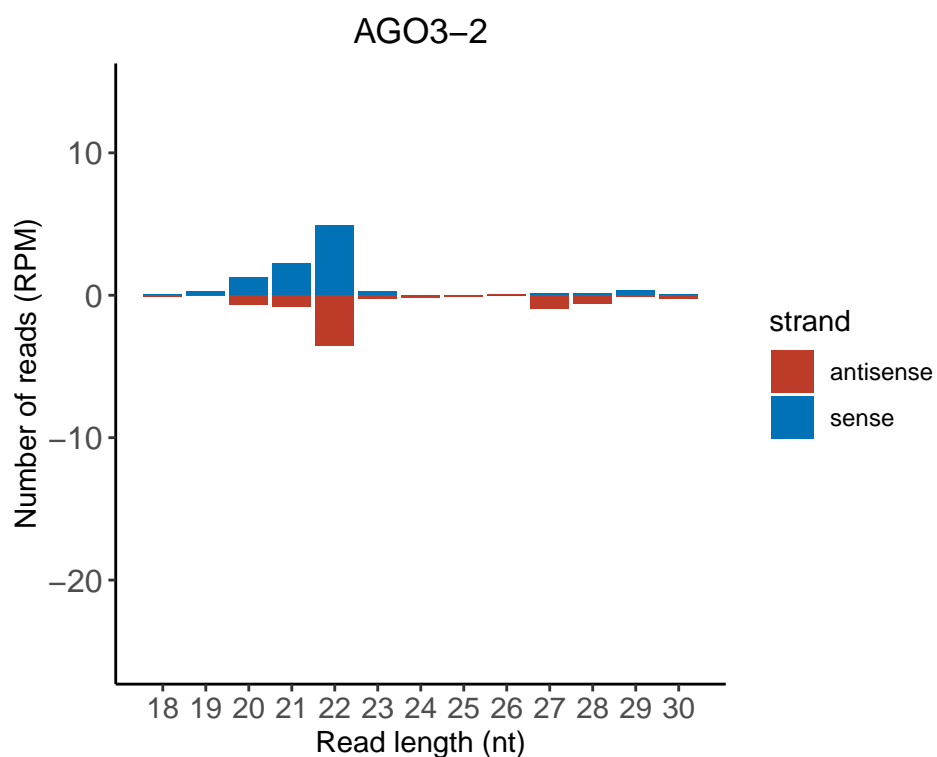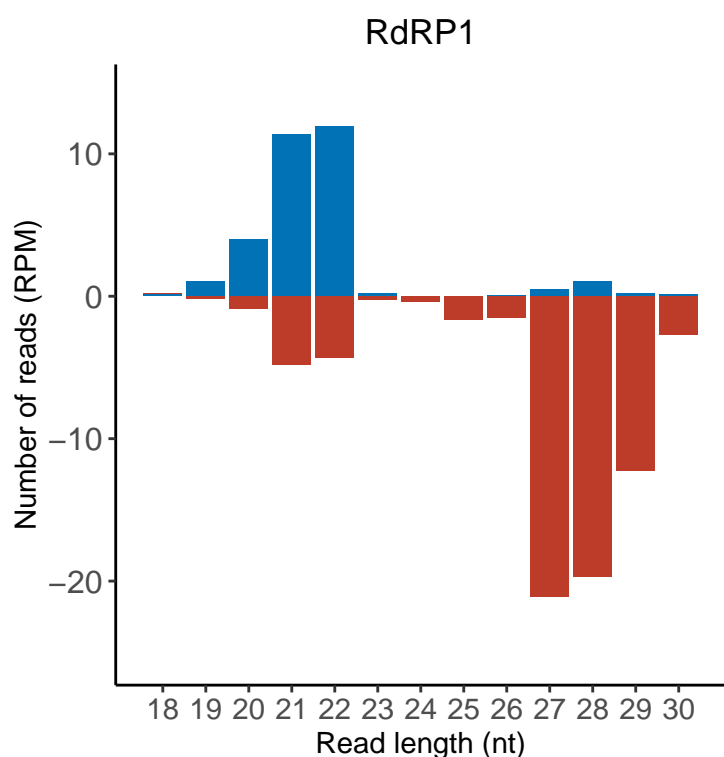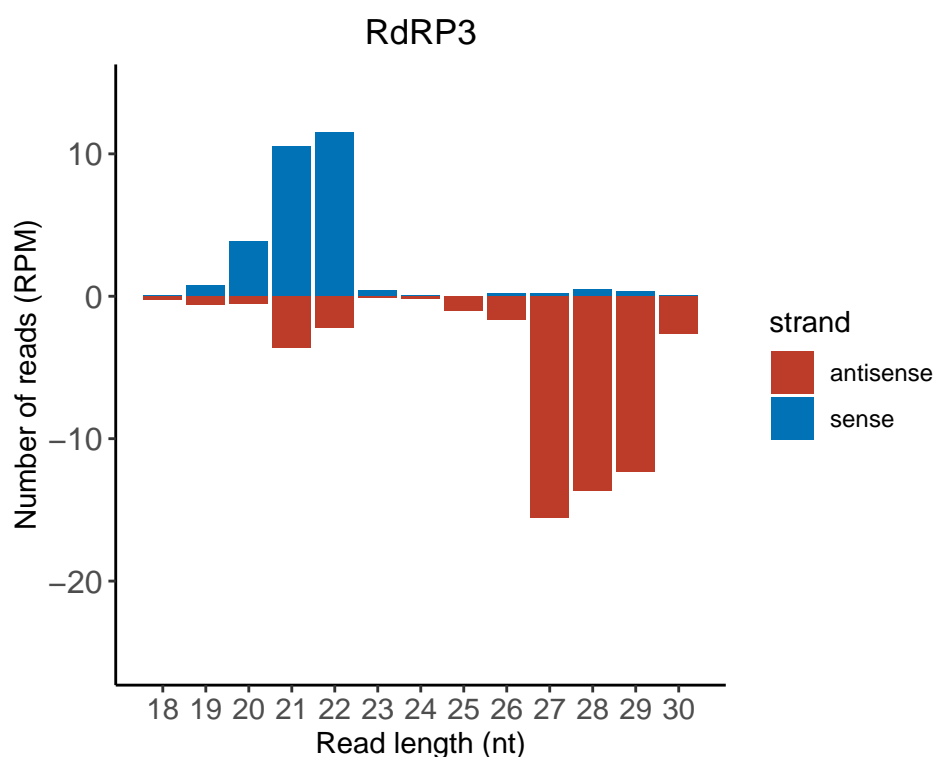

Supplement: S1 Data — On the Normalized and Relative levels pages, normalized read counts (RPM) and relative levels compared to the control (dsGFP) library were used. TEs with more than 50RPM and Coding Genes with more than 3.5RPM on average in KD libraries are included in this website. For viral sequences, reference sequences were generated by assembling sRNA sequences (See the Analysis of sRNAseq data section in Materials and methods.) and size distributions of sRNA reads mapped to each contig are shown. Reads were first grouped by their 5’ nucleotides, and reads of each length were counted. Full tables including TEs and Coding genes with fewer reads are in the “FullTable” folder. (ZIP) [file pone.0281195.s017.zip › SupplementaryData/Links/PDFs/Motif:rnd-1_family-305TE_lengthdistribution.pdf]

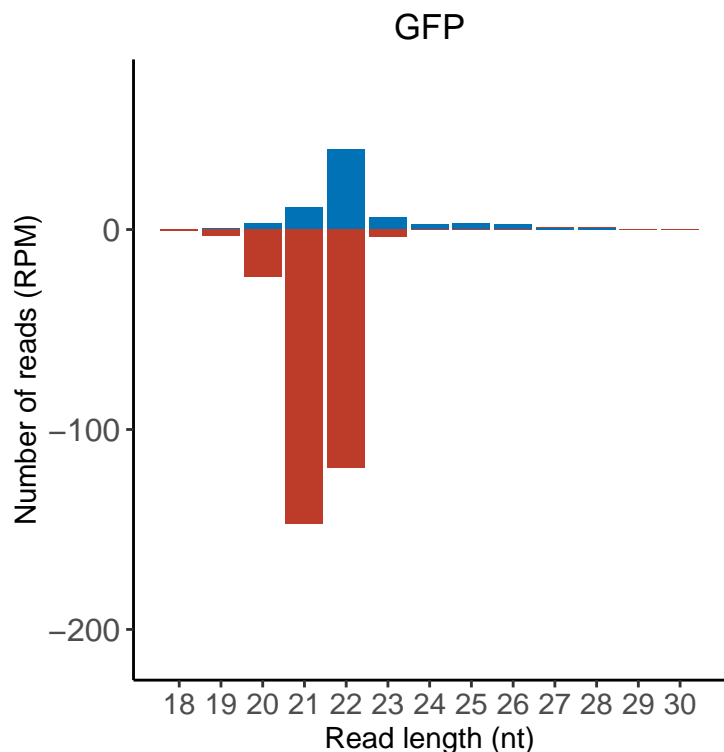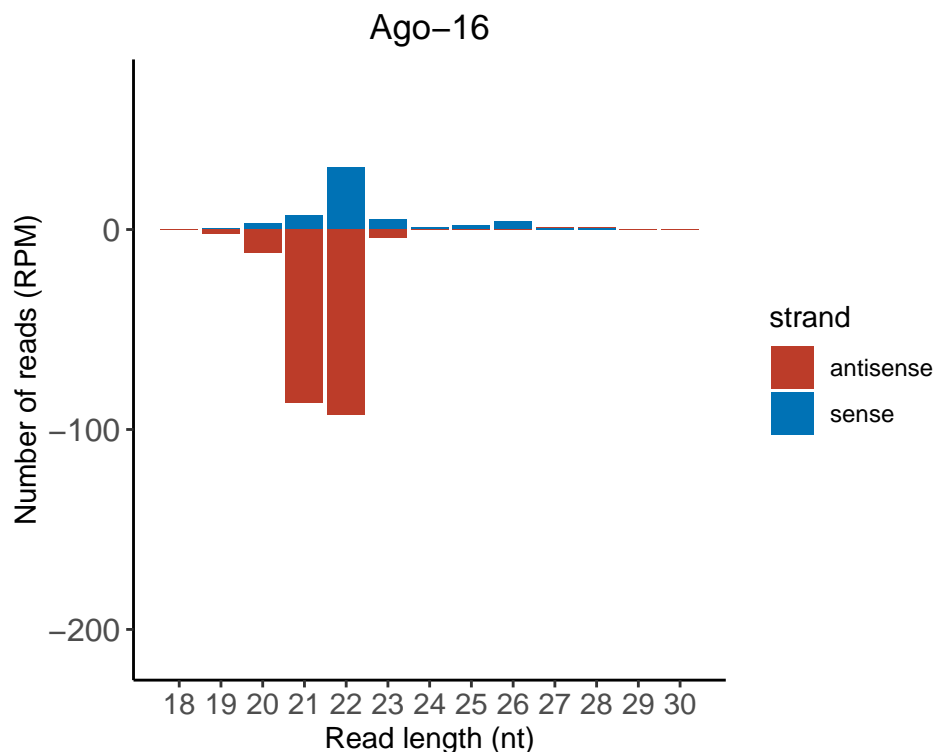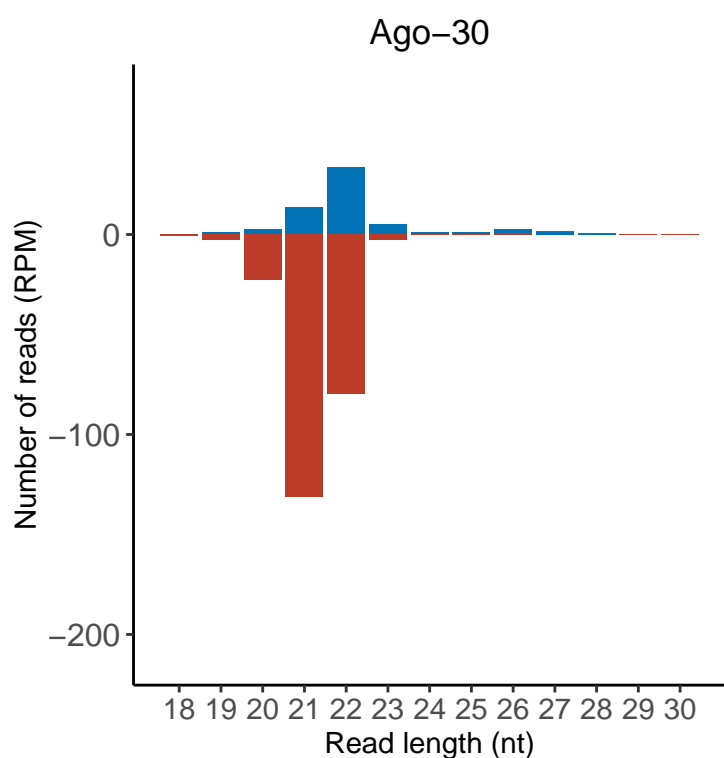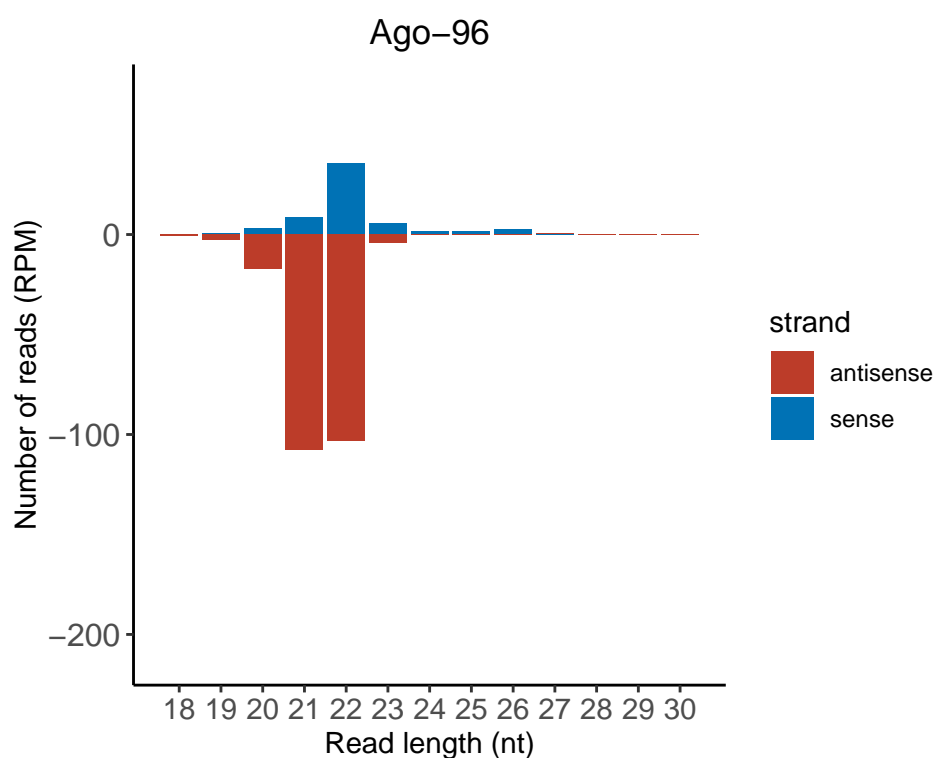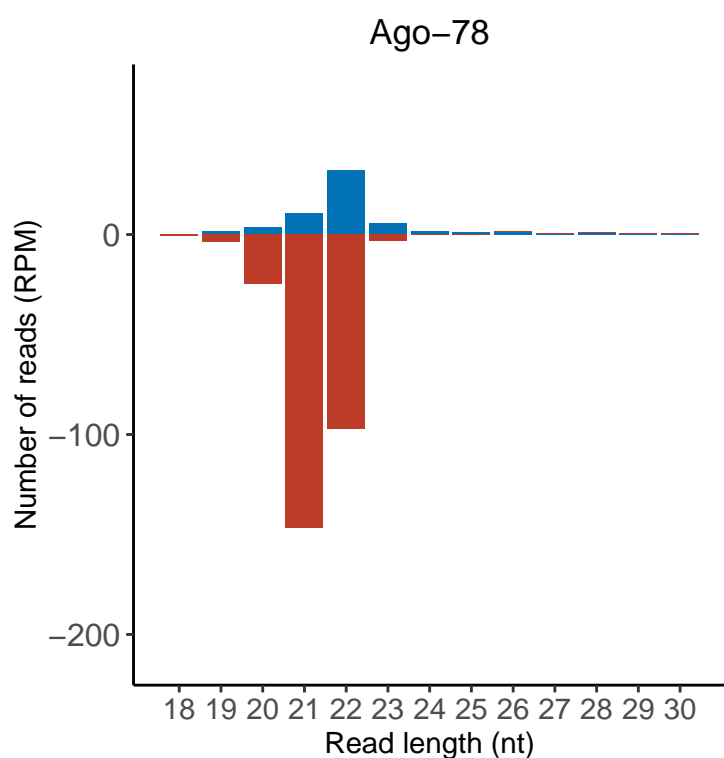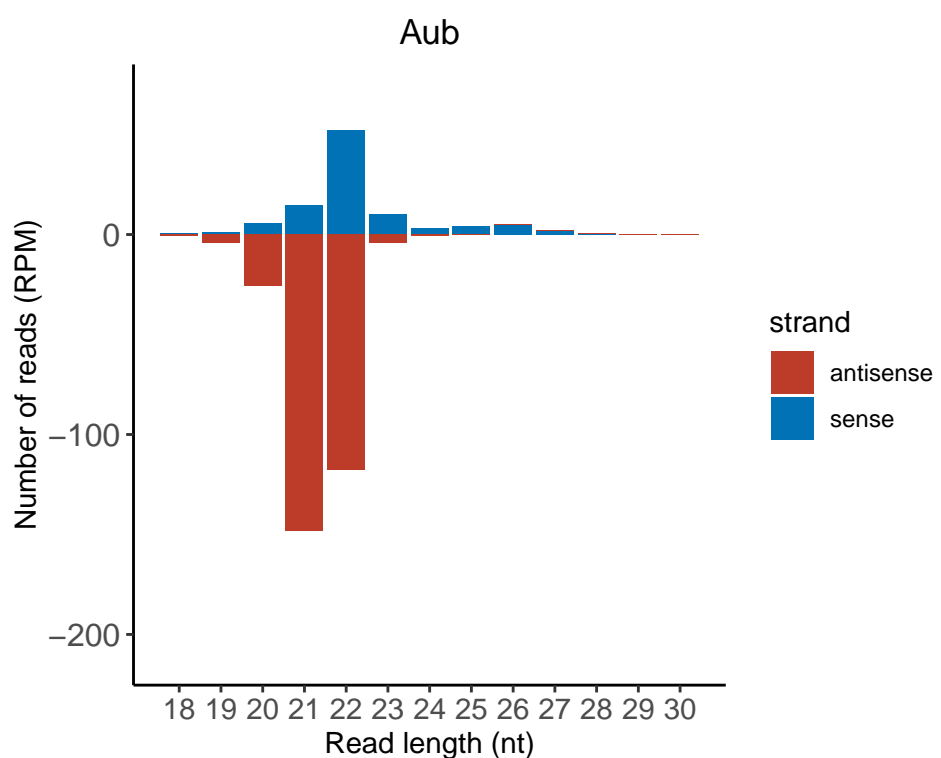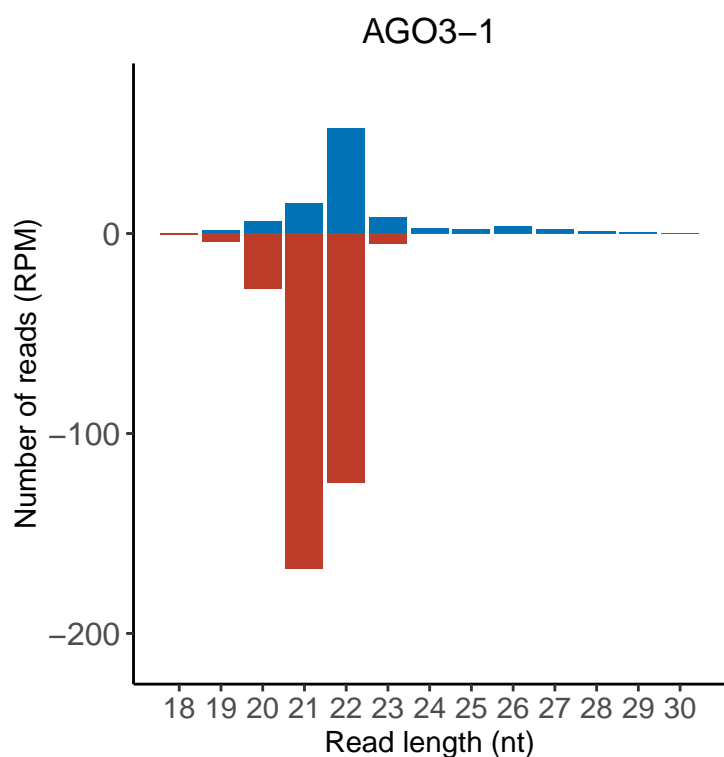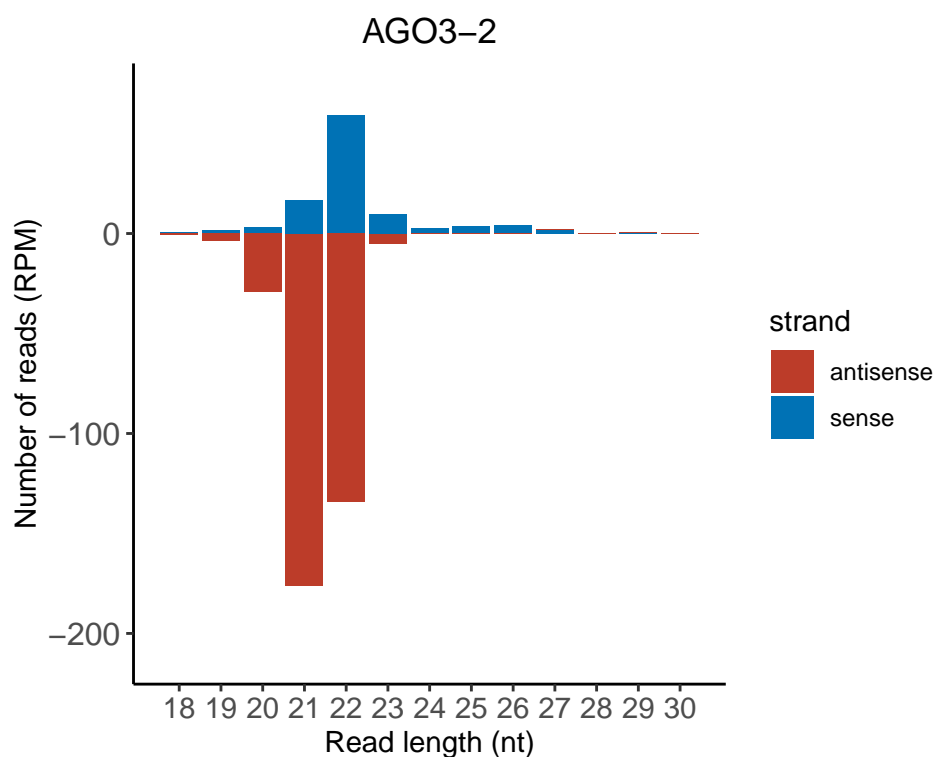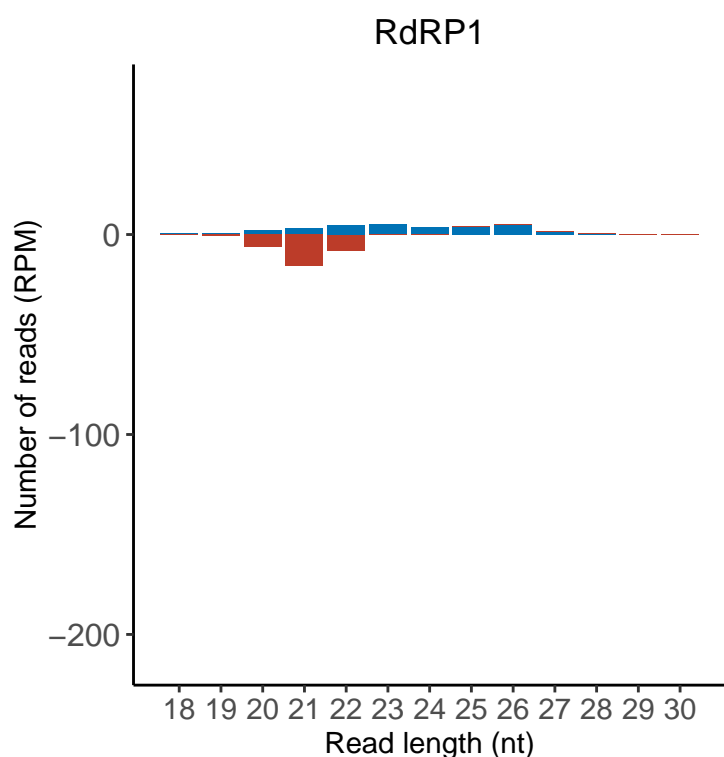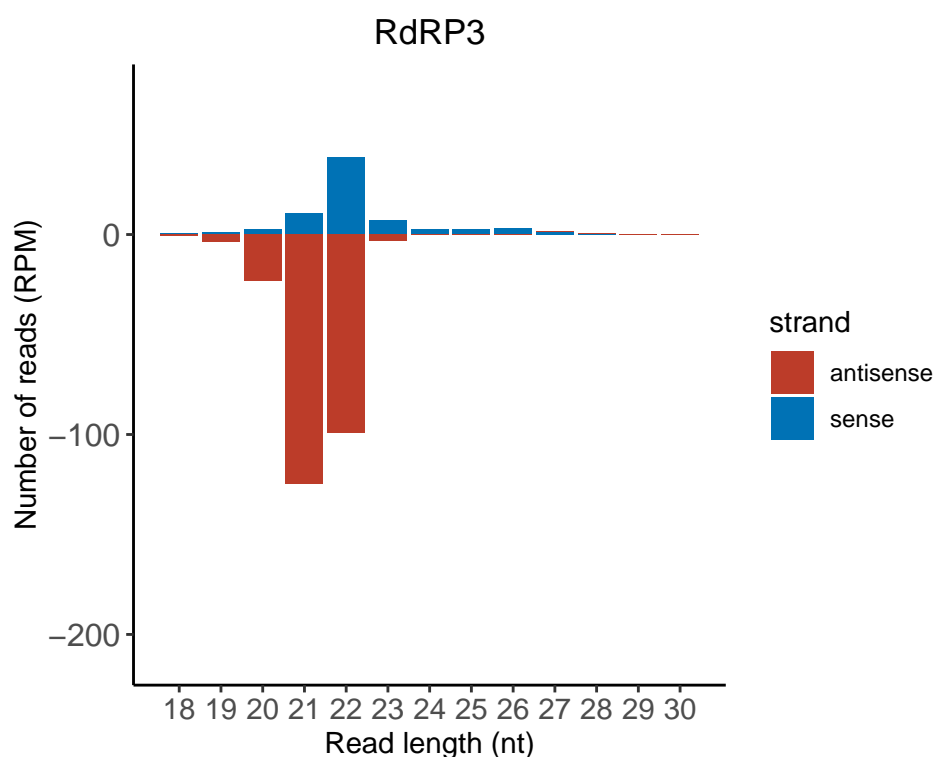

Supplement: S1 Data — On the Normalized and Relative levels pages, normalized read counts (RPM) and relative levels compared to the control (dsGFP) library were used. TEs with more than 50RPM and Coding Genes with more than 3.5RPM on average in KD libraries are included in this website. For viral sequences, reference sequences were generated by assembling sRNA sequences (See the Analysis of sRNAseq data section in Materials and methods.) and size distributions of sRNA reads mapped to each contig are shown. Reads were first grouped by their 5’ nucleotides, and reads of each length were counted. Full tables including TEs and Coding genes with fewer reads are in the “FullTable” folder. (ZIP) [file pone.0281195.s017.zip › SupplementaryData/Links/PDFs/Motif:rnd-1_family-1055TE_lengthdistribution.pdf]

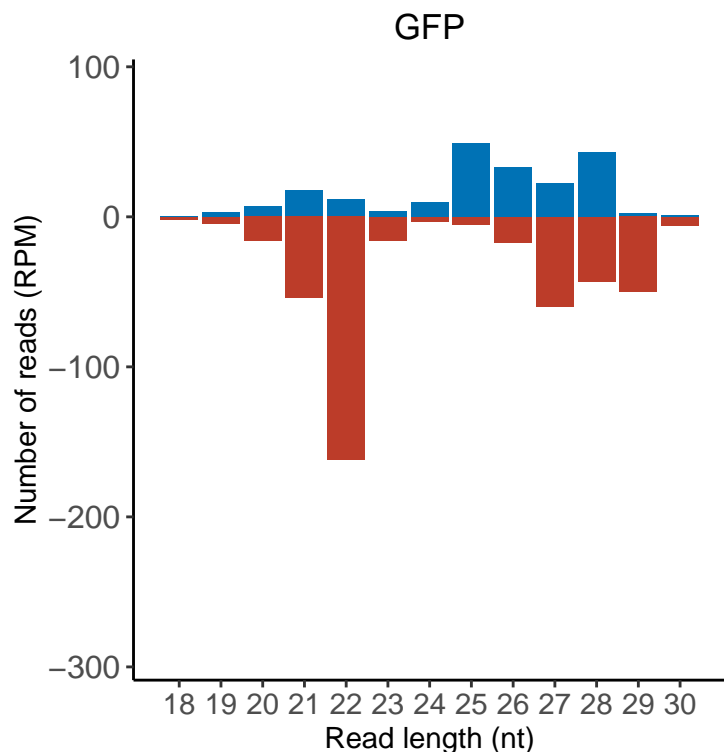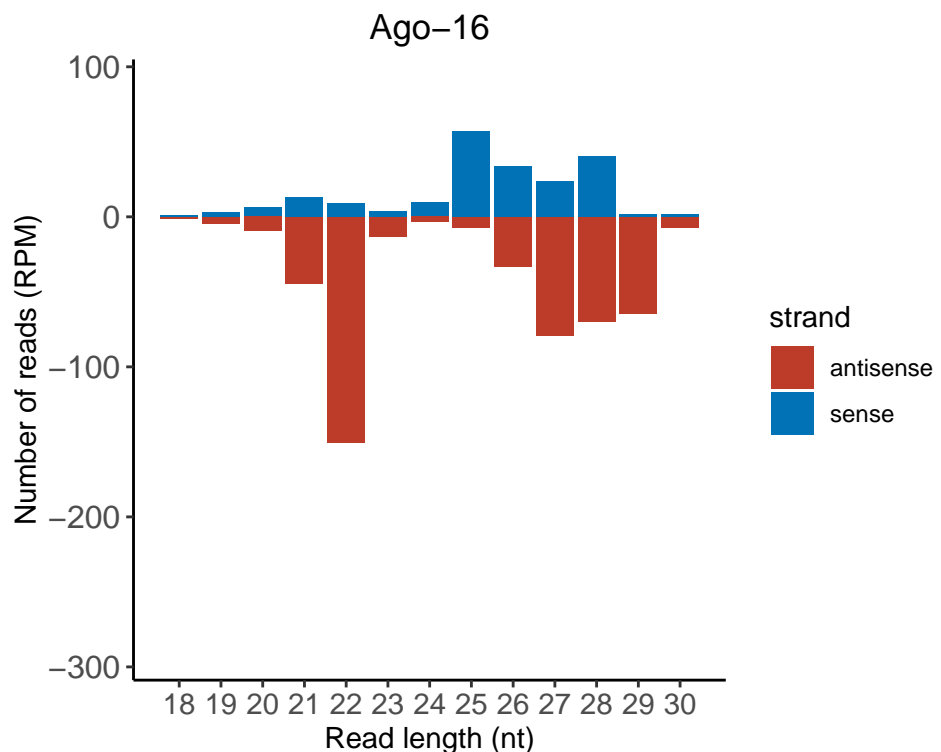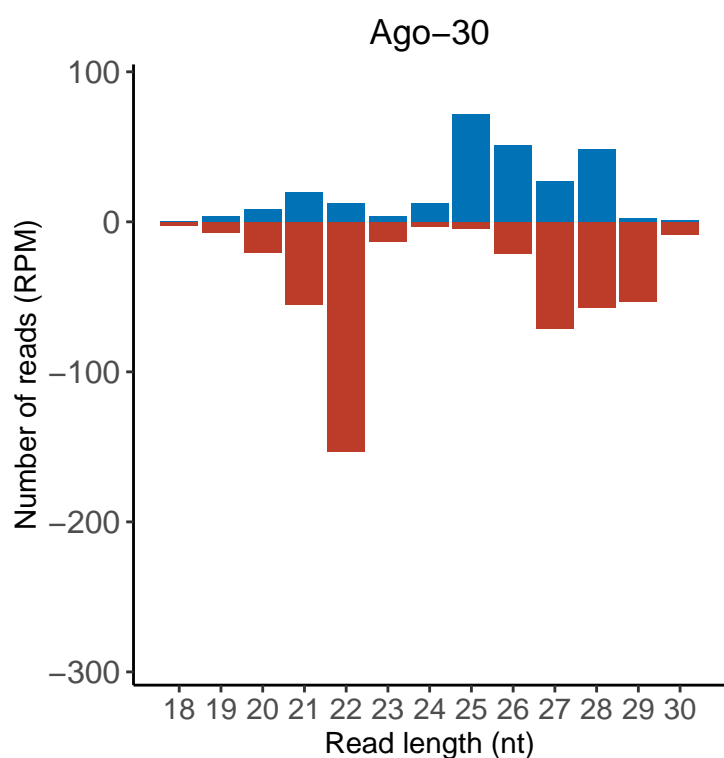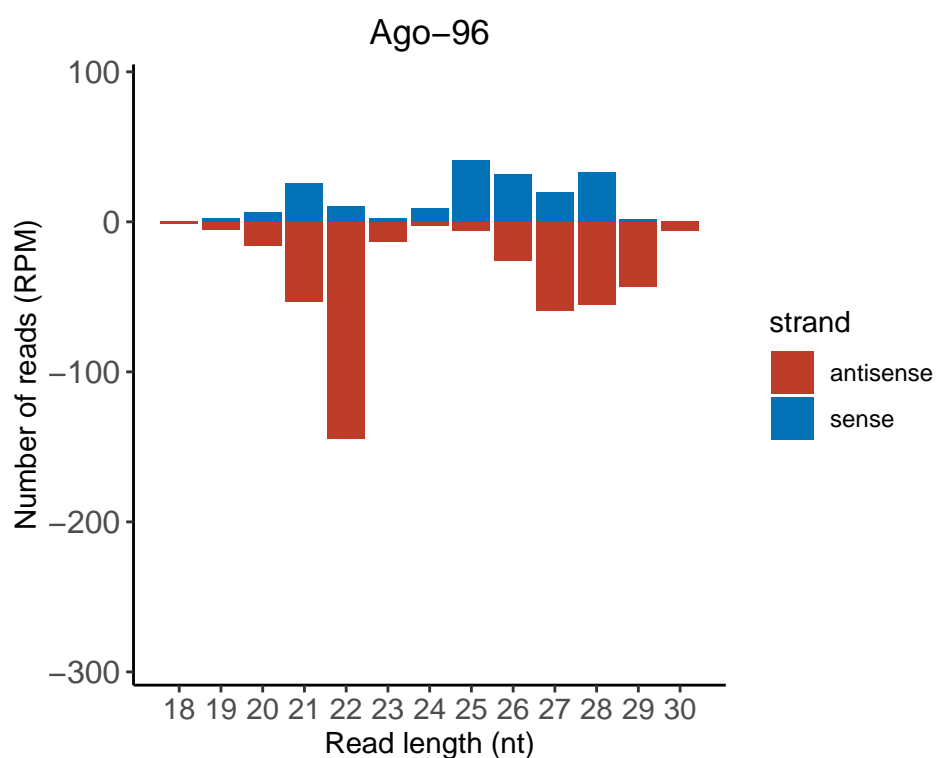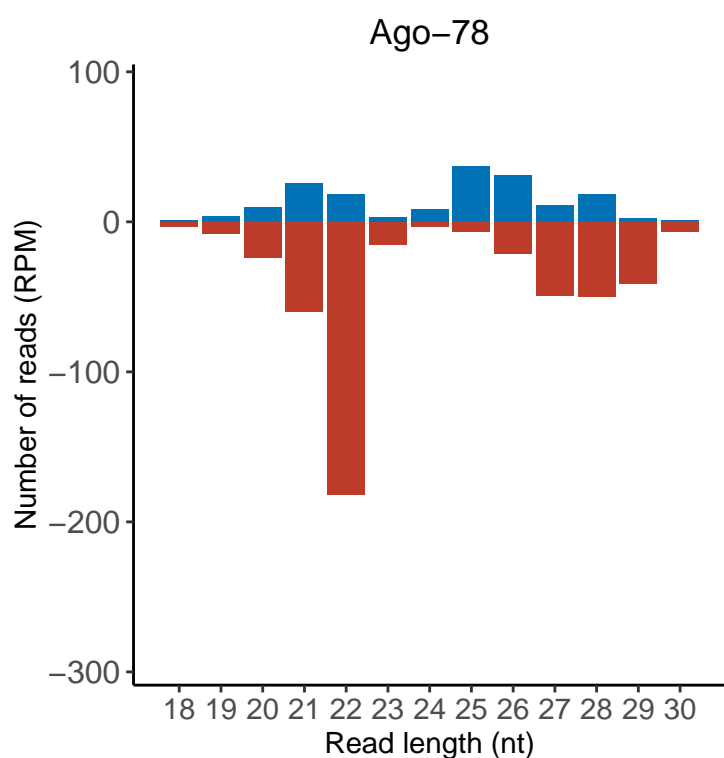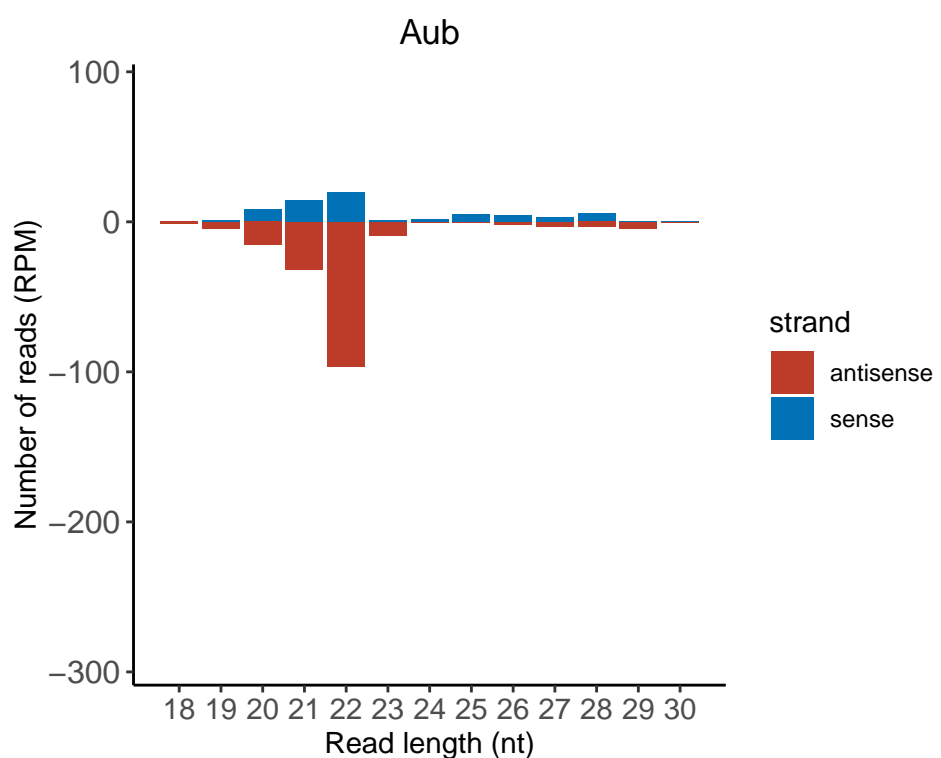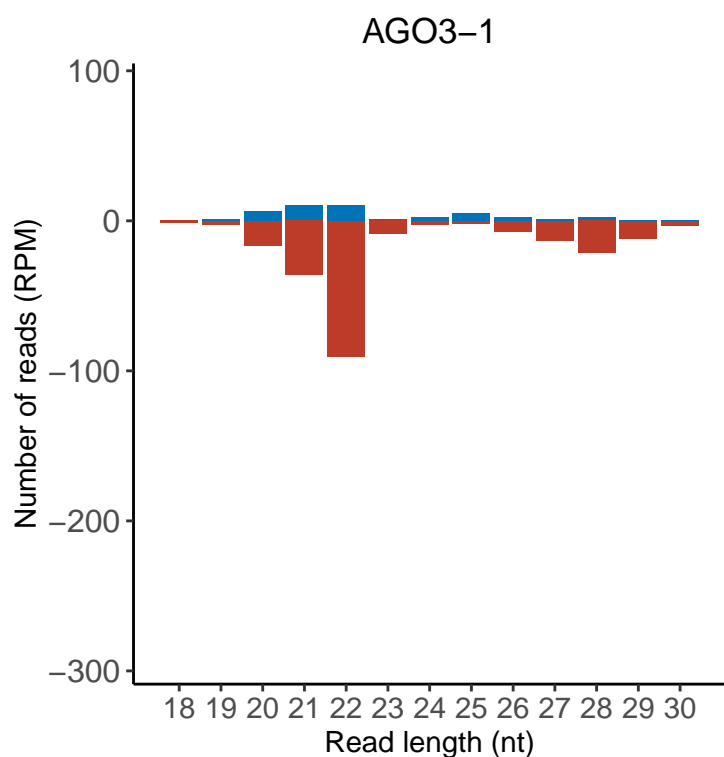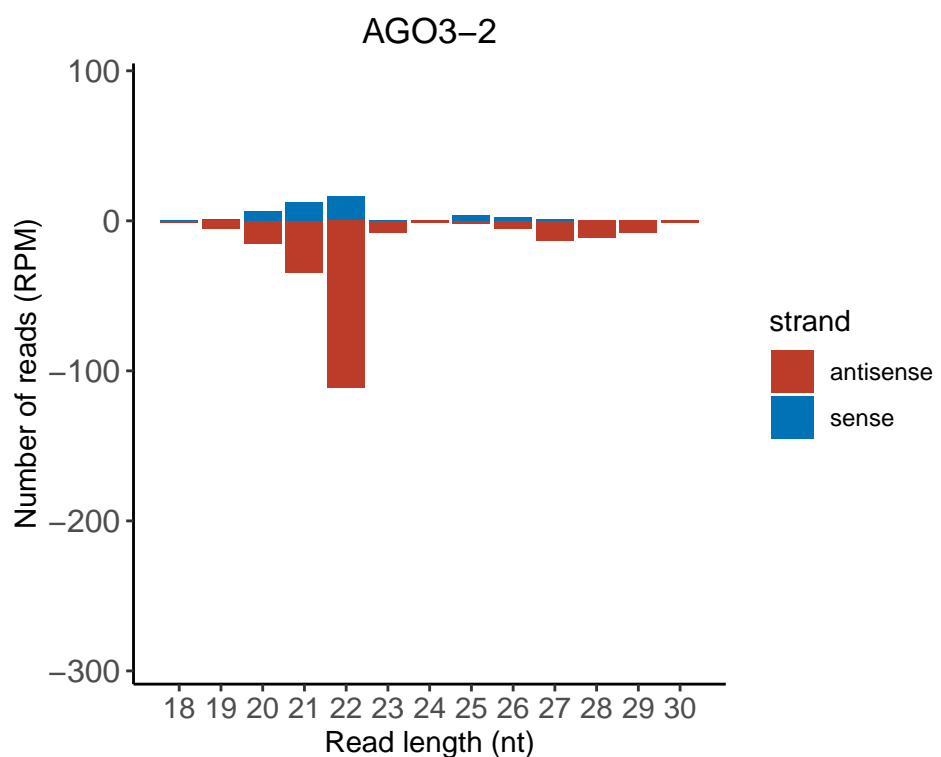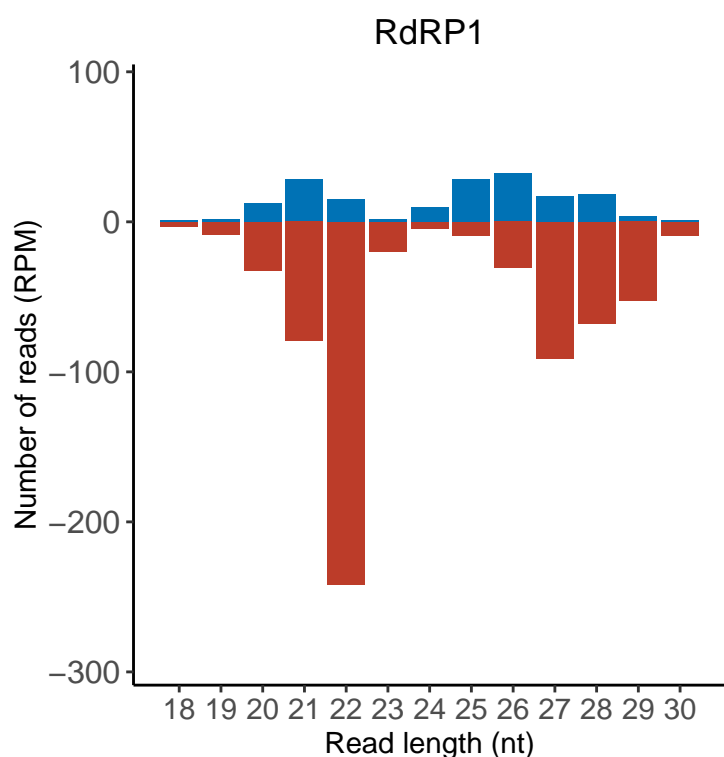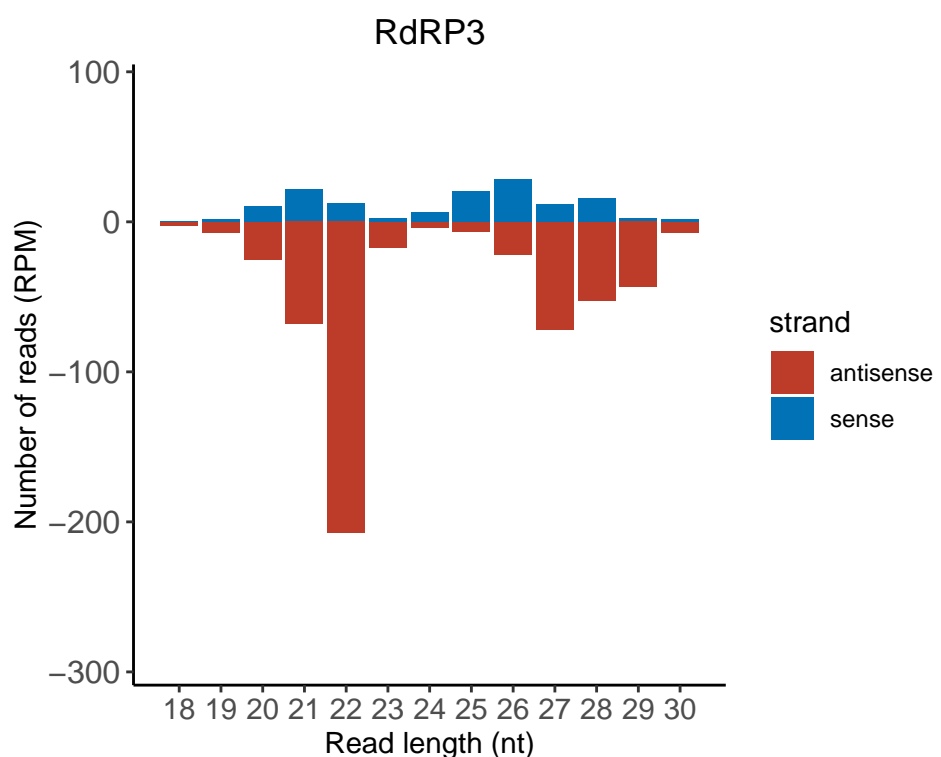

Supplement: S1 Data — On the Normalized and Relative levels pages, normalized read counts (RPM) and relative levels compared to the control (dsGFP) library were used. TEs with more than 50RPM and Coding Genes with more than 3.5RPM on average in KD libraries are included in this website. For viral sequences, reference sequences were generated by assembling sRNA sequences (See the Analysis of sRNAseq data section in Materials and methods.) and size distributions of sRNA reads mapped to each contig are shown. Reads were first grouped by their 5’ nucleotides, and reads of each length were counted. Full tables including TEs and Coding genes with fewer reads are in the “FullTable” folder. (ZIP) [file pone.0281195.s017.zip › SupplementaryData/Links/PDFs/Motif:rnd-6_family-8871TE_lengthdistribution.pdf]

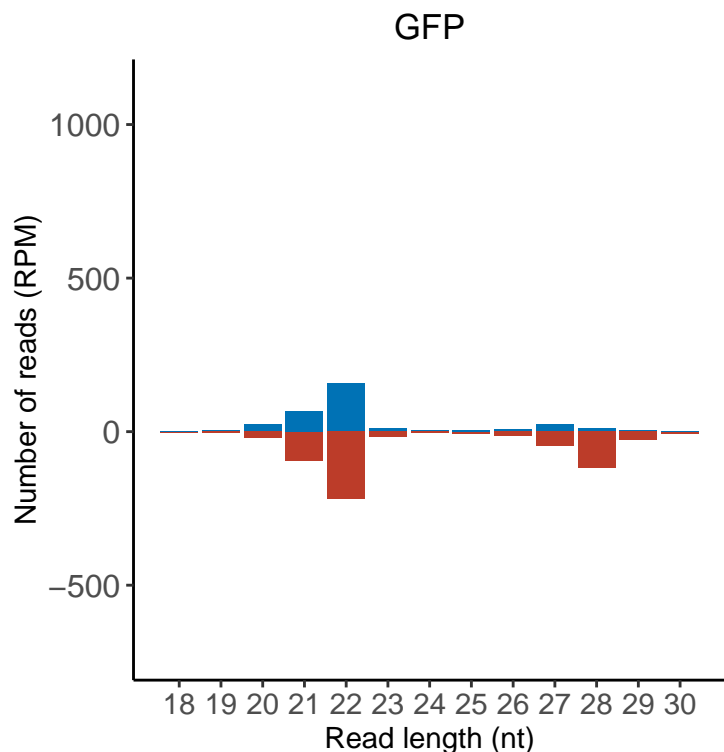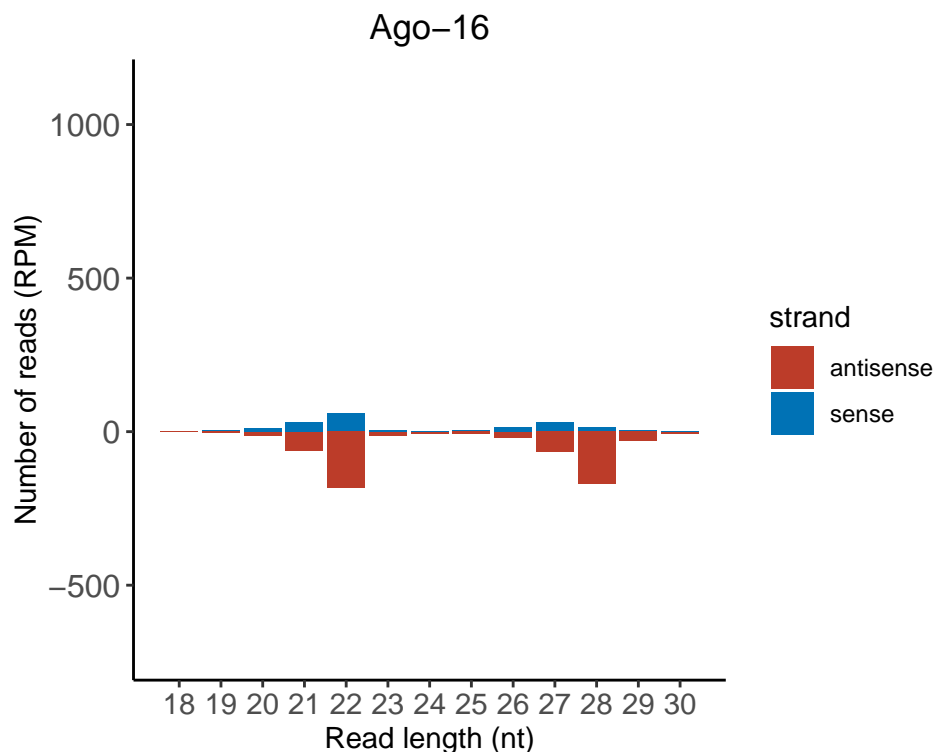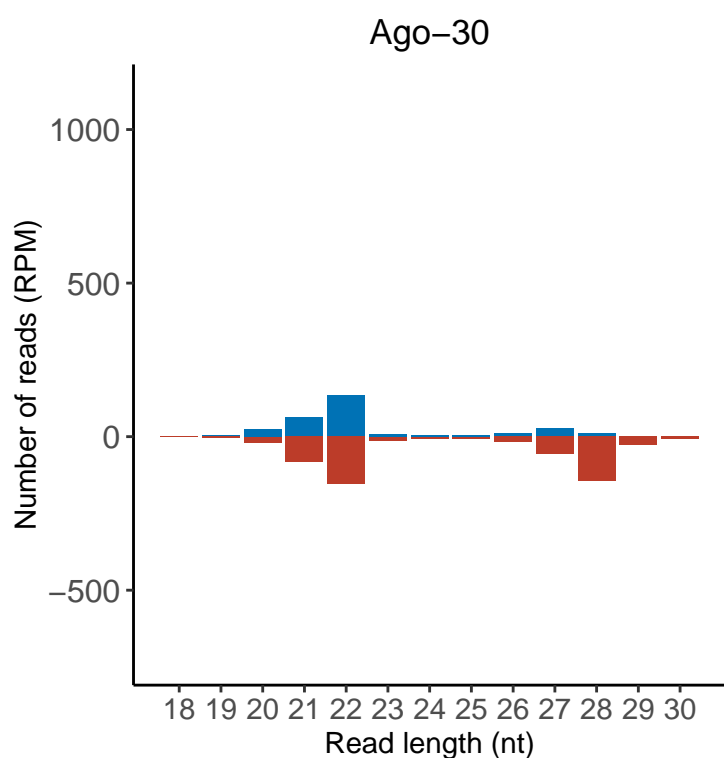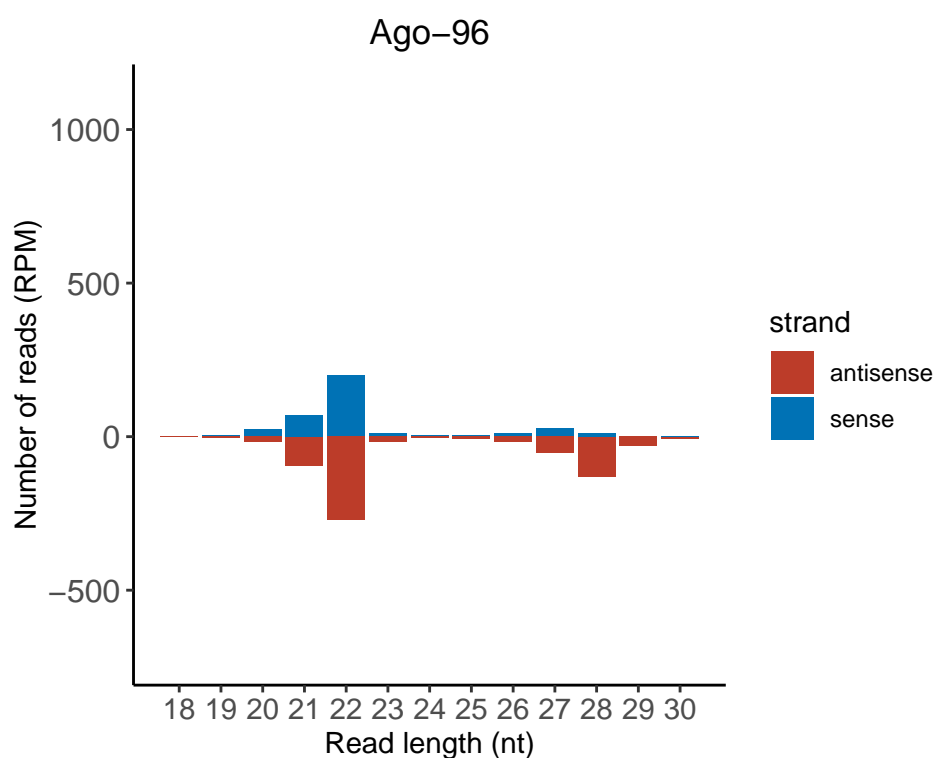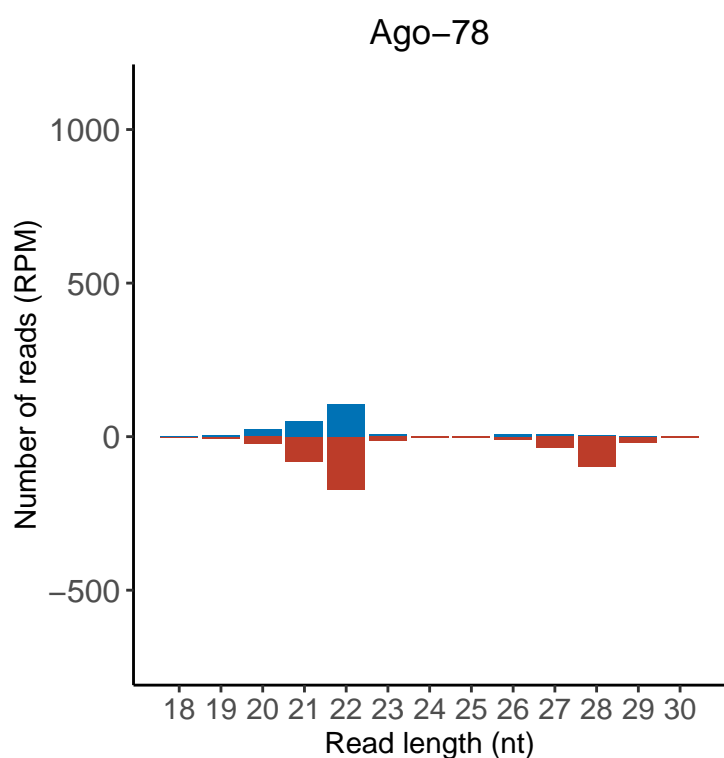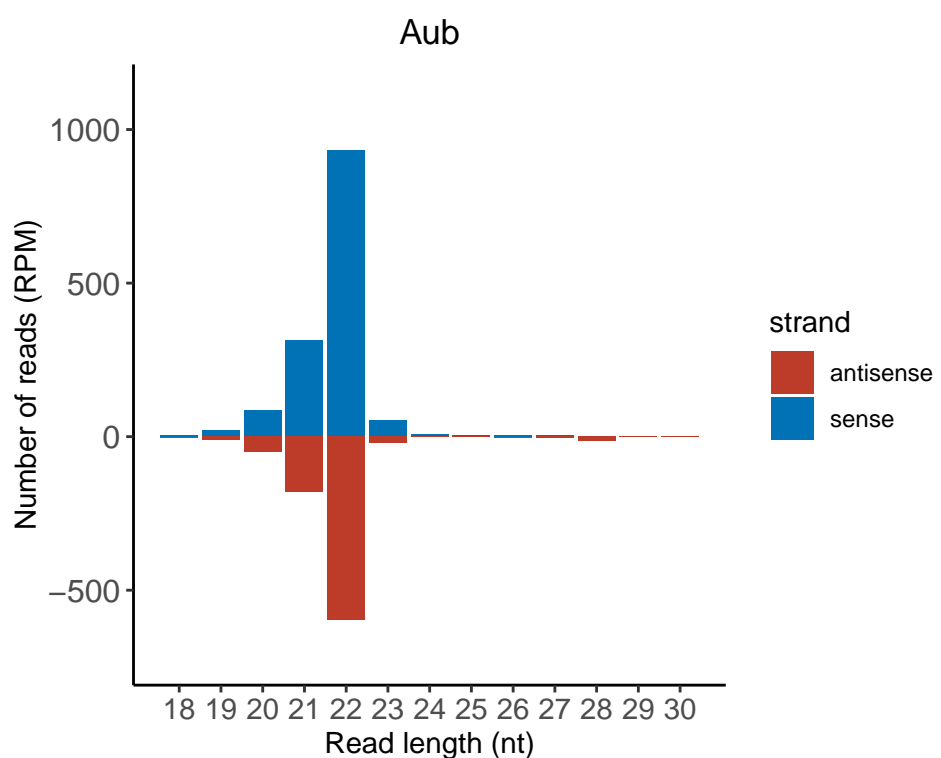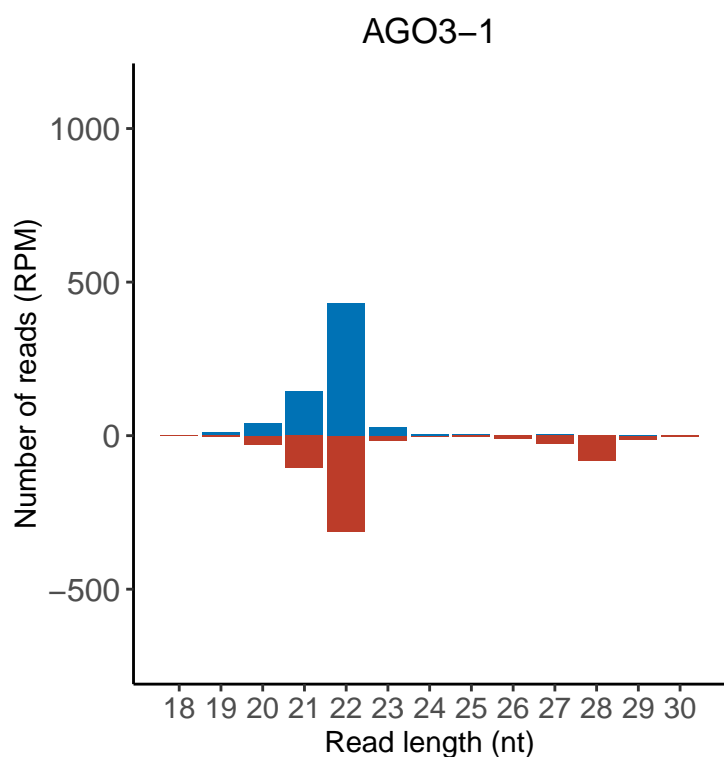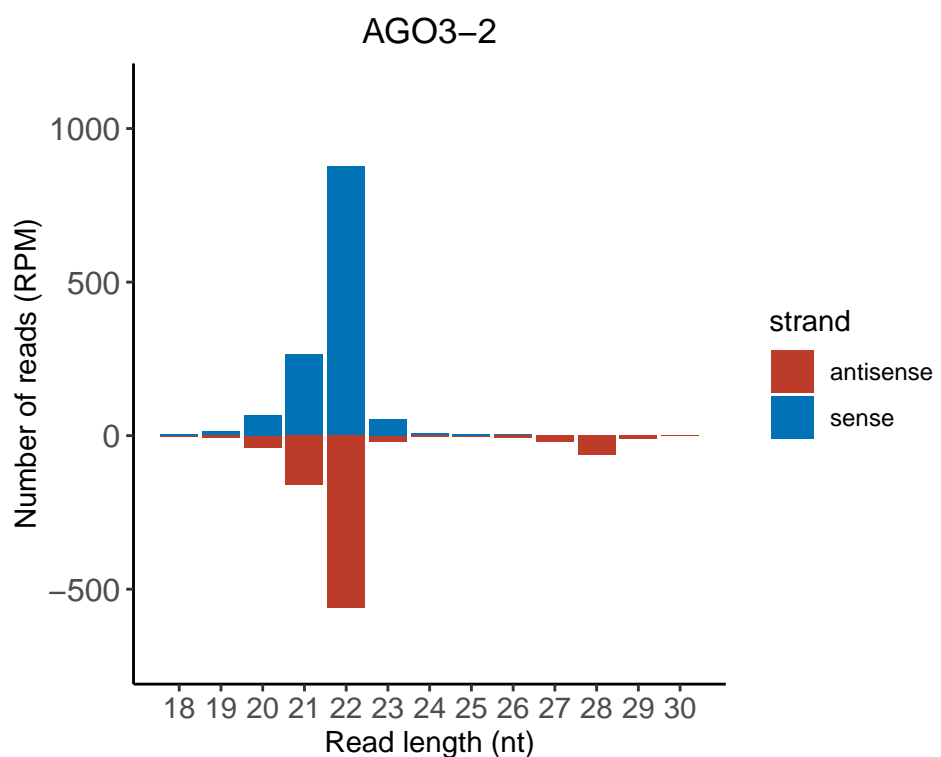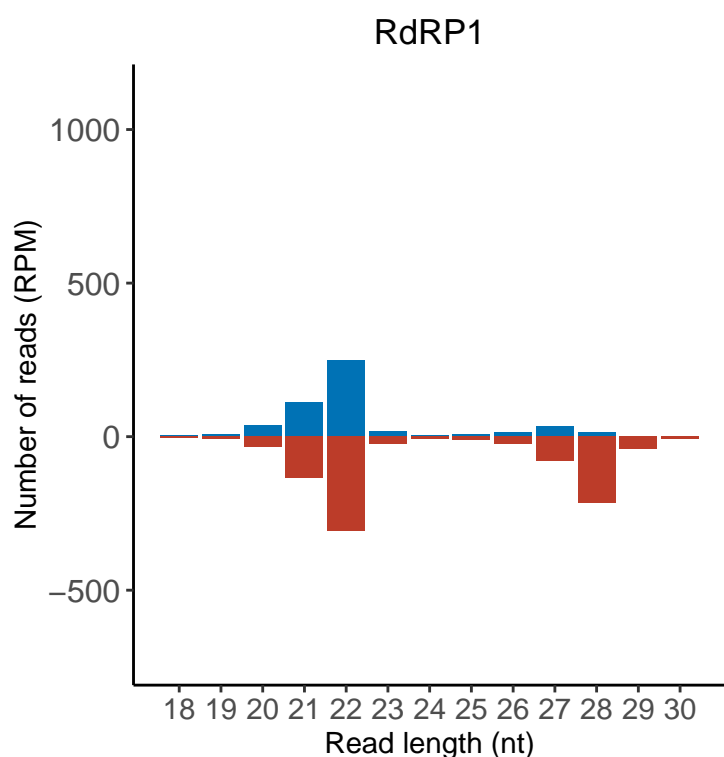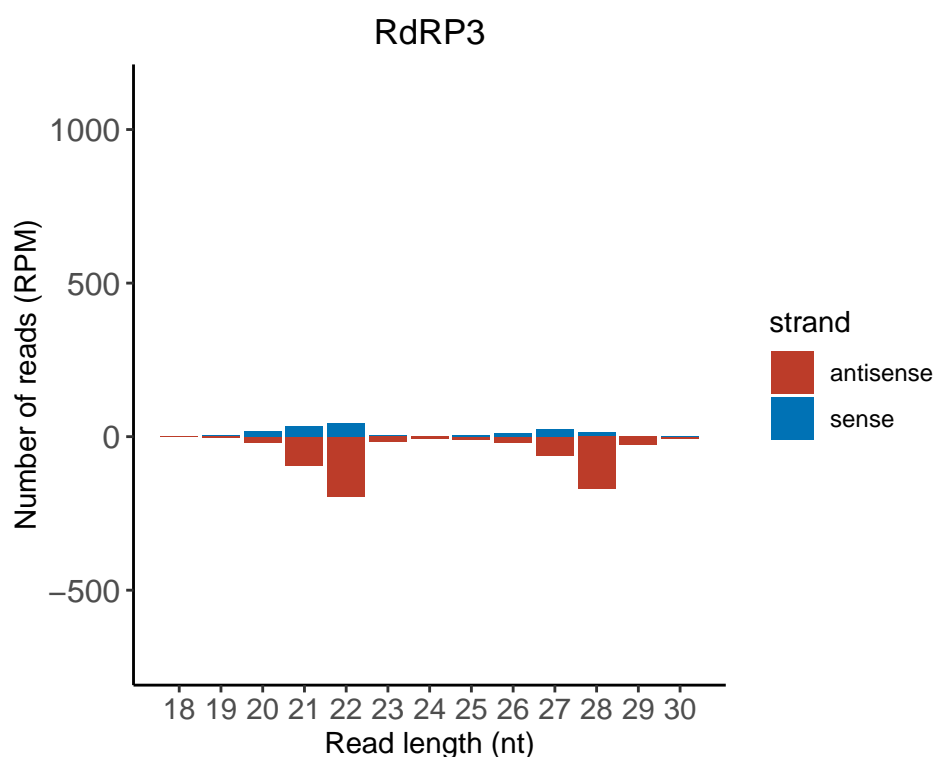

Supplement: S1 Data — On the Normalized and Relative levels pages, normalized read counts (RPM) and relative levels compared to the control (dsGFP) library were used. TEs with more than 50RPM and Coding Genes with more than 3.5RPM on average in KD libraries are included in this website. For viral sequences, reference sequences were generated by assembling sRNA sequences (See the Analysis of sRNAseq data section in Materials and methods.) and size distributions of sRNA reads mapped to each contig are shown. Reads were first grouped by their 5’ nucleotides, and reads of each length were counted. Full tables including TEs and Coding genes with fewer reads are in the “FullTable” folder. (ZIP) [file pone.0281195.s017.zip › SupplementaryData/Links/PDFs/Motif:rnd-5_family-2803TE_lengthdistribution.pdf]

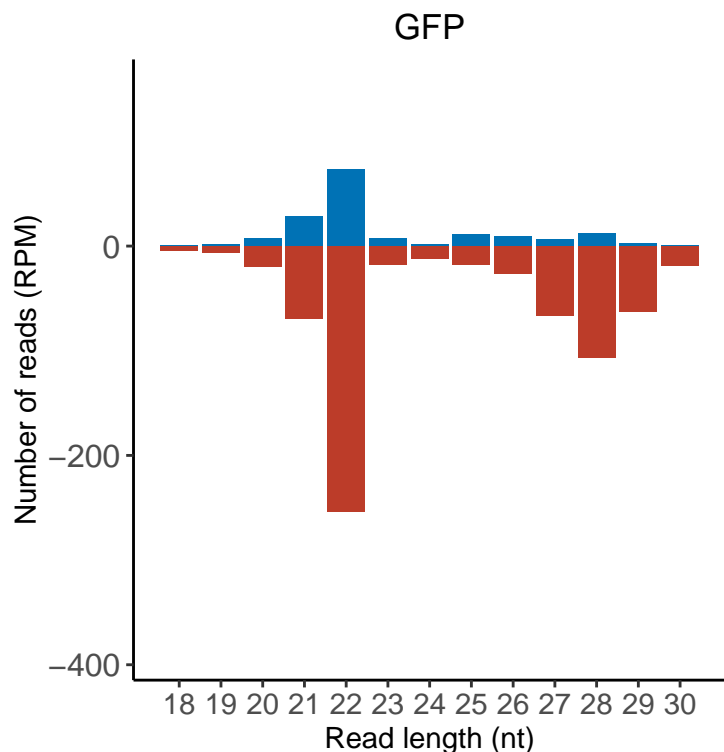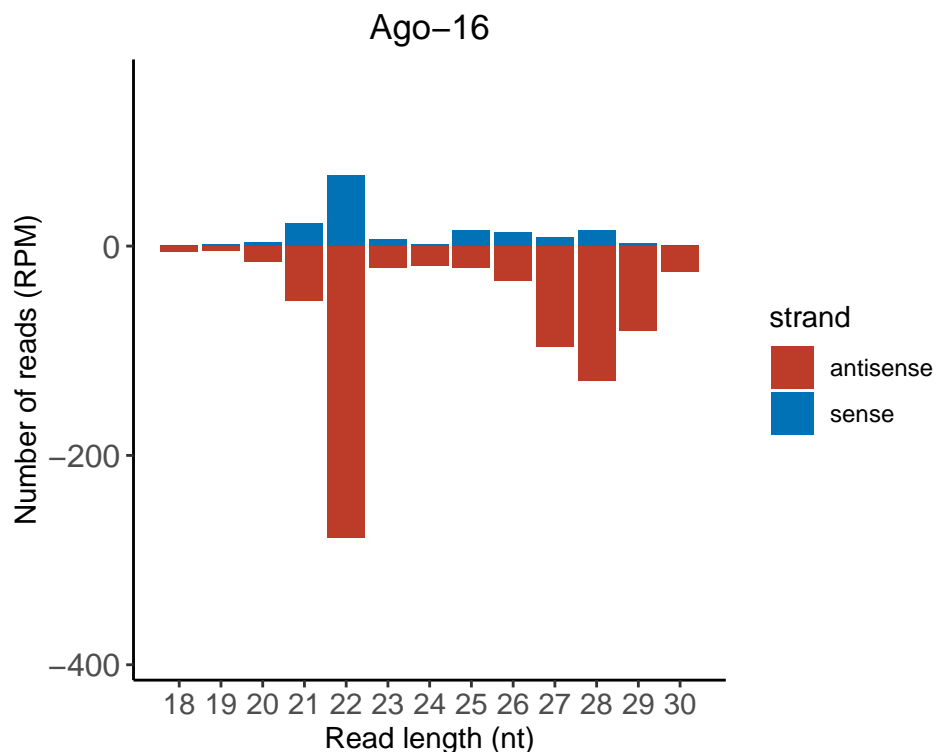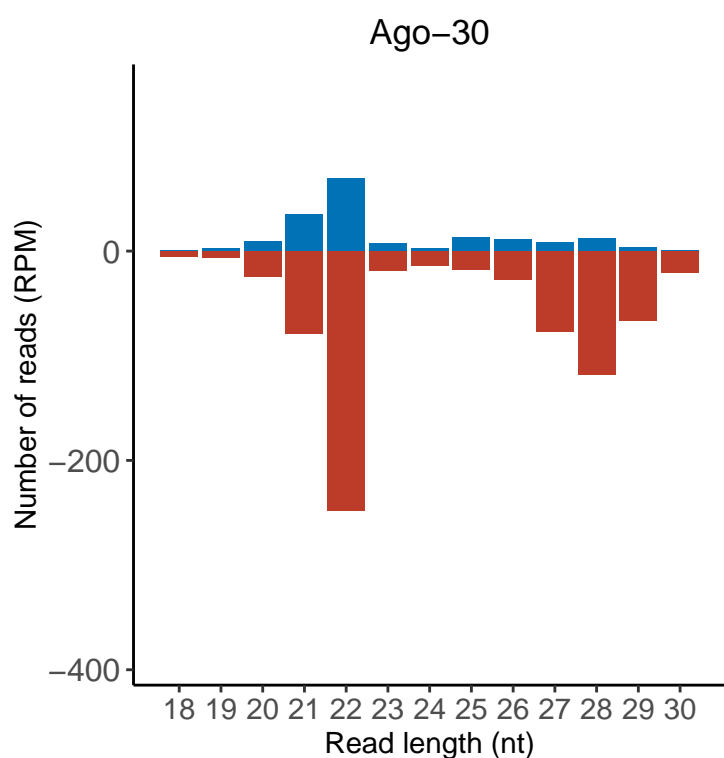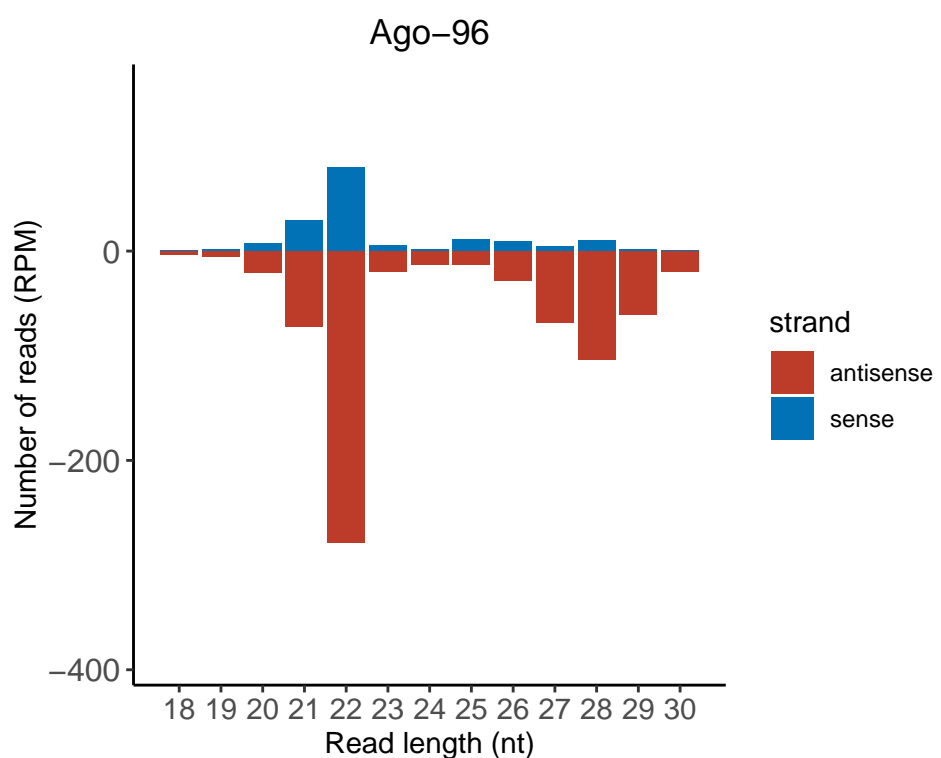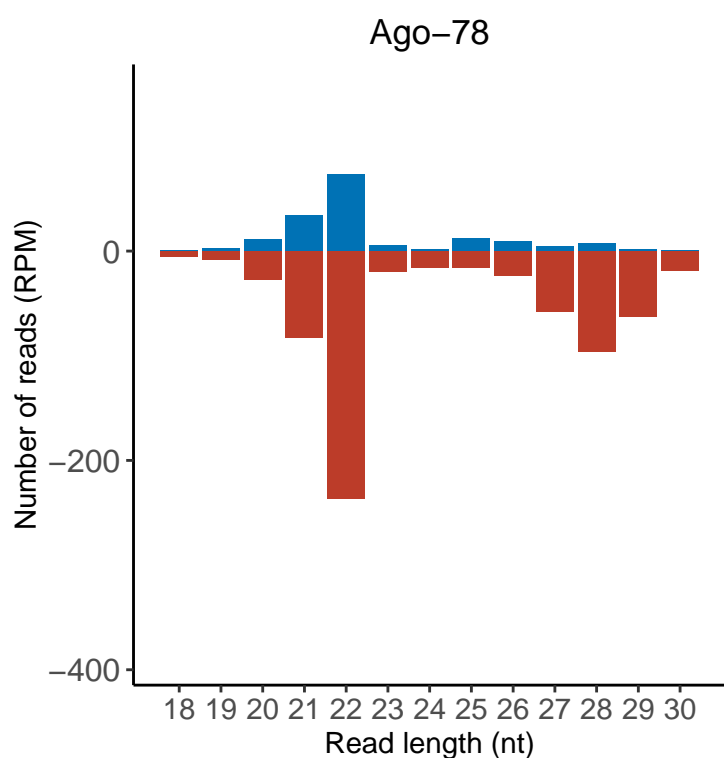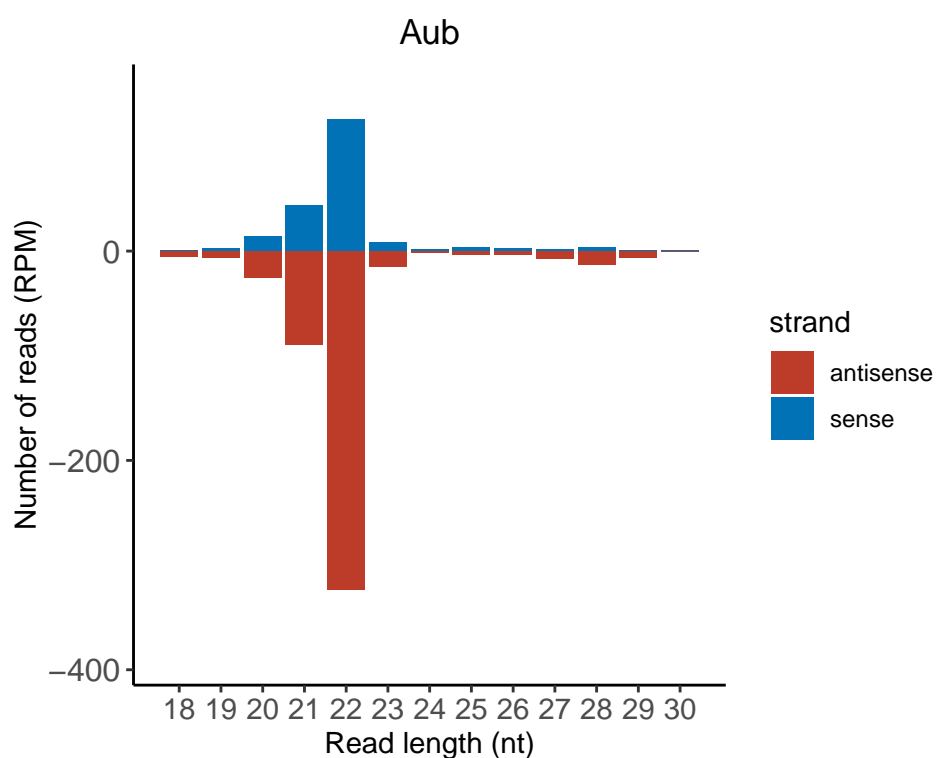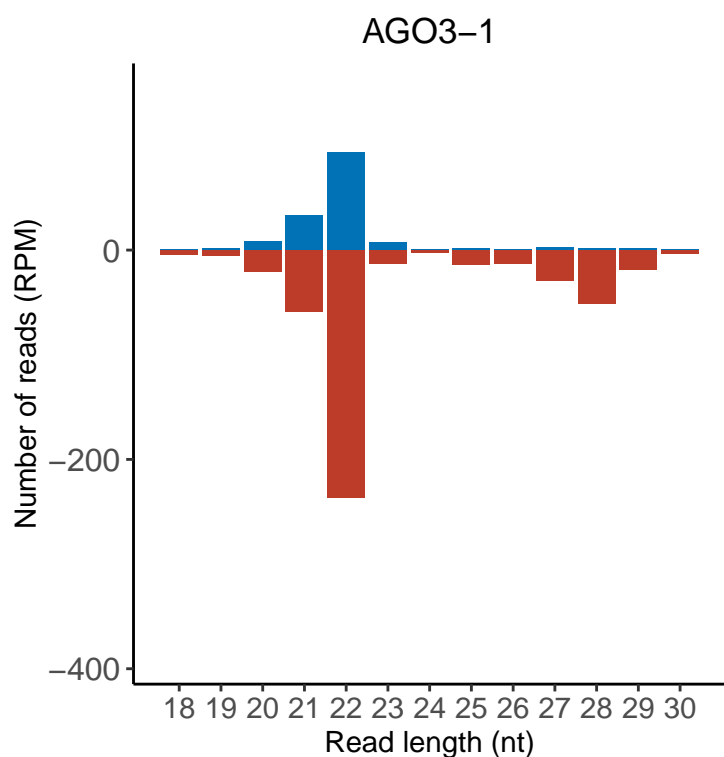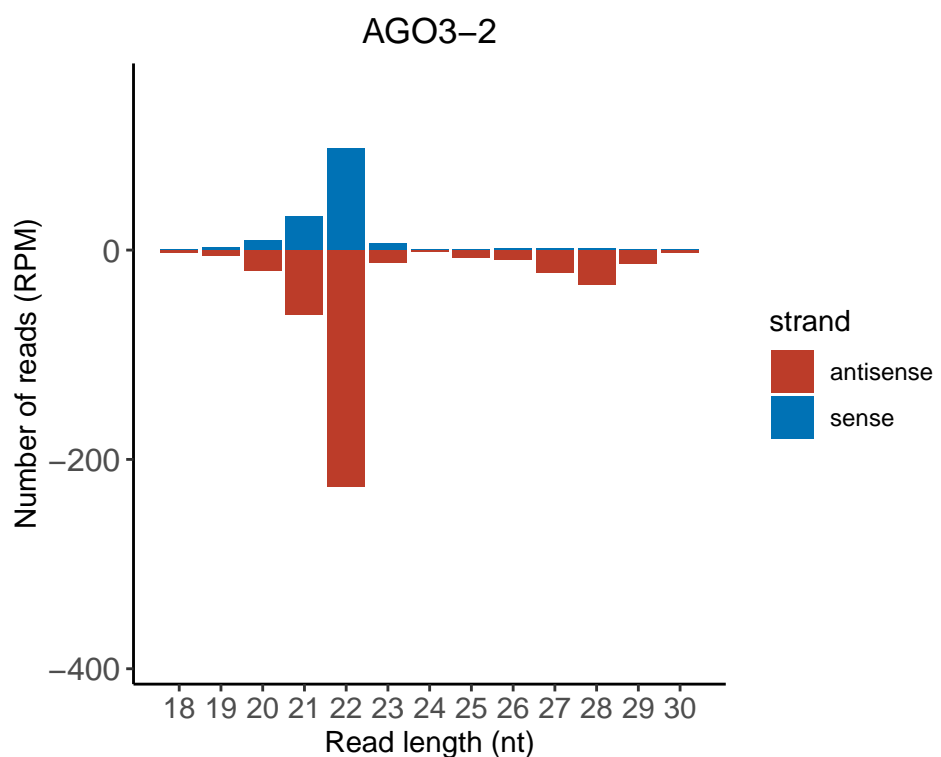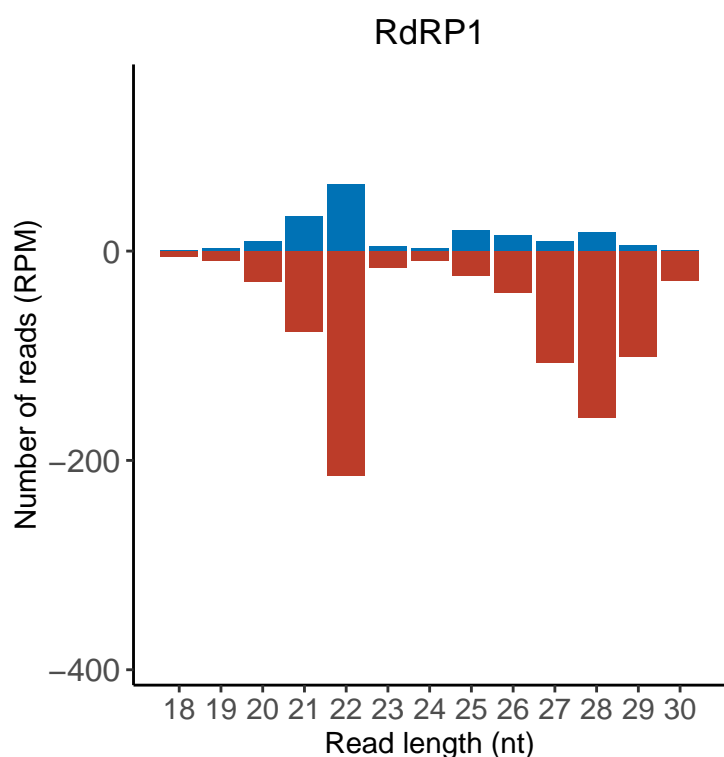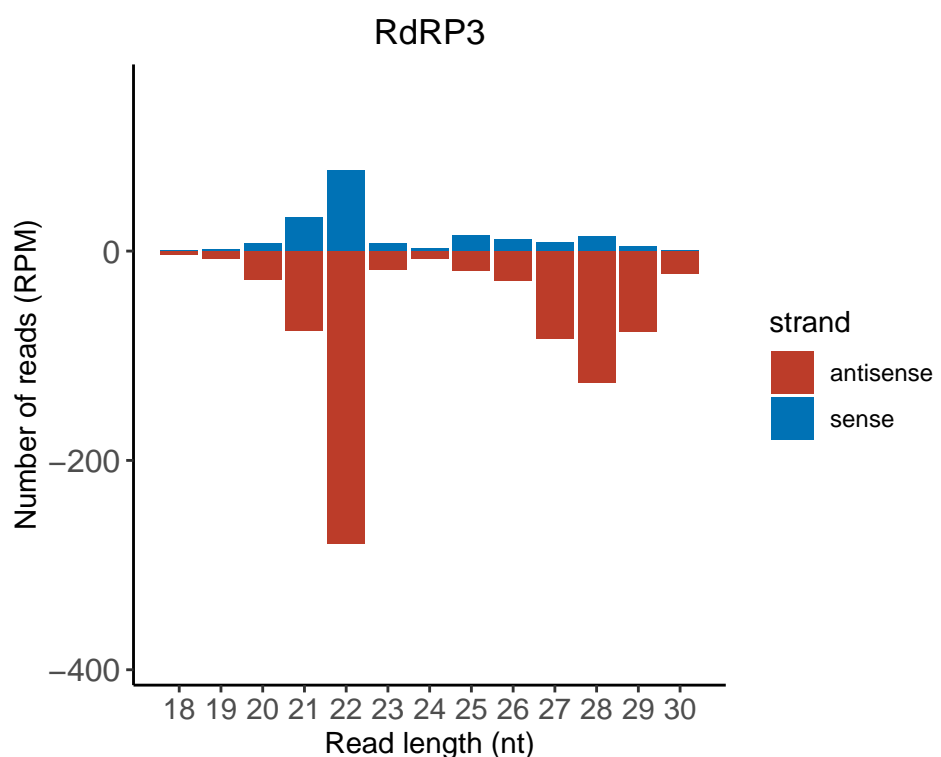

Supplement: S1 Data — On the Normalized and Relative levels pages, normalized read counts (RPM) and relative levels compared to the control (dsGFP) library were used. TEs with more than 50RPM and Coding Genes with more than 3.5RPM on average in KD libraries are included in this website. For viral sequences, reference sequences were generated by assembling sRNA sequences (See the Analysis of sRNAseq data section in Materials and methods.) and size distributions of sRNA reads mapped to each contig are shown. Reads were first grouped by their 5’ nucleotides, and reads of each length were counted. Full tables including TEs and Coding genes with fewer reads are in the “FullTable” folder. (ZIP) [file pone.0281195.s017.zip › SupplementaryData/Links/PDFs/Motif:rnd-1_family-9TE_lengthdistribution.pdf]

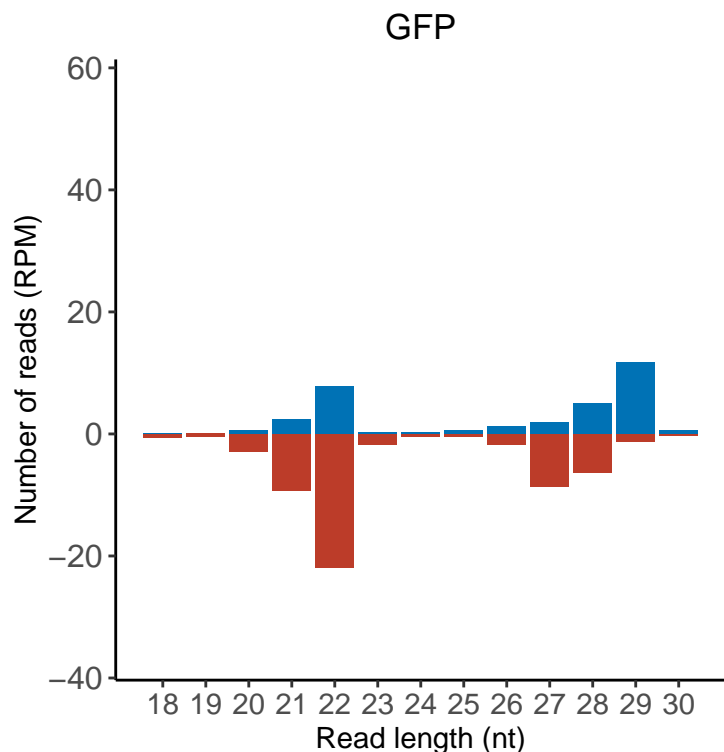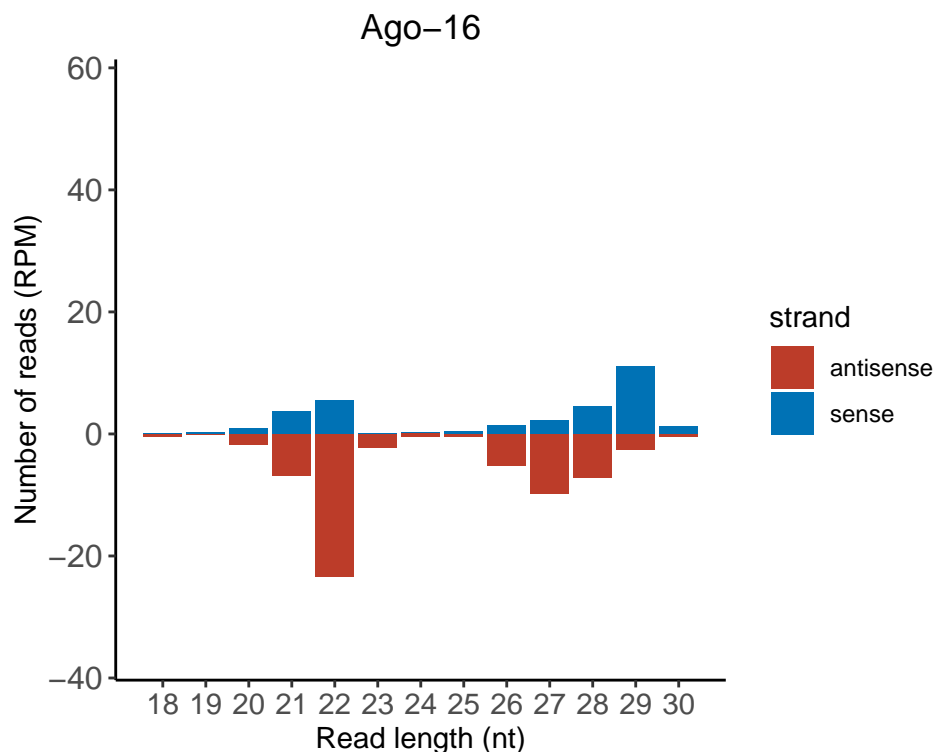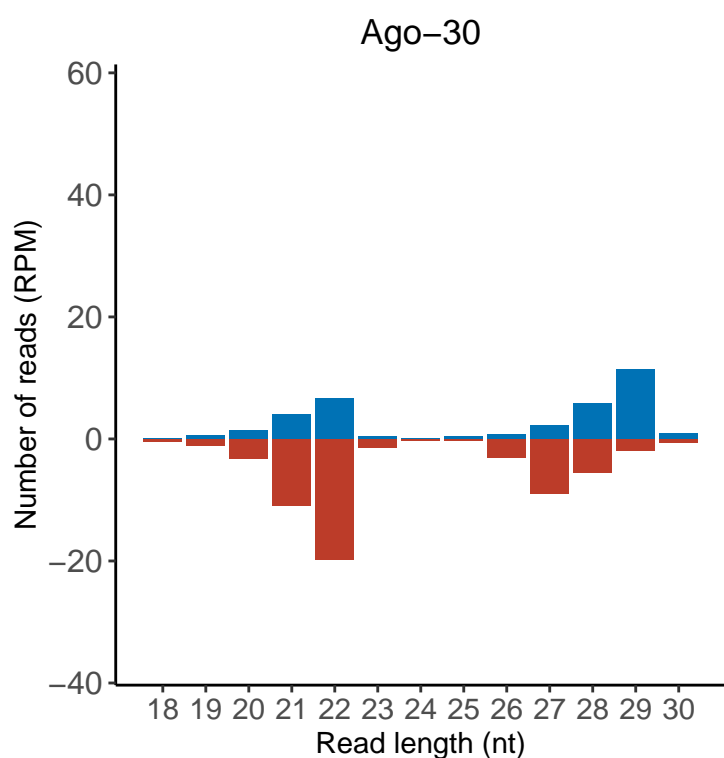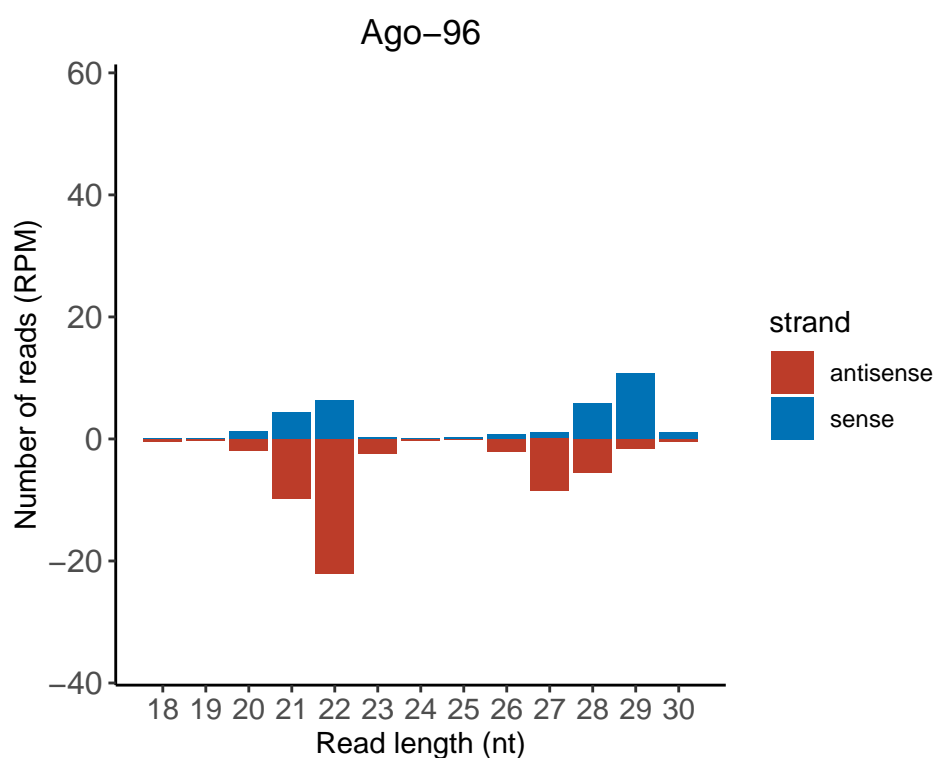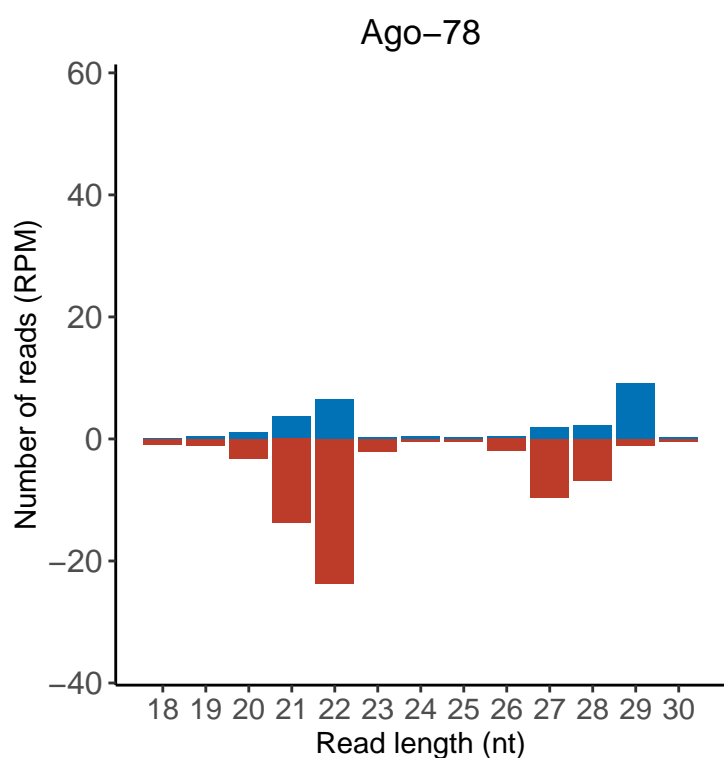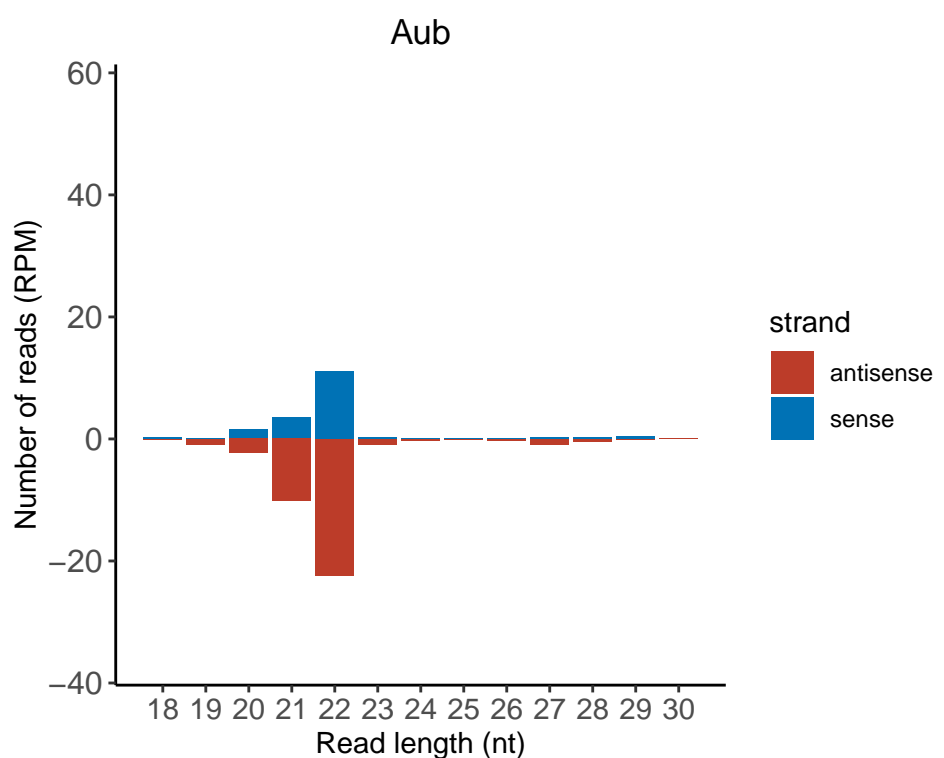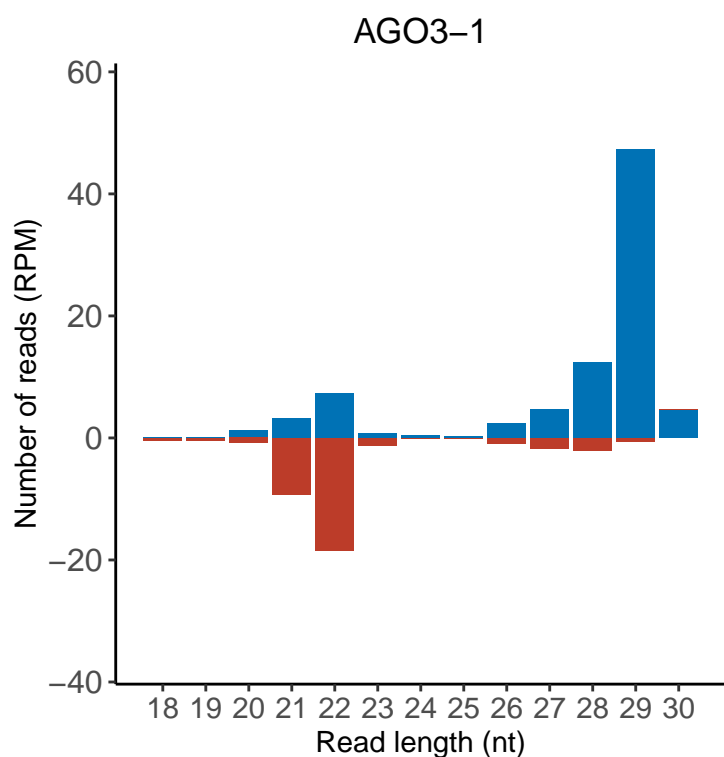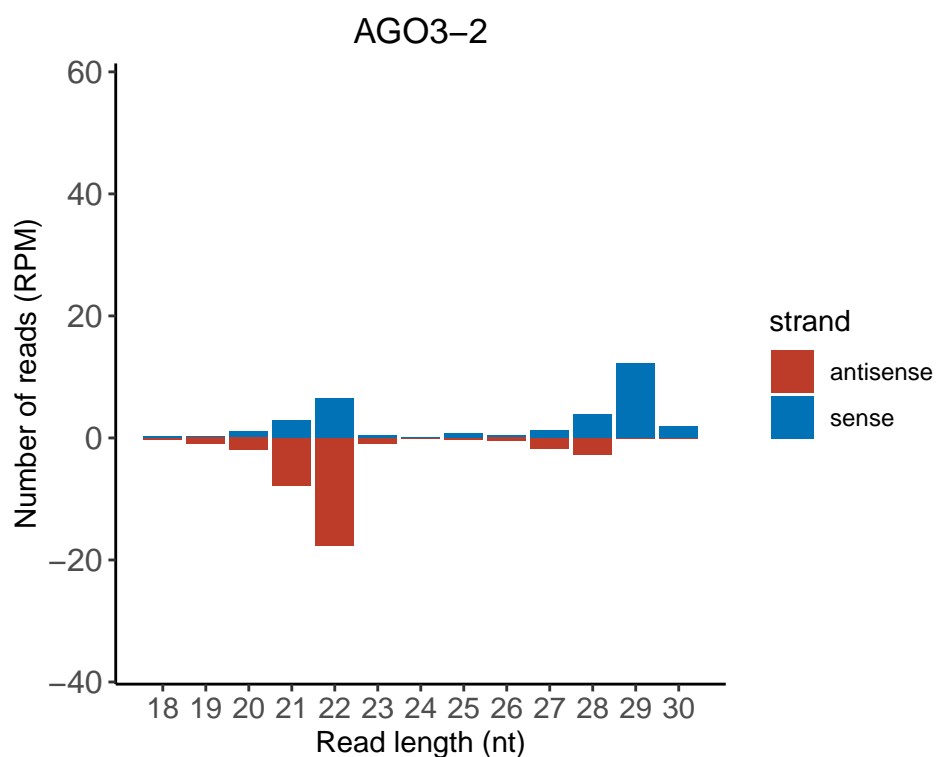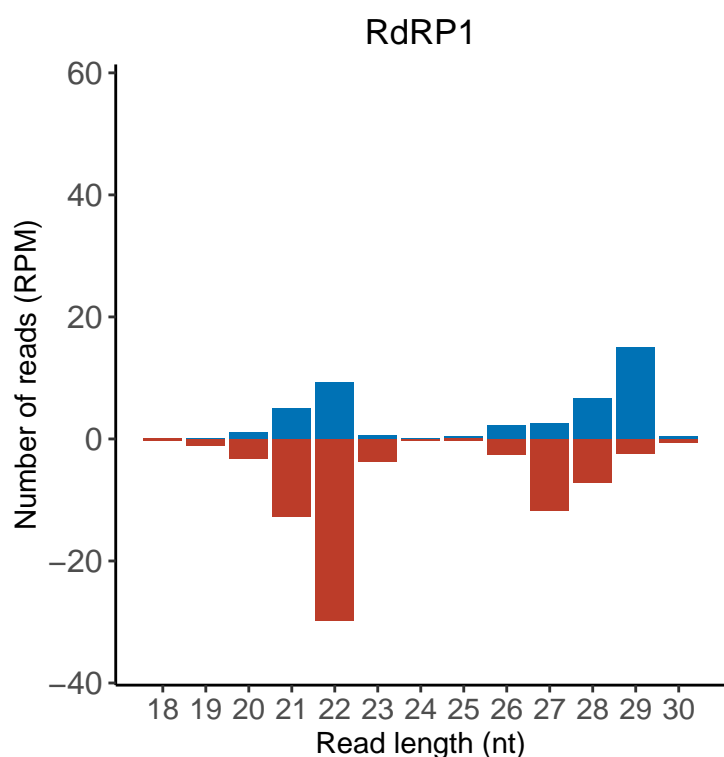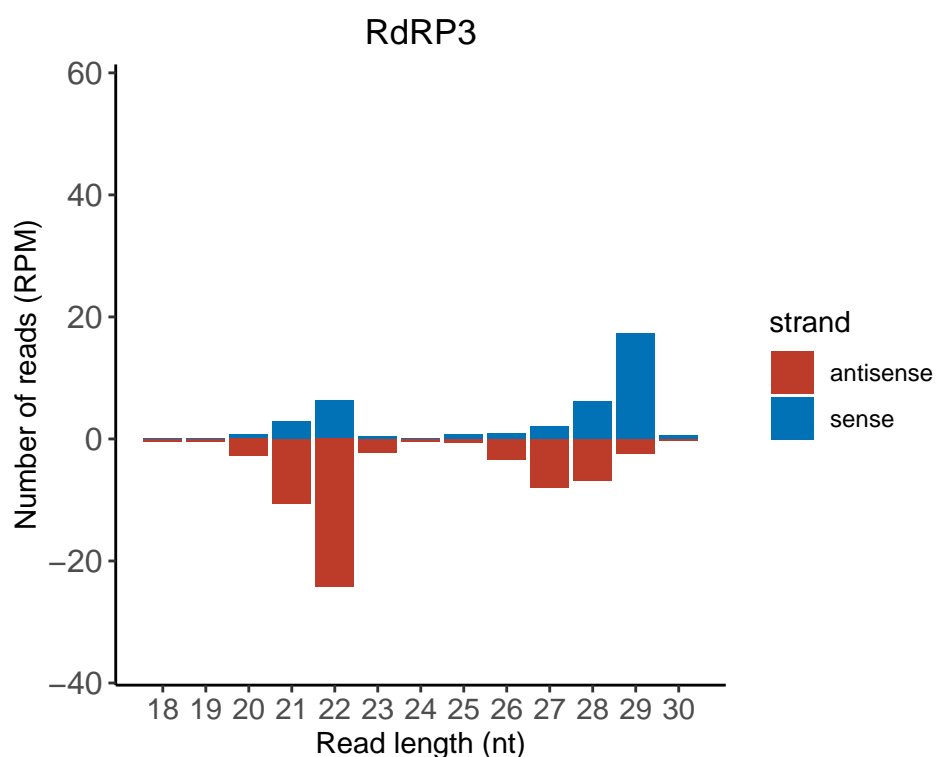

Supplement: S1 Data — On the Normalized and Relative levels pages, normalized read counts (RPM) and relative levels compared to the control (dsGFP) library were used. TEs with more than 50RPM and Coding Genes with more than 3.5RPM on average in KD libraries are included in this website. For viral sequences, reference sequences were generated by assembling sRNA sequences (See the Analysis of sRNAseq data section in Materials and methods.) and size distributions of sRNA reads mapped to each contig are shown. Reads were first grouped by their 5’ nucleotides, and reads of each length were counted. Full tables including TEs and Coding genes with fewer reads are in the “FullTable” folder. (ZIP) [file pone.0281195.s017.zip › SupplementaryData/Links/PDFs/Motif:rnd-1_family-970TE_lengthdistribution.pdf]

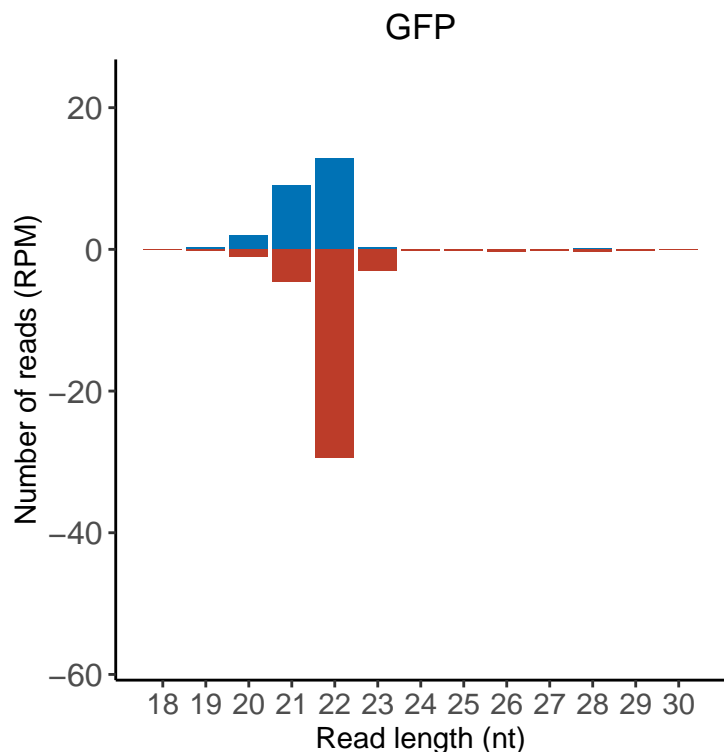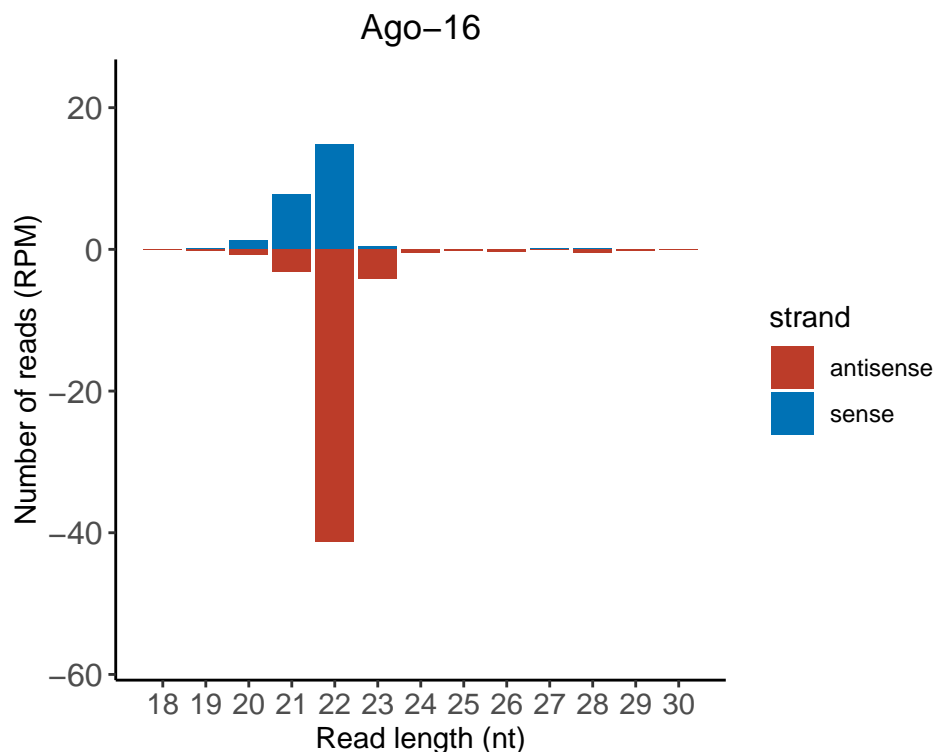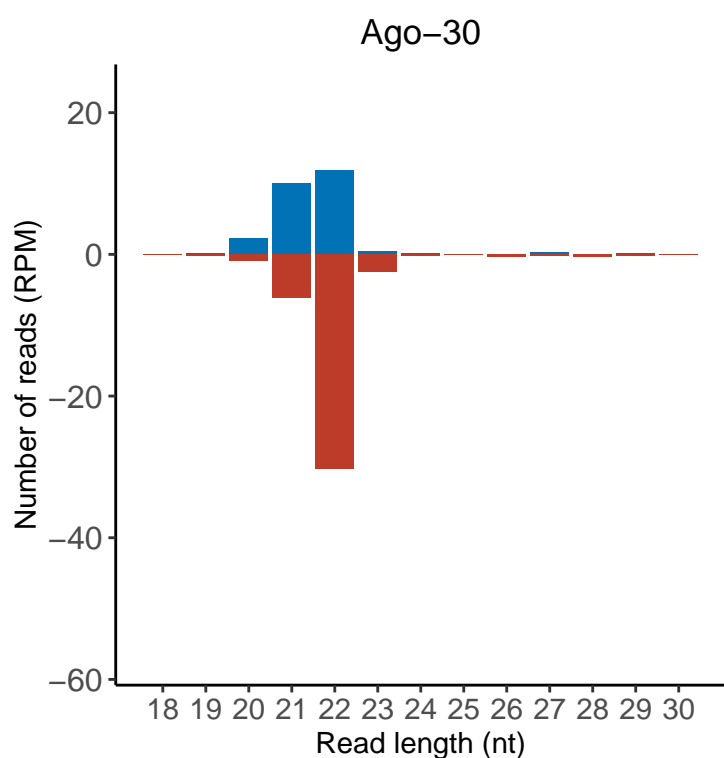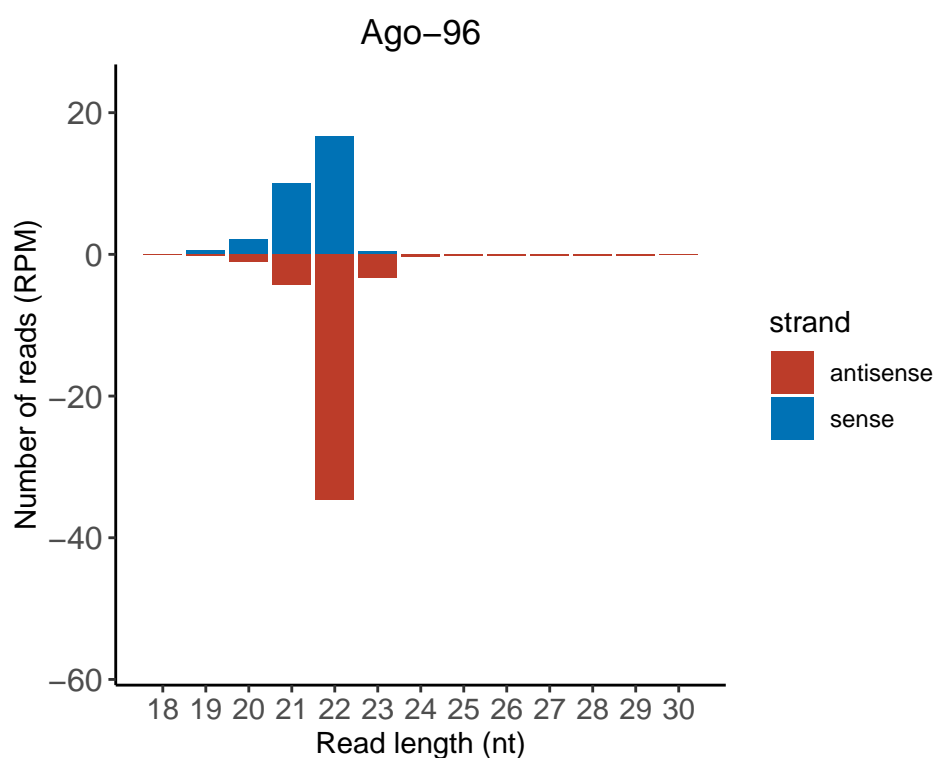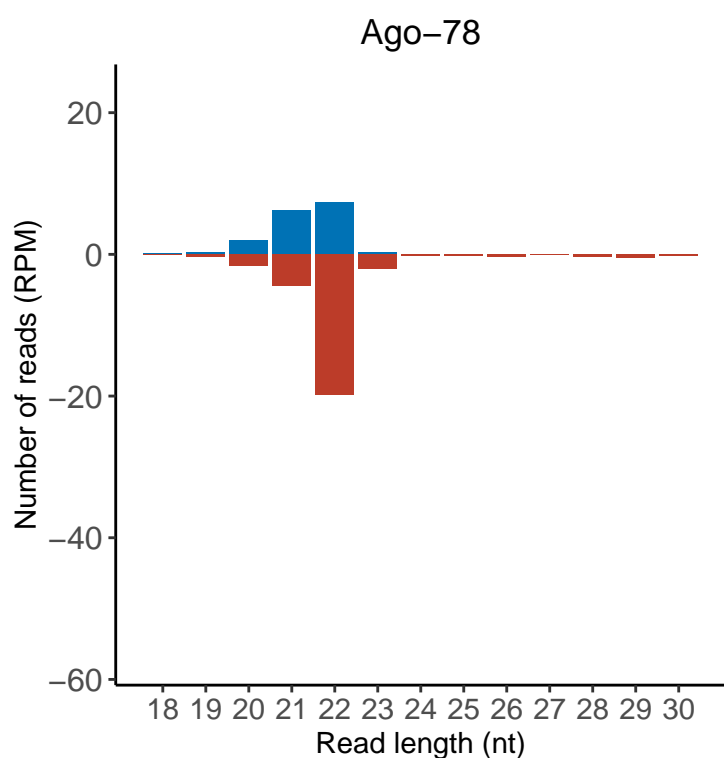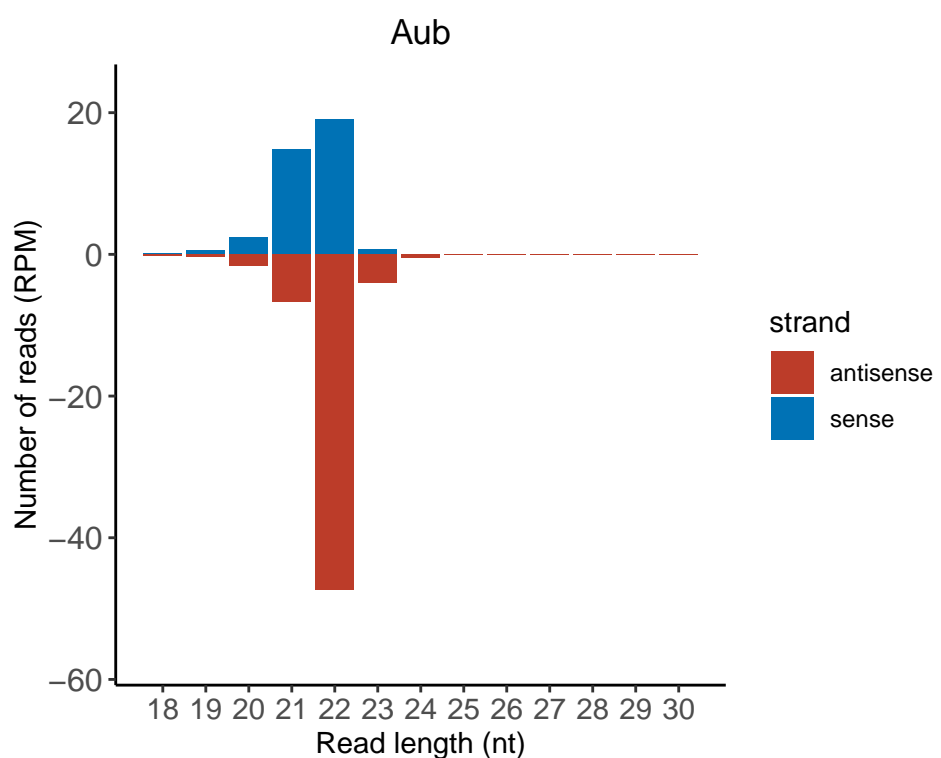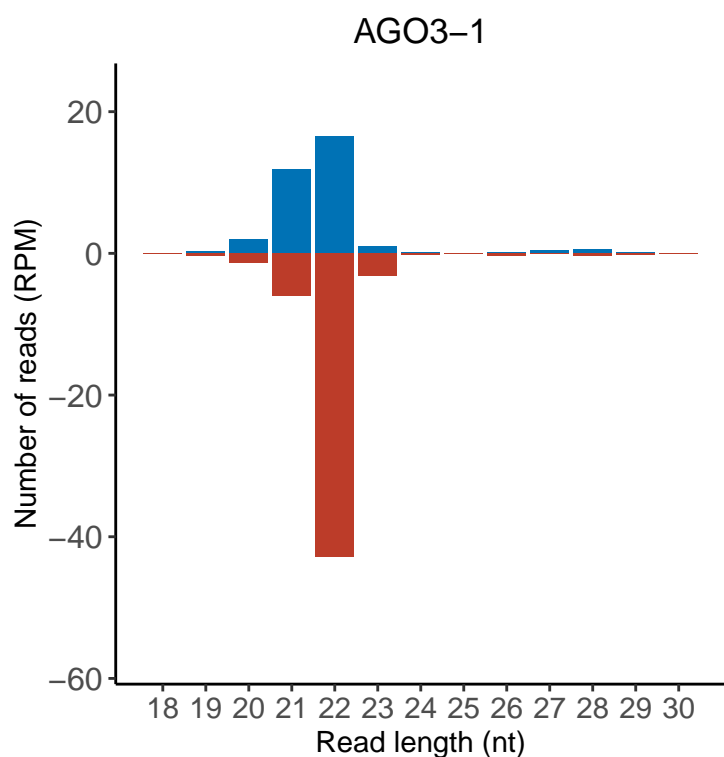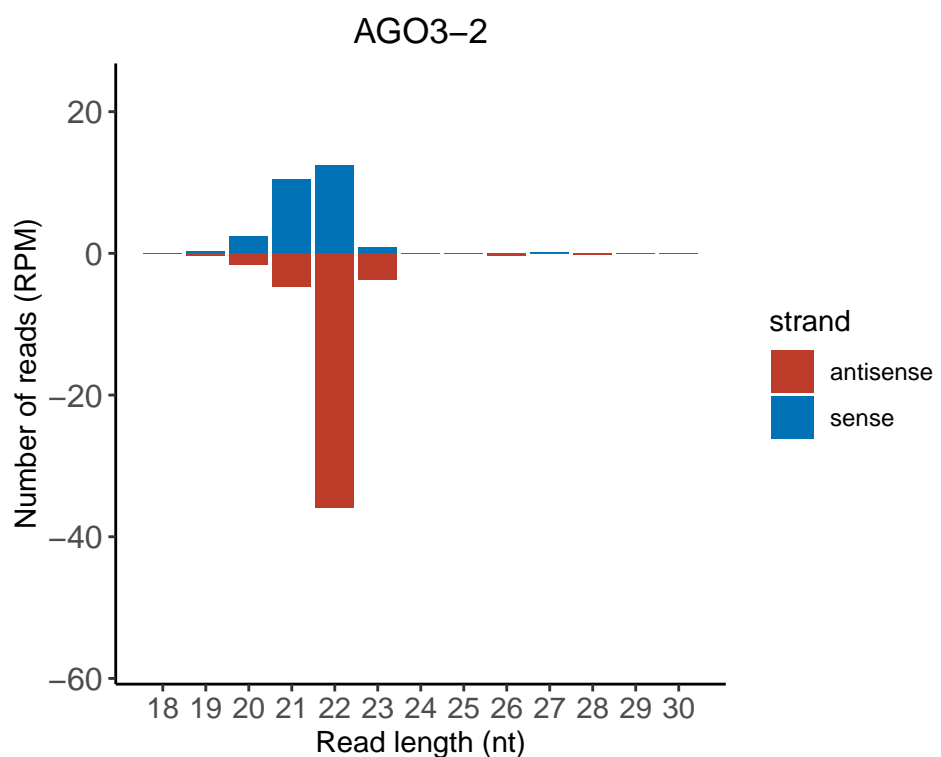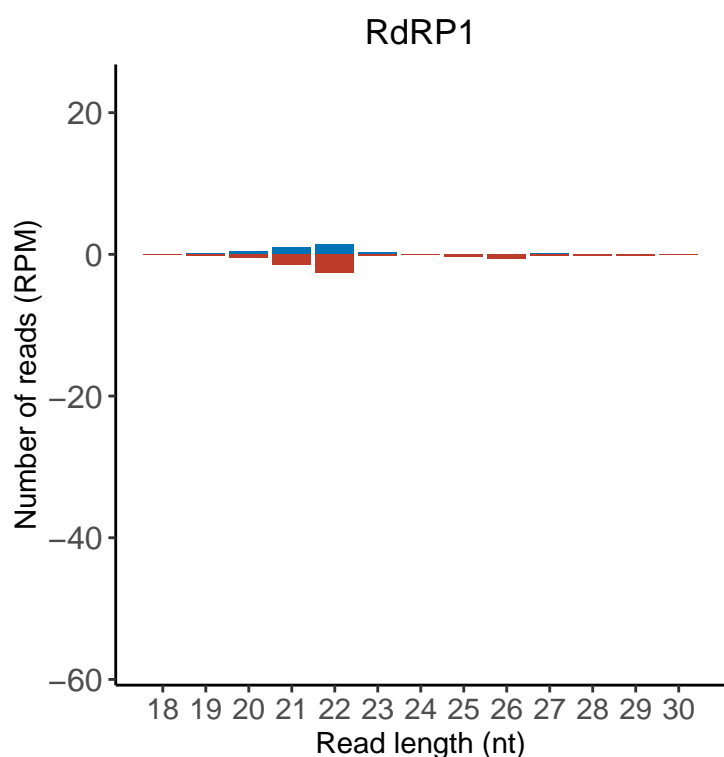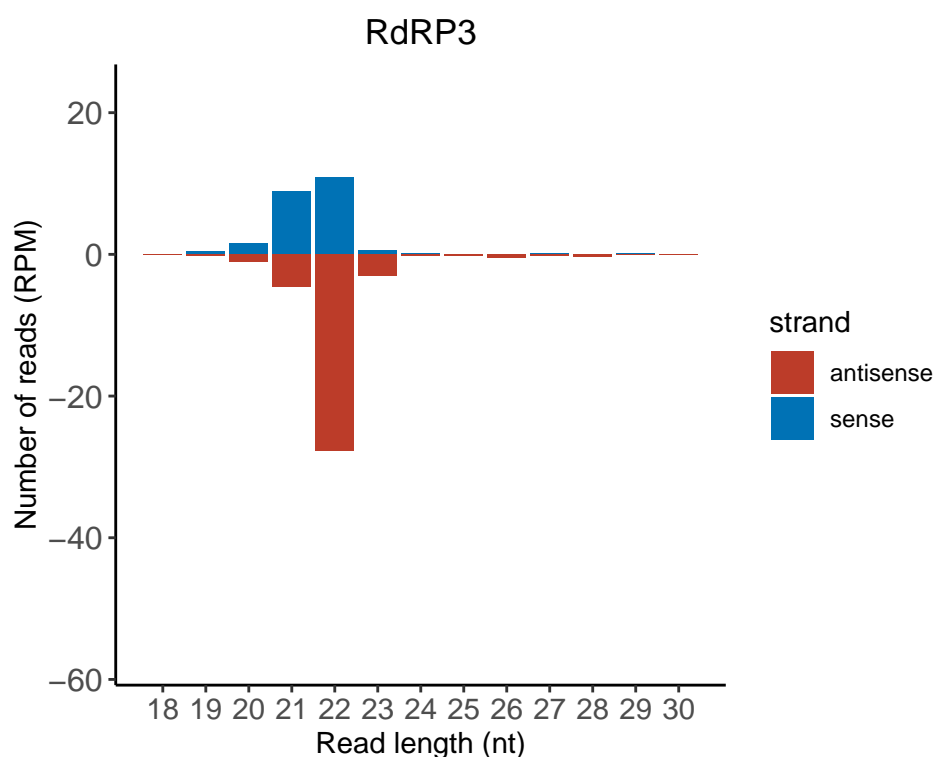

Supplement: S1 Data — On the Normalized and Relative levels pages, normalized read counts (RPM) and relative levels compared to the control (dsGFP) library were used. TEs with more than 50RPM and Coding Genes with more than 3.5RPM on average in KD libraries are included in this website. For viral sequences, reference sequences were generated by assembling sRNA sequences (See the Analysis of sRNAseq data section in Materials and methods.) and size distributions of sRNA reads mapped to each contig are shown. Reads were first grouped by their 5’ nucleotides, and reads of each length were counted. Full tables including TEs and Coding genes with fewer reads are in the “FullTable” folder. (ZIP) [file pone.0281195.s017.zip › SupplementaryData/Links/PDFs/Motif:rnd-1_family-237TE_lengthdistribution.pdf]

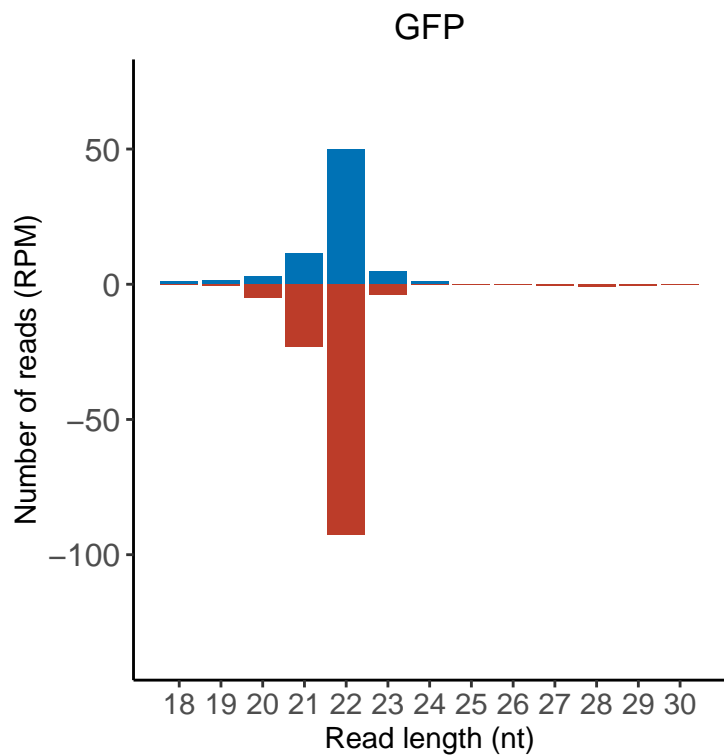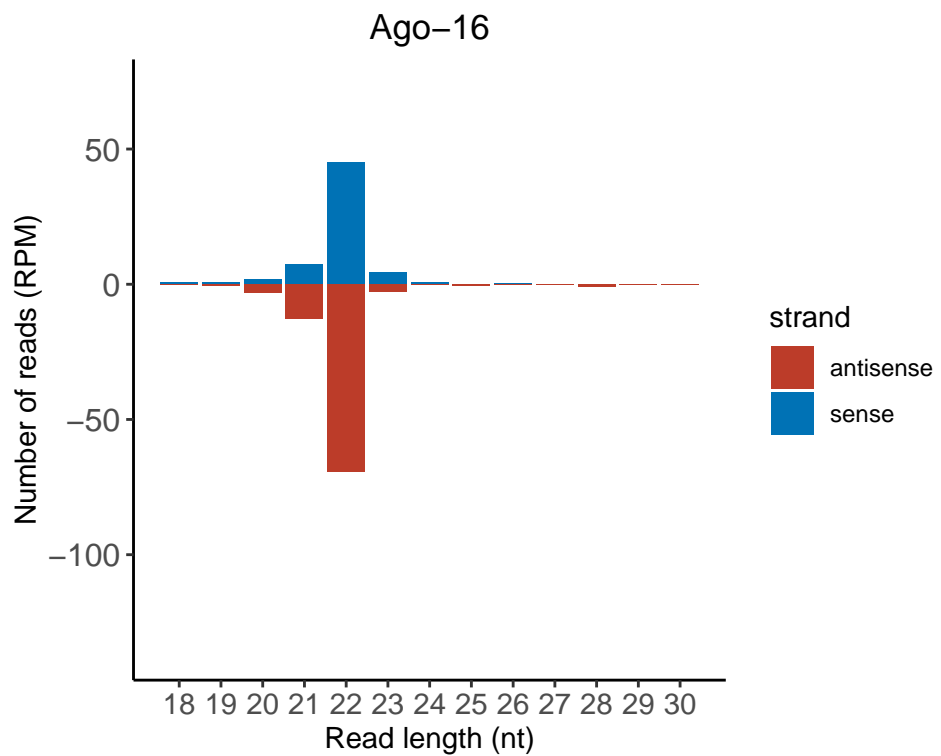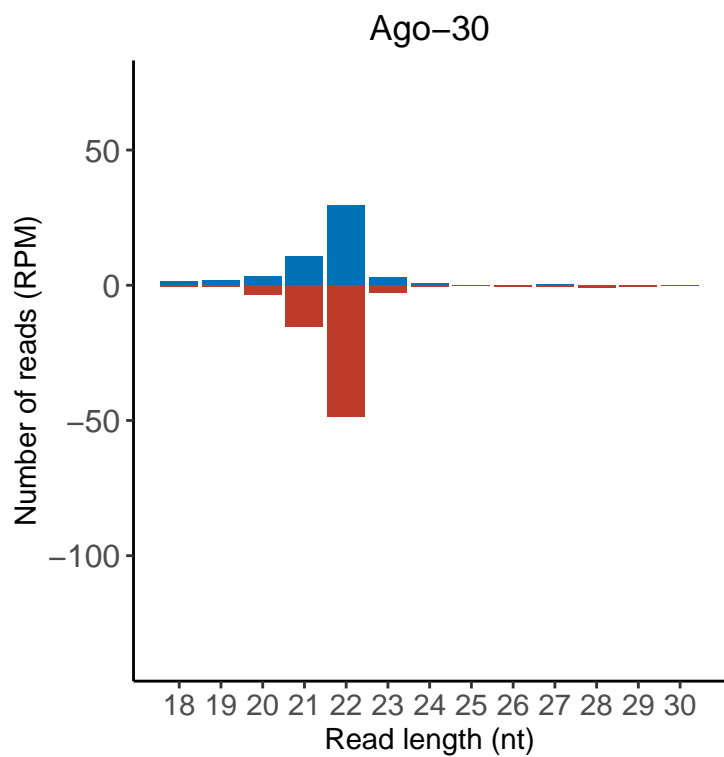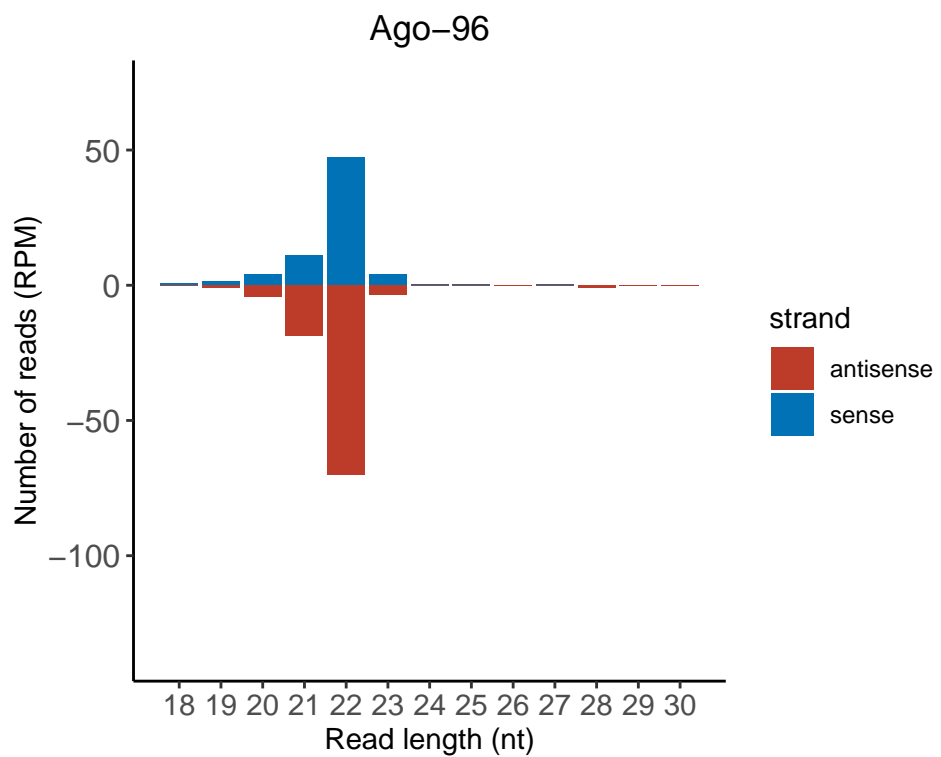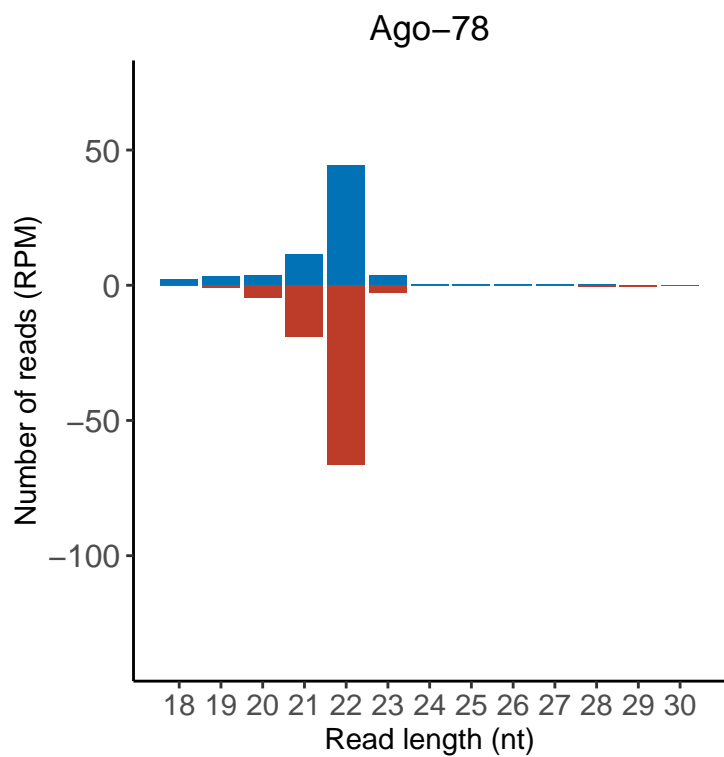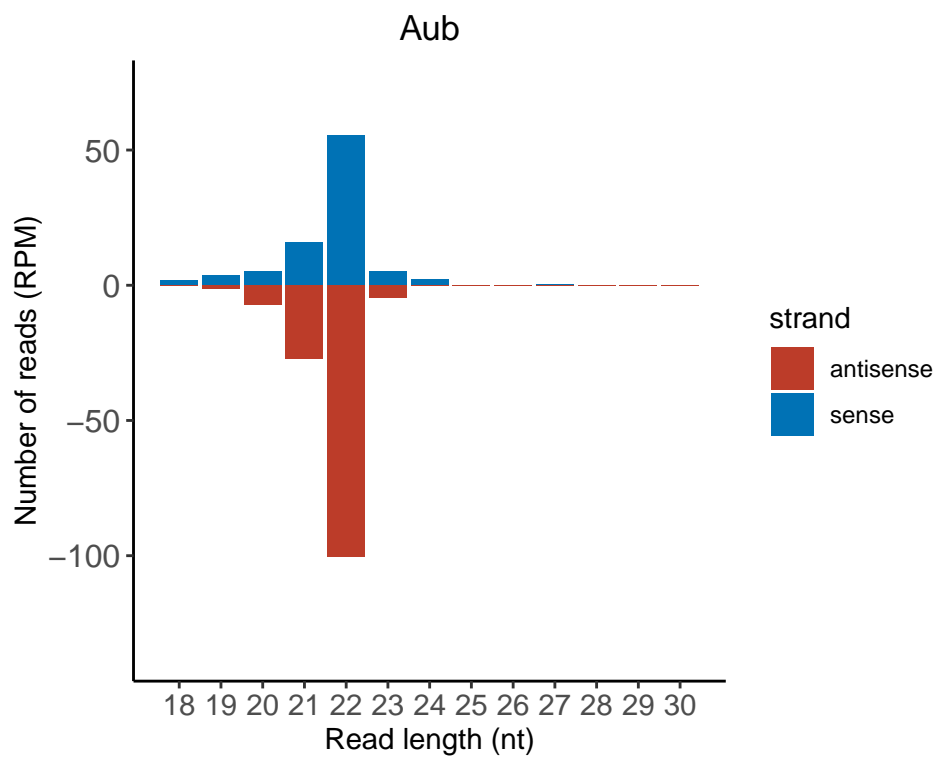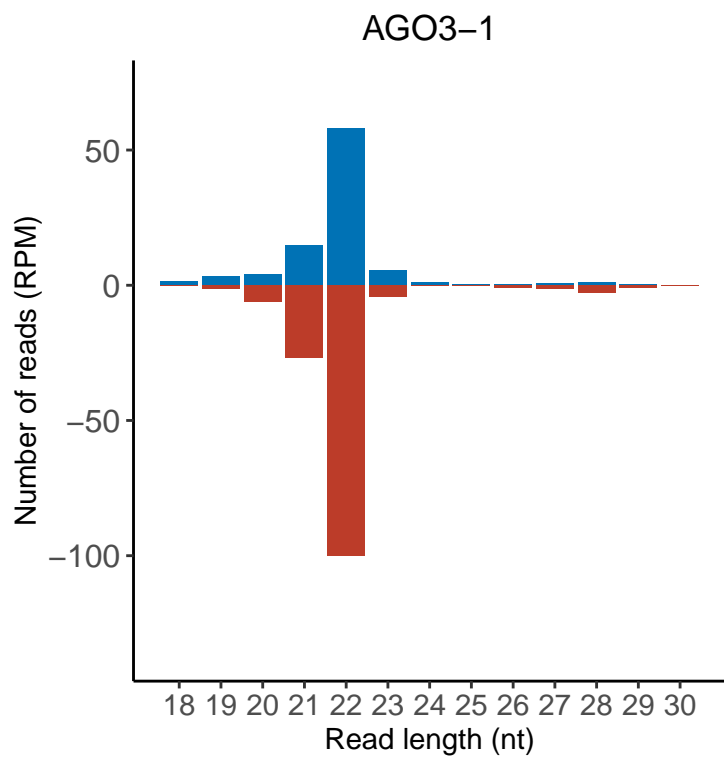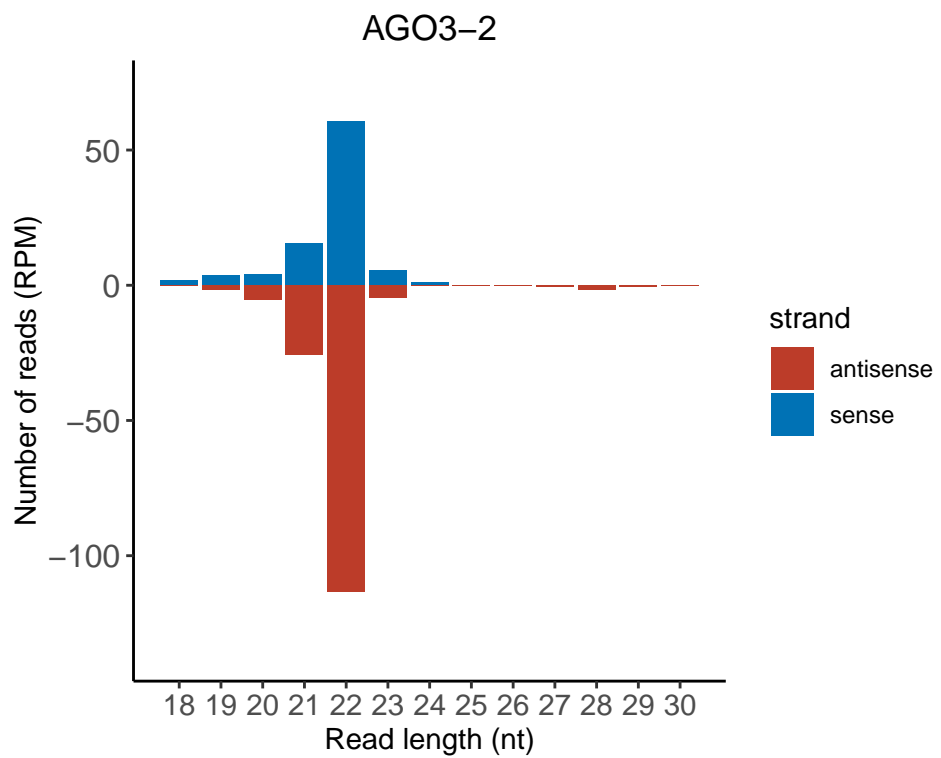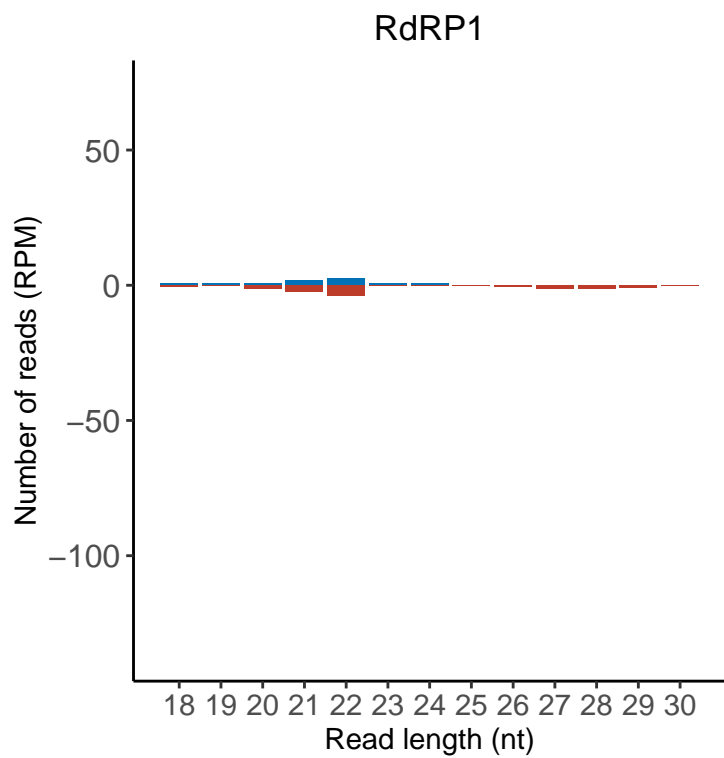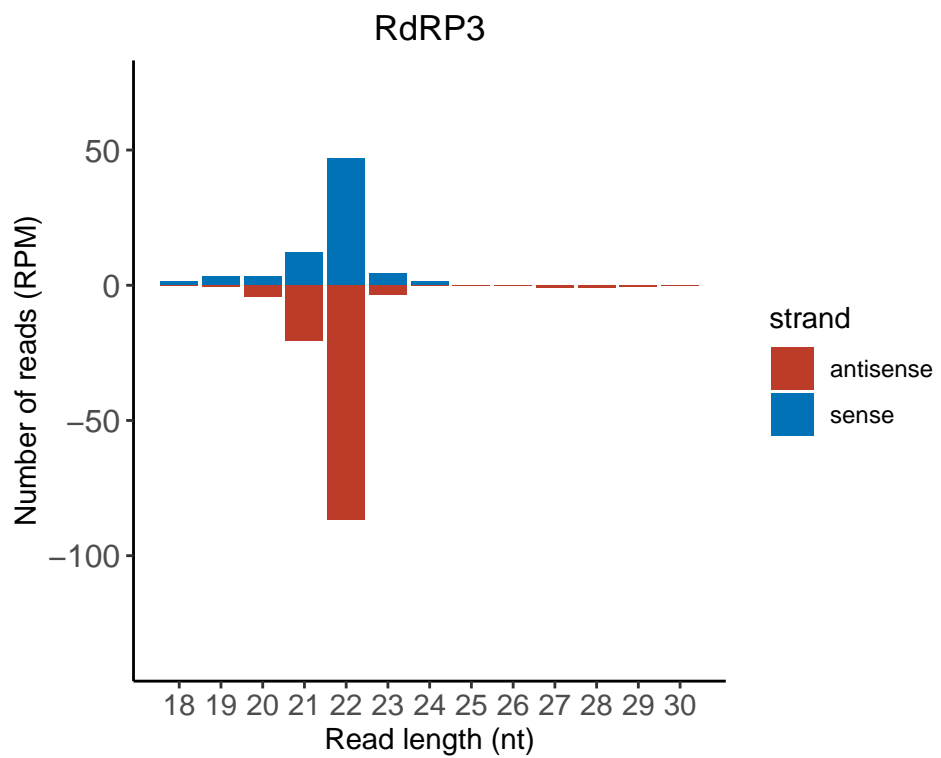

Supplement: S1 Data — On the Normalized and Relative levels pages, normalized read counts (RPM) and relative levels compared to the control (dsGFP) library were used. TEs with more than 50RPM and Coding Genes with more than 3.5RPM on average in KD libraries are included in this website. For viral sequences, reference sequences were generated by assembling sRNA sequences (See the Analysis of sRNAseq data section in Materials and methods.) and size distributions of sRNA reads mapped to each contig are shown. Reads were first grouped by their 5’ nucleotides, and reads of each length were counted. Full tables including TEs and Coding genes with fewer reads are in the “FullTable” folder. (ZIP) [file pone.0281195.s017.zip › SupplementaryData/Links/PDFs/Motif:rnd-5_family-1420TE_lengthdistribution.pdf]

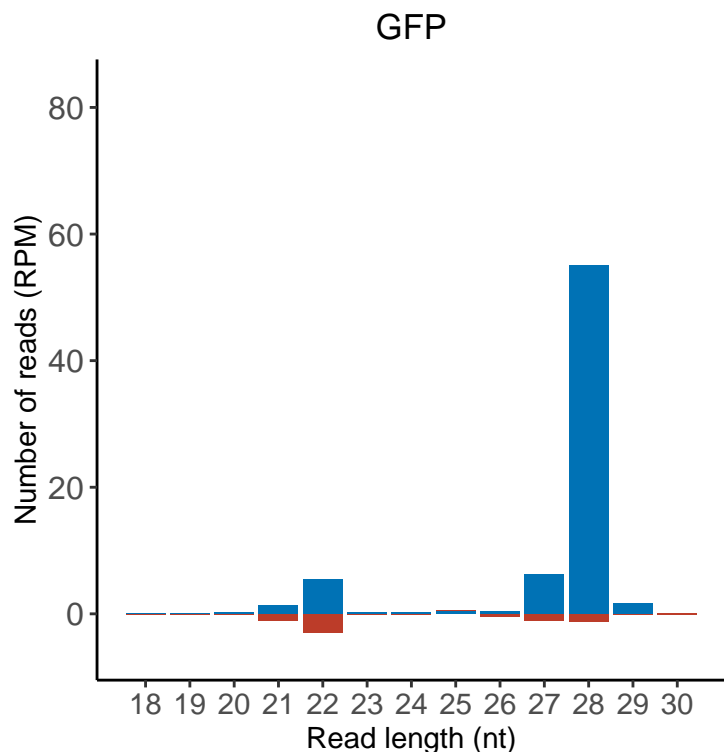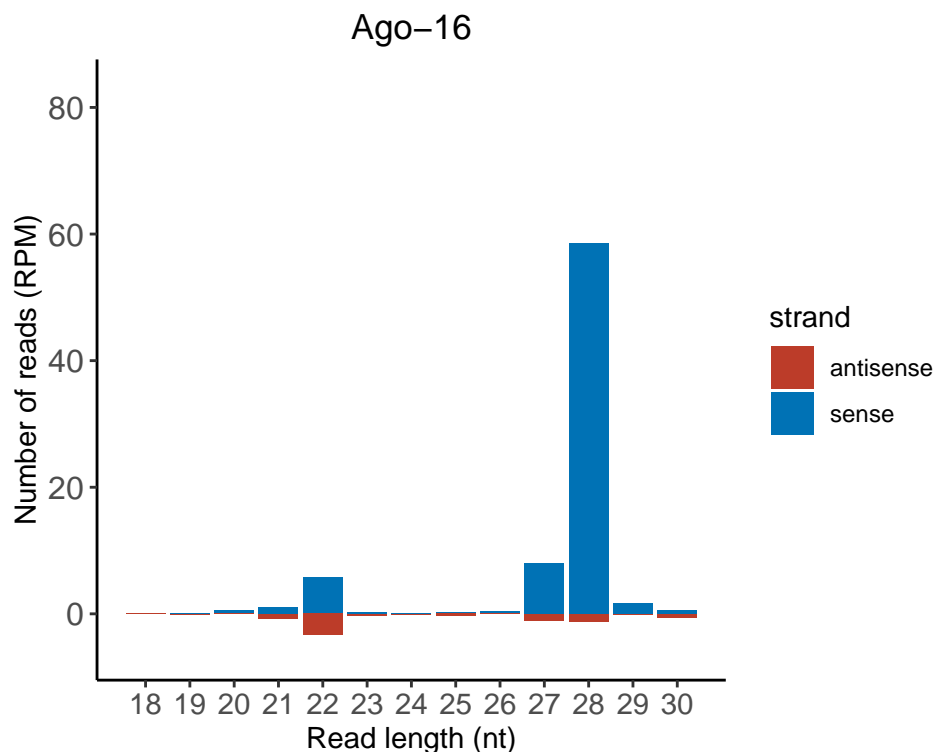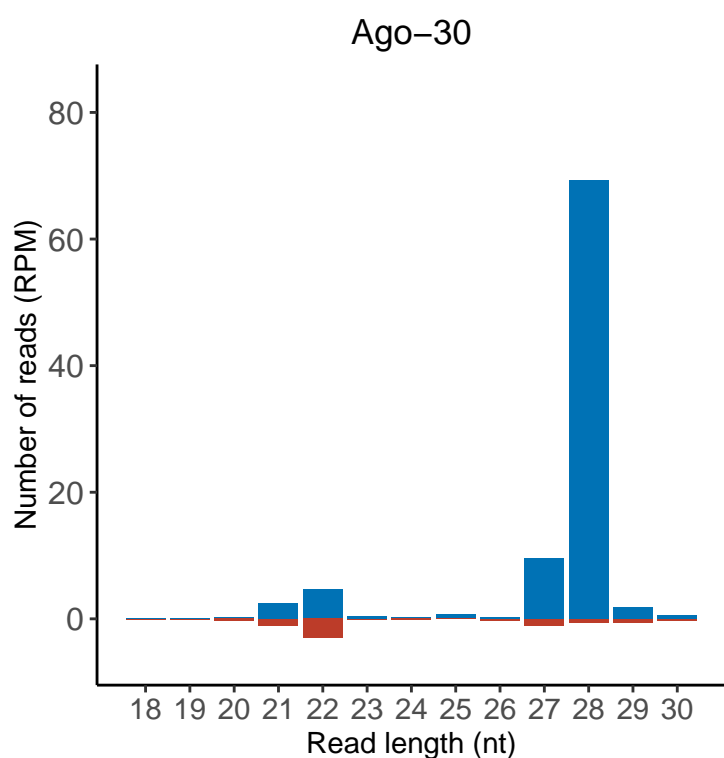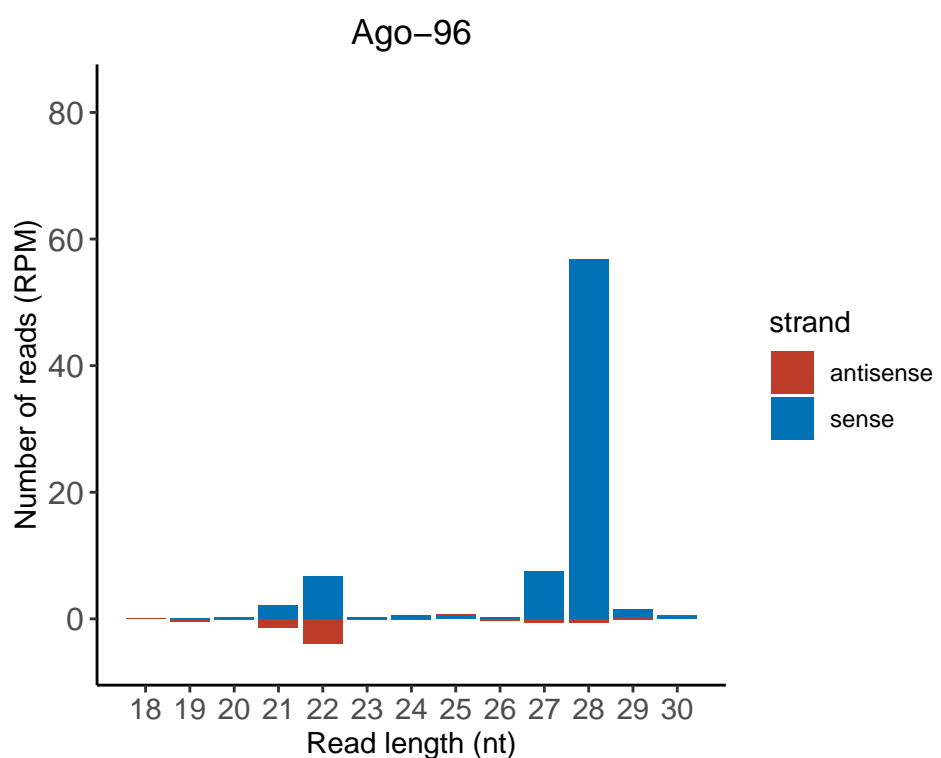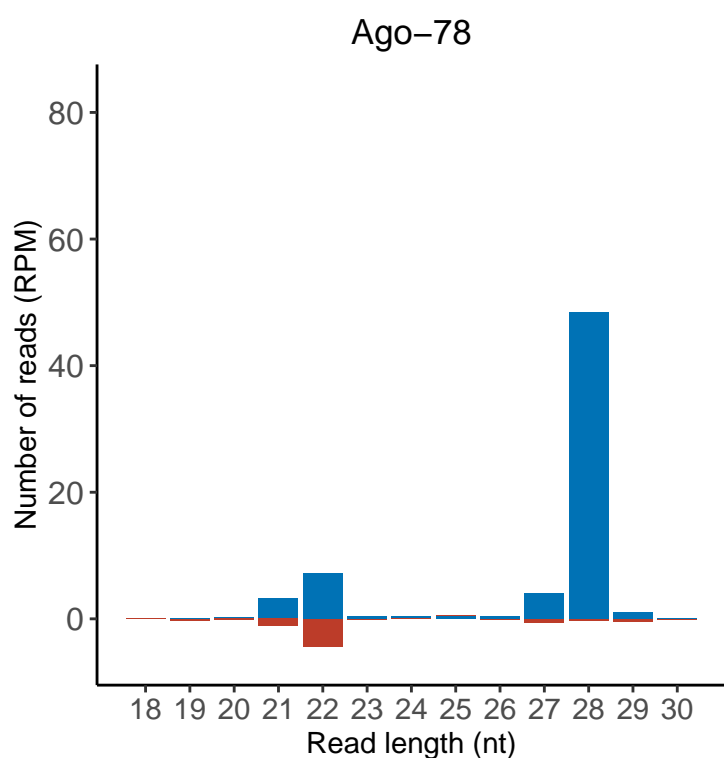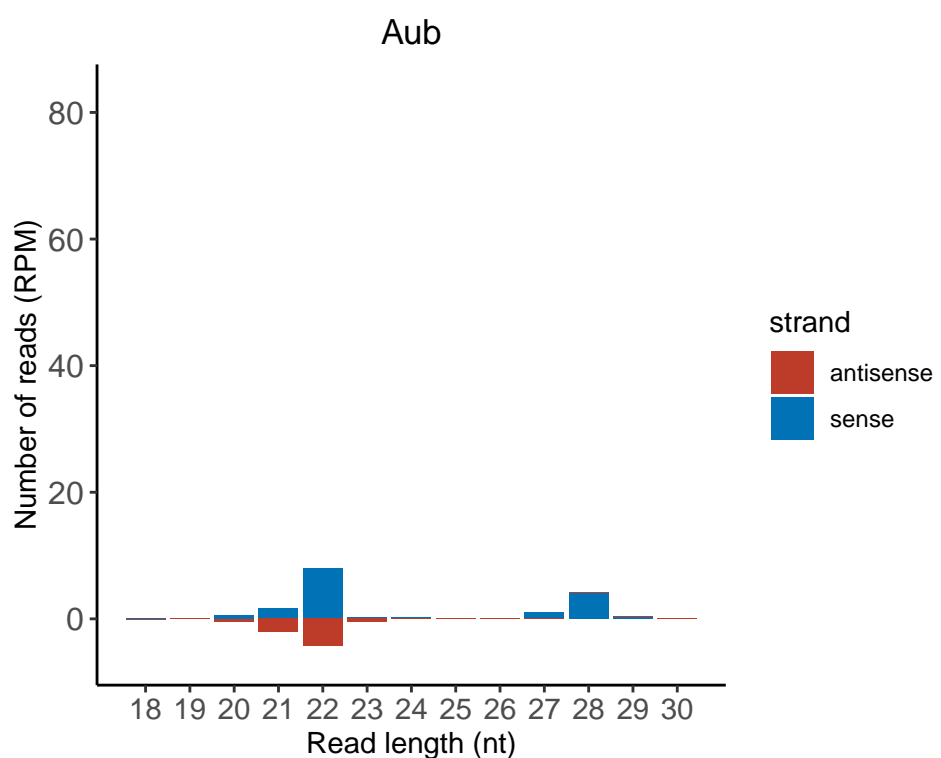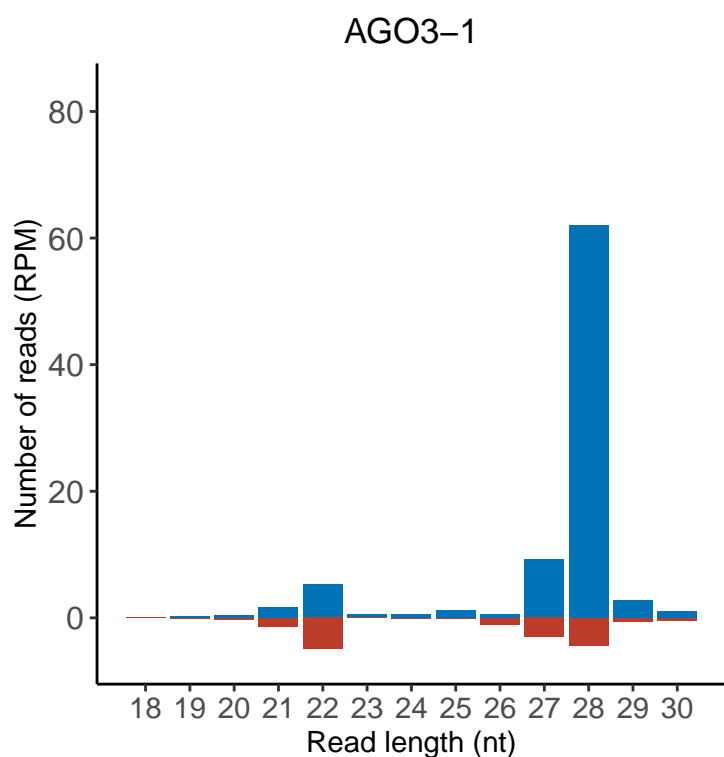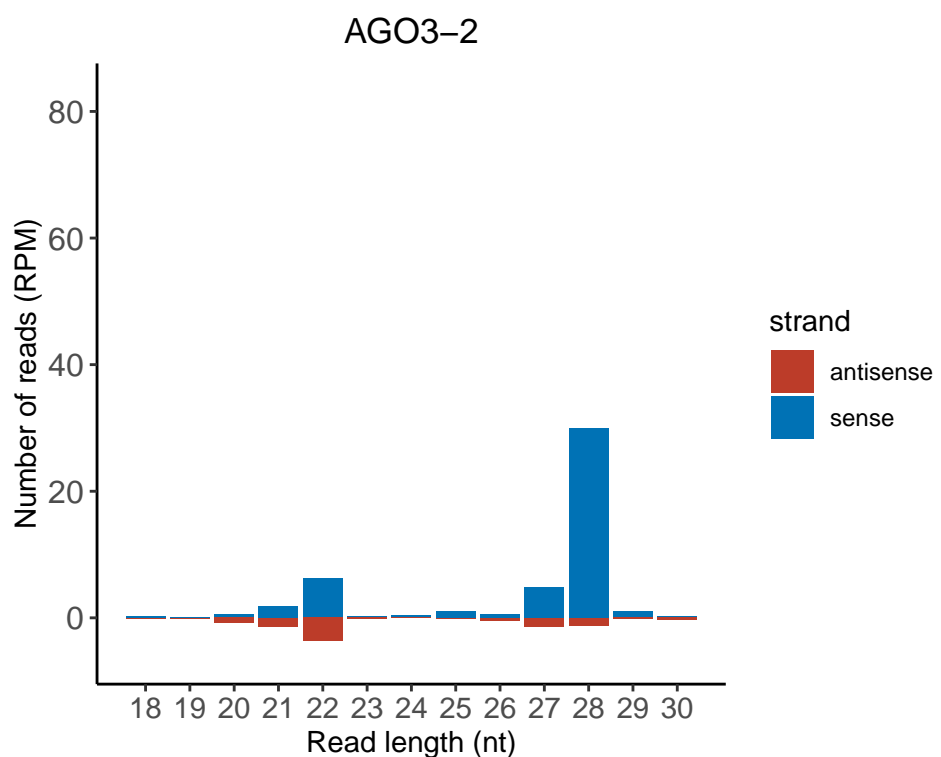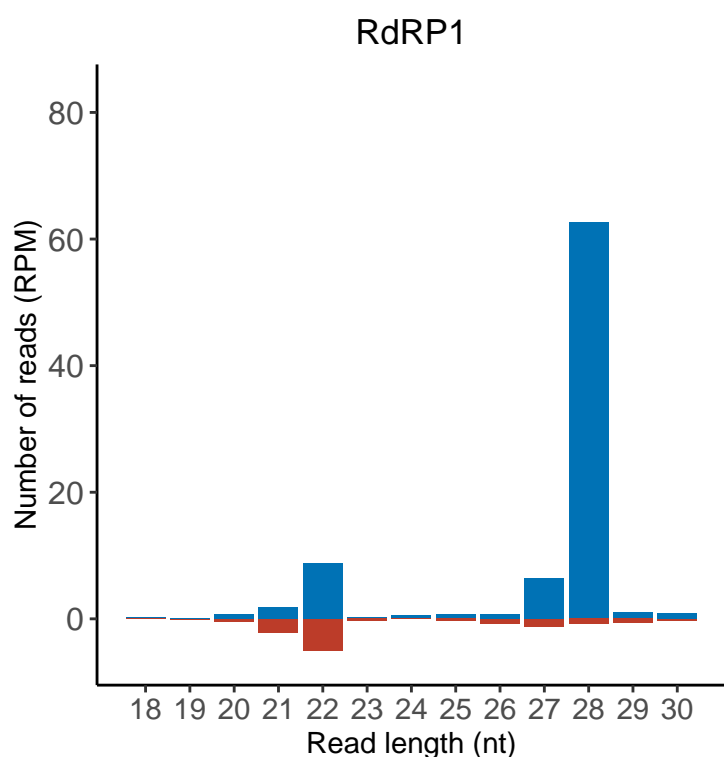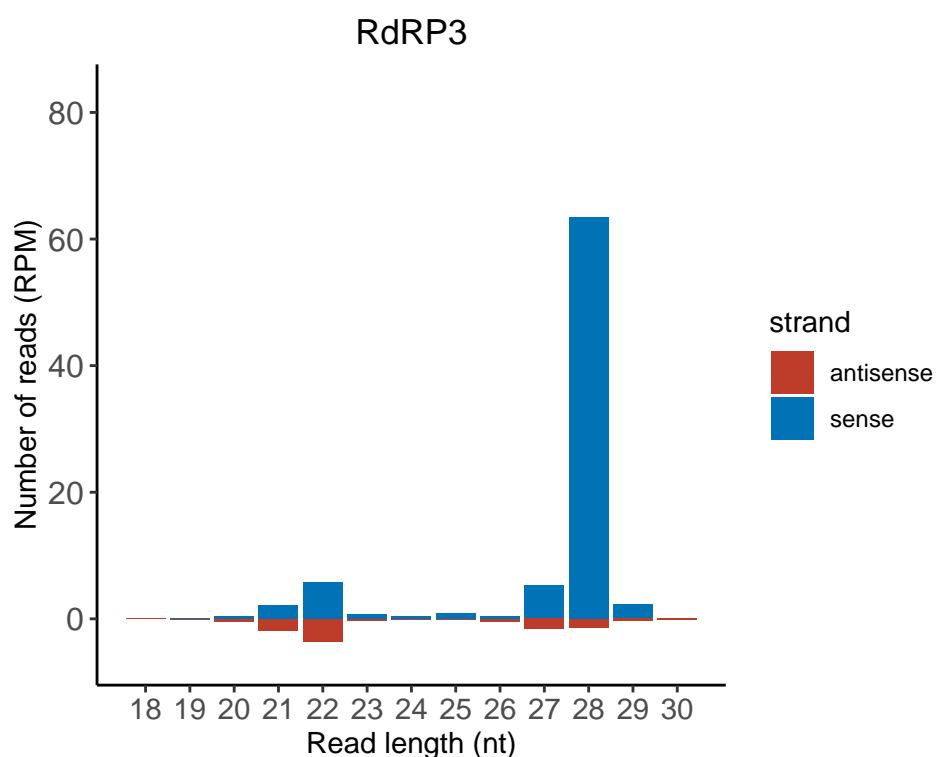

Supplement: S1 Data — On the Normalized and Relative levels pages, normalized read counts (RPM) and relative levels compared to the control (dsGFP) library were used. TEs with more than 50RPM and Coding Genes with more than 3.5RPM on average in KD libraries are included in this website. For viral sequences, reference sequences were generated by assembling sRNA sequences (See the Analysis of sRNAseq data section in Materials and methods.) and size distributions of sRNA reads mapped to each contig are shown. Reads were first grouped by their 5’ nucleotides, and reads of each length were counted. Full tables including TEs and Coding genes with fewer reads are in the “FullTable” folder. (ZIP) [file pone.0281195.s017.zip › SupplementaryData/Links/PDFs/Motif:rnd-1_family-51TE_lengthdistribution.pdf]

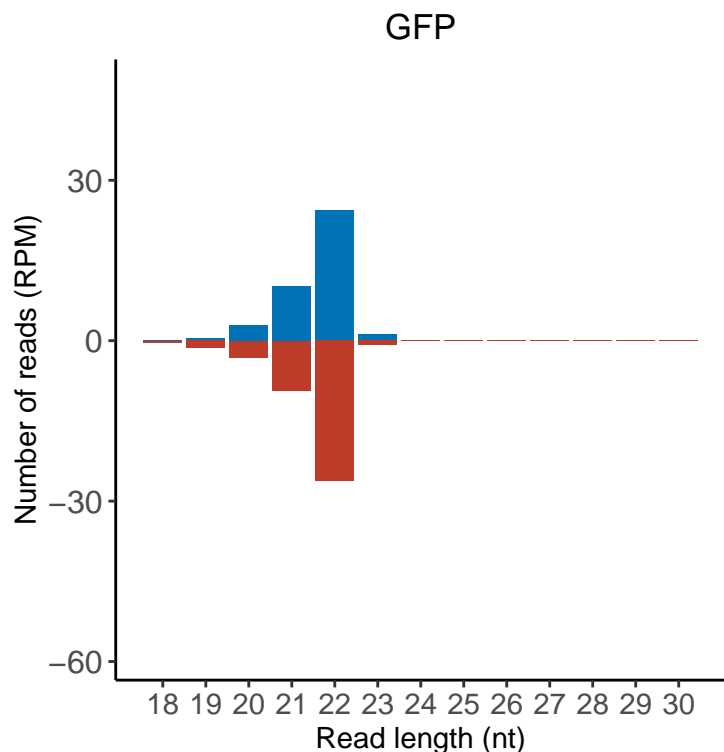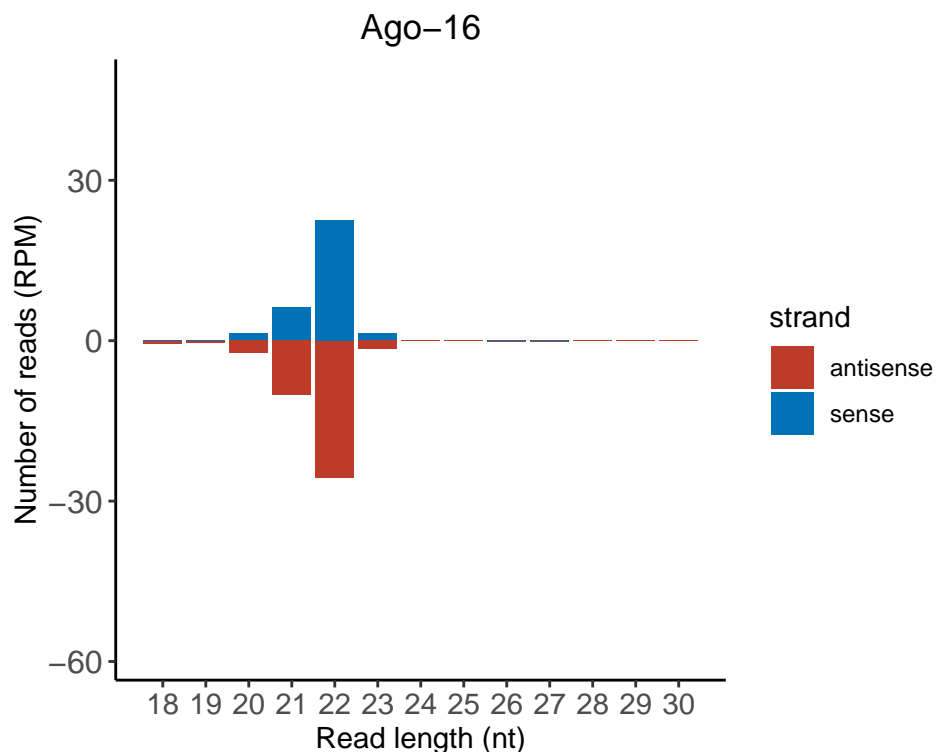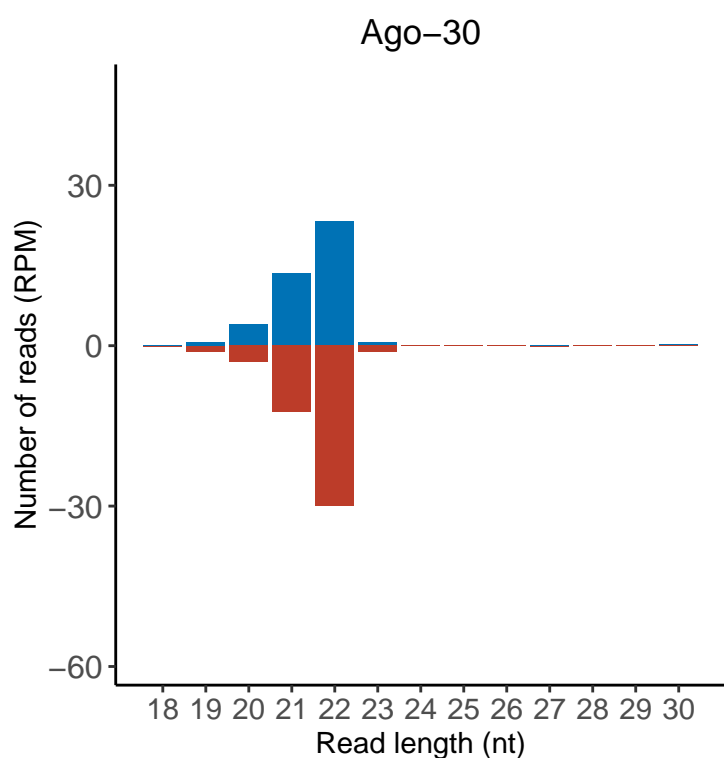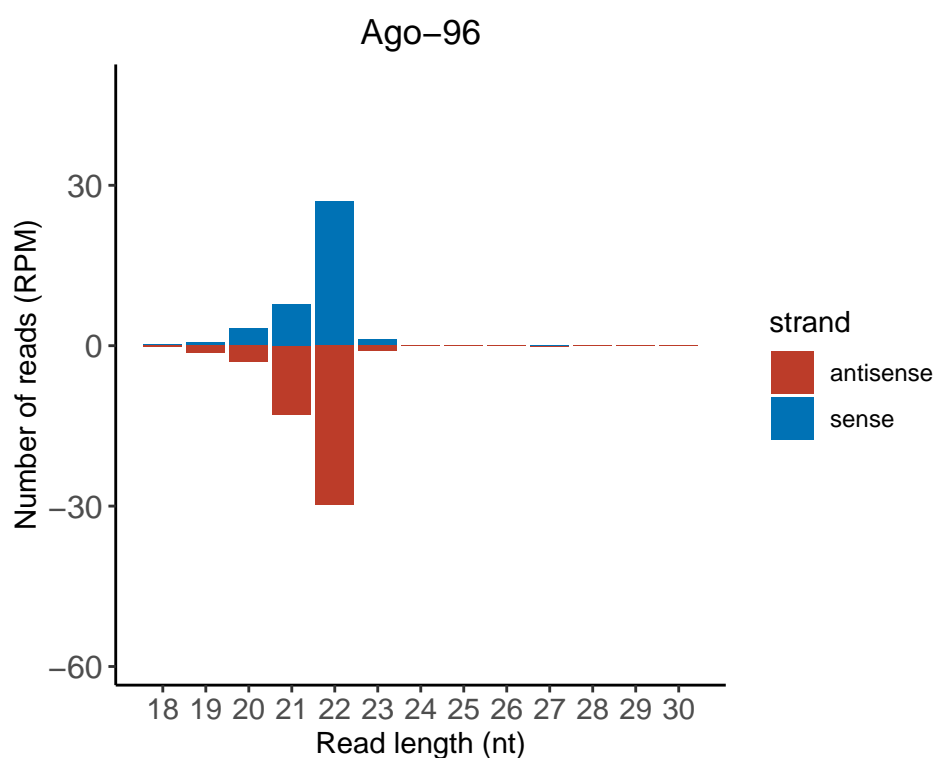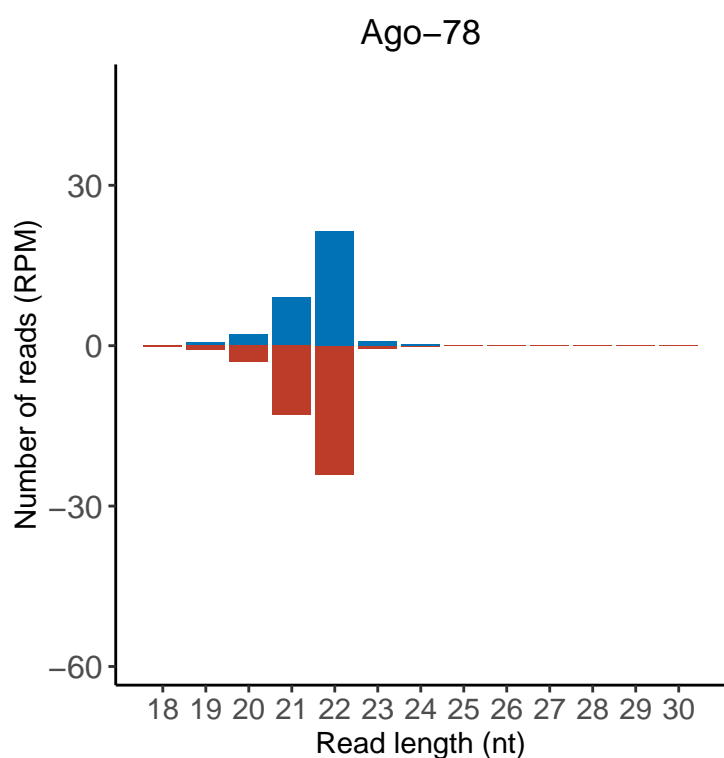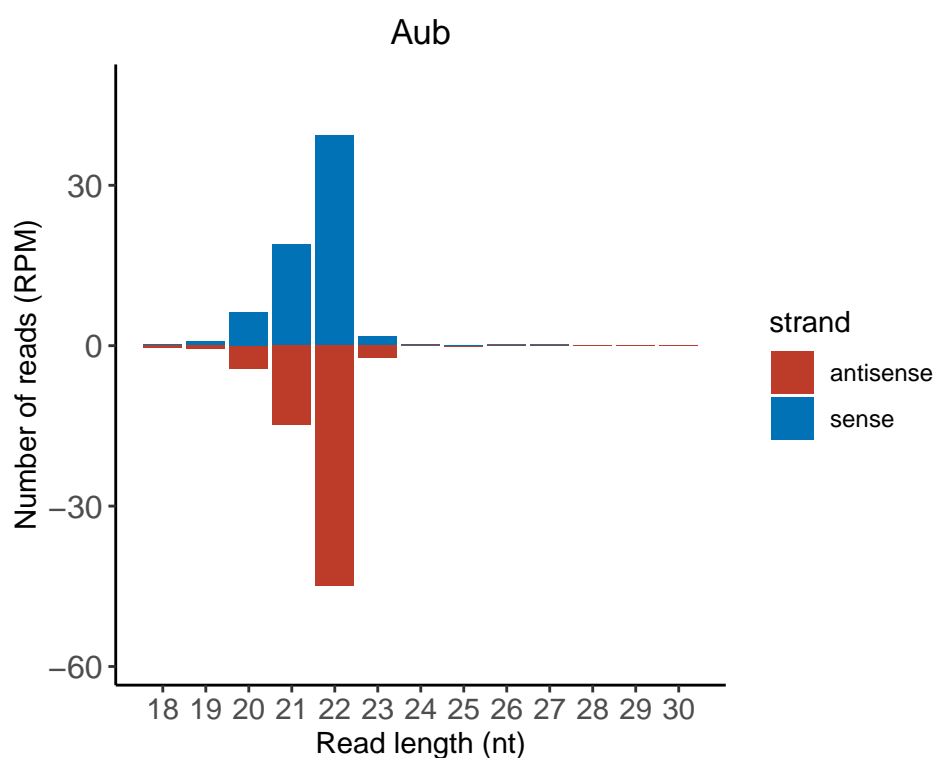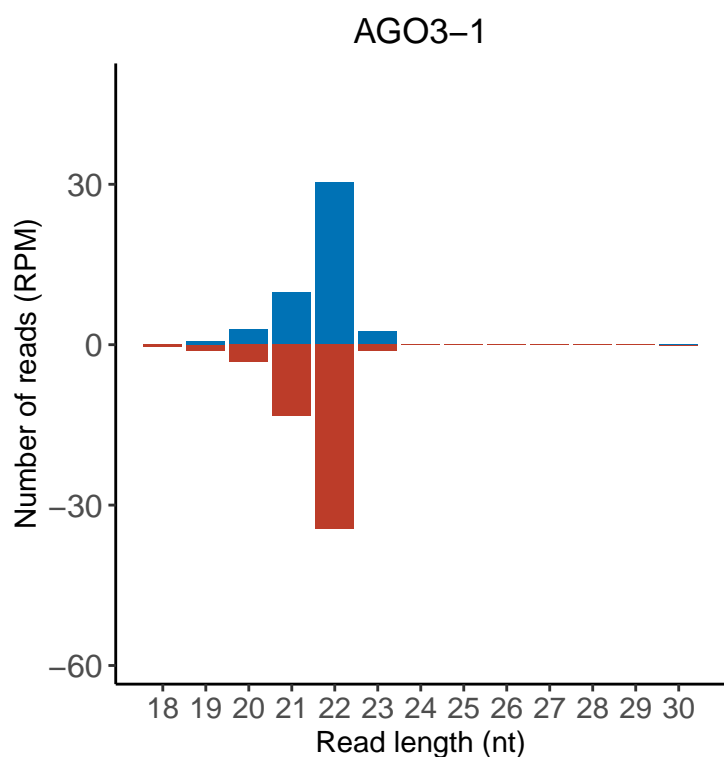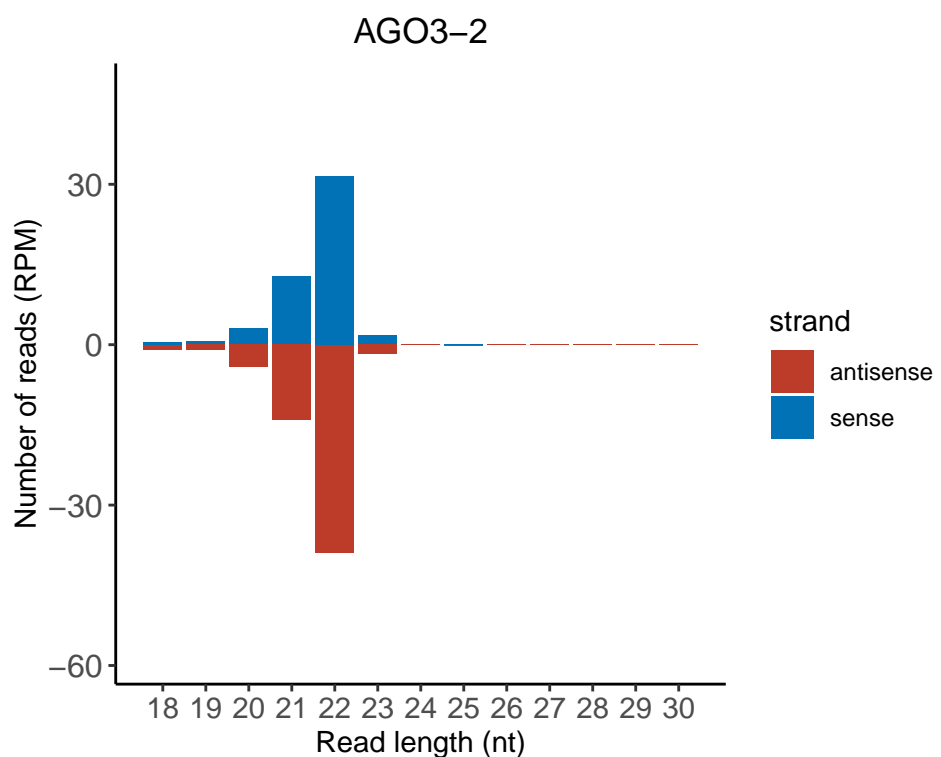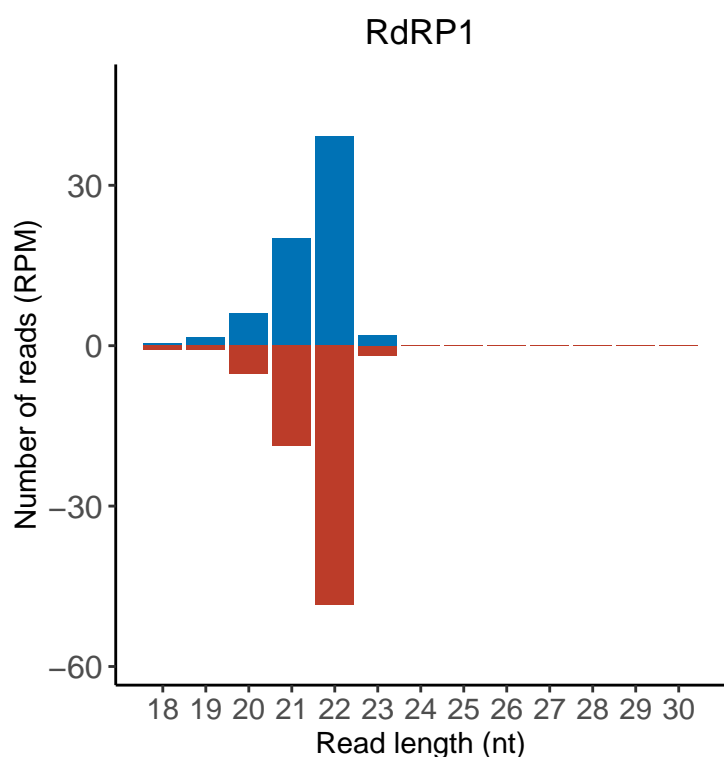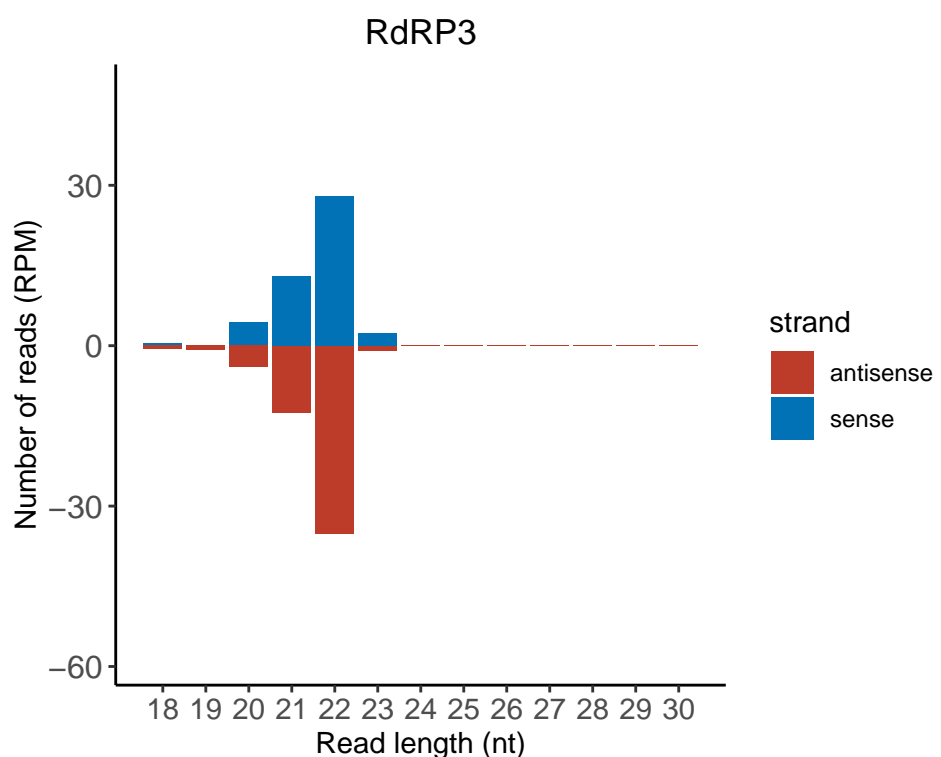

Supplement: S1 Data — On the Normalized and Relative levels pages, normalized read counts (RPM) and relative levels compared to the control (dsGFP) library were used. TEs with more than 50RPM and Coding Genes with more than 3.5RPM on average in KD libraries are included in this website. For viral sequences, reference sequences were generated by assembling sRNA sequences (See the Analysis of sRNAseq data section in Materials and methods.) and size distributions of sRNA reads mapped to each contig are shown. Reads were first grouped by their 5’ nucleotides, and reads of each length were counted. Full tables including TEs and Coding genes with fewer reads are in the “FullTable” folder. (ZIP) [file pone.0281195.s017.zip › SupplementaryData/Links/PDFs/Motif:rnd-6_family-6470TE_lengthdistribution.pdf]
