# Supplementary material for: A novel eukaryotic RdRP-dependent small RNA pathway represses antiviral immunity by controlling an ERK pathway component in the black-legged tick
Source: PLoS One. 2023 Mar 30;18(3):e0281195. doi: 10.1371/journal.pone.0281195 (PMC10062562; doi:10.1371/journal.pone.0281195)
Supplement: S1 Data — On the Normalized and Relative levels pages, normalized read counts (RPM) and relative levels compared to the control (dsGFP) library were used. TEs with more than 50RPM and Coding Genes with more than 3.5RPM on average in KD libraries are included in this website. For viral sequences, reference sequences were generated by assembling sRNA sequences (See the Analysis of sRNAseq data section in Materials and methods.) and size distributions of sRNA reads mapped to each contig are shown. Reads were first grouped by their 5’ nucleotides, and reads of each length were counted. Full tables including TEs and Coding genes with fewer reads are in the “FullTable” folder. (ZIP) [file pone.0281195.s017.zip › SupplementaryData/Links/dsGFP_viralcontig_info_sRNA.html]

|  | contigID | virusID | evalue | pident | virusName | contig\_length | reference | Plot\_18nt30nt | Plot\_25nt30nt |
| --- | --- | --- | --- | --- | --- | --- | --- | --- | --- |
| 0 | CONTIG77 | BBD75430.1 | 0.000000e+00 | 99.242 | hypothetical protein, partial [Ixodes scapularis associated virus 3] | 1540 | LC094965 | ./PDFs/distr\_18nt30nt/B049\_CONTIG77\_sRNA\_size\_profile\_raw\_read\_count.pdf | ./PDFs/distr\_25nt30nt/B049\_CONTIG77\_sRNA\_size\_profile\_raw\_read\_count.pdf |
| 1 | CONTIG67 | BBD75428.1 | 0.000000e+00 | 98.327 | hypothetical protein, partial [Ixodes scapularis associated virus 1] | 2665 | LC094964 | ./PDFs/distr\_18nt30nt/B049\_CONTIG67\_sRNA\_size\_profile\_raw\_read\_count.pdf | ./PDFs/distr\_25nt30nt/B049\_CONTIG67\_sRNA\_size\_profile\_raw\_read\_count.pdf |
| 2 | CONTIG111 | BBD75427.1 | 8.490000e-06 | 100.000 | polyprotein [Ixodes scapularis iflavirus] | 61 | LC094426 | ./PDFs/distr\_18nt30nt/B049\_CONTIG111\_sRNA\_size\_profile\_raw\_read\_count.pdf | ./PDFs/distr\_25nt30nt/B049\_CONTIG111\_sRNA\_size\_profile\_raw\_read\_count.pdf |
| 3 | CONTIG53 | BBD75427.1 | 1.700000e-77 | 99.333 | polyprotein [Ixodes scapularis iflavirus] | 472 | LC094426 | ./PDFs/distr\_18nt30nt/B049\_CONTIG53\_sRNA\_size\_profile\_raw\_read\_count.pdf | ./PDFs/distr\_25nt30nt/B049\_CONTIG53\_sRNA\_size\_profile\_raw\_read\_count.pdf |
| 4 | CONTIG106 | BBD75427.1 | 1.530000e-09 | 100.000 | polyprotein [Ixodes scapularis iflavirus] | 76 | LC094426 | ./PDFs/distr\_18nt30nt/B049\_CONTIG106\_sRNA\_size\_profile\_raw\_read\_count.pdf | ./PDFs/distr\_25nt30nt/B049\_CONTIG106\_sRNA\_size\_profile\_raw\_read\_count.pdf |
| 5 | CONTIG142 | BBD75427.1 | 0.000000e+00 | 99.762 | polyprotein [Ixodes scapularis iflavirus] | 1261 | LC094426 | ./PDFs/distr\_18nt30nt/B049\_CONTIG142\_sRNA\_size\_profile\_raw\_read\_count.pdf | ./PDFs/distr\_25nt30nt/B049\_CONTIG142\_sRNA\_size\_profile\_raw\_read\_count.pdf |
| 6 | CONTIG119 | BBD75427.1 | 2.360000e-136 | 100.000 | polyprotein [Ixodes scapularis iflavirus] | 613 | LC094426 | ./PDFs/distr\_18nt30nt/B049\_CONTIG119\_sRNA\_size\_profile\_raw\_read\_count.pdf | ./PDFs/distr\_25nt30nt/B049\_CONTIG119\_sRNA\_size\_profile\_raw\_read\_count.pdf |
| 7 | CONTIG44 | BBD75427.1 | 8.590000e-60 | 100.000 | polyprotein [Ixodes scapularis iflavirus] | 300 | LC094426 | ./PDFs/distr\_18nt30nt/B049\_CONTIG44\_sRNA\_size\_profile\_raw\_read\_count.pdf | ./PDFs/distr\_25nt30nt/B049\_CONTIG44\_sRNA\_size\_profile\_raw\_read\_count.pdf |
| 8 | CONTIG43 | BBD75427.1 | 1.320000e-23 | 100.000 | polyprotein [Ixodes scapularis iflavirus] | 142 | LC094426 | ./PDFs/distr\_18nt30nt/B049\_CONTIG43\_sRNA\_size\_profile\_raw\_read\_count.pdf | ./PDFs/distr\_25nt30nt/B049\_CONTIG43\_sRNA\_size\_profile\_raw\_read\_count.pdf |
| 9 | CONTIG98 | BBD75427.1 | 7.200000e-57 | 98.913 | polyprotein [Ixodes scapularis iflavirus] | 278 | LC094426 | ./PDFs/distr\_18nt30nt/B049\_CONTIG98\_sRNA\_size\_profile\_raw\_read\_count.pdf | ./PDFs/distr\_25nt30nt/B049\_CONTIG98\_sRNA\_size\_profile\_raw\_read\_count.pdf |
| 10 | CONTIG82 | BBD75427.1 | 3.070000e-54 | 98.795 | polyprotein [Ixodes scapularis iflavirus] | 251 | LC094426 | ./PDFs/distr\_18nt30nt/B049\_CONTIG82\_sRNA\_size\_profile\_raw\_read\_count.pdf | ./PDFs/distr\_25nt30nt/B049\_CONTIG82\_sRNA\_size\_profile\_raw\_read\_count.pdf |
| 11 | CONTIG33 | BBD75427.1 | 4.180000e-48 | 97.674 | polyprotein [Ixodes scapularis iflavirus] | 261 | LC094426 | ./PDFs/distr\_18nt30nt/B049\_CONTIG33\_sRNA\_size\_profile\_raw\_read\_count.pdf | ./PDFs/distr\_25nt30nt/B049\_CONTIG33\_sRNA\_size\_profile\_raw\_read\_count.pdf |
| 12 | CONTIG125 | BBD75427.1 | 4.730000e-47 | 100.000 | polyprotein [Ixodes scapularis iflavirus] | 237 | LC094426 | ./PDFs/distr\_18nt30nt/B049\_CONTIG125\_sRNA\_size\_profile\_raw\_read\_count.pdf | ./PDFs/distr\_25nt30nt/B049\_CONTIG125\_sRNA\_size\_profile\_raw\_read\_count.pdf |
| 13 | CONTIG28 | BBD75427.1 | 0.000000e+00 | 97.794 | polyprotein [Ixodes scapularis iflavirus] | 820 | LC094426 | ./PDFs/distr\_18nt30nt/B049\_CONTIG28\_sRNA\_size\_profile\_raw\_read\_count.pdf | ./PDFs/distr\_25nt30nt/B049\_CONTIG28\_sRNA\_size\_profile\_raw\_read\_count.pdf |
| 14 | CONTIG128 | BBD75427.1 | 0.000000e+00 | 99.804 | polyprotein [Ixodes scapularis iflavirus] | 1532 | LC094426 | ./PDFs/distr\_18nt30nt/B049\_CONTIG128\_sRNA\_size\_profile\_raw\_read\_count.pdf | ./PDFs/distr\_25nt30nt/B049\_CONTIG128\_sRNA\_size\_profile\_raw\_read\_count.pdf |
| 15 | CONTIG24 | BBD75427.1 | 1.610000e-05 | 100.000 | polyprotein [Ixodes scapularis iflavirus] | 64 | LC094426 | ./PDFs/distr\_18nt30nt/B049\_CONTIG24\_sRNA\_size\_profile\_raw\_read\_count.pdf | ./PDFs/distr\_25nt30nt/B049\_CONTIG24\_sRNA\_size\_profile\_raw\_read\_count.pdf |
| 16 | CONTIG62 | BBD75427.1 | 1.370000e-97 | 100.000 | polyprotein [Ixodes scapularis iflavirus] | 442 | LC094426 | ./PDFs/distr\_18nt30nt/B049\_CONTIG62\_sRNA\_size\_profile\_raw\_read\_count.pdf | ./PDFs/distr\_25nt30nt/B049\_CONTIG62\_sRNA\_size\_profile\_raw\_read\_count.pdf |
| 17 | CONTIG80 | BBD75426.1 | 4.220000e-144 | 98.578 | glycoprotein precursor [Ixodes scapularis bunyavirus] | 635 | LC094425 | ./PDFs/distr\_18nt30nt/B049\_CONTIG80\_sRNA\_size\_profile\_raw\_read\_count.pdf | ./PDFs/distr\_25nt30nt/B049\_CONTIG80\_sRNA\_size\_profile\_raw\_read\_count.pdf |
| 18 | CONTIG134 | BBD75426.1 | 4.350000e-58 | 97.000 | glycoprotein precursor [Ixodes scapularis bunyavirus] | 303 | LC094425 | ./PDFs/distr\_18nt30nt/B049\_CONTIG134\_sRNA\_size\_profile\_raw\_read\_count.pdf | ./PDFs/distr\_25nt30nt/B049\_CONTIG134\_sRNA\_size\_profile\_raw\_read\_count.pdf |
| 19 | CONTIG157 | BBD75426.1 | 1.940000e-138 | 99.510 | glycoprotein precursor [Ixodes scapularis bunyavirus] | 615 | LC094425 | ./PDFs/distr\_18nt30nt/B049\_CONTIG157\_sRNA\_size\_profile\_raw\_read\_count.pdf | ./PDFs/distr\_25nt30nt/B049\_CONTIG157\_sRNA\_size\_profile\_raw\_read\_count.pdf |
| 20 | CONTIG160 | BBD75426.1 | 0.000000e+00 | 100.000 | glycoprotein precursor [Ixodes scapularis bunyavirus] | 1399 | LC094425 | ./PDFs/distr\_18nt30nt/B049\_CONTIG160\_sRNA\_size\_profile\_raw\_read\_count.pdf | ./PDFs/distr\_25nt30nt/B049\_CONTIG160\_sRNA\_size\_profile\_raw\_read\_count.pdf |
| 21 | CONTIG90 | BBD75426.1 | 1.360000e-148 | 99.543 | glycoprotein precursor [Ixodes scapularis bunyavirus] | 657 | LC094425 | ./PDFs/distr\_18nt30nt/B049\_CONTIG90\_sRNA\_size\_profile\_raw\_read\_count.pdf | ./PDFs/distr\_25nt30nt/B049\_CONTIG90\_sRNA\_size\_profile\_raw\_read\_count.pdf |
| 22 | CONTIG40 | BBD75426.1 | 4.950000e-115 | 96.721 | glycoprotein precursor [Ixodes scapularis bunyavirus] | 549 | LC094425 | ./PDFs/distr\_18nt30nt/B049\_CONTIG40\_sRNA\_size\_profile\_raw\_read\_count.pdf | ./PDFs/distr\_25nt30nt/B049\_CONTIG40\_sRNA\_size\_profile\_raw\_read\_count.pdf |
| 23 | CONTIG123 | BBD75425.1 | 0.000000e+00 | 99.659 | RNA-dependent RNA polymerase [Ixodes scapularis bunyavirus] | 1827 | LC094424 | ./PDFs/distr\_18nt30nt/B049\_CONTIG123\_sRNA\_size\_profile\_raw\_read\_count.pdf | ./PDFs/distr\_25nt30nt/B049\_CONTIG123\_sRNA\_size\_profile\_raw\_read\_count.pdf |
| 24 | CONTIG124 | BBD75425.1 | 0.000000e+00 | 99.745 | RNA-dependent RNA polymerase [Ixodes scapularis bunyavirus] | 4818 | LC094424 | ./PDFs/distr\_18nt30nt/B049\_CONTIG124\_sRNA\_size\_profile\_raw\_read\_count.pdf | ./PDFs/distr\_25nt30nt/B049\_CONTIG124\_sRNA\_size\_profile\_raw\_read\_count.pdf |
| 25 | CONTIG96 | BBD75425.1 | 2.410000e-18 | 100.000 | RNA-dependent RNA polymerase [Ixodes scapularis bunyavirus] | 130 | LC094424 | ./PDFs/distr\_18nt30nt/B049\_CONTIG96\_sRNA\_size\_profile\_raw\_read\_count.pdf | ./PDFs/distr\_25nt30nt/B049\_CONTIG96\_sRNA\_size\_profile\_raw\_read\_count.pdf |
| 26 | CONTIG130 | BBD75425.1 | 7.430000e-21 | 97.727 | RNA-dependent RNA polymerase [Ixodes scapularis bunyavirus] | 146 | LC094424 | ./PDFs/distr\_18nt30nt/B049\_CONTIG130\_sRNA\_size\_profile\_raw\_read\_count.pdf | ./PDFs/distr\_25nt30nt/B049\_CONTIG130\_sRNA\_size\_profile\_raw\_read\_count.pdf |
| 27 | CONTIG148 | BBD75425.1 | 0.000000e+00 | 99.483 | RNA-dependent RNA polymerase [Ixodes scapularis bunyavirus] | 1740 | LC094424 | ./PDFs/distr\_18nt30nt/B049\_CONTIG148\_sRNA\_size\_profile\_raw\_read\_count.pdf | ./PDFs/distr\_25nt30nt/B049\_CONTIG148\_sRNA\_size\_profile\_raw\_read\_count.pdf |
| 28 | CONTIG154 | BBD75425.1 | 3.220000e-160 | 99.200 | RNA-dependent RNA polymerase [Ixodes scapularis bunyavirus] | 752 | LC094424 | ./PDFs/distr\_18nt30nt/B049\_CONTIG154\_sRNA\_size\_profile\_raw\_read\_count.pdf | ./PDFs/distr\_25nt30nt/B049\_CONTIG154\_sRNA\_size\_profile\_raw\_read\_count.pdf |
| 29 | CONTIG144 | QPI13030.1 | 1.160000e-08 | 100.000 | polyprotein [Iflavirus IricIV-2] | 76 |  | ./PDFs/distr\_18nt30nt/B049\_CONTIG144\_sRNA\_size\_profile\_raw\_read\_count.pdf | ./PDFs/distr\_25nt30nt/B049\_CONTIG144\_sRNA\_size\_profile\_raw\_read\_count.pdf |
| 30 | CONTIG145 | ABG45892.1 | 2.070000e-05 | 100.000 | large T antigen, partial [Betapolyomavirus macacae] | 85 |  | ./PDFs/distr\_18nt30nt/B049\_CONTIG145\_sRNA\_size\_profile\_raw\_read\_count.pdf | ./PDFs/distr\_25nt30nt/B049\_CONTIG145\_sRNA\_size\_profile\_raw\_read\_count.pdf |
| 31 | CONTIG131 | DAD54753.1 | 6.380000e-27 | 100.000 | TPA\_asm: polyprotein [Iflavirus IricIV-3] | 144 |  | ./PDFs/distr\_18nt30nt/B049\_CONTIG131\_sRNA\_size\_profile\_raw\_read\_count.pdf | ./PDFs/distr\_25nt30nt/B049\_CONTIG131\_sRNA\_size\_profile\_raw\_read\_count.pdf |
| 32 | CONTIG141 | QPI13029.1 | 1.440000e-11 | 100.000 | polyprotein [Iflavirus IricIV-1] | 89 |  | ./PDFs/distr\_18nt30nt/B049\_CONTIG141\_sRNA\_size\_profile\_raw\_read\_count.pdf | ./PDFs/distr\_25nt30nt/B049\_CONTIG141\_sRNA\_size\_profile\_raw\_read\_count.pdf |
| 33 | CONTIG139 | USZ80667.1 | 1.700000e-10 | 75.758 | hypothetical protein, partial [Sichuan tick toti-like virus] | 106 |  | ./PDFs/distr\_18nt30nt/B049\_CONTIG139\_sRNA\_size\_profile\_raw\_read\_count.pdf | ./PDFs/distr\_25nt30nt/B049\_CONTIG139\_sRNA\_size\_profile\_raw\_read\_count.pdf |
| 34 | CONTIG136 | QPI13032.1 | 6.400000e-21 | 95.349 | polyprotein [Iflavirus IricIV-5] | 129 |  | ./PDFs/distr\_18nt30nt/B049\_CONTIG136\_sRNA\_size\_profile\_raw\_read\_count.pdf | ./PDFs/distr\_25nt30nt/B049\_CONTIG136\_sRNA\_size\_profile\_raw\_read\_count.pdf |
| 35 | CONTIG165 | AGF91671.1 | 1.020000e-12 | 90.625 | hypothetical protein CDPG\_00067 [Cellulophaga phage phi47:1] | 117 |  | ./PDFs/distr\_18nt30nt/B049\_CONTIG165\_sRNA\_size\_profile\_raw\_read\_count.pdf | ./PDFs/distr\_25nt30nt/B049\_CONTIG165\_sRNA\_size\_profile\_raw\_read\_count.pdf |
| 36 | CONTIG167 | DAD54753.1 | 1.640000e-63 | 100.000 | TPA\_asm: polyprotein [Iflavirus IricIV-3] | 338 |  | ./PDFs/distr\_18nt30nt/B049\_CONTIG167\_sRNA\_size\_profile\_raw\_read\_count.pdf | ./PDFs/distr\_25nt30nt/B049\_CONTIG167\_sRNA\_size\_profile\_raw\_read\_count.pdf |
| 37 | CONTIG175 | USZ80667.1 | 1.580000e-04 | 55.000 | hypothetical protein, partial [Sichuan tick toti-like virus] | 310 |  | ./PDFs/distr\_18nt30nt/B049\_CONTIG175\_sRNA\_size\_profile\_raw\_read\_count.pdf | ./PDFs/distr\_25nt30nt/B049\_CONTIG175\_sRNA\_size\_profile\_raw\_read\_count.pdf |
| 38 | CONTIG177 | USZ80667.1 | 1.260000e-08 | 72.727 | hypothetical protein, partial [Sichuan tick toti-like virus] | 101 |  | ./PDFs/distr\_18nt30nt/B049\_CONTIG177\_sRNA\_size\_profile\_raw\_read\_count.pdf | ./PDFs/distr\_25nt30nt/B049\_CONTIG177\_sRNA\_size\_profile\_raw\_read\_count.pdf |
| 39 | CONTIG179 | USZ80667.1 | 1.490000e-27 | 58.947 | hypothetical protein, partial [Sichuan tick toti-like virus] | 287 |  | ./PDFs/distr\_18nt30nt/B049\_CONTIG179\_sRNA\_size\_profile\_raw\_read\_count.pdf | ./PDFs/distr\_25nt30nt/B049\_CONTIG179\_sRNA\_size\_profile\_raw\_read\_count.pdf |
| 40 | CONTIG180 | YP\_009336907.1 | 3.520000e-22 | 32.443 | hypothetical protein [Hubei toti-like virus 24] | 938 |  | ./PDFs/distr\_18nt30nt/B049\_CONTIG180\_sRNA\_size\_profile\_raw\_read\_count.pdf | ./PDFs/distr\_25nt30nt/B049\_CONTIG180\_sRNA\_size\_profile\_raw\_read\_count.pdf |
| 41 | CONTIG19 | USZ80667.1 | 1.140000e-27 | 52.083 | hypothetical protein, partial [Sichuan tick toti-like virus] | 291 |  | ./PDFs/distr\_18nt30nt/B049\_CONTIG19\_sRNA\_size\_profile\_raw\_read\_count.pdf | ./PDFs/distr\_25nt30nt/B049\_CONTIG19\_sRNA\_size\_profile\_raw\_read\_count.pdf |
| 42 | CONTIG102 | QPI13030.1 | 8.450000e-44 | 93.976 | polyprotein [Iflavirus IricIV-2] | 253 |  | ./PDFs/distr\_18nt30nt/B049\_CONTIG102\_sRNA\_size\_profile\_raw\_read\_count.pdf | ./PDFs/distr\_25nt30nt/B049\_CONTIG102\_sRNA\_size\_profile\_raw\_read\_count.pdf |
| 43 | CONTIG115 | YP\_009336907.1 | 2.580000e-18 | 31.333 | hypothetical protein [Hubei toti-like virus 24] | 622 |  | ./PDFs/distr\_18nt30nt/B049\_CONTIG115\_sRNA\_size\_profile\_raw\_read\_count.pdf | ./PDFs/distr\_25nt30nt/B049\_CONTIG115\_sRNA\_size\_profile\_raw\_read\_count.pdf |
| 44 | CONTIG112 | QPI13030.1 | 3.910000e-64 | 99.038 | polyprotein [Iflavirus IricIV-2] | 315 |  | ./PDFs/distr\_18nt30nt/B049\_CONTIG112\_sRNA\_size\_profile\_raw\_read\_count.pdf | ./PDFs/distr\_25nt30nt/B049\_CONTIG112\_sRNA\_size\_profile\_raw\_read\_count.pdf |
| 45 | CONTIG26 | USZ80667.1 | 3.860000e-08 | 52.000 | hypothetical protein, partial [Sichuan tick toti-like virus] | 152 |  | ./PDFs/distr\_18nt30nt/B049\_CONTIG26\_sRNA\_size\_profile\_raw\_read\_count.pdf | ./PDFs/distr\_25nt30nt/B049\_CONTIG26\_sRNA\_size\_profile\_raw\_read\_count.pdf |
| 46 | CONTIG32 | USZ80667.1 | 2.160000e-46 | 60.156 | hypothetical protein, partial [Sichuan tick toti-like virus] | 386 |  | ./PDFs/distr\_18nt30nt/B049\_CONTIG32\_sRNA\_size\_profile\_raw\_read\_count.pdf | ./PDFs/distr\_25nt30nt/B049\_CONTIG32\_sRNA\_size\_profile\_raw\_read\_count.pdf |
| 47 | CONTIG36 | AAA32208.1 | 3.750000e-13 | 100.000 | ampicillinase, partial [Enterobacteria phage f1] | 90 |  | ./PDFs/distr\_18nt30nt/B049\_CONTIG36\_sRNA\_size\_profile\_raw\_read\_count.pdf | ./PDFs/distr\_25nt30nt/B049\_CONTIG36\_sRNA\_size\_profile\_raw\_read\_count.pdf |
| 48 | CONTIG41 | QGJ03590.1 | 1.450000e-17 | 100.000 | TEM family beta-lactamase, partial [uncultured phage] | 110 |  | ./PDFs/distr\_18nt30nt/B049\_CONTIG41\_sRNA\_size\_profile\_raw\_read\_count.pdf | ./PDFs/distr\_25nt30nt/B049\_CONTIG41\_sRNA\_size\_profile\_raw\_read\_count.pdf |
| 49 | CONTIG45 | QPI13029.1 | 3.520000e-79 | 99.206 | polyprotein [Iflavirus IricIV-1] | 379 |  | ./PDFs/distr\_18nt30nt/B049\_CONTIG45\_sRNA\_size\_profile\_raw\_read\_count.pdf | ./PDFs/distr\_25nt30nt/B049\_CONTIG45\_sRNA\_size\_profile\_raw\_read\_count.pdf |
| 50 | CONTIG49 | USZ80667.1 | 6.140000e-15 | 54.688 | hypothetical protein, partial [Sichuan tick toti-like virus] | 194 |  | ./PDFs/distr\_18nt30nt/B049\_CONTIG49\_sRNA\_size\_profile\_raw\_read\_count.pdf | ./PDFs/distr\_25nt30nt/B049\_CONTIG49\_sRNA\_size\_profile\_raw\_read\_count.pdf |
| 51 | CONTIG52 | USZ80667.1 | 8.420000e-39 | 71.605 | hypothetical protein, partial [Sichuan tick toti-like virus] | 245 |  | ./PDFs/distr\_18nt30nt/B049\_CONTIG52\_sRNA\_size\_profile\_raw\_read\_count.pdf | ./PDFs/distr\_25nt30nt/B049\_CONTIG52\_sRNA\_size\_profile\_raw\_read\_count.pdf |
| 52 | CONTIG54 | BBD75425.1 | 3.140000e-10 | 92.857 | RNA-dependent RNA polymerase [Ixodes scapularis bunyavirus] | 108 |  | ./PDFs/distr\_18nt30nt/B049\_CONTIG54\_sRNA\_size\_profile\_raw\_read\_count.pdf | ./PDFs/distr\_25nt30nt/B049\_CONTIG54\_sRNA\_size\_profile\_raw\_read\_count.pdf |
| 53 | CONTIG64 | AAA32208.1 | 2.960000e-06 | 100.000 | ampicillinase, partial [Enterobacteria phage f1] | 70 |  | ./PDFs/distr\_18nt30nt/B049\_CONTIG64\_sRNA\_size\_profile\_raw\_read\_count.pdf | ./PDFs/distr\_25nt30nt/B049\_CONTIG64\_sRNA\_size\_profile\_raw\_read\_count.pdf |
| 54 | CONTIG72 | QPI13029.1 | 1.080000e-13 | 97.143 | polyprotein [Iflavirus IricIV-1] | 109 |  | ./PDFs/distr\_18nt30nt/B049\_CONTIG72\_sRNA\_size\_profile\_raw\_read\_count.pdf | ./PDFs/distr\_25nt30nt/B049\_CONTIG72\_sRNA\_size\_profile\_raw\_read\_count.pdf |
| 55 | CONTIG78 | ASY03250.1 | 1.850000e-58 | 98.936 | RNA-dependent RNA-polymerase [Bronnoya virus] | 319 |  | ./PDFs/distr\_18nt30nt/B049\_CONTIG78\_sRNA\_size\_profile\_raw\_read\_count.pdf | ./PDFs/distr\_25nt30nt/B049\_CONTIG78\_sRNA\_size\_profile\_raw\_read\_count.pdf |
| 56 | CONTIG88 | QPI13032.1 | 6.600000e-24 | 97.917 | polyprotein [Iflavirus IricIV-5] | 146 |  | ./PDFs/distr\_18nt30nt/B049\_CONTIG88\_sRNA\_size\_profile\_raw\_read\_count.pdf | ./PDFs/distr\_25nt30nt/B049\_CONTIG88\_sRNA\_size\_profile\_raw\_read\_count.pdf |
| 57 | CONTIG99 | USZ80667.1 | 7.260000e-52 | 76.415 | hypothetical protein, partial [Sichuan tick toti-like virus] | 319 |  | ./PDFs/distr\_18nt30nt/B049\_CONTIG99\_sRNA\_size\_profile\_raw\_read\_count.pdf | ./PDFs/distr\_25nt30nt/B049\_CONTIG99\_sRNA\_size\_profile\_raw\_read\_count.pdf |
| 58 | CONTIG22 | DAD54753.1 | 7.130000e-27 | 100.000 | TPA\_asm: polyprotein [Iflavirus IricIV-3] | 155 |  | ./PDFs/distr\_18nt30nt/B049\_CONTIG22\_sRNA\_size\_profile\_raw\_read\_count.pdf | ./PDFs/distr\_25nt30nt/B049\_CONTIG22\_sRNA\_size\_profile\_raw\_read\_count.pdf |
| 59 | CONTIG110 | CAA84692.1 | 6.160000e-04 | 100.000 | beta lactamase [Escherichia phage phiX174] | 54 |  | ./PDFs/distr\_18nt30nt/B049\_CONTIG110\_sRNA\_size\_profile\_raw\_read\_count.pdf | ./PDFs/distr\_25nt30nt/B049\_CONTIG110\_sRNA\_size\_profile\_raw\_read\_count.pdf |
| 60 | CONTIG185 | CAA84692.1 | 1.020000e-07 | 100.000 | beta lactamase [Escherichia phage phiX174] | 104 |  | ./PDFs/distr\_18nt30nt/B049\_CONTIG185\_sRNA\_size\_profile\_raw\_read\_count.pdf | ./PDFs/distr\_25nt30nt/B049\_CONTIG185\_sRNA\_size\_profile\_raw\_read\_count.pdf |
