# Supplementary material for: A novel eukaryotic RdRP-dependent small RNA pathway represses antiviral immunity by controlling an ERK pathway component in the black-legged tick
Source: PLoS One. 2023 Mar 30;18(3):e0281195. doi: 10.1371/journal.pone.0281195 (PMC10062562; doi:10.1371/journal.pone.0281195)
Supplement: S1 Data — On the Normalized and Relative levels pages, normalized read counts (RPM) and relative levels compared to the control (dsGFP) library were used. TEs with more than 50RPM and Coding Genes with more than 3.5RPM on average in KD libraries are included in this website. For viral sequences, reference sequences were generated by assembling sRNA sequences (See the Analysis of sRNAseq data section in Materials and methods.) and size distributions of sRNA reads mapped to each contig are shown. Reads were first grouped by their 5’ nucleotides, and reads of each length were counted. Full tables including TEs and Coding genes with fewer reads are in the “FullTable” folder. (ZIP) [file pone.0281195.s017.zip › SupplementaryData/Links/CDS_Relative.html]

 


## CDS mapping table (Relative level compared to GFP control)

| ID | GFP | Ago-16 | Ago-30 | Ago-96 | Ago-78 | Aub | AGO3-1 | AGO3-2 | RdRP1 | RdRP3 | SUM\_KD |
| --- | --- | --- | --- | --- | --- | --- | --- | --- | --- | --- | --- |
| ISCI012234.RA | 1 | 0.42342196 | 1.42218036 | 0.87947184 | 1.9295286 | 1.51873925 | 1.36087145 | 1.45611576 | 1.15789739 | 0.28251798 | 6437.86105 |
| ISCI005507.RA | 1 | 1.36093553 | 0.7550073 | 1.24797125 | 1.02713914 | 0.43273448 | 0.52918475 | 0.60834739 | 1.59080199 | 1.12014133 | 3624.28861 |
| ISCI011233.RA | 1 | 1.3420158 | 1.18565991 | 0.94078484 | 1.06655343 | 0.14903487 | 0.36870337 | 0.42571768 | 1.91498092 | 1.57375048 | 2638.36889 |
| ISCI013597.RA | 1 | 1.18280005 | 1.02396499 | 1.09105796 | 1.21966699 | 0.817011 | 0.89475289 | 1.07697389 | 1.75069076 | 1.34382898 | 2584.51701 |
| ISCI009240.RA | 1 | 0.93010923 | 1.18977372 | 1.52927103 | 1.43322493 | 1.79573247 | 1.58856255 | 1.33401054 | 0.10252305 | 1.154665 | 2436.48548 |
| ISCI017315.RA | 1 | 0.66627823 | 0.95277939 | 1.05567753 | 1.09767857 | 0.77417672 | 0.52033636 | 0.62694015 | 1.48830685 | 0.79295692 | 1973.45298 |
| ISCI007738.RA | 1 | 1.36745049 | 1.0520388 | 1.09592545 | 1.4007239 | 0.60923367 | 0.18499885 | 0.20212685 | 2.04038035 | 1.60563204 | 1574.67285 |
| ISCI024113.RA | 1 | 1.10989339 | 0.95040506 | 1.06765744 | 0.94685957 | 0.86427168 | 0.50986376 | 0.5786106 | 1.40600087 | 1.1231206 | 1515.60895 |
| ISCI024064.RA | 1 | 1.00205487 | 0.9863126 | 1.18671181 | 1.65343587 | 0.93443159 | 0.80170654 | 1.05148292 | 1.62632229 | 1.01380339 | 1305.92335 |
| ISCI024649.RA | 1 | 1.09266976 | 1.0995202 | 0.94850118 | 0.94818661 | 1.11701215 | 0.55114066 | 0.71952334 | 1.5016125 | 1.16754922 | 1305.61972 |
| ISCI010315.RA | 1 | 1.31911424 | 1.19499245 | 1.00797738 | 0.91591494 | 0.33293701 | 0.32443461 | 0.30616597 | 1.67773277 | 1.32344294 | 1002.29043 |
| ISCI024767.RA | 1 | 1.19903982 | 1.08031561 | 0.89548956 | 1.01018224 | 0.48331819 | 0.41038743 | 0.48830264 | 1.42944309 | 1.11466525 | 928.143144 |
| ISCI017059.RA | 1 | 0.54770171 | 1.07862756 | 0.89875261 | 1.01790806 | 2.34814585 | 0.78128853 | 1.11129414 | 1.73502707 | 0.91113701 | 875.468496 |
| ISCI003449.RA | 1 | 1.14855157 | 0.98549288 | 0.91587585 | 0.54785985 | 1.1682167 | 0.37507589 | 0.33633141 | 1.39257374 | 0.93441614 | 852.163311 |
| ISCI024070.RA | 1 | 0.88000988 | 0.9688217 | 0.9298142 | 1.06346148 | 1.5633218 | 0.6626857 | 1.00828043 | 1.52756791 | 1.14825695 | 851.914112 |
| ISCI009768.RA | 1 | 0.2692515 | 0.9027875 | 1.12520153 | 0.67510223 | 1.01580428 | 1.03086667 | 1.2922099 | 1.56734257 | 0.24780344 | 805.852331 |
| ISCI002834.RA | 1 | 1.18601878 | 1.11372338 | 0.93764298 | 1.0846214 | 0.50469128 | 0.36918691 | 0.37488545 | 1.16321758 | 0.93846402 | 795.331658 |
| ISCI005745.RA | 1 | 0.59222336 | 1.06178774 | 0.81608761 | 0.96480569 | 0.88903502 | 0.97474661 | 1.10071127 | 1.53332819 | 0.87600492 | 773.690497 |
| ISCI019388.RA | 1 | 1.08533716 | 1.20433564 | 0.96518242 | 1.04327477 | 0.21626145 | 0.33340944 | 0.28286933 | 1.49689332 | 1.28831246 | 745.981703 |
| ISCI009034.RA | 1 | 1.06255085 | 1.28843356 | 1.47492414 | 1.51200876 | 1.8890358 | 1.68384108 | 1.30152321 | 0.23502856 | 1.44975838 | 731.14182 |
| ISCI021730.RA | 1 | 0.67190707 | 6.48894037 | 0.72988038 | 2.79525559 | 1.89391615 | 1.14124499 | 1.20239643 | 0.8084677 | 0.76074592 | 694.676419 |
| ISCI022167.RA | 1 | 1.0853487 | 1.01113084 | 1.05825381 | 1.65618546 | 1.6888114 | 1.16404365 | 1.27142399 | 0.29033307 | 0.75346422 | 689.127205 |
| ISCI010273.RA | 1 | 1.01261209 | 1.27727848 | 1.36817232 | 1.38117975 | 1.6068121 | 1.47102266 | 1.14327297 | 0.18996269 | 1.30873244 | 659.818586 |
| ISCI003367.RA | 1 | 0.82441332 | 1.15604142 | 0.97459227 | 1.31868628 | 13.739794 | 3.5432371 | 6.11463446 | 1.51757815 | 1.18645247 | 655.784848 |
| ISCI004588.RA | 1 | 1.31429759 | 1.02710508 | 0.87464769 | 0.94579357 | 0.29774858 | 0.45256813 | 0.42452786 | 1.52460337 | 1.34098014 | 648.031407 |
| ISCI017737.RA | 1 | 1.0522722 | 1.01645405 | 0.85230436 | 0.67089216 | 0.72473946 | 0.6461504 | 0.92729173 | 0.93125348 | 0.77792725 | 607.22761 |
| ISCI011355.RA | 1 | 0.8899317 | 1.05404667 | 1.15167585 | 1.4607084 | 0.47942147 | 0.40927712 | 0.52744169 | 1.31521151 | 1.05447755 | 525.10613 |
| ISCI006668.RA | 1 | 1.12219307 | 1.19034981 | 0.91589779 | 0.88795381 | 0.16842028 | 0.35589447 | 0.27048823 | 1.46113868 | 1.19882602 | 523.660869 |
| ISCI015077.RA | 1 | 1.59820355 | 1.1647996 | 1.18964071 | 1.96574386 | 0.1840426 | 0.39551749 | 0.48397638 | 2.00181324 | 1.75635063 | 485.477834 |
| ISCI012349.RA | 1 | 1.13305235 | 1.22132699 | 0.96206628 | 1.00539879 | 0.24447924 | 0.33099745 | 0.33942888 | 1.19458733 | 0.97747372 | 483.458157 |
| ISCI017738.RA | 1 | 1.1141172 | 1.26621667 | 1.14458925 | 1.22762375 | 0.54482402 | 0.45780021 | 0.48380134 | 1.47945321 | 1.18119998 | 482.586191 |
| ISCI024452.RA | 1 | 0.79812161 | 1.20470618 | 0.96848694 | 1.21379824 | 0.860082 | 0.98659318 | 0.903483 | 1.70650373 | 1.34771656 | 449.491754 |
| ISCI018019.RA | 1 | 1.17981451 | 0.88148605 | 0.97197535 | 0.99018339 | 1.06996479 | 0.48471973 | 0.71010819 | 1.29526363 | 1.09473606 | 442.54089 |
| ISCI009817.RA | 1 | 1.13254187 | 1.16897775 | 1.09098164 | 1.07434602 | 1.72504351 | 0.87007622 | 0.89235993 | 1.70657855 | 1.41124079 | 434.770077 |
| ISCI011625.RA | 1 | 1.20291042 | 1.22818539 | 0.97373619 | 0.98627391 | 2.23995661 | 0.67364933 | 1.05295616 | 1.58330442 | 1.43210888 | 422.531951 |
| ISCI013345.RA | 1 | 1.10650148 | 1.07199218 | 0.80470435 | 0.62330346 | 0.0673543 | 0.17518363 | 0.11318085 | 1.59755554 | 1.18428709 | 375.016615 |
| ISCI007332.RA | 1 | 0.65117463 | 1.2467961 | 1.56547209 | 1.07195398 | 1.49220775 | 1.20892071 | 1.00134735 | 0.04848109 | 0.70720863 | 372.122398 |
| ISCI007053.RA | 1 | 0.27678184 | 1.34605825 | 1.15052821 | 1.12790415 | 1.25644108 | 1.25150575 | 1.49582595 | 1.05389842 | 0.28690078 | 366.031762 |
| ISCI001342.RA | 1 | 1.14232385 | 1.25876046 | 1.25717293 | 1.27682731 | 1.01846106 | 0.88571536 | 0.87342713 | 1.66031182 | 1.15143065 | 355.011236 |
