# Supplementary material for: A novel eukaryotic RdRP-dependent small RNA pathway represses antiviral immunity by controlling an ERK pathway component in the black-legged tick
Source: PLoS One. 2023 Mar 30;18(3):e0281195. doi: 10.1371/journal.pone.0281195 (PMC10062562; doi:10.1371/journal.pone.0281195)
Supplement: S1 File — For dot plots, the size and color represent the number of genes in the GO category and the significance of enrichment, respectively, as indicated in the legend. For Gene-GO networks, the linkage between each enriched GO category and the misregulated genes that are involved in the corresponding GO category is depicted. The color of the dots representing the misreguated genes indicates the log2 fold change level. The size of the dots representing the enriched GO categories indicates the number of misregulated genes in the GO category. (PDF) [file pone.0281195.s018.pdf]

# Gene annotation

- **Gene name**
  - -- Uniprot (for protein coding gene)
  - -- Vectorbase(for ncRNA gene)
  - -- Description from EggNOG-mapper
- **GO term info**
  - -- EggNOG-mapper(GO ids for protein coding gene)
  - -- TRAPID(GO Ids for ncRNA gene)

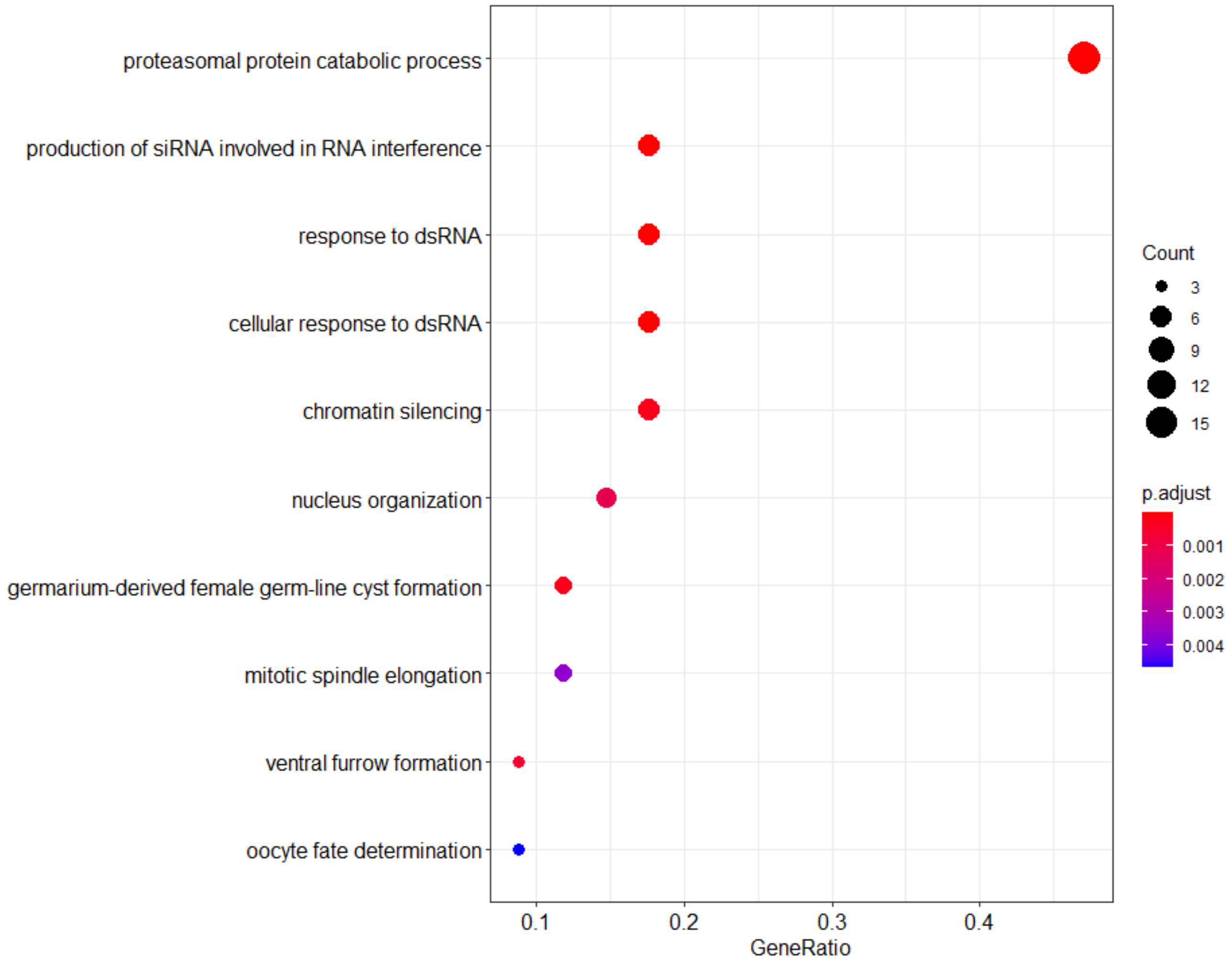


Dot plot showing the result of GO enrichment analysis (Biological Process) for misregulated genes upon Ago-16 knockdown.

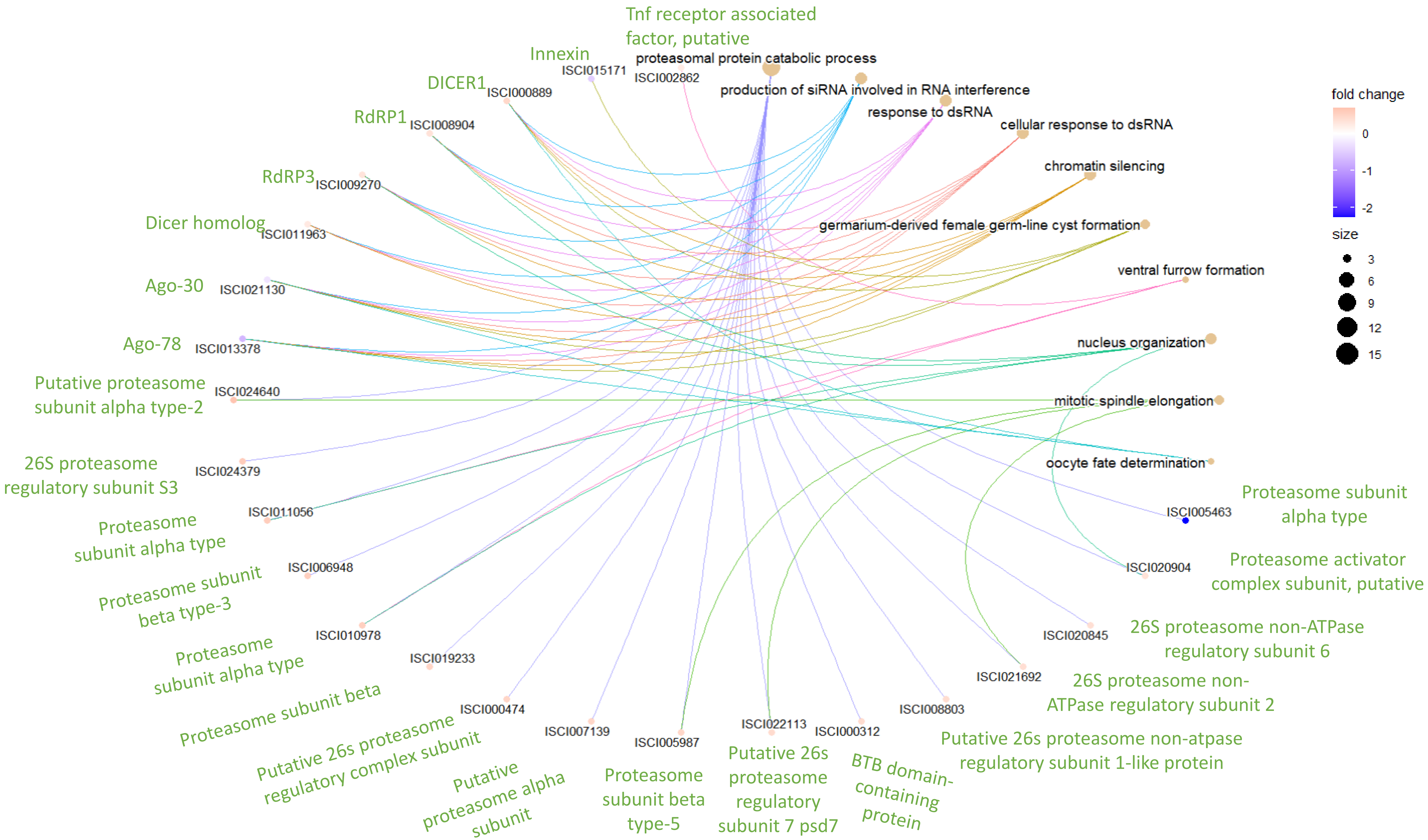


Linkage between GO terms(Biological Process) and genes misregulated upon Ago-16 knockdown.

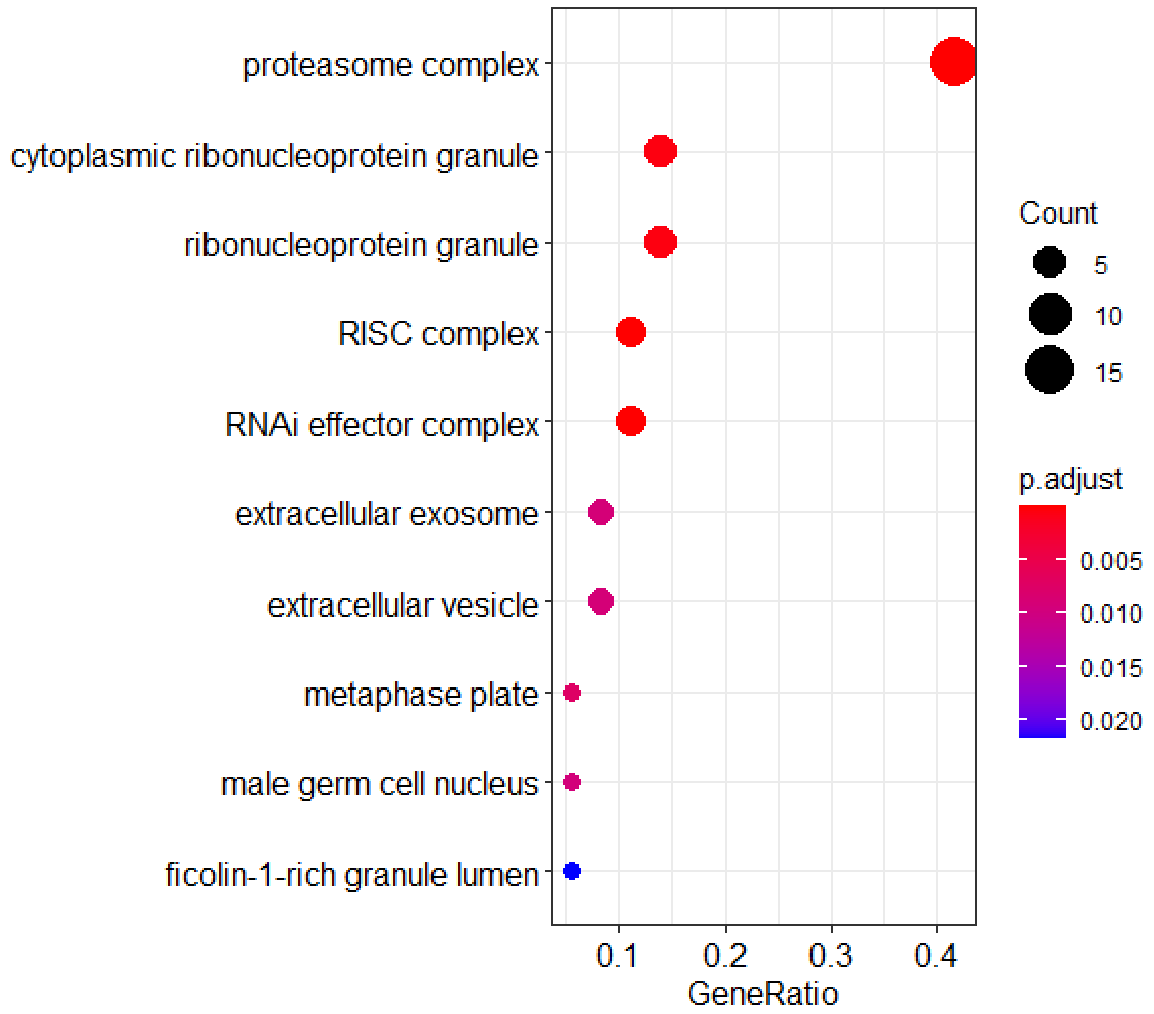


Dot plot showing the result of GO enrichment analysis (Cellular Component) for misregulated genes upon Ago-16 knockdown.

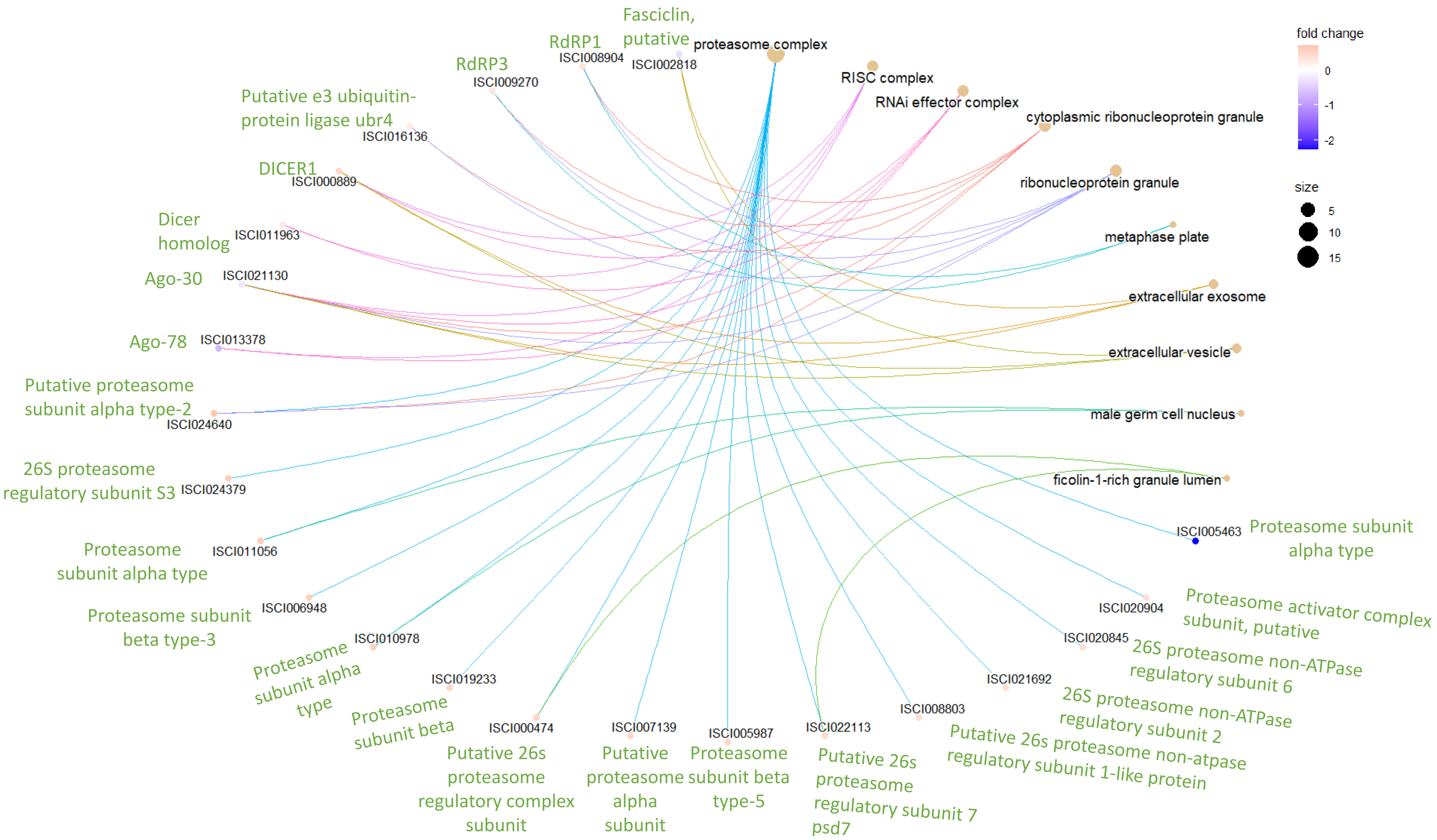


Linkage between GO terms(Cellular Component) and genes misregulated upon Ago-16 knockdown.

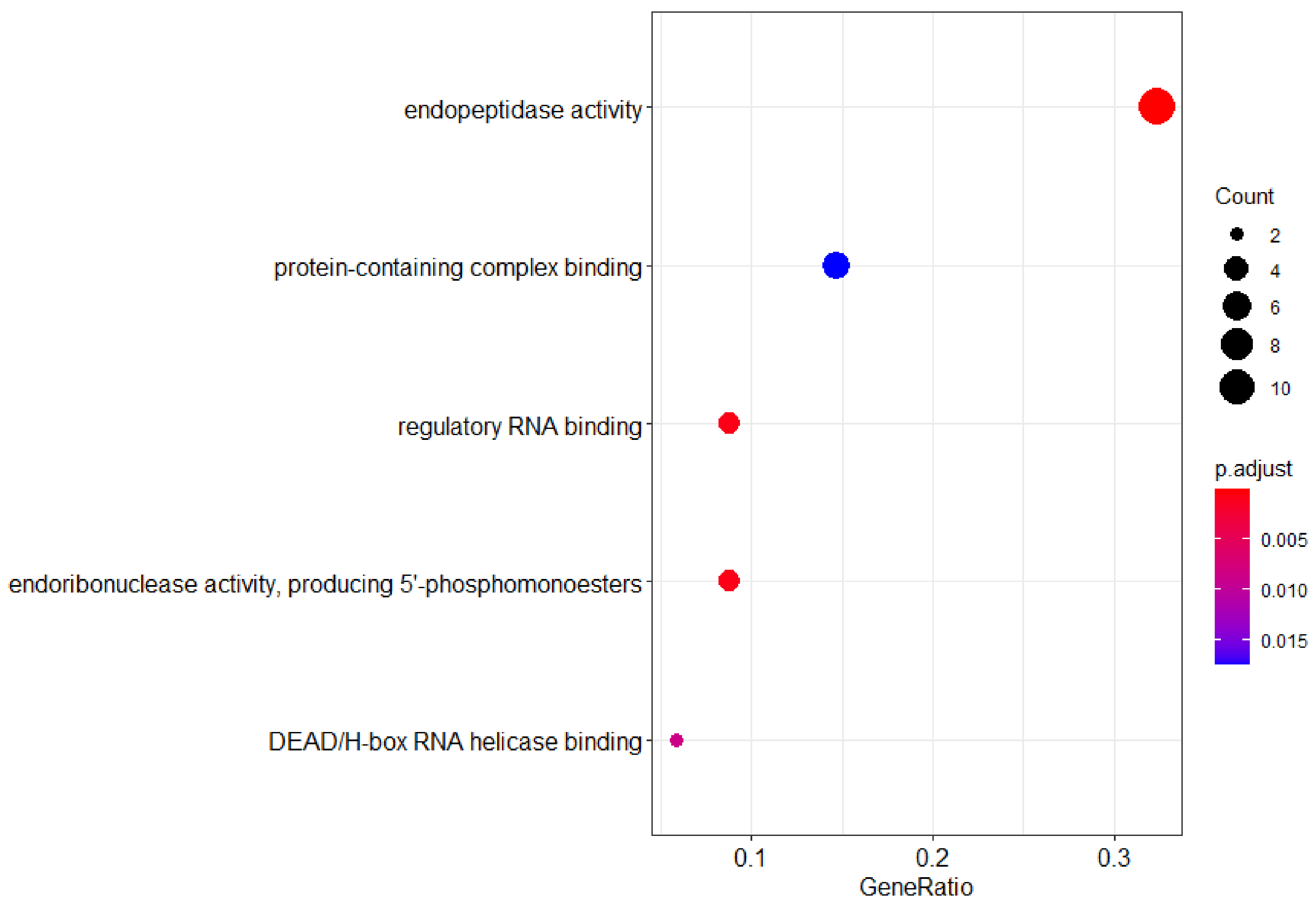


Dot plot showing the result of GO enrichment analysis (Molecular Function) for misregulated genes upon Ago-16 knockdown.

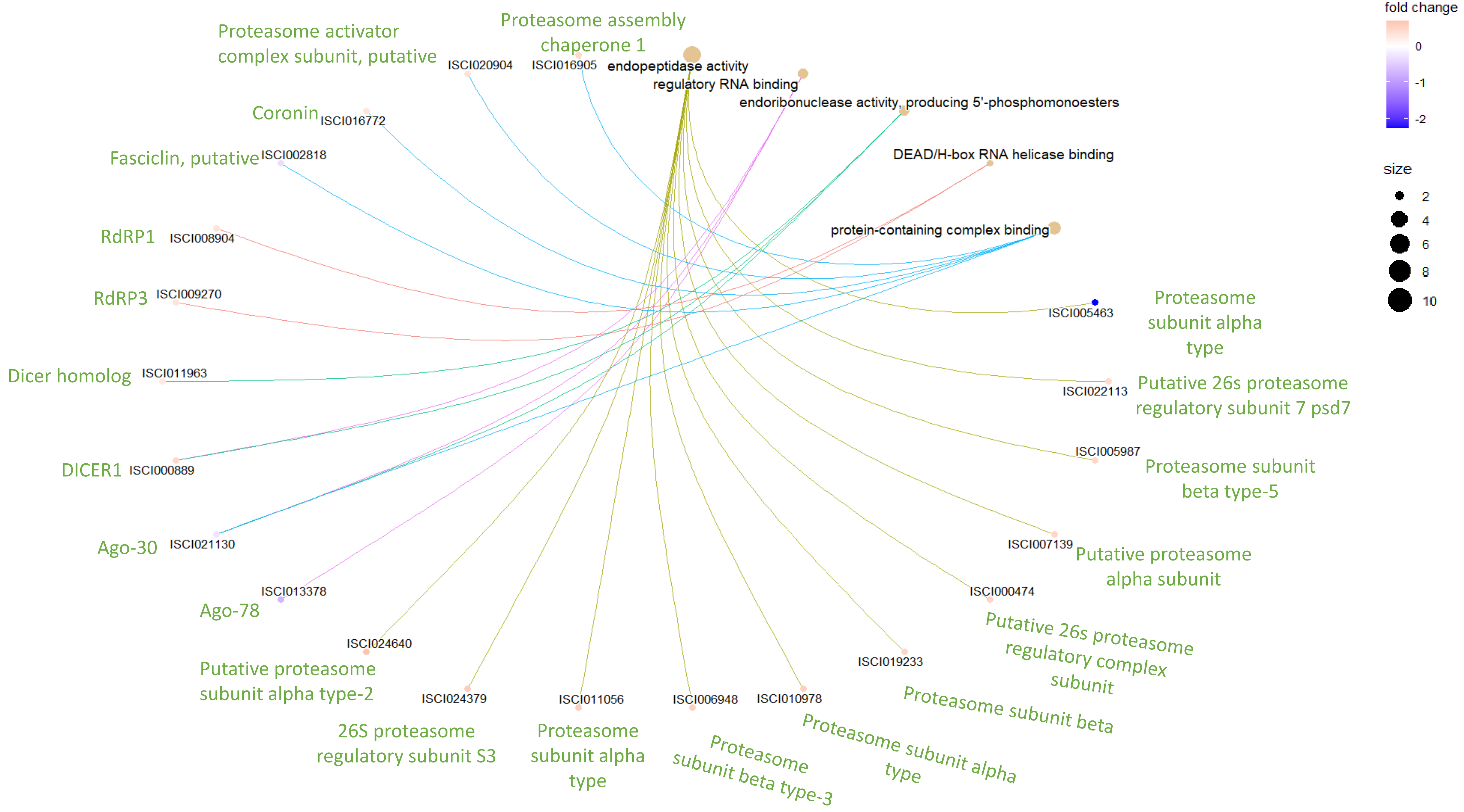


Linkage between GO terms(Molecular Function) and genes misregulated upon Ago-16 knockdown.

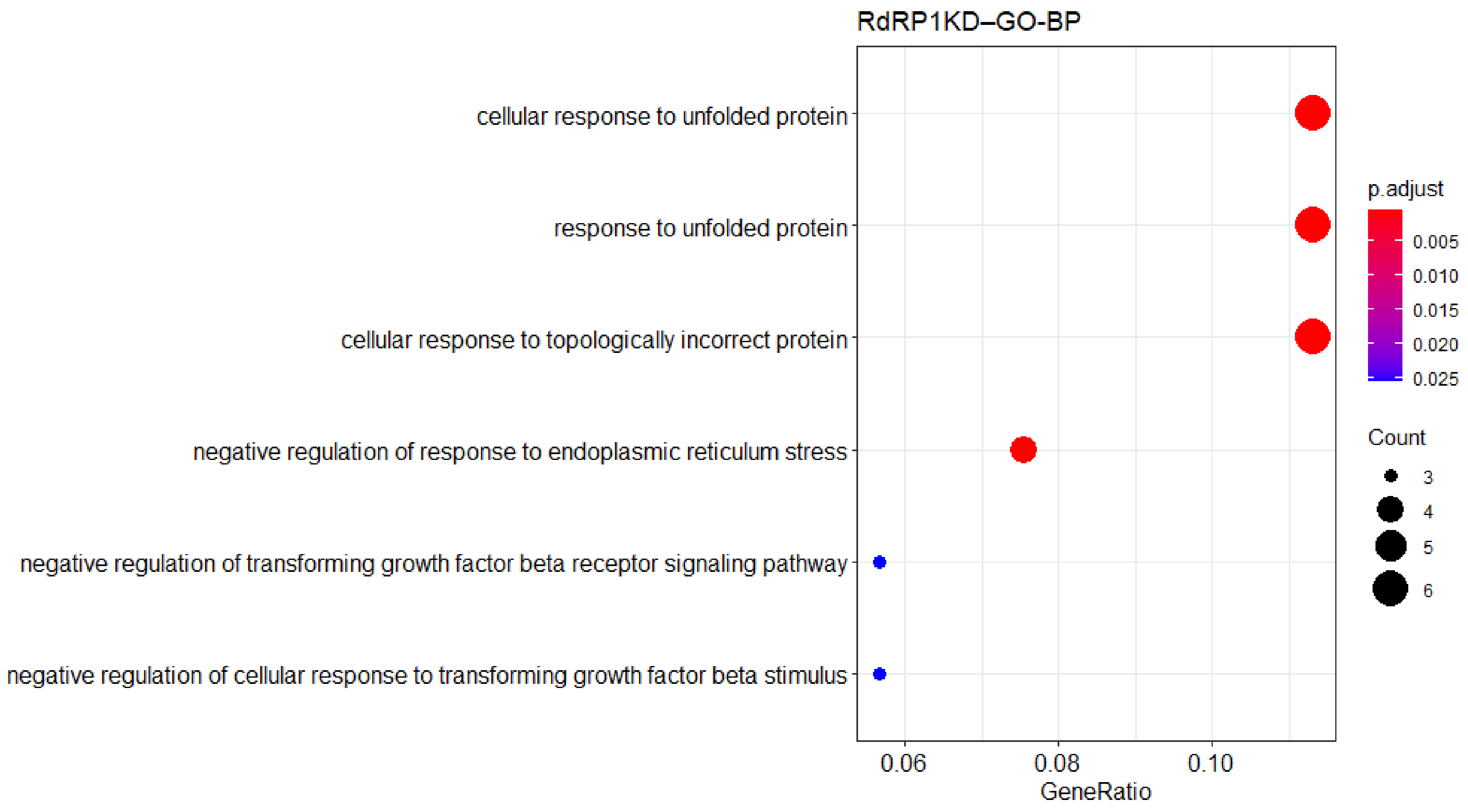


Dot plot showing the result of GO enrichment analysis (Biological Process) for misregulated genes upon RdRP1 knockdown.

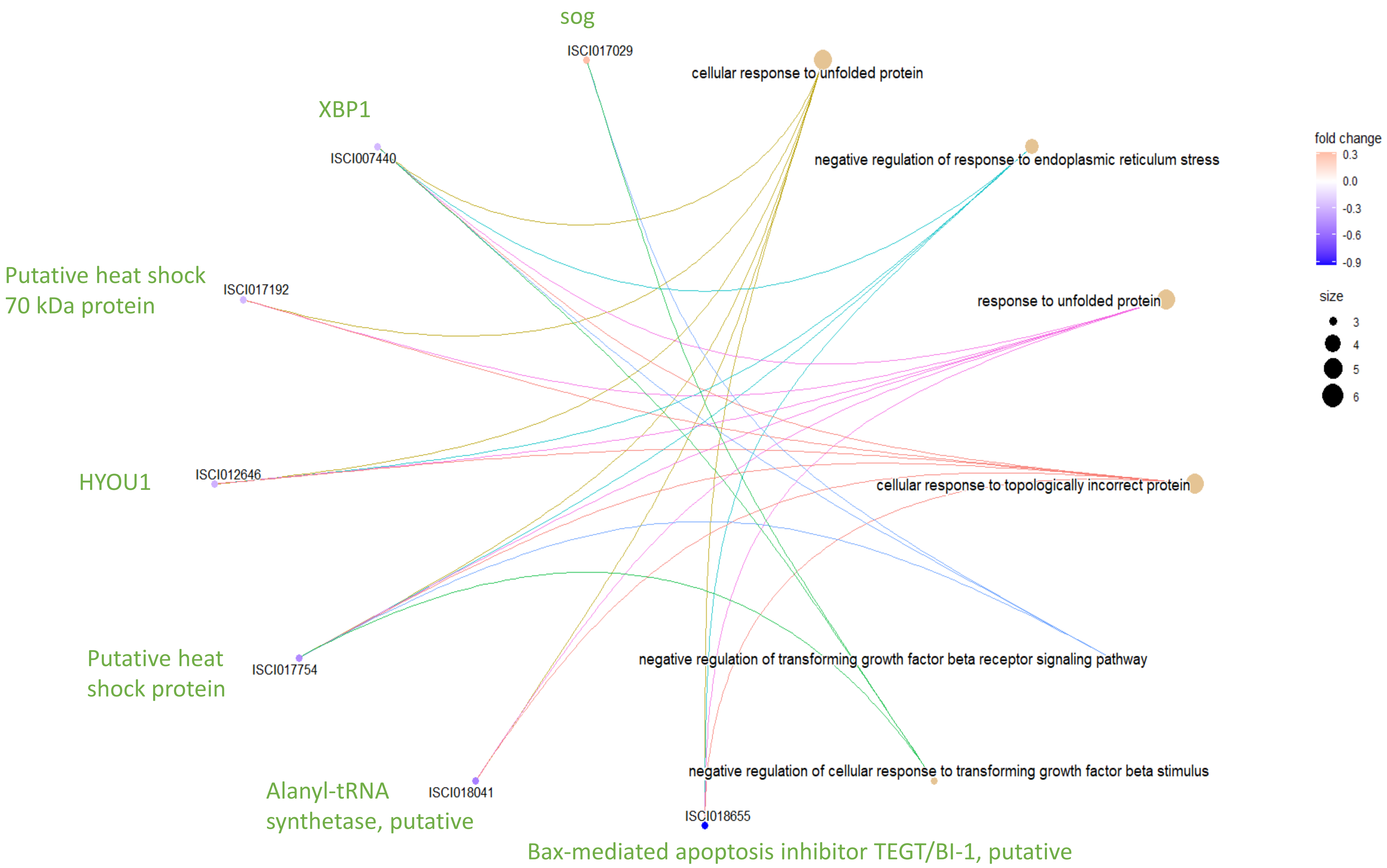


Linkage between GO terms(Biological Process) and genes misregulated upon RdRP1 knockdown.

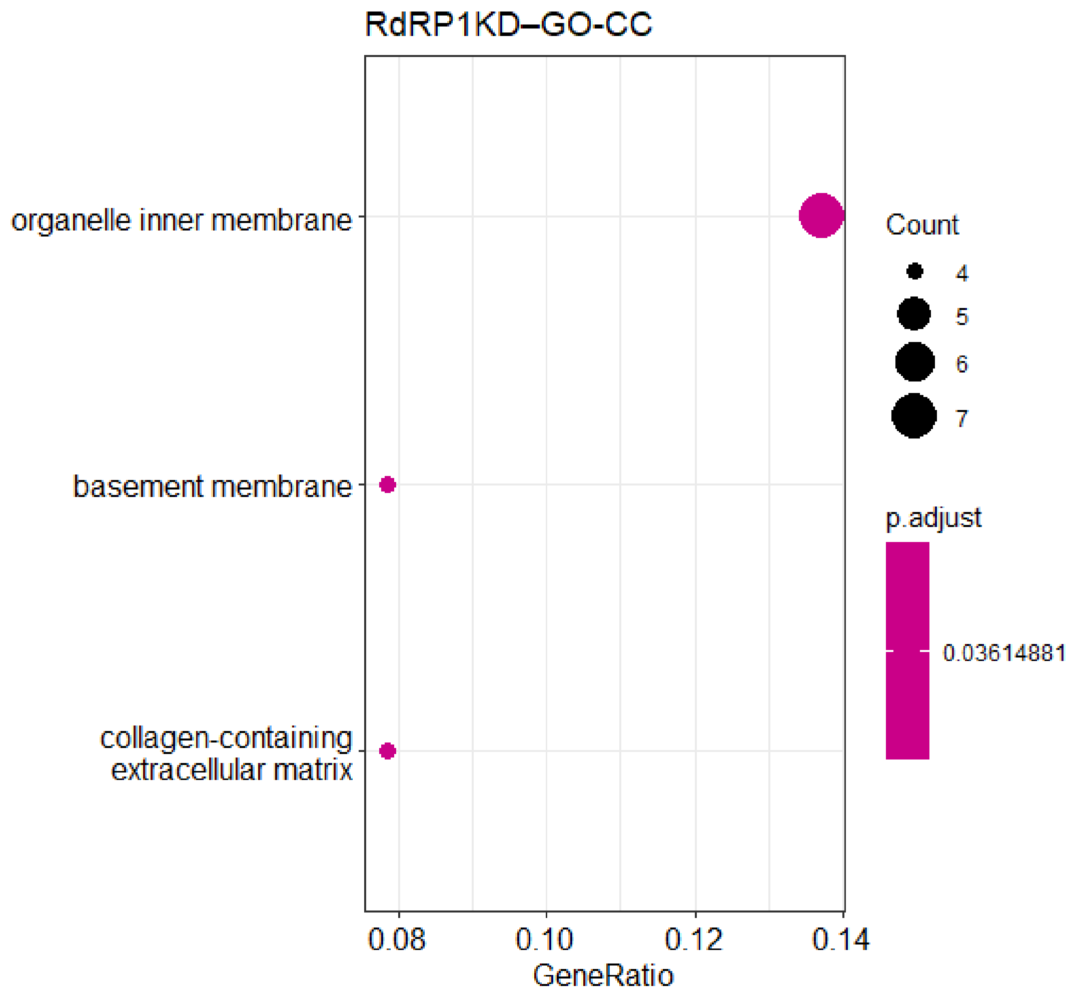


Dot plot showing the result of GO enrichment analysis (Cellular Component) for misregulated genes upon RdRP1 knockdown.

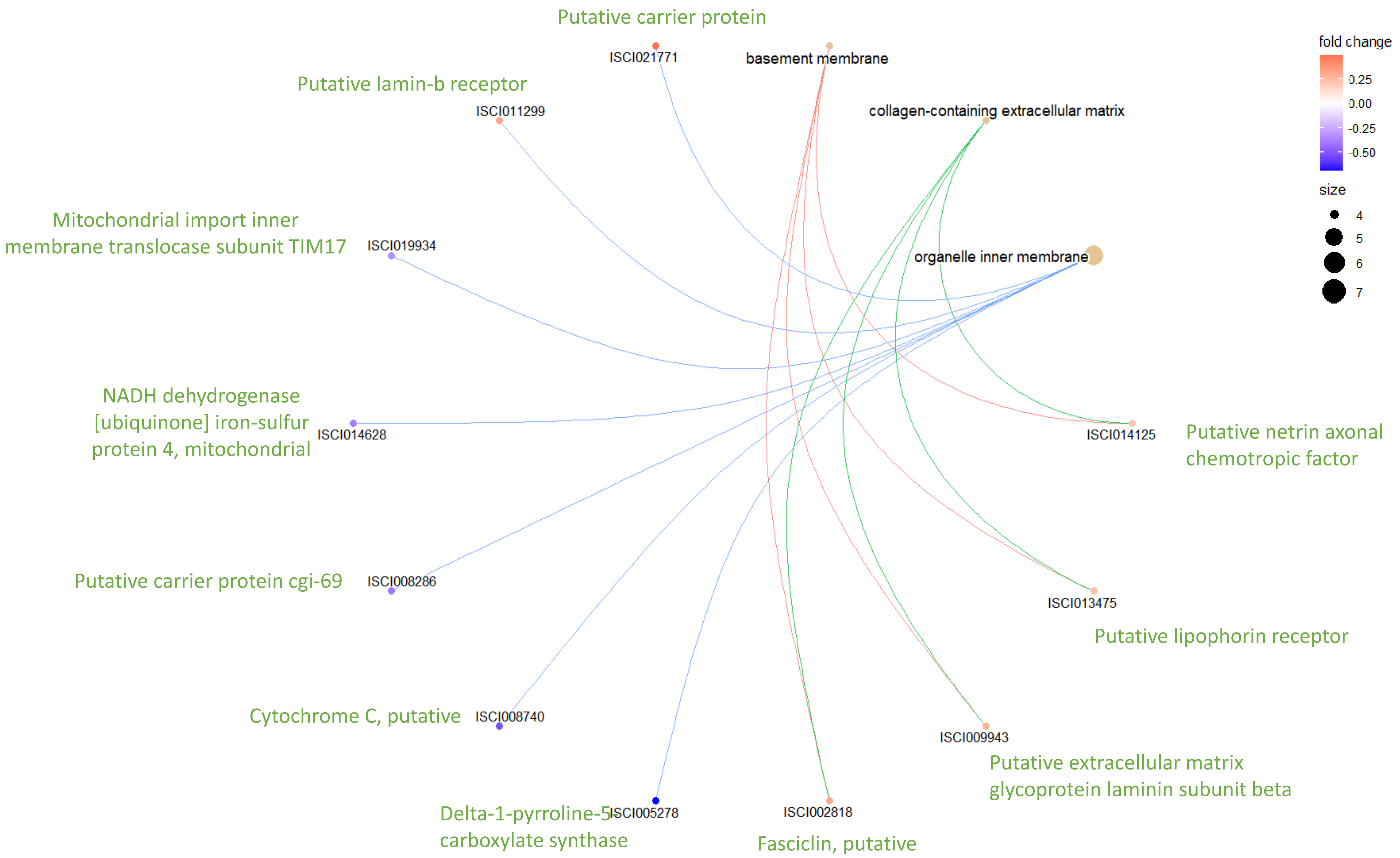


Linkage between GO terms(Cellular Component) and genes misregulated upon RdRP1 knockdown.

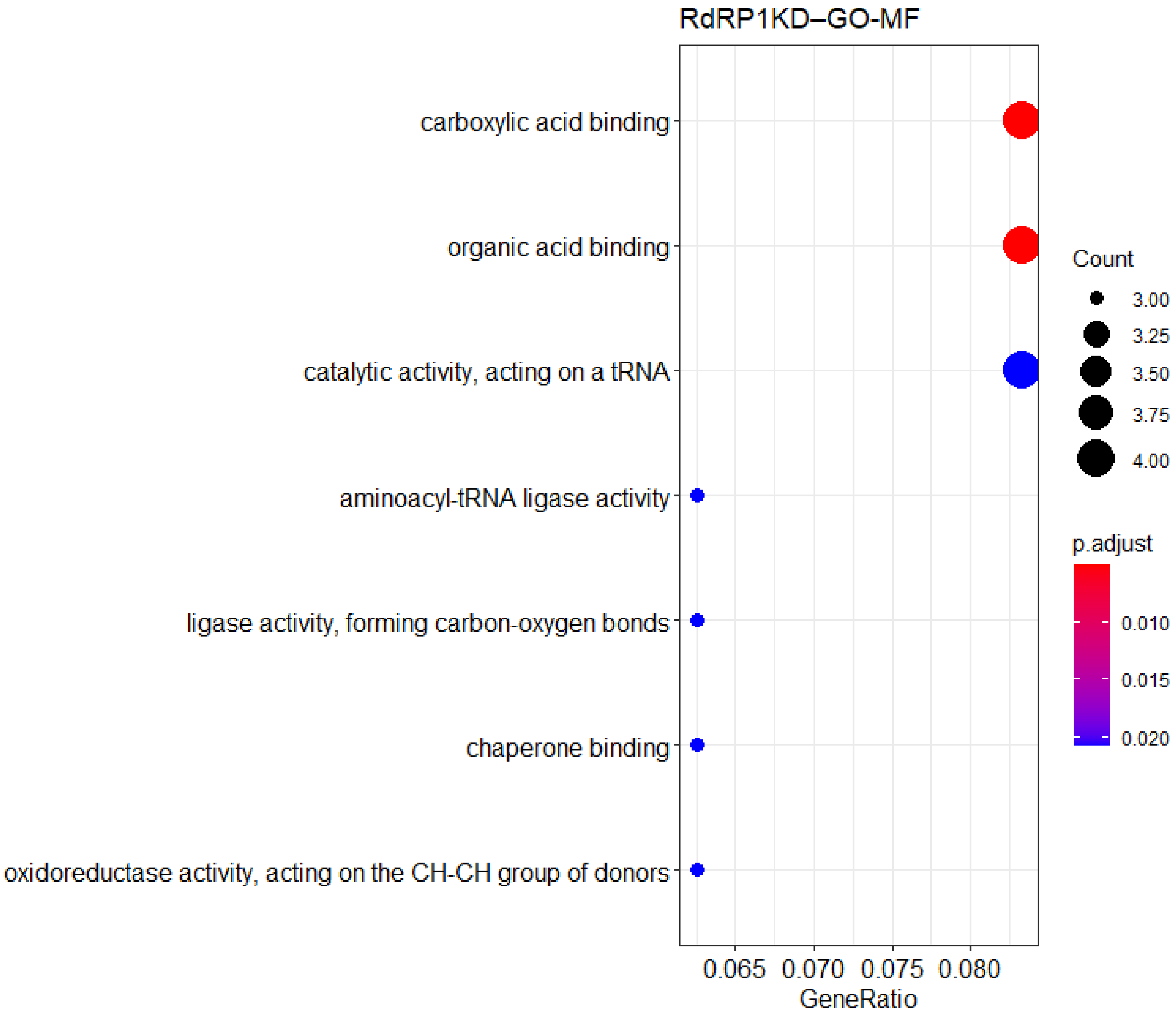


Dot plot showing the result of GO enrichment analysis (Molecular Function) for misregulated genes upon RdRP1 knockdown.

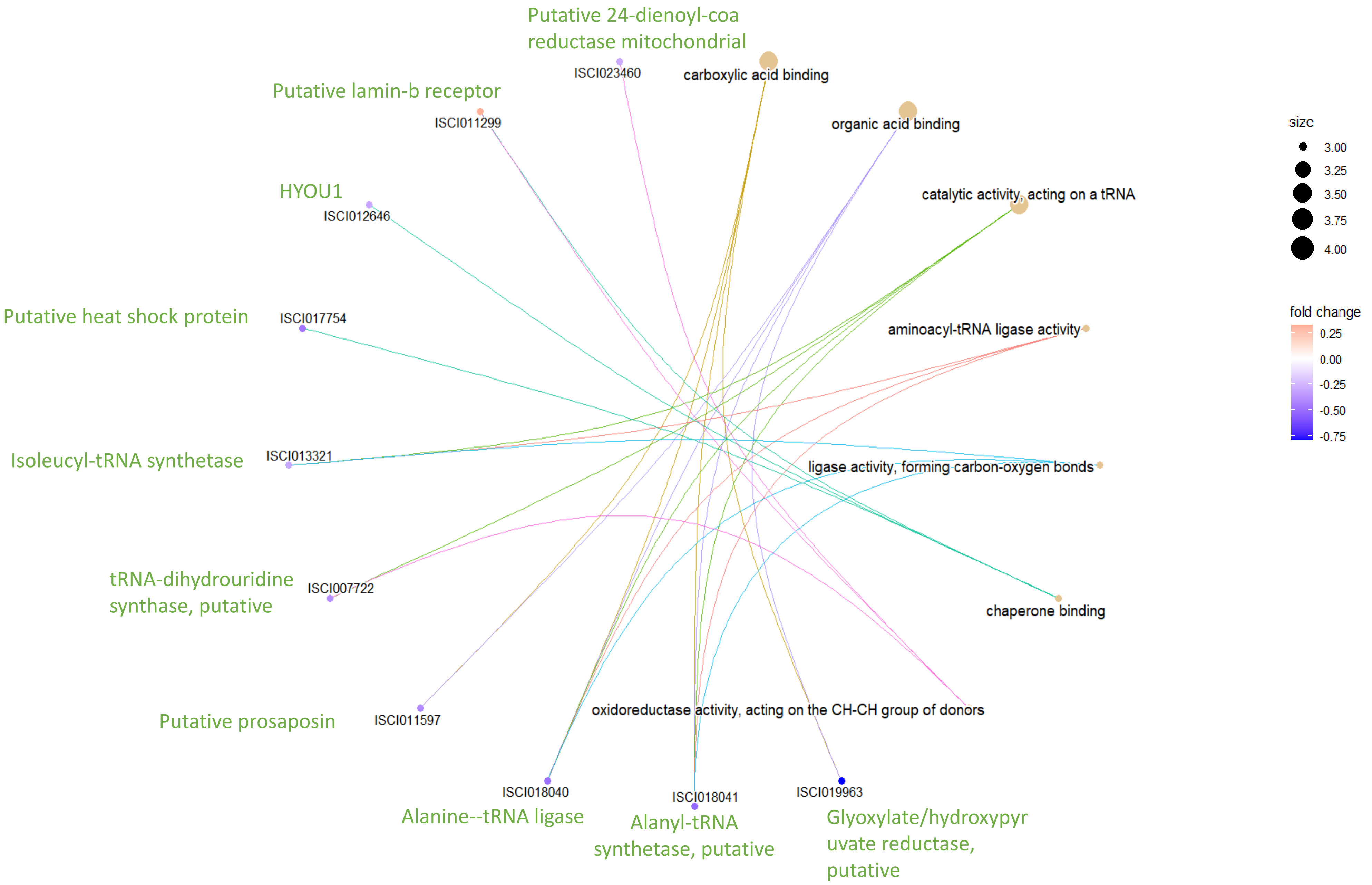


Linkage between GO terms(Molecular Function) and genes misregulated upon RdRP1 knockdown.

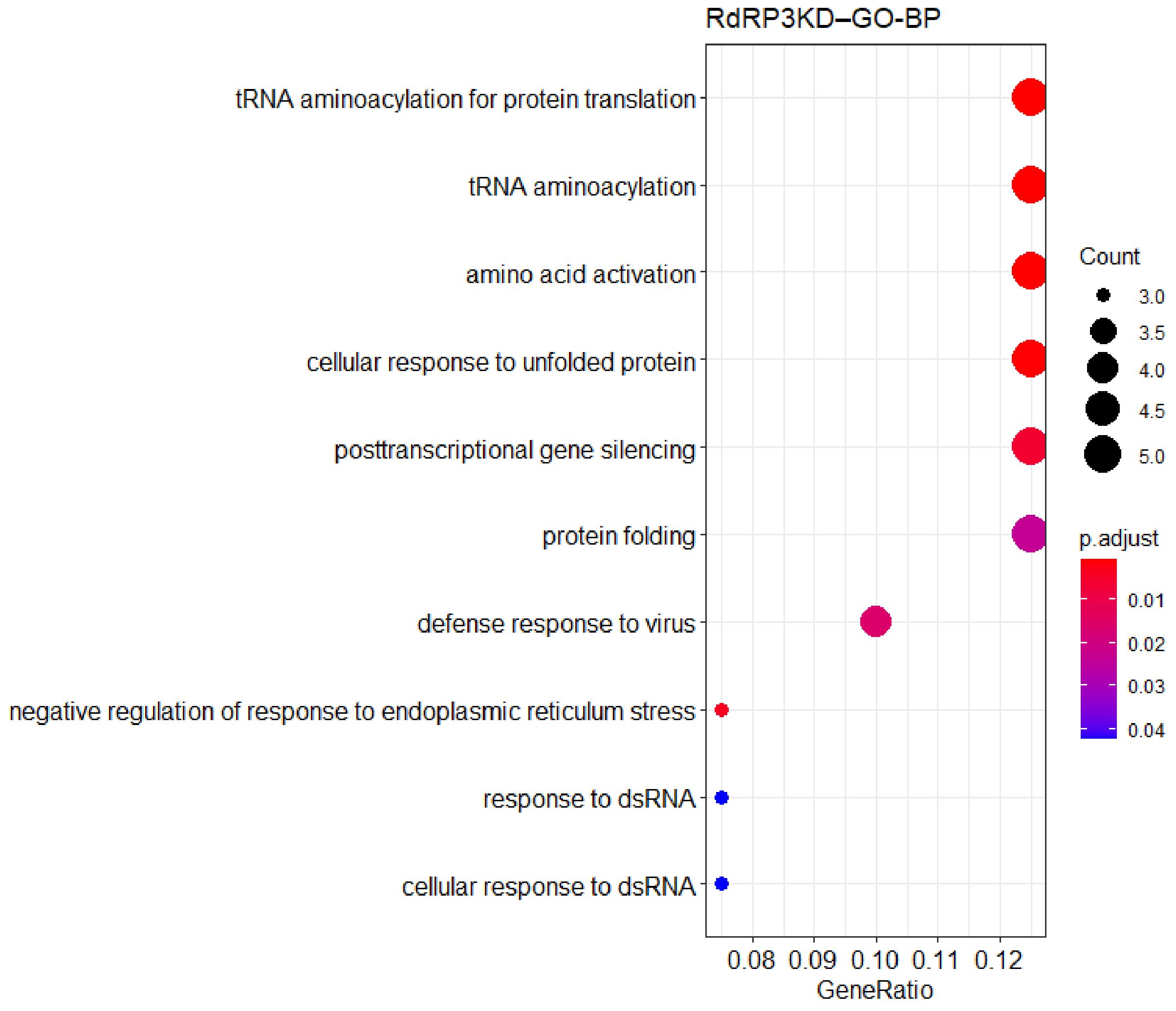


Dot plot showing the result of GO enrichment analysis (Biological Process) for misregulated genes upon RdRP3 knockdown.

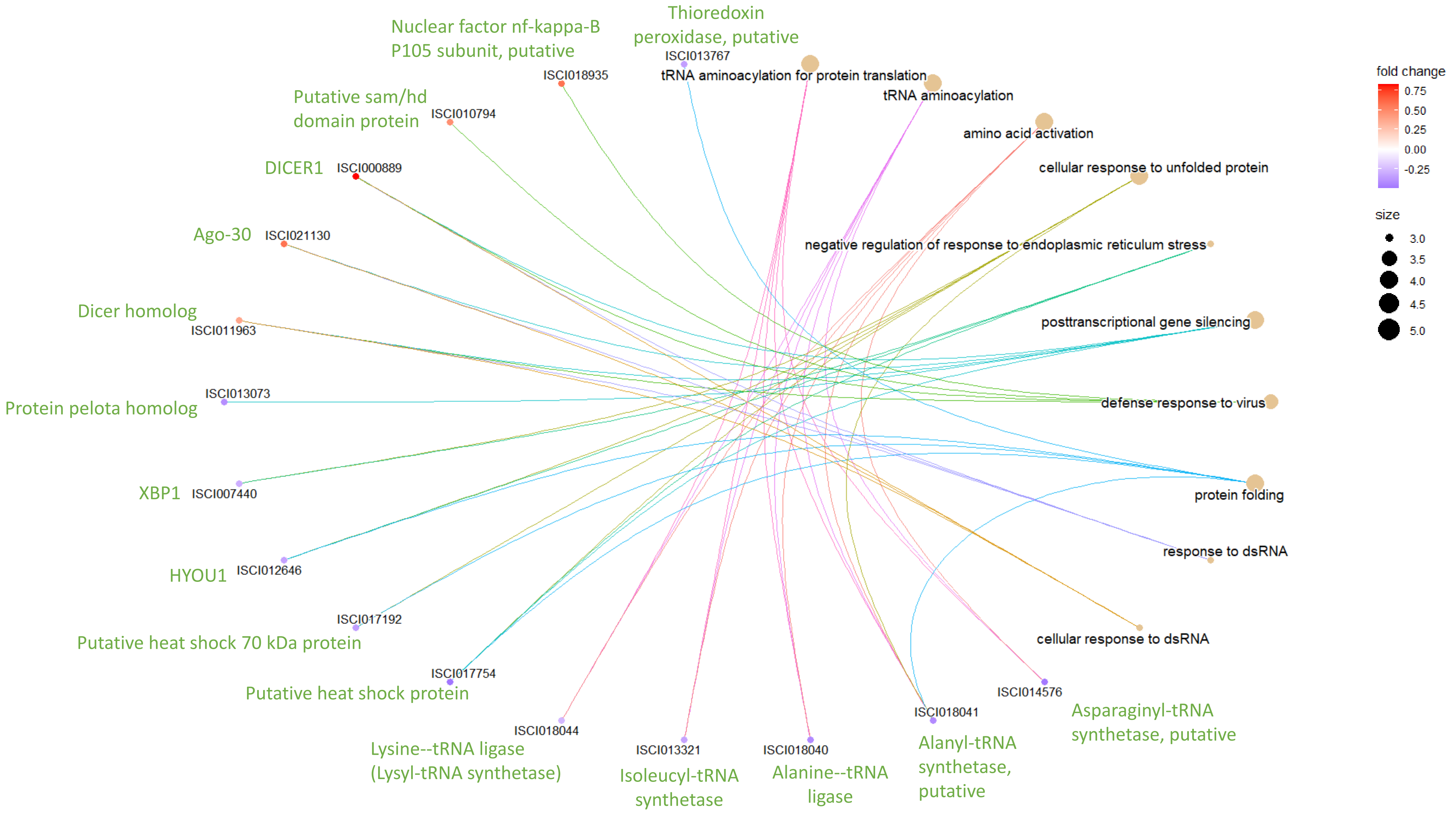


Linkage between GO terms(Biological Process) and genes misregulated upon RdRP3 knockdown.

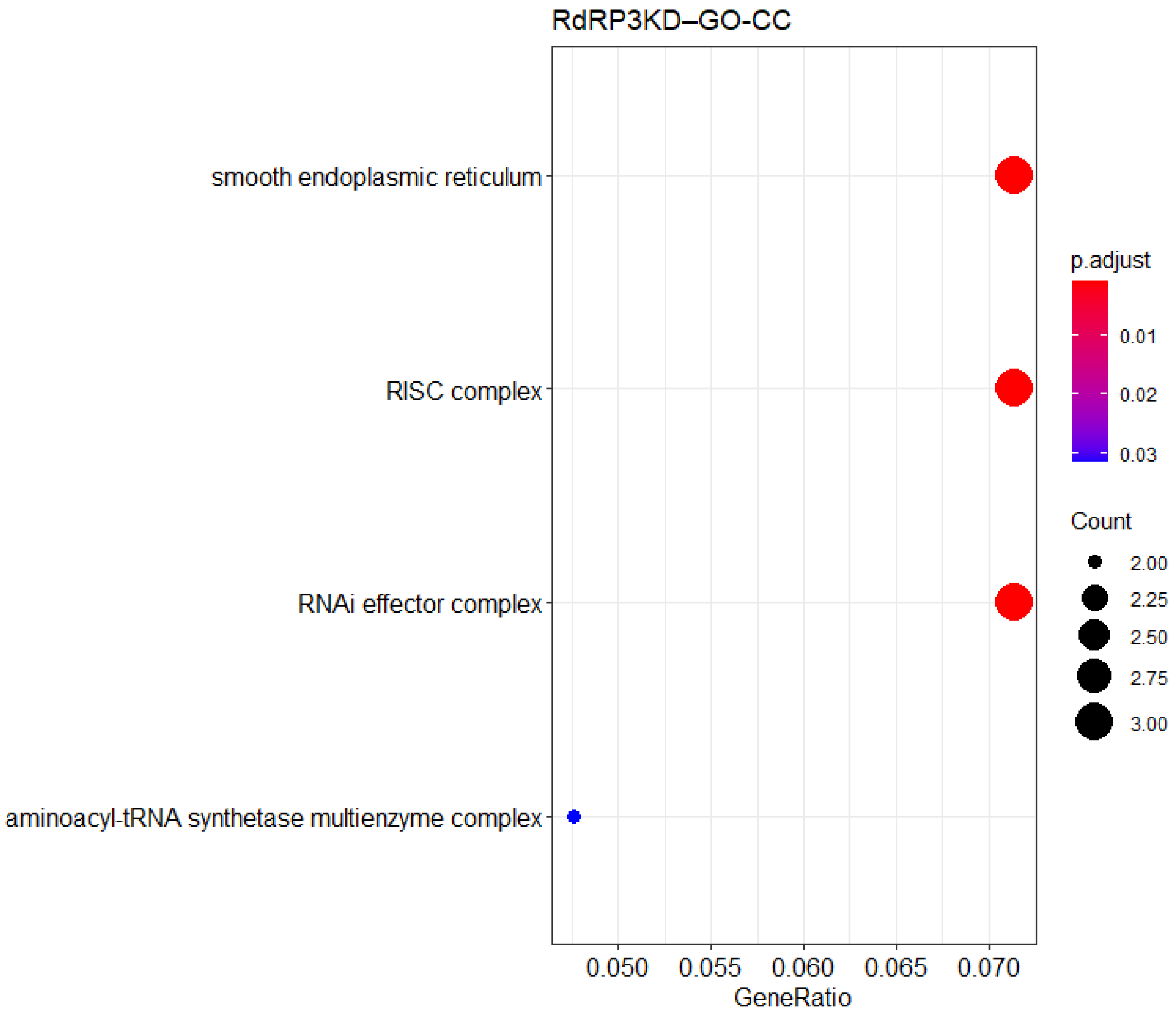


Dot plot showing the result of GO enrichment analysis (Cellular Component) for misregulated genes upon RdRP3 knockdown.

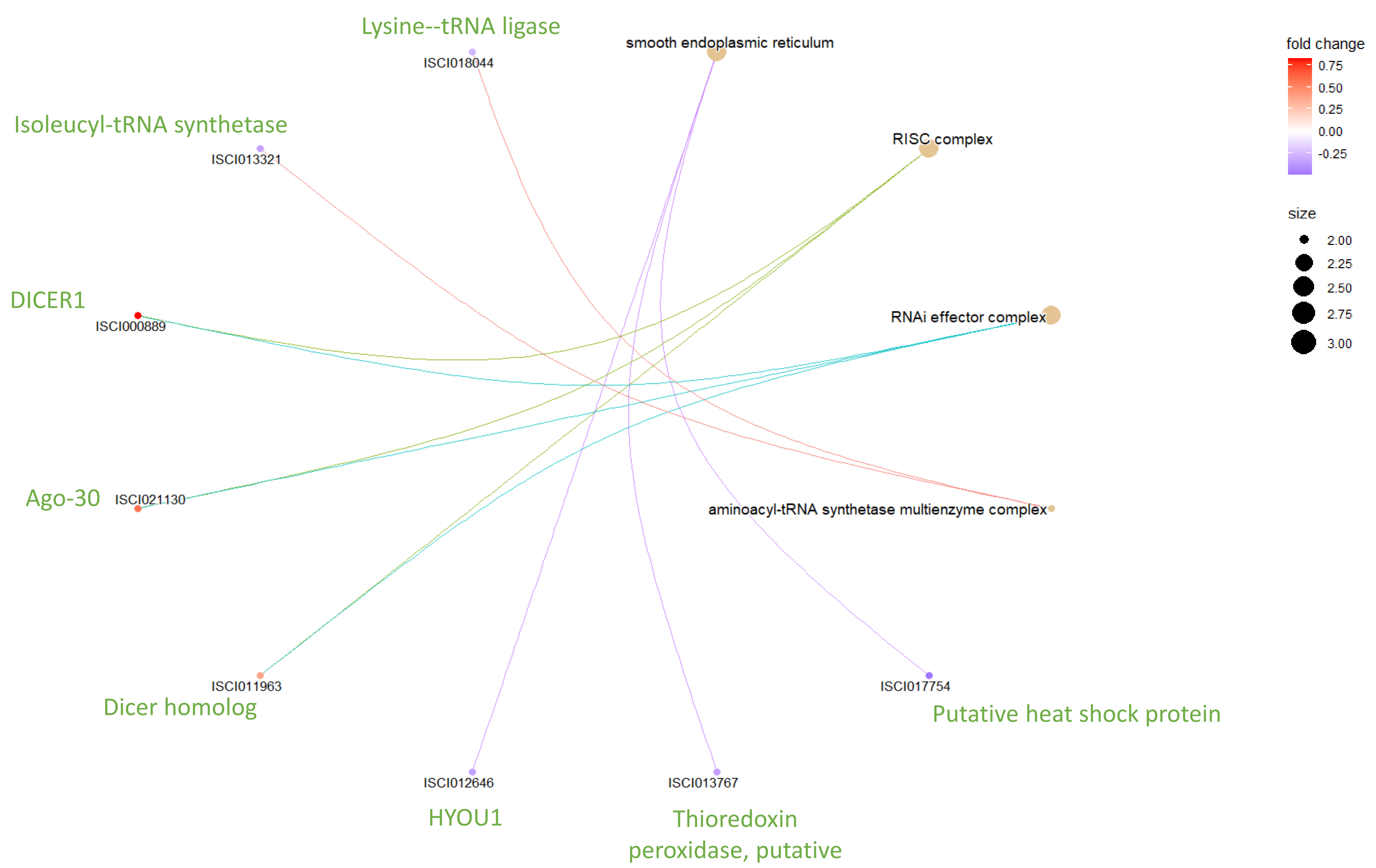


Linkage between GO terms(Cellular Component) and genes misregulated upon RdRP3 knockdown.

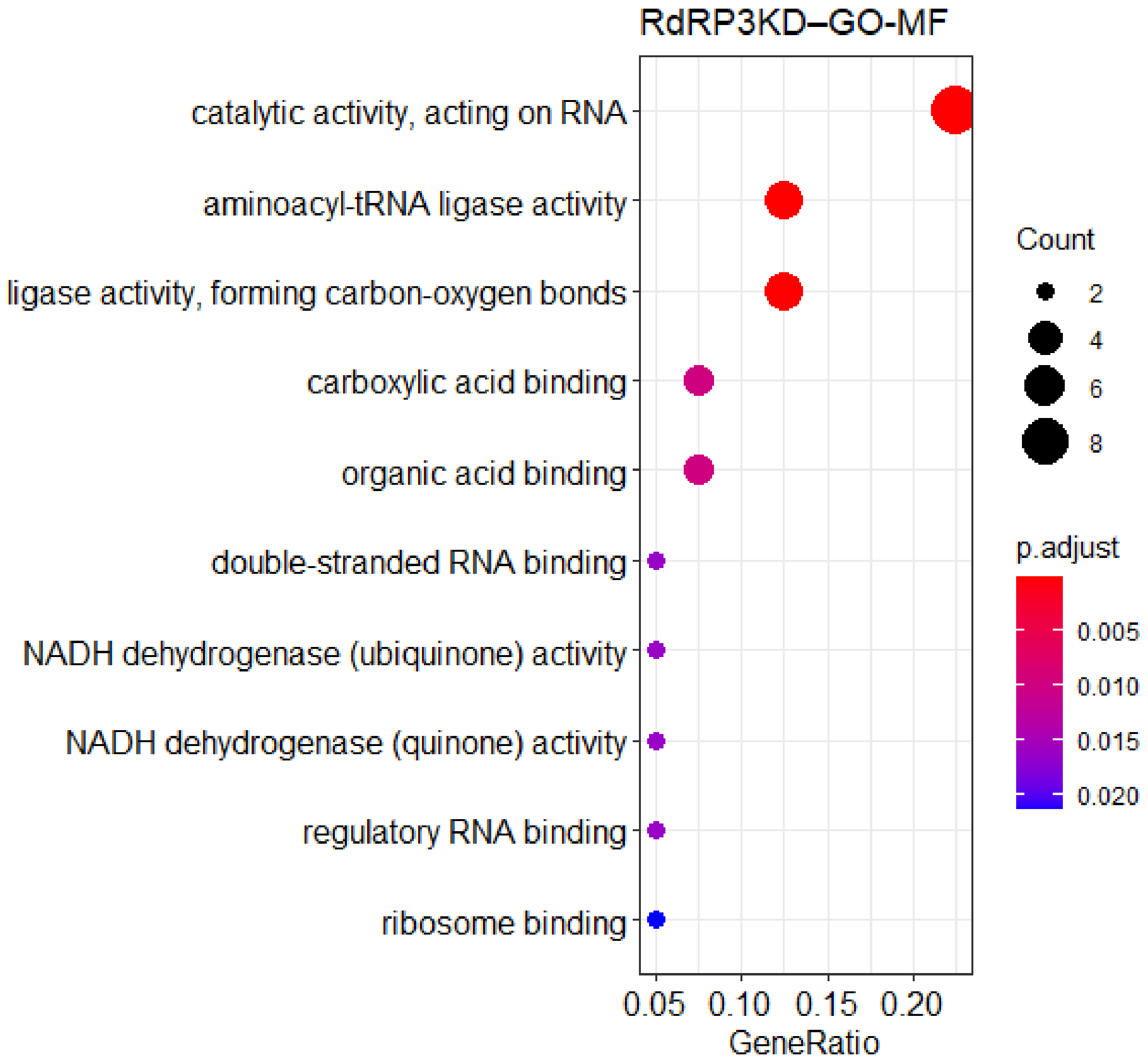


Dot plot showing the result of GO enrichment analysis (Molecular Function) for misregulated genes upon RdRP3 knockdown.

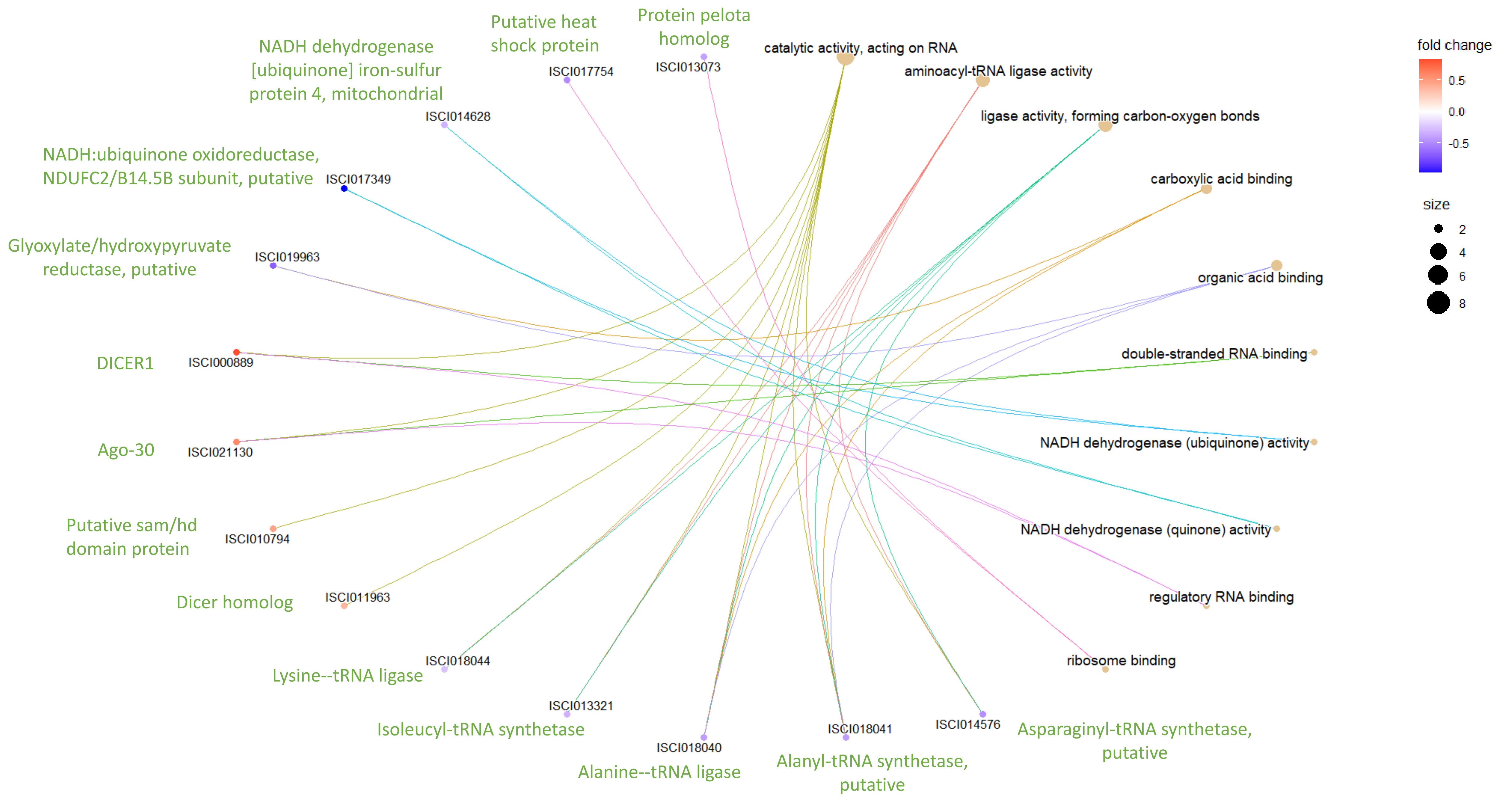


Linkage between GO terms(Molecular Function) and genes misregulated upon RdRP3 knockdown.
